# *De novo* assembly and transcriptome characterization: novel insights into the natural resistance mechanisms of *Microtus fortis* against *Schistosoma japonicum*

**DOI:** 10.1186/1471-2164-15-417

**Published:** 2014-06-02

**Authors:** Yuan Hu, Yuxin Xu, Weiyuan Lu, Zhongying Yuan, Hong Quan, Yujuan Shen, Jianping Cao

**Affiliations:** National Institute of Parasitic Diseases, Chinese Center for Disease Control and Prevention; Key Laboratory of Parasite and Vector Biology, MOH, Shanghai, 200025 China; WHO Collaborating Center for Malaria, Schistosomiasis and Filariasis, Shanghai, 200025 China

**Keywords:** *Microtus fortis*, *Schistosoma japonicum*, Non-permissive host, RNA-seq

## Abstract

**Background:**

*Microtus fortis* is a non-permissive host of *Schistosoma japonicum*. It has natural resistance against schistosomes, although the precise resistance mechanisms remain unclear. The paucity of genetic information for *M. fortis* limits the use of available immunological methods. Thus, studies based on high-throughput sequencing technologies are required to obtain information about resistance mechanisms against *S. japonicum*.

**Results:**

Using Illumina single-end technology, a *de novo* assembly of the *M. fortis* transcriptome produced 67,751 unigenes with an average length of 868 nucleotides. Comparisons were made between *M. fortis* before and after infection with *S. japonicum* using RNA-seq quantification analysis. The highest number of differentially expressed genes (DEGs) occurred two weeks after infection, and the highest number of down-regulated DEGs occurred three weeks after infection. Simultaneously, the strongest pathological changes in the liver were observed at week two. Gene ontology terms and pathways related to the DEGs revealed that up-regulated transcripts were involved in metabolism, immunity and inflammatory responses. Quantitative real-time PCR analysis showed that patterns of gene expression were consistent with RNA-seq results.

**Conclusions:**

After infection with *S. japonicum*, a defensive reaction in *M. fortis* commenced rapidly, increasing dramatically in the second week, and gradually decreasing three weeks after infection. The obtained *M. fortis* transcriptome and DEGs profile data demonstrated that natural and adaptive immune responses, play an important role in *M. fortis* immunity to *S. japonicum.* These findings provide a better understanding of the natural resistance mechanisms of *M. fortis* against schistosomes.

**Electronic supplementary material:**

The online version of this article (doi: 10.1186/1471-2164-15-417) contains supplementary material, which is available to authorized users.

## Background

Schistosome infections occur in over 70 tropical and subtropical countries, with 200 million individuals infected, resulting in 200,000 human deaths annually [1]. The pathology of chronic infections with *S. japonicum* includes granuloma formation in the liver, periportal fibrosis, portal hypertension and the formation of vascular shunts [2]. Serious schistosomiasis reduces the ability of humans to work and impedes the development of agriculture and livestock. It is crucial to control schistosomiasis transmission, improve the level of public health knowledge and increase income in agriculture and livestock. At present, praziquantel (PZQ) is the only safe, cheap and effective drug against human schistosome species. However, massive administration of PZQ in endemic areas has raised serious concerns regarding the development of parasite resistance to PZQ [3]. Isolates of *S. mansoni* from patients in Kisumu, Kenya, were shown to have lower susceptibility to PZQ [4]. Clarifying the immune mechanisms of *S. japonicum* infection is an important foundation to screen effective vaccine candidates and novelty drugs against schistosomiasis.

The reed vole (*Microtus fortis*) has been identified as a non-permissive host of *S. japonicum* that has a natural resistance mechanism against schistosomes [5–8]. Several studies of the resistance of *M. fortis* to *S. japonicum* have been conducted. Eosinophils, neutrophils, macrophages and serum antibodies are thought to be involved in resistance [9] and nitric oxide, albumin, E77, KPNA2, HSP90 were found to be resistance-associated proteins [9–11]. It has been suggested that apoptosis might be employed by *M. fortis* for eliminating schistosomes [12]. This study aimed to provide new suggestions to control the spread of schistosomiasis and to further elucidate the resistance mechanisms against schistosomes in *M. fortis*.

Because *M. fortis* is a new experimental animal model, biological information about it is very limited. To date, only 328 nucleotides, 68 ESTs and 26 genes of *M. fortis* are accessible from all NCBI databases, and no monoclonal antibodies specific for these markers are commercially available. It is therefore extremely difficult to study resistance mechanisms using immunological techniques, such as flow cytometry, Western blot or microarrays. Due to the high cost of genome sequencing, transcriptome sequencing is a useful tool to obtain biological information and to discover and identify new genes of *M. fortis*. The next-generation sequencing (NGS) based RNA-Seq technique has been widely used for *de novo* transcriptome sequencing assembly, discovery of novel genes and the investigation of gene expression in many non-model organisms [13]. It is therefore suitable for the identification of potential resistance-related genes in *M. fortis*.

In this study, we sequenced the *M. fortis* transcriptome using Illumina technology and demonstrated the suitability of short-read sequencing for the *de novo* assembly and annotation of genes expressed in a eukaryote without prior genome information. In addition, we compared the gene expression profiles of *M. fortis* up to four weeks after infection with schistosomes by using an RNA-Seq quantification expression system. The assembled and annotated transcriptome sequences and gene expression profiles showed that natural and adaptive immune responses, such as Toll-like receptors (TLR), natural killer (NK) cell activation and IgG and IgE, play important roles against *S. japonicum* infections. These data provide a valuable resource for the analysis of resistance genes of *M. fortis* against *S. japonicum*.

## Results

### Pathological changes in the liver

**Figure 1:**
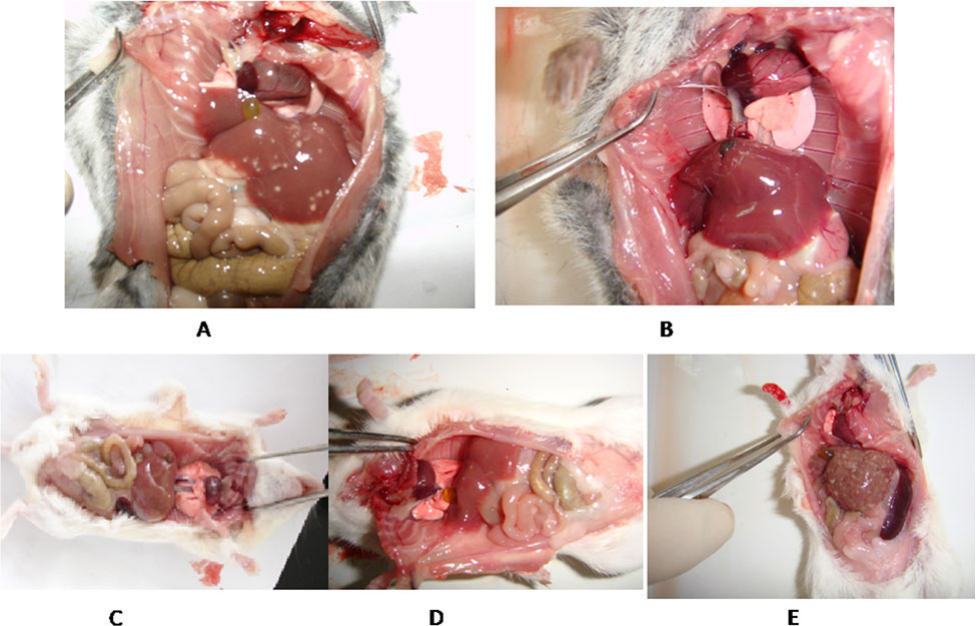
**Pathological liver changes. A**: Liver of *M. fortis* after two weeks of infection. **B**: Liver of *M. fortis* after four weeks of infection. **C**: Liver of BALB/c mouse after two weeks of infection. **D**: Liver of BALB/c mouse after four weeks of infection. **E**: Liver of BALB/c mouse after six weeks of infection.

After infection with *S. japonicum* for 2–3 weeks, several white nodules were observed in the liver of *M. fortis*, which disappeared 3–4 weeks after infection. The liver was then restored to its original color, structure and texture. This phenomenon was also observed by Shao [14] and it is speculated that these white nodules are induced by a schistosomulum [15]. Some reports described many worm-granulomas detected in the liver of *M. fortis*, schistosomula were impaired by inflammatory cells surrouding [15, 16]. Conversely, no changes occurred in the livers of BALB/c mice (which are permissive hosts for *S. japonicum*) after the first four weeks of infection. High numbers of liver granulomas were observed after the deposition of eggs in the liver of mice (Figure 1).

### Illumina sequencing and de novo transcriptome assembly

To obtain an overview of an *M. fortis* gene expression profile, cDNA from the liver of *M. fortis* was generated and sequenced using the Illumina sequencing platform. After removing adaptors and low quality reads, 134,985,444 clean reads were obtained from sequencing. The GC content and Q20 were 48.98% and 98.03%, respectively. These reads were assembled with the Trinity software. After clustering using the TGICL program, we obtained 166,501 contigs and 67,751 unigenes (mean length: 868 nt) with an N50 of 1,631 nt (Additional file 1: Table S1). Simultaneously, an abundance of unannotated unigenes was generated in our study, which might represent a specific gene pool of interest for further *M. fortis* studies.

### Function annotation and classification of assembled transcripts

**Figure 2:**
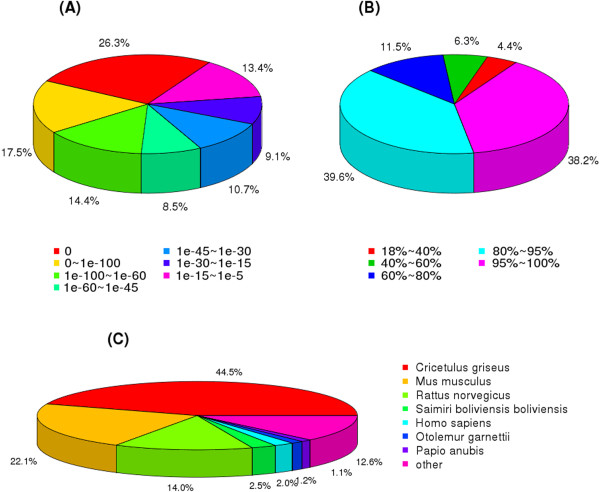
**Homology analysis of** ***M. fortis*** **transcriptome.** All distinct gene sequences that had BLAST annotations within the nr database with a cut-off E-value ≤ 10–5 were analyzed for: **A**: E-value distribution; **B**: similarity distribution; **C**: species distribution.

To annotate these unigenes, we searched the reference sequences using BLASTx against the NCBI non-redundant (nr) and Swiss-Prot protein databases with a cut-off E-value of 10^-5^. A total of 33,384 (49.27% of all distinct sequences) and 31,001 (45.76% of all distinct sequences) were obtained as significant hits using the respective databases (Additional file 2: Table S2). However, there were still many sequences (11,815, 17.44%) without BLASTx hits. These sequences might be new genes or non-coding RNA sequences. Among these, 55,936 unigenes (82.56% of 67,751 unigenes) were annotated with the NR, NT, Swiss-Prot, Kyoto Encyclopedia of Genes and Genomes (KEGG), COG and gene ontology (GO) by BLAST databases, and functional bioinformatics analyses. The annotation results show that the species *Cricetulus griseus* (44.5%), *Mus musculus* (22.1%), and *Rattus norvegicus* (14.0%) were highly homologous with *M. fortis* (Figure 2).

**Figure 3:**
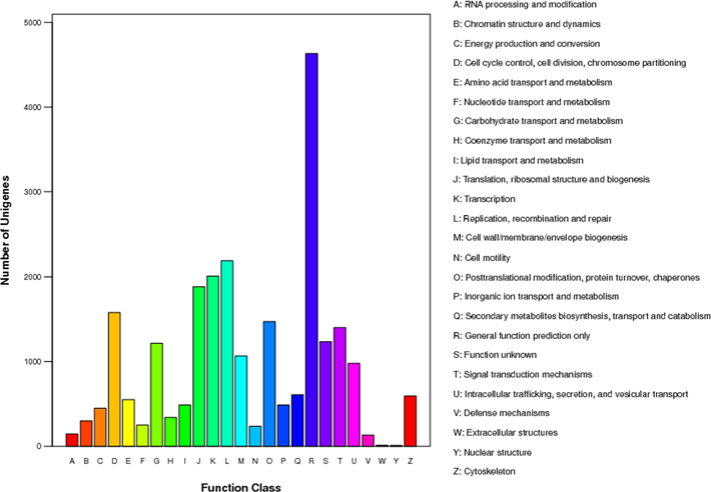
**COG function classification of assembled** ***M. fortis*** **transcripts.**

**Figure 4:**
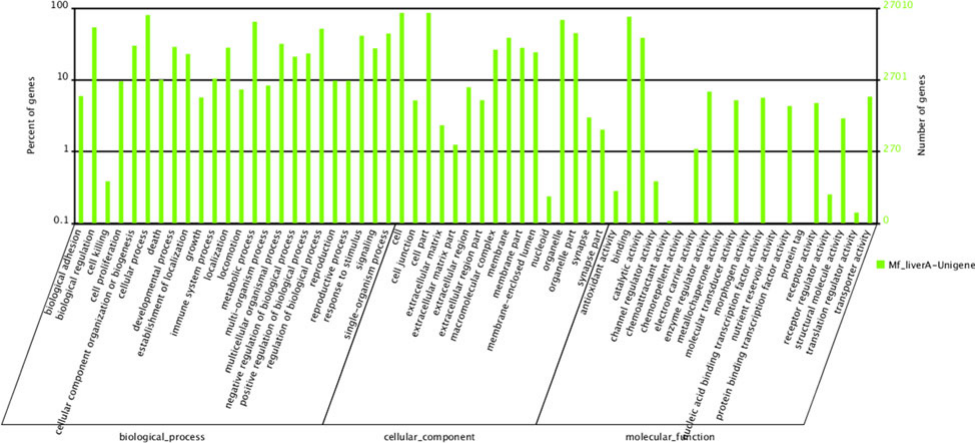
**Gene ontology classification of assembled** ***M. fortis*** **transcripts.**

To evaluate our transcriptome library, we searched the annotated sequences for the genes involved in COG classifications [17]. Unigenes were aligned to the COG database to predict and classify possible functions. A total of 24,238 sequences were functionally classified into 25 COG categories. The largest number of unigenes focused on “the general function of prediction” class, the second-largest groups were in the “replication, recombination and repair” and “transcription” classes, followed by “cell cycle control, cell division, chromosome partitioning”, “chromosome partitioning”, “posttranslational modification” and “protein turnover” (Figure 3). To further evaluate the completeness of the *de novo* assembly transcriptome and predict possible functions, a GO functional annotation was used to classify the functions of the predicted *M. fortis* genes. In total, 27,010 sequences could be categorized into 60 functional groups in the three main categories of “cellular component”, “molecular function” and “biological process”. In these categories the number of unigenes categorized as “biological process” accounted for nearly 40%. This dominant processes were involved in developmental biology, biosynthesis, metabolism, stimulus–response and signal transduction. A few genes were associated with terms such as “cell killing”, “nucleoid”, “chemoattractant activity”, “chemorepellent activity”, “receptor regulator activity” and “translation regulator activity” (Figure 4).

The KEGG is a bioinformatics resource for linking genomes to life and the environment and for understanding high-level gene functions in terms of networks of the biological system (http://www.genome.jp/kegg/). In our research, 23,898 unigenes were mapped to 258 KEGG pathways (Additional file 3: Dataset S1). Among them, “metabolic pathways” (2432 unigenes, 10.18% of sequences), “pathways in cancer” (993 unigenes, 4.16% of sequences 4.16%) and “regulation of actin cytoskeleton” (923 unigenes, 3.86% of sequences) were dominant. In 258 KEGG pathways, there were 20 pathways involved in immune and inflammatory responses (Table 1), such as endocytosis (2.87%), MAPK signaling pathway (2.62%), chemokine signaling pathway (2.12%), wnt signaling pathway (1.8%) and Fc gamma R-mediated phagocytosis (1.74%). The gene catalog provided a comprehensive understanding of the gene transcription profiles of *M. fortis*, and it provided a valuable foundation for screening differential expression genes after infection with *S. japonicum.*

**Table 1:** **Pathways of** ***de novo*** **transcriptome involved in immunity and inflammation responses**

Pathway	All genes with pathway annotation (23898)	Pathway ID
Endocytosis	685 (2.87%)	ko04144
MAPK signaling pathway	627 (2.62%)	ko04010
Chemokine signaling pathway	507 (2.12%)	ko04062
Wnt signaling pathway	430 (1.8%)	ko04310
Fc gamma R-mediated phagocytosis	416 (1.74%)	ko04666
Cytokine-cytokine receptor interaction	328 (1.37%)	ko04060
Leukocyte transendothelial migration	327 (1.37%)	ko04670
JAK-STAT signaling pathway	302 (1.26%)	ko04630
NF-kappa B signaling pathway	288 (1.21%)	ko04064
T cell receptor signaling pathway	256 (1.07%)	ko04660
B cell receptor signaling pathway	254 (1.06%)	ko04662
Complement and coagulation cascades	253 (1.06%)	ko04610
NK cell-mediated cytotoxicity	238 (1%)	ko04650
Notch signaling pathway	231 (0.97%)	ko04330
TGF-beta signaling pathway	225 (0.94%)	ko04350
Apoptosis	216 (0.9%)	ko04210
TLR signaling pathway	203 (0.85%)	ko04620
NOD-like receptor signaling pathway	185 (0.77%)	ko04621
Antigen-processing and presentation	136 (0.57%)	ko04612
Intestinal immune network for IgA production	54 (0.23%)	ko04672

### DEGs involved in the response to infection with S. japonicum

**Figure 5:**
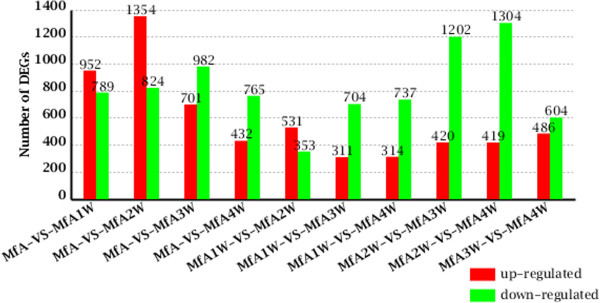
**Overview of differential expression at different points after infection** ***.*** In a pairwise comparison (denoted as MfA-VS-MfA1W, for example), the former (MfA) was considered as the control, and the latter (MfA1W) was considered as the treatment. MfA, *M. fortis* before infection; MfA1W: *M. fortis* after infection for 1 week; MfA2W: *M. fortis* after infection for 2 weeks; MfA3W: *M. fortis* after infection for 3 weeks; MfA4W: *M. fortis* after infection for 4 weeks.

**Figure 6:**
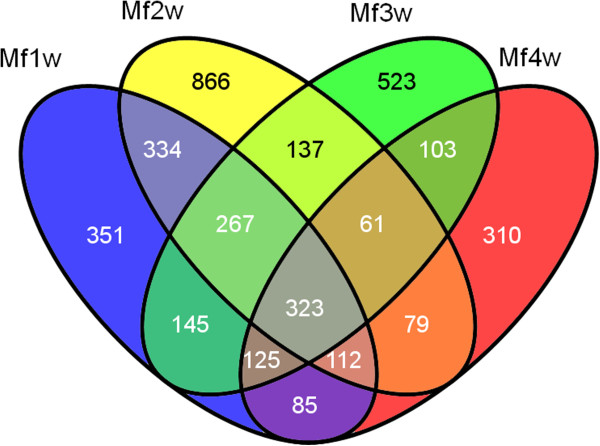
**Overlap between numbers of DEGs responding to** ***S. japonicum*** . MfA1W: *M. fortis* after infection for 1 week; MfA2W: *M. fortis* after infection for 2 weeks; MfA3W: *M. fortis* after infection for 3 weeks; MfA4W: *M. fortis* after infection for 4 weeks.

To investigate the changes in gene expression and understand the critical genes in *M. fortis* responses to *S. japonicum*, we used RNA-Seq technology to study transcriptional changes. Clean reads of *M. fortis* that were infected with *S. japonicum* from 1–4 weeks were respectively mapped to the *de novo* assembly transcriptome reference sequences of *M. fortis* using SOAP aligner/SOAP2 [18]. The clean reads mapped to the genes were approximately 86.93% to 87.70% in the four respective libraries, in which approximately 69.13% to 76.84% reads were perfectly matched (Table 2). The expression levels of the unigenes were calculated using the RPKM algorithm. The RPKM values can be directly used for comparing the differences in gene expression among samples. We used “FDR ≤ 0.001 and the absolute value of log_2_ ratio ≥ 2” as threshold to judge the significance of gene expression difference. From these results, the amounts of DEGs were detected at different time points pre- and post-infection (Figure 5). Based on RNA-Seq (quantification) analysis, it was shown that the number of up-regulated genes at the second week after infection was 1,354, which was much higher than that at other weeks. Compared to the second week after infection, the numbers of down-regulated genes at the third and fourth weeks after infection were 1,202 and 1,304, respectively. A high number of DEGs in the liver of *M. fortis* overlapped at different points after infection (Figure 6).

**Table 2:** **DGE sequencing statistics**

	Map to Gene	Total reads	Total BasePairs	Total mapped Reads	Perfect match	≤ 2 bp mismatch	Unique match	Multi-position match	Total unmapped reads
***M. fortis 0w***	**Number**	14108810	6.91E + 08	12386868	10841524	1545344	8899796	3487072	1721942
	**Percentage**	100.00%	100.00%	87.80%	76.84%	10.95%	63.08%	24.72%	12.20%
***M. fortis 1w***	**Number**	14541376	7.13E + 08	12661913	10539814	2122099	9081999	3579914	1879463
	**Percentage**	100.00%	100.00%	87.08%	72.48%	14.59%	62.46%	24.62%	12.92%
***M. fortis 2w***	**Number**	14414203	7.06E + 08	12569395	10659905	1909490	8823147	3746248	1844808
	**Percentage**	100.00%	100.00%	87.20%	73.95%	13.25%	61.21%	25.99%	12.80%
***M. fortis 3w***	**Number**	14029434	6.87E + 08	12263700	9975484	2288216	8773301	3490399	1765734
	**Percentage**	100.00%	100.00%	87.41%	71.10%	16.31%	62.53%	24.88%	12.59%
***M. fortis 4w***	**Number**	14093913	6.91E + 08	12252516	9743528	2508988	8705359	3547157	1841397
	**Percentage**	100.00%	100.00%	86.93%	69.13%	17.80%	61.77%	25.17%	13.07%

### GO and pathway functional enrichment analysis of DEGs

To understand the functions of the DEGs, we performed GO functional enrichment and KEGG pathway analyses using a hypergeometric distribution model. When comparing *M. fortis* samples before infection and four weeks after infection, the terms “cell periphery”, “extracellular space”, “plasma membrane”, “external side of plasma membrane”, “extracellular region”, “cell surface” and “cytoplasm” showed the most significant differences (Additional files 4, 5, 6 and 7: Datasets S2, S3, S4, S5).

By analyzing the statistically enriched GO function related to the DEGs, it was revealed that the most significant and most frequently enriched terms for up-regulated transcripts after infection were inflammatory- and immunity-correlated genes. The first class included pattern recognition receptors (PRRs), such as mannose-binding lectin (MBL, K03991), C-reactive protein (CRP, K01672), scavenger receptor (SR, GO:0005044), complement receptor (CR, GO:0002430) and TLRs (GO:0002755, GO:0006954, GO:0034162, GO:0034134). The second class consisted of cytokines and complement-related unigenes, such as interferon-gamma (IFN-γ)-related unigenes (GO:0003924, GO:0000975, GO:0005515, GO:0003723), tumor necrosis factor (TNF)-related unigenes (GO:0005164, GO:0033209, GO:0005031, GO:0043120) and complement-related unigenes (GO:0006956, GO:0006957, GO:0006958, GO:0001851). The third class was comprised of lymphocyte activation-related molecules, such as T- and B lymphocyte-related unigenes (GO:0003823, GO:0015643, GO:0005488, GO:0019901, GO:0005068) and macrophage-related unigenes (GO:0008603, GO:0043621, GO:0070891).

Pathways related to the DEGs related to inflammatory and immunity responses were “antigen-processing and presentation”, “intestinal immune network for IgA production”, “natural killer cell-mediated cytotoxicity”, “B cell receptor signaling pathway”, “MAPK signaling”, “NF-kappa B”, “JAK-STAT signaling”, “Toll-like receptor signaling”, “TGF-beta signaling pathway”, “wnt signaling pathway” and “complement and coagulation cascades” (Table 3).

**Table 3:** **Pathway of RNA-seq involved in immunity and inflammation responses after infection for four weeks**

Pathway	DEGs with pathway annotation				All genes with pathway annotation			
	1st w (700)	2nd w (946)	3rd w (665)	4th w (432)	1st w	2nd w	3rd w	4th w
Jak-STAT signaling	15 (2.14%)	22 (2.33%)	13 (1.95%)	5 (1.16%)	577 (1.51%)	577 (1.51%)	577 (1.51%)	577 (1.51%)
MAPK signaling	26 (3.71%)	29 (3.07%)	16 (2.41%)	9 (2.08%)	1058 (2.76%)	1058 (2.76%)	1058 (2.76%)	1058 (2.76%)
apoptosis	9 (1.29%)	12 (1.27%)	5 (0.75%)	3 (0.69%)	373 (0.97%)	373 (0.97%)	373 (0.97%)	373 (0.97%)
NF-kappa B	23 (3.29%)	37 (3.91%)	11 (1.65%)	8 (1.85%)	477 (1.25%)	477 (1.25%)	477 (1.25%)	477 (1.25%)
Toll-like receptor signaling	22 (3.14%)	22 (2.33%)	15 (2.26%)	4 (0.93%)	405 (1.06%)	405 (1.06%)	405 (1.06%)	405 (1.06%)
Natural killer cell mediated cytotoxicity	36 (5.14%)	43 (4.55%)	16 (2.41%)	8 (1.85%)	532 (1.39%)	532 (1.39%)	532 (1.39%)	532 (1.39%)
Fc gamma R-mediated phagocytosis	33 (4.71%)	41 (4.33%)	20 (3.01%)	16 (3.7%)	570 (1.49%)	570 (1.49%)	570 (1.49%)	570 (1.49%)
Complement and coagulation cascades	25 (3.57%)	28 (2.96%)	20 (3.01%)	19 (4.4%)	459 (1.2%)	459 (1.2%)	459 (1.2%)	459 (1.2%)
B cell receptor signaling pathway	22 (3.14%)	35 (3.7%)	15 (2.26%)	11 (2.55%)	424 (1.11%)	424 (1.11%)	424 (1.11%)	424 (1.11%)
T cell receptor signaling pathway	15 (2.14%)	23 (2.43%)	10 (1.5%)	11 (2.55%)	462 (1.21%)	462 (1.21%)	462 (1.21%)	462 (1.21%)
Cytokine-cytokine receptor interaction	21 (3%)	29 (3.07%)	22 (3.31%)	5 (1.16%)	738 (1.93%)	738 (1.93%)	738 (1.93%)	738 (1.93%)
RIG-I-like receptor signaling pathway	8 (1.14%)	10 (1.06%)	6 (0.9%)	2 (0.46%)	245 (0.64%)	245 (0.64%)	245 (0.64%)	245 (0.64%)
NOD-like receptor signaling pathway	8 (1.14%)	9 (0.95%)	6 (0.9%)	2 (0.46%)	343 (0.9%)	343 (0.9%)	343 (0.9%)	343 (0.9%)
TGF-beta signaling pathway	2 (0.29%)	12 (1.27%)	6 (0.9%)	1 (0.23%)	344 (0.9%)	344 (0.9%)	344 (0.9%)	344 (0.9%)
Wnt signaling pathway	5 (0.71%)	12 (1.27%)	3 (0.45%)	3 (0.69%)	630 (1.64%)	630 (1.64%)	630 (1.64%)	630 (1.64%)
Ubiquitin mediated proteolysis	5 (0.71%)	11 (1.16%)	9 (1.35%)	1 (0.23%)	668 (1.74%)	668 (1.74%)	668 (1.74%)	668 (1.74%)

Note: 1st w, first week after infection; 2nd w, second week after infection; 3rd w, third week after infection; 4th w, fourth week after infection.

### Validation of Illumina expression patterns by qRT-PCR analysis

**Figure 7:**
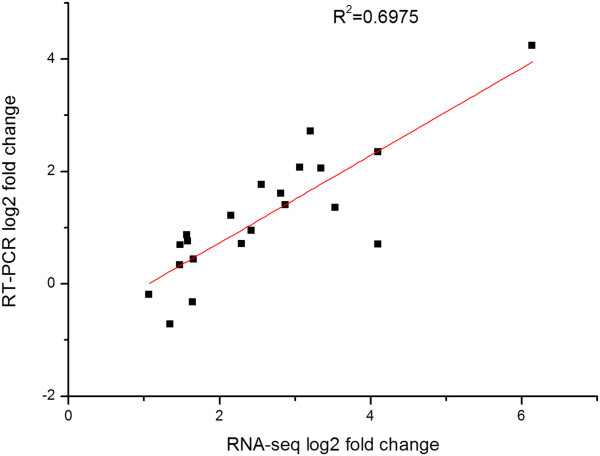
**Comparison of RNA-seq-derived ratios with RT-PCR analysis for selective genes.** R^2^, correlation coefficient.

**Figure 8:**
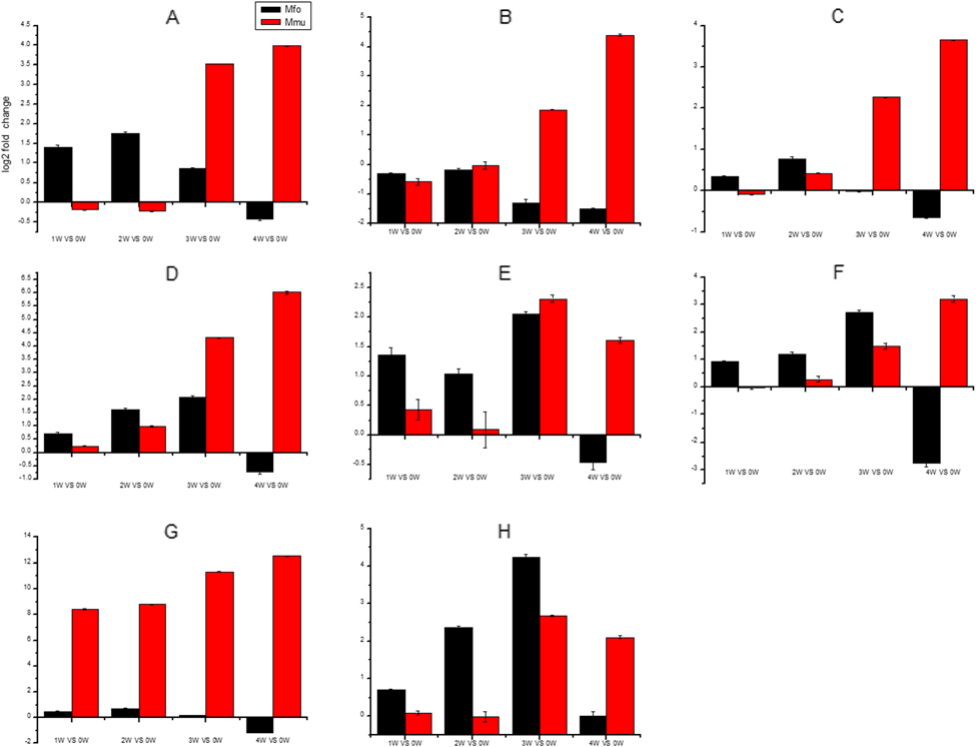
**Expression profiles of eight candidate DEGs in** ***M. fortis*** **and BALB/c mice. A**: CD74 in BALB/c, CL2855 contig 1 in *M. fortis*; **B**, Clec4n in BALB/c, Unigene 29960 in *M. fortis*; **C**, Tmsb4x in BALB/c, Unigene 36673 in *M. fortis*; **D**, Irf7 in BALB/c, Unigene 38015 in *M. fortis*; **E**, Tlr2 in BALB/c, Unigene 6959 in *M. fortis*; **F**, CD14 in BALB/c, Unigene 31333 in *M. fortis*; **G**, FcerIg in BALB/c, Unigene 36889 in *M. fortis*; **H**, Irgm1 in BALB/c, Cl2148 contig 4 in *M. fortis*. The expression value at the mRNA level was detected using quantitative RT-PCR. The expression times and expression levels of unigenes in the different hosts were significantly different.

To confirm the reliability of the sequencing analysis and to verify the relationship of unigenes to resistance against *S. japonicum*, eight candidate DEGs were selected and detected by qRT-PCR. Agarose gel electrophoresis results showed that eight primer pairs obtained a band of the expected size. The profile of their expression was consistent with the results from the RNA-Seq (Figure 7) and the results suggested that the unigene data were highly reliable. In *M. fortis*, unigenes were up-regulated for the first three weeks after infection, and gradually decreased at the fourth week after infection. In mice, the unigenes were up-regulated after the third or the fourth week after infection. The time point of up-regulation for genes in BALB/c mice was delayed by 3–4 weeks compared to *M. fortis*. The expression time points and expression levels of unigenes in different hosts were thus significantly different (Figure 8).

### Potential resistance-associated molecular analysis in M. fortis

We further analyzed DEGs to select up-regulated or down-regulated unigenes in the first three weeks after infection. In this period, the reaction to removing schistosomula from *M. fortis* was the most dramatic. Unigenes showing changes during this period might be potential resistance genes. These up-regulated unigenes were grouped into the following categories:

(i) Metabolism-related unigenes, such as glutathione S-transferase kappa 1, GTPase family M protein 1, 3 beta-hydroxysteroid dehydrogenase, calcium-dependent phospholipase A2, mitochondrial precursor, apolipoprotein L, insulin-like growth factor-binding protein 1, serpin B9-like, cytochrome P450 4A10;
(ii) Immunity-related unigenes, such as immunoglobulin IgG heavy chain, h-2 class I histocompatibility antigen, CD74 antigen, MHC class II antigen, interferon-induced protein with tetratricopeptide repeats 1-like, interferon regulatory factor 1, interferon regulatory factor 7, interferon regulatory factor 8, interferon regulatory factor 9, lymphocyte antigen 86-like, T cell surface glycoprotein CD8 alpha chain-like, B cell linker protein, monocyte differentiation antigen CD14-like;
(iii) Inflammation-related unigenes, such as fibronectin precursor, C-X-C motif chemokine 10-like, C-C motif chemokine 5, Janus kinase 3;
(iv) Apoptosis-related genes, such as caspase-8 and bcl-2 homologous antagonist.

The results showed that unigenes down-regulated after infection were, among others, salivary gland secretion protein 4, cholesterol 7-alpha-monooxygenase, UDP-glucuronosyltransferase 2B1-like isoform 2, UDP-glucuronosyltransferase 1-9-like, hematopoietically expressed homeobox, zinc finger and BTB domain-containing protein 16. These results showed that after infection of *M. fortis* with *S. japonicum*, metabolism, immunity, inflammation, and apoptosis-related gene expression levels increased, whereas synthesis was reduced.

## Discussion

*M. fortis* and BALB/c mice are non-permissive and permissive hosts of *S. japonicum*, respectively. After infection, the profiles of gene expression, immune response and pathological changes of the two hosts were significantly different. A previous study revealed that the lungs and liver of *M. fortis*, rats and mice display different characteristics when infected with *S. japonicum* for 10 days. *M. fortis* and rats, both of which are resistant to *S. japonicum*, developed a stronger immune response and more severe pathological lesions in response to schistosomes than mice during the early phase of the infection [15]. After infection with *S. japonicum*, levels of cytokines, complements and antibodies were significantly higher in *M. fortis* than in mice [9, 19]. In our study, the changes in cytokine expression were detected by using protein microarrays in the sera of *M. fortis* voles and C57BL/6 mice. Levels of Th1, Th2, and Th17 cytokines in *M. fortis* voles increased significantly during the first 3 weeks post-infection, while there was no significant changes in mice (Data not shown). It has been found that after infection with *S. japonicum* for 1 week, serine protease inhibitor gene expression was up-regulated and immune-associated gene expression did not change in the lung tissue of mice. Conversely, some important immune-associated genes (such as CD74, MHC-I and MHC-II) were up-regulated, and serine protease inhibitor gene expression did not change in *M. fortis* [20]. Pathological changes in the liver of *M. fortis* were significant during the first 3 weeks post-infection, while there was no significant changes in the liver of mice (shown in Figure 1). These results help to clarify molecular mechanisms of infection immunity in different hosts of *S. japonicum*, which might provide new ways to prevent and control schistosomiasis.

To investigate the changes in gene expression and understand the critical genes in the response of *M. fortis* to *S. japonicum* infection, RNA-Seq (quantification) analysis was performed and various differently-expressed genes were obtained. The number of up-regulated genes at the second week after infection was 1,354, which was higher than at other time points. Compared to the second week after infection, the number of down-regulated genes at the third and the fourth week after infection was 1,202 and 1,304, respectively, which was higher than at other weeks. It was thus concluded that the in vivo response of *M. fortis* was the most intense at the second week after infection. Three weeks after infection, the response to schistosomes in *M. fortis* gradually leveled off. This result was consistent with pathological changes in the liver after infection. GO and pathway functional enrichment analysis of the DEGs showed that “cell periphery”, “extracellular space”, “plasma membrane”, “external side of plasma membrane”, “extracellular region”, “cell surface” and “cytoplasm” were the most significantly changed categories. These structures include metabolic enzymes, cell surface receptors, signaling molecules, and platelet-derived growth factor-binding structures. The most significantly and most frequently enriched terms that were up-regulated transcripts after infection were inflammatory, immunity-correlated genes, and metabolism-related enzymes (Additional file 8: Dataset S6).

NK cells are a type of cytotoxic lymphocytes critical to the innate immune system. They control immune responses by secreting cytokines and chemokines, including tumor necrosis factor-α (TNF-α) and interferon-γ (IFN-γ), and eliminate target cells by polarized exocytosis of cytotoxic granules containing perforin, granzymes and Fas ligand [21]. Binding of IFN-γ (or type II interferon) to its receptors activates the JAK-STAT pathway and has an immune-modulatory function [22]. IFN-γ (originally called macrophage-activating factor) can stimulate macrophages and induce direct antimicrobial and antitumor mechanisms. It can also up-regulate antigen-processing and antigen-presenting pathways [23]. IFN-γ orchestrates leukocyte attraction, thus enhancing NK cell activity and regulating B cell functions, such as immunoglobulin (Ig) production and class switching [24]. Our results show that unigenes involved in IFN-γ, such as interferon regulatory factor 1, interferon regulatory factor 7, interferon regulatory factor 8, interferon regulatory factor 9 and interferon-induced protein with tetratricopeptide repeats 1-like, were significantly up-regulated. Simultaneously, by using genome oligonucleotide microarrays, some studies also reported that after infection with *S. japonicum* for 10 days, a gene similar to IFN-γ receptor 2 was up-regulated in the lungs of *M. fortis* [15]. Members of the TNF/TNF-receptor (TNF-R) superfamily coordinate the immune response at multiple levels, such as by lymphoid neogenesis, augmenting immune responses and apoptosis dependent on caspase 8. Among these, acute TNF-R engagement can induce the rapid and robust activation of NF-κB and MAPK pathways [25]. In our study, the TNF-related genes and pathways were significantly up-regulated. These included TNF-receptor binding (ligand) superfamily, TNF-mediated signaling pathway, TNF-activated receptor activity member 1B precursor, TNF binding, caspase 8 (K04398), apoptosis, NF-κB and MAPK pathways. This indicates that IFN-γ and TNF might play important roles in the response against *S. japonicum* infections.

TLRs are among the best-characterized PRRs and are key regulators of anti-pathogen immune responses. Schistosomal-derived lysophosphatidylcholine participates in the production of cytokines, such as TNF-α and IL-10, and in eosinophil activation through a TLR2-dependent pathway in *S. mansoni* and *S. haematobium* infection [26, 27]. Antigen-presenting cells (APCs) recognize lysophosphatidylserine and schistosomal glycolipids of schistosomes through TLRs, leading to NF-κB and MAPK signaling pathway activation and inducing inflammatory cytokine production [28].

The CD14 antigen is a glycosylphosphatidylinositol (GPI)-anchored receptor known to serve as a co-receptor for several TLRs that were significantly up-regulated in *M. fortis* after infection with *S. japonicum*. Earlier studies showed a role for CD14 as a co-receptor working with TLR4 and facilitating cellular responses to low doses of lipopolysaccharide (LPS). Upon LPS stimulation, CD14 enables the activation of the TLR4-TRAM-TRIF pathway and leads to the activation of NFAT transcription factor family members [29]. This suggests that CD14 and TLRs play an important role in initiating inflammation by responding to LPS stimulation in *M. fortis*.

Adaptive immunity is an important part of the anti-schistosomiasis mechanism in *M. fortis*. Three weeks after infection, many unigenes were involved in adaptive immunity, including immunoglobulin IgG heavy chain, Fc receptor of IgE and CD8A antigen protein complex, CD74. Antibodies are major components of the immune system. IgG is the main antibody isotype found in blood and extracellular fluid, allowing it to control the infection of body tissues. IgG can mediate a variety of biological functions, such as the classical pathway of complement activation, ADCC, hypersensitivity, and can block pathogens. The Fc receptors of IgE are proteins called Fc-epsilon receptors (FcϵR) found on the surface of certain cells - including mast cells, eosinophils, basophils and Langerhans cells - that contribute to the protective functions of the immune system [30]. The CD8 antigen is a cell surface glycoprotein found on most cytotoxic T lymphocytes that mediates efficient cell-cell interactions within the immune system. CD74, known as the major histocompatibility complex (MHC) class II invariant chain, regulates the trafficking of MHC-II in APCs and acts as a receptor for macrophage migration inhibitory factor (MIF). After the binding of the MIF, NF-κB and Erk1/2 activation occurs, along with the induction of proinflammatory cytokine secretion [31]. It has been reported that the serum of *M. fortis* was passively transferred in mice, which not only reduced worm burden but also led to worm shortening [32]. Another report showed that purified IgG3 antibodies from laboratory-bred *M. fortis* and wild *M. fortis* effectively killed schistosomula, and that IgG3 antibodies of wild *M. fortis* induced a higher worm-reduction rate. The death rates of schistosomula due to IgG3 antibody purified from the sera of laboratory-bred *M. fortis* and wild *M. fortis* were 2.35 and 5.88 times higher than in Km mice, respectively [33]. It has been shown that the sera and/or spleen cells of *M. fortis* had an important effect on killing schistosomula *in vitro*. This study could also show a synergism between sera of *M. fortis* and spleen cells [34]. In our study, after infection with *S. japonicum* for three weeks, cytokines (such as IL-4, IL-5, IL-6, IL-13, TNF-α and GM-CSF) in the sera of *M. fortis* were significantly increased, whereas cytokine levels of the BALB/c mice were much lower (data not shown). These findings suggest that these parasite-killing effects were performed in part by CD8^+^ T and B lymphocytes.

## Conclusions

In summary, we first characterized the *M. fortis de novo* transcriptome and performed RNA-Seq quantification analysis after infection with *S. japonicum*. A total of 134,985,444 clean reads, 166,501 contigs and 67,751 unigenes were obtained. Based on the assembled *de novo* transcriptome, the amount of DEGs that play significant roles in the response to schistosome infection were identified. The numbers of DEGs at weeks 1–4 were 1741, 2178, 1683 and 1197, respectively. We concluded that the in vivo response of *M. fortis* was the most intense at the second week after infection. GO and pathway enrichment analysis revealed that after infection the up-regulated DEGs were predominately involved in metabolism, inflammation response and immunity response. The natural and adaptive immune responses, particularly NK cell activation producing IFN-γ and TGF-β, TLR activation of macrophages, and IgG and IgE antibodies produced by B lymphocytes, play an important role in *M. fortis* resistance against *S. japonicum.* Multiple candidate genes involved in innate immune responses were identified further with qRT-PCR, and expression patterns in *M. fortis* and BALB/c mice were compared. The results reflected significant differences of innate immune responses and different sensitivities in the hosts of schistosomes. This study provides a valuable molecular basis for the analysis of *M. fortis* resistance mechanisms against *S. japonicum*.

## Methods

### Ethics statement

This study was performed in strict accordance with the recommendations of the Laboratory of Animal Welfare & Ethics Committee (LAWEC) of China. No specific permissions from Hunan provincial authorities were required because the study did not involve endangered or protected species. The protocol was approved by the LAWEC Committee of the National Institute of Parasitic Diseases, Chinese Center for Disease Control and Prevention (approval ID: IPD 2009–4). All surgery was performed under sodium pentobarbital anesthesia, and all efforts were made to minimize suffering.

### Animals and cercariae

*M. fortis* voles were live captured in the Hunan Dongtin Lakes region, China. The animals were transported to Shanghai and reared in independent ventilated cages. Fifteen *M. fortis* females (aged 7–8 weeks, weighing 60–70 g) were randomly selected from a sub-generation of wild animals to be used in the experiment. BALB/c mice (aged 7–8 weeks, weighing 20–25 g) were purchased from the Experimental Animal Department of the Chinese Academy of Sciences. The animals were infected cutaneously (see below) with a mainland strain of *S. japonicum* cercariae. Infected *Oncomelanias hupensis* snails were provided by the National Institute of Parasitic Diseases, Chinese Center for Disease Control and Prevention. The infected snails were placed in dechlorinated water under artificial light to induce cercarial shedding before animal infections.

### Sample preparation

*M. fortis* voles and BALB/c mice were infected percutaneously with *S. japonicum* cercariae. Based on the susceptibility of each rodent species to *S. japonicum*, *M. fortis* voles were infected with 1,000 cercariae and BALB/c mice were infected with 40 cercariae per individual. Fifteen *M. fortis* voles and 15 BALB/c mice were divided into five groups randomly. Animals in each group were sacrificed either before infection or at 1, 2, 3 and 4 weeks post-infection. Before the animals were sacrificed, they were anesthetized with 0.75% sodium pentobarbital by intraperitoneal injection, and the livers of the animals were removed, frozen in liquid nitrogen and stored at -80°C until further use.

### Preparation of cDNA library and Illumina sequencing for transcriptome analysis

Total RNA was extracted using Trizol reagent (Invitrogen, USA) following the manufacturer’s protocol. After total RNA extraction and DNase I treatment, magnetic beads with Oligo (dT) were used to isolate mRNA. The mRNA was fragmented into short fragments when it was mixed with the fragmentation buffer. Then, cDNA was synthesized using the mRNA fragments as templates. The short fragments were purified and resolved with EB buffer for end reparation and single nucleotide A (adenine) addition. Afterwards, the short fragments were connected with adapters. The suitable fragments were selected as templates for PCR amplification. During the QC steps, an Agilent 2100 Bioanalyzer and an ABI StepOnePlus Real-Time PCR System were used for quantification and qualification of the sample library. The library was sequenced using Illumina HiSeq™ 2000.

Raw reads were produced from the sequences. Clean reads were obtained after removal of low quality reads, reads with adaptors and reads with unknown nucleotides larger than 5%. A *de novo* transcriptome assembly was performed using the short reads assembling program Trinity [35]. The resulting sequences obtained from Trinity are called unigenes. Then, a BLASTx alignment (E-value < 0.00001) between unigenes and protein databases, such as NR, Swiss-Prot, KEGG and COG, was performed, and the best alignment results were used to determine the sequence direction of the unigenes. With NR annotation, we used the Blast2GO program [36] to obtain the GO annotation of the unigenes. After obtaining the GO annotation for every unigene, we used WEGO software [37] to perform a GO functional classification for all unigenes and to further understand the distribution of gene functions in the species on a macroscopic level. We predicted genes with different expression levels, and performed GO functional analysis and KEGG pathway analysis on them.

### RNA-Seq quantification analysis

Four independent cDNA libraries were constructed for the four liver samples according to the RNA-Seq protocol. Raw image files were collected using the Illumina HiSeqTM2000 sequencing platform in BGI Shenzhen, China (http://en.genomics.cn/navigation/index.action). The analyzed data have been deposited in the NCBI Sequence Read Archive under the Accession No. SRX337491.

### Screening of DEGs

Screening of DEGs included the screening of genes that are differentially expressed among samples followed by a GO functional enrichment analysis and a KEGG pathway enrichment analysis for these DEGs. The expression level of each gene was measured via RNA-Seq quantification analysis using the RPKM [38] method (reads per kb per million reads). Referring to “The significance of digital gene expression profiles” [39], we developed a strict algorithm (the Poisson distribution) to identify DEGs between two samples. We performed a cluster analysis of gene expression patterns with cluster [40] software and Java Treeview [41] software.

GO offers a dynamically-updated controlled vocabulary and a strictly defined concept to comprehensively describe properties of genes and their products in any organism. GO enrichment analysis provides all GO terms that are significantly enriched in DEGs compared to the genome background and filters those DEGs that correspond to biological functions. This method first maps all DEGs to GO terms in a database (http://www.geneontology.org/). Genes usually interact with one another to play roles in certain biological functions. The pathway enrichment analysis identified significantly enriched metabolic pathways or signal transduction pathways in DEGs compared with the entire genome background.

### RNA isolation for reverse transcription and real-time quantitative PCR (qPCR)

Total RNA was extracted from the livers of *M. fortis* and BALB/c mice using a QIAgen RNeasy Mini Kit according to the manufacturer’s specifications. The RNA was determined using a NanoDrop 2000 spectrophotometer (Thermo Scientific, USA), and the integrity was evaluated using electrophoresis on an agarose gel stained with ethidium bromide. Reactions were performed in a GeneAmp® PCR System 9700 (Applied Biosystems, USA) for 15 min at 37°C, followed by heat inactivation of RT for 5 s at 85°C. Real-time PCR was performed using a LightCycler® 480 II Real-time PCR Instrument (Roche, Switzerland) with 10 μl of PCR reaction mixture. Reactions were incubated in a 384-well optical plate (Roche, Switzerland) at 95°C for 10 min, followed by 40 cycles of 95°C for 10 s and 60°C for 30 s. Each sample was analyzed in triplicates. At the end of the PCR cycles, a melting curve analysis was performed to validate the specific generation of the expected PCR product. The primer sequences (Additional file 9: Table S3) were designed in the laboratory and synthesized by Generay Biotech (Generay, China) based on the mRNA sequences obtained from the NCBI database and the RNA-Seq. The expression levels of mRNAs were normalized to GAPDH and were calculated using the 2^-ΔΔ^Ct method [42].

### Availability of supporting data

Raw sequence read data were submitted to the NCBI Sequence Read Archive under the Accession No. SRX337491, http://www.ncbi.nlm.nih.gov/bioproject/PRJNA215691).

## Electronic supplementary material

Additional file 1: Table S1: Statistics of assembly quality. (DOC 24 KB)

Additional file 2: Table S2: Summary of annotation results. (DOC 24 KB)

Additional file 3: Dataset S1: Pathways of *M. fortis* liver-unigenes. (ZIP 163 KB)

Additional file 4: Dataset S2: GO terms for MfA-VS-MfA1W_C. (ZIP 44 KB)

Additional file 5: Dataset S3: GO terms for MfA-VS-MfA2W_C. (ZIP 64 KB)

Additional file 6: Dataset S4: GO terms for MfA-VS-MfA3W_C. (ZIP 42 KB)

Additional file 7: Dataset S5: GO terms for MfA-VS-MfA4W_C. (ZIP 26 KB)

Additional file 8: Dataset S6: Trends of consistent genes for three weeks after infection. (XLS 20 KB)

Additional file 9: Table S3: Primer sequences used in qPCR. (XLS 570 KB)
