# Supplementary material for: De novo assembly and transcriptome characterization: novel insights into the natural resistance mechanisms of Microtus fortis against Schistosoma japonicum
Source: BMC Genomics. 2014 Jun 2;15(1):417. doi: 10.1186/1471-2164-15-417 (PMC4073500; doi:10.1186/1471-2164-15-417)
Supplement: Supplementary file 1 — Additional file 1: Table S1: Statistics of assembly quality. (DOC 24 KB) [file 12864_2013_6159_MOESM1_ESM.doc]

**Table S1 Statistics of assembly quality**

|  | sample | Total Number | Total Length(nt) | Mean Length (nt) | N50 | Total Consensus Sequences | Distinct Clusters | Distinct Singletons |
| --- | --- | --- | --- | --- | --- | --- | --- | --- |
| Contig | M.fortis_liver | 166,501 | 54,048,038 | 325 | 599 | - | - | - |
| Unigene | M.fortis_liver | 67,751 | 58,808,484 | 868 | 1631 | 67,751 | 15,731 | 52,020 |
