# Supplementary material for: De novo assembly and transcriptome characterization: novel insights into the natural resistance mechanisms of Microtus fortis against Schistosoma japonicum
Source: BMC Genomics. 2014 Jun 2;15(1):417. doi: 10.1186/1471-2164-15-417 (PMC4073500; doi:10.1186/1471-2164-15-417)
Supplement: Supplementary file 2 — Additional file 2: Table S2: Summary of annotation results. (DOC 24 KB) [file 12864_2013_6159_MOESM2_ESM.doc]

**Table S2 Summary of annotation results**

| Sequence File | NR | NT | Swiss-Prot | KEGG | COG | GO | ALL |
| --- | --- | --- | --- | --- | --- | --- | --- |
| Mf_liverA-Unigene.fa | 33,384 | 55,525 | 31,001 | 23,898 | 10,836 | 27,010 | 55,936 |
