# Supplementary material for: De novo assembly and transcriptome characterization: novel insights into the natural resistance mechanisms of Microtus fortis against Schistosoma japonicum
Source: BMC Genomics. 2014 Jun 2;15(1):417. doi: 10.1186/1471-2164-15-417 (PMC4073500; doi:10.1186/1471-2164-15-417)
Supplement: Supplementary file 3 — Additional file 3: Dataset S1: Pathways of M. fortis liver-unigenes. (ZIP 163 KB) [file 12864_2013_6159_MOESM3_ESM.zip › 1990354100108772_add3.htm]

Mf\_liverA-Unigene.fa

1. Mf\_liverA-Unigene.fa

| # | Pathway | All genes with pathway annotation (23898) | Pathway ID |
| --- | --- | --- | --- |
| 1 | Metabolic pathways | 2432 (10.18%) (NaN%) | ko01100 |
| 2 | Pathways in cancer | 993 (4.16%) (NaN%) | ko05200 |
| 3 | Regulation of actin cytoskeleton | 923 (3.86%) (NaN%) | ko04810 |
| 4 | Focal adhesion | 865 (3.62%) (NaN%) | ko04510 |
| 5 | Endocytosis | 685 (2.87%) (NaN%) | ko04144 |
| 6 | RNA transport | 642 (2.69%) (NaN%) | ko03013 |
| 7 | HTLV-I infection | 627 (2.62%) (NaN%) | ko05166 |
| 8 | MAPK signaling pathway | 627 (2.62%) (NaN%) | ko04010 |
| 9 | Transcriptional misregulation in cancer | 589 (2.46%) (NaN%) | ko05202 |
| 10 | Amoebiasis | 577 (2.41%) (NaN%) | ko05146 |
| 11 | Epstein-Barr virus infection | 572 (2.39%) (NaN%) | ko05169 |
| 12 | Herpes simplex infection | 568 (2.38%) (NaN%) | ko05168 |
| 13 | Tight junction | 564 (2.36%) (NaN%) | ko04530 |
| 14 | Ubiquitin mediated proteolysis | 550 (2.3%) (NaN%) | ko04120 |
| 15 | Spliceosome | 528 (2.21%) (NaN%) | ko03040 |
| 16 | Chemokine signaling pathway | 507 (2.12%) (NaN%) | ko04062 |
| 17 | Vascular smooth muscle contraction | 503 (2.1%) (NaN%) | ko04270 |
| 18 | Protein processing in endoplasmic reticulum | 481 (2.01%) (NaN%) | ko04141 |
| 19 | Salmonella infection | 474 (1.98%) (NaN%) | ko05132 |
| 20 | Adherens junction | 464 (1.94%) (NaN%) | ko04520 |
| 21 | mRNA surveillance pathway | 455 (1.9%) (NaN%) | ko03015 |
| 22 | ECM-receptor interaction | 451 (1.89%) (NaN%) | ko04512 |
| 23 | Insulin signaling pathway | 438 (1.83%) (NaN%) | ko04910 |
| 24 | Wnt signaling pathway | 430 (1.8%) (NaN%) | ko04310 |
| 25 | Huntington's disease | 422 (1.77%) (NaN%) | ko05016 |
| 26 | Fc gamma R-mediated phagocytosis | 416 (1.74%) (NaN%) | ko04666 |
| 27 | Influenza A | 416 (1.74%) (NaN%) | ko05164 |
| 28 | Purine metabolism | 413 (1.73%) (NaN%) | ko00230 |
| 29 | Phagosome | 400 (1.67%) (NaN%) | ko04145 |
| 30 | Tuberculosis | 398 (1.67%) (NaN%) | ko05152 |
| 31 | Dilated cardiomyopathy | 387 (1.62%) (NaN%) | ko05414 |
| 32 | Viral myocarditis | 373 (1.56%) (NaN%) | ko05416 |
| 33 | Hypertrophic cardiomyopathy (HCM) | 366 (1.53%) (NaN%) | ko05410 |
| 34 | Axon guidance | 364 (1.52%) (NaN%) | ko04360 |
| 35 | Cell cycle | 350 (1.46%) (NaN%) | ko04110 |
| 36 | Lysine degradation | 348 (1.46%) (NaN%) | ko00310 |
| 37 | Bacterial invasion of epithelial cells | 343 (1.44%) (NaN%) | ko05100 |
| 38 | Toxoplasmosis | 341 (1.43%) (NaN%) | ko05145 |
| 39 | Cytokine-cytokine receptor interaction | 328 (1.37%) (NaN%) | ko04060 |
| 40 | Leukocyte transendothelial migration | 327 (1.37%) (NaN%) | ko04670 |
| 41 | Small cell lung cancer | 326 (1.36%) (NaN%) | ko05222 |
| 42 | Shigellosis | 319 (1.33%) (NaN%) | ko05131 |
| 43 | Lysosome | 319 (1.33%) (NaN%) | ko04142 |
| 44 | Protein digestion and absorption | 314 (1.31%) (NaN%) | ko04974 |
| 45 | Calcium signaling pathway | 313 (1.31%) (NaN%) | ko04020 |
| 46 | Osteoclast differentiation | 312 (1.31%) (NaN%) | ko04380 |
| 47 | Phosphatidylinositol signaling system | 304 (1.27%) (NaN%) | ko04070 |
| 48 | Jak-STAT signaling pathway | 302 (1.26%) (NaN%) | ko04630 |
| 49 | Measles | 301 (1.26%) (NaN%) | ko05162 |
| 50 | Neurotrophin signaling pathway | 297 (1.24%) (NaN%) | ko04722 |
| 51 | Bile secretion | 296 (1.24%) (NaN%) | ko04976 |
| 52 | Alzheimer's disease | 294 (1.23%) (NaN%) | ko05010 |
| 53 | NF-kappa B signaling pathway | 288 (1.21%) (NaN%) | ko04064 |
| 54 | Pathogenic Escherichia coli infection | 283 (1.18%) (NaN%) | ko05130 |
| 55 | Salivary secretion | 279 (1.17%) (NaN%) | ko04970 |
| 56 | RNA degradation | 279 (1.17%) (NaN%) | ko03018 |
| 57 | Prostate cancer | 278 (1.16%) (NaN%) | ko05215 |
| 58 | Pyrimidine metabolism | 262 (1.1%) (NaN%) | ko00240 |
| 59 | Oocyte meiosis | 259 (1.08%) (NaN%) | ko04114 |
| 60 | Cardiac muscle contraction | 258 (1.08%) (NaN%) | ko04260 |
| 61 | T cell receptor signaling pathway | 256 (1.07%) (NaN%) | ko04660 |
| 62 | B cell receptor signaling pathway | 254 (1.06%) (NaN%) | ko04662 |
| 63 | Complement and coagulation cascades | 253 (1.06%) (NaN%) | ko04610 |
| 64 | Vibrio cholerae infection | 252 (1.05%) (NaN%) | ko05110 |
| 65 | Hepatitis C | 251 (1.05%) (NaN%) | ko05160 |
| 66 | Ribosome biogenesis in eukaryotes | 247 (1.03%) (NaN%) | ko03008 |
| 67 | Dopaminergic synapse | 245 (1.03%) (NaN%) | ko04728 |
| 68 | ErbB signaling pathway | 241 (1.01%) (NaN%) | ko04012 |
| 69 | Natural killer cell mediated cytotoxicity | 238 (1%) (NaN%) | ko04650 |
| 70 | Serotonergic synapse | 237 (0.99%) (NaN%) | ko04726 |
| 71 | Cell adhesion molecules (CAMs) | 236 (0.99%) (NaN%) | ko04514 |
| 72 | GnRH signaling pathway | 234 (0.98%) (NaN%) | ko04912 |
| 73 | Inositol phosphate metabolism | 233 (0.97%) (NaN%) | ko00562 |
| 74 | Notch signaling pathway | 231 (0.97%) (NaN%) | ko04330 |
| 75 | Metabolism of xenobiotics by cytochrome P450 | 228 (0.95%) (NaN%) | ko00980 |
| 76 | TGF-beta signaling pathway | 225 (0.94%) (NaN%) | ko04350 |
| 77 | Hematopoietic cell lineage | 224 (0.94%) (NaN%) | ko04640 |
| 78 | Melanogenesis | 221 (0.92%) (NaN%) | ko04916 |
| 79 | Alcoholism | 220 (0.92%) (NaN%) | ko05034 |
| 80 | Apoptosis | 216 (0.9%) (NaN%) | ko04210 |
| 81 | Glutamatergic synapse | 215 (0.9%) (NaN%) | ko04724 |
| 82 | Gastric acid secretion | 212 (0.89%) (NaN%) | ko04971 |
| 83 | Progesterone-mediated oocyte maturation | 211 (0.88%) (NaN%) | ko04914 |
| 84 | Chagas disease (American trypanosomiasis) | 208 (0.87%) (NaN%) | ko05142 |
| 85 | Amyotrophic lateral sclerosis (ALS) | 206 (0.86%) (NaN%) | ko05014 |
| 86 | Toll-like receptor signaling pathway | 203 (0.85%) (NaN%) | ko04620 |
| 87 | Arrhythmogenic right ventricular cardiomyopathy (ARVC) | 202 (0.85%) (NaN%) | ko05412 |
| 88 | Chronic myeloid leukemia | 202 (0.85%) (NaN%) | ko05220 |
| 89 | Peroxisome | 201 (0.84%) (NaN%) | ko04146 |
| 90 | Drug metabolism - other enzymes | 199 (0.83%) (NaN%) | ko00983 |
| 91 | VEGF signaling pathway | 196 (0.82%) (NaN%) | ko04370 |
| 92 | Drug metabolism - cytochrome P450 | 196 (0.82%) (NaN%) | ko00982 |
| 93 | Cholinergic synapse | 194 (0.81%) (NaN%) | ko04725 |
| 94 | PPAR signaling pathway | 192 (0.8%) (NaN%) | ko03320 |
| 95 | p53 signaling pathway | 191 (0.8%) (NaN%) | ko04115 |
| 96 | Gap junction | 190 (0.8%) (NaN%) | ko04540 |
| 97 | Legionellosis | 190 (0.8%) (NaN%) | ko05134 |
| 98 | Steroid hormone biosynthesis | 190 (0.8%) (NaN%) | ko00140 |
| 99 | Pancreatic secretion | 189 (0.79%) (NaN%) | ko04972 |
| 100 | Renal cell carcinoma | 188 (0.79%) (NaN%) | ko05211 |
| 101 | Glycerophospholipid metabolism | 188 (0.79%) (NaN%) | ko00564 |
| 102 | Retinol metabolism | 187 (0.78%) (NaN%) | ko00830 |
| 103 | NOD-like receptor signaling pathway | 185 (0.77%) (NaN%) | ko04621 |
| 104 | Parkinson's disease | 180 (0.75%) (NaN%) | ko05012 |
| 105 | Neuroactive ligand-receptor interaction | 177 (0.74%) (NaN%) | ko04080 |
| 106 | Aminoacyl-tRNA biosynthesis | 175 (0.73%) (NaN%) | ko00970 |
| 107 | Fc epsilon RI signaling pathway | 173 (0.72%) (NaN%) | ko04664 |
| 108 | Basal transcription factors | 171 (0.72%) (NaN%) | ko03022 |
| 109 | Pancreatic cancer | 171 (0.72%) (NaN%) | ko05212 |
| 110 | Long-term potentiation | 170 (0.71%) (NaN%) | ko04720 |
| 111 | Acute myeloid leukemia | 168 (0.7%) (NaN%) | ko05221 |
| 112 | Colorectal cancer | 166 (0.69%) (NaN%) | ko05210 |
| 113 | Glioma | 165 (0.69%) (NaN%) | ko05214 |
| 114 | Oxidative phosphorylation | 161 (0.67%) (NaN%) | ko00190 |
| 115 | Dorso-ventral axis formation | 158 (0.66%) (NaN%) | ko04320 |
| 116 | Pertussis | 156 (0.65%) (NaN%) | ko05133 |
| 117 | Adipocytokine signaling pathway | 153 (0.64%) (NaN%) | ko04920 |
| 118 | mTOR signaling pathway | 151 (0.63%) (NaN%) | ko04150 |
| 119 | Retrograde endocannabinoid signaling | 151 (0.63%) (NaN%) | ko04723 |
| 120 | Leishmaniasis | 148 (0.62%) (NaN%) | ko05140 |
| 121 | Endometrial cancer | 148 (0.62%) (NaN%) | ko05213 |
| 122 | ABC transporters | 146 (0.61%) (NaN%) | ko02010 |
| 123 | Prion diseases | 145 (0.61%) (NaN%) | ko05020 |
| 124 | Systemic lupus erythematosus | 144 (0.6%) (NaN%) | ko05322 |
| 125 | Fanconi anemia pathway | 144 (0.6%) (NaN%) | ko03460 |
| 126 | Amino sugar and nucleotide sugar metabolism | 140 (0.59%) (NaN%) | ko00520 |
| 127 | Long-term depression | 139 (0.58%) (NaN%) | ko04730 |
| 128 | Melanoma | 138 (0.58%) (NaN%) | ko05218 |
| 129 | Arachidonic acid metabolism | 137 (0.57%) (NaN%) | ko00590 |
| 130 | Non-small cell lung cancer | 136 (0.57%) (NaN%) | ko05223 |
| 131 | Ribosome | 136 (0.57%) (NaN%) | ko03010 |
| 132 | Antigen processing and presentation | 136 (0.57%) (NaN%) | ko04612 |
| 133 | Morphine addiction | 134 (0.56%) (NaN%) | ko05032 |
| 134 | Synaptic vesicle cycle | 130 (0.54%) (NaN%) | ko04721 |
| 135 | Staphylococcus aureus infection | 130 (0.54%) (NaN%) | ko05150 |
| 136 | Rheumatoid arthritis | 129 (0.54%) (NaN%) | ko05323 |
| 137 | RIG-I-like receptor signaling pathway | 129 (0.54%) (NaN%) | ko04622 |
| 138 | Epithelial cell signaling in Helicobacter pylori infection | 129 (0.54%) (NaN%) | ko05120 |
| 139 | GABAergic synapse | 127 (0.53%) (NaN%) | ko04727 |
| 140 | Amphetamine addiction | 123 (0.51%) (NaN%) | ko05031 |
| 141 | Fructose and mannose metabolism | 123 (0.51%) (NaN%) | ko00051 |
| 142 | Linoleic acid metabolism | 121 (0.51%) (NaN%) | ko00591 |
| 143 | Cytosolic DNA-sensing pathway | 118 (0.49%) (NaN%) | ko04623 |
| 144 | Basal cell carcinoma | 118 (0.49%) (NaN%) | ko05217 |
| 145 | Arginine and proline metabolism | 117 (0.49%) (NaN%) | ko00330 |
| 146 | Bladder cancer | 115 (0.48%) (NaN%) | ko05219 |
| 147 | Base excision repair | 112 (0.47%) (NaN%) | ko03410 |
| 148 | Glycolysis / Gluconeogenesis | 112 (0.47%) (NaN%) | ko00010 |
| 149 | Primary immunodeficiency | 109 (0.46%) (NaN%) | ko05340 |
| 150 | Starch and sucrose metabolism | 109 (0.46%) (NaN%) | ko00500 |
| 151 | Glycerolipid metabolism | 109 (0.46%) (NaN%) | ko00561 |
| 152 | Endocrine and other factor-regulated calcium reabsorption | 108 (0.45%) (NaN%) | ko04961 |
| 153 | Sphingolipid metabolism | 108 (0.45%) (NaN%) | ko00600 |
| 154 | RNA polymerase | 106 (0.44%) (NaN%) | ko03020 |
| 155 | Glutathione metabolism | 105 (0.44%) (NaN%) | ko00480 |
| 156 | Vasopressin-regulated water reabsorption | 104 (0.44%) (NaN%) | ko04962 |
| 157 | Malaria | 101 (0.42%) (NaN%) | ko05144 |
| 158 | Fatty acid metabolism | 101 (0.42%) (NaN%) | ko00071 |
| 159 | Valine, leucine and isoleucine degradation | 101 (0.42%) (NaN%) | ko00280 |
| 160 | Hedgehog signaling pathway | 100 (0.42%) (NaN%) | ko04340 |
| 161 | Type II diabetes mellitus | 95 (0.4%) (NaN%) | ko04930 |
| 162 | Mineral absorption | 94 (0.39%) (NaN%) | ko04978 |
| 163 | Carbohydrate digestion and absorption | 94 (0.39%) (NaN%) | ko04973 |
| 164 | African trypanosomiasis | 93 (0.39%) (NaN%) | ko05143 |
| 165 | Other types of O-glycan biosynthesis | 91 (0.38%) (NaN%) | ko00514 |
| 166 | Cysteine and methionine metabolism | 87 (0.36%) (NaN%) | ko00270 |
| 167 | Nucleotide excision repair | 86 (0.36%) (NaN%) | ko03420 |
| 168 | Glycine, serine and threonine metabolism | 85 (0.36%) (NaN%) | ko00260 |
| 169 | Galactose metabolism | 84 (0.35%) (NaN%) | ko00052 |
| 170 | Pyruvate metabolism | 84 (0.35%) (NaN%) | ko00620 |
| 171 | Tryptophan metabolism | 81 (0.34%) (NaN%) | ko00380 |
| 172 | Aldosterone-regulated sodium reabsorption | 81 (0.34%) (NaN%) | ko04960 |
| 173 | N-Glycan biosynthesis | 80 (0.33%) (NaN%) | ko00510 |
| 174 | Nicotinate and nicotinamide metabolism | 80 (0.33%) (NaN%) | ko00760 |
| 175 | Porphyrin and chlorophyll metabolism | 78 (0.33%) (NaN%) | ko00860 |
| 176 | Cocaine addiction | 78 (0.33%) (NaN%) | ko05030 |
| 177 | Thyroid cancer | 78 (0.33%) (NaN%) | ko05216 |
| 178 | Fat digestion and absorption | 75 (0.31%) (NaN%) | ko04975 |
| 179 | SNARE interactions in vesicular transport | 75 (0.31%) (NaN%) | ko04130 |
| 180 | Ether lipid metabolism | 74 (0.31%) (NaN%) | ko00565 |
| 181 | Tyrosine metabolism | 71 (0.3%) (NaN%) | ko00350 |
| 182 | Phototransduction - fly | 70 (0.29%) (NaN%) | ko04745 |
| 183 | Other glycan degradation | 70 (0.29%) (NaN%) | ko00511 |
| 184 | Olfactory transduction | 69 (0.29%) (NaN%) | ko04740 |
| 185 | Autoimmune thyroid disease | 66 (0.28%) (NaN%) | ko05320 |
| 186 | Propanoate metabolism | 64 (0.27%) (NaN%) | ko00640 |
| 187 | Pentose and glucuronate interconversions | 64 (0.27%) (NaN%) | ko00040 |
| 188 | Butanoate metabolism | 64 (0.27%) (NaN%) | ko00650 |
| 189 | Vitamin digestion and absorption | 63 (0.26%) (NaN%) | ko04977 |
| 190 | DNA replication | 62 (0.26%) (NaN%) | ko03030 |
| 191 | Citrate cycle (TCA cycle) | 62 (0.26%) (NaN%) | ko00020 |
| 192 | Allograft rejection | 60 (0.25%) (NaN%) | ko05330 |
| 193 | Taste transduction | 58 (0.24%) (NaN%) | ko04742 |
| 194 | Glycosylphosphatidylinositol(GPI)-anchor biosynthesis | 57 (0.24%) (NaN%) | ko00563 |
| 195 | Glycosaminoglycan degradation | 56 (0.23%) (NaN%) | ko00531 |
| 196 | Histidine metabolism | 56 (0.23%) (NaN%) | ko00340 |
| 197 | Homologous recombination | 55 (0.23%) (NaN%) | ko03440 |
| 198 | Type I diabetes mellitus | 55 (0.23%) (NaN%) | ko04940 |
| 199 | Ascorbate and aldarate metabolism | 55 (0.23%) (NaN%) | ko00053 |
| 200 | Alanine, aspartate and glutamate metabolism | 54 (0.23%) (NaN%) | ko00250 |
| 201 | Intestinal immune network for IgA production | 54 (0.23%) (NaN%) | ko04672 |
| 202 | Circadian rhythm - mammal | 53 (0.22%) (NaN%) | ko04710 |
| 203 | beta-Alanine metabolism | 53 (0.22%) (NaN%) | ko00410 |
| 204 | Primary bile acid biosynthesis | 52 (0.22%) (NaN%) | ko00120 |
| 205 | Collecting duct acid secretion | 52 (0.22%) (NaN%) | ko04966 |
| 206 | Phenylalanine metabolism | 52 (0.22%) (NaN%) | ko00360 |
| 207 | Pentose phosphate pathway | 52 (0.22%) (NaN%) | ko00030 |
| 208 | Terpenoid backbone biosynthesis | 50 (0.21%) (NaN%) | ko00900 |
| 209 | Graft-versus-host disease | 50 (0.21%) (NaN%) | ko05332 |
| 210 | Proximal tubule bicarbonate reclamation | 50 (0.21%) (NaN%) | ko04964 |
| 211 | Pantothenate and CoA biosynthesis | 49 (0.21%) (NaN%) | ko00770 |
| 212 | Glycosaminoglycan biosynthesis - chondroitin sulfate | 49 (0.21%) (NaN%) | ko00532 |
| 213 | Glycosphingolipid biosynthesis - ganglio series | 48 (0.2%) (NaN%) | ko00604 |
| 214 | Mismatch repair | 48 (0.2%) (NaN%) | ko03430 |
| 215 | Non-homologous end-joining | 48 (0.2%) (NaN%) | ko03450 |
| 216 | Proteasome | 47 (0.2%) (NaN%) | ko03050 |
| 217 | MAPK signaling pathway - fly | 45 (0.19%) (NaN%) | ko04013 |
| 218 | Biosynthesis of unsaturated fatty acids | 45 (0.19%) (NaN%) | ko01040 |
| 219 | Glycosaminoglycan biosynthesis - heparan sulfate | 44 (0.18%) (NaN%) | ko00534 |
| 220 | Fatty acid elongation | 44 (0.18%) (NaN%) | ko00062 |
| 221 | Regulation of autophagy | 42 (0.18%) (NaN%) | ko04140 |
| 222 | Glyoxylate and dicarboxylate metabolism | 41 (0.17%) (NaN%) | ko00630 |
| 223 | Protein export | 39 (0.16%) (NaN%) | ko03060 |
| 224 | Maturity onset diabetes of the young | 38 (0.16%) (NaN%) | ko04950 |
| 225 | Selenocompound metabolism | 37 (0.15%) (NaN%) | ko00450 |
| 226 | Phototransduction | 37 (0.15%) (NaN%) | ko04744 |
| 227 | Steroid biosynthesis | 36 (0.15%) (NaN%) | ko00100 |
| 228 | alpha-Linolenic acid metabolism | 35 (0.15%) (NaN%) | ko00592 |
| 229 | Sulfur metabolism | 34 (0.14%) (NaN%) | ko00920 |
| 230 | Mucin type O-Glycan biosynthesis | 34 (0.14%) (NaN%) | ko00512 |
| 231 | One carbon pool by folate | 32 (0.13%) (NaN%) | ko00670 |
| 232 | Circadian rhythm - fly | 30 (0.13%) (NaN%) | ko04711 |
| 233 | Ubiquinone and other terpenoid-quinone biosynthesis | 27 (0.11%) (NaN%) | ko00130 |
| 234 | Asthma | 26 (0.11%) (NaN%) | ko05310 |
| 235 | Glycosphingolipid biosynthesis - lacto and neolacto series | 25 (0.1%) (NaN%) | ko00601 |
| 236 | Renin-angiotensin system | 25 (0.1%) (NaN%) | ko04614 |
| 237 | Sulfur relay system | 24 (0.1%) (NaN%) | ko04122 |
| 238 | Valine, leucine and isoleucine biosynthesis | 23 (0.1%) (NaN%) | ko00290 |
| 239 | Fatty acid biosynthesis | 19 (0.08%) (NaN%) | ko00061 |
| 240 | Glycosaminoglycan biosynthesis - keratan sulfate | 19 (0.08%) (NaN%) | ko00533 |
| 241 | Caffeine metabolism | 19 (0.08%) (NaN%) | ko00232 |
| 242 | Riboflavin metabolism | 18 (0.08%) (NaN%) | ko00740 |
| 243 | Folate biosynthesis | 18 (0.08%) (NaN%) | ko00790 |
| 244 | Phenylalanine, tyrosine and tryptophan biosynthesis | 15 (0.06%) (NaN%) | ko00400 |
| 245 | Butirosin and neomycin biosynthesis | 15 (0.06%) (NaN%) | ko00524 |
| 246 | Synthesis and degradation of ketone bodies | 15 (0.06%) (NaN%) | ko00072 |
| 247 | Lipoic acid metabolism | 13 (0.05%) (NaN%) | ko00785 |
| 248 | Nicotine addiction | 13 (0.05%) (NaN%) | ko05033 |
| 249 | Taurine and hypotaurine metabolism | 13 (0.05%) (NaN%) | ko00430 |
| 250 | Glycosphingolipid biosynthesis - globo series | 10 (0.04%) (NaN%) | ko00603 |
| 251 | Vitamin B6 metabolism | 9 (0.04%) (NaN%) | ko00750 |
| 252 | D-Arginine and D-ornithine metabolism | 8 (0.03%) (NaN%) | ko00472 |
| 253 | Thiamine metabolism | 8 (0.03%) (NaN%) | ko00730 |
| 254 | Cyanoamino acid metabolism | 7 (0.03%) (NaN%) | ko00460 |
| 255 | D-Glutamine and D-glutamate metabolism | 6 (0.03%) (NaN%) | ko00471 |
| 256 | Polyketide sugar unit biosynthesis | 6 (0.03%) (NaN%) | ko00523 |
| 257 | Biotin metabolism | 4 (0.02%) (NaN%) | ko00780 |
| 258 | Lysine biosynthesis | 2 (0.01%) (NaN%) | ko00300 |

| # | Pathway | Differentially expressed genes |
| --- | --- | --- |
| 1 | Metabolic pathways (no map in kegg database) | CL1072.Contig1\_Mf\_liverA, CL1072.Contig2\_Mf\_liverA, CL1072.Contig3\_Mf\_liverA, CL1072.Contig4\_Mf\_liverA, CL1076.Contig1\_Mf\_liverA, CL1076.Contig4\_Mf\_liverA, CL1076.Contig5\_Mf\_liverA, CL1076.Contig6\_Mf\_liverA, CL1086.Contig1\_Mf\_liverA, CL1086.Contig2\_Mf\_liverA, CL1086.Contig3\_Mf\_liverA, CL109.Contig1\_Mf\_liverA, CL109.Contig2\_Mf\_liverA, CL1093.Contig1\_Mf\_liverA, CL1093.Contig2\_Mf\_liverA, CL1095.Contig1\_Mf\_liverA, CL1096.Contig1\_Mf\_liverA, CL1096.Contig2\_Mf\_liverA, CL1109.Contig1\_Mf\_liverA, CL1109.Contig2\_Mf\_liverA, CL1109.Contig3\_Mf\_liverA, CL1132.Contig1\_Mf\_liverA, CL1132.Contig2\_Mf\_liverA, CL1133.Contig1\_Mf\_liverA, CL1133.Contig2\_Mf\_liverA, CL1133.Contig3\_Mf\_liverA, CL1133.Contig4\_Mf\_liverA, CL1133.Contig5\_Mf\_liverA, CL1133.Contig6\_Mf\_liverA, CL114.Contig1\_Mf\_liverA, CL114.Contig2\_Mf\_liverA, CL1140.Contig1\_Mf\_liverA, CL1140.Contig2\_Mf\_liverA, CL1140.Contig3\_Mf\_liverA, CL1140.Contig4\_Mf\_liverA, CL1140.Contig5\_Mf\_liverA, CL1140.Contig6\_Mf\_liverA, CL1140.Contig7\_Mf\_liverA, CL1146.Contig1\_Mf\_liverA, CL1146.Contig2\_Mf\_liverA, CL1146.Contig3\_Mf\_liverA, CL1146.Contig4\_Mf\_liverA, CL1160.Contig1\_Mf\_liverA, CL1160.Contig2\_Mf\_liverA, CL1160.Contig3\_Mf\_liverA, CL1160.Contig4\_Mf\_liverA, CL117.Contig3\_Mf\_liverA, CL117.Contig4\_Mf\_liverA, CL1176.Contig1\_Mf\_liverA, CL1179.Contig1\_Mf\_liverA, CL1179.Contig2\_Mf\_liverA, CL1179.Contig3\_Mf\_liverA, CL1179.Contig4\_Mf\_liverA, CL1179.Contig5\_Mf\_liverA, CL1179.Contig6\_Mf\_liverA, CL1192.Contig1\_Mf\_liverA, CL1219.Contig1\_Mf\_liverA, CL1219.Contig2\_Mf\_liverA, CL1219.Contig3\_Mf\_liverA, CL1230.Contig10\_Mf\_liverA, CL1230.Contig11\_Mf\_liverA, CL1230.Contig1\_Mf\_liverA, CL1230.Contig2\_Mf\_liverA, CL1230.Contig3\_Mf\_liverA, CL1230.Contig4\_Mf\_liverA, CL1230.Contig5\_Mf\_liverA, CL1230.Contig6\_Mf\_liverA, CL1230.Contig7\_Mf\_liverA, CL1230.Contig8\_Mf\_liverA, CL1230.Contig9\_Mf\_liverA, CL1242.Contig1\_Mf\_liverA, CL1242.Contig2\_Mf\_liverA, CL125.Contig1\_Mf\_liverA, CL125.Contig2\_Mf\_liverA, CL1252.Contig1\_Mf\_liverA, CL1263.Contig1\_Mf\_liverA, CL1263.Contig2\_Mf\_liverA, CL1271.Contig1\_Mf\_liverA, CL1273.Contig1\_Mf\_liverA, CL1273.Contig3\_Mf\_liverA, CL1281.Contig1\_Mf\_liverA, CL1281.Contig2\_Mf\_liverA, CL132.Contig1\_Mf\_liverA, CL132.Contig2\_Mf\_liverA, CL132.Contig3\_Mf\_liverA, CL1323.Contig1\_Mf\_liverA, CL1323.Contig2\_Mf\_liverA, CL1323.Contig3\_Mf\_liverA, CL1389.Contig1\_Mf\_liverA, CL1389.Contig2\_Mf\_liverA, CL1389.Contig3\_Mf\_liverA, CL1389.Contig4\_Mf\_liverA, CL1389.Contig5\_Mf\_liverA, CL1389.Contig6\_Mf\_liverA, CL1389.Contig7\_Mf\_liverA, CL1389.Contig8\_Mf\_liverA, CL1416.Contig1\_Mf\_liverA, CL1416.Contig2\_Mf\_liverA, CL1449.Contig1\_Mf\_liverA, CL1449.Contig2\_Mf\_liverA, CL1449.Contig3\_Mf\_liverA, CL1449.Contig4\_Mf\_liverA, CL1449.Contig5\_Mf\_liverA, CL147.Contig1\_Mf\_liverA, CL1480.Contig1\_Mf\_liverA, CL1480.Contig2\_Mf\_liverA, CL1480.Contig3\_Mf\_liverA, CL1480.Contig4\_Mf\_liverA, CL1485.Contig1\_Mf\_liverA, CL1485.Contig2\_Mf\_liverA, CL1485.Contig3\_Mf\_liverA, CL149.Contig4\_Mf\_liverA, CL1496.Contig1\_Mf\_liverA, CL1496.Contig2\_Mf\_liverA, CL1500.Contig1\_Mf\_liverA, CL1500.Contig2\_Mf\_liverA, CL1502.Contig1\_Mf\_liverA, CL1502.Contig2\_Mf\_liverA, CL1502.Contig3\_Mf\_liverA, CL1502.Contig4\_Mf\_liverA, CL1506.Contig1\_Mf\_liverA, CL1506.Contig2\_Mf\_liverA, CL1506.Contig3\_Mf\_liverA, CL151.Contig1\_Mf\_liverA, CL1530.Contig1\_Mf\_liverA, CL1530.Contig2\_Mf\_liverA, CL1530.Contig3\_Mf\_liverA, CL1530.Contig4\_Mf\_liverA, CL1542.Contig1\_Mf\_liverA, CL1542.Contig2\_Mf\_liverA, CL1542.Contig3\_Mf\_liverA, CL1542.Contig4\_Mf\_liverA, CL1545.Contig1\_Mf\_liverA, CL1545.Contig2\_Mf\_liverA, CL1545.Contig3\_Mf\_liverA, CL1601.Contig1\_Mf\_liverA, CL1601.Contig2\_Mf\_liverA, CL1601.Contig3\_Mf\_liverA, CL1623.Contig1\_Mf\_liverA, CL1623.Contig2\_Mf\_liverA, CL1636.Contig1\_Mf\_liverA, CL1643.Contig1\_Mf\_liverA, CL1643.Contig2\_Mf\_liverA, CL1643.Contig3\_Mf\_liverA, CL1643.Contig4\_Mf\_liverA, CL1643.Contig5\_Mf\_liverA, CL1643.Contig6\_Mf\_liverA, CL1643.Contig7\_Mf\_liverA, CL1643.Contig8\_Mf\_liverA, CL1644.Contig1\_Mf\_liverA, CL1651.Contig1\_Mf\_liverA, CL1651.Contig2\_Mf\_liverA, CL1667.Contig1\_Mf\_liverA, CL1684.Contig10\_Mf\_liverA, CL1684.Contig11\_Mf\_liverA, CL1684.Contig1\_Mf\_liverA, CL1684.Contig2\_Mf\_liverA, CL1684.Contig3\_Mf\_liverA, CL1684.Contig4\_Mf\_liverA, CL1684.Contig5\_Mf\_liverA, CL1684.Contig6\_Mf\_liverA, CL1684.Contig8\_Mf\_liverA, CL1684.Contig9\_Mf\_liverA, CL1685.Contig9\_Mf\_liverA, CL1688.Contig1\_Mf\_liverA, CL1688.Contig2\_Mf\_liverA, CL1688.Contig3\_Mf\_liverA, CL1688.Contig4\_Mf\_liverA, CL1695.Contig1\_Mf\_liverA, CL1695.Contig2\_Mf\_liverA, CL1695.Contig3\_Mf\_liverA, CL1695.Contig4\_Mf\_liverA, CL1695.Contig5\_Mf\_liverA, CL1695.Contig6\_Mf\_liverA, CL1711.Contig1\_Mf\_liverA, CL1711.Contig2\_Mf\_liverA, CL1711.Contig3\_Mf\_liverA, CL1711.Contig4\_Mf\_liverA, CL1715.Contig1\_Mf\_liverA, CL1715.Contig2\_Mf\_liverA, CL1734.Contig1\_Mf\_liverA, CL1734.Contig2\_Mf\_liverA, CL1734.Contig3\_Mf\_liverA, CL1734.Contig4\_Mf\_liverA, CL1736.Contig1\_Mf\_liverA, CL1736.Contig2\_Mf\_liverA, CL1752.Contig1\_Mf\_liverA, CL1752.Contig2\_Mf\_liverA, CL1752.Contig3\_Mf\_liverA, CL1752.Contig4\_Mf\_liverA, CL1754.Contig1\_Mf\_liverA, CL1754.Contig2\_Mf\_liverA, CL1777.Contig1\_Mf\_liverA, CL1777.Contig2\_Mf\_liverA, CL178.Contig1\_Mf\_liverA, CL178.Contig2\_Mf\_liverA, CL1798.Contig1\_Mf\_liverA, CL1798.Contig2\_Mf\_liverA, CL1799.Contig1\_Mf\_liverA, CL1799.Contig2\_Mf\_liverA, CL1808.Contig1\_Mf\_liverA, CL1808.Contig2\_Mf\_liverA, CL1808.Contig3\_Mf\_liverA, CL1808.Contig4\_Mf\_liverA, CL1808.Contig5\_Mf\_liverA, CL181.Contig1\_Mf\_liverA, CL181.Contig2\_Mf\_liverA, CL181.Contig3\_Mf\_liverA, CL181.Contig4\_Mf\_liverA, CL1814.Contig1\_Mf\_liverA, CL1853.Contig1\_Mf\_liverA, CL1853.Contig2\_Mf\_liverA, CL1856.Contig1\_Mf\_liverA, CL1856.Contig2\_Mf\_liverA, CL1857.Contig1\_Mf\_liverA, CL1857.Contig2\_Mf\_liverA, CL1860.Contig10\_Mf\_liverA, CL1860.Contig1\_Mf\_liverA, CL1860.Contig6\_Mf\_liverA, CL1860.Contig7\_Mf\_liverA, CL1870.Contig1\_Mf\_liverA, CL1885.Contig1\_Mf\_liverA, CL1885.Contig2\_Mf\_liverA, CL190.Contig1\_Mf\_liverA, CL190.Contig2\_Mf\_liverA, CL190.Contig3\_Mf\_liverA, CL1916.Contig10\_Mf\_liverA, CL1916.Contig11\_Mf\_liverA, CL1916.Contig12\_Mf\_liverA, CL1916.Contig1\_Mf\_liverA, CL1916.Contig2\_Mf\_liverA, CL1916.Contig3\_Mf\_liverA, CL1916.Contig4\_Mf\_liverA, CL1916.Contig5\_Mf\_liverA, CL1916.Contig6\_Mf\_liverA, CL1916.Contig7\_Mf\_liverA, CL1916.Contig8\_Mf\_liverA, CL1916.Contig9\_Mf\_liverA, CL1923.Contig1\_Mf\_liverA, CL1923.Contig2\_Mf\_liverA, CL1943.Contig1\_Mf\_liverA, CL1943.Contig2\_Mf\_liverA, CL1944.Contig3\_Mf\_liverA, CL1979.Contig1\_Mf\_liverA, CL1979.Contig2\_Mf\_liverA, CL1979.Contig3\_Mf\_liverA, CL1979.Contig4\_Mf\_liverA, CL1979.Contig5\_Mf\_liverA, CL1979.Contig6\_Mf\_liverA, CL1979.Contig7\_Mf\_liverA, CL1979.Contig8\_Mf\_liverA, CL1979.Contig9\_Mf\_liverA, CL1988.Contig1\_Mf\_liverA, CL1988.Contig2\_Mf\_liverA, CL1988.Contig3\_Mf\_liverA, CL2.Contig1\_Mf\_liverA, CL2.Contig2\_Mf\_liverA, CL2.Contig3\_Mf\_liverA, CL2.Contig4\_Mf\_liverA, CL2.Contig5\_Mf\_liverA, CL2.Contig7\_Mf\_liverA, CL2022.Contig1\_Mf\_liverA, CL2022.Contig2\_Mf\_liverA, CL2027.Contig2\_Mf\_liverA, CL2047.Contig1\_Mf\_liverA, CL2047.Contig2\_Mf\_liverA, CL2047.Contig3\_Mf\_liverA, CL2047.Contig4\_Mf\_liverA, CL2047.Contig5\_Mf\_liverA, CL2047.Contig6\_Mf\_liverA, CL2051.Contig1\_Mf\_liverA, CL2051.Contig2\_Mf\_liverA, CL2057.Contig1\_Mf\_liverA, CL2071.Contig1\_Mf\_liverA, CL2071.Contig2\_Mf\_liverA, CL209.Contig1\_Mf\_liverA, CL209.Contig2\_Mf\_liverA, CL2092.Contig1\_Mf\_liverA, CL2092.Contig2\_Mf\_liverA, CL2092.Contig3\_Mf\_liverA, CL2097.Contig1\_Mf\_liverA, CL2097.Contig2\_Mf\_liverA, CL2097.Contig3\_Mf\_liverA, CL2099.Contig1\_Mf\_liverA, CL2099.Contig2\_Mf\_liverA, CL2099.Contig3\_Mf\_liverA, CL2099.Contig4\_Mf\_liverA, CL2099.Contig5\_Mf\_liverA, CL2099.Contig6\_Mf\_liverA, CL2103.Contig1\_Mf\_liverA, CL2111.Contig1\_Mf\_liverA, CL2111.Contig2\_Mf\_liverA, CL2158.Contig1\_Mf\_liverA, CL2158.Contig2\_Mf\_liverA, CL220.Contig1\_Mf\_liverA, CL220.Contig2\_Mf\_liverA, CL2214.Contig1\_Mf\_liverA, CL2214.Contig2\_Mf\_liverA, CL2214.Contig3\_Mf\_liverA, CL2214.Contig4\_Mf\_liverA, CL2226.Contig1\_Mf\_liverA, CL2226.Contig2\_Mf\_liverA, CL2244.Contig1\_Mf\_liverA, CL2250.Contig1\_Mf\_liverA, CL2250.Contig2\_Mf\_liverA, CL2251.Contig1\_Mf\_liverA, CL2251.Contig2\_Mf\_liverA, CL2261.Contig1\_Mf\_liverA, CL2261.Contig2\_Mf\_liverA, CL2270.Contig3\_Mf\_liverA, CL2270.Contig4\_Mf\_liverA, CL2287.Contig1\_Mf\_liverA, CL2287.Contig2\_Mf\_liverA, CL2287.Contig3\_Mf\_liverA, CL2290.Contig1\_Mf\_liverA, CL2290.Contig2\_Mf\_liverA, CL2294.Contig1\_Mf\_liverA, CL2302.Contig1\_Mf\_liverA, CL2302.Contig2\_Mf\_liverA, CL2327.Contig1\_Mf\_liverA, CL2327.Contig2\_Mf\_liverA, CL2351.Contig1\_Mf\_liverA, CL2351.Contig2\_Mf\_liverA, CL2367.Contig1\_Mf\_liverA, CL2367.Contig2\_Mf\_liverA, CL2367.Contig3\_Mf\_liverA, CL2389.Contig1\_Mf\_liverA, CL2389.Contig2\_Mf\_liverA, CL241.Contig1\_Mf\_liverA, CL241.Contig2\_Mf\_liverA, CL2416.Contig1\_Mf\_liverA, CL2416.Contig2\_Mf\_liverA, CL2443.Contig1\_Mf\_liverA, CL2443.Contig2\_Mf\_liverA, CL2443.Contig3\_Mf\_liverA, CL2443.Contig4\_Mf\_liverA, CL2452.Contig1\_Mf\_liverA, CL2452.Contig2\_Mf\_liverA, CL2452.Contig3\_Mf\_liverA, CL2452.Contig4\_Mf\_liverA, CL2459.Contig1\_Mf\_liverA, CL2459.Contig2\_Mf\_liverA, CL2459.Contig3\_Mf\_liverA, CL2459.Contig4\_Mf\_liverA, CL2493.Contig1\_Mf\_liverA, CL2517.Contig1\_Mf\_liverA, CL2517.Contig2\_Mf\_liverA, CL2535.Contig1\_Mf\_liverA, CL2535.Contig2\_Mf\_liverA, CL2543.Contig1\_Mf\_liverA, CL2543.Contig2\_Mf\_liverA, CL2543.Contig3\_Mf\_liverA, CL2543.Contig4\_Mf\_liverA, CL2578.Contig1\_Mf\_liverA, CL2623.Contig1\_Mf\_liverA, CL2623.Contig2\_Mf\_liverA, CL2623.Contig3\_Mf\_liverA, CL2623.Contig4\_Mf\_liverA, CL2623.Contig5\_Mf\_liverA, CL2623.Contig6\_Mf\_liverA, CL2623.Contig7\_Mf\_liverA, CL2625.Contig2\_Mf\_liverA, CL2682.Contig1\_Mf\_liverA, CL2682.Contig2\_Mf\_liverA, CL2682.Contig3\_Mf\_liverA, CL2682.Contig4\_Mf\_liverA, CL2702.Contig1\_Mf\_liverA, CL2702.Contig2\_Mf\_liverA, CL2711.Contig1\_Mf\_liverA, CL2711.Contig2\_Mf\_liverA, CL2711.Contig3\_Mf\_liverA, CL2711.Contig4\_Mf\_liverA, CL2742.Contig1\_Mf\_liverA, CL2748.Contig1\_Mf\_liverA, CL2748.Contig2\_Mf\_liverA, CL2759.Contig1\_Mf\_liverA, CL2759.Contig2\_Mf\_liverA, CL2763.Contig1\_Mf\_liverA, CL2763.Contig2\_Mf\_liverA, CL2763.Contig3\_Mf\_liverA, CL2791.Contig1\_Mf\_liverA, CL2791.Contig2\_Mf\_liverA, CL2794.Contig1\_Mf\_liverA, CL2794.Contig2\_Mf\_liverA, CL2797.Contig1\_Mf\_liverA, CL2797.Contig2\_Mf\_liverA, CL281.Contig2\_Mf\_liverA, CL281.Contig3\_Mf\_liverA, CL281.Contig5\_Mf\_liverA, CL281.Contig7\_Mf\_liverA, CL2831.Contig1\_Mf\_liverA, CL2831.Contig2\_Mf\_liverA, CL2840.Contig1\_Mf\_liverA, CL2840.Contig2\_Mf\_liverA, CL2861.Contig1\_Mf\_liverA, CL2872.Contig1\_Mf\_liverA, CL2872.Contig2\_Mf\_liverA, CL2874.Contig1\_Mf\_liverA, CL2874.Contig2\_Mf\_liverA, CL2874.Contig3\_Mf\_liverA, CL2874.Contig4\_Mf\_liverA, CL2893.Contig1\_Mf\_liverA, CL2893.Contig2\_Mf\_liverA, CL2949.Contig1\_Mf\_liverA, CL2949.Contig2\_Mf\_liverA, CL2959.Contig1\_Mf\_liverA, CL2959.Contig2\_Mf\_liverA, CL2964.Contig1\_Mf\_liverA, CL2964.Contig2\_Mf\_liverA, CL297.Contig3\_Mf\_liverA, CL297.Contig4\_Mf\_liverA, CL2971.Contig1\_Mf\_liverA, CL298.Contig3\_Mf\_liverA, CL2980.Contig1\_Mf\_liverA, CL2980.Contig2\_Mf\_liverA, CL2989.Contig1\_Mf\_liverA, CL2989.Contig2\_Mf\_liverA, CL3027.Contig1\_Mf\_liverA, CL3027.Contig2\_Mf\_liverA, CL303.Contig1\_Mf\_liverA, CL303.Contig2\_Mf\_liverA, CL3064.Contig1\_Mf\_liverA, CL3064.Contig2\_Mf\_liverA, CL3064.Contig3\_Mf\_liverA, CL3064.Contig4\_Mf\_liverA, CL3093.Contig1\_Mf\_liverA, CL3093.Contig2\_Mf\_liverA, CL3093.Contig3\_Mf\_liverA, CL3093.Contig4\_Mf\_liverA, CL3093.Contig5\_Mf\_liverA, CL310.Contig1\_Mf\_liverA, CL310.Contig2\_Mf\_liverA, CL3139.Contig1\_Mf\_liverA, CL3166.Contig1\_Mf\_liverA, CL3166.Contig2\_Mf\_liverA, CL3166.Contig3\_Mf\_liverA, CL3166.Contig4\_Mf\_liverA, CL3166.Contig5\_Mf\_liverA, CL3166.Contig6\_Mf\_liverA, CL3176.Contig1\_Mf\_liverA, CL3176.Contig2\_Mf\_liverA, CL3176.Contig3\_Mf\_liverA, CL3176.Contig4\_Mf\_liverA, CL3176.Contig5\_Mf\_liverA, CL3176.Contig6\_Mf\_liverA, CL3191.Contig2\_Mf\_liverA, CL3194.Contig1\_Mf\_liverA, CL3196.Contig1\_Mf\_liverA, CL3196.Contig2\_Mf\_liverA, CL320.Contig2\_Mf\_liverA, CL320.Contig3\_Mf\_liverA, CL320.Contig4\_Mf\_liverA, CL320.Contig5\_Mf\_liverA, CL3204.Contig1\_Mf\_liverA, CL3204.Contig2\_Mf\_liverA, CL3219.Contig1\_Mf\_liverA, CL3270.Contig1\_Mf\_liverA, CL3270.Contig2\_Mf\_liverA, CL3288.Contig1\_Mf\_liverA, CL3288.Contig2\_Mf\_liverA, CL3288.Contig3\_Mf\_liverA, CL3299.Contig1\_Mf\_liverA, CL3299.Contig2\_Mf\_liverA, CL330.Contig1\_Mf\_liverA, CL330.Contig2\_Mf\_liverA, CL3307.Contig1\_Mf\_liverA, CL3307.Contig2\_Mf\_liverA, CL3324.Contig1\_Mf\_liverA, CL3324.Contig2\_Mf\_liverA, CL3348.Contig1\_Mf\_liverA, CL3348.Contig2\_Mf\_liverA, CL3349.Contig1\_Mf\_liverA, CL3349.Contig2\_Mf\_liverA, CL3353.Contig1\_Mf\_liverA, CL3353.Contig2\_Mf\_liverA, CL3354.Contig1\_Mf\_liverA, CL3354.Contig2\_Mf\_liverA, CL3354.Contig3\_Mf\_liverA, CL3354.Contig4\_Mf\_liverA, CL336.Contig10\_Mf\_liverA, CL336.Contig11\_Mf\_liverA, CL336.Contig12\_Mf\_liverA, CL336.Contig13\_Mf\_liverA, CL336.Contig14\_Mf\_liverA, CL336.Contig15\_Mf\_liverA, CL336.Contig16\_Mf\_liverA, CL336.Contig17\_Mf\_liverA, CL336.Contig1\_Mf\_liverA, CL336.Contig2\_Mf\_liverA, CL336.Contig3\_Mf\_liverA, CL336.Contig4\_Mf\_liverA, CL336.Contig6\_Mf\_liverA, CL336.Contig7\_Mf\_liverA, CL336.Contig8\_Mf\_liverA, CL336.Contig9\_Mf\_liverA, CL3364.Contig1\_Mf\_liverA, CL3364.Contig2\_Mf\_liverA, CL3390.Contig1\_Mf\_liverA, CL3390.Contig2\_Mf\_liverA, CL3391.Contig1\_Mf\_liverA, CL3391.Contig2\_Mf\_liverA, CL3392.Contig1\_Mf\_liverA, CL3392.Contig2\_Mf\_liverA, CL3392.Contig3\_Mf\_liverA, CL3392.Contig4\_Mf\_liverA, CL3424.Contig1\_Mf\_liverA, CL3451.Contig1\_Mf\_liverA, CL3451.Contig2\_Mf\_liverA, CL3479.Contig1\_Mf\_liverA, CL3480.Contig1\_Mf\_liverA, CL3480.Contig2\_Mf\_liverA, CL3487.Contig1\_Mf\_liverA, CL3487.Contig2\_Mf\_liverA, CL3489.Contig1\_Mf\_liverA, CL3489.Contig2\_Mf\_liverA, CL3490.Contig1\_Mf\_liverA, CL3490.Contig2\_Mf\_liverA, CL3498.Contig1\_Mf\_liverA, CL3498.Contig2\_Mf\_liverA, CL3504.Contig1\_Mf\_liverA, CL3504.Contig2\_Mf\_liverA, CL3508.Contig1\_Mf\_liverA, CL3508.Contig2\_Mf\_liverA, CL3522.Contig1\_Mf\_liverA, CL3522.Contig2\_Mf\_liverA, CL355.Contig2\_Mf\_liverA, CL355.Contig3\_Mf\_liverA, CL3550.Contig1\_Mf\_liverA, CL3550.Contig2\_Mf\_liverA, CL356.Contig1\_Mf\_liverA, CL3564.Contig1\_Mf\_liverA, CL3564.Contig2\_Mf\_liverA, CL3582.Contig1\_Mf\_liverA, CL3582.Contig2\_Mf\_liverA, CL3599.Contig1\_Mf\_liverA, CL3599.Contig2\_Mf\_liverA, CL3606.Contig1\_Mf\_liverA, CL3606.Contig2\_Mf\_liverA, CL362.Contig1\_Mf\_liverA, CL362.Contig2\_Mf\_liverA, CL362.Contig3\_Mf\_liverA, CL362.Contig4\_Mf\_liverA, CL362.Contig5\_Mf\_liverA, CL3627.Contig1\_Mf\_liverA, CL3627.Contig2\_Mf\_liverA, CL3635.Contig1\_Mf\_liverA, CL3635.Contig2\_Mf\_liverA, CL3635.Contig3\_Mf\_liverA, CL3635.Contig4\_Mf\_liverA, CL365.Contig10\_Mf\_liverA, CL365.Contig2\_Mf\_liverA, CL365.Contig3\_Mf\_liverA, CL365.Contig4\_Mf\_liverA, CL365.Contig6\_Mf\_liverA, CL3660.Contig1\_Mf\_liverA, CL3660.Contig2\_Mf\_liverA, CL3689.Contig1\_Mf\_liverA, CL3689.Contig2\_Mf\_liverA, CL3699.Contig1\_Mf\_liverA, CL3699.Contig2\_Mf\_liverA, CL3705.Contig1\_Mf\_liverA, CL3705.Contig2\_Mf\_liverA, CL3706.Contig1\_Mf\_liverA, CL3764.Contig1\_Mf\_liverA, CL3764.Contig2\_Mf\_liverA, CL3766.Contig1\_Mf\_liverA, CL377.Contig1\_Mf\_liverA, CL377.Contig2\_Mf\_liverA, CL3793.Contig1\_Mf\_liverA, CL3793.Contig2\_Mf\_liverA, CL3796.Contig1\_Mf\_liverA, CL3796.Contig2\_Mf\_liverA, CL380.Contig1\_Mf\_liverA, CL380.Contig2\_Mf\_liverA, CL380.Contig3\_Mf\_liverA, CL380.Contig4\_Mf\_liverA, CL380.Contig5\_Mf\_liverA, CL380.Contig6\_Mf\_liverA, CL380.Contig7\_Mf\_liverA, CL380.Contig8\_Mf\_liverA, CL3804.Contig1\_Mf\_liverA, CL3804.Contig2\_Mf\_liverA, CL3813.Contig1\_Mf\_liverA, CL3813.Contig2\_Mf\_liverA, CL383.Contig1\_Mf\_liverA, CL3832.Contig1\_Mf\_liverA, CL3832.Contig2\_Mf\_liverA, CL3849.Contig1\_Mf\_liverA, CL3849.Contig2\_Mf\_liverA, CL3849.Contig3\_Mf\_liverA, CL3853.Contig1\_Mf\_liverA, CL3853.Contig2\_Mf\_liverA, CL3868.Contig1\_Mf\_liverA, CL3868.Contig2\_Mf\_liverA, CL389.Contig1\_Mf\_liverA, CL389.Contig2\_Mf\_liverA, CL3891.Contig1\_Mf\_liverA, CL3891.Contig2\_Mf\_liverA, CL3905.Contig1\_Mf\_liverA, CL3905.Contig2\_Mf\_liverA, CL3941.Contig1\_Mf\_liverA, CL3960.Contig1\_Mf\_liverA, CL3960.Contig2\_Mf\_liverA, CL3975.Contig1\_Mf\_liverA, CL3978.Contig1\_Mf\_liverA, CL3978.Contig2\_Mf\_liverA, CL3980.Contig1\_Mf\_liverA, CL3980.Contig2\_Mf\_liverA, CL4007.Contig1\_Mf\_liverA, CL4023.Contig1\_Mf\_liverA, CL4023.Contig2\_Mf\_liverA, CL4038.Contig2\_Mf\_liverA, CL404.Contig10\_Mf\_liverA, CL404.Contig11\_Mf\_liverA, CL404.Contig12\_Mf\_liverA, CL404.Contig13\_Mf\_liverA, CL404.Contig14\_Mf\_liverA, CL404.Contig15\_Mf\_liverA, CL404.Contig16\_Mf\_liverA, CL404.Contig17\_Mf\_liverA, CL404.Contig18\_Mf\_liverA, CL404.Contig19\_Mf\_liverA, CL404.Contig1\_Mf\_liverA, CL404.Contig20\_Mf\_liverA, CL404.Contig21\_Mf\_liverA, CL404.Contig22\_Mf\_liverA, CL404.Contig23\_Mf\_liverA, CL404.Contig24\_Mf\_liverA, CL404.Contig25\_Mf\_liverA, CL404.Contig26\_Mf\_liverA, CL404.Contig27\_Mf\_liverA, CL404.Contig28\_Mf\_liverA, CL404.Contig2\_Mf\_liverA, CL404.Contig3\_Mf\_liverA, CL404.Contig4\_Mf\_liverA, CL404.Contig5\_Mf\_liverA, CL404.Contig6\_Mf\_liverA, CL404.Contig7\_Mf\_liverA, CL404.Contig8\_Mf\_liverA, CL404.Contig9\_Mf\_liverA, CL4058.Contig1\_Mf\_liverA, CL4058.Contig2\_Mf\_liverA, CL4090.Contig1\_Mf\_liverA, CL4090.Contig2\_Mf\_liverA, CL4090.Contig3\_Mf\_liverA, CL4090.Contig4\_Mf\_liverA, CL4092.Contig1\_Mf\_liverA, CL4092.Contig2\_Mf\_liverA, CL4101.Contig1\_Mf\_liverA, CL4101.Contig2\_Mf\_liverA, CL4117.Contig1\_Mf\_liverA, CL4117.Contig2\_Mf\_liverA, CL412.Contig1\_Mf\_liverA, CL412.Contig2\_Mf\_liverA, CL412.Contig3\_Mf\_liverA, CL4131.Contig1\_Mf\_liverA, CL4131.Contig2\_Mf\_liverA, CL4139.Contig1\_Mf\_liverA, CL4145.Contig1\_Mf\_liverA, CL4145.Contig2\_Mf\_liverA, CL415.Contig1\_Mf\_liverA, CL415.Contig2\_Mf\_liverA, CL4154.Contig1\_Mf\_liverA, CL4154.Contig2\_Mf\_liverA, CL4164.Contig1\_Mf\_liverA, CL4181.Contig1\_Mf\_liverA, CL4181.Contig2\_Mf\_liverA, CL4189.Contig1\_Mf\_liverA, CL4189.Contig2\_Mf\_liverA, CL4197.Contig1\_Mf\_liverA, CL4197.Contig2\_Mf\_liverA, CL4197.Contig3\_Mf\_liverA, CL4208.Contig1\_Mf\_liverA, CL4208.Contig2\_Mf\_liverA, CL4220.Contig1\_Mf\_liverA, CL4220.Contig2\_Mf\_liverA, CL4241.Contig1\_Mf\_liverA, CL4241.Contig2\_Mf\_liverA, CL4249.Contig1\_Mf\_liverA, CL4249.Contig2\_Mf\_liverA, CL4252.Contig1\_Mf\_liverA, CL4252.Contig2\_Mf\_liverA, CL4265.Contig2\_Mf\_liverA, CL427.Contig1\_Mf\_liverA, CL4279.Contig1\_Mf\_liverA, CL4279.Contig2\_Mf\_liverA, CL4295.Contig1\_Mf\_liverA, CL4317.Contig1\_Mf\_liverA, CL4328.Contig1\_Mf\_liverA, CL4328.Contig2\_Mf\_liverA, CL4328.Contig3\_Mf\_liverA, CL4335.Contig1\_Mf\_liverA, CL4335.Contig2\_Mf\_liverA, CL4351.Contig1\_Mf\_liverA, CL4351.Contig2\_Mf\_liverA, CL4369.Contig1\_Mf\_liverA, CL4369.Contig2\_Mf\_liverA, CL4372.Contig2\_Mf\_liverA, CL4379.Contig1\_Mf\_liverA, CL4379.Contig2\_Mf\_liverA, CL4382.Contig1\_Mf\_liverA, CL4382.Contig2\_Mf\_liverA, CL4390.Contig1\_Mf\_liverA, CL4390.Contig2\_Mf\_liverA, CL4390.Contig3\_Mf\_liverA, CL4424.Contig1\_Mf\_liverA, CL4424.Contig2\_Mf\_liverA, CL4435.Contig1\_Mf\_liverA, CL4435.Contig2\_Mf\_liverA, CL4435.Contig3\_Mf\_liverA, CL4438.Contig1\_Mf\_liverA, CL4438.Contig2\_Mf\_liverA, CL4447.Contig1\_Mf\_liverA, CL4447.Contig2\_Mf\_liverA, CL4447.Contig3\_Mf\_liverA, CL4447.Contig4\_Mf\_liverA, CL4450.Contig1\_Mf\_liverA, CL4487.Contig1\_Mf\_liverA, CL4487.Contig2\_Mf\_liverA, CL4490.Contig2\_Mf\_liverA, CL4492.Contig1\_Mf\_liverA, CL4492.Contig2\_Mf\_liverA, CL4503.Contig1\_Mf\_liverA, CL4503.Contig2\_Mf\_liverA, CL4503.Contig3\_Mf\_liverA, CL4508.Contig1\_Mf\_liverA, CL4509.Contig1\_Mf\_liverA, CL4509.Contig2\_Mf\_liverA, CL4514.Contig1\_Mf\_liverA, CL4526.Contig1\_Mf\_liverA, CL4526.Contig2\_Mf\_liverA, CL4541.Contig1\_Mf\_liverA, CL4541.Contig2\_Mf\_liverA, CL4550.Contig1\_Mf\_liverA, CL4550.Contig2\_Mf\_liverA, CL4550.Contig3\_Mf\_liverA, CL4554.Contig1\_Mf\_liverA, CL4554.Contig2\_Mf\_liverA, CL4554.Contig3\_Mf\_liverA, CL4563.Contig2\_Mf\_liverA, CL4593.Contig3\_Mf\_liverA, CL4633.Contig1\_Mf\_liverA, CL4633.Contig2\_Mf\_liverA, CL4659.Contig1\_Mf\_liverA, CL4659.Contig2\_Mf\_liverA, CL4659.Contig3\_Mf\_liverA, CL4661.Contig1\_Mf\_liverA, CL4661.Contig2\_Mf\_liverA, CL4672.Contig1\_Mf\_liverA, CL4672.Contig2\_Mf\_liverA, CL4672.Contig3\_Mf\_liverA, CL4678.Contig1\_Mf\_liverA, CL4698.Contig1\_Mf\_liverA, CL4698.Contig2\_Mf\_liverA, CL4700.Contig1\_Mf\_liverA, CL4700.Contig2\_Mf\_liverA, CL4711.Contig1\_Mf\_liverA, CL4711.Contig2\_Mf\_liverA, CL4721.Contig1\_Mf\_liverA, CL4721.Contig2\_Mf\_liverA, CL4753.Contig1\_Mf\_liverA, CL4764.Contig1\_Mf\_liverA, CL4764.Contig2\_Mf\_liverA, CL4787.Contig1\_Mf\_liverA, CL4787.Contig2\_Mf\_liverA, CL4787.Contig3\_Mf\_liverA, CL4812.Contig1\_Mf\_liverA, CL4812.Contig2\_Mf\_liverA, CL4816.Contig1\_Mf\_liverA, CL4816.Contig2\_Mf\_liverA, CL4816.Contig3\_Mf\_liverA, CL4866.Contig1\_Mf\_liverA, CL4866.Contig2\_Mf\_liverA, CL4870.Contig1\_Mf\_liverA, CL4878.Contig1\_Mf\_liverA, CL4878.Contig2\_Mf\_liverA, CL4890.Contig1\_Mf\_liverA, CL4890.Contig2\_Mf\_liverA, CL490.Contig1\_Mf\_liverA, CL490.Contig2\_Mf\_liverA, CL490.Contig3\_Mf\_liverA, CL490.Contig4\_Mf\_liverA, CL490.Contig5\_Mf\_liverA, CL490.Contig6\_Mf\_liverA, CL490.Contig7\_Mf\_liverA, CL490.Contig8\_Mf\_liverA, CL4905.Contig1\_Mf\_liverA, CL4912.Contig1\_Mf\_liverA, CL4912.Contig2\_Mf\_liverA, CL493.Contig1\_Mf\_liverA, CL493.Contig2\_Mf\_liverA, CL493.Contig3\_Mf\_liverA, CL493.Contig4\_Mf\_liverA, CL4946.Contig1\_Mf\_liverA, CL4951.Contig1\_Mf\_liverA, CL4951.Contig2\_Mf\_liverA, CL4955.Contig1\_Mf\_liverA, CL4955.Contig2\_Mf\_liverA, CL4974.Contig1\_Mf\_liverA, CL4974.Contig2\_Mf\_liverA, CL4984.Contig1\_Mf\_liverA, CL4984.Contig2\_Mf\_liverA, CL5001.Contig3\_Mf\_liverA, CL5019.Contig1\_Mf\_liverA, CL5019.Contig2\_Mf\_liverA, CL5024.Contig1\_Mf\_liverA, CL5024.Contig2\_Mf\_liverA, CL5031.Contig1\_Mf\_liverA, CL5078.Contig1\_Mf\_liverA, CL5078.Contig2\_Mf\_liverA, CL5099.Contig1\_Mf\_liverA, CL5099.Contig2\_Mf\_liverA, CL5107.Contig1\_Mf\_liverA, CL5130.Contig1\_Mf\_liverA, CL5130.Contig2\_Mf\_liverA, CL5131.Contig1\_Mf\_liverA, CL5131.Contig2\_Mf\_liverA, CL5134.Contig1\_Mf\_liverA, CL5134.Contig2\_Mf\_liverA, CL5134.Contig3\_Mf\_liverA, CL5152.Contig1\_Mf\_liverA, CL5152.Contig2\_Mf\_liverA, CL5152.Contig3\_Mf\_liverA, CL5162.Contig1\_Mf\_liverA, CL5162.Contig2\_Mf\_liverA, CL517.Contig1\_Mf\_liverA, CL517.Contig2\_Mf\_liverA, CL5176.Contig1\_Mf\_liverA, CL5176.Contig2\_Mf\_liverA, CL5176.Contig3\_Mf\_liverA, CL5179.Contig1\_Mf\_liverA, CL5179.Contig2\_Mf\_liverA, CL5187.Contig1\_Mf\_liverA, CL5187.Contig2\_Mf\_liverA, CL5188.Contig1\_Mf\_liverA, CL5188.Contig2\_Mf\_liverA, CL523.Contig1\_Mf\_liverA, CL523.Contig2\_Mf\_liverA, CL5247.Contig1\_Mf\_liverA, CL5247.Contig2\_Mf\_liverA, CL526.Contig1\_Mf\_liverA, CL526.Contig2\_Mf\_liverA, CL526.Contig3\_Mf\_liverA, CL526.Contig4\_Mf\_liverA, CL5265.Contig1\_Mf\_liverA, CL5265.Contig2\_Mf\_liverA, CL5273.Contig1\_Mf\_liverA, CL5273.Contig2\_Mf\_liverA, CL5280.Contig1\_Mf\_liverA, CL5296.Contig1\_Mf\_liverA, CL5296.Contig2\_Mf\_liverA, CL53.Contig1\_Mf\_liverA, CL53.Contig2\_Mf\_liverA, CL530.Contig1\_Mf\_liverA, CL530.Contig2\_Mf\_liverA, CL5303.Contig1\_Mf\_liverA, CL5303.Contig2\_Mf\_liverA, CL5303.Contig4\_Mf\_liverA, CL5303.Contig5\_Mf\_liverA, CL5303.Contig6\_Mf\_liverA, CL5310.Contig1\_Mf\_liverA, CL5310.Contig2\_Mf\_liverA, CL5322.Contig1\_Mf\_liverA, CL5322.Contig2\_Mf\_liverA, CL533.Contig1\_Mf\_liverA, CL533.Contig2\_Mf\_liverA, CL533.Contig3\_Mf\_liverA, CL533.Contig4\_Mf\_liverA, CL5336.Contig1\_Mf\_liverA, CL5336.Contig2\_Mf\_liverA, CL537.Contig1\_Mf\_liverA, CL537.Contig3\_Mf\_liverA, CL5373.Contig1\_Mf\_liverA, CL5373.Contig2\_Mf\_liverA, CL5390.Contig1\_Mf\_liverA, CL5390.Contig2\_Mf\_liverA, CL5437.Contig1\_Mf\_liverA, CL5437.Contig2\_Mf\_liverA, CL5447.Contig1\_Mf\_liverA, CL5447.Contig2\_Mf\_liverA, CL5448.Contig1\_Mf\_liverA, CL5452.Contig1\_Mf\_liverA, CL5452.Contig2\_Mf\_liverA, CL5466.Contig1\_Mf\_liverA, CL5466.Contig2\_Mf\_liverA, CL5475.Contig1\_Mf\_liverA, CL5475.Contig2\_Mf\_liverA, CL5493.Contig1\_Mf\_liverA, CL5493.Contig2\_Mf\_liverA, CL5499.Contig1\_Mf\_liverA, CL5499.Contig2\_Mf\_liverA, CL5539.Contig1\_Mf\_liverA, CL5539.Contig2\_Mf\_liverA, CL5543.Contig1\_Mf\_liverA, CL5549.Contig1\_Mf\_liverA, CL5549.Contig2\_Mf\_liverA, CL5574.Contig1\_Mf\_liverA, CL5580.Contig1\_Mf\_liverA, CL5580.Contig2\_Mf\_liverA, CL5580.Contig3\_Mf\_liverA, CL5588.Contig1\_Mf\_liverA, CL5588.Contig2\_Mf\_liverA, CL5627.Contig1\_Mf\_liverA, CL5631.Contig1\_Mf\_liverA, CL5634.Contig1\_Mf\_liverA, CL5634.Contig2\_Mf\_liverA, CL567.Contig1\_Mf\_liverA, CL567.Contig2\_Mf\_liverA, CL57.Contig1\_Mf\_liverA, CL57.Contig2\_Mf\_liverA, CL5700.Contig2\_Mf\_liverA, CL5713.Contig1\_Mf\_liverA, CL5713.Contig2\_Mf\_liverA, CL5718.Contig1\_Mf\_liverA, CL5718.Contig2\_Mf\_liverA, CL5740.Contig1\_Mf\_liverA, CL5740.Contig2\_Mf\_liverA, CL5787.Contig1\_Mf\_liverA, CL5787.Contig2\_Mf\_liverA, CL5792.Contig1\_Mf\_liverA, CL5792.Contig2\_Mf\_liverA, CL5796.Contig1\_Mf\_liverA, CL5796.Contig2\_Mf\_liverA, CL5818.Contig1\_Mf\_liverA, CL5820.Contig1\_Mf\_liverA, CL5820.Contig2\_Mf\_liverA, CL5840.Contig1\_Mf\_liverA, CL5840.Contig2\_Mf\_liverA, CL5867.Contig1\_Mf\_liverA, CL5867.Contig2\_Mf\_liverA, CL5870.Contig1\_Mf\_liverA, CL5870.Contig2\_Mf\_liverA, CL5873.Contig1\_Mf\_liverA, CL5873.Contig2\_Mf\_liverA, CL595.Contig1\_Mf\_liverA, CL595.Contig2\_Mf\_liverA, CL5965.Contig1\_Mf\_liverA, CL5965.Contig2\_Mf\_liverA, CL5968.Contig1\_Mf\_liverA, CL597.Contig1\_Mf\_liverA, CL597.Contig3\_Mf\_liverA, CL5981.Contig1\_Mf\_liverA, CL5986.Contig1\_Mf\_liverA, CL5993.Contig1\_Mf\_liverA, CL5993.Contig2\_Mf\_liverA, CL5993.Contig3\_Mf\_liverA, CL5993.Contig4\_Mf\_liverA, CL5993.Contig5\_Mf\_liverA, CL6.Contig1\_Mf\_liverA, CL6.Contig2\_Mf\_liverA, CL6.Contig3\_Mf\_liverA, CL6.Contig4\_Mf\_liverA, CL6.Contig5\_Mf\_liverA, CL6.Contig6\_Mf\_liverA, CL6.Contig7\_Mf\_liverA, CL6.Contig8\_Mf\_liverA, CL60.Contig1\_Mf\_liverA, CL60.Contig2\_Mf\_liverA, CL6010.Contig1\_Mf\_liverA, CL6011.Contig1\_Mf\_liverA, CL6011.Contig2\_Mf\_liverA, CL6011.Contig3\_Mf\_liverA, CL6011.Contig4\_Mf\_liverA, CL611.Contig1\_Mf\_liverA, CL611.Contig2\_Mf\_liverA, CL612.Contig10\_Mf\_liverA, CL612.Contig11\_Mf\_liverA, CL612.Contig12\_Mf\_liverA, CL612.Contig13\_Mf\_liverA, CL612.Contig14\_Mf\_liverA, CL612.Contig15\_Mf\_liverA, CL612.Contig16\_Mf\_liverA, CL612.Contig1\_Mf\_liverA, CL612.Contig2\_Mf\_liverA, CL612.Contig3\_Mf\_liverA, CL612.Contig4\_Mf\_liverA, CL612.Contig5\_Mf\_liverA, CL612.Contig6\_Mf\_liverA, CL612.Contig7\_Mf\_liverA, CL612.Contig8\_Mf\_liverA, CL612.Contig9\_Mf\_liverA, CL62.Contig2\_Mf\_liverA, CL652.Contig1\_Mf\_liverA, CL652.Contig2\_Mf\_liverA, CL652.Contig3\_Mf\_liverA, CL652.Contig4\_Mf\_liverA, CL652.Contig5\_Mf\_liverA, CL652.Contig6\_Mf\_liverA, CL652.Contig7\_Mf\_liverA, CL665.Contig1\_Mf\_liverA, CL679.Contig1\_Mf\_liverA, CL679.Contig2\_Mf\_liverA, CL687.Contig11\_Mf\_liverA, CL687.Contig2\_Mf\_liverA, CL687.Contig4\_Mf\_liverA, CL687.Contig8\_Mf\_liverA, CL687.Contig9\_Mf\_liverA, CL715.Contig1\_Mf\_liverA, CL715.Contig2\_Mf\_liverA, CL719.Contig1\_Mf\_liverA, CL719.Contig2\_Mf\_liverA, CL719.Contig3\_Mf\_liverA, CL719.Contig4\_Mf\_liverA, CL719.Contig5\_Mf\_liverA, CL719.Contig6\_Mf\_liverA, CL719.Contig7\_Mf\_liverA, CL719.Contig8\_Mf\_liverA, CL738.Contig2\_Mf\_liverA, CL739.Contig1\_Mf\_liverA, CL739.Contig2\_Mf\_liverA, CL741.Contig13\_Mf\_liverA, CL741.Contig1\_Mf\_liverA, CL741.Contig3\_Mf\_liverA, CL741.Contig4\_Mf\_liverA, CL741.Contig5\_Mf\_liverA, CL741.Contig6\_Mf\_liverA, CL759.Contig1\_Mf\_liverA, CL759.Contig2\_Mf\_liverA, CL768.Contig1\_Mf\_liverA, CL768.Contig2\_Mf\_liverA, CL781.Contig1\_Mf\_liverA, CL781.Contig2\_Mf\_liverA, CL809.Contig1\_Mf\_liverA, CL809.Contig2\_Mf\_liverA, CL810.Contig1\_Mf\_liverA, CL810.Contig2\_Mf\_liverA, CL811.Contig1\_Mf\_liverA, CL811.Contig2\_Mf\_liverA, CL811.Contig3\_Mf\_liverA, CL855.Contig2\_Mf\_liverA, CL855.Contig4\_Mf\_liverA, CL866.Contig1\_Mf\_liverA, CL866.Contig2\_Mf\_liverA, CL888.Contig1\_Mf\_liverA, CL916.Contig1\_Mf\_liverA, CL916.Contig2\_Mf\_liverA, CL916.Contig3\_Mf\_liverA, CL943.Contig1\_Mf\_liverA, CL943.Contig2\_Mf\_liverA, CL963.Contig1\_Mf\_liverA, CL963.Contig2\_Mf\_liverA, CL963.Contig3\_Mf\_liverA, CL963.Contig4\_Mf\_liverA, CL963.Contig5\_Mf\_liverA, CL989.Contig10\_Mf\_liverA, CL989.Contig11\_Mf\_liverA, CL989.Contig12\_Mf\_liverA, CL989.Contig13\_Mf\_liverA, CL989.Contig14\_Mf\_liverA, CL989.Contig15\_Mf\_liverA, CL989.Contig1\_Mf\_liverA, CL989.Contig23\_Mf\_liverA, CL989.Contig2\_Mf\_liverA, CL989.Contig34\_Mf\_liverA, CL989.Contig39\_Mf\_liverA, CL989.Contig3\_Mf\_liverA, CL989.Contig40\_Mf\_liverA, CL989.Contig41\_Mf\_liverA, CL989.Contig42\_Mf\_liverA, CL989.Contig43\_Mf\_liverA, CL989.Contig4\_Mf\_liverA, CL989.Contig5\_Mf\_liverA, CL989.Contig6\_Mf\_liverA, CL989.Contig7\_Mf\_liverA, CL989.Contig8\_Mf\_liverA, CL989.Contig9\_Mf\_liverA, Unigene1003\_Mf\_liverA, Unigene10136\_Mf\_liverA, Unigene10222\_Mf\_liverA, Unigene10313\_Mf\_liverA, Unigene10386\_Mf\_liverA, Unigene1041\_Mf\_liverA, Unigene10438\_Mf\_liverA, Unigene1049\_Mf\_liverA, Unigene1050\_Mf\_liverA, Unigene10612\_Mf\_liverA, Unigene10661\_Mf\_liverA, Unigene10695\_Mf\_liverA, Unigene10704\_Mf\_liverA, Unigene10705\_Mf\_liverA, Unigene10713\_Mf\_liverA, Unigene10721\_Mf\_liverA, Unigene10769\_Mf\_liverA, Unigene10791\_Mf\_liverA, Unigene10863\_Mf\_liverA, Unigene10934\_Mf\_liverA, Unigene10966\_Mf\_liverA, Unigene1107\_Mf\_liverA, Unigene11082\_Mf\_liverA, Unigene11083\_Mf\_liverA, Unigene110\_Mf\_liverA, Unigene1124\_Mf\_liverA, Unigene11339\_Mf\_liverA, Unigene113\_Mf\_liverA, Unigene1167\_Mf\_liverA, Unigene11812\_Mf\_liverA, Unigene11813\_Mf\_liverA, Unigene11831\_Mf\_liverA, Unigene11858\_Mf\_liverA, Unigene11877\_Mf\_liverA, Unigene1187\_Mf\_liverA, Unigene1188\_Mf\_liverA, Unigene1189\_Mf\_liverA, Unigene11964\_Mf\_liverA, Unigene11\_Mf\_liverA, Unigene1205\_Mf\_liverA, Unigene12153\_Mf\_liverA, Unigene12329\_Mf\_liverA, Unigene12339\_Mf\_liverA, Unigene12503\_Mf\_liverA, Unigene12583\_Mf\_liverA, Unigene1264\_Mf\_liverA, Unigene12676\_Mf\_liverA, Unigene12807\_Mf\_liverA, Unigene1280\_Mf\_liverA, Unigene12837\_Mf\_liverA, Unigene1283\_Mf\_liverA, Unigene12840\_Mf\_liverA, Unigene12891\_Mf\_liverA, Unigene12908\_Mf\_liverA, Unigene12909\_Mf\_liverA, Unigene12910\_Mf\_liverA, Unigene12913\_Mf\_liverA, Unigene12914\_Mf\_liverA, Unigene12918\_Mf\_liverA, Unigene12937\_Mf\_liverA, Unigene13032\_Mf\_liverA, Unigene13049\_Mf\_liverA, Unigene13050\_Mf\_liverA, Unigene13079\_Mf\_liverA, Unigene13153\_Mf\_liverA, Unigene13188\_Mf\_liverA, Unigene13196\_Mf\_liverA, Unigene13236\_Mf\_liverA, Unigene13252\_Mf\_liverA, Unigene13253\_Mf\_liverA, Unigene13314\_Mf\_liverA, Unigene13315\_Mf\_liverA, Unigene13320\_Mf\_liverA, Unigene13333\_Mf\_liverA, Unigene13342\_Mf\_liverA, Unigene13344\_Mf\_liverA, Unigene13347\_Mf\_liverA, Unigene13349\_Mf\_liverA, Unigene13354\_Mf\_liverA, Unigene13386\_Mf\_liverA, Unigene13387\_Mf\_liverA, Unigene13394\_Mf\_liverA, Unigene13398\_Mf\_liverA, Unigene13406\_Mf\_liverA, Unigene13436\_Mf\_liverA, Unigene1344\_Mf\_liverA, Unigene1345\_Mf\_liverA, Unigene1346\_Mf\_liverA, Unigene13478\_Mf\_liverA, Unigene1347\_Mf\_liverA, Unigene13485\_Mf\_liverA, Unigene13536\_Mf\_liverA, Unigene13572\_Mf\_liverA, Unigene1361\_Mf\_liverA, Unigene13630\_Mf\_liverA, Unigene13631\_Mf\_liverA, Unigene13647\_Mf\_liverA, Unigene13657\_Mf\_liverA, Unigene13658\_Mf\_liverA, Unigene13659\_Mf\_liverA, Unigene13665\_Mf\_liverA, Unigene13672\_Mf\_liverA, Unigene13675\_Mf\_liverA, Unigene13676\_Mf\_liverA, Unigene13677\_Mf\_liverA, Unigene13678\_Mf\_liverA, Unigene13752\_Mf\_liverA, Unigene13766\_Mf\_liverA, Unigene13779\_Mf\_liverA, Unigene13819\_Mf\_liverA, Unigene13838\_Mf\_liverA, Unigene1383\_Mf\_liverA, Unigene13851\_Mf\_liverA, Unigene13857\_Mf\_liverA, Unigene13858\_Mf\_liverA, Unigene13864\_Mf\_liverA, Unigene13911\_Mf\_liverA, Unigene13951\_Mf\_liverA, Unigene13961\_Mf\_liverA, Unigene13962\_Mf\_liverA, Unigene13999\_Mf\_liverA, Unigene139\_Mf\_liverA, Unigene14016\_Mf\_liverA, Unigene1401\_Mf\_liverA, Unigene14040\_Mf\_liverA, Unigene14041\_Mf\_liverA, Unigene140\_Mf\_liverA, Unigene14134\_Mf\_liverA, Unigene14145\_Mf\_liverA, Unigene14150\_Mf\_liverA, Unigene14171\_Mf\_liverA, Unigene14199\_Mf\_liverA, Unigene14200\_Mf\_liverA, Unigene14202\_Mf\_liverA, Unigene14235\_Mf\_liverA, Unigene1427\_Mf\_liverA, Unigene14285\_Mf\_liverA, Unigene14322\_Mf\_liverA, Unigene1432\_Mf\_liverA, Unigene14367\_Mf\_liverA, Unigene14380\_Mf\_liverA, Unigene14381\_Mf\_liverA, Unigene14417\_Mf\_liverA, Unigene14456\_Mf\_liverA, Unigene14514\_Mf\_liverA, Unigene14515\_Mf\_liverA, Unigene14529\_Mf\_liverA, Unigene14530\_Mf\_liverA, Unigene14557\_Mf\_liverA, Unigene14558\_Mf\_liverA, Unigene14633\_Mf\_liverA, Unigene14644\_Mf\_liverA, Unigene14699\_Mf\_liverA, Unigene1472\_Mf\_liverA, Unigene14762\_Mf\_liverA, Unigene14789\_Mf\_liverA, Unigene14792\_Mf\_liverA, Unigene14796\_Mf\_liverA, Unigene1479\_Mf\_liverA, Unigene14815\_Mf\_liverA, Unigene14840\_Mf\_liverA, Unigene14870\_Mf\_liverA, Unigene14871\_Mf\_liverA, Unigene14886\_Mf\_liverA, Unigene15016\_Mf\_liverA, Unigene15170\_Mf\_liverA, Unigene15175\_Mf\_liverA, Unigene15176\_Mf\_liverA, Unigene151\_Mf\_liverA, Unigene15224\_Mf\_liverA, Unigene1522\_Mf\_liverA, Unigene15236\_Mf\_liverA, Unigene15237\_Mf\_liverA, Unigene15239\_Mf\_liverA, Unigene15252\_Mf\_liverA, Unigene15280\_Mf\_liverA, Unigene15281\_Mf\_liverA, Unigene15291\_Mf\_liverA, Unigene15320\_Mf\_liverA, Unigene15333\_Mf\_liverA, Unigene15350\_Mf\_liverA, Unigene15354\_Mf\_liverA, Unigene15355\_Mf\_liverA, Unigene15376\_Mf\_liverA, Unigene15377\_Mf\_liverA, Unigene15400\_Mf\_liverA, Unigene15402\_Mf\_liverA, Unigene15424\_Mf\_liverA, Unigene15454\_Mf\_liverA, Unigene15467\_Mf\_liverA, Unigene15588\_Mf\_liverA, Unigene15589\_Mf\_liverA, Unigene15594\_Mf\_liverA, Unigene15606\_Mf\_liverA, Unigene15648\_Mf\_liverA, Unigene15728\_Mf\_liverA, Unigene15794\_Mf\_liverA, Unigene15853\_Mf\_liverA, Unigene15907\_Mf\_liverA, Unigene15924\_Mf\_liverA, Unigene15925\_Mf\_liverA, Unigene15955\_Mf\_liverA, Unigene16176\_Mf\_liverA, Unigene16261\_Mf\_liverA, Unigene162\_Mf\_liverA, Unigene16378\_Mf\_liverA, Unigene16514\_Mf\_liverA, Unigene16566\_Mf\_liverA, Unigene16604\_Mf\_liverA, Unigene16633\_Mf\_liverA, Unigene16711\_Mf\_liverA, Unigene16712\_Mf\_liverA, Unigene16759\_Mf\_liverA, Unigene16760\_Mf\_liverA, Unigene16788\_Mf\_liverA, Unigene16789\_Mf\_liverA, Unigene16817\_Mf\_liverA, Unigene17016\_Mf\_liverA, Unigene1702\_Mf\_liverA, Unigene17052\_Mf\_liverA, Unigene17168\_Mf\_liverA, Unigene17178\_Mf\_liverA, Unigene17317\_Mf\_liverA, Unigene17382\_Mf\_liverA, Unigene17534\_Mf\_liverA, Unigene17566\_Mf\_liverA, Unigene17569\_Mf\_liverA, Unigene17608\_Mf\_liverA, Unigene17629\_Mf\_liverA, Unigene17640\_Mf\_liverA, Unigene17754\_Mf\_liverA, Unigene17814\_Mf\_liverA, Unigene17896\_Mf\_liverA, Unigene17897\_Mf\_liverA, Unigene17969\_Mf\_liverA, Unigene18022\_Mf\_liverA, Unigene18065\_Mf\_liverA, Unigene18092\_Mf\_liverA, Unigene18097\_Mf\_liverA, Unigene1810\_Mf\_liverA, Unigene18200\_Mf\_liverA, Unigene18244\_Mf\_liverA, Unigene18245\_Mf\_liverA, Unigene183\_Mf\_liverA, Unigene18430\_Mf\_liverA, Unigene18431\_Mf\_liverA, Unigene18441\_Mf\_liverA, Unigene18443\_Mf\_liverA, Unigene18603\_Mf\_liverA, Unigene18643\_Mf\_liverA, Unigene18727\_Mf\_liverA, Unigene18731\_Mf\_liverA, Unigene18874\_Mf\_liverA, Unigene18992\_Mf\_liverA, Unigene19002\_Mf\_liverA, Unigene19003\_Mf\_liverA, Unigene19044\_Mf\_liverA, Unigene19045\_Mf\_liverA, Unigene19083\_Mf\_liverA, Unigene19084\_Mf\_liverA, Unigene19085\_Mf\_liverA, Unigene190\_Mf\_liverA, Unigene19155\_Mf\_liverA, Unigene19232\_Mf\_liverA, Unigene19291\_Mf\_liverA, Unigene19392\_Mf\_liverA, Unigene19393\_Mf\_liverA, Unigene19394\_Mf\_liverA, Unigene19399\_Mf\_liverA, Unigene19460\_Mf\_liverA, Unigene19461\_Mf\_liverA, Unigene19462\_Mf\_liverA, Unigene19625\_Mf\_liverA, Unigene19626\_Mf\_liverA, Unigene19627\_Mf\_liverA, Unigene19702\_Mf\_liverA, Unigene19960\_Mf\_liverA, Unigene19961\_Mf\_liverA, Unigene19\_Mf\_liverA, Unigene20070\_Mf\_liverA, Unigene20095\_Mf\_liverA, Unigene20096\_Mf\_liverA, Unigene20166\_Mf\_liverA, Unigene20179\_Mf\_liverA, Unigene20180\_Mf\_liverA, Unigene20249\_Mf\_liverA, Unigene20254\_Mf\_liverA, Unigene20371\_Mf\_liverA, Unigene20372\_Mf\_liverA, Unigene203\_Mf\_liverA, Unigene20492\_Mf\_liverA, Unigene20493\_Mf\_liverA, Unigene20517\_Mf\_liverA, Unigene20557\_Mf\_liverA, Unigene20696\_Mf\_liverA, Unigene20701\_Mf\_liverA, Unigene20734\_Mf\_liverA, Unigene20735\_Mf\_liverA, Unigene20736\_Mf\_liverA, Unigene20766\_Mf\_liverA, Unigene20811\_Mf\_liverA, Unigene20832\_Mf\_liverA, Unigene20926\_Mf\_liverA, Unigene20978\_Mf\_liverA, Unigene21020\_Mf\_liverA, Unigene21021\_Mf\_liverA, Unigene21104\_Mf\_liverA, Unigene21117\_Mf\_liverA, Unigene21124\_Mf\_liverA, Unigene21138\_Mf\_liverA, Unigene21139\_Mf\_liverA, Unigene21155\_Mf\_liverA, Unigene21156\_Mf\_liverA, Unigene21163\_Mf\_liverA, Unigene21318\_Mf\_liverA, Unigene21319\_Mf\_liverA, Unigene21466\_Mf\_liverA, Unigene21478\_Mf\_liverA, Unigene21479\_Mf\_liverA, Unigene21513\_Mf\_liverA, Unigene21546\_Mf\_liverA, Unigene21547\_Mf\_liverA, Unigene21626\_Mf\_liverA, Unigene21646\_Mf\_liverA, Unigene21707\_Mf\_liverA, Unigene21840\_Mf\_liverA, Unigene21863\_Mf\_liverA, Unigene2191\_Mf\_liverA, Unigene21972\_Mf\_liverA, Unigene2204\_Mf\_liverA, Unigene22070\_Mf\_liverA, Unigene22071\_Mf\_liverA, Unigene22072\_Mf\_liverA, Unigene22076\_Mf\_liverA, Unigene22077\_Mf\_liverA, Unigene22096\_Mf\_liverA, Unigene22100\_Mf\_liverA, Unigene22135\_Mf\_liverA, Unigene22499\_Mf\_liverA, Unigene22500\_Mf\_liverA, Unigene22575\_Mf\_liverA, Unigene2257\_Mf\_liverA, Unigene22611\_Mf\_liverA, Unigene22612\_Mf\_liverA, Unigene22672\_Mf\_liverA, Unigene22681\_Mf\_liverA, Unigene22722\_Mf\_liverA, Unigene22723\_Mf\_liverA, Unigene2283\_Mf\_liverA, Unigene22963\_Mf\_liverA, Unigene22967\_Mf\_liverA, Unigene22968\_Mf\_liverA, Unigene22969\_Mf\_liverA, Unigene22970\_Mf\_liverA, Unigene229\_Mf\_liverA, Unigene23066\_Mf\_liverA, Unigene2307\_Mf\_liverA, Unigene23179\_Mf\_liverA, Unigene23185\_Mf\_liverA, Unigene23209\_Mf\_liverA, Unigene23278\_Mf\_liverA, Unigene23279\_Mf\_liverA, Unigene23432\_Mf\_liverA, Unigene23472\_Mf\_liverA, Unigene23473\_Mf\_liverA, Unigene2350\_Mf\_liverA, Unigene23560\_Mf\_liverA, Unigene2359\_Mf\_liverA, Unigene2360\_Mf\_liverA, Unigene2361\_Mf\_liverA, Unigene24047\_Mf\_liverA, Unigene24048\_Mf\_liverA, Unigene24085\_Mf\_liverA, Unigene24093\_Mf\_liverA, Unigene240\_Mf\_liverA, Unigene24159\_Mf\_liverA, Unigene24201\_Mf\_liverA, Unigene24202\_Mf\_liverA, Unigene24211\_Mf\_liverA, Unigene24212\_Mf\_liverA, Unigene24213\_Mf\_liverA, Unigene24238\_Mf\_liverA, Unigene24265\_Mf\_liverA, Unigene24266\_Mf\_liverA, Unigene24271\_Mf\_liverA, Unigene24272\_Mf\_liverA, Unigene24275\_Mf\_liverA, Unigene242\_Mf\_liverA, Unigene24345\_Mf\_liverA, Unigene24354\_Mf\_liverA, Unigene24355\_Mf\_liverA, Unigene24392\_Mf\_liverA, Unigene24402\_Mf\_liverA, Unigene24404\_Mf\_liverA, Unigene24412\_Mf\_liverA, Unigene24423\_Mf\_liverA, Unigene24434\_Mf\_liverA, Unigene24488\_Mf\_liverA, Unigene24522\_Mf\_liverA, Unigene24558\_Mf\_liverA, Unigene24581\_Mf\_liverA, Unigene2458\_Mf\_liverA, Unigene24619\_Mf\_liverA, Unigene24641\_Mf\_liverA, Unigene24653\_Mf\_liverA, Unigene24697\_Mf\_liverA, Unigene24712\_Mf\_liverA, Unigene24713\_Mf\_liverA, Unigene24734\_Mf\_liverA, Unigene24749\_Mf\_liverA, Unigene24765\_Mf\_liverA, Unigene24784\_Mf\_liverA, Unigene24808\_Mf\_liverA, Unigene24812\_Mf\_liverA, Unigene24902\_Mf\_liverA, Unigene24927\_Mf\_liverA, Unigene25005\_Mf\_liverA, Unigene25099\_Mf\_liverA, Unigene25100\_Mf\_liverA, Unigene25101\_Mf\_liverA, Unigene25117\_Mf\_liverA, Unigene25178\_Mf\_liverA, Unigene25179\_Mf\_liverA, Unigene25183\_Mf\_liverA, Unigene2526\_Mf\_liverA, Unigene25304\_Mf\_liverA, Unigene25353\_Mf\_liverA, Unigene2536\_Mf\_liverA, Unigene25373\_Mf\_liverA, Unigene25434\_Mf\_liverA, Unigene2548\_Mf\_liverA, Unigene25509\_Mf\_liverA, Unigene25586\_Mf\_liverA, Unigene25594\_Mf\_liverA, Unigene25595\_Mf\_liverA, Unigene25596\_Mf\_liverA, Unigene25602\_Mf\_liverA, Unigene25615\_Mf\_liverA, Unigene25640\_Mf\_liverA, Unigene25644\_Mf\_liverA, Unigene25677\_Mf\_liverA, Unigene25689\_Mf\_liverA, Unigene25763\_Mf\_liverA, Unigene25784\_Mf\_liverA, Unigene25785\_Mf\_liverA, Unigene25786\_Mf\_liverA, Unigene25840\_Mf\_liverA, Unigene25864\_Mf\_liverA, Unigene25874\_Mf\_liverA, Unigene25880\_Mf\_liverA, Unigene25884\_Mf\_liverA, Unigene25906\_Mf\_liverA, Unigene25941\_Mf\_liverA, Unigene25943\_Mf\_liverA, Unigene25945\_Mf\_liverA, Unigene25994\_Mf\_liverA, Unigene26025\_Mf\_liverA, Unigene26103\_Mf\_liverA, Unigene26123\_Mf\_liverA, Unigene26202\_Mf\_liverA, Unigene26214\_Mf\_liverA, Unigene26274\_Mf\_liverA, Unigene26278\_Mf\_liverA, Unigene2629\_Mf\_liverA, Unigene2633\_Mf\_liverA, Unigene26486\_Mf\_liverA, Unigene26623\_Mf\_liverA, Unigene26624\_Mf\_liverA, Unigene26784\_Mf\_liverA, Unigene26785\_Mf\_liverA, Unigene26786\_Mf\_liverA, Unigene26874\_Mf\_liverA, Unigene26875\_Mf\_liverA, Unigene26904\_Mf\_liverA, Unigene26923\_Mf\_liverA, Unigene26948\_Mf\_liverA, Unigene26949\_Mf\_liverA, Unigene26955\_Mf\_liverA, Unigene26969\_Mf\_liverA, Unigene27017\_Mf\_liverA, Unigene27018\_Mf\_liverA, Unigene27120\_Mf\_liverA, Unigene27121\_Mf\_liverA, Unigene27135\_Mf\_liverA, Unigene27144\_Mf\_liverA, Unigene27208\_Mf\_liverA, Unigene27232\_Mf\_liverA, Unigene27391\_Mf\_liverA, Unigene27424\_Mf\_liverA, Unigene27425\_Mf\_liverA, Unigene27543\_Mf\_liverA, Unigene27544\_Mf\_liverA, Unigene27545\_Mf\_liverA, Unigene27569\_Mf\_liverA, Unigene27592\_Mf\_liverA, Unigene27609\_Mf\_liverA, Unigene27612\_Mf\_liverA, Unigene27619\_Mf\_liverA, Unigene27633\_Mf\_liverA, Unigene27644\_Mf\_liverA, Unigene27645\_Mf\_liverA, Unigene27680\_Mf\_liverA, Unigene27727\_Mf\_liverA, Unigene27730\_Mf\_liverA, Unigene27736\_Mf\_liverA, Unigene27755\_Mf\_liverA, Unigene27756\_Mf\_liverA, Unigene27760\_Mf\_liverA, Unigene27763\_Mf\_liverA, Unigene27764\_Mf\_liverA, Unigene27895\_Mf\_liverA, Unigene27896\_Mf\_liverA, Unigene27898\_Mf\_liverA, Unigene28003\_Mf\_liverA, Unigene28083\_Mf\_liverA, Unigene28106\_Mf\_liverA, Unigene28133\_Mf\_liverA, Unigene28136\_Mf\_liverA, Unigene28153\_Mf\_liverA, Unigene28188\_Mf\_liverA, Unigene28218\_Mf\_liverA, Unigene28255\_Mf\_liverA, Unigene28355\_Mf\_liverA, Unigene28356\_Mf\_liverA, Unigene28379\_Mf\_liverA, Unigene28380\_Mf\_liverA, Unigene28381\_Mf\_liverA, Unigene28392\_Mf\_liverA, Unigene28456\_Mf\_liverA, Unigene28457\_Mf\_liverA, Unigene28458\_Mf\_liverA, Unigene28530\_Mf\_liverA, Unigene28544\_Mf\_liverA, Unigene28545\_Mf\_liverA, Unigene28587\_Mf\_liverA, Unigene28623\_Mf\_liverA, Unigene28656\_Mf\_liverA, Unigene28731\_Mf\_liverA, Unigene2876\_Mf\_liverA, Unigene28818\_Mf\_liverA, Unigene28819\_Mf\_liverA, Unigene28855\_Mf\_liverA, Unigene28891\_Mf\_liverA, Unigene2889\_Mf\_liverA, Unigene28939\_Mf\_liverA, Unigene28983\_Mf\_liverA, Unigene29119\_Mf\_liverA, Unigene29120\_Mf\_liverA, Unigene29146\_Mf\_liverA, Unigene29194\_Mf\_liverA, Unigene29303\_Mf\_liverA, Unigene2930\_Mf\_liverA, Unigene29339\_Mf\_liverA, Unigene29368\_Mf\_liverA, Unigene2939\_Mf\_liverA, Unigene29431\_Mf\_liverA, Unigene29432\_Mf\_liverA, Unigene29504\_Mf\_liverA, Unigene29558\_Mf\_liverA, Unigene29572\_Mf\_liverA, Unigene29585\_Mf\_liverA, Unigene29631\_Mf\_liverA, Unigene29645\_Mf\_liverA, Unigene29727\_Mf\_liverA, Unigene29796\_Mf\_liverA, Unigene29836\_Mf\_liverA, Unigene29851\_Mf\_liverA, Unigene29852\_Mf\_liverA, Unigene29876\_Mf\_liverA, Unigene29882\_Mf\_liverA, Unigene29910\_Mf\_liverA, Unigene29966\_Mf\_liverA, Unigene30002\_Mf\_liverA, Unigene30003\_Mf\_liverA, Unigene30004\_Mf\_liverA, Unigene30031\_Mf\_liverA, Unigene30066\_Mf\_liverA, Unigene30072\_Mf\_liverA, Unigene30132\_Mf\_liverA, Unigene30139\_Mf\_liverA, Unigene30179\_Mf\_liverA, Unigene30180\_Mf\_liverA, Unigene30349\_Mf\_liverA, Unigene30355\_Mf\_liverA, Unigene30413\_Mf\_liverA, Unigene30425\_Mf\_liverA, Unigene30437\_Mf\_liverA, Unigene30481\_Mf\_liverA, Unigene30485\_Mf\_liverA, Unigene30499\_Mf\_liverA, Unigene30543\_Mf\_liverA, Unigene30553\_Mf\_liverA, Unigene30554\_Mf\_liverA, Unigene30627\_Mf\_liverA, Unigene30714\_Mf\_liverA, Unigene30715\_Mf\_liverA, Unigene30730\_Mf\_liverA, Unigene30731\_Mf\_liverA, Unigene30777\_Mf\_liverA, Unigene30789\_Mf\_liverA, Unigene30803\_Mf\_liverA, Unigene30811\_Mf\_liverA, Unigene30818\_Mf\_liverA, Unigene30839\_Mf\_liverA, Unigene30854\_Mf\_liverA, Unigene30858\_Mf\_liverA, Unigene30871\_Mf\_liverA, Unigene30877\_Mf\_liverA, Unigene30900\_Mf\_liverA, Unigene30969\_Mf\_liverA, Unigene3101\_Mf\_liverA, Unigene31040\_Mf\_liverA, Unigene31041\_Mf\_liverA, Unigene31051\_Mf\_liverA, Unigene31081\_Mf\_liverA, Unigene31101\_Mf\_liverA, Unigene31136\_Mf\_liverA, Unigene31164\_Mf\_liverA, Unigene31165\_Mf\_liverA, Unigene31175\_Mf\_liverA, Unigene31180\_Mf\_liverA, Unigene31181\_Mf\_liverA, Unigene31182\_Mf\_liverA, Unigene31189\_Mf\_liverA, Unigene31205\_Mf\_liverA, Unigene31206\_Mf\_liverA, Unigene31207\_Mf\_liverA, Unigene31229\_Mf\_liverA, Unigene31230\_Mf\_liverA, Unigene31231\_Mf\_liverA, Unigene31292\_Mf\_liverA, Unigene31297\_Mf\_liverA, Unigene31300\_Mf\_liverA, Unigene31303\_Mf\_liverA, Unigene31306\_Mf\_liverA, Unigene31368\_Mf\_liverA, Unigene31392\_Mf\_liverA, Unigene31426\_Mf\_liverA, Unigene3142\_Mf\_liverA, Unigene31448\_Mf\_liverA, Unigene31457\_Mf\_liverA, Unigene31458\_Mf\_liverA, Unigene31511\_Mf\_liverA, Unigene31523\_Mf\_liverA, Unigene31533\_Mf\_liverA, Unigene31560\_Mf\_liverA, Unigene31561\_Mf\_liverA, Unigene31583\_Mf\_liverA, Unigene31602\_Mf\_liverA, Unigene31623\_Mf\_liverA, Unigene31627\_Mf\_liverA, Unigene31666\_Mf\_liverA, Unigene31694\_Mf\_liverA, Unigene31697\_Mf\_liverA, Unigene31698\_Mf\_liverA, Unigene31750\_Mf\_liverA, Unigene31767\_Mf\_liverA, Unigene31772\_Mf\_liverA, Unigene31786\_Mf\_liverA, Unigene31919\_Mf\_liverA, Unigene31920\_Mf\_liverA, Unigene31925\_Mf\_liverA, Unigene31998\_Mf\_liverA, Unigene32004\_Mf\_liverA, Unigene32006\_Mf\_liverA, Unigene32012\_Mf\_liverA, Unigene32066\_Mf\_liverA, Unigene32070\_Mf\_liverA, Unigene32071\_Mf\_liverA, Unigene32101\_Mf\_liverA, Unigene32102\_Mf\_liverA, Unigene32142\_Mf\_liverA, Unigene3215\_Mf\_liverA, Unigene32257\_Mf\_liverA, Unigene32271\_Mf\_liverA, Unigene32294\_Mf\_liverA, Unigene32295\_Mf\_liverA, Unigene322\_Mf\_liverA, Unigene32314\_Mf\_liverA, Unigene32331\_Mf\_liverA, Unigene32338\_Mf\_liverA, Unigene323\_Mf\_liverA, Unigene32410\_Mf\_liverA, Unigene32421\_Mf\_liverA, Unigene32529\_Mf\_liverA, Unigene32562\_Mf\_liverA, Unigene32580\_Mf\_liverA, Unigene32614\_Mf\_liverA, Unigene32625\_Mf\_liverA, Unigene32650\_Mf\_liverA, Unigene32695\_Mf\_liverA, Unigene32811\_Mf\_liverA, Unigene32852\_Mf\_liverA, Unigene32858\_Mf\_liverA, Unigene32859\_Mf\_liverA, Unigene32860\_Mf\_liverA, Unigene32889\_Mf\_liverA, Unigene32892\_Mf\_liverA, Unigene32895\_Mf\_liverA, Unigene32906\_Mf\_liverA, Unigene32920\_Mf\_liverA, Unigene32945\_Mf\_liverA, Unigene33043\_Mf\_liverA, Unigene33044\_Mf\_liverA, Unigene33086\_Mf\_liverA, Unigene33127\_Mf\_liverA, Unigene33128\_Mf\_liverA, Unigene3313\_Mf\_liverA, Unigene33214\_Mf\_liverA, Unigene33237\_Mf\_liverA, Unigene33300\_Mf\_liverA, Unigene33317\_Mf\_liverA, Unigene33318\_Mf\_liverA, Unigene33433\_Mf\_liverA, Unigene33502\_Mf\_liverA, Unigene33522\_Mf\_liverA, Unigene33523\_Mf\_liverA, Unigene33524\_Mf\_liverA, Unigene33531\_Mf\_liverA, Unigene33545\_Mf\_liverA, Unigene33552\_Mf\_liverA, Unigene33580\_Mf\_liverA, Unigene33600\_Mf\_liverA, Unigene33660\_Mf\_liverA, Unigene33688\_Mf\_liverA, Unigene33696\_Mf\_liverA, Unigene33730\_Mf\_liverA, Unigene33760\_Mf\_liverA, Unigene3377\_Mf\_liverA, Unigene33799\_Mf\_liverA, Unigene33800\_Mf\_liverA, Unigene33813\_Mf\_liverA, Unigene33836\_Mf\_liverA, Unigene33853\_Mf\_liverA, Unigene33854\_Mf\_liverA, Unigene33875\_Mf\_liverA, Unigene33880\_Mf\_liverA, Unigene33884\_Mf\_liverA, Unigene33936\_Mf\_liverA, Unigene33977\_Mf\_liverA, Unigene33991\_Mf\_liverA, Unigene34174\_Mf\_liverA, Unigene34183\_Mf\_liverA, Unigene34184\_Mf\_liverA, Unigene34185\_Mf\_liverA, Unigene34187\_Mf\_liverA, Unigene34200\_Mf\_liverA, Unigene34223\_Mf\_liverA, Unigene34224\_Mf\_liverA, Unigene34262\_Mf\_liverA, Unigene34263\_Mf\_liverA, Unigene34286\_Mf\_liverA, Unigene34289\_Mf\_liverA, Unigene34290\_Mf\_liverA, Unigene34291\_Mf\_liverA, Unigene34341\_Mf\_liverA, Unigene34342\_Mf\_liverA, Unigene34343\_Mf\_liverA, Unigene34346\_Mf\_liverA, Unigene34347\_Mf\_liverA, Unigene34348\_Mf\_liverA, Unigene34373\_Mf\_liverA, Unigene34383\_Mf\_liverA, Unigene34391\_Mf\_liverA, Unigene34398\_Mf\_liverA, Unigene3439\_Mf\_liverA, Unigene34406\_Mf\_liverA, Unigene34412\_Mf\_liverA, Unigene34413\_Mf\_liverA, Unigene34414\_Mf\_liverA, Unigene34415\_Mf\_liverA, Unigene34416\_Mf\_liverA, Unigene34417\_Mf\_liverA, Unigene34418\_Mf\_liverA, Unigene34419\_Mf\_liverA, Unigene34420\_Mf\_liverA, Unigene34421\_Mf\_liverA, Unigene34422\_Mf\_liverA, Unigene34423\_Mf\_liverA, Unigene34424\_Mf\_liverA, Unigene34425\_Mf\_liverA, Unigene34426\_Mf\_liverA, Unigene34427\_Mf\_liverA, Unigene34428\_Mf\_liverA, Unigene34429\_Mf\_liverA, Unigene34494\_Mf\_liverA, Unigene34497\_Mf\_liverA, Unigene34516\_Mf\_liverA, Unigene34628\_Mf\_liverA, Unigene34630\_Mf\_liverA, Unigene34650\_Mf\_liverA, Unigene34651\_Mf\_liverA, Unigene34652\_Mf\_liverA, Unigene34760\_Mf\_liverA, Unigene34764\_Mf\_liverA, Unigene34770\_Mf\_liverA, Unigene34771\_Mf\_liverA, Unigene34772\_Mf\_liverA, Unigene34808\_Mf\_liverA, Unigene34842\_Mf\_liverA, Unigene34843\_Mf\_liverA, Unigene34844\_Mf\_liverA, Unigene34845\_Mf\_liverA, Unigene34968\_Mf\_liverA, Unigene35046\_Mf\_liverA, Unigene35053\_Mf\_liverA, Unigene35054\_Mf\_liverA, Unigene35055\_Mf\_liverA, Unigene35056\_Mf\_liverA, Unigene35069\_Mf\_liverA, Unigene35135\_Mf\_liverA, Unigene3514\_Mf\_liverA, Unigene35156\_Mf\_liverA, Unigene35157\_Mf\_liverA, Unigene35158\_Mf\_liverA, Unigene35159\_Mf\_liverA, Unigene35160\_Mf\_liverA, Unigene35221\_Mf\_liverA, Unigene35222\_Mf\_liverA, Unigene35269\_Mf\_liverA, Unigene35283\_Mf\_liverA, Unigene35287\_Mf\_liverA, Unigene35288\_Mf\_liverA, Unigene35305\_Mf\_liverA, Unigene35358\_Mf\_liverA, Unigene35359\_Mf\_liverA, Unigene35382\_Mf\_liverA, Unigene353\_Mf\_liverA, Unigene35449\_Mf\_liverA, Unigene35450\_Mf\_liverA, Unigene35455\_Mf\_liverA, Unigene35457\_Mf\_liverA, Unigene35458\_Mf\_liverA, Unigene35486\_Mf\_liverA, Unigene35500\_Mf\_liverA, Unigene35503\_Mf\_liverA, Unigene35504\_Mf\_liverA, Unigene35505\_Mf\_liverA, Unigene35542\_Mf\_liverA, Unigene35543\_Mf\_liverA, Unigene35609\_Mf\_liverA, Unigene35750\_Mf\_liverA, Unigene35752\_Mf\_liverA, Unigene35824\_Mf\_liverA, Unigene35857\_Mf\_liverA, Unigene35875\_Mf\_liverA, Unigene35930\_Mf\_liverA, Unigene35931\_Mf\_liverA, Unigene35932\_Mf\_liverA, Unigene35969\_Mf\_liverA, Unigene35970\_Mf\_liverA, Unigene35978\_Mf\_liverA, Unigene36135\_Mf\_liverA, Unigene36141\_Mf\_liverA, Unigene36144\_Mf\_liverA, Unigene36189\_Mf\_liverA, Unigene36190\_Mf\_liverA, Unigene36313\_Mf\_liverA, Unigene36315\_Mf\_liverA, Unigene36318\_Mf\_liverA, Unigene36319\_Mf\_liverA, Unigene36320\_Mf\_liverA, Unigene36321\_Mf\_liverA, Unigene36357\_Mf\_liverA, Unigene36422\_Mf\_liverA, Unigene36423\_Mf\_liverA, Unigene36424\_Mf\_liverA, Unigene36430\_Mf\_liverA, Unigene36440\_Mf\_liverA, Unigene36504\_Mf\_liverA, Unigene36507\_Mf\_liverA, Unigene36508\_Mf\_liverA, Unigene36509\_Mf\_liverA, Unigene36510\_Mf\_liverA, Unigene36513\_Mf\_liverA, Unigene36514\_Mf\_liverA, Unigene36544\_Mf\_liverA, Unigene36580\_Mf\_liverA, Unigene36581\_Mf\_liverA, Unigene36593\_Mf\_liverA, Unigene36606\_Mf\_liverA, Unigene36608\_Mf\_liverA, Unigene36616\_Mf\_liverA, Unigene36617\_Mf\_liverA, Unigene36624\_Mf\_liverA, Unigene36626\_Mf\_liverA, Unigene36627\_Mf\_liverA, Unigene36628\_Mf\_liverA, Unigene36630\_Mf\_liverA, Unigene36638\_Mf\_liverA, Unigene36640\_Mf\_liverA, Unigene36641\_Mf\_liverA, Unigene36645\_Mf\_liverA, Unigene36646\_Mf\_liverA, Unigene36647\_Mf\_liverA, Unigene36652\_Mf\_liverA, Unigene36653\_Mf\_liverA, Unigene36655\_Mf\_liverA, Unigene36661\_Mf\_liverA, Unigene36662\_Mf\_liverA, Unigene36664\_Mf\_liverA, Unigene36666\_Mf\_liverA, Unigene36671\_Mf\_liverA, Unigene36674\_Mf\_liverA, Unigene36675\_Mf\_liverA, Unigene36678\_Mf\_liverA, Unigene36682\_Mf\_liverA, Unigene36683\_Mf\_liverA, Unigene36684\_Mf\_liverA, Unigene36686\_Mf\_liverA, Unigene36687\_Mf\_liverA, Unigene36690\_Mf\_liverA, Unigene36691\_Mf\_liverA, Unigene36694\_Mf\_liverA, Unigene36698\_Mf\_liverA, Unigene36699\_Mf\_liverA, Unigene36703\_Mf\_liverA, Unigene36704\_Mf\_liverA, Unigene36707\_Mf\_liverA, Unigene36714\_Mf\_liverA, Unigene36716\_Mf\_liverA, Unigene36717\_Mf\_liverA, Unigene36718\_Mf\_liverA, Unigene36721\_Mf\_liverA, Unigene36722\_Mf\_liverA, Unigene36723\_Mf\_liverA, Unigene36724\_Mf\_liverA, Unigene36725\_Mf\_liverA, Unigene36726\_Mf\_liverA, Unigene36727\_Mf\_liverA, Unigene36730\_Mf\_liverA, Unigene36731\_Mf\_liverA, Unigene36733\_Mf\_liverA, Unigene36736\_Mf\_liverA, Unigene36737\_Mf\_liverA, Unigene36738\_Mf\_liverA, Unigene36740\_Mf\_liverA, Unigene36743\_Mf\_liverA, Unigene36749\_Mf\_liverA, Unigene36753\_Mf\_liverA, Unigene36754\_Mf\_liverA, Unigene36758\_Mf\_liverA, Unigene36759\_Mf\_liverA, Unigene36761\_Mf\_liverA, Unigene36764\_Mf\_liverA, Unigene36765\_Mf\_liverA, Unigene36771\_Mf\_liverA, Unigene36776\_Mf\_liverA, Unigene36777\_Mf\_liverA, Unigene36779\_Mf\_liverA, Unigene36780\_Mf\_liverA, Unigene36783\_Mf\_liverA, Unigene36790\_Mf\_liverA, Unigene36795\_Mf\_liverA, Unigene36797\_Mf\_liverA, Unigene36798\_Mf\_liverA, Unigene367\_Mf\_liverA, Unigene36801\_Mf\_liverA, Unigene36811\_Mf\_liverA, Unigene36820\_Mf\_liverA, Unigene36828\_Mf\_liverA, Unigene36830\_Mf\_liverA, Unigene36833\_Mf\_liverA, Unigene36835\_Mf\_liverA, Unigene36837\_Mf\_liverA, Unigene36838\_Mf\_liverA, Unigene36850\_Mf\_liverA, Unigene36855\_Mf\_liverA, Unigene36864\_Mf\_liverA, Unigene36866\_Mf\_liverA, Unigene36872\_Mf\_liverA, Unigene36876\_Mf\_liverA, Unigene3687\_Mf\_liverA, Unigene36880\_Mf\_liverA, Unigene36882\_Mf\_liverA, Unigene36897\_Mf\_liverA, Unigene36905\_Mf\_liverA, Unigene36913\_Mf\_liverA, Unigene36923\_Mf\_liverA, Unigene36925\_Mf\_liverA, Unigene36931\_Mf\_liverA, Unigene36932\_Mf\_liverA, Unigene36960\_Mf\_liverA, Unigene36962\_Mf\_liverA, Unigene36974\_Mf\_liverA, Unigene36977\_Mf\_liverA, Unigene36982\_Mf\_liverA, Unigene36991\_Mf\_liverA, Unigene36998\_Mf\_liverA, Unigene37003\_Mf\_liverA, Unigene37013\_Mf\_liverA, Unigene37018\_Mf\_liverA, Unigene37023\_Mf\_liverA, Unigene37040\_Mf\_liverA, Unigene37051\_Mf\_liverA, Unigene37059\_Mf\_liverA, Unigene37068\_Mf\_liverA, Unigene37071\_Mf\_liverA, Unigene37089\_Mf\_liverA, Unigene37104\_Mf\_liverA, Unigene37105\_Mf\_liverA, Unigene37107\_Mf\_liverA, Unigene37122\_Mf\_liverA, Unigene37157\_Mf\_liverA, Unigene37170\_Mf\_liverA, Unigene37180\_Mf\_liverA, Unigene37197\_Mf\_liverA, Unigene371\_Mf\_liverA, Unigene37201\_Mf\_liverA, Unigene37205\_Mf\_liverA, Unigene37234\_Mf\_liverA, Unigene37259\_Mf\_liverA, Unigene37262\_Mf\_liverA, Unigene37282\_Mf\_liverA, Unigene37290\_Mf\_liverA, Unigene37295\_Mf\_liverA, Unigene37296\_Mf\_liverA, Unigene37344\_Mf\_liverA, Unigene37403\_Mf\_liverA, Unigene37408\_Mf\_liverA, Unigene37428\_Mf\_liverA, Unigene37433\_Mf\_liverA, Unigene37469\_Mf\_liverA, Unigene37472\_Mf\_liverA, Unigene37521\_Mf\_liverA, Unigene37542\_Mf\_liverA, Unigene37555\_Mf\_liverA, Unigene37566\_Mf\_liverA, Unigene37637\_Mf\_liverA, Unigene37669\_Mf\_liverA, Unigene37689\_Mf\_liverA, Unigene37734\_Mf\_liverA, Unigene37744\_Mf\_liverA, Unigene37788\_Mf\_liverA, Unigene37880\_Mf\_liverA, Unigene37973\_Mf\_liverA, Unigene38042\_Mf\_liverA, Unigene3810\_Mf\_liverA, Unigene3811\_Mf\_liverA, Unigene38280\_Mf\_liverA, Unigene3829\_Mf\_liverA, Unigene38331\_Mf\_liverA, Unigene38514\_Mf\_liverA, Unigene38594\_Mf\_liverA, Unigene38622\_Mf\_liverA, Unigene386\_Mf\_liverA, Unigene38829\_Mf\_liverA, Unigene388\_Mf\_liverA, Unigene38925\_Mf\_liverA, Unigene38977\_Mf\_liverA, Unigene39183\_Mf\_liverA, Unigene39397\_Mf\_liverA, Unigene39401\_Mf\_liverA, Unigene39424\_Mf\_liverA, Unigene39489\_Mf\_liverA, Unigene39609\_Mf\_liverA, Unigene39613\_Mf\_liverA, Unigene39626\_Mf\_liverA, Unigene39790\_Mf\_liverA, Unigene39936\_Mf\_liverA, Unigene39970\_Mf\_liverA, Unigene40038\_Mf\_liverA, Unigene40050\_Mf\_liverA, Unigene40136\_Mf\_liverA, Unigene40223\_Mf\_liverA, Unigene4023\_Mf\_liverA, Unigene40254\_Mf\_liverA, Unigene40263\_Mf\_liverA, Unigene4027\_Mf\_liverA, Unigene40322\_Mf\_liverA, Unigene40343\_Mf\_liverA, Unigene40421\_Mf\_liverA, Unigene40583\_Mf\_liverA, Unigene4069\_Mf\_liverA, Unigene4079\_Mf\_liverA, Unigene40915\_Mf\_liverA, Unigene40928\_Mf\_liverA, Unigene41030\_Mf\_liverA, Unigene41109\_Mf\_liverA, Unigene4118\_Mf\_liverA, Unigene41268\_Mf\_liverA, Unigene41384\_Mf\_liverA, Unigene41450\_Mf\_liverA, Unigene41494\_Mf\_liverA, Unigene41501\_Mf\_liverA, Unigene4170\_Mf\_liverA, Unigene41806\_Mf\_liverA, Unigene41877\_Mf\_liverA, Unigene41891\_Mf\_liverA, Unigene41957\_Mf\_liverA, Unigene41988\_Mf\_liverA, Unigene419\_Mf\_liverA, Unigene42040\_Mf\_liverA, Unigene42067\_Mf\_liverA, Unigene42109\_Mf\_liverA, Unigene42252\_Mf\_liverA, Unigene42253\_Mf\_liverA, Unigene42272\_Mf\_liverA, Unigene42497\_Mf\_liverA, Unigene42633\_Mf\_liverA, Unigene42716\_Mf\_liverA, Unigene42736\_Mf\_liverA, Unigene42812\_Mf\_liverA, Unigene42844\_Mf\_liverA, Unigene42924\_Mf\_liverA, Unigene42940\_Mf\_liverA, Unigene42988\_Mf\_liverA, Unigene43069\_Mf\_liverA, Unigene43157\_Mf\_liverA, Unigene43223\_Mf\_liverA, Unigene43419\_Mf\_liverA, Unigene43458\_Mf\_liverA, Unigene43528\_Mf\_liverA, Unigene43534\_Mf\_liverA, Unigene43757\_Mf\_liverA, Unigene43855\_Mf\_liverA, Unigene43863\_Mf\_liverA, Unigene44181\_Mf\_liverA, Unigene44198\_Mf\_liverA, Unigene44285\_Mf\_liverA, Unigene44359\_Mf\_liverA, Unigene44454\_Mf\_liverA, Unigene44492\_Mf\_liverA, Unigene44527\_Mf\_liverA, Unigene4460\_Mf\_liverA, Unigene44650\_Mf\_liverA, Unigene44768\_Mf\_liverA, Unigene44845\_Mf\_liverA, Unigene44912\_Mf\_liverA, Unigene44972\_Mf\_liverA, Unigene45113\_Mf\_liverA, Unigene4527\_Mf\_liverA, Unigene45370\_Mf\_liverA, Unigene45399\_Mf\_liverA, Unigene45409\_Mf\_liverA, Unigene45431\_Mf\_liverA, Unigene45511\_Mf\_liverA, Unigene45643\_Mf\_liverA, Unigene45807\_Mf\_liverA, Unigene46460\_Mf\_liverA, Unigene4654\_Mf\_liverA, Unigene46649\_Mf\_liverA, Unigene46705\_Mf\_liverA, Unigene46822\_Mf\_liverA, Unigene46996\_Mf\_liverA, Unigene4699\_Mf\_liverA, Unigene47034\_Mf\_liverA, Unigene47112\_Mf\_liverA, Unigene47212\_Mf\_liverA, Unigene4723\_Mf\_liverA, Unigene47348\_Mf\_liverA, Unigene47472\_Mf\_liverA, Unigene47554\_Mf\_liverA, Unigene4757\_Mf\_liverA, Unigene4779\_Mf\_liverA, Unigene47832\_Mf\_liverA, Unigene4791\_Mf\_liverA, Unigene47994\_Mf\_liverA, Unigene48160\_Mf\_liverA, Unigene4827\_Mf\_liverA, Unigene48387\_Mf\_liverA, Unigene48506\_Mf\_liverA, Unigene48816\_Mf\_liverA, Unigene4924\_Mf\_liverA, Unigene49430\_Mf\_liverA, Unigene49595\_Mf\_liverA, Unigene49840\_Mf\_liverA, Unigene4\_Mf\_liverA, Unigene50028\_Mf\_liverA, Unigene50200\_Mf\_liverA, Unigene50222\_Mf\_liverA, Unigene5022\_Mf\_liverA, Unigene50332\_Mf\_liverA, Unigene50557\_Mf\_liverA, Unigene50779\_Mf\_liverA, Unigene5082\_Mf\_liverA, Unigene50912\_Mf\_liverA, Unigene5100\_Mf\_liverA, Unigene5101\_Mf\_liverA, Unigene51097\_Mf\_liverA, Unigene51254\_Mf\_liverA, Unigene5130\_Mf\_liverA, Unigene5137\_Mf\_liverA, Unigene51426\_Mf\_liverA, Unigene51500\_Mf\_liverA, Unigene51568\_Mf\_liverA, Unigene5163\_Mf\_liverA, Unigene51671\_Mf\_liverA, Unigene51690\_Mf\_liverA, Unigene5170\_Mf\_liverA, Unigene5173\_Mf\_liverA, Unigene5174\_Mf\_liverA, Unigene5180\_Mf\_liverA, Unigene5212\_Mf\_liverA, Unigene5222\_Mf\_liverA, Unigene5233\_Mf\_liverA, Unigene5236\_Mf\_liverA, Unigene5260\_Mf\_liverA, Unigene5276\_Mf\_liverA, Unigene5277\_Mf\_liverA, Unigene5278\_Mf\_liverA, Unigene5280\_Mf\_liverA, Unigene5282\_Mf\_liverA, Unigene5284\_Mf\_liverA, Unigene5287\_Mf\_liverA, Unigene5290\_Mf\_liverA, Unigene5291\_Mf\_liverA, Unigene5293\_Mf\_liverA, Unigene5296\_Mf\_liverA, Unigene5299\_Mf\_liverA, Unigene5300\_Mf\_liverA, Unigene5309\_Mf\_liverA, Unigene5318\_Mf\_liverA, Unigene5319\_Mf\_liverA, Unigene5320\_Mf\_liverA, Unigene5322\_Mf\_liverA, Unigene5324\_Mf\_liverA, Unigene5326\_Mf\_liverA, Unigene5329\_Mf\_liverA, Unigene5333\_Mf\_liverA, Unigene5334\_Mf\_liverA, Unigene5340\_Mf\_liverA, Unigene5341\_Mf\_liverA, Unigene5342\_Mf\_liverA, Unigene5345\_Mf\_liverA, Unigene5346\_Mf\_liverA, Unigene5347\_Mf\_liverA, Unigene5351\_Mf\_liverA, Unigene5354\_Mf\_liverA, Unigene5359\_Mf\_liverA, Unigene5364\_Mf\_liverA, Unigene5365\_Mf\_liverA, Unigene5380\_Mf\_liverA, Unigene5397\_Mf\_liverA, Unigene5408\_Mf\_liverA, Unigene542\_Mf\_liverA, Unigene5430\_Mf\_liverA, Unigene5442\_Mf\_liverA, Unigene5445\_Mf\_liverA, Unigene5480\_Mf\_liverA, Unigene5495\_Mf\_liverA, Unigene5497\_Mf\_liverA, Unigene5506\_Mf\_liverA, Unigene5516\_Mf\_liverA, Unigene5525\_Mf\_liverA, Unigene5540\_Mf\_liverA, Unigene5543\_Mf\_liverA, Unigene5546\_Mf\_liverA, Unigene5555\_Mf\_liverA, Unigene5578\_Mf\_liverA, Unigene5585\_Mf\_liverA, Unigene5590\_Mf\_liverA, Unigene5617\_Mf\_liverA, Unigene5661\_Mf\_liverA, Unigene5677\_Mf\_liverA, Unigene5689\_Mf\_liverA, Unigene5692\_Mf\_liverA, Unigene5733\_Mf\_liverA, Unigene5735\_Mf\_liverA, Unigene5755\_Mf\_liverA, Unigene5760\_Mf\_liverA, Unigene5761\_Mf\_liverA, Unigene5763\_Mf\_liverA, Unigene5774\_Mf\_liverA, Unigene5775\_Mf\_liverA, Unigene5792\_Mf\_liverA, Unigene5795\_Mf\_liverA, Unigene5810\_Mf\_liverA, Unigene5869\_Mf\_liverA, Unigene5872\_Mf\_liverA, Unigene5873\_Mf\_liverA, Unigene5874\_Mf\_liverA, Unigene5879\_Mf\_liverA, Unigene5884\_Mf\_liverA, Unigene5890\_Mf\_liverA, Unigene5891\_Mf\_liverA, Unigene5892\_Mf\_liverA, Unigene5899\_Mf\_liverA, Unigene6009\_Mf\_liverA, Unigene6110\_Mf\_liverA, Unigene6191\_Mf\_liverA, Unigene6221\_Mf\_liverA, Unigene6338\_Mf\_liverA, Unigene6344\_Mf\_liverA, Unigene6351\_Mf\_liverA, Unigene6541\_Mf\_liverA, Unigene6555\_Mf\_liverA, Unigene6594\_Mf\_liverA, Unigene663\_Mf\_liverA, Unigene674\_Mf\_liverA, Unigene6778\_Mf\_liverA, Unigene67\_Mf\_liverA, Unigene6824\_Mf\_liverA, Unigene688\_Mf\_liverA, Unigene689\_Mf\_liverA, Unigene6906\_Mf\_liverA, Unigene6967\_Mf\_liverA, Unigene6968\_Mf\_liverA, Unigene6976\_Mf\_liverA, Unigene6990\_Mf\_liverA, Unigene6996\_Mf\_liverA, Unigene7022\_Mf\_liverA, Unigene7028\_Mf\_liverA, Unigene7048\_Mf\_liverA, Unigene7078\_Mf\_liverA, Unigene7079\_Mf\_liverA, Unigene7110\_Mf\_liverA, Unigene7116\_Mf\_liverA, Unigene7169\_Mf\_liverA, Unigene7195\_Mf\_liverA, Unigene7204\_Mf\_liverA, Unigene7260\_Mf\_liverA, Unigene7261\_Mf\_liverA, Unigene7336\_Mf\_liverA, Unigene7347\_Mf\_liverA, Unigene7385\_Mf\_liverA, Unigene7395\_Mf\_liverA, Unigene7484\_Mf\_liverA, Unigene7491\_Mf\_liverA, Unigene7495\_Mf\_liverA, Unigene7512\_Mf\_liverA, Unigene7513\_Mf\_liverA, Unigene7536\_Mf\_liverA, Unigene7567\_Mf\_liverA, Unigene762\_Mf\_liverA, Unigene7662\_Mf\_liverA, Unigene7704\_Mf\_liverA, Unigene7740\_Mf\_liverA, Unigene7797\_Mf\_liverA, Unigene7806\_Mf\_liverA, Unigene7807\_Mf\_liverA, Unigene7812\_Mf\_liverA, Unigene7897\_Mf\_liverA, Unigene7970\_Mf\_liverA, Unigene803\_Mf\_liverA, Unigene807\_Mf\_liverA, Unigene8102\_Mf\_liverA, Unigene8146\_Mf\_liverA, Unigene8170\_Mf\_liverA, Unigene8229\_Mf\_liverA, Unigene825\_Mf\_liverA, Unigene827\_Mf\_liverA, Unigene834\_Mf\_liverA, Unigene8469\_Mf\_liverA, Unigene8552\_Mf\_liverA, Unigene8606\_Mf\_liverA, Unigene8764\_Mf\_liverA, Unigene87\_Mf\_liverA, Unigene8831\_Mf\_liverA, Unigene8907\_Mf\_liverA, Unigene8908\_Mf\_liverA, Unigene8917\_Mf\_liverA, Unigene908\_Mf\_liverA, Unigene9398\_Mf\_liverA, Unigene9409\_Mf\_liverA, Unigene9416\_Mf\_liverA, Unigene9487\_Mf\_liverA, Unigene951\_Mf\_liverA, Unigene9541\_Mf\_liverA, Unigene9563\_Mf\_liverA, Unigene9650\_Mf\_liverA, Unigene9682\_Mf\_liverA, Unigene9709\_Mf\_liverA, Unigene9759\_Mf\_liverA, Unigene9807\_Mf\_liverA, Unigene989\_Mf\_liverA |
| 2 | Pathways in cancer | CL1005.Contig1\_Mf\_liverA, CL1005.Contig2\_Mf\_liverA, CL1061.Contig1\_Mf\_liverA, CL1197.Contig1\_Mf\_liverA, CL1197.Contig2\_Mf\_liverA, CL1207.Contig1\_Mf\_liverA, CL1207.Contig2\_Mf\_liverA, CL1232.Contig1\_Mf\_liverA, CL1232.Contig2\_Mf\_liverA, CL1278.Contig1\_Mf\_liverA, CL1278.Contig2\_Mf\_liverA, CL1291.Contig1\_Mf\_liverA, CL1291.Contig2\_Mf\_liverA, CL1362.Contig1\_Mf\_liverA, CL1362.Contig2\_Mf\_liverA, CL1379.Contig1\_Mf\_liverA, CL1379.Contig2\_Mf\_liverA, CL1379.Contig3\_Mf\_liverA, CL1379.Contig4\_Mf\_liverA, CL1380.Contig1\_Mf\_liverA, CL1381.Contig2\_Mf\_liverA, CL1381.Contig3\_Mf\_liverA, CL1475.Contig1\_Mf\_liverA, CL1475.Contig2\_Mf\_liverA, CL1552.Contig1\_Mf\_liverA, CL1553.Contig1\_Mf\_liverA, CL1553.Contig2\_Mf\_liverA, CL1553.Contig3\_Mf\_liverA, CL1553.Contig4\_Mf\_liverA, CL1553.Contig5\_Mf\_liverA, CL1567.Contig10\_Mf\_liverA, CL1567.Contig11\_Mf\_liverA, CL1567.Contig12\_Mf\_liverA, CL1567.Contig13\_Mf\_liverA, CL1567.Contig14\_Mf\_liverA, CL1567.Contig15\_Mf\_liverA, CL1567.Contig1\_Mf\_liverA, CL1567.Contig2\_Mf\_liverA, CL1567.Contig3\_Mf\_liverA, CL1567.Contig4\_Mf\_liverA, CL1567.Contig5\_Mf\_liverA, CL1567.Contig6\_Mf\_liverA, CL1567.Contig7\_Mf\_liverA, CL1567.Contig8\_Mf\_liverA, CL1567.Contig9\_Mf\_liverA, CL1575.Contig1\_Mf\_liverA, CL1575.Contig2\_Mf\_liverA, CL1653.Contig1\_Mf\_liverA, CL1653.Contig2\_Mf\_liverA, CL1659.Contig1\_Mf\_liverA, CL1659.Contig2\_Mf\_liverA, CL1685.Contig1\_Mf\_liverA, CL1685.Contig2\_Mf\_liverA, CL1685.Contig3\_Mf\_liverA, CL1685.Contig4\_Mf\_liverA, CL1685.Contig5\_Mf\_liverA, CL1685.Contig6\_Mf\_liverA, CL1685.Contig7\_Mf\_liverA, CL1685.Contig8\_Mf\_liverA, CL1687.Contig1\_Mf\_liverA, CL1687.Contig2\_Mf\_liverA, CL1690.Contig1\_Mf\_liverA, CL1690.Contig2\_Mf\_liverA, CL1690.Contig3\_Mf\_liverA, CL1690.Contig4\_Mf\_liverA, CL1730.Contig1\_Mf\_liverA, CL1730.Contig2\_Mf\_liverA, CL1805.Contig1\_Mf\_liverA, CL1805.Contig2\_Mf\_liverA, CL1810.Contig1\_Mf\_liverA, CL1810.Contig2\_Mf\_liverA, CL1837.Contig1\_Mf\_liverA, CL1837.Contig2\_Mf\_liverA, CL1837.Contig3\_Mf\_liverA, CL1846.Contig2\_Mf\_liverA, CL1877.Contig1\_Mf\_liverA, CL1877.Contig2\_Mf\_liverA, CL1877.Contig3\_Mf\_liverA, CL1877.Contig4\_Mf\_liverA, CL1900.Contig1\_Mf\_liverA, CL1900.Contig2\_Mf\_liverA, CL1955.Contig1\_Mf\_liverA, CL1955.Contig2\_Mf\_liverA, CL1959.Contig1\_Mf\_liverA, CL1959.Contig2\_Mf\_liverA, CL1959.Contig3\_Mf\_liverA, CL1959.Contig4\_Mf\_liverA, CL1959.Contig5\_Mf\_liverA, CL1961.Contig1\_Mf\_liverA, CL1961.Contig2\_Mf\_liverA, CL1994.Contig1\_Mf\_liverA, CL1994.Contig2\_Mf\_liverA, CL2007.Contig1\_Mf\_liverA, CL2007.Contig2\_Mf\_liverA, CL2007.Contig3\_Mf\_liverA, CL2086.Contig1\_Mf\_liverA, CL210.Contig2\_Mf\_liverA, CL2117.Contig1\_Mf\_liverA, CL2117.Contig2\_Mf\_liverA, CL2128.Contig1\_Mf\_liverA, CL2128.Contig2\_Mf\_liverA, CL2128.Contig3\_Mf\_liverA, CL2128.Contig4\_Mf\_liverA, CL2130.Contig1\_Mf\_liverA, CL2130.Contig2\_Mf\_liverA, CL2130.Contig3\_Mf\_liverA, CL2134.Contig1\_Mf\_liverA, CL2134.Contig2\_Mf\_liverA, CL2263.Contig1\_Mf\_liverA, CL2263.Contig2\_Mf\_liverA, CL2285.Contig1\_Mf\_liverA, CL2285.Contig2\_Mf\_liverA, CL2285.Contig3\_Mf\_liverA, CL2307.Contig1\_Mf\_liverA, CL2307.Contig2\_Mf\_liverA, CL2355.Contig1\_Mf\_liverA, CL2355.Contig2\_Mf\_liverA, CL237.Contig1\_Mf\_liverA, CL237.Contig2\_Mf\_liverA, CL237.Contig3\_Mf\_liverA, CL239.Contig2\_Mf\_liverA, CL2404.Contig10\_Mf\_liverA, CL2404.Contig11\_Mf\_liverA, CL2404.Contig12\_Mf\_liverA, CL2404.Contig13\_Mf\_liverA, CL2404.Contig14\_Mf\_liverA, CL2404.Contig15\_Mf\_liverA, CL2404.Contig1\_Mf\_liverA, CL2404.Contig2\_Mf\_liverA, CL2404.Contig3\_Mf\_liverA, CL2404.Contig4\_Mf\_liverA, CL2404.Contig5\_Mf\_liverA, CL2404.Contig6\_Mf\_liverA, CL2404.Contig7\_Mf\_liverA, CL2404.Contig8\_Mf\_liverA, CL2404.Contig9\_Mf\_liverA, CL2405.Contig1\_Mf\_liverA, CL2405.Contig2\_Mf\_liverA, CL2423.Contig1\_Mf\_liverA, CL2423.Contig2\_Mf\_liverA, CL2434.Contig1\_Mf\_liverA, CL2434.Contig2\_Mf\_liverA, CL2434.Contig3\_Mf\_liverA, CL2434.Contig4\_Mf\_liverA, CL2434.Contig5\_Mf\_liverA, CL2478.Contig1\_Mf\_liverA, CL2478.Contig2\_Mf\_liverA, CL2478.Contig3\_Mf\_liverA, CL2500.Contig1\_Mf\_liverA, CL2500.Contig2\_Mf\_liverA, CL2500.Contig3\_Mf\_liverA, CL2500.Contig4\_Mf\_liverA, CL2500.Contig5\_Mf\_liverA, CL2500.Contig6\_Mf\_liverA, CL2500.Contig7\_Mf\_liverA, CL2500.Contig8\_Mf\_liverA, CL2520.Contig1\_Mf\_liverA, CL2520.Contig2\_Mf\_liverA, CL2546.Contig1\_Mf\_liverA, CL2546.Contig2\_Mf\_liverA, CL2557.Contig1\_Mf\_liverA, CL2557.Contig2\_Mf\_liverA, CL2578.Contig1\_Mf\_liverA, CL2592.Contig1\_Mf\_liverA, CL2592.Contig2\_Mf\_liverA, CL2592.Contig3\_Mf\_liverA, CL2599.Contig1\_Mf\_liverA, CL2599.Contig2\_Mf\_liverA, CL2599.Contig3\_Mf\_liverA, CL260.Contig1\_Mf\_liverA, CL260.Contig2\_Mf\_liverA, CL2638.Contig1\_Mf\_liverA, CL2638.Contig2\_Mf\_liverA, CL2638.Contig3\_Mf\_liverA, CL2638.Contig4\_Mf\_liverA, CL2664.Contig1\_Mf\_liverA, CL2664.Contig2\_Mf\_liverA, CL2664.Contig3\_Mf\_liverA, CL2664.Contig4\_Mf\_liverA, CL2770.Contig1\_Mf\_liverA, CL2770.Contig2\_Mf\_liverA, CL2789.Contig1\_Mf\_liverA, CL2789.Contig2\_Mf\_liverA, CL2876.Contig1\_Mf\_liverA, CL296.Contig1\_Mf\_liverA, CL296.Contig2\_Mf\_liverA, CL2969.Contig1\_Mf\_liverA, CL2969.Contig2\_Mf\_liverA, CL2993.Contig1\_Mf\_liverA, CL2993.Contig2\_Mf\_liverA, CL2994.Contig1\_Mf\_liverA, CL2994.Contig2\_Mf\_liverA, CL2996.Contig10\_Mf\_liverA, CL2996.Contig11\_Mf\_liverA, CL2996.Contig1\_Mf\_liverA, CL2996.Contig2\_Mf\_liverA, CL2996.Contig3\_Mf\_liverA, CL2996.Contig4\_Mf\_liverA, CL2996.Contig5\_Mf\_liverA, CL2996.Contig6\_Mf\_liverA, CL2996.Contig7\_Mf\_liverA, CL2996.Contig8\_Mf\_liverA, CL2996.Contig9\_Mf\_liverA, CL3007.Contig1\_Mf\_liverA, CL3053.Contig1\_Mf\_liverA, CL3066.Contig1\_Mf\_liverA, CL3066.Contig2\_Mf\_liverA, CL3147.Contig1\_Mf\_liverA, CL3155.Contig1\_Mf\_liverA, CL3155.Contig2\_Mf\_liverA, CL3168.Contig1\_Mf\_liverA, CL3168.Contig2\_Mf\_liverA, CL3220.Contig1\_Mf\_liverA, CL3220.Contig2\_Mf\_liverA, CL3220.Contig3\_Mf\_liverA, CL3220.Contig4\_Mf\_liverA, CL3243.Contig1\_Mf\_liverA, CL3243.Contig2\_Mf\_liverA, CL3259.Contig1\_Mf\_liverA, CL3259.Contig2\_Mf\_liverA, CL3548.Contig1\_Mf\_liverA, CL3607.Contig1\_Mf\_liverA, CL3607.Contig2\_Mf\_liverA, CL3616.Contig1\_Mf\_liverA, CL3616.Contig2\_Mf\_liverA, CL3623.Contig1\_Mf\_liverA, CL3623.Contig2\_Mf\_liverA, CL3623.Contig3\_Mf\_liverA, CL3719.Contig1\_Mf\_liverA, CL3719.Contig2\_Mf\_liverA, CL3738.Contig1\_Mf\_liverA, CL3738.Contig2\_Mf\_liverA, CL3786.Contig1\_Mf\_liverA, CL3786.Contig2\_Mf\_liverA, CL3800.Contig1\_Mf\_liverA, CL3800.Contig2\_Mf\_liverA, CL3934.Contig1\_Mf\_liverA, CL4012.Contig1\_Mf\_liverA, CL4012.Contig2\_Mf\_liverA, CL4012.Contig3\_Mf\_liverA, CL4014.Contig1\_Mf\_liverA, CL4015.Contig1\_Mf\_liverA, CL4015.Contig2\_Mf\_liverA, CL4038.Contig2\_Mf\_liverA, CL4076.Contig1\_Mf\_liverA, CL410.Contig13\_Mf\_liverA, CL410.Contig14\_Mf\_liverA, CL410.Contig15\_Mf\_liverA, CL410.Contig16\_Mf\_liverA, CL410.Contig17\_Mf\_liverA, CL410.Contig9\_Mf\_liverA, CL4123.Contig2\_Mf\_liverA, CL4130.Contig1\_Mf\_liverA, CL4130.Contig2\_Mf\_liverA, CL4154.Contig1\_Mf\_liverA, CL4154.Contig2\_Mf\_liverA, CL4208.Contig1\_Mf\_liverA, CL4208.Contig2\_Mf\_liverA, CL4281.Contig2\_Mf\_liverA, CL433.Contig2\_Mf\_liverA, CL4407.Contig1\_Mf\_liverA, CL4407.Contig2\_Mf\_liverA, CL4447.Contig1\_Mf\_liverA, CL4447.Contig2\_Mf\_liverA, CL4447.Contig3\_Mf\_liverA, CL4447.Contig4\_Mf\_liverA, CL4507.Contig1\_Mf\_liverA, CL452.Contig4\_Mf\_liverA, CL4587.Contig1\_Mf\_liverA, CL4587.Contig2\_Mf\_liverA, CL4598.Contig4\_Mf\_liverA, CL4618.Contig1\_Mf\_liverA, CL4618.Contig2\_Mf\_liverA, CL4643.Contig1\_Mf\_liverA, CL4643.Contig2\_Mf\_liverA, CL4643.Contig3\_Mf\_liverA, CL4643.Contig4\_Mf\_liverA, CL4643.Contig5\_Mf\_liverA, CL4643.Contig6\_Mf\_liverA, CL4664.Contig1\_Mf\_liverA, CL4664.Contig2\_Mf\_liverA, CL4665.Contig1\_Mf\_liverA, CL4697.Contig1\_Mf\_liverA, CL4722.Contig1\_Mf\_liverA, CL4722.Contig2\_Mf\_liverA, CL4757.Contig1\_Mf\_liverA, CL4757.Contig2\_Mf\_liverA, CL4784.Contig2\_Mf\_liverA, CL4846.Contig1\_Mf\_liverA, CL4846.Contig2\_Mf\_liverA, CL4848.Contig1\_Mf\_liverA, CL4848.Contig2\_Mf\_liverA, CL4870.Contig1\_Mf\_liverA, CL4901.Contig1\_Mf\_liverA, CL4901.Contig2\_Mf\_liverA, CL4919.Contig1\_Mf\_liverA, CL4919.Contig2\_Mf\_liverA, CL4936.Contig1\_Mf\_liverA, CL4936.Contig2\_Mf\_liverA, CL4952.Contig1\_Mf\_liverA, CL4952.Contig2\_Mf\_liverA, CL5022.Contig1\_Mf\_liverA, CL5022.Contig2\_Mf\_liverA, CL5036.Contig1\_Mf\_liverA, CL5036.Contig2\_Mf\_liverA, CL5138.Contig1\_Mf\_liverA, CL5138.Contig2\_Mf\_liverA, CL5138.Contig3\_Mf\_liverA, CL5184.Contig1\_Mf\_liverA, CL5219.Contig1\_Mf\_liverA, CL5254.Contig1\_Mf\_liverA, CL5254.Contig2\_Mf\_liverA, CL5278.Contig1\_Mf\_liverA, CL5278.Contig2\_Mf\_liverA, CL5405.Contig1\_Mf\_liverA, CL5405.Contig2\_Mf\_liverA, CL5416.Contig1\_Mf\_liverA, CL5416.Contig2\_Mf\_liverA, CL5595.Contig1\_Mf\_liverA, CL5595.Contig2\_Mf\_liverA, CL5655.Contig1\_Mf\_liverA, CL5655.Contig2\_Mf\_liverA, CL5692.Contig1\_Mf\_liverA, CL5692.Contig2\_Mf\_liverA, CL5736.Contig1\_Mf\_liverA, CL5736.Contig2\_Mf\_liverA, CL574.Contig1\_Mf\_liverA, CL574.Contig2\_Mf\_liverA, CL5955.Contig1\_Mf\_liverA, CL6034.Contig1\_Mf\_liverA, CL6034.Contig2\_Mf\_liverA, CL683.Contig1\_Mf\_liverA, CL683.Contig2\_Mf\_liverA, CL683.Contig3\_Mf\_liverA, CL683.Contig4\_Mf\_liverA, CL703.Contig1\_Mf\_liverA, CL704.Contig1\_Mf\_liverA, CL704.Contig2\_Mf\_liverA, CL712.Contig1\_Mf\_liverA, CL712.Contig2\_Mf\_liverA, CL712.Contig3\_Mf\_liverA, CL712.Contig4\_Mf\_liverA, CL712.Contig5\_Mf\_liverA, CL712.Contig6\_Mf\_liverA, CL712.Contig7\_Mf\_liverA, CL712.Contig8\_Mf\_liverA, CL72.Contig10\_Mf\_liverA, CL72.Contig11\_Mf\_liverA, CL72.Contig12\_Mf\_liverA, CL72.Contig13\_Mf\_liverA, CL72.Contig14\_Mf\_liverA, CL72.Contig15\_Mf\_liverA, CL72.Contig16\_Mf\_liverA, CL72.Contig17\_Mf\_liverA, CL72.Contig18\_Mf\_liverA, CL72.Contig19\_Mf\_liverA, CL72.Contig1\_Mf\_liverA, CL72.Contig20\_Mf\_liverA, CL72.Contig21\_Mf\_liverA, CL72.Contig22\_Mf\_liverA, CL72.Contig23\_Mf\_liverA, CL72.Contig24\_Mf\_liverA, CL72.Contig25\_Mf\_liverA, CL72.Contig26\_Mf\_liverA, CL72.Contig27\_Mf\_liverA, CL72.Contig28\_Mf\_liverA, CL72.Contig29\_Mf\_liverA, CL72.Contig2\_Mf\_liverA, CL72.Contig30\_Mf\_liverA, CL72.Contig31\_Mf\_liverA, CL72.Contig32\_Mf\_liverA, CL72.Contig33\_Mf\_liverA, CL72.Contig34\_Mf\_liverA, CL72.Contig35\_Mf\_liverA, CL72.Contig36\_Mf\_liverA, CL72.Contig37\_Mf\_liverA, CL72.Contig38\_Mf\_liverA, CL72.Contig39\_Mf\_liverA, CL72.Contig3\_Mf\_liverA, CL72.Contig40\_Mf\_liverA, CL72.Contig41\_Mf\_liverA, CL72.Contig42\_Mf\_liverA, CL72.Contig43\_Mf\_liverA, CL72.Contig45\_Mf\_liverA, CL72.Contig46\_Mf\_liverA, CL72.Contig47\_Mf\_liverA, CL72.Contig48\_Mf\_liverA, CL72.Contig49\_Mf\_liverA, CL72.Contig4\_Mf\_liverA, CL72.Contig50\_Mf\_liverA, CL72.Contig51\_Mf\_liverA, CL72.Contig52\_Mf\_liverA, CL72.Contig5\_Mf\_liverA, CL72.Contig6\_Mf\_liverA, CL72.Contig7\_Mf\_liverA, CL72.Contig8\_Mf\_liverA, CL72.Contig9\_Mf\_liverA, CL838.Contig1\_Mf\_liverA, CL838.Contig2\_Mf\_liverA, CL838.Contig3\_Mf\_liverA, CL838.Contig4\_Mf\_liverA, CL838.Contig5\_Mf\_liverA, CL838.Contig6\_Mf\_liverA, CL839.Contig1\_Mf\_liverA, CL839.Contig2\_Mf\_liverA, CL864.Contig2\_Mf\_liverA, CL864.Contig3\_Mf\_liverA, CL890.Contig1\_Mf\_liverA, CL890.Contig2\_Mf\_liverA, CL892.Contig1\_Mf\_liverA, CL892.Contig2\_Mf\_liverA, CL892.Contig3\_Mf\_liverA, CL893.Contig1\_Mf\_liverA, CL893.Contig3\_Mf\_liverA, CL932.Contig5\_Mf\_liverA, CL932.Contig6\_Mf\_liverA, CL932.Contig9\_Mf\_liverA, CL951.Contig1\_Mf\_liverA, CL951.Contig2\_Mf\_liverA, CL98.Contig1\_Mf\_liverA, CL98.Contig2\_Mf\_liverA, CL98.Contig3\_Mf\_liverA, CL98.Contig4\_Mf\_liverA, CL988.Contig1\_Mf\_liverA, CL988.Contig2\_Mf\_liverA, CL988.Contig3\_Mf\_liverA, CL988.Contig4\_Mf\_liverA, Unigene10139\_Mf\_liverA, Unigene10249\_Mf\_liverA, Unigene10335\_Mf\_liverA, Unigene10448\_Mf\_liverA, Unigene10589\_Mf\_liverA, Unigene10681\_Mf\_liverA, Unigene10713\_Mf\_liverA, Unigene10820\_Mf\_liverA, Unigene11007\_Mf\_liverA, Unigene11008\_Mf\_liverA, Unigene11092\_Mf\_liverA, Unigene11221\_Mf\_liverA, Unigene1129\_Mf\_liverA, Unigene11662\_Mf\_liverA, Unigene1168\_Mf\_liverA, Unigene11737\_Mf\_liverA, Unigene11782\_Mf\_liverA, Unigene11865\_Mf\_liverA, Unigene12116\_Mf\_liverA, Unigene12117\_Mf\_liverA, Unigene12161\_Mf\_liverA, Unigene12256\_Mf\_liverA, Unigene12262\_Mf\_liverA, Unigene12263\_Mf\_liverA, Unigene12276\_Mf\_liverA, Unigene12277\_Mf\_liverA, Unigene12466\_Mf\_liverA, Unigene12525\_Mf\_liverA, Unigene12623\_Mf\_liverA, Unigene12713\_Mf\_liverA, Unigene12836\_Mf\_liverA, Unigene12877\_Mf\_liverA, Unigene12930\_Mf\_liverA, Unigene12932\_Mf\_liverA, Unigene13062\_Mf\_liverA, Unigene13085\_Mf\_liverA, Unigene1309\_Mf\_liverA, Unigene13170\_Mf\_liverA, Unigene13171\_Mf\_liverA, Unigene13201\_Mf\_liverA, Unigene13379\_Mf\_liverA, Unigene13408\_Mf\_liverA, Unigene13533\_Mf\_liverA, Unigene13695\_Mf\_liverA, Unigene13769\_Mf\_liverA, Unigene14094\_Mf\_liverA, Unigene14154\_Mf\_liverA, Unigene14329\_Mf\_liverA, Unigene14377\_Mf\_liverA, Unigene14378\_Mf\_liverA, Unigene14452\_Mf\_liverA, Unigene14484\_Mf\_liverA, Unigene14588\_Mf\_liverA, Unigene14774\_Mf\_liverA, Unigene14907\_Mf\_liverA, Unigene15040\_Mf\_liverA, Unigene15074\_Mf\_liverA, Unigene15075\_Mf\_liverA, Unigene15076\_Mf\_liverA, Unigene15125\_Mf\_liverA, Unigene15161\_Mf\_liverA, Unigene15186\_Mf\_liverA, Unigene1522\_Mf\_liverA, Unigene152\_Mf\_liverA, Unigene15423\_Mf\_liverA, Unigene15463\_Mf\_liverA, Unigene15464\_Mf\_liverA, Unigene1549\_Mf\_liverA, Unigene15730\_Mf\_liverA, Unigene15845\_Mf\_liverA, Unigene15846\_Mf\_liverA, Unigene15869\_Mf\_liverA, Unigene15874\_Mf\_liverA, Unigene15878\_Mf\_liverA, Unigene16062\_Mf\_liverA, Unigene16240\_Mf\_liverA, Unigene16241\_Mf\_liverA, Unigene16285\_Mf\_liverA, Unigene16310\_Mf\_liverA, Unigene16311\_Mf\_liverA, Unigene16819\_Mf\_liverA, Unigene16820\_Mf\_liverA, Unigene16849\_Mf\_liverA, Unigene16890\_Mf\_liverA, Unigene16922\_Mf\_liverA, Unigene16923\_Mf\_liverA, Unigene16924\_Mf\_liverA, Unigene16925\_Mf\_liverA, Unigene17298\_Mf\_liverA, Unigene17425\_Mf\_liverA, Unigene17426\_Mf\_liverA, Unigene17499\_Mf\_liverA, Unigene17518\_Mf\_liverA, Unigene17696\_Mf\_liverA, Unigene17855\_Mf\_liverA, Unigene18120\_Mf\_liverA, Unigene18129\_Mf\_liverA, Unigene18167\_Mf\_liverA, Unigene18168\_Mf\_liverA, Unigene18177\_Mf\_liverA, Unigene18385\_Mf\_liverA, Unigene18428\_Mf\_liverA, Unigene18653\_Mf\_liverA, Unigene18655\_Mf\_liverA, Unigene18722\_Mf\_liverA, Unigene18723\_Mf\_liverA, Unigene18826\_Mf\_liverA, Unigene18847\_Mf\_liverA, Unigene19017\_Mf\_liverA, Unigene19257\_Mf\_liverA, Unigene19258\_Mf\_liverA, Unigene19260\_Mf\_liverA, Unigene19261\_Mf\_liverA, Unigene19297\_Mf\_liverA, Unigene19353\_Mf\_liverA, Unigene19472\_Mf\_liverA, Unigene19473\_Mf\_liverA, Unigene19665\_Mf\_liverA, Unigene19687\_Mf\_liverA, Unigene19739\_Mf\_liverA, Unigene19795\_Mf\_liverA, Unigene19831\_Mf\_liverA, Unigene19885\_Mf\_liverA, Unigene19893\_Mf\_liverA, Unigene19894\_Mf\_liverA, Unigene20022\_Mf\_liverA, Unigene20262\_Mf\_liverA, Unigene20377\_Mf\_liverA, Unigene20378\_Mf\_liverA, Unigene20379\_Mf\_liverA, Unigene20696\_Mf\_liverA, Unigene20700\_Mf\_liverA, Unigene20720\_Mf\_liverA, Unigene20776\_Mf\_liverA, Unigene20795\_Mf\_liverA, Unigene20896\_Mf\_liverA, Unigene20978\_Mf\_liverA, Unigene21071\_Mf\_liverA, Unigene21224\_Mf\_liverA, Unigene21225\_Mf\_liverA, Unigene21284\_Mf\_liverA, Unigene21416\_Mf\_liverA, Unigene21481\_Mf\_liverA, Unigene21482\_Mf\_liverA, Unigene21483\_Mf\_liverA, Unigene21484\_Mf\_liverA, Unigene21500\_Mf\_liverA, Unigene21621\_Mf\_liverA, Unigene21840\_Mf\_liverA, Unigene21857\_Mf\_liverA, Unigene21991\_Mf\_liverA, Unigene2226\_Mf\_liverA, Unigene22381\_Mf\_liverA, Unigene22852\_Mf\_liverA, Unigene22859\_Mf\_liverA, Unigene22860\_Mf\_liverA, Unigene22919\_Mf\_liverA, Unigene23108\_Mf\_liverA, Unigene23110\_Mf\_liverA, Unigene23111\_Mf\_liverA, Unigene23118\_Mf\_liverA, Unigene23129\_Mf\_liverA, Unigene23130\_Mf\_liverA, Unigene23158\_Mf\_liverA, Unigene23224\_Mf\_liverA, Unigene23334\_Mf\_liverA, Unigene23341\_Mf\_liverA, Unigene23458\_Mf\_liverA, Unigene23560\_Mf\_liverA, Unigene2361\_Mf\_liverA, Unigene2377\_Mf\_liverA, Unigene23\_Mf\_liverA, Unigene24065\_Mf\_liverA, Unigene24068\_Mf\_liverA, Unigene24069\_Mf\_liverA, Unigene24142\_Mf\_liverA, Unigene24350\_Mf\_liverA, Unigene2435\_Mf\_liverA, Unigene2455\_Mf\_liverA, Unigene24576\_Mf\_liverA, Unigene24704\_Mf\_liverA, Unigene24804\_Mf\_liverA, Unigene24853\_Mf\_liverA, Unigene24986\_Mf\_liverA, Unigene24987\_Mf\_liverA, Unigene24988\_Mf\_liverA, Unigene25049\_Mf\_liverA, Unigene25119\_Mf\_liverA, Unigene25162\_Mf\_liverA, Unigene25171\_Mf\_liverA, Unigene25187\_Mf\_liverA, Unigene2521\_Mf\_liverA, Unigene25286\_Mf\_liverA, Unigene25341\_Mf\_liverA, Unigene25454\_Mf\_liverA, Unigene25852\_Mf\_liverA, Unigene25853\_Mf\_liverA, Unigene258\_Mf\_liverA, Unigene26106\_Mf\_liverA, Unigene26183\_Mf\_liverA, Unigene26305\_Mf\_liverA, Unigene26306\_Mf\_liverA, Unigene26307\_Mf\_liverA, Unigene26384\_Mf\_liverA, Unigene26413\_Mf\_liverA, Unigene26422\_Mf\_liverA, Unigene26423\_Mf\_liverA, Unigene26479\_Mf\_liverA, Unigene26480\_Mf\_liverA, Unigene26481\_Mf\_liverA, Unigene27038\_Mf\_liverA, Unigene2721\_Mf\_liverA, Unigene27312\_Mf\_liverA, Unigene27313\_Mf\_liverA, Unigene27398\_Mf\_liverA, Unigene27399\_Mf\_liverA, Unigene27400\_Mf\_liverA, Unigene27450\_Mf\_liverA, Unigene27452\_Mf\_liverA, Unigene27479\_Mf\_liverA, Unigene27571\_Mf\_liverA, Unigene27684\_Mf\_liverA, Unigene27734\_Mf\_liverA, Unigene28022\_Mf\_liverA, Unigene28250\_Mf\_liverA, Unigene28296\_Mf\_liverA, Unigene28335\_Mf\_liverA, Unigene28336\_Mf\_liverA, Unigene28338\_Mf\_liverA, Unigene28395\_Mf\_liverA, Unigene28396\_Mf\_liverA, Unigene28397\_Mf\_liverA, Unigene28398\_Mf\_liverA, Unigene28399\_Mf\_liverA, Unigene28504\_Mf\_liverA, Unigene28505\_Mf\_liverA, Unigene28617\_Mf\_liverA, Unigene28899\_Mf\_liverA, Unigene2897\_Mf\_liverA, Unigene28989\_Mf\_liverA, Unigene29001\_Mf\_liverA, Unigene29002\_Mf\_liverA, Unigene29076\_Mf\_liverA, Unigene29077\_Mf\_liverA, Unigene29129\_Mf\_liverA, Unigene29359\_Mf\_liverA, Unigene29360\_Mf\_liverA, Unigene29363\_Mf\_liverA, Unigene29366\_Mf\_liverA, Unigene29399\_Mf\_liverA, Unigene29617\_Mf\_liverA, Unigene29636\_Mf\_liverA, Unigene29637\_Mf\_liverA, Unigene29642\_Mf\_liverA, Unigene29725\_Mf\_liverA, Unigene29772\_Mf\_liverA, Unigene29775\_Mf\_liverA, Unigene29788\_Mf\_liverA, Unigene29829\_Mf\_liverA, Unigene29831\_Mf\_liverA, Unigene29866\_Mf\_liverA, Unigene30067\_Mf\_liverA, Unigene30153\_Mf\_liverA, Unigene30289\_Mf\_liverA, Unigene3053\_Mf\_liverA, Unigene30563\_Mf\_liverA, Unigene30564\_Mf\_liverA, Unigene30618\_Mf\_liverA, Unigene30619\_Mf\_liverA, Unigene30621\_Mf\_liverA, Unigene30825\_Mf\_liverA, Unigene30842\_Mf\_liverA, Unigene30843\_Mf\_liverA, Unigene30872\_Mf\_liverA, Unigene31010\_Mf\_liverA, Unigene31011\_Mf\_liverA, Unigene31017\_Mf\_liverA, Unigene31129\_Mf\_liverA, Unigene31325\_Mf\_liverA, Unigene31330\_Mf\_liverA, Unigene31438\_Mf\_liverA, Unigene31687\_Mf\_liverA, Unigene31688\_Mf\_liverA, Unigene31691\_Mf\_liverA, Unigene31692\_Mf\_liverA, Unigene31708\_Mf\_liverA, Unigene31709\_Mf\_liverA, Unigene31710\_Mf\_liverA, Unigene31711\_Mf\_liverA, Unigene31751\_Mf\_liverA, Unigene31796\_Mf\_liverA, Unigene31797\_Mf\_liverA, Unigene31814\_Mf\_liverA, Unigene31822\_Mf\_liverA, Unigene31862\_Mf\_liverA, Unigene32002\_Mf\_liverA, Unigene32003\_Mf\_liverA, Unigene32058\_Mf\_liverA, Unigene32090\_Mf\_liverA, Unigene32132\_Mf\_liverA, Unigene32133\_Mf\_liverA, Unigene32182\_Mf\_liverA, Unigene32186\_Mf\_liverA, Unigene32187\_Mf\_liverA, Unigene32188\_Mf\_liverA, Unigene32213\_Mf\_liverA, Unigene32214\_Mf\_liverA, Unigene3222\_Mf\_liverA, Unigene32234\_Mf\_liverA, Unigene32235\_Mf\_liverA, Unigene32334\_Mf\_liverA, Unigene32335\_Mf\_liverA, Unigene32568\_Mf\_liverA, Unigene32643\_Mf\_liverA, Unigene32649\_Mf\_liverA, Unigene32717\_Mf\_liverA, Unigene32810\_Mf\_liverA, Unigene32879\_Mf\_liverA, Unigene32882\_Mf\_liverA, Unigene32883\_Mf\_liverA, Unigene32958\_Mf\_liverA, Unigene33079\_Mf\_liverA, Unigene33080\_Mf\_liverA, Unigene33122\_Mf\_liverA, Unigene33153\_Mf\_liverA, Unigene33154\_Mf\_liverA, Unigene33155\_Mf\_liverA, Unigene33156\_Mf\_liverA, Unigene33161\_Mf\_liverA, Unigene33247\_Mf\_liverA, Unigene33313\_Mf\_liverA, Unigene33314\_Mf\_liverA, Unigene33853\_Mf\_liverA, Unigene33854\_Mf\_liverA, Unigene33855\_Mf\_liverA, Unigene33856\_Mf\_liverA, Unigene33857\_Mf\_liverA, Unigene33965\_Mf\_liverA, Unigene34084\_Mf\_liverA, Unigene34123\_Mf\_liverA, Unigene34124\_Mf\_liverA, Unigene34937\_Mf\_liverA, Unigene34975\_Mf\_liverA, Unigene35096\_Mf\_liverA, Unigene35151\_Mf\_liverA, Unigene35152\_Mf\_liverA, Unigene35207\_Mf\_liverA, Unigene35427\_Mf\_liverA, Unigene35570\_Mf\_liverA, Unigene35571\_Mf\_liverA, Unigene35572\_Mf\_liverA, Unigene35573\_Mf\_liverA, Unigene35612\_Mf\_liverA, Unigene3572\_Mf\_liverA, Unigene35849\_Mf\_liverA, Unigene35851\_Mf\_liverA, Unigene35853\_Mf\_liverA, Unigene35898\_Mf\_liverA, Unigene35899\_Mf\_liverA, Unigene35900\_Mf\_liverA, Unigene35901\_Mf\_liverA, Unigene35902\_Mf\_liverA, Unigene35946\_Mf\_liverA, Unigene35996\_Mf\_liverA, Unigene36162\_Mf\_liverA, Unigene36173\_Mf\_liverA, Unigene36308\_Mf\_liverA, Unigene36309\_Mf\_liverA, Unigene36310\_Mf\_liverA, Unigene36311\_Mf\_liverA, Unigene36357\_Mf\_liverA, Unigene36381\_Mf\_liverA, Unigene36382\_Mf\_liverA, Unigene36383\_Mf\_liverA, Unigene36384\_Mf\_liverA, Unigene36413\_Mf\_liverA, Unigene36414\_Mf\_liverA, Unigene36417\_Mf\_liverA, Unigene36418\_Mf\_liverA, Unigene36420\_Mf\_liverA, Unigene3643\_Mf\_liverA, Unigene36620\_Mf\_liverA, Unigene36648\_Mf\_liverA, Unigene36700\_Mf\_liverA, Unigene36731\_Mf\_liverA, Unigene36748\_Mf\_liverA, Unigene36807\_Mf\_liverA, Unigene36856\_Mf\_liverA, Unigene36882\_Mf\_liverA, Unigene36906\_Mf\_liverA, Unigene37006\_Mf\_liverA, Unigene37007\_Mf\_liverA, Unigene37147\_Mf\_liverA, Unigene37150\_Mf\_liverA, Unigene37320\_Mf\_liverA, Unigene37352\_Mf\_liverA, Unigene37358\_Mf\_liverA, Unigene37503\_Mf\_liverA, Unigene37591\_Mf\_liverA, Unigene37683\_Mf\_liverA, Unigene3775\_Mf\_liverA, Unigene38208\_Mf\_liverA, Unigene38238\_Mf\_liverA, Unigene38311\_Mf\_liverA, Unigene3838\_Mf\_liverA, Unigene38393\_Mf\_liverA, Unigene38514\_Mf\_liverA, Unigene38630\_Mf\_liverA, Unigene38839\_Mf\_liverA, Unigene38919\_Mf\_liverA, Unigene3915\_Mf\_liverA, Unigene39466\_Mf\_liverA, Unigene39575\_Mf\_liverA, Unigene39640\_Mf\_liverA, Unigene39787\_Mf\_liverA, Unigene39828\_Mf\_liverA, Unigene39990\_Mf\_liverA, Unigene40103\_Mf\_liverA, Unigene40116\_Mf\_liverA, Unigene40205\_Mf\_liverA, Unigene40304\_Mf\_liverA, Unigene40352\_Mf\_liverA, Unigene40387\_Mf\_liverA, Unigene40644\_Mf\_liverA, Unigene40933\_Mf\_liverA, Unigene40962\_Mf\_liverA, Unigene41087\_Mf\_liverA, Unigene41217\_Mf\_liverA, Unigene41315\_Mf\_liverA, Unigene41421\_Mf\_liverA, Unigene41505\_Mf\_liverA, Unigene4226\_Mf\_liverA, Unigene42323\_Mf\_liverA, Unigene42335\_Mf\_liverA, Unigene4264\_Mf\_liverA, Unigene42736\_Mf\_liverA, Unigene42940\_Mf\_liverA, Unigene43225\_Mf\_liverA, Unigene43515\_Mf\_liverA, Unigene43599\_Mf\_liverA, Unigene43792\_Mf\_liverA, Unigene4381\_Mf\_liverA, Unigene4382\_Mf\_liverA, Unigene43893\_Mf\_liverA, Unigene43912\_Mf\_liverA, Unigene44288\_Mf\_liverA, Unigene442\_Mf\_liverA, Unigene44310\_Mf\_liverA, Unigene4459\_Mf\_liverA, Unigene44819\_Mf\_liverA, Unigene44965\_Mf\_liverA, Unigene45212\_Mf\_liverA, Unigene4524\_Mf\_liverA, Unigene45261\_Mf\_liverA, Unigene45269\_Mf\_liverA, Unigene4543\_Mf\_liverA, Unigene45485\_Mf\_liverA, Unigene45555\_Mf\_liverA, Unigene4565\_Mf\_liverA, Unigene45703\_Mf\_liverA, Unigene45708\_Mf\_liverA, Unigene45730\_Mf\_liverA, Unigene4574\_Mf\_liverA, Unigene4575\_Mf\_liverA, Unigene45931\_Mf\_liverA, Unigene46009\_Mf\_liverA, Unigene46027\_Mf\_liverA, Unigene46087\_Mf\_liverA, Unigene46117\_Mf\_liverA, Unigene46132\_Mf\_liverA, Unigene46319\_Mf\_liverA, Unigene46359\_Mf\_liverA, Unigene46368\_Mf\_liverA, Unigene46470\_Mf\_liverA, Unigene46522\_Mf\_liverA, Unigene46649\_Mf\_liverA, Unigene4672\_Mf\_liverA, Unigene46876\_Mf\_liverA, Unigene47153\_Mf\_liverA, Unigene47261\_Mf\_liverA, Unigene47573\_Mf\_liverA, Unigene47772\_Mf\_liverA, Unigene47837\_Mf\_liverA, Unigene47869\_Mf\_liverA, Unigene48281\_Mf\_liverA, Unigene48285\_Mf\_liverA, Unigene48313\_Mf\_liverA, Unigene48506\_Mf\_liverA, Unigene48526\_Mf\_liverA, Unigene48539\_Mf\_liverA, Unigene48645\_Mf\_liverA, Unigene48671\_Mf\_liverA, Unigene48762\_Mf\_liverA, Unigene4893\_Mf\_liverA, Unigene49107\_Mf\_liverA, Unigene49578\_Mf\_liverA, Unigene49797\_Mf\_liverA, Unigene49845\_Mf\_liverA, Unigene4985\_Mf\_liverA, Unigene50036\_Mf\_liverA, Unigene50319\_Mf\_liverA, Unigene50332\_Mf\_liverA, Unigene50360\_Mf\_liverA, Unigene50424\_Mf\_liverA, Unigene50600\_Mf\_liverA, Unigene50689\_Mf\_liverA, Unigene50718\_Mf\_liverA, Unigene51086\_Mf\_liverA, Unigene5134\_Mf\_liverA, Unigene51616\_Mf\_liverA, Unigene51795\_Mf\_liverA, Unigene517\_Mf\_liverA, Unigene5226\_Mf\_liverA, Unigene5343\_Mf\_liverA, Unigene5410\_Mf\_liverA, Unigene5434\_Mf\_liverA, Unigene5453\_Mf\_liverA, Unigene557\_Mf\_liverA, Unigene5767\_Mf\_liverA, Unigene5772\_Mf\_liverA, Unigene583\_Mf\_liverA, Unigene5880\_Mf\_liverA, Unigene5975\_Mf\_liverA, Unigene5983\_Mf\_liverA, Unigene5984\_Mf\_liverA, Unigene610\_Mf\_liverA, Unigene6162\_Mf\_liverA, Unigene6259\_Mf\_liverA, Unigene6392\_Mf\_liverA, Unigene6473\_Mf\_liverA, Unigene6534\_Mf\_liverA, Unigene6639\_Mf\_liverA, Unigene6654\_Mf\_liverA, Unigene687\_Mf\_liverA, Unigene6955\_Mf\_liverA, Unigene7150\_Mf\_liverA, Unigene7298\_Mf\_liverA, Unigene7350\_Mf\_liverA, Unigene7351\_Mf\_liverA, Unigene7498\_Mf\_liverA, Unigene7582\_Mf\_liverA, Unigene7589\_Mf\_liverA, Unigene8066\_Mf\_liverA, Unigene8204\_Mf\_liverA, Unigene8270\_Mf\_liverA, Unigene8360\_Mf\_liverA, Unigene8443\_Mf\_liverA, Unigene8488\_Mf\_liverA, Unigene8501\_Mf\_liverA, Unigene8587\_Mf\_liverA, Unigene85\_Mf\_liverA, Unigene8624\_Mf\_liverA, Unigene88\_Mf\_liverA, Unigene9051\_Mf\_liverA, Unigene9059\_Mf\_liverA, Unigene9060\_Mf\_liverA, Unigene9401\_Mf\_liverA, Unigene9436\_Mf\_liverA, Unigene9501\_Mf\_liverA, Unigene9577\_Mf\_liverA, Unigene9680\_Mf\_liverA, Unigene9719\_Mf\_liverA, Unigene9762\_Mf\_liverA, Unigene9896\_Mf\_liverA, Unigene9905\_Mf\_liverA, Unigene994\_Mf\_liverA |
| 3 | Regulation of actin cytoskeleton | CL1013.Contig5\_Mf\_liverA, CL1034.Contig2\_Mf\_liverA, CL1154.Contig1\_Mf\_liverA, CL1154.Contig2\_Mf\_liverA, CL1180.Contig10\_Mf\_liverA, CL1180.Contig1\_Mf\_liverA, CL1180.Contig5\_Mf\_liverA, CL1196.Contig1\_Mf\_liverA, CL1196.Contig2\_Mf\_liverA, CL1197.Contig1\_Mf\_liverA, CL1197.Contig2\_Mf\_liverA, CL1222.Contig1\_Mf\_liverA, CL1222.Contig2\_Mf\_liverA, CL1235.Contig1\_Mf\_liverA, CL1235.Contig2\_Mf\_liverA, CL1235.Contig3\_Mf\_liverA, CL1270.Contig1\_Mf\_liverA, CL1362.Contig1\_Mf\_liverA, CL1362.Contig2\_Mf\_liverA, CL1365.Contig2\_Mf\_liverA, CL1381.Contig2\_Mf\_liverA, CL1381.Contig3\_Mf\_liverA, CL1540.Contig1\_Mf\_liverA, CL1540.Contig2\_Mf\_liverA, CL1559.Contig1\_Mf\_liverA, CL1559.Contig2\_Mf\_liverA, CL1559.Contig3\_Mf\_liverA, CL1569.Contig1\_Mf\_liverA, CL1569.Contig2\_Mf\_liverA, CL1569.Contig3\_Mf\_liverA, CL1569.Contig4\_Mf\_liverA, CL1569.Contig5\_Mf\_liverA, CL1575.Contig1\_Mf\_liverA, CL1575.Contig2\_Mf\_liverA, CL1619.Contig2\_Mf\_liverA, CL1619.Contig3\_Mf\_liverA, CL1619.Contig4\_Mf\_liverA, CL1622.Contig3\_Mf\_liverA, CL1622.Contig4\_Mf\_liverA, CL1622.Contig6\_Mf\_liverA, CL1622.Contig8\_Mf\_liverA, CL1622.Contig9\_Mf\_liverA, CL1727.Contig1\_Mf\_liverA, CL1727.Contig2\_Mf\_liverA, CL1735.Contig3\_Mf\_liverA, CL1735.Contig4\_Mf\_liverA, CL1747.Contig1\_Mf\_liverA, CL175.Contig1\_Mf\_liverA, CL175.Contig2\_Mf\_liverA, CL175.Contig3\_Mf\_liverA, CL1761.Contig10\_Mf\_liverA, CL1761.Contig11\_Mf\_liverA, CL1761.Contig12\_Mf\_liverA, CL1761.Contig13\_Mf\_liverA, CL1761.Contig14\_Mf\_liverA, CL1761.Contig15\_Mf\_liverA, CL1761.Contig1\_Mf\_liverA, CL1761.Contig2\_Mf\_liverA, CL1761.Contig3\_Mf\_liverA, CL1761.Contig4\_Mf\_liverA, CL1761.Contig5\_Mf\_liverA, CL1761.Contig6\_Mf\_liverA, CL1761.Contig7\_Mf\_liverA, CL1761.Contig8\_Mf\_liverA, CL1761.Contig9\_Mf\_liverA, CL1806.Contig1\_Mf\_liverA, CL1806.Contig2\_Mf\_liverA, CL1806.Contig3\_Mf\_liverA, CL1806.Contig4\_Mf\_liverA, CL1806.Contig5\_Mf\_liverA, CL1806.Contig6\_Mf\_liverA, CL1806.Contig7\_Mf\_liverA, CL1806.Contig8\_Mf\_liverA, CL1865.Contig2\_Mf\_liverA, CL1883.Contig7\_Mf\_liverA, CL1905.Contig10\_Mf\_liverA, CL1905.Contig1\_Mf\_liverA, CL1905.Contig2\_Mf\_liverA, CL1905.Contig3\_Mf\_liverA, CL1905.Contig4\_Mf\_liverA, CL1905.Contig5\_Mf\_liverA, CL1905.Contig6\_Mf\_liverA, CL1905.Contig7\_Mf\_liverA, CL1905.Contig8\_Mf\_liverA, CL1905.Contig9\_Mf\_liverA, CL1927.Contig1\_Mf\_liverA, CL1927.Contig2\_Mf\_liverA, CL1927.Contig3\_Mf\_liverA, CL1927.Contig4\_Mf\_liverA, CL1961.Contig1\_Mf\_liverA, CL1961.Contig2\_Mf\_liverA, CL1964.Contig1\_Mf\_liverA, CL1964.Contig2\_Mf\_liverA, CL1970.Contig1\_Mf\_liverA, CL1970.Contig3\_Mf\_liverA, CL1970.Contig6\_Mf\_liverA, CL1995.Contig1\_Mf\_liverA, CL2021.Contig1\_Mf\_liverA, CL2021.Contig2\_Mf\_liverA, CL2048.Contig1\_Mf\_liverA, CL2048.Contig2\_Mf\_liverA, CL2048.Contig3\_Mf\_liverA, CL2068.Contig1\_Mf\_liverA, CL2068.Contig2\_Mf\_liverA, CL2110.Contig1\_Mf\_liverA, CL2110.Contig2\_Mf\_liverA, CL2110.Contig3\_Mf\_liverA, CL2110.Contig4\_Mf\_liverA, CL2117.Contig1\_Mf\_liverA, CL2117.Contig2\_Mf\_liverA, CL2123.Contig1\_Mf\_liverA, CL2123.Contig2\_Mf\_liverA, CL2166.Contig1\_Mf\_liverA, CL2191.Contig1\_Mf\_liverA, CL2191.Contig2\_Mf\_liverA, CL2230.Contig1\_Mf\_liverA, CL2230.Contig2\_Mf\_liverA, CL2246.Contig1\_Mf\_liverA, CL2246.Contig2\_Mf\_liverA, CL2259.Contig1\_Mf\_liverA, CL2259.Contig2\_Mf\_liverA, CL226.Contig3\_Mf\_liverA, CL2277.Contig1\_Mf\_liverA, CL2277.Contig2\_Mf\_liverA, CL2285.Contig1\_Mf\_liverA, CL2285.Contig2\_Mf\_liverA, CL2285.Contig3\_Mf\_liverA, CL2292.Contig1\_Mf\_liverA, CL2343.Contig1\_Mf\_liverA, CL2343.Contig2\_Mf\_liverA, CL2376.Contig1\_Mf\_liverA, CL2378.Contig1\_Mf\_liverA, CL2378.Contig2\_Mf\_liverA, CL2378.Contig3\_Mf\_liverA, CL2378.Contig4\_Mf\_liverA, CL2400.Contig1\_Mf\_liverA, CL2400.Contig2\_Mf\_liverA, CL2405.Contig1\_Mf\_liverA, CL2405.Contig2\_Mf\_liverA, CL2542.Contig1\_Mf\_liverA, CL2551.Contig1\_Mf\_liverA, CL2553.Contig1\_Mf\_liverA, CL2557.Contig1\_Mf\_liverA, CL2557.Contig2\_Mf\_liverA, CL2560.Contig1\_Mf\_liverA, CL2560.Contig2\_Mf\_liverA, CL2603.Contig1\_Mf\_liverA, CL2603.Contig2\_Mf\_liverA, CL2603.Contig3\_Mf\_liverA, CL2603.Contig4\_Mf\_liverA, CL2639.Contig1\_Mf\_liverA, CL2649.Contig1\_Mf\_liverA, CL2649.Contig3\_Mf\_liverA, CL269.Contig1\_Mf\_liverA, CL269.Contig2\_Mf\_liverA, CL2692.Contig1\_Mf\_liverA, CL2692.Contig2\_Mf\_liverA, CL2692.Contig3\_Mf\_liverA, CL2737.Contig2\_Mf\_liverA, CL2737.Contig3\_Mf\_liverA, CL2737.Contig4\_Mf\_liverA, CL2737.Contig5\_Mf\_liverA, CL2737.Contig6\_Mf\_liverA, CL2753.Contig1\_Mf\_liverA, CL2753.Contig2\_Mf\_liverA, CL2784.Contig1\_Mf\_liverA, CL2784.Contig2\_Mf\_liverA, CL2796.Contig1\_Mf\_liverA, CL2796.Contig2\_Mf\_liverA, CL2861.Contig1\_Mf\_liverA, CL2871.Contig1\_Mf\_liverA, CL2898.Contig1\_Mf\_liverA, CL2898.Contig2\_Mf\_liverA, CL2900.Contig1\_Mf\_liverA, CL2900.Contig2\_Mf\_liverA, CL2963.Contig1\_Mf\_liverA, CL2963.Contig2\_Mf\_liverA, CL2963.Contig3\_Mf\_liverA, CL2963.Contig4\_Mf\_liverA, CL2963.Contig5\_Mf\_liverA, CL2975.Contig1\_Mf\_liverA, CL2975.Contig2\_Mf\_liverA, CL2975.Contig3\_Mf\_liverA, CL2975.Contig4\_Mf\_liverA, CL2996.Contig10\_Mf\_liverA, CL2996.Contig11\_Mf\_liverA, CL2996.Contig1\_Mf\_liverA, CL2996.Contig2\_Mf\_liverA, CL2996.Contig3\_Mf\_liverA, CL2996.Contig4\_Mf\_liverA, CL2996.Contig5\_Mf\_liverA, CL2996.Contig6\_Mf\_liverA, CL2996.Contig7\_Mf\_liverA, CL2996.Contig8\_Mf\_liverA, CL2996.Contig9\_Mf\_liverA, CL3012.Contig1\_Mf\_liverA, CL3012.Contig2\_Mf\_liverA, CL3031.Contig1\_Mf\_liverA, CL3031.Contig2\_Mf\_liverA, CL3125.Contig1\_Mf\_liverA, CL3148.Contig1\_Mf\_liverA, CL3154.Contig1\_Mf\_liverA, CL3154.Contig2\_Mf\_liverA, CL3168.Contig1\_Mf\_liverA, CL3168.Contig2\_Mf\_liverA, CL3207.Contig1\_Mf\_liverA, CL3207.Contig2\_Mf\_liverA, CL3213.Contig1\_Mf\_liverA, CL3213.Contig3\_Mf\_liverA, CL3223.Contig2\_Mf\_liverA, CL3251.Contig1\_Mf\_liverA, CL3252.Contig1\_Mf\_liverA, CL3252.Contig2\_Mf\_liverA, CL3253.Contig1\_Mf\_liverA, CL3253.Contig2\_Mf\_liverA, CL3253.Contig3\_Mf\_liverA, CL3253.Contig4\_Mf\_liverA, CL3276.Contig1\_Mf\_liverA, CL3276.Contig2\_Mf\_liverA, CL3276.Contig3\_Mf\_liverA, CL3388.Contig3\_Mf\_liverA, CL3416.Contig1\_Mf\_liverA, CL3416.Contig2\_Mf\_liverA, CL3433.Contig1\_Mf\_liverA, CL3433.Contig2\_Mf\_liverA, CL3446.Contig1\_Mf\_liverA, CL3446.Contig2\_Mf\_liverA, CL3534.Contig1\_Mf\_liverA, CL3534.Contig3\_Mf\_liverA, CL3534.Contig4\_Mf\_liverA, CL3539.Contig2\_Mf\_liverA, CL3637.Contig1\_Mf\_liverA, CL3656.Contig1\_Mf\_liverA, CL3656.Contig2\_Mf\_liverA, CL3675.Contig1\_Mf\_liverA, CL3675.Contig2\_Mf\_liverA, CL371.Contig1\_Mf\_liverA, CL371.Contig3\_Mf\_liverA, CL371.Contig5\_Mf\_liverA, CL3715.Contig3\_Mf\_liverA, CL3845.Contig1\_Mf\_liverA, CL3861.Contig1\_Mf\_liverA, CL3861.Contig2\_Mf\_liverA, CL3865.Contig1\_Mf\_liverA, CL3898.Contig1\_Mf\_liverA, CL3898.Contig2\_Mf\_liverA, CL4005.Contig1\_Mf\_liverA, CL4005.Contig2\_Mf\_liverA, CL4031.Contig1\_Mf\_liverA, CL4052.Contig1\_Mf\_liverA, CL4055.Contig1\_Mf\_liverA, CL4055.Contig2\_Mf\_liverA, CL410.Contig13\_Mf\_liverA, CL410.Contig14\_Mf\_liverA, CL410.Contig15\_Mf\_liverA, CL410.Contig16\_Mf\_liverA, CL410.Contig17\_Mf\_liverA, CL410.Contig9\_Mf\_liverA, CL4123.Contig1\_Mf\_liverA, CL4123.Contig2\_Mf\_liverA, CL4147.Contig2\_Mf\_liverA, CL4147.Contig3\_Mf\_liverA, CL4153.Contig2\_Mf\_liverA, CL4169.Contig1\_Mf\_liverA, CL4169.Contig2\_Mf\_liverA, CL4175.Contig1\_Mf\_liverA, CL4175.Contig2\_Mf\_liverA, CL4175.Contig3\_Mf\_liverA, CL4175.Contig4\_Mf\_liverA, CL42.Contig1\_Mf\_liverA, CL42.Contig2\_Mf\_liverA, CL42.Contig3\_Mf\_liverA, CL42.Contig4\_Mf\_liverA, CL42.Contig5\_Mf\_liverA, CL42.Contig6\_Mf\_liverA, CL4208.Contig1\_Mf\_liverA, CL4208.Contig2\_Mf\_liverA, CL4254.Contig1\_Mf\_liverA, CL4254.Contig2\_Mf\_liverA, CL4269.Contig1\_Mf\_liverA, CL4276.Contig1\_Mf\_liverA, CL4276.Contig2\_Mf\_liverA, CL4281.Contig2\_Mf\_liverA, CL433.Contig1\_Mf\_liverA, CL433.Contig2\_Mf\_liverA, CL4372.Contig1\_Mf\_liverA, CL4388.Contig1\_Mf\_liverA, CL4388.Contig2\_Mf\_liverA, CL4388.Contig3\_Mf\_liverA, CL4434.Contig1\_Mf\_liverA, CL4460.Contig1\_Mf\_liverA, CL4460.Contig2\_Mf\_liverA, CL4518.Contig3\_Mf\_liverA, CL4598.Contig3\_Mf\_liverA, CL4598.Contig4\_Mf\_liverA, CL4604.Contig1\_Mf\_liverA, CL4604.Contig2\_Mf\_liverA, CL4616.Contig1\_Mf\_liverA, CL4616.Contig2\_Mf\_liverA, CL462.Contig1\_Mf\_liverA, CL462.Contig2\_Mf\_liverA, CL462.Contig3\_Mf\_liverA, CL462.Contig4\_Mf\_liverA, CL462.Contig5\_Mf\_liverA, CL462.Contig6\_Mf\_liverA, CL462.Contig7\_Mf\_liverA, CL462.Contig8\_Mf\_liverA, CL4648.Contig1\_Mf\_liverA, CL4648.Contig2\_Mf\_liverA, CL4664.Contig1\_Mf\_liverA, CL4664.Contig2\_Mf\_liverA, CL4731.Contig1\_Mf\_liverA, CL4731.Contig2\_Mf\_liverA, CL4762.Contig1\_Mf\_liverA, CL4772.Contig1\_Mf\_liverA, CL4784.Contig2\_Mf\_liverA, CL4812.Contig2\_Mf\_liverA, CL4827.Contig1\_Mf\_liverA, CL4827.Contig2\_Mf\_liverA, CL4846.Contig1\_Mf\_liverA, CL4846.Contig2\_Mf\_liverA, CL4848.Contig1\_Mf\_liverA, CL4848.Contig2\_Mf\_liverA, CL4892.Contig1\_Mf\_liverA, CL4892.Contig2\_Mf\_liverA, CL490.Contig1\_Mf\_liverA, CL490.Contig2\_Mf\_liverA, CL490.Contig3\_Mf\_liverA, CL490.Contig4\_Mf\_liverA, CL490.Contig6\_Mf\_liverA, CL490.Contig7\_Mf\_liverA, CL4918.Contig1\_Mf\_liverA, CL4918.Contig2\_Mf\_liverA, CL4936.Contig1\_Mf\_liverA, CL4936.Contig2\_Mf\_liverA, CL4998.Contig1\_Mf\_liverA, CL4998.Contig2\_Mf\_liverA, CL5048.Contig1\_Mf\_liverA, CL5069.Contig1\_Mf\_liverA, CL507.Contig1\_Mf\_liverA, CL510.Contig1\_Mf\_liverA, CL510.Contig2\_Mf\_liverA, CL516.Contig1\_Mf\_liverA, CL516.Contig2\_Mf\_liverA, CL516.Contig3\_Mf\_liverA, CL516.Contig4\_Mf\_liverA, CL5184.Contig1\_Mf\_liverA, CL5254.Contig1\_Mf\_liverA, CL5254.Contig2\_Mf\_liverA, CL5261.Contig2\_Mf\_liverA, CL5303.Contig1\_Mf\_liverA, CL5303.Contig2\_Mf\_liverA, CL5303.Contig4\_Mf\_liverA, CL5303.Contig5\_Mf\_liverA, CL5303.Contig6\_Mf\_liverA, CL5312.Contig1\_Mf\_liverA, CL5312.Contig2\_Mf\_liverA, CL5368.Contig1\_Mf\_liverA, CL5368.Contig2\_Mf\_liverA, CL5406.Contig2\_Mf\_liverA, CL5527.Contig1\_Mf\_liverA, CL5527.Contig2\_Mf\_liverA, CL5569.Contig1\_Mf\_liverA, CL5569.Contig2\_Mf\_liverA, CL5570.Contig1\_Mf\_liverA, CL5570.Contig2\_Mf\_liverA, CL5595.Contig1\_Mf\_liverA, CL5595.Contig2\_Mf\_liverA, CL561.Contig1\_Mf\_liverA, CL561.Contig2\_Mf\_liverA, CL561.Contig3\_Mf\_liverA, CL5664.Contig1\_Mf\_liverA, CL5664.Contig2\_Mf\_liverA, CL5669.Contig1\_Mf\_liverA, CL5669.Contig2\_Mf\_liverA, CL5670.Contig1\_Mf\_liverA, CL5829.Contig1\_Mf\_liverA, CL5842.Contig1\_Mf\_liverA, CL5842.Contig2\_Mf\_liverA, CL5878.Contig2\_Mf\_liverA, CL68.Contig1\_Mf\_liverA, CL68.Contig2\_Mf\_liverA, CL68.Contig3\_Mf\_liverA, CL712.Contig5\_Mf\_liverA, CL712.Contig6\_Mf\_liverA, CL712.Contig7\_Mf\_liverA, CL712.Contig8\_Mf\_liverA, CL723.Contig1\_Mf\_liverA, CL723.Contig2\_Mf\_liverA, CL723.Contig3\_Mf\_liverA, CL774.Contig10\_Mf\_liverA, CL774.Contig11\_Mf\_liverA, CL774.Contig12\_Mf\_liverA, CL774.Contig3\_Mf\_liverA, CL774.Contig4\_Mf\_liverA, CL774.Contig5\_Mf\_liverA, CL774.Contig6\_Mf\_liverA, CL774.Contig9\_Mf\_liverA, CL779.Contig10\_Mf\_liverA, CL779.Contig12\_Mf\_liverA, CL779.Contig1\_Mf\_liverA, CL779.Contig3\_Mf\_liverA, CL779.Contig4\_Mf\_liverA, CL779.Contig6\_Mf\_liverA, CL779.Contig7\_Mf\_liverA, CL779.Contig9\_Mf\_liverA, CL795.Contig1\_Mf\_liverA, CL807.Contig1\_Mf\_liverA, CL807.Contig2\_Mf\_liverA, CL807.Contig3\_Mf\_liverA, CL807.Contig4\_Mf\_liverA, CL807.Contig5\_Mf\_liverA, CL807.Contig6\_Mf\_liverA, CL807.Contig7\_Mf\_liverA, CL807.Contig8\_Mf\_liverA, CL812.Contig4\_Mf\_liverA, CL82.Contig3\_Mf\_liverA, CL82.Contig4\_Mf\_liverA, CL833.Contig1\_Mf\_liverA, CL833.Contig2\_Mf\_liverA, CL838.Contig1\_Mf\_liverA, CL838.Contig2\_Mf\_liverA, CL838.Contig3\_Mf\_liverA, CL838.Contig4\_Mf\_liverA, CL838.Contig5\_Mf\_liverA, CL838.Contig6\_Mf\_liverA, CL844.Contig1\_Mf\_liverA, CL844.Contig2\_Mf\_liverA, CL851.Contig1\_Mf\_liverA, CL851.Contig2\_Mf\_liverA, CL863.Contig1\_Mf\_liverA, CL863.Contig2\_Mf\_liverA, CL863.Contig3\_Mf\_liverA, CL863.Contig4\_Mf\_liverA, CL875.Contig1\_Mf\_liverA, CL875.Contig2\_Mf\_liverA, CL875.Contig3\_Mf\_liverA, CL882.Contig2\_Mf\_liverA, CL882.Contig3\_Mf\_liverA, CL882.Contig5\_Mf\_liverA, CL882.Contig6\_Mf\_liverA, CL97.Contig1\_Mf\_liverA, CL97.Contig2\_Mf\_liverA, CL987.Contig1\_Mf\_liverA, CL987.Contig2\_Mf\_liverA, CL987.Contig3\_Mf\_liverA, CL987.Contig4\_Mf\_liverA, CL987.Contig5\_Mf\_liverA, CL987.Contig6\_Mf\_liverA, Unigene1008\_Mf\_liverA, Unigene10139\_Mf\_liverA, Unigene10249\_Mf\_liverA, Unigene10522\_Mf\_liverA, Unigene1055\_Mf\_liverA, Unigene1085\_Mf\_liverA, Unigene10889\_Mf\_liverA, Unigene10970\_Mf\_liverA, Unigene1108\_Mf\_liverA, Unigene1119\_Mf\_liverA, Unigene11227\_Mf\_liverA, Unigene1148\_Mf\_liverA, Unigene11542\_Mf\_liverA, Unigene11737\_Mf\_liverA, Unigene11749\_Mf\_liverA, Unigene11892\_Mf\_liverA, Unigene12525\_Mf\_liverA, Unigene12592\_Mf\_liverA, Unigene12713\_Mf\_liverA, Unigene12814\_Mf\_liverA, Unigene12842\_Mf\_liverA, Unigene12932\_Mf\_liverA, Unigene12951\_Mf\_liverA, Unigene12953\_Mf\_liverA, Unigene1308\_Mf\_liverA, Unigene1318\_Mf\_liverA, Unigene13408\_Mf\_liverA, Unigene13535\_Mf\_liverA, Unigene13628\_Mf\_liverA, Unigene13918\_Mf\_liverA, Unigene13919\_Mf\_liverA, Unigene13960\_Mf\_liverA, Unigene14054\_Mf\_liverA, Unigene14094\_Mf\_liverA, Unigene14168\_Mf\_liverA, Unigene14284\_Mf\_liverA, Unigene14313\_Mf\_liverA, Unigene14427\_Mf\_liverA, Unigene14428\_Mf\_liverA, Unigene14581\_Mf\_liverA, Unigene14588\_Mf\_liverA, Unigene14602\_Mf\_liverA, Unigene14820\_Mf\_liverA, Unigene14941\_Mf\_liverA, Unigene15010\_Mf\_liverA, Unigene15125\_Mf\_liverA, Unigene15338\_Mf\_liverA, Unigene15339\_Mf\_liverA, Unigene15364\_Mf\_liverA, Unigene15572\_Mf\_liverA, Unigene15610\_Mf\_liverA, Unigene15630\_Mf\_liverA, Unigene15911\_Mf\_liverA, Unigene16240\_Mf\_liverA, Unigene16241\_Mf\_liverA, Unigene16545\_Mf\_liverA, Unigene16586\_Mf\_liverA, Unigene16670\_Mf\_liverA, Unigene16671\_Mf\_liverA, Unigene16757\_Mf\_liverA, Unigene16952\_Mf\_liverA, Unigene17025\_Mf\_liverA, Unigene17035\_Mf\_liverA, Unigene17113\_Mf\_liverA, Unigene17392\_Mf\_liverA, Unigene17455\_Mf\_liverA, Unigene17518\_Mf\_liverA, Unigene17855\_Mf\_liverA, Unigene18177\_Mf\_liverA, Unigene1828\_Mf\_liverA, Unigene182\_Mf\_liverA, Unigene18326\_Mf\_liverA, Unigene18340\_Mf\_liverA, Unigene18428\_Mf\_liverA, Unigene18443\_Mf\_liverA, Unigene18792\_Mf\_liverA, Unigene18843\_Mf\_liverA, Unigene1896\_Mf\_liverA, Unigene19195\_Mf\_liverA, Unigene19196\_Mf\_liverA, Unigene19232\_Mf\_liverA, Unigene19269\_Mf\_liverA, Unigene19568\_Mf\_liverA, Unigene19658\_Mf\_liverA, Unigene19659\_Mf\_liverA, Unigene19852\_Mf\_liverA, Unigene19853\_Mf\_liverA, Unigene19881\_Mf\_liverA, Unigene19882\_Mf\_liverA, Unigene19893\_Mf\_liverA, Unigene19894\_Mf\_liverA, Unigene19992\_Mf\_liverA, Unigene20108\_Mf\_liverA, Unigene20216\_Mf\_liverA, Unigene20262\_Mf\_liverA, Unigene20472\_Mf\_liverA, Unigene20612\_Mf\_liverA, Unigene20697\_Mf\_liverA, Unigene20776\_Mf\_liverA, Unigene20829\_Mf\_liverA, Unigene21275\_Mf\_liverA, Unigene21276\_Mf\_liverA, Unigene21423\_Mf\_liverA, Unigene21816\_Mf\_liverA, Unigene21934\_Mf\_liverA, Unigene22012\_Mf\_liverA, Unigene22158\_Mf\_liverA, Unigene22381\_Mf\_liverA, Unigene22390\_Mf\_liverA, Unigene22396\_Mf\_liverA, Unigene22432\_Mf\_liverA, Unigene22433\_Mf\_liverA, Unigene22558\_Mf\_liverA, Unigene22597\_Mf\_liverA, Unigene22919\_Mf\_liverA, Unigene23334\_Mf\_liverA, Unigene2338\_Mf\_liverA, Unigene23869\_Mf\_liverA, Unigene23870\_Mf\_liverA, Unigene23871\_Mf\_liverA, Unigene23920\_Mf\_liverA, Unigene23922\_Mf\_liverA, Unigene23923\_Mf\_liverA, Unigene23924\_Mf\_liverA, Unigene23934\_Mf\_liverA, Unigene23935\_Mf\_liverA, Unigene23936\_Mf\_liverA, Unigene23937\_Mf\_liverA, Unigene23938\_Mf\_liverA, Unigene23\_Mf\_liverA, Unigene24065\_Mf\_liverA, Unigene24068\_Mf\_liverA, Unigene24069\_Mf\_liverA, Unigene24111\_Mf\_liverA, Unigene24112\_Mf\_liverA, Unigene24182\_Mf\_liverA, Unigene24215\_Mf\_liverA, Unigene24335\_Mf\_liverA, Unigene24347\_Mf\_liverA, Unigene24540\_Mf\_liverA, Unigene24576\_Mf\_liverA, Unigene24707\_Mf\_liverA, Unigene24715\_Mf\_liverA, Unigene24755\_Mf\_liverA, Unigene24763\_Mf\_liverA, Unigene24804\_Mf\_liverA, Unigene24805\_Mf\_liverA, Unigene24909\_Mf\_liverA, Unigene24965\_Mf\_liverA, Unigene24981\_Mf\_liverA, Unigene25080\_Mf\_liverA, Unigene25119\_Mf\_liverA, Unigene25132\_Mf\_liverA, Unigene25162\_Mf\_liverA, Unigene25187\_Mf\_liverA, Unigene25200\_Mf\_liverA, Unigene25218\_Mf\_liverA, Unigene2521\_Mf\_liverA, Unigene25272\_Mf\_liverA, Unigene25299\_Mf\_liverA, Unigene25300\_Mf\_liverA, Unigene2530\_Mf\_liverA, Unigene25326\_Mf\_liverA, Unigene25404\_Mf\_liverA, Unigene25416\_Mf\_liverA, Unigene2549\_Mf\_liverA, Unigene25733\_Mf\_liverA, Unigene25734\_Mf\_liverA, Unigene25748\_Mf\_liverA, Unigene25753\_Mf\_liverA, Unigene25826\_Mf\_liverA, Unigene26032\_Mf\_liverA, Unigene26033\_Mf\_liverA, Unigene26034\_Mf\_liverA, Unigene26035\_Mf\_liverA, Unigene26036\_Mf\_liverA, Unigene26052\_Mf\_liverA, Unigene26190\_Mf\_liverA, Unigene26194\_Mf\_liverA, Unigene26210\_Mf\_liverA, Unigene26251\_Mf\_liverA, Unigene26305\_Mf\_liverA, Unigene26306\_Mf\_liverA, Unigene26307\_Mf\_liverA, Unigene26406\_Mf\_liverA, Unigene26440\_Mf\_liverA, Unigene26655\_Mf\_liverA, Unigene26656\_Mf\_liverA, Unigene26657\_Mf\_liverA, Unigene26712\_Mf\_liverA, Unigene26964\_Mf\_liverA, Unigene27221\_Mf\_liverA, Unigene27260\_Mf\_liverA, Unigene27261\_Mf\_liverA, Unigene27312\_Mf\_liverA, Unigene27313\_Mf\_liverA, Unigene27323\_Mf\_liverA, Unigene27337\_Mf\_liverA, Unigene27353\_Mf\_liverA, Unigene27538\_Mf\_liverA, Unigene27539\_Mf\_liverA, Unigene27684\_Mf\_liverA, Unigene27868\_Mf\_liverA, Unigene27939\_Mf\_liverA, Unigene28146\_Mf\_liverA, Unigene28165\_Mf\_liverA, Unigene28166\_Mf\_liverA, Unigene28283\_Mf\_liverA, Unigene28284\_Mf\_liverA, Unigene28285\_Mf\_liverA, Unigene2829\_Mf\_liverA, Unigene28491\_Mf\_liverA, Unigene28504\_Mf\_liverA, Unigene28505\_Mf\_liverA, Unigene28621\_Mf\_liverA, Unigene28722\_Mf\_liverA, Unigene28879\_Mf\_liverA, Unigene28880\_Mf\_liverA, Unigene28899\_Mf\_liverA, Unigene2890\_Mf\_liverA, Unigene28989\_Mf\_liverA, Unigene29041\_Mf\_liverA, Unigene29095\_Mf\_liverA, Unigene29359\_Mf\_liverA, Unigene29360\_Mf\_liverA, Unigene29363\_Mf\_liverA, Unigene29424\_Mf\_liverA, Unigene29850\_Mf\_liverA, Unigene29938\_Mf\_liverA, Unigene29939\_Mf\_liverA, Unigene30099\_Mf\_liverA, Unigene30134\_Mf\_liverA, Unigene30135\_Mf\_liverA, Unigene30192\_Mf\_liverA, Unigene30200\_Mf\_liverA, Unigene30256\_Mf\_liverA, Unigene30618\_Mf\_liverA, Unigene30619\_Mf\_liverA, Unigene30621\_Mf\_liverA, Unigene30642\_Mf\_liverA, Unigene3066\_Mf\_liverA, Unigene30776\_Mf\_liverA, Unigene30800\_Mf\_liverA, Unigene30801\_Mf\_liverA, Unigene30873\_Mf\_liverA, Unigene30877\_Mf\_liverA, Unigene30944\_Mf\_liverA, Unigene30949\_Mf\_liverA, Unigene31028\_Mf\_liverA, Unigene31303\_Mf\_liverA, Unigene31333\_Mf\_liverA, Unigene31368\_Mf\_liverA, Unigene31386\_Mf\_liverA, Unigene31424\_Mf\_liverA, Unigene31430\_Mf\_liverA, Unigene31440\_Mf\_liverA, Unigene31487\_Mf\_liverA, Unigene31511\_Mf\_liverA, Unigene31613\_Mf\_liverA, Unigene31665\_Mf\_liverA, Unigene31888\_Mf\_liverA, Unigene31889\_Mf\_liverA, Unigene31954\_Mf\_liverA, Unigene31968\_Mf\_liverA, Unigene32058\_Mf\_liverA, Unigene32096\_Mf\_liverA, Unigene32097\_Mf\_liverA, Unigene32111\_Mf\_liverA, Unigene32125\_Mf\_liverA, Unigene32168\_Mf\_liverA, Unigene32176\_Mf\_liverA, Unigene32183\_Mf\_liverA, Unigene3222\_Mf\_liverA, Unigene32234\_Mf\_liverA, Unigene32235\_Mf\_liverA, Unigene32334\_Mf\_liverA, Unigene32335\_Mf\_liverA, Unigene32370\_Mf\_liverA, Unigene32396\_Mf\_liverA, Unigene32469\_Mf\_liverA, Unigene32583\_Mf\_liverA, Unigene32659\_Mf\_liverA, Unigene32717\_Mf\_liverA, Unigene32895\_Mf\_liverA, Unigene32909\_Mf\_liverA, Unigene3295\_Mf\_liverA, Unigene33000\_Mf\_liverA, Unigene33076\_Mf\_liverA, Unigene3308\_Mf\_liverA, Unigene33247\_Mf\_liverA, Unigene33262\_Mf\_liverA, Unigene33263\_Mf\_liverA, Unigene33275\_Mf\_liverA, Unigene33296\_Mf\_liverA, Unigene33352\_Mf\_liverA, Unigene33364\_Mf\_liverA, Unigene33617\_Mf\_liverA, Unigene33692\_Mf\_liverA, Unigene33838\_Mf\_liverA, Unigene33937\_Mf\_liverA, Unigene33938\_Mf\_liverA, Unigene33965\_Mf\_liverA, Unigene34055\_Mf\_liverA, Unigene34056\_Mf\_liverA, Unigene34183\_Mf\_liverA, Unigene34411\_Mf\_liverA, Unigene34813\_Mf\_liverA, Unigene34847\_Mf\_liverA, Unigene34930\_Mf\_liverA, Unigene34931\_Mf\_liverA, Unigene35075\_Mf\_liverA, Unigene3514\_Mf\_liverA, Unigene35167\_Mf\_liverA, Unigene35196\_Mf\_liverA, Unigene3528\_Mf\_liverA, Unigene35370\_Mf\_liverA, Unigene35528\_Mf\_liverA, Unigene35570\_Mf\_liverA, Unigene35571\_Mf\_liverA, Unigene35572\_Mf\_liverA, Unigene35573\_Mf\_liverA, Unigene35600\_Mf\_liverA, Unigene35690\_Mf\_liverA, Unigene35748\_Mf\_liverA, Unigene35750\_Mf\_liverA, Unigene35898\_Mf\_liverA, Unigene35899\_Mf\_liverA, Unigene35900\_Mf\_liverA, Unigene35901\_Mf\_liverA, Unigene35902\_Mf\_liverA, Unigene36039\_Mf\_liverA, Unigene36040\_Mf\_liverA, Unigene36337\_Mf\_liverA, Unigene36631\_Mf\_liverA, Unigene36673\_Mf\_liverA, Unigene36748\_Mf\_liverA, Unigene36792\_Mf\_liverA, Unigene36818\_Mf\_liverA, Unigene36819\_Mf\_liverA, Unigene37017\_Mf\_liverA, Unigene37042\_Mf\_liverA, Unigene37132\_Mf\_liverA, Unigene37147\_Mf\_liverA, Unigene37149\_Mf\_liverA, Unigene37176\_Mf\_liverA, Unigene37341\_Mf\_liverA, Unigene373\_Mf\_liverA, Unigene37412\_Mf\_liverA, Unigene37474\_Mf\_liverA, Unigene37683\_Mf\_liverA, Unigene37766\_Mf\_liverA, Unigene37771\_Mf\_liverA, Unigene37802\_Mf\_liverA, Unigene38319\_Mf\_liverA, Unigene38353\_Mf\_liverA, Unigene38516\_Mf\_liverA, Unigene38839\_Mf\_liverA, Unigene38870\_Mf\_liverA, Unigene38919\_Mf\_liverA, Unigene38931\_Mf\_liverA, Unigene39099\_Mf\_liverA, Unigene39252\_Mf\_liverA, Unigene39537\_Mf\_liverA, Unigene3961\_Mf\_liverA, Unigene39640\_Mf\_liverA, Unigene39749\_Mf\_liverA, Unigene39816\_Mf\_liverA, Unigene39890\_Mf\_liverA, Unigene39990\_Mf\_liverA, Unigene40020\_Mf\_liverA, Unigene40060\_Mf\_liverA, Unigene40369\_Mf\_liverA, Unigene40532\_Mf\_liverA, Unigene40610\_Mf\_liverA, Unigene40824\_Mf\_liverA, Unigene41125\_Mf\_liverA, Unigene4133\_Mf\_liverA, Unigene41600\_Mf\_liverA, Unigene4169\_Mf\_liverA, Unigene4170\_Mf\_liverA, Unigene41758\_Mf\_liverA, Unigene41785\_Mf\_liverA, Unigene41957\_Mf\_liverA, Unigene41993\_Mf\_liverA, Unigene42117\_Mf\_liverA, Unigene42182\_Mf\_liverA, Unigene4226\_Mf\_liverA, Unigene42272\_Mf\_liverA, Unigene42852\_Mf\_liverA, Unigene43225\_Mf\_liverA, Unigene43262\_Mf\_liverA, Unigene43339\_Mf\_liverA, Unigene43452\_Mf\_liverA, Unigene43599\_Mf\_liverA, Unigene43624\_Mf\_liverA, Unigene44038\_Mf\_liverA, Unigene44137\_Mf\_liverA, Unigene44395\_Mf\_liverA, Unigene4459\_Mf\_liverA, Unigene44657\_Mf\_liverA, Unigene44670\_Mf\_liverA, Unigene44769\_Mf\_liverA, Unigene44886\_Mf\_liverA, Unigene44975\_Mf\_liverA, Unigene45138\_Mf\_liverA, Unigene45261\_Mf\_liverA, Unigene45703\_Mf\_liverA, Unigene45708\_Mf\_liverA, Unigene46470\_Mf\_liverA, Unigene46484\_Mf\_liverA, Unigene46793\_Mf\_liverA, Unigene4684\_Mf\_liverA, Unigene47034\_Mf\_liverA, Unigene47377\_Mf\_liverA, Unigene47472\_Mf\_liverA, Unigene47554\_Mf\_liverA, Unigene4768\_Mf\_liverA, Unigene47837\_Mf\_liverA, Unigene47936\_Mf\_liverA, Unigene47970\_Mf\_liverA, Unigene4863\_Mf\_liverA, Unigene4871\_Mf\_liverA, Unigene48857\_Mf\_liverA, Unigene49422\_Mf\_liverA, Unigene49797\_Mf\_liverA, Unigene4983\_Mf\_liverA, Unigene50036\_Mf\_liverA, Unigene50319\_Mf\_liverA, Unigene50360\_Mf\_liverA, Unigene50718\_Mf\_liverA, Unigene5078\_Mf\_liverA, Unigene51086\_Mf\_liverA, Unigene51682\_Mf\_liverA, Unigene5172\_Mf\_liverA, Unigene5226\_Mf\_liverA, Unigene5316\_Mf\_liverA, Unigene5434\_Mf\_liverA, Unigene557\_Mf\_liverA, Unigene5648\_Mf\_liverA, Unigene583\_Mf\_liverA, Unigene5875\_Mf\_liverA, Unigene5880\_Mf\_liverA, Unigene5983\_Mf\_liverA, Unigene5984\_Mf\_liverA, Unigene613\_Mf\_liverA, Unigene6318\_Mf\_liverA, Unigene676\_Mf\_liverA, Unigene682\_Mf\_liverA, Unigene6897\_Mf\_liverA, Unigene6991\_Mf\_liverA, Unigene7098\_Mf\_liverA, Unigene7146\_Mf\_liverA, Unigene7341\_Mf\_liverA, Unigene744\_Mf\_liverA, Unigene7498\_Mf\_liverA, Unigene7606\_Mf\_liverA, Unigene7614\_Mf\_liverA, Unigene8066\_Mf\_liverA, Unigene8498\_Mf\_liverA, Unigene875\_Mf\_liverA, Unigene877\_Mf\_liverA, Unigene8878\_Mf\_liverA, Unigene9051\_Mf\_liverA, Unigene9067\_Mf\_liverA, Unigene9197\_Mf\_liverA, Unigene919\_Mf\_liverA, Unigene9201\_Mf\_liverA, Unigene9375\_Mf\_liverA, Unigene938\_Mf\_liverA, Unigene9436\_Mf\_liverA, Unigene9621\_Mf\_liverA, Unigene9762\_Mf\_liverA, Unigene9933\_Mf\_liverA, Unigene9\_Mf\_liverA |
| 4 | Focal adhesion | CL1044.Contig1\_Mf\_liverA, CL1044.Contig2\_Mf\_liverA, CL1101.Contig1\_Mf\_liverA, CL1101.Contig2\_Mf\_liverA, CL1101.Contig3\_Mf\_liverA, CL1101.Contig4\_Mf\_liverA, CL1101.Contig5\_Mf\_liverA, CL1154.Contig1\_Mf\_liverA, CL1154.Contig2\_Mf\_liverA, CL1197.Contig1\_Mf\_liverA, CL1197.Contig2\_Mf\_liverA, CL1207.Contig1\_Mf\_liverA, CL1207.Contig2\_Mf\_liverA, CL1222.Contig1\_Mf\_liverA, CL1222.Contig2\_Mf\_liverA, CL1235.Contig1\_Mf\_liverA, CL1235.Contig2\_Mf\_liverA, CL1235.Contig3\_Mf\_liverA, CL1253.Contig1\_Mf\_liverA, CL1253.Contig2\_Mf\_liverA, CL1362.Contig1\_Mf\_liverA, CL1362.Contig2\_Mf\_liverA, CL1365.Contig1\_Mf\_liverA, CL1370.Contig1\_Mf\_liverA, CL1370.Contig2\_Mf\_liverA, CL1370.Contig3\_Mf\_liverA, CL1370.Contig4\_Mf\_liverA, CL1380.Contig1\_Mf\_liverA, CL1381.Contig2\_Mf\_liverA, CL1381.Contig3\_Mf\_liverA, CL1410.Contig1\_Mf\_liverA, CL1410.Contig2\_Mf\_liverA, CL1410.Contig3\_Mf\_liverA, CL1410.Contig4\_Mf\_liverA, CL1559.Contig1\_Mf\_liverA, CL1559.Contig2\_Mf\_liverA, CL1559.Contig3\_Mf\_liverA, CL1567.Contig10\_Mf\_liverA, CL1567.Contig11\_Mf\_liverA, CL1567.Contig12\_Mf\_liverA, CL1567.Contig13\_Mf\_liverA, CL1567.Contig14\_Mf\_liverA, CL1567.Contig15\_Mf\_liverA, CL1567.Contig1\_Mf\_liverA, CL1567.Contig2\_Mf\_liverA, CL1567.Contig3\_Mf\_liverA, CL1567.Contig4\_Mf\_liverA, CL1567.Contig5\_Mf\_liverA, CL1567.Contig6\_Mf\_liverA, CL1567.Contig7\_Mf\_liverA, CL1567.Contig8\_Mf\_liverA, CL1567.Contig9\_Mf\_liverA, CL1571.Contig2\_Mf\_liverA, CL1575.Contig1\_Mf\_liverA, CL1575.Contig2\_Mf\_liverA, CL1682.Contig1\_Mf\_liverA, CL1685.Contig1\_Mf\_liverA, CL1685.Contig2\_Mf\_liverA, CL1685.Contig3\_Mf\_liverA, CL1685.Contig4\_Mf\_liverA, CL1685.Contig5\_Mf\_liverA, CL1685.Contig6\_Mf\_liverA, CL1685.Contig7\_Mf\_liverA, CL1685.Contig8\_Mf\_liverA, CL1690.Contig1\_Mf\_liverA, CL1690.Contig2\_Mf\_liverA, CL1690.Contig3\_Mf\_liverA, CL1690.Contig4\_Mf\_liverA, CL175.Contig1\_Mf\_liverA, CL175.Contig2\_Mf\_liverA, CL175.Contig3\_Mf\_liverA, CL1757.Contig1\_Mf\_liverA, CL1784.Contig1\_Mf\_liverA, CL1805.Contig1\_Mf\_liverA, CL1805.Contig2\_Mf\_liverA, CL1837.Contig1\_Mf\_liverA, CL1837.Contig2\_Mf\_liverA, CL1837.Contig3\_Mf\_liverA, CL1900.Contig1\_Mf\_liverA, CL1900.Contig2\_Mf\_liverA, CL1961.Contig1\_Mf\_liverA, CL1961.Contig2\_Mf\_liverA, CL1994.Contig1\_Mf\_liverA, CL1994.Contig2\_Mf\_liverA, CL1995.Contig1\_Mf\_liverA, CL2007.Contig1\_Mf\_liverA, CL2007.Contig2\_Mf\_liverA, CL2007.Contig3\_Mf\_liverA, CL2048.Contig1\_Mf\_liverA, CL2048.Contig2\_Mf\_liverA, CL2048.Contig3\_Mf\_liverA, CL2068.Contig1\_Mf\_liverA, CL2068.Contig2\_Mf\_liverA, CL210.Contig2\_Mf\_liverA, CL2134.Contig1\_Mf\_liverA, CL2134.Contig2\_Mf\_liverA, CL2230.Contig1\_Mf\_liverA, CL2230.Contig2\_Mf\_liverA, CL2246.Contig1\_Mf\_liverA, CL2246.Contig2\_Mf\_liverA, CL2285.Contig1\_Mf\_liverA, CL2285.Contig2\_Mf\_liverA, CL2285.Contig3\_Mf\_liverA, CL2293.Contig1\_Mf\_liverA, CL2293.Contig2\_Mf\_liverA, CL2293.Contig3\_Mf\_liverA, CL2293.Contig4\_Mf\_liverA, CL2307.Contig1\_Mf\_liverA, CL2307.Contig2\_Mf\_liverA, CL2376.Contig1\_Mf\_liverA, CL2381.Contig1\_Mf\_liverA, CL2381.Contig2\_Mf\_liverA, CL2381.Contig3\_Mf\_liverA, CL2381.Contig4\_Mf\_liverA, CL239.Contig2\_Mf\_liverA, CL2400.Contig1\_Mf\_liverA, CL2400.Contig2\_Mf\_liverA, CL2405.Contig1\_Mf\_liverA, CL2405.Contig2\_Mf\_liverA, CL243.Contig1\_Mf\_liverA, CL2456.Contig1\_Mf\_liverA, CL2456.Contig2\_Mf\_liverA, CL2456.Contig3\_Mf\_liverA, CL2456.Contig4\_Mf\_liverA, CL2500.Contig1\_Mf\_liverA, CL2500.Contig2\_Mf\_liverA, CL2500.Contig3\_Mf\_liverA, CL2500.Contig4\_Mf\_liverA, CL2500.Contig5\_Mf\_liverA, CL2500.Contig6\_Mf\_liverA, CL2500.Contig7\_Mf\_liverA, CL2500.Contig8\_Mf\_liverA, CL2508.Contig1\_Mf\_liverA, CL2508.Contig2\_Mf\_liverA, CL2546.Contig1\_Mf\_liverA, CL2546.Contig2\_Mf\_liverA, CL2557.Contig1\_Mf\_liverA, CL2557.Contig2\_Mf\_liverA, CL2560.Contig1\_Mf\_liverA, CL2560.Contig2\_Mf\_liverA, CL260.Contig1\_Mf\_liverA, CL260.Contig2\_Mf\_liverA, CL2620.Contig1\_Mf\_liverA, CL2638.Contig1\_Mf\_liverA, CL2638.Contig2\_Mf\_liverA, CL2638.Contig3\_Mf\_liverA, CL2638.Contig4\_Mf\_liverA, CL2664.Contig1\_Mf\_liverA, CL2664.Contig2\_Mf\_liverA, CL2664.Contig3\_Mf\_liverA, CL2664.Contig4\_Mf\_liverA, CL2692.Contig1\_Mf\_liverA, CL2692.Contig2\_Mf\_liverA, CL2692.Contig3\_Mf\_liverA, CL2693.Contig1\_Mf\_liverA, CL2693.Contig2\_Mf\_liverA, CL2753.Contig1\_Mf\_liverA, CL2753.Contig2\_Mf\_liverA, CL2863.Contig1\_Mf\_liverA, CL2863.Contig2\_Mf\_liverA, CL2876.Contig1\_Mf\_liverA, CL2887.Contig1\_Mf\_liverA, CL2887.Contig2\_Mf\_liverA, CL2898.Contig1\_Mf\_liverA, CL2898.Contig2\_Mf\_liverA, CL2908.Contig1\_Mf\_liverA, CL2908.Contig2\_Mf\_liverA, CL296.Contig1\_Mf\_liverA, CL296.Contig2\_Mf\_liverA, CL2963.Contig1\_Mf\_liverA, CL2963.Contig2\_Mf\_liverA, CL2963.Contig3\_Mf\_liverA, CL2963.Contig4\_Mf\_liverA, CL2963.Contig5\_Mf\_liverA, CL3125.Contig1\_Mf\_liverA, CL3147.Contig1\_Mf\_liverA, CL3168.Contig1\_Mf\_liverA, CL3168.Contig2\_Mf\_liverA, CL3207.Contig1\_Mf\_liverA, CL3207.Contig2\_Mf\_liverA, CL3215.Contig1\_Mf\_liverA, CL3215.Contig2\_Mf\_liverA, CL3220.Contig1\_Mf\_liverA, CL3220.Contig2\_Mf\_liverA, CL3220.Contig3\_Mf\_liverA, CL3220.Contig4\_Mf\_liverA, CL3231.Contig1\_Mf\_liverA, CL3231.Contig2\_Mf\_liverA, CL3243.Contig1\_Mf\_liverA, CL3243.Contig2\_Mf\_liverA, CL3253.Contig1\_Mf\_liverA, CL3253.Contig2\_Mf\_liverA, CL3253.Contig3\_Mf\_liverA, CL3253.Contig4\_Mf\_liverA, CL3276.Contig1\_Mf\_liverA, CL3276.Contig2\_Mf\_liverA, CL3276.Contig3\_Mf\_liverA, CL3349.Contig1\_Mf\_liverA, CL3349.Contig2\_Mf\_liverA, CL3397.Contig1\_Mf\_liverA, CL3397.Contig2\_Mf\_liverA, CL3411.Contig1\_Mf\_liverA, CL3411.Contig2\_Mf\_liverA, CL3416.Contig1\_Mf\_liverA, CL3416.Contig2\_Mf\_liverA, CL3500.Contig1\_Mf\_liverA, CL3500.Contig2\_Mf\_liverA, CL3534.Contig1\_Mf\_liverA, CL3534.Contig3\_Mf\_liverA, CL3534.Contig4\_Mf\_liverA, CL3539.Contig2\_Mf\_liverA, CL3898.Contig1\_Mf\_liverA, CL3898.Contig2\_Mf\_liverA, CL4014.Contig1\_Mf\_liverA, CL4015.Contig1\_Mf\_liverA, CL4015.Contig2\_Mf\_liverA, CL4024.Contig1\_Mf\_liverA, CL4024.Contig2\_Mf\_liverA, CL4052.Contig1\_Mf\_liverA, CL4055.Contig1\_Mf\_liverA, CL4055.Contig2\_Mf\_liverA, CL410.Contig13\_Mf\_liverA, CL410.Contig14\_Mf\_liverA, CL410.Contig15\_Mf\_liverA, CL410.Contig16\_Mf\_liverA, CL410.Contig17\_Mf\_liverA, CL410.Contig9\_Mf\_liverA, CL411.Contig1\_Mf\_liverA, CL42.Contig1\_Mf\_liverA, CL42.Contig2\_Mf\_liverA, CL42.Contig3\_Mf\_liverA, CL42.Contig4\_Mf\_liverA, CL42.Contig5\_Mf\_liverA, CL42.Contig6\_Mf\_liverA, CL4254.Contig1\_Mf\_liverA, CL4254.Contig2\_Mf\_liverA, CL4286.Contig1\_Mf\_liverA, CL4286.Contig2\_Mf\_liverA, CL4338.Contig1\_Mf\_liverA, CL4338.Contig2\_Mf\_liverA, CL4388.Contig3\_Mf\_liverA, CL4407.Contig1\_Mf\_liverA, CL4407.Contig2\_Mf\_liverA, CL4447.Contig1\_Mf\_liverA, CL4447.Contig2\_Mf\_liverA, CL4447.Contig3\_Mf\_liverA, CL4447.Contig4\_Mf\_liverA, CL4460.Contig1\_Mf\_liverA, CL4460.Contig2\_Mf\_liverA, CL4507.Contig1\_Mf\_liverA, CL4518.Contig1\_Mf\_liverA, CL4598.Contig4\_Mf\_liverA, CL4605.Contig2\_Mf\_liverA, CL4610.Contig1\_Mf\_liverA, CL4610.Contig2\_Mf\_liverA, CL4616.Contig1\_Mf\_liverA, CL4616.Contig2\_Mf\_liverA, CL4618.Contig1\_Mf\_liverA, CL4618.Contig2\_Mf\_liverA, CL4643.Contig1\_Mf\_liverA, CL4643.Contig2\_Mf\_liverA, CL4643.Contig3\_Mf\_liverA, CL4643.Contig4\_Mf\_liverA, CL4643.Contig5\_Mf\_liverA, CL4643.Contig6\_Mf\_liverA, CL4664.Contig1\_Mf\_liverA, CL4664.Contig2\_Mf\_liverA, CL472.Contig2\_Mf\_liverA, CL474.Contig1\_Mf\_liverA, CL4741.Contig1\_Mf\_liverA, CL4741.Contig2\_Mf\_liverA, CL4784.Contig2\_Mf\_liverA, CL4812.Contig2\_Mf\_liverA, CL4848.Contig1\_Mf\_liverA, CL4848.Contig2\_Mf\_liverA, CL485.Contig1\_Mf\_liverA, CL485.Contig2\_Mf\_liverA, CL4892.Contig1\_Mf\_liverA, CL4918.Contig1\_Mf\_liverA, CL4918.Contig2\_Mf\_liverA, CL4935.Contig1\_Mf\_liverA, CL4935.Contig2\_Mf\_liverA, CL4936.Contig1\_Mf\_liverA, CL4936.Contig2\_Mf\_liverA, CL4952.Contig1\_Mf\_liverA, CL4952.Contig2\_Mf\_liverA, CL4993.Contig1\_Mf\_liverA, CL4993.Contig2\_Mf\_liverA, CL4998.Contig1\_Mf\_liverA, CL4998.Contig2\_Mf\_liverA, CL510.Contig1\_Mf\_liverA, CL510.Contig2\_Mf\_liverA, CL5148.Contig1\_Mf\_liverA, CL5148.Contig2\_Mf\_liverA, CL5254.Contig1\_Mf\_liverA, CL5254.Contig2\_Mf\_liverA, CL5261.Contig2\_Mf\_liverA, CL5303.Contig1\_Mf\_liverA, CL5303.Contig2\_Mf\_liverA, CL5303.Contig4\_Mf\_liverA, CL5303.Contig5\_Mf\_liverA, CL5303.Contig6\_Mf\_liverA, CL5376.Contig1\_Mf\_liverA, CL5384.Contig1\_Mf\_liverA, CL5384.Contig2\_Mf\_liverA, CL5416.Contig1\_Mf\_liverA, CL5416.Contig2\_Mf\_liverA, CL5576.Contig1\_Mf\_liverA, CL5595.Contig1\_Mf\_liverA, CL5595.Contig2\_Mf\_liverA, CL5612.Contig2\_Mf\_liverA, CL5664.Contig1\_Mf\_liverA, CL5664.Contig2\_Mf\_liverA, CL5889.Contig2\_Mf\_liverA, CL647.Contig1\_Mf\_liverA, CL647.Contig2\_Mf\_liverA, CL712.Contig5\_Mf\_liverA, CL712.Contig6\_Mf\_liverA, CL712.Contig7\_Mf\_liverA, CL712.Contig8\_Mf\_liverA, CL723.Contig1\_Mf\_liverA, CL723.Contig2\_Mf\_liverA, CL723.Contig3\_Mf\_liverA, CL734.Contig1\_Mf\_liverA, CL734.Contig2\_Mf\_liverA, CL812.Contig2\_Mf\_liverA, CL82.Contig3\_Mf\_liverA, CL82.Contig4\_Mf\_liverA, CL864.Contig2\_Mf\_liverA, CL864.Contig3\_Mf\_liverA, CL893.Contig1\_Mf\_liverA, CL893.Contig2\_Mf\_liverA, CL893.Contig3\_Mf\_liverA, CL893.Contig4\_Mf\_liverA, CL97.Contig1\_Mf\_liverA, CL97.Contig2\_Mf\_liverA, CL98.Contig1\_Mf\_liverA, CL98.Contig2\_Mf\_liverA, CL98.Contig3\_Mf\_liverA, CL98.Contig4\_Mf\_liverA, Unigene10099\_Mf\_liverA, Unigene10122\_Mf\_liverA, Unigene10139\_Mf\_liverA, Unigene10249\_Mf\_liverA, Unigene1055\_Mf\_liverA, Unigene1085\_Mf\_liverA, Unigene1088\_Mf\_liverA, Unigene10897\_Mf\_liverA, Unigene11009\_Mf\_liverA, Unigene1108\_Mf\_liverA, Unigene11227\_Mf\_liverA, Unigene11544\_Mf\_liverA, Unigene11662\_Mf\_liverA, Unigene11737\_Mf\_liverA, Unigene11749\_Mf\_liverA, Unigene11803\_Mf\_liverA, Unigene11892\_Mf\_liverA, Unigene12161\_Mf\_liverA, Unigene12262\_Mf\_liverA, Unigene12263\_Mf\_liverA, Unigene12706\_Mf\_liverA, Unigene12713\_Mf\_liverA, Unigene12774\_Mf\_liverA, Unigene12814\_Mf\_liverA, Unigene12877\_Mf\_liverA, Unigene12933\_Mf\_liverA, Unigene12951\_Mf\_liverA, Unigene12953\_Mf\_liverA, Unigene12993\_Mf\_liverA, Unigene13062\_Mf\_liverA, Unigene13169\_Mf\_liverA, Unigene13170\_Mf\_liverA, Unigene13171\_Mf\_liverA, Unigene13419\_Mf\_liverA, Unigene13695\_Mf\_liverA, Unigene13723\_Mf\_liverA, Unigene13796\_Mf\_liverA, Unigene13918\_Mf\_liverA, Unigene13939\_Mf\_liverA, Unigene14093\_Mf\_liverA, Unigene14094\_Mf\_liverA, Unigene14427\_Mf\_liverA, Unigene14428\_Mf\_liverA, Unigene14484\_Mf\_liverA, Unigene14581\_Mf\_liverA, Unigene14609\_Mf\_liverA, Unigene1461\_Mf\_liverA, Unigene14724\_Mf\_liverA, Unigene14774\_Mf\_liverA, Unigene14820\_Mf\_liverA, Unigene14841\_Mf\_liverA, Unigene14908\_Mf\_liverA, Unigene14987\_Mf\_liverA, Unigene15074\_Mf\_liverA, Unigene15075\_Mf\_liverA, Unigene15076\_Mf\_liverA, Unigene15125\_Mf\_liverA, Unigene15161\_Mf\_liverA, Unigene15164\_Mf\_liverA, Unigene15181\_Mf\_liverA, Unigene15338\_Mf\_liverA, Unigene15339\_Mf\_liverA, Unigene15352\_Mf\_liverA, Unigene15364\_Mf\_liverA, Unigene15572\_Mf\_liverA, Unigene15630\_Mf\_liverA, Unigene15855\_Mf\_liverA, Unigene15874\_Mf\_liverA, Unigene15914\_Mf\_liverA, Unigene16056\_Mf\_liverA, Unigene16062\_Mf\_liverA, Unigene16240\_Mf\_liverA, Unigene16241\_Mf\_liverA, Unigene16285\_Mf\_liverA, Unigene16545\_Mf\_liverA, Unigene16586\_Mf\_liverA, Unigene16757\_Mf\_liverA, Unigene16849\_Mf\_liverA, Unigene16890\_Mf\_liverA, Unigene16944\_Mf\_liverA, Unigene16952\_Mf\_liverA, Unigene17018\_Mf\_liverA, Unigene17019\_Mf\_liverA, Unigene17035\_Mf\_liverA, Unigene17313\_Mf\_liverA, Unigene17425\_Mf\_liverA, Unigene17426\_Mf\_liverA, Unigene17467\_Mf\_liverA, Unigene17499\_Mf\_liverA, Unigene17518\_Mf\_liverA, Unigene17571\_Mf\_liverA, Unigene17696\_Mf\_liverA, Unigene17982\_Mf\_liverA, Unigene18129\_Mf\_liverA, Unigene18177\_Mf\_liverA, Unigene1828\_Mf\_liverA, Unigene18326\_Mf\_liverA, Unigene18340\_Mf\_liverA, Unigene18428\_Mf\_liverA, Unigene18443\_Mf\_liverA, Unigene18655\_Mf\_liverA, Unigene18843\_Mf\_liverA, Unigene1896\_Mf\_liverA, Unigene19017\_Mf\_liverA, Unigene19092\_Mf\_liverA, Unigene19195\_Mf\_liverA, Unigene19196\_Mf\_liverA, Unigene19257\_Mf\_liverA, Unigene19258\_Mf\_liverA, Unigene19353\_Mf\_liverA, Unigene19522\_Mf\_liverA, Unigene19523\_Mf\_liverA, Unigene19568\_Mf\_liverA, Unigene19715\_Mf\_liverA, Unigene19716\_Mf\_liverA, Unigene19831\_Mf\_liverA, Unigene19893\_Mf\_liverA, Unigene19894\_Mf\_liverA, Unigene19973\_Mf\_liverA, Unigene19985\_Mf\_liverA, Unigene20262\_Mf\_liverA, Unigene20377\_Mf\_liverA, Unigene20378\_Mf\_liverA, Unigene20379\_Mf\_liverA, Unigene20432\_Mf\_liverA, Unigene20433\_Mf\_liverA, Unigene20472\_Mf\_liverA, Unigene2070\_Mf\_liverA, Unigene20776\_Mf\_liverA, Unigene20795\_Mf\_liverA, Unigene20829\_Mf\_liverA, Unigene2087\_Mf\_liverA, Unigene20910\_Mf\_liverA, Unigene20911\_Mf\_liverA, Unigene21275\_Mf\_liverA, Unigene21276\_Mf\_liverA, Unigene21693\_Mf\_liverA, Unigene21721\_Mf\_liverA, Unigene21816\_Mf\_liverA, Unigene21833\_Mf\_liverA, Unigene21857\_Mf\_liverA, Unigene2195\_Mf\_liverA, Unigene21991\_Mf\_liverA, Unigene22308\_Mf\_liverA, Unigene22381\_Mf\_liverA, Unigene22396\_Mf\_liverA, Unigene22556\_Mf\_liverA, Unigene22557\_Mf\_liverA, Unigene22611\_Mf\_liverA, Unigene22852\_Mf\_liverA, Unigene22919\_Mf\_liverA, Unigene22977\_Mf\_liverA, Unigene22978\_Mf\_liverA, Unigene22979\_Mf\_liverA, Unigene22980\_Mf\_liverA, Unigene23067\_Mf\_liverA, Unigene23108\_Mf\_liverA, Unigene23129\_Mf\_liverA, Unigene23130\_Mf\_liverA, Unigene23152\_Mf\_liverA, Unigene23158\_Mf\_liverA, Unigene23224\_Mf\_liverA, Unigene23328\_Mf\_liverA, Unigene23329\_Mf\_liverA, Unigene23330\_Mf\_liverA, Unigene23331\_Mf\_liverA, Unigene23334\_Mf\_liverA, Unigene23341\_Mf\_liverA, Unigene2338\_Mf\_liverA, Unigene23515\_Mf\_liverA, Unigene2376\_Mf\_liverA, Unigene2377\_Mf\_liverA, Unigene23920\_Mf\_liverA, Unigene23922\_Mf\_liverA, Unigene23923\_Mf\_liverA, Unigene23924\_Mf\_liverA, Unigene23934\_Mf\_liverA, Unigene23935\_Mf\_liverA, Unigene23936\_Mf\_liverA, Unigene23937\_Mf\_liverA, Unigene23938\_Mf\_liverA, Unigene23\_Mf\_liverA, Unigene24049\_Mf\_liverA, Unigene24065\_Mf\_liverA, Unigene24068\_Mf\_liverA, Unigene24069\_Mf\_liverA, Unigene24086\_Mf\_liverA, Unigene24142\_Mf\_liverA, Unigene24182\_Mf\_liverA, Unigene24347\_Mf\_liverA, Unigene2435\_Mf\_liverA, Unigene24576\_Mf\_liverA, Unigene24715\_Mf\_liverA, Unigene24755\_Mf\_liverA, Unigene24804\_Mf\_liverA, Unigene24805\_Mf\_liverA, Unigene24869\_Mf\_liverA, Unigene24986\_Mf\_liverA, Unigene24987\_Mf\_liverA, Unigene24988\_Mf\_liverA, Unigene25119\_Mf\_liverA, Unigene25135\_Mf\_liverA, Unigene25172\_Mf\_liverA, Unigene25187\_Mf\_liverA, Unigene25200\_Mf\_liverA, Unigene25218\_Mf\_liverA, Unigene2521\_Mf\_liverA, Unigene25341\_Mf\_liverA, Unigene25416\_Mf\_liverA, Unigene25661\_Mf\_liverA, Unigene25748\_Mf\_liverA, Unigene25765\_Mf\_liverA, Unigene25839\_Mf\_liverA, Unigene25853\_Mf\_liverA, Unigene26183\_Mf\_liverA, Unigene26195\_Mf\_liverA, Unigene26305\_Mf\_liverA, Unigene26306\_Mf\_liverA, Unigene26307\_Mf\_liverA, Unigene26384\_Mf\_liverA, Unigene26440\_Mf\_liverA, Unigene26813\_Mf\_liverA, Unigene27063\_Mf\_liverA, Unigene27221\_Mf\_liverA, Unigene27260\_Mf\_liverA, Unigene27261\_Mf\_liverA, Unigene27281\_Mf\_liverA, Unigene27323\_Mf\_liverA, Unigene27684\_Mf\_liverA, Unigene27868\_Mf\_liverA, Unigene27933\_Mf\_liverA, Unigene27934\_Mf\_liverA, Unigene27935\_Mf\_liverA, Unigene27936\_Mf\_liverA, Unigene27951\_Mf\_liverA, Unigene28283\_Mf\_liverA, Unigene28284\_Mf\_liverA, Unigene28285\_Mf\_liverA, Unigene28296\_Mf\_liverA, Unigene28331\_Mf\_liverA, Unigene28395\_Mf\_liverA, Unigene28396\_Mf\_liverA, Unigene28397\_Mf\_liverA, Unigene28398\_Mf\_liverA, Unigene28399\_Mf\_liverA, Unigene28504\_Mf\_liverA, Unigene28505\_Mf\_liverA, Unigene28564\_Mf\_liverA, Unigene28565\_Mf\_liverA, Unigene28610\_Mf\_liverA, Unigene28621\_Mf\_liverA, Unigene28623\_Mf\_liverA, Unigene2890\_Mf\_liverA, Unigene29041\_Mf\_liverA, Unigene29269\_Mf\_liverA, Unigene29301\_Mf\_liverA, Unigene29938\_Mf\_liverA, Unigene29939\_Mf\_liverA, Unigene30009\_Mf\_liverA, Unigene30010\_Mf\_liverA, Unigene30135\_Mf\_liverA, Unigene30200\_Mf\_liverA, Unigene30346\_Mf\_liverA, Unigene30458\_Mf\_liverA, Unigene30459\_Mf\_liverA, Unigene30493\_Mf\_liverA, Unigene30494\_Mf\_liverA, Unigene30495\_Mf\_liverA, Unigene3053\_Mf\_liverA, Unigene30584\_Mf\_liverA, Unigene30585\_Mf\_liverA, Unigene30586\_Mf\_liverA, Unigene30587\_Mf\_liverA, Unigene30588\_Mf\_liverA, Unigene30618\_Mf\_liverA, Unigene30619\_Mf\_liverA, Unigene30621\_Mf\_liverA, Unigene30877\_Mf\_liverA, Unigene31017\_Mf\_liverA, Unigene31325\_Mf\_liverA, Unigene31330\_Mf\_liverA, Unigene31346\_Mf\_liverA, Unigene31382\_Mf\_liverA, Unigene31440\_Mf\_liverA, Unigene31511\_Mf\_liverA, Unigene31732\_Mf\_liverA, Unigene31733\_Mf\_liverA, Unigene31852\_Mf\_liverA, Unigene31897\_Mf\_liverA, Unigene31954\_Mf\_liverA, Unigene32002\_Mf\_liverA, Unigene32003\_Mf\_liverA, Unigene32058\_Mf\_liverA, Unigene32096\_Mf\_liverA, Unigene32299\_Mf\_liverA, Unigene32334\_Mf\_liverA, Unigene32335\_Mf\_liverA, Unigene32346\_Mf\_liverA, Unigene32370\_Mf\_liverA, Unigene32396\_Mf\_liverA, Unigene32469\_Mf\_liverA, Unigene32570\_Mf\_liverA, Unigene32571\_Mf\_liverA, Unigene32631\_Mf\_liverA, Unigene32716\_Mf\_liverA, Unigene32717\_Mf\_liverA, Unigene32895\_Mf\_liverA, Unigene32909\_Mf\_liverA, Unigene32987\_Mf\_liverA, Unigene33010\_Mf\_liverA, Unigene33012\_Mf\_liverA, Unigene33024\_Mf\_liverA, Unigene33025\_Mf\_liverA, Unigene3308\_Mf\_liverA, Unigene33103\_Mf\_liverA, Unigene33104\_Mf\_liverA, Unigene33163\_Mf\_liverA, Unigene33165\_Mf\_liverA, Unigene33247\_Mf\_liverA, Unigene33262\_Mf\_liverA, Unigene33263\_Mf\_liverA, Unigene33597\_Mf\_liverA, Unigene33746\_Mf\_liverA, Unigene33768\_Mf\_liverA, Unigene33965\_Mf\_liverA, Unigene33970\_Mf\_liverA, Unigene33971\_Mf\_liverA, Unigene33972\_Mf\_liverA, Unigene34060\_Mf\_liverA, Unigene34123\_Mf\_liverA, Unigene34124\_Mf\_liverA, Unigene34179\_Mf\_liverA, Unigene34183\_Mf\_liverA, Unigene3462\_Mf\_liverA, Unigene34813\_Mf\_liverA, Unigene34847\_Mf\_liverA, Unigene34931\_Mf\_liverA, Unigene35014\_Mf\_liverA, Unigene35167\_Mf\_liverA, Unigene35196\_Mf\_liverA, Unigene3528\_Mf\_liverA, Unigene35307\_Mf\_liverA, Unigene35312\_Mf\_liverA, Unigene35313\_Mf\_liverA, Unigene35441\_Mf\_liverA, Unigene35528\_Mf\_liverA, Unigene35570\_Mf\_liverA, Unigene35571\_Mf\_liverA, Unigene35572\_Mf\_liverA, Unigene35573\_Mf\_liverA, Unigene3572\_Mf\_liverA, Unigene35737\_Mf\_liverA, Unigene35750\_Mf\_liverA, Unigene36260\_Mf\_liverA, Unigene36261\_Mf\_liverA, Unigene36273\_Mf\_liverA, Unigene36308\_Mf\_liverA, Unigene36309\_Mf\_liverA, Unigene36310\_Mf\_liverA, Unigene36311\_Mf\_liverA, Unigene36385\_Mf\_liverA, Unigene3643\_Mf\_liverA, Unigene36748\_Mf\_liverA, Unigene36843\_Mf\_liverA, Unigene37124\_Mf\_liverA, Unigene37147\_Mf\_liverA, Unigene37176\_Mf\_liverA, Unigene37358\_Mf\_liverA, Unigene37474\_Mf\_liverA, Unigene37591\_Mf\_liverA, Unigene37683\_Mf\_liverA, Unigene37729\_Mf\_liverA, Unigene37771\_Mf\_liverA, Unigene38319\_Mf\_liverA, Unigene38839\_Mf\_liverA, Unigene38870\_Mf\_liverA, Unigene38919\_Mf\_liverA, Unigene38994\_Mf\_liverA, Unigene389\_Mf\_liverA, Unigene3902\_Mf\_liverA, Unigene39099\_Mf\_liverA, Unigene3915\_Mf\_liverA, Unigene39212\_Mf\_liverA, Unigene39252\_Mf\_liverA, Unigene39355\_Mf\_liverA, Unigene39575\_Mf\_liverA, Unigene39640\_Mf\_liverA, Unigene39721\_Mf\_liverA, Unigene39816\_Mf\_liverA, Unigene39828\_Mf\_liverA, Unigene39890\_Mf\_liverA, Unigene39990\_Mf\_liverA, Unigene40205\_Mf\_liverA, Unigene40369\_Mf\_liverA, Unigene40507\_Mf\_liverA, Unigene40557\_Mf\_liverA, Unigene40962\_Mf\_liverA, Unigene41087\_Mf\_liverA, Unigene41299\_Mf\_liverA, Unigene41315\_Mf\_liverA, Unigene41505\_Mf\_liverA, Unigene41600\_Mf\_liverA, Unigene4170\_Mf\_liverA, Unigene41758\_Mf\_liverA, Unigene4195\_Mf\_liverA, Unigene4226\_Mf\_liverA, Unigene42335\_Mf\_liverA, Unigene4264\_Mf\_liverA, Unigene42852\_Mf\_liverA, Unigene43225\_Mf\_liverA, Unigene43599\_Mf\_liverA, Unigene43792\_Mf\_liverA, Unigene43912\_Mf\_liverA, Unigene44038\_Mf\_liverA, Unigene44101\_Mf\_liverA, Unigene44288\_Mf\_liverA, Unigene4459\_Mf\_liverA, Unigene44657\_Mf\_liverA, Unigene44670\_Mf\_liverA, Unigene44769\_Mf\_liverA, Unigene44819\_Mf\_liverA, Unigene4513\_Mf\_liverA, Unigene45269\_Mf\_liverA, Unigene4543\_Mf\_liverA, Unigene45703\_Mf\_liverA, Unigene45708\_Mf\_liverA, Unigene45730\_Mf\_liverA, Unigene4574\_Mf\_liverA, Unigene4575\_Mf\_liverA, Unigene45883\_Mf\_liverA, Unigene46009\_Mf\_liverA, Unigene46299\_Mf\_liverA, Unigene46368\_Mf\_liverA, Unigene46470\_Mf\_liverA, Unigene46635\_Mf\_liverA, Unigene46649\_Mf\_liverA, Unigene46720\_Mf\_liverA, Unigene4672\_Mf\_liverA, Unigene46793\_Mf\_liverA, Unigene4684\_Mf\_liverA, Unigene47261\_Mf\_liverA, Unigene47472\_Mf\_liverA, Unigene47554\_Mf\_liverA, Unigene47634\_Mf\_liverA, Unigene47772\_Mf\_liverA, Unigene47837\_Mf\_liverA, Unigene47869\_Mf\_liverA, Unigene47936\_Mf\_liverA, Unigene48072\_Mf\_liverA, Unigene48285\_Mf\_liverA, Unigene48313\_Mf\_liverA, Unigene48493\_Mf\_liverA, Unigene48526\_Mf\_liverA, Unigene48539\_Mf\_liverA, Unigene48671\_Mf\_liverA, Unigene486\_Mf\_liverA, Unigene4871\_Mf\_liverA, Unigene48762\_Mf\_liverA, Unigene48857\_Mf\_liverA, Unigene4916\_Mf\_liverA, Unigene49422\_Mf\_liverA, Unigene496\_Mf\_liverA, Unigene49797\_Mf\_liverA, Unigene49845\_Mf\_liverA, Unigene4985\_Mf\_liverA, Unigene49920\_Mf\_liverA, Unigene50036\_Mf\_liverA, Unigene50319\_Mf\_liverA, Unigene50360\_Mf\_liverA, Unigene50600\_Mf\_liverA, Unigene5088\_Mf\_liverA, Unigene51086\_Mf\_liverA, Unigene51321\_Mf\_liverA, Unigene5134\_Mf\_liverA, Unigene51616\_Mf\_liverA, Unigene5226\_Mf\_liverA, Unigene5245\_Mf\_liverA, Unigene5381\_Mf\_liverA, Unigene5405\_Mf\_liverA, Unigene5434\_Mf\_liverA, Unigene5501\_Mf\_liverA, Unigene5606\_Mf\_liverA, Unigene5648\_Mf\_liverA, Unigene5772\_Mf\_liverA, Unigene583\_Mf\_liverA, Unigene5880\_Mf\_liverA, Unigene5983\_Mf\_liverA, Unigene5984\_Mf\_liverA, Unigene6063\_Mf\_liverA, Unigene6654\_Mf\_liverA, Unigene7104\_Mf\_liverA, Unigene7105\_Mf\_liverA, Unigene7146\_Mf\_liverA, Unigene7150\_Mf\_liverA, Unigene7350\_Mf\_liverA, Unigene7351\_Mf\_liverA, Unigene7498\_Mf\_liverA, Unigene7514\_Mf\_liverA, Unigene7539\_Mf\_liverA, Unigene7612\_Mf\_liverA, Unigene7725\_Mf\_liverA, Unigene7906\_Mf\_liverA, Unigene80\_Mf\_liverA, Unigene8109\_Mf\_liverA, Unigene8270\_Mf\_liverA, Unigene8360\_Mf\_liverA, Unigene8498\_Mf\_liverA, Unigene8587\_Mf\_liverA, Unigene8624\_Mf\_liverA, Unigene8811\_Mf\_liverA, Unigene8878\_Mf\_liverA, Unigene88\_Mf\_liverA, Unigene9051\_Mf\_liverA, Unigene9059\_Mf\_liverA, Unigene9060\_Mf\_liverA, Unigene919\_Mf\_liverA, Unigene9201\_Mf\_liverA, Unigene9375\_Mf\_liverA, Unigene93\_Mf\_liverA, Unigene9466\_Mf\_liverA, Unigene9501\_Mf\_liverA, Unigene9577\_Mf\_liverA, Unigene9621\_Mf\_liverA, Unigene9762\_Mf\_liverA, Unigene9905\_Mf\_liverA |
| 5 | Endocytosis | CL1002.Contig1\_Mf\_liverA, CL1002.Contig2\_Mf\_liverA, CL1013.Contig5\_Mf\_liverA, CL1038.Contig1\_Mf\_liverA, CL1038.Contig2\_Mf\_liverA, CL1038.Contig3\_Mf\_liverA, CL1038.Contig4\_Mf\_liverA, CL1038.Contig5\_Mf\_liverA, CL107.Contig1\_Mf\_liverA, CL107.Contig2\_Mf\_liverA, CL107.Contig3\_Mf\_liverA, CL107.Contig4\_Mf\_liverA, CL107.Contig5\_Mf\_liverA, CL1129.Contig1\_Mf\_liverA, CL1129.Contig2\_Mf\_liverA, CL1147.Contig1\_Mf\_liverA, CL1147.Contig2\_Mf\_liverA, CL1147.Contig3\_Mf\_liverA, CL116.Contig1\_Mf\_liverA, CL1199.Contig10\_Mf\_liverA, CL1199.Contig11\_Mf\_liverA, CL1199.Contig12\_Mf\_liverA, CL1199.Contig14\_Mf\_liverA, CL1199.Contig15\_Mf\_liverA, CL1199.Contig16\_Mf\_liverA, CL1199.Contig17\_Mf\_liverA, CL1199.Contig1\_Mf\_liverA, CL1199.Contig2\_Mf\_liverA, CL1199.Contig3\_Mf\_liverA, CL1199.Contig6\_Mf\_liverA, CL1199.Contig7\_Mf\_liverA, CL1199.Contig8\_Mf\_liverA, CL1199.Contig9\_Mf\_liverA, CL1202.Contig1\_Mf\_liverA, CL1265.Contig1\_Mf\_liverA, CL1322.Contig2\_Mf\_liverA, CL1362.Contig1\_Mf\_liverA, CL1362.Contig2\_Mf\_liverA, CL1384.Contig1\_Mf\_liverA, CL1384.Contig2\_Mf\_liverA, CL1422.Contig1\_Mf\_liverA, CL1422.Contig2\_Mf\_liverA, CL143.Contig1\_Mf\_liverA, CL143.Contig2\_Mf\_liverA, CL143.Contig3\_Mf\_liverA, CL143.Contig4\_Mf\_liverA, CL1437.Contig1\_Mf\_liverA, CL1437.Contig2\_Mf\_liverA, CL1575.Contig1\_Mf\_liverA, CL1575.Contig2\_Mf\_liverA, CL1598.Contig1\_Mf\_liverA, CL1598.Contig2\_Mf\_liverA, CL1598.Contig3\_Mf\_liverA, CL1598.Contig4\_Mf\_liverA, CL1598.Contig5\_Mf\_liverA, CL1598.Contig6\_Mf\_liverA, CL1613.Contig1\_Mf\_liverA, CL1613.Contig2\_Mf\_liverA, CL1619.Contig1\_Mf\_liverA, CL1619.Contig3\_Mf\_liverA, CL1622.Contig5\_Mf\_liverA, CL1622.Contig7\_Mf\_liverA, CL1646.Contig1\_Mf\_liverA, CL1647.Contig1\_Mf\_liverA, CL1647.Contig2\_Mf\_liverA, CL1653.Contig1\_Mf\_liverA, CL1653.Contig2\_Mf\_liverA, CL1743.Contig14\_Mf\_liverA, CL1743.Contig1\_Mf\_liverA, CL1743.Contig9\_Mf\_liverA, CL1805.Contig1\_Mf\_liverA, CL1805.Contig2\_Mf\_liverA, CL1840.Contig1\_Mf\_liverA, CL1919.Contig1\_Mf\_liverA, CL1919.Contig2\_Mf\_liverA, CL1958.Contig1\_Mf\_liverA, CL2001.Contig1\_Mf\_liverA, CL2001.Contig2\_Mf\_liverA, CL2029.Contig1\_Mf\_liverA, CL2029.Contig2\_Mf\_liverA, CL2033.Contig3\_Mf\_liverA, CL2033.Contig4\_Mf\_liverA, CL2047.Contig2\_Mf\_liverA, CL2047.Contig4\_Mf\_liverA, CL2047.Contig6\_Mf\_liverA, CL2075.Contig1\_Mf\_liverA, CL2075.Contig2\_Mf\_liverA, CL2084.Contig1\_Mf\_liverA, CL2084.Contig2\_Mf\_liverA, CL2084.Contig3\_Mf\_liverA, CL2084.Contig4\_Mf\_liverA, CL2125.Contig1\_Mf\_liverA, CL2125.Contig2\_Mf\_liverA, CL2133.Contig1\_Mf\_liverA, CL2133.Contig2\_Mf\_liverA, CL2189.Contig1\_Mf\_liverA, CL2189.Contig2\_Mf\_liverA, CL2211.Contig1\_Mf\_liverA, CL2211.Contig2\_Mf\_liverA, CL2211.Contig3\_Mf\_liverA, CL2211.Contig4\_Mf\_liverA, CL2229.Contig1\_Mf\_liverA, CL2229.Contig2\_Mf\_liverA, CL2271.Contig1\_Mf\_liverA, CL2271.Contig2\_Mf\_liverA, CL233.Contig2\_Mf\_liverA, CL233.Contig3\_Mf\_liverA, CL233.Contig4\_Mf\_liverA, CL233.Contig5\_Mf\_liverA, CL233.Contig6\_Mf\_liverA, CL233.Contig7\_Mf\_liverA, CL233.Contig8\_Mf\_liverA, CL2405.Contig2\_Mf\_liverA, CL2457.Contig1\_Mf\_liverA, CL2457.Contig2\_Mf\_liverA, CL2457.Contig3\_Mf\_liverA, CL2457.Contig4\_Mf\_liverA, CL2457.Contig5\_Mf\_liverA, CL2457.Contig6\_Mf\_liverA, CL2457.Contig7\_Mf\_liverA, CL2483.Contig1\_Mf\_liverA, CL2483.Contig2\_Mf\_liverA, CL2553.Contig1\_Mf\_liverA, CL2615.Contig1\_Mf\_liverA, CL2615.Contig2\_Mf\_liverA, CL2616.Contig1\_Mf\_liverA, CL2635.Contig1\_Mf\_liverA, CL2635.Contig2\_Mf\_liverA, CL2635.Contig3\_Mf\_liverA, CL2664.Contig1\_Mf\_liverA, CL2664.Contig2\_Mf\_liverA, CL2664.Contig3\_Mf\_liverA, CL2664.Contig4\_Mf\_liverA, CL2737.Contig1\_Mf\_liverA, CL2737.Contig2\_Mf\_liverA, CL2737.Contig5\_Mf\_liverA, CL2737.Contig6\_Mf\_liverA, CL275.Contig1\_Mf\_liverA, CL275.Contig2\_Mf\_liverA, CL275.Contig3\_Mf\_liverA, CL275.Contig4\_Mf\_liverA, CL275.Contig5\_Mf\_liverA, CL2969.Contig1\_Mf\_liverA, CL2969.Contig2\_Mf\_liverA, CL2994.Contig1\_Mf\_liverA, CL2994.Contig2\_Mf\_liverA, CL2996.Contig10\_Mf\_liverA, CL2996.Contig11\_Mf\_liverA, CL2996.Contig1\_Mf\_liverA, CL2996.Contig2\_Mf\_liverA, CL2996.Contig3\_Mf\_liverA, CL2996.Contig4\_Mf\_liverA, CL2996.Contig5\_Mf\_liverA, CL2996.Contig6\_Mf\_liverA, CL2996.Contig7\_Mf\_liverA, CL2996.Contig8\_Mf\_liverA, CL2996.Contig9\_Mf\_liverA, CL3063.Contig1\_Mf\_liverA, CL3081.Contig1\_Mf\_liverA, CL3154.Contig1\_Mf\_liverA, CL3154.Contig2\_Mf\_liverA, CL3173.Contig1\_Mf\_liverA, CL3173.Contig2\_Mf\_liverA, CL3231.Contig2\_Mf\_liverA, CL3239.Contig1\_Mf\_liverA, CL3239.Contig2\_Mf\_liverA, CL3338.Contig1\_Mf\_liverA, CL3338.Contig2\_Mf\_liverA, CL3350.Contig1\_Mf\_liverA, CL3350.Contig2\_Mf\_liverA, CL3359.Contig1\_Mf\_liverA, CL3359.Contig2\_Mf\_liverA, CL3446.Contig1\_Mf\_liverA, CL3446.Contig2\_Mf\_liverA, CL3616.Contig1\_Mf\_liverA, CL3616.Contig2\_Mf\_liverA, CL371.Contig2\_Mf\_liverA, CL371.Contig3\_Mf\_liverA, CL371.Contig4\_Mf\_liverA, CL371.Contig5\_Mf\_liverA, CL3946.Contig1\_Mf\_liverA, CL3998.Contig1\_Mf\_liverA, CL3998.Contig2\_Mf\_liverA, CL4008.Contig2\_Mf\_liverA, CL4038.Contig2\_Mf\_liverA, CL4208.Contig1\_Mf\_liverA, CL4208.Contig2\_Mf\_liverA, CL4217.Contig1\_Mf\_liverA, CL4217.Contig2\_Mf\_liverA, CL4264.Contig1\_Mf\_liverA, CL4264.Contig2\_Mf\_liverA, CL4303.Contig1\_Mf\_liverA, CL4303.Contig2\_Mf\_liverA, CL4323.Contig1\_Mf\_liverA, CL4338.Contig1\_Mf\_liverA, CL4338.Contig2\_Mf\_liverA, CL4375.Contig1\_Mf\_liverA, CL4375.Contig2\_Mf\_liverA, CL4409.Contig1\_Mf\_liverA, CL4420.Contig1\_Mf\_liverA, CL4447.Contig3\_Mf\_liverA, CL4447.Contig4\_Mf\_liverA, CL4459.Contig1\_Mf\_liverA, CL4459.Contig2\_Mf\_liverA, CL4655.Contig1\_Mf\_liverA, CL4655.Contig2\_Mf\_liverA, CL4655.Contig3\_Mf\_liverA, CL4713.Contig1\_Mf\_liverA, CL4713.Contig2\_Mf\_liverA, CL4724.Contig1\_Mf\_liverA, CL4724.Contig2\_Mf\_liverA, CL4724.Contig3\_Mf\_liverA, CL4801.Contig1\_Mf\_liverA, CL4823.Contig1\_Mf\_liverA, CL4823.Contig2\_Mf\_liverA, CL488.Contig1\_Mf\_liverA, CL488.Contig2\_Mf\_liverA, CL4901.Contig1\_Mf\_liverA, CL4901.Contig2\_Mf\_liverA, CL4921.Contig1\_Mf\_liverA, CL4921.Contig2\_Mf\_liverA, CL4943.Contig1\_Mf\_liverA, CL4943.Contig2\_Mf\_liverA, CL497.Contig1\_Mf\_liverA, CL497.Contig2\_Mf\_liverA, CL5005.Contig1\_Mf\_liverA, CL5005.Contig2\_Mf\_liverA, CL5018.Contig1\_Mf\_liverA, CL5018.Contig2\_Mf\_liverA, CL503.Contig1\_Mf\_liverA, CL503.Contig2\_Mf\_liverA, CL503.Contig3\_Mf\_liverA, CL503.Contig4\_Mf\_liverA, CL503.Contig5\_Mf\_liverA, CL5040.Contig1\_Mf\_liverA, CL5061.Contig2\_Mf\_liverA, CL515.Contig1\_Mf\_liverA, CL515.Contig3\_Mf\_liverA, CL515.Contig4\_Mf\_liverA, CL5157.Contig1\_Mf\_liverA, CL5207.Contig1\_Mf\_liverA, CL5207.Contig2\_Mf\_liverA, CL5249.Contig1\_Mf\_liverA, CL5249.Contig2\_Mf\_liverA, CL5303.Contig1\_Mf\_liverA, CL5303.Contig2\_Mf\_liverA, CL5303.Contig4\_Mf\_liverA, CL5303.Contig5\_Mf\_liverA, CL5303.Contig6\_Mf\_liverA, CL5444.Contig1\_Mf\_liverA, CL5486.Contig1\_Mf\_liverA, CL5486.Contig2\_Mf\_liverA, CL5507.Contig1\_Mf\_liverA, CL5507.Contig2\_Mf\_liverA, CL5507.Contig3\_Mf\_liverA, CL5594.Contig1\_Mf\_liverA, CL5595.Contig1\_Mf\_liverA, CL5595.Contig2\_Mf\_liverA, CL5614.Contig1\_Mf\_liverA, CL5614.Contig2\_Mf\_liverA, CL5672.Contig1\_Mf\_liverA, CL5672.Contig3\_Mf\_liverA, CL5772.Contig1\_Mf\_liverA, CL5772.Contig2\_Mf\_liverA, CL5772.Contig3\_Mf\_liverA, CL5821.Contig1\_Mf\_liverA, CL5823.Contig1\_Mf\_liverA, CL5950.Contig1\_Mf\_liverA, CL5950.Contig2\_Mf\_liverA, CL5950.Contig3\_Mf\_liverA, CL596.Contig1\_Mf\_liverA, CL596.Contig2\_Mf\_liverA, CL5970.Contig1\_Mf\_liverA, CL5970.Contig2\_Mf\_liverA, CL5974.Contig1\_Mf\_liverA, CL5974.Contig2\_Mf\_liverA, CL5978.Contig2\_Mf\_liverA, CL5978.Contig3\_Mf\_liverA, CL5987.Contig1\_Mf\_liverA, CL5987.Contig2\_Mf\_liverA, CL692.Contig1\_Mf\_liverA, CL692.Contig2\_Mf\_liverA, CL692.Contig3\_Mf\_liverA, CL729.Contig1\_Mf\_liverA, CL729.Contig2\_Mf\_liverA, CL729.Contig3\_Mf\_liverA, CL729.Contig4\_Mf\_liverA, CL777.Contig7\_Mf\_liverA, CL777.Contig8\_Mf\_liverA, CL779.Contig2\_Mf\_liverA, CL779.Contig3\_Mf\_liverA, CL779.Contig4\_Mf\_liverA, CL825.Contig3\_Mf\_liverA, CL838.Contig1\_Mf\_liverA, CL838.Contig3\_Mf\_liverA, CL838.Contig4\_Mf\_liverA, CL838.Contig5\_Mf\_liverA, CL838.Contig6\_Mf\_liverA, CL839.Contig1\_Mf\_liverA, CL839.Contig2\_Mf\_liverA, CL863.Contig3\_Mf\_liverA, CL863.Contig4\_Mf\_liverA, CL946.Contig1\_Mf\_liverA, CL946.Contig2\_Mf\_liverA, CL953.Contig1\_Mf\_liverA, CL953.Contig2\_Mf\_liverA, CL953.Contig3\_Mf\_liverA, Unigene10029\_Mf\_liverA, Unigene10249\_Mf\_liverA, Unigene10279\_Mf\_liverA, Unigene10378\_Mf\_liverA, Unigene10481\_Mf\_liverA, Unigene10482\_Mf\_liverA, Unigene10534\_Mf\_liverA, Unigene10589\_Mf\_liverA, Unigene11420\_Mf\_liverA, Unigene1164\_Mf\_liverA, Unigene11673\_Mf\_liverA, Unigene11688\_Mf\_liverA, Unigene1168\_Mf\_liverA, Unigene1178\_Mf\_liverA, Unigene1183\_Mf\_liverA, Unigene1184\_Mf\_liverA, Unigene1185\_Mf\_liverA, Unigene1186\_Mf\_liverA, Unigene11890\_Mf\_liverA, Unigene1200\_Mf\_liverA, Unigene1221\_Mf\_liverA, Unigene12491\_Mf\_liverA, Unigene12538\_Mf\_liverA, Unigene12713\_Mf\_liverA, Unigene12774\_Mf\_liverA, Unigene12814\_Mf\_liverA, Unigene1292\_Mf\_liverA, Unigene12964\_Mf\_liverA, Unigene13000\_Mf\_liverA, Unigene13001\_Mf\_liverA, Unigene13033\_Mf\_liverA, Unigene1309\_Mf\_liverA, Unigene13114\_Mf\_liverA, Unigene13147\_Mf\_liverA, Unigene13437\_Mf\_liverA, Unigene13463\_Mf\_liverA, Unigene13572\_Mf\_liverA, Unigene13761\_Mf\_liverA, Unigene13981\_Mf\_liverA, Unigene13993\_Mf\_liverA, Unigene14020\_Mf\_liverA, Unigene14021\_Mf\_liverA, Unigene14082\_Mf\_liverA, Unigene1408\_Mf\_liverA, Unigene14094\_Mf\_liverA, Unigene14237\_Mf\_liverA, Unigene14244\_Mf\_liverA, Unigene1440\_Mf\_liverA, Unigene14549\_Mf\_liverA, Unigene14609\_Mf\_liverA, Unigene14719\_Mf\_liverA, Unigene15077\_Mf\_liverA, Unigene15186\_Mf\_liverA, Unigene15206\_Mf\_liverA, Unigene15316\_Mf\_liverA, Unigene15669\_Mf\_liverA, Unigene16107\_Mf\_liverA, Unigene16180\_Mf\_liverA, Unigene16240\_Mf\_liverA, Unigene16241\_Mf\_liverA, Unigene16364\_Mf\_liverA, Unigene16365\_Mf\_liverA, Unigene16366\_Mf\_liverA, Unigene16427\_Mf\_liverA, Unigene16458\_Mf\_liverA, Unigene17073\_Mf\_liverA, Unigene17074\_Mf\_liverA, Unigene1708\_Mf\_liverA, Unigene18017\_Mf\_liverA, Unigene18018\_Mf\_liverA, Unigene18129\_Mf\_liverA, Unigene18385\_Mf\_liverA, Unigene18443\_Mf\_liverA, Unigene18537\_Mf\_liverA, Unigene18708\_Mf\_liverA, Unigene18722\_Mf\_liverA, Unigene18723\_Mf\_liverA, Unigene19274\_Mf\_liverA, Unigene19275\_Mf\_liverA, Unigene19687\_Mf\_liverA, Unigene19991\_Mf\_liverA, Unigene20178\_Mf\_liverA, Unigene20466\_Mf\_liverA, Unigene20692\_Mf\_liverA, Unigene20693\_Mf\_liverA, Unigene20696\_Mf\_liverA, Unigene20978\_Mf\_liverA, Unigene21421\_Mf\_liverA, Unigene21666\_Mf\_liverA, Unigene22161\_Mf\_liverA, Unigene22162\_Mf\_liverA, Unigene22163\_Mf\_liverA, Unigene22165\_Mf\_liverA, Unigene22166\_Mf\_liverA, Unigene22167\_Mf\_liverA, Unigene22168\_Mf\_liverA, Unigene2226\_Mf\_liverA, Unigene22330\_Mf\_liverA, Unigene22825\_Mf\_liverA, Unigene2282\_Mf\_liverA, Unigene230\_Mf\_liverA, Unigene23274\_Mf\_liverA, Unigene23275\_Mf\_liverA, Unigene23435\_Mf\_liverA, Unigene23560\_Mf\_liverA, Unigene23697\_Mf\_liverA, Unigene24233\_Mf\_liverA, Unigene24255\_Mf\_liverA, Unigene24333\_Mf\_liverA, Unigene24489\_Mf\_liverA, Unigene24909\_Mf\_liverA, Unigene25044\_Mf\_liverA, Unigene25045\_Mf\_liverA, Unigene2530\_Mf\_liverA, Unigene25430\_Mf\_liverA, Unigene25431\_Mf\_liverA, Unigene25504\_Mf\_liverA, Unigene25506\_Mf\_liverA, Unigene25576\_Mf\_liverA, Unigene25645\_Mf\_liverA, Unigene25683\_Mf\_liverA, Unigene25724\_Mf\_liverA, Unigene25726\_Mf\_liverA, Unigene25825\_Mf\_liverA, Unigene2584\_Mf\_liverA, Unigene25854\_Mf\_liverA, Unigene2587\_Mf\_liverA, Unigene26029\_Mf\_liverA, Unigene26117\_Mf\_liverA, Unigene26360\_Mf\_liverA, Unigene26377\_Mf\_liverA, Unigene26378\_Mf\_liverA, Unigene26413\_Mf\_liverA, Unigene26491\_Mf\_liverA, Unigene266\_Mf\_liverA, Unigene26712\_Mf\_liverA, Unigene26924\_Mf\_liverA, Unigene26985\_Mf\_liverA, Unigene27022\_Mf\_liverA, Unigene27137\_Mf\_liverA, Unigene27138\_Mf\_liverA, Unigene27227\_Mf\_liverA, Unigene27230\_Mf\_liverA, Unigene27231\_Mf\_liverA, Unigene27260\_Mf\_liverA, Unigene27261\_Mf\_liverA, Unigene27385\_Mf\_liverA, Unigene27386\_Mf\_liverA, Unigene27387\_Mf\_liverA, Unigene27553\_Mf\_liverA, Unigene27593\_Mf\_liverA, Unigene27594\_Mf\_liverA, Unigene27630\_Mf\_liverA, Unigene27631\_Mf\_liverA, Unigene2791\_Mf\_liverA, Unigene27937\_Mf\_liverA, Unigene27966\_Mf\_liverA, Unigene2798\_Mf\_liverA, Unigene28244\_Mf\_liverA, Unigene28245\_Mf\_liverA, Unigene28331\_Mf\_liverA, Unigene28335\_Mf\_liverA, Unigene28336\_Mf\_liverA, Unigene28338\_Mf\_liverA, Unigene28405\_Mf\_liverA, Unigene28443\_Mf\_liverA, Unigene28444\_Mf\_liverA, Unigene28445\_Mf\_liverA, Unigene28446\_Mf\_liverA, Unigene28520\_Mf\_liverA, Unigene28842\_Mf\_liverA, Unigene28922\_Mf\_liverA, Unigene28950\_Mf\_liverA, Unigene28952\_Mf\_liverA, Unigene29115\_Mf\_liverA, Unigene29399\_Mf\_liverA, Unigene29486\_Mf\_liverA, Unigene29488\_Mf\_liverA, Unigene29497\_Mf\_liverA, Unigene29788\_Mf\_liverA, Unigene29849\_Mf\_liverA, Unigene29899\_Mf\_liverA, Unigene29969\_Mf\_liverA, Unigene29987\_Mf\_liverA, Unigene29988\_Mf\_liverA, Unigene29989\_Mf\_liverA, Unigene30122\_Mf\_liverA, Unigene30373\_Mf\_liverA, Unigene30448\_Mf\_liverA, Unigene30530\_Mf\_liverA, Unigene30642\_Mf\_liverA, Unigene30691\_Mf\_liverA, Unigene30692\_Mf\_liverA, Unigene30694\_Mf\_liverA, Unigene30825\_Mf\_liverA, Unigene30877\_Mf\_liverA, Unigene30911\_Mf\_liverA, Unigene31022\_Mf\_liverA, Unigene31118\_Mf\_liverA, Unigene31119\_Mf\_liverA, Unigene31196\_Mf\_liverA, Unigene31325\_Mf\_liverA, Unigene31366\_Mf\_liverA, Unigene31521\_Mf\_liverA, Unigene31673\_Mf\_liverA, Unigene31709\_Mf\_liverA, Unigene31710\_Mf\_liverA, Unigene31711\_Mf\_liverA, Unigene31727\_Mf\_liverA, Unigene31789\_Mf\_liverA, Unigene31872\_Mf\_liverA, Unigene31941\_Mf\_liverA, Unigene32029\_Mf\_liverA, Unigene32058\_Mf\_liverA, Unigene32126\_Mf\_liverA, Unigene32169\_Mf\_liverA, Unigene32206\_Mf\_liverA, Unigene32310\_Mf\_liverA, Unigene32334\_Mf\_liverA, Unigene32335\_Mf\_liverA, Unigene32568\_Mf\_liverA, Unigene32583\_Mf\_liverA, Unigene32592\_Mf\_liverA, Unigene32633\_Mf\_liverA, Unigene32783\_Mf\_liverA, Unigene32935\_Mf\_liverA, Unigene32987\_Mf\_liverA, Unigene33079\_Mf\_liverA, Unigene33080\_Mf\_liverA, Unigene33184\_Mf\_liverA, Unigene33364\_Mf\_liverA, Unigene33365\_Mf\_liverA, Unigene33451\_Mf\_liverA, Unigene33642\_Mf\_liverA, Unigene33698\_Mf\_liverA, Unigene33746\_Mf\_liverA, Unigene33848\_Mf\_liverA, Unigene33953\_Mf\_liverA, Unigene33954\_Mf\_liverA, Unigene33965\_Mf\_liverA, Unigene34236\_Mf\_liverA, Unigene34299\_Mf\_liverA, Unigene34333\_Mf\_liverA, Unigene34446\_Mf\_liverA, Unigene34588\_Mf\_liverA, Unigene34712\_Mf\_liverA, Unigene34777\_Mf\_liverA, Unigene34973\_Mf\_liverA, Unigene34974\_Mf\_liverA, Unigene34975\_Mf\_liverA, Unigene35147\_Mf\_liverA, Unigene35276\_Mf\_liverA, Unigene35312\_Mf\_liverA, Unigene35313\_Mf\_liverA, Unigene35341\_Mf\_liverA, Unigene35535\_Mf\_liverA, Unigene35537\_Mf\_liverA, Unigene3568\_Mf\_liverA, Unigene35695\_Mf\_liverA, Unigene35750\_Mf\_liverA, Unigene35811\_Mf\_liverA, Unigene35816\_Mf\_liverA, Unigene35866\_Mf\_liverA, Unigene35867\_Mf\_liverA, Unigene35898\_Mf\_liverA, Unigene35899\_Mf\_liverA, Unigene35900\_Mf\_liverA, Unigene35901\_Mf\_liverA, Unigene35902\_Mf\_liverA, Unigene35996\_Mf\_liverA, Unigene36049\_Mf\_liverA, Unigene36123\_Mf\_liverA, Unigene36124\_Mf\_liverA, Unigene36288\_Mf\_liverA, Unigene36289\_Mf\_liverA, Unigene36357\_Mf\_liverA, Unigene36539\_Mf\_liverA, Unigene36543\_Mf\_liverA, Unigene36748\_Mf\_liverA, Unigene36819\_Mf\_liverA, Unigene36849\_Mf\_liverA, Unigene36868\_Mf\_liverA, Unigene36870\_Mf\_liverA, Unigene36893\_Mf\_liverA, Unigene36912\_Mf\_liverA, Unigene36961\_Mf\_liverA, Unigene37083\_Mf\_liverA, Unigene37150\_Mf\_liverA, Unigene37188\_Mf\_liverA, Unigene37553\_Mf\_liverA, Unigene37656\_Mf\_liverA, Unigene37802\_Mf\_liverA, Unigene38831\_Mf\_liverA, Unigene38839\_Mf\_liverA, Unigene39466\_Mf\_liverA, Unigene3951\_Mf\_liverA, Unigene39537\_Mf\_liverA, Unigene39806\_Mf\_liverA, Unigene40190\_Mf\_liverA, Unigene40629\_Mf\_liverA, Unigene40741\_Mf\_liverA, Unigene40778\_Mf\_liverA, Unigene40871\_Mf\_liverA, Unigene41299\_Mf\_liverA, Unigene41436\_Mf\_liverA, Unigene41645\_Mf\_liverA, Unigene41679\_Mf\_liverA, Unigene4170\_Mf\_liverA, Unigene41876\_Mf\_liverA, Unigene42323\_Mf\_liverA, Unigene42352\_Mf\_liverA, Unigene42489\_Mf\_liverA, Unigene42736\_Mf\_liverA, Unigene42830\_Mf\_liverA, Unigene43163\_Mf\_liverA, Unigene43225\_Mf\_liverA, Unigene43344\_Mf\_liverA, Unigene43346\_Mf\_liverA, Unigene43368\_Mf\_liverA, Unigene43640\_Mf\_liverA, Unigene43763\_Mf\_liverA, Unigene43900\_Mf\_liverA, Unigene43908\_Mf\_liverA, Unigene4422\_Mf\_liverA, Unigene442\_Mf\_liverA, Unigene44372\_Mf\_liverA, Unigene44741\_Mf\_liverA, Unigene44842\_Mf\_liverA, Unigene45781\_Mf\_liverA, Unigene4653\_Mf\_liverA, Unigene47132\_Mf\_liverA, Unigene47448\_Mf\_liverA, Unigene47472\_Mf\_liverA, Unigene47798\_Mf\_liverA, Unigene4803\_Mf\_liverA, Unigene48052\_Mf\_liverA, Unigene48645\_Mf\_liverA, Unigene48792\_Mf\_liverA, Unigene48879\_Mf\_liverA, Unigene48987\_Mf\_liverA, Unigene4938\_Mf\_liverA, Unigene49422\_Mf\_liverA, Unigene49725\_Mf\_liverA, Unigene49889\_Mf\_liverA, Unigene51973\_Mf\_liverA, Unigene5199\_Mf\_liverA, Unigene5438\_Mf\_liverA, Unigene5605\_Mf\_liverA, Unigene5864\_Mf\_liverA, Unigene5975\_Mf\_liverA, Unigene5983\_Mf\_liverA, Unigene5984\_Mf\_liverA, Unigene6143\_Mf\_liverA, Unigene6151\_Mf\_liverA, Unigene6497\_Mf\_liverA, Unigene6540\_Mf\_liverA, Unigene6753\_Mf\_liverA, Unigene6816\_Mf\_liverA, Unigene6817\_Mf\_liverA, Unigene7003\_Mf\_liverA, Unigene7118\_Mf\_liverA, Unigene717\_Mf\_liverA, Unigene7312\_Mf\_liverA, Unigene7330\_Mf\_liverA, Unigene742\_Mf\_liverA, Unigene7589\_Mf\_liverA, Unigene7709\_Mf\_liverA, Unigene8024\_Mf\_liverA, Unigene8025\_Mf\_liverA, Unigene8031\_Mf\_liverA, Unigene8199\_Mf\_liverA, Unigene8204\_Mf\_liverA, Unigene836\_Mf\_liverA, Unigene897\_Mf\_liverA, Unigene898\_Mf\_liverA, Unigene9470\_Mf\_liverA, Unigene94\_Mf\_liverA, Unigene9893\_Mf\_liverA |
| 6 | RNA transport | CL1000.Contig1\_Mf\_liverA, CL1000.Contig2\_Mf\_liverA, CL1000.Contig3\_Mf\_liverA, CL1064.Contig1\_Mf\_liverA, CL1138.Contig2\_Mf\_liverA, CL1171.Contig4\_Mf\_liverA, CL1234.Contig1\_Mf\_liverA, CL1234.Contig2\_Mf\_liverA, CL1250.Contig1\_Mf\_liverA, CL1250.Contig2\_Mf\_liverA, CL1250.Contig3\_Mf\_liverA, CL1250.Contig4\_Mf\_liverA, CL1250.Contig5\_Mf\_liverA, CL1250.Contig6\_Mf\_liverA, CL1250.Contig7\_Mf\_liverA, CL1250.Contig8\_Mf\_liverA, CL1273.Contig2\_Mf\_liverA, CL1282.Contig1\_Mf\_liverA, CL1282.Contig2\_Mf\_liverA, CL1402.Contig1\_Mf\_liverA, CL1402.Contig2\_Mf\_liverA, CL1407.Contig2\_Mf\_liverA, CL1407.Contig3\_Mf\_liverA, CL1444.Contig1\_Mf\_liverA, CL1444.Contig2\_Mf\_liverA, CL1444.Contig3\_Mf\_liverA, CL1444.Contig4\_Mf\_liverA, CL1444.Contig5\_Mf\_liverA, CL1444.Contig6\_Mf\_liverA, CL1460.Contig1\_Mf\_liverA, CL1460.Contig2\_Mf\_liverA, CL1585.Contig3\_Mf\_liverA, CL1685.Contig9\_Mf\_liverA, CL1731.Contig1\_Mf\_liverA, CL1735.Contig1\_Mf\_liverA, CL1735.Contig2\_Mf\_liverA, CL1758.Contig1\_Mf\_liverA, CL1780.Contig1\_Mf\_liverA, CL1780.Contig2\_Mf\_liverA, CL1782.Contig1\_Mf\_liverA, CL1782.Contig2\_Mf\_liverA, CL1782.Contig3\_Mf\_liverA, CL1939.Contig3\_Mf\_liverA, CL1939.Contig4\_Mf\_liverA, CL2017.Contig1\_Mf\_liverA, CL2017.Contig2\_Mf\_liverA, CL2017.Contig3\_Mf\_liverA, CL2017.Contig4\_Mf\_liverA, CL2018.Contig1\_Mf\_liverA, CL2018.Contig2\_Mf\_liverA, CL2024.Contig1\_Mf\_liverA, CL2024.Contig2\_Mf\_liverA, CL2033.Contig3\_Mf\_liverA, CL2033.Contig4\_Mf\_liverA, CL2039.Contig1\_Mf\_liverA, CL2040.Contig1\_Mf\_liverA, CL2064.Contig1\_Mf\_liverA, CL2064.Contig2\_Mf\_liverA, CL2108.Contig1\_Mf\_liverA, CL2108.Contig2\_Mf\_liverA, CL2108.Contig3\_Mf\_liverA, CL2108.Contig4\_Mf\_liverA, CL2108.Contig5\_Mf\_liverA, CL2132.Contig1\_Mf\_liverA, CL2132.Contig2\_Mf\_liverA, CL2284.Contig1\_Mf\_liverA, CL2284.Contig2\_Mf\_liverA, CL2301.Contig1\_Mf\_liverA, CL2301.Contig2\_Mf\_liverA, CL2301.Contig3\_Mf\_liverA, CL2301.Contig4\_Mf\_liverA, CL231.Contig1\_Mf\_liverA, CL231.Contig2\_Mf\_liverA, CL2329.Contig1\_Mf\_liverA, CL2329.Contig2\_Mf\_liverA, CL2332.Contig1\_Mf\_liverA, CL2332.Contig2\_Mf\_liverA, CL2342.Contig2\_Mf\_liverA, CL2425.Contig1\_Mf\_liverA, CL2446.Contig2\_Mf\_liverA, CL2450.Contig1\_Mf\_liverA, CL2450.Contig2\_Mf\_liverA, CL2450.Contig3\_Mf\_liverA, CL2487.Contig4\_Mf\_liverA, CL2487.Contig5\_Mf\_liverA, CL2493.Contig2\_Mf\_liverA, CL2516.Contig1\_Mf\_liverA, CL2516.Contig2\_Mf\_liverA, CL2542.Contig1\_Mf\_liverA, CL256.Contig1\_Mf\_liverA, CL256.Contig3\_Mf\_liverA, CL256.Contig5\_Mf\_liverA, CL256.Contig7\_Mf\_liverA, CL2631.Contig1\_Mf\_liverA, CL2631.Contig2\_Mf\_liverA, CL2741.Contig1\_Mf\_liverA, CL2741.Contig2\_Mf\_liverA, CL2764.Contig1\_Mf\_liverA, CL2764.Contig2\_Mf\_liverA, CL2764.Contig3\_Mf\_liverA, CL2764.Contig4\_Mf\_liverA, CL2768.Contig1\_Mf\_liverA, CL2865.Contig2\_Mf\_liverA, CL2871.Contig2\_Mf\_liverA, CL288.Contig1\_Mf\_liverA, CL288.Contig2\_Mf\_liverA, CL2900.Contig1\_Mf\_liverA, CL2900.Contig2\_Mf\_liverA, CL2951.Contig1\_Mf\_liverA, CL2951.Contig2\_Mf\_liverA, CL2953.Contig1\_Mf\_liverA, CL2953.Contig2\_Mf\_liverA, CL2953.Contig3\_Mf\_liverA, CL2953.Contig4\_Mf\_liverA, CL307.Contig12\_Mf\_liverA, CL307.Contig16\_Mf\_liverA, CL307.Contig18\_Mf\_liverA, CL307.Contig19\_Mf\_liverA, CL307.Contig1\_Mf\_liverA, CL307.Contig20\_Mf\_liverA, CL307.Contig21\_Mf\_liverA, CL307.Contig22\_Mf\_liverA, CL307.Contig23\_Mf\_liverA, CL307.Contig24\_Mf\_liverA, CL307.Contig2\_Mf\_liverA, CL307.Contig3\_Mf\_liverA, CL307.Contig4\_Mf\_liverA, CL307.Contig5\_Mf\_liverA, CL307.Contig8\_Mf\_liverA, CL3077.Contig1\_Mf\_liverA, CL312.Contig1\_Mf\_liverA, CL312.Contig2\_Mf\_liverA, CL312.Contig3\_Mf\_liverA, CL3138.Contig1\_Mf\_liverA, CL3138.Contig2\_Mf\_liverA, CL3148.Contig1\_Mf\_liverA, CL3148.Contig2\_Mf\_liverA, CL3173.Contig1\_Mf\_liverA, CL3173.Contig2\_Mf\_liverA, CL3175.Contig1\_Mf\_liverA, CL3175.Contig2\_Mf\_liverA, CL3211.Contig2\_Mf\_liverA, CL3213.Contig2\_Mf\_liverA, CL3217.Contig1\_Mf\_liverA, CL3217.Contig2\_Mf\_liverA, CL3280.Contig2\_Mf\_liverA, CL3297.Contig2\_Mf\_liverA, CL3304.Contig1\_Mf\_liverA, CL3316.Contig1\_Mf\_liverA, CL3447.Contig1\_Mf\_liverA, CL3465.Contig1\_Mf\_liverA, CL3465.Contig2\_Mf\_liverA, CL3466.Contig2\_Mf\_liverA, CL3477.Contig1\_Mf\_liverA, CL3486.Contig1\_Mf\_liverA, CL3486.Contig2\_Mf\_liverA, CL3486.Contig3\_Mf\_liverA, CL3533.Contig1\_Mf\_liverA, CL3581.Contig2\_Mf\_liverA, CL3608.Contig1\_Mf\_liverA, CL3608.Contig2\_Mf\_liverA, CL363.Contig1\_Mf\_liverA, CL363.Contig2\_Mf\_liverA, CL3634.Contig1\_Mf\_liverA, CL3634.Contig2\_Mf\_liverA, CL3645.Contig1\_Mf\_liverA, CL3802.Contig1\_Mf\_liverA, CL3802.Contig2\_Mf\_liverA, CL3818.Contig1\_Mf\_liverA, CL3818.Contig2\_Mf\_liverA, CL3871.Contig1\_Mf\_liverA, CL3871.Contig2\_Mf\_liverA, CL3871.Contig3\_Mf\_liverA, CL3897.Contig1\_Mf\_liverA, CL3897.Contig2\_Mf\_liverA, CL3897.Contig5\_Mf\_liverA, CL3958.Contig1\_Mf\_liverA, CL3958.Contig2\_Mf\_liverA, CL396.Contig7\_Mf\_liverA, CL3975.Contig2\_Mf\_liverA, CL3989.Contig1\_Mf\_liverA, CL3989.Contig2\_Mf\_liverA, CL4102.Contig1\_Mf\_liverA, CL4102.Contig2\_Mf\_liverA, CL4115.Contig1\_Mf\_liverA, CL4123.Contig2\_Mf\_liverA, CL4148.Contig1\_Mf\_liverA, CL4157.Contig1\_Mf\_liverA, CL4157.Contig2\_Mf\_liverA, CL424.Contig1\_Mf\_liverA, CL424.Contig2\_Mf\_liverA, CL424.Contig3\_Mf\_liverA, CL4255.Contig1\_Mf\_liverA, CL4255.Contig2\_Mf\_liverA, CL4281.Contig2\_Mf\_liverA, CL4325.Contig1\_Mf\_liverA, CL4325.Contig2\_Mf\_liverA, CL435.Contig1\_Mf\_liverA, CL435.Contig2\_Mf\_liverA, CL435.Contig3\_Mf\_liverA, CL435.Contig4\_Mf\_liverA, CL435.Contig5\_Mf\_liverA, CL435.Contig6\_Mf\_liverA, CL435.Contig7\_Mf\_liverA, CL4371.Contig1\_Mf\_liverA, CL4371.Contig2\_Mf\_liverA, CL447.Contig33\_Mf\_liverA, CL4518.Contig3\_Mf\_liverA, CL452.Contig4\_Mf\_liverA, CL4548.Contig2\_Mf\_liverA, CL4592.Contig1\_Mf\_liverA, CL4593.Contig3\_Mf\_liverA, CL4598.Contig4\_Mf\_liverA, CL4605.Contig1\_Mf\_liverA, CL4646.Contig2\_Mf\_liverA, CL4647.Contig1\_Mf\_liverA, CL4647.Contig2\_Mf\_liverA, CL4653.Contig1\_Mf\_liverA, CL4729.Contig1\_Mf\_liverA, CL4729.Contig2\_Mf\_liverA, CL4736.Contig1\_Mf\_liverA, CL4793.Contig1\_Mf\_liverA, CL4809.Contig1\_Mf\_liverA, CL4809.Contig2\_Mf\_liverA, CL4899.Contig1\_Mf\_liverA, CL4899.Contig2\_Mf\_liverA, CL5047.Contig1\_Mf\_liverA, CL5093.Contig1\_Mf\_liverA, CL5093.Contig2\_Mf\_liverA, CL5184.Contig1\_Mf\_liverA, CL5198.Contig1\_Mf\_liverA, CL5198.Contig2\_Mf\_liverA, CL5303.Contig3\_Mf\_liverA, CL5329.Contig1\_Mf\_liverA, CL5329.Contig2\_Mf\_liverA, CL5360.Contig1\_Mf\_liverA, CL5360.Contig2\_Mf\_liverA, CL5433.Contig1\_Mf\_liverA, CL5433.Contig2\_Mf\_liverA, CL5503.Contig1\_Mf\_liverA, CL5503.Contig2\_Mf\_liverA, CL5519.Contig1\_Mf\_liverA, CL5519.Contig2\_Mf\_liverA, CL5612.Contig2\_Mf\_liverA, CL5664.Contig1\_Mf\_liverA, CL5664.Contig2\_Mf\_liverA, CL5676.Contig1\_Mf\_liverA, CL5676.Contig2\_Mf\_liverA, CL5789.Contig1\_Mf\_liverA, CL5789.Contig2\_Mf\_liverA, CL5865.Contig1\_Mf\_liverA, CL590.Contig1\_Mf\_liverA, CL590.Contig2\_Mf\_liverA, CL5978.Contig2\_Mf\_liverA, CL5978.Contig3\_Mf\_liverA, CL6037.Contig1\_Mf\_liverA, CL6037.Contig2\_Mf\_liverA, CL6037.Contig3\_Mf\_liverA, CL633.Contig1\_Mf\_liverA, CL638.Contig1\_Mf\_liverA, CL639.Contig3\_Mf\_liverA, CL639.Contig4\_Mf\_liverA, CL708.Contig12\_Mf\_liverA, CL708.Contig13\_Mf\_liverA, CL708.Contig16\_Mf\_liverA, CL708.Contig17\_Mf\_liverA, CL708.Contig1\_Mf\_liverA, CL708.Contig4\_Mf\_liverA, CL708.Contig6\_Mf\_liverA, CL740.Contig1\_Mf\_liverA, CL740.Contig2\_Mf\_liverA, CL741.Contig11\_Mf\_liverA, CL741.Contig13\_Mf\_liverA, CL741.Contig1\_Mf\_liverA, CL741.Contig3\_Mf\_liverA, CL741.Contig4\_Mf\_liverA, CL741.Contig5\_Mf\_liverA, CL741.Contig6\_Mf\_liverA, CL741.Contig9\_Mf\_liverA, CL762.Contig1\_Mf\_liverA, CL762.Contig2\_Mf\_liverA, CL762.Contig3\_Mf\_liverA, CL766.Contig1\_Mf\_liverA, CL766.Contig2\_Mf\_liverA, CL766.Contig3\_Mf\_liverA, CL766.Contig4\_Mf\_liverA, CL771.Contig1\_Mf\_liverA, CL771.Contig2\_Mf\_liverA, CL771.Contig3\_Mf\_liverA, CL771.Contig4\_Mf\_liverA, CL808.Contig1\_Mf\_liverA, CL808.Contig2\_Mf\_liverA, CL808.Contig3\_Mf\_liverA, CL813.Contig1\_Mf\_liverA, CL813.Contig2\_Mf\_liverA, CL82.Contig1\_Mf\_liverA, CL82.Contig2\_Mf\_liverA, CL871.Contig1\_Mf\_liverA, CL894.Contig1\_Mf\_liverA, CL894.Contig2\_Mf\_liverA, CL93.Contig1\_Mf\_liverA, CL93.Contig2\_Mf\_liverA, CL93.Contig3\_Mf\_liverA, CL93.Contig4\_Mf\_liverA, CL986.Contig1\_Mf\_liverA, CL986.Contig2\_Mf\_liverA, Unigene10024\_Mf\_liverA, Unigene10040\_Mf\_liverA, Unigene1007\_Mf\_liverA, Unigene10607\_Mf\_liverA, Unigene114\_Mf\_liverA, Unigene1166\_Mf\_liverA, Unigene124\_Mf\_liverA, Unigene12818\_Mf\_liverA, Unigene12882\_Mf\_liverA, Unigene12889\_Mf\_liverA, Unigene12895\_Mf\_liverA, Unigene12898\_Mf\_liverA, Unigene12906\_Mf\_liverA, Unigene1303\_Mf\_liverA, Unigene13114\_Mf\_liverA, Unigene13275\_Mf\_liverA, Unigene13276\_Mf\_liverA, Unigene13312\_Mf\_liverA, Unigene13358\_Mf\_liverA, Unigene13604\_Mf\_liverA, Unigene13806\_Mf\_liverA, Unigene13807\_Mf\_liverA, Unigene13900\_Mf\_liverA, Unigene14227\_Mf\_liverA, Unigene14270\_Mf\_liverA, Unigene14569\_Mf\_liverA, Unigene14570\_Mf\_liverA, Unigene14581\_Mf\_liverA, Unigene14588\_Mf\_liverA, Unigene14805\_Mf\_liverA, Unigene14826\_Mf\_liverA, Unigene14905\_Mf\_liverA, Unigene14906\_Mf\_liverA, Unigene15138\_Mf\_liverA, Unigene15209\_Mf\_liverA, Unigene15304\_Mf\_liverA, Unigene15433\_Mf\_liverA, Unigene15533\_Mf\_liverA, Unigene15559\_Mf\_liverA, Unigene15580\_Mf\_liverA, Unigene15610\_Mf\_liverA, Unigene15887\_Mf\_liverA, Unigene16096\_Mf\_liverA, Unigene16097\_Mf\_liverA, Unigene16299\_Mf\_liverA, Unigene17036\_Mf\_liverA, Unigene17113\_Mf\_liverA, Unigene17137\_Mf\_liverA, Unigene17138\_Mf\_liverA, Unigene17410\_Mf\_liverA, Unigene18027\_Mf\_liverA, Unigene18328\_Mf\_liverA, Unigene18539\_Mf\_liverA, Unigene18953\_Mf\_liverA, Unigene18\_Mf\_liverA, Unigene19288\_Mf\_liverA, Unigene1939\_Mf\_liverA, Unigene19526\_Mf\_liverA, Unigene19527\_Mf\_liverA, Unigene19795\_Mf\_liverA, Unigene19827\_Mf\_liverA, Unigene20202\_Mf\_liverA, Unigene20365\_Mf\_liverA, Unigene20839\_Mf\_liverA, Unigene20842\_Mf\_liverA, Unigene20861\_Mf\_liverA, Unigene20862\_Mf\_liverA, Unigene21819\_Mf\_liverA, Unigene21820\_Mf\_liverA, Unigene21870\_Mf\_liverA, Unigene21871\_Mf\_liverA, Unigene21872\_Mf\_liverA, Unigene21938\_Mf\_liverA, Unigene2230\_Mf\_liverA, Unigene22611\_Mf\_liverA, Unigene22652\_Mf\_liverA, Unigene22788\_Mf\_liverA, Unigene23265\_Mf\_liverA, Unigene23438\_Mf\_liverA, Unigene23439\_Mf\_liverA, Unigene23462\_Mf\_liverA, Unigene23549\_Mf\_liverA, Unigene23664\_Mf\_liverA, Unigene23943\_Mf\_liverA, Unigene24276\_Mf\_liverA, Unigene24327\_Mf\_liverA, Unigene24328\_Mf\_liverA, Unigene24329\_Mf\_liverA, Unigene24330\_Mf\_liverA, Unigene2441\_Mf\_liverA, Unigene24477\_Mf\_liverA, Unigene24579\_Mf\_liverA, Unigene24628\_Mf\_liverA, Unigene24662\_Mf\_liverA, Unigene24663\_Mf\_liverA, Unigene24732\_Mf\_liverA, Unigene24760\_Mf\_liverA, Unigene25019\_Mf\_liverA, Unigene25094\_Mf\_liverA, Unigene25095\_Mf\_liverA, Unigene25119\_Mf\_liverA, Unigene25181\_Mf\_liverA, Unigene25193\_Mf\_liverA, Unigene25194\_Mf\_liverA, Unigene25195\_Mf\_liverA, Unigene25341\_Mf\_liverA, Unigene25421\_Mf\_liverA, Unigene25553\_Mf\_liverA, Unigene25554\_Mf\_liverA, Unigene25571\_Mf\_liverA, Unigene25630\_Mf\_liverA, Unigene25692\_Mf\_liverA, Unigene25693\_Mf\_liverA, Unigene25694\_Mf\_liverA, Unigene25812\_Mf\_liverA, Unigene25813\_Mf\_liverA, Unigene25826\_Mf\_liverA, Unigene25980\_Mf\_liverA, Unigene26020\_Mf\_liverA, Unigene26021\_Mf\_liverA, Unigene26073\_Mf\_liverA, Unigene26115\_Mf\_liverA, Unigene26199\_Mf\_liverA, Unigene26325\_Mf\_liverA, Unigene26511\_Mf\_liverA, Unigene26512\_Mf\_liverA, Unigene26523\_Mf\_liverA, Unigene26535\_Mf\_liverA, Unigene26683\_Mf\_liverA, Unigene26723\_Mf\_liverA, Unigene26962\_Mf\_liverA, Unigene27103\_Mf\_liverA, Unigene27104\_Mf\_liverA, Unigene27105\_Mf\_liverA, Unigene27259\_Mf\_liverA, Unigene27286\_Mf\_liverA, Unigene27293\_Mf\_liverA, Unigene27751\_Mf\_liverA, Unigene27752\_Mf\_liverA, Unigene27778\_Mf\_liverA, Unigene27779\_Mf\_liverA, Unigene27780\_Mf\_liverA, Unigene28148\_Mf\_liverA, Unigene28149\_Mf\_liverA, Unigene28150\_Mf\_liverA, Unigene28197\_Mf\_liverA, Unigene28214\_Mf\_liverA, Unigene28215\_Mf\_liverA, Unigene28268\_Mf\_liverA, Unigene28290\_Mf\_liverA, Unigene28298\_Mf\_liverA, Unigene28440\_Mf\_liverA, Unigene28693\_Mf\_liverA, Unigene28696\_Mf\_liverA, Unigene28736\_Mf\_liverA, Unigene28806\_Mf\_liverA, Unigene28807\_Mf\_liverA, Unigene28955\_Mf\_liverA, Unigene28983\_Mf\_liverA, Unigene29065\_Mf\_liverA, Unigene29406\_Mf\_liverA, Unigene29407\_Mf\_liverA, Unigene29447\_Mf\_liverA, Unigene29720\_Mf\_liverA, Unigene29818\_Mf\_liverA, Unigene29849\_Mf\_liverA, Unigene29892\_Mf\_liverA, Unigene29981\_Mf\_liverA, Unigene30041\_Mf\_liverA, Unigene30134\_Mf\_liverA, Unigene30164\_Mf\_liverA, Unigene30283\_Mf\_liverA, Unigene30336\_Mf\_liverA, Unigene30355\_Mf\_liverA, Unigene3047\_Mf\_liverA, Unigene30599\_Mf\_liverA, Unigene30600\_Mf\_liverA, Unigene30664\_Mf\_liverA, Unigene30883\_Mf\_liverA, Unigene30928\_Mf\_liverA, Unigene30936\_Mf\_liverA, Unigene31091\_Mf\_liverA, Unigene31145\_Mf\_liverA, Unigene31189\_Mf\_liverA, Unigene31270\_Mf\_liverA, Unigene31303\_Mf\_liverA, Unigene31646\_Mf\_liverA, Unigene31788\_Mf\_liverA, Unigene31840\_Mf\_liverA, Unigene31883\_Mf\_liverA, Unigene31942\_Mf\_liverA, Unigene31943\_Mf\_liverA, Unigene31999\_Mf\_liverA, Unigene32013\_Mf\_liverA, Unigene32029\_Mf\_liverA, Unigene32045\_Mf\_liverA, Unigene32163\_Mf\_liverA, Unigene32165\_Mf\_liverA, Unigene32166\_Mf\_liverA, Unigene32182\_Mf\_liverA, Unigene32186\_Mf\_liverA, Unigene32187\_Mf\_liverA, Unigene32188\_Mf\_liverA, Unigene32192\_Mf\_liverA, Unigene32341\_Mf\_liverA, Unigene32431\_Mf\_liverA, Unigene32558\_Mf\_liverA, Unigene32620\_Mf\_liverA, Unigene32749\_Mf\_liverA, Unigene32942\_Mf\_liverA, Unigene33111\_Mf\_liverA, Unigene33200\_Mf\_liverA, Unigene33274\_Mf\_liverA, Unigene33301\_Mf\_liverA, Unigene33597\_Mf\_liverA, Unigene33727\_Mf\_liverA, Unigene33939\_Mf\_liverA, Unigene34038\_Mf\_liverA, Unigene34052\_Mf\_liverA, Unigene34173\_Mf\_liverA, Unigene34593\_Mf\_liverA, Unigene34594\_Mf\_liverA, Unigene34606\_Mf\_liverA, Unigene34873\_Mf\_liverA, Unigene34874\_Mf\_liverA, Unigene35149\_Mf\_liverA, Unigene35373\_Mf\_liverA, Unigene35374\_Mf\_liverA, Unigene35375\_Mf\_liverA, Unigene35376\_Mf\_liverA, Unigene35377\_Mf\_liverA, Unigene35378\_Mf\_liverA, Unigene35379\_Mf\_liverA, Unigene35380\_Mf\_liverA, Unigene35681\_Mf\_liverA, Unigene35685\_Mf\_liverA, Unigene35686\_Mf\_liverA, Unigene35688\_Mf\_liverA, Unigene35689\_Mf\_liverA, Unigene35822\_Mf\_liverA, Unigene35828\_Mf\_liverA, Unigene36136\_Mf\_liverA, Unigene36275\_Mf\_liverA, Unigene3661\_Mf\_liverA, Unigene36862\_Mf\_liverA, Unigene36869\_Mf\_liverA, Unigene36886\_Mf\_liverA, Unigene36936\_Mf\_liverA, Unigene36976\_Mf\_liverA, Unigene37016\_Mf\_liverA, Unigene37069\_Mf\_liverA, Unigene37098\_Mf\_liverA, Unigene37169\_Mf\_liverA, Unigene37189\_Mf\_liverA, Unigene37357\_Mf\_liverA, Unigene37472\_Mf\_liverA, Unigene37494\_Mf\_liverA, Unigene37504\_Mf\_liverA, Unigene37585\_Mf\_liverA, Unigene3771\_Mf\_liverA, Unigene37778\_Mf\_liverA, Unigene3777\_Mf\_liverA, Unigene37892\_Mf\_liverA, Unigene37908\_Mf\_liverA, Unigene37938\_Mf\_liverA, Unigene38109\_Mf\_liverA, Unigene38310\_Mf\_liverA, Unigene38815\_Mf\_liverA, Unigene39035\_Mf\_liverA, Unigene39145\_Mf\_liverA, Unigene39251\_Mf\_liverA, Unigene39507\_Mf\_liverA, Unigene39527\_Mf\_liverA, Unigene39787\_Mf\_liverA, Unigene40498\_Mf\_liverA, Unigene40576\_Mf\_liverA, Unigene40897\_Mf\_liverA, Unigene40974\_Mf\_liverA, Unigene41613\_Mf\_liverA, Unigene4161\_Mf\_liverA, Unigene43076\_Mf\_liverA, Unigene43579\_Mf\_liverA, Unigene44219\_Mf\_liverA, Unigene442\_Mf\_liverA, Unigene44820\_Mf\_liverA, Unigene4561\_Mf\_liverA, Unigene4611\_Mf\_liverA, Unigene46821\_Mf\_liverA, Unigene4712\_Mf\_liverA, Unigene4730\_Mf\_liverA, Unigene47634\_Mf\_liverA, Unigene47768\_Mf\_liverA, Unigene49299\_Mf\_liverA, Unigene4929\_Mf\_liverA, Unigene4930\_Mf\_liverA, Unigene5044\_Mf\_liverA, Unigene5048\_Mf\_liverA, Unigene5049\_Mf\_liverA, Unigene51110\_Mf\_liverA, Unigene5134\_Mf\_liverA, Unigene5140\_Mf\_liverA, Unigene5145\_Mf\_liverA, Unigene5167\_Mf\_liverA, Unigene5198\_Mf\_liverA, Unigene5219\_Mf\_liverA, Unigene534\_Mf\_liverA, Unigene5395\_Mf\_liverA, Unigene5396\_Mf\_liverA, Unigene5574\_Mf\_liverA, Unigene5705\_Mf\_liverA, Unigene5729\_Mf\_liverA, Unigene5744\_Mf\_liverA, Unigene5766\_Mf\_liverA, Unigene587\_Mf\_liverA, Unigene5898\_Mf\_liverA, Unigene5900\_Mf\_liverA, Unigene5904\_Mf\_liverA, Unigene6035\_Mf\_liverA, Unigene6121\_Mf\_liverA, Unigene7380\_Mf\_liverA, Unigene7408\_Mf\_liverA, Unigene7603\_Mf\_liverA, Unigene8032\_Mf\_liverA, Unigene8066\_Mf\_liverA, Unigene8196\_Mf\_liverA, Unigene8197\_Mf\_liverA, Unigene8207\_Mf\_liverA, Unigene841\_Mf\_liverA, Unigene8653\_Mf\_liverA, Unigene8877\_Mf\_liverA, Unigene8969\_Mf\_liverA, Unigene9240\_Mf\_liverA, Unigene938\_Mf\_liverA, Unigene9436\_Mf\_liverA, Unigene95\_Mf\_liverA, Unigene9664\_Mf\_liverA |
| 7 | HTLV-I infection | CL1017.Contig1\_Mf\_liverA, CL1017.Contig2\_Mf\_liverA, CL1020.Contig1\_Mf\_liverA, CL1020.Contig2\_Mf\_liverA, CL1114.Contig1\_Mf\_liverA, CL1114.Contig2\_Mf\_liverA, CL120.Contig1\_Mf\_liverA, CL120.Contig2\_Mf\_liverA, CL120.Contig3\_Mf\_liverA, CL120.Contig4\_Mf\_liverA, CL1291.Contig1\_Mf\_liverA, CL1291.Contig2\_Mf\_liverA, CL1336.Contig1\_Mf\_liverA, CL1336.Contig2\_Mf\_liverA, CL1336.Contig3\_Mf\_liverA, CL1362.Contig1\_Mf\_liverA, CL1362.Contig2\_Mf\_liverA, CL1371.Contig1\_Mf\_liverA, CL1371.Contig2\_Mf\_liverA, CL1371.Contig3\_Mf\_liverA, CL1371.Contig4\_Mf\_liverA, CL1447.Contig1\_Mf\_liverA, CL1447.Contig2\_Mf\_liverA, CL1447.Contig3\_Mf\_liverA, CL1447.Contig4\_Mf\_liverA, CL1475.Contig1\_Mf\_liverA, CL1475.Contig2\_Mf\_liverA, CL1551.Contig1\_Mf\_liverA, CL1551.Contig2\_Mf\_liverA, CL1659.Contig1\_Mf\_liverA, CL1659.Contig2\_Mf\_liverA, CL1687.Contig1\_Mf\_liverA, CL1687.Contig2\_Mf\_liverA, CL1690.Contig1\_Mf\_liverA, CL1690.Contig2\_Mf\_liverA, CL1690.Contig3\_Mf\_liverA, CL1690.Contig4\_Mf\_liverA, CL1695.Contig1\_Mf\_liverA, CL1695.Contig2\_Mf\_liverA, CL1695.Contig3\_Mf\_liverA, CL1695.Contig4\_Mf\_liverA, CL1695.Contig5\_Mf\_liverA, CL1695.Contig6\_Mf\_liverA, CL1730.Contig1\_Mf\_liverA, CL1730.Contig2\_Mf\_liverA, CL1805.Contig1\_Mf\_liverA, CL1805.Contig2\_Mf\_liverA, CL1815.Contig1\_Mf\_liverA, CL1815.Contig2\_Mf\_liverA, CL1815.Contig3\_Mf\_liverA, CL1818.Contig1\_Mf\_liverA, CL1818.Contig2\_Mf\_liverA, CL1840.Contig1\_Mf\_liverA, CL1898.Contig1\_Mf\_liverA, CL1898.Contig2\_Mf\_liverA, CL1898.Contig3\_Mf\_liverA, CL1902.Contig1\_Mf\_liverA, CL1902.Contig2\_Mf\_liverA, CL1902.Contig3\_Mf\_liverA, CL193.Contig1\_Mf\_liverA, CL193.Contig2\_Mf\_liverA, CL1940.Contig1\_Mf\_liverA, CL1940.Contig2\_Mf\_liverA, CL1940.Contig3\_Mf\_liverA, CL1940.Contig4\_Mf\_liverA, CL1940.Contig5\_Mf\_liverA, CL1940.Contig6\_Mf\_liverA, CL1955.Contig1\_Mf\_liverA, CL1955.Contig2\_Mf\_liverA, CL2033.Contig3\_Mf\_liverA, CL2033.Contig4\_Mf\_liverA, CL2117.Contig1\_Mf\_liverA, CL2117.Contig2\_Mf\_liverA, CL215.Contig1\_Mf\_liverA, CL215.Contig2\_Mf\_liverA, CL232.Contig1\_Mf\_liverA, CL232.Contig2\_Mf\_liverA, CL232.Contig3\_Mf\_liverA, CL232.Contig4\_Mf\_liverA, CL2393.Contig1\_Mf\_liverA, CL2393.Contig2\_Mf\_liverA, CL2393.Contig3\_Mf\_liverA, CL2393.Contig4\_Mf\_liverA, CL2520.Contig1\_Mf\_liverA, CL2520.Contig2\_Mf\_liverA, CL2548.Contig1\_Mf\_liverA, CL2548.Contig2\_Mf\_liverA, CL2557.Contig1\_Mf\_liverA, CL2557.Contig2\_Mf\_liverA, CL2599.Contig1\_Mf\_liverA, CL2599.Contig2\_Mf\_liverA, CL2599.Contig3\_Mf\_liverA, CL275.Contig1\_Mf\_liverA, CL275.Contig2\_Mf\_liverA, CL275.Contig3\_Mf\_liverA, CL275.Contig4\_Mf\_liverA, CL275.Contig5\_Mf\_liverA, CL2750.Contig1\_Mf\_liverA, CL2750.Contig2\_Mf\_liverA, CL2770.Contig1\_Mf\_liverA, CL2770.Contig2\_Mf\_liverA, CL2778.Contig1\_Mf\_liverA, CL2778.Contig2\_Mf\_liverA, CL2954.Contig1\_Mf\_liverA, CL2954.Contig2\_Mf\_liverA, CL2954.Contig3\_Mf\_liverA, CL296.Contig1\_Mf\_liverA, CL296.Contig2\_Mf\_liverA, CL2969.Contig1\_Mf\_liverA, CL2969.Contig2\_Mf\_liverA, CL2993.Contig1\_Mf\_liverA, CL2993.Contig2\_Mf\_liverA, CL2999.Contig1\_Mf\_liverA, CL2999.Contig2\_Mf\_liverA, CL3002.Contig1\_Mf\_liverA, CL3007.Contig1\_Mf\_liverA, CL3053.Contig1\_Mf\_liverA, CL3063.Contig1\_Mf\_liverA, CL3147.Contig1\_Mf\_liverA, CL3211.Contig2\_Mf\_liverA, CL3243.Contig1\_Mf\_liverA, CL3243.Contig2\_Mf\_liverA, CL3429.Contig1\_Mf\_liverA, CL3429.Contig2\_Mf\_liverA, CL3631.Contig1\_Mf\_liverA, CL3631.Contig2\_Mf\_liverA, CL3632.Contig1\_Mf\_liverA, CL3749.Contig1\_Mf\_liverA, CL3749.Contig2\_Mf\_liverA, CL3800.Contig1\_Mf\_liverA, CL3800.Contig2\_Mf\_liverA, CL3835.Contig1\_Mf\_liverA, CL3835.Contig2\_Mf\_liverA, CL3916.Contig1\_Mf\_liverA, CL3916.Contig2\_Mf\_liverA, CL3934.Contig1\_Mf\_liverA, CL4002.Contig1\_Mf\_liverA, CL4002.Contig2\_Mf\_liverA, CL4227.Contig1\_Mf\_liverA, CL4227.Contig2\_Mf\_liverA, CL4227.Contig3\_Mf\_liverA, CL4280.Contig1\_Mf\_liverA, CL4280.Contig2\_Mf\_liverA, CL4281.Contig2\_Mf\_liverA, CL433.Contig2\_Mf\_liverA, CL4442.Contig1\_Mf\_liverA, CL4442.Contig2\_Mf\_liverA, CL4558.Contig1\_Mf\_liverA, CL4558.Contig2\_Mf\_liverA, CL459.Contig1\_Mf\_liverA, CL459.Contig2\_Mf\_liverA, CL4597.Contig1\_Mf\_liverA, CL4597.Contig2\_Mf\_liverA, CL4653.Contig1\_Mf\_liverA, CL4669.Contig1\_Mf\_liverA, CL4678.Contig1\_Mf\_liverA, CL4722.Contig1\_Mf\_liverA, CL4722.Contig2\_Mf\_liverA, CL4745.Contig1\_Mf\_liverA, CL4757.Contig1\_Mf\_liverA, CL4757.Contig2\_Mf\_liverA, CL4784.Contig2\_Mf\_liverA, CL4792.Contig1\_Mf\_liverA, CL4792.Contig2\_Mf\_liverA, CL4846.Contig1\_Mf\_liverA, CL4846.Contig2\_Mf\_liverA, CL4901.Contig1\_Mf\_liverA, CL4901.Contig2\_Mf\_liverA, CL4919.Contig1\_Mf\_liverA, CL4919.Contig2\_Mf\_liverA, CL4935.Contig1\_Mf\_liverA, CL4935.Contig2\_Mf\_liverA, CL5018.Contig1\_Mf\_liverA, CL5018.Contig2\_Mf\_liverA, CL5132.Contig2\_Mf\_liverA, CL5148.Contig1\_Mf\_liverA, CL5148.Contig2\_Mf\_liverA, CL5287.Contig1\_Mf\_liverA, CL5287.Contig2\_Mf\_liverA, CL5329.Contig2\_Mf\_liverA, CL5337.Contig1\_Mf\_liverA, CL5337.Contig2\_Mf\_liverA, CL5384.Contig1\_Mf\_liverA, CL5384.Contig2\_Mf\_liverA, CL5415.Contig1\_Mf\_liverA, CL5415.Contig2\_Mf\_liverA, CL5416.Contig1\_Mf\_liverA, CL5416.Contig2\_Mf\_liverA, CL5430.Contig1\_Mf\_liverA, CL5430.Contig2\_Mf\_liverA, CL5478.Contig1\_Mf\_liverA, CL5478.Contig2\_Mf\_liverA, CL5491.Contig1\_Mf\_liverA, CL5491.Contig2\_Mf\_liverA, CL5501.Contig1\_Mf\_liverA, CL5501.Contig2\_Mf\_liverA, CL5576.Contig1\_Mf\_liverA, CL5595.Contig1\_Mf\_liverA, CL5595.Contig2\_Mf\_liverA, CL5614.Contig1\_Mf\_liverA, CL5614.Contig2\_Mf\_liverA, CL5651.Contig1\_Mf\_liverA, CL5651.Contig2\_Mf\_liverA, CL5749.Contig1\_Mf\_liverA, CL5749.Contig2\_Mf\_liverA, CL5766.Contig1\_Mf\_liverA, CL5766.Contig2\_Mf\_liverA, CL582.Contig1\_Mf\_liverA, CL5896.Contig1\_Mf\_liverA, CL5896.Contig2\_Mf\_liverA, CL6034.Contig1\_Mf\_liverA, CL6034.Contig2\_Mf\_liverA, CL63.Contig1\_Mf\_liverA, CL634.Contig1\_Mf\_liverA, CL634.Contig2\_Mf\_liverA, CL683.Contig1\_Mf\_liverA, CL683.Contig2\_Mf\_liverA, CL683.Contig3\_Mf\_liverA, CL683.Contig4\_Mf\_liverA, CL697.Contig1\_Mf\_liverA, CL703.Contig1\_Mf\_liverA, CL870.Contig1\_Mf\_liverA, CL870.Contig2\_Mf\_liverA, CL873.Contig1\_Mf\_liverA, CL873.Contig2\_Mf\_liverA, CL890.Contig1\_Mf\_liverA, CL890.Contig2\_Mf\_liverA, CL892.Contig1\_Mf\_liverA, CL892.Contig2\_Mf\_liverA, CL892.Contig3\_Mf\_liverA, CL907.Contig1\_Mf\_liverA, CL907.Contig2\_Mf\_liverA, CL907.Contig3\_Mf\_liverA, CL907.Contig4\_Mf\_liverA, CL932.Contig5\_Mf\_liverA, CL932.Contig6\_Mf\_liverA, CL932.Contig9\_Mf\_liverA, CL951.Contig1\_Mf\_liverA, CL951.Contig2\_Mf\_liverA, CL99.Contig1\_Mf\_liverA, CL99.Contig2\_Mf\_liverA, Unigene10122\_Mf\_liverA, Unigene10249\_Mf\_liverA, Unigene10273\_Mf\_liverA, Unigene10282\_Mf\_liverA, Unigene10335\_Mf\_liverA, Unigene10780\_Mf\_liverA, Unigene11092\_Mf\_liverA, Unigene11782\_Mf\_liverA, Unigene12256\_Mf\_liverA, Unigene12525\_Mf\_liverA, Unigene1309\_Mf\_liverA, Unigene13170\_Mf\_liverA, Unigene13171\_Mf\_liverA, Unigene13265\_Mf\_liverA, Unigene13379\_Mf\_liverA, Unigene13408\_Mf\_liverA, Unigene13418\_Mf\_liverA, Unigene13533\_Mf\_liverA, Unigene13586\_Mf\_liverA, Unigene13695\_Mf\_liverA, Unigene13919\_Mf\_liverA, Unigene13981\_Mf\_liverA, Unigene13988\_Mf\_liverA, Unigene14035\_Mf\_liverA, Unigene14094\_Mf\_liverA, Unigene14284\_Mf\_liverA, Unigene1438\_Mf\_liverA, Unigene1463\_Mf\_liverA, Unigene14695\_Mf\_liverA, Unigene14807\_Mf\_liverA, Unigene1489\_Mf\_liverA, Unigene14907\_Mf\_liverA, Unigene15295\_Mf\_liverA, Unigene15296\_Mf\_liverA, Unigene152\_Mf\_liverA, Unigene15401\_Mf\_liverA, Unigene15463\_Mf\_liverA, Unigene15464\_Mf\_liverA, Unigene15714\_Mf\_liverA, Unigene15715\_Mf\_liverA, Unigene15730\_Mf\_liverA, Unigene15982\_Mf\_liverA, Unigene16048\_Mf\_liverA, Unigene16321\_Mf\_liverA, Unigene16547\_Mf\_liverA, Unigene16548\_Mf\_liverA, Unigene16819\_Mf\_liverA, Unigene16820\_Mf\_liverA, Unigene17260\_Mf\_liverA, Unigene17261\_Mf\_liverA, Unigene17298\_Mf\_liverA, Unigene17317\_Mf\_liverA, Unigene17518\_Mf\_liverA, Unigene17657\_Mf\_liverA, Unigene17982\_Mf\_liverA, Unigene18167\_Mf\_liverA, Unigene18168\_Mf\_liverA, Unigene18177\_Mf\_liverA, Unigene18385\_Mf\_liverA, Unigene18722\_Mf\_liverA, Unigene18723\_Mf\_liverA, Unigene18735\_Mf\_liverA, Unigene19041\_Mf\_liverA, Unigene19079\_Mf\_liverA, Unigene19080\_Mf\_liverA, Unigene19116\_Mf\_liverA, Unigene19145\_Mf\_liverA, Unigene19260\_Mf\_liverA, Unigene19261\_Mf\_liverA, Unigene19742\_Mf\_liverA, Unigene19795\_Mf\_liverA, Unigene19885\_Mf\_liverA, Unigene19893\_Mf\_liverA, Unigene19894\_Mf\_liverA, Unigene19985\_Mf\_liverA, Unigene20022\_Mf\_liverA, Unigene20149\_Mf\_liverA, Unigene2070\_Mf\_liverA, Unigene20842\_Mf\_liverA, Unigene20855\_Mf\_liverA, Unigene2086\_Mf\_liverA, Unigene20896\_Mf\_liverA, Unigene21163\_Mf\_liverA, Unigene21193\_Mf\_liverA, Unigene21284\_Mf\_liverA, Unigene21416\_Mf\_liverA, Unigene21464\_Mf\_liverA, Unigene21710\_Mf\_liverA, Unigene21711\_Mf\_liverA, Unigene21712\_Mf\_liverA, Unigene21713\_Mf\_liverA, Unigene22381\_Mf\_liverA, Unigene22432\_Mf\_liverA, Unigene22433\_Mf\_liverA, Unigene22859\_Mf\_liverA, Unigene22860\_Mf\_liverA, Unigene22919\_Mf\_liverA, Unigene23266\_Mf\_liverA, Unigene23341\_Mf\_liverA, Unigene23668\_Mf\_liverA, Unigene23669\_Mf\_liverA, Unigene23670\_Mf\_liverA, Unigene24065\_Mf\_liverA, Unigene24111\_Mf\_liverA, Unigene24112\_Mf\_liverA, Unigene24160\_Mf\_liverA, Unigene24281\_Mf\_liverA, Unigene24294\_Mf\_liverA, Unigene24334\_Mf\_liverA, Unigene24527\_Mf\_liverA, Unigene24528\_Mf\_liverA, Unigene2455\_Mf\_liverA, Unigene24785\_Mf\_liverA, Unigene24801\_Mf\_liverA, Unigene24804\_Mf\_liverA, Unigene24832\_Mf\_liverA, Unigene24833\_Mf\_liverA, Unigene25070\_Mf\_liverA, Unigene2521\_Mf\_liverA, Unigene25454\_Mf\_liverA, Unigene25504\_Mf\_liverA, Unigene25506\_Mf\_liverA, Unigene25541\_Mf\_liverA, Unigene25542\_Mf\_liverA, Unigene25586\_Mf\_liverA, Unigene25753\_Mf\_liverA, Unigene25792\_Mf\_liverA, Unigene25852\_Mf\_liverA, Unigene25887\_Mf\_liverA, Unigene26098\_Mf\_liverA, Unigene26099\_Mf\_liverA, Unigene26106\_Mf\_liverA, Unigene26117\_Mf\_liverA, Unigene26305\_Mf\_liverA, Unigene26306\_Mf\_liverA, Unigene26307\_Mf\_liverA, Unigene26413\_Mf\_liverA, Unigene26422\_Mf\_liverA, Unigene26423\_Mf\_liverA, Unigene26479\_Mf\_liverA, Unigene26480\_Mf\_liverA, Unigene26481\_Mf\_liverA, Unigene26573\_Mf\_liverA, Unigene26574\_Mf\_liverA, Unigene26664\_Mf\_liverA, Unigene26665\_Mf\_liverA, Unigene27181\_Mf\_liverA, Unigene2721\_Mf\_liverA, Unigene27478\_Mf\_liverA, Unigene27553\_Mf\_liverA, Unigene27571\_Mf\_liverA, Unigene27593\_Mf\_liverA, Unigene27594\_Mf\_liverA, Unigene2798\_Mf\_liverA, Unigene28104\_Mf\_liverA, Unigene28144\_Mf\_liverA, Unigene28145\_Mf\_liverA, Unigene28244\_Mf\_liverA, Unigene28245\_Mf\_liverA, Unigene28250\_Mf\_liverA, Unigene28413\_Mf\_liverA, Unigene28437\_Mf\_liverA, Unigene2854\_Mf\_liverA, Unigene28617\_Mf\_liverA, Unigene28736\_Mf\_liverA, Unigene28802\_Mf\_liverA, Unigene28893\_Mf\_liverA, Unigene28955\_Mf\_liverA, Unigene28989\_Mf\_liverA, Unigene29018\_Mf\_liverA, Unigene29129\_Mf\_liverA, Unigene29231\_Mf\_liverA, Unigene2929\_Mf\_liverA, Unigene2930\_Mf\_liverA, Unigene29399\_Mf\_liverA, Unigene29636\_Mf\_liverA, Unigene29637\_Mf\_liverA, Unigene29725\_Mf\_liverA, Unigene29829\_Mf\_liverA, Unigene29831\_Mf\_liverA, Unigene29921\_Mf\_liverA, Unigene30009\_Mf\_liverA, Unigene30010\_Mf\_liverA, Unigene30411\_Mf\_liverA, Unigene30412\_Mf\_liverA, Unigene30539\_Mf\_liverA, Unigene30540\_Mf\_liverA, Unigene30541\_Mf\_liverA, Unigene30616\_Mf\_liverA, Unigene30618\_Mf\_liverA, Unigene30619\_Mf\_liverA, Unigene30621\_Mf\_liverA, Unigene30680\_Mf\_liverA, Unigene30878\_Mf\_liverA, Unigene30879\_Mf\_liverA, Unigene30907\_Mf\_liverA, Unigene30908\_Mf\_liverA, Unigene30909\_Mf\_liverA, Unigene30981\_Mf\_liverA, Unigene31129\_Mf\_liverA, Unigene31196\_Mf\_liverA, Unigene31304\_Mf\_liverA, Unigene31305\_Mf\_liverA, Unigene31392\_Mf\_liverA, Unigene31521\_Mf\_liverA, Unigene31592\_Mf\_liverA, Unigene31593\_Mf\_liverA, Unigene31594\_Mf\_liverA, Unigene31595\_Mf\_liverA, Unigene31597\_Mf\_liverA, Unigene31599\_Mf\_liverA, Unigene31687\_Mf\_liverA, Unigene31688\_Mf\_liverA, Unigene31691\_Mf\_liverA, Unigene31692\_Mf\_liverA, Unigene31708\_Mf\_liverA, Unigene3170\_Mf\_liverA, Unigene31768\_Mf\_liverA, Unigene31769\_Mf\_liverA, Unigene31796\_Mf\_liverA, Unigene31797\_Mf\_liverA, Unigene31822\_Mf\_liverA, Unigene31862\_Mf\_liverA, Unigene31930\_Mf\_liverA, Unigene31933\_Mf\_liverA, Unigene31934\_Mf\_liverA, Unigene31935\_Mf\_liverA, Unigene31936\_Mf\_liverA, Unigene32002\_Mf\_liverA, Unigene32003\_Mf\_liverA, Unigene32090\_Mf\_liverA, Unigene32234\_Mf\_liverA, Unigene32235\_Mf\_liverA, Unigene32239\_Mf\_liverA, Unigene32361\_Mf\_liverA, Unigene32362\_Mf\_liverA, Unigene32425\_Mf\_liverA, Unigene32484\_Mf\_liverA, Unigene32650\_Mf\_liverA, Unigene32810\_Mf\_liverA, Unigene32879\_Mf\_liverA, Unigene32882\_Mf\_liverA, Unigene32883\_Mf\_liverA, Unigene32924\_Mf\_liverA, Unigene33122\_Mf\_liverA, Unigene33138\_Mf\_liverA, Unigene33455\_Mf\_liverA, Unigene33512\_Mf\_liverA, Unigene33513\_Mf\_liverA, Unigene33855\_Mf\_liverA, Unigene33856\_Mf\_liverA, Unigene33857\_Mf\_liverA, Unigene33965\_Mf\_liverA, Unigene33970\_Mf\_liverA, Unigene33971\_Mf\_liverA, Unigene33972\_Mf\_liverA, Unigene34084\_Mf\_liverA, Unigene34176\_Mf\_liverA, Unigene34711\_Mf\_liverA, Unigene34746\_Mf\_liverA, Unigene3478\_Mf\_liverA, Unigene34975\_Mf\_liverA, Unigene35418\_Mf\_liverA, Unigene35459\_Mf\_liverA, Unigene35612\_Mf\_liverA, Unigene36539\_Mf\_liverA, Unigene36543\_Mf\_liverA, Unigene36648\_Mf\_liverA, Unigene36799\_Mf\_liverA, Unigene36849\_Mf\_liverA, Unigene36856\_Mf\_liverA, Unigene36926\_Mf\_liverA, Unigene37037\_Mf\_liverA, Unigene37132\_Mf\_liverA, Unigene37245\_Mf\_liverA, Unigene37255\_Mf\_liverA, Unigene37296\_Mf\_liverA, Unigene37358\_Mf\_liverA, Unigene37492\_Mf\_liverA, Unigene38085\_Mf\_liverA, Unigene38616\_Mf\_liverA, Unigene38681\_Mf\_liverA, Unigene38919\_Mf\_liverA, Unigene3908\_Mf\_liverA, Unigene39137\_Mf\_liverA, Unigene39200\_Mf\_liverA, Unigene39212\_Mf\_liverA, Unigene39266\_Mf\_liverA, Unigene39380\_Mf\_liverA, Unigene39632\_Mf\_liverA, Unigene39640\_Mf\_liverA, Unigene39666\_Mf\_liverA, Unigene39749\_Mf\_liverA, Unigene40101\_Mf\_liverA, Unigene40103\_Mf\_liverA, Unigene40126\_Mf\_liverA, Unigene40507\_Mf\_liverA, Unigene40557\_Mf\_liverA, Unigene40644\_Mf\_liverA, Unigene41280\_Mf\_liverA, Unigene41421\_Mf\_liverA, Unigene41494\_Mf\_liverA, Unigene41527\_Mf\_liverA, Unigene41703\_Mf\_liverA, Unigene41852\_Mf\_liverA, Unigene41894\_Mf\_liverA, Unigene42335\_Mf\_liverA, Unigene42819\_Mf\_liverA, Unigene43517\_Mf\_liverA, Unigene43640\_Mf\_liverA, Unigene43966\_Mf\_liverA, Unigene4459\_Mf\_liverA, Unigene44701\_Mf\_liverA, Unigene44808\_Mf\_liverA, Unigene45329\_Mf\_liverA, Unigene45413\_Mf\_liverA, Unigene45485\_Mf\_liverA, Unigene45643\_Mf\_liverA, Unigene45708\_Mf\_liverA, Unigene45981\_Mf\_liverA, Unigene4616\_Mf\_liverA, Unigene46474\_Mf\_liverA, Unigene46619\_Mf\_liverA, Unigene4681\_Mf\_liverA, Unigene47448\_Mf\_liverA, Unigene4781\_Mf\_liverA, Unigene47837\_Mf\_liverA, Unigene48460\_Mf\_liverA, Unigene48539\_Mf\_liverA, Unigene48645\_Mf\_liverA, Unigene48792\_Mf\_liverA, Unigene48880\_Mf\_liverA, Unigene48890\_Mf\_liverA, Unigene488\_Mf\_liverA, Unigene4893\_Mf\_liverA, Unigene49208\_Mf\_liverA, Unigene4938\_Mf\_liverA, Unigene50319\_Mf\_liverA, Unigene50360\_Mf\_liverA, Unigene50600\_Mf\_liverA, Unigene50718\_Mf\_liverA, Unigene51063\_Mf\_liverA, Unigene51348\_Mf\_liverA, Unigene5142\_Mf\_liverA, Unigene51487\_Mf\_liverA, Unigene51614\_Mf\_liverA, Unigene5190\_Mf\_liverA, Unigene51985\_Mf\_liverA, Unigene5226\_Mf\_liverA, Unigene534\_Mf\_liverA, Unigene5453\_Mf\_liverA, Unigene5484\_Mf\_liverA, Unigene550\_Mf\_liverA, Unigene5540\_Mf\_liverA, Unigene5818\_Mf\_liverA, Unigene5825\_Mf\_liverA, Unigene583\_Mf\_liverA, Unigene5983\_Mf\_liverA, Unigene5984\_Mf\_liverA, Unigene6560\_Mf\_liverA, Unigene6561\_Mf\_liverA, Unigene6710\_Mf\_liverA, Unigene6711\_Mf\_liverA, Unigene6922\_Mf\_liverA, Unigene7108\_Mf\_liverA, Unigene7298\_Mf\_liverA, Unigene7350\_Mf\_liverA, Unigene7351\_Mf\_liverA, Unigene7582\_Mf\_liverA, Unigene7589\_Mf\_liverA, Unigene7729\_Mf\_liverA, Unigene7730\_Mf\_liverA, Unigene774\_Mf\_liverA, Unigene8077\_Mf\_liverA, Unigene8109\_Mf\_liverA, Unigene8277\_Mf\_liverA, Unigene8554\_Mf\_liverA, Unigene8604\_Mf\_liverA, Unigene8624\_Mf\_liverA, Unigene8831\_Mf\_liverA, Unigene897\_Mf\_liverA, Unigene898\_Mf\_liverA, Unigene9466\_Mf\_liverA, Unigene9762\_Mf\_liverA, Unigene994\_Mf\_liverA, Unigene995\_Mf\_liverA |
| 8 | MAPK signaling pathway | CL1017.Contig1\_Mf\_liverA, CL1017.Contig2\_Mf\_liverA, CL1018.Contig1\_Mf\_liverA, CL1018.Contig2\_Mf\_liverA, CL1018.Contig3\_Mf\_liverA, CL1101.Contig1\_Mf\_liverA, CL1101.Contig2\_Mf\_liverA, CL1101.Contig3\_Mf\_liverA, CL1101.Contig4\_Mf\_liverA, CL1101.Contig5\_Mf\_liverA, CL1207.Contig1\_Mf\_liverA, CL1207.Contig2\_Mf\_liverA, CL1253.Contig1\_Mf\_liverA, CL1253.Contig2\_Mf\_liverA, CL1265.Contig1\_Mf\_liverA, CL1362.Contig1\_Mf\_liverA, CL1362.Contig2\_Mf\_liverA, CL1373.Contig1\_Mf\_liverA, CL1437.Contig1\_Mf\_liverA, CL1437.Contig2\_Mf\_liverA, CL1553.Contig1\_Mf\_liverA, CL1553.Contig2\_Mf\_liverA, CL1553.Contig3\_Mf\_liverA, CL1553.Contig4\_Mf\_liverA, CL1553.Contig5\_Mf\_liverA, CL1575.Contig1\_Mf\_liverA, CL1575.Contig2\_Mf\_liverA, CL1687.Contig1\_Mf\_liverA, CL1687.Contig2\_Mf\_liverA, CL1784.Contig1\_Mf\_liverA, CL1784.Contig2\_Mf\_liverA, CL1797.Contig1\_Mf\_liverA, CL1797.Contig2\_Mf\_liverA, CL1805.Contig1\_Mf\_liverA, CL1805.Contig2\_Mf\_liverA, CL1844.Contig1\_Mf\_liverA, CL1844.Contig2\_Mf\_liverA, CL1844.Contig4\_Mf\_liverA, CL1866.Contig1\_Mf\_liverA, CL1866.Contig2\_Mf\_liverA, CL1902.Contig1\_Mf\_liverA, CL1902.Contig2\_Mf\_liverA, CL1902.Contig3\_Mf\_liverA, CL193.Contig1\_Mf\_liverA, CL193.Contig2\_Mf\_liverA, CL1998.Contig1\_Mf\_liverA, CL2004.Contig1\_Mf\_liverA, CL2004.Contig2\_Mf\_liverA, CL2163.Contig1\_Mf\_liverA, CL2163.Contig2\_Mf\_liverA, CL2176.Contig1\_Mf\_liverA, CL2176.Contig2\_Mf\_liverA, CL2191.Contig1\_Mf\_liverA, CL2191.Contig2\_Mf\_liverA, CL2191.Contig3\_Mf\_liverA, CL2213.Contig1\_Mf\_liverA, CL2213.Contig2\_Mf\_liverA, CL232.Contig1\_Mf\_liverA, CL232.Contig2\_Mf\_liverA, CL232.Contig3\_Mf\_liverA, CL232.Contig4\_Mf\_liverA, CL2334.Contig1\_Mf\_liverA, CL2334.Contig2\_Mf\_liverA, CL2334.Contig3\_Mf\_liverA, CL2334.Contig4\_Mf\_liverA, CL2336.Contig1\_Mf\_liverA, CL2336.Contig2\_Mf\_liverA, CL2336.Contig3\_Mf\_liverA, CL2363.Contig1\_Mf\_liverA, CL2363.Contig2\_Mf\_liverA, CL2371.Contig1\_Mf\_liverA, CL2405.Contig1\_Mf\_liverA, CL2405.Contig2\_Mf\_liverA, CL2431.Contig1\_Mf\_liverA, CL2431.Contig2\_Mf\_liverA, CL2431.Contig3\_Mf\_liverA, CL2448.Contig1\_Mf\_liverA, CL2448.Contig2\_Mf\_liverA, CL2448.Contig3\_Mf\_liverA, CL2483.Contig1\_Mf\_liverA, CL2483.Contig2\_Mf\_liverA, CL2546.Contig1\_Mf\_liverA, CL2546.Contig2\_Mf\_liverA, CL2548.Contig1\_Mf\_liverA, CL2548.Contig2\_Mf\_liverA, CL2608.Contig2\_Mf\_liverA, CL2628.Contig1\_Mf\_liverA, CL2628.Contig2\_Mf\_liverA, CL2628.Contig3\_Mf\_liverA, CL2628.Contig4\_Mf\_liverA, CL2628.Contig5\_Mf\_liverA, CL2628.Contig6\_Mf\_liverA, CL2688.Contig1\_Mf\_liverA, CL2688.Contig2\_Mf\_liverA, CL2688.Contig3\_Mf\_liverA, CL2853.Contig1\_Mf\_liverA, CL2853.Contig2\_Mf\_liverA, CL2853.Contig3\_Mf\_liverA, CL2869.Contig1\_Mf\_liverA, CL2869.Contig2\_Mf\_liverA, CL2869.Contig3\_Mf\_liverA, CL2869.Contig4\_Mf\_liverA, CL2876.Contig1\_Mf\_liverA, CL2954.Contig1\_Mf\_liverA, CL2954.Contig2\_Mf\_liverA, CL2954.Contig3\_Mf\_liverA, CL296.Contig1\_Mf\_liverA, CL296.Contig2\_Mf\_liverA, CL2993.Contig1\_Mf\_liverA, CL2993.Contig2\_Mf\_liverA, CL2996.Contig10\_Mf\_liverA, CL2996.Contig11\_Mf\_liverA, CL2996.Contig1\_Mf\_liverA, CL2996.Contig2\_Mf\_liverA, CL2996.Contig3\_Mf\_liverA, CL2996.Contig4\_Mf\_liverA, CL2996.Contig5\_Mf\_liverA, CL2996.Contig6\_Mf\_liverA, CL2996.Contig7\_Mf\_liverA, CL2996.Contig8\_Mf\_liverA, CL2996.Contig9\_Mf\_liverA, CL301.Contig10\_Mf\_liverA, CL301.Contig11\_Mf\_liverA, CL301.Contig12\_Mf\_liverA, CL301.Contig1\_Mf\_liverA, CL301.Contig2\_Mf\_liverA, CL301.Contig3\_Mf\_liverA, CL301.Contig4\_Mf\_liverA, CL301.Contig5\_Mf\_liverA, CL301.Contig6\_Mf\_liverA, CL301.Contig7\_Mf\_liverA, CL301.Contig8\_Mf\_liverA, CL301.Contig9\_Mf\_liverA, CL302.Contig2\_Mf\_liverA, CL3081.Contig1\_Mf\_liverA, CL3112.Contig1\_Mf\_liverA, CL3112.Contig6\_Mf\_liverA, CL3112.Contig7\_Mf\_liverA, CL3112.Contig8\_Mf\_liverA, CL3112.Contig9\_Mf\_liverA, CL3147.Contig1\_Mf\_liverA, CL3168.Contig1\_Mf\_liverA, CL3168.Contig2\_Mf\_liverA, CL3173.Contig1\_Mf\_liverA, CL3173.Contig2\_Mf\_liverA, CL3198.Contig1\_Mf\_liverA, CL3227.Contig1\_Mf\_liverA, CL3227.Contig2\_Mf\_liverA, CL3227.Contig3\_Mf\_liverA, CL3227.Contig4\_Mf\_liverA, CL3469.Contig1\_Mf\_liverA, CL3469.Contig2\_Mf\_liverA, CL3507.Contig1\_Mf\_liverA, CL3507.Contig2\_Mf\_liverA, CL3616.Contig1\_Mf\_liverA, CL3616.Contig2\_Mf\_liverA, CL3631.Contig1\_Mf\_liverA, CL3631.Contig2\_Mf\_liverA, CL3681.Contig1\_Mf\_liverA, CL3681.Contig2\_Mf\_liverA, CL3709.Contig1\_Mf\_liverA, CL3709.Contig2\_Mf\_liverA, CL3749.Contig1\_Mf\_liverA, CL3749.Contig2\_Mf\_liverA, CL3813.Contig1\_Mf\_liverA, CL3813.Contig2\_Mf\_liverA, CL3827.Contig1\_Mf\_liverA, CL3827.Contig2\_Mf\_liverA, CL3831.Contig1\_Mf\_liverA, CL3859.Contig1\_Mf\_liverA, CL3859.Contig2\_Mf\_liverA, CL3916.Contig1\_Mf\_liverA, CL3916.Contig2\_Mf\_liverA, CL3975.Contig1\_Mf\_liverA, CL3984.Contig1\_Mf\_liverA, CL3984.Contig2\_Mf\_liverA, CL410.Contig13\_Mf\_liverA, CL410.Contig14\_Mf\_liverA, CL410.Contig15\_Mf\_liverA, CL410.Contig16\_Mf\_liverA, CL410.Contig17\_Mf\_liverA, CL410.Contig9\_Mf\_liverA, CL4142.Contig1\_Mf\_liverA, CL4142.Contig2\_Mf\_liverA, CL4208.Contig1\_Mf\_liverA, CL4208.Contig2\_Mf\_liverA, CL4236.Contig1\_Mf\_liverA, CL4236.Contig2\_Mf\_liverA, CL4265.Contig1\_Mf\_liverA, CL4276.Contig2\_Mf\_liverA, CL44.Contig1\_Mf\_liverA, CL44.Contig2\_Mf\_liverA, CL4404.Contig1\_Mf\_liverA, CL4404.Contig2\_Mf\_liverA, CL4409.Contig1\_Mf\_liverA, CL4415.Contig1\_Mf\_liverA, CL4437.Contig1\_Mf\_liverA, CL4557.Contig1\_Mf\_liverA, CL4557.Contig2\_Mf\_liverA, CL4633.Contig1\_Mf\_liverA, CL4633.Contig2\_Mf\_liverA, CL4664.Contig1\_Mf\_liverA, CL4664.Contig2\_Mf\_liverA, CL4722.Contig1\_Mf\_liverA, CL4722.Contig2\_Mf\_liverA, CL4741.Contig1\_Mf\_liverA, CL4741.Contig2\_Mf\_liverA, CL4757.Contig1\_Mf\_liverA, CL4757.Contig2\_Mf\_liverA, CL4846.Contig1\_Mf\_liverA, CL4846.Contig2\_Mf\_liverA, CL4848.Contig1\_Mf\_liverA, CL4848.Contig2\_Mf\_liverA, CL4883.Contig1\_Mf\_liverA, CL4901.Contig1\_Mf\_liverA, CL4901.Contig2\_Mf\_liverA, CL4918.Contig1\_Mf\_liverA, CL4918.Contig2\_Mf\_liverA, CL4919.Contig1\_Mf\_liverA, CL4919.Contig2\_Mf\_liverA, CL4952.Contig1\_Mf\_liverA, CL4952.Contig2\_Mf\_liverA, CL4985.Contig1\_Mf\_liverA, CL4985.Contig2\_Mf\_liverA, CL5132.Contig2\_Mf\_liverA, CL5235.Contig1\_Mf\_liverA, CL5235.Contig2\_Mf\_liverA, CL5254.Contig1\_Mf\_liverA, CL5254.Contig2\_Mf\_liverA, CL532.Contig1\_Mf\_liverA, CL532.Contig2\_Mf\_liverA, CL5448.Contig1\_Mf\_liverA, CL5461.Contig1\_Mf\_liverA, CL5461.Contig2\_Mf\_liverA, CL5552.Contig1\_Mf\_liverA, CL5552.Contig2\_Mf\_liverA, CL5570.Contig1\_Mf\_liverA, CL5570.Contig2\_Mf\_liverA, CL5595.Contig1\_Mf\_liverA, CL5595.Contig2\_Mf\_liverA, CL5643.Contig1\_Mf\_liverA, CL5674.Contig1\_Mf\_liverA, CL5674.Contig2\_Mf\_liverA, CL5674.Contig3\_Mf\_liverA, CL5691.Contig1\_Mf\_liverA, CL5691.Contig2\_Mf\_liverA, CL5771.Contig1\_Mf\_liverA, CL5771.Contig2\_Mf\_liverA, CL5782.Contig1\_Mf\_liverA, CL5782.Contig2\_Mf\_liverA, CL5782.Contig3\_Mf\_liverA, CL5861.Contig1\_Mf\_liverA, CL5861.Contig2\_Mf\_liverA, CL5975.Contig1\_Mf\_liverA, CL5975.Contig2\_Mf\_liverA, CL6029.Contig1\_Mf\_liverA, CL6029.Contig2\_Mf\_liverA, CL6029.Contig3\_Mf\_liverA, CL6029.Contig4\_Mf\_liverA, CL831.Contig1\_Mf\_liverA, CL831.Contig2\_Mf\_liverA, CL831.Contig3\_Mf\_liverA, CL831.Contig4\_Mf\_liverA, CL831.Contig5\_Mf\_liverA, CL838.Contig1\_Mf\_liverA, CL838.Contig2\_Mf\_liverA, CL838.Contig3\_Mf\_liverA, CL838.Contig4\_Mf\_liverA, CL838.Contig5\_Mf\_liverA, CL838.Contig6\_Mf\_liverA, CL890.Contig1\_Mf\_liverA, CL890.Contig2\_Mf\_liverA, CL960.Contig10\_Mf\_liverA, CL960.Contig11\_Mf\_liverA, CL960.Contig12\_Mf\_liverA, CL960.Contig13\_Mf\_liverA, CL960.Contig14\_Mf\_liverA, CL960.Contig15\_Mf\_liverA, CL960.Contig1\_Mf\_liverA, CL960.Contig2\_Mf\_liverA, CL960.Contig3\_Mf\_liverA, CL960.Contig4\_Mf\_liverA, CL960.Contig5\_Mf\_liverA, Unigene10145\_Mf\_liverA, Unigene10249\_Mf\_liverA, Unigene10341\_Mf\_liverA, Unigene10780\_Mf\_liverA, Unigene11673\_Mf\_liverA, Unigene11737\_Mf\_liverA, Unigene11782\_Mf\_liverA, Unigene11913\_Mf\_liverA, Unigene11991\_Mf\_liverA, Unigene11\_Mf\_liverA, Unigene1200\_Mf\_liverA, Unigene12161\_Mf\_liverA, Unigene12189\_Mf\_liverA, Unigene12713\_Mf\_liverA, Unigene12877\_Mf\_liverA, Unigene12932\_Mf\_liverA, Unigene13067\_Mf\_liverA, Unigene1309\_Mf\_liverA, Unigene13153\_Mf\_liverA, Unigene13265\_Mf\_liverA, Unigene13379\_Mf\_liverA, Unigene13438\_Mf\_liverA, Unigene13439\_Mf\_liverA, Unigene13474\_Mf\_liverA, Unigene13475\_Mf\_liverA, Unigene13537\_Mf\_liverA, Unigene13538\_Mf\_liverA, Unigene13695\_Mf\_liverA, Unigene13919\_Mf\_liverA, Unigene13988\_Mf\_liverA, Unigene14093\_Mf\_liverA, Unigene14094\_Mf\_liverA, Unigene14108\_Mf\_liverA, Unigene14109\_Mf\_liverA, Unigene14377\_Mf\_liverA, Unigene14378\_Mf\_liverA, Unigene14602\_Mf\_liverA, Unigene1461\_Mf\_liverA, Unigene14695\_Mf\_liverA, Unigene14807\_Mf\_liverA, Unigene15002\_Mf\_liverA, Unigene15125\_Mf\_liverA, Unigene15295\_Mf\_liverA, Unigene15296\_Mf\_liverA, Unigene152\_Mf\_liverA, Unigene15401\_Mf\_liverA, Unigene1549\_Mf\_liverA, Unigene15588\_Mf\_liverA, Unigene16048\_Mf\_liverA, Unigene16240\_Mf\_liverA, Unigene16241\_Mf\_liverA, Unigene16321\_Mf\_liverA, Unigene16373\_Mf\_liverA, Unigene16374\_Mf\_liverA, Unigene16529\_Mf\_liverA, Unigene16547\_Mf\_liverA, Unigene16548\_Mf\_liverA, Unigene16566\_Mf\_liverA, Unigene16759\_Mf\_liverA, Unigene16788\_Mf\_liverA, Unigene16789\_Mf\_liverA, Unigene16819\_Mf\_liverA, Unigene16820\_Mf\_liverA, Unigene17039\_Mf\_liverA, Unigene17040\_Mf\_liverA, Unigene17069\_Mf\_liverA, Unigene17657\_Mf\_liverA, Unigene17855\_Mf\_liverA, Unigene17893\_Mf\_liverA, Unigene1825\_Mf\_liverA, Unigene18385\_Mf\_liverA, Unigene18420\_Mf\_liverA, Unigene18428\_Mf\_liverA, Unigene18656\_Mf\_liverA, Unigene18657\_Mf\_liverA, Unigene18722\_Mf\_liverA, Unigene18723\_Mf\_liverA, Unigene18757\_Mf\_liverA, Unigene19715\_Mf\_liverA, Unigene19716\_Mf\_liverA, Unigene19967\_Mf\_liverA, Unigene20149\_Mf\_liverA, Unigene20340\_Mf\_liverA, Unigene20382\_Mf\_liverA, Unigene20383\_Mf\_liverA, Unigene20795\_Mf\_liverA, Unigene21013\_Mf\_liverA, Unigene2139\_Mf\_liverA, Unigene21558\_Mf\_liverA, Unigene21560\_Mf\_liverA, Unigene2161\_Mf\_liverA, Unigene21622\_Mf\_liverA, Unigene21972\_Mf\_liverA, Unigene21979\_Mf\_liverA, Unigene22381\_Mf\_liverA, Unigene22801\_Mf\_liverA, Unigene22919\_Mf\_liverA, Unigene22921\_Mf\_liverA, Unigene23006\_Mf\_liverA, Unigene23108\_Mf\_liverA, Unigene23341\_Mf\_liverA, Unigene23600\_Mf\_liverA, Unigene23601\_Mf\_liverA, Unigene23652\_Mf\_liverA, Unigene23653\_Mf\_liverA, Unigene23654\_Mf\_liverA, Unigene2377\_Mf\_liverA, Unigene24065\_Mf\_liverA, Unigene24068\_Mf\_liverA, Unigene24069\_Mf\_liverA, Unigene24448\_Mf\_liverA, Unigene24449\_Mf\_liverA, Unigene24576\_Mf\_liverA, Unigene24689\_Mf\_liverA, Unigene24794\_Mf\_liverA, Unigene24797\_Mf\_liverA, Unigene24798\_Mf\_liverA, Unigene24799\_Mf\_liverA, Unigene24800\_Mf\_liverA, Unigene24804\_Mf\_liverA, Unigene25070\_Mf\_liverA, Unigene25119\_Mf\_liverA, Unigene25162\_Mf\_liverA, Unigene25172\_Mf\_liverA, Unigene25200\_Mf\_liverA, Unigene25748\_Mf\_liverA, Unigene25753\_Mf\_liverA, Unigene25821\_Mf\_liverA, Unigene25853\_Mf\_liverA, Unigene26250\_Mf\_liverA, Unigene26413\_Mf\_liverA, Unigene26468\_Mf\_liverA, Unigene2689\_Mf\_liverA, Unigene27007\_Mf\_liverA, Unigene27010\_Mf\_liverA, Unigene2721\_Mf\_liverA, Unigene27230\_Mf\_liverA, Unigene27231\_Mf\_liverA, Unigene27312\_Mf\_liverA, Unigene27313\_Mf\_liverA, Unigene27478\_Mf\_liverA, Unigene27633\_Mf\_liverA, Unigene27643\_Mf\_liverA, Unigene28104\_Mf\_liverA, Unigene28307\_Mf\_liverA, Unigene28308\_Mf\_liverA, Unigene28309\_Mf\_liverA, Unigene28405\_Mf\_liverA, Unigene28409\_Mf\_liverA, Unigene28437\_Mf\_liverA, Unigene28504\_Mf\_liverA, Unigene28505\_Mf\_liverA, Unigene2854\_Mf\_liverA, Unigene28718\_Mf\_liverA, Unigene28719\_Mf\_liverA, Unigene2873\_Mf\_liverA, Unigene28899\_Mf\_liverA, Unigene28989\_Mf\_liverA, Unigene29095\_Mf\_liverA, Unigene29119\_Mf\_liverA, Unigene29120\_Mf\_liverA, Unigene29134\_Mf\_liverA, Unigene29231\_Mf\_liverA, Unigene29399\_Mf\_liverA, Unigene29416\_Mf\_liverA, Unigene29617\_Mf\_liverA, Unigene29725\_Mf\_liverA, Unigene29858\_Mf\_liverA, Unigene29921\_Mf\_liverA, Unigene30517\_Mf\_liverA, Unigene30518\_Mf\_liverA, Unigene30642\_Mf\_liverA, Unigene30839\_Mf\_liverA, Unigene30872\_Mf\_liverA, Unigene30981\_Mf\_liverA, Unigene31006\_Mf\_liverA, Unigene31304\_Mf\_liverA, Unigene31305\_Mf\_liverA, Unigene31333\_Mf\_liverA, Unigene31822\_Mf\_liverA, Unigene32002\_Mf\_liverA, Unigene32003\_Mf\_liverA, Unigene32058\_Mf\_liverA, Unigene32088\_Mf\_liverA, Unigene3222\_Mf\_liverA, Unigene32239\_Mf\_liverA, Unigene32334\_Mf\_liverA, Unigene32335\_Mf\_liverA, Unigene32375\_Mf\_liverA, Unigene32376\_Mf\_liverA, Unigene32568\_Mf\_liverA, Unigene32863\_Mf\_liverA, Unigene32864\_Mf\_liverA, Unigene32865\_Mf\_liverA, Unigene32879\_Mf\_liverA, Unigene32882\_Mf\_liverA, Unigene32883\_Mf\_liverA, Unigene32912\_Mf\_liverA, Unigene32913\_Mf\_liverA, Unigene33024\_Mf\_liverA, Unigene33025\_Mf\_liverA, Unigene33039\_Mf\_liverA, Unigene33074\_Mf\_liverA, Unigene33122\_Mf\_liverA, Unigene33273\_Mf\_liverA, Unigene33316\_Mf\_liverA, Unigene33338\_Mf\_liverA, Unigene33339\_Mf\_liverA, Unigene33340\_Mf\_liverA, Unigene33341\_Mf\_liverA, Unigene33342\_Mf\_liverA, Unigene33343\_Mf\_liverA, Unigene33353\_Mf\_liverA, Unigene33409\_Mf\_liverA, Unigene33886\_Mf\_liverA, Unigene33887\_Mf\_liverA, Unigene33889\_Mf\_liverA, Unigene33965\_Mf\_liverA, Unigene33970\_Mf\_liverA, Unigene33971\_Mf\_liverA, Unigene33972\_Mf\_liverA, Unigene34411\_Mf\_liverA, Unigene34777\_Mf\_liverA, Unigene34823\_Mf\_liverA, Unigene34975\_Mf\_liverA, Unigene35459\_Mf\_liverA, Unigene35570\_Mf\_liverA, Unigene35571\_Mf\_liverA, Unigene35572\_Mf\_liverA, Unigene35573\_Mf\_liverA, Unigene35627\_Mf\_liverA, Unigene35816\_Mf\_liverA, Unigene35875\_Mf\_liverA, Unigene35898\_Mf\_liverA, Unigene35899\_Mf\_liverA, Unigene35900\_Mf\_liverA, Unigene35901\_Mf\_liverA, Unigene35902\_Mf\_liverA, Unigene36260\_Mf\_liverA, Unigene36261\_Mf\_liverA, Unigene36613\_Mf\_liverA, Unigene36799\_Mf\_liverA, Unigene36901\_Mf\_liverA, Unigene36926\_Mf\_liverA, Unigene37025\_Mf\_liverA, Unigene37101\_Mf\_liverA, Unigene37132\_Mf\_liverA, Unigene37147\_Mf\_liverA, Unigene37358\_Mf\_liverA, Unigene37683\_Mf\_liverA, Unigene3788\_Mf\_liverA, Unigene38065\_Mf\_liverA, Unigene38563\_Mf\_liverA, Unigene38681\_Mf\_liverA, Unigene38816\_Mf\_liverA, Unigene38839\_Mf\_liverA, Unigene38919\_Mf\_liverA, Unigene39200\_Mf\_liverA, Unigene39263\_Mf\_liverA, Unigene39640\_Mf\_liverA, Unigene40101\_Mf\_liverA, Unigene40126\_Mf\_liverA, Unigene40223\_Mf\_liverA, Unigene40428\_Mf\_liverA, Unigene40622\_Mf\_liverA, Unigene40644\_Mf\_liverA, Unigene41078\_Mf\_liverA, Unigene41505\_Mf\_liverA, Unigene42223\_Mf\_liverA, Unigene4226\_Mf\_liverA, Unigene42817\_Mf\_liverA, Unigene43225\_Mf\_liverA, Unigene43534\_Mf\_liverA, Unigene43765\_Mf\_liverA, Unigene43966\_Mf\_liverA, Unigene44118\_Mf\_liverA, Unigene44310\_Mf\_liverA, Unigene44469\_Mf\_liverA, Unigene45261\_Mf\_liverA, Unigene45585\_Mf\_liverA, Unigene45634\_Mf\_liverA, Unigene4565\_Mf\_liverA, Unigene45685\_Mf\_liverA, Unigene45768\_Mf\_liverA, Unigene46145\_Mf\_liverA, Unigene46281\_Mf\_liverA, Unigene46368\_Mf\_liverA, Unigene46474\_Mf\_liverA, Unigene466\_Mf\_liverA, Unigene467\_Mf\_liverA, Unigene468\_Mf\_liverA, Unigene47869\_Mf\_liverA, Unigene48313\_Mf\_liverA, Unigene48509\_Mf\_liverA, Unigene48539\_Mf\_liverA, Unigene48553\_Mf\_liverA, Unigene48645\_Mf\_liverA, Unigene48762\_Mf\_liverA, Unigene49007\_Mf\_liverA, Unigene49035\_Mf\_liverA, Unigene49037\_Mf\_liverA, Unigene49384\_Mf\_liverA, Unigene49797\_Mf\_liverA, Unigene50004\_Mf\_liverA, Unigene50057\_Mf\_liverA, Unigene50600\_Mf\_liverA, Unigene5147\_Mf\_liverA, Unigene51487\_Mf\_liverA, Unigene51985\_Mf\_liverA, Unigene5434\_Mf\_liverA, Unigene557\_Mf\_liverA, Unigene5725\_Mf\_liverA, Unigene5818\_Mf\_liverA, Unigene583\_Mf\_liverA, Unigene5983\_Mf\_liverA, Unigene5984\_Mf\_liverA, Unigene610\_Mf\_liverA, Unigene6481\_Mf\_liverA, Unigene6560\_Mf\_liverA, Unigene6561\_Mf\_liverA, Unigene6734\_Mf\_liverA, Unigene6735\_Mf\_liverA, Unigene6781\_Mf\_liverA, Unigene6782\_Mf\_liverA, Unigene7104\_Mf\_liverA, Unigene7105\_Mf\_liverA, Unigene7108\_Mf\_liverA, Unigene7477\_Mf\_liverA, Unigene7478\_Mf\_liverA, Unigene7589\_Mf\_liverA, Unigene7729\_Mf\_liverA, Unigene7730\_Mf\_liverA, Unigene774\_Mf\_liverA, Unigene7897\_Mf\_liverA, Unigene8102\_Mf\_liverA, Unigene8233\_Mf\_liverA, Unigene8566\_Mf\_liverA, Unigene8604\_Mf\_liverA, Unigene8624\_Mf\_liverA, Unigene8878\_Mf\_liverA, Unigene8907\_Mf\_liverA, Unigene9423\_Mf\_liverA, Unigene9728\_Mf\_liverA, Unigene991\_Mf\_liverA |
| 9 | Transcriptional misregulation in cancer | CL1039.Contig1\_Mf\_liverA, CL1039.Contig2\_Mf\_liverA, CL1075.Contig1\_Mf\_liverA, CL1075.Contig2\_Mf\_liverA, CL1197.Contig1\_Mf\_liverA, CL1197.Contig2\_Mf\_liverA, CL1278.Contig1\_Mf\_liverA, CL1278.Contig2\_Mf\_liverA, CL1309.Contig1\_Mf\_liverA, CL1309.Contig2\_Mf\_liverA, CL1338.Contig7\_Mf\_liverA, CL1404.Contig1\_Mf\_liverA, CL1404.Contig2\_Mf\_liverA, CL1404.Contig3\_Mf\_liverA, CL1404.Contig4\_Mf\_liverA, CL1404.Contig5\_Mf\_liverA, CL1503.Contig1\_Mf\_liverA, CL1503.Contig2\_Mf\_liverA, CL1503.Contig3\_Mf\_liverA, CL1553.Contig1\_Mf\_liverA, CL1553.Contig2\_Mf\_liverA, CL1553.Contig3\_Mf\_liverA, CL1553.Contig4\_Mf\_liverA, CL1553.Contig5\_Mf\_liverA, CL1567.Contig10\_Mf\_liverA, CL1567.Contig11\_Mf\_liverA, CL1567.Contig12\_Mf\_liverA, CL1567.Contig13\_Mf\_liverA, CL1567.Contig14\_Mf\_liverA, CL1567.Contig15\_Mf\_liverA, CL1567.Contig1\_Mf\_liverA, CL1567.Contig2\_Mf\_liverA, CL1567.Contig3\_Mf\_liverA, CL1567.Contig4\_Mf\_liverA, CL1567.Contig5\_Mf\_liverA, CL1567.Contig6\_Mf\_liverA, CL1567.Contig7\_Mf\_liverA, CL1567.Contig8\_Mf\_liverA, CL1567.Contig9\_Mf\_liverA, CL1653.Contig1\_Mf\_liverA, CL1653.Contig2\_Mf\_liverA, CL1717.Contig1\_Mf\_liverA, CL1717.Contig2\_Mf\_liverA, CL1717.Contig3\_Mf\_liverA, CL1730.Contig1\_Mf\_liverA, CL1730.Contig2\_Mf\_liverA, CL1753.Contig1\_Mf\_liverA, CL1753.Contig2\_Mf\_liverA, CL1820.Contig1\_Mf\_liverA, CL1820.Contig2\_Mf\_liverA, CL1861.Contig1\_Mf\_liverA, CL1861.Contig2\_Mf\_liverA, CL1921.Contig1\_Mf\_liverA, CL1921.Contig2\_Mf\_liverA, CL1921.Contig3\_Mf\_liverA, CL1939.Contig1\_Mf\_liverA, CL1939.Contig2\_Mf\_liverA, CL197.Contig1\_Mf\_liverA, CL197.Contig2\_Mf\_liverA, CL197.Contig3\_Mf\_liverA, CL197.Contig4\_Mf\_liverA, CL197.Contig5\_Mf\_liverA, CL197.Contig6\_Mf\_liverA, CL197.Contig7\_Mf\_liverA, CL197.Contig8\_Mf\_liverA, CL2004.Contig1\_Mf\_liverA, CL2004.Contig2\_Mf\_liverA, CL2052.Contig1\_Mf\_liverA, CL2052.Contig2\_Mf\_liverA, CL2052.Contig3\_Mf\_liverA, CL2052.Contig4\_Mf\_liverA, CL2052.Contig5\_Mf\_liverA, CL2052.Contig6\_Mf\_liverA, CL2061.Contig1\_Mf\_liverA, CL2061.Contig2\_Mf\_liverA, CL2079.Contig1\_Mf\_liverA, CL2128.Contig1\_Mf\_liverA, CL2128.Contig2\_Mf\_liverA, CL2128.Contig3\_Mf\_liverA, CL2128.Contig4\_Mf\_liverA, CL2130.Contig1\_Mf\_liverA, CL2130.Contig2\_Mf\_liverA, CL2130.Contig3\_Mf\_liverA, CL2132.Contig1\_Mf\_liverA, CL2135.Contig1\_Mf\_liverA, CL2135.Contig2\_Mf\_liverA, CL2135.Contig3\_Mf\_liverA, CL2174.Contig1\_Mf\_liverA, CL2191.Contig1\_Mf\_liverA, CL2191.Contig2\_Mf\_liverA, CL2191.Contig3\_Mf\_liverA, CL2195.Contig1\_Mf\_liverA, CL2249.Contig1\_Mf\_liverA, CL2249.Contig2\_Mf\_liverA, CL2355.Contig1\_Mf\_liverA, CL2355.Contig2\_Mf\_liverA, CL2384.Contig1\_Mf\_liverA, CL2384.Contig2\_Mf\_liverA, CL2439.Contig1\_Mf\_liverA, CL2439.Contig2\_Mf\_liverA, CL244.Contig10\_Mf\_liverA, CL244.Contig11\_Mf\_liverA, CL244.Contig12\_Mf\_liverA, CL244.Contig13\_Mf\_liverA, CL244.Contig14\_Mf\_liverA, CL244.Contig15\_Mf\_liverA, CL244.Contig16\_Mf\_liverA, CL244.Contig17\_Mf\_liverA, CL244.Contig18\_Mf\_liverA, CL244.Contig19\_Mf\_liverA, CL244.Contig1\_Mf\_liverA, CL244.Contig20\_Mf\_liverA, CL244.Contig21\_Mf\_liverA, CL244.Contig22\_Mf\_liverA, CL244.Contig23\_Mf\_liverA, CL244.Contig24\_Mf\_liverA, CL244.Contig25\_Mf\_liverA, CL244.Contig26\_Mf\_liverA, CL244.Contig27\_Mf\_liverA, CL244.Contig28\_Mf\_liverA, CL244.Contig29\_Mf\_liverA, CL244.Contig2\_Mf\_liverA, CL244.Contig30\_Mf\_liverA, CL244.Contig31\_Mf\_liverA, CL244.Contig32\_Mf\_liverA, CL244.Contig33\_Mf\_liverA, CL244.Contig34\_Mf\_liverA, CL244.Contig35\_Mf\_liverA, CL244.Contig36\_Mf\_liverA, CL244.Contig37\_Mf\_liverA, CL244.Contig38\_Mf\_liverA, CL244.Contig39\_Mf\_liverA, CL244.Contig3\_Mf\_liverA, CL244.Contig4\_Mf\_liverA, CL244.Contig5\_Mf\_liverA, CL244.Contig6\_Mf\_liverA, CL244.Contig7\_Mf\_liverA, CL244.Contig8\_Mf\_liverA, CL244.Contig9\_Mf\_liverA, CL246.Contig1\_Mf\_liverA, CL246.Contig2\_Mf\_liverA, CL246.Contig3\_Mf\_liverA, CL2549.Contig1\_Mf\_liverA, CL2549.Contig2\_Mf\_liverA, CL255.Contig2\_Mf\_liverA, CL255.Contig3\_Mf\_liverA, CL255.Contig8\_Mf\_liverA, CL2610.Contig1\_Mf\_liverA, CL2610.Contig2\_Mf\_liverA, CL2638.Contig1\_Mf\_liverA, CL2638.Contig2\_Mf\_liverA, CL2638.Contig3\_Mf\_liverA, CL2638.Contig4\_Mf\_liverA, CL2664.Contig1\_Mf\_liverA, CL2664.Contig2\_Mf\_liverA, CL2664.Contig3\_Mf\_liverA, CL2664.Contig4\_Mf\_liverA, CL2686.Contig1\_Mf\_liverA, CL2686.Contig2\_Mf\_liverA, CL2696.Contig1\_Mf\_liverA, CL2696.Contig2\_Mf\_liverA, CL2696.Contig3\_Mf\_liverA, CL2696.Contig4\_Mf\_liverA, CL2696.Contig5\_Mf\_liverA, CL2718.Contig1\_Mf\_liverA, CL2718.Contig2\_Mf\_liverA, CL2718.Contig3\_Mf\_liverA, CL2718.Contig4\_Mf\_liverA, CL2802.Contig1\_Mf\_liverA, CL2802.Contig2\_Mf\_liverA, CL2802.Contig3\_Mf\_liverA, CL2805.Contig1\_Mf\_liverA, CL2805.Contig2\_Mf\_liverA, CL2805.Contig3\_Mf\_liverA, CL2805.Contig4\_Mf\_liverA, CL3019.Contig1\_Mf\_liverA, CL3019.Contig2\_Mf\_liverA, CL3143.Contig1\_Mf\_liverA, CL3162.Contig1\_Mf\_liverA, CL3162.Contig2\_Mf\_liverA, CL3162.Contig3\_Mf\_liverA, CL3162.Contig4\_Mf\_liverA, CL3162.Contig5\_Mf\_liverA, CL3258.Contig1\_Mf\_liverA, CL340.Contig1\_Mf\_liverA, CL340.Contig2\_Mf\_liverA, CL340.Contig3\_Mf\_liverA, CL340.Contig4\_Mf\_liverA, CL340.Contig5\_Mf\_liverA, CL340.Contig6\_Mf\_liverA, CL340.Contig7\_Mf\_liverA, CL349.Contig10\_Mf\_liverA, CL349.Contig11\_Mf\_liverA, CL349.Contig12\_Mf\_liverA, CL349.Contig13\_Mf\_liverA, CL349.Contig14\_Mf\_liverA, CL349.Contig15\_Mf\_liverA, CL349.Contig16\_Mf\_liverA, CL349.Contig1\_Mf\_liverA, CL349.Contig2\_Mf\_liverA, CL349.Contig3\_Mf\_liverA, CL349.Contig4\_Mf\_liverA, CL349.Contig5\_Mf\_liverA, CL349.Contig6\_Mf\_liverA, CL349.Contig7\_Mf\_liverA, CL349.Contig8\_Mf\_liverA, CL349.Contig9\_Mf\_liverA, CL3501.Contig1\_Mf\_liverA, CL3501.Contig2\_Mf\_liverA, CL3501.Contig3\_Mf\_liverA, CL3534.Contig1\_Mf\_liverA, CL3534.Contig2\_Mf\_liverA, CL3639.Contig1\_Mf\_liverA, CL3727.Contig1\_Mf\_liverA, CL3727.Contig2\_Mf\_liverA, CL3994.Contig1\_Mf\_liverA, CL4015.Contig1\_Mf\_liverA, CL4015.Contig2\_Mf\_liverA, CL4022.Contig1\_Mf\_liverA, CL4022.Contig2\_Mf\_liverA, CL4022.Contig3\_Mf\_liverA, CL4086.Contig1\_Mf\_liverA, CL4086.Contig2\_Mf\_liverA, CL4142.Contig1\_Mf\_liverA, CL4142.Contig2\_Mf\_liverA, CL4329.Contig1\_Mf\_liverA, CL4329.Contig2\_Mf\_liverA, CL4337.Contig1\_Mf\_liverA, CL4337.Contig2\_Mf\_liverA, CL4338.Contig1\_Mf\_liverA, CL4338.Contig2\_Mf\_liverA, CL4444.Contig1\_Mf\_liverA, CL4444.Contig2\_Mf\_liverA, CL4525.Contig1\_Mf\_liverA, CL4525.Contig2\_Mf\_liverA, CL4720.Contig1\_Mf\_liverA, CL4720.Contig2\_Mf\_liverA, CL4722.Contig1\_Mf\_liverA, CL4722.Contig2\_Mf\_liverA, CL4757.Contig1\_Mf\_liverA, CL4757.Contig2\_Mf\_liverA, CL4770.Contig1\_Mf\_liverA, CL4770.Contig2\_Mf\_liverA, CL4776.Contig1\_Mf\_liverA, CL4776.Contig2\_Mf\_liverA, CL4776.Contig3\_Mf\_liverA, CL4807.Contig1\_Mf\_liverA, CL4807.Contig2\_Mf\_liverA, CL4841.Contig1\_Mf\_liverA, CL4841.Contig2\_Mf\_liverA, CL4850.Contig1\_Mf\_liverA, CL4850.Contig2\_Mf\_liverA, CL4859.Contig1\_Mf\_liverA, CL4859.Contig2\_Mf\_liverA, CL4870.Contig1\_Mf\_liverA, CL4888.Contig1\_Mf\_liverA, CL4901.Contig1\_Mf\_liverA, CL4901.Contig2\_Mf\_liverA, CL4926.Contig1\_Mf\_liverA, CL4945.Contig1\_Mf\_liverA, CL4945.Contig2\_Mf\_liverA, CL4975.Contig1\_Mf\_liverA, CL4975.Contig2\_Mf\_liverA, CL4985.Contig1\_Mf\_liverA, CL4985.Contig2\_Mf\_liverA, CL5083.Contig1\_Mf\_liverA, CL5083.Contig2\_Mf\_liverA, CL5184.Contig1\_Mf\_liverA, CL52.Contig1\_Mf\_liverA, CL52.Contig2\_Mf\_liverA, CL5219.Contig1\_Mf\_liverA, CL5246.Contig1\_Mf\_liverA, CL5246.Contig2\_Mf\_liverA, CL5278.Contig1\_Mf\_liverA, CL5278.Contig2\_Mf\_liverA, CL5375.Contig1\_Mf\_liverA, CL5375.Contig2\_Mf\_liverA, CL5570.Contig1\_Mf\_liverA, CL5570.Contig2\_Mf\_liverA, CL5630.Contig1\_Mf\_liverA, CL5630.Contig2\_Mf\_liverA, CL5955.Contig3\_Mf\_liverA, CL62.Contig1\_Mf\_liverA, CL708.Contig6\_Mf\_liverA, CL730.Contig1\_Mf\_liverA, CL730.Contig2\_Mf\_liverA, CL730.Contig3\_Mf\_liverA, CL730.Contig4\_Mf\_liverA, CL730.Contig5\_Mf\_liverA, CL730.Contig6\_Mf\_liverA, CL730.Contig7\_Mf\_liverA, CL730.Contig8\_Mf\_liverA, CL740.Contig2\_Mf\_liverA, CL839.Contig1\_Mf\_liverA, CL839.Contig2\_Mf\_liverA, CL872.Contig1\_Mf\_liverA, CL872.Contig2\_Mf\_liverA, CL922.Contig1\_Mf\_liverA, CL922.Contig2\_Mf\_liverA, CL922.Contig4\_Mf\_liverA, CL977.Contig1\_Mf\_liverA, CL977.Contig2\_Mf\_liverA, CL977.Contig3\_Mf\_liverA, Unigene10040\_Mf\_liverA, Unigene10168\_Mf\_liverA, Unigene10351\_Mf\_liverA, Unigene10606\_Mf\_liverA, Unigene10820\_Mf\_liverA, Unigene11007\_Mf\_liverA, Unigene11008\_Mf\_liverA, Unigene11390\_Mf\_liverA, Unigene11782\_Mf\_liverA, Unigene11797\_Mf\_liverA, Unigene11927\_Mf\_liverA, Unigene1204\_Mf\_liverA, Unigene12213\_Mf\_liverA, Unigene12374\_Mf\_liverA, Unigene12444\_Mf\_liverA, Unigene12733\_Mf\_liverA, Unigene13438\_Mf\_liverA, Unigene13439\_Mf\_liverA, Unigene1350\_Mf\_liverA, Unigene13790\_Mf\_liverA, Unigene13791\_Mf\_liverA, Unigene13930\_Mf\_liverA, Unigene14035\_Mf\_liverA, Unigene14108\_Mf\_liverA, Unigene14109\_Mf\_liverA, Unigene14774\_Mf\_liverA, Unigene14907\_Mf\_liverA, Unigene14998\_Mf\_liverA, Unigene14\_Mf\_liverA, Unigene15018\_Mf\_liverA, Unigene15066\_Mf\_liverA, Unigene15229\_Mf\_liverA, Unigene15361\_Mf\_liverA, Unigene15440\_Mf\_liverA, Unigene15552\_Mf\_liverA, Unigene15553\_Mf\_liverA, Unigene15561\_Mf\_liverA, Unigene15624\_Mf\_liverA, Unigene15867\_Mf\_liverA, Unigene15878\_Mf\_liverA, Unigene15982\_Mf\_liverA, Unigene15986\_Mf\_liverA, Unigene16284\_Mf\_liverA, Unigene16411\_Mf\_liverA, Unigene16799\_Mf\_liverA, Unigene16800\_Mf\_liverA, Unigene1710\_Mf\_liverA, Unigene17166\_Mf\_liverA, Unigene17167\_Mf\_liverA, Unigene17639\_Mf\_liverA, Unigene18129\_Mf\_liverA, Unigene18340\_Mf\_liverA, Unigene18420\_Mf\_liverA, Unigene18757\_Mf\_liverA, Unigene18847\_Mf\_liverA, Unigene19297\_Mf\_liverA, Unigene19658\_Mf\_liverA, Unigene19659\_Mf\_liverA, Unigene19885\_Mf\_liverA, Unigene19967\_Mf\_liverA, Unigene20500\_Mf\_liverA, Unigene20501\_Mf\_liverA, Unigene20616\_Mf\_liverA, Unigene20617\_Mf\_liverA, Unigene20618\_Mf\_liverA, Unigene20619\_Mf\_liverA, Unigene20937\_Mf\_liverA, Unigene21842\_Mf\_liverA, Unigene21843\_Mf\_liverA, Unigene22381\_Mf\_liverA, Unigene22548\_Mf\_liverA, Unigene23031\_Mf\_liverA, Unigene23032\_Mf\_liverA, Unigene23033\_Mf\_liverA, Unigene23292\_Mf\_liverA, Unigene2344\_Mf\_liverA, Unigene23534\_Mf\_liverA, Unigene23537\_Mf\_liverA, Unigene23538\_Mf\_liverA, Unigene23551\_Mf\_liverA, Unigene23668\_Mf\_liverA, Unigene23669\_Mf\_liverA, Unigene23670\_Mf\_liverA, Unigene2377\_Mf\_liverA, Unigene24078\_Mf\_liverA, Unigene24079\_Mf\_liverA, Unigene24209\_Mf\_liverA, Unigene243\_Mf\_liverA, Unigene24448\_Mf\_liverA, Unigene24449\_Mf\_liverA, Unigene24508\_Mf\_liverA, Unigene24669\_Mf\_liverA, Unigene25074\_Mf\_liverA, Unigene25142\_Mf\_liverA, Unigene25171\_Mf\_liverA, Unigene251\_Mf\_liverA, Unigene25541\_Mf\_liverA, Unigene25542\_Mf\_liverA, Unigene25912\_Mf\_liverA, Unigene25914\_Mf\_liverA, Unigene26098\_Mf\_liverA, Unigene26099\_Mf\_liverA, Unigene26106\_Mf\_liverA, Unigene26164\_Mf\_liverA, Unigene26165\_Mf\_liverA, Unigene26166\_Mf\_liverA, Unigene26167\_Mf\_liverA, Unigene26168\_Mf\_liverA, Unigene26297\_Mf\_liverA, Unigene26332\_Mf\_liverA, Unigene26333\_Mf\_liverA, Unigene26413\_Mf\_liverA, Unigene26742\_Mf\_liverA, Unigene27038\_Mf\_liverA, Unigene27099\_Mf\_liverA, Unigene27337\_Mf\_liverA, Unigene27398\_Mf\_liverA, Unigene27399\_Mf\_liverA, Unigene27400\_Mf\_liverA, Unigene27576\_Mf\_liverA, Unigene27577\_Mf\_liverA, Unigene27640\_Mf\_liverA, Unigene27924\_Mf\_liverA, Unigene28355\_Mf\_liverA, Unigene28356\_Mf\_liverA, Unigene28609\_Mf\_liverA, Unigene28613\_Mf\_liverA, Unigene29026\_Mf\_liverA, Unigene29027\_Mf\_liverA, Unigene29028\_Mf\_liverA, Unigene29134\_Mf\_liverA, Unigene29141\_Mf\_liverA, Unigene29270\_Mf\_liverA, Unigene2929\_Mf\_liverA, Unigene29315\_Mf\_liverA, Unigene29445\_Mf\_liverA, Unigene29642\_Mf\_liverA, Unigene29749\_Mf\_liverA, Unigene29788\_Mf\_liverA, Unigene29833\_Mf\_liverA, Unigene29991\_Mf\_liverA, Unigene29992\_Mf\_liverA, Unigene29993\_Mf\_liverA, Unigene30203\_Mf\_liverA, Unigene30289\_Mf\_liverA, Unigene30539\_Mf\_liverA, Unigene30540\_Mf\_liverA, Unigene30541\_Mf\_liverA, Unigene30561\_Mf\_liverA, Unigene31014\_Mf\_liverA, Unigene31015\_Mf\_liverA, Unigene31016\_Mf\_liverA, Unigene31315\_Mf\_liverA, Unigene31325\_Mf\_liverA, Unigene31333\_Mf\_liverA, Unigene31339\_Mf\_liverA, Unigene31523\_Mf\_liverA, Unigene31857\_Mf\_liverA, Unigene31890\_Mf\_liverA, Unigene31957\_Mf\_liverA, Unigene31958\_Mf\_liverA, Unigene31959\_Mf\_liverA, Unigene32013\_Mf\_liverA, Unigene3214\_Mf\_liverA, Unigene32230\_Mf\_liverA, Unigene32239\_Mf\_liverA, Unigene32425\_Mf\_liverA, Unigene32536\_Mf\_liverA, Unigene32537\_Mf\_liverA, Unigene32659\_Mf\_liverA, Unigene32879\_Mf\_liverA, Unigene32882\_Mf\_liverA, Unigene32883\_Mf\_liverA, Unigene33247\_Mf\_liverA, Unigene33413\_Mf\_liverA, Unigene34062\_Mf\_liverA, Unigene34063\_Mf\_liverA, Unigene34064\_Mf\_liverA, Unigene34065\_Mf\_liverA, Unigene34066\_Mf\_liverA, Unigene34176\_Mf\_liverA, Unigene341\_Mf\_liverA, Unigene34687\_Mf\_liverA, Unigene34713\_Mf\_liverA, Unigene34714\_Mf\_liverA, Unigene3478\_Mf\_liverA, Unigene34975\_Mf\_liverA, Unigene35015\_Mf\_liverA, Unigene35016\_Mf\_liverA, Unigene35017\_Mf\_liverA, Unigene35259\_Mf\_liverA, Unigene35284\_Mf\_liverA, Unigene35312\_Mf\_liverA, Unigene35313\_Mf\_liverA, Unigene35461\_Mf\_liverA, Unigene35462\_Mf\_liverA, Unigene35463\_Mf\_liverA, Unigene35464\_Mf\_liverA, Unigene35698\_Mf\_liverA, Unigene35755\_Mf\_liverA, Unigene35996\_Mf\_liverA, Unigene36162\_Mf\_liverA, Unigene36173\_Mf\_liverA, Unigene36308\_Mf\_liverA, Unigene36309\_Mf\_liverA, Unigene36310\_Mf\_liverA, Unigene36311\_Mf\_liverA, Unigene36741\_Mf\_liverA, Unigene37150\_Mf\_liverA, Unigene37172\_Mf\_liverA, Unigene37320\_Mf\_liverA, Unigene37432\_Mf\_liverA, Unigene37435\_Mf\_liverA, Unigene38140\_Mf\_liverA, Unigene3850\_Mf\_liverA, Unigene38538\_Mf\_liverA, Unigene3859\_Mf\_liverA, Unigene38630\_Mf\_liverA, Unigene387\_Mf\_liverA, Unigene3908\_Mf\_liverA, Unigene39212\_Mf\_liverA, Unigene39385\_Mf\_liverA, Unigene39640\_Mf\_liverA, Unigene40304\_Mf\_liverA, Unigene40520\_Mf\_liverA, Unigene40601\_Mf\_liverA, Unigene40610\_Mf\_liverA, Unigene41280\_Mf\_liverA, Unigene41724\_Mf\_liverA, Unigene41957\_Mf\_liverA, Unigene4280\_Mf\_liverA, Unigene4311\_Mf\_liverA, Unigene43640\_Mf\_liverA, Unigene44026\_Mf\_liverA, Unigene44118\_Mf\_liverA, Unigene44893\_Mf\_liverA, Unigene45003\_Mf\_liverA, Unigene45417\_Mf\_liverA, Unigene45585\_Mf\_liverA, Unigene46178\_Mf\_liverA, Unigene46281\_Mf\_liverA, Unigene46319\_Mf\_liverA, Unigene46359\_Mf\_liverA, Unigene46432\_Mf\_liverA, Unigene46649\_Mf\_liverA, Unigene47015\_Mf\_liverA, Unigene47668\_Mf\_liverA, Unigene48462\_Mf\_liverA, Unigene48792\_Mf\_liverA, Unigene488\_Mf\_liverA, Unigene49037\_Mf\_liverA, Unigene4907\_Mf\_liverA, Unigene49717\_Mf\_liverA, Unigene4983\_Mf\_liverA, Unigene49924\_Mf\_liverA, Unigene50298\_Mf\_liverA, Unigene51063\_Mf\_liverA, Unigene5171\_Mf\_liverA, Unigene51858\_Mf\_liverA, Unigene5213\_Mf\_liverA, Unigene5252\_Mf\_liverA, Unigene5381\_Mf\_liverA, Unigene5449\_Mf\_liverA, Unigene5754\_Mf\_liverA, Unigene5798\_Mf\_liverA, Unigene5990\_Mf\_liverA, Unigene6037\_Mf\_liverA, Unigene6101\_Mf\_liverA, Unigene6119\_Mf\_liverA, Unigene6259\_Mf\_liverA, Unigene6392\_Mf\_liverA, Unigene6610\_Mf\_liverA, Unigene6734\_Mf\_liverA, Unigene6735\_Mf\_liverA, Unigene7339\_Mf\_liverA, Unigene7627\_Mf\_liverA, Unigene766\_Mf\_liverA, Unigene8078\_Mf\_liverA, Unigene8106\_Mf\_liverA, Unigene826\_Mf\_liverA, Unigene8479\_Mf\_liverA, Unigene9599\_Mf\_liverA, Unigene9619\_Mf\_liverA, Unigene9690\_Mf\_liverA, Unigene9719\_Mf\_liverA, Unigene9896\_Mf\_liverA |
| 10 | Amoebiasis | CL1044.Contig1\_Mf\_liverA, CL1044.Contig2\_Mf\_liverA, CL1103.Contig1\_Mf\_liverA, CL1190.Contig1\_Mf\_liverA, CL1190.Contig2\_Mf\_liverA, CL1190.Contig3\_Mf\_liverA, CL1197.Contig1\_Mf\_liverA, CL1197.Contig2\_Mf\_liverA, CL1199.Contig10\_Mf\_liverA, CL1199.Contig11\_Mf\_liverA, CL1199.Contig12\_Mf\_liverA, CL1199.Contig13\_Mf\_liverA, CL1199.Contig14\_Mf\_liverA, CL1199.Contig15\_Mf\_liverA, CL1199.Contig16\_Mf\_liverA, CL1199.Contig17\_Mf\_liverA, CL1199.Contig1\_Mf\_liverA, CL1199.Contig2\_Mf\_liverA, CL1199.Contig3\_Mf\_liverA, CL1199.Contig4\_Mf\_liverA, CL1365.Contig1\_Mf\_liverA, CL1370.Contig1\_Mf\_liverA, CL1370.Contig2\_Mf\_liverA, CL1370.Contig3\_Mf\_liverA, CL1370.Contig4\_Mf\_liverA, CL1380.Contig1\_Mf\_liverA, CL1381.Contig2\_Mf\_liverA, CL1381.Contig3\_Mf\_liverA, CL1410.Contig1\_Mf\_liverA, CL1410.Contig2\_Mf\_liverA, CL1410.Contig3\_Mf\_liverA, CL1410.Contig4\_Mf\_liverA, CL1437.Contig1\_Mf\_liverA, CL1437.Contig2\_Mf\_liverA, CL1437.Contig3\_Mf\_liverA, CL1486.Contig1\_Mf\_liverA, CL1486.Contig2\_Mf\_liverA, CL1486.Contig3\_Mf\_liverA, CL1571.Contig2\_Mf\_liverA, CL163.Contig1\_Mf\_liverA, CL1682.Contig1\_Mf\_liverA, CL1731.Contig1\_Mf\_liverA, CL1757.Contig1\_Mf\_liverA, CL1805.Contig1\_Mf\_liverA, CL1805.Contig2\_Mf\_liverA, CL1861.Contig1\_Mf\_liverA, CL1861.Contig2\_Mf\_liverA, CL1900.Contig1\_Mf\_liverA, CL1900.Contig2\_Mf\_liverA, CL1911.Contig1\_Mf\_liverA, CL1911.Contig2\_Mf\_liverA, CL194.Contig1\_Mf\_liverA, CL194.Contig2\_Mf\_liverA, CL1994.Contig1\_Mf\_liverA, CL1994.Contig2\_Mf\_liverA, CL2007.Contig1\_Mf\_liverA, CL2007.Contig2\_Mf\_liverA, CL2007.Contig3\_Mf\_liverA, CL2032.Contig1\_Mf\_liverA, CL2033.Contig3\_Mf\_liverA, CL2033.Contig4\_Mf\_liverA, CL2098.Contig1\_Mf\_liverA, CL210.Contig2\_Mf\_liverA, CL2116.Contig1\_Mf\_liverA, CL2116.Contig2\_Mf\_liverA, CL221.Contig1\_Mf\_liverA, CL221.Contig2\_Mf\_liverA, CL221.Contig3\_Mf\_liverA, CL221.Contig4\_Mf\_liverA, CL2285.Contig1\_Mf\_liverA, CL2285.Contig2\_Mf\_liverA, CL2285.Contig3\_Mf\_liverA, CL2307.Contig1\_Mf\_liverA, CL2307.Contig2\_Mf\_liverA, CL2339.Contig1\_Mf\_liverA, CL2339.Contig2\_Mf\_liverA, CL2343.Contig2\_Mf\_liverA, CL2381.Contig1\_Mf\_liverA, CL2381.Contig2\_Mf\_liverA, CL2381.Contig3\_Mf\_liverA, CL2381.Contig4\_Mf\_liverA, CL239.Contig2\_Mf\_liverA, CL2416.Contig1\_Mf\_liverA, CL2416.Contig2\_Mf\_liverA, CL2416.Contig3\_Mf\_liverA, CL243.Contig1\_Mf\_liverA, CL2546.Contig1\_Mf\_liverA, CL2546.Contig2\_Mf\_liverA, CL2557.Contig1\_Mf\_liverA, CL2557.Contig2\_Mf\_liverA, CL260.Contig1\_Mf\_liverA, CL260.Contig2\_Mf\_liverA, CL2620.Contig1\_Mf\_liverA, CL2692.Contig1\_Mf\_liverA, CL2692.Contig2\_Mf\_liverA, CL2692.Contig3\_Mf\_liverA, CL2693.Contig1\_Mf\_liverA, CL2693.Contig2\_Mf\_liverA, CL2764.Contig1\_Mf\_liverA, CL2764.Contig2\_Mf\_liverA, CL2764.Contig3\_Mf\_liverA, CL2764.Contig4\_Mf\_liverA, CL2768.Contig2\_Mf\_liverA, CL2845.Contig1\_Mf\_liverA, CL2845.Contig2\_Mf\_liverA, CL2863.Contig1\_Mf\_liverA, CL2863.Contig2\_Mf\_liverA, CL2876.Contig1\_Mf\_liverA, CL2887.Contig1\_Mf\_liverA, CL2887.Contig2\_Mf\_liverA, CL2900.Contig1\_Mf\_liverA, CL2900.Contig2\_Mf\_liverA, CL2954.Contig1\_Mf\_liverA, CL2954.Contig2\_Mf\_liverA, CL2954.Contig3\_Mf\_liverA, CL3116.Contig2\_Mf\_liverA, CL3143.Contig1\_Mf\_liverA, CL3213.Contig2\_Mf\_liverA, CL3215.Contig1\_Mf\_liverA, CL3215.Contig2\_Mf\_liverA, CL3220.Contig1\_Mf\_liverA, CL3220.Contig2\_Mf\_liverA, CL3220.Contig3\_Mf\_liverA, CL3220.Contig4\_Mf\_liverA, CL3276.Contig1\_Mf\_liverA, CL3276.Contig2\_Mf\_liverA, CL3276.Contig3\_Mf\_liverA, CL3316.Contig1\_Mf\_liverA, CL3316.Contig2\_Mf\_liverA, CL3349.Contig1\_Mf\_liverA, CL3349.Contig2\_Mf\_liverA, CL3416.Contig1\_Mf\_liverA, CL3416.Contig2\_Mf\_liverA, CL3631.Contig1\_Mf\_liverA, CL3631.Contig2\_Mf\_liverA, CL3787.Contig1\_Mf\_liverA, CL3858.Contig1\_Mf\_liverA, CL3898.Contig1\_Mf\_liverA, CL3898.Contig2\_Mf\_liverA, CL3928.Contig1\_Mf\_liverA, CL3928.Contig2\_Mf\_liverA, CL3958.Contig1\_Mf\_liverA, CL3958.Contig2\_Mf\_liverA, CL4086.Contig1\_Mf\_liverA, CL4086.Contig2\_Mf\_liverA, CL411.Contig1\_Mf\_liverA, CL4121.Contig1\_Mf\_liverA, CL4286.Contig1\_Mf\_liverA, CL4286.Contig2\_Mf\_liverA, CL4303.Contig1\_Mf\_liverA, CL4303.Contig2\_Mf\_liverA, CL433.Contig2\_Mf\_liverA, CL4335.Contig1\_Mf\_liverA, CL4335.Contig2\_Mf\_liverA, CL44.Contig1\_Mf\_liverA, CL44.Contig2\_Mf\_liverA, CL4409.Contig1\_Mf\_liverA, CL4507.Contig1\_Mf\_liverA, CL4605.Contig2\_Mf\_liverA, CL4610.Contig1\_Mf\_liverA, CL4610.Contig2\_Mf\_liverA, CL4618.Contig1\_Mf\_liverA, CL4618.Contig2\_Mf\_liverA, CL4643.Contig1\_Mf\_liverA, CL4643.Contig2\_Mf\_liverA, CL4643.Contig3\_Mf\_liverA, CL4643.Contig4\_Mf\_liverA, CL4643.Contig5\_Mf\_liverA, CL4643.Contig6\_Mf\_liverA, CL4644.Contig1\_Mf\_liverA, CL4644.Contig2\_Mf\_liverA, CL474.Contig1\_Mf\_liverA, CL4784.Contig2\_Mf\_liverA, CL4811.Contig1\_Mf\_liverA, CL4861.Contig1\_Mf\_liverA, CL4883.Contig2\_Mf\_liverA, CL4888.Contig1\_Mf\_liverA, CL4952.Contig1\_Mf\_liverA, CL4952.Contig2\_Mf\_liverA, CL4993.Contig1\_Mf\_liverA, CL4993.Contig2\_Mf\_liverA, CL5141.Contig1\_Mf\_liverA, CL5376.Contig1\_Mf\_liverA, CL5406.Contig1\_Mf\_liverA, CL5453.Contig2\_Mf\_liverA, CL550.Contig1\_Mf\_liverA, CL550.Contig2\_Mf\_liverA, CL5570.Contig1\_Mf\_liverA, CL5570.Contig2\_Mf\_liverA, CL5612.Contig2\_Mf\_liverA, CL5670.Contig2\_Mf\_liverA, CL5889.Contig2\_Mf\_liverA, CL5913.Contig1\_Mf\_liverA, CL5913.Contig2\_Mf\_liverA, CL628.Contig1\_Mf\_liverA, CL628.Contig4\_Mf\_liverA, CL628.Contig5\_Mf\_liverA, CL628.Contig6\_Mf\_liverA, CL628.Contig7\_Mf\_liverA, CL628.Contig8\_Mf\_liverA, CL628.Contig9\_Mf\_liverA, CL674.Contig1\_Mf\_liverA, CL708.Contig1\_Mf\_liverA, CL708.Contig4\_Mf\_liverA, CL708.Contig6\_Mf\_liverA, CL734.Contig1\_Mf\_liverA, CL734.Contig2\_Mf\_liverA, CL771.Contig1\_Mf\_liverA, CL771.Contig2\_Mf\_liverA, CL771.Contig3\_Mf\_liverA, CL771.Contig4\_Mf\_liverA, CL864.Contig2\_Mf\_liverA, CL864.Contig3\_Mf\_liverA, CL893.Contig1\_Mf\_liverA, CL893.Contig2\_Mf\_liverA, CL893.Contig3\_Mf\_liverA, CL893.Contig4\_Mf\_liverA, CL900.Contig1\_Mf\_liverA, CL932.Contig3\_Mf\_liverA, CL932.Contig5\_Mf\_liverA, CL932.Contig6\_Mf\_liverA, CL932.Contig9\_Mf\_liverA, CL97.Contig1\_Mf\_liverA, CL97.Contig2\_Mf\_liverA, CL98.Contig1\_Mf\_liverA, CL98.Contig2\_Mf\_liverA, CL98.Contig3\_Mf\_liverA, CL98.Contig4\_Mf\_liverA, Unigene1085\_Mf\_liverA, Unigene1088\_Mf\_liverA, Unigene11009\_Mf\_liverA, Unigene11662\_Mf\_liverA, Unigene11782\_Mf\_liverA, Unigene11803\_Mf\_liverA, Unigene11927\_Mf\_liverA, Unigene1212\_Mf\_liverA, Unigene12153\_Mf\_liverA, Unigene12262\_Mf\_liverA, Unigene12263\_Mf\_liverA, Unigene12503\_Mf\_liverA, Unigene12706\_Mf\_liverA, Unigene12877\_Mf\_liverA, Unigene12933\_Mf\_liverA, Unigene12993\_Mf\_liverA, Unigene13000\_Mf\_liverA, Unigene13001\_Mf\_liverA, Unigene13032\_Mf\_liverA, Unigene13062\_Mf\_liverA, Unigene13260\_Mf\_liverA, Unigene13261\_Mf\_liverA, Unigene13419\_Mf\_liverA, Unigene1363\_Mf\_liverA, Unigene13648\_Mf\_liverA, Unigene13779\_Mf\_liverA, Unigene13796\_Mf\_liverA, Unigene14050\_Mf\_liverA, Unigene14168\_Mf\_liverA, Unigene14190\_Mf\_liverA, Unigene14284\_Mf\_liverA, Unigene1448\_Mf\_liverA, Unigene14724\_Mf\_liverA, Unigene14807\_Mf\_liverA, Unigene14841\_Mf\_liverA, Unigene14908\_Mf\_liverA, Unigene14987\_Mf\_liverA, Unigene15074\_Mf\_liverA, Unigene15075\_Mf\_liverA, Unigene15076\_Mf\_liverA, Unigene15181\_Mf\_liverA, Unigene15295\_Mf\_liverA, Unigene15296\_Mf\_liverA, Unigene15352\_Mf\_liverA, Unigene15389\_Mf\_liverA, Unigene15855\_Mf\_liverA, Unigene15867\_Mf\_liverA, Unigene15874\_Mf\_liverA, Unigene15914\_Mf\_liverA, Unigene15940\_Mf\_liverA, Unigene16056\_Mf\_liverA, Unigene16062\_Mf\_liverA, Unigene16285\_Mf\_liverA, Unigene16849\_Mf\_liverA, Unigene16890\_Mf\_liverA, Unigene17313\_Mf\_liverA, Unigene17425\_Mf\_liverA, Unigene17426\_Mf\_liverA, Unigene17499\_Mf\_liverA, Unigene17518\_Mf\_liverA, Unigene17534\_Mf\_liverA, Unigene17571\_Mf\_liverA, Unigene17696\_Mf\_liverA, Unigene17760\_Mf\_liverA, Unigene17842\_Mf\_liverA, Unigene18177\_Mf\_liverA, Unigene18199\_Mf\_liverA, Unigene18326\_Mf\_liverA, Unigene18385\_Mf\_liverA, Unigene18655\_Mf\_liverA, Unigene18722\_Mf\_liverA, Unigene18723\_Mf\_liverA, Unigene19017\_Mf\_liverA, Unigene19044\_Mf\_liverA, Unigene19045\_Mf\_liverA, Unigene19092\_Mf\_liverA, Unigene19257\_Mf\_liverA, Unigene19258\_Mf\_liverA, Unigene19348\_Mf\_liverA, Unigene19414\_Mf\_liverA, Unigene19415\_Mf\_liverA, Unigene19658\_Mf\_liverA, Unigene19659\_Mf\_liverA, Unigene19893\_Mf\_liverA, Unigene19894\_Mf\_liverA, Unigene19973\_Mf\_liverA, Unigene19976\_Mf\_liverA, Unigene20249\_Mf\_liverA, Unigene20377\_Mf\_liverA, Unigene20378\_Mf\_liverA, Unigene20379\_Mf\_liverA, Unigene20394\_Mf\_liverA, Unigene20795\_Mf\_liverA, Unigene21693\_Mf\_liverA, Unigene21857\_Mf\_liverA, Unigene21905\_Mf\_liverA, Unigene21906\_Mf\_liverA, Unigene21991\_Mf\_liverA, Unigene22390\_Mf\_liverA, Unigene22611\_Mf\_liverA, Unigene22852\_Mf\_liverA, Unigene22977\_Mf\_liverA, Unigene22978\_Mf\_liverA, Unigene22979\_Mf\_liverA, Unigene22980\_Mf\_liverA, Unigene23067\_Mf\_liverA, Unigene23108\_Mf\_liverA, Unigene23129\_Mf\_liverA, Unigene23130\_Mf\_liverA, Unigene23158\_Mf\_liverA, Unigene23224\_Mf\_liverA, Unigene23328\_Mf\_liverA, Unigene23329\_Mf\_liverA, Unigene23330\_Mf\_liverA, Unigene23331\_Mf\_liverA, Unigene23334\_Mf\_liverA, Unigene2376\_Mf\_liverA, Unigene2377\_Mf\_liverA, Unigene23\_Mf\_liverA, Unigene24065\_Mf\_liverA, Unigene24086\_Mf\_liverA, Unigene24187\_Mf\_liverA, Unigene24255\_Mf\_liverA, Unigene2435\_Mf\_liverA, Unigene24468\_Mf\_liverA, Unigene24781\_Mf\_liverA, Unigene24804\_Mf\_liverA, Unigene24869\_Mf\_liverA, Unigene24912\_Mf\_liverA, Unigene24955\_Mf\_liverA, Unigene24956\_Mf\_liverA, Unigene24957\_Mf\_liverA, Unigene24958\_Mf\_liverA, Unigene24986\_Mf\_liverA, Unigene24987\_Mf\_liverA, Unigene24988\_Mf\_liverA, Unigene25135\_Mf\_liverA, Unigene25142\_Mf\_liverA, Unigene2521\_Mf\_liverA, Unigene25341\_Mf\_liverA, Unigene25364\_Mf\_liverA, Unigene25661\_Mf\_liverA, Unigene25662\_Mf\_liverA, Unigene25765\_Mf\_liverA, Unigene25853\_Mf\_liverA, Unigene26083\_Mf\_liverA, Unigene26183\_Mf\_liverA, Unigene26250\_Mf\_liverA, Unigene26273\_Mf\_liverA, Unigene26305\_Mf\_liverA, Unigene26306\_Mf\_liverA, Unigene26307\_Mf\_liverA, Unigene26923\_Mf\_liverA, Unigene27024\_Mf\_liverA, Unigene27068\_Mf\_liverA, Unigene27151\_Mf\_liverA, Unigene27217\_Mf\_liverA, Unigene27228\_Mf\_liverA, Unigene27281\_Mf\_liverA, Unigene27507\_Mf\_liverA, Unigene27951\_Mf\_liverA, Unigene28290\_Mf\_liverA, Unigene28296\_Mf\_liverA, Unigene28395\_Mf\_liverA, Unigene28396\_Mf\_liverA, Unigene28397\_Mf\_liverA, Unigene28398\_Mf\_liverA, Unigene28399\_Mf\_liverA, Unigene28610\_Mf\_liverA, Unigene28623\_Mf\_liverA, Unigene28624\_Mf\_liverA, Unigene28977\_Mf\_liverA, Unigene29126\_Mf\_liverA, Unigene2919\_Mf\_liverA, Unigene29269\_Mf\_liverA, Unigene29301\_Mf\_liverA, Unigene29315\_Mf\_liverA, Unigene29399\_Mf\_liverA, Unigene29467\_Mf\_liverA, Unigene29569\_Mf\_liverA, Unigene29617\_Mf\_liverA, Unigene30308\_Mf\_liverA, Unigene30346\_Mf\_liverA, Unigene30458\_Mf\_liverA, Unigene30459\_Mf\_liverA, Unigene30493\_Mf\_liverA, Unigene30494\_Mf\_liverA, Unigene30495\_Mf\_liverA, Unigene3053\_Mf\_liverA, Unigene30618\_Mf\_liverA, Unigene30619\_Mf\_liverA, Unigene30621\_Mf\_liverA, Unigene30771\_Mf\_liverA, Unigene30772\_Mf\_liverA, Unigene30877\_Mf\_liverA, Unigene31017\_Mf\_liverA, Unigene31276\_Mf\_liverA, Unigene31277\_Mf\_liverA, Unigene31278\_Mf\_liverA, Unigene31330\_Mf\_liverA, Unigene31333\_Mf\_liverA, Unigene31346\_Mf\_liverA, Unigene31382\_Mf\_liverA, Unigene31446\_Mf\_liverA, Unigene31897\_Mf\_liverA, Unigene32006\_Mf\_liverA, Unigene32090\_Mf\_liverA, Unigene32096\_Mf\_liverA, Unigene3215\_Mf\_liverA, Unigene32346\_Mf\_liverA, Unigene32559\_Mf\_liverA, Unigene32631\_Mf\_liverA, Unigene32879\_Mf\_liverA, Unigene32882\_Mf\_liverA, Unigene32883\_Mf\_liverA, Unigene32895\_Mf\_liverA, Unigene32987\_Mf\_liverA, Unigene33010\_Mf\_liverA, Unigene33012\_Mf\_liverA, Unigene33217\_Mf\_liverA, Unigene33218\_Mf\_liverA, Unigene33247\_Mf\_liverA, Unigene33597\_Mf\_liverA, Unigene33768\_Mf\_liverA, Unigene33772\_Mf\_liverA, Unigene34123\_Mf\_liverA, Unigene34124\_Mf\_liverA, Unigene34258\_Mf\_liverA, Unigene34847\_Mf\_liverA, Unigene35014\_Mf\_liverA, Unigene35199\_Mf\_liverA, Unigene35307\_Mf\_liverA, Unigene35441\_Mf\_liverA, Unigene35689\_Mf\_liverA, Unigene3572\_Mf\_liverA, Unigene35737\_Mf\_liverA, Unigene36385\_Mf\_liverA, Unigene3643\_Mf\_liverA, Unigene36801\_Mf\_liverA, Unigene36843\_Mf\_liverA, Unigene36911\_Mf\_liverA, Unigene37124\_Mf\_liverA, Unigene37591\_Mf\_liverA, Unigene37729\_Mf\_liverA, Unigene38219\_Mf\_liverA, Unigene38681\_Mf\_liverA, Unigene38994\_Mf\_liverA, Unigene389\_Mf\_liverA, Unigene39099\_Mf\_liverA, Unigene3915\_Mf\_liverA, Unigene3922\_Mf\_liverA, Unigene39816\_Mf\_liverA, Unigene39828\_Mf\_liverA, Unigene39990\_Mf\_liverA, Unigene40205\_Mf\_liverA, Unigene40253\_Mf\_liverA, Unigene40610\_Mf\_liverA, Unigene40962\_Mf\_liverA, Unigene40977\_Mf\_liverA, Unigene41087\_Mf\_liverA, Unigene41299\_Mf\_liverA, Unigene41315\_Mf\_liverA, Unigene41505\_Mf\_liverA, Unigene41613\_Mf\_liverA, Unigene41645\_Mf\_liverA, Unigene41724\_Mf\_liverA, Unigene42552\_Mf\_liverA, Unigene4264\_Mf\_liverA, Unigene4311\_Mf\_liverA, Unigene43599\_Mf\_liverA, Unigene43792\_Mf\_liverA, Unigene43912\_Mf\_liverA, Unigene44288\_Mf\_liverA, Unigene4459\_Mf\_liverA, Unigene44819\_Mf\_liverA, Unigene4513\_Mf\_liverA, Unigene45269\_Mf\_liverA, Unigene4543\_Mf\_liverA, Unigene45676\_Mf\_liverA, Unigene45703\_Mf\_liverA, Unigene45708\_Mf\_liverA, Unigene45730\_Mf\_liverA, Unigene46009\_Mf\_liverA, Unigene46368\_Mf\_liverA, Unigene46470\_Mf\_liverA, Unigene46649\_Mf\_liverA, Unigene4672\_Mf\_liverA, Unigene47212\_Mf\_liverA, Unigene47261\_Mf\_liverA, Unigene47634\_Mf\_liverA, Unigene47772\_Mf\_liverA, Unigene47837\_Mf\_liverA, Unigene47869\_Mf\_liverA, Unigene48285\_Mf\_liverA, Unigene48313\_Mf\_liverA, Unigene48526\_Mf\_liverA, Unigene48645\_Mf\_liverA, Unigene48671\_Mf\_liverA, Unigene486\_Mf\_liverA, Unigene4871\_Mf\_liverA, Unigene48762\_Mf\_liverA, Unigene4916\_Mf\_liverA, Unigene496\_Mf\_liverA, Unigene4983\_Mf\_liverA, Unigene49845\_Mf\_liverA, Unigene4985\_Mf\_liverA, Unigene49920\_Mf\_liverA, Unigene50319\_Mf\_liverA, Unigene50360\_Mf\_liverA, Unigene5134\_Mf\_liverA, Unigene5148\_Mf\_liverA, Unigene51616\_Mf\_liverA, Unigene5226\_Mf\_liverA, Unigene5245\_Mf\_liverA, Unigene5331\_Mf\_liverA, Unigene5381\_Mf\_liverA, Unigene5501\_Mf\_liverA, Unigene5606\_Mf\_liverA, Unigene5772\_Mf\_liverA, Unigene583\_Mf\_liverA, Unigene6063\_Mf\_liverA, Unigene6654\_Mf\_liverA, Unigene67\_Mf\_liverA, Unigene6959\_Mf\_liverA, Unigene7150\_Mf\_liverA, Unigene7204\_Mf\_liverA, Unigene7506\_Mf\_liverA, Unigene7514\_Mf\_liverA, Unigene7539\_Mf\_liverA, Unigene7612\_Mf\_liverA, Unigene766\_Mf\_liverA, Unigene8042\_Mf\_liverA, Unigene80\_Mf\_liverA, Unigene8199\_Mf\_liverA, Unigene8270\_Mf\_liverA, Unigene8360\_Mf\_liverA, Unigene851\_Mf\_liverA, Unigene8587\_Mf\_liverA, Unigene865\_Mf\_liverA, Unigene8718\_Mf\_liverA, Unigene88\_Mf\_liverA, Unigene9059\_Mf\_liverA, Unigene9060\_Mf\_liverA, Unigene919\_Mf\_liverA, Unigene9501\_Mf\_liverA, Unigene9577\_Mf\_liverA, Unigene9650\_Mf\_liverA, Unigene9762\_Mf\_liverA, Unigene9905\_Mf\_liverA |
| 11 | Epstein-Barr virus infection | CL1114.Contig1\_Mf\_liverA, CL1114.Contig2\_Mf\_liverA, CL1265.Contig1\_Mf\_liverA, CL1273.Contig1\_Mf\_liverA, CL1273.Contig3\_Mf\_liverA, CL1278.Contig1\_Mf\_liverA, CL1278.Contig2\_Mf\_liverA, CL1373.Contig1\_Mf\_liverA, CL1437.Contig1\_Mf\_liverA, CL1437.Contig2\_Mf\_liverA, CL149.Contig4\_Mf\_liverA, CL1500.Contig1\_Mf\_liverA, CL1500.Contig2\_Mf\_liverA, CL1543.Contig1\_Mf\_liverA, CL1543.Contig2\_Mf\_liverA, CL1543.Contig3\_Mf\_liverA, CL1684.Contig10\_Mf\_liverA, CL1684.Contig11\_Mf\_liverA, CL1684.Contig1\_Mf\_liverA, CL1684.Contig2\_Mf\_liverA, CL1684.Contig3\_Mf\_liverA, CL1684.Contig4\_Mf\_liverA, CL1684.Contig5\_Mf\_liverA, CL1684.Contig6\_Mf\_liverA, CL1684.Contig8\_Mf\_liverA, CL1684.Contig9\_Mf\_liverA, CL1685.Contig9\_Mf\_liverA, CL1687.Contig1\_Mf\_liverA, CL1687.Contig2\_Mf\_liverA, CL1743.Contig14\_Mf\_liverA, CL1743.Contig1\_Mf\_liverA, CL1743.Contig9\_Mf\_liverA, CL1752.Contig1\_Mf\_liverA, CL1752.Contig2\_Mf\_liverA, CL1752.Contig3\_Mf\_liverA, CL1752.Contig4\_Mf\_liverA, CL1805.Contig1\_Mf\_liverA, CL1805.Contig2\_Mf\_liverA, CL1856.Contig1\_Mf\_liverA, CL1856.Contig2\_Mf\_liverA, CL1860.Contig10\_Mf\_liverA, CL1860.Contig1\_Mf\_liverA, CL1860.Contig6\_Mf\_liverA, CL1860.Contig7\_Mf\_liverA, CL1861.Contig1\_Mf\_liverA, CL1861.Contig2\_Mf\_liverA, CL1949.Contig2\_Mf\_liverA, CL2033.Contig3\_Mf\_liverA, CL2033.Contig4\_Mf\_liverA, CL2069.Contig1\_Mf\_liverA, CL2069.Contig2\_Mf\_liverA, CL2069.Contig3\_Mf\_liverA, CL2069.Contig4\_Mf\_liverA, CL2163.Contig1\_Mf\_liverA, CL2163.Contig2\_Mf\_liverA, CL2174.Contig1\_Mf\_liverA, CL2176.Contig1\_Mf\_liverA, CL2176.Contig2\_Mf\_liverA, CL218.Contig1\_Mf\_liverA, CL218.Contig2\_Mf\_liverA, CL22.Contig1\_Mf\_liverA, CL22.Contig2\_Mf\_liverA, CL22.Contig5\_Mf\_liverA, CL2334.Contig1\_Mf\_liverA, CL2334.Contig2\_Mf\_liverA, CL2334.Contig3\_Mf\_liverA, CL2334.Contig4\_Mf\_liverA, CL2416.Contig1\_Mf\_liverA, CL2416.Contig2\_Mf\_liverA, CL2434.Contig1\_Mf\_liverA, CL2434.Contig2\_Mf\_liverA, CL2434.Contig3\_Mf\_liverA, CL2434.Contig4\_Mf\_liverA, CL2434.Contig5\_Mf\_liverA, CL2557.Contig1\_Mf\_liverA, CL2557.Contig2\_Mf\_liverA, CL2608.Contig2\_Mf\_liverA, CL2736.Contig1\_Mf\_liverA, CL2736.Contig2\_Mf\_liverA, CL2736.Contig3\_Mf\_liverA, CL275.Contig1\_Mf\_liverA, CL275.Contig2\_Mf\_liverA, CL275.Contig3\_Mf\_liverA, CL275.Contig4\_Mf\_liverA, CL275.Contig5\_Mf\_liverA, CL2764.Contig1\_Mf\_liverA, CL2764.Contig2\_Mf\_liverA, CL2764.Contig3\_Mf\_liverA, CL2764.Contig4\_Mf\_liverA, CL2861.Contig1\_Mf\_liverA, CL296.Contig1\_Mf\_liverA, CL296.Contig2\_Mf\_liverA, CL2993.Contig1\_Mf\_liverA, CL2993.Contig2\_Mf\_liverA, CL3002.Contig1\_Mf\_liverA, CL3053.Contig1\_Mf\_liverA, CL3063.Contig1\_Mf\_liverA, CL3081.Contig1\_Mf\_liverA, CL3143.Contig1\_Mf\_liverA, CL3147.Contig1\_Mf\_liverA, CL3173.Contig1\_Mf\_liverA, CL3173.Contig2\_Mf\_liverA, CL3267.Contig1\_Mf\_liverA, CL3267.Contig2\_Mf\_liverA, CL3270.Contig1\_Mf\_liverA, CL3270.Contig2\_Mf\_liverA, CL3349.Contig1\_Mf\_liverA, CL3349.Contig2\_Mf\_liverA, CL3398.Contig1\_Mf\_liverA, CL3398.Contig2\_Mf\_liverA, CL3616.Contig1\_Mf\_liverA, CL3616.Contig2\_Mf\_liverA, CL3623.Contig1\_Mf\_liverA, CL3623.Contig2\_Mf\_liverA, CL3623.Contig3\_Mf\_liverA, CL3631.Contig1\_Mf\_liverA, CL3631.Contig2\_Mf\_liverA, CL3800.Contig1\_Mf\_liverA, CL3800.Contig2\_Mf\_liverA, CL3804.Contig1\_Mf\_liverA, CL3804.Contig2\_Mf\_liverA, CL3814.Contig1\_Mf\_liverA, CL3814.Contig2\_Mf\_liverA, CL3832.Contig1\_Mf\_liverA, CL3832.Contig2\_Mf\_liverA, CL3835.Contig1\_Mf\_liverA, CL3835.Contig2\_Mf\_liverA, CL3916.Contig1\_Mf\_liverA, CL3916.Contig2\_Mf\_liverA, CL3941.Contig1\_Mf\_liverA, CL4008.Contig2\_Mf\_liverA, CL4038.Contig2\_Mf\_liverA, CL4086.Contig1\_Mf\_liverA, CL4086.Contig2\_Mf\_liverA, CL4106.Contig1\_Mf\_liverA, CL4106.Contig2\_Mf\_liverA, CL4149.Contig2\_Mf\_liverA, CL4149.Contig3\_Mf\_liverA, CL4154.Contig1\_Mf\_liverA, CL4154.Contig2\_Mf\_liverA, CL4160.Contig1\_Mf\_liverA, CL4160.Contig2\_Mf\_liverA, CL4249.Contig1\_Mf\_liverA, CL4249.Contig2\_Mf\_liverA, CL4304.Contig1\_Mf\_liverA, CL4364.Contig1\_Mf\_liverA, CL4364.Contig2\_Mf\_liverA, CL4409.Contig1\_Mf\_liverA, CL4414.Contig1\_Mf\_liverA, CL4414.Contig2\_Mf\_liverA, CL444.Contig1\_Mf\_liverA, CL444.Contig2\_Mf\_liverA, CL4450.Contig1\_Mf\_liverA, CL4462.Contig1\_Mf\_liverA, CL4462.Contig2\_Mf\_liverA, CL4487.Contig1\_Mf\_liverA, CL4487.Contig2\_Mf\_liverA, CL4513.Contig1\_Mf\_liverA, CL4513.Contig2\_Mf\_liverA, CL4558.Contig1\_Mf\_liverA, CL4558.Contig2\_Mf\_liverA, CL459.Contig1\_Mf\_liverA, CL459.Contig2\_Mf\_liverA, CL4653.Contig1\_Mf\_liverA, CL4722.Contig1\_Mf\_liverA, CL4722.Contig2\_Mf\_liverA, CL4725.Contig1\_Mf\_liverA, CL4725.Contig2\_Mf\_liverA, CL4757.Contig1\_Mf\_liverA, CL4757.Contig2\_Mf\_liverA, CL4784.Contig2\_Mf\_liverA, CL4888.Contig1\_Mf\_liverA, CL4919.Contig1\_Mf\_liverA, CL4919.Contig2\_Mf\_liverA, CL4988.Contig1\_Mf\_liverA, CL4988.Contig2\_Mf\_liverA, CL5018.Contig1\_Mf\_liverA, CL5018.Contig2\_Mf\_liverA, CL5329.Contig1\_Mf\_liverA, CL5329.Contig2\_Mf\_liverA, CL5336.Contig1\_Mf\_liverA, CL5336.Contig2\_Mf\_liverA, CL5405.Contig1\_Mf\_liverA, CL5405.Contig2\_Mf\_liverA, CL5416.Contig1\_Mf\_liverA, CL5416.Contig2\_Mf\_liverA, CL5486.Contig1\_Mf\_liverA, CL5486.Contig2\_Mf\_liverA, CL5549.Contig1\_Mf\_liverA, CL5549.Contig2\_Mf\_liverA, CL5552.Contig1\_Mf\_liverA, CL5552.Contig2\_Mf\_liverA, CL5614.Contig1\_Mf\_liverA, CL5614.Contig2\_Mf\_liverA, CL5713.Contig1\_Mf\_liverA, CL5713.Contig2\_Mf\_liverA, CL5771.Contig1\_Mf\_liverA, CL5771.Contig2\_Mf\_liverA, CL5891.Contig1\_Mf\_liverA, CL5891.Contig2\_Mf\_liverA, CL5947.Contig1\_Mf\_liverA, CL5947.Contig2\_Mf\_liverA, CL5950.Contig1\_Mf\_liverA, CL5950.Contig2\_Mf\_liverA, CL5950.Contig3\_Mf\_liverA, CL5955.Contig1\_Mf\_liverA, CL5970.Contig1\_Mf\_liverA, CL5970.Contig2\_Mf\_liverA, CL6034.Contig1\_Mf\_liverA, CL6034.Contig2\_Mf\_liverA, CL687.Contig11\_Mf\_liverA, CL687.Contig2\_Mf\_liverA, CL687.Contig4\_Mf\_liverA, CL687.Contig8\_Mf\_liverA, CL687.Contig9\_Mf\_liverA, CL771.Contig1\_Mf\_liverA, CL771.Contig2\_Mf\_liverA, CL771.Contig3\_Mf\_liverA, CL771.Contig4\_Mf\_liverA, CL777.Contig7\_Mf\_liverA, CL777.Contig8\_Mf\_liverA, CL830.Contig1\_Mf\_liverA, CL830.Contig2\_Mf\_liverA, CL830.Contig3\_Mf\_liverA, CL830.Contig4\_Mf\_liverA, CL830.Contig5\_Mf\_liverA, CL830.Contig6\_Mf\_liverA, CL830.Contig7\_Mf\_liverA, CL830.Contig8\_Mf\_liverA, CL839.Contig1\_Mf\_liverA, CL839.Contig2\_Mf\_liverA, CL874.Contig1\_Mf\_liverA, CL874.Contig2\_Mf\_liverA, CL874.Contig3\_Mf\_liverA, CL874.Contig4\_Mf\_liverA, CL874.Contig5\_Mf\_liverA, CL874.Contig6\_Mf\_liverA, CL890.Contig1\_Mf\_liverA, CL890.Contig2\_Mf\_liverA, CL928.Contig10\_Mf\_liverA, CL928.Contig11\_Mf\_liverA, CL928.Contig12\_Mf\_liverA, CL928.Contig13\_Mf\_liverA, CL928.Contig14\_Mf\_liverA, CL928.Contig15\_Mf\_liverA, CL928.Contig16\_Mf\_liverA, CL928.Contig17\_Mf\_liverA, CL928.Contig18\_Mf\_liverA, CL928.Contig19\_Mf\_liverA, CL928.Contig1\_Mf\_liverA, CL928.Contig20\_Mf\_liverA, CL928.Contig21\_Mf\_liverA, CL928.Contig22\_Mf\_liverA, CL928.Contig23\_Mf\_liverA, CL928.Contig24\_Mf\_liverA, CL928.Contig2\_Mf\_liverA, CL928.Contig3\_Mf\_liverA, CL928.Contig4\_Mf\_liverA, CL928.Contig5\_Mf\_liverA, CL928.Contig6\_Mf\_liverA, CL928.Contig7\_Mf\_liverA, CL928.Contig8\_Mf\_liverA, CL928.Contig9\_Mf\_liverA, CL932.Contig5\_Mf\_liverA, CL932.Contig6\_Mf\_liverA, CL932.Contig9\_Mf\_liverA, CL951.Contig1\_Mf\_liverA, CL951.Contig2\_Mf\_liverA, Unigene10029\_Mf\_liverA, Unigene10335\_Mf\_liverA, Unigene10438\_Mf\_liverA, Unigene10661\_Mf\_liverA, Unigene10820\_Mf\_liverA, Unigene11007\_Mf\_liverA, Unigene11008\_Mf\_liverA, Unigene11673\_Mf\_liverA, Unigene11782\_Mf\_liverA, Unigene11927\_Mf\_liverA, Unigene12538\_Mf\_liverA, Unigene12557\_Mf\_liverA, Unigene13379\_Mf\_liverA, Unigene13695\_Mf\_liverA, Unigene13961\_Mf\_liverA, Unigene13962\_Mf\_liverA, Unigene13981\_Mf\_liverA, Unigene14199\_Mf\_liverA, Unigene14200\_Mf\_liverA, Unigene14227\_Mf\_liverA, Unigene14699\_Mf\_liverA, Unigene14807\_Mf\_liverA, Unigene14870\_Mf\_liverA, Unigene14871\_Mf\_liverA, Unigene14907\_Mf\_liverA, Unigene14957\_Mf\_liverA, Unigene15085\_Mf\_liverA, Unigene15208\_Mf\_liverA, Unigene15295\_Mf\_liverA, Unigene15296\_Mf\_liverA, Unigene152\_Mf\_liverA, Unigene15344\_Mf\_liverA, Unigene15427\_Mf\_liverA, Unigene15845\_Mf\_liverA, Unigene15846\_Mf\_liverA, Unigene15867\_Mf\_liverA, Unigene15982\_Mf\_liverA, Unigene16321\_Mf\_liverA, Unigene16378\_Mf\_liverA, Unigene17518\_Mf\_liverA, Unigene17657\_Mf\_liverA, Unigene17754\_Mf\_liverA, Unigene18177\_Mf\_liverA, Unigene18567\_Mf\_liverA, Unigene1902\_Mf\_liverA, Unigene19297\_Mf\_liverA, Unigene19795\_Mf\_liverA, Unigene19812\_Mf\_liverA, Unigene19885\_Mf\_liverA, Unigene19893\_Mf\_liverA, Unigene19894\_Mf\_liverA, Unigene20070\_Mf\_liverA, Unigene20166\_Mf\_liverA, Unigene20842\_Mf\_liverA, Unigene20896\_Mf\_liverA, Unigene21284\_Mf\_liverA, Unigene21393\_Mf\_liverA, Unigene21541\_Mf\_liverA, Unigene21558\_Mf\_liverA, Unigene21560\_Mf\_liverA, Unigene21638\_Mf\_liverA, Unigene22427\_Mf\_liverA, Unigene22432\_Mf\_liverA, Unigene22433\_Mf\_liverA, Unigene22672\_Mf\_liverA, Unigene22919\_Mf\_liverA, Unigene231\_Mf\_liverA, Unigene23341\_Mf\_liverA, Unigene23484\_Mf\_liverA, Unigene24065\_Mf\_liverA, Unigene24111\_Mf\_liverA, Unigene24112\_Mf\_liverA, Unigene24160\_Mf\_liverA, Unigene24211\_Mf\_liverA, Unigene24212\_Mf\_liverA, Unigene24213\_Mf\_liverA, Unigene24246\_Mf\_liverA, Unigene24247\_Mf\_liverA, Unigene24509\_Mf\_liverA, Unigene24510\_Mf\_liverA, Unigene24804\_Mf\_liverA, Unigene25070\_Mf\_liverA, Unigene25142\_Mf\_liverA, Unigene2521\_Mf\_liverA, Unigene25341\_Mf\_liverA, Unigene25390\_Mf\_liverA, Unigene25404\_Mf\_liverA, Unigene25464\_Mf\_liverA, Unigene25504\_Mf\_liverA, Unigene25506\_Mf\_liverA, Unigene25677\_Mf\_liverA, Unigene25821\_Mf\_liverA, Unigene26079\_Mf\_liverA, Unigene26080\_Mf\_liverA, Unigene26081\_Mf\_liverA, Unigene26082\_Mf\_liverA, Unigene26117\_Mf\_liverA, Unigene26250\_Mf\_liverA, Unigene26267\_Mf\_liverA, Unigene26268\_Mf\_liverA, Unigene26305\_Mf\_liverA, Unigene26306\_Mf\_liverA, Unigene26307\_Mf\_liverA, Unigene26664\_Mf\_liverA, Unigene26665\_Mf\_liverA, Unigene27230\_Mf\_liverA, Unigene27231\_Mf\_liverA, Unigene27478\_Mf\_liverA, Unigene27553\_Mf\_liverA, Unigene27643\_Mf\_liverA, Unigene27748\_Mf\_liverA, Unigene27920\_Mf\_liverA, Unigene2798\_Mf\_liverA, Unigene28186\_Mf\_liverA, Unigene28244\_Mf\_liverA, Unigene28245\_Mf\_liverA, Unigene28290\_Mf\_liverA, Unigene28405\_Mf\_liverA, Unigene28481\_Mf\_liverA, Unigene28736\_Mf\_liverA, Unigene29076\_Mf\_liverA, Unigene29077\_Mf\_liverA, Unigene29078\_Mf\_liverA, Unigene29079\_Mf\_liverA, Unigene29129\_Mf\_liverA, Unigene29248\_Mf\_liverA, Unigene29725\_Mf\_liverA, Unigene29768\_Mf\_liverA, Unigene29829\_Mf\_liverA, Unigene29831\_Mf\_liverA, Unigene29849\_Mf\_liverA, Unigene29877\_Mf\_liverA, Unigene29878\_Mf\_liverA, Unigene30143\_Mf\_liverA, Unigene30289\_Mf\_liverA, Unigene30334\_Mf\_liverA, Unigene30340\_Mf\_liverA, Unigene30411\_Mf\_liverA, Unigene30412\_Mf\_liverA, Unigene30599\_Mf\_liverA, Unigene30600\_Mf\_liverA, Unigene30609\_Mf\_liverA, Unigene30610\_Mf\_liverA, Unigene30618\_Mf\_liverA, Unigene30619\_Mf\_liverA, Unigene30621\_Mf\_liverA, Unigene30643\_Mf\_liverA, Unigene30878\_Mf\_liverA, Unigene30879\_Mf\_liverA, Unigene30926\_Mf\_liverA, Unigene30927\_Mf\_liverA, Unigene31010\_Mf\_liverA, Unigene31011\_Mf\_liverA, Unigene31022\_Mf\_liverA, Unigene31196\_Mf\_liverA, Unigene31521\_Mf\_liverA, Unigene31583\_Mf\_liverA, Unigene31666\_Mf\_liverA, Unigene31687\_Mf\_liverA, Unigene31688\_Mf\_liverA, Unigene31691\_Mf\_liverA, Unigene31692\_Mf\_liverA, Unigene31822\_Mf\_liverA, Unigene31824\_Mf\_liverA, Unigene32002\_Mf\_liverA, Unigene32003\_Mf\_liverA, Unigene32170\_Mf\_liverA, Unigene32228\_Mf\_liverA, Unigene32229\_Mf\_liverA, Unigene32338\_Mf\_liverA, Unigene32357\_Mf\_liverA, Unigene32402\_Mf\_liverA, Unigene32568\_Mf\_liverA, Unigene32810\_Mf\_liverA, Unigene32852\_Mf\_liverA, Unigene32879\_Mf\_liverA, Unigene32882\_Mf\_liverA, Unigene32883\_Mf\_liverA, Unigene33039\_Mf\_liverA, Unigene33122\_Mf\_liverA, Unigene33353\_Mf\_liverA, Unigene33853\_Mf\_liverA, Unigene33854\_Mf\_liverA, Unigene34446\_Mf\_liverA, Unigene34588\_Mf\_liverA, Unigene34707\_Mf\_liverA, Unigene34711\_Mf\_liverA, Unigene34746\_Mf\_liverA, Unigene34777\_Mf\_liverA, Unigene35147\_Mf\_liverA, Unigene35198\_Mf\_liverA, Unigene35276\_Mf\_liverA, Unigene35335\_Mf\_liverA, Unigene35336\_Mf\_liverA, Unigene35338\_Mf\_liverA, Unigene35443\_Mf\_liverA, Unigene35444\_Mf\_liverA, Unigene35455\_Mf\_liverA, Unigene35457\_Mf\_liverA, Unigene35612\_Mf\_liverA, Unigene35761\_Mf\_liverA, Unigene35816\_Mf\_liverA, Unigene36049\_Mf\_liverA, Unigene36288\_Mf\_liverA, Unigene36289\_Mf\_liverA, Unigene36413\_Mf\_liverA, Unigene36539\_Mf\_liverA, Unigene36543\_Mf\_liverA, Unigene36623\_Mf\_liverA, Unigene36701\_Mf\_liverA, Unigene36793\_Mf\_liverA, Unigene36829\_Mf\_liverA, Unigene36839\_Mf\_liverA, Unigene36849\_Mf\_liverA, Unigene36901\_Mf\_liverA, Unigene36986\_Mf\_liverA, Unigene37003\_Mf\_liverA, Unigene37025\_Mf\_liverA, Unigene37075\_Mf\_liverA, Unigene37076\_Mf\_liverA, Unigene37082\_Mf\_liverA, Unigene37170\_Mf\_liverA, Unigene37245\_Mf\_liverA, Unigene37282\_Mf\_liverA, Unigene37350\_Mf\_liverA, Unigene37358\_Mf\_liverA, Unigene37366\_Mf\_liverA, Unigene37558\_Mf\_liverA, Unigene37566\_Mf\_liverA, Unigene37744\_Mf\_liverA, Unigene38010\_Mf\_liverA, Unigene38042\_Mf\_liverA, Unigene38514\_Mf\_liverA, Unigene38616\_Mf\_liverA, Unigene38630\_Mf\_liverA, Unigene38681\_Mf\_liverA, Unigene38685\_Mf\_liverA, Unigene38925\_Mf\_liverA, Unigene39035\_Mf\_liverA, Unigene39749\_Mf\_liverA, Unigene40304\_Mf\_liverA, Unigene40644\_Mf\_liverA, Unigene41082\_Mf\_liverA, Unigene41724\_Mf\_liverA, Unigene42335\_Mf\_liverA, Unigene42830\_Mf\_liverA, Unigene4311\_Mf\_liverA, Unigene43198\_Mf\_liverA, Unigene43855\_Mf\_liverA, Unigene43900\_Mf\_liverA, Unigene44067\_Mf\_liverA, Unigene4459\_Mf\_liverA, Unigene45166\_Mf\_liverA, Unigene4565\_Mf\_liverA, Unigene45708\_Mf\_liverA, Unigene46319\_Mf\_liverA, Unigene4681\_Mf\_liverA, Unigene47448\_Mf\_liverA, Unigene47837\_Mf\_liverA, Unigene48052\_Mf\_liverA, Unigene48462\_Mf\_liverA, Unigene4848\_Mf\_liverA, Unigene48506\_Mf\_liverA, Unigene48539\_Mf\_liverA, Unigene4893\_Mf\_liverA, Unigene49035\_Mf\_liverA, Unigene4938\_Mf\_liverA, Unigene50099\_Mf\_liverA, Unigene502\_Mf\_liverA, Unigene50319\_Mf\_liverA, Unigene50332\_Mf\_liverA, Unigene50360\_Mf\_liverA, Unigene50600\_Mf\_liverA, Unigene51426\_Mf\_liverA, Unigene5165\_Mf\_liverA, Unigene5185\_Mf\_liverA, Unigene5186\_Mf\_liverA, Unigene5219\_Mf\_liverA, Unigene5226\_Mf\_liverA, Unigene5303\_Mf\_liverA, Unigene534\_Mf\_liverA, Unigene5370\_Mf\_liverA, Unigene5380\_Mf\_liverA, Unigene5453\_Mf\_liverA, Unigene550\_Mf\_liverA, Unigene5585\_Mf\_liverA, Unigene5702\_Mf\_liverA, Unigene5762\_Mf\_liverA, Unigene5763\_Mf\_liverA, Unigene583\_Mf\_liverA, Unigene6560\_Mf\_liverA, Unigene6561\_Mf\_liverA, Unigene663\_Mf\_liverA, Unigene6852\_Mf\_liverA, Unigene690\_Mf\_liverA, Unigene742\_Mf\_liverA, Unigene7567\_Mf\_liverA, Unigene7729\_Mf\_liverA, Unigene7730\_Mf\_liverA, Unigene7734\_Mf\_liverA, Unigene8624\_Mf\_liverA, Unigene897\_Mf\_liverA, Unigene898\_Mf\_liverA, Unigene9762\_Mf\_liverA |
| 12 | Herpes simplex infection | CL1033.Contig1\_Mf\_liverA, CL1033.Contig4\_Mf\_liverA, CL1066.Contig1\_Mf\_liverA, CL1211.Contig1\_Mf\_liverA, CL1211.Contig2\_Mf\_liverA, CL1211.Contig3\_Mf\_liverA, CL124.Contig1\_Mf\_liverA, CL124.Contig2\_Mf\_liverA, CL1266.Contig2\_Mf\_liverA, CL1460.Contig1\_Mf\_liverA, CL1460.Contig2\_Mf\_liverA, CL149.Contig4\_Mf\_liverA, CL1497.Contig1\_Mf\_liverA, CL1497.Contig2\_Mf\_liverA, CL1529.Contig1\_Mf\_liverA, CL1529.Contig2\_Mf\_liverA, CL1529.Contig3\_Mf\_liverA, CL1552.Contig1\_Mf\_liverA, CL1582.Contig1\_Mf\_liverA, CL1582.Contig2\_Mf\_liverA, CL1582.Contig3\_Mf\_liverA, CL1626.Contig2\_Mf\_liverA, CL1653.Contig1\_Mf\_liverA, CL1653.Contig2\_Mf\_liverA, CL1673.Contig1\_Mf\_liverA, CL1673.Contig2\_Mf\_liverA, CL1673.Contig3\_Mf\_liverA, CL1673.Contig4\_Mf\_liverA, CL1673.Contig5\_Mf\_liverA, CL1673.Contig6\_Mf\_liverA, CL1684.Contig10\_Mf\_liverA, CL1684.Contig11\_Mf\_liverA, CL1684.Contig1\_Mf\_liverA, CL1684.Contig2\_Mf\_liverA, CL1684.Contig3\_Mf\_liverA, CL1684.Contig4\_Mf\_liverA, CL1684.Contig5\_Mf\_liverA, CL1684.Contig6\_Mf\_liverA, CL1684.Contig8\_Mf\_liverA, CL1684.Contig9\_Mf\_liverA, CL1685.Contig9\_Mf\_liverA, CL1687.Contig1\_Mf\_liverA, CL1687.Contig2\_Mf\_liverA, CL1810.Contig1\_Mf\_liverA, CL1810.Contig2\_Mf\_liverA, CL1860.Contig10\_Mf\_liverA, CL1860.Contig1\_Mf\_liverA, CL1860.Contig6\_Mf\_liverA, CL1860.Contig7\_Mf\_liverA, CL19.Contig1\_Mf\_liverA, CL19.Contig2\_Mf\_liverA, CL19.Contig3\_Mf\_liverA, CL19.Contig4\_Mf\_liverA, CL19.Contig5\_Mf\_liverA, CL1939.Contig1\_Mf\_liverA, CL1939.Contig2\_Mf\_liverA, CL1939.Contig3\_Mf\_liverA, CL1939.Contig4\_Mf\_liverA, CL1999.Contig3\_Mf\_liverA, CL1999.Contig4\_Mf\_liverA, CL2000.Contig1\_Mf\_liverA, CL2163.Contig1\_Mf\_liverA, CL2163.Contig2\_Mf\_liverA, CL2188.Contig1\_Mf\_liverA, CL2188.Contig2\_Mf\_liverA, CL2188.Contig3\_Mf\_liverA, CL2188.Contig4\_Mf\_liverA, CL2188.Contig5\_Mf\_liverA, CL2188.Contig6\_Mf\_liverA, CL2212.Contig1\_Mf\_liverA, CL2212.Contig2\_Mf\_liverA, CL2294.Contig1\_Mf\_liverA, CL2325.Contig1\_Mf\_liverA, CL2325.Contig2\_Mf\_liverA, CL2329.Contig1\_Mf\_liverA, CL2329.Contig2\_Mf\_liverA, CL2416.Contig1\_Mf\_liverA, CL2416.Contig2\_Mf\_liverA, CL2434.Contig1\_Mf\_liverA, CL2434.Contig2\_Mf\_liverA, CL2434.Contig3\_Mf\_liverA, CL2434.Contig4\_Mf\_liverA, CL2434.Contig5\_Mf\_liverA, CL2549.Contig1\_Mf\_liverA, CL2549.Contig2\_Mf\_liverA, CL2616.Contig1\_Mf\_liverA, CL2687.Contig1\_Mf\_liverA, CL2687.Contig2\_Mf\_liverA, CL271.Contig1\_Mf\_liverA, CL271.Contig2\_Mf\_liverA, CL2736.Contig1\_Mf\_liverA, CL2736.Contig2\_Mf\_liverA, CL2736.Contig3\_Mf\_liverA, CL275.Contig1\_Mf\_liverA, CL275.Contig2\_Mf\_liverA, CL275.Contig3\_Mf\_liverA, CL275.Contig4\_Mf\_liverA, CL275.Contig5\_Mf\_liverA, CL2757.Contig1\_Mf\_liverA, CL2757.Contig2\_Mf\_liverA, CL2826.Contig1\_Mf\_liverA, CL2826.Contig3\_Mf\_liverA, CL2847.Contig1\_Mf\_liverA, CL2847.Contig2\_Mf\_liverA, CL2855.Contig1\_Mf\_liverA, CL2855.Contig2\_Mf\_liverA, CL2861.Contig1\_Mf\_liverA, CL2895.Contig1\_Mf\_liverA, CL2895.Contig2\_Mf\_liverA, CL296.Contig1\_Mf\_liverA, CL296.Contig2\_Mf\_liverA, CL2978.Contig1\_Mf\_liverA, CL2978.Contig2\_Mf\_liverA, CL2993.Contig1\_Mf\_liverA, CL2993.Contig2\_Mf\_liverA, CL3002.Contig1\_Mf\_liverA, CL3013.Contig1\_Mf\_liverA, CL3013.Contig2\_Mf\_liverA, CL3053.Contig1\_Mf\_liverA, CL306.Contig1\_Mf\_liverA, CL306.Contig2\_Mf\_liverA, CL3063.Contig1\_Mf\_liverA, CL3223.Contig2\_Mf\_liverA, CL3253.Contig1\_Mf\_liverA, CL3253.Contig2\_Mf\_liverA, CL3253.Contig3\_Mf\_liverA, CL3253.Contig4\_Mf\_liverA, CL3267.Contig1\_Mf\_liverA, CL3267.Contig2\_Mf\_liverA, CL3284.Contig1\_Mf\_liverA, CL3284.Contig2\_Mf\_liverA, CL3284.Contig3\_Mf\_liverA, CL3349.Contig1\_Mf\_liverA, CL3349.Contig2\_Mf\_liverA, CL3398.Contig1\_Mf\_liverA, CL3398.Contig2\_Mf\_liverA, CL3431.Contig1\_Mf\_liverA, CL3463.Contig1\_Mf\_liverA, CL3463.Contig2\_Mf\_liverA, CL3463.Contig3\_Mf\_liverA, CL3465.Contig2\_Mf\_liverA, CL3468.Contig1\_Mf\_liverA, CL3468.Contig2\_Mf\_liverA, CL3483.Contig1\_Mf\_liverA, CL3567.Contig1\_Mf\_liverA, CL3567.Contig2\_Mf\_liverA, CL3581.Contig1\_Mf\_liverA, CL3581.Contig2\_Mf\_liverA, CL3616.Contig1\_Mf\_liverA, CL3616.Contig2\_Mf\_liverA, CL3623.Contig1\_Mf\_liverA, CL3623.Contig2\_Mf\_liverA, CL3623.Contig3\_Mf\_liverA, CL3756.Contig1\_Mf\_liverA, CL3756.Contig2\_Mf\_liverA, CL3756.Contig3\_Mf\_liverA, CL3756.Contig4\_Mf\_liverA, CL3775.Contig1\_Mf\_liverA, CL3775.Contig2\_Mf\_liverA, CL3800.Contig1\_Mf\_liverA, CL3800.Contig2\_Mf\_liverA, CL3814.Contig1\_Mf\_liverA, CL3814.Contig2\_Mf\_liverA, CL3822.Contig1\_Mf\_liverA, CL3822.Contig2\_Mf\_liverA, CL3832.Contig1\_Mf\_liverA, CL3832.Contig2\_Mf\_liverA, CL3835.Contig1\_Mf\_liverA, CL3835.Contig2\_Mf\_liverA, CL3841.Contig1\_Mf\_liverA, CL3841.Contig2\_Mf\_liverA, CL3930.Contig1\_Mf\_liverA, CL3930.Contig2\_Mf\_liverA, CL3942.Contig1\_Mf\_liverA, CL3942.Contig2\_Mf\_liverA, CL3963.Contig1\_Mf\_liverA, CL3963.Contig2\_Mf\_liverA, CL3975.Contig2\_Mf\_liverA, CL4039.Contig1\_Mf\_liverA, CL4039.Contig2\_Mf\_liverA, CL4106.Contig1\_Mf\_liverA, CL4106.Contig2\_Mf\_liverA, CL4148.Contig1\_Mf\_liverA, CL4170.Contig1\_Mf\_liverA, CL4170.Contig2\_Mf\_liverA, CL4183.Contig2\_Mf\_liverA, CL4244.Contig1\_Mf\_liverA, CL4248.Contig1\_Mf\_liverA, CL4248.Contig2\_Mf\_liverA, CL4304.Contig1\_Mf\_liverA, CL4364.Contig1\_Mf\_liverA, CL4364.Contig2\_Mf\_liverA, CL438.Contig1\_Mf\_liverA, CL439.Contig2\_Mf\_liverA, CL439.Contig3\_Mf\_liverA, CL439.Contig4\_Mf\_liverA, CL44.Contig1\_Mf\_liverA, CL44.Contig2\_Mf\_liverA, CL4427.Contig1\_Mf\_liverA, CL4427.Contig2\_Mf\_liverA, CL4450.Contig1\_Mf\_liverA, CL447.Contig33\_Mf\_liverA, CL4475.Contig1\_Mf\_liverA, CL4558.Contig1\_Mf\_liverA, CL4558.Contig2\_Mf\_liverA, CL459.Contig1\_Mf\_liverA, CL459.Contig2\_Mf\_liverA, CL462.Contig5\_Mf\_liverA, CL4757.Contig1\_Mf\_liverA, CL4757.Contig2\_Mf\_liverA, CL4770.Contig1\_Mf\_liverA, CL4770.Contig2\_Mf\_liverA, CL479.Contig1\_Mf\_liverA, CL479.Contig2\_Mf\_liverA, CL4820.Contig1\_Mf\_liverA, CL4820.Contig2\_Mf\_liverA, CL4841.Contig1\_Mf\_liverA, CL4841.Contig2\_Mf\_liverA, CL4919.Contig1\_Mf\_liverA, CL4919.Contig2\_Mf\_liverA, CL4945.Contig1\_Mf\_liverA, CL4945.Contig2\_Mf\_liverA, CL4962.Contig1\_Mf\_liverA, CL4962.Contig2\_Mf\_liverA, CL5018.Contig1\_Mf\_liverA, CL5018.Contig2\_Mf\_liverA, CL5033.Contig1\_Mf\_liverA, CL5033.Contig2\_Mf\_liverA, CL5083.Contig1\_Mf\_liverA, CL5083.Contig2\_Mf\_liverA, CL510.Contig1\_Mf\_liverA, CL510.Contig2\_Mf\_liverA, CL5256.Contig2\_Mf\_liverA, CL5405.Contig1\_Mf\_liverA, CL5405.Contig2\_Mf\_liverA, CL5538.Contig1\_Mf\_liverA, CL5538.Contig2\_Mf\_liverA, CL5549.Contig1\_Mf\_liverA, CL5549.Contig2\_Mf\_liverA, CL5552.Contig1\_Mf\_liverA, CL5552.Contig2\_Mf\_liverA, CL5554.Contig1\_Mf\_liverA, CL5554.Contig2\_Mf\_liverA, CL5614.Contig1\_Mf\_liverA, CL5614.Contig2\_Mf\_liverA, CL5630.Contig1\_Mf\_liverA, CL5630.Contig2\_Mf\_liverA, CL5674.Contig1\_Mf\_liverA, CL5674.Contig2\_Mf\_liverA, CL5674.Contig3\_Mf\_liverA, CL575.Contig1\_Mf\_liverA, CL5891.Contig2\_Mf\_liverA, CL6034.Contig1\_Mf\_liverA, CL6034.Contig2\_Mf\_liverA, CL618.Contig1\_Mf\_liverA, CL618.Contig2\_Mf\_liverA, CL618.Contig3\_Mf\_liverA, CL618.Contig4\_Mf\_liverA, CL624.Contig1\_Mf\_liverA, CL624.Contig2\_Mf\_liverA, CL624.Contig3\_Mf\_liverA, CL741.Contig10\_Mf\_liverA, CL741.Contig3\_Mf\_liverA, CL741.Contig7\_Mf\_liverA, CL741.Contig8\_Mf\_liverA, CL741.Contig9\_Mf\_liverA, CL808.Contig1\_Mf\_liverA, CL808.Contig2\_Mf\_liverA, CL808.Contig3\_Mf\_liverA, CL874.Contig1\_Mf\_liverA, CL874.Contig2\_Mf\_liverA, CL874.Contig3\_Mf\_liverA, CL874.Contig4\_Mf\_liverA, CL874.Contig5\_Mf\_liverA, CL874.Contig6\_Mf\_liverA, CL890.Contig1\_Mf\_liverA, CL890.Contig2\_Mf\_liverA, CL932.Contig5\_Mf\_liverA, CL932.Contig6\_Mf\_liverA, CL932.Contig9\_Mf\_liverA, CL951.Contig1\_Mf\_liverA, CL951.Contig2\_Mf\_liverA, Unigene10112\_Mf\_liverA, Unigene1018\_Mf\_liverA, Unigene10335\_Mf\_liverA, Unigene10438\_Mf\_liverA, Unigene10661\_Mf\_liverA, Unigene10724\_Mf\_liverA, Unigene10954\_Mf\_liverA, Unigene11183\_Mf\_liverA, Unigene11782\_Mf\_liverA, Unigene12048\_Mf\_liverA, Unigene12669\_Mf\_liverA, Unigene12743\_Mf\_liverA, Unigene12989\_Mf\_liverA, Unigene13249\_Mf\_liverA, Unigene13250\_Mf\_liverA, Unigene1326\_Mf\_liverA, Unigene13450\_Mf\_liverA, Unigene13597\_Mf\_liverA, Unigene13695\_Mf\_liverA, Unigene13796\_Mf\_liverA, Unigene13894\_Mf\_liverA, Unigene13981\_Mf\_liverA, Unigene14212\_Mf\_liverA, Unigene14253\_Mf\_liverA, Unigene14329\_Mf\_liverA, Unigene14377\_Mf\_liverA, Unigene14378\_Mf\_liverA, Unigene14514\_Mf\_liverA, Unigene14515\_Mf\_liverA, Unigene14588\_Mf\_liverA, Unigene14924\_Mf\_liverA, Unigene15001\_Mf\_liverA, Unigene15064\_Mf\_liverA, Unigene15138\_Mf\_liverA, Unigene15427\_Mf\_liverA, Unigene15678\_Mf\_liverA, Unigene15845\_Mf\_liverA, Unigene15846\_Mf\_liverA, Unigene15940\_Mf\_liverA, Unigene15961\_Mf\_liverA, Unigene16284\_Mf\_liverA, Unigene16378\_Mf\_liverA, Unigene16462\_Mf\_liverA, Unigene16757\_Mf\_liverA, Unigene16819\_Mf\_liverA, Unigene16820\_Mf\_liverA, Unigene16965\_Mf\_liverA, Unigene17208\_Mf\_liverA, Unigene17754\_Mf\_liverA, Unigene17903\_Mf\_liverA, Unigene18199\_Mf\_liverA, Unigene18467\_Mf\_liverA, Unigene18848\_Mf\_liverA, Unigene1902\_Mf\_liverA, Unigene19451\_Mf\_liverA, Unigene19452\_Mf\_liverA, Unigene19795\_Mf\_liverA, Unigene19797\_Mf\_liverA, Unigene20315\_Mf\_liverA, Unigene20340\_Mf\_liverA, Unigene20957\_Mf\_liverA, Unigene20958\_Mf\_liverA, Unigene21284\_Mf\_liverA, Unigene21423\_Mf\_liverA, Unigene21594\_Mf\_liverA, Unigene21664\_Mf\_liverA, Unigene21665\_Mf\_liverA, Unigene21717\_Mf\_liverA, Unigene21958\_Mf\_liverA, Unigene21959\_Mf\_liverA, Unigene2241\_Mf\_liverA, Unigene22652\_Mf\_liverA, Unigene226\_Mf\_liverA, Unigene231\_Mf\_liverA, Unigene23445\_Mf\_liverA, Unigene23589\_Mf\_liverA, Unigene23791\_Mf\_liverA, Unigene24160\_Mf\_liverA, Unigene241\_Mf\_liverA, Unigene24257\_Mf\_liverA, Unigene24549\_Mf\_liverA, Unigene24551\_Mf\_liverA, Unigene24606\_Mf\_liverA, Unigene25404\_Mf\_liverA, Unigene25416\_Mf\_liverA, Unigene25464\_Mf\_liverA, Unigene25504\_Mf\_liverA, Unigene25506\_Mf\_liverA, Unigene25821\_Mf\_liverA, Unigene26079\_Mf\_liverA, Unigene26080\_Mf\_liverA, Unigene26081\_Mf\_liverA, Unigene26082\_Mf\_liverA, Unigene26100\_Mf\_liverA, Unigene26117\_Mf\_liverA, Unigene26131\_Mf\_liverA, Unigene26132\_Mf\_liverA, Unigene26664\_Mf\_liverA, Unigene26665\_Mf\_liverA, Unigene26817\_Mf\_liverA, Unigene2721\_Mf\_liverA, Unigene27224\_Mf\_liverA, Unigene27225\_Mf\_liverA, Unigene27339\_Mf\_liverA, Unigene27366\_Mf\_liverA, Unigene273\_Mf\_liverA, Unigene27553\_Mf\_liverA, Unigene27643\_Mf\_liverA, Unigene27778\_Mf\_liverA, Unigene27779\_Mf\_liverA, Unigene27780\_Mf\_liverA, Unigene27895\_Mf\_liverA, Unigene2798\_Mf\_liverA, Unigene28085\_Mf\_liverA, Unigene28086\_Mf\_liverA, Unigene28197\_Mf\_liverA, Unigene28244\_Mf\_liverA, Unigene28245\_Mf\_liverA, Unigene28277\_Mf\_liverA, Unigene28278\_Mf\_liverA, Unigene28341\_Mf\_liverA, Unigene28374\_Mf\_liverA, Unigene28375\_Mf\_liverA, Unigene28609\_Mf\_liverA, Unigene28623\_Mf\_liverA, Unigene28624\_Mf\_liverA, Unigene28980\_Mf\_liverA, Unigene28981\_Mf\_liverA, Unigene29076\_Mf\_liverA, Unigene29077\_Mf\_liverA, Unigene29129\_Mf\_liverA, Unigene29231\_Mf\_liverA, Unigene29248\_Mf\_liverA, Unigene29427\_Mf\_liverA, Unigene29490\_Mf\_liverA, Unigene29492\_Mf\_liverA, Unigene29617\_Mf\_liverA, Unigene29725\_Mf\_liverA, Unigene29829\_Mf\_liverA, Unigene29831\_Mf\_liverA, Unigene29849\_Mf\_liverA, Unigene30453\_Mf\_liverA, Unigene30563\_Mf\_liverA, Unigene30878\_Mf\_liverA, Unigene30879\_Mf\_liverA, Unigene31010\_Mf\_liverA, Unigene31011\_Mf\_liverA, Unigene31196\_Mf\_liverA, Unigene31273\_Mf\_liverA, Unigene31521\_Mf\_liverA, Unigene31570\_Mf\_liverA, Unigene31687\_Mf\_liverA, Unigene31688\_Mf\_liverA, Unigene31691\_Mf\_liverA, Unigene31692\_Mf\_liverA, Unigene31845\_Mf\_liverA, Unigene31846\_Mf\_liverA, Unigene32357\_Mf\_liverA, Unigene32432\_Mf\_liverA, Unigene32511\_Mf\_liverA, Unigene32512\_Mf\_liverA, Unigene32568\_Mf\_liverA, Unigene32676\_Mf\_liverA, Unigene32724\_Mf\_liverA, Unigene32879\_Mf\_liverA, Unigene32882\_Mf\_liverA, Unigene32883\_Mf\_liverA, Unigene32922\_Mf\_liverA, Unigene32923\_Mf\_liverA, Unigene33122\_Mf\_liverA, Unigene332\_Mf\_liverA, Unigene33316\_Mf\_liverA, Unigene33401\_Mf\_liverA, Unigene33646\_Mf\_liverA, Unigene33768\_Mf\_liverA, Unigene34014\_Mf\_liverA, Unigene34052\_Mf\_liverA, Unigene34183\_Mf\_liverA, Unigene34707\_Mf\_liverA, Unigene34711\_Mf\_liverA, Unigene35210\_Mf\_liverA, Unigene35211\_Mf\_liverA, Unigene35335\_Mf\_liverA, Unigene35336\_Mf\_liverA, Unigene35338\_Mf\_liverA, Unigene35417\_Mf\_liverA, Unigene35423\_Mf\_liverA, Unigene35498\_Mf\_liverA, Unigene35612\_Mf\_liverA, Unigene35822\_Mf\_liverA, Unigene35996\_Mf\_liverA, Unigene36414\_Mf\_liverA, Unigene36417\_Mf\_liverA, Unigene36418\_Mf\_liverA, Unigene36420\_Mf\_liverA, Unigene364\_Mf\_liverA, Unigene36515\_Mf\_liverA, Unigene36516\_Mf\_liverA, Unigene36517\_Mf\_liverA, Unigene36518\_Mf\_liverA, Unigene36519\_Mf\_liverA, Unigene36520\_Mf\_liverA, Unigene36521\_Mf\_liverA, Unigene36522\_Mf\_liverA, Unigene36523\_Mf\_liverA, Unigene36526\_Mf\_liverA, Unigene36527\_Mf\_liverA, Unigene36539\_Mf\_liverA, Unigene36543\_Mf\_liverA, Unigene36667\_Mf\_liverA, Unigene36692\_Mf\_liverA, Unigene36779\_Mf\_liverA, Unigene36849\_Mf\_liverA, Unigene36871\_Mf\_liverA, Unigene36901\_Mf\_liverA, Unigene36935\_Mf\_liverA, Unigene37003\_Mf\_liverA, Unigene37056\_Mf\_liverA, Unigene37082\_Mf\_liverA, Unigene37162\_Mf\_liverA, Unigene37189\_Mf\_liverA, Unigene37245\_Mf\_liverA, Unigene37282\_Mf\_liverA, Unigene37358\_Mf\_liverA, Unigene37412\_Mf\_liverA, Unigene37421\_Mf\_liverA, Unigene37458\_Mf\_liverA, Unigene37504\_Mf\_liverA, Unigene37744\_Mf\_liverA, Unigene37930\_Mf\_liverA, Unigene38015\_Mf\_liverA, Unigene3835\_Mf\_liverA, Unigene38477\_Mf\_liverA, Unigene38616\_Mf\_liverA, Unigene38685\_Mf\_liverA, Unigene38755\_Mf\_liverA, Unigene38925\_Mf\_liverA, Unigene3922\_Mf\_liverA, Unigene40289\_Mf\_liverA, Unigene40343\_Mf\_liverA, Unigene4164\_Mf\_liverA, Unigene425\_Mf\_liverA, Unigene42834\_Mf\_liverA, Unigene43198\_Mf\_liverA, Unigene44237\_Mf\_liverA, Unigene45555\_Mf\_liverA, Unigene4565\_Mf\_liverA, Unigene45\_Mf\_liverA, Unigene46319\_Mf\_liverA, Unigene4681\_Mf\_liverA, Unigene47448\_Mf\_liverA, Unigene4848\_Mf\_liverA, Unigene48648\_Mf\_liverA, Unigene48778\_Mf\_liverA, Unigene49296\_Mf\_liverA, Unigene4938\_Mf\_liverA, Unigene502\_Mf\_liverA, Unigene50424\_Mf\_liverA, Unigene5245\_Mf\_liverA, Unigene5251\_Mf\_liverA, Unigene5380\_Mf\_liverA, Unigene5453\_Mf\_liverA, Unigene551\_Mf\_liverA, Unigene5560\_Mf\_liverA, Unigene5674\_Mf\_liverA, Unigene5702\_Mf\_liverA, Unigene5842\_Mf\_liverA, Unigene6216\_Mf\_liverA, Unigene655\_Mf\_liverA, Unigene663\_Mf\_liverA, Unigene6806\_Mf\_liverA, Unigene6959\_Mf\_liverA, Unigene7339\_Mf\_liverA, Unigene77\_Mf\_liverA, Unigene7845\_Mf\_liverA, Unigene7846\_Mf\_liverA, Unigene7950\_Mf\_liverA, Unigene8117\_Mf\_liverA, Unigene8118\_Mf\_liverA, Unigene834\_Mf\_liverA, Unigene83\_Mf\_liverA, Unigene8560\_Mf\_liverA, Unigene894\_Mf\_liverA, Unigene897\_Mf\_liverA, Unigene898\_Mf\_liverA, Unigene9550\_Mf\_liverA |
| 13 | Tight junction | CL1013.Contig5\_Mf\_liverA, CL1046.Contig1\_Mf\_liverA, CL1046.Contig2\_Mf\_liverA, CL1046.Contig3\_Mf\_liverA, CL1102.Contig1\_Mf\_liverA, CL1102.Contig2\_Mf\_liverA, CL1102.Contig3\_Mf\_liverA, CL1102.Contig4\_Mf\_liverA, CL1102.Contig5\_Mf\_liverA, CL1102.Contig6\_Mf\_liverA, CL1102.Contig7\_Mf\_liverA, CL1102.Contig8\_Mf\_liverA, CL1102.Contig9\_Mf\_liverA, CL1121.Contig1\_Mf\_liverA, CL1121.Contig2\_Mf\_liverA, CL1121.Contig3\_Mf\_liverA, CL1170.Contig1\_Mf\_liverA, CL1170.Contig2\_Mf\_liverA, CL1170.Contig3\_Mf\_liverA, CL1170.Contig4\_Mf\_liverA, CL1187.Contig2\_Mf\_liverA, CL1187.Contig3\_Mf\_liverA, CL1202.Contig1\_Mf\_liverA, CL1222.Contig1\_Mf\_liverA, CL1222.Contig2\_Mf\_liverA, CL1321.Contig1\_Mf\_liverA, CL1321.Contig2\_Mf\_liverA, CL1321.Contig3\_Mf\_liverA, CL1321.Contig4\_Mf\_liverA, CL1321.Contig5\_Mf\_liverA, CL1321.Contig6\_Mf\_liverA, CL1321.Contig7\_Mf\_liverA, CL1321.Contig8\_Mf\_liverA, CL1321.Contig9\_Mf\_liverA, CL1430.Contig1\_Mf\_liverA, CL1430.Contig2\_Mf\_liverA, CL1434.Contig1\_Mf\_liverA, CL1434.Contig2\_Mf\_liverA, CL1434.Contig3\_Mf\_liverA, CL1434.Contig4\_Mf\_liverA, CL1434.Contig5\_Mf\_liverA, CL1434.Contig6\_Mf\_liverA, CL1451.Contig1\_Mf\_liverA, CL1451.Contig2\_Mf\_liverA, CL1451.Contig3\_Mf\_liverA, CL1451.Contig4\_Mf\_liverA, CL1538.Contig1\_Mf\_liverA, CL1540.Contig1\_Mf\_liverA, CL1540.Contig2\_Mf\_liverA, CL1569.Contig1\_Mf\_liverA, CL1569.Contig2\_Mf\_liverA, CL1569.Contig3\_Mf\_liverA, CL1569.Contig4\_Mf\_liverA, CL1569.Contig5\_Mf\_liverA, CL1598.Contig1\_Mf\_liverA, CL1598.Contig2\_Mf\_liverA, CL1598.Contig3\_Mf\_liverA, CL1598.Contig4\_Mf\_liverA, CL1598.Contig5\_Mf\_liverA, CL1598.Contig6\_Mf\_liverA, CL1619.Contig2\_Mf\_liverA, CL1619.Contig3\_Mf\_liverA, CL1619.Contig4\_Mf\_liverA, CL1622.Contig3\_Mf\_liverA, CL1622.Contig4\_Mf\_liverA, CL1622.Contig6\_Mf\_liverA, CL1622.Contig8\_Mf\_liverA, CL1622.Contig9\_Mf\_liverA, CL1628.Contig1\_Mf\_liverA, CL1628.Contig2\_Mf\_liverA, CL1633.Contig1\_Mf\_liverA, CL1633.Contig2\_Mf\_liverA, CL1685.Contig1\_Mf\_liverA, CL1685.Contig2\_Mf\_liverA, CL1685.Contig3\_Mf\_liverA, CL1685.Contig4\_Mf\_liverA, CL1685.Contig5\_Mf\_liverA, CL1685.Contig6\_Mf\_liverA, CL1685.Contig7\_Mf\_liverA, CL1685.Contig8\_Mf\_liverA, CL1735.Contig3\_Mf\_liverA, CL1735.Contig4\_Mf\_liverA, CL1770.Contig1\_Mf\_liverA, CL1770.Contig2\_Mf\_liverA, CL1806.Contig1\_Mf\_liverA, CL1806.Contig2\_Mf\_liverA, CL1806.Contig3\_Mf\_liverA, CL1806.Contig4\_Mf\_liverA, CL1806.Contig5\_Mf\_liverA, CL1806.Contig6\_Mf\_liverA, CL1806.Contig7\_Mf\_liverA, CL1806.Contig8\_Mf\_liverA, CL2062.Contig1\_Mf\_liverA, CL2062.Contig2\_Mf\_liverA, CL2170.Contig1\_Mf\_liverA, CL2170.Contig2\_Mf\_liverA, CL2254.Contig1\_Mf\_liverA, CL2254.Contig2\_Mf\_liverA, CL2291.Contig1\_Mf\_liverA, CL2291.Contig2\_Mf\_liverA, CL2291.Contig3\_Mf\_liverA, CL2291.Contig4\_Mf\_liverA, CL2292.Contig1\_Mf\_liverA, CL2378.Contig1\_Mf\_liverA, CL2378.Contig2\_Mf\_liverA, CL2378.Contig3\_Mf\_liverA, CL2378.Contig4\_Mf\_liverA, CL24.Contig11\_Mf\_liverA, CL24.Contig13\_Mf\_liverA, CL24.Contig7\_Mf\_liverA, CL24.Contig8\_Mf\_liverA, CL2405.Contig2\_Mf\_liverA, CL2431.Contig1\_Mf\_liverA, CL2431.Contig2\_Mf\_liverA, CL2431.Contig3\_Mf\_liverA, CL248.Contig1\_Mf\_liverA, CL248.Contig2\_Mf\_liverA, CL248.Contig3\_Mf\_liverA, CL2481.Contig1\_Mf\_liverA, CL2481.Contig2\_Mf\_liverA, CL2481.Contig3\_Mf\_liverA, CL2511.Contig1\_Mf\_liverA, CL2511.Contig2\_Mf\_liverA, CL2511.Contig3\_Mf\_liverA, CL2546.Contig1\_Mf\_liverA, CL2546.Contig2\_Mf\_liverA, CL2551.Contig1\_Mf\_liverA, CL2553.Contig1\_Mf\_liverA, CL2599.Contig1\_Mf\_liverA, CL2599.Contig2\_Mf\_liverA, CL2599.Contig3\_Mf\_liverA, CL2603.Contig1\_Mf\_liverA, CL2603.Contig2\_Mf\_liverA, CL2603.Contig3\_Mf\_liverA, CL2603.Contig4\_Mf\_liverA, CL2639.Contig1\_Mf\_liverA, CL2675.Contig1\_Mf\_liverA, CL2675.Contig2\_Mf\_liverA, CL2679.Contig1\_Mf\_liverA, CL2679.Contig2\_Mf\_liverA, CL2692.Contig1\_Mf\_liverA, CL2692.Contig2\_Mf\_liverA, CL2692.Contig3\_Mf\_liverA, CL2737.Contig2\_Mf\_liverA, CL2737.Contig3\_Mf\_liverA, CL2737.Contig4\_Mf\_liverA, CL2737.Contig5\_Mf\_liverA, CL2737.Contig6\_Mf\_liverA, CL2753.Contig1\_Mf\_liverA, CL2753.Contig2\_Mf\_liverA, CL2806.Contig1\_Mf\_liverA, CL2806.Contig2\_Mf\_liverA, CL2806.Contig3\_Mf\_liverA, CL2876.Contig1\_Mf\_liverA, CL2958.Contig1\_Mf\_liverA, CL2958.Contig2\_Mf\_liverA, CL2958.Contig3\_Mf\_liverA, CL2958.Contig4\_Mf\_liverA, CL2963.Contig1\_Mf\_liverA, CL2963.Contig2\_Mf\_liverA, CL2963.Contig3\_Mf\_liverA, CL2963.Contig4\_Mf\_liverA, CL2963.Contig5\_Mf\_liverA, CL2975.Contig1\_Mf\_liverA, CL2975.Contig2\_Mf\_liverA, CL2975.Contig3\_Mf\_liverA, CL2975.Contig4\_Mf\_liverA, CL3038.Contig1\_Mf\_liverA, CL3038.Contig2\_Mf\_liverA, CL3038.Contig3\_Mf\_liverA, CL3038.Contig4\_Mf\_liverA, CL3144.Contig1\_Mf\_liverA, CL3144.Contig2\_Mf\_liverA, CL3144.Contig3\_Mf\_liverA, CL3144.Contig4\_Mf\_liverA, CL3144.Contig5\_Mf\_liverA, CL3144.Contig6\_Mf\_liverA, CL3147.Contig1\_Mf\_liverA, CL3203.Contig1\_Mf\_liverA, CL321.Contig1\_Mf\_liverA, CL321.Contig2\_Mf\_liverA, CL3252.Contig1\_Mf\_liverA, CL3252.Contig2\_Mf\_liverA, CL3267.Contig1\_Mf\_liverA, CL3267.Contig2\_Mf\_liverA, CL3276.Contig1\_Mf\_liverA, CL3276.Contig2\_Mf\_liverA, CL3276.Contig3\_Mf\_liverA, CL3530.Contig1\_Mf\_liverA, CL3530.Contig2\_Mf\_liverA, CL3587.Contig1\_Mf\_liverA, CL3587.Contig2\_Mf\_liverA, CL3656.Contig1\_Mf\_liverA, CL3656.Contig2\_Mf\_liverA, CL3703.Contig1\_Mf\_liverA, CL3703.Contig2\_Mf\_liverA, CL371.Contig1\_Mf\_liverA, CL371.Contig3\_Mf\_liverA, CL371.Contig5\_Mf\_liverA, CL3734.Contig1\_Mf\_liverA, CL3734.Contig2\_Mf\_liverA, CL3734.Contig3\_Mf\_liverA, CL3734.Contig4\_Mf\_liverA, CL3734.Contig5\_Mf\_liverA, CL3790.Contig1\_Mf\_liverA, CL3790.Contig2\_Mf\_liverA, CL3845.Contig1\_Mf\_liverA, CL3865.Contig1\_Mf\_liverA, CL3984.Contig1\_Mf\_liverA, CL3984.Contig2\_Mf\_liverA, CL3988.Contig1\_Mf\_liverA, CL3988.Contig2\_Mf\_liverA, CL4005.Contig1\_Mf\_liverA, CL4005.Contig2\_Mf\_liverA, CL4014.Contig1\_Mf\_liverA, CL4024.Contig1\_Mf\_liverA, CL4123.Contig1\_Mf\_liverA, CL4123.Contig2\_Mf\_liverA, CL4153.Contig1\_Mf\_liverA, CL4153.Contig2\_Mf\_liverA, CL4264.Contig1\_Mf\_liverA, CL4264.Contig2\_Mf\_liverA, CL4276.Contig1\_Mf\_liverA, CL4276.Contig2\_Mf\_liverA, CL4302.Contig1\_Mf\_liverA, CL4302.Contig2\_Mf\_liverA, CL4304.Contig1\_Mf\_liverA, CL4388.Contig1\_Mf\_liverA, CL4388.Contig2\_Mf\_liverA, CL4434.Contig1\_Mf\_liverA, CL4437.Contig1\_Mf\_liverA, CL4437.Contig2\_Mf\_liverA, CL4447.Contig1\_Mf\_liverA, CL4447.Contig2\_Mf\_liverA, CL4447.Contig3\_Mf\_liverA, CL4447.Contig4\_Mf\_liverA, CL4459.Contig1\_Mf\_liverA, CL4459.Contig2\_Mf\_liverA, CL4598.Contig3\_Mf\_liverA, CL462.Contig1\_Mf\_liverA, CL462.Contig2\_Mf\_liverA, CL462.Contig3\_Mf\_liverA, CL462.Contig4\_Mf\_liverA, CL462.Contig5\_Mf\_liverA, CL462.Contig6\_Mf\_liverA, CL462.Contig7\_Mf\_liverA, CL462.Contig8\_Mf\_liverA, CL4648.Contig1\_Mf\_liverA, CL4648.Contig2\_Mf\_liverA, CL4762.Contig1\_Mf\_liverA, CL4846.Contig1\_Mf\_liverA, CL4846.Contig2\_Mf\_liverA, CL4952.Contig1\_Mf\_liverA, CL4952.Contig2\_Mf\_liverA, CL5048.Contig1\_Mf\_liverA, CL507.Contig1\_Mf\_liverA, CL5157.Contig1\_Mf\_liverA, CL5559.Contig1\_Mf\_liverA, CL5569.Contig1\_Mf\_liverA, CL5569.Contig2\_Mf\_liverA, CL5595.Contig1\_Mf\_liverA, CL5595.Contig2\_Mf\_liverA, CL561.Contig1\_Mf\_liverA, CL561.Contig2\_Mf\_liverA, CL561.Contig3\_Mf\_liverA, CL5669.Contig1\_Mf\_liverA, CL5669.Contig2\_Mf\_liverA, CL5672.Contig1\_Mf\_liverA, CL5672.Contig3\_Mf\_liverA, CL5784.Contig1\_Mf\_liverA, CL5784.Contig2\_Mf\_liverA, CL5797.Contig1\_Mf\_liverA, CL5797.Contig2\_Mf\_liverA, CL5843.Contig1\_Mf\_liverA, CL5843.Contig2\_Mf\_liverA, CL5878.Contig2\_Mf\_liverA, CL5978.Contig2\_Mf\_liverA, CL5978.Contig3\_Mf\_liverA, CL628.Contig4\_Mf\_liverA, CL628.Contig8\_Mf\_liverA, CL671.Contig3\_Mf\_liverA, CL671.Contig4\_Mf\_liverA, CL750.Contig1\_Mf\_liverA, CL750.Contig2\_Mf\_liverA, CL750.Contig3\_Mf\_liverA, CL750.Contig4\_Mf\_liverA, CL750.Contig5\_Mf\_liverA, CL779.Contig10\_Mf\_liverA, CL779.Contig12\_Mf\_liverA, CL779.Contig1\_Mf\_liverA, CL779.Contig2\_Mf\_liverA, CL779.Contig3\_Mf\_liverA, CL779.Contig4\_Mf\_liverA, CL779.Contig6\_Mf\_liverA, CL779.Contig7\_Mf\_liverA, CL779.Contig9\_Mf\_liverA, CL807.Contig1\_Mf\_liverA, CL807.Contig2\_Mf\_liverA, CL807.Contig3\_Mf\_liverA, CL807.Contig4\_Mf\_liverA, CL807.Contig5\_Mf\_liverA, CL807.Contig6\_Mf\_liverA, CL807.Contig7\_Mf\_liverA, CL807.Contig8\_Mf\_liverA, CL851.Contig1\_Mf\_liverA, CL851.Contig2\_Mf\_liverA, CL863.Contig1\_Mf\_liverA, CL863.Contig2\_Mf\_liverA, CL863.Contig3\_Mf\_liverA, CL863.Contig4\_Mf\_liverA, CL882.Contig2\_Mf\_liverA, CL882.Contig3\_Mf\_liverA, CL882.Contig5\_Mf\_liverA, CL882.Contig6\_Mf\_liverA, CL964.Contig1\_Mf\_liverA, CL964.Contig2\_Mf\_liverA, CL964.Contig3\_Mf\_liverA, CL97.Contig1\_Mf\_liverA, CL97.Contig2\_Mf\_liverA, Unigene1008\_Mf\_liverA, Unigene10249\_Mf\_liverA, Unigene10279\_Mf\_liverA, Unigene1085\_Mf\_liverA, Unigene10889\_Mf\_liverA, Unigene11594\_Mf\_liverA, Unigene12877\_Mf\_liverA, Unigene12907\_Mf\_liverA, Unigene13170\_Mf\_liverA, Unigene13171\_Mf\_liverA, Unigene13535\_Mf\_liverA, Unigene13628\_Mf\_liverA, Unigene13919\_Mf\_liverA, Unigene14030\_Mf\_liverA, Unigene14094\_Mf\_liverA, Unigene14662\_Mf\_liverA, Unigene1470\_Mf\_liverA, Unigene14732\_Mf\_liverA, Unigene14733\_Mf\_liverA, Unigene14759\_Mf\_liverA, Unigene14760\_Mf\_liverA, Unigene14765\_Mf\_liverA, Unigene14786\_Mf\_liverA, Unigene15010\_Mf\_liverA, Unigene15161\_Mf\_liverA, Unigene15442\_Mf\_liverA, Unigene15544\_Mf\_liverA, Unigene15545\_Mf\_liverA, Unigene15593\_Mf\_liverA, Unigene15656\_Mf\_liverA, Unigene15965\_Mf\_liverA, Unigene16384\_Mf\_liverA, Unigene16385\_Mf\_liverA, Unigene16458\_Mf\_liverA, Unigene16896\_Mf\_liverA, Unigene17392\_Mf\_liverA, Unigene17455\_Mf\_liverA, Unigene1779\_Mf\_liverA, Unigene18326\_Mf\_liverA, Unigene19274\_Mf\_liverA, Unigene19275\_Mf\_liverA, Unigene19992\_Mf\_liverA, Unigene20108\_Mf\_liverA, Unigene20141\_Mf\_liverA, Unigene20142\_Mf\_liverA, Unigene20143\_Mf\_liverA, Unigene20675\_Mf\_liverA, Unigene20705\_Mf\_liverA, Unigene20795\_Mf\_liverA, Unigene21463\_Mf\_liverA, Unigene22012\_Mf\_liverA, Unigene22754\_Mf\_liverA, Unigene22755\_Mf\_liverA, Unigene23102\_Mf\_liverA, Unigene23108\_Mf\_liverA, Unigene23341\_Mf\_liverA, Unigene23376\_Mf\_liverA, Unigene23377\_Mf\_liverA, Unigene23696\_Mf\_liverA, Unigene24182\_Mf\_liverA, Unigene24252\_Mf\_liverA, Unigene24281\_Mf\_liverA, Unigene24540\_Mf\_liverA, Unigene24755\_Mf\_liverA, Unigene24805\_Mf\_liverA, Unigene24909\_Mf\_liverA, Unigene25029\_Mf\_liverA, Unigene25132\_Mf\_liverA, Unigene25226\_Mf\_liverA, Unigene25298\_Mf\_liverA, Unigene25299\_Mf\_liverA, Unigene25300\_Mf\_liverA, Unigene2530\_Mf\_liverA, Unigene25751\_Mf\_liverA, Unigene25753\_Mf\_liverA, Unigene25853\_Mf\_liverA, Unigene2587\_Mf\_liverA, Unigene26190\_Mf\_liverA, Unigene26406\_Mf\_liverA, Unigene26648\_Mf\_liverA, Unigene26712\_Mf\_liverA, Unigene26829\_Mf\_liverA, Unigene26830\_Mf\_liverA, Unigene26924\_Mf\_liverA, Unigene26964\_Mf\_liverA, Unigene27221\_Mf\_liverA, Unigene27260\_Mf\_liverA, Unigene27261\_Mf\_liverA, Unigene27565\_Mf\_liverA, Unigene27939\_Mf\_liverA, Unigene27956\_Mf\_liverA, Unigene28491\_Mf\_liverA, Unigene28989\_Mf\_liverA, Unigene29676\_Mf\_liverA, Unigene29677\_Mf\_liverA, Unigene29678\_Mf\_liverA, Unigene29808\_Mf\_liverA, Unigene29809\_Mf\_liverA, Unigene29884\_Mf\_liverA, Unigene29885\_Mf\_liverA, Unigene29899\_Mf\_liverA, Unigene29900\_Mf\_liverA, Unigene30134\_Mf\_liverA, Unigene30135\_Mf\_liverA, Unigene30256\_Mf\_liverA, Unigene30380\_Mf\_liverA, Unigene30381\_Mf\_liverA, Unigene30382\_Mf\_liverA, Unigene30401\_Mf\_liverA, Unigene30568\_Mf\_liverA, Unigene30707\_Mf\_liverA, Unigene30776\_Mf\_liverA, Unigene30786\_Mf\_liverA, Unigene30873\_Mf\_liverA, Unigene30944\_Mf\_liverA, Unigene31336\_Mf\_liverA, Unigene31441\_Mf\_liverA, Unigene31442\_Mf\_liverA, Unigene31443\_Mf\_liverA, Unigene31444\_Mf\_liverA, Unigene31445\_Mf\_liverA, Unigene31487\_Mf\_liverA, Unigene31613\_Mf\_liverA, Unigene31941\_Mf\_liverA, Unigene31954\_Mf\_liverA, Unigene31968\_Mf\_liverA, Unigene32002\_Mf\_liverA, Unigene32003\_Mf\_liverA, Unigene32027\_Mf\_liverA, Unigene32096\_Mf\_liverA, Unigene32097\_Mf\_liverA, Unigene32168\_Mf\_liverA, Unigene32176\_Mf\_liverA, Unigene32182\_Mf\_liverA, Unigene32183\_Mf\_liverA, Unigene32357\_Mf\_liverA, Unigene32583\_Mf\_liverA, Unigene32592\_Mf\_liverA, Unigene32946\_Mf\_liverA, Unigene32948\_Mf\_liverA, Unigene32950\_Mf\_liverA, Unigene3295\_Mf\_liverA, Unigene32999\_Mf\_liverA, Unigene33076\_Mf\_liverA, Unigene33262\_Mf\_liverA, Unigene33263\_Mf\_liverA, Unigene33293\_Mf\_liverA, Unigene33364\_Mf\_liverA, Unigene33867\_Mf\_liverA, Unigene33903\_Mf\_liverA, Unigene34202\_Mf\_liverA, Unigene34215\_Mf\_liverA, Unigene34217\_Mf\_liverA, Unigene34218\_Mf\_liverA, Unigene34490\_Mf\_liverA, Unigene34847\_Mf\_liverA, Unigene35235\_Mf\_liverA, Unigene35399\_Mf\_liverA, Unigene35600\_Mf\_liverA, Unigene36014\_Mf\_liverA, Unigene36337\_Mf\_liverA, Unigene36648\_Mf\_liverA, Unigene36748\_Mf\_liverA, Unigene36856\_Mf\_liverA, Unigene36944\_Mf\_liverA, Unigene36979\_Mf\_liverA, Unigene37042\_Mf\_liverA, Unigene37078\_Mf\_liverA, Unigene3712\_Mf\_liverA, Unigene37132\_Mf\_liverA, Unigene37474\_Mf\_liverA, Unigene37487\_Mf\_liverA, Unigene38145\_Mf\_liverA, Unigene3886\_Mf\_liverA, Unigene39335\_Mf\_liverA, Unigene39537\_Mf\_liverA, Unigene39816\_Mf\_liverA, Unigene39890\_Mf\_liverA, Unigene40241\_Mf\_liverA, Unigene40388\_Mf\_liverA, Unigene41129\_Mf\_liverA, Unigene41402\_Mf\_liverA, Unigene41505\_Mf\_liverA, Unigene42546\_Mf\_liverA, Unigene42957\_Mf\_liverA, Unigene43225\_Mf\_liverA, Unigene43533\_Mf\_liverA, Unigene43624\_Mf\_liverA, Unigene44137\_Mf\_liverA, Unigene44657\_Mf\_liverA, Unigene44741\_Mf\_liverA, Unigene44886\_Mf\_liverA, Unigene448\_Mf\_liverA, Unigene4496\_Mf\_liverA, Unigene455\_Mf\_liverA, Unigene45719\_Mf\_liverA, Unigene459\_Mf\_liverA, Unigene4602\_Mf\_liverA, Unigene460\_Mf\_liverA, Unigene46368\_Mf\_liverA, Unigene46416\_Mf\_liverA, Unigene47134\_Mf\_liverA, Unigene4742\_Mf\_liverA, Unigene47848\_Mf\_liverA, Unigene47869\_Mf\_liverA, Unigene47970\_Mf\_liverA, Unigene48313\_Mf\_liverA, Unigene48405\_Mf\_liverA, Unigene48539\_Mf\_liverA, Unigene4863\_Mf\_liverA, Unigene4871\_Mf\_liverA, Unigene48762\_Mf\_liverA, Unigene48879\_Mf\_liverA, Unigene49422\_Mf\_liverA, Unigene50209\_Mf\_liverA, Unigene50600\_Mf\_liverA, Unigene50853\_Mf\_liverA, Unigene51104\_Mf\_liverA, Unigene51286\_Mf\_liverA, Unigene51408\_Mf\_liverA, Unigene5423\_Mf\_liverA, Unigene5636\_Mf\_liverA, Unigene5767\_Mf\_liverA, Unigene613\_Mf\_liverA, Unigene6151\_Mf\_liverA, Unigene6387\_Mf\_liverA, Unigene669\_Mf\_liverA, Unigene676\_Mf\_liverA, Unigene6816\_Mf\_liverA, Unigene6817\_Mf\_liverA, Unigene6897\_Mf\_liverA, Unigene6964\_Mf\_liverA, Unigene7146\_Mf\_liverA, Unigene7188\_Mf\_liverA, Unigene7581\_Mf\_liverA, Unigene7732\_Mf\_liverA, Unigene8066\_Mf\_liverA, Unigene8252\_Mf\_liverA, Unigene8308\_Mf\_liverA, Unigene8535\_Mf\_liverA, Unigene855\_Mf\_liverA, Unigene8624\_Mf\_liverA, Unigene8725\_Mf\_liverA, Unigene9351\_Mf\_liverA, Unigene9436\_Mf\_liverA |
| 14 | Ubiquitin mediated proteolysis | CL1002.Contig1\_Mf\_liverA, CL1002.Contig2\_Mf\_liverA, CL1020.Contig1\_Mf\_liverA, CL1020.Contig2\_Mf\_liverA, CL1051.Contig1\_Mf\_liverA, CL1051.Contig2\_Mf\_liverA, CL1051.Contig3\_Mf\_liverA, CL1051.Contig4\_Mf\_liverA, CL1051.Contig5\_Mf\_liverA, CL1051.Contig6\_Mf\_liverA, CL1087.Contig1\_Mf\_liverA, CL1087.Contig2\_Mf\_liverA, CL1087.Contig3\_Mf\_liverA, CL1087.Contig4\_Mf\_liverA, CL1087.Contig5\_Mf\_liverA, CL1087.Contig6\_Mf\_liverA, CL1147.Contig2\_Mf\_liverA, CL1147.Contig3\_Mf\_liverA, CL1260.Contig1\_Mf\_liverA, CL1260.Contig2\_Mf\_liverA, CL1260.Contig3\_Mf\_liverA, CL1260.Contig4\_Mf\_liverA, CL1371.Contig1\_Mf\_liverA, CL1371.Contig2\_Mf\_liverA, CL1371.Contig3\_Mf\_liverA, CL1371.Contig4\_Mf\_liverA, CL1379.Contig1\_Mf\_liverA, CL1379.Contig2\_Mf\_liverA, CL1379.Contig3\_Mf\_liverA, CL1379.Contig4\_Mf\_liverA, CL1427.Contig1\_Mf\_liverA, CL1427.Contig2\_Mf\_liverA, CL1567.Contig10\_Mf\_liverA, CL1567.Contig11\_Mf\_liverA, CL1567.Contig12\_Mf\_liverA, CL1567.Contig13\_Mf\_liverA, CL1567.Contig14\_Mf\_liverA, CL1567.Contig15\_Mf\_liverA, CL1567.Contig1\_Mf\_liverA, CL1567.Contig2\_Mf\_liverA, CL1567.Contig3\_Mf\_liverA, CL1567.Contig4\_Mf\_liverA, CL1567.Contig5\_Mf\_liverA, CL1567.Contig6\_Mf\_liverA, CL1567.Contig7\_Mf\_liverA, CL1567.Contig8\_Mf\_liverA, CL1567.Contig9\_Mf\_liverA, CL1585.Contig3\_Mf\_liverA, CL1604.Contig1\_Mf\_liverA, CL1604.Contig2\_Mf\_liverA, CL161.Contig1\_Mf\_liverA, CL161.Contig2\_Mf\_liverA, CL1653.Contig1\_Mf\_liverA, CL1653.Contig2\_Mf\_liverA, CL1690.Contig1\_Mf\_liverA, CL1690.Contig2\_Mf\_liverA, CL1690.Contig3\_Mf\_liverA, CL1690.Contig4\_Mf\_liverA, CL1743.Contig14\_Mf\_liverA, CL1743.Contig1\_Mf\_liverA, CL1743.Contig9\_Mf\_liverA, CL1756.Contig1\_Mf\_liverA, CL1756.Contig2\_Mf\_liverA, CL1787.Contig10\_Mf\_liverA, CL1787.Contig11\_Mf\_liverA, CL1787.Contig12\_Mf\_liverA, CL1787.Contig1\_Mf\_liverA, CL1787.Contig2\_Mf\_liverA, CL1787.Contig3\_Mf\_liverA, CL1787.Contig4\_Mf\_liverA, CL1787.Contig5\_Mf\_liverA, CL1787.Contig6\_Mf\_liverA, CL1787.Contig7\_Mf\_liverA, CL1787.Contig8\_Mf\_liverA, CL1787.Contig9\_Mf\_liverA, CL1813.Contig1\_Mf\_liverA, CL1813.Contig2\_Mf\_liverA, CL1815.Contig1\_Mf\_liverA, CL1815.Contig2\_Mf\_liverA, CL1815.Contig3\_Mf\_liverA, CL1845.Contig1\_Mf\_liverA, CL1845.Contig2\_Mf\_liverA, CL1845.Contig3\_Mf\_liverA, CL1845.Contig4\_Mf\_liverA, CL1860.Contig10\_Mf\_liverA, CL1860.Contig11\_Mf\_liverA, CL1860.Contig1\_Mf\_liverA, CL1860.Contig5\_Mf\_liverA, CL1860.Contig6\_Mf\_liverA, CL1860.Contig7\_Mf\_liverA, CL1860.Contig8\_Mf\_liverA, CL1860.Contig9\_Mf\_liverA, CL1926.Contig1\_Mf\_liverA, CL1926.Contig2\_Mf\_liverA, CL2068.Contig1\_Mf\_liverA, CL2068.Contig2\_Mf\_liverA, CL2119.Contig1\_Mf\_liverA, CL2119.Contig2\_Mf\_liverA, CL215.Contig1\_Mf\_liverA, CL215.Contig2\_Mf\_liverA, CL2294.Contig1\_Mf\_liverA, CL2311.Contig1\_Mf\_liverA, CL2371.Contig2\_Mf\_liverA, CL2393.Contig1\_Mf\_liverA, CL2393.Contig2\_Mf\_liverA, CL2393.Contig3\_Mf\_liverA, CL2393.Contig4\_Mf\_liverA, CL2434.Contig1\_Mf\_liverA, CL2434.Contig2\_Mf\_liverA, CL2434.Contig3\_Mf\_liverA, CL2434.Contig4\_Mf\_liverA, CL2434.Contig5\_Mf\_liverA, CL2453.Contig1\_Mf\_liverA, CL2453.Contig2\_Mf\_liverA, CL257.Contig2\_Mf\_liverA, CL257.Contig3\_Mf\_liverA, CL257.Contig4\_Mf\_liverA, CL257.Contig5\_Mf\_liverA, CL2592.Contig1\_Mf\_liverA, CL2592.Contig2\_Mf\_liverA, CL2592.Contig3\_Mf\_liverA, CL2607.Contig1\_Mf\_liverA, CL2607.Contig2\_Mf\_liverA, CL264.Contig1\_Mf\_liverA, CL264.Contig2\_Mf\_liverA, CL2750.Contig1\_Mf\_liverA, CL2750.Contig2\_Mf\_liverA, CL2810.Contig1\_Mf\_liverA, CL2895.Contig1\_Mf\_liverA, CL2895.Contig2\_Mf\_liverA, CL2916.Contig1\_Mf\_liverA, CL2916.Contig2\_Mf\_liverA, CL2916.Contig3\_Mf\_liverA, CL2928.Contig1\_Mf\_liverA, CL2928.Contig2\_Mf\_liverA, CL2928.Contig3\_Mf\_liverA, CL2928.Contig4\_Mf\_liverA, CL2994.Contig1\_Mf\_liverA, CL2994.Contig2\_Mf\_liverA, CL3026.Contig1\_Mf\_liverA, CL3149.Contig1\_Mf\_liverA, CL3149.Contig2\_Mf\_liverA, CL3149.Contig3\_Mf\_liverA, CL3149.Contig4\_Mf\_liverA, CL3149.Contig5\_Mf\_liverA, CL3149.Contig6\_Mf\_liverA, CL3184.Contig1\_Mf\_liverA, CL3184.Contig2\_Mf\_liverA, CL3243.Contig1\_Mf\_liverA, CL3243.Contig2\_Mf\_liverA, CL3247.Contig1\_Mf\_liverA, CL3247.Contig2\_Mf\_liverA, CL325.Contig1\_Mf\_liverA, CL3286.Contig1\_Mf\_liverA, CL3286.Contig2\_Mf\_liverA, CL3381.Contig1\_Mf\_liverA, CL3381.Contig2\_Mf\_liverA, CL3425.Contig1\_Mf\_liverA, CL3425.Contig2\_Mf\_liverA, CL3616.Contig1\_Mf\_liverA, CL3616.Contig2\_Mf\_liverA, CL3680.Contig1\_Mf\_liverA, CL3680.Contig2\_Mf\_liverA, CL3680.Contig3\_Mf\_liverA, CL3773.Contig1\_Mf\_liverA, CL3773.Contig2\_Mf\_liverA, CL3871.Contig1\_Mf\_liverA, CL3871.Contig2\_Mf\_liverA, CL3871.Contig3\_Mf\_liverA, CL4008.Contig2\_Mf\_liverA, CL4015.Contig1\_Mf\_liverA, CL4015.Contig2\_Mf\_liverA, CL4038.Contig2\_Mf\_liverA, CL4106.Contig1\_Mf\_liverA, CL4106.Contig2\_Mf\_liverA, CL4161.Contig1\_Mf\_liverA, CL4161.Contig2\_Mf\_liverA, CL4262.Contig2\_Mf\_liverA, CL4262.Contig3\_Mf\_liverA, CL4281.Contig1\_Mf\_liverA, CL4281.Contig2\_Mf\_liverA, CL4362.Contig1\_Mf\_liverA, CL4362.Contig2\_Mf\_liverA, CL452.Contig1\_Mf\_liverA, CL452.Contig2\_Mf\_liverA, CL4556.Contig1\_Mf\_liverA, CL4556.Contig2\_Mf\_liverA, CL464.Contig10\_Mf\_liverA, CL464.Contig11\_Mf\_liverA, CL464.Contig12\_Mf\_liverA, CL464.Contig13\_Mf\_liverA, CL464.Contig14\_Mf\_liverA, CL464.Contig15\_Mf\_liverA, CL464.Contig16\_Mf\_liverA, CL464.Contig17\_Mf\_liverA, CL464.Contig18\_Mf\_liverA, CL464.Contig19\_Mf\_liverA, CL464.Contig1\_Mf\_liverA, CL464.Contig20\_Mf\_liverA, CL464.Contig21\_Mf\_liverA, CL464.Contig22\_Mf\_liverA, CL464.Contig23\_Mf\_liverA, CL464.Contig24\_Mf\_liverA, CL464.Contig25\_Mf\_liverA, CL464.Contig26\_Mf\_liverA, CL464.Contig27\_Mf\_liverA, CL464.Contig28\_Mf\_liverA, CL464.Contig29\_Mf\_liverA, CL464.Contig2\_Mf\_liverA, CL464.Contig30\_Mf\_liverA, CL464.Contig31\_Mf\_liverA, CL464.Contig3\_Mf\_liverA, CL464.Contig4\_Mf\_liverA, CL464.Contig5\_Mf\_liverA, CL464.Contig6\_Mf\_liverA, CL464.Contig7\_Mf\_liverA, CL464.Contig8\_Mf\_liverA, CL464.Contig9\_Mf\_liverA, CL4724.Contig1\_Mf\_liverA, CL4724.Contig2\_Mf\_liverA, CL4724.Contig3\_Mf\_liverA, CL4765.Contig1\_Mf\_liverA, CL4765.Contig2\_Mf\_liverA, CL4788.Contig1\_Mf\_liverA, CL4788.Contig2\_Mf\_liverA, CL4911.Contig1\_Mf\_liverA, CL4911.Contig2\_Mf\_liverA, CL4962.Contig1\_Mf\_liverA, CL4962.Contig2\_Mf\_liverA, CL5033.Contig1\_Mf\_liverA, CL5033.Contig2\_Mf\_liverA, CL5149.Contig1\_Mf\_liverA, CL5149.Contig2\_Mf\_liverA, CL5157.Contig1\_Mf\_liverA, CL5222.Contig1\_Mf\_liverA, CL5222.Contig2\_Mf\_liverA, CL5287.Contig1\_Mf\_liverA, CL5287.Contig2\_Mf\_liverA, CL534.Contig1\_Mf\_liverA, CL534.Contig2\_Mf\_liverA, CL534.Contig3\_Mf\_liverA, CL5348.Contig1\_Mf\_liverA, CL5348.Contig2\_Mf\_liverA, CL5481.Contig1\_Mf\_liverA, CL5486.Contig1\_Mf\_liverA, CL5486.Contig2\_Mf\_liverA, CL5491.Contig1\_Mf\_liverA, CL5491.Contig2\_Mf\_liverA, CL5496.Contig1\_Mf\_liverA, CL5496.Contig2\_Mf\_liverA, CL5496.Contig3\_Mf\_liverA, CL5496.Contig4\_Mf\_liverA, CL5496.Contig5\_Mf\_liverA, CL5617.Contig1\_Mf\_liverA, CL5617.Contig2\_Mf\_liverA, CL5703.Contig1\_Mf\_liverA, CL5703.Contig2\_Mf\_liverA, CL5737.Contig1\_Mf\_liverA, CL5737.Contig2\_Mf\_liverA, CL5772.Contig1\_Mf\_liverA, CL5772.Contig2\_Mf\_liverA, CL5772.Contig3\_Mf\_liverA, CL5919.Contig1\_Mf\_liverA, CL5919.Contig2\_Mf\_liverA, CL5950.Contig1\_Mf\_liverA, CL5950.Contig2\_Mf\_liverA, CL5950.Contig3\_Mf\_liverA, CL5970.Contig1\_Mf\_liverA, CL5970.Contig2\_Mf\_liverA, CL644.Contig1\_Mf\_liverA, CL644.Contig2\_Mf\_liverA, CL644.Contig3\_Mf\_liverA, CL644.Contig4\_Mf\_liverA, CL775.Contig1\_Mf\_liverA, CL775.Contig2\_Mf\_liverA, CL775.Contig3\_Mf\_liverA, CL777.Contig7\_Mf\_liverA, CL777.Contig8\_Mf\_liverA, CL791.Contig1\_Mf\_liverA, CL791.Contig2\_Mf\_liverA, CL839.Contig1\_Mf\_liverA, CL839.Contig2\_Mf\_liverA, CL873.Contig1\_Mf\_liverA, CL873.Contig2\_Mf\_liverA, CL907.Contig1\_Mf\_liverA, CL907.Contig2\_Mf\_liverA, CL907.Contig3\_Mf\_liverA, CL907.Contig4\_Mf\_liverA, CL988.Contig1\_Mf\_liverA, CL988.Contig2\_Mf\_liverA, CL988.Contig3\_Mf\_liverA, CL988.Contig4\_Mf\_liverA, Unigene10029\_Mf\_liverA, Unigene10087\_Mf\_liverA, Unigene10273\_Mf\_liverA, Unigene10516\_Mf\_liverA, Unigene10517\_Mf\_liverA, Unigene10618\_Mf\_liverA, Unigene10780\_Mf\_liverA, Unigene1116\_Mf\_liverA, Unigene11366\_Mf\_liverA, Unigene1156\_Mf\_liverA, Unigene11845\_Mf\_liverA, Unigene12538\_Mf\_liverA, Unigene12660\_Mf\_liverA, Unigene12661\_Mf\_liverA, Unigene12819\_Mf\_liverA, Unigene1282\_Mf\_liverA, Unigene13225\_Mf\_liverA, Unigene13247\_Mf\_liverA, Unigene13248\_Mf\_liverA, Unigene13769\_Mf\_liverA, Unigene13798\_Mf\_liverA, Unigene13799\_Mf\_liverA, Unigene13800\_Mf\_liverA, Unigene13801\_Mf\_liverA, Unigene14078\_Mf\_liverA, Unigene141\_Mf\_liverA, Unigene14203\_Mf\_liverA, Unigene14217\_Mf\_liverA, Unigene14218\_Mf\_liverA, Unigene142\_Mf\_liverA, Unigene143\_Mf\_liverA, Unigene1468\_Mf\_liverA, Unigene14747\_Mf\_liverA, Unigene14774\_Mf\_liverA, Unigene14971\_Mf\_liverA, Unigene15115\_Mf\_liverA, Unigene15186\_Mf\_liverA, Unigene15196\_Mf\_liverA, Unigene15286\_Mf\_liverA, Unigene15476\_Mf\_liverA, Unigene15477\_Mf\_liverA, Unigene15478\_Mf\_liverA, Unigene15661\_Mf\_liverA, Unigene17330\_Mf\_liverA, Unigene18095\_Mf\_liverA, Unigene18813\_Mf\_liverA, Unigene19116\_Mf\_liverA, Unigene19392\_Mf\_liverA, Unigene19742\_Mf\_liverA, Unigene19767\_Mf\_liverA, Unigene20321\_Mf\_liverA, Unigene20855\_Mf\_liverA, Unigene2086\_Mf\_liverA, Unigene212\_Mf\_liverA, Unigene21741\_Mf\_liverA, Unigene21742\_Mf\_liverA, Unigene21757\_Mf\_liverA, Unigene22255\_Mf\_liverA, Unigene22256\_Mf\_liverA, Unigene22257\_Mf\_liverA, Unigene22285\_Mf\_liverA, Unigene22286\_Mf\_liverA, Unigene22825\_Mf\_liverA, Unigene23408\_Mf\_liverA, Unigene23409\_Mf\_liverA, Unigene23519\_Mf\_liverA, Unigene23589\_Mf\_liverA, Unigene23894\_Mf\_liverA, Unigene23895\_Mf\_liverA, Unigene24067\_Mf\_liverA, Unigene24164\_Mf\_liverA, Unigene24165\_Mf\_liverA, Unigene24463\_Mf\_liverA, Unigene24511\_Mf\_liverA, Unigene24512\_Mf\_liverA, Unigene24527\_Mf\_liverA, Unigene24528\_Mf\_liverA, Unigene24832\_Mf\_liverA, Unigene24833\_Mf\_liverA, Unigene24991\_Mf\_liverA, Unigene24992\_Mf\_liverA, Unigene25066\_Mf\_liverA, Unigene2530\_Mf\_liverA, Unigene25887\_Mf\_liverA, Unigene258\_Mf\_liverA, Unigene26037\_Mf\_liverA, Unigene26854\_Mf\_liverA, Unigene26855\_Mf\_liverA, Unigene27144\_Mf\_liverA, Unigene27181\_Mf\_liverA, Unigene27242\_Mf\_liverA, Unigene27244\_Mf\_liverA, Unigene27292\_Mf\_liverA, Unigene27339\_Mf\_liverA, Unigene27366\_Mf\_liverA, Unigene27481\_Mf\_liverA, Unigene27823\_Mf\_liverA, Unigene28070\_Mf\_liverA, Unigene28335\_Mf\_liverA, Unigene28336\_Mf\_liverA, Unigene28338\_Mf\_liverA, Unigene28413\_Mf\_liverA, Unigene28436\_Mf\_liverA, Unigene28437\_Mf\_liverA, Unigene2854\_Mf\_liverA, Unigene28888\_Mf\_liverA, Unigene28893\_Mf\_liverA, Unigene29252\_Mf\_liverA, Unigene29326\_Mf\_liverA, Unigene29327\_Mf\_liverA, Unigene29334\_Mf\_liverA, Unigene29949\_Mf\_liverA, Unigene30785\_Mf\_liverA, Unigene30825\_Mf\_liverA, Unigene30846\_Mf\_liverA, Unigene31010\_Mf\_liverA, Unigene31011\_Mf\_liverA, Unigene31022\_Mf\_liverA, Unigene31024\_Mf\_liverA, Unigene3129\_Mf\_liverA, Unigene31353\_Mf\_liverA, Unigene31354\_Mf\_liverA, Unigene31355\_Mf\_liverA, Unigene31405\_Mf\_liverA, Unigene31407\_Mf\_liverA, Unigene31474\_Mf\_liverA, Unigene31475\_Mf\_liverA, Unigene31592\_Mf\_liverA, Unigene31593\_Mf\_liverA, Unigene31594\_Mf\_liverA, Unigene31595\_Mf\_liverA, Unigene31597\_Mf\_liverA, Unigene31599\_Mf\_liverA, Unigene31709\_Mf\_liverA, Unigene31710\_Mf\_liverA, Unigene31711\_Mf\_liverA, Unigene31804\_Mf\_liverA, Unigene32213\_Mf\_liverA, Unigene32214\_Mf\_liverA, Unigene32484\_Mf\_liverA, Unigene32489\_Mf\_liverA, Unigene32568\_Mf\_liverA, Unigene32922\_Mf\_liverA, Unigene32923\_Mf\_liverA, Unigene33235\_Mf\_liverA, Unigene33478\_Mf\_liverA, Unigene33681\_Mf\_liverA, Unigene337\_Mf\_liverA, Unigene34201\_Mf\_liverA, Unigene34446\_Mf\_liverA, Unigene34479\_Mf\_liverA, Unigene34588\_Mf\_liverA, Unigene34618\_Mf\_liverA, Unigene34619\_Mf\_liverA, Unigene34767\_Mf\_liverA, Unigene34849\_Mf\_liverA, Unigene35147\_Mf\_liverA, Unigene35276\_Mf\_liverA, Unigene35371\_Mf\_liverA, Unigene35372\_Mf\_liverA, Unigene35491\_Mf\_liverA, Unigene35747\_Mf\_liverA, Unigene35996\_Mf\_liverA, Unigene36049\_Mf\_liverA, Unigene36288\_Mf\_liverA, Unigene36289\_Mf\_liverA, Unigene36308\_Mf\_liverA, Unigene36309\_Mf\_liverA, Unigene36310\_Mf\_liverA, Unigene36311\_Mf\_liverA, Unigene36615\_Mf\_liverA, Unigene36692\_Mf\_liverA, Unigene36744\_Mf\_liverA, Unigene36807\_Mf\_liverA, Unigene36916\_Mf\_liverA, Unigene36934\_Mf\_liverA, Unigene37006\_Mf\_liverA, Unigene37090\_Mf\_liverA, Unigene37182\_Mf\_liverA, Unigene37276\_Mf\_liverA, Unigene37492\_Mf\_liverA, Unigene37516\_Mf\_liverA, Unigene37925\_Mf\_liverA, Unigene37945\_Mf\_liverA, Unigene38011\_Mf\_liverA, Unigene38265\_Mf\_liverA, Unigene38508\_Mf\_liverA, Unigene38994\_Mf\_liverA, Unigene39104\_Mf\_liverA, Unigene39165\_Mf\_liverA, Unigene39200\_Mf\_liverA, Unigene39466\_Mf\_liverA, Unigene39618\_Mf\_liverA, Unigene39632\_Mf\_liverA, Unigene39666\_Mf\_liverA, Unigene40101\_Mf\_liverA, Unigene41234\_Mf\_liverA, Unigene41298\_Mf\_liverA, Unigene416\_Mf\_liverA, Unigene42323\_Mf\_liverA, Unigene42506\_Mf\_liverA, Unigene42830\_Mf\_liverA, Unigene42869\_Mf\_liverA, Unigene43900\_Mf\_liverA, Unigene43966\_Mf\_liverA, Unigene44701\_Mf\_liverA, Unigene44779\_Mf\_liverA, Unigene45240\_Mf\_liverA, Unigene4615\_Mf\_liverA, Unigene4657\_Mf\_liverA, Unigene4658\_Mf\_liverA, Unigene46807\_Mf\_liverA, Unigene48052\_Mf\_liverA, Unigene4818\_Mf\_liverA, Unigene481\_Mf\_liverA, Unigene4821\_Mf\_liverA, Unigene48460\_Mf\_liverA, Unigene50031\_Mf\_liverA, Unigene50203\_Mf\_liverA, Unigene5111\_Mf\_liverA, Unigene5141\_Mf\_liverA, Unigene5151\_Mf\_liverA, Unigene5176\_Mf\_liverA, Unigene5190\_Mf\_liverA, Unigene5248\_Mf\_liverA, Unigene524\_Mf\_liverA, Unigene5289\_Mf\_liverA, Unigene5417\_Mf\_liverA, Unigene555\_Mf\_liverA, Unigene5560\_Mf\_liverA, Unigene558\_Mf\_liverA, Unigene5813\_Mf\_liverA, Unigene5815\_Mf\_liverA, Unigene5825\_Mf\_liverA, Unigene5856\_Mf\_liverA, Unigene5938\_Mf\_liverA, Unigene593\_Mf\_liverA, Unigene5954\_Mf\_liverA, Unigene6175\_Mf\_liverA, Unigene6176\_Mf\_liverA, Unigene6259\_Mf\_liverA, Unigene6305\_Mf\_liverA, Unigene6306\_Mf\_liverA, Unigene6393\_Mf\_liverA, Unigene6540\_Mf\_liverA, Unigene6559\_Mf\_liverA, Unigene7034\_Mf\_liverA, Unigene7257\_Mf\_liverA, Unigene7297\_Mf\_liverA, Unigene742\_Mf\_liverA, Unigene7447\_Mf\_liverA, Unigene7833\_Mf\_liverA, Unigene836\_Mf\_liverA, Unigene852\_Mf\_liverA, Unigene857\_Mf\_liverA, Unigene8906\_Mf\_liverA, Unigene9252\_Mf\_liverA, Unigene970\_Mf\_liverA |
| 15 | Spliceosome | CL1000.Contig1\_Mf\_liverA, CL1000.Contig2\_Mf\_liverA, CL1000.Contig3\_Mf\_liverA, CL1033.Contig1\_Mf\_liverA, CL1033.Contig4\_Mf\_liverA, CL1055.Contig1\_Mf\_liverA, CL1055.Contig2\_Mf\_liverA, CL1185.Contig1\_Mf\_liverA, CL1185.Contig2\_Mf\_liverA, CL1254.Contig1\_Mf\_liverA, CL1254.Contig2\_Mf\_liverA, CL1254.Contig3\_Mf\_liverA, CL1254.Contig4\_Mf\_liverA, CL1254.Contig5\_Mf\_liverA, CL1254.Contig6\_Mf\_liverA, CL1265.Contig1\_Mf\_liverA, CL1266.Contig2\_Mf\_liverA, CL1282.Contig1\_Mf\_liverA, CL1282.Contig2\_Mf\_liverA, CL1304.Contig1\_Mf\_liverA, CL1304.Contig2\_Mf\_liverA, CL1304.Contig3\_Mf\_liverA, CL1304.Contig4\_Mf\_liverA, CL1332.Contig1\_Mf\_liverA, CL1332.Contig2\_Mf\_liverA, CL1338.Contig1\_Mf\_liverA, CL1338.Contig2\_Mf\_liverA, CL1338.Contig3\_Mf\_liverA, CL1338.Contig4\_Mf\_liverA, CL1338.Contig5\_Mf\_liverA, CL1338.Contig6\_Mf\_liverA, CL1437.Contig1\_Mf\_liverA, CL1437.Contig2\_Mf\_liverA, CL1460.Contig1\_Mf\_liverA, CL1460.Contig2\_Mf\_liverA, CL1494.Contig1\_Mf\_liverA, CL1497.Contig1\_Mf\_liverA, CL1497.Contig2\_Mf\_liverA, CL1590.Contig1\_Mf\_liverA, CL1590.Contig2\_Mf\_liverA, CL1590.Contig3\_Mf\_liverA, CL1590.Contig4\_Mf\_liverA, CL1701.Contig1\_Mf\_liverA, CL1701.Contig2\_Mf\_liverA, CL1805.Contig1\_Mf\_liverA, CL1805.Contig2\_Mf\_liverA, CL1872.Contig1\_Mf\_liverA, CL1872.Contig3\_Mf\_liverA, CL1872.Contig4\_Mf\_liverA, CL1872.Contig7\_Mf\_liverA, CL1970.Contig1\_Mf\_liverA, CL1970.Contig3\_Mf\_liverA, CL1970.Contig6\_Mf\_liverA, CL2000.Contig1\_Mf\_liverA, CL2042.Contig1\_Mf\_liverA, CL2042.Contig2\_Mf\_liverA, CL2042.Contig3\_Mf\_liverA, CL2135.Contig1\_Mf\_liverA, CL2135.Contig2\_Mf\_liverA, CL2135.Contig3\_Mf\_liverA, CL2154.Contig2\_Mf\_liverA, CL2209.Contig1\_Mf\_liverA, CL2209.Contig2\_Mf\_liverA, CL2292.Contig2\_Mf\_liverA, CL2301.Contig1\_Mf\_liverA, CL2301.Contig2\_Mf\_liverA, CL2301.Contig3\_Mf\_liverA, CL2301.Contig4\_Mf\_liverA, CL231.Contig1\_Mf\_liverA, CL231.Contig2\_Mf\_liverA, CL2325.Contig1\_Mf\_liverA, CL2325.Contig2\_Mf\_liverA, CL2329.Contig1\_Mf\_liverA, CL2329.Contig2\_Mf\_liverA, CL2330.Contig1\_Mf\_liverA, CL2330.Contig2\_Mf\_liverA, CL2330.Contig3\_Mf\_liverA, CL2330.Contig4\_Mf\_liverA, CL2487.Contig4\_Mf\_liverA, CL2487.Contig5\_Mf\_liverA, CL2569.Contig1\_Mf\_liverA, CL2569.Contig2\_Mf\_liverA, CL2668.Contig1\_Mf\_liverA, CL2668.Contig2\_Mf\_liverA, CL2668.Contig3\_Mf\_liverA, CL2676.Contig1\_Mf\_liverA, CL2676.Contig2\_Mf\_liverA, CL2723.Contig1\_Mf\_liverA, CL2723.Contig2\_Mf\_liverA, CL2861.Contig1\_Mf\_liverA, CL2861.Contig2\_Mf\_liverA, CL2871.Contig1\_Mf\_liverA, CL288.Contig1\_Mf\_liverA, CL288.Contig2\_Mf\_liverA, CL2978.Contig1\_Mf\_liverA, CL2978.Contig2\_Mf\_liverA, CL3013.Contig1\_Mf\_liverA, CL3013.Contig2\_Mf\_liverA, CL3076.Contig1\_Mf\_liverA, CL3076.Contig2\_Mf\_liverA, CL3076.Contig3\_Mf\_liverA, CL3076.Contig4\_Mf\_liverA, CL3076.Contig5\_Mf\_liverA, CL3076.Contig6\_Mf\_liverA, CL3076.Contig7\_Mf\_liverA, CL3076.Contig8\_Mf\_liverA, CL3081.Contig1\_Mf\_liverA, CL3138.Contig1\_Mf\_liverA, CL3138.Contig2\_Mf\_liverA, CL3173.Contig1\_Mf\_liverA, CL3173.Contig2\_Mf\_liverA, CL3244.Contig1\_Mf\_liverA, CL3244.Contig2\_Mf\_liverA, CL3246.Contig1\_Mf\_liverA, CL3246.Contig2\_Mf\_liverA, CL3250.Contig1\_Mf\_liverA, CL3250.Contig2\_Mf\_liverA, CL3268.Contig1\_Mf\_liverA, CL3268.Contig2\_Mf\_liverA, CL3311.Contig1\_Mf\_liverA, CL3311.Contig2\_Mf\_liverA, CL3311.Contig3\_Mf\_liverA, CL3362.Contig3\_Mf\_liverA, CL3419.Contig1\_Mf\_liverA, CL3419.Contig2\_Mf\_liverA, CL3452.Contig1\_Mf\_liverA, CL3452.Contig2\_Mf\_liverA, CL3468.Contig1\_Mf\_liverA, CL3468.Contig2\_Mf\_liverA, CL3486.Contig1\_Mf\_liverA, CL3486.Contig2\_Mf\_liverA, CL3486.Contig3\_Mf\_liverA, CL3523.Contig1\_Mf\_liverA, CL3523.Contig2\_Mf\_liverA, CL356.Contig1\_Mf\_liverA, CL3581.Contig1\_Mf\_liverA, CL3581.Contig2\_Mf\_liverA, CL3645.Contig1\_Mf\_liverA, CL3775.Contig1\_Mf\_liverA, CL3775.Contig2\_Mf\_liverA, CL3801.Contig1\_Mf\_liverA, CL3801.Contig2\_Mf\_liverA, CL3887.Contig1\_Mf\_liverA, CL3887.Contig2\_Mf\_liverA, CL3896.Contig1\_Mf\_liverA, CL3896.Contig2\_Mf\_liverA, CL396.Contig1\_Mf\_liverA, CL396.Contig2\_Mf\_liverA, CL396.Contig3\_Mf\_liverA, CL396.Contig4\_Mf\_liverA, CL396.Contig5\_Mf\_liverA, CL396.Contig6\_Mf\_liverA, CL4043.Contig1\_Mf\_liverA, CL4057.Contig1\_Mf\_liverA, CL4057.Contig2\_Mf\_liverA, CL4057.Contig3\_Mf\_liverA, CL4057.Contig4\_Mf\_liverA, CL4074.Contig1\_Mf\_liverA, CL4074.Contig2\_Mf\_liverA, CL4115.Contig1\_Mf\_liverA, CL4126.Contig1\_Mf\_liverA, CL4126.Contig2\_Mf\_liverA, CL4159.Contig1\_Mf\_liverA, CL4159.Contig2\_Mf\_liverA, CL4159.Contig3\_Mf\_liverA, CL4159.Contig4\_Mf\_liverA, CL4183.Contig2\_Mf\_liverA, CL4237.Contig1\_Mf\_liverA, CL4237.Contig2\_Mf\_liverA, CL4255.Contig1\_Mf\_liverA, CL4255.Contig2\_Mf\_liverA, CL4285.Contig1\_Mf\_liverA, CL4285.Contig2\_Mf\_liverA, CL4331.Contig1\_Mf\_liverA, CL4331.Contig2\_Mf\_liverA, CL4365.Contig1\_Mf\_liverA, CL4365.Contig2\_Mf\_liverA, CL4403.Contig1\_Mf\_liverA, CL4403.Contig2\_Mf\_liverA, CL4409.Contig1\_Mf\_liverA, CL4454.Contig1\_Mf\_liverA, CL4454.Contig2\_Mf\_liverA, CL4466.Contig1\_Mf\_liverA, CL4466.Contig2\_Mf\_liverA, CL4485.Contig1\_Mf\_liverA, CL4485.Contig2\_Mf\_liverA, CL4502.Contig1\_Mf\_liverA, CL4502.Contig2\_Mf\_liverA, CL4506.Contig1\_Mf\_liverA, CL4506.Contig2\_Mf\_liverA, CL4547.Contig1\_Mf\_liverA, CL4547.Contig2\_Mf\_liverA, CL4547.Contig3\_Mf\_liverA, CL4647.Contig1\_Mf\_liverA, CL4647.Contig2\_Mf\_liverA, CL4795.Contig1\_Mf\_liverA, CL4795.Contig2\_Mf\_liverA, CL4800.Contig1\_Mf\_liverA, CL4800.Contig2\_Mf\_liverA, CL481.Contig1\_Mf\_liverA, CL481.Contig2\_Mf\_liverA, CL4812.Contig1\_Mf\_liverA, CL4820.Contig1\_Mf\_liverA, CL4820.Contig2\_Mf\_liverA, CL4855.Contig1\_Mf\_liverA, CL4855.Contig2\_Mf\_liverA, CL4869.Contig1\_Mf\_liverA, CL4869.Contig2\_Mf\_liverA, CL4869.Contig3\_Mf\_liverA, CL4869.Contig4\_Mf\_liverA, CL5001.Contig1\_Mf\_liverA, CL5001.Contig2\_Mf\_liverA, CL5033.Contig1\_Mf\_liverA, CL5033.Contig2\_Mf\_liverA, CL5047.Contig1\_Mf\_liverA, CL5109.Contig1\_Mf\_liverA, CL5229.Contig1\_Mf\_liverA, CL5333.Contig1\_Mf\_liverA, CL5333.Contig2\_Mf\_liverA, CL5460.Contig1\_Mf\_liverA, CL55.Contig1\_Mf\_liverA, CL5527.Contig1\_Mf\_liverA, CL5538.Contig1\_Mf\_liverA, CL5538.Contig2\_Mf\_liverA, CL5635.Contig1\_Mf\_liverA, CL5635.Contig2\_Mf\_liverA, CL5637.Contig1\_Mf\_liverA, CL5637.Contig2\_Mf\_liverA, CL5637.Contig3\_Mf\_liverA, CL5656.Contig1\_Mf\_liverA, CL5656.Contig2\_Mf\_liverA, CL5670.Contig1\_Mf\_liverA, CL5670.Contig2\_Mf\_liverA, CL5789.Contig1\_Mf\_liverA, CL5789.Contig2\_Mf\_liverA, CL5794.Contig1\_Mf\_liverA, CL5794.Contig2\_Mf\_liverA, CL624.Contig1\_Mf\_liverA, CL624.Contig2\_Mf\_liverA, CL624.Contig3\_Mf\_liverA, CL629.Contig1\_Mf\_liverA, CL629.Contig2\_Mf\_liverA, CL629.Contig3\_Mf\_liverA, CL658.Contig1\_Mf\_liverA, CL658.Contig2\_Mf\_liverA, CL658.Contig3\_Mf\_liverA, CL708.Contig12\_Mf\_liverA, CL708.Contig16\_Mf\_liverA, CL708.Contig1\_Mf\_liverA, CL708.Contig4\_Mf\_liverA, CL708.Contig6\_Mf\_liverA, CL715.Contig2\_Mf\_liverA, CL763.Contig1\_Mf\_liverA, CL764.Contig1\_Mf\_liverA, CL764.Contig2\_Mf\_liverA, CL764.Contig3\_Mf\_liverA, CL774.Contig10\_Mf\_liverA, CL774.Contig11\_Mf\_liverA, CL774.Contig12\_Mf\_liverA, CL774.Contig3\_Mf\_liverA, CL774.Contig4\_Mf\_liverA, CL774.Contig5\_Mf\_liverA, CL774.Contig6\_Mf\_liverA, CL774.Contig9\_Mf\_liverA, CL793.Contig2\_Mf\_liverA, CL798.Contig10\_Mf\_liverA, CL798.Contig13\_Mf\_liverA, CL798.Contig16\_Mf\_liverA, CL798.Contig17\_Mf\_liverA, CL798.Contig18\_Mf\_liverA, CL798.Contig19\_Mf\_liverA, CL798.Contig20\_Mf\_liverA, CL798.Contig21\_Mf\_liverA, CL798.Contig22\_Mf\_liverA, CL798.Contig2\_Mf\_liverA, CL798.Contig3\_Mf\_liverA, CL798.Contig4\_Mf\_liverA, CL798.Contig5\_Mf\_liverA, CL798.Contig6\_Mf\_liverA, CL798.Contig7\_Mf\_liverA, CL798.Contig8\_Mf\_liverA, CL798.Contig9\_Mf\_liverA, CL808.Contig1\_Mf\_liverA, CL808.Contig2\_Mf\_liverA, CL808.Contig3\_Mf\_liverA, CL812.Contig2\_Mf\_liverA, CL836.Contig1\_Mf\_liverA, CL836.Contig2\_Mf\_liverA, CL836.Contig3\_Mf\_liverA, CL836.Contig4\_Mf\_liverA, CL836.Contig5\_Mf\_liverA, CL836.Contig6\_Mf\_liverA, CL836.Contig7\_Mf\_liverA, CL836.Contig8\_Mf\_liverA, CL878.Contig1\_Mf\_liverA, CL878.Contig2\_Mf\_liverA, Unigene1007\_Mf\_liverA, Unigene11245\_Mf\_liverA, Unigene11493\_Mf\_liverA, Unigene11673\_Mf\_liverA, Unigene12992\_Mf\_liverA, Unigene13116\_Mf\_liverA, Unigene13225\_Mf\_liverA, Unigene1357\_Mf\_liverA, Unigene13597\_Mf\_liverA, Unigene1372\_Mf\_liverA, Unigene13865\_Mf\_liverA, Unigene14039\_Mf\_liverA, Unigene14376\_Mf\_liverA, Unigene14431\_Mf\_liverA, Unigene14500\_Mf\_liverA, Unigene14652\_Mf\_liverA, Unigene14655\_Mf\_liverA, Unigene1471\_Mf\_liverA, Unigene14758\_Mf\_liverA, Unigene15204\_Mf\_liverA, Unigene15205\_Mf\_liverA, Unigene15215\_Mf\_liverA, Unigene15397\_Mf\_liverA, Unigene15619\_Mf\_liverA, Unigene15645\_Mf\_liverA, Unigene15670\_Mf\_liverA, Unigene15689\_Mf\_liverA, Unigene17784\_Mf\_liverA, Unigene18387\_Mf\_liverA, Unigene18792\_Mf\_liverA, Unigene19821\_Mf\_liverA, Unigene19881\_Mf\_liverA, Unigene21389\_Mf\_liverA, Unigene21423\_Mf\_liverA, Unigene21591\_Mf\_liverA, Unigene23664\_Mf\_liverA, Unigene24242\_Mf\_liverA, Unigene24432\_Mf\_liverA, Unigene24628\_Mf\_liverA, Unigene24789\_Mf\_liverA, Unigene24879\_Mf\_liverA, Unigene25025\_Mf\_liverA, Unigene25218\_Mf\_liverA, Unigene25661\_Mf\_liverA, Unigene25681\_Mf\_liverA, Unigene25736\_Mf\_liverA, Unigene25744\_Mf\_liverA, Unigene25780\_Mf\_liverA, Unigene25783\_Mf\_liverA, Unigene26470\_Mf\_liverA, Unigene26471\_Mf\_liverA, Unigene26726\_Mf\_liverA, Unigene26912\_Mf\_liverA, Unigene27184\_Mf\_liverA, Unigene27230\_Mf\_liverA, Unigene27231\_Mf\_liverA, Unigene27366\_Mf\_liverA, Unigene2815\_Mf\_liverA, Unigene28357\_Mf\_liverA, Unigene28377\_Mf\_liverA, Unigene28378\_Mf\_liverA, Unigene28405\_Mf\_liverA, Unigene28440\_Mf\_liverA, Unigene28450\_Mf\_liverA, Unigene28498\_Mf\_liverA, Unigene2886\_Mf\_liverA, Unigene28955\_Mf\_liverA, Unigene28983\_Mf\_liverA, Unigene29147\_Mf\_liverA, Unigene29208\_Mf\_liverA, Unigene29209\_Mf\_liverA, Unigene29262\_Mf\_liverA, Unigene29400\_Mf\_liverA, Unigene29427\_Mf\_liverA, Unigene29729\_Mf\_liverA, Unigene29755\_Mf\_liverA, Unigene29756\_Mf\_liverA, Unigene29757\_Mf\_liverA, Unigene29768\_Mf\_liverA, Unigene29843\_Mf\_liverA, Unigene29894\_Mf\_liverA, Unigene29895\_Mf\_liverA, Unigene30159\_Mf\_liverA, Unigene30396\_Mf\_liverA, Unigene30397\_Mf\_liverA, Unigene30447\_Mf\_liverA, Unigene30666\_Mf\_liverA, Unigene30667\_Mf\_liverA, Unigene30936\_Mf\_liverA, Unigene30939\_Mf\_liverA, Unigene30954\_Mf\_liverA, Unigene31145\_Mf\_liverA, Unigene31227\_Mf\_liverA, Unigene31295\_Mf\_liverA, Unigene31299\_Mf\_liverA, Unigene31329\_Mf\_liverA, Unigene31330\_Mf\_liverA, Unigene31368\_Mf\_liverA, Unigene31626\_Mf\_liverA, Unigene31694\_Mf\_liverA, Unigene31814\_Mf\_liverA, Unigene31845\_Mf\_liverA, Unigene31846\_Mf\_liverA, Unigene31981\_Mf\_liverA, Unigene32033\_Mf\_liverA, Unigene32034\_Mf\_liverA, Unigene32113\_Mf\_liverA, Unigene32114\_Mf\_liverA, Unigene32350\_Mf\_liverA, Unigene32351\_Mf\_liverA, Unigene32388\_Mf\_liverA, Unigene32412\_Mf\_liverA, Unigene32432\_Mf\_liverA, Unigene32511\_Mf\_liverA, Unigene32512\_Mf\_liverA, Unigene32589\_Mf\_liverA, Unigene32620\_Mf\_liverA, Unigene32724\_Mf\_liverA, Unigene3277\_Mf\_liverA, Unigene32997\_Mf\_liverA, Unigene33221\_Mf\_liverA, Unigene33222\_Mf\_liverA, Unigene33646\_Mf\_liverA, Unigene33694\_Mf\_liverA, Unigene33820\_Mf\_liverA, Unigene33894\_Mf\_liverA, Unigene34052\_Mf\_liverA, Unigene34576\_Mf\_liverA, Unigene34593\_Mf\_liverA, Unigene34594\_Mf\_liverA, Unigene34600\_Mf\_liverA, Unigene34656\_Mf\_liverA, Unigene34678\_Mf\_liverA, Unigene34679\_Mf\_liverA, Unigene34777\_Mf\_liverA, Unigene35094\_Mf\_liverA, Unigene35149\_Mf\_liverA, Unigene35210\_Mf\_liverA, Unigene35211\_Mf\_liverA, Unigene35373\_Mf\_liverA, Unigene35374\_Mf\_liverA, Unigene35375\_Mf\_liverA, Unigene35376\_Mf\_liverA, Unigene35377\_Mf\_liverA, Unigene35378\_Mf\_liverA, Unigene35379\_Mf\_liverA, Unigene35380\_Mf\_liverA, Unigene35423\_Mf\_liverA, Unigene35500\_Mf\_liverA, Unigene35685\_Mf\_liverA, Unigene35686\_Mf\_liverA, Unigene35687\_Mf\_liverA, Unigene35688\_Mf\_liverA, Unigene35689\_Mf\_liverA, Unigene35748\_Mf\_liverA, Unigene35749\_Mf\_liverA, Unigene35816\_Mf\_liverA, Unigene35831\_Mf\_liverA, Unigene3687\_Mf\_liverA, Unigene36922\_Mf\_liverA, Unigene36936\_Mf\_liverA, Unigene36941\_Mf\_liverA, Unigene37033\_Mf\_liverA, Unigene37048\_Mf\_liverA, Unigene37050\_Mf\_liverA, Unigene37088\_Mf\_liverA, Unigene37173\_Mf\_liverA, Unigene37195\_Mf\_liverA, Unigene37236\_Mf\_liverA, Unigene37264\_Mf\_liverA, Unigene37266\_Mf\_liverA, Unigene37277\_Mf\_liverA, Unigene37339\_Mf\_liverA, Unigene37346\_Mf\_liverA, Unigene37421\_Mf\_liverA, Unigene37430\_Mf\_liverA, Unigene37504\_Mf\_liverA, Unigene37518\_Mf\_liverA, Unigene37519\_Mf\_liverA, Unigene37535\_Mf\_liverA, Unigene37569\_Mf\_liverA, Unigene37603\_Mf\_liverA, Unigene37660\_Mf\_liverA, Unigene3771\_Mf\_liverA, Unigene37821\_Mf\_liverA, Unigene38083\_Mf\_liverA, Unigene38242\_Mf\_liverA, Unigene38249\_Mf\_liverA, Unigene38353\_Mf\_liverA, Unigene38569\_Mf\_liverA, Unigene40343\_Mf\_liverA, Unigene40532\_Mf\_liverA, Unigene40902\_Mf\_liverA, Unigene4133\_Mf\_liverA, Unigene41342\_Mf\_liverA, Unigene41886\_Mf\_liverA, Unigene42049\_Mf\_liverA, Unigene42435\_Mf\_liverA, Unigene42852\_Mf\_liverA, Unigene43504\_Mf\_liverA, Unigene4364\_Mf\_liverA, Unigene4674\_Mf\_liverA, Unigene46821\_Mf\_liverA, Unigene47557\_Mf\_liverA, Unigene4827\_Mf\_liverA, Unigene48462\_Mf\_liverA, Unigene48726\_Mf\_liverA, Unigene5053\_Mf\_liverA, Unigene5193\_Mf\_liverA, Unigene5249\_Mf\_liverA, Unigene5355\_Mf\_liverA, Unigene539\_Mf\_liverA, Unigene5400\_Mf\_liverA, Unigene5616\_Mf\_liverA, Unigene5668\_Mf\_liverA, Unigene5819\_Mf\_liverA, Unigene5831\_Mf\_liverA, Unigene5870\_Mf\_liverA, Unigene5898\_Mf\_liverA, Unigene6267\_Mf\_liverA, Unigene6268\_Mf\_liverA, Unigene6629\_Mf\_liverA, Unigene7004\_Mf\_liverA, Unigene7309\_Mf\_liverA, Unigene7341\_Mf\_liverA, Unigene7381\_Mf\_liverA, Unigene750\_Mf\_liverA, Unigene754\_Mf\_liverA, Unigene83\_Mf\_liverA, Unigene868\_Mf\_liverA, Unigene8969\_Mf\_liverA, Unigene9654\_Mf\_liverA |
| 16 | Chemokine signaling pathway | CL1034.Contig2\_Mf\_liverA, CL1114.Contig1\_Mf\_liverA, CL1114.Contig2\_Mf\_liverA, CL1180.Contig10\_Mf\_liverA, CL1180.Contig1\_Mf\_liverA, CL1180.Contig2\_Mf\_liverA, CL1180.Contig5\_Mf\_liverA, CL1180.Contig8\_Mf\_liverA, CL1180.Contig9\_Mf\_liverA, CL1197.Contig1\_Mf\_liverA, CL1197.Contig2\_Mf\_liverA, CL1202.Contig1\_Mf\_liverA, CL1207.Contig1\_Mf\_liverA, CL1207.Contig2\_Mf\_liverA, CL1365.Contig2\_Mf\_liverA, CL1377.Contig1\_Mf\_liverA, CL1377.Contig2\_Mf\_liverA, CL1598.Contig1\_Mf\_liverA, CL1598.Contig2\_Mf\_liverA, CL1598.Contig3\_Mf\_liverA, CL1598.Contig4\_Mf\_liverA, CL1598.Contig5\_Mf\_liverA, CL1598.Contig6\_Mf\_liverA, CL1687.Contig1\_Mf\_liverA, CL1687.Contig2\_Mf\_liverA, CL1727.Contig2\_Mf\_liverA, CL1747.Contig1\_Mf\_liverA, CL1865.Contig2\_Mf\_liverA, CL1883.Contig7\_Mf\_liverA, CL1958.Contig1\_Mf\_liverA, CL1970.Contig1\_Mf\_liverA, CL1970.Contig3\_Mf\_liverA, CL1970.Contig6\_Mf\_liverA, CL2021.Contig1\_Mf\_liverA, CL2021.Contig2\_Mf\_liverA, CL2110.Contig1\_Mf\_liverA, CL2110.Contig2\_Mf\_liverA, CL2110.Contig3\_Mf\_liverA, CL2110.Contig4\_Mf\_liverA, CL213.Contig1\_Mf\_liverA, CL213.Contig2\_Mf\_liverA, CL2144.Contig1\_Mf\_liverA, CL2144.Contig2\_Mf\_liverA, CL2166.Contig1\_Mf\_liverA, CL218.Contig1\_Mf\_liverA, CL218.Contig2\_Mf\_liverA, CL2191.Contig1\_Mf\_liverA, CL2191.Contig2\_Mf\_liverA, CL2259.Contig1\_Mf\_liverA, CL2259.Contig2\_Mf\_liverA, CL226.Contig3\_Mf\_liverA, CL2376.Contig1\_Mf\_liverA, CL2400.Contig1\_Mf\_liverA, CL2400.Contig2\_Mf\_liverA, CL2405.Contig1\_Mf\_liverA, CL2405.Contig2\_Mf\_liverA, CL2483.Contig1\_Mf\_liverA, CL2483.Contig2\_Mf\_liverA, CL2546.Contig1\_Mf\_liverA, CL2546.Contig2\_Mf\_liverA, CL2557.Contig1\_Mf\_liverA, CL2557.Contig2\_Mf\_liverA, CL2796.Contig1\_Mf\_liverA, CL2861.Contig1\_Mf\_liverA, CL2869.Contig1\_Mf\_liverA, CL2869.Contig2\_Mf\_liverA, CL2869.Contig3\_Mf\_liverA, CL2869.Contig4\_Mf\_liverA, CL2871.Contig1\_Mf\_liverA, CL2876.Contig1\_Mf\_liverA, CL2898.Contig1\_Mf\_liverA, CL2898.Contig2\_Mf\_liverA, CL2993.Contig1\_Mf\_liverA, CL2993.Contig2\_Mf\_liverA, CL2999.Contig1\_Mf\_liverA, CL2999.Contig2\_Mf\_liverA, CL3125.Contig1\_Mf\_liverA, CL3147.Contig1\_Mf\_liverA, CL3168.Contig1\_Mf\_liverA, CL3168.Contig2\_Mf\_liverA, CL3223.Contig2\_Mf\_liverA, CL3231.Contig1\_Mf\_liverA, CL3231.Contig2\_Mf\_liverA, CL3388.Contig3\_Mf\_liverA, CL3398.Contig1\_Mf\_liverA, CL3398.Contig2\_Mf\_liverA, CL3515.Contig1\_Mf\_liverA, CL3515.Contig2\_Mf\_liverA, CL3567.Contig1\_Mf\_liverA, CL3567.Contig2\_Mf\_liverA, CL3631.Contig1\_Mf\_liverA, CL3631.Contig2\_Mf\_liverA, CL3637.Contig1\_Mf\_liverA, CL3715.Contig3\_Mf\_liverA, CL3800.Contig1\_Mf\_liverA, CL3800.Contig2\_Mf\_liverA, CL3861.Contig1\_Mf\_liverA, CL3861.Contig2\_Mf\_liverA, CL3930.Contig1\_Mf\_liverA, CL3930.Contig2\_Mf\_liverA, CL395.Contig1\_Mf\_liverA, CL395.Contig2\_Mf\_liverA, CL395.Contig3\_Mf\_liverA, CL4055.Contig1\_Mf\_liverA, CL4055.Contig2\_Mf\_liverA, CL410.Contig13\_Mf\_liverA, CL410.Contig14\_Mf\_liverA, CL410.Contig15\_Mf\_liverA, CL410.Contig16\_Mf\_liverA, CL410.Contig17\_Mf\_liverA, CL410.Contig9\_Mf\_liverA, CL4147.Contig2\_Mf\_liverA, CL4147.Contig3\_Mf\_liverA, CL416.Contig1\_Mf\_liverA, CL416.Contig2\_Mf\_liverA, CL416.Contig3\_Mf\_liverA, CL416.Contig4\_Mf\_liverA, CL416.Contig5\_Mf\_liverA, CL416.Contig6\_Mf\_liverA, CL416.Contig7\_Mf\_liverA, CL416.Contig8\_Mf\_liverA, CL4264.Contig1\_Mf\_liverA, CL4264.Contig2\_Mf\_liverA, CL4335.Contig1\_Mf\_liverA, CL4335.Contig2\_Mf\_liverA, CL4406.Contig1\_Mf\_liverA, CL444.Contig1\_Mf\_liverA, CL444.Contig2\_Mf\_liverA, CL4459.Contig1\_Mf\_liverA, CL4459.Contig2\_Mf\_liverA, CL4518.Contig3\_Mf\_liverA, CL4598.Contig4\_Mf\_liverA, CL4616.Contig1\_Mf\_liverA, CL4616.Contig2\_Mf\_liverA, CL4655.Contig1\_Mf\_liverA, CL4655.Contig2\_Mf\_liverA, CL4655.Contig3\_Mf\_liverA, CL4664.Contig1\_Mf\_liverA, CL4664.Contig2\_Mf\_liverA, CL4731.Contig1\_Mf\_liverA, CL4731.Contig2\_Mf\_liverA, CL4772.Contig1\_Mf\_liverA, CL4784.Contig2\_Mf\_liverA, CL4846.Contig1\_Mf\_liverA, CL4846.Contig2\_Mf\_liverA, CL4848.Contig1\_Mf\_liverA, CL4848.Contig2\_Mf\_liverA, CL4892.Contig1\_Mf\_liverA, CL4892.Contig2\_Mf\_liverA, CL4918.Contig1\_Mf\_liverA, CL4918.Contig2\_Mf\_liverA, CL4919.Contig1\_Mf\_liverA, CL4919.Contig2\_Mf\_liverA, CL4952.Contig1\_Mf\_liverA, CL4952.Contig2\_Mf\_liverA, CL4957.Contig1\_Mf\_liverA, CL4957.Contig2\_Mf\_liverA, CL5191.Contig1\_Mf\_liverA, CL5191.Contig2\_Mf\_liverA, CL5254.Contig1\_Mf\_liverA, CL5254.Contig2\_Mf\_liverA, CL5261.Contig1\_Mf\_liverA, CL5264.Contig1\_Mf\_liverA, CL5264.Contig2\_Mf\_liverA, CL5406.Contig2\_Mf\_liverA, CL5416.Contig1\_Mf\_liverA, CL5416.Contig2\_Mf\_liverA, CL5501.Contig1\_Mf\_liverA, CL5501.Contig2\_Mf\_liverA, CL5527.Contig1\_Mf\_liverA, CL5527.Contig2\_Mf\_liverA, CL5595.Contig1\_Mf\_liverA, CL5595.Contig2\_Mf\_liverA, CL5672.Contig1\_Mf\_liverA, CL5672.Contig3\_Mf\_liverA, CL5896.Contig1\_Mf\_liverA, CL5896.Contig2\_Mf\_liverA, CL5955.Contig1\_Mf\_liverA, CL63.Contig1\_Mf\_liverA, CL632.Contig1\_Mf\_liverA, CL632.Contig2\_Mf\_liverA, CL632.Contig3\_Mf\_liverA, CL632.Contig4\_Mf\_liverA, CL683.Contig1\_Mf\_liverA, CL683.Contig2\_Mf\_liverA, CL683.Contig3\_Mf\_liverA, CL774.Contig10\_Mf\_liverA, CL774.Contig11\_Mf\_liverA, CL774.Contig12\_Mf\_liverA, CL774.Contig3\_Mf\_liverA, CL774.Contig4\_Mf\_liverA, CL774.Contig5\_Mf\_liverA, CL774.Contig6\_Mf\_liverA, CL774.Contig9\_Mf\_liverA, CL777.Contig14\_Mf\_liverA, CL777.Contig15\_Mf\_liverA, CL890.Contig1\_Mf\_liverA, CL890.Contig2\_Mf\_liverA, Unigene10018\_Mf\_liverA, Unigene10241\_Mf\_liverA, Unigene10249\_Mf\_liverA, Unigene10279\_Mf\_liverA, Unigene10481\_Mf\_liverA, Unigene10482\_Mf\_liverA, Unigene1055\_Mf\_liverA, Unigene1137\_Mf\_liverA, Unigene11534\_Mf\_liverA, Unigene11737\_Mf\_liverA, Unigene11782\_Mf\_liverA, Unigene11890\_Mf\_liverA, Unigene1200\_Mf\_liverA, Unigene12061\_Mf\_liverA, Unigene12153\_Mf\_liverA, Unigene12161\_Mf\_liverA, Unigene12187\_Mf\_liverA, Unigene12503\_Mf\_liverA, Unigene12724\_Mf\_liverA, Unigene12877\_Mf\_liverA, Unigene13396\_Mf\_liverA, Unigene13779\_Mf\_liverA, Unigene1377\_Mf\_liverA, Unigene13894\_Mf\_liverA, Unigene13918\_Mf\_liverA, Unigene14093\_Mf\_liverA, Unigene14094\_Mf\_liverA, Unigene14168\_Mf\_liverA, Unigene14184\_Mf\_liverA, Unigene14427\_Mf\_liverA, Unigene14428\_Mf\_liverA, Unigene14588\_Mf\_liverA, Unigene14759\_Mf\_liverA, Unigene14760\_Mf\_liverA, Unigene14807\_Mf\_liverA, Unigene15125\_Mf\_liverA, Unigene15295\_Mf\_liverA, Unigene15296\_Mf\_liverA, Unigene15338\_Mf\_liverA, Unigene15339\_Mf\_liverA, Unigene15364\_Mf\_liverA, Unigene15572\_Mf\_liverA, Unigene15630\_Mf\_liverA, Unigene16458\_Mf\_liverA, Unigene16952\_Mf\_liverA, Unigene16960\_Mf\_liverA, Unigene17035\_Mf\_liverA, Unigene17220\_Mf\_liverA, Unigene17518\_Mf\_liverA, Unigene17534\_Mf\_liverA, Unigene1806\_Mf\_liverA, Unigene18177\_Mf\_liverA, Unigene18428\_Mf\_liverA, Unigene18792\_Mf\_liverA, Unigene19041\_Mf\_liverA, Unigene19044\_Mf\_liverA, Unigene19045\_Mf\_liverA, Unigene19881\_Mf\_liverA, Unigene19882\_Mf\_liverA, Unigene19893\_Mf\_liverA, Unigene19894\_Mf\_liverA, Unigene20214\_Mf\_liverA, Unigene20249\_Mf\_liverA, Unigene20472\_Mf\_liverA, Unigene20795\_Mf\_liverA, Unigene21276\_Mf\_liverA, Unigene22165\_Mf\_liverA, Unigene22166\_Mf\_liverA, Unigene22167\_Mf\_liverA, Unigene22168\_Mf\_liverA, Unigene22396\_Mf\_liverA, Unigene23108\_Mf\_liverA, Unigene23266\_Mf\_liverA, Unigene23341\_Mf\_liverA, Unigene23920\_Mf\_liverA, Unigene23922\_Mf\_liverA, Unigene23923\_Mf\_liverA, Unigene23924\_Mf\_liverA, Unigene23934\_Mf\_liverA, Unigene23935\_Mf\_liverA, Unigene23936\_Mf\_liverA, Unigene23937\_Mf\_liverA, Unigene23938\_Mf\_liverA, Unigene24065\_Mf\_liverA, Unigene24068\_Mf\_liverA, Unigene24069\_Mf\_liverA, Unigene24334\_Mf\_liverA, Unigene24335\_Mf\_liverA, Unigene24336\_Mf\_liverA, Unigene24547\_Mf\_liverA, Unigene24576\_Mf\_liverA, Unigene24763\_Mf\_liverA, Unigene24804\_Mf\_liverA, Unigene25119\_Mf\_liverA, Unigene25172\_Mf\_liverA, Unigene2521\_Mf\_liverA, Unigene25404\_Mf\_liverA, Unigene25447\_Mf\_liverA, Unigene25454\_Mf\_liverA, Unigene25826\_Mf\_liverA, Unigene25853\_Mf\_liverA, Unigene2587\_Mf\_liverA, Unigene26195\_Mf\_liverA, Unigene26305\_Mf\_liverA, Unigene26306\_Mf\_liverA, Unigene26307\_Mf\_liverA, Unigene26573\_Mf\_liverA, Unigene26574\_Mf\_liverA, Unigene26729\_Mf\_liverA, Unigene26730\_Mf\_liverA, Unigene26923\_Mf\_liverA, Unigene27224\_Mf\_liverA, Unigene27225\_Mf\_liverA, Unigene27248\_Mf\_liverA, Unigene27249\_Mf\_liverA, Unigene27260\_Mf\_liverA, Unigene27261\_Mf\_liverA, Unigene27323\_Mf\_liverA, Unigene27343\_Mf\_liverA, Unigene27344\_Mf\_liverA, Unigene2829\_Mf\_liverA, Unigene28341\_Mf\_liverA, Unigene28342\_Mf\_liverA, Unigene28504\_Mf\_liverA, Unigene28505\_Mf\_liverA, Unigene28621\_Mf\_liverA, Unigene28989\_Mf\_liverA, Unigene29041\_Mf\_liverA, Unigene29095\_Mf\_liverA, Unigene29676\_Mf\_liverA, Unigene29677\_Mf\_liverA, Unigene29678\_Mf\_liverA, Unigene29725\_Mf\_liverA, Unigene29850\_Mf\_liverA, Unigene29884\_Mf\_liverA, Unigene29885\_Mf\_liverA, Unigene30099\_Mf\_liverA, Unigene30135\_Mf\_liverA, Unigene30192\_Mf\_liverA, Unigene30321\_Mf\_liverA, Unigene30322\_Mf\_liverA, Unigene30411\_Mf\_liverA, Unigene30412\_Mf\_liverA, Unigene30618\_Mf\_liverA, Unigene30619\_Mf\_liverA, Unigene30621\_Mf\_liverA, Unigene30680\_Mf\_liverA, Unigene30877\_Mf\_liverA, Unigene30907\_Mf\_liverA, Unigene30908\_Mf\_liverA, Unigene30909\_Mf\_liverA, Unigene31109\_Mf\_liverA, Unigene31249\_Mf\_liverA, Unigene31251\_Mf\_liverA, Unigene31303\_Mf\_liverA, Unigene31368\_Mf\_liverA, Unigene31424\_Mf\_liverA, Unigene31665\_Mf\_liverA, Unigene32002\_Mf\_liverA, Unigene32003\_Mf\_liverA, Unigene32125\_Mf\_liverA, Unigene3215\_Mf\_liverA, Unigene32396\_Mf\_liverA, Unigene32659\_Mf\_liverA, Unigene32879\_Mf\_liverA, Unigene32882\_Mf\_liverA, Unigene32883\_Mf\_liverA, Unigene32895\_Mf\_liverA, Unigene32935\_Mf\_liverA, Unigene33122\_Mf\_liverA, Unigene33163\_Mf\_liverA, Unigene33165\_Mf\_liverA, Unigene33247\_Mf\_liverA, Unigene33352\_Mf\_liverA, Unigene33692\_Mf\_liverA, Unigene34055\_Mf\_liverA, Unigene34056\_Mf\_liverA, Unigene34215\_Mf\_liverA, Unigene34217\_Mf\_liverA, Unigene34218\_Mf\_liverA, Unigene34930\_Mf\_liverA, Unigene35104\_Mf\_liverA, Unigene35106\_Mf\_liverA, Unigene35108\_Mf\_liverA, Unigene3514\_Mf\_liverA, Unigene35322\_Mf\_liverA, Unigene35323\_Mf\_liverA, Unigene35344\_Mf\_liverA, Unigene35370\_Mf\_liverA, Unigene35570\_Mf\_liverA, Unigene35571\_Mf\_liverA, Unigene35572\_Mf\_liverA, Unigene35573\_Mf\_liverA, Unigene3568\_Mf\_liverA, Unigene35748\_Mf\_liverA, Unigene36413\_Mf\_liverA, Unigene36414\_Mf\_liverA, Unigene36417\_Mf\_liverA, Unigene36418\_Mf\_liverA, Unigene36420\_Mf\_liverA, Unigene36650\_Mf\_liverA, Unigene36748\_Mf\_liverA, Unigene36818\_Mf\_liverA, Unigene36907\_Mf\_liverA, Unigene37099\_Mf\_liverA, Unigene3712\_Mf\_liverA, Unigene37147\_Mf\_liverA, Unigene37176\_Mf\_liverA, Unigene37179\_Mf\_liverA, Unigene37341\_Mf\_liverA, Unigene373\_Mf\_liverA, Unigene37412\_Mf\_liverA, Unigene37683\_Mf\_liverA, Unigene37771\_Mf\_liverA, Unigene37802\_Mf\_liverA, Unigene3798\_Mf\_liverA, Unigene38110\_Mf\_liverA, Unigene38219\_Mf\_liverA, Unigene38353\_Mf\_liverA, Unigene38520\_Mf\_liverA, Unigene38681\_Mf\_liverA, Unigene38689\_Mf\_liverA, Unigene39099\_Mf\_liverA, Unigene40020\_Mf\_liverA, Unigene40369\_Mf\_liverA, Unigene40388\_Mf\_liverA, Unigene4133\_Mf\_liverA, Unigene41457\_Mf\_liverA, Unigene41505\_Mf\_liverA, Unigene41527\_Mf\_liverA, Unigene41586\_Mf\_liverA, Unigene41600\_Mf\_liverA, Unigene41703\_Mf\_liverA, Unigene41758\_Mf\_liverA, Unigene41957\_Mf\_liverA, Unigene4226\_Mf\_liverA, Unigene42272\_Mf\_liverA, Unigene42335\_Mf\_liverA, Unigene43225\_Mf\_liverA, Unigene43339\_Mf\_liverA, Unigene43533\_Mf\_liverA, Unigene43599\_Mf\_liverA, Unigene4422\_Mf\_liverA, Unigene44372\_Mf\_liverA, Unigene44395\_Mf\_liverA, Unigene4459\_Mf\_liverA, Unigene45138\_Mf\_liverA, Unigene45164\_Mf\_liverA, Unigene45329\_Mf\_liverA, Unigene45708\_Mf\_liverA, Unigene45728\_Mf\_liverA, Unigene4630\_Mf\_liverA, Unigene46368\_Mf\_liverA, Unigene46484\_Mf\_liverA, Unigene4684\_Mf\_liverA, Unigene47034\_Mf\_liverA, Unigene47212\_Mf\_liverA, Unigene47554\_Mf\_liverA, Unigene47837\_Mf\_liverA, Unigene47869\_Mf\_liverA, Unigene47936\_Mf\_liverA, Unigene48313\_Mf\_liverA, Unigene48539\_Mf\_liverA, Unigene48762\_Mf\_liverA, Unigene48879\_Mf\_liverA, Unigene49422\_Mf\_liverA, Unigene49797\_Mf\_liverA, Unigene49889\_Mf\_liverA, Unigene50319\_Mf\_liverA, Unigene50360\_Mf\_liverA, Unigene50600\_Mf\_liverA, Unigene51211\_Mf\_liverA, Unigene51532\_Mf\_liverA, Unigene51614\_Mf\_liverA, Unigene5226\_Mf\_liverA, Unigene5274\_Mf\_liverA, Unigene5434\_Mf\_liverA, Unigene5484\_Mf\_liverA, Unigene550\_Mf\_liverA, Unigene5648\_Mf\_liverA, Unigene5680\_Mf\_liverA, Unigene583\_Mf\_liverA, Unigene5875\_Mf\_liverA, Unigene591\_Mf\_liverA, Unigene6151\_Mf\_liverA, Unigene67\_Mf\_liverA, Unigene6816\_Mf\_liverA, Unigene6817\_Mf\_liverA, Unigene682\_Mf\_liverA, Unigene6857\_Mf\_liverA, Unigene6914\_Mf\_liverA, Unigene7477\_Mf\_liverA, Unigene7478\_Mf\_liverA, Unigene7950\_Mf\_liverA, Unigene8277\_Mf\_liverA, Unigene855\_Mf\_liverA, Unigene8624\_Mf\_liverA, Unigene8725\_Mf\_liverA, Unigene8740\_Mf\_liverA, Unigene8878\_Mf\_liverA, Unigene9067\_Mf\_liverA, Unigene9197\_Mf\_liverA, Unigene919\_Mf\_liverA, Unigene9201\_Mf\_liverA, Unigene9499\_Mf\_liverA, Unigene9621\_Mf\_liverA, Unigene9650\_Mf\_liverA, Unigene9762\_Mf\_liverA, Unigene9893\_Mf\_liverA |
| 17 | Vascular smooth muscle contraction | CL1013.Contig5\_Mf\_liverA, CL1235.Contig1\_Mf\_liverA, CL1235.Contig2\_Mf\_liverA, CL1235.Contig3\_Mf\_liverA, CL153.Contig1\_Mf\_liverA, CL153.Contig2\_Mf\_liverA, CL153.Contig3\_Mf\_liverA, CL1540.Contig1\_Mf\_liverA, CL1540.Contig2\_Mf\_liverA, CL1569.Contig1\_Mf\_liverA, CL1569.Contig2\_Mf\_liverA, CL1569.Contig3\_Mf\_liverA, CL1569.Contig4\_Mf\_liverA, CL1569.Contig5\_Mf\_liverA, CL1619.Contig2\_Mf\_liverA, CL1619.Contig3\_Mf\_liverA, CL1619.Contig4\_Mf\_liverA, CL1622.Contig3\_Mf\_liverA, CL1622.Contig4\_Mf\_liverA, CL1622.Contig6\_Mf\_liverA, CL1622.Contig8\_Mf\_liverA, CL1622.Contig9\_Mf\_liverA, CL1686.Contig10\_Mf\_liverA, CL1686.Contig1\_Mf\_liverA, CL1686.Contig2\_Mf\_liverA, CL1686.Contig3\_Mf\_liverA, CL1686.Contig4\_Mf\_liverA, CL1686.Contig5\_Mf\_liverA, CL1686.Contig6\_Mf\_liverA, CL1686.Contig7\_Mf\_liverA, CL1686.Contig8\_Mf\_liverA, CL1686.Contig9\_Mf\_liverA, CL1735.Contig3\_Mf\_liverA, CL1735.Contig4\_Mf\_liverA, CL175.Contig1\_Mf\_liverA, CL175.Contig2\_Mf\_liverA, CL175.Contig3\_Mf\_liverA, CL1784.Contig1\_Mf\_liverA, CL1784.Contig2\_Mf\_liverA, CL1806.Contig1\_Mf\_liverA, CL1806.Contig2\_Mf\_liverA, CL1806.Contig3\_Mf\_liverA, CL1806.Contig4\_Mf\_liverA, CL1806.Contig5\_Mf\_liverA, CL1806.Contig6\_Mf\_liverA, CL1806.Contig7\_Mf\_liverA, CL1806.Contig8\_Mf\_liverA, CL1936.Contig1\_Mf\_liverA, CL1936.Contig2\_Mf\_liverA, CL1995.Contig1\_Mf\_liverA, CL2048.Contig1\_Mf\_liverA, CL2048.Contig2\_Mf\_liverA, CL2048.Contig3\_Mf\_liverA, CL2050.Contig1\_Mf\_liverA, CL2050.Contig2\_Mf\_liverA, CL2050.Contig3\_Mf\_liverA, CL2050.Contig4\_Mf\_liverA, CL2050.Contig5\_Mf\_liverA, CL207.Contig1\_Mf\_liverA, CL207.Contig2\_Mf\_liverA, CL2181.Contig1\_Mf\_liverA, CL2181.Contig2\_Mf\_liverA, CL2181.Contig3\_Mf\_liverA, CL2181.Contig4\_Mf\_liverA, CL2181.Contig5\_Mf\_liverA, CL2181.Contig6\_Mf\_liverA, CL2230.Contig1\_Mf\_liverA, CL2230.Contig2\_Mf\_liverA, CL2292.Contig1\_Mf\_liverA, CL2378.Contig1\_Mf\_liverA, CL2378.Contig2\_Mf\_liverA, CL2378.Contig3\_Mf\_liverA, CL2378.Contig4\_Mf\_liverA, CL2546.Contig1\_Mf\_liverA, CL2546.Contig2\_Mf\_liverA, CL2551.Contig1\_Mf\_liverA, CL2553.Contig1\_Mf\_liverA, CL2603.Contig1\_Mf\_liverA, CL2603.Contig2\_Mf\_liverA, CL2603.Contig3\_Mf\_liverA, CL2603.Contig4\_Mf\_liverA, CL2639.Contig1\_Mf\_liverA, CL2737.Contig2\_Mf\_liverA, CL2737.Contig3\_Mf\_liverA, CL2737.Contig4\_Mf\_liverA, CL2737.Contig5\_Mf\_liverA, CL2737.Contig6\_Mf\_liverA, CL2753.Contig1\_Mf\_liverA, CL2753.Contig2\_Mf\_liverA, CL2784.Contig1\_Mf\_liverA, CL2784.Contig2\_Mf\_liverA, CL2876.Contig1\_Mf\_liverA, CL2963.Contig1\_Mf\_liverA, CL2975.Contig1\_Mf\_liverA, CL2975.Contig2\_Mf\_liverA, CL2975.Contig3\_Mf\_liverA, CL2975.Contig4\_Mf\_liverA, CL2999.Contig1\_Mf\_liverA, CL2999.Contig2\_Mf\_liverA, CL3031.Contig1\_Mf\_liverA, CL3031.Contig2\_Mf\_liverA, CL3125.Contig1\_Mf\_liverA, CL3168.Contig1\_Mf\_liverA, CL3168.Contig2\_Mf\_liverA, CL3252.Contig1\_Mf\_liverA, CL3252.Contig2\_Mf\_liverA, CL3253.Contig1\_Mf\_liverA, CL3253.Contig2\_Mf\_liverA, CL3253.Contig3\_Mf\_liverA, CL3253.Contig4\_Mf\_liverA, CL336.Contig10\_Mf\_liverA, CL336.Contig11\_Mf\_liverA, CL336.Contig12\_Mf\_liverA, CL336.Contig13\_Mf\_liverA, CL336.Contig14\_Mf\_liverA, CL336.Contig15\_Mf\_liverA, CL336.Contig16\_Mf\_liverA, CL336.Contig17\_Mf\_liverA, CL336.Contig1\_Mf\_liverA, CL336.Contig2\_Mf\_liverA, CL336.Contig3\_Mf\_liverA, CL336.Contig4\_Mf\_liverA, CL336.Contig6\_Mf\_liverA, CL336.Contig7\_Mf\_liverA, CL336.Contig8\_Mf\_liverA, CL336.Contig9\_Mf\_liverA, CL3534.Contig1\_Mf\_liverA, CL3534.Contig3\_Mf\_liverA, CL3534.Contig4\_Mf\_liverA, CL3539.Contig2\_Mf\_liverA, CL3631.Contig1\_Mf\_liverA, CL3631.Contig2\_Mf\_liverA, CL3656.Contig1\_Mf\_liverA, CL3656.Contig2\_Mf\_liverA, CL371.Contig1\_Mf\_liverA, CL371.Contig3\_Mf\_liverA, CL371.Contig5\_Mf\_liverA, CL3813.Contig1\_Mf\_liverA, CL3813.Contig2\_Mf\_liverA, CL3845.Contig1\_Mf\_liverA, CL3865.Contig1\_Mf\_liverA, CL3928.Contig1\_Mf\_liverA, CL3928.Contig2\_Mf\_liverA, CL3945.Contig1\_Mf\_liverA, CL4005.Contig1\_Mf\_liverA, CL4005.Contig2\_Mf\_liverA, CL4031.Contig1\_Mf\_liverA, CL4038.Contig1\_Mf\_liverA, CL4123.Contig1\_Mf\_liverA, CL4123.Contig2\_Mf\_liverA, CL4153.Contig2\_Mf\_liverA, CL4169.Contig1\_Mf\_liverA, CL4169.Contig2\_Mf\_liverA, CL4175.Contig1\_Mf\_liverA, CL4175.Contig2\_Mf\_liverA, CL4175.Contig3\_Mf\_liverA, CL4175.Contig4\_Mf\_liverA, CL4254.Contig1\_Mf\_liverA, CL4254.Contig2\_Mf\_liverA, CL4276.Contig1\_Mf\_liverA, CL4276.Contig2\_Mf\_liverA, CL4335.Contig1\_Mf\_liverA, CL4335.Contig2\_Mf\_liverA, CL4388.Contig1\_Mf\_liverA, CL4388.Contig2\_Mf\_liverA, CL4388.Contig3\_Mf\_liverA, CL4434.Contig1\_Mf\_liverA, CL4437.Contig2\_Mf\_liverA, CL4460.Contig1\_Mf\_liverA, CL4460.Contig2\_Mf\_liverA, CL4598.Contig3\_Mf\_liverA, CL4598.Contig4\_Mf\_liverA, CL4616.Contig1\_Mf\_liverA, CL4616.Contig2\_Mf\_liverA, CL462.Contig1\_Mf\_liverA, CL462.Contig2\_Mf\_liverA, CL462.Contig3\_Mf\_liverA, CL462.Contig4\_Mf\_liverA, CL462.Contig5\_Mf\_liverA, CL462.Contig6\_Mf\_liverA, CL462.Contig7\_Mf\_liverA, CL462.Contig8\_Mf\_liverA, CL4633.Contig1\_Mf\_liverA, CL4633.Contig2\_Mf\_liverA, CL4644.Contig1\_Mf\_liverA, CL4644.Contig2\_Mf\_liverA, CL4648.Contig1\_Mf\_liverA, CL4648.Contig2\_Mf\_liverA, CL4762.Contig1\_Mf\_liverA, CL4812.Contig2\_Mf\_liverA, CL4837.Contig1\_Mf\_liverA, CL4883.Contig2\_Mf\_liverA, CL4892.Contig1\_Mf\_liverA, CL4952.Contig1\_Mf\_liverA, CL4952.Contig2\_Mf\_liverA, CL496.Contig1\_Mf\_liverA, CL496.Contig2\_Mf\_liverA, CL496.Contig3\_Mf\_liverA, CL5048.Contig1\_Mf\_liverA, CL507.Contig1\_Mf\_liverA, CL510.Contig1\_Mf\_liverA, CL510.Contig2\_Mf\_liverA, CL5448.Contig1\_Mf\_liverA, CL5501.Contig1\_Mf\_liverA, CL5501.Contig2\_Mf\_liverA, CL5569.Contig1\_Mf\_liverA, CL5569.Contig2\_Mf\_liverA, CL561.Contig2\_Mf\_liverA, CL561.Contig3\_Mf\_liverA, CL5664.Contig1\_Mf\_liverA, CL5664.Contig2\_Mf\_liverA, CL5669.Contig1\_Mf\_liverA, CL5669.Contig2\_Mf\_liverA, CL5878.Contig2\_Mf\_liverA, CL5896.Contig1\_Mf\_liverA, CL5896.Contig2\_Mf\_liverA, CL63.Contig1\_Mf\_liverA, CL723.Contig1\_Mf\_liverA, CL723.Contig2\_Mf\_liverA, CL723.Contig3\_Mf\_liverA, CL779.Contig10\_Mf\_liverA, CL779.Contig12\_Mf\_liverA, CL779.Contig1\_Mf\_liverA, CL779.Contig3\_Mf\_liverA, CL779.Contig4\_Mf\_liverA, CL779.Contig6\_Mf\_liverA, CL779.Contig7\_Mf\_liverA, CL779.Contig9\_Mf\_liverA, CL807.Contig1\_Mf\_liverA, CL807.Contig2\_Mf\_liverA, CL807.Contig3\_Mf\_liverA, CL807.Contig4\_Mf\_liverA, CL807.Contig5\_Mf\_liverA, CL807.Contig6\_Mf\_liverA, CL807.Contig7\_Mf\_liverA, CL807.Contig8\_Mf\_liverA, CL851.Contig1\_Mf\_liverA, CL851.Contig2\_Mf\_liverA, CL863.Contig1\_Mf\_liverA, CL863.Contig2\_Mf\_liverA, CL863.Contig3\_Mf\_liverA, CL863.Contig4\_Mf\_liverA, CL882.Contig2\_Mf\_liverA, CL882.Contig3\_Mf\_liverA, CL882.Contig5\_Mf\_liverA, CL882.Contig6\_Mf\_liverA, Unigene1008\_Mf\_liverA, Unigene10341\_Mf\_liverA, Unigene1055\_Mf\_liverA, Unigene10889\_Mf\_liverA, Unigene11196\_Mf\_liverA, Unigene111\_Mf\_liverA, Unigene11737\_Mf\_liverA, Unigene11892\_Mf\_liverA, Unigene11\_Mf\_liverA, Unigene12153\_Mf\_liverA, Unigene12248\_Mf\_liverA, Unigene12503\_Mf\_liverA, Unigene12814\_Mf\_liverA, Unigene12877\_Mf\_liverA, Unigene13153\_Mf\_liverA, Unigene13535\_Mf\_liverA, Unigene13628\_Mf\_liverA, Unigene13779\_Mf\_liverA, Unigene139\_Mf\_liverA, Unigene140\_Mf\_liverA, Unigene14581\_Mf\_liverA, Unigene14807\_Mf\_liverA, Unigene15010\_Mf\_liverA, Unigene15125\_Mf\_liverA, Unigene15295\_Mf\_liverA, Unigene15296\_Mf\_liverA, Unigene15588\_Mf\_liverA, Unigene16355\_Mf\_liverA, Unigene16566\_Mf\_liverA, Unigene16574\_Mf\_liverA, Unigene16788\_Mf\_liverA, Unigene16789\_Mf\_liverA, Unigene17040\_Mf\_liverA, Unigene17069\_Mf\_liverA, Unigene17265\_Mf\_liverA, Unigene17266\_Mf\_liverA, Unigene17392\_Mf\_liverA, Unigene17455\_Mf\_liverA, Unigene17534\_Mf\_liverA, Unigene18428\_Mf\_liverA, Unigene18941\_Mf\_liverA, Unigene19044\_Mf\_liverA, Unigene19045\_Mf\_liverA, Unigene19386\_Mf\_liverA, Unigene19387\_Mf\_liverA, Unigene19388\_Mf\_liverA, Unigene19992\_Mf\_liverA, Unigene20108\_Mf\_liverA, Unigene20249\_Mf\_liverA, Unigene20795\_Mf\_liverA, Unigene20829\_Mf\_liverA, Unigene21275\_Mf\_liverA, Unigene21571\_Mf\_liverA, Unigene21622\_Mf\_liverA, Unigene21816\_Mf\_liverA, Unigene21927\_Mf\_liverA, Unigene21928\_Mf\_liverA, Unigene21972\_Mf\_liverA, Unigene22012\_Mf\_liverA, Unigene23108\_Mf\_liverA, Unigene23266\_Mf\_liverA, Unigene2338\_Mf\_liverA, Unigene23934\_Mf\_liverA, Unigene23935\_Mf\_liverA, Unigene23936\_Mf\_liverA, Unigene23937\_Mf\_liverA, Unigene23938\_Mf\_liverA, Unigene24068\_Mf\_liverA, Unigene24069\_Mf\_liverA, Unigene24334\_Mf\_liverA, Unigene24540\_Mf\_liverA, Unigene24707\_Mf\_liverA, Unigene24715\_Mf\_liverA, Unigene24716\_Mf\_liverA, Unigene24755\_Mf\_liverA, Unigene24791\_Mf\_liverA, Unigene24792\_Mf\_liverA, Unigene24909\_Mf\_liverA, Unigene25132\_Mf\_liverA, Unigene25200\_Mf\_liverA, Unigene25299\_Mf\_liverA, Unigene25300\_Mf\_liverA, Unigene2530\_Mf\_liverA, Unigene25341\_Mf\_liverA, Unigene25416\_Mf\_liverA, Unigene25733\_Mf\_liverA, Unigene25734\_Mf\_liverA, Unigene25853\_Mf\_liverA, Unigene26052\_Mf\_liverA, Unigene2613\_Mf\_liverA, Unigene26190\_Mf\_liverA, Unigene26406\_Mf\_liverA, Unigene26573\_Mf\_liverA, Unigene26574\_Mf\_liverA, Unigene26611\_Mf\_liverA, Unigene26712\_Mf\_liverA, Unigene26923\_Mf\_liverA, Unigene26951\_Mf\_liverA, Unigene26964\_Mf\_liverA, Unigene27309\_Mf\_liverA, Unigene27623\_Mf\_liverA, Unigene27633\_Mf\_liverA, Unigene27673\_Mf\_liverA, Unigene27868\_Mf\_liverA, Unigene27939\_Mf\_liverA, Unigene28185\_Mf\_liverA, Unigene28491\_Mf\_liverA, Unigene28621\_Mf\_liverA, Unigene29119\_Mf\_liverA, Unigene29120\_Mf\_liverA, Unigene29359\_Mf\_liverA, Unigene29360\_Mf\_liverA, Unigene29363\_Mf\_liverA, Unigene29426\_Mf\_liverA, Unigene29670\_Mf\_liverA, Unigene29676\_Mf\_liverA, Unigene29677\_Mf\_liverA, Unigene29678\_Mf\_liverA, Unigene29884\_Mf\_liverA, Unigene29885\_Mf\_liverA, Unigene30134\_Mf\_liverA, Unigene30135\_Mf\_liverA, Unigene30209\_Mf\_liverA, Unigene30210\_Mf\_liverA, Unigene30211\_Mf\_liverA, Unigene30212\_Mf\_liverA, Unigene30256\_Mf\_liverA, Unigene30680\_Mf\_liverA, Unigene30776\_Mf\_liverA, Unigene30839\_Mf\_liverA, Unigene30873\_Mf\_liverA, Unigene30907\_Mf\_liverA, Unigene30908\_Mf\_liverA, Unigene30909\_Mf\_liverA, Unigene30944\_Mf\_liverA, Unigene31276\_Mf\_liverA, Unigene31277\_Mf\_liverA, Unigene31278\_Mf\_liverA, Unigene31440\_Mf\_liverA, Unigene31487\_Mf\_liverA, Unigene31613\_Mf\_liverA, Unigene31770\_Mf\_liverA, Unigene31968\_Mf\_liverA, Unigene32097\_Mf\_liverA, Unigene3215\_Mf\_liverA, Unigene32168\_Mf\_liverA, Unigene32176\_Mf\_liverA, Unigene32183\_Mf\_liverA, Unigene32247\_Mf\_liverA, Unigene32248\_Mf\_liverA, Unigene322\_Mf\_liverA, Unigene32370\_Mf\_liverA, Unigene32396\_Mf\_liverA, Unigene323\_Mf\_liverA, Unigene32469\_Mf\_liverA, Unigene32583\_Mf\_liverA, Unigene32635\_Mf\_liverA, Unigene32636\_Mf\_liverA, Unigene3295\_Mf\_liverA, Unigene33000\_Mf\_liverA, Unigene33076\_Mf\_liverA, Unigene3308\_Mf\_liverA, Unigene33296\_Mf\_liverA, Unigene33364\_Mf\_liverA, Unigene33514\_Mf\_liverA, Unigene34036\_Mf\_liverA, Unigene34183\_Mf\_liverA, Unigene34253\_Mf\_liverA, Unigene34411\_Mf\_liverA, Unigene34582\_Mf\_liverA, Unigene34583\_Mf\_liverA, Unigene3460\_Mf\_liverA, Unigene35196\_Mf\_liverA, Unigene35358\_Mf\_liverA, Unigene35359\_Mf\_liverA, Unigene35600\_Mf\_liverA, Unigene35618\_Mf\_liverA, Unigene35875\_Mf\_liverA, Unigene36337\_Mf\_liverA, Unigene36748\_Mf\_liverA, Unigene36824\_Mf\_liverA, Unigene37017\_Mf\_liverA, Unigene37042\_Mf\_liverA, Unigene3712\_Mf\_liverA, Unigene37147\_Mf\_liverA, Unigene37232\_Mf\_liverA, Unigene37662\_Mf\_liverA, Unigene37683\_Mf\_liverA, Unigene38109\_Mf\_liverA, Unigene38319\_Mf\_liverA, Unigene38681\_Mf\_liverA, Unigene39099\_Mf\_liverA, Unigene39537\_Mf\_liverA, Unigene40223\_Mf\_liverA, Unigene40262\_Mf\_liverA, Unigene40388\_Mf\_liverA, Unigene40977\_Mf\_liverA, Unigene41505\_Mf\_liverA, Unigene41527\_Mf\_liverA, Unigene41703\_Mf\_liverA, Unigene41785\_Mf\_liverA, Unigene4226\_Mf\_liverA, Unigene43533\_Mf\_liverA, Unigene43534\_Mf\_liverA, Unigene43624\_Mf\_liverA, Unigene43848\_Mf\_liverA, Unigene43969\_Mf\_liverA, Unigene44025\_Mf\_liverA, Unigene44038\_Mf\_liverA, Unigene44137\_Mf\_liverA, Unigene44590\_Mf\_liverA, Unigene44886\_Mf\_liverA, Unigene45329\_Mf\_liverA, Unigene4556\_Mf\_liverA, Unigene4557\_Mf\_liverA, Unigene46352\_Mf\_liverA, Unigene46368\_Mf\_liverA, Unigene46492\_Mf\_liverA, Unigene46723\_Mf\_liverA, Unigene46793\_Mf\_liverA, Unigene4684\_Mf\_liverA, Unigene46920\_Mf\_liverA, Unigene47097\_Mf\_liverA, Unigene47209\_Mf\_liverA, Unigene47212\_Mf\_liverA, Unigene47869\_Mf\_liverA, Unigene47970\_Mf\_liverA, Unigene48313\_Mf\_liverA, Unigene48618\_Mf\_liverA, Unigene4863\_Mf\_liverA, Unigene48762\_Mf\_liverA, Unigene49990\_Mf\_liverA, Unigene50352\_Mf\_liverA, Unigene51614\_Mf\_liverA, Unigene5472\_Mf\_liverA, Unigene5484\_Mf\_liverA, Unigene557\_Mf\_liverA, Unigene5648\_Mf\_liverA, Unigene613\_Mf\_liverA, Unigene6249\_Mf\_liverA, Unigene676\_Mf\_liverA, Unigene67\_Mf\_liverA, Unigene6897\_Mf\_liverA, Unigene7129\_Mf\_liverA, Unigene7130\_Mf\_liverA, Unigene7897\_Mf\_liverA, Unigene8066\_Mf\_liverA, Unigene8102\_Mf\_liverA, Unigene8277\_Mf\_liverA, Unigene8498\_Mf\_liverA, Unigene855\_Mf\_liverA, Unigene8725\_Mf\_liverA, Unigene8907\_Mf\_liverA, Unigene9436\_Mf\_liverA, Unigene9621\_Mf\_liverA, Unigene9647\_Mf\_liverA, Unigene9650\_Mf\_liverA |
| 18 | Protein processing in endoplasmic reticulum | CL1051.Contig1\_Mf\_liverA, CL1051.Contig2\_Mf\_liverA, CL1051.Contig3\_Mf\_liverA, CL1051.Contig4\_Mf\_liverA, CL1051.Contig5\_Mf\_liverA, CL1051.Contig6\_Mf\_liverA, CL1064.Contig1\_Mf\_liverA, CL1092.Contig1\_Mf\_liverA, CL1108.Contig1\_Mf\_liverA, CL1108.Contig2\_Mf\_liverA, CL1108.Contig3\_Mf\_liverA, CL1147.Contig2\_Mf\_liverA, CL1147.Contig3\_Mf\_liverA, CL1159.Contig1\_Mf\_liverA, CL1159.Contig2\_Mf\_liverA, CL119.Contig1\_Mf\_liverA, CL119.Contig2\_Mf\_liverA, CL119.Contig3\_Mf\_liverA, CL119.Contig4\_Mf\_liverA, CL1232.Contig1\_Mf\_liverA, CL1232.Contig2\_Mf\_liverA, CL1265.Contig1\_Mf\_liverA, CL1273.Contig1\_Mf\_liverA, CL1291.Contig1\_Mf\_liverA, CL1291.Contig2\_Mf\_liverA, CL1410.Contig1\_Mf\_liverA, CL1410.Contig2\_Mf\_liverA, CL1410.Contig3\_Mf\_liverA, CL1410.Contig4\_Mf\_liverA, CL1437.Contig1\_Mf\_liverA, CL1437.Contig2\_Mf\_liverA, CL162.Contig1\_Mf\_liverA, CL162.Contig4\_Mf\_liverA, CL1640.Contig1\_Mf\_liverA, CL1640.Contig2\_Mf\_liverA, CL1640.Contig3\_Mf\_liverA, CL1640.Contig4\_Mf\_liverA, CL1640.Contig5\_Mf\_liverA, CL1665.Contig1\_Mf\_liverA, CL1665.Contig2\_Mf\_liverA, CL1765.Contig1\_Mf\_liverA, CL1765.Contig2\_Mf\_liverA, CL1765.Contig3\_Mf\_liverA, CL1805.Contig1\_Mf\_liverA, CL1805.Contig2\_Mf\_liverA, CL1806.Contig1\_Mf\_liverA, CL1806.Contig2\_Mf\_liverA, CL1806.Contig4\_Mf\_liverA, CL1806.Contig6\_Mf\_liverA, CL1809.Contig1\_Mf\_liverA, CL1809.Contig2\_Mf\_liverA, CL1813.Contig1\_Mf\_liverA, CL1813.Contig2\_Mf\_liverA, CL1823.Contig1\_Mf\_liverA, CL1956.Contig1\_Mf\_liverA, CL1956.Contig2\_Mf\_liverA, CL1984.Contig1\_Mf\_liverA, CL1984.Contig2\_Mf\_liverA, CL1998.Contig1\_Mf\_liverA, CL20.Contig1\_Mf\_liverA, CL20.Contig2\_Mf\_liverA, CL2085.Contig2\_Mf\_liverA, CL2179.Contig1\_Mf\_liverA, CL2253.Contig1\_Mf\_liverA, CL2294.Contig1\_Mf\_liverA, CL2294.Contig2\_Mf\_liverA, CL2371.Contig2\_Mf\_liverA, CL2443.Contig1\_Mf\_liverA, CL2443.Contig2\_Mf\_liverA, CL2443.Contig3\_Mf\_liverA, CL2443.Contig4\_Mf\_liverA, CL2501.Contig1\_Mf\_liverA, CL2501.Contig2\_Mf\_liverA, CL2501.Contig3\_Mf\_liverA, CL2502.Contig2\_Mf\_liverA, CL2502.Contig3\_Mf\_liverA, CL2580.Contig1\_Mf\_liverA, CL2580.Contig2\_Mf\_liverA, CL2580.Contig3\_Mf\_liverA, CL2580.Contig4\_Mf\_liverA, CL2580.Contig5\_Mf\_liverA, CL2655.Contig1\_Mf\_liverA, CL2655.Contig2\_Mf\_liverA, CL2732.Contig1\_Mf\_liverA, CL2732.Contig2\_Mf\_liverA, CL2732.Contig3\_Mf\_liverA, CL2732.Contig4\_Mf\_liverA, CL2736.Contig1\_Mf\_liverA, CL2736.Contig2\_Mf\_liverA, CL2736.Contig3\_Mf\_liverA, CL2774.Contig1\_Mf\_liverA, CL2774.Contig2\_Mf\_liverA, CL2807.Contig2\_Mf\_liverA, CL2877.Contig1\_Mf\_liverA, CL2877.Contig2\_Mf\_liverA, CL2893.Contig1\_Mf\_liverA, CL2893.Contig2\_Mf\_liverA, CL2895.Contig1\_Mf\_liverA, CL2895.Contig2\_Mf\_liverA, CL2920.Contig1\_Mf\_liverA, CL2920.Contig2\_Mf\_liverA, CL2920.Contig3\_Mf\_liverA, CL2920.Contig4\_Mf\_liverA, CL296.Contig1\_Mf\_liverA, CL296.Contig2\_Mf\_liverA, CL2975.Contig1\_Mf\_liverA, CL2975.Contig2\_Mf\_liverA, CL2975.Contig3\_Mf\_liverA, CL2975.Contig4\_Mf\_liverA, CL3026.Contig1\_Mf\_liverA, CL3026.Contig2\_Mf\_liverA, CL3039.Contig1\_Mf\_liverA, CL3039.Contig2\_Mf\_liverA, CL3039.Contig3\_Mf\_liverA, CL3081.Contig1\_Mf\_liverA, CL3104.Contig1\_Mf\_liverA, CL3104.Contig2\_Mf\_liverA, CL3148.Contig1\_Mf\_liverA, CL3148.Contig2\_Mf\_liverA, CL3149.Contig1\_Mf\_liverA, CL3149.Contig2\_Mf\_liverA, CL3149.Contig3\_Mf\_liverA, CL3149.Contig4\_Mf\_liverA, CL3149.Contig5\_Mf\_liverA, CL3149.Contig6\_Mf\_liverA, CL3152.Contig2\_Mf\_liverA, CL3173.Contig1\_Mf\_liverA, CL3173.Contig2\_Mf\_liverA, CL3220.Contig1\_Mf\_liverA, CL3220.Contig2\_Mf\_liverA, CL3220.Contig3\_Mf\_liverA, CL3220.Contig4\_Mf\_liverA, CL3288.Contig1\_Mf\_liverA, CL3288.Contig2\_Mf\_liverA, CL3288.Contig3\_Mf\_liverA, CL3311.Contig3\_Mf\_liverA, CL3330.Contig1\_Mf\_liverA, CL3330.Contig2\_Mf\_liverA, CL3349.Contig1\_Mf\_liverA, CL3349.Contig2\_Mf\_liverA, CL3408.Contig1\_Mf\_liverA, CL3408.Contig2\_Mf\_liverA, CL3569.Contig1\_Mf\_liverA, CL3569.Contig2\_Mf\_liverA, CL3569.Contig3\_Mf\_liverA, CL3616.Contig1\_Mf\_liverA, CL3616.Contig2\_Mf\_liverA, CL3680.Contig1\_Mf\_liverA, CL3680.Contig2\_Mf\_liverA, CL3680.Contig3\_Mf\_liverA, CL3719.Contig1\_Mf\_liverA, CL3719.Contig2\_Mf\_liverA, CL3723.Contig1\_Mf\_liverA, CL3723.Contig2\_Mf\_liverA, CL3748.Contig1\_Mf\_liverA, CL3748.Contig2\_Mf\_liverA, CL3748.Contig3\_Mf\_liverA, CL3974.Contig1\_Mf\_liverA, CL3974.Contig2\_Mf\_liverA, CL3975.Contig1\_Mf\_liverA, CL4010.Contig1\_Mf\_liverA, CL4010.Contig2\_Mf\_liverA, CL4010.Contig3\_Mf\_liverA, CL409.Contig1\_Mf\_liverA, CL409.Contig2\_Mf\_liverA, CL409.Contig3\_Mf\_liverA, CL409.Contig4\_Mf\_liverA, CL409.Contig5\_Mf\_liverA, CL4106.Contig1\_Mf\_liverA, CL4106.Contig2\_Mf\_liverA, CL4139.Contig1\_Mf\_liverA, CL4153.Contig1\_Mf\_liverA, CL4263.Contig1\_Mf\_liverA, CL4280.Contig1\_Mf\_liverA, CL4280.Contig2\_Mf\_liverA, CL4281.Contig1\_Mf\_liverA, CL4281.Contig2\_Mf\_liverA, CL4307.Contig1\_Mf\_liverA, CL4307.Contig2\_Mf\_liverA, CL4327.Contig1\_Mf\_liverA, CL4327.Contig2\_Mf\_liverA, CL4330.Contig1\_Mf\_liverA, CL4330.Contig2\_Mf\_liverA, CL4371.Contig1\_Mf\_liverA, CL4371.Contig2\_Mf\_liverA, CL4388.Contig3\_Mf\_liverA, CL4409.Contig1\_Mf\_liverA, CL4470.Contig1\_Mf\_liverA, CL4470.Contig2\_Mf\_liverA, CL4520.Contig1\_Mf\_liverA, CL4520.Contig2\_Mf\_liverA, CL4522.Contig1\_Mf\_liverA, CL4522.Contig2\_Mf\_liverA, CL4594.Contig1\_Mf\_liverA, CL4594.Contig2\_Mf\_liverA, CL4643.Contig1\_Mf\_liverA, CL4643.Contig2\_Mf\_liverA, CL4643.Contig3\_Mf\_liverA, CL4643.Contig4\_Mf\_liverA, CL4643.Contig5\_Mf\_liverA, CL4643.Contig6\_Mf\_liverA, CL4771.Contig1\_Mf\_liverA, CL4771.Contig2\_Mf\_liverA, CL4771.Contig3\_Mf\_liverA, CL4787.Contig1\_Mf\_liverA, CL4787.Contig2\_Mf\_liverA, CL4787.Contig3\_Mf\_liverA, CL4821.Contig1\_Mf\_liverA, CL4897.Contig1\_Mf\_liverA, CL4897.Contig2\_Mf\_liverA, CL4908.Contig1\_Mf\_liverA, CL4908.Contig2\_Mf\_liverA, CL5101.Contig1\_Mf\_liverA, CL5169.Contig1\_Mf\_liverA, CL5169.Contig2\_Mf\_liverA, CL5309.Contig1\_Mf\_liverA, CL5496.Contig1\_Mf\_liverA, CL5496.Contig2\_Mf\_liverA, CL5496.Contig3\_Mf\_liverA, CL5496.Contig4\_Mf\_liverA, CL5496.Contig5\_Mf\_liverA, CL5510.Contig1\_Mf\_liverA, CL5510.Contig2\_Mf\_liverA, CL5549.Contig2\_Mf\_liverA, CL5560.Contig1\_Mf\_liverA, CL5560.Contig2\_Mf\_liverA, CL5682.Contig1\_Mf\_liverA, CL5682.Contig2\_Mf\_liverA, CL5737.Contig1\_Mf\_liverA, CL5737.Contig2\_Mf\_liverA, CL5769.Contig1\_Mf\_liverA, CL5769.Contig2\_Mf\_liverA, CL5771.Contig1\_Mf\_liverA, CL5771.Contig2\_Mf\_liverA, CL608.Contig1\_Mf\_liverA, CL608.Contig2\_Mf\_liverA, CL608.Contig3\_Mf\_liverA, CL608.Contig4\_Mf\_liverA, CL644.Contig1\_Mf\_liverA, CL644.Contig2\_Mf\_liverA, CL644.Contig3\_Mf\_liverA, CL644.Contig4\_Mf\_liverA, CL680.Contig1\_Mf\_liverA, CL697.Contig1\_Mf\_liverA, CL851.Contig1\_Mf\_liverA, CL877.Contig1\_Mf\_liverA, CL877.Contig2\_Mf\_liverA, CL913.Contig1\_Mf\_liverA, CL913.Contig2\_Mf\_liverA, CL913.Contig3\_Mf\_liverA, CL930.Contig1\_Mf\_liverA, CL930.Contig2\_Mf\_liverA, CL930.Contig3\_Mf\_liverA, CL930.Contig4\_Mf\_liverA, CL931.Contig1\_Mf\_liverA, CL931.Contig2\_Mf\_liverA, CL950.Contig4\_Mf\_liverA, Unigene10296\_Mf\_liverA, Unigene1063\_Mf\_liverA, Unigene1094\_Mf\_liverA, Unigene11099\_Mf\_liverA, Unigene11104\_Mf\_liverA, Unigene11387\_Mf\_liverA, Unigene11673\_Mf\_liverA, Unigene13137\_Mf\_liverA, Unigene13231\_Mf\_liverA, Unigene13236\_Mf\_liverA, Unigene13347\_Mf\_liverA, Unigene13404\_Mf\_liverA, Unigene13405\_Mf\_liverA, Unigene13485\_Mf\_liverA, Unigene13695\_Mf\_liverA, Unigene13769\_Mf\_liverA, Unigene13853\_Mf\_liverA, Unigene13866\_Mf\_liverA, Unigene13924\_Mf\_liverA, Unigene13937\_Mf\_liverA, Unigene13951\_Mf\_liverA, Unigene141\_Mf\_liverA, Unigene142\_Mf\_liverA, Unigene143\_Mf\_liverA, Unigene14789\_Mf\_liverA, Unigene14826\_Mf\_liverA, Unigene14921\_Mf\_liverA, Unigene14971\_Mf\_liverA, Unigene15138\_Mf\_liverA, Unigene15210\_Mf\_liverA, Unigene15323\_Mf\_liverA, Unigene15376\_Mf\_liverA, Unigene15377\_Mf\_liverA, Unigene15427\_Mf\_liverA, Unigene15564\_Mf\_liverA, Unigene15610\_Mf\_liverA, Unigene15661\_Mf\_liverA, Unigene15888\_Mf\_liverA, Unigene16349\_Mf\_liverA, Unigene16463\_Mf\_liverA, Unigene16464\_Mf\_liverA, Unigene16465\_Mf\_liverA, Unigene17313\_Mf\_liverA, Unigene17362\_Mf\_liverA, Unigene17363\_Mf\_liverA, Unigene18120\_Mf\_liverA, Unigene1825\_Mf\_liverA, Unigene18643\_Mf\_liverA, Unigene19116\_Mf\_liverA, Unigene19571\_Mf\_liverA, Unigene20240\_Mf\_liverA, Unigene20241\_Mf\_liverA, Unigene20242\_Mf\_liverA, Unigene20243\_Mf\_liverA, Unigene20699\_Mf\_liverA, Unigene20865\_Mf\_liverA, Unigene208\_Mf\_liverA, Unigene2135\_Mf\_liverA, Unigene21558\_Mf\_liverA, Unigene21560\_Mf\_liverA, Unigene21934\_Mf\_liverA, Unigene22253\_Mf\_liverA, Unigene23445\_Mf\_liverA, Unigene23458\_Mf\_liverA, Unigene23529\_Mf\_liverA, Unigene23531\_Mf\_liverA, Unigene24154\_Mf\_liverA, Unigene24155\_Mf\_liverA, Unigene24294\_Mf\_liverA, Unigene24350\_Mf\_liverA, Unigene24466\_Mf\_liverA, Unigene24511\_Mf\_liverA, Unigene24512\_Mf\_liverA, Unigene24594\_Mf\_liverA, Unigene24595\_Mf\_liverA, Unigene24629\_Mf\_liverA, Unigene24631\_Mf\_liverA, Unigene25148\_Mf\_liverA, Unigene25223\_Mf\_liverA, Unigene25670\_Mf\_liverA, Unigene25712\_Mf\_liverA, Unigene25785\_Mf\_liverA, Unigene25786\_Mf\_liverA, Unigene25791\_Mf\_liverA, Unigene25974\_Mf\_liverA, Unigene26079\_Mf\_liverA, Unigene26080\_Mf\_liverA, Unigene26081\_Mf\_liverA, Unigene26082\_Mf\_liverA, Unigene27230\_Mf\_liverA, Unigene27231\_Mf\_liverA, Unigene27417\_Mf\_liverA, Unigene27733\_Mf\_liverA, Unigene27848\_Mf\_liverA, Unigene27925\_Mf\_liverA, Unigene28131\_Mf\_liverA, Unigene28275\_Mf\_liverA, Unigene28405\_Mf\_liverA, Unigene28656\_Mf\_liverA, Unigene28823\_Mf\_liverA, Unigene28824\_Mf\_liverA, Unigene28825\_Mf\_liverA, Unigene29368\_Mf\_liverA, Unigene29412\_Mf\_liverA, Unigene29413\_Mf\_liverA, Unigene2945\_Mf\_liverA, Unigene2946\_Mf\_liverA, Unigene29949\_Mf\_liverA, Unigene31017\_Mf\_liverA, Unigene31162\_Mf\_liverA, Unigene31252\_Mf\_liverA, Unigene31474\_Mf\_liverA, Unigene31475\_Mf\_liverA, Unigene31516\_Mf\_liverA, Unigene32088\_Mf\_liverA, Unigene32094\_Mf\_liverA, Unigene32142\_Mf\_liverA, Unigene32161\_Mf\_liverA, Unigene32426\_Mf\_liverA, Unigene32850\_Mf\_liverA, Unigene33012\_Mf\_liverA, Unigene33068\_Mf\_liverA, Unigene33134\_Mf\_liverA, Unigene33135\_Mf\_liverA, Unigene33169\_Mf\_liverA, Unigene33531\_Mf\_liverA, Unigene33567\_Mf\_liverA, Unigene33569\_Mf\_liverA, Unigene33808\_Mf\_liverA, Unigene33809\_Mf\_liverA, Unigene33810\_Mf\_liverA, Unigene34146\_Mf\_liverA, Unigene34180\_Mf\_liverA, Unigene34623\_Mf\_liverA, Unigene34707\_Mf\_liverA, Unigene34767\_Mf\_liverA, Unigene34773\_Mf\_liverA, Unigene34777\_Mf\_liverA, Unigene35072\_Mf\_liverA, Unigene35414\_Mf\_liverA, Unigene35491\_Mf\_liverA, Unigene35634\_Mf\_liverA, Unigene35635\_Mf\_liverA, Unigene35638\_Mf\_liverA, Unigene35747\_Mf\_liverA, Unigene35816\_Mf\_liverA, Unigene36505\_Mf\_liverA, Unigene36564\_Mf\_liverA, Unigene36565\_Mf\_liverA, Unigene36692\_Mf\_liverA, Unigene36696\_Mf\_liverA, Unigene36769\_Mf\_liverA, Unigene36785\_Mf\_liverA, Unigene36787\_Mf\_liverA, Unigene36799\_Mf\_liverA, Unigene36803\_Mf\_liverA, Unigene36809\_Mf\_liverA, Unigene36832\_Mf\_liverA, Unigene36844\_Mf\_liverA, Unigene36904\_Mf\_liverA, Unigene36927\_Mf\_liverA, Unigene36932\_Mf\_liverA, Unigene36937\_Mf\_liverA, Unigene36951\_Mf\_liverA, Unigene36952\_Mf\_liverA, Unigene36954\_Mf\_liverA, Unigene36969\_Mf\_liverA, Unigene36978\_Mf\_liverA, Unigene36984\_Mf\_liverA, Unigene37082\_Mf\_liverA, Unigene37103\_Mf\_liverA, Unigene37182\_Mf\_liverA, Unigene37282\_Mf\_liverA, Unigene37346\_Mf\_liverA, Unigene37925\_Mf\_liverA, Unigene38477\_Mf\_liverA, Unigene38685\_Mf\_liverA, Unigene392\_Mf\_liverA, Unigene39812\_Mf\_liverA, Unigene40172\_Mf\_liverA, Unigene41\_Mf\_liverA, Unigene43198\_Mf\_liverA, Unigene43335\_Mf\_liverA, Unigene44654\_Mf\_liverA, Unigene4565\_Mf\_liverA, Unigene45750\_Mf\_liverA, Unigene46117\_Mf\_liverA, Unigene4684\_Mf\_liverA, Unigene47034\_Mf\_liverA, Unigene4848\_Mf\_liverA, Unigene48890\_Mf\_liverA, Unigene49208\_Mf\_liverA, Unigene49662\_Mf\_liverA, Unigene502\_Mf\_liverA, Unigene5151\_Mf\_liverA, Unigene5168\_Mf\_liverA, Unigene51705\_Mf\_liverA, Unigene51980\_Mf\_liverA, Unigene5212\_Mf\_liverA, Unigene5219\_Mf\_liverA, Unigene5229\_Mf\_liverA, Unigene5289\_Mf\_liverA, Unigene5306\_Mf\_liverA, Unigene5308\_Mf\_liverA, Unigene5393\_Mf\_liverA, Unigene5394\_Mf\_liverA, Unigene5401\_Mf\_liverA, Unigene5412\_Mf\_liverA, Unigene5431\_Mf\_liverA, Unigene5432\_Mf\_liverA, Unigene5444\_Mf\_liverA, Unigene5702\_Mf\_liverA, Unigene5894\_Mf\_liverA, Unigene600\_Mf\_liverA, Unigene6705\_Mf\_liverA, Unigene6737\_Mf\_liverA, Unigene777\_Mf\_liverA, Unigene779\_Mf\_liverA, Unigene8162\_Mf\_liverA, Unigene8163\_Mf\_liverA, Unigene837\_Mf\_liverA, Unigene851\_Mf\_liverA, Unigene8792\_Mf\_liverA, Unigene93\_Mf\_liverA |
| 19 | Salmonella infection | CL1013.Contig5\_Mf\_liverA, CL1034.Contig2\_Mf\_liverA, CL1101.Contig1\_Mf\_liverA, CL1101.Contig2\_Mf\_liverA, CL1101.Contig3\_Mf\_liverA, CL1101.Contig4\_Mf\_liverA, CL1101.Contig5\_Mf\_liverA, CL1222.Contig1\_Mf\_liverA, CL1222.Contig2\_Mf\_liverA, CL124.Contig1\_Mf\_liverA, CL124.Contig2\_Mf\_liverA, CL1365.Contig2\_Mf\_liverA, CL1424.Contig1\_Mf\_liverA, CL1424.Contig2\_Mf\_liverA, CL1424.Contig3\_Mf\_liverA, CL1424.Contig4\_Mf\_liverA, CL1540.Contig1\_Mf\_liverA, CL1540.Contig2\_Mf\_liverA, CL1569.Contig1\_Mf\_liverA, CL1569.Contig2\_Mf\_liverA, CL1569.Contig3\_Mf\_liverA, CL1569.Contig4\_Mf\_liverA, CL1569.Contig5\_Mf\_liverA, CL1619.Contig2\_Mf\_liverA, CL1619.Contig3\_Mf\_liverA, CL1619.Contig4\_Mf\_liverA, CL1622.Contig3\_Mf\_liverA, CL1622.Contig4\_Mf\_liverA, CL1622.Contig6\_Mf\_liverA, CL1622.Contig8\_Mf\_liverA, CL1622.Contig9\_Mf\_liverA, CL1639.Contig1\_Mf\_liverA, CL1639.Contig2\_Mf\_liverA, CL1639.Contig3\_Mf\_liverA, CL1639.Contig4\_Mf\_liverA, CL1727.Contig2\_Mf\_liverA, CL1735.Contig3\_Mf\_liverA, CL1735.Contig4\_Mf\_liverA, CL1747.Contig1\_Mf\_liverA, CL1806.Contig1\_Mf\_liverA, CL1806.Contig2\_Mf\_liverA, CL1806.Contig3\_Mf\_liverA, CL1806.Contig4\_Mf\_liverA, CL1806.Contig5\_Mf\_liverA, CL1806.Contig6\_Mf\_liverA, CL1806.Contig7\_Mf\_liverA, CL1806.Contig8\_Mf\_liverA, CL1865.Contig2\_Mf\_liverA, CL1883.Contig7\_Mf\_liverA, CL1970.Contig1\_Mf\_liverA, CL1970.Contig3\_Mf\_liverA, CL1970.Contig6\_Mf\_liverA, CL2021.Contig1\_Mf\_liverA, CL2021.Contig2\_Mf\_liverA, CL2033.Contig3\_Mf\_liverA, CL2033.Contig4\_Mf\_liverA, CL2166.Contig1\_Mf\_liverA, CL2176.Contig1\_Mf\_liverA, CL2176.Contig2\_Mf\_liverA, CL2191.Contig1\_Mf\_liverA, CL2191.Contig2\_Mf\_liverA, CL2259.Contig1\_Mf\_liverA, CL2259.Contig2\_Mf\_liverA, CL226.Contig3\_Mf\_liverA, CL2292.Contig1\_Mf\_liverA, CL2334.Contig1\_Mf\_liverA, CL2334.Contig2\_Mf\_liverA, CL2334.Contig3\_Mf\_liverA, CL2334.Contig4\_Mf\_liverA, CL2368.Contig1\_Mf\_liverA, CL2368.Contig2\_Mf\_liverA, CL2376.Contig1\_Mf\_liverA, CL2378.Contig1\_Mf\_liverA, CL2378.Contig2\_Mf\_liverA, CL2378.Contig3\_Mf\_liverA, CL2378.Contig4\_Mf\_liverA, CL2405.Contig1\_Mf\_liverA, CL2405.Contig2\_Mf\_liverA, CL2551.Contig1\_Mf\_liverA, CL2553.Contig1\_Mf\_liverA, CL2603.Contig1\_Mf\_liverA, CL2603.Contig2\_Mf\_liverA, CL2603.Contig3\_Mf\_liverA, CL2603.Contig4\_Mf\_liverA, CL2608.Contig2\_Mf\_liverA, CL2611.Contig1\_Mf\_liverA, CL2611.Contig2\_Mf\_liverA, CL2611.Contig3\_Mf\_liverA, CL2611.Contig4\_Mf\_liverA, CL2639.Contig1\_Mf\_liverA, CL2737.Contig2\_Mf\_liverA, CL2737.Contig3\_Mf\_liverA, CL2737.Contig4\_Mf\_liverA, CL2737.Contig5\_Mf\_liverA, CL2737.Contig6\_Mf\_liverA, CL2796.Contig1\_Mf\_liverA, CL2861.Contig1\_Mf\_liverA, CL2871.Contig1\_Mf\_liverA, CL2900.Contig1\_Mf\_liverA, CL2900.Contig2\_Mf\_liverA, CL296.Contig1\_Mf\_liverA, CL296.Contig2\_Mf\_liverA, CL2975.Contig1\_Mf\_liverA, CL2975.Contig2\_Mf\_liverA, CL2975.Contig3\_Mf\_liverA, CL2975.Contig4\_Mf\_liverA, CL2990.Contig1\_Mf\_liverA, CL2990.Contig2\_Mf\_liverA, CL2990.Contig3\_Mf\_liverA, CL3125.Contig1\_Mf\_liverA, CL3144.Contig1\_Mf\_liverA, CL3144.Contig2\_Mf\_liverA, CL3144.Contig3\_Mf\_liverA, CL3144.Contig4\_Mf\_liverA, CL3144.Contig5\_Mf\_liverA, CL3144.Contig6\_Mf\_liverA, CL3168.Contig1\_Mf\_liverA, CL3168.Contig2\_Mf\_liverA, CL3223.Contig2\_Mf\_liverA, CL3252.Contig1\_Mf\_liverA, CL3252.Contig2\_Mf\_liverA, CL3388.Contig3\_Mf\_liverA, CL3637.Contig1\_Mf\_liverA, CL3656.Contig1\_Mf\_liverA, CL3656.Contig2\_Mf\_liverA, CL371.Contig1\_Mf\_liverA, CL371.Contig3\_Mf\_liverA, CL371.Contig5\_Mf\_liverA, CL3715.Contig3\_Mf\_liverA, CL3845.Contig1\_Mf\_liverA, CL3861.Contig1\_Mf\_liverA, CL3861.Contig2\_Mf\_liverA, CL3865.Contig1\_Mf\_liverA, CL4005.Contig1\_Mf\_liverA, CL4005.Contig2\_Mf\_liverA, CL4123.Contig1\_Mf\_liverA, CL4123.Contig2\_Mf\_liverA, CL4147.Contig2\_Mf\_liverA, CL4147.Contig3\_Mf\_liverA, CL4153.Contig2\_Mf\_liverA, CL4247.Contig1\_Mf\_liverA, CL4247.Contig2\_Mf\_liverA, CL4276.Contig1\_Mf\_liverA, CL4276.Contig2\_Mf\_liverA, CL4388.Contig1\_Mf\_liverA, CL4388.Contig2\_Mf\_liverA, CL44.Contig1\_Mf\_liverA, CL44.Contig2\_Mf\_liverA, CL4434.Contig1\_Mf\_liverA, CL4518.Contig3\_Mf\_liverA, CL4598.Contig3\_Mf\_liverA, CL4598.Contig4\_Mf\_liverA, CL4604.Contig1\_Mf\_liverA, CL4604.Contig2\_Mf\_liverA, CL4616.Contig1\_Mf\_liverA, CL4616.Contig2\_Mf\_liverA, CL462.Contig1\_Mf\_liverA, CL462.Contig2\_Mf\_liverA, CL462.Contig3\_Mf\_liverA, CL462.Contig4\_Mf\_liverA, CL462.Contig5\_Mf\_liverA, CL462.Contig6\_Mf\_liverA, CL462.Contig7\_Mf\_liverA, CL462.Contig8\_Mf\_liverA, CL4648.Contig1\_Mf\_liverA, CL4648.Contig2\_Mf\_liverA, CL4731.Contig1\_Mf\_liverA, CL4731.Contig2\_Mf\_liverA, CL4762.Contig1\_Mf\_liverA, CL4772.Contig1\_Mf\_liverA, CL479.Contig1\_Mf\_liverA, CL479.Contig2\_Mf\_liverA, CL4892.Contig1\_Mf\_liverA, CL4892.Contig2\_Mf\_liverA, CL504.Contig10\_Mf\_liverA, CL504.Contig11\_Mf\_liverA, CL504.Contig12\_Mf\_liverA, CL504.Contig13\_Mf\_liverA, CL504.Contig14\_Mf\_liverA, CL504.Contig1\_Mf\_liverA, CL504.Contig2\_Mf\_liverA, CL504.Contig3\_Mf\_liverA, CL504.Contig4\_Mf\_liverA, CL504.Contig5\_Mf\_liverA, CL504.Contig6\_Mf\_liverA, CL504.Contig7\_Mf\_liverA, CL504.Contig8\_Mf\_liverA, CL504.Contig9\_Mf\_liverA, CL5048.Contig1\_Mf\_liverA, CL507.Contig1\_Mf\_liverA, CL5184.Contig1\_Mf\_liverA, CL5189.Contig1\_Mf\_liverA, CL5189.Contig2\_Mf\_liverA, CL5254.Contig2\_Mf\_liverA, CL5406.Contig2\_Mf\_liverA, CL5527.Contig1\_Mf\_liverA, CL5527.Contig2\_Mf\_liverA, CL5569.Contig1\_Mf\_liverA, CL5569.Contig2\_Mf\_liverA, CL5570.Contig1\_Mf\_liverA, CL5570.Contig2\_Mf\_liverA, CL561.Contig2\_Mf\_liverA, CL561.Contig3\_Mf\_liverA, CL5669.Contig1\_Mf\_liverA, CL5669.Contig2\_Mf\_liverA, CL5878.Contig2\_Mf\_liverA, CL5928.Contig1\_Mf\_liverA, CL5928.Contig2\_Mf\_liverA, CL774.Contig10\_Mf\_liverA, CL774.Contig11\_Mf\_liverA, CL774.Contig12\_Mf\_liverA, CL774.Contig3\_Mf\_liverA, CL774.Contig4\_Mf\_liverA, CL774.Contig5\_Mf\_liverA, CL774.Contig6\_Mf\_liverA, CL774.Contig9\_Mf\_liverA, CL779.Contig10\_Mf\_liverA, CL779.Contig12\_Mf\_liverA, CL779.Contig1\_Mf\_liverA, CL779.Contig3\_Mf\_liverA, CL779.Contig4\_Mf\_liverA, CL779.Contig6\_Mf\_liverA, CL779.Contig7\_Mf\_liverA, CL779.Contig9\_Mf\_liverA, CL807.Contig1\_Mf\_liverA, CL807.Contig2\_Mf\_liverA, CL807.Contig3\_Mf\_liverA, CL807.Contig4\_Mf\_liverA, CL807.Contig5\_Mf\_liverA, CL807.Contig6\_Mf\_liverA, CL807.Contig7\_Mf\_liverA, CL807.Contig8\_Mf\_liverA, CL812.Contig4\_Mf\_liverA, CL851.Contig1\_Mf\_liverA, CL851.Contig2\_Mf\_liverA, CL863.Contig1\_Mf\_liverA, CL863.Contig2\_Mf\_liverA, CL863.Contig3\_Mf\_liverA, CL863.Contig4\_Mf\_liverA, CL882.Contig2\_Mf\_liverA, CL882.Contig3\_Mf\_liverA, CL882.Contig5\_Mf\_liverA, CL882.Contig6\_Mf\_liverA, Unigene1008\_Mf\_liverA, Unigene1055\_Mf\_liverA, Unigene10657\_Mf\_liverA, Unigene10889\_Mf\_liverA, Unigene11782\_Mf\_liverA, Unigene13177\_Mf\_liverA, Unigene13178\_Mf\_liverA, Unigene13535\_Mf\_liverA, Unigene13628\_Mf\_liverA, Unigene13695\_Mf\_liverA, Unigene13945\_Mf\_liverA, Unigene14168\_Mf\_liverA, Unigene14253\_Mf\_liverA, Unigene14588\_Mf\_liverA, Unigene1461\_Mf\_liverA, Unigene14941\_Mf\_liverA, Unigene15010\_Mf\_liverA, Unigene16819\_Mf\_liverA, Unigene16820\_Mf\_liverA, Unigene16952\_Mf\_liverA, Unigene17113\_Mf\_liverA, Unigene17392\_Mf\_liverA, Unigene17455\_Mf\_liverA, Unigene18199\_Mf\_liverA, Unigene18792\_Mf\_liverA, Unigene19177\_Mf\_liverA, Unigene19232\_Mf\_liverA, Unigene19715\_Mf\_liverA, Unigene19716\_Mf\_liverA, Unigene19881\_Mf\_liverA, Unigene19882\_Mf\_liverA, Unigene19992\_Mf\_liverA, Unigene20108\_Mf\_liverA, Unigene21043\_Mf\_liverA, Unigene21463\_Mf\_liverA, Unigene21620\_Mf\_liverA, Unigene21905\_Mf\_liverA, Unigene21906\_Mf\_liverA, Unigene21935\_Mf\_liverA, Unigene22012\_Mf\_liverA, Unigene22158\_Mf\_liverA, Unigene2235\_Mf\_liverA, Unigene22390\_Mf\_liverA, Unigene22676\_Mf\_liverA, Unigene22762\_Mf\_liverA, Unigene23934\_Mf\_liverA, Unigene23935\_Mf\_liverA, Unigene23936\_Mf\_liverA, Unigene23937\_Mf\_liverA, Unigene23938\_Mf\_liverA, Unigene24182\_Mf\_liverA, Unigene24215\_Mf\_liverA, Unigene24255\_Mf\_liverA, Unigene24335\_Mf\_liverA, Unigene24540\_Mf\_liverA, Unigene24763\_Mf\_liverA, Unigene24805\_Mf\_liverA, Unigene24909\_Mf\_liverA, Unigene24955\_Mf\_liverA, Unigene24956\_Mf\_liverA, Unigene24957\_Mf\_liverA, Unigene24958\_Mf\_liverA, Unigene24965\_Mf\_liverA, Unigene25132\_Mf\_liverA, Unigene25299\_Mf\_liverA, Unigene25300\_Mf\_liverA, Unigene2530\_Mf\_liverA, Unigene25404\_Mf\_liverA, Unigene25826\_Mf\_liverA, Unigene26190\_Mf\_liverA, Unigene26406\_Mf\_liverA, Unigene26712\_Mf\_liverA, Unigene26964\_Mf\_liverA, Unigene2721\_Mf\_liverA, Unigene27221\_Mf\_liverA, Unigene27939\_Mf\_liverA, Unigene28085\_Mf\_liverA, Unigene28086\_Mf\_liverA, Unigene2829\_Mf\_liverA, Unigene28491\_Mf\_liverA, Unigene28621\_Mf\_liverA, Unigene28624\_Mf\_liverA, Unigene28722\_Mf\_liverA, Unigene28725\_Mf\_liverA, Unigene29850\_Mf\_liverA, Unigene30005\_Mf\_liverA, Unigene30006\_Mf\_liverA, Unigene30099\_Mf\_liverA, Unigene30134\_Mf\_liverA, Unigene30135\_Mf\_liverA, Unigene30192\_Mf\_liverA, Unigene30256\_Mf\_liverA, Unigene30601\_Mf\_liverA, Unigene30776\_Mf\_liverA, Unigene30873\_Mf\_liverA, Unigene30877\_Mf\_liverA, Unigene30944\_Mf\_liverA, Unigene30949\_Mf\_liverA, Unigene31303\_Mf\_liverA, Unigene31333\_Mf\_liverA, Unigene31368\_Mf\_liverA, Unigene31424\_Mf\_liverA, Unigene31487\_Mf\_liverA, Unigene31613\_Mf\_liverA, Unigene31665\_Mf\_liverA, Unigene31888\_Mf\_liverA, Unigene31889\_Mf\_liverA, Unigene31954\_Mf\_liverA, Unigene31968\_Mf\_liverA, Unigene32097\_Mf\_liverA, Unigene32125\_Mf\_liverA, Unigene32168\_Mf\_liverA, Unigene32176\_Mf\_liverA, Unigene32183\_Mf\_liverA, Unigene32359\_Mf\_liverA, Unigene32396\_Mf\_liverA, Unigene32583\_Mf\_liverA, Unigene32659\_Mf\_liverA, Unigene32714\_Mf\_liverA, Unigene32879\_Mf\_liverA, Unigene32882\_Mf\_liverA, Unigene32883\_Mf\_liverA, Unigene32895\_Mf\_liverA, Unigene3295\_Mf\_liverA, Unigene33024\_Mf\_liverA, Unigene33025\_Mf\_liverA, Unigene33076\_Mf\_liverA, Unigene33262\_Mf\_liverA, Unigene33263\_Mf\_liverA, Unigene33275\_Mf\_liverA, Unigene33352\_Mf\_liverA, Unigene33353\_Mf\_liverA, Unigene33364\_Mf\_liverA, Unigene33533\_Mf\_liverA, Unigene33534\_Mf\_liverA, Unigene33692\_Mf\_liverA, Unigene33838\_Mf\_liverA, Unigene33937\_Mf\_liverA, Unigene33938\_Mf\_liverA, Unigene34034\_Mf\_liverA, Unigene34055\_Mf\_liverA, Unigene34056\_Mf\_liverA, Unigene34803\_Mf\_liverA, Unigene34930\_Mf\_liverA, Unigene3514\_Mf\_liverA, Unigene35224\_Mf\_liverA, Unigene35370\_Mf\_liverA, Unigene35600\_Mf\_liverA, Unigene35748\_Mf\_liverA, Unigene36260\_Mf\_liverA, Unigene36261\_Mf\_liverA, Unigene36337\_Mf\_liverA, Unigene36631\_Mf\_liverA, Unigene36818\_Mf\_liverA, Unigene37042\_Mf\_liverA, Unigene37096\_Mf\_liverA, Unigene37099\_Mf\_liverA, Unigene37147\_Mf\_liverA, Unigene37341\_Mf\_liverA, Unigene37358\_Mf\_liverA, Unigene373\_Mf\_liverA, Unigene37412\_Mf\_liverA, Unigene37448\_Mf\_liverA, Unigene37474\_Mf\_liverA, Unigene37802\_Mf\_liverA, Unigene38219\_Mf\_liverA, Unigene38353\_Mf\_liverA, Unigene38520\_Mf\_liverA, Unigene38657\_Mf\_liverA, Unigene38931\_Mf\_liverA, Unigene38994\_Mf\_liverA, Unigene39099\_Mf\_liverA, Unigene39537\_Mf\_liverA, Unigene40020\_Mf\_liverA, Unigene40060\_Mf\_liverA, Unigene40533\_Mf\_liverA, Unigene40824\_Mf\_liverA, Unigene4133\_Mf\_liverA, Unigene41594\_Mf\_liverA, Unigene41957\_Mf\_liverA, Unigene4214\_Mf\_liverA, Unigene42272\_Mf\_liverA, Unigene43225\_Mf\_liverA, Unigene43339\_Mf\_liverA, Unigene43599\_Mf\_liverA, Unigene43624\_Mf\_liverA, Unigene44137\_Mf\_liverA, Unigene44189\_Mf\_liverA, Unigene44395\_Mf\_liverA, Unigene44619\_Mf\_liverA, Unigene44657\_Mf\_liverA, Unigene44886\_Mf\_liverA, Unigene45138\_Mf\_liverA, Unigene45324\_Mf\_liverA, Unigene45662\_Mf\_liverA, Unigene46484\_Mf\_liverA, Unigene4684\_Mf\_liverA, Unigene46897\_Mf\_liverA, Unigene47034\_Mf\_liverA, Unigene47377\_Mf\_liverA, Unigene47554\_Mf\_liverA, Unigene47970\_Mf\_liverA, Unigene48463\_Mf\_liverA, Unigene4863\_Mf\_liverA, Unigene49035\_Mf\_liverA, Unigene4947\_Mf\_liverA, Unigene5172\_Mf\_liverA, Unigene5263\_Mf\_liverA, Unigene5316\_Mf\_liverA, Unigene5434\_Mf\_liverA, Unigene5624\_Mf\_liverA, Unigene5648\_Mf\_liverA, Unigene5875\_Mf\_liverA, Unigene613\_Mf\_liverA, Unigene676\_Mf\_liverA, Unigene682\_Mf\_liverA, Unigene6897\_Mf\_liverA, Unigene7104\_Mf\_liverA, Unigene7105\_Mf\_liverA, Unigene7126\_Mf\_liverA, Unigene7146\_Mf\_liverA, Unigene7244\_Mf\_liverA, Unigene8042\_Mf\_liverA, Unigene8066\_Mf\_liverA, Unigene9067\_Mf\_liverA, Unigene9197\_Mf\_liverA, Unigene919\_Mf\_liverA, Unigene9436\_Mf\_liverA, Unigene9499\_Mf\_liverA, Unigene9621\_Mf\_liverA, Unigene9895\_Mf\_liverA |
| 20 | Adherens junction | CL1019.Contig1\_Mf\_liverA, CL1019.Contig2\_Mf\_liverA, CL1034.Contig2\_Mf\_liverA, CL1196.Contig1\_Mf\_liverA, CL1196.Contig2\_Mf\_liverA, CL1202.Contig1\_Mf\_liverA, CL1222.Contig1\_Mf\_liverA, CL1222.Contig2\_Mf\_liverA, CL13.Contig1\_Mf\_liverA, CL13.Contig2\_Mf\_liverA, CL1365.Contig2\_Mf\_liverA, CL1401.Contig1\_Mf\_liverA, CL1401.Contig2\_Mf\_liverA, CL1401.Contig3\_Mf\_liverA, CL1401.Contig4\_Mf\_liverA, CL1401.Contig5\_Mf\_liverA, CL1401.Contig6\_Mf\_liverA, CL1401.Contig7\_Mf\_liverA, CL1401.Contig8\_Mf\_liverA, CL1538.Contig1\_Mf\_liverA, CL1598.Contig1\_Mf\_liverA, CL1598.Contig2\_Mf\_liverA, CL1598.Contig3\_Mf\_liverA, CL1598.Contig4\_Mf\_liverA, CL1598.Contig5\_Mf\_liverA, CL1598.Contig6\_Mf\_liverA, CL1727.Contig2\_Mf\_liverA, CL1743.Contig10\_Mf\_liverA, CL1743.Contig11\_Mf\_liverA, CL1743.Contig12\_Mf\_liverA, CL1743.Contig13\_Mf\_liverA, CL1743.Contig15\_Mf\_liverA, CL1743.Contig16\_Mf\_liverA, CL1743.Contig7\_Mf\_liverA, CL1743.Contig8\_Mf\_liverA, CL1747.Contig1\_Mf\_liverA, CL1761.Contig10\_Mf\_liverA, CL1761.Contig11\_Mf\_liverA, CL1761.Contig12\_Mf\_liverA, CL1761.Contig13\_Mf\_liverA, CL1761.Contig14\_Mf\_liverA, CL1761.Contig15\_Mf\_liverA, CL1761.Contig1\_Mf\_liverA, CL1761.Contig2\_Mf\_liverA, CL1761.Contig3\_Mf\_liverA, CL1761.Contig4\_Mf\_liverA, CL1761.Contig5\_Mf\_liverA, CL1761.Contig6\_Mf\_liverA, CL1761.Contig7\_Mf\_liverA, CL1761.Contig8\_Mf\_liverA, CL1761.Contig9\_Mf\_liverA, CL1817.Contig1\_Mf\_liverA, CL1817.Contig2\_Mf\_liverA, CL1817.Contig3\_Mf\_liverA, CL1865.Contig2\_Mf\_liverA, CL1877.Contig1\_Mf\_liverA, CL1877.Contig2\_Mf\_liverA, CL1877.Contig3\_Mf\_liverA, CL1877.Contig4\_Mf\_liverA, CL1883.Contig7\_Mf\_liverA, CL1970.Contig1\_Mf\_liverA, CL1970.Contig3\_Mf\_liverA, CL1970.Contig6\_Mf\_liverA, CL2021.Contig1\_Mf\_liverA, CL2021.Contig2\_Mf\_liverA, CL2062.Contig1\_Mf\_liverA, CL2062.Contig2\_Mf\_liverA, CL2145.Contig3\_Mf\_liverA, CL2145.Contig4\_Mf\_liverA, CL2166.Contig1\_Mf\_liverA, CL2191.Contig1\_Mf\_liverA, CL2191.Contig2\_Mf\_liverA, CL2259.Contig1\_Mf\_liverA, CL2259.Contig2\_Mf\_liverA, CL226.Contig3\_Mf\_liverA, CL2376.Contig1\_Mf\_liverA, CL2404.Contig10\_Mf\_liverA, CL2404.Contig11\_Mf\_liverA, CL2404.Contig12\_Mf\_liverA, CL2404.Contig13\_Mf\_liverA, CL2404.Contig14\_Mf\_liverA, CL2404.Contig15\_Mf\_liverA, CL2404.Contig1\_Mf\_liverA, CL2404.Contig2\_Mf\_liverA, CL2404.Contig3\_Mf\_liverA, CL2404.Contig4\_Mf\_liverA, CL2404.Contig5\_Mf\_liverA, CL2404.Contig6\_Mf\_liverA, CL2404.Contig7\_Mf\_liverA, CL2404.Contig8\_Mf\_liverA, CL2404.Contig9\_Mf\_liverA, CL2405.Contig1\_Mf\_liverA, CL2405.Contig2\_Mf\_liverA, CL248.Contig1\_Mf\_liverA, CL248.Contig2\_Mf\_liverA, CL248.Contig3\_Mf\_liverA, CL2664.Contig1\_Mf\_liverA, CL2664.Contig2\_Mf\_liverA, CL2664.Contig3\_Mf\_liverA, CL2664.Contig4\_Mf\_liverA, CL2692.Contig1\_Mf\_liverA, CL2692.Contig2\_Mf\_liverA, CL2692.Contig3\_Mf\_liverA, CL2796.Contig1\_Mf\_liverA, CL2806.Contig1\_Mf\_liverA, CL2806.Contig2\_Mf\_liverA, CL2806.Contig3\_Mf\_liverA, CL2861.Contig1\_Mf\_liverA, CL2871.Contig1\_Mf\_liverA, CL2900.Contig1\_Mf\_liverA, CL2900.Contig2\_Mf\_liverA, CL2969.Contig1\_Mf\_liverA, CL2969.Contig2\_Mf\_liverA, CL3053.Contig1\_Mf\_liverA, CL3144.Contig1\_Mf\_liverA, CL3144.Contig2\_Mf\_liverA, CL3144.Contig3\_Mf\_liverA, CL3144.Contig4\_Mf\_liverA, CL3144.Contig5\_Mf\_liverA, CL3144.Contig6\_Mf\_liverA, CL3168.Contig1\_Mf\_liverA, CL3168.Contig2\_Mf\_liverA, CL3223.Contig2\_Mf\_liverA, CL3267.Contig1\_Mf\_liverA, CL3267.Contig2\_Mf\_liverA, CL3272.Contig1\_Mf\_liverA, CL3272.Contig2\_Mf\_liverA, CL3276.Contig1\_Mf\_liverA, CL3276.Contig2\_Mf\_liverA, CL3276.Contig3\_Mf\_liverA, CL3301.Contig1\_Mf\_liverA, CL3301.Contig2\_Mf\_liverA, CL3301.Contig3\_Mf\_liverA, CL3301.Contig4\_Mf\_liverA, CL3388.Contig3\_Mf\_liverA, CL3615.Contig1\_Mf\_liverA, CL3615.Contig2\_Mf\_liverA, CL3637.Contig1\_Mf\_liverA, CL3715.Contig3\_Mf\_liverA, CL3724.Contig1\_Mf\_liverA, CL3724.Contig2\_Mf\_liverA, CL3724.Contig3\_Mf\_liverA, CL3724.Contig4\_Mf\_liverA, CL3724.Contig5\_Mf\_liverA, CL373.Contig1\_Mf\_liverA, CL373.Contig2\_Mf\_liverA, CL373.Contig3\_Mf\_liverA, CL373.Contig4\_Mf\_liverA, CL373.Contig5\_Mf\_liverA, CL373.Contig6\_Mf\_liverA, CL373.Contig7\_Mf\_liverA, CL373.Contig8\_Mf\_liverA, CL3861.Contig1\_Mf\_liverA, CL3861.Contig2\_Mf\_liverA, CL3898.Contig1\_Mf\_liverA, CL3898.Contig2\_Mf\_liverA, CL3963.Contig1\_Mf\_liverA, CL3963.Contig2\_Mf\_liverA, CL4147.Contig2\_Mf\_liverA, CL4147.Contig3\_Mf\_liverA, CL4304.Contig1\_Mf\_liverA, CL4425.Contig1\_Mf\_liverA, CL4425.Contig2\_Mf\_liverA, CL4437.Contig1\_Mf\_liverA, CL4437.Contig2\_Mf\_liverA, CL4459.Contig1\_Mf\_liverA, CL4459.Contig2\_Mf\_liverA, CL4518.Contig3\_Mf\_liverA, CL4562.Contig1\_Mf\_liverA, CL4562.Contig2\_Mf\_liverA, CL4731.Contig1\_Mf\_liverA, CL4731.Contig2\_Mf\_liverA, CL4772.Contig1\_Mf\_liverA, CL4892.Contig1\_Mf\_liverA, CL4892.Contig2\_Mf\_liverA, CL4898.Contig1\_Mf\_liverA, CL4898.Contig2\_Mf\_liverA, CL4901.Contig1\_Mf\_liverA, CL4901.Contig2\_Mf\_liverA, CL5048.Contig1\_Mf\_liverA, CL5201.Contig1\_Mf\_liverA, CL5201.Contig2\_Mf\_liverA, CL5209.Contig1\_Mf\_liverA, CL5209.Contig2\_Mf\_liverA, CL5243.Contig1\_Mf\_liverA, CL5243.Contig2\_Mf\_liverA, CL5254.Contig1\_Mf\_liverA, CL5254.Contig2\_Mf\_liverA, CL5406.Contig2\_Mf\_liverA, CL5527.Contig1\_Mf\_liverA, CL5527.Contig2\_Mf\_liverA, CL5552.Contig1\_Mf\_liverA, CL5552.Contig2\_Mf\_liverA, CL561.Contig1\_Mf\_liverA, CL578.Contig10\_Mf\_liverA, CL578.Contig11\_Mf\_liverA, CL578.Contig12\_Mf\_liverA, CL578.Contig13\_Mf\_liverA, CL578.Contig14\_Mf\_liverA, CL578.Contig15\_Mf\_liverA, CL578.Contig16\_Mf\_liverA, CL578.Contig17\_Mf\_liverA, CL578.Contig18\_Mf\_liverA, CL578.Contig19\_Mf\_liverA, CL578.Contig1\_Mf\_liverA, CL578.Contig20\_Mf\_liverA, CL578.Contig21\_Mf\_liverA, CL578.Contig22\_Mf\_liverA, CL578.Contig23\_Mf\_liverA, CL578.Contig24\_Mf\_liverA, CL578.Contig25\_Mf\_liverA, CL578.Contig26\_Mf\_liverA, CL578.Contig27\_Mf\_liverA, CL578.Contig2\_Mf\_liverA, CL578.Contig3\_Mf\_liverA, CL578.Contig4\_Mf\_liverA, CL578.Contig5\_Mf\_liverA, CL578.Contig6\_Mf\_liverA, CL578.Contig7\_Mf\_liverA, CL578.Contig8\_Mf\_liverA, CL578.Contig9\_Mf\_liverA, CL5799.Contig1\_Mf\_liverA, CL5799.Contig2\_Mf\_liverA, CL5804.Contig1\_Mf\_liverA, CL5804.Contig2\_Mf\_liverA, CL5871.Contig10\_Mf\_liverA, CL5871.Contig11\_Mf\_liverA, CL5871.Contig2\_Mf\_liverA, CL5871.Contig4\_Mf\_liverA, CL5871.Contig5\_Mf\_liverA, CL5871.Contig6\_Mf\_liverA, CL5871.Contig7\_Mf\_liverA, CL5871.Contig8\_Mf\_liverA, CL5871.Contig9\_Mf\_liverA, CL6034.Contig1\_Mf\_liverA, CL6034.Contig2\_Mf\_liverA, CL605.Contig2\_Mf\_liverA, CL628.Contig10\_Mf\_liverA, CL628.Contig2\_Mf\_liverA, CL628.Contig3\_Mf\_liverA, CL628.Contig4\_Mf\_liverA, CL628.Contig5\_Mf\_liverA, CL628.Contig6\_Mf\_liverA, CL628.Contig7\_Mf\_liverA, CL628.Contig8\_Mf\_liverA, CL774.Contig10\_Mf\_liverA, CL774.Contig11\_Mf\_liverA, CL774.Contig12\_Mf\_liverA, CL774.Contig3\_Mf\_liverA, CL774.Contig4\_Mf\_liverA, CL774.Contig5\_Mf\_liverA, CL774.Contig6\_Mf\_liverA, CL774.Contig9\_Mf\_liverA, CL812.Contig4\_Mf\_liverA, CL833.Contig1\_Mf\_liverA, CL833.Contig2\_Mf\_liverA, CL838.Contig2\_Mf\_liverA, CL932.Contig5\_Mf\_liverA, CL932.Contig6\_Mf\_liverA, CL932.Contig9\_Mf\_liverA, CL951.Contig1\_Mf\_liverA, CL951.Contig2\_Mf\_liverA, CL97.Contig1\_Mf\_liverA, CL97.Contig2\_Mf\_liverA, CL987.Contig1\_Mf\_liverA, CL987.Contig2\_Mf\_liverA, CL987.Contig3\_Mf\_liverA, CL987.Contig4\_Mf\_liverA, CL987.Contig5\_Mf\_liverA, CL987.Contig6\_Mf\_liverA, Unigene10279\_Mf\_liverA, Unigene10335\_Mf\_liverA, Unigene10356\_Mf\_liverA, Unigene1085\_Mf\_liverA, Unigene11564\_Mf\_liverA, Unigene13060\_Mf\_liverA, Unigene1309\_Mf\_liverA, Unigene13169\_Mf\_liverA, Unigene13170\_Mf\_liverA, Unigene13171\_Mf\_liverA, Unigene1322\_Mf\_liverA, Unigene13249\_Mf\_liverA, Unigene13250\_Mf\_liverA, Unigene13845\_Mf\_liverA, Unigene13846\_Mf\_liverA, Unigene14168\_Mf\_liverA, Unigene14420\_Mf\_liverA, Unigene14468\_Mf\_liverA, Unigene14469\_Mf\_liverA, Unigene14588\_Mf\_liverA, Unigene14637\_Mf\_liverA, Unigene15040\_Mf\_liverA, Unigene15449\_Mf\_liverA, Unigene16262\_Mf\_liverA, Unigene16952\_Mf\_liverA, Unigene17113\_Mf\_liverA, Unigene18129\_Mf\_liverA, Unigene18326\_Mf\_liverA, Unigene18404\_Mf\_liverA, Unigene18405\_Mf\_liverA, Unigene18487\_Mf\_liverA, Unigene18792\_Mf\_liverA, Unigene19269\_Mf\_liverA, Unigene19795\_Mf\_liverA, Unigene19881\_Mf\_liverA, Unigene19882\_Mf\_liverA, Unigene19991\_Mf\_liverA, Unigene20043\_Mf\_liverA, Unigene20044\_Mf\_liverA, Unigene20700\_Mf\_liverA, Unigene20705\_Mf\_liverA, Unigene21284\_Mf\_liverA, Unigene21463\_Mf\_liverA, Unigene22390\_Mf\_liverA, Unigene22754\_Mf\_liverA, Unigene22755\_Mf\_liverA, Unigene22926\_Mf\_liverA, Unigene23137\_Mf\_liverA, Unigene23138\_Mf\_liverA, Unigene23139\_Mf\_liverA, Unigene23140\_Mf\_liverA, Unigene23344\_Mf\_liverA, Unigene23869\_Mf\_liverA, Unigene23870\_Mf\_liverA, Unigene23871\_Mf\_liverA, Unigene24182\_Mf\_liverA, Unigene24335\_Mf\_liverA, Unigene24763\_Mf\_liverA, Unigene24805\_Mf\_liverA, Unigene25162\_Mf\_liverA, Unigene25272\_Mf\_liverA, Unigene25286\_Mf\_liverA, Unigene25404\_Mf\_liverA, Unigene25826\_Mf\_liverA, Unigene26195\_Mf\_liverA, Unigene26413\_Mf\_liverA, Unigene27140\_Mf\_liverA, Unigene27221\_Mf\_liverA, Unigene27260\_Mf\_liverA, Unigene27261\_Mf\_liverA, Unigene27312\_Mf\_liverA, Unigene27313\_Mf\_liverA, Unigene2829\_Mf\_liverA, Unigene28722\_Mf\_liverA, Unigene29829\_Mf\_liverA, Unigene29831\_Mf\_liverA, Unigene29850\_Mf\_liverA, Unigene29899\_Mf\_liverA, Unigene30099\_Mf\_liverA, Unigene30192\_Mf\_liverA, Unigene30517\_Mf\_liverA, Unigene30518\_Mf\_liverA, Unigene30877\_Mf\_liverA, Unigene30949\_Mf\_liverA, Unigene31303\_Mf\_liverA, Unigene31325\_Mf\_liverA, Unigene31368\_Mf\_liverA, Unigene31424\_Mf\_liverA, Unigene31665\_Mf\_liverA, Unigene31687\_Mf\_liverA, Unigene31688\_Mf\_liverA, Unigene31691\_Mf\_liverA, Unigene31692\_Mf\_liverA, Unigene31708\_Mf\_liverA, Unigene31921\_Mf\_liverA, Unigene31954\_Mf\_liverA, Unigene32096\_Mf\_liverA, Unigene32125\_Mf\_liverA, Unigene32203\_Mf\_liverA, Unigene32204\_Mf\_liverA, Unigene3222\_Mf\_liverA, Unigene32334\_Mf\_liverA, Unigene32335\_Mf\_liverA, Unigene32357\_Mf\_liverA, Unigene32362\_Mf\_liverA, Unigene32570\_Mf\_liverA, Unigene32571\_Mf\_liverA, Unigene32621\_Mf\_liverA, Unigene32649\_Mf\_liverA, Unigene32659\_Mf\_liverA, Unigene32895\_Mf\_liverA, Unigene33262\_Mf\_liverA, Unigene33263\_Mf\_liverA, Unigene33275\_Mf\_liverA, Unigene33352\_Mf\_liverA, Unigene33692\_Mf\_liverA, Unigene33838\_Mf\_liverA, Unigene33937\_Mf\_liverA, Unigene33938\_Mf\_liverA, Unigene34055\_Mf\_liverA, Unigene34056\_Mf\_liverA, Unigene34847\_Mf\_liverA, Unigene34930\_Mf\_liverA, Unigene34975\_Mf\_liverA, Unigene3514\_Mf\_liverA, Unigene35370\_Mf\_liverA, Unigene35406\_Mf\_liverA, Unigene35407\_Mf\_liverA, Unigene35612\_Mf\_liverA, Unigene35748\_Mf\_liverA, Unigene36182\_Mf\_liverA, Unigene36187\_Mf\_liverA, Unigene36748\_Mf\_liverA, Unigene36818\_Mf\_liverA, Unigene36909\_Mf\_liverA, Unigene37029\_Mf\_liverA, Unigene37147\_Mf\_liverA, Unigene37341\_Mf\_liverA, Unigene373\_Mf\_liverA, Unigene37412\_Mf\_liverA, Unigene37474\_Mf\_liverA, Unigene37535\_Mf\_liverA, Unigene37802\_Mf\_liverA, Unigene38353\_Mf\_liverA, Unigene38369\_Mf\_liverA, Unigene38931\_Mf\_liverA, Unigene38972\_Mf\_liverA, Unigene3907\_Mf\_liverA, Unigene39816\_Mf\_liverA, Unigene40020\_Mf\_liverA, Unigene40060\_Mf\_liverA, Unigene40824\_Mf\_liverA, Unigene41248\_Mf\_liverA, Unigene4133\_Mf\_liverA, Unigene41957\_Mf\_liverA, Unigene41993\_Mf\_liverA, Unigene42272\_Mf\_liverA, Unigene43225\_Mf\_liverA, Unigene43339\_Mf\_liverA, Unigene43599\_Mf\_liverA, Unigene44395\_Mf\_liverA, Unigene44657\_Mf\_liverA, Unigene45138\_Mf\_liverA, Unigene4538\_Mf\_liverA, Unigene46484\_Mf\_liverA, Unigene469\_Mf\_liverA, Unigene47034\_Mf\_liverA, Unigene4742\_Mf\_liverA, Unigene47554\_Mf\_liverA, Unigene4871\_Mf\_liverA, Unigene48879\_Mf\_liverA, Unigene49422\_Mf\_liverA, Unigene5172\_Mf\_liverA, Unigene517\_Mf\_liverA, Unigene5434\_Mf\_liverA, Unigene5453\_Mf\_liverA, Unigene5486\_Mf\_liverA, Unigene5767\_Mf\_liverA, Unigene5875\_Mf\_liverA, Unigene6151\_Mf\_liverA, Unigene668\_Mf\_liverA, Unigene682\_Mf\_liverA, Unigene7066\_Mf\_liverA, Unigene7067\_Mf\_liverA, Unigene7146\_Mf\_liverA, Unigene7581\_Mf\_liverA, Unigene7589\_Mf\_liverA, Unigene882\_Mf\_liverA, Unigene894\_Mf\_liverA, Unigene9067\_Mf\_liverA, Unigene9197\_Mf\_liverA, Unigene919\_Mf\_liverA, Unigene9517\_Mf\_liverA, Unigene969\_Mf\_liverA |
[truncated: 949,571 more chars]
