# Supplementary material for: De novo assembly and transcriptome characterization: novel insights into the natural resistance mechanisms of Microtus fortis against Schistosoma japonicum
Source: BMC Genomics. 2014 Jun 2;15(1):417. doi: 10.1186/1471-2164-15-417 (PMC4073500; doi:10.1186/1471-2164-15-417)
Supplement: Supplementary file 4 — Additional file 4: Dataset S2: GO terms for MfA-VS-MfA1W_C. (ZIP 44 KB) [file 12864_2013_6159_MOESM4_ESM.zip › 1990354100108772_add4.html]

Terms for MfA-VS-MfA1W\_C


## Terms for MfA-VS-MfA1W\_C

---


### Result Table

|  |
| --- |
| **Terms from the Component Ontology with p-value as good or better than 1** |

| Gene Ontology term | Cluster frequency | Genome frequency of use | Corrected P-value | Expression Profile |
| --- | --- | --- | --- | --- |
| cell periphery | 251 out of 690 genes, 36.4% | 6038 out of 37603 genes, 16.1% | 1.10e-36 | View Result |
| plasma membrane | 244 out of 690 genes, 35.4% | 5905 out of 37603 genes, 15.7% | 6.42e-35 | View Result |
| external side of plasma membrane | 48 out of 690 genes, 7.0% | 286 out of 37603 genes, 0.8% | 5.24e-29 | View Result |
| extracellular region | 148 out of 690 genes, 21.4% | 2987 out of 37603 genes, 7.9% | 1.15e-26 | View Result |
| extracellular space | 78 out of 690 genes, 11.3% | 1015 out of 37603 genes, 2.7% | 4.15e-24 | View Result |
| cell surface | 74 out of 690 genes, 10.7% | 1006 out of 37603 genes, 2.7% | 1.27e-21 | View Result |
| extracellular region part | 109 out of 690 genes, 15.8% | 2343 out of 37603 genes, 6.2% | 1.02e-16 | View Result |
| integral to membrane | 207 out of 690 genes, 30.0% | 6371 out of 37603 genes, 16.9% | 3.18e-15 | View Result |
| plasma membrane part | 151 out of 690 genes, 21.9% | 4083 out of 37603 genes, 10.9% | 8.64e-15 | View Result |
| MHC protein complex | 19 out of 690 genes, 2.8% | 71 out of 37603 genes, 0.2% | 9.72e-15 | View Result |
| cytoplasm | 430 out of 690 genes, 62.3% | 17507 out of 37603 genes, 46.6% | 1.19e-14 | View Result |
| endoplasmic reticulum | 96 out of 690 genes, 13.9% | 2107 out of 37603 genes, 5.6% | 7.20e-14 | View Result |
| nuclear outer membrane-endoplasmic reticulum membrane network | 63 out of 690 genes, 9.1% | 1173 out of 37603 genes, 3.1% | 1.38e-11 | View Result |
| membrane | 409 out of 690 genes, 59.3% | 17065 out of 37603 genes, 45.4% | 3.52e-11 | View Result |
| intrinsic to plasma membrane | 71 out of 690 genes, 10.3% | 1463 out of 37603 genes, 3.9% | 4.39e-11 | View Result |
| endoplasmic reticulum membrane | 61 out of 690 genes, 8.8% | 1142 out of 37603 genes, 3.0% | 4.57e-11 | View Result |
| endoplasmic reticulum part | 69 out of 690 genes, 10.0% | 1424 out of 37603 genes, 3.8% | 1.07e-10 | View Result |
| MHC class II protein complex | 9 out of 690 genes, 1.3% | 15 out of 37603 genes, 0.0% | 3.47e-10 | View Result |
| integral to plasma membrane | 67 out of 690 genes, 9.7% | 1399 out of 37603 genes, 3.7% | 4.12e-10 | View Result |
| cytosol | 109 out of 690 genes, 15.8% | 2945 out of 37603 genes, 7.8% | 5.68e-10 | View Result |
| cytoplasmic part | 355 out of 690 genes, 51.4% | 14592 out of 37603 genes, 38.8% | 2.54e-09 | View Result |
| extracellular organelle | 12 out of 690 genes, 1.7% | 45 out of 37603 genes, 0.1% | 7.49e-09 | View Result |
| extracellular membrane-bounded organelle | 12 out of 690 genes, 1.7% | 45 out of 37603 genes, 0.1% | 7.49e-09 | View Result |
| symbiont-containing vacuole | 10 out of 690 genes, 1.4% | 27 out of 37603 genes, 0.1% | 8.83e-09 | View Result |
| host cell cytoplasm | 10 out of 690 genes, 1.4% | 30 out of 37603 genes, 0.1% | 2.99e-08 | View Result |
| host cell cytoplasm part | 10 out of 690 genes, 1.4% | 30 out of 37603 genes, 0.1% | 2.99e-08 | View Result |
| integrin complex | 12 out of 690 genes, 1.7% | 53 out of 37603 genes, 0.1% | 6.08e-08 | View Result |
| host intracellular part | 10 out of 690 genes, 1.4% | 32 out of 37603 genes, 0.1% | 6.21e-08 | View Result |
| intracellular region of host | 10 out of 690 genes, 1.4% | 32 out of 37603 genes, 0.1% | 6.21e-08 | View Result |
| host | 10 out of 690 genes, 1.4% | 34 out of 37603 genes, 0.1% | 1.22e-07 | View Result |
| host cell part | 10 out of 690 genes, 1.4% | 34 out of 37603 genes, 0.1% | 1.22e-07 | View Result |
| extraorganismal space | 10 out of 690 genes, 1.4% | 34 out of 37603 genes, 0.1% | 1.22e-07 | View Result |
| host cell | 10 out of 690 genes, 1.4% | 34 out of 37603 genes, 0.1% | 1.22e-07 | View Result |
| other organism | 10 out of 690 genes, 1.4% | 34 out of 37603 genes, 0.1% | 1.22e-07 | View Result |
| other organism cell | 10 out of 690 genes, 1.4% | 34 out of 37603 genes, 0.1% | 1.22e-07 | View Result |
| other organism part | 10 out of 690 genes, 1.4% | 34 out of 37603 genes, 0.1% | 1.22e-07 | View Result |
| lysosome | 34 out of 690 genes, 4.9% | 560 out of 37603 genes, 1.5% | 5.64e-07 | View Result |
| immunological synapse | 8 out of 690 genes, 1.2% | 22 out of 37603 genes, 0.1% | 1.07e-06 | View Result |
| organelle membrane | 143 out of 690 genes, 20.7% | 4856 out of 37603 genes, 12.9% | 1.79e-06 | View Result |
| endomembrane system | 111 out of 690 genes, 16.1% | 3480 out of 37603 genes, 9.3% | 2.20e-06 | View Result |
| multivesicular body | 9 out of 690 genes, 1.3% | 35 out of 37603 genes, 0.1% | 3.53e-06 | View Result |
| membrane raft | 24 out of 690 genes, 3.5% | 335 out of 37603 genes, 0.9% | 6.53e-06 | View Result |
| symbiont-containing vacuole membrane | 7 out of 690 genes, 1.0% | 20 out of 37603 genes, 0.1% | 1.46e-05 | View Result |
| receptor complex | 22 out of 690 genes, 3.2% | 299 out of 37603 genes, 0.8% | 1.58e-05 | View Result |
| apical plasma membrane | 24 out of 690 genes, 3.5% | 360 out of 37603 genes, 1.0% | 2.54e-05 | View Result |
| apical part of cell | 28 out of 690 genes, 4.1% | 488 out of 37603 genes, 1.3% | 5.00e-05 | View Result |
| membrane part | 307 out of 690 genes, 44.5% | 13186 out of 37603 genes, 35.1% | 5.34e-05 | View Result |
| proteinaceous extracellular matrix | 29 out of 690 genes, 4.2% | 554 out of 37603 genes, 1.5% | 0.00020 | View Result |
| lytic vacuole | 35 out of 690 genes, 5.1% | 750 out of 37603 genes, 2.0% | 0.00021 | View Result |
| perinuclear region of cytoplasm | 33 out of 690 genes, 4.8% | 703 out of 37603 genes, 1.9% | 0.00038 | View Result |
| extracellular matrix | 37 out of 690 genes, 5.4% | 854 out of 37603 genes, 2.3% | 0.00058 | View Result |
| late endosome | 19 out of 690 genes, 2.8% | 301 out of 37603 genes, 0.8% | 0.00127 | View Result |
| lysosomal membrane | 13 out of 690 genes, 1.9% | 151 out of 37603 genes, 0.4% | 0.00161 | View Result |
| complement component C1 complex | 3 out of 690 genes, 0.4% | 3 out of 37603 genes, 0.0% | 0.00210 | View Result |
| secretory granule | 28 out of 690 genes, 4.1% | 610 out of 37603 genes, 1.6% | 0.00376 | View Result |
| proteasome core complex | 6 out of 690 genes, 0.9% | 29 out of 37603 genes, 0.1% | 0.00423 | View Result |
| vacuolar part | 18 out of 690 genes, 2.6% | 304 out of 37603 genes, 0.8% | 0.00542 | View Result |
| vacuole | 35 out of 690 genes, 5.1% | 875 out of 37603 genes, 2.3% | 0.00612 | View Result |
| cytoplasmic vesicle part | 28 out of 690 genes, 4.1% | 629 out of 37603 genes, 1.7% | 0.00653 | View Result |
| MHC class I protein complex | 5 out of 690 genes, 0.7% | 20 out of 37603 genes, 0.1% | 0.00865 | View Result |
| phagocytic vesicle | 8 out of 690 genes, 1.2% | 67 out of 37603 genes, 0.2% | 0.01061 | View Result |
| collagen | 10 out of 690 genes, 1.4% | 112 out of 37603 genes, 0.3% | 0.01463 | View Result |
| endocytic vesicle membrane | 10 out of 690 genes, 1.4% | 113 out of 37603 genes, 0.3% | 0.01579 | View Result |
| varicosity | 3 out of 690 genes, 0.4% | 5 out of 37603 genes, 0.0% | 0.02046 | View Result |
| mitochondrion | 81 out of 690 genes, 11.7% | 2848 out of 37603 genes, 7.6% | 0.02050 | View Result |
| connexon complex | 3 out of 690 genes, 0.4% | 7 out of 37603 genes, 0.0% | 0.06969 | View Result |
| vacuolar membrane | 14 out of 690 genes, 2.0% | 247 out of 37603 genes, 0.7% | 0.07288 | View Result |
| Golgi apparatus | 57 out of 690 genes, 8.3% | 1902 out of 37603 genes, 5.1% | 0.07412 | View Result |
| condensed nuclear chromosome outer kinetochore | 2 out of 690 genes, 0.3% | 2 out of 37603 genes, 0.0% | 0.11499 | View Result |
| macrophage migration inhibitory factor receptor complex | 2 out of 690 genes, 0.3% | 2 out of 37603 genes, 0.0% | 0.11499 | View Result |
| NOS2-CD74 complex | 2 out of 690 genes, 0.3% | 2 out of 37603 genes, 0.0% | 0.11499 | View Result |
| endosome | 37 out of 690 genes, 5.4% | 1107 out of 37603 genes, 2.9% | 0.13569 | View Result |
| anchored to membrane | 10 out of 690 genes, 1.4% | 151 out of 37603 genes, 0.4% | 0.17151 | View Result |
| secretory granule membrane | 10 out of 690 genes, 1.4% | 154 out of 37603 genes, 0.4% | 0.20007 | View Result |
| cytoplasmic vesicle membrane | 22 out of 690 genes, 3.2% | 549 out of 37603 genes, 1.5% | 0.20813 | View Result |
| intrinsic to membrane | 237 out of 690 genes, 34.3% | 10799 out of 37603 genes, 28.7% | 0.22983 | View Result |
| mitochondrial intermembrane space | 7 out of 690 genes, 1.0% | 80 out of 37603 genes, 0.2% | 0.23173 | View Result |
| endocytic vesicle | 13 out of 690 genes, 1.9% | 252 out of 37603 genes, 0.7% | 0.29243 | View Result |
| platelet alpha granule | 9 out of 690 genes, 1.3% | 135 out of 37603 genes, 0.4% | 0.30831 | View Result |
| Weibel-Palade body | 3 out of 690 genes, 0.4% | 11 out of 37603 genes, 0.0% | 0.31099 | View Result |
| phagocytic vesicle membrane | 5 out of 690 genes, 0.7% | 42 out of 37603 genes, 0.1% | 0.34027 | View Result |
| cell cortex | 16 out of 690 genes, 2.3% | 358 out of 37603 genes, 1.0% | 0.36696 | View Result |
| high-density lipoprotein particle | 5 out of 690 genes, 0.7% | 43 out of 37603 genes, 0.1% | 0.37929 | View Result |
| lysosomal lumen | 6 out of 690 genes, 0.9% | 65 out of 37603 genes, 0.2% | 0.42130 | View Result |
| organelle envelope lumen | 7 out of 690 genes, 1.0% | 89 out of 37603 genes, 0.2% | 0.43642 | View Result |
| vacuolar lumen | 6 out of 690 genes, 0.9% | 66 out of 37603 genes, 0.2% | 0.45635 | View Result |
| extracellular matrix part | 15 out of 690 genes, 2.2% | 335 out of 37603 genes, 0.9% | 0.50910 | View Result |
| dendritic shaft | 5 out of 690 genes, 0.7% | 46 out of 37603 genes, 0.1% | 0.51630 | View Result |
| vesicle | 67 out of 690 genes, 9.7% | 2526 out of 37603 genes, 6.7% | 0.56271 | View Result |
| actin cytoskeleton | 28 out of 690 genes, 4.1% | 830 out of 37603 genes, 2.2% | 0.58010 | View Result |
| vesicle membrane | 24 out of 690 genes, 3.5% | 672 out of 37603 genes, 1.8% | 0.58071 | View Result |
| endosomal part | 16 out of 690 genes, 2.3% | 375 out of 37603 genes, 1.0% | 0.58990 | View Result |
| intercellular canaliculus | 3 out of 690 genes, 0.4% | 14 out of 37603 genes, 0.0% | 0.65847 | View Result |
| actin filament | 10 out of 690 genes, 1.4% | 183 out of 37603 genes, 0.5% | 0.73695 | View Result |
| anchored to external side of plasma membrane | 3 out of 690 genes, 0.4% | 15 out of 37603 genes, 0.0% | 0.81192 | View Result |
| cytoplasmic vesicle | 60 out of 690 genes, 8.7% | 2245 out of 37603 genes, 6.0% | 0.82475 | View Result |
| proteasome complex | 7 out of 690 genes, 1.0% | 100 out of 37603 genes, 0.3% | 0.85357 | View Result |
| late endosome membrane | 7 out of 690 genes, 1.0% | 100 out of 37603 genes, 0.3% | 0.85357 | View Result |
| lamellipodium | 10 out of 690 genes, 1.4% | 191 out of 37603 genes, 0.5% | 1 | View Result |
| cytoplasmic membrane-bounded vesicle lumen | 6 out of 690 genes, 0.9% | 77 out of 37603 genes, 0.2% | 1 | View Result |
| Ndc80 complex | 2 out of 690 genes, 0.3% | 5 out of 37603 genes, 0.0% | 1 | View Result |
| chromaffin granule membrane | 2 out of 690 genes, 0.3% | 5 out of 37603 genes, 0.0% | 1 | View Result |
| membrane-bounded vesicle | 55 out of 690 genes, 8.0% | 2048 out of 37603 genes, 5.4% | 1 | View Result |
| cell projection | 76 out of 690 genes, 11.0% | 3024 out of 37603 genes, 8.0% | 1 | View Result |
| endosome membrane | 15 out of 690 genes, 2.2% | 366 out of 37603 genes, 1.0% | 1 | View Result |
| neuron projection | 46 out of 690 genes, 6.7% | 1654 out of 37603 genes, 4.4% | 1 | View Result |
| filopodium | 7 out of 690 genes, 1.0% | 112 out of 37603 genes, 0.3% | 1 | View Result |
| NADPH oxidase complex | 2 out of 690 genes, 0.3% | 6 out of 37603 genes, 0.0% | 1 | View Result |
| cytoplasmic membrane-bounded vesicle | 51 out of 690 genes, 7.4% | 1912 out of 37603 genes, 5.1% | 1 | View Result |
| zymogen granule | 3 out of 690 genes, 0.4% | 20 out of 37603 genes, 0.1% | 1 | View Result |
| vesicle lumen | 6 out of 690 genes, 0.9% | 88 out of 37603 genes, 0.2% | 1 | View Result |
| intrinsic to external side of plasma membrane | 3 out of 690 genes, 0.4% | 21 out of 37603 genes, 0.1% | 1 | View Result |
| condensed nuclear chromosome kinetochore | 2 out of 690 genes, 0.3% | 8 out of 37603 genes, 0.0% | 1 | View Result |
| chromaffin granule | 2 out of 690 genes, 0.3% | 8 out of 37603 genes, 0.0% | 1 | View Result |
| vesicular fraction | 17 out of 690 genes, 2.5% | 487 out of 37603 genes, 1.3% | 1 | View Result |
| trans-Golgi network membrane | 3 out of 690 genes, 0.4% | 24 out of 37603 genes, 0.1% | 1 | View Result |
| dendrite | 21 out of 690 genes, 3.0% | 655 out of 37603 genes, 1.7% | 1 | View Result |
| type III intermediate filament | 2 out of 690 genes, 0.3% | 9 out of 37603 genes, 0.0% | 1 | View Result |
| anchored to plasma membrane | 4 out of 690 genes, 0.6% | 48 out of 37603 genes, 0.1% | 1 | View Result |
| platelet alpha granule lumen | 5 out of 690 genes, 0.7% | 74 out of 37603 genes, 0.2% | 1 | View Result |
| cortical cytoskeleton | 6 out of 690 genes, 0.9% | 103 out of 37603 genes, 0.3% | 1 | View Result |
| secretory granule lumen | 5 out of 690 genes, 0.7% | 75 out of 37603 genes, 0.2% | 1 | View Result |
| cell cortex part | 8 out of 690 genes, 1.2% | 171 out of 37603 genes, 0.5% | 1 | View Result |
| main axon | 4 out of 690 genes, 0.6% | 52 out of 37603 genes, 0.1% | 1 | View Result |
| intermediate filament | 7 out of 690 genes, 1.0% | 144 out of 37603 genes, 0.4% | 1 | View Result |
| organelle outer membrane | 10 out of 690 genes, 1.4% | 251 out of 37603 genes, 0.7% | 1 | View Result |
| neuronal cell body | 16 out of 690 genes, 2.3% | 494 out of 37603 genes, 1.3% | 1 | View Result |
| axon | 17 out of 690 genes, 2.5% | 539 out of 37603 genes, 1.4% | 1 | View Result |
| zymogen granule membrane | 2 out of 690 genes, 0.3% | 13 out of 37603 genes, 0.0% | 1 | View Result |
| microvillus | 7 out of 690 genes, 1.0% | 154 out of 37603 genes, 0.4% | 1 | View Result |
| early endosome | 11 out of 690 genes, 1.6% | 301 out of 37603 genes, 0.8% | 1 | View Result |
| uropod | 2 out of 690 genes, 0.3% | 14 out of 37603 genes, 0.0% | 1 | View Result |
| I-kappaB/NF-kappaB complex | 2 out of 690 genes, 0.3% | 14 out of 37603 genes, 0.0% | 1 | View Result |
| gap junction | 3 out of 690 genes, 0.4% | 36 out of 37603 genes, 0.1% | 1 | View Result |
| outer membrane | 10 out of 690 genes, 1.4% | 271 out of 37603 genes, 0.7% | 1 | View Result |
| condensed nuclear chromosome, centromeric region | 2 out of 690 genes, 0.3% | 15 out of 37603 genes, 0.0% | 1 | View Result |
| chromatin assembly complex | 2 out of 690 genes, 0.3% | 15 out of 37603 genes, 0.0% | 1 | View Result |
| growth cone | 7 out of 690 genes, 1.0% | 162 out of 37603 genes, 0.4% | 1 | View Result |
| recycling endosome membrane | 3 out of 690 genes, 0.4% | 38 out of 37603 genes, 0.1% | 1 | View Result |
| filopodium membrane | 2 out of 690 genes, 0.3% | 16 out of 37603 genes, 0.0% | 1 | View Result |
| melanosome | 5 out of 690 genes, 0.7% | 102 out of 37603 genes, 0.3% | 1 | View Result |
| extracellular vesicular exosome | 2 out of 690 genes, 0.3% | 18 out of 37603 genes, 0.0% | 1 | View Result |
| mitochondrial outer membrane | 8 out of 690 genes, 1.2% | 214 out of 37603 genes, 0.6% | 1 | View Result |
| cell-cell adherens junction | 6 out of 690 genes, 0.9% | 140 out of 37603 genes, 0.4% | 1 | View Result |
| cortical actin cytoskeleton | 4 out of 690 genes, 0.6% | 75 out of 37603 genes, 0.2% | 1 | View Result |
| integral to lumenal side of endoplasmic reticulum membrane | 2 out of 690 genes, 0.3% | 20 out of 37603 genes, 0.1% | 1 | View Result |
| clathrin coated vesicle membrane | 6 out of 690 genes, 0.9% | 145 out of 37603 genes, 0.4% | 1 | View Result |
| protein-lipid complex | 5 out of 690 genes, 0.7% | 112 out of 37603 genes, 0.3% | 1 | View Result |
| plasma lipoprotein particle | 5 out of 690 genes, 0.7% | 112 out of 37603 genes, 0.3% | 1 | View Result |
| extrinsic to membrane | 9 out of 690 genes, 1.3% | 264 out of 37603 genes, 0.7% | 1 | View Result |
| trailing edge | 2 out of 690 genes, 0.3% | 21 out of 37603 genes, 0.1% | 1 | View Result |
| cell-cell junction | 20 out of 690 genes, 2.9% | 742 out of 37603 genes, 2.0% | 1 | View Result |
| condensed chromosome outer kinetochore | 2 out of 690 genes, 0.3% | 22 out of 37603 genes, 0.1% | 1 | View Result |
| CD40 receptor complex | 2 out of 690 genes, 0.3% | 22 out of 37603 genes, 0.1% | 1 | View Result |
| internal side of plasma membrane | 8 out of 690 genes, 1.2% | 229 out of 37603 genes, 0.6% | 1 | View Result |
| acrosomal membrane | 3 out of 690 genes, 0.4% | 52 out of 37603 genes, 0.1% | 1 | View Result |
| fascia adherens | 2 out of 690 genes, 0.3% | 24 out of 37603 genes, 0.1% | 1 | View Result |
| intermediate filament cytoskeleton | 7 out of 690 genes, 1.0% | 197 out of 37603 genes, 0.5% | 1 | View Result |
| hemidesmosome | 2 out of 690 genes, 0.3% | 25 out of 37603 genes, 0.1% | 1 | View Result |
| cell leading edge | 20 out of 690 genes, 2.9% | 771 out of 37603 genes, 2.1% | 1 | View Result |
| nuclear envelope | 17 out of 690 genes, 2.5% | 638 out of 37603 genes, 1.7% | 1 | View Result |
| recycling endosome | 4 out of 690 genes, 0.6% | 91 out of 37603 genes, 0.2% | 1 | View Result |
| phagocytic cup | 3 out of 690 genes, 0.4% | 57 out of 37603 genes, 0.2% | 1 | View Result |
| intercalated disc | 3 out of 690 genes, 0.4% | 57 out of 37603 genes, 0.2% | 1 | View Result |
| organelle envelope | 44 out of 690 genes, 6.4% | 1940 out of 37603 genes, 5.2% | 1 | View Result |
| mitochondrial matrix | 12 out of 690 genes, 1.7% | 422 out of 37603 genes, 1.1% | 1 | View Result |
| coated vesicle membrane | 7 out of 690 genes, 1.0% | 209 out of 37603 genes, 0.6% | 1 | View Result |
| membrane fraction | 19 out of 690 genes, 2.8% | 745 out of 37603 genes, 2.0% | 1 | View Result |
| SCF ubiquitin ligase complex | 2 out of 690 genes, 0.3% | 29 out of 37603 genes, 0.1% | 1 | View Result |
| endoplasmic reticulum lumen | 6 out of 690 genes, 0.9% | 174 out of 37603 genes, 0.5% | 1 | View Result |
| envelope | 44 out of 690 genes, 6.4% | 1966 out of 37603 genes, 5.2% | 1 | View Result |
| Golgi-associated vesicle | 5 out of 690 genes, 0.7% | 135 out of 37603 genes, 0.4% | 1 | View Result |
| intrinsic to endoplasmic reticulum membrane | 7 out of 690 genes, 1.0% | 217 out of 37603 genes, 0.6% | 1 | View Result |
| mitochondrial part | 36 out of 690 genes, 5.2% | 1586 out of 37603 genes, 4.2% | 1 | View Result |
| rough endoplasmic reticulum | 3 out of 690 genes, 0.4% | 64 out of 37603 genes, 0.2% | 1 | View Result |
| contractile fiber | 12 out of 690 genes, 1.7% | 441 out of 37603 genes, 1.2% | 1 | View Result |
| heterotrimeric G-protein complex | 2 out of 690 genes, 0.3% | 32 out of 37603 genes, 0.1% | 1 | View Result |
| clathrin adaptor complex | 2 out of 690 genes, 0.3% | 32 out of 37603 genes, 0.1% | 1 | View Result |
| Golgi membrane | 20 out of 690 genes, 2.9% | 819 out of 37603 genes, 2.2% | 1 | View Result |
| aggresome | 2 out of 690 genes, 0.3% | 33 out of 37603 genes, 0.1% | 1 | View Result |
| mitochondrial envelope | 25 out of 690 genes, 3.6% | 1070 out of 37603 genes, 2.8% | 1 | View Result |
| cell part | 601 out of 690 genes, 87.1% | 32173 out of 37603 genes, 85.6% | 1 | View Result |
| lateral plasma membrane | 3 out of 690 genes, 0.4% | 69 out of 37603 genes, 0.2% | 1 | View Result |
| cell-cell contact zone | 3 out of 690 genes, 0.4% | 69 out of 37603 genes, 0.2% | 1 | View Result |
| cell | 601 out of 690 genes, 87.1% | 32174 out of 37603 genes, 85.6% | 1 | View Result |
| ER to Golgi transport vesicle membrane | 2 out of 690 genes, 0.3% | 35 out of 37603 genes, 0.1% | 1 | View Result |
| clathrin-coated endocytic vesicle membrane | 2 out of 690 genes, 0.3% | 36 out of 37603 genes, 0.1% | 1 | View Result |
| cell body | 22 out of 690 genes, 3.2% | 936 out of 37603 genes, 2.5% | 1 | View Result |
| photoreceptor outer segment | 2 out of 690 genes, 0.3% | 37 out of 37603 genes, 0.1% | 1 | View Result |
| nuclear outer membrane | 2 out of 690 genes, 0.3% | 37 out of 37603 genes, 0.1% | 1 | View Result |
| ruffle membrane | 4 out of 690 genes, 0.6% | 112 out of 37603 genes, 0.3% | 1 | View Result |
| acrosomal vesicle | 5 out of 690 genes, 0.7% | 153 out of 37603 genes, 0.4% | 1 | View Result |
| Golgi apparatus part | 26 out of 690 genes, 3.8% | 1145 out of 37603 genes, 3.0% | 1 | View Result |
| clathrin-coated vesicle | 11 out of 690 genes, 1.6% | 423 out of 37603 genes, 1.1% | 1 | View Result |
| extrinsic to plasma membrane | 5 out of 690 genes, 0.7% | 156 out of 37603 genes, 0.4% | 1 | View Result |
| adherens junction | 13 out of 690 genes, 1.9% | 518 out of 37603 genes, 1.4% | 1 | View Result |
| cell junction | 33 out of 690 genes, 4.8% | 1502 out of 37603 genes, 4.0% | 1 | View Result |
| striated muscle thin filament | 2 out of 690 genes, 0.3% | 40 out of 37603 genes, 0.1% | 1 | View Result |
| cell projection part | 25 out of 690 genes, 3.6% | 1106 out of 37603 genes, 2.9% | 1 | View Result |
| mitochondrial inner membrane | 14 out of 690 genes, 2.0% | 577 out of 37603 genes, 1.5% | 1 | View Result |
| pigment granule | 6 out of 690 genes, 0.9% | 206 out of 37603 genes, 0.5% | 1 | View Result |
| clathrin-coated endocytic vesicle | 2 out of 690 genes, 0.3% | 42 out of 37603 genes, 0.1% | 1 | View Result |
| Golgi-associated vesicle membrane | 3 out of 690 genes, 0.4% | 80 out of 37603 genes, 0.2% | 1 | View Result |
| protein complex | 133 out of 690 genes, 19.3% | 6728 out of 37603 genes, 17.9% | 1 | View Result |
| caveola | 4 out of 690 genes, 0.6% | 122 out of 37603 genes, 0.3% | 1 | View Result |
| integral to endoplasmic reticulum membrane | 4 out of 690 genes, 0.6% | 122 out of 37603 genes, 0.3% | 1 | View Result |
| synaptic vesicle membrane | 2 out of 690 genes, 0.3% | 44 out of 37603 genes, 0.1% | 1 | View Result |
| trans-Golgi network | 5 out of 690 genes, 0.7% | 167 out of 37603 genes, 0.4% | 1 | View Result |
| leading edge membrane | 6 out of 690 genes, 0.9% | 212 out of 37603 genes, 0.6% | 1 | View Result |
| H4/H2A histone acetyltransferase complex | 2 out of 690 genes, 0.3% | 45 out of 37603 genes, 0.1% | 1 | View Result |
| mitochondrial membrane | 21 out of 690 genes, 3.0% | 943 out of 37603 genes, 2.5% | 1 | View Result |
| ER to Golgi transport vesicle | 2 out of 690 genes, 0.3% | 48 out of 37603 genes, 0.1% | 1 | View Result |
| nuclear matrix | 4 out of 690 genes, 0.6% | 132 out of 37603 genes, 0.4% | 1 | View Result |
| transport vesicle | 6 out of 690 genes, 0.9% | 228 out of 37603 genes, 0.6% | 1 | View Result |
| DNA-directed RNA polymerase II, holoenzyme | 4 out of 690 genes, 0.6% | 140 out of 37603 genes, 0.4% | 1 | View Result |
| Golgi lumen | 2 out of 690 genes, 0.3% | 54 out of 37603 genes, 0.1% | 1 | View Result |
| perikaryon | 2 out of 690 genes, 0.3% | 54 out of 37603 genes, 0.1% | 1 | View Result |
| coated vesicle | 12 out of 690 genes, 1.7% | 526 out of 37603 genes, 1.4% | 1 | View Result |
| anchoring junction | 13 out of 690 genes, 1.9% | 581 out of 37603 genes, 1.5% | 1 | View Result |
| sarcolemma | 5 out of 690 genes, 0.7% | 193 out of 37603 genes, 0.5% | 1 | View Result |
| Golgi cisterna | 3 out of 690 genes, 0.4% | 103 out of 37603 genes, 0.3% | 1 | View Result |
| organelle subcompartment | 3 out of 690 genes, 0.4% | 105 out of 37603 genes, 0.3% | 1 | View Result |
| dendritic spine | 6 out of 690 genes, 0.9% | 248 out of 37603 genes, 0.7% | 1 | View Result |
| neuron spine | 6 out of 690 genes, 0.9% | 248 out of 37603 genes, 0.7% | 1 | View Result |
| nuclear periphery | 6 out of 690 genes, 0.9% | 252 out of 37603 genes, 0.7% | 1 | View Result |
| integral to organelle membrane | 6 out of 690 genes, 0.9% | 257 out of 37603 genes, 0.7% | 1 | View Result |
| DNA-directed RNA polymerase complex | 4 out of 690 genes, 0.6% | 159 out of 37603 genes, 0.4% | 1 | View Result |
| nuclear DNA-directed RNA polymerase complex | 4 out of 690 genes, 0.6% | 159 out of 37603 genes, 0.4% | 1 | View Result |
| pore complex | 3 out of 690 genes, 0.4% | 112 out of 37603 genes, 0.3% | 1 | View Result |
| AP-type membrane coat adaptor complex | 2 out of 690 genes, 0.3% | 66 out of 37603 genes, 0.2% | 1 | View Result |
| transport vesicle membrane | 3 out of 690 genes, 0.4% | 113 out of 37603 genes, 0.3% | 1 | View Result |
| site of polarized growth | 7 out of 690 genes, 1.0% | 311 out of 37603 genes, 0.8% | 1 | View Result |
| extrinsic to internal side of plasma membrane | 3 out of 690 genes, 0.4% | 114 out of 37603 genes, 0.3% | 1 | View Result |
| basal plasma membrane | 2 out of 690 genes, 0.3% | 67 out of 37603 genes, 0.2% | 1 | View Result |
| organelle inner membrane | 19 out of 690 genes, 2.8% | 932 out of 37603 genes, 2.5% | 1 | View Result |
| RNA polymerase complex | 4 out of 690 genes, 0.6% | 164 out of 37603 genes, 0.4% | 1 | View Result |
| replication fork | 2 out of 690 genes, 0.3% | 77 out of 37603 genes, 0.2% | 1 | View Result |
| endoplasmic reticulum-Golgi intermediate compartment | 2 out of 690 genes, 0.3% | 79 out of 37603 genes, 0.2% | 1 | View Result |
| basal part of cell | 2 out of 690 genes, 0.3% | 79 out of 37603 genes, 0.2% | 1 | View Result |
| stress fiber | 2 out of 690 genes, 0.3% | 81 out of 37603 genes, 0.2% | 1 | View Result |
| clathrin coat | 2 out of 690 genes, 0.3% | 81 out of 37603 genes, 0.2% | 1 | View Result |
| cell projection membrane | 7 out of 690 genes, 1.0% | 349 out of 37603 genes, 0.9% | 1 | View Result |
| ruffle | 6 out of 690 genes, 0.9% | 296 out of 37603 genes, 0.8% | 1 | View Result |
| lipid particle | 2 out of 690 genes, 0.3% | 89 out of 37603 genes, 0.2% | 1 | View Result |
| intrinsic to organelle membrane | 9 out of 690 genes, 1.3% | 470 out of 37603 genes, 1.2% | 1 | View Result |
| myelin sheath | 2 out of 690 genes, 0.3% | 91 out of 37603 genes, 0.2% | 1 | View Result |
| flagellum | 2 out of 690 genes, 0.3% | 93 out of 37603 genes, 0.2% | 1 | View Result |
| histone deacetylase complex | 3 out of 690 genes, 0.4% | 150 out of 37603 genes, 0.4% | 1 | View Result |
| basement membrane | 4 out of 690 genes, 0.6% | 206 out of 37603 genes, 0.5% | 1 | View Result |
| focal adhesion | 4 out of 690 genes, 0.6% | 207 out of 37603 genes, 0.6% | 1 | View Result |
| I band | 4 out of 690 genes, 0.6% | 208 out of 37603 genes, 0.6% | 1 | View Result |
| ion channel complex | 4 out of 690 genes, 0.6% | 208 out of 37603 genes, 0.6% | 1 | View Result |
| Golgi stack | 3 out of 690 genes, 0.4% | 155 out of 37603 genes, 0.4% | 1 | View Result |
| actomyosin | 2 out of 690 genes, 0.3% | 100 out of 37603 genes, 0.3% | 1 | View Result |
| cell-substrate junction | 7 out of 690 genes, 1.0% | 381 out of 37603 genes, 1.0% | 1 | View Result |
| tight junction | 3 out of 690 genes, 0.4% | 157 out of 37603 genes, 0.4% | 1 | View Result |
| membrane coat | 3 out of 690 genes, 0.4% | 157 out of 37603 genes, 0.4% | 1 | View Result |
| coated membrane | 3 out of 690 genes, 0.4% | 157 out of 37603 genes, 0.4% | 1 | View Result |
| occluding junction | 3 out of 690 genes, 0.4% | 157 out of 37603 genes, 0.4% | 1 | View Result |
| inclusion body | 2 out of 690 genes, 0.3% | 101 out of 37603 genes, 0.3% | 1 | View Result |
| myosin complex | 2 out of 690 genes, 0.3% | 101 out of 37603 genes, 0.3% | 1 | View Result |
| Z disc | 2 out of 690 genes, 0.3% | 101 out of 37603 genes, 0.3% | 1 | View Result |
| synaptic vesicle | 3 out of 690 genes, 0.4% | 161 out of 37603 genes, 0.4% | 1 | View Result |
| actin filament bundle | 3 out of 690 genes, 0.4% | 165 out of 37603 genes, 0.4% | 1 | View Result |
| myofibril | 7 out of 690 genes, 1.0% | 395 out of 37603 genes, 1.1% | 1 | View Result |
| peroxisome | 5 out of 690 genes, 0.7% | 284 out of 37603 genes, 0.8% | 1 | View Result |
| synapse | 13 out of 690 genes, 1.9% | 741 out of 37603 genes, 2.0% | 1 | View Result |
| basolateral plasma membrane | 12 out of 690 genes, 1.7% | 687 out of 37603 genes, 1.8% | 1 | View Result |
| sarcomere | 5 out of 690 genes, 0.7% | 302 out of 37603 genes, 0.8% | 1 | View Result |
| condensed chromosome kinetochore | 2 out of 690 genes, 0.3% | 121 out of 37603 genes, 0.3% | 1 | View Result |
| axon part | 5 out of 690 genes, 0.7% | 304 out of 37603 genes, 0.8% | 1 | View Result |
| protein serine/threonine phosphatase complex | 2 out of 690 genes, 0.3% | 123 out of 37603 genes, 0.3% | 1 | View Result |
| midbody | 2 out of 690 genes, 0.3% | 123 out of 37603 genes, 0.3% | 1 | View Result |
| cytoskeleton | 71 out of 690 genes, 10.3% | 4034 out of 37603 genes, 10.7% | 1 | View Result |
| chromatin remodeling complex | 6 out of 690 genes, 0.9% | 368 out of 37603 genes, 1.0% | 1 | View Result |
| transcriptional repressor complex | 2 out of 690 genes, 0.3% | 128 out of 37603 genes, 0.3% | 1 | View Result |
| condensed chromosome, centromeric region | 2 out of 690 genes, 0.3% | 130 out of 37603 genes, 0.3% | 1 | View Result |
| histone acetyltransferase complex | 3 out of 690 genes, 0.4% | 198 out of 37603 genes, 0.5% | 1 | View Result |
| protein-DNA complex | 2 out of 690 genes, 0.3% | 141 out of 37603 genes, 0.4% | 1 | View Result |
| contractile fiber part | 5 out of 690 genes, 0.7% | 339 out of 37603 genes, 0.9% | 1 | View Result |
| microbody | 5 out of 690 genes, 0.7% | 344 out of 37603 genes, 0.9% | 1 | View Result |
| PML body | 2 out of 690 genes, 0.3% | 150 out of 37603 genes, 0.4% | 1 | View Result |
| histone methyltransferase complex | 2 out of 690 genes, 0.3% | 155 out of 37603 genes, 0.4% | 1 | View Result |
| cell-substrate adherens junction | 5 out of 690 genes, 0.7% | 359 out of 37603 genes, 1.0% | 1 | View Result |
| brush border | 2 out of 690 genes, 0.3% | 160 out of 37603 genes, 0.4% | 1 | View Result |
| insoluble fraction | 24 out of 690 genes, 3.5% | 1517 out of 37603 genes, 4.0% | 1 | View Result |
| condensed nuclear chromosome | 2 out of 690 genes, 0.3% | 162 out of 37603 genes, 0.4% | 1 | View Result |
| cilium | 5 out of 690 genes, 0.7% | 367 out of 37603 genes, 1.0% | 1 | View Result |
| ubiquitin ligase complex | 4 out of 690 genes, 0.6% | 303 out of 37603 genes, 0.8% | 1 | View Result |
| primary cilium | 3 out of 690 genes, 0.4% | 241 out of 37603 genes, 0.6% | 1 | View Result |
| postsynaptic density | 2 out of 690 genes, 0.3% | 170 out of 37603 genes, 0.5% | 1 | View Result |
| dendritic spine head | 2 out of 690 genes, 0.3% | 170 out of 37603 genes, 0.5% | 1 | View Result |
| cullin-RING ubiquitin ligase complex | 2 out of 690 genes, 0.3% | 173 out of 37603 genes, 0.5% | 1 | View Result |
| macromolecular complex | 146 out of 690 genes, 21.2% | 8498 out of 37603 genes, 22.6% | 1 | View Result |
| methyltransferase complex | 2 out of 690 genes, 0.3% | 176 out of 37603 genes, 0.5% | 1 | View Result |
| synapse part | 6 out of 690 genes, 0.9% | 453 out of 37603 genes, 1.2% | 1 | View Result |
| synaptic membrane | 2 out of 690 genes, 0.3% | 184 out of 37603 genes, 0.5% | 1 | View Result |
| intracellular organelle part | 244 out of 690 genes, 35.4% | 13998 out of 37603 genes, 37.2% | 1 | View Result |
| nonmotile primary cilium | 2 out of 690 genes, 0.3% | 192 out of 37603 genes, 0.5% | 1 | View Result |
| membrane-bounded organelle | 403 out of 690 genes, 58.4% | 22716 out of 37603 genes, 60.4% | 1 | View Result |
| apical junction complex | 4 out of 690 genes, 0.6% | 339 out of 37603 genes, 0.9% | 1 | View Result |
| apicolateral plasma membrane | 4 out of 690 genes, 0.6% | 348 out of 37603 genes, 0.9% | 1 | View Result |
| intracellular part | 515 out of 690 genes, 74.6% | 28781 out of 37603 genes, 76.5% | 1 | View Result |
| organelle part | 257 out of 690 genes, 37.2% | 14834 out of 37603 genes, 39.4% | 1 | View Result |
| nuclear membrane | 3 out of 690 genes, 0.4% | 287 out of 37603 genes, 0.8% | 1 | View Result |
| nuclear speck | 2 out of 690 genes, 0.3% | 213 out of 37603 genes, 0.6% | 1 | View Result |
| cytoskeletal part | 40 out of 690 genes, 5.8% | 2623 out of 37603 genes, 7.0% | 1 | View Result |
| cell fraction | 30 out of 690 genes, 4.3% | 2030 out of 37603 genes, 5.4% | 1 | View Result |
| intracellular | 522 out of 690 genes, 75.7% | 29285 out of 37603 genes, 77.9% | 1 | View Result |
| intracellular membrane-bounded organelle | 395 out of 690 genes, 57.2% | 22524 out of 37603 genes, 59.9% | 1 | View Result |
| mitochondrial membrane part | 2 out of 690 genes, 0.3% | 234 out of 37603 genes, 0.6% | 1 | View Result |
| nuclear chromosome part | 6 out of 690 genes, 0.9% | 547 out of 37603 genes, 1.5% | 1 | View Result |
| kinetochore | 2 out of 690 genes, 0.3% | 242 out of 37603 genes, 0.6% | 1 | View Result |
| transcription factor complex | 6 out of 690 genes, 0.9% | 555 out of 37603 genes, 1.5% | 1 | View Result |
| microtubule | 5 out of 690 genes, 0.7% | 499 out of 37603 genes, 1.3% | 1 | View Result |
| nuclear chromatin | 3 out of 690 genes, 0.4% | 346 out of 37603 genes, 0.9% | 1 | View Result |
| chromosome, centromeric region | 3 out of 690 genes, 0.4% | 354 out of 37603 genes, 0.9% | 1 | View Result |
| nuclear chromosome | 7 out of 690 genes, 1.0% | 677 out of 37603 genes, 1.8% | 1 | View Result |
| intracellular organelle | 433 out of 690 genes, 62.8% | 24874 out of 37603 genes, 66.1% | 1 | View Result |
| organelle | 435 out of 690 genes, 63.0% | 25054 out of 37603 genes, 66.6% | 1 | View Result |
| condensed chromosome | 2 out of 690 genes, 0.3% | 318 out of 37603 genes, 0.8% | 1 | View Result |
| ribosome | 3 out of 690 genes, 0.4% | 410 out of 37603 genes, 1.1% | 1 | View Result |
| chromatin | 7 out of 690 genes, 1.0% | 737 out of 37603 genes, 2.0% | 1 | View Result |
| nucleus | 165 out of 690 genes, 23.9% | 10441 out of 37603 genes, 27.8% | 1 | View Result |
| nucleolus | 25 out of 690 genes, 3.6% | 2075 out of 37603 genes, 5.5% | 1 | View Result |
| centrosome | 5 out of 690 genes, 0.7% | 672 out of 37603 genes, 1.8% | 1 | View Result |
| nucleoplasm | 33 out of 690 genes, 4.8% | 2713 out of 37603 genes, 7.2% | 1 | View Result |
| spindle | 2 out of 690 genes, 0.3% | 496 out of 37603 genes, 1.3% | 1 | View Result |
| microtubule organizing center | 8 out of 690 genes, 1.2% | 1077 out of 37603 genes, 2.9% | 1 | View Result |
| nuclear body | 4 out of 690 genes, 0.6% | 729 out of 37603 genes, 1.9% | 1 | View Result |
| non-membrane-bounded organelle | 110 out of 690 genes, 15.9% | 7901 out of 37603 genes, 21.0% | 1 | View Result |
| intracellular non-membrane-bounded organelle | 110 out of 690 genes, 15.9% | 7901 out of 37603 genes, 21.0% | 1 | View Result |
| nucleoplasm part | 16 out of 690 genes, 2.3% | 1841 out of 37603 genes, 4.9% | 1 | View Result |
| chromosomal part | 10 out of 690 genes, 1.4% | 1456 out of 37603 genes, 3.9% | 1 | View Result |
| membrane-enclosed lumen | 93 out of 690 genes, 13.5% | 7219 out of 37603 genes, 19.2% | 1 | View Result |
| microtubule cytoskeleton | 17 out of 690 genes, 2.5% | 2086 out of 37603 genes, 5.5% | 1 | View Result |
| ribonucleoprotein complex | 8 out of 690 genes, 1.2% | 1367 out of 37603 genes, 3.6% | 1 | View Result |
| chromosome | 11 out of 690 genes, 1.6% | 1683 out of 37603 genes, 4.5% | 1 | View Result |
| organelle lumen | 86 out of 690 genes, 12.5% | 7139 out of 37603 genes, 19.0% | 1 | View Result |
| intracellular organelle lumen | 81 out of 690 genes, 11.7% | 7060 out of 37603 genes, 18.8% | 1 | View Result |
| nuclear part | 77 out of 690 genes, 11.2% | 7093 out of 37603 genes, 18.9% | 1 | View Result |
| nuclear lumen | 62 out of 690 genes, 9.0% | 6408 out of 37603 genes, 17.0% | 1 | View Result |

| Gene Ontology term | Genes annotated to the term |
| --- | --- |
| cell periphery | Unigene26055\_Mf\_liverA, Unigene21684\_Mf\_liverA, CL5640.Contig1\_Mf\_liverA, NM\_009898, Unigene25721\_Mf\_liverA, CL3835.Contig2\_Mf\_liverA, Unigene38831\_Mf\_liverA, Unigene22874\_Mf\_liverA, Unigene6959\_Mf\_liverA, Unigene28899\_Mf\_liverA, Unigene24111\_Mf\_liverA, Unigene28186\_Mf\_liverA, Unigene14603\_Mf\_liverA, Unigene29308\_Mf\_liverA, Unigene24883\_Mf\_liverA, NM\_010378, Unigene29424\_Mf\_liverA, Unigene8473\_Mf\_liverA, Unigene36849\_Mf\_liverA, Unigene26053\_Mf\_liverA, Unigene29008\_Mf\_liverA, Unigene25055\_Mf\_liverA, Unigene27593\_Mf\_liverA, CL2339.Contig1\_Mf\_liverA, Unigene22433\_Mf\_liverA, Unigene29788\_Mf\_liverA, Unigene36762\_Mf\_liverA, Unigene16463\_Mf\_liverA, CL777.Contig8\_Mf\_liverA, Unigene14816\_Mf\_liverA, Unigene37698\_Mf\_liverA, Unigene38689\_Mf\_liverA, Unigene30878\_Mf\_liverA, Unigene1137\_Mf\_liverA, Unigene27294\_Mf\_liverA, Unigene24252\_Mf\_liverA, NR\_004446, Unigene24344\_Mf\_liverA, CL1555.Contig1\_Mf\_liverA, Unigene18500\_Mf\_liverA, CL5586.Contig1\_Mf\_liverA, Unigene36889\_Mf\_liverA, Unigene32564\_Mf\_liverA, Unigene37535\_Mf\_liverA, Unigene24112\_Mf\_liverA, Unigene15703\_Mf\_liverA, CL2001.Contig1\_Mf\_liverA, Unigene38110\_Mf\_liverA, Unigene35816\_Mf\_liverA, Unigene31623\_Mf\_liverA, Unigene26194\_Mf\_liverA, Unigene31333\_Mf\_liverA, CL593.Contig2\_Mf\_liverA, CL2260.Contig1\_Mf\_liverA, Unigene13593\_Mf\_liverA, Unigene30369\_Mf\_liverA, NM\_010233, Unigene4686\_Mf\_liverA, Unigene4630\_Mf\_liverA, Unigene11\_Mf\_liverA, Unigene34394\_Mf\_liverA, Unigene843\_Mf\_liverA, Unigene5138\_Mf\_liverA, CL1493.Contig2\_Mf\_liverA, CL3575.Contig1\_Mf\_liverA, Unigene12153\_Mf\_liverA, Unigene37150\_Mf\_liverA, Unigene22432\_Mf\_liverA, Unigene25046\_Mf\_liverA, Unigene2745\_Mf\_liverA, Unigene25462\_Mf\_liverA, Unigene802\_Mf\_liverA, Unigene5552\_Mf\_liverA, CL5807.Contig1\_Mf\_liverA, NM\_153795, Unigene13363\_Mf\_liverA, Unigene28822\_Mf\_liverA, Unigene26065\_Mf\_liverA, CL2855.Contig2\_Mf\_liverA, Unigene31988\_Mf\_liverA, Unigene18125\_Mf\_liverA, CL4293.Contig1\_Mf\_liverA, Unigene13097\_Mf\_liverA, Unigene25226\_Mf\_liverA, Unigene33459\_Mf\_liverA, CL848.Contig2\_Mf\_liverA, Unigene23870\_Mf\_liverA, Unigene21561\_Mf\_liverA, Unigene4922\_Mf\_liverA, Unigene13143\_Mf\_liverA, CL1263.Contig1\_Mf\_liverA, Unigene30261\_Mf\_liverA, Unigene34746\_Mf\_liverA, Unigene24503\_Mf\_liverA, Unigene139\_Mf\_liverA, Unigene15583\_Mf\_liverA, Unigene27422\_Mf\_liverA, Unigene33560\_Mf\_liverA, CL3816.Contig1\_Mf\_liverA, CL4701.Contig1\_Mf\_liverA, Unigene25333\_Mf\_liverA, CL993.Contig2\_Mf\_liverA, CL5631.Contig1\_Mf\_liverA, Unigene37575\_Mf\_liverA, Unigene30584\_Mf\_liverA, Unigene17579\_Mf\_liverA, CL4220.Contig2\_Mf\_liverA, Unigene27081\_Mf\_liverA, Unigene431\_Mf\_liverA, Unigene27547\_Mf\_liverA, NM\_134156, NM\_010391, CL4156.Contig1\_Mf\_liverA, Unigene27248\_Mf\_liverA, CL5254.Contig1\_Mf\_liverA, Unigene24613\_Mf\_liverA, Unigene31852\_Mf\_liverA, Unigene27082\_Mf\_liverA, Unigene37076\_Mf\_liverA, Unigene43107\_Mf\_liverA, Unigene37497\_Mf\_liverA, Unigene35698\_Mf\_liverA, NM\_010380, CL275.Contig5\_Mf\_liverA, Unigene33632\_Mf\_liverA, CL6039.Contig1\_Mf\_liverA, CL2855.Contig1\_Mf\_liverA, Unigene2746\_Mf\_liverA, Unigene16465\_Mf\_liverA, Unigene5169\_Mf\_liverA, Unigene31206\_Mf\_liverA, Unigene10496\_Mf\_liverA, NM\_010392, CL4086.Contig1\_Mf\_liverA, Unigene31251\_Mf\_liverA, CL3822.Contig2\_Mf\_liverA, Unigene40610\_Mf\_liverA, CL186.Contig3\_Mf\_liverA, Unigene15318\_Mf\_liverA, Unigene29823\_Mf\_liverA, CL425.Contig1\_Mf\_liverA, Unigene36190\_Mf\_liverA, NM\_009255, Unigene15982\_Mf\_liverA, Unigene30154\_Mf\_liverA, Unigene37243\_Mf\_liverA, Unigene27026\_Mf\_liverA, Unigene35237\_Mf\_liverA, Unigene112\_Mf\_liverA, Unigene30587\_Mf\_liverA, Unigene39403\_Mf\_liverA, Unigene3752\_Mf\_liverA, CL4600.Contig1\_Mf\_liverA, Unigene591\_Mf\_liverA, Unigene38598\_Mf\_liverA, Unigene12907\_Mf\_liverA, Unigene29231\_Mf\_liverA, Unigene15681\_Mf\_liverA, Unigene5693\_Mf\_liverA, Unigene22052\_Mf\_liverA, Unigene5165\_Mf\_liverA, Unigene30707\_Mf\_liverA, CL3800.Contig1\_Mf\_liverA, Unigene26585\_Mf\_liverA, Unigene36543\_Mf\_liverA, Unigene4938\_Mf\_liverA, Unigene28687\_Mf\_liverA, Unigene36328\_Mf\_liverA, CL3002.Contig1\_Mf\_liverA, Unigene30155\_Mf\_liverA, Unigene6895\_Mf\_liverA, CL1810.Contig1\_Mf\_liverA, Unigene5758\_Mf\_liverA, CL6039.Contig2\_Mf\_liverA, CL4033.Contig1\_Mf\_liverA, Unigene38870\_Mf\_liverA, Unigene38199\_Mf\_liverA, Unigene24547\_Mf\_liverA, Unigene34983\_Mf\_liverA, Unigene28662\_Mf\_liverA, CL887.Contig2\_Mf\_liverA, Unigene4781\_Mf\_liverA, Unigene37139\_Mf\_liverA, CL3519.Contig1\_Mf\_liverA, Unigene29426\_Mf\_liverA, Unigene13462\_Mf\_liverA, CL3207.Contig1\_Mf\_liverA, Unigene5906\_Mf\_liverA, Unigene20371\_Mf\_liverA, Unigene28731\_Mf\_liverA, CL1493.Contig1\_Mf\_liverA, Unigene4597\_Mf\_liverA, Unigene14170\_Mf\_liverA, Unigene30585\_Mf\_liverA, CL5764.Contig1\_Mf\_liverA, Unigene32294\_Mf\_liverA, Unigene4909\_Mf\_liverA, Unigene24471\_Mf\_liverA, Unigene33080\_Mf\_liverA, Unigene18796\_Mf\_liverA, Unigene21337\_Mf\_liverA, Unigene39252\_Mf\_liverA, Unigene30947\_Mf\_liverA, Unigene28873\_Mf\_liverA, Unigene18126\_Mf\_liverA, Unigene8740\_Mf\_liverA, NM\_178405, CL3816.Contig2\_Mf\_liverA, CL1052.Contig1\_Mf\_liverA, Unigene32515\_Mf\_liverA, Unigene35090\_Mf\_liverA, NM\_012030, Unigene39011\_Mf\_liverA, Unigene20432\_Mf\_liverA, Unigene120\_Mf\_liverA, Unigene39749\_Mf\_liverA, Unigene35609\_Mf\_liverA, CL4007.Contig1\_Mf\_liverA, Unigene25052\_Mf\_liverA, CL3835.Contig1\_Mf\_liverA, Unigene13950\_Mf\_liverA, Unigene37245\_Mf\_liverA, CL2355.Contig1\_Mf\_liverA, Unigene20372\_Mf\_liverA, Unigene26580\_Mf\_liverA, Unigene32295\_Mf\_liverA, Unigene21562\_Mf\_liverA, Unigene19658\_Mf\_liverA, Unigene26336\_Mf\_liverA, Unigene4636\_Mf\_liverA, Unigene5745\_Mf\_liverA, Unigene21336\_Mf\_liverA, Unigene38514\_Mf\_liverA, Unigene12889\_Mf\_liverA, Unigene36699\_Mf\_liverA, Unigene30892\_Mf\_liverA, Unigene18340\_Mf\_liverA, Unigene496\_Mf\_liverA, Unigene42803\_Mf\_liverA, NM\_001099634, Unigene14765\_Mf\_liverA, Unigene4983\_Mf\_liverA, CL5698.Contig1\_Mf\_liverA, Unigene4681\_Mf\_liverA, Unigene16891\_Mf\_liverA, Unigene12519\_Mf\_liverA, Unigene37819\_Mf\_liverA, CL591.Contig1\_Mf\_liverA, Unigene14284\_Mf\_liverA, Unigene5886\_Mf\_liverA, CL336.Contig3\_Mf\_liverA |
| plasma membrane | Unigene26055\_Mf\_liverA, Unigene21684\_Mf\_liverA, CL5640.Contig1\_Mf\_liverA, NM\_009898, Unigene25721\_Mf\_liverA, CL3835.Contig2\_Mf\_liverA, Unigene38831\_Mf\_liverA, Unigene22874\_Mf\_liverA, Unigene6959\_Mf\_liverA, Unigene24111\_Mf\_liverA, Unigene28186\_Mf\_liverA, Unigene14603\_Mf\_liverA, Unigene29308\_Mf\_liverA, Unigene24883\_Mf\_liverA, NM\_010378, Unigene29424\_Mf\_liverA, Unigene8473\_Mf\_liverA, Unigene36849\_Mf\_liverA, Unigene26053\_Mf\_liverA, Unigene29008\_Mf\_liverA, Unigene25055\_Mf\_liverA, Unigene27593\_Mf\_liverA, CL2339.Contig1\_Mf\_liverA, Unigene22433\_Mf\_liverA, Unigene29788\_Mf\_liverA, Unigene36762\_Mf\_liverA, Unigene16463\_Mf\_liverA, CL777.Contig8\_Mf\_liverA, Unigene14816\_Mf\_liverA, Unigene37698\_Mf\_liverA, Unigene38689\_Mf\_liverA, Unigene30878\_Mf\_liverA, Unigene1137\_Mf\_liverA, Unigene27294\_Mf\_liverA, NR\_004446, Unigene24344\_Mf\_liverA, CL1555.Contig1\_Mf\_liverA, Unigene18500\_Mf\_liverA, CL5586.Contig1\_Mf\_liverA, Unigene36889\_Mf\_liverA, Unigene32564\_Mf\_liverA, Unigene37535\_Mf\_liverA, Unigene24112\_Mf\_liverA, Unigene15703\_Mf\_liverA, CL2001.Contig1\_Mf\_liverA, Unigene38110\_Mf\_liverA, Unigene35816\_Mf\_liverA, Unigene31623\_Mf\_liverA, Unigene26194\_Mf\_liverA, Unigene31333\_Mf\_liverA, CL593.Contig2\_Mf\_liverA, CL2260.Contig1\_Mf\_liverA, Unigene13593\_Mf\_liverA, Unigene30369\_Mf\_liverA, NM\_010233, Unigene4686\_Mf\_liverA, Unigene4630\_Mf\_liverA, Unigene11\_Mf\_liverA, Unigene34394\_Mf\_liverA, Unigene843\_Mf\_liverA, Unigene5138\_Mf\_liverA, CL1493.Contig2\_Mf\_liverA, CL3575.Contig1\_Mf\_liverA, Unigene12153\_Mf\_liverA, Unigene37150\_Mf\_liverA, Unigene22432\_Mf\_liverA, Unigene25046\_Mf\_liverA, Unigene2745\_Mf\_liverA, Unigene25462\_Mf\_liverA, Unigene802\_Mf\_liverA, Unigene5552\_Mf\_liverA, CL5807.Contig1\_Mf\_liverA, NM\_153795, Unigene13363\_Mf\_liverA, Unigene26065\_Mf\_liverA, CL2855.Contig2\_Mf\_liverA, Unigene31988\_Mf\_liverA, Unigene18125\_Mf\_liverA, CL4293.Contig1\_Mf\_liverA, Unigene13097\_Mf\_liverA, Unigene25226\_Mf\_liverA, Unigene33459\_Mf\_liverA, Unigene23870\_Mf\_liverA, Unigene21561\_Mf\_liverA, Unigene4922\_Mf\_liverA, Unigene13143\_Mf\_liverA, CL1263.Contig1\_Mf\_liverA, Unigene30261\_Mf\_liverA, Unigene34746\_Mf\_liverA, Unigene24503\_Mf\_liverA, Unigene139\_Mf\_liverA, Unigene15583\_Mf\_liverA, Unigene27422\_Mf\_liverA, Unigene33560\_Mf\_liverA, CL3816.Contig1\_Mf\_liverA, Unigene25333\_Mf\_liverA, CL993.Contig2\_Mf\_liverA, CL5631.Contig1\_Mf\_liverA, Unigene37575\_Mf\_liverA, Unigene30584\_Mf\_liverA, Unigene17579\_Mf\_liverA, CL4220.Contig2\_Mf\_liverA, Unigene27081\_Mf\_liverA, Unigene431\_Mf\_liverA, Unigene27547\_Mf\_liverA, NM\_134156, NM\_010391, CL4156.Contig1\_Mf\_liverA, Unigene27248\_Mf\_liverA, CL5254.Contig1\_Mf\_liverA, Unigene24613\_Mf\_liverA, Unigene31852\_Mf\_liverA, Unigene27082\_Mf\_liverA, Unigene37076\_Mf\_liverA, Unigene43107\_Mf\_liverA, Unigene37497\_Mf\_liverA, Unigene35698\_Mf\_liverA, NM\_010380, CL275.Contig5\_Mf\_liverA, Unigene33632\_Mf\_liverA, CL6039.Contig1\_Mf\_liverA, CL2855.Contig1\_Mf\_liverA, Unigene2746\_Mf\_liverA, Unigene16465\_Mf\_liverA, Unigene5169\_Mf\_liverA, Unigene31206\_Mf\_liverA, Unigene10496\_Mf\_liverA, NM\_010392, CL4086.Contig1\_Mf\_liverA, Unigene31251\_Mf\_liverA, CL3822.Contig2\_Mf\_liverA, Unigene40610\_Mf\_liverA, Unigene15318\_Mf\_liverA, Unigene29823\_Mf\_liverA, CL425.Contig1\_Mf\_liverA, Unigene36190\_Mf\_liverA, NM\_009255, Unigene15982\_Mf\_liverA, Unigene30154\_Mf\_liverA, Unigene37243\_Mf\_liverA, Unigene27026\_Mf\_liverA, Unigene35237\_Mf\_liverA, Unigene112\_Mf\_liverA, Unigene30587\_Mf\_liverA, Unigene39403\_Mf\_liverA, Unigene3752\_Mf\_liverA, CL4600.Contig1\_Mf\_liverA, Unigene591\_Mf\_liverA, Unigene38598\_Mf\_liverA, Unigene12907\_Mf\_liverA, Unigene29231\_Mf\_liverA, Unigene15681\_Mf\_liverA, Unigene5693\_Mf\_liverA, Unigene22052\_Mf\_liverA, Unigene5165\_Mf\_liverA, Unigene30707\_Mf\_liverA, CL3800.Contig1\_Mf\_liverA, Unigene26585\_Mf\_liverA, Unigene36543\_Mf\_liverA, Unigene4938\_Mf\_liverA, Unigene28687\_Mf\_liverA, Unigene36328\_Mf\_liverA, CL3002.Contig1\_Mf\_liverA, Unigene30155\_Mf\_liverA, Unigene6895\_Mf\_liverA, CL1810.Contig1\_Mf\_liverA, Unigene5758\_Mf\_liverA, CL6039.Contig2\_Mf\_liverA, CL4033.Contig1\_Mf\_liverA, Unigene38870\_Mf\_liverA, Unigene38199\_Mf\_liverA, Unigene24547\_Mf\_liverA, Unigene34983\_Mf\_liverA, Unigene28662\_Mf\_liverA, CL887.Contig2\_Mf\_liverA, Unigene4781\_Mf\_liverA, Unigene37139\_Mf\_liverA, CL3519.Contig1\_Mf\_liverA, Unigene29426\_Mf\_liverA, Unigene13462\_Mf\_liverA, CL3207.Contig1\_Mf\_liverA, Unigene5906\_Mf\_liverA, Unigene20371\_Mf\_liverA, Unigene28731\_Mf\_liverA, CL1493.Contig1\_Mf\_liverA, Unigene4597\_Mf\_liverA, Unigene14170\_Mf\_liverA, Unigene30585\_Mf\_liverA, CL5764.Contig1\_Mf\_liverA, Unigene32294\_Mf\_liverA, Unigene4909\_Mf\_liverA, Unigene24471\_Mf\_liverA, Unigene33080\_Mf\_liverA, Unigene18796\_Mf\_liverA, Unigene21337\_Mf\_liverA, Unigene39252\_Mf\_liverA, Unigene30947\_Mf\_liverA, Unigene28873\_Mf\_liverA, Unigene18126\_Mf\_liverA, Unigene8740\_Mf\_liverA, NM\_178405, CL3816.Contig2\_Mf\_liverA, CL1052.Contig1\_Mf\_liverA, Unigene35090\_Mf\_liverA, NM\_012030, Unigene39011\_Mf\_liverA, Unigene20432\_Mf\_liverA, Unigene120\_Mf\_liverA, Unigene39749\_Mf\_liverA, Unigene35609\_Mf\_liverA, CL4007.Contig1\_Mf\_liverA, Unigene25052\_Mf\_liverA, CL3835.Contig1\_Mf\_liverA, Unigene13950\_Mf\_liverA, Unigene37245\_Mf\_liverA, CL2355.Contig1\_Mf\_liverA, Unigene20372\_Mf\_liverA, Unigene26580\_Mf\_liverA, Unigene32295\_Mf\_liverA, Unigene21562\_Mf\_liverA, Unigene19658\_Mf\_liverA, Unigene26336\_Mf\_liverA, Unigene4636\_Mf\_liverA, Unigene5745\_Mf\_liverA, Unigene21336\_Mf\_liverA, Unigene38514\_Mf\_liverA, Unigene12889\_Mf\_liverA, Unigene36699\_Mf\_liverA, Unigene30892\_Mf\_liverA, Unigene18340\_Mf\_liverA, Unigene496\_Mf\_liverA, Unigene42803\_Mf\_liverA, NM\_001099634, Unigene14765\_Mf\_liverA, Unigene4983\_Mf\_liverA, CL5698.Contig1\_Mf\_liverA, Unigene4681\_Mf\_liverA, Unigene16891\_Mf\_liverA, Unigene12519\_Mf\_liverA, Unigene37819\_Mf\_liverA, CL591.Contig1\_Mf\_liverA, Unigene14284\_Mf\_liverA, Unigene5886\_Mf\_liverA, CL336.Contig3\_Mf\_liverA |
| external side of plasma membrane | Unigene4909\_Mf\_liverA, Unigene24471\_Mf\_liverA, Unigene5552\_Mf\_liverA, CL4086.Contig1\_Mf\_liverA, Unigene33080\_Mf\_liverA, Unigene40610\_Mf\_liverA, CL3835.Contig2\_Mf\_liverA, Unigene26065\_Mf\_liverA, CL2855.Contig2\_Mf\_liverA, Unigene18125\_Mf\_liverA, Unigene6959\_Mf\_liverA, Unigene24111\_Mf\_liverA, Unigene28186\_Mf\_liverA, Unigene21561\_Mf\_liverA, Unigene18126\_Mf\_liverA, NM\_009255, Unigene13143\_Mf\_liverA, Unigene15982\_Mf\_liverA, Unigene27593\_Mf\_liverA, Unigene22433\_Mf\_liverA, Unigene30587\_Mf\_liverA, Unigene34746\_Mf\_liverA, Unigene24503\_Mf\_liverA, Unigene14816\_Mf\_liverA, Unigene38689\_Mf\_liverA, Unigene39749\_Mf\_liverA, Unigene1137\_Mf\_liverA, CL3835.Contig1\_Mf\_liverA, Unigene30584\_Mf\_liverA, Unigene37245\_Mf\_liverA, Unigene36889\_Mf\_liverA, CL3002.Contig1\_Mf\_liverA, Unigene32564\_Mf\_liverA, Unigene6895\_Mf\_liverA, Unigene24112\_Mf\_liverA, CL4156.Contig1\_Mf\_liverA, Unigene21562\_Mf\_liverA, Unigene19658\_Mf\_liverA, CL887.Contig2\_Mf\_liverA, CL2001.Contig1\_Mf\_liverA, Unigene38110\_Mf\_liverA, Unigene4630\_Mf\_liverA, Unigene42803\_Mf\_liverA, Unigene4983\_Mf\_liverA, Unigene22432\_Mf\_liverA, CL275.Contig5\_Mf\_liverA, CL2855.Contig1\_Mf\_liverA, Unigene30585\_Mf\_liverA |
| extracellular region | Unigene29399\_Mf\_liverA, Unigene38831\_Mf\_liverA, CL3669.Contig2\_Mf\_liverA, Unigene14536\_Mf\_liverA, Unigene28899\_Mf\_liverA, Unigene14603\_Mf\_liverA, Unigene9081\_Mf\_liverA, CL482.Contig1\_Mf\_liverA, CL2339.Contig1\_Mf\_liverA, CL81.Contig1\_Mf\_liverA, Unigene18181\_Mf\_liverA, Unigene36762\_Mf\_liverA, Unigene34852\_Mf\_liverA, Unigene7065\_Mf\_liverA, Unigene37698\_Mf\_liverA, Unigene38689\_Mf\_liverA, Unigene1137\_Mf\_liverA, Unigene34810\_Mf\_liverA, CL44.Contig1\_Mf\_liverA, Unigene27420\_Mf\_liverA, Unigene14594\_Mf\_liverA, Unigene17397\_Mf\_liverA, Unigene37904\_Mf\_liverA, Unigene37535\_Mf\_liverA, Unigene14276\_Mf\_liverA, CL2001.Contig1\_Mf\_liverA, Unigene38110\_Mf\_liverA, Unigene31333\_Mf\_liverA, NM\_010233, Unigene11\_Mf\_liverA, Unigene4630\_Mf\_liverA, NM\_009776, Unigene30003\_Mf\_liverA, CL3575.Contig1\_Mf\_liverA, Unigene14050\_Mf\_liverA, Unigene30002\_Mf\_liverA, Unigene8054\_Mf\_liverA, Unigene5134\_Mf\_liverA, CL787.Contig1\_Mf\_liverA, Unigene9150\_Mf\_liverA, Unigene26380\_Mf\_liverA, Unigene5382\_Mf\_liverA, Unigene33459\_Mf\_liverA, Unigene13143\_Mf\_liverA, Unigene36669\_Mf\_liverA, Unigene30261\_Mf\_liverA, Unigene36698\_Mf\_liverA, Unigene34746\_Mf\_liverA, Unigene14582\_Mf\_liverA, Unigene941\_Mf\_liverA, Unigene13945\_Mf\_liverA, Unigene33560\_Mf\_liverA, CL993.Contig2\_Mf\_liverA, Unigene15145\_Mf\_liverA, Unigene28315\_Mf\_liverA, Unigene30584\_Mf\_liverA, Unigene17579\_Mf\_liverA, CL4040.Contig2\_Mf\_liverA, CL1736.Contig2\_Mf\_liverA, NM\_001081372, Unigene5606\_Mf\_liverA, Unigene425\_Mf\_liverA, Unigene30493\_Mf\_liverA, Unigene13296\_Mf\_liverA, Unigene27248\_Mf\_liverA, Unigene8132\_Mf\_liverA, Unigene30839\_Mf\_liverA, Unigene24157\_Mf\_liverA, NM\_010776, Unigene32791\_Mf\_liverA, CL442.Contig2\_Mf\_liverA, Unigene35698\_Mf\_liverA, CL1347.Contig3\_Mf\_liverA, CL5189.Contig1\_Mf\_liverA, CL6039.Contig1\_Mf\_liverA, CL3339.Contig1\_Mf\_liverA, NM\_177033, CL4086.Contig1\_Mf\_liverA, Unigene4363\_Mf\_liverA, CL1811.Contig2\_Mf\_liverA, Unigene15318\_Mf\_liverA, Unigene24375\_Mf\_liverA, Unigene30815\_Mf\_liverA, NM\_009255, Unigene10351\_Mf\_liverA, Unigene15982\_Mf\_liverA, Unigene35237\_Mf\_liverA, Unigene30587\_Mf\_liverA, Unigene36650\_Mf\_liverA, Unigene3752\_Mf\_liverA, CL1372.Contig2\_Mf\_liverA, Unigene591\_Mf\_liverA, CL4040.Contig1\_Mf\_liverA, Unigene29231\_Mf\_liverA, CL3030.Contig2\_Mf\_liverA, Unigene22052\_Mf\_liverA, Unigene13894\_Mf\_liverA, Unigene36328\_Mf\_liverA, CL6039.Contig2\_Mf\_liverA, Unigene38870\_Mf\_liverA, Unigene20512\_Mf\_liverA, Unigene28662\_Mf\_liverA, Unigene34983\_Mf\_liverA, Unigene8033\_Mf\_liverA, Unigene29426\_Mf\_liverA, CL1811.Contig1\_Mf\_liverA, Unigene20371\_Mf\_liverA, Unigene393\_Mf\_liverA, Unigene36987\_Mf\_liverA, Unigene14810\_Mf\_liverA, Unigene1212\_Mf\_liverA, Unigene30585\_Mf\_liverA, CL1372.Contig1\_Mf\_liverA, Unigene32110\_Mf\_liverA, Unigene13106\_Mf\_liverA, Unigene33080\_Mf\_liverA, Unigene15529\_Mf\_liverA, Unigene33832\_Mf\_liverA, Unigene13616\_Mf\_liverA, Unigene21337\_Mf\_liverA, Unigene39252\_Mf\_liverA, CL5293.Contig2\_Mf\_liverA, CL5978.Contig2\_Mf\_liverA, CL2251.Contig1\_Mf\_liverA, CL5293.Contig1\_Mf\_liverA, Unigene26381\_Mf\_liverA, Unigene25595\_Mf\_liverA, CL442.Contig5\_Mf\_liverA, CL5459.Contig2\_Mf\_liverA, Unigene35090\_Mf\_liverA, Unigene13950\_Mf\_liverA, CL2333.Contig1\_Mf\_liverA, Unigene37063\_Mf\_liverA, Unigene20372\_Mf\_liverA, CL4790.Contig2\_Mf\_liverA, Unigene14809\_Mf\_liverA, Unigene677\_Mf\_liverA, Unigene15592\_Mf\_liverA, Unigene21336\_Mf\_liverA, Unigene2195\_Mf\_liverA, Unigene30814\_Mf\_liverA, Unigene36673\_Mf\_liverA, Unigene42803\_Mf\_liverA, Unigene18340\_Mf\_liverA, Unigene36439\_Mf\_liverA, Unigene30786\_Mf\_liverA, Unigene36426\_Mf\_liverA, Unigene37559\_Mf\_liverA |
| extracellular space | Unigene29399\_Mf\_liverA, CL4086.Contig1\_Mf\_liverA, CL1811.Contig2\_Mf\_liverA, CL3669.Contig2\_Mf\_liverA, Unigene14536\_Mf\_liverA, Unigene15318\_Mf\_liverA, Unigene28899\_Mf\_liverA, Unigene14603\_Mf\_liverA, Unigene9081\_Mf\_liverA, Unigene30815\_Mf\_liverA, Unigene10351\_Mf\_liverA, Unigene15982\_Mf\_liverA, CL2339.Contig1\_Mf\_liverA, CL81.Contig1\_Mf\_liverA, Unigene35237\_Mf\_liverA, CL1372.Contig2\_Mf\_liverA, CL4040.Contig1\_Mf\_liverA, Unigene7065\_Mf\_liverA, Unigene29231\_Mf\_liverA, Unigene37698\_Mf\_liverA, Unigene38689\_Mf\_liverA, Unigene1137\_Mf\_liverA, CL44.Contig1\_Mf\_liverA, Unigene14594\_Mf\_liverA, Unigene13894\_Mf\_liverA, Unigene37904\_Mf\_liverA, Unigene37535\_Mf\_liverA, Unigene38870\_Mf\_liverA, Unigene34983\_Mf\_liverA, Unigene28662\_Mf\_liverA, Unigene38110\_Mf\_liverA, Unigene31333\_Mf\_liverA, NM\_010233, Unigene4630\_Mf\_liverA, Unigene29426\_Mf\_liverA, CL3575.Contig1\_Mf\_liverA, CL1811.Contig1\_Mf\_liverA, Unigene1212\_Mf\_liverA, Unigene14050\_Mf\_liverA, Unigene32110\_Mf\_liverA, CL1372.Contig1\_Mf\_liverA, CL787.Contig1\_Mf\_liverA, Unigene13106\_Mf\_liverA, Unigene33080\_Mf\_liverA, Unigene15529\_Mf\_liverA, Unigene33832\_Mf\_liverA, Unigene5382\_Mf\_liverA, Unigene13616\_Mf\_liverA, Unigene21337\_Mf\_liverA, Unigene13143\_Mf\_liverA, Unigene36698\_Mf\_liverA, Unigene25595\_Mf\_liverA, Unigene14582\_Mf\_liverA, Unigene34746\_Mf\_liverA, CL5459.Contig2\_Mf\_liverA, Unigene941\_Mf\_liverA, Unigene13950\_Mf\_liverA, Unigene17579\_Mf\_liverA, CL4040.Contig2\_Mf\_liverA, CL2333.Contig1\_Mf\_liverA, Unigene425\_Mf\_liverA, Unigene37063\_Mf\_liverA, Unigene30493\_Mf\_liverA, Unigene677\_Mf\_liverA, Unigene15592\_Mf\_liverA, Unigene27248\_Mf\_liverA, Unigene13296\_Mf\_liverA, Unigene8132\_Mf\_liverA, Unigene21336\_Mf\_liverA, Unigene2195\_Mf\_liverA, Unigene30814\_Mf\_liverA, Unigene24157\_Mf\_liverA, Unigene42803\_Mf\_liverA, Unigene18340\_Mf\_liverA, CL1347.Contig3\_Mf\_liverA, Unigene36439\_Mf\_liverA, Unigene30786\_Mf\_liverA, Unigene36426\_Mf\_liverA |
| cell surface | NM\_010392, Unigene29399\_Mf\_liverA, CL4086.Contig1\_Mf\_liverA, Unigene40610\_Mf\_liverA, CL3835.Contig2\_Mf\_liverA, Unigene6959\_Mf\_liverA, Unigene24111\_Mf\_liverA, Unigene28186\_Mf\_liverA, NM\_010378, NM\_009255, Unigene29424\_Mf\_liverA, Unigene15982\_Mf\_liverA, Unigene27593\_Mf\_liverA, Unigene27026\_Mf\_liverA, Unigene22433\_Mf\_liverA, Unigene29788\_Mf\_liverA, Unigene30587\_Mf\_liverA, Unigene29231\_Mf\_liverA, Unigene14816\_Mf\_liverA, Unigene38689\_Mf\_liverA, Unigene1137\_Mf\_liverA, NR\_004446, Unigene36889\_Mf\_liverA, CL3002.Contig1\_Mf\_liverA, Unigene32564\_Mf\_liverA, Unigene37904\_Mf\_liverA, Unigene6895\_Mf\_liverA, Unigene24112\_Mf\_liverA, Unigene38870\_Mf\_liverA, CL887.Contig2\_Mf\_liverA, CL2001.Contig1\_Mf\_liverA, Unigene38110\_Mf\_liverA, Unigene31333\_Mf\_liverA, CL3519.Contig1\_Mf\_liverA, Unigene4630\_Mf\_liverA, Unigene11\_Mf\_liverA, Unigene29426\_Mf\_liverA, Unigene13462\_Mf\_liverA, Unigene20371\_Mf\_liverA, Unigene37150\_Mf\_liverA, Unigene22432\_Mf\_liverA, Unigene30585\_Mf\_liverA, Unigene4909\_Mf\_liverA, Unigene5552\_Mf\_liverA, Unigene24471\_Mf\_liverA, Unigene33080\_Mf\_liverA, Unigene26065\_Mf\_liverA, CL2855.Contig2\_Mf\_liverA, Unigene18125\_Mf\_liverA, Unigene39252\_Mf\_liverA, Unigene21561\_Mf\_liverA, Unigene18126\_Mf\_liverA, Unigene13143\_Mf\_liverA, Unigene36698\_Mf\_liverA, Unigene14582\_Mf\_liverA, Unigene34746\_Mf\_liverA, Unigene24503\_Mf\_liverA, Unigene39749\_Mf\_liverA, CL3835.Contig1\_Mf\_liverA, Unigene30584\_Mf\_liverA, Unigene35046\_Mf\_liverA, Unigene37245\_Mf\_liverA, NM\_010391, Unigene26580\_Mf\_liverA, CL4156.Contig1\_Mf\_liverA, Unigene27248\_Mf\_liverA, Unigene19658\_Mf\_liverA, Unigene21562\_Mf\_liverA, Unigene42803\_Mf\_liverA, Unigene4983\_Mf\_liverA, NM\_010380, CL275.Contig5\_Mf\_liverA, Unigene14284\_Mf\_liverA, CL2855.Contig1\_Mf\_liverA |
| extracellular region part | Unigene29399\_Mf\_liverA, NM\_177033, CL4086.Contig1\_Mf\_liverA, CL1811.Contig2\_Mf\_liverA, CL3669.Contig2\_Mf\_liverA, Unigene14536\_Mf\_liverA, Unigene15318\_Mf\_liverA, Unigene28899\_Mf\_liverA, Unigene14603\_Mf\_liverA, Unigene9081\_Mf\_liverA, Unigene30815\_Mf\_liverA, Unigene10351\_Mf\_liverA, NM\_009255, Unigene15982\_Mf\_liverA, CL482.Contig1\_Mf\_liverA, CL2339.Contig1\_Mf\_liverA, CL81.Contig1\_Mf\_liverA, Unigene35237\_Mf\_liverA, Unigene18181\_Mf\_liverA, Unigene30587\_Mf\_liverA, CL1372.Contig2\_Mf\_liverA, CL4040.Contig1\_Mf\_liverA, Unigene7065\_Mf\_liverA, Unigene29231\_Mf\_liverA, Unigene37698\_Mf\_liverA, Unigene38689\_Mf\_liverA, Unigene22052\_Mf\_liverA, Unigene1137\_Mf\_liverA, Unigene34810\_Mf\_liverA, CL44.Contig1\_Mf\_liverA, Unigene14594\_Mf\_liverA, Unigene13894\_Mf\_liverA, Unigene36328\_Mf\_liverA, Unigene17397\_Mf\_liverA, Unigene37904\_Mf\_liverA, Unigene37535\_Mf\_liverA, CL6039.Contig2\_Mf\_liverA, Unigene20512\_Mf\_liverA, Unigene38870\_Mf\_liverA, Unigene34983\_Mf\_liverA, Unigene28662\_Mf\_liverA, CL2001.Contig1\_Mf\_liverA, Unigene38110\_Mf\_liverA, Unigene31333\_Mf\_liverA, Unigene8033\_Mf\_liverA, NM\_010233, Unigene4630\_Mf\_liverA, Unigene29426\_Mf\_liverA, NM\_009776, CL3575.Contig1\_Mf\_liverA, CL1811.Contig1\_Mf\_liverA, Unigene1212\_Mf\_liverA, Unigene14050\_Mf\_liverA, Unigene30585\_Mf\_liverA, Unigene8054\_Mf\_liverA, Unigene32110\_Mf\_liverA, CL1372.Contig1\_Mf\_liverA, CL787.Contig1\_Mf\_liverA, Unigene13106\_Mf\_liverA, Unigene33080\_Mf\_liverA, Unigene15529\_Mf\_liverA, Unigene33832\_Mf\_liverA, Unigene5382\_Mf\_liverA, Unigene13616\_Mf\_liverA, Unigene21337\_Mf\_liverA, Unigene39252\_Mf\_liverA, Unigene33459\_Mf\_liverA, CL2251.Contig1\_Mf\_liverA, CL5978.Contig2\_Mf\_liverA, Unigene13143\_Mf\_liverA, Unigene25595\_Mf\_liverA, Unigene30261\_Mf\_liverA, Unigene36698\_Mf\_liverA, CL442.Contig5\_Mf\_liverA, Unigene14582\_Mf\_liverA, Unigene34746\_Mf\_liverA, Unigene941\_Mf\_liverA, CL5459.Contig2\_Mf\_liverA, Unigene35090\_Mf\_liverA, CL993.Contig2\_Mf\_liverA, Unigene13950\_Mf\_liverA, Unigene30584\_Mf\_liverA, Unigene17579\_Mf\_liverA, CL4040.Contig2\_Mf\_liverA, NM\_001081372, Unigene425\_Mf\_liverA, Unigene5606\_Mf\_liverA, CL2333.Contig1\_Mf\_liverA, Unigene37063\_Mf\_liverA, Unigene30493\_Mf\_liverA, Unigene677\_Mf\_liverA, Unigene15592\_Mf\_liverA, Unigene27248\_Mf\_liverA, Unigene13296\_Mf\_liverA, Unigene8132\_Mf\_liverA, Unigene21336\_Mf\_liverA, Unigene2195\_Mf\_liverA, Unigene30814\_Mf\_liverA, Unigene24157\_Mf\_liverA, Unigene18340\_Mf\_liverA, Unigene42803\_Mf\_liverA, NM\_010776, Unigene32791\_Mf\_liverA, CL442.Contig2\_Mf\_liverA, CL6039.Contig1\_Mf\_liverA, CL1347.Contig3\_Mf\_liverA, Unigene30786\_Mf\_liverA, Unigene36439\_Mf\_liverA, Unigene36426\_Mf\_liverA |
| integral to membrane | Unigene26055\_Mf\_liverA, Unigene21684\_Mf\_liverA, CL3835.Contig2\_Mf\_liverA, Unigene22874\_Mf\_liverA, Unigene32891\_Mf\_liverA, Unigene6959\_Mf\_liverA, Unigene7682\_Mf\_liverA, Unigene24111\_Mf\_liverA, Unigene28186\_Mf\_liverA, Unigene24883\_Mf\_liverA, NM\_011082, Unigene36849\_Mf\_liverA, Unigene26053\_Mf\_liverA, Unigene25055\_Mf\_liverA, Unigene27593\_Mf\_liverA, CL2339.Contig1\_Mf\_liverA, Unigene22433\_Mf\_liverA, Unigene29788\_Mf\_liverA, Unigene36762\_Mf\_liverA, Unigene34852\_Mf\_liverA, CL3847.Contig1\_Mf\_liverA, Unigene14816\_Mf\_liverA, Unigene39886\_Mf\_liverA, Unigene37698\_Mf\_liverA, Unigene30878\_Mf\_liverA, Unigene27294\_Mf\_liverA, Unigene24344\_Mf\_liverA, Unigene37880\_Mf\_liverA, CL1555.Contig1\_Mf\_liverA, Unigene18500\_Mf\_liverA, CL5586.Contig1\_Mf\_liverA, Unigene36889\_Mf\_liverA, Unigene32564\_Mf\_liverA, Unigene24112\_Mf\_liverA, CL695.Contig1\_Mf\_liverA, Unigene15703\_Mf\_liverA, Unigene25426\_Mf\_liverA, CL2001.Contig1\_Mf\_liverA, Unigene31333\_Mf\_liverA, CL593.Contig2\_Mf\_liverA, CL2260.Contig1\_Mf\_liverA, Unigene13593\_Mf\_liverA, Unigene30369\_Mf\_liverA, Unigene4686\_Mf\_liverA, Unigene34394\_Mf\_liverA, Unigene31694\_Mf\_liverA, Unigene843\_Mf\_liverA, Unigene37150\_Mf\_liverA, Unigene22432\_Mf\_liverA, Unigene34143\_Mf\_liverA, Unigene5294\_Mf\_liverA, Unigene8054\_Mf\_liverA, Unigene25462\_Mf\_liverA, CL787.Contig1\_Mf\_liverA, Unigene17048\_Mf\_liverA, Unigene5552\_Mf\_liverA, CL5807.Contig1\_Mf\_liverA, Unigene31958\_Mf\_liverA, Unigene13363\_Mf\_liverA, Unigene26065\_Mf\_liverA, Unigene13230\_Mf\_liverA, CL2855.Contig2\_Mf\_liverA, Unigene18125\_Mf\_liverA, CL4293.Contig1\_Mf\_liverA, Unigene25226\_Mf\_liverA, Unigene5366\_Mf\_liverA, Unigene4922\_Mf\_liverA, Unigene5632\_Mf\_liverA, NM\_153505, Unigene941\_Mf\_liverA, CL2142.Contig2\_Mf\_liverA, Unigene24503\_Mf\_liverA, Unigene15583\_Mf\_liverA, CL3816.Contig1\_Mf\_liverA, Unigene37420\_Mf\_liverA, NM\_153589, CL529.Contig2\_Mf\_liverA, Unigene37575\_Mf\_liverA, CL4220.Contig2\_Mf\_liverA, Unigene25398\_Mf\_liverA, Unigene27081\_Mf\_liverA, Unigene27547\_Mf\_liverA, CL5316.Contig1\_Mf\_liverA, Unigene37389\_Mf\_liverA, Unigene32332\_Mf\_liverA, Unigene14070\_Mf\_liverA, Unigene860\_Mf\_liverA, Unigene29960\_Mf\_liverA, Unigene27248\_Mf\_liverA, Unigene27082\_Mf\_liverA, Unigene5712\_Mf\_liverA, Unigene37497\_Mf\_liverA, Unigene35698\_Mf\_liverA, CL275.Contig5\_Mf\_liverA, Unigene33632\_Mf\_liverA, Unigene15184\_Mf\_liverA, CL2855.Contig1\_Mf\_liverA, Unigene33760\_Mf\_liverA, Unigene5169\_Mf\_liverA, Unigene40796\_Mf\_liverA, Unigene72\_Mf\_liverA, Unigene13233\_Mf\_liverA, Unigene4363\_Mf\_liverA, Unigene40610\_Mf\_liverA, Unigene20167\_Mf\_liverA, Unigene15318\_Mf\_liverA, Unigene29823\_Mf\_liverA, CL425.Contig1\_Mf\_liverA, Unigene32421\_Mf\_liverA, Unigene7970\_Mf\_liverA, CL2797.Contig2\_Mf\_liverA, Unigene15982\_Mf\_liverA, Unigene27026\_Mf\_liverA, Unigene112\_Mf\_liverA, Unigene39403\_Mf\_liverA, Unigene3752\_Mf\_liverA, CL4600.Contig1\_Mf\_liverA, Unigene38598\_Mf\_liverA, Unigene29231\_Mf\_liverA, CL5796.Contig2\_Mf\_liverA, Unigene26585\_Mf\_liverA, Unigene36543\_Mf\_liverA, CL1988.Contig1\_Mf\_liverA, Unigene4938\_Mf\_liverA, Unigene5292\_Mf\_liverA, NM\_010141, CL3002.Contig1\_Mf\_liverA, CL1988.Contig3\_Mf\_liverA, Unigene6895\_Mf\_liverA, Unigene5758\_Mf\_liverA, CL4033.Contig1\_Mf\_liverA, Unigene38870\_Mf\_liverA, Unigene12843\_Mf\_liverA, Unigene38199\_Mf\_liverA, CL887.Contig2\_Mf\_liverA, Unigene4781\_Mf\_liverA, Unigene37139\_Mf\_liverA, Unigene25994\_Mf\_liverA, CL1988.Contig2\_Mf\_liverA, Unigene29426\_Mf\_liverA, Unigene32695\_Mf\_liverA, Unigene13462\_Mf\_liverA, Unigene5906\_Mf\_liverA, Unigene20371\_Mf\_liverA, Unigene28731\_Mf\_liverA, NM\_033444, Unigene4597\_Mf\_liverA, Unigene28499\_Mf\_liverA, CL5764.Contig1\_Mf\_liverA, Unigene10135\_Mf\_liverA, Unigene4909\_Mf\_liverA, Unigene24471\_Mf\_liverA, Unigene33080\_Mf\_liverA, Unigene7683\_Mf\_liverA, CL4220.Contig1\_Mf\_liverA, Unigene23255\_Mf\_liverA, Unigene21337\_Mf\_liverA, Unigene39252\_Mf\_liverA, Unigene30947\_Mf\_liverA, CL5307.Contig1\_Mf\_liverA, Unigene3377\_Mf\_liverA, Unigene28873\_Mf\_liverA, Unigene18126\_Mf\_liverA, Unigene8740\_Mf\_liverA, NM\_178405, CL738.Contig2\_Mf\_liverA, CL3816.Contig2\_Mf\_liverA, Unigene14270\_Mf\_liverA, CL4490.Contig2\_Mf\_liverA, Unigene35090\_Mf\_liverA, Unigene9406\_Mf\_liverA, CL5062.Contig2\_Mf\_liverA, Unigene39011\_Mf\_liverA, Unigene39749\_Mf\_liverA, Unigene35609\_Mf\_liverA, CL4007.Contig1\_Mf\_liverA, Unigene25052\_Mf\_liverA, CL3835.Contig1\_Mf\_liverA, Unigene7048\_Mf\_liverA, Unigene23185\_Mf\_liverA, Unigene13950\_Mf\_liverA, Unigene35046\_Mf\_liverA, Unigene37245\_Mf\_liverA, Unigene20372\_Mf\_liverA, CL3104.Contig1\_Mf\_liverA, Unigene26580\_Mf\_liverA, Unigene677\_Mf\_liverA, Unigene36593\_Mf\_liverA, Unigene19658\_Mf\_liverA, Unigene26336\_Mf\_liverA, Unigene5745\_Mf\_liverA, Unigene21336\_Mf\_liverA, Unigene36699\_Mf\_liverA, Unigene35417\_Mf\_liverA, Unigene18340\_Mf\_liverA, Unigene33720\_Mf\_liverA, Unigene34866\_Mf\_liverA, Unigene14765\_Mf\_liverA, Unigene4983\_Mf\_liverA, CL5698.Contig1\_Mf\_liverA, Unigene4681\_Mf\_liverA, Unigene7387\_Mf\_liverA, Unigene979\_Mf\_liverA, Unigene31080\_Mf\_liverA, Unigene37819\_Mf\_liverA, Unigene14284\_Mf\_liverA, Unigene5886\_Mf\_liverA |
| plasma membrane part | NM\_009898, CL3835.Contig2\_Mf\_liverA, Unigene22874\_Mf\_liverA, Unigene6959\_Mf\_liverA, Unigene24111\_Mf\_liverA, Unigene28186\_Mf\_liverA, Unigene24883\_Mf\_liverA, NM\_010378, Unigene29424\_Mf\_liverA, Unigene36849\_Mf\_liverA, Unigene29008\_Mf\_liverA, Unigene25055\_Mf\_liverA, Unigene27593\_Mf\_liverA, CL2339.Contig1\_Mf\_liverA, Unigene22433\_Mf\_liverA, Unigene29788\_Mf\_liverA, Unigene36762\_Mf\_liverA, Unigene14816\_Mf\_liverA, Unigene38689\_Mf\_liverA, Unigene30878\_Mf\_liverA, Unigene1137\_Mf\_liverA, NR\_004446, Unigene24344\_Mf\_liverA, Unigene18500\_Mf\_liverA, CL5586.Contig1\_Mf\_liverA, Unigene36889\_Mf\_liverA, Unigene32564\_Mf\_liverA, Unigene24112\_Mf\_liverA, Unigene15703\_Mf\_liverA, CL2001.Contig1\_Mf\_liverA, Unigene38110\_Mf\_liverA, Unigene35816\_Mf\_liverA, CL2260.Contig1\_Mf\_liverA, Unigene13593\_Mf\_liverA, Unigene30369\_Mf\_liverA, NM\_010233, Unigene4686\_Mf\_liverA, Unigene4630\_Mf\_liverA, Unigene843\_Mf\_liverA, Unigene5138\_Mf\_liverA, CL3575.Contig1\_Mf\_liverA, Unigene22432\_Mf\_liverA, Unigene5552\_Mf\_liverA, NM\_153795, Unigene13363\_Mf\_liverA, Unigene26065\_Mf\_liverA, CL2855.Contig2\_Mf\_liverA, Unigene18125\_Mf\_liverA, CL4293.Contig1\_Mf\_liverA, Unigene25226\_Mf\_liverA, Unigene23870\_Mf\_liverA, Unigene21561\_Mf\_liverA, Unigene13143\_Mf\_liverA, Unigene34746\_Mf\_liverA, Unigene24503\_Mf\_liverA, Unigene139\_Mf\_liverA, Unigene15583\_Mf\_liverA, CL5631.Contig1\_Mf\_liverA, Unigene37575\_Mf\_liverA, Unigene30584\_Mf\_liverA, CL4220.Contig2\_Mf\_liverA, Unigene27081\_Mf\_liverA, Unigene431\_Mf\_liverA, NM\_134156, NM\_010391, CL4156.Contig1\_Mf\_liverA, Unigene31852\_Mf\_liverA, Unigene27082\_Mf\_liverA, Unigene37497\_Mf\_liverA, Unigene35698\_Mf\_liverA, NM\_010380, CL275.Contig5\_Mf\_liverA, Unigene33632\_Mf\_liverA, CL2855.Contig1\_Mf\_liverA, Unigene5169\_Mf\_liverA, NM\_010392, CL4086.Contig1\_Mf\_liverA, Unigene40610\_Mf\_liverA, CL425.Contig1\_Mf\_liverA, NM\_009255, Unigene15982\_Mf\_liverA, Unigene37243\_Mf\_liverA, Unigene35237\_Mf\_liverA, Unigene112\_Mf\_liverA, Unigene30587\_Mf\_liverA, CL4600.Contig1\_Mf\_liverA, Unigene591\_Mf\_liverA, Unigene29231\_Mf\_liverA, Unigene5693\_Mf\_liverA, Unigene30707\_Mf\_liverA, Unigene26585\_Mf\_liverA, Unigene36543\_Mf\_liverA, Unigene4938\_Mf\_liverA, Unigene36328\_Mf\_liverA, CL3002.Contig1\_Mf\_liverA, Unigene6895\_Mf\_liverA, Unigene5758\_Mf\_liverA, CL1810.Contig1\_Mf\_liverA, CL4033.Contig1\_Mf\_liverA, Unigene38870\_Mf\_liverA, Unigene38199\_Mf\_liverA, CL887.Contig2\_Mf\_liverA, Unigene4781\_Mf\_liverA, Unigene37139\_Mf\_liverA, CL3519.Contig1\_Mf\_liverA, Unigene29426\_Mf\_liverA, Unigene13462\_Mf\_liverA, Unigene5906\_Mf\_liverA, CL3207.Contig1\_Mf\_liverA, Unigene20371\_Mf\_liverA, Unigene4597\_Mf\_liverA, Unigene30585\_Mf\_liverA, Unigene4909\_Mf\_liverA, Unigene24471\_Mf\_liverA, Unigene33080\_Mf\_liverA, Unigene21337\_Mf\_liverA, Unigene30947\_Mf\_liverA, Unigene39252\_Mf\_liverA, Unigene28873\_Mf\_liverA, Unigene18126\_Mf\_liverA, Unigene8740\_Mf\_liverA, NM\_178405, CL1052.Contig1\_Mf\_liverA, Unigene35090\_Mf\_liverA, NM\_012030, Unigene39011\_Mf\_liverA, Unigene35609\_Mf\_liverA, Unigene39749\_Mf\_liverA, CL4007.Contig1\_Mf\_liverA, CL3835.Contig1\_Mf\_liverA, Unigene25052\_Mf\_liverA, Unigene13950\_Mf\_liverA, Unigene37245\_Mf\_liverA, Unigene20372\_Mf\_liverA, Unigene26580\_Mf\_liverA, Unigene19658\_Mf\_liverA, Unigene21562\_Mf\_liverA, Unigene26336\_Mf\_liverA, Unigene4636\_Mf\_liverA, Unigene21336\_Mf\_liverA, Unigene12889\_Mf\_liverA, Unigene30892\_Mf\_liverA, Unigene42803\_Mf\_liverA, NM\_001099634, Unigene18340\_Mf\_liverA, Unigene14765\_Mf\_liverA, Unigene4983\_Mf\_liverA, Unigene4681\_Mf\_liverA, CL591.Contig1\_Mf\_liverA, Unigene14284\_Mf\_liverA, CL336.Contig3\_Mf\_liverA |
| MHC protein complex | NM\_010392, Unigene37245\_Mf\_liverA, Unigene36543\_Mf\_liverA, Unigene4938\_Mf\_liverA, Unigene24471\_Mf\_liverA, CL3002.Contig1\_Mf\_liverA, CL3835.Contig2\_Mf\_liverA, CL2855.Contig2\_Mf\_liverA, NM\_010391, NM\_010378, Unigene36849\_Mf\_liverA, Unigene13143\_Mf\_liverA, Unigene4681\_Mf\_liverA, NM\_010380, CL275.Contig5\_Mf\_liverA, CL2855.Contig1\_Mf\_liverA, Unigene30878\_Mf\_liverA, CL3835.Contig1\_Mf\_liverA, NR\_004446 |
| cytoplasm | Unigene21684\_Mf\_liverA, Unigene5287\_Mf\_liverA, NM\_144940, Unigene34609\_Mf\_liverA, NM\_009898, Unigene25721\_Mf\_liverA, CL3669.Contig2\_Mf\_liverA, Unigene28186\_Mf\_liverA, NM\_010378, Unigene34727\_Mf\_liverA, NM\_011082, Unigene29424\_Mf\_liverA, CL4160.Contig2\_Mf\_liverA, CL854.Contig1\_Mf\_liverA, Unigene26053\_Mf\_liverA, CL4105.Contig1\_Mf\_liverA, Unigene11007\_Mf\_liverA, Unigene16463\_Mf\_liverA, Unigene785\_Mf\_liverA, Unigene39886\_Mf\_liverA, Unigene15064\_Mf\_liverA, NM\_021273, Unigene27339\_Mf\_liverA, Unigene24252\_Mf\_liverA, CL4757.Contig1\_Mf\_liverA, NR\_004446, CL5586.Contig1\_Mf\_liverA, Unigene34341\_Mf\_liverA, Unigene37535\_Mf\_liverA, Unigene30142\_Mf\_liverA, CL695.Contig1\_Mf\_liverA, NM\_011921, Unigene35816\_Mf\_liverA, Unigene31623\_Mf\_liverA, NM\_010233, Unigene13379\_Mf\_liverA, NM\_009776, Unigene34394\_Mf\_liverA, Unigene30003\_Mf\_liverA, NM\_031165, Unigene25594\_Mf\_liverA, NM\_153193, Unigene5138\_Mf\_liverA, Unigene14508\_Mf\_liverA, Unigene14050\_Mf\_liverA, Unigene30002\_Mf\_liverA, Unigene8054\_Mf\_liverA, Unigene18430\_Mf\_liverA, Unigene802\_Mf\_liverA, Unigene9150\_Mf\_liverA, Unigene5639\_Mf\_liverA, Unigene17048\_Mf\_liverA, CL5807.Contig1\_Mf\_liverA, Unigene33522\_Mf\_liverA, Unigene13363\_Mf\_liverA, Unigene36417\_Mf\_liverA, NM\_021278, CL2855.Contig2\_Mf\_liverA, NM\_177093, CL4293.Contig1\_Mf\_liverA, Unigene25226\_Mf\_liverA, Unigene33512\_Mf\_liverA, Unigene21561\_Mf\_liverA, Unigene39655\_Mf\_liverA, Unigene4922\_Mf\_liverA, Unigene25976\_Mf\_liverA, NM\_153505, Unigene30261\_Mf\_liverA, NM\_145942, Unigene36698\_Mf\_liverA, Unigene13945\_Mf\_liverA, Unigene139\_Mf\_liverA, Unigene4944\_Mf\_liverA, Unigene37575\_Mf\_liverA, Unigene5774\_Mf\_liverA, Unigene25398\_Mf\_liverA, Unigene28217\_Mf\_liverA, Unigene27547\_Mf\_liverA, CL1736.Contig2\_Mf\_liverA, Unigene36836\_Mf\_liverA, Unigene33366\_Mf\_liverA, NM\_134156, CL5316.Contig1\_Mf\_liverA, Unigene37389\_Mf\_liverA, Unigene4720\_Mf\_liverA, Unigene45530\_Mf\_liverA, Unigene32332\_Mf\_liverA, Unigene37470\_Mf\_liverA, Unigene27248\_Mf\_liverA, CL5254.Contig1\_Mf\_liverA, CL2266.Contig2\_Mf\_liverA, Unigene30839\_Mf\_liverA, Unigene34010\_Mf\_liverA, Unigene24613\_Mf\_liverA, Unigene152\_Mf\_liverA, Unigene31852\_Mf\_liverA, CL532.Contig1\_Mf\_liverA, Unigene35169\_Mf\_liverA, Unigene37076\_Mf\_liverA, Unigene1280\_Mf\_liverA, Unigene30288\_Mf\_liverA, CL3166.Contig4\_Mf\_liverA, Unigene14171\_Mf\_liverA, Unigene35698\_Mf\_liverA, Unigene33632\_Mf\_liverA, CL5189.Contig1\_Mf\_liverA, CL3339.Contig1\_Mf\_liverA, Unigene2746\_Mf\_liverA, Unigene30528\_Mf\_liverA, Unigene16465\_Mf\_liverA, Unigene31251\_Mf\_liverA, Unigene4363\_Mf\_liverA, CL4925.Contig1\_Mf\_liverA, CL186.Contig3\_Mf\_liverA, CL425.Contig1\_Mf\_liverA, CL2797.Contig2\_Mf\_liverA, CL1803.Contig1\_Mf\_liverA, CL4736.Contig1\_Mf\_liverA, Unigene14916\_Mf\_liverA, Unigene37243\_Mf\_liverA, Unigene30587\_Mf\_liverA, Unigene15077\_Mf\_liverA, CL4600.Contig1\_Mf\_liverA, Unigene36034\_Mf\_liverA, Unigene5693\_Mf\_liverA, Unigene22052\_Mf\_liverA, Unigene39970\_Mf\_liverA, CL3393.Contig1\_Mf\_liverA, Unigene34867\_Mf\_liverA, Unigene29876\_Mf\_liverA, CL3800.Contig1\_Mf\_liverA, CL2478.Contig3\_Mf\_liverA, Unigene28687\_Mf\_liverA, Unigene5815\_Mf\_liverA, CL1988.Contig3\_Mf\_liverA, NM\_028785, NM\_009609, CL1810.Contig1\_Mf\_liverA, Unigene38870\_Mf\_liverA, CL4033.Contig1\_Mf\_liverA, Unigene24547\_Mf\_liverA, Unigene34983\_Mf\_liverA, Unigene28662\_Mf\_liverA, CL2439.Contig1\_Mf\_liverA, Unigene4781\_Mf\_liverA, NR\_003623, CL3519.Contig1\_Mf\_liverA, Unigene8033\_Mf\_liverA, CL1988.Contig2\_Mf\_liverA, Unigene20371\_Mf\_liverA, Unigene13683\_Mf\_liverA, CL1493.Contig1\_Mf\_liverA, Unigene9698\_Mf\_liverA, Unigene28499\_Mf\_liverA, Unigene36420\_Mf\_liverA, Unigene30585\_Mf\_liverA, Unigene542\_Mf\_liverA, Unigene35476\_Mf\_liverA, Unigene24758\_Mf\_liverA, Unigene4909\_Mf\_liverA, Unigene24471\_Mf\_liverA, Unigene31571\_Mf\_liverA, Unigene33080\_Mf\_liverA, NM\_010162, Unigene15529\_Mf\_liverA, CL4220.Contig1\_Mf\_liverA, Unigene37262\_Mf\_liverA, NM\_019879, NR\_033215, CL5293.Contig2\_Mf\_liverA, CL2251.Contig1\_Mf\_liverA, CL5293.Contig1\_Mf\_liverA, CL4490.Contig2\_Mf\_liverA, Unigene25595\_Mf\_liverA, Unigene14286\_Mf\_liverA, Unigene32515\_Mf\_liverA, CL4816.Contig3\_Mf\_liverA, CL840.Contig1\_Mf\_liverA, Unigene39011\_Mf\_liverA, Unigene20432\_Mf\_liverA, NM\_009022, Unigene35609\_Mf\_liverA, Unigene23185\_Mf\_liverA, Unigene7048\_Mf\_liverA, Unigene13950\_Mf\_liverA, Unigene35046\_Mf\_liverA, Unigene28459\_Mf\_liverA, Unigene37245\_Mf\_liverA, Unigene20372\_Mf\_liverA, Unigene29985\_Mf\_liverA, CL3104.Contig1\_Mf\_liverA, Unigene13018\_Mf\_liverA, Unigene36593\_Mf\_liverA, Unigene37616\_Mf\_liverA, NM\_027406, Unigene36699\_Mf\_liverA, Unigene36176\_Mf\_liverA, Unigene36673\_Mf\_liverA, Unigene18340\_Mf\_liverA, Unigene496\_Mf\_liverA, CL5698.Contig1\_Mf\_liverA, Unigene6110\_Mf\_liverA, Unigene4556\_Mf\_liverA, Unigene29889\_Mf\_liverA, Unigene2939\_Mf\_liverA, Unigene9466\_Mf\_liverA, Unigene26055\_Mf\_liverA, Unigene29399\_Mf\_liverA, CL5640.Contig1\_Mf\_liverA, NM\_020559, Unigene35884\_Mf\_liverA, NM\_008292, Unigene28142\_Mf\_liverA, Unigene38015\_Mf\_liverA, CL3835.Contig2\_Mf\_liverA, Unigene31199\_Mf\_liverA, Unigene6959\_Mf\_liverA, Unigene28899\_Mf\_liverA, Unigene29308\_Mf\_liverA, CL523.Contig1\_Mf\_liverA, Unigene13153\_Mf\_liverA, CL4117.Contig1\_Mf\_liverA, Unigene8473\_Mf\_liverA, Unigene29008\_Mf\_liverA, CL482.Contig1\_Mf\_liverA, Unigene33508\_Mf\_liverA, Unigene36762\_Mf\_liverA, CL777.Contig8\_Mf\_liverA, CL3725.Contig1\_Mf\_liverA, NM\_011072, Unigene21466\_Mf\_liverA, Unigene30878\_Mf\_liverA, Unigene40289\_Mf\_liverA, Unigene34810\_Mf\_liverA, CL44.Contig1\_Mf\_liverA, Unigene27420\_Mf\_liverA, Unigene37880\_Mf\_liverA, Unigene38331\_Mf\_liverA, Unigene20602\_Mf\_liverA, Unigene37999\_Mf\_liverA, Unigene40020\_Mf\_liverA, Unigene37904\_Mf\_liverA, Unigene41336\_Mf\_liverA, CL3483.Contig1\_Mf\_liverA, Unigene25070\_Mf\_liverA, CL4141.Contig1\_Mf\_liverA, Unigene34184\_Mf\_liverA, CL5191.Contig2\_Mf\_liverA, CL2001.Contig1\_Mf\_liverA, Unigene26194\_Mf\_liverA, Unigene13593\_Mf\_liverA, Unigene4686\_Mf\_liverA, Unigene11\_Mf\_liverA, Unigene843\_Mf\_liverA, Unigene25596\_Mf\_liverA, CL1493.Contig2\_Mf\_liverA, Unigene12153\_Mf\_liverA, Unigene25046\_Mf\_liverA, CL5268.Contig1\_Mf\_liverA, Unigene5294\_Mf\_liverA, Unigene2745\_Mf\_liverA, Unigene38104\_Mf\_liverA, Unigene25462\_Mf\_liverA, Unigene5775\_Mf\_liverA, Unigene36626\_Mf\_liverA, CL4076.Contig1\_Mf\_liverA, CL787.Contig1\_Mf\_liverA, Unigene39507\_Mf\_liverA, Unigene25524\_Mf\_liverA, Unigene28822\_Mf\_liverA, Unigene4\_Mf\_liverA, CL3750.Contig2\_Mf\_liverA, Unigene5382\_Mf\_liverA, Unigene151\_Mf\_liverA, Unigene33459\_Mf\_liverA, CL848.Contig2\_Mf\_liverA, Unigene23870\_Mf\_liverA, Unigene5632\_Mf\_liverA, Unigene36669\_Mf\_liverA, CL1263.Contig1\_Mf\_liverA, Unigene37460\_Mf\_liverA, Unigene31517\_Mf\_liverA, CL2632.Contig2\_Mf\_liverA, Unigene24323\_Mf\_liverA, CL2142.Contig2\_Mf\_liverA, Unigene24503\_Mf\_liverA, Unigene27422\_Mf\_liverA, CL3816.Contig1\_Mf\_liverA, CL4701.Contig1\_Mf\_liverA, Unigene25333\_Mf\_liverA, CL993.Contig2\_Mf\_liverA, Unigene36757\_Mf\_liverA, CL5631.Contig1\_Mf\_liverA, Unigene31427\_Mf\_liverA, CL529.Contig2\_Mf\_liverA, Unigene30584\_Mf\_liverA, CL4220.Contig2\_Mf\_liverA, Unigene17579\_Mf\_liverA, Unigene27081\_Mf\_liverA, Unigene431\_Mf\_liverA, NM\_001081372, NM\_010391, NM\_010227, Unigene21317\_Mf\_liverA, Unigene30493\_Mf\_liverA, Unigene7195\_Mf\_liverA, Unigene8132\_Mf\_liverA, Unigene37178\_Mf\_liverA, Unigene27082\_Mf\_liverA, Unigene5712\_Mf\_liverA, Unigene43107\_Mf\_liverA, Unigene1205\_Mf\_liverA, CL442.Contig2\_Mf\_liverA, NM\_033374, CL1352.Contig1\_Mf\_liverA, CL6039.Contig1\_Mf\_liverA, CL2855.Contig1\_Mf\_liverA, CL4411.Contig4\_Mf\_liverA, NM\_001025388, Unigene24804\_Mf\_liverA, Unigene31206\_Mf\_liverA, NM\_013821, Unigene40796\_Mf\_liverA, Unigene13233\_Mf\_liverA, NM\_011099, NM\_010481, CL4086.Contig1\_Mf\_liverA, NM\_009128, Unigene15318\_Mf\_liverA, CL2240.Contig1\_Mf\_liverA, Unigene36190\_Mf\_liverA, NM\_008293, Unigene7970\_Mf\_liverA, NM\_009255, Unigene30154\_Mf\_liverA, CL1125.Contig1\_Mf\_liverA, CL4162.Contig1\_Mf\_liverA, Unigene35237\_Mf\_liverA, Unigene112\_Mf\_liverA, Unigene12907\_Mf\_liverA, Unigene15681\_Mf\_liverA, Unigene25292\_Mf\_liverA, Unigene5165\_Mf\_liverA, Unigene15588\_Mf\_liverA, CL5796.Contig2\_Mf\_liverA, Unigene30707\_Mf\_liverA, Unigene36414\_Mf\_liverA, CL1988.Contig1\_Mf\_liverA, NM\_133838, Unigene5175\_Mf\_liverA, Unigene13894\_Mf\_liverA, Unigene36328\_Mf\_liverA, CL3002.Contig1\_Mf\_liverA, Unigene30155\_Mf\_liverA, CL5057.Contig1\_Mf\_liverA, Unigene36514\_Mf\_liverA, CL6039.Contig2\_Mf\_liverA, CL4816.Contig2\_Mf\_liverA, CL887.Contig2\_Mf\_liverA, Unigene33523\_Mf\_liverA, Unigene32695\_Mf\_liverA, CL3207.Contig1\_Mf\_liverA, Unigene5906\_Mf\_liverA, Unigene43357\_Mf\_liverA, Unigene28731\_Mf\_liverA, Unigene14810\_Mf\_liverA, Unigene1212\_Mf\_liverA, CL2791.Contig1\_Mf\_liverA, CL4770.Contig1\_Mf\_liverA, CL3549.Contig1\_Mf\_liverA, Unigene10135\_Mf\_liverA, Unigene1483\_Mf\_liverA, Unigene32294\_Mf\_liverA, CL3750.Contig1\_Mf\_liverA, CL114.Contig2\_Mf\_liverA, Unigene18796\_Mf\_liverA, Unigene33526\_Mf\_liverA, Unigene1327\_Mf\_liverA, Unigene39252\_Mf\_liverA, CL5307.Contig1\_Mf\_liverA, Unigene3377\_Mf\_liverA, NM\_001081172, CL5978.Contig2\_Mf\_liverA, Unigene8740\_Mf\_liverA, CL738.Contig2\_Mf\_liverA, CL3816.Contig2\_Mf\_liverA, Unigene14270\_Mf\_liverA, Unigene33525\_Mf\_liverA, CL4048.Contig1\_Mf\_liverA, CL1052.Contig1\_Mf\_liverA, CL442.Contig5\_Mf\_liverA, CL5459.Contig2\_Mf\_liverA, NM\_012030, Unigene9406\_Mf\_liverA, Unigene24477\_Mf\_liverA, Unigene120\_Mf\_liverA, Unigene30731\_Mf\_liverA, Unigene30983\_Mf\_liverA, CL4007.Contig1\_Mf\_liverA, Unigene15026\_Mf\_liverA, Unigene36418\_Mf\_liverA, Unigene14940\_Mf\_liverA, Unigene1221\_Mf\_liverA, CL3835.Contig1\_Mf\_liverA, Unigene39875\_Mf\_liverA, Unigene38311\_Mf\_liverA, Unigene550\_Mf\_liverA, Unigene31198\_Mf\_liverA, Unigene1292\_Mf\_liverA, CL2355.Contig1\_Mf\_liverA, Unigene28314\_Mf\_liverA, Unigene37153\_Mf\_liverA, Unigene14809\_Mf\_liverA, Unigene32295\_Mf\_liverA, Unigene21562\_Mf\_liverA, Unigene15592\_Mf\_liverA, Unigene5745\_Mf\_liverA, Unigene38514\_Mf\_liverA, Unigene665\_Mf\_liverA, Unigene2195\_Mf\_liverA, Unigene12889\_Mf\_liverA, Unigene30892\_Mf\_liverA, Unigene8560\_Mf\_liverA, Unigene34866\_Mf\_liverA, Unigene4681\_Mf\_liverA, Unigene16891\_Mf\_liverA, Unigene12519\_Mf\_liverA, Unigene37819\_Mf\_liverA, Unigene5886\_Mf\_liverA, CL336.Contig3\_Mf\_liverA |
| endoplasmic reticulum | NM\_009128, CL2240.Contig1\_Mf\_liverA, CL523.Contig1\_Mf\_liverA, Unigene13153\_Mf\_liverA, CL2797.Contig2\_Mf\_liverA, Unigene7970\_Mf\_liverA, CL482.Contig1\_Mf\_liverA, Unigene37243\_Mf\_liverA, Unigene35237\_Mf\_liverA, Unigene30587\_Mf\_liverA, Unigene21466\_Mf\_liverA, Unigene22052\_Mf\_liverA, Unigene40289\_Mf\_liverA, CL5796.Contig2\_Mf\_liverA, CL4757.Contig1\_Mf\_liverA, Unigene37880\_Mf\_liverA, CL1988.Contig1\_Mf\_liverA, Unigene36328\_Mf\_liverA, Unigene34341\_Mf\_liverA, CL1988.Contig3\_Mf\_liverA, Unigene37535\_Mf\_liverA, CL695.Contig1\_Mf\_liverA, Unigene36514\_Mf\_liverA, CL4816.Contig2\_Mf\_liverA, CL887.Contig2\_Mf\_liverA, CL1988.Contig2\_Mf\_liverA, Unigene4686\_Mf\_liverA, Unigene25596\_Mf\_liverA, Unigene32695\_Mf\_liverA, Unigene25594\_Mf\_liverA, CL1493.Contig2\_Mf\_liverA, Unigene43357\_Mf\_liverA, Unigene28731\_Mf\_liverA, CL1493.Contig1\_Mf\_liverA, Unigene5294\_Mf\_liverA, Unigene30585\_Mf\_liverA, Unigene10135\_Mf\_liverA, Unigene25462\_Mf\_liverA, Unigene542\_Mf\_liverA, Unigene5775\_Mf\_liverA, CL787.Contig1\_Mf\_liverA, Unigene32294\_Mf\_liverA, CL114.Contig2\_Mf\_liverA, NM\_010162, CL2855.Contig2\_Mf\_liverA, CL4220.Contig1\_Mf\_liverA, Unigene25226\_Mf\_liverA, CL5307.Contig1\_Mf\_liverA, Unigene21561\_Mf\_liverA, Unigene3377\_Mf\_liverA, Unigene8740\_Mf\_liverA, Unigene4922\_Mf\_liverA, Unigene5632\_Mf\_liverA, CL738.Contig2\_Mf\_liverA, Unigene36669\_Mf\_liverA, CL4490.Contig2\_Mf\_liverA, Unigene25595\_Mf\_liverA, CL4816.Contig3\_Mf\_liverA, CL2142.Contig2\_Mf\_liverA, Unigene139\_Mf\_liverA, Unigene35609\_Mf\_liverA, CL4007.Contig1\_Mf\_liverA, Unigene7048\_Mf\_liverA, Unigene23185\_Mf\_liverA, CL529.Contig2\_Mf\_liverA, Unigene30584\_Mf\_liverA, Unigene5774\_Mf\_liverA, CL4220.Contig2\_Mf\_liverA, Unigene35046\_Mf\_liverA, Unigene27081\_Mf\_liverA, Unigene36836\_Mf\_liverA, NM\_001081372, Unigene37389\_Mf\_liverA, CL3104.Contig1\_Mf\_liverA, Unigene30493\_Mf\_liverA, Unigene32295\_Mf\_liverA, Unigene36593\_Mf\_liverA, Unigene21562\_Mf\_liverA, Unigene7195\_Mf\_liverA, Unigene37616\_Mf\_liverA, Unigene8132\_Mf\_liverA, Unigene2195\_Mf\_liverA, Unigene12889\_Mf\_liverA, Unigene36699\_Mf\_liverA, Unigene27082\_Mf\_liverA, Unigene5712\_Mf\_liverA, CL5698.Contig1\_Mf\_liverA, Unigene1280\_Mf\_liverA, Unigene1205\_Mf\_liverA, CL442.Contig2\_Mf\_liverA, CL3166.Contig4\_Mf\_liverA, CL2855.Contig1\_Mf\_liverA, CL4411.Contig4\_Mf\_liverA, Unigene5886\_Mf\_liverA, CL336.Contig3\_Mf\_liverA, Unigene40796\_Mf\_liverA |
| nuclear outer membrane-endoplasmic reticulum membrane network | Unigene32294\_Mf\_liverA, CL114.Contig2\_Mf\_liverA, NM\_009128, NM\_010162, CL2855.Contig2\_Mf\_liverA, CL4220.Contig1\_Mf\_liverA, CL2240.Contig1\_Mf\_liverA, CL5307.Contig1\_Mf\_liverA, CL523.Contig1\_Mf\_liverA, Unigene13153\_Mf\_liverA, CL2797.Contig2\_Mf\_liverA, Unigene7970\_Mf\_liverA, Unigene3377\_Mf\_liverA, Unigene4922\_Mf\_liverA, Unigene5632\_Mf\_liverA, CL738.Contig2\_Mf\_liverA, CL482.Contig1\_Mf\_liverA, CL4490.Contig2\_Mf\_liverA, Unigene35237\_Mf\_liverA, CL4816.Contig3\_Mf\_liverA, Unigene139\_Mf\_liverA, Unigene35609\_Mf\_liverA, CL4007.Contig1\_Mf\_liverA, CL529.Contig2\_Mf\_liverA, Unigene7048\_Mf\_liverA, Unigene23185\_Mf\_liverA, Unigene5774\_Mf\_liverA, CL4220.Contig2\_Mf\_liverA, Unigene35046\_Mf\_liverA, Unigene37880\_Mf\_liverA, CL2478.Contig3\_Mf\_liverA, Unigene27081\_Mf\_liverA, CL1988.Contig1\_Mf\_liverA, Unigene34341\_Mf\_liverA, CL1988.Contig3\_Mf\_liverA, Unigene37535\_Mf\_liverA, Unigene37389\_Mf\_liverA, CL695.Contig1\_Mf\_liverA, Unigene36514\_Mf\_liverA, Unigene32295\_Mf\_liverA, Unigene36593\_Mf\_liverA, Unigene7195\_Mf\_liverA, CL4816.Contig2\_Mf\_liverA, Unigene36699\_Mf\_liverA, CL1988.Contig2\_Mf\_liverA, Unigene27082\_Mf\_liverA, Unigene32695\_Mf\_liverA, Unigene5712\_Mf\_liverA, CL5698.Contig1\_Mf\_liverA, Unigene43357\_Mf\_liverA, Unigene1280\_Mf\_liverA, Unigene28731\_Mf\_liverA, Unigene1205\_Mf\_liverA, CL3166.Contig4\_Mf\_liverA, CL2855.Contig1\_Mf\_liverA, Unigene5294\_Mf\_liverA, Unigene5886\_Mf\_liverA, CL336.Contig3\_Mf\_liverA, Unigene40796\_Mf\_liverA, Unigene542\_Mf\_liverA, Unigene10135\_Mf\_liverA, Unigene25462\_Mf\_liverA, Unigene5775\_Mf\_liverA |
| membrane | Unigene21684\_Mf\_liverA, NM\_176843, NM\_009898, Unigene25721\_Mf\_liverA, NM\_001025208, Unigene22874\_Mf\_liverA, Unigene32891\_Mf\_liverA, NM\_010807, Unigene24111\_Mf\_liverA, NM\_018815, Unigene28186\_Mf\_liverA, NM\_010378, Unigene34727\_Mf\_liverA, NM\_011082, Unigene29424\_Mf\_liverA, Unigene26053\_Mf\_liverA, NM\_028862, Unigene16463\_Mf\_liverA, Unigene34852\_Mf\_liverA, CL3847.Contig1\_Mf\_liverA, Unigene39886\_Mf\_liverA, Unigene38689\_Mf\_liverA, NM\_021273, Unigene1137\_Mf\_liverA, Unigene27294\_Mf\_liverA, NR\_004446, CL1555.Contig1\_Mf\_liverA, CL5586.Contig1\_Mf\_liverA, Unigene32564\_Mf\_liverA, Unigene34341\_Mf\_liverA, Unigene37535\_Mf\_liverA, Unigene30142\_Mf\_liverA, CL695.Contig1\_Mf\_liverA, Unigene15703\_Mf\_liverA, Unigene35816\_Mf\_liverA, Unigene31623\_Mf\_liverA, CL593.Contig2\_Mf\_liverA, NM\_145474, CL2260.Contig1\_Mf\_liverA, NM\_010233, Unigene13379\_Mf\_liverA, Unigene34394\_Mf\_liverA, Unigene31694\_Mf\_liverA, NM\_031165, NM\_153193, Unigene5138\_Mf\_liverA, Unigene37150\_Mf\_liverA, Unigene14508\_Mf\_liverA, Unigene34143\_Mf\_liverA, Unigene748\_Mf\_liverA, Unigene8054\_Mf\_liverA, Unigene802\_Mf\_liverA, Unigene17048\_Mf\_liverA, Unigene5552\_Mf\_liverA, CL5807.Contig1\_Mf\_liverA, Unigene13363\_Mf\_liverA, Unigene31958\_Mf\_liverA, Unigene26065\_Mf\_liverA, Unigene13230\_Mf\_liverA, CL2855.Contig2\_Mf\_liverA, Unigene18125\_Mf\_liverA, CL4293.Contig1\_Mf\_liverA, Unigene13097\_Mf\_liverA, Unigene25226\_Mf\_liverA, Unigene21561\_Mf\_liverA, Unigene5366\_Mf\_liverA, NM\_010001, Unigene4922\_Mf\_liverA, NM\_153505, Unigene30261\_Mf\_liverA, Unigene941\_Mf\_liverA, NM\_001104531, Unigene139\_Mf\_liverA, Unigene33560\_Mf\_liverA, Unigene37420\_Mf\_liverA, Unigene37575\_Mf\_liverA, Unigene5774\_Mf\_liverA, Unigene25398\_Mf\_liverA, Unigene27547\_Mf\_liverA, NM\_134156, CL5316.Contig1\_Mf\_liverA, Unigene37389\_Mf\_liverA, Unigene4720\_Mf\_liverA, Unigene32332\_Mf\_liverA, Unigene4723\_Mf\_liverA, Unigene14070\_Mf\_liverA, Unigene29960\_Mf\_liverA, CL4156.Contig1\_Mf\_liverA, Unigene27248\_Mf\_liverA, CL5254.Contig1\_Mf\_liverA, Unigene34010\_Mf\_liverA, Unigene24613\_Mf\_liverA, Unigene31852\_Mf\_liverA, Unigene37076\_Mf\_liverA, Unigene1280\_Mf\_liverA, CL3166.Contig4\_Mf\_liverA, Unigene35698\_Mf\_liverA, CL275.Contig5\_Mf\_liverA, Unigene33632\_Mf\_liverA, Unigene15184\_Mf\_liverA, Unigene2746\_Mf\_liverA, Unigene16465\_Mf\_liverA, Unigene5169\_Mf\_liverA, Unigene10496\_Mf\_liverA, NM\_010392, Unigene31251\_Mf\_liverA, Unigene4363\_Mf\_liverA, Unigene40610\_Mf\_liverA, CL4925.Contig1\_Mf\_liverA, CL425.Contig1\_Mf\_liverA, Unigene32421\_Mf\_liverA, CL2797.Contig2\_Mf\_liverA, Unigene37243\_Mf\_liverA, Unigene27026\_Mf\_liverA, Unigene30587\_Mf\_liverA, Unigene39403\_Mf\_liverA, Unigene15077\_Mf\_liverA, CL4600.Contig1\_Mf\_liverA, Unigene38843\_Mf\_liverA, Unigene29231\_Mf\_liverA, Unigene5693\_Mf\_liverA, Unigene22052\_Mf\_liverA, NM\_010003, Unigene34867\_Mf\_liverA, CL3800.Contig1\_Mf\_liverA, CL2478.Contig3\_Mf\_liverA, Unigene26585\_Mf\_liverA, Unigene4938\_Mf\_liverA, Unigene28687\_Mf\_liverA, CL422.Contig1\_Mf\_liverA, Unigene5292\_Mf\_liverA, CL1988.Contig3\_Mf\_liverA, CL1810.Contig1\_Mf\_liverA, Unigene5758\_Mf\_liverA, CL4033.Contig1\_Mf\_liverA, Unigene38870\_Mf\_liverA, Unigene12843\_Mf\_liverA, Unigene38199\_Mf\_liverA, Unigene24547\_Mf\_liverA, Unigene34983\_Mf\_liverA, Unigene28662\_Mf\_liverA, Unigene4781\_Mf\_liverA, CL3519.Contig1\_Mf\_liverA, CL1988.Contig2\_Mf\_liverA, Unigene29426\_Mf\_liverA, Unigene13462\_Mf\_liverA, Unigene20371\_Mf\_liverA, CL1493.Contig1\_Mf\_liverA, Unigene28499\_Mf\_liverA, Unigene30585\_Mf\_liverA, CL5764.Contig1\_Mf\_liverA, Unigene542\_Mf\_liverA, Unigene4909\_Mf\_liverA, Unigene24471\_Mf\_liverA, Unigene33080\_Mf\_liverA, NM\_010162, Unigene15529\_Mf\_liverA, CL4220.Contig1\_Mf\_liverA, Unigene30947\_Mf\_liverA, NM\_019879, Unigene18126\_Mf\_liverA, CL2251.Contig1\_Mf\_liverA, NM\_007822, CL4490.Contig2\_Mf\_liverA, CL4816.Contig3\_Mf\_liverA, Unigene35090\_Mf\_liverA, Unigene39011\_Mf\_liverA, Unigene20432\_Mf\_liverA, NM\_025336, Unigene35609\_Mf\_liverA, NM\_145218, Unigene23185\_Mf\_liverA, Unigene7048\_Mf\_liverA, Unigene13950\_Mf\_liverA, Unigene35046\_Mf\_liverA, Unigene37245\_Mf\_liverA, Unigene20372\_Mf\_liverA, CL3104.Contig1\_Mf\_liverA, Unigene26580\_Mf\_liverA, Unigene36593\_Mf\_liverA, Unigene19658\_Mf\_liverA, Unigene26336\_Mf\_liverA, Unigene36699\_Mf\_liverA, Unigene18340\_Mf\_liverA, Unigene496\_Mf\_liverA, Unigene42803\_Mf\_liverA, Unigene4983\_Mf\_liverA, CL5698.Contig1\_Mf\_liverA, Unigene7387\_Mf\_liverA, CL591.Contig1\_Mf\_liverA, Unigene9466\_Mf\_liverA, Unigene26055\_Mf\_liverA, NM\_016751, CL5640.Contig1\_Mf\_liverA, Unigene38015\_Mf\_liverA, CL3835.Contig2\_Mf\_liverA, Unigene38831\_Mf\_liverA, Unigene6959\_Mf\_liverA, Unigene7682\_Mf\_liverA, Unigene14603\_Mf\_liverA, Unigene24883\_Mf\_liverA, Unigene29308\_Mf\_liverA, CL523.Contig1\_Mf\_liverA, Unigene13153\_Mf\_liverA, Unigene36849\_Mf\_liverA, Unigene8473\_Mf\_liverA, Unigene29008\_Mf\_liverA, Unigene25055\_Mf\_liverA, CL482.Contig1\_Mf\_liverA, Unigene27593\_Mf\_liverA, CL2339.Contig1\_Mf\_liverA, Unigene22433\_Mf\_liverA, Unigene29788\_Mf\_liverA, Unigene36762\_Mf\_liverA, CL777.Contig8\_Mf\_liverA, NM\_007820, Unigene14816\_Mf\_liverA, Unigene37698\_Mf\_liverA, Unigene30878\_Mf\_liverA, Unigene34810\_Mf\_liverA, Unigene24344\_Mf\_liverA, Unigene37880\_Mf\_liverA, Unigene18500\_Mf\_liverA, Unigene36889\_Mf\_liverA, Unigene40020\_Mf\_liverA, Unigene41336\_Mf\_liverA, NM\_028279, Unigene24112\_Mf\_liverA, Unigene25426\_Mf\_liverA, CL5191.Contig2\_Mf\_liverA, CL2001.Contig1\_Mf\_liverA, Unigene38110\_Mf\_liverA, Unigene26194\_Mf\_liverA, Unigene31333\_Mf\_liverA, Unigene13593\_Mf\_liverA, Unigene30369\_Mf\_liverA, Unigene4686\_Mf\_liverA, Unigene11\_Mf\_liverA, Unigene4630\_Mf\_liverA, Unigene843\_Mf\_liverA, CL1493.Contig2\_Mf\_liverA, CL3575.Contig1\_Mf\_liverA, Unigene12153\_Mf\_liverA, Unigene22432\_Mf\_liverA, Unigene25046\_Mf\_liverA, Unigene5294\_Mf\_liverA, Unigene2745\_Mf\_liverA, Unigene25462\_Mf\_liverA, Unigene5775\_Mf\_liverA, CL787.Contig1\_Mf\_liverA, Unigene25524\_Mf\_liverA, NM\_153795, Unigene31988\_Mf\_liverA, NM\_201360, Unigene33459\_Mf\_liverA, Unigene23870\_Mf\_liverA, Unigene13143\_Mf\_liverA, Unigene5632\_Mf\_liverA, CL1263.Contig1\_Mf\_liverA, NM\_007811, Unigene34746\_Mf\_liverA, CL2142.Contig2\_Mf\_liverA, Unigene24503\_Mf\_liverA, Unigene15583\_Mf\_liverA, Unigene27422\_Mf\_liverA, CL3816.Contig1\_Mf\_liverA, CL993.Contig2\_Mf\_liverA, Unigene25333\_Mf\_liverA, Unigene36757\_Mf\_liverA, CL5631.Contig1\_Mf\_liverA, NM\_153589, CL529.Contig2\_Mf\_liverA, Unigene30584\_Mf\_liverA, CL4220.Contig2\_Mf\_liverA, Unigene17579\_Mf\_liverA, Unigene27081\_Mf\_liverA, Unigene431\_Mf\_liverA, NM\_001101488, NM\_010391, NM\_010227, Unigene860\_Mf\_liverA, Unigene7195\_Mf\_liverA, Unigene8132\_Mf\_liverA, NM\_019717, Unigene27082\_Mf\_liverA, Unigene5712\_Mf\_liverA, Unigene43107\_Mf\_liverA, Unigene1205\_Mf\_liverA, CL442.Contig2\_Mf\_liverA, Unigene37497\_Mf\_liverA, NM\_010380, CL6039.Contig1\_Mf\_liverA, CL2855.Contig1\_Mf\_liverA, Unigene33760\_Mf\_liverA, NM\_001025388, Unigene24804\_Mf\_liverA, Unigene31206\_Mf\_liverA, NM\_013821, Unigene40796\_Mf\_liverA, Unigene72\_Mf\_liverA, Unigene13233\_Mf\_liverA, NM\_011099, NM\_001100182, CL4086.Contig1\_Mf\_liverA, CL3822.Contig2\_Mf\_liverA, NM\_009128, Unigene20167\_Mf\_liverA, Unigene15318\_Mf\_liverA, CL2240.Contig1\_Mf\_liverA, Unigene29823\_Mf\_liverA, Unigene36190\_Mf\_liverA, NM\_008293, Unigene7970\_Mf\_liverA, NM\_009255, Unigene15982\_Mf\_liverA, Unigene30154\_Mf\_liverA, CL1125.Contig1\_Mf\_liverA, Unigene35237\_Mf\_liverA, Unigene112\_Mf\_liverA, Unigene3752\_Mf\_liverA, Unigene591\_Mf\_liverA, Unigene38598\_Mf\_liverA, Unigene12907\_Mf\_liverA, Unigene15681\_Mf\_liverA, Unigene5165\_Mf\_liverA, CL5796.Contig2\_Mf\_liverA, NM\_011170, Unigene30707\_Mf\_liverA, Unigene36543\_Mf\_liverA, CL1988.Contig1\_Mf\_liverA, NM\_133838, Unigene36328\_Mf\_liverA, NM\_010141, CL3002.Contig1\_Mf\_liverA, Unigene30155\_Mf\_liverA, Unigene6895\_Mf\_liverA, Unigene36514\_Mf\_liverA, Unigene24250\_Mf\_liverA, CL6039.Contig2\_Mf\_liverA, CL4816.Contig2\_Mf\_liverA, CL887.Contig2\_Mf\_liverA, Unigene37139\_Mf\_liverA, Unigene25994\_Mf\_liverA, Unigene32695\_Mf\_liverA, CL3207.Contig1\_Mf\_liverA, Unigene5906\_Mf\_liverA, NM\_001081148, Unigene43357\_Mf\_liverA, Unigene28731\_Mf\_liverA, NM\_033444, Unigene4597\_Mf\_liverA, Unigene14170\_Mf\_liverA, Unigene10135\_Mf\_liverA, Unigene32294\_Mf\_liverA, CL114.Contig2\_Mf\_liverA, Unigene7683\_Mf\_liverA, Unigene18796\_Mf\_liverA, Unigene23255\_Mf\_liverA, Unigene13616\_Mf\_liverA, Unigene21337\_Mf\_liverA, Unigene39252\_Mf\_liverA, CL5307.Contig1\_Mf\_liverA, Unigene3377\_Mf\_liverA, Unigene28873\_Mf\_liverA, NM\_001081172, Unigene8740\_Mf\_liverA, NM\_178405, CL738.Contig2\_Mf\_liverA, CL3816.Contig2\_Mf\_liverA, Unigene14270\_Mf\_liverA, CL1052.Contig1\_Mf\_liverA, Unigene14580\_Mf\_liverA, CL5459.Contig2\_Mf\_liverA, NM\_012030, Unigene9406\_Mf\_liverA, CL5062.Contig2\_Mf\_liverA, Unigene120\_Mf\_liverA, Unigene39749\_Mf\_liverA, CL4007.Contig1\_Mf\_liverA, Unigene1221\_Mf\_liverA, Unigene25052\_Mf\_liverA, CL3835.Contig1\_Mf\_liverA, Unigene550\_Mf\_liverA, Unigene1292\_Mf\_liverA, CL2355.Contig1\_Mf\_liverA, Unigene677\_Mf\_liverA, Unigene32295\_Mf\_liverA, Unigene21562\_Mf\_liverA, Unigene4636\_Mf\_liverA, Unigene5745\_Mf\_liverA, Unigene21336\_Mf\_liverA, Unigene38514\_Mf\_liverA, Unigene2195\_Mf\_liverA, Unigene12889\_Mf\_liverA, Unigene35417\_Mf\_liverA, Unigene30892\_Mf\_liverA, NM\_001099634, Unigene33720\_Mf\_liverA, Unigene34866\_Mf\_liverA, Unigene14765\_Mf\_liverA, Unigene4681\_Mf\_liverA, Unigene16891\_Mf\_liverA, Unigene12519\_Mf\_liverA, Unigene979\_Mf\_liverA, Unigene31080\_Mf\_liverA, Unigene37819\_Mf\_liverA, Unigene14284\_Mf\_liverA, Unigene5886\_Mf\_liverA, CL336.Contig3\_Mf\_liverA |
| intrinsic to plasma membrane | Unigene40610\_Mf\_liverA, Unigene22874\_Mf\_liverA, CL425.Contig1\_Mf\_liverA, Unigene24111\_Mf\_liverA, Unigene24883\_Mf\_liverA, Unigene15982\_Mf\_liverA, Unigene25055\_Mf\_liverA, Unigene27593\_Mf\_liverA, CL2339.Contig1\_Mf\_liverA, Unigene22433\_Mf\_liverA, Unigene29788\_Mf\_liverA, Unigene36762\_Mf\_liverA, CL4600.Contig1\_Mf\_liverA, Unigene29231\_Mf\_liverA, Unigene24344\_Mf\_liverA, Unigene18500\_Mf\_liverA, CL5586.Contig1\_Mf\_liverA, Unigene36889\_Mf\_liverA, Unigene32564\_Mf\_liverA, Unigene24112\_Mf\_liverA, Unigene5758\_Mf\_liverA, Unigene15703\_Mf\_liverA, Unigene38870\_Mf\_liverA, Unigene38199\_Mf\_liverA, Unigene4781\_Mf\_liverA, Unigene37139\_Mf\_liverA, Unigene13593\_Mf\_liverA, Unigene30369\_Mf\_liverA, Unigene4686\_Mf\_liverA, Unigene29426\_Mf\_liverA, Unigene843\_Mf\_liverA, Unigene13462\_Mf\_liverA, Unigene5906\_Mf\_liverA, Unigene20371\_Mf\_liverA, CL3575.Contig1\_Mf\_liverA, Unigene22432\_Mf\_liverA, Unigene4909\_Mf\_liverA, Unigene13363\_Mf\_liverA, Unigene26065\_Mf\_liverA, CL2855.Contig2\_Mf\_liverA, Unigene18125\_Mf\_liverA, CL4293.Contig1\_Mf\_liverA, Unigene21337\_Mf\_liverA, Unigene39252\_Mf\_liverA, Unigene30947\_Mf\_liverA, Unigene21561\_Mf\_liverA, Unigene18126\_Mf\_liverA, Unigene8740\_Mf\_liverA, NM\_178405, Unigene35090\_Mf\_liverA, Unigene39011\_Mf\_liverA, Unigene39749\_Mf\_liverA, Unigene37575\_Mf\_liverA, Unigene25052\_Mf\_liverA, Unigene13950\_Mf\_liverA, Unigene27081\_Mf\_liverA, Unigene20372\_Mf\_liverA, CL4156.Contig1\_Mf\_liverA, Unigene19658\_Mf\_liverA, Unigene21562\_Mf\_liverA, Unigene21336\_Mf\_liverA, Unigene18340\_Mf\_liverA, Unigene27082\_Mf\_liverA, Unigene14765\_Mf\_liverA, Unigene4983\_Mf\_liverA, Unigene37497\_Mf\_liverA, Unigene35698\_Mf\_liverA, Unigene33632\_Mf\_liverA, CL2855.Contig1\_Mf\_liverA, Unigene14284\_Mf\_liverA, Unigene5169\_Mf\_liverA |
| endoplasmic reticulum membrane | Unigene32294\_Mf\_liverA, CL114.Contig2\_Mf\_liverA, NM\_009128, NM\_010162, CL2855.Contig2\_Mf\_liverA, CL4220.Contig1\_Mf\_liverA, CL2240.Contig1\_Mf\_liverA, CL5307.Contig1\_Mf\_liverA, CL523.Contig1\_Mf\_liverA, Unigene13153\_Mf\_liverA, CL2797.Contig2\_Mf\_liverA, Unigene7970\_Mf\_liverA, Unigene3377\_Mf\_liverA, Unigene4922\_Mf\_liverA, Unigene5632\_Mf\_liverA, CL738.Contig2\_Mf\_liverA, CL482.Contig1\_Mf\_liverA, CL4490.Contig2\_Mf\_liverA, Unigene35237\_Mf\_liverA, CL4816.Contig3\_Mf\_liverA, Unigene139\_Mf\_liverA, Unigene35609\_Mf\_liverA, CL4007.Contig1\_Mf\_liverA, CL529.Contig2\_Mf\_liverA, Unigene7048\_Mf\_liverA, Unigene23185\_Mf\_liverA, Unigene5774\_Mf\_liverA, CL4220.Contig2\_Mf\_liverA, Unigene35046\_Mf\_liverA, Unigene37880\_Mf\_liverA, Unigene27081\_Mf\_liverA, CL1988.Contig1\_Mf\_liverA, Unigene34341\_Mf\_liverA, CL1988.Contig3\_Mf\_liverA, Unigene37389\_Mf\_liverA, CL695.Contig1\_Mf\_liverA, Unigene36514\_Mf\_liverA, Unigene32295\_Mf\_liverA, Unigene36593\_Mf\_liverA, Unigene7195\_Mf\_liverA, CL4816.Contig2\_Mf\_liverA, Unigene36699\_Mf\_liverA, CL1988.Contig2\_Mf\_liverA, Unigene27082\_Mf\_liverA, Unigene32695\_Mf\_liverA, Unigene5712\_Mf\_liverA, CL5698.Contig1\_Mf\_liverA, Unigene43357\_Mf\_liverA, Unigene1280\_Mf\_liverA, Unigene28731\_Mf\_liverA, Unigene1205\_Mf\_liverA, CL3166.Contig4\_Mf\_liverA, CL2855.Contig1\_Mf\_liverA, Unigene5294\_Mf\_liverA, Unigene5886\_Mf\_liverA, CL336.Contig3\_Mf\_liverA, Unigene40796\_Mf\_liverA, Unigene542\_Mf\_liverA, Unigene10135\_Mf\_liverA, Unigene25462\_Mf\_liverA, Unigene5775\_Mf\_liverA |
| endoplasmic reticulum part | NM\_009128, CL2240.Contig1\_Mf\_liverA, CL523.Contig1\_Mf\_liverA, Unigene13153\_Mf\_liverA, CL2797.Contig2\_Mf\_liverA, Unigene7970\_Mf\_liverA, CL482.Contig1\_Mf\_liverA, Unigene35237\_Mf\_liverA, Unigene21466\_Mf\_liverA, Unigene22052\_Mf\_liverA, Unigene37880\_Mf\_liverA, CL1988.Contig1\_Mf\_liverA, Unigene34341\_Mf\_liverA, CL1988.Contig3\_Mf\_liverA, CL695.Contig1\_Mf\_liverA, Unigene36514\_Mf\_liverA, CL4816.Contig2\_Mf\_liverA, CL1988.Contig2\_Mf\_liverA, Unigene4686\_Mf\_liverA, Unigene25596\_Mf\_liverA, Unigene32695\_Mf\_liverA, Unigene43357\_Mf\_liverA, Unigene28731\_Mf\_liverA, Unigene5294\_Mf\_liverA, Unigene542\_Mf\_liverA, Unigene10135\_Mf\_liverA, Unigene25462\_Mf\_liverA, Unigene5775\_Mf\_liverA, Unigene32294\_Mf\_liverA, CL114.Contig2\_Mf\_liverA, NM\_010162, CL2855.Contig2\_Mf\_liverA, CL4220.Contig1\_Mf\_liverA, CL5307.Contig1\_Mf\_liverA, Unigene3377\_Mf\_liverA, Unigene5632\_Mf\_liverA, Unigene4922\_Mf\_liverA, CL738.Contig2\_Mf\_liverA, CL4490.Contig2\_Mf\_liverA, Unigene36669\_Mf\_liverA, Unigene25595\_Mf\_liverA, CL4816.Contig3\_Mf\_liverA, Unigene139\_Mf\_liverA, Unigene35609\_Mf\_liverA, CL4007.Contig1\_Mf\_liverA, Unigene23185\_Mf\_liverA, Unigene7048\_Mf\_liverA, CL529.Contig2\_Mf\_liverA, CL4220.Contig2\_Mf\_liverA, Unigene35046\_Mf\_liverA, Unigene5774\_Mf\_liverA, Unigene27081\_Mf\_liverA, NM\_001081372, Unigene37389\_Mf\_liverA, Unigene30493\_Mf\_liverA, Unigene32295\_Mf\_liverA, Unigene36593\_Mf\_liverA, Unigene7195\_Mf\_liverA, Unigene36699\_Mf\_liverA, Unigene27082\_Mf\_liverA, Unigene5712\_Mf\_liverA, Unigene1280\_Mf\_liverA, CL5698.Contig1\_Mf\_liverA, Unigene1205\_Mf\_liverA, CL3166.Contig4\_Mf\_liverA, CL2855.Contig1\_Mf\_liverA, Unigene5886\_Mf\_liverA, CL336.Contig3\_Mf\_liverA, Unigene40796\_Mf\_liverA |
| MHC class II protein complex | Unigene37245\_Mf\_liverA, Unigene24471\_Mf\_liverA, CL3002.Contig1\_Mf\_liverA, CL3835.Contig2\_Mf\_liverA, CL2855.Contig2\_Mf\_liverA, Unigene4681\_Mf\_liverA, CL2855.Contig1\_Mf\_liverA, Unigene30878\_Mf\_liverA, CL3835.Contig1\_Mf\_liverA |
| integral to plasma membrane | Unigene40610\_Mf\_liverA, Unigene22874\_Mf\_liverA, CL425.Contig1\_Mf\_liverA, Unigene24111\_Mf\_liverA, Unigene24883\_Mf\_liverA, Unigene15982\_Mf\_liverA, Unigene25055\_Mf\_liverA, Unigene27593\_Mf\_liverA, CL2339.Contig1\_Mf\_liverA, Unigene22433\_Mf\_liverA, Unigene29788\_Mf\_liverA, Unigene36762\_Mf\_liverA, CL4600.Contig1\_Mf\_liverA, Unigene29231\_Mf\_liverA, Unigene24344\_Mf\_liverA, Unigene18500\_Mf\_liverA, CL5586.Contig1\_Mf\_liverA, Unigene36889\_Mf\_liverA, Unigene32564\_Mf\_liverA, Unigene24112\_Mf\_liverA, Unigene5758\_Mf\_liverA, Unigene15703\_Mf\_liverA, Unigene38870\_Mf\_liverA, Unigene38199\_Mf\_liverA, Unigene4781\_Mf\_liverA, Unigene37139\_Mf\_liverA, Unigene13593\_Mf\_liverA, Unigene30369\_Mf\_liverA, Unigene4686\_Mf\_liverA, Unigene29426\_Mf\_liverA, Unigene843\_Mf\_liverA, Unigene13462\_Mf\_liverA, Unigene5906\_Mf\_liverA, Unigene20371\_Mf\_liverA, Unigene22432\_Mf\_liverA, Unigene4909\_Mf\_liverA, Unigene13363\_Mf\_liverA, Unigene26065\_Mf\_liverA, CL2855.Contig2\_Mf\_liverA, Unigene18125\_Mf\_liverA, CL4293.Contig1\_Mf\_liverA, Unigene21337\_Mf\_liverA, Unigene39252\_Mf\_liverA, Unigene30947\_Mf\_liverA, Unigene18126\_Mf\_liverA, Unigene8740\_Mf\_liverA, NM\_178405, Unigene35090\_Mf\_liverA, Unigene39011\_Mf\_liverA, Unigene39749\_Mf\_liverA, Unigene37575\_Mf\_liverA, Unigene25052\_Mf\_liverA, Unigene13950\_Mf\_liverA, Unigene27081\_Mf\_liverA, Unigene20372\_Mf\_liverA, Unigene19658\_Mf\_liverA, Unigene21336\_Mf\_liverA, Unigene18340\_Mf\_liverA, Unigene27082\_Mf\_liverA, Unigene14765\_Mf\_liverA, Unigene4983\_Mf\_liverA, Unigene37497\_Mf\_liverA, Unigene35698\_Mf\_liverA, Unigene33632\_Mf\_liverA, CL2855.Contig1\_Mf\_liverA, Unigene14284\_Mf\_liverA, Unigene5169\_Mf\_liverA |
| cytosol | CL5640.Contig1\_Mf\_liverA, Unigene35884\_Mf\_liverA, Unigene38015\_Mf\_liverA, Unigene31251\_Mf\_liverA, CL4925.Contig1\_Mf\_liverA, CL186.Contig3\_Mf\_liverA, Unigene28899\_Mf\_liverA, Unigene36190\_Mf\_liverA, Unigene29308\_Mf\_liverA, Unigene13153\_Mf\_liverA, CL4160.Contig2\_Mf\_liverA, CL4105.Contig1\_Mf\_liverA, Unigene30154\_Mf\_liverA, Unigene35237\_Mf\_liverA, CL777.Contig8\_Mf\_liverA, Unigene39886\_Mf\_liverA, Unigene15681\_Mf\_liverA, Unigene5693\_Mf\_liverA, Unigene15064\_Mf\_liverA, Unigene27339\_Mf\_liverA, Unigene5165\_Mf\_liverA, CL4757.Contig1\_Mf\_liverA, CL44.Contig1\_Mf\_liverA, CL3800.Contig1\_Mf\_liverA, CL2478.Contig3\_Mf\_liverA, Unigene28687\_Mf\_liverA, Unigene20602\_Mf\_liverA, Unigene5815\_Mf\_liverA, Unigene40020\_Mf\_liverA, Unigene37535\_Mf\_liverA, Unigene25070\_Mf\_liverA, CL1810.Contig1\_Mf\_liverA, CL4141.Contig1\_Mf\_liverA, CL6039.Contig2\_Mf\_liverA, Unigene34184\_Mf\_liverA, Unigene24547\_Mf\_liverA, CL5191.Contig2\_Mf\_liverA, Unigene35816\_Mf\_liverA, Unigene26194\_Mf\_liverA, Unigene33523\_Mf\_liverA, Unigene30003\_Mf\_liverA, Unigene25596\_Mf\_liverA, Unigene843\_Mf\_liverA, Unigene5138\_Mf\_liverA, Unigene12153\_Mf\_liverA, CL1493.Contig1\_Mf\_liverA, Unigene1212\_Mf\_liverA, Unigene14050\_Mf\_liverA, Unigene25046\_Mf\_liverA, CL5268.Contig1\_Mf\_liverA, Unigene28499\_Mf\_liverA, Unigene2745\_Mf\_liverA, Unigene30002\_Mf\_liverA, CL3549.Contig1\_Mf\_liverA, Unigene35476\_Mf\_liverA, Unigene24758\_Mf\_liverA, Unigene24471\_Mf\_liverA, Unigene33522\_Mf\_liverA, Unigene4\_Mf\_liverA, Unigene37262\_Mf\_liverA, Unigene151\_Mf\_liverA, CL848.Contig2\_Mf\_liverA, Unigene21561\_Mf\_liverA, CL2251.Contig1\_Mf\_liverA, Unigene8740\_Mf\_liverA, CL1263.Contig1\_Mf\_liverA, CL1052.Contig1\_Mf\_liverA, Unigene30261\_Mf\_liverA, CL442.Contig5\_Mf\_liverA, Unigene24323\_Mf\_liverA, CL2142.Contig2\_Mf\_liverA, Unigene13945\_Mf\_liverA, Unigene24477\_Mf\_liverA, Unigene120\_Mf\_liverA, Unigene30983\_Mf\_liverA, CL5631.Contig1\_Mf\_liverA, Unigene15026\_Mf\_liverA, Unigene31427\_Mf\_liverA, Unigene39875\_Mf\_liverA, Unigene38311\_Mf\_liverA, Unigene27081\_Mf\_liverA, Unigene550\_Mf\_liverA, Unigene431\_Mf\_liverA, CL2355.Contig1\_Mf\_liverA, Unigene37153\_Mf\_liverA, Unigene45530\_Mf\_liverA, Unigene21317\_Mf\_liverA, CL3104.Contig1\_Mf\_liverA, Unigene21562\_Mf\_liverA, Unigene38514\_Mf\_liverA, Unigene665\_Mf\_liverA, CL5254.Contig1\_Mf\_liverA, Unigene12889\_Mf\_liverA, Unigene36673\_Mf\_liverA, Unigene152\_Mf\_liverA, Unigene37178\_Mf\_liverA, Unigene27082\_Mf\_liverA, Unigene31852\_Mf\_liverA, Unigene37076\_Mf\_liverA, CL5698.Contig1\_Mf\_liverA, Unigene30288\_Mf\_liverA, CL442.Contig2\_Mf\_liverA, Unigene16891\_Mf\_liverA, CL6039.Contig1\_Mf\_liverA, CL5189.Contig1\_Mf\_liverA, Unigene2746\_Mf\_liverA, Unigene4556\_Mf\_liverA, Unigene24804\_Mf\_liverA, Unigene2939\_Mf\_liverA |
| cytoplasmic part | Unigene21684\_Mf\_liverA, Unigene5287\_Mf\_liverA, NM\_144940, NM\_009898, Unigene25721\_Mf\_liverA, CL3669.Contig2\_Mf\_liverA, Unigene28186\_Mf\_liverA, NM\_010378, Unigene34727\_Mf\_liverA, NM\_011082, Unigene29424\_Mf\_liverA, CL4160.Contig2\_Mf\_liverA, CL854.Contig1\_Mf\_liverA, Unigene26053\_Mf\_liverA, CL4105.Contig1\_Mf\_liverA, Unigene39886\_Mf\_liverA, Unigene15064\_Mf\_liverA, NM\_021273, Unigene27339\_Mf\_liverA, Unigene24252\_Mf\_liverA, CL4757.Contig1\_Mf\_liverA, NR\_004446, CL5586.Contig1\_Mf\_liverA, Unigene34341\_Mf\_liverA, Unigene37535\_Mf\_liverA, Unigene30142\_Mf\_liverA, CL695.Contig1\_Mf\_liverA, NM\_011921, Unigene35816\_Mf\_liverA, Unigene31623\_Mf\_liverA, NM\_010233, Unigene13379\_Mf\_liverA, NM\_009776, Unigene34394\_Mf\_liverA, Unigene30003\_Mf\_liverA, NM\_031165, Unigene25594\_Mf\_liverA, NM\_153193, Unigene5138\_Mf\_liverA, Unigene14508\_Mf\_liverA, Unigene14050\_Mf\_liverA, Unigene30002\_Mf\_liverA, Unigene8054\_Mf\_liverA, Unigene18430\_Mf\_liverA, Unigene5639\_Mf\_liverA, Unigene17048\_Mf\_liverA, CL5807.Contig1\_Mf\_liverA, Unigene33522\_Mf\_liverA, Unigene13363\_Mf\_liverA, NM\_021278, CL2855.Contig2\_Mf\_liverA, NM\_177093, Unigene25226\_Mf\_liverA, Unigene21561\_Mf\_liverA, Unigene39655\_Mf\_liverA, Unigene4922\_Mf\_liverA, NM\_153505, Unigene30261\_Mf\_liverA, NM\_145942, Unigene36698\_Mf\_liverA, Unigene13945\_Mf\_liverA, Unigene139\_Mf\_liverA, Unigene37575\_Mf\_liverA, Unigene5774\_Mf\_liverA, Unigene25398\_Mf\_liverA, Unigene27547\_Mf\_liverA, CL1736.Contig2\_Mf\_liverA, Unigene36836\_Mf\_liverA, NM\_134156, CL5316.Contig1\_Mf\_liverA, Unigene37389\_Mf\_liverA, Unigene4720\_Mf\_liverA, Unigene45530\_Mf\_liverA, Unigene32332\_Mf\_liverA, Unigene27248\_Mf\_liverA, CL5254.Contig1\_Mf\_liverA, Unigene34010\_Mf\_liverA, Unigene152\_Mf\_liverA, Unigene31852\_Mf\_liverA, CL532.Contig1\_Mf\_liverA, Unigene37076\_Mf\_liverA, Unigene1280\_Mf\_liverA, Unigene30288\_Mf\_liverA, CL3166.Contig4\_Mf\_liverA, Unigene14171\_Mf\_liverA, Unigene33632\_Mf\_liverA, CL5189.Contig1\_Mf\_liverA, CL3339.Contig1\_Mf\_liverA, Unigene2746\_Mf\_liverA, Unigene30528\_Mf\_liverA, Unigene31251\_Mf\_liverA, Unigene4363\_Mf\_liverA, CL186.Contig3\_Mf\_liverA, CL4925.Contig1\_Mf\_liverA, CL425.Contig1\_Mf\_liverA, CL2797.Contig2\_Mf\_liverA, Unigene14916\_Mf\_liverA, Unigene37243\_Mf\_liverA, Unigene30587\_Mf\_liverA, Unigene15077\_Mf\_liverA, CL4600.Contig1\_Mf\_liverA, Unigene36034\_Mf\_liverA, Unigene5693\_Mf\_liverA, Unigene22052\_Mf\_liverA, Unigene34867\_Mf\_liverA, Unigene29876\_Mf\_liverA, CL3800.Contig1\_Mf\_liverA, CL2478.Contig3\_Mf\_liverA, Unigene28687\_Mf\_liverA, Unigene5815\_Mf\_liverA, CL1988.Contig3\_Mf\_liverA, NM\_028785, CL1810.Contig1\_Mf\_liverA, NM\_009609, CL4033.Contig1\_Mf\_liverA, Unigene38870\_Mf\_liverA, Unigene24547\_Mf\_liverA, Unigene28662\_Mf\_liverA, NR\_003623, Unigene4781\_Mf\_liverA, Unigene8033\_Mf\_liverA, CL1988.Contig2\_Mf\_liverA, Unigene20371\_Mf\_liverA, Unigene13683\_Mf\_liverA, CL1493.Contig1\_Mf\_liverA, Unigene28499\_Mf\_liverA, Unigene30585\_Mf\_liverA, Unigene542\_Mf\_liverA, Unigene35476\_Mf\_liverA, Unigene24758\_Mf\_liverA, Unigene4909\_Mf\_liverA, Unigene24471\_Mf\_liverA, Unigene33080\_Mf\_liverA, NM\_010162, Unigene15529\_Mf\_liverA, CL4220.Contig1\_Mf\_liverA, Unigene37262\_Mf\_liverA, NM\_019879, NR\_033215, CL2251.Contig1\_Mf\_liverA, CL4490.Contig2\_Mf\_liverA, Unigene25595\_Mf\_liverA, Unigene14286\_Mf\_liverA, Unigene32515\_Mf\_liverA, CL4816.Contig3\_Mf\_liverA, CL840.Contig1\_Mf\_liverA, Unigene39011\_Mf\_liverA, NM\_009022, Unigene35609\_Mf\_liverA, Unigene7048\_Mf\_liverA, Unigene23185\_Mf\_liverA, Unigene13950\_Mf\_liverA, Unigene35046\_Mf\_liverA, Unigene28459\_Mf\_liverA, Unigene37245\_Mf\_liverA, Unigene20372\_Mf\_liverA, CL3104.Contig1\_Mf\_liverA, Unigene36593\_Mf\_liverA, Unigene37616\_Mf\_liverA, NM\_027406, Unigene36699\_Mf\_liverA, Unigene36673\_Mf\_liverA, Unigene496\_Mf\_liverA, CL5698.Contig1\_Mf\_liverA, Unigene6110\_Mf\_liverA, Unigene4556\_Mf\_liverA, Unigene2939\_Mf\_liverA, Unigene26055\_Mf\_liverA, Unigene29399\_Mf\_liverA, NM\_020559, Unigene35884\_Mf\_liverA, CL5640.Contig1\_Mf\_liverA, NM\_008292, Unigene38015\_Mf\_liverA, CL3835.Contig2\_Mf\_liverA, Unigene31199\_Mf\_liverA, Unigene6959\_Mf\_liverA, Unigene28899\_Mf\_liverA, Unigene29308\_Mf\_liverA, CL523.Contig1\_Mf\_liverA, CL4117.Contig1\_Mf\_liverA, Unigene13153\_Mf\_liverA, Unigene29008\_Mf\_liverA, CL482.Contig1\_Mf\_liverA, Unigene36762\_Mf\_liverA, CL777.Contig8\_Mf\_liverA, CL3725.Contig1\_Mf\_liverA, NM\_011072, Unigene21466\_Mf\_liverA, Unigene30878\_Mf\_liverA, Unigene40289\_Mf\_liverA, Unigene34810\_Mf\_liverA, CL44.Contig1\_Mf\_liverA, Unigene37880\_Mf\_liverA, Unigene38331\_Mf\_liverA, Unigene20602\_Mf\_liverA, Unigene37999\_Mf\_liverA, Unigene40020\_Mf\_liverA, Unigene41336\_Mf\_liverA, Unigene25070\_Mf\_liverA, CL4141.Contig1\_Mf\_liverA, Unigene34184\_Mf\_liverA, CL5191.Contig2\_Mf\_liverA, CL2001.Contig1\_Mf\_liverA, Unigene26194\_Mf\_liverA, Unigene13593\_Mf\_liverA, Unigene11\_Mf\_liverA, Unigene4686\_Mf\_liverA, Unigene843\_Mf\_liverA, Unigene25596\_Mf\_liverA, CL1493.Contig2\_Mf\_liverA, Unigene12153\_Mf\_liverA, Unigene25046\_Mf\_liverA, CL5268.Contig1\_Mf\_liverA, Unigene5294\_Mf\_liverA, Unigene2745\_Mf\_liverA, Unigene38104\_Mf\_liverA, Unigene25462\_Mf\_liverA, Unigene5775\_Mf\_liverA, Unigene36626\_Mf\_liverA, CL787.Contig1\_Mf\_liverA, Unigene25524\_Mf\_liverA, Unigene28822\_Mf\_liverA, Unigene4\_Mf\_liverA, CL3750.Contig2\_Mf\_liverA, Unigene151\_Mf\_liverA, Unigene5382\_Mf\_liverA, Unigene33459\_Mf\_liverA, CL848.Contig2\_Mf\_liverA, Unigene5632\_Mf\_liverA, Unigene36669\_Mf\_liverA, Unigene37460\_Mf\_liverA, CL1263.Contig1\_Mf\_liverA, Unigene24323\_Mf\_liverA, Unigene24503\_Mf\_liverA, CL2142.Contig2\_Mf\_liverA, CL3816.Contig1\_Mf\_liverA, CL4701.Contig1\_Mf\_liverA, CL993.Contig2\_Mf\_liverA, Unigene25333\_Mf\_liverA, Unigene36757\_Mf\_liverA, CL5631.Contig1\_Mf\_liverA, Unigene31427\_Mf\_liverA, CL529.Contig2\_Mf\_liverA, Unigene30584\_Mf\_liverA, CL4220.Contig2\_Mf\_liverA, Unigene431\_Mf\_liverA, Unigene27081\_Mf\_liverA, NM\_001081372, NM\_010391, NM\_010227, Unigene21317\_Mf\_liverA, Unigene30493\_Mf\_liverA, Unigene7195\_Mf\_liverA, Unigene8132\_Mf\_liverA, Unigene37178\_Mf\_liverA, Unigene27082\_Mf\_liverA, Unigene5712\_Mf\_liverA, Unigene1205\_Mf\_liverA, CL442.Contig2\_Mf\_liverA, NM\_033374, CL6039.Contig1\_Mf\_liverA, CL1352.Contig1\_Mf\_liverA, CL2855.Contig1\_Mf\_liverA, CL4411.Contig4\_Mf\_liverA, NM\_001025388, NM\_013821, Unigene31206\_Mf\_liverA, Unigene24804\_Mf\_liverA, Unigene40796\_Mf\_liverA, Unigene13233\_Mf\_liverA, NM\_011099, NM\_010481, CL4086.Contig1\_Mf\_liverA, NM\_009128, Unigene15318\_Mf\_liverA, CL2240.Contig1\_Mf\_liverA, Unigene36190\_Mf\_liverA, NM\_008293, Unigene7970\_Mf\_liverA, NM\_009255, Unigene30154\_Mf\_liverA, CL1125.Contig1\_Mf\_liverA, CL4162.Contig1\_Mf\_liverA, Unigene35237\_Mf\_liverA, Unigene112\_Mf\_liverA, Unigene12907\_Mf\_liverA, Unigene15681\_Mf\_liverA, Unigene25292\_Mf\_liverA, Unigene5165\_Mf\_liverA, CL5796.Contig2\_Mf\_liverA, Unigene30707\_Mf\_liverA, CL1988.Contig1\_Mf\_liverA, NM\_133838, Unigene36328\_Mf\_liverA, CL3002.Contig1\_Mf\_liverA, Unigene36514\_Mf\_liverA, CL6039.Contig2\_Mf\_liverA, CL887.Contig2\_Mf\_liverA, CL4816.Contig2\_Mf\_liverA, Unigene33523\_Mf\_liverA, Unigene32695\_Mf\_liverA, Unigene5906\_Mf\_liverA, Unigene43357\_Mf\_liverA, Unigene28731\_Mf\_liverA, Unigene14810\_Mf\_liverA, Unigene1212\_Mf\_liverA, CL2791.Contig1\_Mf\_liverA, CL3549.Contig1\_Mf\_liverA, Unigene10135\_Mf\_liverA, Unigene32294\_Mf\_liverA, CL3750.Contig1\_Mf\_liverA, CL114.Contig2\_Mf\_liverA, Unigene39252\_Mf\_liverA, CL5307.Contig1\_Mf\_liverA, Unigene3377\_Mf\_liverA, CL5978.Contig2\_Mf\_liverA, Unigene8740\_Mf\_liverA, CL738.Contig2\_Mf\_liverA, CL3816.Contig2\_Mf\_liverA, CL4048.Contig1\_Mf\_liverA, CL1052.Contig1\_Mf\_liverA, CL442.Contig5\_Mf\_liverA, NM\_012030, Unigene24477\_Mf\_liverA, Unigene120\_Mf\_liverA, Unigene30731\_Mf\_liverA, Unigene30983\_Mf\_liverA, CL4007.Contig1\_Mf\_liverA, Unigene15026\_Mf\_liverA, Unigene1221\_Mf\_liverA, CL3835.Contig1\_Mf\_liverA, Unigene39875\_Mf\_liverA, Unigene38311\_Mf\_liverA, Unigene550\_Mf\_liverA, Unigene31198\_Mf\_liverA, Unigene1292\_Mf\_liverA, CL2355.Contig1\_Mf\_liverA, Unigene37153\_Mf\_liverA, Unigene14809\_Mf\_liverA, Unigene32295\_Mf\_liverA, Unigene21562\_Mf\_liverA, Unigene15592\_Mf\_liverA, Unigene5745\_Mf\_liverA, Unigene38514\_Mf\_liverA, Unigene665\_Mf\_liverA, Unigene2195\_Mf\_liverA, Unigene12889\_Mf\_liverA, Unigene8560\_Mf\_liverA, Unigene34866\_Mf\_liverA, Unigene4681\_Mf\_liverA, Unigene16891\_Mf\_liverA, Unigene37819\_Mf\_liverA, Unigene5886\_Mf\_liverA, CL336.Contig3\_Mf\_liverA |
| extracellular organelle | CL482.Contig1\_Mf\_liverA, Unigene36328\_Mf\_liverA, CL442.Contig5\_Mf\_liverA, CL442.Contig2\_Mf\_liverA, CL6039.Contig1\_Mf\_liverA, CL6039.Contig2\_Mf\_liverA, CL993.Contig2\_Mf\_liverA, Unigene33459\_Mf\_liverA, CL2001.Contig1\_Mf\_liverA, CL2251.Contig1\_Mf\_liverA, CL5978.Contig2\_Mf\_liverA, Unigene8033\_Mf\_liverA |
| extracellular membrane-bounded organelle | CL482.Contig1\_Mf\_liverA, Unigene36328\_Mf\_liverA, CL442.Contig5\_Mf\_liverA, CL442.Contig2\_Mf\_liverA, CL6039.Contig1\_Mf\_liverA, CL6039.Contig2\_Mf\_liverA, CL993.Contig2\_Mf\_liverA, Unigene33459\_Mf\_liverA, CL2001.Contig1\_Mf\_liverA, CL2251.Contig1\_Mf\_liverA, CL5978.Contig2\_Mf\_liverA, Unigene8033\_Mf\_liverA |
| symbiont-containing vacuole | CL482.Contig1\_Mf\_liverA, Unigene36328\_Mf\_liverA, CL442.Contig5\_Mf\_liverA, CL442.Contig2\_Mf\_liverA, CL6039.Contig1\_Mf\_liverA, CL6039.Contig2\_Mf\_liverA, CL993.Contig2\_Mf\_liverA, Unigene33459\_Mf\_liverA, CL5978.Contig2\_Mf\_liverA, Unigene8033\_Mf\_liverA |
| host cell cytoplasm | CL482.Contig1\_Mf\_liverA, Unigene36328\_Mf\_liverA, CL442.Contig5\_Mf\_liverA, CL442.Contig2\_Mf\_liverA, CL6039.Contig1\_Mf\_liverA, CL6039.Contig2\_Mf\_liverA, CL993.Contig2\_Mf\_liverA, Unigene33459\_Mf\_liverA, CL5978.Contig2\_Mf\_liverA, Unigene8033\_Mf\_liverA |
| host cell cytoplasm part | CL482.Contig1\_Mf\_liverA, Unigene36328\_Mf\_liverA, CL442.Contig5\_Mf\_liverA, CL442.Contig2\_Mf\_liverA, CL6039.Contig1\_Mf\_liverA, CL6039.Contig2\_Mf\_liverA, CL993.Contig2\_Mf\_liverA, Unigene33459\_Mf\_liverA, CL5978.Contig2\_Mf\_liverA, Unigene8033\_Mf\_liverA |
| integrin complex | Unigene18340\_Mf\_liverA, Unigene22433\_Mf\_liverA, Unigene4983\_Mf\_liverA, Unigene40610\_Mf\_liverA, Unigene24112\_Mf\_liverA, Unigene22432\_Mf\_liverA, Unigene19658\_Mf\_liverA, Unigene39252\_Mf\_liverA, Unigene14284\_Mf\_liverA, Unigene38870\_Mf\_liverA, Unigene24111\_Mf\_liverA, Unigene39749\_Mf\_liverA |
| host intracellular part | CL482.Contig1\_Mf\_liverA, Unigene36328\_Mf\_liverA, CL442.Contig5\_Mf\_liverA, CL442.Contig2\_Mf\_liverA, CL6039.Contig1\_Mf\_liverA, CL6039.Contig2\_Mf\_liverA, CL993.Contig2\_Mf\_liverA, Unigene33459\_Mf\_liverA, CL5978.Contig2\_Mf\_liverA, Unigene8033\_Mf\_liverA |
| intracellular region of host | CL482.Contig1\_Mf\_liverA, Unigene36328\_Mf\_liverA, CL442.Contig5\_Mf\_liverA, CL442.Contig2\_Mf\_liverA, CL6039.Contig1\_Mf\_liverA, CL6039.Contig2\_Mf\_liverA, CL993.Contig2\_Mf\_liverA, Unigene33459\_Mf\_liverA, CL5978.Contig2\_Mf\_liverA, Unigene8033\_Mf\_liverA |
| host | CL482.Contig1\_Mf\_liverA, Unigene36328\_Mf\_liverA, CL442.Contig5\_Mf\_liverA, CL442.Contig2\_Mf\_liverA, CL6039.Contig1\_Mf\_liverA, CL6039.Contig2\_Mf\_liverA, CL993.Contig2\_Mf\_liverA, Unigene33459\_Mf\_liverA, CL5978.Contig2\_Mf\_liverA, Unigene8033\_Mf\_liverA |
| host cell part | CL482.Contig1\_Mf\_liverA, Unigene36328\_Mf\_liverA, CL442.Contig5\_Mf\_liverA, CL442.Contig2\_Mf\_liverA, CL6039.Contig1\_Mf\_liverA, CL6039.Contig2\_Mf\_liverA, CL993.Contig2\_Mf\_liverA, Unigene33459\_Mf\_liverA, CL5978.Contig2\_Mf\_liverA, Unigene8033\_Mf\_liverA |
| extraorganismal space | CL482.Contig1\_Mf\_liverA, Unigene36328\_Mf\_liverA, CL442.Contig5\_Mf\_liverA, CL442.Contig2\_Mf\_liverA, CL6039.Contig1\_Mf\_liverA, CL6039.Contig2\_Mf\_liverA, CL993.Contig2\_Mf\_liverA, Unigene33459\_Mf\_liverA, CL5978.Contig2\_Mf\_liverA, Unigene8033\_Mf\_liverA |
| host cell | CL482.Contig1\_Mf\_liverA, Unigene36328\_Mf\_liverA, CL442.Contig5\_Mf\_liverA, CL442.Contig2\_Mf\_liverA, CL6039.Contig1\_Mf\_liverA, CL6039.Contig2\_Mf\_liverA, CL993.Contig2\_Mf\_liverA, Unigene33459\_Mf\_liverA, CL5978.Contig2\_Mf\_liverA, Unigene8033\_Mf\_liverA |
| other organism | CL482.Contig1\_Mf\_liverA, Unigene36328\_Mf\_liverA, CL442.Contig5\_Mf\_liverA, CL442.Contig2\_Mf\_liverA, CL6039.Contig1\_Mf\_liverA, CL6039.Contig2\_Mf\_liverA, CL993.Contig2\_Mf\_liverA, Unigene33459\_Mf\_liverA, CL5978.Contig2\_Mf\_liverA, Unigene8033\_Mf\_liverA |
| other organism cell | CL482.Contig1\_Mf\_liverA, Unigene36328\_Mf\_liverA, CL442.Contig5\_Mf\_liverA, CL442.Contig2\_Mf\_liverA, CL6039.Contig1\_Mf\_liverA, CL6039.Contig2\_Mf\_liverA, CL993.Contig2\_Mf\_liverA, Unigene33459\_Mf\_liverA, CL5978.Contig2\_Mf\_liverA, Unigene8033\_Mf\_liverA |
| other organism part | CL482.Contig1\_Mf\_liverA, Unigene36328\_Mf\_liverA, CL442.Contig5\_Mf\_liverA, CL442.Contig2\_Mf\_liverA, CL6039.Contig1\_Mf\_liverA, CL6039.Contig2\_Mf\_liverA, CL993.Contig2\_Mf\_liverA, Unigene33459\_Mf\_liverA, CL5978.Contig2\_Mf\_liverA, Unigene8033\_Mf\_liverA |
| lysosome | Unigene24471\_Mf\_liverA, Unigene25524\_Mf\_liverA, CL3835.Contig2\_Mf\_liverA, CL2855.Contig2\_Mf\_liverA, CL3669.Contig2\_Mf\_liverA, Unigene15529\_Mf\_liverA, CL4925.Contig1\_Mf\_liverA, Unigene5382\_Mf\_liverA, CL854.Contig1\_Mf\_liverA, Unigene8740\_Mf\_liverA, Unigene39886\_Mf\_liverA, Unigene21466\_Mf\_liverA, Unigene30878\_Mf\_liverA, CL3835.Contig1\_Mf\_liverA, Unigene37575\_Mf\_liverA, Unigene27081\_Mf\_liverA, Unigene36836\_Mf\_liverA, CL1736.Contig2\_Mf\_liverA, Unigene36328\_Mf\_liverA, CL3002.Contig1\_Mf\_liverA, Unigene37389\_Mf\_liverA, Unigene32332\_Mf\_liverA, Unigene15592\_Mf\_liverA, Unigene5745\_Mf\_liverA, Unigene8132\_Mf\_liverA, Unigene27082\_Mf\_liverA, Unigene5906\_Mf\_liverA, CL1493.Contig2\_Mf\_liverA, CL1493.Contig1\_Mf\_liverA, Unigene4681\_Mf\_liverA, CL442.Contig2\_Mf\_liverA, Unigene33632\_Mf\_liverA, CL2855.Contig1\_Mf\_liverA, Unigene30528\_Mf\_liverA |
| immunological synapse | Unigene22433\_Mf\_liverA, Unigene34746\_Mf\_liverA, Unigene22432\_Mf\_liverA, Unigene24112\_Mf\_liverA, Unigene26580\_Mf\_liverA, Unigene24111\_Mf\_liverA, Unigene39749\_Mf\_liverA, Unigene29008\_Mf\_liverA |
| organelle membrane | Unigene38015\_Mf\_liverA, NM\_009898, CL3835.Contig2\_Mf\_liverA, NM\_018815, CL523.Contig1\_Mf\_liverA, Unigene13153\_Mf\_liverA, Unigene34727\_Mf\_liverA, NM\_011082, Unigene29424\_Mf\_liverA, Unigene29008\_Mf\_liverA, CL482.Contig1\_Mf\_liverA, NM\_007820, Unigene39886\_Mf\_liverA, Unigene30878\_Mf\_liverA, Unigene34810\_Mf\_liverA, Unigene37880\_Mf\_liverA, CL5586.Contig1\_Mf\_liverA, Unigene40020\_Mf\_liverA, Unigene34341\_Mf\_liverA, Unigene37535\_Mf\_liverA, CL695.Contig1\_Mf\_liverA, CL2001.Contig1\_Mf\_liverA, NM\_145474, Unigene4686\_Mf\_liverA, Unigene34394\_Mf\_liverA, NM\_031165, NM\_153193, CL1493.Contig2\_Mf\_liverA, Unigene14508\_Mf\_liverA, Unigene5294\_Mf\_liverA, Unigene2745\_Mf\_liverA, Unigene8054\_Mf\_liverA, Unigene25462\_Mf\_liverA, Unigene5775\_Mf\_liverA, CL787.Contig1\_Mf\_liverA, Unigene17048\_Mf\_liverA, CL5807.Contig1\_Mf\_liverA, CL2855.Contig2\_Mf\_liverA, NM\_201360, NM\_010001, Unigene4922\_Mf\_liverA, Unigene5632\_Mf\_liverA, NM\_007811, NM\_001104531, Unigene139\_Mf\_liverA, CL3816.Contig1\_Mf\_liverA, Unigene36757\_Mf\_liverA, CL5631.Contig1\_Mf\_liverA, CL529.Contig2\_Mf\_liverA, Unigene5774\_Mf\_liverA, Unigene25398\_Mf\_liverA, CL4220.Contig2\_Mf\_liverA, Unigene27081\_Mf\_liverA, NM\_134156, CL5316.Contig1\_Mf\_liverA, Unigene37389\_Mf\_liverA, Unigene32332\_Mf\_liverA, Unigene7195\_Mf\_liverA, Unigene8132\_Mf\_liverA, Unigene34010\_Mf\_liverA, NM\_019717, Unigene27082\_Mf\_liverA, Unigene5712\_Mf\_liverA, Unigene1280\_Mf\_liverA, Unigene1205\_Mf\_liverA, CL442.Contig2\_Mf\_liverA, CL3166.Contig4\_Mf\_liverA, Unigene33632\_Mf\_liverA, CL2855.Contig1\_Mf\_liverA, Unigene2746\_Mf\_liverA, NM\_013821, Unigene40796\_Mf\_liverA, Unigene13233\_Mf\_liverA, NM\_001100182, NM\_009128, CL4925.Contig1\_Mf\_liverA, CL2240.Contig1\_Mf\_liverA, NM\_008293, Unigene7970\_Mf\_liverA, CL2797.Contig2\_Mf\_liverA, CL1125.Contig1\_Mf\_liverA, Unigene35237\_Mf\_liverA, Unigene112\_Mf\_liverA, Unigene15077\_Mf\_liverA, CL4600.Contig1\_Mf\_liverA, Unigene5693\_Mf\_liverA, NM\_010003, CL5796.Contig2\_Mf\_liverA, CL2478.Contig3\_Mf\_liverA, CL1988.Contig1\_Mf\_liverA, NM\_133838, Unigene36328\_Mf\_liverA, CL3002.Contig1\_Mf\_liverA, CL1988.Contig3\_Mf\_liverA, Unigene36514\_Mf\_liverA, CL1810.Contig1\_Mf\_liverA, CL4033.Contig1\_Mf\_liverA, CL4816.Contig2\_Mf\_liverA, Unigene4781\_Mf\_liverA, CL1988.Contig2\_Mf\_liverA, Unigene32695\_Mf\_liverA, NM\_001081148, Unigene5906\_Mf\_liverA, Unigene43357\_Mf\_liverA, Unigene28731\_Mf\_liverA, CL1493.Contig1\_Mf\_liverA, Unigene28499\_Mf\_liverA, Unigene10135\_Mf\_liverA, Unigene542\_Mf\_liverA, Unigene32294\_Mf\_liverA, CL114.Contig2\_Mf\_liverA, Unigene4909\_Mf\_liverA, Unigene24471\_Mf\_liverA, NM\_010162, Unigene15529\_Mf\_liverA, CL4220.Contig1\_Mf\_liverA, CL5307.Contig1\_Mf\_liverA, Unigene3377\_Mf\_liverA, NM\_019879, CL738.Contig2\_Mf\_liverA, CL3816.Contig2\_Mf\_liverA, CL4490.Contig2\_Mf\_liverA, NM\_007822, CL4816.Contig3\_Mf\_liverA, Unigene35609\_Mf\_liverA, NM\_025336, CL4007.Contig1\_Mf\_liverA, Unigene1221\_Mf\_liverA, Unigene23185\_Mf\_liverA, CL3835.Contig1\_Mf\_liverA, Unigene7048\_Mf\_liverA, Unigene35046\_Mf\_liverA, Unigene1292\_Mf\_liverA, CL3104.Contig1\_Mf\_liverA, Unigene32295\_Mf\_liverA, Unigene36593\_Mf\_liverA, Unigene5745\_Mf\_liverA, Unigene36699\_Mf\_liverA, NM\_001099634, CL5698.Contig1\_Mf\_liverA, Unigene4681\_Mf\_liverA, Unigene5886\_Mf\_liverA, CL336.Contig3\_Mf\_liverA |
| endomembrane system | Unigene26055\_Mf\_liverA, Unigene28142\_Mf\_liverA, NM\_009898, NM\_009128, CL4925.Contig1\_Mf\_liverA, CL2240.Contig1\_Mf\_liverA, NM\_018815, CL1575.Contig1\_Mf\_liverA, CL523.Contig1\_Mf\_liverA, NM\_080638, Unigene13153\_Mf\_liverA, CL2797.Contig2\_Mf\_liverA, Unigene7970\_Mf\_liverA, Unigene29424\_Mf\_liverA, Unigene26053\_Mf\_liverA, Unigene29008\_Mf\_liverA, CL482.Contig1\_Mf\_liverA, CL4162.Contig1\_Mf\_liverA, Unigene35237\_Mf\_liverA, Unigene112\_Mf\_liverA, CL4600.Contig1\_Mf\_liverA, NR\_004446, Unigene34810\_Mf\_liverA, Unigene37880\_Mf\_liverA, CL2478.Contig3\_Mf\_liverA, CL1988.Contig1\_Mf\_liverA, CL5586.Contig1\_Mf\_liverA, Unigene36328\_Mf\_liverA, Unigene40020\_Mf\_liverA, Unigene34341\_Mf\_liverA, CL1988.Contig3\_Mf\_liverA, Unigene37535\_Mf\_liverA, CL695.Contig1\_Mf\_liverA, Unigene36514\_Mf\_liverA, Unigene15703\_Mf\_liverA, CL4033.Contig1\_Mf\_liverA, CL4816.Contig2\_Mf\_liverA, CL2001.Contig1\_Mf\_liverA, Unigene31623\_Mf\_liverA, CL1988.Contig2\_Mf\_liverA, Unigene4686\_Mf\_liverA, Unigene34394\_Mf\_liverA, Unigene32695\_Mf\_liverA, NM\_031165, CL1493.Contig2\_Mf\_liverA, Unigene43357\_Mf\_liverA, Unigene28731\_Mf\_liverA, CL1493.Contig1\_Mf\_liverA, NM\_033444, Unigene28499\_Mf\_liverA, Unigene5294\_Mf\_liverA, Unigene8054\_Mf\_liverA, Unigene542\_Mf\_liverA, Unigene25462\_Mf\_liverA, Unigene10135\_Mf\_liverA, Unigene5775\_Mf\_liverA, CL787.Contig1\_Mf\_liverA, Unigene32294\_Mf\_liverA, CL114.Contig2\_Mf\_liverA, Unigene4909\_Mf\_liverA, CL5807.Contig1\_Mf\_liverA, NM\_010162, CL2855.Contig2\_Mf\_liverA, Unigene15529\_Mf\_liverA, CL4220.Contig1\_Mf\_liverA, CL5307.Contig1\_Mf\_liverA, Unigene3377\_Mf\_liverA, Unigene4922\_Mf\_liverA, Unigene5632\_Mf\_liverA, Unigene14270\_Mf\_liverA, CL3816.Contig2\_Mf\_liverA, CL738.Contig2\_Mf\_liverA, CL1263.Contig1\_Mf\_liverA, CL4490.Contig2\_Mf\_liverA, CL4816.Contig3\_Mf\_liverA, Unigene139\_Mf\_liverA, CL3816.Contig1\_Mf\_liverA, Unigene36757\_Mf\_liverA, Unigene35609\_Mf\_liverA, CL4007.Contig1\_Mf\_liverA, Unigene1221\_Mf\_liverA, CL529.Contig2\_Mf\_liverA, Unigene7048\_Mf\_liverA, Unigene23185\_Mf\_liverA, Unigene5774\_Mf\_liverA, CL4220.Contig2\_Mf\_liverA, Unigene35046\_Mf\_liverA, Unigene27081\_Mf\_liverA, Unigene550\_Mf\_liverA, NM\_134156, NM\_010391, Unigene37389\_Mf\_liverA, Unigene32295\_Mf\_liverA, Unigene36593\_Mf\_liverA, Unigene7195\_Mf\_liverA, Unigene8132\_Mf\_liverA, CL5254.Contig1\_Mf\_liverA, Unigene36699\_Mf\_liverA, Unigene34010\_Mf\_liverA, NM\_001099634, Unigene27082\_Mf\_liverA, Unigene5712\_Mf\_liverA, CL5698.Contig1\_Mf\_liverA, Unigene1280\_Mf\_liverA, Unigene1205\_Mf\_liverA, CL3166.Contig4\_Mf\_liverA, Unigene16891\_Mf\_liverA, CL2855.Contig1\_Mf\_liverA, Unigene5886\_Mf\_liverA, CL336.Contig3\_Mf\_liverA, Unigene40796\_Mf\_liverA |
| multivesicular body | Unigene27081\_Mf\_liverA, Unigene37245\_Mf\_liverA, Unigene27082\_Mf\_liverA, CL2855.Contig1\_Mf\_liverA, Unigene30878\_Mf\_liverA, Unigene8132\_Mf\_liverA, CL3002.Contig1\_Mf\_liverA, CL2855.Contig2\_Mf\_liverA, Unigene4681\_Mf\_liverA |
| membrane raft | Unigene27081\_Mf\_liverA, Unigene36889\_Mf\_liverA, Unigene32564\_Mf\_liverA, Unigene4720\_Mf\_liverA, CL1810.Contig1\_Mf\_liverA, Unigene21562\_Mf\_liverA, Unigene21561\_Mf\_liverA, CL887.Contig2\_Mf\_liverA, Unigene12889\_Mf\_liverA, Unigene31333\_Mf\_liverA, CL3816.Contig2\_Mf\_liverA, Unigene34866\_Mf\_liverA, Unigene27026\_Mf\_liverA, Unigene27082\_Mf\_liverA, Unigene35237\_Mf\_liverA, CL777.Contig8\_Mf\_liverA, CL5698.Contig1\_Mf\_liverA, CL3816.Contig1\_Mf\_liverA, Unigene29231\_Mf\_liverA, Unigene12519\_Mf\_liverA, Unigene14284\_Mf\_liverA, CL5631.Contig1\_Mf\_liverA, Unigene34867\_Mf\_liverA, Unigene13950\_Mf\_liverA |
| symbiont-containing vacuole membrane | CL482.Contig1\_Mf\_liverA, CL6039.Contig2\_Mf\_liverA, CL6039.Contig1\_Mf\_liverA, CL993.Contig2\_Mf\_liverA, Unigene33459\_Mf\_liverA, CL5978.Contig2\_Mf\_liverA, Unigene8033\_Mf\_liverA |
| receptor complex | CL4086.Contig1\_Mf\_liverA, Unigene40610\_Mf\_liverA, CL2855.Contig2\_Mf\_liverA, Unigene24112\_Mf\_liverA, Unigene6959\_Mf\_liverA, Unigene24111\_Mf\_liverA, Unigene19658\_Mf\_liverA, Unigene39252\_Mf\_liverA, Unigene38870\_Mf\_liverA, Unigene31333\_Mf\_liverA, Unigene15982\_Mf\_liverA, Unigene18340\_Mf\_liverA, Unigene29426\_Mf\_liverA, Unigene22433\_Mf\_liverA, Unigene843\_Mf\_liverA, Unigene29788\_Mf\_liverA, Unigene4983\_Mf\_liverA, Unigene37150\_Mf\_liverA, Unigene22432\_Mf\_liverA, Unigene14284\_Mf\_liverA, CL2855.Contig1\_Mf\_liverA, Unigene39749\_Mf\_liverA |
| apical plasma membrane | CL4220.Contig2\_Mf\_liverA, Unigene27081\_Mf\_liverA, Unigene4909\_Mf\_liverA, CL5586.Contig1\_Mf\_liverA, Unigene21562\_Mf\_liverA, Unigene28186\_Mf\_liverA, CL4033.Contig1\_Mf\_liverA, Unigene21561\_Mf\_liverA, Unigene26336\_Mf\_liverA, Unigene28873\_Mf\_liverA, Unigene29424\_Mf\_liverA, Unigene35816\_Mf\_liverA, Unigene30892\_Mf\_liverA, CL3519.Contig1\_Mf\_liverA, Unigene4686\_Mf\_liverA, Unigene27082\_Mf\_liverA, Unigene112\_Mf\_liverA, Unigene139\_Mf\_liverA, Unigene15583\_Mf\_liverA, Unigene4597\_Mf\_liverA, CL591.Contig1\_Mf\_liverA, Unigene35609\_Mf\_liverA, CL336.Contig3\_Mf\_liverA, CL4007.Contig1\_Mf\_liverA |
| apical part of cell | CL4220.Contig2\_Mf\_liverA, Unigene27081\_Mf\_liverA, Unigene4909\_Mf\_liverA, CL5586.Contig1\_Mf\_liverA, Unigene21562\_Mf\_liverA, Unigene28186\_Mf\_liverA, CL4033.Contig1\_Mf\_liverA, Unigene21561\_Mf\_liverA, Unigene26336\_Mf\_liverA, CL887.Contig2\_Mf\_liverA, Unigene28873\_Mf\_liverA, Unigene2195\_Mf\_liverA, Unigene29424\_Mf\_liverA, Unigene35816\_Mf\_liverA, Unigene30892\_Mf\_liverA, Unigene13593\_Mf\_liverA, CL3519.Contig1\_Mf\_liverA, Unigene4686\_Mf\_liverA, Unigene27082\_Mf\_liverA, Unigene112\_Mf\_liverA, Unigene139\_Mf\_liverA, Unigene15583\_Mf\_liverA, Unigene4597\_Mf\_liverA, CL591.Contig1\_Mf\_liverA, Unigene35609\_Mf\_liverA, CL336.Contig3\_Mf\_liverA, CL4007.Contig1\_Mf\_liverA, Unigene37575\_Mf\_liverA |
| membrane part | Unigene21684\_Mf\_liverA, NM\_176843, NM\_009898, NM\_001025208, Unigene22874\_Mf\_liverA, Unigene32891\_Mf\_liverA, Unigene24111\_Mf\_liverA, NM\_018815, Unigene28186\_Mf\_liverA, NM\_010378, Unigene34727\_Mf\_liverA, NM\_011082, Unigene29424\_Mf\_liverA, Unigene26053\_Mf\_liverA, NM\_028862, Unigene34852\_Mf\_liverA, CL3847.Contig1\_Mf\_liverA, Unigene39886\_Mf\_liverA, Unigene38689\_Mf\_liverA, Unigene1137\_Mf\_liverA, Unigene27294\_Mf\_liverA, NR\_004446, CL1555.Contig1\_Mf\_liverA, CL5586.Contig1\_Mf\_liverA, Unigene32564\_Mf\_liverA, Unigene34341\_Mf\_liverA, Unigene37535\_Mf\_liverA, CL695.Contig1\_Mf\_liverA, Unigene15703\_Mf\_liverA, Unigene35816\_Mf\_liverA, CL593.Contig2\_Mf\_liverA, CL2260.Contig1\_Mf\_liverA, NM\_010233, Unigene34394\_Mf\_liverA, Unigene31694\_Mf\_liverA, NM\_153193, Unigene5138\_Mf\_liverA, Unigene37150\_Mf\_liverA, Unigene34143\_Mf\_liverA, Unigene8054\_Mf\_liverA, Unigene17048\_Mf\_liverA, Unigene5552\_Mf\_liverA, CL5807.Contig1\_Mf\_liverA, Unigene13363\_Mf\_liverA, Unigene31958\_Mf\_liverA, Unigene26065\_Mf\_liverA, Unigene13230\_Mf\_liverA, CL2855.Contig2\_Mf\_liverA, Unigene18125\_Mf\_liverA, CL4293.Contig1\_Mf\_liverA, Unigene13097\_Mf\_liverA, Unigene25226\_Mf\_liverA, Unigene21561\_Mf\_liverA, Unigene5366\_Mf\_liverA, Unigene4922\_Mf\_liverA, NM\_153505, Unigene941\_Mf\_liverA, Unigene139\_Mf\_liverA, Unigene37420\_Mf\_liverA, Unigene37575\_Mf\_liverA, Unigene5774\_Mf\_liverA, Unigene25398\_Mf\_liverA, Unigene27547\_Mf\_liverA, NM\_134156, CL5316.Contig1\_Mf\_liverA, Unigene37389\_Mf\_liverA, Unigene4720\_Mf\_liverA, Unigene32332\_Mf\_liverA, Unigene14070\_Mf\_liverA, Unigene29960\_Mf\_liverA, CL4156.Contig1\_Mf\_liverA, Unigene27248\_Mf\_liverA, Unigene31852\_Mf\_liverA, Unigene1280\_Mf\_liverA, CL3166.Contig4\_Mf\_liverA, Unigene35698\_Mf\_liverA, CL275.Contig5\_Mf\_liverA, Unigene33632\_Mf\_liverA, Unigene15184\_Mf\_liverA, Unigene5169\_Mf\_liverA, NM\_010392, Unigene4363\_Mf\_liverA, Unigene40610\_Mf\_liverA, CL4925.Contig1\_Mf\_liverA, CL425.Contig1\_Mf\_liverA, Unigene32421\_Mf\_liverA, CL2797.Contig2\_Mf\_liverA, Unigene37243\_Mf\_liverA, Unigene27026\_Mf\_liverA, Unigene30587\_Mf\_liverA, Unigene39403\_Mf\_liverA, CL4600.Contig1\_Mf\_liverA, Unigene29231\_Mf\_liverA, Unigene5693\_Mf\_liverA, Unigene34867\_Mf\_liverA, CL2478.Contig3\_Mf\_liverA, Unigene26585\_Mf\_liverA, Unigene4938\_Mf\_liverA, CL422.Contig1\_Mf\_liverA, Unigene5292\_Mf\_liverA, CL1988.Contig3\_Mf\_liverA, CL1810.Contig1\_Mf\_liverA, Unigene5758\_Mf\_liverA, CL4033.Contig1\_Mf\_liverA, Unigene38870\_Mf\_liverA, Unigene12843\_Mf\_liverA, Unigene38199\_Mf\_liverA, Unigene4781\_Mf\_liverA, CL3519.Contig1\_Mf\_liverA, CL1988.Contig2\_Mf\_liverA, Unigene29426\_Mf\_liverA, Unigene13462\_Mf\_liverA, Unigene20371\_Mf\_liverA, Unigene28499\_Mf\_liverA, Unigene30585\_Mf\_liverA, CL5764.Contig1\_Mf\_liverA, Unigene542\_Mf\_liverA, Unigene4909\_Mf\_liverA, Unigene24471\_Mf\_liverA, Unigene33080\_Mf\_liverA, NM\_010162, CL4220.Contig1\_Mf\_liverA, Unigene30947\_Mf\_liverA, Unigene18126\_Mf\_liverA, NM\_007822, CL4490.Contig2\_Mf\_liverA, CL4816.Contig3\_Mf\_liverA, Unigene35090\_Mf\_liverA, Unigene39011\_Mf\_liverA, Unigene35609\_Mf\_liverA, NM\_145218, Unigene7048\_Mf\_liverA, Unigene23185\_Mf\_liverA, Unigene13950\_Mf\_liverA, Unigene35046\_Mf\_liverA, Unigene37245\_Mf\_liverA, Unigene20372\_Mf\_liverA, CL3104.Contig1\_Mf\_liverA, Unigene26580\_Mf\_liverA, Unigene36593\_Mf\_liverA, Unigene19658\_Mf\_liverA, Unigene26336\_Mf\_liverA, Unigene36699\_Mf\_liverA, Unigene18340\_Mf\_liverA, Unigene42803\_Mf\_liverA, Unigene4983\_Mf\_liverA, CL5698.Contig1\_Mf\_liverA, Unigene7387\_Mf\_liverA, CL591.Contig1\_Mf\_liverA, Unigene26055\_Mf\_liverA, NM\_016751, CL3835.Contig2\_Mf\_liverA, Unigene38831\_Mf\_liverA, Unigene6959\_Mf\_liverA, Unigene7682\_Mf\_liverA, Unigene24883\_Mf\_liverA, CL523.Contig1\_Mf\_liverA, Unigene13153\_Mf\_liverA, Unigene36849\_Mf\_liverA, Unigene29008\_Mf\_liverA, Unigene25055\_Mf\_liverA, CL482.Contig1\_Mf\_liverA, CL2339.Contig1\_Mf\_liverA, Unigene27593\_Mf\_liverA, Unigene22433\_Mf\_liverA, Unigene29788\_Mf\_liverA, Unigene36762\_Mf\_liverA, CL777.Contig8\_Mf\_liverA, Unigene37698\_Mf\_liverA, Unigene14816\_Mf\_liverA, Unigene30878\_Mf\_liverA, Unigene34810\_Mf\_liverA, Unigene24344\_Mf\_liverA, Unigene37880\_Mf\_liverA, Unigene18500\_Mf\_liverA, Unigene36889\_Mf\_liverA, Unigene41336\_Mf\_liverA, NM\_028279, Unigene24112\_Mf\_liverA, Unigene25426\_Mf\_liverA, CL2001.Contig1\_Mf\_liverA, Unigene38110\_Mf\_liverA, Unigene31333\_Mf\_liverA, Unigene30369\_Mf\_liverA, Unigene13593\_Mf\_liverA, Unigene4630\_Mf\_liverA, Unigene4686\_Mf\_liverA, Unigene843\_Mf\_liverA, CL3575.Contig1\_Mf\_liverA, Unigene22432\_Mf\_liverA, Unigene5294\_Mf\_liverA, Unigene25462\_Mf\_liverA, Unigene5775\_Mf\_liverA, CL787.Contig1\_Mf\_liverA, NM\_153795, Unigene23870\_Mf\_liverA, Unigene13143\_Mf\_liverA, Unigene5632\_Mf\_liverA, Unigene34746\_Mf\_liverA, Unigene24503\_Mf\_liverA, CL2142.Contig2\_Mf\_liverA, Unigene15583\_Mf\_liverA, CL3816.Contig1\_Mf\_liverA, CL5631.Contig1\_Mf\_liverA, NM\_153589, CL529.Contig2\_Mf\_liverA, Unigene30584\_Mf\_liverA, CL4220.Contig2\_Mf\_liverA, Unigene431\_Mf\_liverA, Unigene27081\_Mf\_liverA, NM\_001101488, NM\_010391, Unigene860\_Mf\_liverA, Unigene7195\_Mf\_liverA, NM\_019717, Unigene27082\_Mf\_liverA, Unigene5712\_Mf\_liverA, Unigene1205\_Mf\_liverA, Unigene37497\_Mf\_liverA, NM\_010380, CL2855.Contig1\_Mf\_liverA, Unigene33760\_Mf\_liverA, NM\_001025388, NM\_013821, Unigene40796\_Mf\_liverA, Unigene13233\_Mf\_liverA, Unigene72\_Mf\_liverA, CL4086.Contig1\_Mf\_liverA, NM\_009128, Unigene20167\_Mf\_liverA, Unigene15318\_Mf\_liverA, CL2240.Contig1\_Mf\_liverA, Unigene29823\_Mf\_liverA, NM\_008293, Unigene7970\_Mf\_liverA, NM\_009255, Unigene15982\_Mf\_liverA, Unigene35237\_Mf\_liverA, Unigene112\_Mf\_liverA, Unigene3752\_Mf\_liverA, Unigene591\_Mf\_liverA, Unigene38598\_Mf\_liverA, CL5796.Contig2\_Mf\_liverA, NM\_011170, Unigene30707\_Mf\_liverA, Unigene36543\_Mf\_liverA, CL1988.Contig1\_Mf\_liverA, Unigene36328\_Mf\_liverA, NM\_010141, CL3002.Contig1\_Mf\_liverA, Unigene6895\_Mf\_liverA, Unigene36514\_Mf\_liverA, CL887.Contig2\_Mf\_liverA, CL4816.Contig2\_Mf\_liverA, Unigene37139\_Mf\_liverA, Unigene25994\_Mf\_liverA, Unigene32695\_Mf\_liverA, Unigene5906\_Mf\_liverA, CL3207.Contig1\_Mf\_liverA, Unigene43357\_Mf\_liverA, Unigene28731\_Mf\_liverA, NM\_033444, Unigene4597\_Mf\_liverA, Unigene10135\_Mf\_liverA, Unigene32294\_Mf\_liverA, CL114.Contig2\_Mf\_liverA, Unigene7683\_Mf\_liverA, Unigene23255\_Mf\_liverA, Unigene21337\_Mf\_liverA, Unigene39252\_Mf\_liverA, CL5307.Contig1\_Mf\_liverA, Unigene3377\_Mf\_liverA, Unigene28873\_Mf\_liverA, Unigene8740\_Mf\_liverA, NM\_178405, Unigene14270\_Mf\_liverA, CL3816.Contig2\_Mf\_liverA, CL738.Contig2\_Mf\_liverA, CL1052.Contig1\_Mf\_liverA, Unigene9406\_Mf\_liverA, NM\_012030, CL5062.Contig2\_Mf\_liverA, Unigene39749\_Mf\_liverA, CL4007.Contig1\_Mf\_liverA, CL3835.Contig1\_Mf\_liverA, Unigene25052\_Mf\_liverA, Unigene677\_Mf\_liverA, Unigene32295\_Mf\_liverA, Unigene21562\_Mf\_liverA, Unigene4636\_Mf\_liverA, Unigene5745\_Mf\_liverA, Unigene21336\_Mf\_liverA, Unigene12889\_Mf\_liverA, Unigene35417\_Mf\_liverA, Unigene30892\_Mf\_liverA, NM\_001099634, Unigene33720\_Mf\_liverA, Unigene34866\_Mf\_liverA, Unigene14765\_Mf\_liverA, Unigene4681\_Mf\_liverA, Unigene12519\_Mf\_liverA, Unigene979\_Mf\_liverA, Unigene31080\_Mf\_liverA, Unigene37819\_Mf\_liverA, Unigene14284\_Mf\_liverA, Unigene5886\_Mf\_liverA, CL336.Contig3\_Mf\_liverA |
| proteinaceous extracellular matrix | Unigene32110\_Mf\_liverA, Unigene29399\_Mf\_liverA, NM\_177033, CL4040.Contig2\_Mf\_liverA, Unigene13106\_Mf\_liverA, Unigene17397\_Mf\_liverA, Unigene5606\_Mf\_liverA, Unigene37904\_Mf\_liverA, Unigene30493\_Mf\_liverA, Unigene28899\_Mf\_liverA, Unigene5382\_Mf\_liverA, Unigene13616\_Mf\_liverA, Unigene20512\_Mf\_liverA, Unigene38870\_Mf\_liverA, Unigene39252\_Mf\_liverA, Unigene8132\_Mf\_liverA, NM\_010233, Unigene30261\_Mf\_liverA, Unigene18181\_Mf\_liverA, Unigene30587\_Mf\_liverA, Unigene14582\_Mf\_liverA, Unigene32791\_Mf\_liverA, Unigene35090\_Mf\_liverA, CL4040.Contig1\_Mf\_liverA, Unigene22052\_Mf\_liverA, Unigene30585\_Mf\_liverA, Unigene8054\_Mf\_liverA, Unigene34810\_Mf\_liverA, Unigene30584\_Mf\_liverA |
| lytic vacuole | Unigene24471\_Mf\_liverA, Unigene25524\_Mf\_liverA, CL3835.Contig2\_Mf\_liverA, CL2855.Contig2\_Mf\_liverA, CL3669.Contig2\_Mf\_liverA, Unigene15529\_Mf\_liverA, CL4925.Contig1\_Mf\_liverA, Unigene5382\_Mf\_liverA, NM\_010378, CL854.Contig1\_Mf\_liverA, Unigene8740\_Mf\_liverA, Unigene39886\_Mf\_liverA, Unigene21466\_Mf\_liverA, Unigene30878\_Mf\_liverA, CL3835.Contig1\_Mf\_liverA, Unigene37575\_Mf\_liverA, Unigene27081\_Mf\_liverA, Unigene36836\_Mf\_liverA, CL1736.Contig2\_Mf\_liverA, Unigene36328\_Mf\_liverA, CL3002.Contig1\_Mf\_liverA, Unigene37389\_Mf\_liverA, Unigene32332\_Mf\_liverA, Unigene15592\_Mf\_liverA, Unigene5745\_Mf\_liverA, Unigene8132\_Mf\_liverA, Unigene27082\_Mf\_liverA, Unigene5906\_Mf\_liverA, CL1493.Contig2\_Mf\_liverA, CL1493.Contig1\_Mf\_liverA, Unigene4681\_Mf\_liverA, CL442.Contig2\_Mf\_liverA, Unigene33632\_Mf\_liverA, CL2855.Contig1\_Mf\_liverA, Unigene30528\_Mf\_liverA |
| perinuclear region of cytoplasm | Unigene26055\_Mf\_liverA, Unigene21684\_Mf\_liverA, CL4086.Contig1\_Mf\_liverA, Unigene31251\_Mf\_liverA, CL186.Contig3\_Mf\_liverA, CL4117.Contig1\_Mf\_liverA, Unigene26053\_Mf\_liverA, CL4162.Contig1\_Mf\_liverA, CL777.Contig8\_Mf\_liverA, CL2142.Contig2\_Mf\_liverA, Unigene39011\_Mf\_liverA, Unigene34867\_Mf\_liverA, Unigene5165\_Mf\_liverA, Unigene20372\_Mf\_liverA, Unigene30142\_Mf\_liverA, Unigene4720\_Mf\_liverA, Unigene27248\_Mf\_liverA, Unigene8132\_Mf\_liverA, Unigene24547\_Mf\_liverA, Unigene35816\_Mf\_liverA, Unigene2195\_Mf\_liverA, CL2001.Contig1\_Mf\_liverA, Unigene11\_Mf\_liverA, Unigene4686\_Mf\_liverA, Unigene34866\_Mf\_liverA, Unigene5138\_Mf\_liverA, Unigene37076\_Mf\_liverA, Unigene5712\_Mf\_liverA, Unigene20371\_Mf\_liverA, CL1352.Contig1\_Mf\_liverA, Unigene2746\_Mf\_liverA, Unigene2745\_Mf\_liverA, Unigene25462\_Mf\_liverA |
| extracellular matrix | CL1372.Contig1\_Mf\_liverA, Unigene32110\_Mf\_liverA, Unigene29399\_Mf\_liverA, NM\_177033, Unigene13106\_Mf\_liverA, Unigene28899\_Mf\_liverA, Unigene5382\_Mf\_liverA, Unigene13616\_Mf\_liverA, Unigene39252\_Mf\_liverA, NM\_145584, Unigene30261\_Mf\_liverA, Unigene18181\_Mf\_liverA, Unigene30587\_Mf\_liverA, Unigene14582\_Mf\_liverA, CL5459.Contig2\_Mf\_liverA, Unigene35090\_Mf\_liverA, CL1372.Contig2\_Mf\_liverA, CL4040.Contig1\_Mf\_liverA, Unigene22052\_Mf\_liverA, Unigene30584\_Mf\_liverA, Unigene34810\_Mf\_liverA, Unigene24344\_Mf\_liverA, CL4040.Contig2\_Mf\_liverA, Unigene17397\_Mf\_liverA, Unigene5606\_Mf\_liverA, Unigene425\_Mf\_liverA, Unigene37904\_Mf\_liverA, NM\_024474, Unigene30493\_Mf\_liverA, Unigene38870\_Mf\_liverA, Unigene20512\_Mf\_liverA, Unigene12843\_Mf\_liverA, Unigene8132\_Mf\_liverA, NM\_010233, Unigene32791\_Mf\_liverA, Unigene30585\_Mf\_liverA, Unigene8054\_Mf\_liverA |
| late endosome | Unigene27081\_Mf\_liverA, Unigene37245\_Mf\_liverA, Unigene24471\_Mf\_liverA, Unigene1292\_Mf\_liverA, Unigene36328\_Mf\_liverA, CL3002.Contig1\_Mf\_liverA, CL3835.Contig2\_Mf\_liverA, CL2855.Contig2\_Mf\_liverA, Unigene8132\_Mf\_liverA, Unigene27082\_Mf\_liverA, Unigene36698\_Mf\_liverA, CL1493.Contig2\_Mf\_liverA, CL1493.Contig1\_Mf\_liverA, Unigene4681\_Mf\_liverA, CL442.Contig2\_Mf\_liverA, CL2855.Contig1\_Mf\_liverA, Unigene5693\_Mf\_liverA, Unigene30878\_Mf\_liverA, CL3835.Contig1\_Mf\_liverA |
| lysosomal membrane | Unigene24471\_Mf\_liverA, Unigene5906\_Mf\_liverA, CL3002.Contig1\_Mf\_liverA, CL3835.Contig2\_Mf\_liverA, CL2855.Contig2\_Mf\_liverA, Unigene4681\_Mf\_liverA, CL4925.Contig1\_Mf\_liverA, Unigene32332\_Mf\_liverA, Unigene39886\_Mf\_liverA, Unigene33632\_Mf\_liverA, CL2855.Contig1\_Mf\_liverA, Unigene30878\_Mf\_liverA, CL3835.Contig1\_Mf\_liverA |
| complement component C1 complex | CL4040.Contig2\_Mf\_liverA, Unigene13106\_Mf\_liverA, CL4040.Contig1\_Mf\_liverA |
| secretory granule | CL44.Contig1\_Mf\_liverA, Unigene29399\_Mf\_liverA, Unigene4909\_Mf\_liverA, CL5807.Contig1\_Mf\_liverA, Unigene33080\_Mf\_liverA, NM\_134156, NM\_021278, Unigene15529\_Mf\_liverA, Unigene14809\_Mf\_liverA, Unigene28662\_Mf\_liverA, NM\_009255, Unigene29424\_Mf\_liverA, CL2251.Contig1\_Mf\_liverA, Unigene36673\_Mf\_liverA, Unigene4686\_Mf\_liverA, CL3816.Contig2\_Mf\_liverA, Unigene36669\_Mf\_liverA, Unigene34394\_Mf\_liverA, NM\_009776, Unigene30587\_Mf\_liverA, Unigene14810\_Mf\_liverA, CL3816.Contig1\_Mf\_liverA, Unigene36757\_Mf\_liverA, Unigene30585\_Mf\_liverA, Unigene8054\_Mf\_liverA, Unigene13950\_Mf\_liverA, Unigene34810\_Mf\_liverA, Unigene30584\_Mf\_liverA |
| proteasome core complex | Unigene35884\_Mf\_liverA, Unigene24471\_Mf\_liverA, CL3002.Contig1\_Mf\_liverA, CL3835.Contig2\_Mf\_liverA, CL3835.Contig1\_Mf\_liverA, Unigene37153\_Mf\_liverA |
| vacuolar part | Unigene24471\_Mf\_liverA, Unigene36328\_Mf\_liverA, Unigene5906\_Mf\_liverA, CL3002.Contig1\_Mf\_liverA, CL3835.Contig2\_Mf\_liverA, CL2855.Contig2\_Mf\_liverA, Unigene4681\_Mf\_liverA, CL4925.Contig1\_Mf\_liverA, Unigene32332\_Mf\_liverA, Unigene33632\_Mf\_liverA, Unigene39886\_Mf\_liverA, Unigene21466\_Mf\_liverA, Unigene5382\_Mf\_liverA, Unigene15592\_Mf\_liverA, CL2855.Contig1\_Mf\_liverA, Unigene30878\_Mf\_liverA, CL3835.Contig1\_Mf\_liverA, Unigene37575\_Mf\_liverA |
| vacuole | Unigene24471\_Mf\_liverA, Unigene25524\_Mf\_liverA, CL3835.Contig2\_Mf\_liverA, CL2855.Contig2\_Mf\_liverA, CL3669.Contig2\_Mf\_liverA, Unigene15529\_Mf\_liverA, CL4925.Contig1\_Mf\_liverA, Unigene5382\_Mf\_liverA, NM\_010378, CL854.Contig1\_Mf\_liverA, Unigene8740\_Mf\_liverA, Unigene39886\_Mf\_liverA, Unigene21466\_Mf\_liverA, Unigene30878\_Mf\_liverA, CL3835.Contig1\_Mf\_liverA, Unigene37575\_Mf\_liverA, Unigene27081\_Mf\_liverA, Unigene36836\_Mf\_liverA, CL1736.Contig2\_Mf\_liverA, Unigene36328\_Mf\_liverA, CL3002.Contig1\_Mf\_liverA, Unigene37389\_Mf\_liverA, Unigene32332\_Mf\_liverA, Unigene15592\_Mf\_liverA, Unigene5745\_Mf\_liverA, Unigene8132\_Mf\_liverA, Unigene27082\_Mf\_liverA, Unigene5906\_Mf\_liverA, CL1493.Contig2\_Mf\_liverA, CL1493.Contig1\_Mf\_liverA, Unigene4681\_Mf\_liverA, CL442.Contig2\_Mf\_liverA, Unigene33632\_Mf\_liverA, CL2855.Contig1\_Mf\_liverA, Unigene30528\_Mf\_liverA |
| cytoplasmic vesicle part | Unigene29399\_Mf\_liverA, Unigene27081\_Mf\_liverA, Unigene4909\_Mf\_liverA, CL5807.Contig1\_Mf\_liverA, Unigene36328\_Mf\_liverA, NM\_009898, NM\_134156, CL2855.Contig2\_Mf\_liverA, Unigene15529\_Mf\_liverA, CL4925.Contig1\_Mf\_liverA, Unigene14809\_Mf\_liverA, Unigene28662\_Mf\_liverA, Unigene29424\_Mf\_liverA, CL2251.Contig1\_Mf\_liverA, Unigene36673\_Mf\_liverA, Unigene4922\_Mf\_liverA, Unigene29008\_Mf\_liverA, Unigene5632\_Mf\_liverA, Unigene4686\_Mf\_liverA, CL3816.Contig2\_Mf\_liverA, Unigene34394\_Mf\_liverA, Unigene27082\_Mf\_liverA, NM\_031165, CL3816.Contig1\_Mf\_liverA, CL2855.Contig1\_Mf\_liverA, Unigene36757\_Mf\_liverA, Unigene8054\_Mf\_liverA, Unigene34810\_Mf\_liverA |
| MHC class I protein complex | CL275.Contig5\_Mf\_liverA, Unigene36543\_Mf\_liverA, Unigene4938\_Mf\_liverA, Unigene36849\_Mf\_liverA, Unigene13143\_Mf\_liverA |
| phagocytic vesicle | Unigene4686\_Mf\_liverA, Unigene14809\_Mf\_liverA, Unigene36328\_Mf\_liverA, Unigene8054\_Mf\_liverA, Unigene8740\_Mf\_liverA, Unigene29008\_Mf\_liverA, Unigene37389\_Mf\_liverA, Unigene34810\_Mf\_liverA |
| collagen | Unigene32110\_Mf\_liverA, CL4040.Contig2\_Mf\_liverA, Unigene13106\_Mf\_liverA, Unigene17397\_Mf\_liverA, Unigene5606\_Mf\_liverA, Unigene32791\_Mf\_liverA, Unigene35090\_Mf\_liverA, CL4040.Contig1\_Mf\_liverA, Unigene30493\_Mf\_liverA, Unigene22052\_Mf\_liverA |
| endocytic vesicle membrane | Unigene4686\_Mf\_liverA, Unigene34394\_Mf\_liverA, CL2855.Contig1\_Mf\_liverA, CL5807.Contig1\_Mf\_liverA, Unigene36328\_Mf\_liverA, Unigene8054\_Mf\_liverA, NM\_009898, CL2855.Contig2\_Mf\_liverA, Unigene29008\_Mf\_liverA, Unigene34810\_Mf\_liverA |
| varicosity | Unigene34866\_Mf\_liverA, Unigene34867\_Mf\_liverA, Unigene4720\_Mf\_liverA |
| mitochondrion | NM\_020559, NM\_010481, Unigene31199\_Mf\_liverA, CL3669.Contig2\_Mf\_liverA, CL425.Contig1\_Mf\_liverA, NM\_008293, Unigene36190\_Mf\_liverA, CL4117.Contig1\_Mf\_liverA, CL2797.Contig2\_Mf\_liverA, Unigene34727\_Mf\_liverA, Unigene14916\_Mf\_liverA, CL1125.Contig1\_Mf\_liverA, CL3725.Contig1\_Mf\_liverA, Unigene36034\_Mf\_liverA, Unigene12907\_Mf\_liverA, Unigene21466\_Mf\_liverA, Unigene5693\_Mf\_liverA, Unigene25292\_Mf\_liverA, Unigene40289\_Mf\_liverA, CL5796.Contig2\_Mf\_liverA, CL4757.Contig1\_Mf\_liverA, Unigene29876\_Mf\_liverA, Unigene38331\_Mf\_liverA, Unigene37999\_Mf\_liverA, CL1810.Contig1\_Mf\_liverA, CL887.Contig2\_Mf\_liverA, CL2001.Contig1\_Mf\_liverA, Unigene35816\_Mf\_liverA, Unigene4781\_Mf\_liverA, Unigene4686\_Mf\_liverA, NM\_153193, Unigene43357\_Mf\_liverA, Unigene14508\_Mf\_liverA, CL2791.Contig1\_Mf\_liverA, Unigene38104\_Mf\_liverA, Unigene18430\_Mf\_liverA, Unigene25462\_Mf\_liverA, Unigene36626\_Mf\_liverA, Unigene35476\_Mf\_liverA, CL3750.Contig1\_Mf\_liverA, CL114.Contig2\_Mf\_liverA, Unigene17048\_Mf\_liverA, CL3750.Contig2\_Mf\_liverA, Unigene3377\_Mf\_liverA, NM\_019879, CL2251.Contig1\_Mf\_liverA, Unigene39655\_Mf\_liverA, CL3816.Contig2\_Mf\_liverA, CL738.Contig2\_Mf\_liverA, Unigene37460\_Mf\_liverA, CL4490.Contig2\_Mf\_liverA, CL4048.Contig1\_Mf\_liverA, Unigene30261\_Mf\_liverA, Unigene14286\_Mf\_liverA, Unigene32515\_Mf\_liverA, Unigene24503\_Mf\_liverA, CL840.Contig1\_Mf\_liverA, CL3816.Contig1\_Mf\_liverA, Unigene30731\_Mf\_liverA, CL5631.Contig1\_Mf\_liverA, Unigene37575\_Mf\_liverA, Unigene23185\_Mf\_liverA, Unigene25398\_Mf\_liverA, Unigene28459\_Mf\_liverA, Unigene27547\_Mf\_liverA, Unigene27081\_Mf\_liverA, CL1736.Contig2\_Mf\_liverA, Unigene31198\_Mf\_liverA, CL5316.Contig1\_Mf\_liverA, CL3104.Contig1\_Mf\_liverA, Unigene2195\_Mf\_liverA, Unigene12889\_Mf\_liverA, CL532.Contig1\_Mf\_liverA, Unigene27082\_Mf\_liverA, Unigene6110\_Mf\_liverA, CL3166.Contig4\_Mf\_liverA, Unigene14171\_Mf\_liverA, CL3339.Contig1\_Mf\_liverA, NM\_013821, Unigene31206\_Mf\_liverA, Unigene13233\_Mf\_liverA |
| connexon complex | Unigene27081\_Mf\_liverA, Unigene27082\_Mf\_liverA, CL2260.Contig1\_Mf\_liverA |
| vacuolar membrane | Unigene24471\_Mf\_liverA, Unigene36328\_Mf\_liverA, Unigene5906\_Mf\_liverA, CL3002.Contig1\_Mf\_liverA, CL3835.Contig2\_Mf\_liverA, CL2855.Contig2\_Mf\_liverA, Unigene4681\_Mf\_liverA, CL4925.Contig1\_Mf\_liverA, Unigene32332\_Mf\_liverA, Unigene39886\_Mf\_liverA, Unigene33632\_Mf\_liverA, CL2855.Contig1\_Mf\_liverA, Unigene30878\_Mf\_liverA, CL3835.Contig1\_Mf\_liverA |
| Golgi apparatus | CL787.Contig1\_Mf\_liverA, Unigene32294\_Mf\_liverA, Unigene29399\_Mf\_liverA, Unigene25721\_Mf\_liverA, Unigene4363\_Mf\_liverA, CL2855.Contig2\_Mf\_liverA, CL3669.Contig2\_Mf\_liverA, Unigene15529\_Mf\_liverA, CL4925.Contig1\_Mf\_liverA, Unigene5382\_Mf\_liverA, Unigene28186\_Mf\_liverA, Unigene36190\_Mf\_liverA, CL4160.Contig2\_Mf\_liverA, Unigene8740\_Mf\_liverA, Unigene4922\_Mf\_liverA, Unigene5632\_Mf\_liverA, CL3816.Contig2\_Mf\_liverA, Unigene36669\_Mf\_liverA, CL482.Contig1\_Mf\_liverA, CL1263.Contig1\_Mf\_liverA, CL1052.Contig1\_Mf\_liverA, Unigene112\_Mf\_liverA, CL442.Contig5\_Mf\_liverA, CL4600.Contig1\_Mf\_liverA, CL777.Contig8\_Mf\_liverA, Unigene24503\_Mf\_liverA, CL3816.Contig1\_Mf\_liverA, Unigene21466\_Mf\_liverA, Unigene22052\_Mf\_liverA, Unigene1221\_Mf\_liverA, Unigene27081\_Mf\_liverA, Unigene37245\_Mf\_liverA, CL5586.Contig1\_Mf\_liverA, Unigene36836\_Mf\_liverA, Unigene36328\_Mf\_liverA, CL3002.Contig1\_Mf\_liverA, Unigene41336\_Mf\_liverA, Unigene37389\_Mf\_liverA, Unigene37535\_Mf\_liverA, NM\_010227, Unigene32295\_Mf\_liverA, CL4033.Contig1\_Mf\_liverA, Unigene37616\_Mf\_liverA, CL887.Contig2\_Mf\_liverA, Unigene31623\_Mf\_liverA, Unigene2195\_Mf\_liverA, Unigene34010\_Mf\_liverA, Unigene4686\_Mf\_liverA, Unigene11\_Mf\_liverA, Unigene27082\_Mf\_liverA, CL1493.Contig2\_Mf\_liverA, Unigene12153\_Mf\_liverA, CL442.Contig2\_Mf\_liverA, CL1493.Contig1\_Mf\_liverA, CL2855.Contig1\_Mf\_liverA, Unigene28499\_Mf\_liverA, Unigene25462\_Mf\_liverA |
| condensed nuclear chromosome outer kinetochore | CL5268.Contig1\_Mf\_liverA, Unigene45530\_Mf\_liverA |
| macrophage migration inhibitory factor receptor complex | CL2855.Contig1\_Mf\_liverA, CL2855.Contig2\_Mf\_liverA |
| NOS2-CD74 complex | CL2855.Contig1\_Mf\_liverA, CL2855.Contig2\_Mf\_liverA |
| endosome | Unigene24471\_Mf\_liverA, Unigene38015\_Mf\_liverA, CL3835.Contig2\_Mf\_liverA, CL2855.Contig2\_Mf\_liverA, NM\_011082, Unigene36698\_Mf\_liverA, Unigene15077\_Mf\_liverA, Unigene36034\_Mf\_liverA, Unigene24503\_Mf\_liverA, Unigene5693\_Mf\_liverA, Unigene25333\_Mf\_liverA, Unigene36757\_Mf\_liverA, Unigene30878\_Mf\_liverA, CL3835.Contig1\_Mf\_liverA, NR\_004446, Unigene37245\_Mf\_liverA, Unigene27081\_Mf\_liverA, NM\_133838, Unigene1292\_Mf\_liverA, Unigene36328\_Mf\_liverA, CL3002.Contig1\_Mf\_liverA, Unigene41336\_Mf\_liverA, NM\_010391, Unigene37389\_Mf\_liverA, Unigene15592\_Mf\_liverA, Unigene5745\_Mf\_liverA, Unigene8132\_Mf\_liverA, CL2001.Contig1\_Mf\_liverA, Unigene13379\_Mf\_liverA, Unigene27082\_Mf\_liverA, CL1493.Contig2\_Mf\_liverA, CL442.Contig2\_Mf\_liverA, CL1493.Contig1\_Mf\_liverA, Unigene4681\_Mf\_liverA, CL2855.Contig1\_Mf\_liverA, Unigene2746\_Mf\_liverA, Unigene2745\_Mf\_liverA |
| anchored to membrane | CL3575.Contig1\_Mf\_liverA, Unigene38831\_Mf\_liverA, Unigene13097\_Mf\_liverA, Unigene37698\_Mf\_liverA, CL4156.Contig1\_Mf\_liverA, Unigene21562\_Mf\_liverA, Unigene21561\_Mf\_liverA, Unigene5169\_Mf\_liverA, NM\_011170, Unigene31333\_Mf\_liverA |
| secretory granule membrane | CL3816.Contig2\_Mf\_liverA, Unigene4909\_Mf\_liverA, Unigene34394\_Mf\_liverA, CL5807.Contig1\_Mf\_liverA, NM\_134156, CL3816.Contig1\_Mf\_liverA, Unigene36757\_Mf\_liverA, Unigene8054\_Mf\_liverA, Unigene29424\_Mf\_liverA, Unigene34810\_Mf\_liverA |
| cytoplasmic vesicle membrane | Unigene27081\_Mf\_liverA, Unigene4909\_Mf\_liverA, CL5807.Contig1\_Mf\_liverA, Unigene36328\_Mf\_liverA, NM\_009898, NM\_134156, CL2855.Contig2\_Mf\_liverA, CL4925.Contig1\_Mf\_liverA, Unigene29424\_Mf\_liverA, Unigene4922\_Mf\_liverA, Unigene29008\_Mf\_liverA, Unigene5632\_Mf\_liverA, CL3816.Contig2\_Mf\_liverA, Unigene4686\_Mf\_liverA, Unigene34394\_Mf\_liverA, Unigene27082\_Mf\_liverA, NM\_031165, CL3816.Contig1\_Mf\_liverA, CL2855.Contig1\_Mf\_liverA, Unigene36757\_Mf\_liverA, Unigene8054\_Mf\_liverA, Unigene34810\_Mf\_liverA |
| intrinsic to membrane | NM\_016751, Unigene26055\_Mf\_liverA, Unigene21684\_Mf\_liverA, NM\_176843, CL3835.Contig2\_Mf\_liverA, Unigene38831\_Mf\_liverA, NM\_001025208, Unigene22874\_Mf\_liverA, Unigene32891\_Mf\_liverA, Unigene6959\_Mf\_liverA, Unigene7682\_Mf\_liverA, Unigene24111\_Mf\_liverA, NM\_018815, Unigene28186\_Mf\_liverA, Unigene24883\_Mf\_liverA, NM\_010378, NM\_011082, Unigene36849\_Mf\_liverA, Unigene26053\_Mf\_liverA, Unigene25055\_Mf\_liverA, Unigene27593\_Mf\_liverA, CL2339.Contig1\_Mf\_liverA, Unigene22433\_Mf\_liverA, Unigene29788\_Mf\_liverA, Unigene36762\_Mf\_liverA, NM\_028862, Unigene34852\_Mf\_liverA, CL3847.Contig1\_Mf\_liverA, Unigene14816\_Mf\_liverA, Unigene39886\_Mf\_liverA, Unigene37698\_Mf\_liverA, Unigene30878\_Mf\_liverA, Unigene27294\_Mf\_liverA, NR\_004446, Unigene24344\_Mf\_liverA, Unigene37880\_Mf\_liverA, CL1555.Contig1\_Mf\_liverA, Unigene18500\_Mf\_liverA, CL5586.Contig1\_Mf\_liverA, Unigene36889\_Mf\_liverA, Unigene32564\_Mf\_liverA, NM\_028279, Unigene24112\_Mf\_liverA, CL695.Contig1\_Mf\_liverA, Unigene15703\_Mf\_liverA, Unigene25426\_Mf\_liverA, CL2001.Contig1\_Mf\_liverA, Unigene31333\_Mf\_liverA, CL593.Contig2\_Mf\_liverA, CL2260.Contig1\_Mf\_liverA, Unigene13593\_Mf\_liverA, Unigene30369\_Mf\_liverA, Unigene4686\_Mf\_liverA, Unigene34394\_Mf\_liverA, Unigene31694\_Mf\_liverA, Unigene843\_Mf\_liverA, NM\_153193, CL3575.Contig1\_Mf\_liverA, Unigene37150\_Mf\_liverA, Unigene22432\_Mf\_liverA, Unigene34143\_Mf\_liverA, Unigene5294\_Mf\_liverA, Unigene8054\_Mf\_liverA, Unigene25462\_Mf\_liverA, CL787.Contig1\_Mf\_liverA, Unigene17048\_Mf\_liverA, Unigene5552\_Mf\_liverA, CL5807.Contig1\_Mf\_liverA, Unigene31958\_Mf\_liverA, Unigene13363\_Mf\_liverA, Unigene26065\_Mf\_liverA, Unigene13230\_Mf\_liverA, CL2855.Contig2\_Mf\_liverA, Unigene18125\_Mf\_liverA, CL4293.Contig1\_Mf\_liverA, Unigene13097\_Mf\_liverA, Unigene25226\_Mf\_liverA, Unigene5366\_Mf\_liverA, Unigene21561\_Mf\_liverA, Unigene4922\_Mf\_liverA, Unigene5632\_Mf\_liverA, NM\_153505, Unigene941\_Mf\_liverA, CL2142.Contig2\_Mf\_liverA, Unigene24503\_Mf\_liverA, Unigene15583\_Mf\_liverA, CL3816.Contig1\_Mf\_liverA, Unigene37420\_Mf\_liverA, NM\_153589, CL529.Contig2\_Mf\_liverA, Unigene37575\_Mf\_liverA, CL4220.Contig2\_Mf\_liverA, Unigene25398\_Mf\_liverA, Unigene27081\_Mf\_liverA, Unigene27547\_Mf\_liverA, NM\_001101488, CL5316.Contig1\_Mf\_liverA, NM\_010391, Unigene37389\_Mf\_liverA, Unigene32332\_Mf\_liverA, Unigene14070\_Mf\_liverA, Unigene860\_Mf\_liverA, Unigene29960\_Mf\_liverA, CL4156.Contig1\_Mf\_liverA, Unigene27248\_Mf\_liverA, NM\_019717, Unigene27082\_Mf\_liverA, Unigene5712\_Mf\_liverA, Unigene37497\_Mf\_liverA, Unigene35698\_Mf\_liverA, NM\_010380, CL275.Contig5\_Mf\_liverA, Unigene33632\_Mf\_liverA, Unigene15184\_Mf\_liverA, CL2855.Contig1\_Mf\_liverA, Unigene33760\_Mf\_liverA, Unigene5169\_Mf\_liverA, NM\_013821, Unigene40796\_Mf\_liverA, Unigene72\_Mf\_liverA, Unigene13233\_Mf\_liverA, NM\_010392, Unigene4363\_Mf\_liverA, NM\_009128, Unigene40610\_Mf\_liverA, Unigene20167\_Mf\_liverA, Unigene15318\_Mf\_liverA, Unigene29823\_Mf\_liverA, CL425.Contig1\_Mf\_liverA, Unigene32421\_Mf\_liverA, NM\_008293, CL2797.Contig2\_Mf\_liverA, Unigene7970\_Mf\_liverA, Unigene15982\_Mf\_liverA, Unigene27026\_Mf\_liverA, Unigene112\_Mf\_liverA, Unigene39403\_Mf\_liverA, Unigene3752\_Mf\_liverA, CL4600.Contig1\_Mf\_liverA, Unigene38598\_Mf\_liverA, Unigene29231\_Mf\_liverA, CL5796.Contig2\_Mf\_liverA, NM\_011170, Unigene26585\_Mf\_liverA, Unigene36543\_Mf\_liverA, CL1988.Contig1\_Mf\_liverA, Unigene4938\_Mf\_liverA, CL422.Contig1\_Mf\_liverA, Unigene5292\_Mf\_liverA, NM\_010141, CL3002.Contig1\_Mf\_liverA, CL1988.Contig3\_Mf\_liverA, Unigene6895\_Mf\_liverA, Unigene5758\_Mf\_liverA, CL4033.Contig1\_Mf\_liverA, Unigene38870\_Mf\_liverA, Unigene12843\_Mf\_liverA, Unigene38199\_Mf\_liverA, CL887.Contig2\_Mf\_liverA, Unigene4781\_Mf\_liverA, Unigene37139\_Mf\_liverA, Unigene25994\_Mf\_liverA, CL3519.Contig1\_Mf\_liverA, CL1988.Contig2\_Mf\_liverA, Unigene29426\_Mf\_liverA, Unigene32695\_Mf\_liverA, Unigene13462\_Mf\_liverA, Unigene5906\_Mf\_liverA, Unigene20371\_Mf\_liverA, Unigene28731\_Mf\_liverA, Unigene4597\_Mf\_liverA, NM\_033444, Unigene28499\_Mf\_liverA, CL5764.Contig1\_Mf\_liverA, Unigene10135\_Mf\_liverA, Unigene4909\_Mf\_liverA, Unigene24471\_Mf\_liverA, Unigene33080\_Mf\_liverA, Unigene7683\_Mf\_liverA, NM\_010162, CL4220.Contig1\_Mf\_liverA, Unigene23255\_Mf\_liverA, Unigene21337\_Mf\_liverA, Unigene39252\_Mf\_liverA, Unigene30947\_Mf\_liverA, CL5307.Contig1\_Mf\_liverA, Unigene3377\_Mf\_liverA, Unigene28873\_Mf\_liverA, Unigene18126\_Mf\_liverA, Unigene8740\_Mf\_liverA, NM\_178405, CL738.Contig2\_Mf\_liverA, CL3816.Contig2\_Mf\_liverA, Unigene14270\_Mf\_liverA, NM\_007822, CL4490.Contig2\_Mf\_liverA, Unigene35090\_Mf\_liverA, Unigene9406\_Mf\_liverA, Unigene39011\_Mf\_liverA, CL5062.Contig2\_Mf\_liverA, Unigene39749\_Mf\_liverA, Unigene35609\_Mf\_liverA, NM\_145218, CL4007.Contig1\_Mf\_liverA, Unigene25052\_Mf\_liverA, CL3835.Contig1\_Mf\_liverA, Unigene7048\_Mf\_liverA, Unigene23185\_Mf\_liverA, Unigene13950\_Mf\_liverA, Unigene35046\_Mf\_liverA, Unigene37245\_Mf\_liverA, Unigene20372\_Mf\_liverA, CL3104.Contig1\_Mf\_liverA, Unigene26580\_Mf\_liverA, Unigene677\_Mf\_liverA, Unigene36593\_Mf\_liverA, Unigene21562\_Mf\_liverA, Unigene19658\_Mf\_liverA, Unigene26336\_Mf\_liverA, Unigene5745\_Mf\_liverA, Unigene21336\_Mf\_liverA, Unigene36699\_Mf\_liverA, Unigene35417\_Mf\_liverA, Unigene18340\_Mf\_liverA, NM\_001099634, Unigene33720\_Mf\_liverA, Unigene34866\_Mf\_liverA, Unigene14765\_Mf\_liverA, Unigene4983\_Mf\_liverA, CL5698.Contig1\_Mf\_liverA, Unigene4681\_Mf\_liverA, Unigene7387\_Mf\_liverA, Unigene979\_Mf\_liverA, Unigene31080\_Mf\_liverA, Unigene37819\_Mf\_liverA, Unigene14284\_Mf\_liverA, Unigene5886\_Mf\_liverA |
| mitochondrial intermembrane space | Unigene35476\_Mf\_liverA, CL738.Contig2\_Mf\_liverA, CL4490.Contig2\_Mf\_liverA, CL2797.Contig2\_Mf\_liverA, Unigene3377\_Mf\_liverA, Unigene23185\_Mf\_liverA, CL5796.Contig2\_Mf\_liverA |
| endocytic vesicle | Unigene4686\_Mf\_liverA, Unigene34394\_Mf\_liverA, CL5807.Contig1\_Mf\_liverA, Unigene36328\_Mf\_liverA, NM\_009898, CL2855.Contig2\_Mf\_liverA, Unigene37389\_Mf\_liverA, Unigene14809\_Mf\_liverA, CL2855.Contig1\_Mf\_liverA, Unigene8054\_Mf\_liverA, Unigene8740\_Mf\_liverA, Unigene29008\_Mf\_liverA, Unigene34810\_Mf\_liverA |
| platelet alpha granule | Unigene29399\_Mf\_liverA, Unigene4909\_Mf\_liverA, NM\_009776, NM\_134156, NM\_021278, Unigene15529\_Mf\_liverA, Unigene28662\_Mf\_liverA, CL2251.Contig1\_Mf\_liverA, Unigene36673\_Mf\_liverA |
| Weibel-Palade body | Unigene30587\_Mf\_liverA, Unigene30585\_Mf\_liverA, Unigene30584\_Mf\_liverA |
| phagocytic vesicle membrane | Unigene4686\_Mf\_liverA, Unigene36328\_Mf\_liverA, Unigene8054\_Mf\_liverA, Unigene29008\_Mf\_liverA, Unigene34810\_Mf\_liverA |
| cell cortex | Unigene30707\_Mf\_liverA, Unigene28822\_Mf\_liverA, Unigene28899\_Mf\_liverA, Unigene39252\_Mf\_liverA, Unigene38870\_Mf\_liverA, CL848.Contig2\_Mf\_liverA, Unigene29308\_Mf\_liverA, Unigene4922\_Mf\_liverA, Unigene29008\_Mf\_liverA, CL777.Contig8\_Mf\_liverA, Unigene32515\_Mf\_liverA, Unigene25046\_Mf\_liverA, Unigene5693\_Mf\_liverA, CL4701.Contig1\_Mf\_liverA, Unigene37575\_Mf\_liverA, Unigene24252\_Mf\_liverA |
| high-density lipoprotein particle | Unigene36698\_Mf\_liverA, Unigene9081\_Mf\_liverA, Unigene30815\_Mf\_liverA, Unigene30814\_Mf\_liverA, Unigene24157\_Mf\_liverA |
| lysosomal lumen | Unigene5382\_Mf\_liverA, Unigene21466\_Mf\_liverA, CL2855.Contig1\_Mf\_liverA, Unigene15592\_Mf\_liverA, CL2855.Contig2\_Mf\_liverA, Unigene37575\_Mf\_liverA |
| organelle envelope lumen | Unigene35476\_Mf\_liverA, CL738.Contig2\_Mf\_liverA, CL4490.Contig2\_Mf\_liverA, CL2797.Contig2\_Mf\_liverA, Unigene3377\_Mf\_liverA, Unigene23185\_Mf\_liverA, CL5796.Contig2\_Mf\_liverA |
| vacuolar lumen | Unigene5382\_Mf\_liverA, Unigene21466\_Mf\_liverA, CL2855.Contig1\_Mf\_liverA, Unigene15592\_Mf\_liverA, CL2855.Contig2\_Mf\_liverA, Unigene37575\_Mf\_liverA |
| extracellular matrix part | Unigene32110\_Mf\_liverA, CL4040.Contig2\_Mf\_liverA, Unigene13106\_Mf\_liverA, Unigene17397\_Mf\_liverA, Unigene5606\_Mf\_liverA, Unigene14582\_Mf\_liverA, CL5459.Contig2\_Mf\_liverA, Unigene32791\_Mf\_liverA, Unigene35090\_Mf\_liverA, CL4040.Contig1\_Mf\_liverA, Unigene30493\_Mf\_liverA, Unigene39252\_Mf\_liverA, Unigene38870\_Mf\_liverA, Unigene22052\_Mf\_liverA, Unigene8132\_Mf\_liverA |
| dendritic shaft | Unigene18500\_Mf\_liverA, Unigene31251\_Mf\_liverA, Unigene24883\_Mf\_liverA, Unigene24547\_Mf\_liverA, Unigene13593\_Mf\_liverA |
| vesicle | Unigene29399\_Mf\_liverA, NM\_009898, CL4925.Contig1\_Mf\_liverA, Unigene6959\_Mf\_liverA, Unigene15318\_Mf\_liverA, Unigene36190\_Mf\_liverA, NM\_009255, NM\_011082, Unigene29424\_Mf\_liverA, Unigene29008\_Mf\_liverA, Unigene30587\_Mf\_liverA, Unigene36762\_Mf\_liverA, Unigene34810\_Mf\_liverA, CL44.Contig1\_Mf\_liverA, CL1555.Contig1\_Mf\_liverA, Unigene36328\_Mf\_liverA, Unigene40020\_Mf\_liverA, Unigene41336\_Mf\_liverA, CL6039.Contig2\_Mf\_liverA, Unigene28662\_Mf\_liverA, CL2001.Contig1\_Mf\_liverA, Unigene8033\_Mf\_liverA, Unigene4686\_Mf\_liverA, Unigene34394\_Mf\_liverA, NM\_009776, NM\_031165, CL1493.Contig2\_Mf\_liverA, Unigene14810\_Mf\_liverA, CL1493.Contig1\_Mf\_liverA, Unigene28499\_Mf\_liverA, Unigene30585\_Mf\_liverA, Unigene8054\_Mf\_liverA, Unigene4909\_Mf\_liverA, CL5807.Contig1\_Mf\_liverA, Unigene13363\_Mf\_liverA, Unigene33080\_Mf\_liverA, Unigene15529\_Mf\_liverA, CL2855.Contig2\_Mf\_liverA, NM\_021278, Unigene33459\_Mf\_liverA, CL2251.Contig1\_Mf\_liverA, Unigene8740\_Mf\_liverA, CL5978.Contig2\_Mf\_liverA, Unigene5632\_Mf\_liverA, Unigene4922\_Mf\_liverA, CL3816.Contig2\_Mf\_liverA, CL1052.Contig1\_Mf\_liverA, Unigene36669\_Mf\_liverA, CL3816.Contig1\_Mf\_liverA, CL993.Contig2\_Mf\_liverA, Unigene36757\_Mf\_liverA, Unigene30584\_Mf\_liverA, Unigene13950\_Mf\_liverA, Unigene27081\_Mf\_liverA, NM\_134156, Unigene37389\_Mf\_liverA, Unigene14809\_Mf\_liverA, Unigene8132\_Mf\_liverA, Unigene2195\_Mf\_liverA, Unigene36673\_Mf\_liverA, NM\_001099634, Unigene27082\_Mf\_liverA, CL6039.Contig1\_Mf\_liverA, Unigene33632\_Mf\_liverA, Unigene16891\_Mf\_liverA, Unigene37819\_Mf\_liverA, CL2855.Contig1\_Mf\_liverA |
| actin cytoskeleton | Unigene30707\_Mf\_liverA, Unigene5639\_Mf\_liverA, NM\_153795, NM\_009898, Unigene25721\_Mf\_liverA, Unigene28822\_Mf\_liverA, Unigene40020\_Mf\_liverA, NM\_134156, NM\_010227, NM\_177093, NM\_009609, Unigene26309\_Mf\_liverA, CL848.Contig2\_Mf\_liverA, Unigene29308\_Mf\_liverA, Unigene31623\_Mf\_liverA, Unigene2195\_Mf\_liverA, CL2251.Contig1\_Mf\_liverA, Unigene29008\_Mf\_liverA, Unigene14637\_Mf\_liverA, Unigene31852\_Mf\_liverA, Unigene5138\_Mf\_liverA, CL5698.Contig1\_Mf\_liverA, Unigene24477\_Mf\_liverA, Unigene25046\_Mf\_liverA, CL5576.Contig1\_Mf\_liverA, CL591.Contig1\_Mf\_liverA, Unigene28499\_Mf\_liverA, Unigene4556\_Mf\_liverA |
| vesicle membrane | Unigene27081\_Mf\_liverA, Unigene4909\_Mf\_liverA, CL5807.Contig1\_Mf\_liverA, Unigene36328\_Mf\_liverA, NM\_009898, Unigene40020\_Mf\_liverA, NM\_134156, CL2855.Contig2\_Mf\_liverA, CL4925.Contig1\_Mf\_liverA, Unigene29424\_Mf\_liverA, Unigene4922\_Mf\_liverA, Unigene29008\_Mf\_liverA, Unigene5632\_Mf\_liverA, Unigene4686\_Mf\_liverA, CL3816.Contig2\_Mf\_liverA, NM\_001099634, Unigene34394\_Mf\_liverA, Unigene27082\_Mf\_liverA, NM\_031165, CL3816.Contig1\_Mf\_liverA, CL2855.Contig1\_Mf\_liverA, Unigene36757\_Mf\_liverA, Unigene8054\_Mf\_liverA, Unigene34810\_Mf\_liverA |
| endosomal part | NM\_133838, Unigene24471\_Mf\_liverA, Unigene38015\_Mf\_liverA, Unigene1292\_Mf\_liverA, CL3002.Contig1\_Mf\_liverA, CL3835.Contig2\_Mf\_liverA, Unigene15592\_Mf\_liverA, CL2001.Contig1\_Mf\_liverA, NM\_011082, Unigene15077\_Mf\_liverA, Unigene4681\_Mf\_liverA, Unigene5693\_Mf\_liverA, Unigene30878\_Mf\_liverA, Unigene2746\_Mf\_liverA, Unigene2745\_Mf\_liverA, CL3835.Contig1\_Mf\_liverA |
| intercellular canaliculus | CL5586.Contig1\_Mf\_liverA, CL4033.Contig1\_Mf\_liverA, Unigene112\_Mf\_liverA |
| actin filament | Unigene5138\_Mf\_liverA, NM\_009898, CL5698.Contig1\_Mf\_liverA, Unigene28822\_Mf\_liverA, NM\_009609, NM\_177093, Unigene25046\_Mf\_liverA, Unigene4556\_Mf\_liverA, Unigene29308\_Mf\_liverA, Unigene29008\_Mf\_liverA |
| anchored to external side of plasma membrane | CL4156.Contig1\_Mf\_liverA, Unigene21562\_Mf\_liverA, Unigene21561\_Mf\_liverA |
| cytoplasmic vesicle | Unigene29399\_Mf\_liverA, Unigene4909\_Mf\_liverA, CL5807.Contig1\_Mf\_liverA, NM\_009898, Unigene33080\_Mf\_liverA, Unigene13363\_Mf\_liverA, NM\_021278, CL2855.Contig2\_Mf\_liverA, Unigene15529\_Mf\_liverA, CL4925.Contig1\_Mf\_liverA, Unigene6959\_Mf\_liverA, Unigene33459\_Mf\_liverA, Unigene36190\_Mf\_liverA, NM\_009255, NM\_011082, Unigene29424\_Mf\_liverA, CL5978.Contig2\_Mf\_liverA, Unigene8740\_Mf\_liverA, CL2251.Contig1\_Mf\_liverA, Unigene4922\_Mf\_liverA, Unigene29008\_Mf\_liverA, Unigene5632\_Mf\_liverA, CL3816.Contig2\_Mf\_liverA, Unigene36669\_Mf\_liverA, CL1052.Contig1\_Mf\_liverA, Unigene30587\_Mf\_liverA, Unigene36762\_Mf\_liverA, CL3816.Contig1\_Mf\_liverA, CL993.Contig2\_Mf\_liverA, Unigene36757\_Mf\_liverA, Unigene13950\_Mf\_liverA, Unigene30584\_Mf\_liverA, Unigene34810\_Mf\_liverA, CL44.Contig1\_Mf\_liverA, Unigene27081\_Mf\_liverA, Unigene36328\_Mf\_liverA, NM\_134156, Unigene41336\_Mf\_liverA, Unigene37389\_Mf\_liverA, Unigene14809\_Mf\_liverA, CL6039.Contig2\_Mf\_liverA, Unigene28662\_Mf\_liverA, CL2001.Contig1\_Mf\_liverA, Unigene36673\_Mf\_liverA, Unigene8033\_Mf\_liverA, Unigene4686\_Mf\_liverA, Unigene34394\_Mf\_liverA, NM\_009776, Unigene27082\_Mf\_liverA, NM\_031165, CL1493.Contig2\_Mf\_liverA, Unigene14810\_Mf\_liverA, CL1493.Contig1\_Mf\_liverA, Unigene33632\_Mf\_liverA, CL6039.Contig1\_Mf\_liverA, Unigene37819\_Mf\_liverA, CL2855.Contig1\_Mf\_liverA, Unigene28499\_Mf\_liverA, Unigene30585\_Mf\_liverA, Unigene8054\_Mf\_liverA |
| proteasome complex | CL4141.Contig1\_Mf\_liverA, Unigene35884\_Mf\_liverA, Unigene24471\_Mf\_liverA, CL3002.Contig1\_Mf\_liverA, CL3835.Contig2\_Mf\_liverA, CL3835.Contig1\_Mf\_liverA, Unigene37153\_Mf\_liverA |
| late endosome membrane | Unigene24471\_Mf\_liverA, Unigene5693\_Mf\_liverA, Unigene1292\_Mf\_liverA, Unigene30878\_Mf\_liverA, CL3002.Contig1\_Mf\_liverA, CL3835.Contig2\_Mf\_liverA, CL3835.Contig1\_Mf\_liverA |
| lamellipodium | Unigene30707\_Mf\_liverA, Unigene21684\_Mf\_liverA, Unigene31852\_Mf\_liverA, Unigene5138\_Mf\_liverA, Unigene27422\_Mf\_liverA, Unigene39011\_Mf\_liverA, CL1352.Contig1\_Mf\_liverA, Unigene5693\_Mf\_liverA, Unigene24252\_Mf\_liverA, Unigene29008\_Mf\_liverA |
| cytoplasmic membrane-bounded vesicle lumen | Unigene29399\_Mf\_liverA, Unigene14809\_Mf\_liverA, Unigene28662\_Mf\_liverA, CL2251.Contig1\_Mf\_liverA, Unigene15529\_Mf\_liverA, Unigene36673\_Mf\_liverA |
| Ndc80 complex | CL5268.Contig1\_Mf\_liverA, Unigene45530\_Mf\_liverA |
| chromaffin granule membrane | Unigene34394\_Mf\_liverA, CL5807.Contig1\_Mf\_liverA |
| membrane-bounded vesicle | Unigene29399\_Mf\_liverA, Unigene4909\_Mf\_liverA, CL5807.Contig1\_Mf\_liverA, NM\_009898, Unigene33080\_Mf\_liverA, Unigene13363\_Mf\_liverA, NM\_021278, CL2855.Contig2\_Mf\_liverA, Unigene15529\_Mf\_liverA, CL4925.Contig1\_Mf\_liverA, Unigene36190\_Mf\_liverA, NM\_009255, NM\_011082, Unigene29424\_Mf\_liverA, Unigene8740\_Mf\_liverA, CL2251.Contig1\_Mf\_liverA, Unigene4922\_Mf\_liverA, Unigene29008\_Mf\_liverA, Unigene5632\_Mf\_liverA, CL3816.Contig2\_Mf\_liverA, Unigene36669\_Mf\_liverA, CL1052.Contig1\_Mf\_liverA, Unigene30587\_Mf\_liverA, Unigene36762\_Mf\_liverA, CL3816.Contig1\_Mf\_liverA, Unigene36757\_Mf\_liverA, Unigene13950\_Mf\_liverA, Unigene30584\_Mf\_liverA, Unigene34810\_Mf\_liverA, CL44.Contig1\_Mf\_liverA, Unigene27081\_Mf\_liverA, CL1555.Contig1\_Mf\_liverA, Unigene36328\_Mf\_liverA, NM\_134156, Unigene40020\_Mf\_liverA, Unigene41336\_Mf\_liverA, Unigene37389\_Mf\_liverA, Unigene14809\_Mf\_liverA, Unigene28662\_Mf\_liverA, CL2001.Contig1\_Mf\_liverA, Unigene2195\_Mf\_liverA, Unigene36673\_Mf\_liverA, Unigene4686\_Mf\_liverA, NM\_001099634, NM\_009776, Unigene34394\_Mf\_liverA, Unigene27082\_Mf\_liverA, NM\_031165, Unigene14810\_Mf\_liverA, Unigene33632\_Mf\_liverA, Unigene37819\_Mf\_liverA, CL2855.Contig1\_Mf\_liverA, Unigene28499\_Mf\_liverA, Unigene30585\_Mf\_liverA, Unigene8054\_Mf\_liverA |
| cell projection | Unigene21684\_Mf\_liverA, Unigene29399\_Mf\_liverA, Unigene31251\_Mf\_liverA, Unigene22874\_Mf\_liverA, CL425.Contig1\_Mf\_liverA, Unigene24883\_Mf\_liverA, Unigene29424\_Mf\_liverA, Unigene29008\_Mf\_liverA, Unigene36762\_Mf\_liverA, CL777.Contig8\_Mf\_liverA, Unigene29231\_Mf\_liverA, Unigene5693\_Mf\_liverA, Unigene34867\_Mf\_liverA, Unigene5165\_Mf\_liverA, Unigene24252\_Mf\_liverA, Unigene30707\_Mf\_liverA, Unigene36414\_Mf\_liverA, Unigene18500\_Mf\_liverA, Unigene5175\_Mf\_liverA, Unigene20602\_Mf\_liverA, Unigene36328\_Mf\_liverA, Unigene37488\_Mf\_liverA, NM\_010141, NM\_009609, CL1810.Contig1\_Mf\_liverA, Unigene24547\_Mf\_liverA, Unigene13593\_Mf\_liverA, Unigene4686\_Mf\_liverA, Unigene5138\_Mf\_liverA, CL3207.Contig1\_Mf\_liverA, CL1493.Contig2\_Mf\_liverA, CL1493.Contig1\_Mf\_liverA, NM\_033444, Unigene36420\_Mf\_liverA, Unigene1483\_Mf\_liverA, Unigene32294\_Mf\_liverA, Unigene13363\_Mf\_liverA, Unigene36417\_Mf\_liverA, NM\_177093, Unigene23870\_Mf\_liverA, Unigene21561\_Mf\_liverA, CL2251.Contig1\_Mf\_liverA, Unigene8740\_Mf\_liverA, NM\_178405, Unigene36669\_Mf\_liverA, Unigene36698\_Mf\_liverA, CL2632.Contig2\_Mf\_liverA, Unigene14582\_Mf\_liverA, Unigene13945\_Mf\_liverA, NM\_011305, NM\_012030, Unigene27422\_Mf\_liverA, Unigene39011\_Mf\_liverA, Unigene25333\_Mf\_liverA, Unigene36418\_Mf\_liverA, Unigene13950\_Mf\_liverA, Unigene27547\_Mf\_liverA, NM\_134156, Unigene4720\_Mf\_liverA, Unigene32295\_Mf\_liverA, Unigene21562\_Mf\_liverA, Unigene27248\_Mf\_liverA, Unigene4636\_Mf\_liverA, Unigene8132\_Mf\_liverA, Unigene2195\_Mf\_liverA, Unigene496\_Mf\_liverA, Unigene34866\_Mf\_liverA, Unigene31852\_Mf\_liverA, Unigene37076\_Mf\_liverA, Unigene5712\_Mf\_liverA, CL3166.Contig4\_Mf\_liverA, CL5189.Contig1\_Mf\_liverA, CL5576.Contig1\_Mf\_liverA, CL1352.Contig1\_Mf\_liverA, Unigene31206\_Mf\_liverA, NM\_011099 |
| endosome membrane | NM\_133838, Unigene24471\_Mf\_liverA, Unigene38015\_Mf\_liverA, Unigene1292\_Mf\_liverA, Unigene15077\_Mf\_liverA, CL3002.Contig1\_Mf\_liverA, CL3835.Contig2\_Mf\_liverA, Unigene4681\_Mf\_liverA, Unigene5693\_Mf\_liverA, Unigene30878\_Mf\_liverA, Unigene2746\_Mf\_liverA, Unigene2745\_Mf\_liverA, CL2001.Contig1\_Mf\_liverA, NM\_011082, CL3835.Contig1\_Mf\_liverA |
| neuron projection | Unigene29399\_Mf\_liverA, Unigene31251\_Mf\_liverA, Unigene13363\_Mf\_liverA, Unigene36417\_Mf\_liverA, NM\_177093, CL425.Contig1\_Mf\_liverA, Unigene21561\_Mf\_liverA, Unigene24883\_Mf\_liverA, Unigene23870\_Mf\_liverA, Unigene8740\_Mf\_liverA, NM\_178405, CL2632.Contig2\_Mf\_liverA, Unigene36762\_Mf\_liverA, Unigene14582\_Mf\_liverA, NM\_011305, Unigene13945\_Mf\_liverA, Unigene29231\_Mf\_liverA, Unigene36418\_Mf\_liverA, Unigene34867\_Mf\_liverA, Unigene5165\_Mf\_liverA, Unigene13950\_Mf\_liverA, Unigene24252\_Mf\_liverA, Unigene30707\_Mf\_liverA, Unigene27547\_Mf\_liverA, Unigene18500\_Mf\_liverA, Unigene36414\_Mf\_liverA, Unigene37488\_Mf\_liverA, NM\_010141, NM\_134156, Unigene4720\_Mf\_liverA, CL1810.Contig1\_Mf\_liverA, NM\_009609, Unigene21562\_Mf\_liverA, Unigene4636\_Mf\_liverA, Unigene8132\_Mf\_liverA, Unigene24547\_Mf\_liverA, Unigene13593\_Mf\_liverA, Unigene4686\_Mf\_liverA, Unigene34866\_Mf\_liverA, Unigene37076\_Mf\_liverA, Unigene5712\_Mf\_liverA, CL3207.Contig1\_Mf\_liverA, CL3166.Contig4\_Mf\_liverA, CL1352.Contig1\_Mf\_liverA, CL5189.Contig1\_Mf\_liverA, Unigene36420\_Mf\_liverA |
| filopodium | Unigene30707\_Mf\_liverA, Unigene39011\_Mf\_liverA, Unigene21684\_Mf\_liverA, CL1352.Contig1\_Mf\_liverA, Unigene31852\_Mf\_liverA, Unigene29424\_Mf\_liverA, Unigene13950\_Mf\_liverA |
| NADPH oxidase complex | Unigene4686\_Mf\_liverA, Unigene8740\_Mf\_liverA |
| cytoplasmic membrane-bounded vesicle | Unigene29399\_Mf\_liverA, Unigene4909\_Mf\_liverA, CL5807.Contig1\_Mf\_liverA, NM\_009898, Unigene33080\_Mf\_liverA, Unigene13363\_Mf\_liverA, NM\_021278, CL2855.Contig2\_Mf\_liverA, Unigene15529\_Mf\_liverA, CL4925.Contig1\_Mf\_liverA, Unigene36190\_Mf\_liverA, NM\_009255, NM\_011082, Unigene29424\_Mf\_liverA, CL2251.Contig1\_Mf\_liverA, Unigene8740\_Mf\_liverA, Unigene4922\_Mf\_liverA, Unigene29008\_Mf\_liverA, Unigene5632\_Mf\_liverA, CL3816.Contig2\_Mf\_liverA, Unigene36669\_Mf\_liverA, CL1052.Contig1\_Mf\_liverA, Unigene30587\_Mf\_liverA, Unigene36762\_Mf\_liverA, CL3816.Contig1\_Mf\_liverA, Unigene36757\_Mf\_liverA, Unigene13950\_Mf\_liverA, Unigene30584\_Mf\_liverA, Unigene34810\_Mf\_liverA, CL44.Contig1\_Mf\_liverA, Unigene27081\_Mf\_liverA, Unigene36328\_Mf\_liverA, NM\_134156, Unigene41336\_Mf\_liverA, Unigene37389\_Mf\_liverA, Unigene14809\_Mf\_liverA, Unigene28662\_Mf\_liverA, CL2001.Contig1\_Mf\_liverA, Unigene36673\_Mf\_liverA, Unigene4686\_Mf\_liverA, NM\_009776, Unigene34394\_Mf\_liverA, Unigene27082\_Mf\_liverA, NM\_031165, Unigene14810\_Mf\_liverA, Unigene33632\_Mf\_liverA, Unigene37819\_Mf\_liverA, CL2855.Contig1\_Mf\_liverA, Unigene28499\_Mf\_liverA, Unigene30585\_Mf\_liverA, Unigene8054\_Mf\_liverA |
| zymogen granule | Unigene8054\_Mf\_liverA, Unigene15529\_Mf\_liverA, Unigene34810\_Mf\_liverA |
| vesicle lumen | Unigene29399\_Mf\_liverA, Unigene14809\_Mf\_liverA, Unigene28662\_Mf\_liverA, CL2251.Contig1\_Mf\_liverA, Unigene15529\_Mf\_liverA, Unigene36673\_Mf\_liverA |
| intrinsic to external side of plasma membrane | CL4156.Contig1\_Mf\_liverA, Unigene21562\_Mf\_liverA, Unigene21561\_Mf\_liverA |
| condensed nuclear chromosome kinetochore | CL5268.Contig1\_Mf\_liverA, Unigene45530\_Mf\_liverA |
| chromaffin granule | Unigene34394\_Mf\_liverA, CL5807.Contig1\_Mf\_liverA |
| vesicular fraction | NM\_001100182, NM\_001081372, NM\_009128, NM\_201360, NM\_008293, NM\_010001, NM\_145474, NM\_178405, NM\_007822, NM\_007811, NM\_153193, NM\_001081148, NM\_001253832, NM\_001104531, NM\_007820, NM\_010003, NM\_013821 |
| trans-Golgi network membrane | CL2855.Contig1\_Mf\_liverA, CL2855.Contig2\_Mf\_liverA, CL4925.Contig1\_Mf\_liverA |
| dendrite | Unigene30707\_Mf\_liverA, Unigene36414\_Mf\_liverA, Unigene18500\_Mf\_liverA, Unigene27547\_Mf\_liverA, Unigene37488\_Mf\_liverA, Unigene31251\_Mf\_liverA, Unigene13363\_Mf\_liverA, NM\_134156, Unigene36417\_Mf\_liverA, Unigene21562\_Mf\_liverA, CL425.Contig1\_Mf\_liverA, Unigene24883\_Mf\_liverA, Unigene21561\_Mf\_liverA, Unigene24547\_Mf\_liverA, Unigene8740\_Mf\_liverA, Unigene13593\_Mf\_liverA, NM\_178405, Unigene4686\_Mf\_liverA, Unigene36420\_Mf\_liverA, Unigene36418\_Mf\_liverA, Unigene24252\_Mf\_liverA |
| type III intermediate filament | Unigene37076\_Mf\_liverA, Unigene5165\_Mf\_liverA |
| anchored to plasma membrane | CL4156.Contig1\_Mf\_liverA, Unigene21562\_Mf\_liverA, Unigene21561\_Mf\_liverA, CL3575.Contig1\_Mf\_liverA |
| platelet alpha granule lumen | Unigene29399\_Mf\_liverA, Unigene28662\_Mf\_liverA, CL2251.Contig1\_Mf\_liverA, Unigene15529\_Mf\_liverA, Unigene36673\_Mf\_liverA |
| cortical cytoskeleton | Unigene30707\_Mf\_liverA, Unigene25046\_Mf\_liverA, CL848.Contig2\_Mf\_liverA, Unigene29308\_Mf\_liverA, Unigene28822\_Mf\_liverA, Unigene29008\_Mf\_liverA |
| secretory granule lumen | Unigene29399\_Mf\_liverA, Unigene28662\_Mf\_liverA, CL2251.Contig1\_Mf\_liverA, Unigene15529\_Mf\_liverA, Unigene36673\_Mf\_liverA |
| cell cortex part | Unigene30707\_Mf\_liverA, Unigene25046\_Mf\_liverA, CL4701.Contig1\_Mf\_liverA, CL848.Contig2\_Mf\_liverA, Unigene29308\_Mf\_liverA, Unigene28822\_Mf\_liverA, Unigene37575\_Mf\_liverA, Unigene29008\_Mf\_liverA |
| main axon | Unigene34866\_Mf\_liverA, Unigene24883\_Mf\_liverA, Unigene34867\_Mf\_liverA, Unigene4720\_Mf\_liverA |
| intermediate filament | Unigene27081\_Mf\_liverA, Unigene27082\_Mf\_liverA, CL4162.Contig1\_Mf\_liverA, Unigene5941\_Mf\_liverA, Unigene37076\_Mf\_liverA, Unigene5940\_Mf\_liverA, Unigene5165\_Mf\_liverA |
| organelle outer membrane | CL2478.Contig3\_Mf\_liverA, Unigene27081\_Mf\_liverA, Unigene27082\_Mf\_liverA, Unigene43357\_Mf\_liverA, Unigene37535\_Mf\_liverA, CL1810.Contig1\_Mf\_liverA, CL3104.Contig1\_Mf\_liverA, CL5631.Contig1\_Mf\_liverA, Unigene25462\_Mf\_liverA, Unigene4781\_Mf\_liverA |
| neuronal cell body | Unigene29399\_Mf\_liverA, Unigene18500\_Mf\_liverA, Unigene27547\_Mf\_liverA, Unigene4720\_Mf\_liverA, CL425.Contig1\_Mf\_liverA, Unigene8132\_Mf\_liverA, Unigene8740\_Mf\_liverA, Unigene13593\_Mf\_liverA, Unigene4686\_Mf\_liverA, Unigene34866\_Mf\_liverA, Unigene5138\_Mf\_liverA, CL2632.Contig2\_Mf\_liverA, Unigene5712\_Mf\_liverA, Unigene14582\_Mf\_liverA, CL3166.Contig4\_Mf\_liverA, Unigene34867\_Mf\_liverA |
| axon | Unigene29399\_Mf\_liverA, Unigene36414\_Mf\_liverA, Unigene18500\_Mf\_liverA, Unigene13363\_Mf\_liverA, Unigene36417\_Mf\_liverA, Unigene4720\_Mf\_liverA, Unigene24883\_Mf\_liverA, Unigene8132\_Mf\_liverA, Unigene34866\_Mf\_liverA, Unigene37076\_Mf\_liverA, CL2632.Contig2\_Mf\_liverA, CL3166.Contig4\_Mf\_liverA, Unigene29231\_Mf\_liverA, Unigene36420\_Mf\_liverA, Unigene36418\_Mf\_liverA, Unigene34867\_Mf\_liverA, Unigene5165\_Mf\_liverA |
| zymogen granule membrane | Unigene8054\_Mf\_liverA, Unigene34810\_Mf\_liverA |
| microvillus | Unigene36669\_Mf\_liverA, Unigene29399\_Mf\_liverA, CL1352.Contig1\_Mf\_liverA, Unigene36698\_Mf\_liverA, CL777.Contig8\_Mf\_liverA, Unigene29424\_Mf\_liverA, NM\_012030 |
| early endosome | Unigene27081\_Mf\_liverA, Unigene37245\_Mf\_liverA, Unigene27082\_Mf\_liverA, NM\_133838, Unigene36698\_Mf\_liverA, Unigene15077\_Mf\_liverA, CL3002.Contig1\_Mf\_liverA, Unigene36034\_Mf\_liverA, NM\_010391, Unigene36757\_Mf\_liverA, NR\_004446 |
| uropod | Unigene29424\_Mf\_liverA, Unigene22874\_Mf\_liverA |
| I-kappaB/NF-kappaB complex | CL3800.Contig1\_Mf\_liverA, Unigene152\_Mf\_liverA |
| gap junction | Unigene27081\_Mf\_liverA, Unigene27082\_Mf\_liverA, CL2260.Contig1\_Mf\_liverA |
| outer membrane | CL2478.Contig3\_Mf\_liverA, Unigene27081\_Mf\_liverA, Unigene27082\_Mf\_liverA, Unigene43357\_Mf\_liverA, Unigene37535\_Mf\_liverA, CL1810.Contig1\_Mf\_liverA, CL3104.Contig1\_Mf\_liverA, CL5631.Contig1\_Mf\_liverA, Unigene25462\_Mf\_liverA, Unigene4781\_Mf\_liverA |
| condensed nuclear chromosome, centromeric region | CL5268.Contig1\_Mf\_liverA, Unigene45530\_Mf\_liverA |
| chromatin assembly complex | CL3900.Contig1\_Mf\_liverA, CL4757.Contig1\_Mf\_liverA |
| growth cone | CL1352.Contig1\_Mf\_liverA, Unigene21562\_Mf\_liverA, Unigene31251\_Mf\_liverA, Unigene21561\_Mf\_liverA, Unigene24547\_Mf\_liverA, Unigene14582\_Mf\_liverA, Unigene13950\_Mf\_liverA |
| recycling endosome membrane | Unigene2746\_Mf\_liverA, Unigene2745\_Mf\_liverA, CL2001.Contig1\_Mf\_liverA |
| filopodium membrane | Unigene31852\_Mf\_liverA, Unigene29424\_Mf\_liverA |
| melanosome | CL3816.Contig2\_Mf\_liverA, CL3816.Contig1\_Mf\_liverA, Unigene36190\_Mf\_liverA, Unigene36762\_Mf\_liverA, CL2001.Contig1\_Mf\_liverA |
| extracellular vesicular exosome | CL2001.Contig1\_Mf\_liverA, CL2251.Contig1\_Mf\_liverA |
| mitochondrial outer membrane | Unigene27081\_Mf\_liverA, Unigene27082\_Mf\_liverA, Unigene43357\_Mf\_liverA, CL1810.Contig1\_Mf\_liverA, CL3104.Contig1\_Mf\_liverA, CL5631.Contig1\_Mf\_liverA, Unigene25462\_Mf\_liverA, Unigene4781\_Mf\_liverA |
| cell-cell adherens junction | Unigene27081\_Mf\_liverA, Unigene27082\_Mf\_liverA, CL422.Contig1\_Mf\_liverA, Unigene2746\_Mf\_liverA, Unigene2745\_Mf\_liverA, NM\_134156 |
| cortical actin cytoskeleton | Unigene30707\_Mf\_liverA, CL848.Contig2\_Mf\_liverA, Unigene28822\_Mf\_liverA, Unigene29008\_Mf\_liverA |
| integral to lumenal side of endoplasmic reticulum membrane | CL2855.Contig1\_Mf\_liverA, CL2855.Contig2\_Mf\_liverA |
| clathrin coated vesicle membrane | Unigene34394\_Mf\_liverA, CL2855.Contig1\_Mf\_liverA, CL5807.Contig1\_Mf\_liverA, NM\_031165, CL2855.Contig2\_Mf\_liverA, CL4925.Contig1\_Mf\_liverA |
| protein-lipid complex | Unigene36698\_Mf\_liverA, Unigene9081\_Mf\_liverA, Unigene30815\_Mf\_liverA, Unigene30814\_Mf\_liverA, Unigene24157\_Mf\_liverA |
| plasma lipoprotein particle | Unigene36698\_Mf\_liverA, Unigene9081\_Mf\_liverA, Unigene30815\_Mf\_liverA, Unigene30814\_Mf\_liverA, Unigene24157\_Mf\_liverA |
| extrinsic to membrane | CL1052.Contig1\_Mf\_liverA, Unigene37243\_Mf\_liverA, Unigene591\_Mf\_liverA, CL4156.Contig1\_Mf\_liverA, Unigene4636\_Mf\_liverA, Unigene8054\_Mf\_liverA, NM\_001025388, Unigene29424\_Mf\_liverA, Unigene34810\_Mf\_liverA |
| trailing edge | Unigene29424\_Mf\_liverA, Unigene22874\_Mf\_liverA |
| cell-cell junction | Unigene21684\_Mf\_liverA, Unigene27081\_Mf\_liverA, CL5586.Contig1\_Mf\_liverA, CL422.Contig1\_Mf\_liverA, NM\_134156, Unigene25226\_Mf\_liverA, CL4033.Contig1\_Mf\_liverA, Unigene23870\_Mf\_liverA, CL2260.Contig1\_Mf\_liverA, Unigene25055\_Mf\_liverA, Unigene14637\_Mf\_liverA, Unigene27082\_Mf\_liverA, Unigene112\_Mf\_liverA, Unigene14765\_Mf\_liverA, Unigene39011\_Mf\_liverA, Unigene5693\_Mf\_liverA, Unigene2746\_Mf\_liverA, Unigene2745\_Mf\_liverA, Unigene25052\_Mf\_liverA, Unigene13950\_Mf\_liverA |
| condensed chromosome outer kinetochore | CL5268.Contig1\_Mf\_liverA, Unigene45530\_Mf\_liverA |
| CD40 receptor complex | Unigene15982\_Mf\_liverA, Unigene843\_Mf\_liverA |
| internal side of plasma membrane | CL1052.Contig1\_Mf\_liverA, Unigene843\_Mf\_liverA, Unigene33080\_Mf\_liverA, Unigene32564\_Mf\_liverA, Unigene591\_Mf\_liverA, Unigene4636\_Mf\_liverA, Unigene29424\_Mf\_liverA, Unigene37575\_Mf\_liverA |
| acrosomal membrane | CL3816.Contig2\_Mf\_liverA, CL3816.Contig1\_Mf\_liverA, Unigene36757\_Mf\_liverA |
| fascia adherens | Unigene27081\_Mf\_liverA, Unigene27082\_Mf\_liverA |
| intermediate filament cytoskeleton | Unigene27081\_Mf\_liverA, Unigene27082\_Mf\_liverA, CL4162.Contig1\_Mf\_liverA, Unigene5941\_Mf\_liverA, Unigene37076\_Mf\_liverA, Unigene5940\_Mf\_liverA, Unigene5165\_Mf\_liverA |
| hemidesmosome | Unigene38870\_Mf\_liverA, Unigene39252\_Mf\_liverA |
| cell leading edge | Unigene30707\_Mf\_liverA, Unigene21684\_Mf\_liverA, Unigene5175\_Mf\_liverA, NM\_009898, Unigene39252\_Mf\_liverA, Unigene38870\_Mf\_liverA, Unigene24883\_Mf\_liverA, Unigene23870\_Mf\_liverA, Unigene29008\_Mf\_liverA, Unigene31852\_Mf\_liverA, Unigene37076\_Mf\_liverA, Unigene5138\_Mf\_liverA, CL3207.Contig1\_Mf\_liverA, NM\_012030, Unigene27422\_Mf\_liverA, Unigene39011\_Mf\_liverA, CL1352.Contig1\_Mf\_liverA, Unigene5693\_Mf\_liverA, Unigene5165\_Mf\_liverA, Unigene24252\_Mf\_liverA |
| nuclear envelope | Unigene32294\_Mf\_liverA, CL2478.Contig3\_Mf\_liverA, Unigene28142\_Mf\_liverA, Unigene37535\_Mf\_liverA, Unigene15703\_Mf\_liverA, Unigene32295\_Mf\_liverA, NM\_018815, CL1575.Contig1\_Mf\_liverA, NM\_080638, Unigene8132\_Mf\_liverA, CL5254.Contig1\_Mf\_liverA, Unigene14270\_Mf\_liverA, NM\_001099634, CL1263.Contig1\_Mf\_liverA, CL4162.Contig1\_Mf\_liverA, NM\_033444, Unigene16891\_Mf\_liverA |
| recycling endosome | Unigene2746\_Mf\_liverA, Unigene2745\_Mf\_liverA, CL2001.Contig1\_Mf\_liverA, Unigene24503\_Mf\_liverA |
| phagocytic cup | Unigene36328\_Mf\_liverA, Unigene5138\_Mf\_liverA, Unigene29008\_Mf\_liverA |
| intercalated disc | Unigene27081\_Mf\_liverA, Unigene27082\_Mf\_liverA, Unigene13950\_Mf\_liverA |
| organelle envelope | Unigene35476\_Mf\_liverA, Unigene32294\_Mf\_liverA, Unigene17048\_Mf\_liverA, Unigene28142\_Mf\_liverA, NM\_018815, NM\_008293, CL1575.Contig1\_Mf\_liverA, NM\_080638, CL2797.Contig2\_Mf\_liverA, Unigene3377\_Mf\_liverA, NM\_019879, Unigene34727\_Mf\_liverA, Unigene14270\_Mf\_liverA, CL738.Contig2\_Mf\_liverA, CL1125.Contig1\_Mf\_liverA, CL1263.Contig1\_Mf\_liverA, CL4490.Contig2\_Mf\_liverA, CL4162.Contig1\_Mf\_liverA, NM\_025336, CL5631.Contig1\_Mf\_liverA, Unigene23185\_Mf\_liverA, CL5796.Contig2\_Mf\_liverA, Unigene25398\_Mf\_liverA, CL2478.Contig3\_Mf\_liverA, Unigene27081\_Mf\_liverA, CL5316.Contig1\_Mf\_liverA, Unigene37535\_Mf\_liverA, CL1810.Contig1\_Mf\_liverA, CL3104.Contig1\_Mf\_liverA, Unigene15703\_Mf\_liverA, Unigene32295\_Mf\_liverA, Unigene8132\_Mf\_liverA, CL5254.Contig1\_Mf\_liverA, Unigene4781\_Mf\_liverA, NM\_001099634, Unigene27082\_Mf\_liverA, NM\_153193, Unigene43357\_Mf\_liverA, Unigene14508\_Mf\_liverA, NM\_033444, Unigene16891\_Mf\_liverA, NM\_013821, Unigene25462\_Mf\_liverA, Unigene13233\_Mf\_liverA |
| mitochondrial matrix | Unigene14916\_Mf\_liverA, CL1125.Contig1\_Mf\_liverA, Unigene37460\_Mf\_liverA, CL532.Contig1\_Mf\_liverA, NM\_010481, Unigene6110\_Mf\_liverA, Unigene14171\_Mf\_liverA, CL2791.Contig1\_Mf\_liverA, Unigene30731\_Mf\_liverA, NM\_019879, Unigene34727\_Mf\_liverA, Unigene35816\_Mf\_liverA |
| coated vesicle membrane | Unigene34394\_Mf\_liverA, CL2855.Contig1\_Mf\_liverA, CL5807.Contig1\_Mf\_liverA, NM\_031165, CL2855.Contig2\_Mf\_liverA, Unigene5632\_Mf\_liverA, CL4925.Contig1\_Mf\_liverA |
| membrane fraction | NM\_001100182, NM\_001081372, NM\_009128, NM\_201360, NM\_008293, NM\_010001, NM\_145474, NM\_178405, NM\_007822, NM\_007811, NM\_153193, NM\_001081148, NM\_001253832, NM\_001104531, NM\_007820, NM\_011072, NM\_010003, NM\_001025388, NM\_013821 |
| SCF ubiquitin ligase complex | Unigene29063\_Mf\_liverA, Unigene13683\_Mf\_liverA |
| endoplasmic reticulum lumen | Unigene36669\_Mf\_liverA, Unigene30493\_Mf\_liverA, Unigene21466\_Mf\_liverA, Unigene25595\_Mf\_liverA, Unigene25596\_Mf\_liverA, Unigene22052\_Mf\_liverA |
| envelope | Unigene35476\_Mf\_liverA, Unigene32294\_Mf\_liverA, Unigene17048\_Mf\_liverA, Unigene28142\_Mf\_liverA, NM\_018815, NM\_008293, CL1575.Contig1\_Mf\_liverA, NM\_080638, CL2797.Contig2\_Mf\_liverA, Unigene3377\_Mf\_liverA, NM\_019879, Unigene34727\_Mf\_liverA, Unigene14270\_Mf\_liverA, CL738.Contig2\_Mf\_liverA, CL1125.Contig1\_Mf\_liverA, CL1263.Contig1\_Mf\_liverA, CL4490.Contig2\_Mf\_liverA, CL4162.Contig1\_Mf\_liverA, NM\_025336, CL5631.Contig1\_Mf\_liverA, Unigene23185\_Mf\_liverA, CL5796.Contig2\_Mf\_liverA, Unigene25398\_Mf\_liverA, CL2478.Contig3\_Mf\_liverA, Unigene27081\_Mf\_liverA, CL5316.Contig1\_Mf\_liverA, Unigene37535\_Mf\_liverA, CL1810.Contig1\_Mf\_liverA, CL3104.Contig1\_Mf\_liverA, Unigene15703\_Mf\_liverA, Unigene32295\_Mf\_liverA, Unigene8132\_Mf\_liverA, CL5254.Contig1\_Mf\_liverA, Unigene4781\_Mf\_liverA, NM\_001099634, Unigene27082\_Mf\_liverA, NM\_153193, Unigene43357\_Mf\_liverA, Unigene14508\_Mf\_liverA, NM\_033444, Unigene16891\_Mf\_liverA, NM\_013821, Unigene25462\_Mf\_liverA, Unigene13233\_Mf\_liverA |
| Golgi-associated vesicle | Unigene36669\_Mf\_liverA, Unigene27081\_Mf\_liverA, Unigene27082\_Mf\_liverA, Unigene41336\_Mf\_liverA, Unigene5632\_Mf\_liverA |
| intrinsic to endoplasmic reticulum membrane | CL695.Contig1\_Mf\_liverA, CL2855.Contig1\_Mf\_liverA, Unigene32695\_Mf\_liverA, NM\_009128, NM\_010162, Unigene10135\_Mf\_liverA, CL2855.Contig2\_Mf\_liverA |
| mitochondrial part | Unigene35476\_Mf\_liverA, Unigene17048\_Mf\_liverA, NM\_020559, NM\_010481, NM\_008293, CL2797.Contig2\_Mf\_liverA, Unigene3377\_Mf\_liverA, NM\_019879, Unigene34727\_Mf\_liverA, CL738.Contig2\_Mf\_liverA, Unigene14916\_Mf\_liverA, CL1125.Contig1\_Mf\_liverA, CL4490.Contig2\_Mf\_liverA, Unigene37460\_Mf\_liverA, Unigene30731\_Mf\_liverA, CL5631.Contig1\_Mf\_liverA, Unigene23185\_Mf\_liverA, CL5796.Contig2\_Mf\_liverA, Unigene25398\_Mf\_liverA, Unigene27081\_Mf\_liverA, CL5316.Contig1\_Mf\_liverA, CL1810.Contig1\_Mf\_liverA, CL3104.Contig1\_Mf\_liverA, Unigene35816\_Mf\_liverA, Unigene4781\_Mf\_liverA, CL532.Contig1\_Mf\_liverA, Unigene27082\_Mf\_liverA, NM\_153193, Unigene6110\_Mf\_liverA, Unigene43357\_Mf\_liverA, Unigene14508\_Mf\_liverA, Unigene14171\_Mf\_liverA, CL2791.Contig1\_Mf\_liverA, NM\_013821, Unigene25462\_Mf\_liverA, Unigene13233\_Mf\_liverA |
| rough endoplasmic reticulum | Unigene36669\_Mf\_liverA, Unigene21466\_Mf\_liverA, Unigene8740\_Mf\_liverA |
| contractile fiber | Unigene5639\_Mf\_liverA, Unigene27081\_Mf\_liverA, Unigene27082\_Mf\_liverA, NM\_134156, NM\_009609, NM\_177093, CL1352.Contig1\_Mf\_liverA, Unigene4556\_Mf\_liverA, NM\_001025388, Unigene8132\_Mf\_liverA, CL2251.Contig1\_Mf\_liverA, Unigene13593\_Mf\_liverA |
| heterotrimeric G-protein complex | Unigene4636\_Mf\_liverA, Unigene591\_Mf\_liverA |
| clathrin adaptor complex | Unigene41336\_Mf\_liverA, CL4925.Contig1\_Mf\_liverA |
| Golgi membrane | CL787.Contig1\_Mf\_liverA, Unigene27081\_Mf\_liverA, CL5586.Contig1\_Mf\_liverA, Unigene36328\_Mf\_liverA, CL2855.Contig2\_Mf\_liverA, Unigene15529\_Mf\_liverA, Unigene37389\_Mf\_liverA, CL4925.Contig1\_Mf\_liverA, CL4033.Contig1\_Mf\_liverA, Unigene4922\_Mf\_liverA, Unigene34010\_Mf\_liverA, Unigene5632\_Mf\_liverA, CL3816.Contig2\_Mf\_liverA, Unigene27082\_Mf\_liverA, Unigene112\_Mf\_liverA, CL4600.Contig1\_Mf\_liverA, CL3816.Contig1\_Mf\_liverA, CL2855.Contig1\_Mf\_liverA, Unigene28499\_Mf\_liverA, Unigene1221\_Mf\_liverA |
| aggresome | Unigene35816\_Mf\_liverA, Unigene9406\_Mf\_liverA |
| mitochondrial envelope | Unigene35476\_Mf\_liverA, Unigene25398\_Mf\_liverA, Unigene27081\_Mf\_liverA, Unigene17048\_Mf\_liverA, CL5316.Contig1\_Mf\_liverA, CL1810.Contig1\_Mf\_liverA, CL3104.Contig1\_Mf\_liverA, NM\_008293, CL2797.Contig2\_Mf\_liverA, Unigene3377\_Mf\_liverA, Unigene34727\_Mf\_liverA, Unigene4781\_Mf\_liverA, CL738.Contig2\_Mf\_liverA, CL1125.Contig1\_Mf\_liverA, CL4490.Contig2\_Mf\_liverA, Unigene27082\_Mf\_liverA, NM\_153193, Unigene43357\_Mf\_liverA, Unigene14508\_Mf\_liverA, CL5631.Contig1\_Mf\_liverA, NM\_013821, Unigene25462\_Mf\_liverA, Unigene13233\_Mf\_liverA, Unigene23185\_Mf\_liverA, CL5796.Contig2\_Mf\_liverA |
| cell part | Unigene21684\_Mf\_liverA, NM\_176843, NM\_009898, Unigene22874\_Mf\_liverA, NM\_025593, Unigene24111\_Mf\_liverA, Unigene28186\_Mf\_liverA, Unigene15553\_Mf\_liverA, NM\_010378, CL854.Contig1\_Mf\_liverA, Unigene16463\_Mf\_liverA, NM\_025613, Unigene39886\_Mf\_liverA, Unigene15064\_Mf\_liverA, Unigene24252\_Mf\_liverA, CL4757.Contig1\_Mf\_liverA, NR\_004446, CL1555.Contig1\_Mf\_liverA, Unigene37488\_Mf\_liverA, Unigene32564\_Mf\_liverA, Unigene37535\_Mf\_liverA, CL695.Contig1\_Mf\_liverA, Unigene35431\_Mf\_liverA, Unigene15703\_Mf\_liverA, Unigene31623\_Mf\_liverA, NM\_010233, Unigene13379\_Mf\_liverA, Unigene34394\_Mf\_liverA, NM\_172409, Unigene25594\_Mf\_liverA, Unigene14050\_Mf\_liverA, CL4995.Contig1\_Mf\_liverA, Unigene8054\_Mf\_liverA, Unigene18430\_Mf\_liverA, Unigene5552\_Mf\_liverA, CL5807.Contig1\_Mf\_liverA, CL2384.Contig1\_Mf\_liverA, Unigene26065\_Mf\_liverA, Unigene36417\_Mf\_liverA, Unigene18125\_Mf\_liverA, NM\_177093, CL4293.Contig1\_Mf\_liverA, Unigene13097\_Mf\_liverA, Unigene25226\_Mf\_liverA, Unigene33512\_Mf\_liverA, Unigene14907\_Mf\_liverA, Unigene25976\_Mf\_liverA, Unigene36698\_Mf\_liverA, NM\_145942, Unigene30261\_Mf\_liverA, Unigene13945\_Mf\_liverA, Unigene139\_Mf\_liverA, Unigene28217\_Mf\_liverA, Unigene36836\_Mf\_liverA, Unigene33366\_Mf\_liverA, NM\_134156, Unigene37389\_Mf\_liverA, Unigene4720\_Mf\_liverA, CL4156.Contig1\_Mf\_liverA, Unigene27248\_Mf\_liverA, CL2266.Contig2\_Mf\_liverA, Unigene30839\_Mf\_liverA, Unigene34010\_Mf\_liverA, Unigene24613\_Mf\_liverA, Unigene152\_Mf\_liverA, Unigene31852\_Mf\_liverA, CL532.Contig1\_Mf\_liverA, Unigene37076\_Mf\_liverA, Unigene1280\_Mf\_liverA, Unigene30288\_Mf\_liverA, Unigene14171\_Mf\_liverA, CL275.Contig5\_Mf\_liverA, CL5576.Contig1\_Mf\_liverA, Unigene33632\_Mf\_liverA, CL5189.Contig1\_Mf\_liverA, CL3339.Contig1\_Mf\_liverA, Unigene2746\_Mf\_liverA, Unigene4593\_Mf\_liverA, Unigene10496\_Mf\_liverA, Unigene4363\_Mf\_liverA, CL425.Contig1\_Mf\_liverA, CL2797.Contig2\_Mf\_liverA, CL1803.Contig1\_Mf\_liverA, Unigene37243\_Mf\_liverA, Unigene30587\_Mf\_liverA, Unigene39403\_Mf\_liverA, Unigene15077\_Mf\_liverA, Unigene36034\_Mf\_liverA, NM\_009253, Unigene5693\_Mf\_liverA, Unigene39970\_Mf\_liverA, CL3393.Contig1\_Mf\_liverA, Unigene29876\_Mf\_liverA, CL3800.Contig1\_Mf\_liverA, Unigene26585\_Mf\_liverA, Unigene28687\_Mf\_liverA, CL422.Contig1\_Mf\_liverA, CL1988.Contig3\_Mf\_liverA, NM\_028785, Unigene5758\_Mf\_liverA, NM\_009609, CL1810.Contig1\_Mf\_liverA, CL4033.Contig1\_Mf\_liverA, Unigene24547\_Mf\_liverA, Unigene34983\_Mf\_liverA, NR\_003623, CL3519.Contig1\_Mf\_liverA, Unigene8033\_Mf\_liverA, CL1493.Contig1\_Mf\_liverA, CL5764.Contig1\_Mf\_liverA, Unigene35476\_Mf\_liverA, Unigene4909\_Mf\_liverA, Unigene24471\_Mf\_liverA, Unigene31571\_Mf\_liverA, NM\_010162, Unigene15529\_Mf\_liverA, CL4220.Contig1\_Mf\_liverA, Unigene37262\_Mf\_liverA, Unigene18126\_Mf\_liverA, NR\_033215, CL2251.Contig1\_Mf\_liverA, NM\_007822, CL5293.Contig1\_Mf\_liverA, CL4490.Contig2\_Mf\_liverA, Unigene25595\_Mf\_liverA, Unigene14286\_Mf\_liverA, Unigene32515\_Mf\_liverA, CL4816.Contig3\_Mf\_liverA, Unigene39011\_Mf\_liverA, Unigene20432\_Mf\_liverA, NM\_009022, Unigene35609\_Mf\_liverA, Unigene7048\_Mf\_liverA, Unigene13950\_Mf\_liverA, Unigene35046\_Mf\_liverA, Unigene28459\_Mf\_liverA, Unigene37245\_Mf\_liverA, Unigene13847\_Mf\_liverA, Unigene19658\_Mf\_liverA, NM\_027406, Unigene36699\_Mf\_liverA, Unigene36176\_Mf\_liverA, Unigene18340\_Mf\_liverA, Unigene496\_Mf\_liverA, Unigene42803\_Mf\_liverA, NM\_001164598, Unigene4983\_Mf\_liverA, Unigene29889\_Mf\_liverA, Unigene2939\_Mf\_liverA, Unigene9466\_Mf\_liverA, CL5640.Contig1\_Mf\_liverA, NM\_020559, Unigene35884\_Mf\_liverA, Unigene28142\_Mf\_liverA, CL3835.Contig2\_Mf\_liverA, Unigene38831\_Mf\_liverA, Unigene28899\_Mf\_liverA, Unigene14603\_Mf\_liverA, Unigene46870\_Mf\_liverA, Unigene24883\_Mf\_liverA, CL1575.Contig1\_Mf\_liverA, NM\_019651, Unigene13153\_Mf\_liverA, CL4117.Contig1\_Mf\_liverA, Unigene36849\_Mf\_liverA, Unigene8473\_Mf\_liverA, Unigene29008\_Mf\_liverA, Unigene25055\_Mf\_liverA, Unigene27593\_Mf\_liverA, CL2339.Contig1\_Mf\_liverA, Unigene29788\_Mf\_liverA, Unigene33508\_Mf\_liverA, CL777.Contig8\_Mf\_liverA, NM\_011072, Unigene14816\_Mf\_liverA, Unigene4776\_Mf\_liverA, Unigene21466\_Mf\_liverA, Unigene34810\_Mf\_liverA, Unigene27420\_Mf\_liverA, Unigene20602\_Mf\_liverA, Unigene36889\_Mf\_liverA, Unigene37999\_Mf\_liverA, Unigene40020\_Mf\_liverA, Unigene37904\_Mf\_liverA, Unigene41336\_Mf\_liverA, CL3483.Contig1\_Mf\_liverA, CL4141.Contig1\_Mf\_liverA, Unigene34184\_Mf\_liverA, CL5191.Contig2\_Mf\_liverA, Unigene13593\_Mf\_liverA, Unigene4686\_Mf\_liverA, Unigene11\_Mf\_liverA, Unigene4630\_Mf\_liverA, Unigene45131\_Mf\_liverA, Unigene5994\_Mf\_liverA, CL1493.Contig2\_Mf\_liverA, CL3575.Contig1\_Mf\_liverA, Unigene5294\_Mf\_liverA, Unigene5941\_Mf\_liverA, Unigene38104\_Mf\_liverA, Unigene38065\_Mf\_liverA, Unigene25462\_Mf\_liverA, Unigene36626\_Mf\_liverA, NM\_153795, Unigene28822\_Mf\_liverA, Unigene31988\_Mf\_liverA, Unigene5382\_Mf\_liverA, Unigene151\_Mf\_liverA, NM\_201360, CL848.Contig2\_Mf\_liverA, Unigene23870\_Mf\_liverA, Unigene13143\_Mf\_liverA, CL1263.Contig1\_Mf\_liverA, Unigene37460\_Mf\_liverA, Unigene31517\_Mf\_liverA, NM\_007811, Unigene24323\_Mf\_liverA, CL2142.Contig2\_Mf\_liverA, NM\_011305, CL3816.Contig1\_Mf\_liverA, Unigene25333\_Mf\_liverA, CL993.Contig2\_Mf\_liverA, CL529.Contig2\_Mf\_liverA, Unigene27081\_Mf\_liverA, Unigene37148\_Mf\_liverA, Unigene7195\_Mf\_liverA, Unigene1205\_Mf\_liverA, CL1352.Contig1\_Mf\_liverA, CL6039.Contig1\_Mf\_liverA, Unigene24804\_Mf\_liverA, Unigene31206\_Mf\_liverA, NM\_009128, CL2240.Contig1\_Mf\_liverA, Unigene29823\_Mf\_liverA, CL1125.Contig1\_Mf\_liverA, Unigene3752\_Mf\_liverA, Unigene38598\_Mf\_liverA, Unigene15681\_Mf\_liverA, Unigene5165\_Mf\_liverA, Unigene30707\_Mf\_liverA, NM\_133838, Unigene15552\_Mf\_liverA, CL3002.Contig1\_Mf\_liverA, Unigene30155\_Mf\_liverA, CL5057.Contig1\_Mf\_liverA, Unigene36514\_Mf\_liverA, NM\_145836, CL4816.Contig2\_Mf\_liverA, Unigene37139\_Mf\_liverA, Unigene33523\_Mf\_liverA, CL3207.Contig1\_Mf\_liverA, Unigene5906\_Mf\_liverA, NM\_001081148, Unigene14810\_Mf\_liverA, NM\_033444, Unigene4597\_Mf\_liverA, Unigene14170\_Mf\_liverA, CL2791.Contig1\_Mf\_liverA, CL4770.Contig1\_Mf\_liverA, Unigene10135\_Mf\_liverA, Unigene1483\_Mf\_liverA, CL114.Contig2\_Mf\_liverA, NM\_009883, Unigene33526\_Mf\_liverA, Unigene1327\_Mf\_liverA, Unigene39252\_Mf\_liverA, NM\_001081172, CL5978.Contig2\_Mf\_liverA, Unigene8740\_Mf\_liverA, Unigene14270\_Mf\_liverA, CL3816.Contig2\_Mf\_liverA, CL738.Contig2\_Mf\_liverA, CL442.Contig5\_Mf\_liverA, CL5459.Contig2\_Mf\_liverA, NM\_012030, Unigene24477\_Mf\_liverA, Unigene30983\_Mf\_liverA, Unigene30731\_Mf\_liverA, Unigene39749\_Mf\_liverA, Unigene36418\_Mf\_liverA, Unigene25052\_Mf\_liverA, CL3835.Contig1\_Mf\_liverA, Unigene38311\_Mf\_liverA, Unigene550\_Mf\_liverA, Unigene31198\_Mf\_liverA, CL2355.Contig1\_Mf\_liverA, Unigene28314\_Mf\_liverA, Unigene37153\_Mf\_liverA, Unigene14809\_Mf\_liverA, Unigene21562\_Mf\_liverA, Unigene15592\_Mf\_liverA, Unigene2195\_Mf\_liverA, Unigene30892\_Mf\_liverA, Unigene34866\_Mf\_liverA, Unigene31436\_Mf\_liverA, Unigene14765\_Mf\_liverA, Unigene4681\_Mf\_liverA, Unigene14284\_Mf\_liverA, Unigene5287\_Mf\_liverA, Unigene34609\_Mf\_liverA, NM\_144940, Unigene25721\_Mf\_liverA, CL3669.Contig2\_Mf\_liverA, Unigene37334\_Mf\_liverA, NM\_018815, NM\_080638, Unigene29424\_Mf\_liverA, NM\_011082, Unigene34727\_Mf\_liverA, CL4160.Contig2\_Mf\_liverA, CL4105.Contig1\_Mf\_liverA, Unigene26053\_Mf\_liverA, Unigene11007\_Mf\_liverA, CL4577.Contig1\_Mf\_liverA, Unigene785\_Mf\_liverA, Unigene38689\_Mf\_liverA, Unigene27339\_Mf\_liverA, NM\_021273, Unigene27294\_Mf\_liverA, Unigene1137\_Mf\_liverA, CL5586.Contig1\_Mf\_liverA, Unigene34341\_Mf\_liverA, Unigene30142\_Mf\_liverA, NM\_011921, Unigene35816\_Mf\_liverA, Unigene7897\_Mf\_liverA, CL2260.Contig1\_Mf\_liverA, NM\_145474, CL593.Contig2\_Mf\_liverA, NM\_009776, Unigene30003\_Mf\_liverA, Unigene5138\_Mf\_liverA, NM\_153193, NM\_031165, Unigene37150\_Mf\_liverA, Unigene14508\_Mf\_liverA, Unigene34143\_Mf\_liverA, Unigene30002\_Mf\_liverA, Unigene802\_Mf\_liverA, Unigene9150\_Mf\_liverA, Unigene17048\_Mf\_liverA, Unigene5639\_Mf\_liverA, Unigene33522\_Mf\_liverA, Unigene13363\_Mf\_liverA, CL2855.Contig2\_Mf\_liverA, NM\_021278, Unigene21561\_Mf\_liverA, NM\_010001, Unigene39655\_Mf\_liverA, Unigene4922\_Mf\_liverA, Unigene14637\_Mf\_liverA, NM\_153505, NM\_007622, NM\_001104531, Unigene4944\_Mf\_liverA, Unigene33560\_Mf\_liverA, Unigene23158\_Mf\_liverA, Unigene37575\_Mf\_liverA, Unigene25398\_Mf\_liverA, Unigene5774\_Mf\_liverA, Unigene27547\_Mf\_liverA, CL1736.Contig2\_Mf\_liverA, CL5316.Contig1\_Mf\_liverA, Unigene45530\_Mf\_liverA, Unigene4723\_Mf\_liverA, Unigene32332\_Mf\_liverA, Unigene29063\_Mf\_liverA, Unigene37470\_Mf\_liverA, CL5254.Contig1\_Mf\_liverA, Unigene35169\_Mf\_liverA, CL3166.Contig4\_Mf\_liverA, Unigene35698\_Mf\_liverA, Unigene32436\_Mf\_liverA, Unigene5169\_Mf\_liverA, Unigene16465\_Mf\_liverA, Unigene30528\_Mf\_liverA, NM\_007678, NM\_010392, Unigene31251\_Mf\_liverA, Unigene40610\_Mf\_liverA, CL186.Contig3\_Mf\_liverA, CL4925.Contig1\_Mf\_liverA, Unigene32421\_Mf\_liverA, Unigene583\_Mf\_liverA, CL4736.Contig1\_Mf\_liverA, Unigene27026\_Mf\_liverA, Unigene14916\_Mf\_liverA, Unigene35858\_Mf\_liverA, CL4600.Contig1\_Mf\_liverA, Unigene29231\_Mf\_liverA, NM\_010003, Unigene22052\_Mf\_liverA, Unigene34867\_Mf\_liverA, CL2478.Contig3\_Mf\_liverA, Unigene4938\_Mf\_liverA, Unigene5815\_Mf\_liverA, NM\_024474, Unigene18499\_Mf\_liverA, Unigene38870\_Mf\_liverA, Unigene38199\_Mf\_liverA, Unigene28662\_Mf\_liverA, Unigene4781\_Mf\_liverA, CL2439.Contig1\_Mf\_liverA, CL1988.Contig2\_Mf\_liverA, Unigene35935\_Mf\_liverA, Unigene29426\_Mf\_liverA, Unigene13462\_Mf\_liverA, Unigene20371\_Mf\_liverA, CL3900.Contig1\_Mf\_liverA, Unigene13683\_Mf\_liverA, Unigene9698\_Mf\_liverA, Unigene30585\_Mf\_liverA, Unigene36420\_Mf\_liverA, Unigene28499\_Mf\_liverA, Unigene542\_Mf\_liverA, Unigene24758\_Mf\_liverA, Unigene33080\_Mf\_liverA, Unigene30947\_Mf\_liverA, NM\_019879, CL5293.Contig2\_Mf\_liverA, Unigene35090\_Mf\_liverA, CL840.Contig1\_Mf\_liverA, NM\_145218, NM\_025336, Unigene4841\_Mf\_liverA, Unigene23185\_Mf\_liverA, NM\_010158, Unigene20372\_Mf\_liverA, Unigene29985\_Mf\_liverA, CL3104.Contig1\_Mf\_liverA, Unigene13018\_Mf\_liverA, Unigene26580\_Mf\_liverA, Unigene36593\_Mf\_liverA, Unigene26336\_Mf\_liverA, Unigene37616\_Mf\_liverA, NM\_146016, Unigene36673\_Mf\_liverA, CL5698.Contig1\_Mf\_liverA, NM\_001253832, Unigene6110\_Mf\_liverA, Unigene31392\_Mf\_liverA, NM\_023256, NR\_024097, CL591.Contig1\_Mf\_liverA, Unigene4556\_Mf\_liverA, NM\_007763, CL139.Contig2\_Mf\_liverA, Unigene26055\_Mf\_liverA, Unigene29399\_Mf\_liverA, Unigene33607\_Mf\_liverA, NM\_008292, Unigene38015\_Mf\_liverA, Unigene31199\_Mf\_liverA, Unigene6959\_Mf\_liverA, Unigene29308\_Mf\_liverA, CL523.Contig1\_Mf\_liverA, CL482.Contig1\_Mf\_liverA, Unigene22433\_Mf\_liverA, Unigene36762\_Mf\_liverA, CL3725.Contig1\_Mf\_liverA, NM\_007820, Unigene37698\_Mf\_liverA, Unigene30878\_Mf\_liverA, Unigene40289\_Mf\_liverA, CL44.Contig1\_Mf\_liverA, Unigene24344\_Mf\_liverA, Unigene37880\_Mf\_liverA, Unigene18500\_Mf\_liverA, Unigene38331\_Mf\_liverA, Unigene25070\_Mf\_liverA, Unigene24112\_Mf\_liverA, CL2001.Contig1\_Mf\_liverA, Unigene38110\_Mf\_liverA, Unigene26194\_Mf\_liverA, Unigene31333\_Mf\_liverA, Unigene30369\_Mf\_liverA, Unigene25596\_Mf\_liverA, Unigene843\_Mf\_liverA, Unigene12153\_Mf\_liverA, Unigene22432\_Mf\_liverA, Unigene25046\_Mf\_liverA, Unigene13918\_Mf\_liverA, CL5268.Contig1\_Mf\_liverA, Unigene2745\_Mf\_liverA, Unigene5775\_Mf\_liverA, CL4076.Contig1\_Mf\_liverA, CL787.Contig1\_Mf\_liverA, Unigene39507\_Mf\_liverA, Unigene25524\_Mf\_liverA, Unigene4\_Mf\_liverA, CL3750.Contig2\_Mf\_liverA, Unigene39141\_Mf\_liverA, Unigene33459\_Mf\_liverA, Unigene5632\_Mf\_liverA, Unigene36669\_Mf\_liverA, CL2632.Contig2\_Mf\_liverA, Unigene34746\_Mf\_liverA, Unigene14582\_Mf\_liverA, Unigene24503\_Mf\_liverA, Unigene15583\_Mf\_liverA, Unigene27422\_Mf\_liverA, CL4701.Contig1\_Mf\_liverA, Unigene36757\_Mf\_liverA, CL5631.Contig1\_Mf\_liverA, Unigene31427\_Mf\_liverA, Unigene30584\_Mf\_liverA, Unigene17579\_Mf\_liverA, CL4220.Contig2\_Mf\_liverA, Unigene431\_Mf\_liverA, NM\_001081372, NM\_010391, Unigene21317\_Mf\_liverA, NM\_010227, Unigene30493\_Mf\_liverA, Unigene8132\_Mf\_liverA, NM\_019717, Unigene37178\_Mf\_liverA, Unigene27082\_Mf\_liverA, Unigene5712\_Mf\_liverA, Unigene43107\_Mf\_liverA, CL442.Contig2\_Mf\_liverA, Unigene37497\_Mf\_liverA, NM\_010380, NM\_033374, CL2855.Contig1\_Mf\_liverA, CL4411.Contig4\_Mf\_liverA, NM\_001025388, NM\_013821, Unigene40796\_Mf\_liverA, Unigene13233\_Mf\_liverA, NM\_011099, NM\_001100182, NM\_010481, CL4086.Contig1\_Mf\_liverA, CL3822.Contig2\_Mf\_liverA, Unigene15318\_Mf\_liverA, Unigene36190\_Mf\_liverA, NM\_008293, Unigene7970\_Mf\_liverA, NM\_009255, Unigene15982\_Mf\_liverA, Unigene30154\_Mf\_liverA, CL4162.Contig1\_Mf\_liverA, Unigene35237\_Mf\_liverA, Unigene112\_Mf\_liverA, Unigene591\_Mf\_liverA, Unigene12907\_Mf\_liverA, Unigene25292\_Mf\_liverA, Unigene15588\_Mf\_liverA, CL5796.Contig2\_Mf\_liverA, NM\_011170, Unigene36414\_Mf\_liverA, Unigene36543\_Mf\_liverA, CL1988.Contig1\_Mf\_liverA, Unigene5175\_Mf\_liverA, Unigene13894\_Mf\_liverA, Unigene36328\_Mf\_liverA, NM\_010141, Unigene6895\_Mf\_liverA, CL6039.Contig2\_Mf\_liverA, CL887.Contig2\_Mf\_liverA, Unigene32695\_Mf\_liverA, Unigene5940\_Mf\_liverA, Unigene43357\_Mf\_liverA, Unigene28731\_Mf\_liverA, Unigene1212\_Mf\_liverA, CL3549.Contig1\_Mf\_liverA, Unigene32294\_Mf\_liverA, CL3750.Contig1\_Mf\_liverA, Unigene18796\_Mf\_liverA, Unigene21337\_Mf\_liverA, CL5307.Contig1\_Mf\_liverA, Unigene3377\_Mf\_liverA, Unigene28873\_Mf\_liverA, NM\_178405, Unigene33525\_Mf\_liverA, CL4048.Contig1\_Mf\_liverA, CL1052.Contig1\_Mf\_liverA, Unigene9406\_Mf\_liverA, Unigene120\_Mf\_liverA, CL4007.Contig1\_Mf\_liverA, Unigene15026\_Mf\_liverA, Unigene14940\_Mf\_liverA, Unigene1221\_Mf\_liverA, Unigene39875\_Mf\_liverA, Unigene1292\_Mf\_liverA, Unigene26309\_Mf\_liverA, Unigene32295\_Mf\_liverA, Unigene4636\_Mf\_liverA, Unigene5745\_Mf\_liverA, Unigene21336\_Mf\_liverA, Unigene38514\_Mf\_liverA, Unigene665\_Mf\_liverA, Unigene12889\_Mf\_liverA, NM\_001099634, Unigene8560\_Mf\_liverA, Unigene16891\_Mf\_liverA, Unigene12519\_Mf\_liverA, Unigene37819\_Mf\_liverA, Unigene5886\_Mf\_liverA, CL336.Contig3\_Mf\_liverA |
| lateral plasma membrane | Unigene27081\_Mf\_liverA, Unigene27082\_Mf\_liverA, Unigene23870\_Mf\_liverA |
| cell-cell contact zone | Unigene27081\_Mf\_liverA, Unigene27082\_Mf\_liverA, Unigene13950\_Mf\_liverA |
| cell | Unigene21684\_Mf\_liverA, NM\_176843, NM\_009898, Unigene22874\_Mf\_liverA, NM\_025593, Unigene24111\_Mf\_liverA, Unigene28186\_Mf\_liverA, Unigene15553\_Mf\_liverA, NM\_010378, CL854.Contig1\_Mf\_liverA, Unigene16463\_Mf\_liverA, NM\_025613, Unigene39886\_Mf\_liverA, Unigene15064\_Mf\_liverA, Unigene24252\_Mf\_liverA, CL4757.Contig1\_Mf\_liverA, NR\_004446, CL1555.Contig1\_Mf\_liverA, Unigene37488\_Mf\_liverA, Unigene32564\_Mf\_liverA, Unigene37535\_Mf\_liverA, CL695.Contig1\_Mf\_liverA, Unigene35431\_Mf\_liverA, Unigene15703\_Mf\_liverA, Unigene31623\_Mf\_liverA, NM\_010233, Unigene13379\_Mf\_liverA, Unigene34394\_Mf\_liverA, NM\_172409, Unigene25594\_Mf\_liverA, Unigene14050\_Mf\_liverA, CL4995.Contig1\_Mf\_liverA, Unigene8054\_Mf\_liverA, Unigene18430\_Mf\_liverA, Unigene5552\_Mf\_liverA, CL5807.Contig1\_Mf\_liverA, CL2384.Contig1\_Mf\_liverA, Unigene26065\_Mf\_liverA, Unigene36417\_Mf\_liverA, Unigene18125\_Mf\_liverA, NM\_177093, CL4293.Contig1\_Mf\_liverA, Unigene13097\_Mf\_liverA, Unigene25226\_Mf\_liverA, Unigene33512\_Mf\_liverA, Unigene14907\_Mf\_liverA, Unigene25976\_Mf\_liverA, Unigene36698\_Mf\_liverA, NM\_145942, Unigene30261\_Mf\_liverA, Unigene13945\_Mf\_liverA, Unigene139\_Mf\_liverA, Unigene28217\_Mf\_liverA, Unigene36836\_Mf\_liverA, Unigene33366\_Mf\_liverA, NM\_134156, Unigene37389\_Mf\_liverA, Unigene4720\_Mf\_liverA, CL4156.Contig1\_Mf\_liverA, Unigene27248\_Mf\_liverA, CL2266.Contig2\_Mf\_liverA, Unigene30839\_Mf\_liverA, Unigene34010\_Mf\_liverA, Unigene24613\_Mf\_liverA, Unigene152\_Mf\_liverA, Unigene31852\_Mf\_liverA, CL532.Contig1\_Mf\_liverA, Unigene37076\_Mf\_liverA, Unigene1280\_Mf\_liverA, Unigene30288\_Mf\_liverA, Unigene14171\_Mf\_liverA, CL275.Contig5\_Mf\_liverA, CL5576.Contig1\_Mf\_liverA, Unigene33632\_Mf\_liverA, CL5189.Contig1\_Mf\_liverA, CL3339.Contig1\_Mf\_liverA, Unigene2746\_Mf\_liverA, Unigene4593\_Mf\_liverA, Unigene10496\_Mf\_liverA, Unigene4363\_Mf\_liverA, CL425.Contig1\_Mf\_liverA, CL2797.Contig2\_Mf\_liverA, CL1803.Contig1\_Mf\_liverA, Unigene37243\_Mf\_liverA, Unigene30587\_Mf\_liverA, Unigene39403\_Mf\_liverA, Unigene15077\_Mf\_liverA, Unigene36034\_Mf\_liverA, NM\_009253, Unigene5693\_Mf\_liverA, Unigene39970\_Mf\_liverA, CL3393.Contig1\_Mf\_liverA, Unigene29876\_Mf\_liverA, CL3800.Contig1\_Mf\_liverA, Unigene26585\_Mf\_liverA, Unigene28687\_Mf\_liverA, CL422.Contig1\_Mf\_liverA, CL1988.Contig3\_Mf\_liverA, NM\_028785, Unigene5758\_Mf\_liverA, NM\_009609, CL1810.Contig1\_Mf\_liverA, CL4033.Contig1\_Mf\_liverA, Unigene24547\_Mf\_liverA, Unigene34983\_Mf\_liverA, NR\_003623, CL3519.Contig1\_Mf\_liverA, Unigene8033\_Mf\_liverA, CL1493.Contig1\_Mf\_liverA, CL5764.Contig1\_Mf\_liverA, Unigene35476\_Mf\_liverA, Unigene4909\_Mf\_liverA, Unigene24471\_Mf\_liverA, Unigene31571\_Mf\_liverA, NM\_010162, Unigene15529\_Mf\_liverA, CL4220.Contig1\_Mf\_liverA, Unigene37262\_Mf\_liverA, Unigene18126\_Mf\_liverA, NR\_033215, CL2251.Contig1\_Mf\_liverA, NM\_007822, CL5293.Contig1\_Mf\_liverA, CL4490.Contig2\_Mf\_liverA, Unigene25595\_Mf\_liverA, Unigene14286\_Mf\_liverA, Unigene32515\_Mf\_liverA, CL4816.Contig3\_Mf\_liverA, Unigene39011\_Mf\_liverA, Unigene20432\_Mf\_liverA, NM\_009022, Unigene35609\_Mf\_liverA, Unigene7048\_Mf\_liverA, Unigene13950\_Mf\_liverA, Unigene35046\_Mf\_liverA, Unigene28459\_Mf\_liverA, Unigene37245\_Mf\_liverA, Unigene13847\_Mf\_liverA, Unigene19658\_Mf\_liverA, NM\_027406, Unigene36699\_Mf\_liverA, Unigene36176\_Mf\_liverA, Unigene18340\_Mf\_liverA, Unigene496\_Mf\_liverA, Unigene42803\_Mf\_liverA, NM\_001164598, Unigene4983\_Mf\_liverA, Unigene29889\_Mf\_liverA, Unigene2939\_Mf\_liverA, Unigene9466\_Mf\_liverA, CL5640.Contig1\_Mf\_liverA, NM\_020559, Unigene35884\_Mf\_liverA, Unigene28142\_Mf\_liverA, CL3835.Contig2\_Mf\_liverA, Unigene38831\_Mf\_liverA, Unigene28899\_Mf\_liverA, Unigene14603\_Mf\_liverA, Unigene46870\_Mf\_liverA, Unigene24883\_Mf\_liverA, CL1575.Contig1\_Mf\_liverA, NM\_019651, Unigene13153\_Mf\_liverA, CL4117.Contig1\_Mf\_liverA, Unigene36849\_Mf\_liverA, Unigene8473\_Mf\_liverA, Unigene29008\_Mf\_liverA, Unigene25055\_Mf\_liverA, Unigene27593\_Mf\_liverA, CL2339.Contig1\_Mf\_liverA, Unigene29788\_Mf\_liverA, Unigene33508\_Mf\_liverA, CL777.Contig8\_Mf\_liverA, NM\_011072, Unigene14816\_Mf\_liverA, Unigene4776\_Mf\_liverA, Unigene21466\_Mf\_liverA, Unigene34810\_Mf\_liverA, Unigene27420\_Mf\_liverA, Unigene20602\_Mf\_liverA, Unigene36889\_Mf\_liverA, Unigene37999\_Mf\_liverA, Unigene40020\_Mf\_liverA, Unigene37904\_Mf\_liverA, Unigene41336\_Mf\_liverA, CL3483.Contig1\_Mf\_liverA, CL4141.Contig1\_Mf\_liverA, Unigene34184\_Mf\_liverA, CL5191.Contig2\_Mf\_liverA, Unigene13593\_Mf\_liverA, Unigene4686\_Mf\_liverA, Unigene11\_Mf\_liverA, Unigene4630\_Mf\_liverA, Unigene45131\_Mf\_liverA, Unigene5994\_Mf\_liverA, CL1493.Contig2\_Mf\_liverA, CL3575.Contig1\_Mf\_liverA, Unigene5294\_Mf\_liverA, Unigene5941\_Mf\_liverA, Unigene38104\_Mf\_liverA, Unigene38065\_Mf\_liverA, Unigene25462\_Mf\_liverA, Unigene36626\_Mf\_liverA, NM\_153795, Unigene28822\_Mf\_liverA, Unigene31988\_Mf\_liverA, Unigene5382\_Mf\_liverA, Unigene151\_Mf\_liverA, NM\_201360, CL848.Contig2\_Mf\_liverA, Unigene23870\_Mf\_liverA, Unigene13143\_Mf\_liverA, CL1263.Contig1\_Mf\_liverA, Unigene37460\_Mf\_liverA, Unigene31517\_Mf\_liverA, NM\_007811, Unigene24323\_Mf\_liverA, CL2142.Contig2\_Mf\_liverA, NM\_011305, CL3816.Contig1\_Mf\_liverA, Unigene25333\_Mf\_liverA, CL993.Contig2\_Mf\_liverA, CL529.Contig2\_Mf\_liverA, Unigene27081\_Mf\_liverA, Unigene37148\_Mf\_liverA, Unigene7195\_Mf\_liverA, Unigene1205\_Mf\_liverA, CL1352.Contig1\_Mf\_liverA, CL6039.Contig1\_Mf\_liverA, Unigene24804\_Mf\_liverA, Unigene31206\_Mf\_liverA, NM\_009128, CL2240.Contig1\_Mf\_liverA, Unigene29823\_Mf\_liverA, CL1125.Contig1\_Mf\_liverA, Unigene3752\_Mf\_liverA, Unigene38598\_Mf\_liverA, Unigene15681\_Mf\_liverA, Unigene5165\_Mf\_liverA, Unigene30707\_Mf\_liverA, NM\_133838, Unigene15552\_Mf\_liverA, CL3002.Contig1\_Mf\_liverA, Unigene30155\_Mf\_liverA, CL5057.Contig1\_Mf\_liverA, Unigene36514\_Mf\_liverA, NM\_145836, CL4816.Contig2\_Mf\_liverA, Unigene37139\_Mf\_liverA, Unigene33523\_Mf\_liverA, CL3207.Contig1\_Mf\_liverA, Unigene5906\_Mf\_liverA, NM\_001081148, Unigene14810\_Mf\_liverA, NM\_033444, Unigene4597\_Mf\_liverA, Unigene14170\_Mf\_liverA, CL2791.Contig1\_Mf\_liverA, CL4770.Contig1\_Mf\_liverA, Unigene10135\_Mf\_liverA, Unigene1483\_Mf\_liverA, CL114.Contig2\_Mf\_liverA, NM\_009883, Unigene33526\_Mf\_liverA, Unigene1327\_Mf\_liverA, Unigene39252\_Mf\_liverA, NM\_001081172, CL5978.Contig2\_Mf\_liverA, Unigene8740\_Mf\_liverA, Unigene14270\_Mf\_liverA, CL3816.Contig2\_Mf\_liverA, CL738.Contig2\_Mf\_liverA, CL442.Contig5\_Mf\_liverA, CL5459.Contig2\_Mf\_liverA, NM\_012030, Unigene24477\_Mf\_liverA, Unigene30983\_Mf\_liverA, Unigene30731\_Mf\_liverA, Unigene39749\_Mf\_liverA, Unigene36418\_Mf\_liverA, Unigene25052\_Mf\_liverA, CL3835.Contig1\_Mf\_liverA, Unigene38311\_Mf\_liverA, Unigene550\_Mf\_liverA, Unigene31198\_Mf\_liverA, CL2355.Contig1\_Mf\_liverA, Unigene28314\_Mf\_liverA, Unigene37153\_Mf\_liverA, Unigene14809\_Mf\_liverA, Unigene21562\_Mf\_liverA, Unigene15592\_Mf\_liverA, Unigene2195\_Mf\_liverA, Unigene30892\_Mf\_liverA, Unigene34866\_Mf\_liverA, Unigene31436\_Mf\_liverA, Unigene14765\_Mf\_liverA, Unigene4681\_Mf\_liverA, Unigene14284\_Mf\_liverA, Unigene5287\_Mf\_liverA, Unigene34609\_Mf\_liverA, NM\_144940, Unigene25721\_Mf\_liverA, CL3669.Contig2\_Mf\_liverA, Unigene37334\_Mf\_liverA, NM\_018815, NM\_080638, Unigene29424\_Mf\_liverA, NM\_011082, Unigene34727\_Mf\_liverA, CL4160.Contig2\_Mf\_liverA, CL4105.Contig1\_Mf\_liverA, Unigene26053\_Mf\_liverA, Unigene11007\_Mf\_liverA, CL4577.Contig1\_Mf\_liverA, Unigene785\_Mf\_liverA, Unigene38689\_Mf\_liverA, Unigene27339\_Mf\_liverA, NM\_021273, Unigene27294\_Mf\_liverA, Unigene1137\_Mf\_liverA, CL5586.Contig1\_Mf\_liverA, Unigene34341\_Mf\_liverA, Unigene30142\_Mf\_liverA, NM\_011921, Unigene35816\_Mf\_liverA, Unigene7897\_Mf\_liverA, CL2260.Contig1\_Mf\_liverA, NM\_145474, CL593.Contig2\_Mf\_liverA, NM\_009776, Unigene30003\_Mf\_liverA, Unigene5138\_Mf\_liverA, NM\_153193, NM\_031165, Unigene37150\_Mf\_liverA, Unigene14508\_Mf\_liverA, Unigene34143\_Mf\_liverA, Unigene30002\_Mf\_liverA, Unigene802\_Mf\_liverA, Unigene9150\_Mf\_liverA, Unigene17048\_Mf\_liverA, Unigene5639\_Mf\_liverA, Unigene33522\_Mf\_liverA, Unigene13363\_Mf\_liverA, CL2855.Contig2\_Mf\_liverA, NM\_021278, Unigene21561\_Mf\_liverA, NM\_010001, Unigene39655\_Mf\_liverA, Unigene4922\_Mf\_liverA, Unigene14637\_Mf\_liverA, NM\_153505, NM\_007622, NM\_001104531, Unigene4944\_Mf\_liverA, Unigene33560\_Mf\_liverA, Unigene23158\_Mf\_liverA, Unigene37575\_Mf\_liverA, Unigene25398\_Mf\_liverA, Unigene5774\_Mf\_liverA, Unigene27547\_Mf\_liverA, CL1736.Contig2\_Mf\_liverA, CL5316.Contig1\_Mf\_liverA, Unigene45530\_Mf\_liverA, Unigene4723\_Mf\_liverA, Unigene32332\_Mf\_liverA, Unigene29063\_Mf\_liverA, Unigene37470\_Mf\_liverA, CL5254.Contig1\_Mf\_liverA, Unigene35169\_Mf\_liverA, CL3166.Contig4\_Mf\_liverA, Unigene35698\_Mf\_liverA, Unigene32436\_Mf\_liverA, Unigene5169\_Mf\_liverA, Unigene16465\_Mf\_liverA, Unigene30528\_Mf\_liverA, NM\_007678, NM\_010392, Unigene31251\_Mf\_liverA, Unigene40610\_Mf\_liverA, CL186.Contig3\_Mf\_liverA, CL4925.Contig1\_Mf\_liverA, Unigene32421\_Mf\_liverA, Unigene583\_Mf\_liverA, CL4736.Contig1\_Mf\_liverA, Unigene27026\_Mf\_liverA, Unigene14916\_Mf\_liverA, Unigene35858\_Mf\_liverA, CL4600.Contig1\_Mf\_liverA, Unigene29231\_Mf\_liverA, NM\_010003, Unigene22052\_Mf\_liverA, Unigene34867\_Mf\_liverA, CL2478.Contig3\_Mf\_liverA, Unigene4938\_Mf\_liverA, Unigene5815\_Mf\_liverA, NM\_024474, Unigene18499\_Mf\_liverA, Unigene38870\_Mf\_liverA, Unigene38199\_Mf\_liverA, Unigene28662\_Mf\_liverA, Unigene4781\_Mf\_liverA, CL2439.Contig1\_Mf\_liverA, CL1988.Contig2\_Mf\_liverA, Unigene35935\_Mf\_liverA, Unigene29426\_Mf\_liverA, Unigene13462\_Mf\_liverA, Unigene20371\_Mf\_liverA, CL3900.Contig1\_Mf\_liverA, Unigene13683\_Mf\_liverA, Unigene9698\_Mf\_liverA, Unigene30585\_Mf\_liverA, Unigene36420\_Mf\_liverA, Unigene28499\_Mf\_liverA, Unigene542\_Mf\_liverA, Unigene24758\_Mf\_liverA, Unigene33080\_Mf\_liverA, Unigene30947\_Mf\_liverA, NM\_019879, CL5293.Contig2\_Mf\_liverA, Unigene35090\_Mf\_liverA, CL840.Contig1\_Mf\_liverA, NM\_145218, NM\_025336, Unigene4841\_Mf\_liverA, Unigene23185\_Mf\_liverA, NM\_010158, Unigene20372\_Mf\_liverA, Unigene29985\_Mf\_liverA, CL3104.Contig1\_Mf\_liverA, Unigene13018\_Mf\_liverA, Unigene26580\_Mf\_liverA, Unigene36593\_Mf\_liverA, Unigene26336\_Mf\_liverA, Unigene37616\_Mf\_liverA, NM\_146016, Unigene36673\_Mf\_liverA, CL5698.Contig1\_Mf\_liverA, NM\_001253832, Unigene6110\_Mf\_liverA, Unigene31392\_Mf\_liverA, NM\_023256, NR\_024097, CL591.Contig1\_Mf\_liverA, Unigene4556\_Mf\_liverA, NM\_007763, CL139.Contig2\_Mf\_liverA, Unigene26055\_Mf\_liverA, Unigene29399\_Mf\_liverA, Unigene33607\_Mf\_liverA, NM\_008292, Unigene38015\_Mf\_liverA, Unigene31199\_Mf\_liverA, Unigene6959\_Mf\_liverA, Unigene29308\_Mf\_liverA, CL523.Contig1\_Mf\_liverA, CL482.Contig1\_Mf\_liverA, Unigene22433\_Mf\_liverA, Unigene36762\_Mf\_liverA, CL3725.Contig1\_Mf\_liverA, NM\_007820, Unigene37698\_Mf\_liverA, Unigene30878\_Mf\_liverA, Unigene40289\_Mf\_liverA, CL44.Contig1\_Mf\_liverA, Unigene24344\_Mf\_liverA, Unigene37880\_Mf\_liverA, Unigene18500\_Mf\_liverA, Unigene38331\_Mf\_liverA, Unigene25070\_Mf\_liverA, Unigene24112\_Mf\_liverA, CL2001.Contig1\_Mf\_liverA, Unigene38110\_Mf\_liverA, Unigene26194\_Mf\_liverA, Unigene31333\_Mf\_liverA, Unigene30369\_Mf\_liverA, Unigene25596\_Mf\_liverA, Unigene843\_Mf\_liverA, Unigene12153\_Mf\_liverA, Unigene22432\_Mf\_liverA, Unigene25046\_Mf\_liverA, Unigene13918\_Mf\_liverA, CL5268.Contig1\_Mf\_liverA, Unigene2745\_Mf\_liverA, Unigene5775\_Mf\_liverA, CL4076.Contig1\_Mf\_liverA, CL787.Contig1\_Mf\_liverA, Unigene39507\_Mf\_liverA, Unigene25524\_Mf\_liverA, Unigene4\_Mf\_liverA, CL3750.Contig2\_Mf\_liverA, Unigene39141\_Mf\_liverA, Unigene33459\_Mf\_liverA, Unigene5632\_Mf\_liverA, Unigene36669\_Mf\_liverA, CL2632.Contig2\_Mf\_liverA, Unigene34746\_Mf\_liverA, Unigene14582\_Mf\_liverA, Unigene24503\_Mf\_liverA, Unigene15583\_Mf\_liverA, Unigene27422\_Mf\_liverA, CL4701.Contig1\_Mf\_liverA, Unigene36757\_Mf\_liverA, CL5631.Contig1\_Mf\_liverA, Unigene31427\_Mf\_liverA, Unigene30584\_Mf\_liverA, Unigene17579\_Mf\_liverA, CL4220.Contig2\_Mf\_liverA, Unigene431\_Mf\_liverA, NM\_001081372, NM\_010391, Unigene21317\_Mf\_liverA, NM\_010227, Unigene30493\_Mf\_liverA, Unigene8132\_Mf\_liverA, NM\_019717, Unigene37178\_Mf\_liverA, Unigene27082\_Mf\_liverA, Unigene5712\_Mf\_liverA, Unigene43107\_Mf\_liverA, CL442.Contig2\_Mf\_liverA, Unigene37497\_Mf\_liverA, NM\_010380, NM\_033374, CL2855.Contig1\_Mf\_liverA, CL4411.Contig4\_Mf\_liverA, NM\_001025388, NM\_013821, Unigene40796\_Mf\_liverA, Unigene13233\_Mf\_liverA, NM\_011099, NM\_001100182, NM\_010481, CL4086.Contig1\_Mf\_liverA, CL3822.Contig2\_Mf\_liverA, Unigene15318\_Mf\_liverA, Unigene36190\_Mf\_liverA, NM\_008293, Unigene7970\_Mf\_liverA, NM\_009255, Unigene15982\_Mf\_liverA, Unigene30154\_Mf\_liverA, CL4162.Contig1\_Mf\_liverA, Unigene35237\_Mf\_liverA, Unigene112\_Mf\_liverA, Unigene591\_Mf\_liverA, Unigene12907\_Mf\_liverA, Unigene25292\_Mf\_liverA, Unigene15588\_Mf\_liverA, CL5796.Contig2\_Mf\_liverA, NM\_011170, Unigene36414\_Mf\_liverA, Unigene36543\_Mf\_liverA, CL1988.Contig1\_Mf\_liverA, Unigene5175\_Mf\_liverA, Unigene13894\_Mf\_liverA, Unigene36328\_Mf\_liverA, NM\_010141, Unigene6895\_Mf\_liverA, CL6039.Contig2\_Mf\_liverA, CL887.Contig2\_Mf\_liverA, Unigene32695\_Mf\_liverA, Unigene5940\_Mf\_liverA, Unigene43357\_Mf\_liverA, Unigene28731\_Mf\_liverA, Unigene1212\_Mf\_liverA, CL3549.Contig1\_Mf\_liverA, Unigene32294\_Mf\_liverA, CL3750.Contig1\_Mf\_liverA, Unigene18796\_Mf\_liverA, Unigene21337\_Mf\_liverA, CL5307.Contig1\_Mf\_liverA, Unigene3377\_Mf\_liverA, Unigene28873\_Mf\_liverA, NM\_178405, Unigene33525\_Mf\_liverA, CL4048.Contig1\_Mf\_liverA, CL1052.Contig1\_Mf\_liverA, Unigene9406\_Mf\_liverA, Unigene120\_Mf\_liverA, CL4007.Contig1\_Mf\_liverA, Unigene15026\_Mf\_liverA, Unigene14940\_Mf\_liverA, Unigene1221\_Mf\_liverA, Unigene39875\_Mf\_liverA, Unigene1292\_Mf\_liverA, Unigene26309\_Mf\_liverA, Unigene32295\_Mf\_liverA, Unigene4636\_Mf\_liverA, Unigene5745\_Mf\_liverA, Unigene21336\_Mf\_liverA, Unigene38514\_Mf\_liverA, Unigene665\_Mf\_liverA, Unigene12889\_Mf\_liverA, NM\_001099634, Unigene8560\_Mf\_liverA, Unigene16891\_Mf\_liverA, Unigene12519\_Mf\_liverA, Unigene37819\_Mf\_liverA, Unigene5886\_Mf\_liverA, CL336.Contig3\_Mf\_liverA |
| ER to Golgi transport vesicle membrane | CL2855.Contig1\_Mf\_liverA, CL2855.Contig2\_Mf\_liverA |
| clathrin-coated endocytic vesicle membrane | CL2855.Contig1\_Mf\_liverA, CL2855.Contig2\_Mf\_liverA |
| cell body | Unigene29399\_Mf\_liverA, Unigene18500\_Mf\_liverA, Unigene27547\_Mf\_liverA, NM\_010141, Unigene4720\_Mf\_liverA, CL1810.Contig1\_Mf\_liverA, CL425.Contig1\_Mf\_liverA, Unigene8132\_Mf\_liverA, NM\_009255, Unigene8740\_Mf\_liverA, Unigene13593\_Mf\_liverA, Unigene4686\_Mf\_liverA, Unigene34866\_Mf\_liverA, Unigene37076\_Mf\_liverA, Unigene5138\_Mf\_liverA, CL2632.Contig2\_Mf\_liverA, Unigene5712\_Mf\_liverA, Unigene14582\_Mf\_liverA, CL3166.Contig4\_Mf\_liverA, Unigene34867\_Mf\_liverA, Unigene5165\_Mf\_liverA, Unigene13950\_Mf\_liverA |
| photoreceptor outer segment | Unigene32294\_Mf\_liverA, Unigene32295\_Mf\_liverA |
| nuclear outer membrane | CL2478.Contig3\_Mf\_liverA, Unigene37535\_Mf\_liverA |
| ruffle membrane | Unigene30707\_Mf\_liverA, Unigene5693\_Mf\_liverA, Unigene5138\_Mf\_liverA, CL3207.Contig1\_Mf\_liverA |
| acrosomal vesicle | CL3816.Contig2\_Mf\_liverA, CL3816.Contig1\_Mf\_liverA, Unigene36757\_Mf\_liverA, Unigene33080\_Mf\_liverA, Unigene13950\_Mf\_liverA |
| Golgi apparatus part | CL787.Contig1\_Mf\_liverA, Unigene29399\_Mf\_liverA, Unigene27081\_Mf\_liverA, CL5586.Contig1\_Mf\_liverA, Unigene36328\_Mf\_liverA, Unigene41336\_Mf\_liverA, CL2855.Contig2\_Mf\_liverA, Unigene15529\_Mf\_liverA, Unigene37389\_Mf\_liverA, Unigene37535\_Mf\_liverA, NM\_010227, CL4925.Contig1\_Mf\_liverA, Unigene5382\_Mf\_liverA, CL4033.Contig1\_Mf\_liverA, Unigene4922\_Mf\_liverA, Unigene34010\_Mf\_liverA, Unigene5632\_Mf\_liverA, CL3816.Contig2\_Mf\_liverA, Unigene36669\_Mf\_liverA, Unigene27082\_Mf\_liverA, Unigene112\_Mf\_liverA, CL4600.Contig1\_Mf\_liverA, CL3816.Contig1\_Mf\_liverA, CL2855.Contig1\_Mf\_liverA, Unigene28499\_Mf\_liverA, Unigene1221\_Mf\_liverA |
| clathrin-coated vesicle | Unigene36669\_Mf\_liverA, Unigene34394\_Mf\_liverA, CL5807.Contig1\_Mf\_liverA, NM\_031165, Unigene30587\_Mf\_liverA, Unigene13363\_Mf\_liverA, CL2855.Contig2\_Mf\_liverA, CL4925.Contig1\_Mf\_liverA, CL2855.Contig1\_Mf\_liverA, Unigene30585\_Mf\_liverA, Unigene30584\_Mf\_liverA |
| extrinsic to plasma membrane | CL4156.Contig1\_Mf\_liverA, CL1052.Contig1\_Mf\_liverA, Unigene37243\_Mf\_liverA, Unigene4636\_Mf\_liverA, Unigene591\_Mf\_liverA |
| adherens junction | Unigene30707\_Mf\_liverA, Unigene39875\_Mf\_liverA, Unigene27081\_Mf\_liverA, Unigene31852\_Mf\_liverA, CL1052.Contig1\_Mf\_liverA, Unigene27082\_Mf\_liverA, CL422.Contig1\_Mf\_liverA, Unigene32564\_Mf\_liverA, NM\_134156, Unigene2746\_Mf\_liverA, Unigene2745\_Mf\_liverA, Unigene29424\_Mf\_liverA, Unigene13950\_Mf\_liverA |
| cell junction | Unigene21684\_Mf\_liverA, CL5807.Contig1\_Mf\_liverA, Unigene25226\_Mf\_liverA, Unigene39252\_Mf\_liverA, Unigene23870\_Mf\_liverA, Unigene29424\_Mf\_liverA, Unigene25055\_Mf\_liverA, Unigene14637\_Mf\_liverA, CL1052.Contig1\_Mf\_liverA, Unigene112\_Mf\_liverA, Unigene39011\_Mf\_liverA, Unigene20432\_Mf\_liverA, Unigene5693\_Mf\_liverA, Unigene25052\_Mf\_liverA, Unigene13950\_Mf\_liverA, Unigene30707\_Mf\_liverA, Unigene39875\_Mf\_liverA, Unigene27081\_Mf\_liverA, CL5586.Contig1\_Mf\_liverA, CL422.Contig1\_Mf\_liverA, NM\_134156, Unigene32564\_Mf\_liverA, CL4033.Contig1\_Mf\_liverA, Unigene38870\_Mf\_liverA, Unigene13593\_Mf\_liverA, CL2260.Contig1\_Mf\_liverA, Unigene31852\_Mf\_liverA, Unigene27082\_Mf\_liverA, Unigene34394\_Mf\_liverA, Unigene14765\_Mf\_liverA, CL5576.Contig1\_Mf\_liverA, Unigene2746\_Mf\_liverA, Unigene2745\_Mf\_liverA |
| striated muscle thin filament | Unigene5639\_Mf\_liverA, Unigene4556\_Mf\_liverA |
| cell projection part | Unigene30707\_Mf\_liverA, Unigene18500\_Mf\_liverA, Unigene27547\_Mf\_liverA, Unigene31251\_Mf\_liverA, Unigene13363\_Mf\_liverA, Unigene4720\_Mf\_liverA, Unigene21562\_Mf\_liverA, CL425.Contig1\_Mf\_liverA, Unigene24883\_Mf\_liverA, Unigene21561\_Mf\_liverA, Unigene24547\_Mf\_liverA, Unigene8132\_Mf\_liverA, Unigene29424\_Mf\_liverA, Unigene13593\_Mf\_liverA, Unigene496\_Mf\_liverA, Unigene34866\_Mf\_liverA, Unigene31852\_Mf\_liverA, Unigene5138\_Mf\_liverA, CL3207.Contig1\_Mf\_liverA, Unigene14582\_Mf\_liverA, CL1352.Contig1\_Mf\_liverA, Unigene5693\_Mf\_liverA, Unigene34867\_Mf\_liverA, Unigene13950\_Mf\_liverA, Unigene24252\_Mf\_liverA |
| mitochondrial inner membrane | Unigene25398\_Mf\_liverA, CL738.Contig2\_Mf\_liverA, CL1125.Contig1\_Mf\_liverA, Unigene17048\_Mf\_liverA, CL4490.Contig2\_Mf\_liverA, Unigene43357\_Mf\_liverA, CL5316.Contig1\_Mf\_liverA, CL2797.Contig2\_Mf\_liverA, Unigene3377\_Mf\_liverA, Unigene34727\_Mf\_liverA, Unigene4781\_Mf\_liverA, Unigene13233\_Mf\_liverA, Unigene23185\_Mf\_liverA, CL5796.Contig2\_Mf\_liverA |
| pigment granule | CL3816.Contig2\_Mf\_liverA, CL3816.Contig1\_Mf\_liverA, NM\_031165, Unigene36190\_Mf\_liverA, Unigene36762\_Mf\_liverA, CL2001.Contig1\_Mf\_liverA |
| clathrin-coated endocytic vesicle | CL2855.Contig1\_Mf\_liverA, CL2855.Contig2\_Mf\_liverA |
| Golgi-associated vesicle membrane | Unigene27081\_Mf\_liverA, Unigene27082\_Mf\_liverA, Unigene5632\_Mf\_liverA |
| protein complex | Unigene9466\_Mf\_liverA, NM\_176843, Unigene35884\_Mf\_liverA, NM\_009898, CL3835.Contig2\_Mf\_liverA, Unigene6959\_Mf\_liverA, NM\_025593, Unigene24111\_Mf\_liverA, Unigene28186\_Mf\_liverA, Unigene29308\_Mf\_liverA, NM\_010378, CL4160.Contig2\_Mf\_liverA, Unigene36849\_Mf\_liverA, Unigene29008\_Mf\_liverA, CL2339.Contig1\_Mf\_liverA, Unigene22433\_Mf\_liverA, Unigene29788\_Mf\_liverA, CL777.Contig8\_Mf\_liverA, Unigene30878\_Mf\_liverA, CL4757.Contig1\_Mf\_liverA, NR\_004446, Unigene18500\_Mf\_liverA, Unigene38331\_Mf\_liverA, Unigene36889\_Mf\_liverA, Unigene41336\_Mf\_liverA, Unigene24112\_Mf\_liverA, CL4141.Contig1\_Mf\_liverA, Unigene35816\_Mf\_liverA, Unigene31333\_Mf\_liverA, CL2260.Contig1\_Mf\_liverA, Unigene4686\_Mf\_liverA, Unigene843\_Mf\_liverA, Unigene5138\_Mf\_liverA, Unigene12153\_Mf\_liverA, Unigene37150\_Mf\_liverA, Unigene22432\_Mf\_liverA, Unigene25046\_Mf\_liverA, CL5268.Contig1\_Mf\_liverA, Unigene5294\_Mf\_liverA, Unigene5941\_Mf\_liverA, Unigene2745\_Mf\_liverA, Unigene5639\_Mf\_liverA, Unigene28822\_Mf\_liverA, CL2855.Contig2\_Mf\_liverA, NM\_177093, Unigene14907\_Mf\_liverA, Unigene23870\_Mf\_liverA, Unigene39655\_Mf\_liverA, Unigene25976\_Mf\_liverA, Unigene13143\_Mf\_liverA, Unigene5632\_Mf\_liverA, Unigene31517\_Mf\_liverA, NM\_153505, Unigene13945\_Mf\_liverA, Unigene15583\_Mf\_liverA, CL4701.Contig1\_Mf\_liverA, CL5631.Contig1\_Mf\_liverA, NM\_153589, Unigene37575\_Mf\_liverA, Unigene27081\_Mf\_liverA, Unigene27547\_Mf\_liverA, CL4040.Contig2\_Mf\_liverA, Unigene33366\_Mf\_liverA, NM\_010391, Unigene45530\_Mf\_liverA, Unigene29063\_Mf\_liverA, Unigene152\_Mf\_liverA, CL532.Contig1\_Mf\_liverA, Unigene27082\_Mf\_liverA, Unigene37076\_Mf\_liverA, Unigene43107\_Mf\_liverA, Unigene30288\_Mf\_liverA, Unigene32436\_Mf\_liverA, NM\_010380, CL275.Contig5\_Mf\_liverA, CL5576.Contig1\_Mf\_liverA, CL5189.Contig1\_Mf\_liverA, CL2855.Contig1\_Mf\_liverA, Unigene2746\_Mf\_liverA, NM\_001025388, NM\_007678, NM\_010392, CL4086.Contig1\_Mf\_liverA, Unigene40610\_Mf\_liverA, CL4925.Contig1\_Mf\_liverA, CL425.Contig1\_Mf\_liverA, Unigene15982\_Mf\_liverA, CL4162.Contig1\_Mf\_liverA, Unigene591\_Mf\_liverA, CL4040.Contig1\_Mf\_liverA, Unigene29231\_Mf\_liverA, Unigene5165\_Mf\_liverA, CL3800.Contig1\_Mf\_liverA, Unigene30707\_Mf\_liverA, Unigene36543\_Mf\_liverA, Unigene4938\_Mf\_liverA, CL3002.Contig1\_Mf\_liverA, CL1810.Contig1\_Mf\_liverA, NM\_009609, Unigene5758\_Mf\_liverA, Unigene38870\_Mf\_liverA, CL887.Contig2\_Mf\_liverA, Unigene35935\_Mf\_liverA, Unigene29426\_Mf\_liverA, Unigene5940\_Mf\_liverA, CL3900.Contig1\_Mf\_liverA, NM\_033444, Unigene13683\_Mf\_liverA, Unigene24471\_Mf\_liverA, Unigene13106\_Mf\_liverA, Unigene39252\_Mf\_liverA, NM\_019879, Unigene8740\_Mf\_liverA, CL2251.Contig1\_Mf\_liverA, Unigene14270\_Mf\_liverA, Unigene39749\_Mf\_liverA, CL3835.Contig1\_Mf\_liverA, Unigene13950\_Mf\_liverA, Unigene37245\_Mf\_liverA, CL2355.Contig1\_Mf\_liverA, Unigene37153\_Mf\_liverA, CL3104.Contig1\_Mf\_liverA, Unigene19658\_Mf\_liverA, Unigene4636\_Mf\_liverA, Unigene18340\_Mf\_liverA, Unigene4983\_Mf\_liverA, CL5698.Contig1\_Mf\_liverA, NM\_023256, Unigene31392\_Mf\_liverA, Unigene4681\_Mf\_liverA, Unigene14284\_Mf\_liverA, CL591.Contig1\_Mf\_liverA, Unigene4556\_Mf\_liverA |
| caveola | Unigene35237\_Mf\_liverA, CL5631.Contig1\_Mf\_liverA, CL887.Contig2\_Mf\_liverA, Unigene12889\_Mf\_liverA |
| integral to endoplasmic reticulum membrane | CL695.Contig1\_Mf\_liverA, CL2855.Contig1\_Mf\_liverA, Unigene10135\_Mf\_liverA, CL2855.Contig2\_Mf\_liverA |
| synaptic vesicle membrane | Unigene34394\_Mf\_liverA, CL5807.Contig1\_Mf\_liverA |
| trans-Golgi network | CL2855.Contig1\_Mf\_liverA, Unigene41336\_Mf\_liverA, CL2855.Contig2\_Mf\_liverA, Unigene31988\_Mf\_liverA, CL4925.Contig1\_Mf\_liverA |
| leading edge membrane | Unigene30707\_Mf\_liverA, Unigene31852\_Mf\_liverA, Unigene5693\_Mf\_liverA, Unigene5138\_Mf\_liverA, Unigene24883\_Mf\_liverA, CL3207.Contig1\_Mf\_liverA |
| H4/H2A histone acetyltransferase complex | NM\_177093, NM\_009609 |
| mitochondrial membrane | Unigene25398\_Mf\_liverA, Unigene27081\_Mf\_liverA, Unigene17048\_Mf\_liverA, CL5316.Contig1\_Mf\_liverA, CL1810.Contig1\_Mf\_liverA, CL3104.Contig1\_Mf\_liverA, CL2797.Contig2\_Mf\_liverA, Unigene3377\_Mf\_liverA, Unigene34727\_Mf\_liverA, Unigene4781\_Mf\_liverA, CL738.Contig2\_Mf\_liverA, CL4490.Contig2\_Mf\_liverA, Unigene27082\_Mf\_liverA, CL1125.Contig1\_Mf\_liverA, Unigene43357\_Mf\_liverA, Unigene14508\_Mf\_liverA, CL5631.Contig1\_Mf\_liverA, Unigene25462\_Mf\_liverA, Unigene13233\_Mf\_liverA, Unigene23185\_Mf\_liverA, CL5796.Contig2\_Mf\_liverA |
| ER to Golgi transport vesicle | CL2855.Contig1\_Mf\_liverA, CL2855.Contig2\_Mf\_liverA |
| nuclear matrix | CL4162.Contig1\_Mf\_liverA, Unigene37076\_Mf\_liverA, Unigene5165\_Mf\_liverA, CL4757.Contig1\_Mf\_liverA |
| transport vesicle | Unigene36669\_Mf\_liverA, Unigene37819\_Mf\_liverA, CL2855.Contig1\_Mf\_liverA, Unigene8054\_Mf\_liverA, Unigene41336\_Mf\_liverA, CL2855.Contig2\_Mf\_liverA |
| DNA-directed RNA polymerase II, holoenzyme | NM\_025593, NM\_176843, Unigene33366\_Mf\_liverA, CL4757.Contig1\_Mf\_liverA |
| Golgi lumen | Unigene29399\_Mf\_liverA, Unigene5382\_Mf\_liverA |
| perikaryon | CL2632.Contig2\_Mf\_liverA, Unigene5138\_Mf\_liverA |
| coated vesicle | Unigene36669\_Mf\_liverA, Unigene34394\_Mf\_liverA, CL5807.Contig1\_Mf\_liverA, NM\_031165, Unigene30587\_Mf\_liverA, Unigene13363\_Mf\_liverA, CL2855.Contig2\_Mf\_liverA, CL4925.Contig1\_Mf\_liverA, CL2855.Contig1\_Mf\_liverA, Unigene30585\_Mf\_liverA, Unigene5632\_Mf\_liverA, Unigene30584\_Mf\_liverA |
| anchoring junction | Unigene30707\_Mf\_liverA, Unigene39875\_Mf\_liverA, Unigene27081\_Mf\_liverA, Unigene31852\_Mf\_liverA, CL1052.Contig1\_Mf\_liverA, Unigene27082\_Mf\_liverA, CL422.Contig1\_Mf\_liverA, Unigene32564\_Mf\_liverA, NM\_134156, Unigene2746\_Mf\_liverA, Unigene2745\_Mf\_liverA, Unigene29424\_Mf\_liverA, Unigene13950\_Mf\_liverA |
| sarcolemma | CL3816.Contig2\_Mf\_liverA, CL3816.Contig1\_Mf\_liverA, Unigene27547\_Mf\_liverA, CL425.Contig1\_Mf\_liverA, CL887.Contig2\_Mf\_liverA |
| Golgi cisterna | Unigene36669\_Mf\_liverA, Unigene37535\_Mf\_liverA, Unigene5632\_Mf\_liverA |
| organelle subcompartment | Unigene36669\_Mf\_liverA, Unigene37535\_Mf\_liverA, Unigene5632\_Mf\_liverA |
| dendritic spine | Unigene30707\_Mf\_liverA, Unigene27547\_Mf\_liverA, CL425.Contig1\_Mf\_liverA, Unigene24883\_Mf\_liverA, Unigene13593\_Mf\_liverA, Unigene24252\_Mf\_liverA |
| neuron spine | Unigene30707\_Mf\_liverA, Unigene27547\_Mf\_liverA, CL425.Contig1\_Mf\_liverA, Unigene24883\_Mf\_liverA, Unigene13593\_Mf\_liverA, Unigene24252\_Mf\_liverA |
| nuclear periphery | CL4162.Contig1\_Mf\_liverA, Unigene37076\_Mf\_liverA, NM\_009883, NM\_007678, Unigene5165\_Mf\_liverA, CL4757.Contig1\_Mf\_liverA |
| integral to organelle membrane | CL787.Contig1\_Mf\_liverA, CL695.Contig1\_Mf\_liverA, CL3104.Contig1\_Mf\_liverA, CL2855.Contig1\_Mf\_liverA, Unigene10135\_Mf\_liverA, CL2855.Contig2\_Mf\_liverA |
| DNA-directed RNA polymerase complex | NM\_025593, NM\_176843, Unigene33366\_Mf\_liverA, CL4757.Contig1\_Mf\_liverA |
| nuclear DNA-directed RNA polymerase complex | NM\_025593, NM\_176843, Unigene33366\_Mf\_liverA, CL4757.Contig1\_Mf\_liverA |
| pore complex | Unigene14270\_Mf\_liverA, CL3104.Contig1\_Mf\_liverA, CL2339.Contig1\_Mf\_liverA |
| AP-type membrane coat adaptor complex | Unigene41336\_Mf\_liverA, CL4925.Contig1\_Mf\_liverA |
| transport vesicle membrane | CL2855.Contig1\_Mf\_liverA, Unigene8054\_Mf\_liverA, CL2855.Contig2\_Mf\_liverA |
| site of polarized growth | CL1352.Contig1\_Mf\_liverA, Unigene21562\_Mf\_liverA, Unigene31251\_Mf\_liverA, Unigene21561\_Mf\_liverA, Unigene24547\_Mf\_liverA, Unigene14582\_Mf\_liverA, Unigene13950\_Mf\_liverA |
| extrinsic to internal side of plasma membrane | CL1052.Contig1\_Mf\_liverA, Unigene4636\_Mf\_liverA, Unigene591\_Mf\_liverA |
| basal plasma membrane | Unigene38870\_Mf\_liverA, Unigene39252\_Mf\_liverA |
| organelle inner membrane | Unigene25398\_Mf\_liverA, Unigene17048\_Mf\_liverA, CL5316.Contig1\_Mf\_liverA, NM\_008293, CL2797.Contig2\_Mf\_liverA, Unigene3377\_Mf\_liverA, NM\_019879, Unigene34727\_Mf\_liverA, Unigene4781\_Mf\_liverA, CL738.Contig2\_Mf\_liverA, CL4490.Contig2\_Mf\_liverA, CL1125.Contig1\_Mf\_liverA, NM\_153193, Unigene43357\_Mf\_liverA, NM\_025336, NM\_013821, Unigene13233\_Mf\_liverA, Unigene23185\_Mf\_liverA, CL5796.Contig2\_Mf\_liverA |
| RNA polymerase complex | NM\_025593, NM\_176843, Unigene33366\_Mf\_liverA, CL4757.Contig1\_Mf\_liverA |
| replication fork | Unigene31392\_Mf\_liverA, CL4757.Contig1\_Mf\_liverA |
| endoplasmic reticulum-Golgi intermediate compartment | Unigene37535\_Mf\_liverA, Unigene5632\_Mf\_liverA |
| basal part of cell | Unigene38870\_Mf\_liverA, Unigene39252\_Mf\_liverA |
| stress fiber | Unigene14637\_Mf\_liverA, Unigene4556\_Mf\_liverA |
| clathrin coat | Unigene41336\_Mf\_liverA, CL4925.Contig1\_Mf\_liverA |
| cell projection membrane | Unigene30707\_Mf\_liverA, Unigene31852\_Mf\_liverA, Unigene5693\_Mf\_liverA, Unigene5138\_Mf\_liverA, Unigene24883\_Mf\_liverA, CL3207.Contig1\_Mf\_liverA, Unigene29424\_Mf\_liverA |
| ruffle | Unigene30707\_Mf\_liverA, Unigene5693\_Mf\_liverA, Unigene5175\_Mf\_liverA, Unigene5138\_Mf\_liverA, CL3207.Contig1\_Mf\_liverA, Unigene24252\_Mf\_liverA |
| lipid particle | Unigene25596\_Mf\_liverA, Unigene43357\_Mf\_liverA |
| intrinsic to organelle membrane | CL787.Contig1\_Mf\_liverA, CL695.Contig1\_Mf\_liverA, CL3104.Contig1\_Mf\_liverA, CL2855.Contig1\_Mf\_liverA, Unigene32695\_Mf\_liverA, NM\_009128, NM\_010162, Unigene10135\_Mf\_liverA, CL2855.Contig2\_Mf\_liverA |
| myelin sheath | Unigene5712\_Mf\_liverA, CL2260.Contig1\_Mf\_liverA |
| flagellum | Unigene31206\_Mf\_liverA, CL2251.Contig1\_Mf\_liverA |
| histone deacetylase complex | Unigene31517\_Mf\_liverA, Unigene43107\_Mf\_liverA, CL4160.Contig2\_Mf\_liverA |
| basement membrane | Unigene38870\_Mf\_liverA, Unigene39252\_Mf\_liverA, Unigene8132\_Mf\_liverA, Unigene14582\_Mf\_liverA |
| focal adhesion | Unigene31852\_Mf\_liverA, CL1052.Contig1\_Mf\_liverA, Unigene32564\_Mf\_liverA, Unigene29424\_Mf\_liverA |
| I band | Unigene4556\_Mf\_liverA, NM\_134156, CL2251.Contig1\_Mf\_liverA, Unigene13593\_Mf\_liverA |
| ion channel complex | Unigene5758\_Mf\_liverA, Unigene18500\_Mf\_liverA, NM\_153589, NM\_033444 |
| Golgi stack | Unigene36669\_Mf\_liverA, Unigene37535\_Mf\_liverA, Unigene5632\_Mf\_liverA |
| actomyosin | Unigene14637\_Mf\_liverA, Unigene4556\_Mf\_liverA |
| cell-substrate junction | Unigene31852\_Mf\_liverA, CL1052.Contig1\_Mf\_liverA, Unigene38870\_Mf\_liverA, Unigene39252\_Mf\_liverA, Unigene32564\_Mf\_liverA, Unigene29424\_Mf\_liverA, NM\_134156 |
| tight junction | Unigene25226\_Mf\_liverA, Unigene14765\_Mf\_liverA, Unigene13950\_Mf\_liverA |
| membrane coat | Unigene41336\_Mf\_liverA, Unigene5632\_Mf\_liverA, CL4925.Contig1\_Mf\_liverA |
| coated membrane | Unigene41336\_Mf\_liverA, Unigene5632\_Mf\_liverA, CL4925.Contig1\_Mf\_liverA |
| occluding junction | Unigene25226\_Mf\_liverA, Unigene14765\_Mf\_liverA, Unigene13950\_Mf\_liverA |
| inclusion body | Unigene35816\_Mf\_liverA, Unigene9406\_Mf\_liverA |
| myosin complex | CL591.Contig1\_Mf\_liverA, Unigene28822\_Mf\_liverA |
| Z disc | CL2251.Contig1\_Mf\_liverA, Unigene13593\_Mf\_liverA |
| synaptic vesicle | Unigene34394\_Mf\_liverA, CL5807.Contig1\_Mf\_liverA, Unigene13363\_Mf\_liverA |
| actin filament bundle | Unigene14637\_Mf\_liverA, Unigene4556\_Mf\_liverA, NM\_134156 |
| myofibril | Unigene5639\_Mf\_liverA, CL1352.Contig1\_Mf\_liverA, Unigene4556\_Mf\_liverA, NM\_001025388, NM\_134156, CL2251.Contig1\_Mf\_liverA, Unigene13593\_Mf\_liverA |
| peroxisome | Unigene5287\_Mf\_liverA, CL1125.Contig1\_Mf\_liverA, NM\_008292, Unigene37076\_Mf\_liverA, Unigene5165\_Mf\_liverA |
| synapse | Unigene30707\_Mf\_liverA, Unigene18500\_Mf\_liverA, Unigene34394\_Mf\_liverA, CL5807.Contig1\_Mf\_liverA, NM\_010141, Unigene13363\_Mf\_liverA, Unigene24883\_Mf\_liverA, Unigene24804\_Mf\_liverA, Unigene1221\_Mf\_liverA, NM\_009255, Unigene13593\_Mf\_liverA, Unigene24252\_Mf\_liverA, Unigene13950\_Mf\_liverA |
| basolateral plasma membrane | Unigene26585\_Mf\_liverA, Unigene31852\_Mf\_liverA, CL1052.Contig1\_Mf\_liverA, Unigene431\_Mf\_liverA, Unigene32564\_Mf\_liverA, NM\_134156, Unigene28186\_Mf\_liverA, Unigene39252\_Mf\_liverA, Unigene38870\_Mf\_liverA, Unigene29424\_Mf\_liverA, Unigene35816\_Mf\_liverA, Unigene13950\_Mf\_liverA |
| sarcomere | Unigene5639\_Mf\_liverA, Unigene4556\_Mf\_liverA, NM\_134156, CL2251.Contig1\_Mf\_liverA, Unigene13593\_Mf\_liverA |
| condensed chromosome kinetochore | CL5268.Contig1\_Mf\_liverA, Unigene45530\_Mf\_liverA |
| axon part | Unigene34866\_Mf\_liverA, Unigene24883\_Mf\_liverA, Unigene8132\_Mf\_liverA, Unigene34867\_Mf\_liverA, Unigene4720\_Mf\_liverA |
| protein serine/threonine phosphatase complex | Unigene30707\_Mf\_liverA, CL532.Contig1\_Mf\_liverA |
| midbody | Unigene5693\_Mf\_liverA, Unigene23870\_Mf\_liverA |
| cytoskeleton | Unigene34609\_Mf\_liverA, NM\_009898, Unigene25721\_Mf\_liverA, Unigene15318\_Mf\_liverA, CL425.Contig1\_Mf\_liverA, Unigene29308\_Mf\_liverA, Unigene29424\_Mf\_liverA, Unigene29008\_Mf\_liverA, Unigene11007\_Mf\_liverA, CL4162.Contig1\_Mf\_liverA, CL3725.Contig1\_Mf\_liverA, NM\_011072, Unigene5693\_Mf\_liverA, Unigene5165\_Mf\_liverA, Unigene24252\_Mf\_liverA, Unigene30707\_Mf\_liverA, CL422.Contig1\_Mf\_liverA, Unigene40020\_Mf\_liverA, Unigene25070\_Mf\_liverA, CL1810.Contig1\_Mf\_liverA, NM\_009609, CL5191.Contig2\_Mf\_liverA, Unigene31623\_Mf\_liverA, Unigene13593\_Mf\_liverA, Unigene5138\_Mf\_liverA, CL3207.Contig1\_Mf\_liverA, Unigene5940\_Mf\_liverA, Unigene25046\_Mf\_liverA, Unigene28499\_Mf\_liverA, Unigene5941\_Mf\_liverA, Unigene2745\_Mf\_liverA, Unigene5639\_Mf\_liverA, NM\_153795, Unigene28822\_Mf\_liverA, NM\_177093, Unigene1327\_Mf\_liverA, Unigene23870\_Mf\_liverA, CL848.Contig2\_Mf\_liverA, CL2251.Contig1\_Mf\_liverA, CL1052.Contig1\_Mf\_liverA, Unigene14637\_Mf\_liverA, NM\_007622, Unigene32515\_Mf\_liverA, NM\_012030, Unigene24477\_Mf\_liverA, Unigene20432\_Mf\_liverA, CL4701.Contig1\_Mf\_liverA, Unigene25333\_Mf\_liverA, Unigene27547\_Mf\_liverA, Unigene550\_Mf\_liverA, Unigene27081\_Mf\_liverA, Unigene33366\_Mf\_liverA, NM\_134156, Unigene21317\_Mf\_liverA, NM\_010227, Unigene26309\_Mf\_liverA, Unigene37470\_Mf\_liverA, Unigene2195\_Mf\_liverA, NM\_146016, Unigene36673\_Mf\_liverA, Unigene496\_Mf\_liverA, Unigene27082\_Mf\_liverA, Unigene31852\_Mf\_liverA, Unigene37076\_Mf\_liverA, Unigene35169\_Mf\_liverA, CL5698.Contig1\_Mf\_liverA, CL5576.Contig1\_Mf\_liverA, CL1352.Contig1\_Mf\_liverA, CL591.Contig1\_Mf\_liverA, Unigene4556\_Mf\_liverA, Unigene2746\_Mf\_liverA |
| chromatin remodeling complex | NM\_177093, NM\_009609, Unigene31517\_Mf\_liverA, CL3900.Contig1\_Mf\_liverA, Unigene43107\_Mf\_liverA, CL4160.Contig2\_Mf\_liverA |
| transcriptional repressor complex | Unigene31517\_Mf\_liverA, CL2355.Contig1\_Mf\_liverA |
| condensed chromosome, centromeric region | CL5268.Contig1\_Mf\_liverA, Unigene45530\_Mf\_liverA |
| histone acetyltransferase complex | NM\_177093, NM\_009609, Unigene30288\_Mf\_liverA |
| protein-DNA complex | CL2439.Contig1\_Mf\_liverA, Unigene31392\_Mf\_liverA |
| contractile fiber part | Unigene5639\_Mf\_liverA, Unigene4556\_Mf\_liverA, NM\_134156, CL2251.Contig1\_Mf\_liverA, Unigene13593\_Mf\_liverA |
| microbody | Unigene5287\_Mf\_liverA, CL1125.Contig1\_Mf\_liverA, NM\_008292, Unigene37076\_Mf\_liverA, Unigene5165\_Mf\_liverA |
| PML body | CL2355.Contig1\_Mf\_liverA, CL4757.Contig1\_Mf\_liverA |
| histone methyltransferase complex | NM\_177093, NM\_009609 |
| cell-substrate adherens junction | Unigene31852\_Mf\_liverA, CL1052.Contig1\_Mf\_liverA, Unigene32564\_Mf\_liverA, Unigene29424\_Mf\_liverA, NM\_134156 |
| brush border | Unigene13363\_Mf\_liverA, NM\_012030 |
| insoluble fraction | NM\_001100182, NM\_001081372, NM\_009128, NM\_201360, NM\_008293, NM\_009255, NM\_010001, NM\_145474, NM\_178405, NM\_007822, NM\_153505, NM\_007811, NM\_153193, NM\_001081148, NM\_001253832, NM\_001104531, NM\_033444, NM\_011072, NM\_007820, NR\_024097, NM\_010003, NM\_001025388, NM\_013821, NM\_011170 |
| condensed nuclear chromosome | CL5268.Contig1\_Mf\_liverA, Unigene45530\_Mf\_liverA |
| cilium | Unigene1483\_Mf\_liverA, Unigene496\_Mf\_liverA, Unigene32294\_Mf\_liverA, Unigene32295\_Mf\_liverA, Unigene20602\_Mf\_liverA |
| ubiquitin ligase complex | Unigene29063\_Mf\_liverA, Unigene843\_Mf\_liverA, CL777.Contig8\_Mf\_liverA, Unigene13683\_Mf\_liverA |
| primary cilium | Unigene32294\_Mf\_liverA, Unigene32295\_Mf\_liverA, Unigene20602\_Mf\_liverA |
| postsynaptic density | Unigene13593\_Mf\_liverA, Unigene24252\_Mf\_liverA |
| dendritic spine head | Unigene13593\_Mf\_liverA, Unigene24252\_Mf\_liverA |
| cullin-RING ubiquitin ligase complex | Unigene29063\_Mf\_liverA, Unigene13683\_Mf\_liverA |
| macromolecular complex | Unigene9466\_Mf\_liverA, NM\_176843, Unigene35884\_Mf\_liverA, NM\_009898, CL3835.Contig2\_Mf\_liverA, Unigene31199\_Mf\_liverA, Unigene6959\_Mf\_liverA, NM\_025593, Unigene24111\_Mf\_liverA, Unigene28186\_Mf\_liverA, Unigene29308\_Mf\_liverA, Unigene9081\_Mf\_liverA, NM\_080638, NM\_010378, CL4160.Contig2\_Mf\_liverA, Unigene36849\_Mf\_liverA, Unigene29008\_Mf\_liverA, CL2339.Contig1\_Mf\_liverA, Unigene22433\_Mf\_liverA, Unigene29788\_Mf\_liverA, CL777.Contig8\_Mf\_liverA, Unigene30878\_Mf\_liverA, CL4757.Contig1\_Mf\_liverA, NR\_004446, Unigene18500\_Mf\_liverA, Unigene38331\_Mf\_liverA, Unigene36889\_Mf\_liverA, Unigene41336\_Mf\_liverA, Unigene24112\_Mf\_liverA, CL4141.Contig1\_Mf\_liverA, Unigene35431\_Mf\_liverA, Unigene35816\_Mf\_liverA, Unigene31333\_Mf\_liverA, CL2260.Contig1\_Mf\_liverA, Unigene4686\_Mf\_liverA, Unigene843\_Mf\_liverA, NM\_031165, Unigene5138\_Mf\_liverA, Unigene12153\_Mf\_liverA, Unigene37150\_Mf\_liverA, Unigene22432\_Mf\_liverA, Unigene25046\_Mf\_liverA, CL5268.Contig1\_Mf\_liverA, Unigene5294\_Mf\_liverA, Unigene5941\_Mf\_liverA, Unigene2745\_Mf\_liverA, Unigene5639\_Mf\_liverA, Unigene28822\_Mf\_liverA, CL2855.Contig2\_Mf\_liverA, NM\_177093, Unigene14907\_Mf\_liverA, Unigene23870\_Mf\_liverA, Unigene39655\_Mf\_liverA, Unigene25976\_Mf\_liverA, Unigene13143\_Mf\_liverA, Unigene5632\_Mf\_liverA, Unigene37460\_Mf\_liverA, Unigene36698\_Mf\_liverA, Unigene31517\_Mf\_liverA, NM\_153505, Unigene13945\_Mf\_liverA, Unigene15583\_Mf\_liverA, CL4701.Contig1\_Mf\_liverA, CL5631.Contig1\_Mf\_liverA, NM\_153589, Unigene37575\_Mf\_liverA, Unigene27081\_Mf\_liverA, Unigene27547\_Mf\_liverA, CL4040.Contig2\_Mf\_liverA, Unigene33366\_Mf\_liverA, NM\_010391, Unigene45530\_Mf\_liverA, Unigene29063\_Mf\_liverA, Unigene24157\_Mf\_liverA, Unigene152\_Mf\_liverA, CL532.Contig1\_Mf\_liverA, Unigene27082\_Mf\_liverA, Unigene37076\_Mf\_liverA, Unigene43107\_Mf\_liverA, Unigene30288\_Mf\_liverA, Unigene32436\_Mf\_liverA, NM\_010380, CL275.Contig5\_Mf\_liverA, CL5576.Contig1\_Mf\_liverA, CL5189.Contig1\_Mf\_liverA, CL2855.Contig1\_Mf\_liverA, Unigene2746\_Mf\_liverA, NM\_001025388, NM\_007678, NM\_010392, CL4086.Contig1\_Mf\_liverA, Unigene40610\_Mf\_liverA, CL4925.Contig1\_Mf\_liverA, CL425.Contig1\_Mf\_liverA, Unigene30815\_Mf\_liverA, Unigene15982\_Mf\_liverA, CL4162.Contig1\_Mf\_liverA, Unigene591\_Mf\_liverA, CL4040.Contig1\_Mf\_liverA, Unigene29231\_Mf\_liverA, Unigene5165\_Mf\_liverA, CL3800.Contig1\_Mf\_liverA, Unigene30707\_Mf\_liverA, Unigene36543\_Mf\_liverA, Unigene4938\_Mf\_liverA, CL3002.Contig1\_Mf\_liverA, CL1810.Contig1\_Mf\_liverA, NM\_009609, Unigene5758\_Mf\_liverA, Unigene38870\_Mf\_liverA, CL887.Contig2\_Mf\_liverA, CL2439.Contig1\_Mf\_liverA, Unigene35935\_Mf\_liverA, Unigene29426\_Mf\_liverA, Unigene5940\_Mf\_liverA, CL3900.Contig1\_Mf\_liverA, NM\_033444, Unigene13683\_Mf\_liverA, Unigene24471\_Mf\_liverA, Unigene13106\_Mf\_liverA, Unigene39252\_Mf\_liverA, NM\_019879, Unigene8740\_Mf\_liverA, CL2251.Contig1\_Mf\_liverA, Unigene14270\_Mf\_liverA, Unigene39749\_Mf\_liverA, CL3835.Contig1\_Mf\_liverA, Unigene13950\_Mf\_liverA, Unigene37245\_Mf\_liverA, Unigene31198\_Mf\_liverA, CL2355.Contig1\_Mf\_liverA, Unigene37153\_Mf\_liverA, CL3104.Contig1\_Mf\_liverA, Unigene19658\_Mf\_liverA, Unigene4636\_Mf\_liverA, Unigene30814\_Mf\_liverA, NM\_027406, Unigene18340\_Mf\_liverA, Unigene4983\_Mf\_liverA, CL5698.Contig1\_Mf\_liverA, NM\_023256, Unigene31392\_Mf\_liverA, Unigene4681\_Mf\_liverA, Unigene14284\_Mf\_liverA, CL591.Contig1\_Mf\_liverA, Unigene4556\_Mf\_liverA |
| methyltransferase complex | NM\_177093, NM\_009609 |
| synapse part | Unigene34394\_Mf\_liverA, CL5807.Contig1\_Mf\_liverA, Unigene24883\_Mf\_liverA, Unigene13363\_Mf\_liverA, Unigene13593\_Mf\_liverA, Unigene24252\_Mf\_liverA |
| synaptic membrane | Unigene24883\_Mf\_liverA, Unigene13593\_Mf\_liverA |
| intracellular organelle part | Unigene29399\_Mf\_liverA, NM\_176843, Unigene35884\_Mf\_liverA, NM\_020559, NM\_008292, Unigene38015\_Mf\_liverA, Unigene28142\_Mf\_liverA, NM\_009898, CL3835.Contig2\_Mf\_liverA, NM\_025593, Unigene28899\_Mf\_liverA, NM\_018815, Unigene29308\_Mf\_liverA, CL1575.Contig1\_Mf\_liverA, CL523.Contig1\_Mf\_liverA, NM\_080638, Unigene13153\_Mf\_liverA, Unigene34727\_Mf\_liverA, NM\_011082, Unigene29424\_Mf\_liverA, CL4160.Contig2\_Mf\_liverA, Unigene29008\_Mf\_liverA, CL4105.Contig1\_Mf\_liverA, CL482.Contig1\_Mf\_liverA, CL777.Contig8\_Mf\_liverA, Unigene39886\_Mf\_liverA, Unigene21466\_Mf\_liverA, Unigene15064\_Mf\_liverA, Unigene30878\_Mf\_liverA, Unigene24252\_Mf\_liverA, CL4757.Contig1\_Mf\_liverA, Unigene34810\_Mf\_liverA, Unigene37880\_Mf\_liverA, CL5586.Contig1\_Mf\_liverA, Unigene41336\_Mf\_liverA, Unigene34341\_Mf\_liverA, Unigene37535\_Mf\_liverA, Unigene25070\_Mf\_liverA, CL695.Contig1\_Mf\_liverA, CL4141.Contig1\_Mf\_liverA, Unigene35431\_Mf\_liverA, Unigene15703\_Mf\_liverA, CL2001.Contig1\_Mf\_liverA, Unigene35816\_Mf\_liverA, Unigene13593\_Mf\_liverA, Unigene4686\_Mf\_liverA, Unigene34394\_Mf\_liverA, Unigene25596\_Mf\_liverA, NM\_031165, NM\_153193, Unigene5138\_Mf\_liverA, CL1493.Contig2\_Mf\_liverA, Unigene14508\_Mf\_liverA, Unigene25046\_Mf\_liverA, CL5268.Contig1\_Mf\_liverA, Unigene5294\_Mf\_liverA, Unigene5941\_Mf\_liverA, Unigene2745\_Mf\_liverA, Unigene8054\_Mf\_liverA, Unigene25462\_Mf\_liverA, Unigene802\_Mf\_liverA, Unigene5775\_Mf\_liverA, CL787.Contig1\_Mf\_liverA, Unigene5639\_Mf\_liverA, Unigene17048\_Mf\_liverA, CL5807.Contig1\_Mf\_liverA, Unigene28822\_Mf\_liverA, Unigene36417\_Mf\_liverA, CL2855.Contig2\_Mf\_liverA, NM\_177093, Unigene14907\_Mf\_liverA, Unigene33512\_Mf\_liverA, Unigene5382\_Mf\_liverA, CL848.Contig2\_Mf\_liverA, Unigene23870\_Mf\_liverA, Unigene4922\_Mf\_liverA, Unigene5632\_Mf\_liverA, Unigene25976\_Mf\_liverA, Unigene36669\_Mf\_liverA, Unigene14637\_Mf\_liverA, CL1263.Contig1\_Mf\_liverA, Unigene37460\_Mf\_liverA, Unigene31517\_Mf\_liverA, NM\_007622, NM\_011305, Unigene139\_Mf\_liverA, CL3816.Contig1\_Mf\_liverA, CL4701.Contig1\_Mf\_liverA, Unigene36757\_Mf\_liverA, CL5631.Contig1\_Mf\_liverA, CL529.Contig2\_Mf\_liverA, Unigene37575\_Mf\_liverA, Unigene5774\_Mf\_liverA, CL4220.Contig2\_Mf\_liverA, Unigene25398\_Mf\_liverA, Unigene27081\_Mf\_liverA, Unigene27547\_Mf\_liverA, NM\_001081372, Unigene33366\_Mf\_liverA, NM\_134156, CL5316.Contig1\_Mf\_liverA, Unigene37389\_Mf\_liverA, Unigene21317\_Mf\_liverA, NM\_010227, Unigene45530\_Mf\_liverA, Unigene32332\_Mf\_liverA, Unigene30493\_Mf\_liverA, Unigene7195\_Mf\_liverA, Unigene8132\_Mf\_liverA, CL5254.Contig1\_Mf\_liverA, Unigene34010\_Mf\_liverA, Unigene152\_Mf\_liverA, Unigene24613\_Mf\_liverA, Unigene27082\_Mf\_liverA, CL532.Contig1\_Mf\_liverA, Unigene5712\_Mf\_liverA, Unigene37076\_Mf\_liverA, Unigene1280\_Mf\_liverA, Unigene43107\_Mf\_liverA, Unigene1205\_Mf\_liverA, Unigene30288\_Mf\_liverA, CL442.Contig2\_Mf\_liverA, CL3166.Contig4\_Mf\_liverA, Unigene14171\_Mf\_liverA, Unigene32436\_Mf\_liverA, CL5576.Contig1\_Mf\_liverA, Unigene33632\_Mf\_liverA, CL2855.Contig1\_Mf\_liverA, Unigene2746\_Mf\_liverA, NM\_013821, Unigene40796\_Mf\_liverA, Unigene13233\_Mf\_liverA, NM\_007678, NM\_010481, NM\_009128, CL4925.Contig1\_Mf\_liverA, Unigene15318\_Mf\_liverA, CL2240.Contig1\_Mf\_liverA, CL425.Contig1\_Mf\_liverA, NM\_008293, CL2797.Contig2\_Mf\_liverA, Unigene7970\_Mf\_liverA, CL1803.Contig1\_Mf\_liverA, Unigene14916\_Mf\_liverA, CL1125.Contig1\_Mf\_liverA, CL4162.Contig1\_Mf\_liverA, Unigene35237\_Mf\_liverA, Unigene112\_Mf\_liverA, Unigene15077\_Mf\_liverA, CL4600.Contig1\_Mf\_liverA, Unigene5693\_Mf\_liverA, Unigene22052\_Mf\_liverA, Unigene5165\_Mf\_liverA, CL5796.Contig2\_Mf\_liverA, Unigene30707\_Mf\_liverA, Unigene29876\_Mf\_liverA, CL2478.Contig3\_Mf\_liverA, Unigene36414\_Mf\_liverA, CL1988.Contig1\_Mf\_liverA, NM\_133838, Unigene36328\_Mf\_liverA, CL3002.Contig1\_Mf\_liverA, CL1988.Contig3\_Mf\_liverA, CL1810.Contig1\_Mf\_liverA, NM\_009609, Unigene36514\_Mf\_liverA, CL4033.Contig1\_Mf\_liverA, CL4816.Contig2\_Mf\_liverA, Unigene28662\_Mf\_liverA, Unigene4781\_Mf\_liverA, Unigene35935\_Mf\_liverA, CL1988.Contig2\_Mf\_liverA, Unigene32695\_Mf\_liverA, CL3207.Contig1\_Mf\_liverA, Unigene5906\_Mf\_liverA, Unigene5940\_Mf\_liverA, CL3900.Contig1\_Mf\_liverA, Unigene43357\_Mf\_liverA, Unigene28731\_Mf\_liverA, Unigene13683\_Mf\_liverA, CL1493.Contig1\_Mf\_liverA, NM\_033444, CL2791.Contig1\_Mf\_liverA, Unigene9698\_Mf\_liverA, Unigene28499\_Mf\_liverA, Unigene36420\_Mf\_liverA, Unigene10135\_Mf\_liverA, Unigene542\_Mf\_liverA, Unigene35476\_Mf\_liverA, Unigene24758\_Mf\_liverA, Unigene32294\_Mf\_liverA, Unigene4909\_Mf\_liverA, CL114.Contig2\_Mf\_liverA, Unigene24471\_Mf\_liverA, NM\_009883, NM\_010162, Unigene15529\_Mf\_liverA, CL4220.Contig1\_Mf\_liverA, CL5307.Contig1\_Mf\_liverA, Unigene3377\_Mf\_liverA, NM\_019879, CL2251.Contig1\_Mf\_liverA, CL738.Contig2\_Mf\_liverA, CL3816.Contig2\_Mf\_liverA, Unigene14270\_Mf\_liverA, CL4490.Contig2\_Mf\_liverA, Unigene25595\_Mf\_liverA, CL4816.Contig3\_Mf\_liverA, NM\_012030, Unigene30731\_Mf\_liverA, NM\_025336, Unigene35609\_Mf\_liverA, CL4007.Contig1\_Mf\_liverA, Unigene36418\_Mf\_liverA, Unigene1221\_Mf\_liverA, Unigene4841\_Mf\_liverA, CL3835.Contig1\_Mf\_liverA, Unigene7048\_Mf\_liverA, Unigene23185\_Mf\_liverA, Unigene35046\_Mf\_liverA, NM\_010158, Unigene1292\_Mf\_liverA, CL2355.Contig1\_Mf\_liverA, Unigene37153\_Mf\_liverA, CL3104.Contig1\_Mf\_liverA, Unigene14809\_Mf\_liverA, Unigene32295\_Mf\_liverA, Unigene36593\_Mf\_liverA, Unigene15592\_Mf\_liverA, Unigene5745\_Mf\_liverA, Unigene12889\_Mf\_liverA, Unigene36699\_Mf\_liverA, Unigene36673\_Mf\_liverA, Unigene496\_Mf\_liverA, NM\_001099634, CL5698.Contig1\_Mf\_liverA, Unigene6110\_Mf\_liverA, Unigene31392\_Mf\_liverA, Unigene4681\_Mf\_liverA, Unigene16891\_Mf\_liverA, CL591.Contig1\_Mf\_liverA, Unigene4556\_Mf\_liverA, Unigene5886\_Mf\_liverA, CL336.Contig3\_Mf\_liverA |
| nonmotile primary cilium | Unigene32294\_Mf\_liverA, Unigene32295\_Mf\_liverA |
| membrane-bounded organelle | Unigene21684\_Mf\_liverA, Unigene5287\_Mf\_liverA, NM\_176843, NM\_009898, Unigene25721\_Mf\_liverA, CL3669.Contig2\_Mf\_liverA, NM\_025593, NM\_018815, Unigene28186\_Mf\_liverA, NM\_080638, Unigene15553\_Mf\_liverA, NM\_010378, Unigene34727\_Mf\_liverA, NM\_011082, Unigene29424\_Mf\_liverA, CL4160.Contig2\_Mf\_liverA, CL854.Contig1\_Mf\_liverA, CL4105.Contig1\_Mf\_liverA, Unigene11007\_Mf\_liverA, CL4577.Contig1\_Mf\_liverA, NM\_025613, Unigene39886\_Mf\_liverA, Unigene15064\_Mf\_liverA, NM\_021273, CL4757.Contig1\_Mf\_liverA, NR\_004446, CL5586.Contig1\_Mf\_liverA, Unigene34341\_Mf\_liverA, Unigene37535\_Mf\_liverA, Unigene30142\_Mf\_liverA, CL695.Contig1\_Mf\_liverA, Unigene35431\_Mf\_liverA, Unigene15703\_Mf\_liverA, Unigene35816\_Mf\_liverA, Unigene31623\_Mf\_liverA, Unigene7897\_Mf\_liverA, NM\_145474, Unigene13379\_Mf\_liverA, NM\_009776, Unigene34394\_Mf\_liverA, NM\_031165, Unigene25594\_Mf\_liverA, NM\_153193, Unigene5138\_Mf\_liverA, Unigene14508\_Mf\_liverA, Unigene14050\_Mf\_liverA, CL4995.Contig1\_Mf\_liverA, Unigene8054\_Mf\_liverA, Unigene18430\_Mf\_liverA, Unigene802\_Mf\_liverA, Unigene17048\_Mf\_liverA, CL5807.Contig1\_Mf\_liverA, Unigene13363\_Mf\_liverA, CL2384.Contig1\_Mf\_liverA, Unigene36417\_Mf\_liverA, NM\_021278, CL2855.Contig2\_Mf\_liverA, NM\_177093, Unigene25226\_Mf\_liverA, Unigene33512\_Mf\_liverA, Unigene14907\_Mf\_liverA, Unigene21561\_Mf\_liverA, Unigene39655\_Mf\_liverA, NM\_010001, Unigene4922\_Mf\_liverA, Unigene25976\_Mf\_liverA, Unigene36698\_Mf\_liverA, Unigene30261\_Mf\_liverA, NM\_007622, Unigene13945\_Mf\_liverA, NM\_001104531, Unigene139\_Mf\_liverA, Unigene4944\_Mf\_liverA, Unigene23158\_Mf\_liverA, Unigene37575\_Mf\_liverA, Unigene5774\_Mf\_liverA, Unigene25398\_Mf\_liverA, Unigene27547\_Mf\_liverA, CL1736.Contig2\_Mf\_liverA, Unigene36836\_Mf\_liverA, Unigene33366\_Mf\_liverA, NM\_134156, CL5316.Contig1\_Mf\_liverA, Unigene37389\_Mf\_liverA, Unigene4720\_Mf\_liverA, Unigene45530\_Mf\_liverA, Unigene29063\_Mf\_liverA, Unigene32332\_Mf\_liverA, Unigene4723\_Mf\_liverA, CL5254.Contig1\_Mf\_liverA, Unigene34010\_Mf\_liverA, Unigene24613\_Mf\_liverA, Unigene152\_Mf\_liverA, CL532.Contig1\_Mf\_liverA, Unigene37076\_Mf\_liverA, Unigene1280\_Mf\_liverA, Unigene30288\_Mf\_liverA, CL3166.Contig4\_Mf\_liverA, Unigene14171\_Mf\_liverA, Unigene32436\_Mf\_liverA, Unigene33632\_Mf\_liverA, CL5189.Contig1\_Mf\_liverA, CL3339.Contig1\_Mf\_liverA, Unigene2746\_Mf\_liverA, Unigene30528\_Mf\_liverA, NM\_007678, Unigene31251\_Mf\_liverA, Unigene4363\_Mf\_liverA, Unigene40610\_Mf\_liverA, CL4925.Contig1\_Mf\_liverA, CL186.Contig3\_Mf\_liverA, CL425.Contig1\_Mf\_liverA, Unigene32421\_Mf\_liverA, CL2797.Contig2\_Mf\_liverA, CL1803.Contig1\_Mf\_liverA, Unigene14916\_Mf\_liverA, Unigene37243\_Mf\_liverA, Unigene35858\_Mf\_liverA, Unigene30587\_Mf\_liverA, Unigene15077\_Mf\_liverA, CL4600.Contig1\_Mf\_liverA, Unigene36034\_Mf\_liverA, Unigene29231\_Mf\_liverA, Unigene5693\_Mf\_liverA, Unigene22052\_Mf\_liverA, NM\_010003, Unigene34867\_Mf\_liverA, Unigene29876\_Mf\_liverA, CL3800.Contig1\_Mf\_liverA, CL2478.Contig3\_Mf\_liverA, Unigene28687\_Mf\_liverA, CL1988.Contig3\_Mf\_liverA, NM\_024474, NM\_009609, CL1810.Contig1\_Mf\_liverA, Unigene18499\_Mf\_liverA, CL4033.Contig1\_Mf\_liverA, Unigene24547\_Mf\_liverA, Unigene34983\_Mf\_liverA, Unigene28662\_Mf\_liverA, CL2439.Contig1\_Mf\_liverA, Unigene4781\_Mf\_liverA, NR\_003623, Unigene8033\_Mf\_liverA, Unigene35935\_Mf\_liverA, CL1988.Contig2\_Mf\_liverA, CL3900.Contig1\_Mf\_liverA, Unigene13683\_Mf\_liverA, CL1493.Contig1\_Mf\_liverA, Unigene9698\_Mf\_liverA, Unigene28499\_Mf\_liverA, Unigene36420\_Mf\_liverA, Unigene30585\_Mf\_liverA, Unigene542\_Mf\_liverA, Unigene35476\_Mf\_liverA, Unigene24758\_Mf\_liverA, Unigene4909\_Mf\_liverA, Unigene24471\_Mf\_liverA, Unigene33080\_Mf\_liverA, NM\_010162, Unigene15529\_Mf\_liverA, CL4220.Contig1\_Mf\_liverA, NM\_019879, NR\_033215, CL2251.Contig1\_Mf\_liverA, NM\_007822, CL4490.Contig2\_Mf\_liverA, Unigene25595\_Mf\_liverA, Unigene14286\_Mf\_liverA, Unigene32515\_Mf\_liverA, CL4816.Contig3\_Mf\_liverA, CL840.Contig1\_Mf\_liverA, Unigene39011\_Mf\_liverA, NM\_009022, NM\_025336, Unigene35609\_Mf\_liverA, NM\_145218, Unigene4841\_Mf\_liverA, Unigene23185\_Mf\_liverA, Unigene7048\_Mf\_liverA, Unigene13950\_Mf\_liverA, Unigene35046\_Mf\_liverA, Unigene28459\_Mf\_liverA, Unigene37245\_Mf\_liverA, NM\_010158, Unigene13847\_Mf\_liverA, Unigene29985\_Mf\_liverA, CL3104.Contig1\_Mf\_liverA, Unigene36593\_Mf\_liverA, Unigene19658\_Mf\_liverA, Unigene37616\_Mf\_liverA, NM\_027406, Unigene36699\_Mf\_liverA, Unigene36673\_Mf\_liverA, Unigene496\_Mf\_liverA, NM\_001164598, Unigene4983\_Mf\_liverA, CL5698.Contig1\_Mf\_liverA, Unigene6110\_Mf\_liverA, NM\_001253832, Unigene31392\_Mf\_liverA, NM\_023256, Unigene9466\_Mf\_liverA, Unigene29399\_Mf\_liverA, NM\_020559, Unigene35884\_Mf\_liverA, Unigene33607\_Mf\_liverA, NM\_008292, Unigene28142\_Mf\_liverA, Unigene38015\_Mf\_liverA, CL3835.Contig2\_Mf\_liverA, Unigene31199\_Mf\_liverA, Unigene28899\_Mf\_liverA, Unigene46870\_Mf\_liverA, CL1575.Contig1\_Mf\_liverA, CL523.Contig1\_Mf\_liverA, Unigene13153\_Mf\_liverA, CL4117.Contig1\_Mf\_liverA, Unigene29008\_Mf\_liverA, CL482.Contig1\_Mf\_liverA, Unigene36762\_Mf\_liverA, CL777.Contig8\_Mf\_liverA, CL3725.Contig1\_Mf\_liverA, NM\_011072, NM\_007820, Unigene4776\_Mf\_liverA, Unigene21466\_Mf\_liverA, Unigene30878\_Mf\_liverA, Unigene40289\_Mf\_liverA, Unigene34810\_Mf\_liverA, CL44.Contig1\_Mf\_liverA, Unigene37880\_Mf\_liverA, Unigene38331\_Mf\_liverA, Unigene37999\_Mf\_liverA, Unigene40020\_Mf\_liverA, Unigene37904\_Mf\_liverA, Unigene41336\_Mf\_liverA, Unigene25070\_Mf\_liverA, CL4141.Contig1\_Mf\_liverA, CL2001.Contig1\_Mf\_liverA, Unigene26194\_Mf\_liverA, Unigene4686\_Mf\_liverA, Unigene11\_Mf\_liverA, Unigene25596\_Mf\_liverA, Unigene45131\_Mf\_liverA, Unigene5994\_Mf\_liverA, CL1493.Contig2\_Mf\_liverA, Unigene12153\_Mf\_liverA, CL5268.Contig1\_Mf\_liverA, Unigene5294\_Mf\_liverA, Unigene2745\_Mf\_liverA, Unigene38104\_Mf\_liverA, Unigene38065\_Mf\_liverA, Unigene25462\_Mf\_liverA, Unigene5775\_Mf\_liverA, Unigene36626\_Mf\_liverA, CL787.Contig1\_Mf\_liverA, Unigene39507\_Mf\_liverA, Unigene25524\_Mf\_liverA, Unigene31988\_Mf\_liverA, CL3750.Contig2\_Mf\_liverA, Unigene39141\_Mf\_liverA, Unigene5382\_Mf\_liverA, NM\_201360, Unigene33459\_Mf\_liverA, Unigene23870\_Mf\_liverA, Unigene5632\_Mf\_liverA, Unigene36669\_Mf\_liverA, CL1263.Contig1\_Mf\_liverA, Unigene37460\_Mf\_liverA, Unigene31517\_Mf\_liverA, NM\_007811, Unigene14582\_Mf\_liverA, CL2142.Contig2\_Mf\_liverA, NM\_011305, Unigene24503\_Mf\_liverA, Unigene27422\_Mf\_liverA, CL3816.Contig1\_Mf\_liverA, Unigene25333\_Mf\_liverA, CL993.Contig2\_Mf\_liverA, Unigene36757\_Mf\_liverA, CL5631.Contig1\_Mf\_liverA, CL529.Contig2\_Mf\_liverA, Unigene30584\_Mf\_liverA, CL4220.Contig2\_Mf\_liverA, Unigene17579\_Mf\_liverA, Unigene27081\_Mf\_liverA, NM\_001081372, NM\_010391, Unigene37148\_Mf\_liverA, NM\_010227, Unigene30493\_Mf\_liverA, Unigene7195\_Mf\_liverA, Unigene8132\_Mf\_liverA, NM\_019717, Unigene37178\_Mf\_liverA, Unigene27082\_Mf\_liverA, Unigene5712\_Mf\_liverA, Unigene43107\_Mf\_liverA, Unigene1205\_Mf\_liverA, CL442.Contig2\_Mf\_liverA, CL6039.Contig1\_Mf\_liverA, CL2855.Contig1\_Mf\_liverA, CL4411.Contig4\_Mf\_liverA, NM\_001025388, Unigene31206\_Mf\_liverA, NM\_013821, Unigene40796\_Mf\_liverA, Unigene13233\_Mf\_liverA, NM\_011099, NM\_001100182, NM\_010481, NM\_009128, Unigene15318\_Mf\_liverA, CL2240.Contig1\_Mf\_liverA, Unigene36190\_Mf\_liverA, NM\_008293, Unigene7970\_Mf\_liverA, NM\_009255, Unigene15982\_Mf\_liverA, CL1125.Contig1\_Mf\_liverA, CL4162.Contig1\_Mf\_liverA, Unigene35237\_Mf\_liverA, Unigene112\_Mf\_liverA, Unigene12907\_Mf\_liverA, Unigene25292\_Mf\_liverA, Unigene5165\_Mf\_liverA, Unigene15588\_Mf\_liverA, CL5796.Contig2\_Mf\_liverA, NM\_011170, Unigene30707\_Mf\_liverA, Unigene36414\_Mf\_liverA, CL1988.Contig1\_Mf\_liverA, NM\_133838, Unigene5175\_Mf\_liverA, Unigene36328\_Mf\_liverA, Unigene15552\_Mf\_liverA, CL3002.Contig1\_Mf\_liverA, Unigene36514\_Mf\_liverA, CL6039.Contig2\_Mf\_liverA, NM\_145836, CL4816.Contig2\_Mf\_liverA, CL887.Contig2\_Mf\_liverA, Unigene32695\_Mf\_liverA, Unigene5906\_Mf\_liverA, NM\_001081148, Unigene43357\_Mf\_liverA, Unigene28731\_Mf\_liverA, Unigene14810\_Mf\_liverA, NM\_033444, Unigene1212\_Mf\_liverA, CL2791.Contig1\_Mf\_liverA, CL4770.Contig1\_Mf\_liverA, Unigene10135\_Mf\_liverA, Unigene32294\_Mf\_liverA, CL3750.Contig1\_Mf\_liverA, CL114.Contig2\_Mf\_liverA, NM\_009883, Unigene18796\_Mf\_liverA, CL5307.Contig1\_Mf\_liverA, Unigene3377\_Mf\_liverA, CL5978.Contig2\_Mf\_liverA, Unigene8740\_Mf\_liverA, NM\_178405, CL738.Contig2\_Mf\_liverA, CL3816.Contig2\_Mf\_liverA, Unigene14270\_Mf\_liverA, CL4048.Contig1\_Mf\_liverA, CL1052.Contig1\_Mf\_liverA, CL442.Contig5\_Mf\_liverA, NM\_012030, Unigene9406\_Mf\_liverA, Unigene24477\_Mf\_liverA, Unigene120\_Mf\_liverA, Unigene30731\_Mf\_liverA, CL4007.Contig1\_Mf\_liverA, Unigene15026\_Mf\_liverA, Unigene36418\_Mf\_liverA, Unigene14940\_Mf\_liverA, Unigene1221\_Mf\_liverA, CL3835.Contig1\_Mf\_liverA, Unigene39875\_Mf\_liverA, Unigene31198\_Mf\_liverA, Unigene1292\_Mf\_liverA, CL2355.Contig1\_Mf\_liverA, Unigene37153\_Mf\_liverA, Unigene14809\_Mf\_liverA, Unigene26309\_Mf\_liverA, Unigene32295\_Mf\_liverA, Unigene21562\_Mf\_liverA, Unigene15592\_Mf\_liverA, Unigene5745\_Mf\_liverA, Unigene665\_Mf\_liverA, Unigene2195\_Mf\_liverA, Unigene12889\_Mf\_liverA, NM\_001099634, Unigene8560\_Mf\_liverA, Unigene34866\_Mf\_liverA, Unigene4681\_Mf\_liverA, Unigene16891\_Mf\_liverA, Unigene37819\_Mf\_liverA, Unigene5886\_Mf\_liverA, CL336.Contig3\_Mf\_liverA |
| apical junction complex | Unigene25226\_Mf\_liverA, Unigene5693\_Mf\_liverA, Unigene14765\_Mf\_liverA, Unigene13950\_Mf\_liverA |
| apicolateral plasma membrane | Unigene25226\_Mf\_liverA, Unigene5693\_Mf\_liverA, Unigene14765\_Mf\_liverA, Unigene13950\_Mf\_liverA |
| intracellular part | Unigene21684\_Mf\_liverA, NM\_176843, NM\_009898, NM\_025593, Unigene28186\_Mf\_liverA, Unigene15553\_Mf\_liverA, NM\_010378, CL854.Contig1\_Mf\_liverA, Unigene16463\_Mf\_liverA, NM\_025613, Unigene39886\_Mf\_liverA, Unigene15064\_Mf\_liverA, Unigene24252\_Mf\_liverA, CL4757.Contig1\_Mf\_liverA, NR\_004446, Unigene37535\_Mf\_liverA, CL695.Contig1\_Mf\_liverA, Unigene35431\_Mf\_liverA, Unigene15703\_Mf\_liverA, Unigene31623\_Mf\_liverA, NM\_010233, Unigene13379\_Mf\_liverA, Unigene34394\_Mf\_liverA, NM\_172409, Unigene25594\_Mf\_liverA, Unigene14050\_Mf\_liverA, CL4995.Contig1\_Mf\_liverA, Unigene8054\_Mf\_liverA, Unigene18430\_Mf\_liverA, CL5807.Contig1\_Mf\_liverA, CL2384.Contig1\_Mf\_liverA, Unigene36417\_Mf\_liverA, NM\_177093, CL4293.Contig1\_Mf\_liverA, Unigene25226\_Mf\_liverA, Unigene33512\_Mf\_liverA, Unigene14907\_Mf\_liverA, Unigene25976\_Mf\_liverA, Unigene36698\_Mf\_liverA, NM\_145942, Unigene30261\_Mf\_liverA, Unigene13945\_Mf\_liverA, Unigene139\_Mf\_liverA, Unigene28217\_Mf\_liverA, Unigene36836\_Mf\_liverA, Unigene33366\_Mf\_liverA, NM\_134156, Unigene37389\_Mf\_liverA, Unigene4720\_Mf\_liverA, Unigene27248\_Mf\_liverA, CL2266.Contig2\_Mf\_liverA, Unigene30839\_Mf\_liverA, Unigene34010\_Mf\_liverA, Unigene24613\_Mf\_liverA, Unigene152\_Mf\_liverA, Unigene31852\_Mf\_liverA, CL532.Contig1\_Mf\_liverA, Unigene37076\_Mf\_liverA, Unigene1280\_Mf\_liverA, Unigene30288\_Mf\_liverA, Unigene14171\_Mf\_liverA, CL5576.Contig1\_Mf\_liverA, Unigene33632\_Mf\_liverA, CL5189.Contig1\_Mf\_liverA, CL3339.Contig1\_Mf\_liverA, Unigene2746\_Mf\_liverA, Unigene4363\_Mf\_liverA, CL425.Contig1\_Mf\_liverA, CL2797.Contig2\_Mf\_liverA, CL1803.Contig1\_Mf\_liverA, Unigene37243\_Mf\_liverA, Unigene30587\_Mf\_liverA, Unigene15077\_Mf\_liverA, Unigene36034\_Mf\_liverA, Unigene5693\_Mf\_liverA, Unigene39970\_Mf\_liverA, CL3393.Contig1\_Mf\_liverA, Unigene29876\_Mf\_liverA, CL3800.Contig1\_Mf\_liverA, Unigene28687\_Mf\_liverA, CL422.Contig1\_Mf\_liverA, CL1988.Contig3\_Mf\_liverA, NM\_028785, NM\_009609, CL1810.Contig1\_Mf\_liverA, CL4033.Contig1\_Mf\_liverA, Unigene24547\_Mf\_liverA, Unigene34983\_Mf\_liverA, NR\_003623, CL3519.Contig1\_Mf\_liverA, Unigene8033\_Mf\_liverA, CL1493.Contig1\_Mf\_liverA, Unigene35476\_Mf\_liverA, Unigene4909\_Mf\_liverA, Unigene24471\_Mf\_liverA, Unigene31571\_Mf\_liverA, NM\_010162, Unigene15529\_Mf\_liverA, CL4220.Contig1\_Mf\_liverA, Unigene37262\_Mf\_liverA, NR\_033215, CL2251.Contig1\_Mf\_liverA, NM\_007822, CL5293.Contig1\_Mf\_liverA, CL4490.Contig2\_Mf\_liverA, Unigene25595\_Mf\_liverA, Unigene14286\_Mf\_liverA, Unigene32515\_Mf\_liverA, CL4816.Contig3\_Mf\_liverA, Unigene39011\_Mf\_liverA, Unigene20432\_Mf\_liverA, NM\_009022, Unigene35609\_Mf\_liverA, Unigene7048\_Mf\_liverA, Unigene13950\_Mf\_liverA, Unigene35046\_Mf\_liverA, Unigene28459\_Mf\_liverA, Unigene37245\_Mf\_liverA, Unigene13847\_Mf\_liverA, Unigene19658\_Mf\_liverA, NM\_027406, Unigene36699\_Mf\_liverA, Unigene36176\_Mf\_liverA, Unigene18340\_Mf\_liverA, Unigene496\_Mf\_liverA, NM\_001164598, Unigene4983\_Mf\_liverA, Unigene29889\_Mf\_liverA, Unigene2939\_Mf\_liverA, Unigene9466\_Mf\_liverA, CL5640.Contig1\_Mf\_liverA, NM\_020559, Unigene35884\_Mf\_liverA, Unigene28142\_Mf\_liverA, CL3835.Contig2\_Mf\_liverA, Unigene28899\_Mf\_liverA, Unigene46870\_Mf\_liverA, CL1575.Contig1\_Mf\_liverA, NM\_019651, Unigene13153\_Mf\_liverA, CL4117.Contig1\_Mf\_liverA, Unigene8473\_Mf\_liverA, Unigene29008\_Mf\_liverA, Unigene33508\_Mf\_liverA, CL777.Contig8\_Mf\_liverA, NM\_011072, Unigene4776\_Mf\_liverA, Unigene21466\_Mf\_liverA, Unigene34810\_Mf\_liverA, Unigene27420\_Mf\_liverA, Unigene20602\_Mf\_liverA, Unigene37999\_Mf\_liverA, Unigene40020\_Mf\_liverA, Unigene37904\_Mf\_liverA, Unigene41336\_Mf\_liverA, CL3483.Contig1\_Mf\_liverA, CL4141.Contig1\_Mf\_liverA, Unigene34184\_Mf\_liverA, CL5191.Contig2\_Mf\_liverA, Unigene13593\_Mf\_liverA, Unigene4686\_Mf\_liverA, Unigene11\_Mf\_liverA, Unigene45131\_Mf\_liverA, Unigene5994\_Mf\_liverA, CL1493.Contig2\_Mf\_liverA, Unigene5294\_Mf\_liverA, Unigene5941\_Mf\_liverA, Unigene38104\_Mf\_liverA, Unigene38065\_Mf\_liverA, Unigene25462\_Mf\_liverA, Unigene36626\_Mf\_liverA, NM\_153795, Unigene28822\_Mf\_liverA, Unigene31988\_Mf\_liverA, Unigene5382\_Mf\_liverA, Unigene151\_Mf\_liverA, NM\_201360, CL848.Contig2\_Mf\_liverA, Unigene23870\_Mf\_liverA, CL1263.Contig1\_Mf\_liverA, Unigene37460\_Mf\_liverA, Unigene31517\_Mf\_liverA, NM\_007811, Unigene24323\_Mf\_liverA, CL2142.Contig2\_Mf\_liverA, NM\_011305, CL3816.Contig1\_Mf\_liverA, Unigene25333\_Mf\_liverA, CL993.Contig2\_Mf\_liverA, CL529.Contig2\_Mf\_liverA, Unigene27081\_Mf\_liverA, Unigene37148\_Mf\_liverA, Unigene7195\_Mf\_liverA, Unigene1205\_Mf\_liverA, CL1352.Contig1\_Mf\_liverA, CL6039.Contig1\_Mf\_liverA, Unigene24804\_Mf\_liverA, Unigene31206\_Mf\_liverA, NM\_009128, CL2240.Contig1\_Mf\_liverA, CL1125.Contig1\_Mf\_liverA, Unigene15681\_Mf\_liverA, Unigene5165\_Mf\_liverA, Unigene30707\_Mf\_liverA, NM\_133838, Unigene15552\_Mf\_liverA, CL3002.Contig1\_Mf\_liverA, Unigene30155\_Mf\_liverA, CL5057.Contig1\_Mf\_liverA, Unigene36514\_Mf\_liverA, NM\_145836, CL4816.Contig2\_Mf\_liverA, Unigene33523\_Mf\_liverA, CL3207.Contig1\_Mf\_liverA, Unigene5906\_Mf\_liverA, Unigene14810\_Mf\_liverA, NM\_033444, CL2791.Contig1\_Mf\_liverA, CL4770.Contig1\_Mf\_liverA, Unigene10135\_Mf\_liverA, Unigene1483\_Mf\_liverA, CL114.Contig2\_Mf\_liverA, NM\_009883, Unigene33526\_Mf\_liverA, Unigene1327\_Mf\_liverA, Unigene39252\_Mf\_liverA, NM\_001081172, CL5978.Contig2\_Mf\_liverA, Unigene8740\_Mf\_liverA, CL738.Contig2\_Mf\_liverA, CL3816.Contig2\_Mf\_liverA, Unigene14270\_Mf\_liverA, CL442.Contig5\_Mf\_liverA, CL5459.Contig2\_Mf\_liverA, NM\_012030, Unigene24477\_Mf\_liverA, Unigene30731\_Mf\_liverA, Unigene30983\_Mf\_liverA, Unigene36418\_Mf\_liverA, CL3835.Contig1\_Mf\_liverA, Unigene38311\_Mf\_liverA, Unigene550\_Mf\_liverA, Unigene31198\_Mf\_liverA, CL2355.Contig1\_Mf\_liverA, Unigene28314\_Mf\_liverA, Unigene37153\_Mf\_liverA, Unigene14809\_Mf\_liverA, Unigene21562\_Mf\_liverA, Unigene15592\_Mf\_liverA, Unigene2195\_Mf\_liverA, Unigene30892\_Mf\_liverA, Unigene34866\_Mf\_liverA, Unigene4681\_Mf\_liverA, Unigene5287\_Mf\_liverA, Unigene34609\_Mf\_liverA, NM\_144940, Unigene25721\_Mf\_liverA, CL3669.Contig2\_Mf\_liverA, Unigene37334\_Mf\_liverA, NM\_018815, NM\_080638, Unigene29424\_Mf\_liverA, NM\_011082, Unigene34727\_Mf\_liverA, CL4160.Contig2\_Mf\_liverA, CL4105.Contig1\_Mf\_liverA, Unigene26053\_Mf\_liverA, Unigene11007\_Mf\_liverA, CL4577.Contig1\_Mf\_liverA, Unigene785\_Mf\_liverA, Unigene27339\_Mf\_liverA, NM\_021273, CL5586.Contig1\_Mf\_liverA, Unigene34341\_Mf\_liverA, Unigene30142\_Mf\_liverA, NM\_011921, Unigene35816\_Mf\_liverA, Unigene7897\_Mf\_liverA, NM\_145474, NM\_009776, Unigene30003\_Mf\_liverA, Unigene5138\_Mf\_liverA, NM\_153193, NM\_031165, Unigene14508\_Mf\_liverA, Unigene30002\_Mf\_liverA, Unigene802\_Mf\_liverA, Unigene9150\_Mf\_liverA, Unigene17048\_Mf\_liverA, Unigene5639\_Mf\_liverA, Unigene33522\_Mf\_liverA, Unigene13363\_Mf\_liverA, CL2855.Contig2\_Mf\_liverA, NM\_021278, Unigene21561\_Mf\_liverA, NM\_010001, Unigene39655\_Mf\_liverA, Unigene4922\_Mf\_liverA, Unigene14637\_Mf\_liverA, NM\_153505, NM\_007622, NM\_001104531, Unigene4944\_Mf\_liverA, Unigene23158\_Mf\_liverA, Unigene37575\_Mf\_liverA, Unigene25398\_Mf\_liverA, Unigene5774\_Mf\_liverA, Unigene27547\_Mf\_liverA, CL1736.Contig2\_Mf\_liverA, CL5316.Contig1\_Mf\_liverA, Unigene45530\_Mf\_liverA, Unigene4723\_Mf\_liverA, Unigene32332\_Mf\_liverA, Unigene29063\_Mf\_liverA, Unigene37470\_Mf\_liverA, CL5254.Contig1\_Mf\_liverA, Unigene35169\_Mf\_liverA, CL3166.Contig4\_Mf\_liverA, Unigene35698\_Mf\_liverA, Unigene32436\_Mf\_liverA, Unigene16465\_Mf\_liverA, Unigene30528\_Mf\_liverA, NM\_007678, Unigene31251\_Mf\_liverA, Unigene40610\_Mf\_liverA, CL186.Contig3\_Mf\_liverA, CL4925.Contig1\_Mf\_liverA, Unigene32421\_Mf\_liverA, CL4736.Contig1\_Mf\_liverA, Unigene14916\_Mf\_liverA, Unigene35858\_Mf\_liverA, CL4600.Contig1\_Mf\_liverA, Unigene29231\_Mf\_liverA, NM\_010003, Unigene22052\_Mf\_liverA, Unigene34867\_Mf\_liverA, CL2478.Contig3\_Mf\_liverA, Unigene5815\_Mf\_liverA, NM\_024474, Unigene18499\_Mf\_liverA, Unigene38870\_Mf\_liverA, Unigene28662\_Mf\_liverA, Unigene4781\_Mf\_liverA, CL2439.Contig1\_Mf\_liverA, CL1988.Contig2\_Mf\_liverA, Unigene35935\_Mf\_liverA, Unigene29426\_Mf\_liverA, Unigene20371\_Mf\_liverA, CL3900.Contig1\_Mf\_liverA, Unigene13683\_Mf\_liverA, Unigene9698\_Mf\_liverA, Unigene30585\_Mf\_liverA, Unigene36420\_Mf\_liverA, Unigene28499\_Mf\_liverA, Unigene542\_Mf\_liverA, Unigene24758\_Mf\_liverA, Unigene33080\_Mf\_liverA, NM\_019879, CL5293.Contig2\_Mf\_liverA, CL840.Contig1\_Mf\_liverA, NM\_145218, NM\_025336, Unigene4841\_Mf\_liverA, Unigene23185\_Mf\_liverA, NM\_010158, Unigene20372\_Mf\_liverA, Unigene29985\_Mf\_liverA, Unigene13018\_Mf\_liverA, CL3104.Contig1\_Mf\_liverA, Unigene36593\_Mf\_liverA, Unigene37616\_Mf\_liverA, NM\_146016, Unigene36673\_Mf\_liverA, NM\_001253832, Unigene6110\_Mf\_liverA, CL5698.Contig1\_Mf\_liverA, NM\_023256, Unigene31392\_Mf\_liverA, CL591.Contig1\_Mf\_liverA, Unigene4556\_Mf\_liverA, NM\_007763, Unigene26055\_Mf\_liverA, CL139.Contig2\_Mf\_liverA, Unigene29399\_Mf\_liverA, Unigene38015\_Mf\_liverA, NM\_008292, Unigene33607\_Mf\_liverA, Unigene31199\_Mf\_liverA, Unigene6959\_Mf\_liverA, Unigene29308\_Mf\_liverA, CL523.Contig1\_Mf\_liverA, CL482.Contig1\_Mf\_liverA, Unigene36762\_Mf\_liverA, CL3725.Contig1\_Mf\_liverA, NM\_007820, Unigene30878\_Mf\_liverA, Unigene40289\_Mf\_liverA, CL44.Contig1\_Mf\_liverA, Unigene37880\_Mf\_liverA, Unigene38331\_Mf\_liverA, Unigene25070\_Mf\_liverA, CL2001.Contig1\_Mf\_liverA, Unigene26194\_Mf\_liverA, Unigene25596\_Mf\_liverA, Unigene843\_Mf\_liverA, Unigene12153\_Mf\_liverA, Unigene25046\_Mf\_liverA, CL5268.Contig1\_Mf\_liverA, Unigene2745\_Mf\_liverA, Unigene5775\_Mf\_liverA, CL787.Contig1\_Mf\_liverA, CL4076.Contig1\_Mf\_liverA, Unigene39507\_Mf\_liverA, Unigene25524\_Mf\_liverA, Unigene4\_Mf\_liverA, CL3750.Contig2\_Mf\_liverA, Unigene39141\_Mf\_liverA, Unigene33459\_Mf\_liverA, Unigene5632\_Mf\_liverA, Unigene36669\_Mf\_liverA, CL2632.Contig2\_Mf\_liverA, Unigene14582\_Mf\_liverA, Unigene24503\_Mf\_liverA, Unigene27422\_Mf\_liverA, CL4701.Contig1\_Mf\_liverA, CL5631.Contig1\_Mf\_liverA, Unigene36757\_Mf\_liverA, Unigene31427\_Mf\_liverA, Unigene30584\_Mf\_liverA, Unigene17579\_Mf\_liverA, CL4220.Contig2\_Mf\_liverA, Unigene431\_Mf\_liverA, NM\_001081372, NM\_010391, Unigene21317\_Mf\_liverA, NM\_010227, Unigene30493\_Mf\_liverA, Unigene8132\_Mf\_liverA, NM\_019717, Unigene27082\_Mf\_liverA, Unigene37178\_Mf\_liverA, Unigene5712\_Mf\_liverA, Unigene43107\_Mf\_liverA, CL442.Contig2\_Mf\_liverA, NM\_033374, CL2855.Contig1\_Mf\_liverA, CL4411.Contig4\_Mf\_liverA, NM\_013821, NM\_001025388, Unigene13233\_Mf\_liverA, Unigene40796\_Mf\_liverA, NM\_011099, NM\_001100182, NM\_010481, CL4086.Contig1\_Mf\_liverA, Unigene15318\_Mf\_liverA, NM\_008293, Unigene36190\_Mf\_liverA, Unigene7970\_Mf\_liverA, NM\_009255, Unigene30154\_Mf\_liverA, Unigene15982\_Mf\_liverA, CL4162.Contig1\_Mf\_liverA, Unigene112\_Mf\_liverA, Unigene35237\_Mf\_liverA, Unigene591\_Mf\_liverA, Unigene12907\_Mf\_liverA, Unigene25292\_Mf\_liverA, Unigene15588\_Mf\_liverA, NM\_011170, CL5796.Contig2\_Mf\_liverA, Unigene36414\_Mf\_liverA, Unigene13894\_Mf\_liverA, Unigene5175\_Mf\_liverA, CL1988.Contig1\_Mf\_liverA, Unigene36328\_Mf\_liverA, CL6039.Contig2\_Mf\_liverA, CL887.Contig2\_Mf\_liverA, Unigene32695\_Mf\_liverA, Unigene5940\_Mf\_liverA, Unigene43357\_Mf\_liverA, Unigene28731\_Mf\_liverA, Unigene1212\_Mf\_liverA, CL3549.Contig1\_Mf\_liverA, CL3750.Contig1\_Mf\_liverA, Unigene32294\_Mf\_liverA, Unigene18796\_Mf\_liverA, CL5307.Contig1\_Mf\_liverA, Unigene3377\_Mf\_liverA, NM\_178405, CL1052.Contig1\_Mf\_liverA, CL4048.Contig1\_Mf\_liverA, Unigene33525\_Mf\_liverA, Unigene9406\_Mf\_liverA, Unigene120\_Mf\_liverA, Unigene14940\_Mf\_liverA, Unigene15026\_Mf\_liverA, CL4007.Contig1\_Mf\_liverA, Unigene1221\_Mf\_liverA, Unigene39875\_Mf\_liverA, Unigene1292\_Mf\_liverA, Unigene26309\_Mf\_liverA, Unigene32295\_Mf\_liverA, Unigene5745\_Mf\_liverA, Unigene4636\_Mf\_liverA, Unigene12889\_Mf\_liverA, Unigene665\_Mf\_liverA, Unigene38514\_Mf\_liverA, NM\_001099634, Unigene8560\_Mf\_liverA, Unigene12519\_Mf\_liverA, Unigene16891\_Mf\_liverA, Unigene37819\_Mf\_liverA, Unigene5886\_Mf\_liverA, CL336.Contig3\_Mf\_liverA |
| organelle part | Unigene29399\_Mf\_liverA, NM\_176843, Unigene35884\_Mf\_liverA, NM\_020559, NM\_008292, Unigene38015\_Mf\_liverA, Unigene28142\_Mf\_liverA, NM\_009898, CL3835.Contig2\_Mf\_liverA, NM\_025593, Unigene28899\_Mf\_liverA, NM\_018815, Unigene29308\_Mf\_liverA, CL1575.Contig1\_Mf\_liverA, CL523.Contig1\_Mf\_liverA, NM\_080638, Unigene13153\_Mf\_liverA, Unigene34727\_Mf\_liverA, NM\_011082, Unigene29424\_Mf\_liverA, CL4160.Contig2\_Mf\_liverA, Unigene29008\_Mf\_liverA, CL4105.Contig1\_Mf\_liverA, CL482.Contig1\_Mf\_liverA, CL777.Contig8\_Mf\_liverA, NM\_007820, Unigene39886\_Mf\_liverA, Unigene21466\_Mf\_liverA, Unigene15064\_Mf\_liverA, Unigene30878\_Mf\_liverA, Unigene24252\_Mf\_liverA, CL4757.Contig1\_Mf\_liverA, Unigene34810\_Mf\_liverA, Unigene37880\_Mf\_liverA, CL5586.Contig1\_Mf\_liverA, Unigene41336\_Mf\_liverA, Unigene40020\_Mf\_liverA, Unigene34341\_Mf\_liverA, Unigene37535\_Mf\_liverA, Unigene25070\_Mf\_liverA, CL695.Contig1\_Mf\_liverA, CL4141.Contig1\_Mf\_liverA, Unigene35431\_Mf\_liverA, Unigene15703\_Mf\_liverA, CL2001.Contig1\_Mf\_liverA, Unigene35816\_Mf\_liverA, NM\_145474, Unigene13593\_Mf\_liverA, Unigene4686\_Mf\_liverA, Unigene34394\_Mf\_liverA, Unigene25596\_Mf\_liverA, NM\_031165, NM\_153193, Unigene5138\_Mf\_liverA, CL1493.Contig2\_Mf\_liverA, Unigene14508\_Mf\_liverA, Unigene25046\_Mf\_liverA, CL5268.Contig1\_Mf\_liverA, Unigene5294\_Mf\_liverA, Unigene5941\_Mf\_liverA, Unigene2745\_Mf\_liverA, Unigene8054\_Mf\_liverA, Unigene25462\_Mf\_liverA, Unigene802\_Mf\_liverA, Unigene5775\_Mf\_liverA, CL787.Contig1\_Mf\_liverA, Unigene5639\_Mf\_liverA, Unigene17048\_Mf\_liverA, CL5807.Contig1\_Mf\_liverA, Unigene28822\_Mf\_liverA, Unigene36417\_Mf\_liverA, CL2855.Contig2\_Mf\_liverA, NM\_177093, Unigene14907\_Mf\_liverA, Unigene33512\_Mf\_liverA, Unigene5382\_Mf\_liverA, NM\_201360, CL848.Contig2\_Mf\_liverA, Unigene23870\_Mf\_liverA, NM\_010001, Unigene4922\_Mf\_liverA, Unigene5632\_Mf\_liverA, Unigene25976\_Mf\_liverA, Unigene36669\_Mf\_liverA, Unigene14637\_Mf\_liverA, CL1263.Contig1\_Mf\_liverA, Unigene37460\_Mf\_liverA, Unigene31517\_Mf\_liverA, NM\_007811, NM\_007622, NM\_011305, NM\_001104531, Unigene139\_Mf\_liverA, CL3816.Contig1\_Mf\_liverA, CL4701.Contig1\_Mf\_liverA, Unigene36757\_Mf\_liverA, CL5631.Contig1\_Mf\_liverA, CL529.Contig2\_Mf\_liverA, Unigene37575\_Mf\_liverA, Unigene5774\_Mf\_liverA, CL4220.Contig2\_Mf\_liverA, Unigene25398\_Mf\_liverA, Unigene27081\_Mf\_liverA, Unigene27547\_Mf\_liverA, NM\_001081372, Unigene33366\_Mf\_liverA, NM\_134156, CL5316.Contig1\_Mf\_liverA, Unigene37389\_Mf\_liverA, Unigene21317\_Mf\_liverA, NM\_010227, Unigene45530\_Mf\_liverA, Unigene32332\_Mf\_liverA, Unigene30493\_Mf\_liverA, Unigene7195\_Mf\_liverA, Unigene8132\_Mf\_liverA, CL5254.Contig1\_Mf\_liverA, Unigene34010\_Mf\_liverA, NM\_019717, Unigene152\_Mf\_liverA, Unigene24613\_Mf\_liverA, Unigene27082\_Mf\_liverA, CL532.Contig1\_Mf\_liverA, Unigene5712\_Mf\_liverA, Unigene37076\_Mf\_liverA, Unigene1280\_Mf\_liverA, Unigene43107\_Mf\_liverA, Unigene1205\_Mf\_liverA, Unigene30288\_Mf\_liverA, CL442.Contig2\_Mf\_liverA, CL3166.Contig4\_Mf\_liverA, Unigene14171\_Mf\_liverA, Unigene32436\_Mf\_liverA, CL1352.Contig1\_Mf\_liverA, CL5576.Contig1\_Mf\_liverA, Unigene33632\_Mf\_liverA, CL2855.Contig1\_Mf\_liverA, Unigene2746\_Mf\_liverA, NM\_013821, Unigene40796\_Mf\_liverA, Unigene13233\_Mf\_liverA, NM\_007678, NM\_001100182, NM\_010481, NM\_009128, CL4925.Contig1\_Mf\_liverA, Unigene15318\_Mf\_liverA, CL2240.Contig1\_Mf\_liverA, CL425.Contig1\_Mf\_liverA, NM\_008293, CL2797.Contig2\_Mf\_liverA, Unigene7970\_Mf\_liverA, CL1803.Contig1\_Mf\_liverA, Unigene14916\_Mf\_liverA, CL1125.Contig1\_Mf\_liverA, CL4162.Contig1\_Mf\_liverA, Unigene35237\_Mf\_liverA, Unigene112\_Mf\_liverA, Unigene15077\_Mf\_liverA, CL4600.Contig1\_Mf\_liverA, Unigene5693\_Mf\_liverA, Unigene22052\_Mf\_liverA, NM\_010003, Unigene5165\_Mf\_liverA, CL5796.Contig2\_Mf\_liverA, Unigene30707\_Mf\_liverA, Unigene29876\_Mf\_liverA, CL2478.Contig3\_Mf\_liverA, Unigene36414\_Mf\_liverA, CL1988.Contig1\_Mf\_liverA, NM\_133838, Unigene36328\_Mf\_liverA, CL3002.Contig1\_Mf\_liverA, CL1988.Contig3\_Mf\_liverA, CL1810.Contig1\_Mf\_liverA, NM\_009609, Unigene36514\_Mf\_liverA, CL4033.Contig1\_Mf\_liverA, CL4816.Contig2\_Mf\_liverA, Unigene28662\_Mf\_liverA, Unigene4781\_Mf\_liverA, Unigene35935\_Mf\_liverA, CL1988.Contig2\_Mf\_liverA, Unigene32695\_Mf\_liverA, CL3207.Contig1\_Mf\_liverA, Unigene5906\_Mf\_liverA, NM\_001081148, Unigene5940\_Mf\_liverA, CL3900.Contig1\_Mf\_liverA, Unigene43357\_Mf\_liverA, Unigene28731\_Mf\_liverA, Unigene13683\_Mf\_liverA, CL1493.Contig1\_Mf\_liverA, NM\_033444, CL2791.Contig1\_Mf\_liverA, Unigene9698\_Mf\_liverA, Unigene28499\_Mf\_liverA, Unigene36420\_Mf\_liverA, Unigene10135\_Mf\_liverA, Unigene542\_Mf\_liverA, Unigene35476\_Mf\_liverA, Unigene24758\_Mf\_liverA, Unigene32294\_Mf\_liverA, Unigene4909\_Mf\_liverA, CL114.Contig2\_Mf\_liverA, Unigene24471\_Mf\_liverA, NM\_009883, NM\_010162, Unigene15529\_Mf\_liverA, CL4220.Contig1\_Mf\_liverA, CL5307.Contig1\_Mf\_liverA, Unigene3377\_Mf\_liverA, NM\_019879, CL2251.Contig1\_Mf\_liverA, CL738.Contig2\_Mf\_liverA, CL3816.Contig2\_Mf\_liverA, Unigene14270\_Mf\_liverA, NM\_007822, CL4490.Contig2\_Mf\_liverA, Unigene25595\_Mf\_liverA, CL4816.Contig3\_Mf\_liverA, NM\_012030, Unigene30731\_Mf\_liverA, NM\_025336, Unigene35609\_Mf\_liverA, CL4007.Contig1\_Mf\_liverA, Unigene36418\_Mf\_liverA, Unigene1221\_Mf\_liverA, Unigene4841\_Mf\_liverA, CL3835.Contig1\_Mf\_liverA, Unigene7048\_Mf\_liverA, Unigene23185\_Mf\_liverA, Unigene35046\_Mf\_liverA, NM\_010158, Unigene1292\_Mf\_liverA, CL2355.Contig1\_Mf\_liverA, Unigene37153\_Mf\_liverA, CL3104.Contig1\_Mf\_liverA, Unigene14809\_Mf\_liverA, Unigene32295\_Mf\_liverA, Unigene36593\_Mf\_liverA, Unigene15592\_Mf\_liverA, Unigene5745\_Mf\_liverA, Unigene12889\_Mf\_liverA, Unigene36699\_Mf\_liverA, Unigene36673\_Mf\_liverA, Unigene496\_Mf\_liverA, NM\_001099634, CL5698.Contig1\_Mf\_liverA, Unigene6110\_Mf\_liverA, Unigene31392\_Mf\_liverA, Unigene4681\_Mf\_liverA, Unigene16891\_Mf\_liverA, CL591.Contig1\_Mf\_liverA, Unigene4556\_Mf\_liverA, Unigene5886\_Mf\_liverA, CL336.Contig3\_Mf\_liverA |
| nuclear membrane | CL2478.Contig3\_Mf\_liverA, Unigene8132\_Mf\_liverA, Unigene37535\_Mf\_liverA |
| nuclear speck | CL2355.Contig1\_Mf\_liverA, Unigene35816\_Mf\_liverA |
| cytoskeletal part | Unigene5639\_Mf\_liverA, NM\_009898, Unigene28822\_Mf\_liverA, NM\_177093, Unigene15318\_Mf\_liverA, CL425.Contig1\_Mf\_liverA, CL848.Contig2\_Mf\_liverA, Unigene29308\_Mf\_liverA, Unigene23870\_Mf\_liverA, Unigene29008\_Mf\_liverA, Unigene14637\_Mf\_liverA, CL4162.Contig1\_Mf\_liverA, NM\_012030, CL4701.Contig1\_Mf\_liverA, Unigene5165\_Mf\_liverA, Unigene24252\_Mf\_liverA, Unigene30707\_Mf\_liverA, Unigene27547\_Mf\_liverA, Unigene27081\_Mf\_liverA, Unigene33366\_Mf\_liverA, NM\_134156, Unigene21317\_Mf\_liverA, Unigene25070\_Mf\_liverA, CL1810.Contig1\_Mf\_liverA, NM\_009609, Unigene13593\_Mf\_liverA, Unigene496\_Mf\_liverA, Unigene27082\_Mf\_liverA, Unigene5138\_Mf\_liverA, Unigene37076\_Mf\_liverA, Unigene5940\_Mf\_liverA, CL3207.Contig1\_Mf\_liverA, CL5698.Contig1\_Mf\_liverA, Unigene25046\_Mf\_liverA, CL5576.Contig1\_Mf\_liverA, CL591.Contig1\_Mf\_liverA, Unigene4556\_Mf\_liverA, Unigene5941\_Mf\_liverA, Unigene2746\_Mf\_liverA, Unigene2745\_Mf\_liverA |
| cell fraction | NM\_001100182, NM\_001081372, NM\_009128, NM\_177093, NM\_009609, NM\_201360, NM\_008293, NM\_011921, NM\_009255, NR\_003623, NM\_010001, NM\_145474, NM\_178405, NM\_007822, NM\_153505, NM\_031165, NM\_007811, NM\_153193, NM\_001081148, NM\_001253832, NM\_001104531, NM\_033444, NM\_011072, NM\_007820, NR\_024097, NM\_010003, NM\_001025388, NM\_013821, NM\_011099, NM\_011170 |
| intracellular | Unigene21684\_Mf\_liverA, NM\_176843, NM\_009898, NM\_025593, Unigene28186\_Mf\_liverA, Unigene15553\_Mf\_liverA, NM\_010378, CL854.Contig1\_Mf\_liverA, Unigene16463\_Mf\_liverA, NM\_025613, Unigene39886\_Mf\_liverA, Unigene15064\_Mf\_liverA, Unigene24252\_Mf\_liverA, CL4757.Contig1\_Mf\_liverA, NR\_004446, Unigene37535\_Mf\_liverA, CL695.Contig1\_Mf\_liverA, Unigene35431\_Mf\_liverA, Unigene15703\_Mf\_liverA, Unigene31623\_Mf\_liverA, NM\_010233, Unigene13379\_Mf\_liverA, Unigene34394\_Mf\_liverA, NM\_172409, Unigene25594\_Mf\_liverA, Unigene14050\_Mf\_liverA, CL4995.Contig1\_Mf\_liverA, Unigene8054\_Mf\_liverA, Unigene18430\_Mf\_liverA, CL5807.Contig1\_Mf\_liverA, CL2384.Contig1\_Mf\_liverA, Unigene36417\_Mf\_liverA, NM\_177093, CL4293.Contig1\_Mf\_liverA, Unigene25226\_Mf\_liverA, Unigene33512\_Mf\_liverA, Unigene14907\_Mf\_liverA, Unigene25976\_Mf\_liverA, Unigene36698\_Mf\_liverA, NM\_145942, Unigene30261\_Mf\_liverA, Unigene13945\_Mf\_liverA, Unigene139\_Mf\_liverA, Unigene28217\_Mf\_liverA, Unigene36836\_Mf\_liverA, Unigene33366\_Mf\_liverA, NM\_134156, Unigene37389\_Mf\_liverA, Unigene4720\_Mf\_liverA, Unigene27248\_Mf\_liverA, CL2266.Contig2\_Mf\_liverA, Unigene30839\_Mf\_liverA, Unigene34010\_Mf\_liverA, Unigene24613\_Mf\_liverA, Unigene152\_Mf\_liverA, Unigene31852\_Mf\_liverA, CL532.Contig1\_Mf\_liverA, Unigene37076\_Mf\_liverA, Unigene1280\_Mf\_liverA, Unigene30288\_Mf\_liverA, Unigene14171\_Mf\_liverA, CL5576.Contig1\_Mf\_liverA, Unigene33632\_Mf\_liverA, CL5189.Contig1\_Mf\_liverA, CL3339.Contig1\_Mf\_liverA, Unigene2746\_Mf\_liverA, Unigene4593\_Mf\_liverA, Unigene4363\_Mf\_liverA, CL425.Contig1\_Mf\_liverA, CL2797.Contig2\_Mf\_liverA, CL1803.Contig1\_Mf\_liverA, Unigene37243\_Mf\_liverA, Unigene30587\_Mf\_liverA, Unigene15077\_Mf\_liverA, Unigene36034\_Mf\_liverA, Unigene5693\_Mf\_liverA, Unigene39970\_Mf\_liverA, CL3393.Contig1\_Mf\_liverA, Unigene29876\_Mf\_liverA, CL3800.Contig1\_Mf\_liverA, Unigene28687\_Mf\_liverA, CL422.Contig1\_Mf\_liverA, CL1988.Contig3\_Mf\_liverA, NM\_028785, NM\_009609, CL1810.Contig1\_Mf\_liverA, CL4033.Contig1\_Mf\_liverA, Unigene24547\_Mf\_liverA, Unigene34983\_Mf\_liverA, NR\_003623, CL3519.Contig1\_Mf\_liverA, Unigene8033\_Mf\_liverA, CL1493.Contig1\_Mf\_liverA, Unigene35476\_Mf\_liverA, Unigene4909\_Mf\_liverA, Unigene24471\_Mf\_liverA, Unigene31571\_Mf\_liverA, NM\_010162, Unigene15529\_Mf\_liverA, CL4220.Contig1\_Mf\_liverA, Unigene37262\_Mf\_liverA, NR\_033215, CL2251.Contig1\_Mf\_liverA, NM\_007822, CL5293.Contig1\_Mf\_liverA, CL4490.Contig2\_Mf\_liverA, Unigene25595\_Mf\_liverA, Unigene14286\_Mf\_liverA, Unigene32515\_Mf\_liverA, CL4816.Contig3\_Mf\_liverA, Unigene39011\_Mf\_liverA, Unigene20432\_Mf\_liverA, NM\_009022, Unigene35609\_Mf\_liverA, Unigene7048\_Mf\_liverA, Unigene13950\_Mf\_liverA, Unigene35046\_Mf\_liverA, Unigene28459\_Mf\_liverA, Unigene37245\_Mf\_liverA, Unigene13847\_Mf\_liverA, Unigene19658\_Mf\_liverA, NM\_027406, Unigene36699\_Mf\_liverA, Unigene36176\_Mf\_liverA, Unigene18340\_Mf\_liverA, Unigene496\_Mf\_liverA, NM\_001164598, Unigene4983\_Mf\_liverA, Unigene29889\_Mf\_liverA, Unigene2939\_Mf\_liverA, Unigene9466\_Mf\_liverA, CL5640.Contig1\_Mf\_liverA, NM\_020559, Unigene35884\_Mf\_liverA, Unigene28142\_Mf\_liverA, CL3835.Contig2\_Mf\_liverA, Unigene28899\_Mf\_liverA, Unigene46870\_Mf\_liverA, CL1575.Contig1\_Mf\_liverA, NM\_019651, Unigene13153\_Mf\_liverA, CL4117.Contig1\_Mf\_liverA, Unigene8473\_Mf\_liverA, Unigene29008\_Mf\_liverA, Unigene33508\_Mf\_liverA, CL777.Contig8\_Mf\_liverA, NM\_011072, Unigene4776\_Mf\_liverA, Unigene21466\_Mf\_liverA, Unigene34810\_Mf\_liverA, Unigene27420\_Mf\_liverA, Unigene20602\_Mf\_liverA, Unigene37999\_Mf\_liverA, Unigene40020\_Mf\_liverA, Unigene37904\_Mf\_liverA, Unigene41336\_Mf\_liverA, CL3483.Contig1\_Mf\_liverA, CL4141.Contig1\_Mf\_liverA, Unigene34184\_Mf\_liverA, CL5191.Contig2\_Mf\_liverA, Unigene13593\_Mf\_liverA, Unigene4686\_Mf\_liverA, Unigene11\_Mf\_liverA, Unigene45131\_Mf\_liverA, Unigene5994\_Mf\_liverA, CL1493.Contig2\_Mf\_liverA, Unigene5294\_Mf\_liverA, Unigene5941\_Mf\_liverA, Unigene38104\_Mf\_liverA, Unigene38065\_Mf\_liverA, Unigene25462\_Mf\_liverA, Unigene36626\_Mf\_liverA, NM\_153795, Unigene28822\_Mf\_liverA, Unigene31988\_Mf\_liverA, Unigene5382\_Mf\_liverA, Unigene151\_Mf\_liverA, NM\_201360, CL848.Contig2\_Mf\_liverA, Unigene23870\_Mf\_liverA, CL1263.Contig1\_Mf\_liverA, Unigene37460\_Mf\_liverA, Unigene31517\_Mf\_liverA, NM\_007811, Unigene24323\_Mf\_liverA, CL2142.Contig2\_Mf\_liverA, NM\_011305, CL3816.Contig1\_Mf\_liverA, Unigene25333\_Mf\_liverA, CL993.Contig2\_Mf\_liverA, CL529.Contig2\_Mf\_liverA, Unigene27081\_Mf\_liverA, Unigene37148\_Mf\_liverA, Unigene7195\_Mf\_liverA, Unigene1205\_Mf\_liverA, CL1352.Contig1\_Mf\_liverA, CL6039.Contig1\_Mf\_liverA, Unigene24804\_Mf\_liverA, Unigene31206\_Mf\_liverA, NM\_009128, CL2240.Contig1\_Mf\_liverA, CL1125.Contig1\_Mf\_liverA, Unigene15681\_Mf\_liverA, Unigene5165\_Mf\_liverA, Unigene30707\_Mf\_liverA, NM\_133838, Unigene15552\_Mf\_liverA, CL3002.Contig1\_Mf\_liverA, Unigene30155\_Mf\_liverA, CL5057.Contig1\_Mf\_liverA, Unigene36514\_Mf\_liverA, NM\_145836, CL4816.Contig2\_Mf\_liverA, Unigene37139\_Mf\_liverA, Unigene33523\_Mf\_liverA, CL3207.Contig1\_Mf\_liverA, Unigene5906\_Mf\_liverA, Unigene14810\_Mf\_liverA, NM\_033444, CL2791.Contig1\_Mf\_liverA, CL4770.Contig1\_Mf\_liverA, Unigene10135\_Mf\_liverA, Unigene1483\_Mf\_liverA, CL114.Contig2\_Mf\_liverA, NM\_009883, Unigene33526\_Mf\_liverA, Unigene1327\_Mf\_liverA, Unigene39252\_Mf\_liverA, NM\_001081172, CL5978.Contig2\_Mf\_liverA, Unigene8740\_Mf\_liverA, CL738.Contig2\_Mf\_liverA, CL3816.Contig2\_Mf\_liverA, Unigene14270\_Mf\_liverA, CL442.Contig5\_Mf\_liverA, CL5459.Contig2\_Mf\_liverA, NM\_012030, Unigene24477\_Mf\_liverA, Unigene30731\_Mf\_liverA, Unigene30983\_Mf\_liverA, Unigene36418\_Mf\_liverA, CL3835.Contig1\_Mf\_liverA, Unigene38311\_Mf\_liverA, Unigene550\_Mf\_liverA, Unigene31198\_Mf\_liverA, CL2355.Contig1\_Mf\_liverA, Unigene28314\_Mf\_liverA, Unigene37153\_Mf\_liverA, Unigene14809\_Mf\_liverA, Unigene21562\_Mf\_liverA, Unigene15592\_Mf\_liverA, Unigene2195\_Mf\_liverA, Unigene30892\_Mf\_liverA, Unigene34866\_Mf\_liverA, Unigene31436\_Mf\_liverA, Unigene4681\_Mf\_liverA, Unigene5287\_Mf\_liverA, Unigene34609\_Mf\_liverA, NM\_144940, Unigene25721\_Mf\_liverA, CL3669.Contig2\_Mf\_liverA, Unigene37334\_Mf\_liverA, NM\_018815, NM\_080638, Unigene29424\_Mf\_liverA, NM\_011082, Unigene34727\_Mf\_liverA, CL4160.Contig2\_Mf\_liverA, CL4105.Contig1\_Mf\_liverA, Unigene26053\_Mf\_liverA, Unigene11007\_Mf\_liverA, CL4577.Contig1\_Mf\_liverA, Unigene785\_Mf\_liverA, Unigene27339\_Mf\_liverA, NM\_021273, CL5586.Contig1\_Mf\_liverA, Unigene34341\_Mf\_liverA, Unigene30142\_Mf\_liverA, NM\_011921, Unigene35816\_Mf\_liverA, Unigene7897\_Mf\_liverA, NM\_145474, NM\_009776, Unigene30003\_Mf\_liverA, Unigene5138\_Mf\_liverA, NM\_153193, NM\_031165, Unigene14508\_Mf\_liverA, Unigene34143\_Mf\_liverA, Unigene30002\_Mf\_liverA, Unigene802\_Mf\_liverA, Unigene9150\_Mf\_liverA, Unigene17048\_Mf\_liverA, Unigene5639\_Mf\_liverA, Unigene33522\_Mf\_liverA, Unigene13363\_Mf\_liverA, CL2855.Contig2\_Mf\_liverA, NM\_021278, Unigene21561\_Mf\_liverA, NM\_010001, Unigene39655\_Mf\_liverA, Unigene4922\_Mf\_liverA, Unigene14637\_Mf\_liverA, NM\_153505, NM\_007622, NM\_001104531, Unigene4944\_Mf\_liverA, Unigene23158\_Mf\_liverA, Unigene37575\_Mf\_liverA, Unigene25398\_Mf\_liverA, Unigene5774\_Mf\_liverA, Unigene27547\_Mf\_liverA, CL1736.Contig2\_Mf\_liverA, CL5316.Contig1\_Mf\_liverA, Unigene45530\_Mf\_liverA, Unigene4723\_Mf\_liverA, Unigene32332\_Mf\_liverA, Unigene29063\_Mf\_liverA, Unigene37470\_Mf\_liverA, CL5254.Contig1\_Mf\_liverA, Unigene35169\_Mf\_liverA, CL3166.Contig4\_Mf\_liverA, Unigene35698\_Mf\_liverA, Unigene32436\_Mf\_liverA, Unigene16465\_Mf\_liverA, Unigene30528\_Mf\_liverA, NM\_007678, Unigene31251\_Mf\_liverA, Unigene40610\_Mf\_liverA, CL186.Contig3\_Mf\_liverA, CL4925.Contig1\_Mf\_liverA, Unigene32421\_Mf\_liverA, Unigene583\_Mf\_liverA, CL4736.Contig1\_Mf\_liverA, Unigene27026\_Mf\_liverA, Unigene14916\_Mf\_liverA, Unigene35858\_Mf\_liverA, CL4600.Contig1\_Mf\_liverA, Unigene29231\_Mf\_liverA, NM\_010003, Unigene22052\_Mf\_liverA, Unigene34867\_Mf\_liverA, CL2478.Contig3\_Mf\_liverA, Unigene5815\_Mf\_liverA, NM\_024474, Unigene18499\_Mf\_liverA, Unigene38870\_Mf\_liverA, Unigene28662\_Mf\_liverA, Unigene4781\_Mf\_liverA, CL2439.Contig1\_Mf\_liverA, CL1988.Contig2\_Mf\_liverA, Unigene35935\_Mf\_liverA, Unigene29426\_Mf\_liverA, Unigene20371\_Mf\_liverA, CL3900.Contig1\_Mf\_liverA, Unigene13683\_Mf\_liverA, Unigene9698\_Mf\_liverA, Unigene30585\_Mf\_liverA, Unigene36420\_Mf\_liverA, Unigene28499\_Mf\_liverA, Unigene542\_Mf\_liverA, Unigene24758\_Mf\_liverA, Unigene33080\_Mf\_liverA, NM\_019879, CL5293.Contig2\_Mf\_liverA, CL840.Contig1\_Mf\_liverA, NM\_145218, NM\_025336, Unigene4841\_Mf\_liverA, Unigene23185\_Mf\_liverA, NM\_010158, Unigene20372\_Mf\_liverA, Unigene29985\_Mf\_liverA, Unigene13018\_Mf\_liverA, CL3104.Contig1\_Mf\_liverA, Unigene36593\_Mf\_liverA, Unigene37616\_Mf\_liverA, NM\_146016, Unigene36673\_Mf\_liverA, NM\_001253832, Unigene6110\_Mf\_liverA, CL5698.Contig1\_Mf\_liverA, NM\_023256, Unigene31392\_Mf\_liverA, CL591.Contig1\_Mf\_liverA, Unigene4556\_Mf\_liverA, NM\_007763, Unigene26055\_Mf\_liverA, CL139.Contig2\_Mf\_liverA, Unigene29399\_Mf\_liverA, Unigene38015\_Mf\_liverA, NM\_008292, Unigene33607\_Mf\_liverA, Unigene31199\_Mf\_liverA, Unigene6959\_Mf\_liverA, Unigene29308\_Mf\_liverA, CL523.Contig1\_Mf\_liverA, CL482.Contig1\_Mf\_liverA, Unigene36762\_Mf\_liverA, CL3725.Contig1\_Mf\_liverA, NM\_007820, Unigene30878\_Mf\_liverA, Unigene40289\_Mf\_liverA, CL44.Contig1\_Mf\_liverA, Unigene37880\_Mf\_liverA, Unigene38331\_Mf\_liverA, Unigene25070\_Mf\_liverA, CL2001.Contig1\_Mf\_liverA, Unigene26194\_Mf\_liverA, Unigene25596\_Mf\_liverA, Unigene843\_Mf\_liverA, Unigene12153\_Mf\_liverA, Unigene25046\_Mf\_liverA, Unigene13918\_Mf\_liverA, CL5268.Contig1\_Mf\_liverA, Unigene2745\_Mf\_liverA, Unigene5775\_Mf\_liverA, CL787.Contig1\_Mf\_liverA, CL4076.Contig1\_Mf\_liverA, Unigene39507\_Mf\_liverA, Unigene25524\_Mf\_liverA, Unigene4\_Mf\_liverA, CL3750.Contig2\_Mf\_liverA, Unigene39141\_Mf\_liverA, Unigene33459\_Mf\_liverA, Unigene5632\_Mf\_liverA, Unigene36669\_Mf\_liverA, CL2632.Contig2\_Mf\_liverA, Unigene14582\_Mf\_liverA, Unigene24503\_Mf\_liverA, Unigene27422\_Mf\_liverA, CL4701.Contig1\_Mf\_liverA, CL5631.Contig1\_Mf\_liverA, Unigene36757\_Mf\_liverA, Unigene31427\_Mf\_liverA, Unigene30584\_Mf\_liverA, Unigene17579\_Mf\_liverA, CL4220.Contig2\_Mf\_liverA, Unigene431\_Mf\_liverA, NM\_001081372, NM\_010391, Unigene21317\_Mf\_liverA, NM\_010227, Unigene30493\_Mf\_liverA, Unigene8132\_Mf\_liverA, NM\_019717, Unigene27082\_Mf\_liverA, Unigene37178\_Mf\_liverA, Unigene5712\_Mf\_liverA, Unigene43107\_Mf\_liverA, CL442.Contig2\_Mf\_liverA, NM\_033374, CL2855.Contig1\_Mf\_liverA, CL4411.Contig4\_Mf\_liverA, NM\_013821, NM\_001025388, Unigene13233\_Mf\_liverA, Unigene40796\_Mf\_liverA, NM\_011099, NM\_001100182, NM\_010481, CL4086.Contig1\_Mf\_liverA, Unigene15318\_Mf\_liverA, NM\_008293, Unigene36190\_Mf\_liverA, Unigene7970\_Mf\_liverA, NM\_009255, Unigene30154\_Mf\_liverA, Unigene15982\_Mf\_liverA, CL4162.Contig1\_Mf\_liverA, Unigene112\_Mf\_liverA, Unigene35237\_Mf\_liverA, Unigene591\_Mf\_liverA, Unigene12907\_Mf\_liverA, Unigene25292\_Mf\_liverA, Unigene15588\_Mf\_liverA, NM\_011170, CL5796.Contig2\_Mf\_liverA, Unigene36414\_Mf\_liverA, Unigene13894\_Mf\_liverA, Unigene5175\_Mf\_liverA, CL1988.Contig1\_Mf\_liverA, Unigene36328\_Mf\_liverA, CL6039.Contig2\_Mf\_liverA, CL887.Contig2\_Mf\_liverA, Unigene32695\_Mf\_liverA, Unigene5940\_Mf\_liverA, Unigene43357\_Mf\_liverA, Unigene28731\_Mf\_liverA, Unigene1212\_Mf\_liverA, CL3549.Contig1\_Mf\_liverA, CL3750.Contig1\_Mf\_liverA, Unigene32294\_Mf\_liverA, Unigene18796\_Mf\_liverA, CL5307.Contig1\_Mf\_liverA, Unigene3377\_Mf\_liverA, NM\_178405, Unigene33525\_Mf\_liverA, CL4048.Contig1\_Mf\_liverA, CL1052.Contig1\_Mf\_liverA, Unigene9406\_Mf\_liverA, Unigene120\_Mf\_liverA, Unigene14940\_Mf\_liverA, Unigene15026\_Mf\_liverA, CL4007.Contig1\_Mf\_liverA, Unigene1221\_Mf\_liverA, Unigene39875\_Mf\_liverA, Unigene1292\_Mf\_liverA, Unigene26309\_Mf\_liverA, Unigene32295\_Mf\_liverA, Unigene4636\_Mf\_liverA, Unigene5745\_Mf\_liverA, Unigene38514\_Mf\_liverA, Unigene665\_Mf\_liverA, Unigene12889\_Mf\_liverA, NM\_001099634, Unigene8560\_Mf\_liverA, Unigene16891\_Mf\_liverA, Unigene12519\_Mf\_liverA, Unigene37819\_Mf\_liverA, Unigene5886\_Mf\_liverA, CL336.Contig3\_Mf\_liverA |
| intracellular membrane-bounded organelle | Unigene21684\_Mf\_liverA, Unigene5287\_Mf\_liverA, NM\_176843, NM\_009898, Unigene25721\_Mf\_liverA, CL3669.Contig2\_Mf\_liverA, NM\_025593, NM\_018815, Unigene28186\_Mf\_liverA, NM\_080638, Unigene15553\_Mf\_liverA, NM\_010378, Unigene34727\_Mf\_liverA, NM\_011082, Unigene29424\_Mf\_liverA, CL4160.Contig2\_Mf\_liverA, CL854.Contig1\_Mf\_liverA, CL4105.Contig1\_Mf\_liverA, Unigene11007\_Mf\_liverA, CL4577.Contig1\_Mf\_liverA, NM\_025613, Unigene39886\_Mf\_liverA, Unigene15064\_Mf\_liverA, NM\_021273, CL4757.Contig1\_Mf\_liverA, NR\_004446, CL5586.Contig1\_Mf\_liverA, Unigene34341\_Mf\_liverA, Unigene37535\_Mf\_liverA, Unigene30142\_Mf\_liverA, CL695.Contig1\_Mf\_liverA, Unigene35431\_Mf\_liverA, Unigene15703\_Mf\_liverA, Unigene35816\_Mf\_liverA, Unigene31623\_Mf\_liverA, Unigene7897\_Mf\_liverA, NM\_145474, Unigene13379\_Mf\_liverA, NM\_009776, Unigene34394\_Mf\_liverA, NM\_031165, Unigene25594\_Mf\_liverA, NM\_153193, Unigene5138\_Mf\_liverA, Unigene14508\_Mf\_liverA, Unigene14050\_Mf\_liverA, CL4995.Contig1\_Mf\_liverA, Unigene8054\_Mf\_liverA, Unigene18430\_Mf\_liverA, Unigene802\_Mf\_liverA, Unigene17048\_Mf\_liverA, CL5807.Contig1\_Mf\_liverA, Unigene13363\_Mf\_liverA, CL2384.Contig1\_Mf\_liverA, Unigene36417\_Mf\_liverA, NM\_021278, CL2855.Contig2\_Mf\_liverA, NM\_177093, Unigene25226\_Mf\_liverA, Unigene33512\_Mf\_liverA, Unigene14907\_Mf\_liverA, Unigene21561\_Mf\_liverA, Unigene39655\_Mf\_liverA, NM\_010001, Unigene4922\_Mf\_liverA, Unigene25976\_Mf\_liverA, Unigene36698\_Mf\_liverA, Unigene30261\_Mf\_liverA, NM\_007622, Unigene13945\_Mf\_liverA, NM\_001104531, Unigene139\_Mf\_liverA, Unigene4944\_Mf\_liverA, Unigene23158\_Mf\_liverA, Unigene37575\_Mf\_liverA, Unigene5774\_Mf\_liverA, Unigene25398\_Mf\_liverA, Unigene27547\_Mf\_liverA, CL1736.Contig2\_Mf\_liverA, Unigene36836\_Mf\_liverA, Unigene33366\_Mf\_liverA, NM\_134156, CL5316.Contig1\_Mf\_liverA, Unigene37389\_Mf\_liverA, Unigene4720\_Mf\_liverA, Unigene45530\_Mf\_liverA, Unigene29063\_Mf\_liverA, Unigene32332\_Mf\_liverA, Unigene4723\_Mf\_liverA, CL5254.Contig1\_Mf\_liverA, Unigene34010\_Mf\_liverA, Unigene24613\_Mf\_liverA, Unigene152\_Mf\_liverA, CL532.Contig1\_Mf\_liverA, Unigene37076\_Mf\_liverA, Unigene1280\_Mf\_liverA, Unigene30288\_Mf\_liverA, CL3166.Contig4\_Mf\_liverA, Unigene14171\_Mf\_liverA, Unigene32436\_Mf\_liverA, Unigene33632\_Mf\_liverA, CL5189.Contig1\_Mf\_liverA, CL3339.Contig1\_Mf\_liverA, Unigene2746\_Mf\_liverA, Unigene30528\_Mf\_liverA, NM\_007678, Unigene31251\_Mf\_liverA, Unigene4363\_Mf\_liverA, Unigene40610\_Mf\_liverA, CL186.Contig3\_Mf\_liverA, CL4925.Contig1\_Mf\_liverA, CL425.Contig1\_Mf\_liverA, Unigene32421\_Mf\_liverA, CL2797.Contig2\_Mf\_liverA, CL1803.Contig1\_Mf\_liverA, Unigene14916\_Mf\_liverA, Unigene37243\_Mf\_liverA, Unigene35858\_Mf\_liverA, Unigene30587\_Mf\_liverA, Unigene15077\_Mf\_liverA, CL4600.Contig1\_Mf\_liverA, Unigene36034\_Mf\_liverA, Unigene29231\_Mf\_liverA, Unigene5693\_Mf\_liverA, Unigene22052\_Mf\_liverA, NM\_010003, Unigene34867\_Mf\_liverA, Unigene29876\_Mf\_liverA, CL3800.Contig1\_Mf\_liverA, CL2478.Contig3\_Mf\_liverA, Unigene28687\_Mf\_liverA, CL1988.Contig3\_Mf\_liverA, NM\_024474, NM\_009609, CL1810.Contig1\_Mf\_liverA, Unigene18499\_Mf\_liverA, CL4033.Contig1\_Mf\_liverA, Unigene24547\_Mf\_liverA, Unigene34983\_Mf\_liverA, Unigene28662\_Mf\_liverA, CL2439.Contig1\_Mf\_liverA, Unigene4781\_Mf\_liverA, NR\_003623, Unigene35935\_Mf\_liverA, CL1988.Contig2\_Mf\_liverA, CL3900.Contig1\_Mf\_liverA, Unigene13683\_Mf\_liverA, CL1493.Contig1\_Mf\_liverA, Unigene9698\_Mf\_liverA, Unigene28499\_Mf\_liverA, Unigene36420\_Mf\_liverA, Unigene30585\_Mf\_liverA, Unigene542\_Mf\_liverA, Unigene35476\_Mf\_liverA, Unigene24758\_Mf\_liverA, Unigene4909\_Mf\_liverA, Unigene24471\_Mf\_liverA, Unigene33080\_Mf\_liverA, NM\_010162, Unigene15529\_Mf\_liverA, CL4220.Contig1\_Mf\_liverA, NM\_019879, NR\_033215, CL2251.Contig1\_Mf\_liverA, NM\_007822, CL4490.Contig2\_Mf\_liverA, Unigene25595\_Mf\_liverA, Unigene14286\_Mf\_liverA, Unigene32515\_Mf\_liverA, CL4816.Contig3\_Mf\_liverA, CL840.Contig1\_Mf\_liverA, Unigene39011\_Mf\_liverA, NM\_009022, NM\_025336, Unigene35609\_Mf\_liverA, NM\_145218, Unigene4841\_Mf\_liverA, Unigene23185\_Mf\_liverA, Unigene7048\_Mf\_liverA, Unigene13950\_Mf\_liverA, Unigene35046\_Mf\_liverA, Unigene28459\_Mf\_liverA, Unigene37245\_Mf\_liverA, NM\_010158, Unigene13847\_Mf\_liverA, Unigene29985\_Mf\_liverA, CL3104.Contig1\_Mf\_liverA, Unigene36593\_Mf\_liverA, Unigene19658\_Mf\_liverA, Unigene37616\_Mf\_liverA, NM\_027406, Unigene36699\_Mf\_liverA, Unigene36673\_Mf\_liverA, Unigene496\_Mf\_liverA, NM\_001164598, Unigene4983\_Mf\_liverA, CL5698.Contig1\_Mf\_liverA, Unigene6110\_Mf\_liverA, NM\_001253832, Unigene31392\_Mf\_liverA, NM\_023256, Unigene9466\_Mf\_liverA, Unigene29399\_Mf\_liverA, NM\_020559, Unigene35884\_Mf\_liverA, Unigene33607\_Mf\_liverA, NM\_008292, Unigene28142\_Mf\_liverA, Unigene38015\_Mf\_liverA, CL3835.Contig2\_Mf\_liverA, Unigene31199\_Mf\_liverA, Unigene28899\_Mf\_liverA, CL1575.Contig1\_Mf\_liverA, Unigene46870\_Mf\_liverA, CL523.Contig1\_Mf\_liverA, CL4117.Contig1\_Mf\_liverA, Unigene13153\_Mf\_liverA, Unigene29008\_Mf\_liverA, CL482.Contig1\_Mf\_liverA, Unigene36762\_Mf\_liverA, CL777.Contig8\_Mf\_liverA, CL3725.Contig1\_Mf\_liverA, NM\_011072, NM\_007820, Unigene4776\_Mf\_liverA, Unigene21466\_Mf\_liverA, Unigene30878\_Mf\_liverA, Unigene40289\_Mf\_liverA, Unigene34810\_Mf\_liverA, CL44.Contig1\_Mf\_liverA, Unigene37880\_Mf\_liverA, Unigene38331\_Mf\_liverA, Unigene37999\_Mf\_liverA, Unigene37904\_Mf\_liverA, Unigene41336\_Mf\_liverA, Unigene25070\_Mf\_liverA, CL4141.Contig1\_Mf\_liverA, CL2001.Contig1\_Mf\_liverA, Unigene26194\_Mf\_liverA, Unigene4686\_Mf\_liverA, Unigene11\_Mf\_liverA, Unigene25596\_Mf\_liverA, Unigene45131\_Mf\_liverA, Unigene5994\_Mf\_liverA, CL1493.Contig2\_Mf\_liverA, Unigene12153\_Mf\_liverA, CL5268.Contig1\_Mf\_liverA, Unigene5294\_Mf\_liverA, Unigene2745\_Mf\_liverA, Unigene38104\_Mf\_liverA, Unigene38065\_Mf\_liverA, Unigene25462\_Mf\_liverA, Unigene5775\_Mf\_liverA, Unigene36626\_Mf\_liverA, CL787.Contig1\_Mf\_liverA, Unigene39507\_Mf\_liverA, Unigene25524\_Mf\_liverA, Unigene31988\_Mf\_liverA, CL3750.Contig2\_Mf\_liverA, Unigene39141\_Mf\_liverA, Unigene5382\_Mf\_liverA, NM\_201360, Unigene23870\_Mf\_liverA, Unigene5632\_Mf\_liverA, Unigene36669\_Mf\_liverA, CL1263.Contig1\_Mf\_liverA, Unigene37460\_Mf\_liverA, Unigene31517\_Mf\_liverA, NM\_007811, Unigene14582\_Mf\_liverA, CL2142.Contig2\_Mf\_liverA, NM\_011305, Unigene24503\_Mf\_liverA, Unigene27422\_Mf\_liverA, CL3816.Contig1\_Mf\_liverA, Unigene25333\_Mf\_liverA, Unigene36757\_Mf\_liverA, CL5631.Contig1\_Mf\_liverA, CL529.Contig2\_Mf\_liverA, Unigene30584\_Mf\_liverA, CL4220.Contig2\_Mf\_liverA, Unigene17579\_Mf\_liverA, Unigene27081\_Mf\_liverA, NM\_001081372, NM\_010391, Unigene37148\_Mf\_liverA, NM\_010227, Unigene30493\_Mf\_liverA, Unigene7195\_Mf\_liverA, Unigene8132\_Mf\_liverA, NM\_019717, Unigene37178\_Mf\_liverA, Unigene27082\_Mf\_liverA, Unigene5712\_Mf\_liverA, Unigene43107\_Mf\_liverA, Unigene1205\_Mf\_liverA, CL442.Contig2\_Mf\_liverA, CL2855.Contig1\_Mf\_liverA, CL4411.Contig4\_Mf\_liverA, NM\_001025388, Unigene31206\_Mf\_liverA, NM\_013821, Unigene40796\_Mf\_liverA, Unigene13233\_Mf\_liverA, NM\_011099, NM\_001100182, NM\_010481, NM\_009128, Unigene15318\_Mf\_liverA, CL2240.Contig1\_Mf\_liverA, Unigene36190\_Mf\_liverA, NM\_008293, Unigene7970\_Mf\_liverA, NM\_009255, Unigene15982\_Mf\_liverA, CL1125.Contig1\_Mf\_liverA, CL4162.Contig1\_Mf\_liverA, Unigene35237\_Mf\_liverA, Unigene112\_Mf\_liverA, Unigene12907\_Mf\_liverA, Unigene25292\_Mf\_liverA, Unigene5165\_Mf\_liverA, Unigene15588\_Mf\_liverA, CL5796.Contig2\_Mf\_liverA, NM\_011170, Unigene30707\_Mf\_liverA, Unigene36414\_Mf\_liverA, CL1988.Contig1\_Mf\_liverA, NM\_133838, Unigene5175\_Mf\_liverA, Unigene36328\_Mf\_liverA, Unigene15552\_Mf\_liverA, CL3002.Contig1\_Mf\_liverA, Unigene36514\_Mf\_liverA, NM\_145836, CL4816.Contig2\_Mf\_liverA, CL887.Contig2\_Mf\_liverA, Unigene32695\_Mf\_liverA, Unigene5906\_Mf\_liverA, Unigene43357\_Mf\_liverA, Unigene28731\_Mf\_liverA, Unigene14810\_Mf\_liverA, NM\_033444, Unigene1212\_Mf\_liverA, CL2791.Contig1\_Mf\_liverA, CL4770.Contig1\_Mf\_liverA, Unigene10135\_Mf\_liverA, Unigene32294\_Mf\_liverA, CL3750.Contig1\_Mf\_liverA, CL114.Contig2\_Mf\_liverA, NM\_009883, Unigene18796\_Mf\_liverA, CL5307.Contig1\_Mf\_liverA, Unigene3377\_Mf\_liverA, Unigene8740\_Mf\_liverA, NM\_178405, CL738.Contig2\_Mf\_liverA, CL3816.Contig2\_Mf\_liverA, Unigene14270\_Mf\_liverA, CL4048.Contig1\_Mf\_liverA, CL1052.Contig1\_Mf\_liverA, CL442.Contig5\_Mf\_liverA, NM\_012030, Unigene9406\_Mf\_liverA, Unigene24477\_Mf\_liverA, Unigene120\_Mf\_liverA, Unigene30731\_Mf\_liverA, CL4007.Contig1\_Mf\_liverA, Unigene15026\_Mf\_liverA, Unigene36418\_Mf\_liverA, Unigene14940\_Mf\_liverA, Unigene1221\_Mf\_liverA, CL3835.Contig1\_Mf\_liverA, Unigene39875\_Mf\_liverA, Unigene31198\_Mf\_liverA, Unigene1292\_Mf\_liverA, CL2355.Contig1\_Mf\_liverA, Unigene37153\_Mf\_liverA, Unigene14809\_Mf\_liverA, Unigene26309\_Mf\_liverA, Unigene32295\_Mf\_liverA, Unigene21562\_Mf\_liverA, Unigene15592\_Mf\_liverA, Unigene5745\_Mf\_liverA, Unigene665\_Mf\_liverA, Unigene2195\_Mf\_liverA, Unigene12889\_Mf\_liverA, NM\_001099634, Unigene8560\_Mf\_liverA, Unigene34866\_Mf\_liverA, Unigene4681\_Mf\_liverA, Unigene16891\_Mf\_liverA, Unigene37819\_Mf\_liverA, Unigene5886\_Mf\_liverA, CL336.Contig3\_Mf\_liverA |
| mitochondrial membrane part | CL3104.Contig1\_Mf\_liverA, Unigene34727\_Mf\_liverA |
| nuclear chromosome part | Unigene14907\_Mf\_liverA, CL5268.Contig1\_Mf\_liverA, NM\_007622, Unigene31392\_Mf\_liverA, CL4757.Contig1\_Mf\_liverA, Unigene45530\_Mf\_liverA |
| kinetochore | CL5268.Contig1\_Mf\_liverA, Unigene45530\_Mf\_liverA |
| transcription factor complex | Unigene35935\_Mf\_liverA, Unigene32436\_Mf\_liverA, Unigene14907\_Mf\_liverA, Unigene33366\_Mf\_liverA, NM\_007678, CL4757.Contig1\_Mf\_liverA |
| microtubule | Unigene27547\_Mf\_liverA, CL425.Contig1\_Mf\_liverA, Unigene2746\_Mf\_liverA, Unigene23870\_Mf\_liverA, Unigene2745\_Mf\_liverA |
| nuclear chromatin | Unigene14907\_Mf\_liverA, NM\_007622, CL4757.Contig1\_Mf\_liverA |
| chromosome, centromeric region | CL5268.Contig1\_Mf\_liverA, NM\_007622, Unigene45530\_Mf\_liverA |
| nuclear chromosome | Unigene14907\_Mf\_liverA, CL5268.Contig1\_Mf\_liverA, NM\_007622, CL3900.Contig1\_Mf\_liverA, Unigene31392\_Mf\_liverA, CL4757.Contig1\_Mf\_liverA, Unigene45530\_Mf\_liverA |
| intracellular organelle | Unigene21684\_Mf\_liverA, Unigene5287\_Mf\_liverA, NM\_176843, Unigene34609\_Mf\_liverA, NM\_009898, Unigene25721\_Mf\_liverA, CL3669.Contig2\_Mf\_liverA, NM\_025593, NM\_018815, Unigene28186\_Mf\_liverA, NM\_080638, Unigene15553\_Mf\_liverA, NM\_010378, Unigene34727\_Mf\_liverA, NM\_011082, Unigene29424\_Mf\_liverA, CL4160.Contig2\_Mf\_liverA, CL854.Contig1\_Mf\_liverA, CL4105.Contig1\_Mf\_liverA, Unigene11007\_Mf\_liverA, CL4577.Contig1\_Mf\_liverA, NM\_025613, Unigene39886\_Mf\_liverA, Unigene15064\_Mf\_liverA, NM\_021273, Unigene24252\_Mf\_liverA, CL4757.Contig1\_Mf\_liverA, NR\_004446, CL5586.Contig1\_Mf\_liverA, Unigene34341\_Mf\_liverA, Unigene37535\_Mf\_liverA, Unigene30142\_Mf\_liverA, CL695.Contig1\_Mf\_liverA, Unigene35431\_Mf\_liverA, Unigene15703\_Mf\_liverA, Unigene35816\_Mf\_liverA, Unigene31623\_Mf\_liverA, Unigene7897\_Mf\_liverA, NM\_145474, Unigene13379\_Mf\_liverA, NM\_009776, Unigene34394\_Mf\_liverA, NM\_031165, Unigene25594\_Mf\_liverA, NM\_153193, Unigene5138\_Mf\_liverA, Unigene14508\_Mf\_liverA, Unigene14050\_Mf\_liverA, CL4995.Contig1\_Mf\_liverA, Unigene8054\_Mf\_liverA, Unigene18430\_Mf\_liverA, Unigene802\_Mf\_liverA, Unigene5639\_Mf\_liverA, Unigene17048\_Mf\_liverA, CL5807.Contig1\_Mf\_liverA, Unigene13363\_Mf\_liverA, CL2384.Contig1\_Mf\_liverA, Unigene36417\_Mf\_liverA, NM\_021278, CL2855.Contig2\_Mf\_liverA, NM\_177093, Unigene25226\_Mf\_liverA, Unigene33512\_Mf\_liverA, Unigene14907\_Mf\_liverA, Unigene21561\_Mf\_liverA, Unigene39655\_Mf\_liverA, NM\_010001, Unigene4922\_Mf\_liverA, Unigene25976\_Mf\_liverA, Unigene14637\_Mf\_liverA, Unigene36698\_Mf\_liverA, Unigene30261\_Mf\_liverA, NM\_007622, Unigene13945\_Mf\_liverA, NM\_001104531, Unigene139\_Mf\_liverA, Unigene4944\_Mf\_liverA, Unigene23158\_Mf\_liverA, Unigene37575\_Mf\_liverA, Unigene5774\_Mf\_liverA, Unigene25398\_Mf\_liverA, Unigene27547\_Mf\_liverA, CL1736.Contig2\_Mf\_liverA, Unigene36836\_Mf\_liverA, Unigene33366\_Mf\_liverA, NM\_134156, CL5316.Contig1\_Mf\_liverA, Unigene37389\_Mf\_liverA, Unigene4720\_Mf\_liverA, Unigene45530\_Mf\_liverA, Unigene29063\_Mf\_liverA, Unigene32332\_Mf\_liverA, Unigene4723\_Mf\_liverA, Unigene37470\_Mf\_liverA, CL5254.Contig1\_Mf\_liverA, Unigene34010\_Mf\_liverA, Unigene24613\_Mf\_liverA, Unigene152\_Mf\_liverA, Unigene31852\_Mf\_liverA, CL532.Contig1\_Mf\_liverA, Unigene35169\_Mf\_liverA, Unigene37076\_Mf\_liverA, Unigene1280\_Mf\_liverA, Unigene30288\_Mf\_liverA, CL3166.Contig4\_Mf\_liverA, Unigene14171\_Mf\_liverA, Unigene32436\_Mf\_liverA, CL5576.Contig1\_Mf\_liverA, Unigene33632\_Mf\_liverA, CL5189.Contig1\_Mf\_liverA, CL3339.Contig1\_Mf\_liverA, Unigene2746\_Mf\_liverA, Unigene30528\_Mf\_liverA, NM\_007678, Unigene31251\_Mf\_liverA, Unigene40610\_Mf\_liverA, Unigene4363\_Mf\_liverA, CL4925.Contig1\_Mf\_liverA, CL186.Contig3\_Mf\_liverA, CL425.Contig1\_Mf\_liverA, Unigene32421\_Mf\_liverA, CL2797.Contig2\_Mf\_liverA, CL1803.Contig1\_Mf\_liverA, Unigene14916\_Mf\_liverA, Unigene37243\_Mf\_liverA, Unigene35858\_Mf\_liverA, Unigene30587\_Mf\_liverA, Unigene15077\_Mf\_liverA, CL4600.Contig1\_Mf\_liverA, Unigene36034\_Mf\_liverA, Unigene29231\_Mf\_liverA, Unigene5693\_Mf\_liverA, Unigene22052\_Mf\_liverA, NM\_010003, Unigene34867\_Mf\_liverA, Unigene29876\_Mf\_liverA, CL3800.Contig1\_Mf\_liverA, CL2478.Contig3\_Mf\_liverA, Unigene28687\_Mf\_liverA, CL422.Contig1\_Mf\_liverA, CL1988.Contig3\_Mf\_liverA, NM\_024474, NM\_009609, CL1810.Contig1\_Mf\_liverA, Unigene18499\_Mf\_liverA, CL4033.Contig1\_Mf\_liverA, Unigene24547\_Mf\_liverA, Unigene34983\_Mf\_liverA, Unigene28662\_Mf\_liverA, CL2439.Contig1\_Mf\_liverA, Unigene4781\_Mf\_liverA, NR\_003623, Unigene8033\_Mf\_liverA, Unigene35935\_Mf\_liverA, CL1988.Contig2\_Mf\_liverA, CL3900.Contig1\_Mf\_liverA, Unigene13683\_Mf\_liverA, CL1493.Contig1\_Mf\_liverA, Unigene9698\_Mf\_liverA, Unigene28499\_Mf\_liverA, Unigene36420\_Mf\_liverA, Unigene30585\_Mf\_liverA, Unigene542\_Mf\_liverA, Unigene35476\_Mf\_liverA, Unigene24758\_Mf\_liverA, Unigene4909\_Mf\_liverA, Unigene24471\_Mf\_liverA, Unigene33080\_Mf\_liverA, NM\_010162, Unigene15529\_Mf\_liverA, CL4220.Contig1\_Mf\_liverA, NM\_019879, NR\_033215, CL2251.Contig1\_Mf\_liverA, NM\_007822, CL4490.Contig2\_Mf\_liverA, Unigene25595\_Mf\_liverA, Unigene14286\_Mf\_liverA, Unigene32515\_Mf\_liverA, CL4816.Contig3\_Mf\_liverA, CL840.Contig1\_Mf\_liverA, Unigene39011\_Mf\_liverA, Unigene20432\_Mf\_liverA, NM\_009022, NM\_025336, Unigene35609\_Mf\_liverA, NM\_145218, Unigene4841\_Mf\_liverA, Unigene23185\_Mf\_liverA, Unigene7048\_Mf\_liverA, Unigene13950\_Mf\_liverA, Unigene35046\_Mf\_liverA, Unigene28459\_Mf\_liverA, Unigene37245\_Mf\_liverA, NM\_010158, Unigene13847\_Mf\_liverA, Unigene29985\_Mf\_liverA, CL3104.Contig1\_Mf\_liverA, Unigene36593\_Mf\_liverA, Unigene19658\_Mf\_liverA, Unigene37616\_Mf\_liverA, NM\_027406, Unigene36699\_Mf\_liverA, NM\_146016, Unigene36673\_Mf\_liverA, Unigene496\_Mf\_liverA, NM\_001164598, Unigene4983\_Mf\_liverA, CL5698.Contig1\_Mf\_liverA, Unigene6110\_Mf\_liverA, NM\_001253832, Unigene31392\_Mf\_liverA, NM\_023256, CL591.Contig1\_Mf\_liverA, Unigene4556\_Mf\_liverA, Unigene9466\_Mf\_liverA, Unigene29399\_Mf\_liverA, NM\_020559, Unigene35884\_Mf\_liverA, Unigene33607\_Mf\_liverA, NM\_008292, Unigene28142\_Mf\_liverA, Unigene38015\_Mf\_liverA, CL3835.Contig2\_Mf\_liverA, Unigene31199\_Mf\_liverA, Unigene6959\_Mf\_liverA, Unigene28899\_Mf\_liverA, Unigene46870\_Mf\_liverA, CL1575.Contig1\_Mf\_liverA, Unigene29308\_Mf\_liverA, CL523.Contig1\_Mf\_liverA, Unigene13153\_Mf\_liverA, CL4117.Contig1\_Mf\_liverA, Unigene29008\_Mf\_liverA, CL482.Contig1\_Mf\_liverA, Unigene36762\_Mf\_liverA, CL777.Contig8\_Mf\_liverA, CL3725.Contig1\_Mf\_liverA, NM\_011072, NM\_007820, Unigene4776\_Mf\_liverA, Unigene21466\_Mf\_liverA, Unigene30878\_Mf\_liverA, Unigene40289\_Mf\_liverA, Unigene34810\_Mf\_liverA, CL44.Contig1\_Mf\_liverA, Unigene37880\_Mf\_liverA, Unigene38331\_Mf\_liverA, Unigene37999\_Mf\_liverA, Unigene40020\_Mf\_liverA, Unigene37904\_Mf\_liverA, Unigene41336\_Mf\_liverA, Unigene25070\_Mf\_liverA, CL4141.Contig1\_Mf\_liverA, CL5191.Contig2\_Mf\_liverA, CL2001.Contig1\_Mf\_liverA, Unigene26194\_Mf\_liverA, Unigene13593\_Mf\_liverA, Unigene4686\_Mf\_liverA, Unigene11\_Mf\_liverA, Unigene25596\_Mf\_liverA, Unigene45131\_Mf\_liverA, Unigene5994\_Mf\_liverA, CL1493.Contig2\_Mf\_liverA, Unigene12153\_Mf\_liverA, Unigene25046\_Mf\_liverA, CL5268.Contig1\_Mf\_liverA, Unigene5294\_Mf\_liverA, Unigene5941\_Mf\_liverA, Unigene2745\_Mf\_liverA, Unigene38104\_Mf\_liverA, Unigene38065\_Mf\_liverA, Unigene25462\_Mf\_liverA, Unigene5775\_Mf\_liverA, Unigene36626\_Mf\_liverA, CL787.Contig1\_Mf\_liverA, Unigene39507\_Mf\_liverA, Unigene25524\_Mf\_liverA, NM\_153795, Unigene28822\_Mf\_liverA, Unigene31988\_Mf\_liverA, CL3750.Contig2\_Mf\_liverA, Unigene39141\_Mf\_liverA, Unigene5382\_Mf\_liverA, NM\_201360, Unigene33459\_Mf\_liverA, CL848.Contig2\_Mf\_liverA, Unigene23870\_Mf\_liverA, Unigene5632\_Mf\_liverA, Unigene36669\_Mf\_liverA, CL1263.Contig1\_Mf\_liverA, Unigene37460\_Mf\_liverA, Unigene31517\_Mf\_liverA, NM\_007811, Unigene14582\_Mf\_liverA, CL2142.Contig2\_Mf\_liverA, NM\_011305, Unigene24503\_Mf\_liverA, Unigene27422\_Mf\_liverA, CL3816.Contig1\_Mf\_liverA, CL4701.Contig1\_Mf\_liverA, Unigene25333\_Mf\_liverA, CL993.Contig2\_Mf\_liverA, Unigene36757\_Mf\_liverA, CL5631.Contig1\_Mf\_liverA, CL529.Contig2\_Mf\_liverA, Unigene30584\_Mf\_liverA, CL4220.Contig2\_Mf\_liverA, Unigene17579\_Mf\_liverA, Unigene27081\_Mf\_liverA, NM\_001081372, NM\_010391, Unigene37148\_Mf\_liverA, NM\_010227, Unigene21317\_Mf\_liverA, Unigene30493\_Mf\_liverA, Unigene7195\_Mf\_liverA, Unigene8132\_Mf\_liverA, NM\_019717, Unigene37178\_Mf\_liverA, Unigene27082\_Mf\_liverA, Unigene5712\_Mf\_liverA, Unigene43107\_Mf\_liverA, Unigene1205\_Mf\_liverA, CL442.Contig2\_Mf\_liverA, NM\_033374, CL1352.Contig1\_Mf\_liverA, CL6039.Contig1\_Mf\_liverA, CL2855.Contig1\_Mf\_liverA, CL4411.Contig4\_Mf\_liverA, NM\_001025388, Unigene31206\_Mf\_liverA, NM\_013821, Unigene40796\_Mf\_liverA, Unigene13233\_Mf\_liverA, NM\_011099, NM\_001100182, NM\_010481, NM\_009128, Unigene15318\_Mf\_liverA, CL2240.Contig1\_Mf\_liverA, Unigene36190\_Mf\_liverA, NM\_008293, Unigene7970\_Mf\_liverA, NM\_009255, Unigene15982\_Mf\_liverA, CL1125.Contig1\_Mf\_liverA, CL4162.Contig1\_Mf\_liverA, Unigene35237\_Mf\_liverA, Unigene112\_Mf\_liverA, Unigene12907\_Mf\_liverA, Unigene25292\_Mf\_liverA, Unigene5165\_Mf\_liverA, Unigene15588\_Mf\_liverA, CL5796.Contig2\_Mf\_liverA, NM\_011170, Unigene30707\_Mf\_liverA, Unigene36414\_Mf\_liverA, CL1988.Contig1\_Mf\_liverA, NM\_133838, Unigene5175\_Mf\_liverA, Unigene36328\_Mf\_liverA, Unigene15552\_Mf\_liverA, CL3002.Contig1\_Mf\_liverA, Unigene36514\_Mf\_liverA, CL6039.Contig2\_Mf\_liverA, NM\_145836, CL4816.Contig2\_Mf\_liverA, CL887.Contig2\_Mf\_liverA, Unigene32695\_Mf\_liverA, CL3207.Contig1\_Mf\_liverA, Unigene5906\_Mf\_liverA, Unigene5940\_Mf\_liverA, Unigene43357\_Mf\_liverA, Unigene28731\_Mf\_liverA, Unigene14810\_Mf\_liverA, NM\_033444, Unigene1212\_Mf\_liverA, CL2791.Contig1\_Mf\_liverA, CL4770.Contig1\_Mf\_liverA, Unigene10135\_Mf\_liverA, Unigene32294\_Mf\_liverA, CL3750.Contig1\_Mf\_liverA, CL114.Contig2\_Mf\_liverA, NM\_009883, Unigene18796\_Mf\_liverA, Unigene1327\_Mf\_liverA, CL5307.Contig1\_Mf\_liverA, Unigene3377\_Mf\_liverA, NM\_001081172, CL5978.Contig2\_Mf\_liverA, Unigene8740\_Mf\_liverA, NM\_178405, CL738.Contig2\_Mf\_liverA, CL3816.Contig2\_Mf\_liverA, Unigene14270\_Mf\_liverA, CL4048.Contig1\_Mf\_liverA, CL1052.Contig1\_Mf\_liverA, CL442.Contig5\_Mf\_liverA, NM\_012030, Unigene9406\_Mf\_liverA, Unigene24477\_Mf\_liverA, Unigene120\_Mf\_liverA, Unigene30731\_Mf\_liverA, CL4007.Contig1\_Mf\_liverA, Unigene15026\_Mf\_liverA, Unigene36418\_Mf\_liverA, Unigene14940\_Mf\_liverA, Unigene1221\_Mf\_liverA, CL3835.Contig1\_Mf\_liverA, Unigene39875\_Mf\_liverA, Unigene550\_Mf\_liverA, Unigene31198\_Mf\_liverA, Unigene1292\_Mf\_liverA, CL2355.Contig1\_Mf\_liverA, Unigene37153\_Mf\_liverA, Unigene14809\_Mf\_liverA, Unigene26309\_Mf\_liverA, Unigene32295\_Mf\_liverA, Unigene21562\_Mf\_liverA, Unigene15592\_Mf\_liverA, Unigene5745\_Mf\_liverA, Unigene665\_Mf\_liverA, Unigene2195\_Mf\_liverA, Unigene12889\_Mf\_liverA, NM\_001099634, Unigene8560\_Mf\_liverA, Unigene34866\_Mf\_liverA, Unigene4681\_Mf\_liverA, Unigene16891\_Mf\_liverA, Unigene37819\_Mf\_liverA, Unigene5886\_Mf\_liverA, CL336.Contig3\_Mf\_liverA |
| organelle | Unigene21684\_Mf\_liverA, Unigene5287\_Mf\_liverA, NM\_176843, Unigene34609\_Mf\_liverA, NM\_009898, Unigene25721\_Mf\_liverA, CL3669.Contig2\_Mf\_liverA, NM\_025593, NM\_018815, Unigene28186\_Mf\_liverA, NM\_080638, Unigene15553\_Mf\_liverA, NM\_010378, Unigene34727\_Mf\_liverA, NM\_011082, Unigene29424\_Mf\_liverA, CL4160.Contig2\_Mf\_liverA, CL854.Contig1\_Mf\_liverA, CL4105.Contig1\_Mf\_liverA, Unigene11007\_Mf\_liverA, CL4577.Contig1\_Mf\_liverA, NM\_025613, Unigene39886\_Mf\_liverA, Unigene15064\_Mf\_liverA, NM\_021273, Unigene24252\_Mf\_liverA, CL4757.Contig1\_Mf\_liverA, NR\_004446, CL1555.Contig1\_Mf\_liverA, CL5586.Contig1\_Mf\_liverA, Unigene34341\_Mf\_liverA, Unigene37535\_Mf\_liverA, Unigene30142\_Mf\_liverA, CL695.Contig1\_Mf\_liverA, Unigene35431\_Mf\_liverA, Unigene15703\_Mf\_liverA, Unigene35816\_Mf\_liverA, Unigene31623\_Mf\_liverA, Unigene7897\_Mf\_liverA, NM\_145474, Unigene13379\_Mf\_liverA, NM\_009776, Unigene34394\_Mf\_liverA, NM\_031165, Unigene25594\_Mf\_liverA, NM\_153193, Unigene5138\_Mf\_liverA, Unigene14508\_Mf\_liverA, Unigene14050\_Mf\_liverA, CL4995.Contig1\_Mf\_liverA, Unigene8054\_Mf\_liverA, Unigene18430\_Mf\_liverA, Unigene802\_Mf\_liverA, Unigene5639\_Mf\_liverA, Unigene17048\_Mf\_liverA, CL5807.Contig1\_Mf\_liverA, Unigene13363\_Mf\_liverA, CL2384.Contig1\_Mf\_liverA, Unigene36417\_Mf\_liverA, NM\_021278, CL2855.Contig2\_Mf\_liverA, NM\_177093, Unigene25226\_Mf\_liverA, Unigene33512\_Mf\_liverA, Unigene14907\_Mf\_liverA, Unigene21561\_Mf\_liverA, Unigene39655\_Mf\_liverA, NM\_010001, Unigene4922\_Mf\_liverA, Unigene25976\_Mf\_liverA, Unigene14637\_Mf\_liverA, Unigene36698\_Mf\_liverA, Unigene30261\_Mf\_liverA, NM\_007622, Unigene13945\_Mf\_liverA, NM\_001104531, Unigene139\_Mf\_liverA, Unigene4944\_Mf\_liverA, Unigene23158\_Mf\_liverA, Unigene37575\_Mf\_liverA, Unigene5774\_Mf\_liverA, Unigene25398\_Mf\_liverA, Unigene27547\_Mf\_liverA, CL1736.Contig2\_Mf\_liverA, Unigene36836\_Mf\_liverA, Unigene33366\_Mf\_liverA, NM\_134156, CL5316.Contig1\_Mf\_liverA, Unigene37389\_Mf\_liverA, Unigene4720\_Mf\_liverA, Unigene45530\_Mf\_liverA, Unigene29063\_Mf\_liverA, Unigene32332\_Mf\_liverA, Unigene4723\_Mf\_liverA, Unigene37470\_Mf\_liverA, CL5254.Contig1\_Mf\_liverA, Unigene34010\_Mf\_liverA, Unigene24613\_Mf\_liverA, Unigene152\_Mf\_liverA, Unigene31852\_Mf\_liverA, CL532.Contig1\_Mf\_liverA, Unigene35169\_Mf\_liverA, Unigene37076\_Mf\_liverA, Unigene1280\_Mf\_liverA, Unigene30288\_Mf\_liverA, CL3166.Contig4\_Mf\_liverA, Unigene14171\_Mf\_liverA, Unigene32436\_Mf\_liverA, CL5576.Contig1\_Mf\_liverA, Unigene33632\_Mf\_liverA, CL5189.Contig1\_Mf\_liverA, CL3339.Contig1\_Mf\_liverA, Unigene2746\_Mf\_liverA, Unigene30528\_Mf\_liverA, NM\_007678, Unigene31251\_Mf\_liverA, Unigene40610\_Mf\_liverA, Unigene4363\_Mf\_liverA, CL4925.Contig1\_Mf\_liverA, CL186.Contig3\_Mf\_liverA, CL425.Contig1\_Mf\_liverA, Unigene32421\_Mf\_liverA, CL2797.Contig2\_Mf\_liverA, CL1803.Contig1\_Mf\_liverA, Unigene14916\_Mf\_liverA, Unigene37243\_Mf\_liverA, Unigene35858\_Mf\_liverA, Unigene30587\_Mf\_liverA, Unigene15077\_Mf\_liverA, CL4600.Contig1\_Mf\_liverA, Unigene36034\_Mf\_liverA, Unigene29231\_Mf\_liverA, Unigene5693\_Mf\_liverA, Unigene22052\_Mf\_liverA, NM\_010003, Unigene34867\_Mf\_liverA, Unigene29876\_Mf\_liverA, CL3800.Contig1\_Mf\_liverA, CL2478.Contig3\_Mf\_liverA, Unigene28687\_Mf\_liverA, CL422.Contig1\_Mf\_liverA, CL1988.Contig3\_Mf\_liverA, NM\_024474, NM\_009609, CL1810.Contig1\_Mf\_liverA, Unigene18499\_Mf\_liverA, CL4033.Contig1\_Mf\_liverA, Unigene24547\_Mf\_liverA, Unigene34983\_Mf\_liverA, Unigene28662\_Mf\_liverA, CL2439.Contig1\_Mf\_liverA, Unigene4781\_Mf\_liverA, NR\_003623, Unigene8033\_Mf\_liverA, Unigene35935\_Mf\_liverA, CL1988.Contig2\_Mf\_liverA, CL3900.Contig1\_Mf\_liverA, Unigene13683\_Mf\_liverA, CL1493.Contig1\_Mf\_liverA, Unigene9698\_Mf\_liverA, Unigene28499\_Mf\_liverA, Unigene36420\_Mf\_liverA, Unigene30585\_Mf\_liverA, Unigene542\_Mf\_liverA, Unigene35476\_Mf\_liverA, Unigene24758\_Mf\_liverA, Unigene4909\_Mf\_liverA, Unigene24471\_Mf\_liverA, Unigene33080\_Mf\_liverA, NM\_010162, Unigene15529\_Mf\_liverA, CL4220.Contig1\_Mf\_liverA, NM\_019879, NR\_033215, CL2251.Contig1\_Mf\_liverA, NM\_007822, CL4490.Contig2\_Mf\_liverA, Unigene25595\_Mf\_liverA, Unigene14286\_Mf\_liverA, Unigene32515\_Mf\_liverA, CL4816.Contig3\_Mf\_liverA, CL840.Contig1\_Mf\_liverA, Unigene39011\_Mf\_liverA, Unigene20432\_Mf\_liverA, NM\_009022, NM\_025336, Unigene35609\_Mf\_liverA, NM\_145218, Unigene4841\_Mf\_liverA, Unigene23185\_Mf\_liverA, Unigene7048\_Mf\_liverA, Unigene13950\_Mf\_liverA, Unigene35046\_Mf\_liverA, Unigene28459\_Mf\_liverA, Unigene37245\_Mf\_liverA, NM\_010158, Unigene13847\_Mf\_liverA, Unigene29985\_Mf\_liverA, CL3104.Contig1\_Mf\_liverA, Unigene36593\_Mf\_liverA, Unigene19658\_Mf\_liverA, Unigene37616\_Mf\_liverA, NM\_027406, Unigene36699\_Mf\_liverA, NM\_146016, Unigene36673\_Mf\_liverA, Unigene496\_Mf\_liverA, NM\_001164598, Unigene4983\_Mf\_liverA, CL5698.Contig1\_Mf\_liverA, Unigene6110\_Mf\_liverA, NM\_001253832, Unigene31392\_Mf\_liverA, NM\_023256, CL591.Contig1\_Mf\_liverA, Unigene4556\_Mf\_liverA, Unigene9466\_Mf\_liverA, Unigene29399\_Mf\_liverA, NM\_020559, Unigene35884\_Mf\_liverA, Unigene33607\_Mf\_liverA, NM\_008292, Unigene28142\_Mf\_liverA, Unigene38015\_Mf\_liverA, CL3835.Contig2\_Mf\_liverA, Unigene31199\_Mf\_liverA, Unigene6959\_Mf\_liverA, Unigene28899\_Mf\_liverA, Unigene46870\_Mf\_liverA, CL1575.Contig1\_Mf\_liverA, Unigene29308\_Mf\_liverA, CL523.Contig1\_Mf\_liverA, Unigene13153\_Mf\_liverA, CL4117.Contig1\_Mf\_liverA, Unigene29008\_Mf\_liverA, CL482.Contig1\_Mf\_liverA, Unigene36762\_Mf\_liverA, CL777.Contig8\_Mf\_liverA, CL3725.Contig1\_Mf\_liverA, NM\_011072, NM\_007820, Unigene4776\_Mf\_liverA, Unigene21466\_Mf\_liverA, Unigene30878\_Mf\_liverA, Unigene40289\_Mf\_liverA, Unigene34810\_Mf\_liverA, CL44.Contig1\_Mf\_liverA, Unigene37880\_Mf\_liverA, Unigene38331\_Mf\_liverA, Unigene37999\_Mf\_liverA, Unigene40020\_Mf\_liverA, Unigene37904\_Mf\_liverA, Unigene41336\_Mf\_liverA, Unigene25070\_Mf\_liverA, CL4141.Contig1\_Mf\_liverA, CL5191.Contig2\_Mf\_liverA, CL2001.Contig1\_Mf\_liverA, Unigene26194\_Mf\_liverA, Unigene13593\_Mf\_liverA, Unigene4686\_Mf\_liverA, Unigene11\_Mf\_liverA, Unigene25596\_Mf\_liverA, Unigene45131\_Mf\_liverA, Unigene5994\_Mf\_liverA, CL1493.Contig2\_Mf\_liverA, Unigene12153\_Mf\_liverA, Unigene25046\_Mf\_liverA, CL5268.Contig1\_Mf\_liverA, Unigene5294\_Mf\_liverA, Unigene5941\_Mf\_liverA, Unigene2745\_Mf\_liverA, Unigene38104\_Mf\_liverA, Unigene38065\_Mf\_liverA, Unigene25462\_Mf\_liverA, Unigene5775\_Mf\_liverA, Unigene36626\_Mf\_liverA, CL787.Contig1\_Mf\_liverA, Unigene39507\_Mf\_liverA, Unigene25524\_Mf\_liverA, NM\_153795, Unigene28822\_Mf\_liverA, Unigene31988\_Mf\_liverA, CL3750.Contig2\_Mf\_liverA, Unigene39141\_Mf\_liverA, Unigene5382\_Mf\_liverA, NM\_201360, Unigene33459\_Mf\_liverA, CL848.Contig2\_Mf\_liverA, Unigene23870\_Mf\_liverA, Unigene5632\_Mf\_liverA, Unigene36669\_Mf\_liverA, CL1263.Contig1\_Mf\_liverA, Unigene37460\_Mf\_liverA, Unigene31517\_Mf\_liverA, NM\_007811, Unigene14582\_Mf\_liverA, CL2142.Contig2\_Mf\_liverA, NM\_011305, Unigene24503\_Mf\_liverA, Unigene27422\_Mf\_liverA, CL3816.Contig1\_Mf\_liverA, CL4701.Contig1\_Mf\_liverA, Unigene25333\_Mf\_liverA, CL993.Contig2\_Mf\_liverA, Unigene36757\_Mf\_liverA, CL5631.Contig1\_Mf\_liverA, CL529.Contig2\_Mf\_liverA, Unigene30584\_Mf\_liverA, CL4220.Contig2\_Mf\_liverA, Unigene17579\_Mf\_liverA, Unigene27081\_Mf\_liverA, NM\_001081372, NM\_010391, Unigene37148\_Mf\_liverA, NM\_010227, Unigene21317\_Mf\_liverA, Unigene30493\_Mf\_liverA, Unigene7195\_Mf\_liverA, Unigene8132\_Mf\_liverA, NM\_019717, Unigene37178\_Mf\_liverA, Unigene27082\_Mf\_liverA, Unigene5712\_Mf\_liverA, Unigene43107\_Mf\_liverA, Unigene1205\_Mf\_liverA, CL442.Contig2\_Mf\_liverA, NM\_033374, CL1352.Contig1\_Mf\_liverA, CL6039.Contig1\_Mf\_liverA, CL2855.Contig1\_Mf\_liverA, CL4411.Contig4\_Mf\_liverA, NM\_001025388, Unigene31206\_Mf\_liverA, NM\_013821, Unigene40796\_Mf\_liverA, Unigene13233\_Mf\_liverA, NM\_011099, NM\_001100182, NM\_010481, NM\_009128, Unigene15318\_Mf\_liverA, CL2240.Contig1\_Mf\_liverA, Unigene36190\_Mf\_liverA, NM\_008293, Unigene7970\_Mf\_liverA, NM\_009255, Unigene15982\_Mf\_liverA, CL1125.Contig1\_Mf\_liverA, CL4162.Contig1\_Mf\_liverA, Unigene35237\_Mf\_liverA, Unigene112\_Mf\_liverA, Unigene12907\_Mf\_liverA, Unigene25292\_Mf\_liverA, Unigene5165\_Mf\_liverA, Unigene15588\_Mf\_liverA, CL5796.Contig2\_Mf\_liverA, NM\_011170, Unigene30707\_Mf\_liverA, Unigene36414\_Mf\_liverA, CL1988.Contig1\_Mf\_liverA, NM\_133838, Unigene5175\_Mf\_liverA, Unigene36328\_Mf\_liverA, Unigene15552\_Mf\_liverA, CL3002.Contig1\_Mf\_liverA, Unigene36514\_Mf\_liverA, CL6039.Contig2\_Mf\_liverA, NM\_145836, CL4816.Contig2\_Mf\_liverA, CL887.Contig2\_Mf\_liverA, Unigene32695\_Mf\_liverA, CL3207.Contig1\_Mf\_liverA, Unigene5906\_Mf\_liverA, NM\_001081148, Unigene5940\_Mf\_liverA, Unigene43357\_Mf\_liverA, Unigene28731\_Mf\_liverA, Unigene14810\_Mf\_liverA, NM\_033444, Unigene1212\_Mf\_liverA, CL2791.Contig1\_Mf\_liverA, CL4770.Contig1\_Mf\_liverA, Unigene10135\_Mf\_liverA, Unigene32294\_Mf\_liverA, CL3750.Contig1\_Mf\_liverA, CL114.Contig2\_Mf\_liverA, NM\_009883, Unigene18796\_Mf\_liverA, Unigene1327\_Mf\_liverA, CL5307.Contig1\_Mf\_liverA, Unigene3377\_Mf\_liverA, NM\_001081172, CL5978.Contig2\_Mf\_liverA, Unigene8740\_Mf\_liverA, NM\_178405, CL738.Contig2\_Mf\_liverA, CL3816.Contig2\_Mf\_liverA, Unigene14270\_Mf\_liverA, CL4048.Contig1\_Mf\_liverA, CL1052.Contig1\_Mf\_liverA, CL442.Contig5\_Mf\_liverA, NM\_012030, Unigene9406\_Mf\_liverA, Unigene24477\_Mf\_liverA, Unigene120\_Mf\_liverA, Unigene30731\_Mf\_liverA, CL4007.Contig1\_Mf\_liverA, Unigene15026\_Mf\_liverA, Unigene36418\_Mf\_liverA, Unigene14940\_Mf\_liverA, Unigene1221\_Mf\_liverA, CL3835.Contig1\_Mf\_liverA, Unigene39875\_Mf\_liverA, Unigene550\_Mf\_liverA, Unigene31198\_Mf\_liverA, Unigene1292\_Mf\_liverA, CL2355.Contig1\_Mf\_liverA, Unigene37153\_Mf\_liverA, Unigene14809\_Mf\_liverA, Unigene26309\_Mf\_liverA, Unigene32295\_Mf\_liverA, Unigene21562\_Mf\_liverA, Unigene15592\_Mf\_liverA, Unigene5745\_Mf\_liverA, Unigene665\_Mf\_liverA, Unigene2195\_Mf\_liverA, Unigene12889\_Mf\_liverA, NM\_001099634, Unigene8560\_Mf\_liverA, Unigene34866\_Mf\_liverA, Unigene4681\_Mf\_liverA, Unigene16891\_Mf\_liverA, Unigene37819\_Mf\_liverA, Unigene5886\_Mf\_liverA, CL336.Contig3\_Mf\_liverA |
| condensed chromosome | CL5268.Contig1\_Mf\_liverA, Unigene45530\_Mf\_liverA |
| ribosome | Unigene37460\_Mf\_liverA, Unigene31198\_Mf\_liverA, Unigene31199\_Mf\_liverA |
| chromatin | Unigene14907\_Mf\_liverA, Unigene31517\_Mf\_liverA, NM\_007622, CL777.Contig8\_Mf\_liverA, NM\_009883, NM\_011305, CL4757.Contig1\_Mf\_liverA |
| nucleus | Unigene9466\_Mf\_liverA, Unigene21684\_Mf\_liverA, Unigene29399\_Mf\_liverA, NM\_176843, Unigene35884\_Mf\_liverA, Unigene33607\_Mf\_liverA, Unigene38015\_Mf\_liverA, Unigene28142\_Mf\_liverA, CL3835.Contig2\_Mf\_liverA, NM\_025593, Unigene28899\_Mf\_liverA, NM\_018815, Unigene28186\_Mf\_liverA, Unigene46870\_Mf\_liverA, CL1575.Contig1\_Mf\_liverA, NM\_080638, Unigene15553\_Mf\_liverA, Unigene29424\_Mf\_liverA, CL4160.Contig2\_Mf\_liverA, Unigene29008\_Mf\_liverA, CL4105.Contig1\_Mf\_liverA, Unigene11007\_Mf\_liverA, CL482.Contig1\_Mf\_liverA, CL4577.Contig1\_Mf\_liverA, CL777.Contig8\_Mf\_liverA, Unigene39886\_Mf\_liverA, Unigene15064\_Mf\_liverA, Unigene40289\_Mf\_liverA, CL4757.Contig1\_Mf\_liverA, Unigene37904\_Mf\_liverA, Unigene37535\_Mf\_liverA, Unigene30142\_Mf\_liverA, Unigene25070\_Mf\_liverA, CL4141.Contig1\_Mf\_liverA, Unigene35431\_Mf\_liverA, Unigene15703\_Mf\_liverA, Unigene35816\_Mf\_liverA, Unigene26194\_Mf\_liverA, Unigene7897\_Mf\_liverA, Unigene5138\_Mf\_liverA, Unigene45131\_Mf\_liverA, Unigene5994\_Mf\_liverA, Unigene14050\_Mf\_liverA, CL5268.Contig1\_Mf\_liverA, CL4995.Contig1\_Mf\_liverA, Unigene2745\_Mf\_liverA, Unigene38065\_Mf\_liverA, Unigene802\_Mf\_liverA, Unigene39507\_Mf\_liverA, CL2384.Contig1\_Mf\_liverA, Unigene36417\_Mf\_liverA, NM\_177093, Unigene39141\_Mf\_liverA, Unigene33512\_Mf\_liverA, Unigene14907\_Mf\_liverA, Unigene23870\_Mf\_liverA, Unigene25976\_Mf\_liverA, CL1263.Contig1\_Mf\_liverA, Unigene31517\_Mf\_liverA, Unigene14582\_Mf\_liverA, NM\_007622, NM\_011305, Unigene13945\_Mf\_liverA, Unigene27422\_Mf\_liverA, Unigene4944\_Mf\_liverA, Unigene25333\_Mf\_liverA, CL5631.Contig1\_Mf\_liverA, Unigene23158\_Mf\_liverA, Unigene37575\_Mf\_liverA, Unigene17579\_Mf\_liverA, Unigene33366\_Mf\_liverA, NM\_134156, Unigene4720\_Mf\_liverA, Unigene37148\_Mf\_liverA, Unigene45530\_Mf\_liverA, Unigene29063\_Mf\_liverA, Unigene8132\_Mf\_liverA, CL5254.Contig1\_Mf\_liverA, Unigene152\_Mf\_liverA, Unigene24613\_Mf\_liverA, Unigene37178\_Mf\_liverA, Unigene37076\_Mf\_liverA, Unigene43107\_Mf\_liverA, Unigene30288\_Mf\_liverA, Unigene32436\_Mf\_liverA, CL5189.Contig1\_Mf\_liverA, CL3339.Contig1\_Mf\_liverA, Unigene2746\_Mf\_liverA, Unigene31206\_Mf\_liverA, NM\_007678, Unigene40610\_Mf\_liverA, CL186.Contig3\_Mf\_liverA, Unigene15318\_Mf\_liverA, CL1803.Contig1\_Mf\_liverA, Unigene14916\_Mf\_liverA, CL4162.Contig1\_Mf\_liverA, Unigene35858\_Mf\_liverA, Unigene35237\_Mf\_liverA, Unigene36034\_Mf\_liverA, Unigene12907\_Mf\_liverA, Unigene29231\_Mf\_liverA, Unigene5693\_Mf\_liverA, Unigene34867\_Mf\_liverA, Unigene15588\_Mf\_liverA, Unigene5165\_Mf\_liverA, CL3800.Contig1\_Mf\_liverA, Unigene29876\_Mf\_liverA, Unigene30707\_Mf\_liverA, CL2478.Contig3\_Mf\_liverA, Unigene36414\_Mf\_liverA, Unigene5175\_Mf\_liverA, Unigene28687\_Mf\_liverA, CL3002.Contig1\_Mf\_liverA, Unigene15552\_Mf\_liverA, CL1810.Contig1\_Mf\_liverA, NM\_009609, Unigene18499\_Mf\_liverA, Unigene34983\_Mf\_liverA, CL2439.Contig1\_Mf\_liverA, Unigene35935\_Mf\_liverA, CL3900.Contig1\_Mf\_liverA, Unigene13683\_Mf\_liverA, NM\_033444, Unigene1212\_Mf\_liverA, CL2791.Contig1\_Mf\_liverA, Unigene9698\_Mf\_liverA, CL4770.Contig1\_Mf\_liverA, Unigene36420\_Mf\_liverA, Unigene24758\_Mf\_liverA, Unigene35476\_Mf\_liverA, Unigene32294\_Mf\_liverA, Unigene24471\_Mf\_liverA, Unigene33080\_Mf\_liverA, NM\_009883, Unigene18796\_Mf\_liverA, Unigene14270\_Mf\_liverA, CL840.Contig1\_Mf\_liverA, Unigene9406\_Mf\_liverA, Unigene24477\_Mf\_liverA, Unigene39011\_Mf\_liverA, Unigene30731\_Mf\_liverA, Unigene15026\_Mf\_liverA, Unigene14940\_Mf\_liverA, Unigene36418\_Mf\_liverA, Unigene4841\_Mf\_liverA, CL3835.Contig1\_Mf\_liverA, Unigene13950\_Mf\_liverA, Unigene39875\_Mf\_liverA, NM\_010158, Unigene13847\_Mf\_liverA, CL2355.Contig1\_Mf\_liverA, Unigene37153\_Mf\_liverA, Unigene29985\_Mf\_liverA, Unigene26309\_Mf\_liverA, Unigene32295\_Mf\_liverA, Unigene19658\_Mf\_liverA, Unigene665\_Mf\_liverA, Unigene12889\_Mf\_liverA, Unigene36673\_Mf\_liverA, NM\_001099634, Unigene8560\_Mf\_liverA, Unigene34866\_Mf\_liverA, Unigene4983\_Mf\_liverA, Unigene31392\_Mf\_liverA, Unigene16891\_Mf\_liverA |
| nucleolus | Unigene29876\_Mf\_liverA, Unigene24758\_Mf\_liverA, Unigene36414\_Mf\_liverA, Unigene36417\_Mf\_liverA, CL1810.Contig1\_Mf\_liverA, Unigene35431\_Mf\_liverA, Unigene28899\_Mf\_liverA, CL1803.Contig1\_Mf\_liverA, Unigene29424\_Mf\_liverA, CL4160.Contig2\_Mf\_liverA, Unigene152\_Mf\_liverA, Unigene24613\_Mf\_liverA, Unigene14916\_Mf\_liverA, Unigene35237\_Mf\_liverA, Unigene31392\_Mf\_liverA, Unigene13683\_Mf\_liverA, Unigene32436\_Mf\_liverA, CL2791.Contig1\_Mf\_liverA, Unigene30731\_Mf\_liverA, Unigene9698\_Mf\_liverA, Unigene36420\_Mf\_liverA, Unigene36418\_Mf\_liverA, Unigene802\_Mf\_liverA, Unigene37575\_Mf\_liverA, CL4757.Contig1\_Mf\_liverA |
| centrosome | CL1810.Contig1\_Mf\_liverA, Unigene15318\_Mf\_liverA, Unigene2746\_Mf\_liverA, Unigene2745\_Mf\_liverA, Unigene21317\_Mf\_liverA |
| nucleoplasm | NM\_176843, Unigene35884\_Mf\_liverA, Unigene24471\_Mf\_liverA, Unigene38015\_Mf\_liverA, NM\_177093, NM\_025593, Unigene33512\_Mf\_liverA, Unigene14907\_Mf\_liverA, CL4160.Contig2\_Mf\_liverA, CL4105.Contig1\_Mf\_liverA, Unigene25976\_Mf\_liverA, Unigene14270\_Mf\_liverA, CL4162.Contig1\_Mf\_liverA, Unigene31517\_Mf\_liverA, Unigene15064\_Mf\_liverA, Unigene4841\_Mf\_liverA, CL4757.Contig1\_Mf\_liverA, Unigene30707\_Mf\_liverA, Unigene33366\_Mf\_liverA, CL2355.Contig1\_Mf\_liverA, Unigene37153\_Mf\_liverA, NM\_009609, Unigene35431\_Mf\_liverA, CL4141.Contig1\_Mf\_liverA, Unigene35816\_Mf\_liverA, Unigene12889\_Mf\_liverA, Unigene152\_Mf\_liverA, Unigene35935\_Mf\_liverA, Unigene30288\_Mf\_liverA, Unigene31392\_Mf\_liverA, Unigene43107\_Mf\_liverA, Unigene32436\_Mf\_liverA, NM\_007678 |
| spindle | Unigene33366\_Mf\_liverA, CL3207.Contig1\_Mf\_liverA |
| microtubule organizing center | Unigene25070\_Mf\_liverA, Unigene496\_Mf\_liverA, CL1810.Contig1\_Mf\_liverA, Unigene15318\_Mf\_liverA, Unigene2746\_Mf\_liverA, Unigene2745\_Mf\_liverA, NM\_012030, Unigene21317\_Mf\_liverA |
| nuclear body | CL2355.Contig1\_Mf\_liverA, Unigene35816\_Mf\_liverA, CL4160.Contig2\_Mf\_liverA, CL4757.Contig1\_Mf\_liverA |
| non-membrane-bounded organelle | Unigene34609\_Mf\_liverA, NM\_009898, Unigene25721\_Mf\_liverA, Unigene31199\_Mf\_liverA, Unigene15318\_Mf\_liverA, Unigene28899\_Mf\_liverA, CL425.Contig1\_Mf\_liverA, Unigene29308\_Mf\_liverA, CL1803.Contig1\_Mf\_liverA, Unigene29424\_Mf\_liverA, CL4160.Contig2\_Mf\_liverA, Unigene29008\_Mf\_liverA, Unigene11007\_Mf\_liverA, Unigene14916\_Mf\_liverA, CL4162.Contig1\_Mf\_liverA, Unigene35237\_Mf\_liverA, CL777.Contig8\_Mf\_liverA, CL3725.Contig1\_Mf\_liverA, NM\_011072, Unigene5693\_Mf\_liverA, Unigene5165\_Mf\_liverA, Unigene24252\_Mf\_liverA, CL4757.Contig1\_Mf\_liverA, Unigene30707\_Mf\_liverA, Unigene29876\_Mf\_liverA, Unigene36414\_Mf\_liverA, CL422.Contig1\_Mf\_liverA, Unigene40020\_Mf\_liverA, Unigene25070\_Mf\_liverA, NM\_009609, CL1810.Contig1\_Mf\_liverA, Unigene35431\_Mf\_liverA, CL5191.Contig2\_Mf\_liverA, Unigene31623\_Mf\_liverA, Unigene13593\_Mf\_liverA, Unigene5138\_Mf\_liverA, CL3207.Contig1\_Mf\_liverA, Unigene5940\_Mf\_liverA, CL3900.Contig1\_Mf\_liverA, Unigene13683\_Mf\_liverA, Unigene25046\_Mf\_liverA, CL2791.Contig1\_Mf\_liverA, Unigene9698\_Mf\_liverA, CL5268.Contig1\_Mf\_liverA, Unigene28499\_Mf\_liverA, Unigene36420\_Mf\_liverA, Unigene5941\_Mf\_liverA, Unigene2745\_Mf\_liverA, Unigene802\_Mf\_liverA, Unigene24758\_Mf\_liverA, Unigene5639\_Mf\_liverA, NM\_153795, NM\_009883, Unigene28822\_Mf\_liverA, Unigene36417\_Mf\_liverA, NM\_021278, NM\_177093, Unigene14907\_Mf\_liverA, Unigene1327\_Mf\_liverA, CL848.Contig2\_Mf\_liverA, Unigene23870\_Mf\_liverA, NM\_001081172, CL2251.Contig1\_Mf\_liverA, Unigene14637\_Mf\_liverA, Unigene37460\_Mf\_liverA, CL1052.Contig1\_Mf\_liverA, Unigene31517\_Mf\_liverA, NM\_007622, NM\_011305, Unigene32515\_Mf\_liverA, NM\_012030, Unigene24477\_Mf\_liverA, Unigene20432\_Mf\_liverA, CL4701.Contig1\_Mf\_liverA, Unigene25333\_Mf\_liverA, Unigene30731\_Mf\_liverA, Unigene36418\_Mf\_liverA, Unigene37575\_Mf\_liverA, Unigene27081\_Mf\_liverA, Unigene550\_Mf\_liverA, Unigene27547\_Mf\_liverA, Unigene31198\_Mf\_liverA, Unigene33366\_Mf\_liverA, NM\_134156, Unigene45530\_Mf\_liverA, NM\_010227, Unigene21317\_Mf\_liverA, Unigene26309\_Mf\_liverA, Unigene37470\_Mf\_liverA, Unigene8132\_Mf\_liverA, Unigene2195\_Mf\_liverA, NM\_146016, Unigene36673\_Mf\_liverA, Unigene496\_Mf\_liverA, Unigene152\_Mf\_liverA, Unigene24613\_Mf\_liverA, Unigene27082\_Mf\_liverA, Unigene31852\_Mf\_liverA, Unigene35169\_Mf\_liverA, Unigene37076\_Mf\_liverA, CL5698.Contig1\_Mf\_liverA, Unigene31392\_Mf\_liverA, Unigene32436\_Mf\_liverA, NM\_033374, CL1352.Contig1\_Mf\_liverA, CL5576.Contig1\_Mf\_liverA, CL591.Contig1\_Mf\_liverA, Unigene4556\_Mf\_liverA, Unigene2746\_Mf\_liverA, NM\_001025388 |
| intracellular non-membrane-bounded organelle | Unigene34609\_Mf\_liverA, NM\_009898, Unigene25721\_Mf\_liverA, Unigene31199\_Mf\_liverA, Unigene15318\_Mf\_liverA, Unigene28899\_Mf\_liverA, CL425.Contig1\_Mf\_liverA, Unigene29308\_Mf\_liverA, CL1803.Contig1\_Mf\_liverA, Unigene29424\_Mf\_liverA, CL4160.Contig2\_Mf\_liverA, Unigene29008\_Mf\_liverA, Unigene11007\_Mf\_liverA, Unigene14916\_Mf\_liverA, CL4162.Contig1\_Mf\_liverA, Unigene35237\_Mf\_liverA, CL777.Contig8\_Mf\_liverA, CL3725.Contig1\_Mf\_liverA, NM\_011072, Unigene5693\_Mf\_liverA, Unigene5165\_Mf\_liverA, Unigene24252\_Mf\_liverA, CL4757.Contig1\_Mf\_liverA, Unigene30707\_Mf\_liverA, Unigene29876\_Mf\_liverA, Unigene36414\_Mf\_liverA, CL422.Contig1\_Mf\_liverA, Unigene40020\_Mf\_liverA, Unigene25070\_Mf\_liverA, NM\_009609, CL1810.Contig1\_Mf\_liverA, Unigene35431\_Mf\_liverA, CL5191.Contig2\_Mf\_liverA, Unigene31623\_Mf\_liverA, Unigene13593\_Mf\_liverA, Unigene5138\_Mf\_liverA, CL3207.Contig1\_Mf\_liverA, Unigene5940\_Mf\_liverA, CL3900.Contig1\_Mf\_liverA, Unigene13683\_Mf\_liverA, Unigene25046\_Mf\_liverA, CL2791.Contig1\_Mf\_liverA, Unigene9698\_Mf\_liverA, CL5268.Contig1\_Mf\_liverA, Unigene28499\_Mf\_liverA, Unigene36420\_Mf\_liverA, Unigene5941\_Mf\_liverA, Unigene2745\_Mf\_liverA, Unigene802\_Mf\_liverA, Unigene24758\_Mf\_liverA, Unigene5639\_Mf\_liverA, NM\_153795, NM\_009883, Unigene28822\_Mf\_liverA, Unigene36417\_Mf\_liverA, NM\_021278, NM\_177093, Unigene14907\_Mf\_liverA, Unigene1327\_Mf\_liverA, CL848.Contig2\_Mf\_liverA, Unigene23870\_Mf\_liverA, NM\_001081172, CL2251.Contig1\_Mf\_liverA, Unigene14637\_Mf\_liverA, Unigene37460\_Mf\_liverA, CL1052.Contig1\_Mf\_liverA, Unigene31517\_Mf\_liverA, NM\_007622, NM\_011305, Unigene32515\_Mf\_liverA, NM\_012030, Unigene24477\_Mf\_liverA, Unigene20432\_Mf\_liverA, CL4701.Contig1\_Mf\_liverA, Unigene25333\_Mf\_liverA, Unigene30731\_Mf\_liverA, Unigene36418\_Mf\_liverA, Unigene37575\_Mf\_liverA, Unigene27081\_Mf\_liverA, Unigene550\_Mf\_liverA, Unigene27547\_Mf\_liverA, Unigene31198\_Mf\_liverA, Unigene33366\_Mf\_liverA, NM\_134156, Unigene45530\_Mf\_liverA, NM\_010227, Unigene21317\_Mf\_liverA, Unigene26309\_Mf\_liverA, Unigene37470\_Mf\_liverA, Unigene8132\_Mf\_liverA, Unigene2195\_Mf\_liverA, NM\_146016, Unigene36673\_Mf\_liverA, Unigene496\_Mf\_liverA, Unigene152\_Mf\_liverA, Unigene24613\_Mf\_liverA, Unigene27082\_Mf\_liverA, Unigene31852\_Mf\_liverA, Unigene35169\_Mf\_liverA, Unigene37076\_Mf\_liverA, CL5698.Contig1\_Mf\_liverA, Unigene31392\_Mf\_liverA, Unigene32436\_Mf\_liverA, NM\_033374, CL1352.Contig1\_Mf\_liverA, CL5576.Contig1\_Mf\_liverA, CL591.Contig1\_Mf\_liverA, Unigene4556\_Mf\_liverA, Unigene2746\_Mf\_liverA, NM\_001025388 |
| nucleoplasm part | NM\_176843, Unigene33366\_Mf\_liverA, CL2355.Contig1\_Mf\_liverA, NM\_177093, NM\_025593, NM\_009609, Unigene14907\_Mf\_liverA, Unigene35816\_Mf\_liverA, CL4160.Contig2\_Mf\_liverA, Unigene35935\_Mf\_liverA, Unigene31517\_Mf\_liverA, Unigene30288\_Mf\_liverA, Unigene43107\_Mf\_liverA, Unigene32436\_Mf\_liverA, NM\_007678, CL4757.Contig1\_Mf\_liverA |
| chromosomal part | Unigene31517\_Mf\_liverA, NM\_007622, CL777.Contig8\_Mf\_liverA, NM\_009883, NM\_011305, Unigene31392\_Mf\_liverA, Unigene45530\_Mf\_liverA, Unigene14907\_Mf\_liverA, CL5268.Contig1\_Mf\_liverA, CL4757.Contig1\_Mf\_liverA |
| membrane-enclosed lumen | Unigene29399\_Mf\_liverA, NM\_176843, Unigene35884\_Mf\_liverA, NM\_010481, Unigene38015\_Mf\_liverA, NM\_025593, Unigene28899\_Mf\_liverA, CL2797.Contig2\_Mf\_liverA, Unigene34727\_Mf\_liverA, CL1803.Contig1\_Mf\_liverA, Unigene29424\_Mf\_liverA, CL4160.Contig2\_Mf\_liverA, CL4105.Contig1\_Mf\_liverA, Unigene14916\_Mf\_liverA, CL1125.Contig1\_Mf\_liverA, CL4162.Contig1\_Mf\_liverA, Unigene35237\_Mf\_liverA, Unigene21466\_Mf\_liverA, Unigene22052\_Mf\_liverA, Unigene15064\_Mf\_liverA, Unigene5165\_Mf\_liverA, CL5796.Contig2\_Mf\_liverA, CL4757.Contig1\_Mf\_liverA, Unigene30707\_Mf\_liverA, Unigene29876\_Mf\_liverA, Unigene36414\_Mf\_liverA, NM\_009609, CL1810.Contig1\_Mf\_liverA, CL4141.Contig1\_Mf\_liverA, Unigene35431\_Mf\_liverA, Unigene28662\_Mf\_liverA, Unigene35816\_Mf\_liverA, Unigene35935\_Mf\_liverA, Unigene25596\_Mf\_liverA, CL3900.Contig1\_Mf\_liverA, Unigene13683\_Mf\_liverA, CL2791.Contig1\_Mf\_liverA, Unigene9698\_Mf\_liverA, CL5268.Contig1\_Mf\_liverA, Unigene36420\_Mf\_liverA, Unigene802\_Mf\_liverA, Unigene24758\_Mf\_liverA, Unigene35476\_Mf\_liverA, Unigene24471\_Mf\_liverA, NM\_009883, Unigene36417\_Mf\_liverA, CL2855.Contig2\_Mf\_liverA, Unigene15529\_Mf\_liverA, NM\_177093, Unigene14907\_Mf\_liverA, Unigene33512\_Mf\_liverA, Unigene5382\_Mf\_liverA, Unigene3377\_Mf\_liverA, NM\_019879, CL2251.Contig1\_Mf\_liverA, Unigene25976\_Mf\_liverA, Unigene14270\_Mf\_liverA, CL738.Contig2\_Mf\_liverA, Unigene36669\_Mf\_liverA, CL4490.Contig2\_Mf\_liverA, Unigene37460\_Mf\_liverA, Unigene31517\_Mf\_liverA, Unigene25595\_Mf\_liverA, NM\_007622, NM\_011305, Unigene30731\_Mf\_liverA, Unigene36418\_Mf\_liverA, Unigene4841\_Mf\_liverA, Unigene37575\_Mf\_liverA, Unigene23185\_Mf\_liverA, NM\_010158, Unigene33366\_Mf\_liverA, CL2355.Contig1\_Mf\_liverA, NM\_134156, Unigene37153\_Mf\_liverA, Unigene45530\_Mf\_liverA, Unigene14809\_Mf\_liverA, Unigene30493\_Mf\_liverA, Unigene15592\_Mf\_liverA, Unigene12889\_Mf\_liverA, Unigene36673\_Mf\_liverA, Unigene152\_Mf\_liverA, Unigene24613\_Mf\_liverA, CL532.Contig1\_Mf\_liverA, Unigene37076\_Mf\_liverA, Unigene6110\_Mf\_liverA, Unigene43107\_Mf\_liverA, Unigene31392\_Mf\_liverA, Unigene30288\_Mf\_liverA, Unigene14171\_Mf\_liverA, Unigene32436\_Mf\_liverA, CL2855.Contig1\_Mf\_liverA, NM\_007678 |
| microtubule cytoskeleton | Unigene496\_Mf\_liverA, Unigene11007\_Mf\_liverA, CL1052.Contig1\_Mf\_liverA, Unigene27547\_Mf\_liverA, Unigene33366\_Mf\_liverA, CL3207.Contig1\_Mf\_liverA, NM\_007622, NM\_012030, Unigene21317\_Mf\_liverA, Unigene25070\_Mf\_liverA, CL1810.Contig1\_Mf\_liverA, Unigene15318\_Mf\_liverA, CL425.Contig1\_Mf\_liverA, Unigene2746\_Mf\_liverA, Unigene23870\_Mf\_liverA, Unigene2745\_Mf\_liverA, NM\_146016 |
| ribonucleoprotein complex | Unigene37460\_Mf\_liverA, Unigene31198\_Mf\_liverA, Unigene31199\_Mf\_liverA, Unigene13683\_Mf\_liverA, CL1810.Contig1\_Mf\_liverA, Unigene35431\_Mf\_liverA, Unigene23870\_Mf\_liverA, Unigene35816\_Mf\_liverA |
| chromosome | Unigene31517\_Mf\_liverA, NM\_007622, CL777.Contig8\_Mf\_liverA, CL3900.Contig1\_Mf\_liverA, NM\_009883, NM\_011305, Unigene31392\_Mf\_liverA, Unigene45530\_Mf\_liverA, Unigene14907\_Mf\_liverA, CL5268.Contig1\_Mf\_liverA, CL4757.Contig1\_Mf\_liverA |
| organelle lumen | Unigene29399\_Mf\_liverA, NM\_176843, Unigene35884\_Mf\_liverA, NM\_010481, Unigene38015\_Mf\_liverA, NM\_025593, Unigene28899\_Mf\_liverA, Unigene34727\_Mf\_liverA, CL1803.Contig1\_Mf\_liverA, Unigene29424\_Mf\_liverA, CL4160.Contig2\_Mf\_liverA, CL4105.Contig1\_Mf\_liverA, Unigene14916\_Mf\_liverA, CL1125.Contig1\_Mf\_liverA, CL4162.Contig1\_Mf\_liverA, Unigene35237\_Mf\_liverA, Unigene21466\_Mf\_liverA, Unigene22052\_Mf\_liverA, Unigene15064\_Mf\_liverA, Unigene5165\_Mf\_liverA, CL4757.Contig1\_Mf\_liverA, Unigene30707\_Mf\_liverA, Unigene29876\_Mf\_liverA, Unigene36414\_Mf\_liverA, NM\_009609, CL1810.Contig1\_Mf\_liverA, CL4141.Contig1\_Mf\_liverA, Unigene35431\_Mf\_liverA, Unigene28662\_Mf\_liverA, Unigene35816\_Mf\_liverA, Unigene35935\_Mf\_liverA, Unigene25596\_Mf\_liverA, CL3900.Contig1\_Mf\_liverA, Unigene13683\_Mf\_liverA, CL2791.Contig1\_Mf\_liverA, Unigene9698\_Mf\_liverA, CL5268.Contig1\_Mf\_liverA, Unigene36420\_Mf\_liverA, Unigene802\_Mf\_liverA, Unigene24758\_Mf\_liverA, Unigene24471\_Mf\_liverA, Unigene36417\_Mf\_liverA, NM\_009883, CL2855.Contig2\_Mf\_liverA, Unigene15529\_Mf\_liverA, NM\_177093, Unigene14907\_Mf\_liverA, Unigene33512\_Mf\_liverA, Unigene5382\_Mf\_liverA, NM\_019879, CL2251.Contig1\_Mf\_liverA, Unigene25976\_Mf\_liverA, Unigene14270\_Mf\_liverA, Unigene36669\_Mf\_liverA, Unigene37460\_Mf\_liverA, Unigene31517\_Mf\_liverA, Unigene25595\_Mf\_liverA, NM\_007622, NM\_011305, Unigene30731\_Mf\_liverA, Unigene36418\_Mf\_liverA, Unigene4841\_Mf\_liverA, Unigene37575\_Mf\_liverA, NM\_010158, Unigene33366\_Mf\_liverA, CL2355.Contig1\_Mf\_liverA, NM\_134156, Unigene45530\_Mf\_liverA, Unigene37153\_Mf\_liverA, Unigene14809\_Mf\_liverA, Unigene30493\_Mf\_liverA, Unigene15592\_Mf\_liverA, Unigene12889\_Mf\_liverA, Unigene36673\_Mf\_liverA, Unigene152\_Mf\_liverA, Unigene24613\_Mf\_liverA, CL532.Contig1\_Mf\_liverA, Unigene37076\_Mf\_liverA, Unigene6110\_Mf\_liverA, Unigene43107\_Mf\_liverA, Unigene31392\_Mf\_liverA, Unigene30288\_Mf\_liverA, Unigene14171\_Mf\_liverA, Unigene32436\_Mf\_liverA, CL2855.Contig1\_Mf\_liverA, NM\_007678 |
| intracellular organelle lumen | Unigene29399\_Mf\_liverA, NM\_176843, Unigene35884\_Mf\_liverA, NM\_010481, Unigene38015\_Mf\_liverA, NM\_025593, Unigene28899\_Mf\_liverA, Unigene34727\_Mf\_liverA, CL1803.Contig1\_Mf\_liverA, Unigene29424\_Mf\_liverA, CL4160.Contig2\_Mf\_liverA, CL4105.Contig1\_Mf\_liverA, Unigene14916\_Mf\_liverA, CL1125.Contig1\_Mf\_liverA, CL4162.Contig1\_Mf\_liverA, Unigene35237\_Mf\_liverA, Unigene21466\_Mf\_liverA, Unigene22052\_Mf\_liverA, Unigene15064\_Mf\_liverA, Unigene5165\_Mf\_liverA, CL4757.Contig1\_Mf\_liverA, Unigene30707\_Mf\_liverA, Unigene29876\_Mf\_liverA, Unigene36414\_Mf\_liverA, NM\_009609, CL1810.Contig1\_Mf\_liverA, CL4141.Contig1\_Mf\_liverA, Unigene35431\_Mf\_liverA, Unigene35816\_Mf\_liverA, Unigene35935\_Mf\_liverA, Unigene25596\_Mf\_liverA, CL3900.Contig1\_Mf\_liverA, Unigene13683\_Mf\_liverA, CL2791.Contig1\_Mf\_liverA, Unigene9698\_Mf\_liverA, CL5268.Contig1\_Mf\_liverA, Unigene36420\_Mf\_liverA, Unigene802\_Mf\_liverA, Unigene24758\_Mf\_liverA, Unigene24471\_Mf\_liverA, Unigene36417\_Mf\_liverA, NM\_009883, CL2855.Contig2\_Mf\_liverA, NM\_177093, Unigene14907\_Mf\_liverA, Unigene33512\_Mf\_liverA, Unigene5382\_Mf\_liverA, NM\_019879, Unigene25976\_Mf\_liverA, Unigene14270\_Mf\_liverA, Unigene36669\_Mf\_liverA, Unigene37460\_Mf\_liverA, Unigene31517\_Mf\_liverA, Unigene25595\_Mf\_liverA, NM\_007622, NM\_011305, Unigene30731\_Mf\_liverA, Unigene36418\_Mf\_liverA, Unigene4841\_Mf\_liverA, Unigene37575\_Mf\_liverA, NM\_010158, Unigene33366\_Mf\_liverA, CL2355.Contig1\_Mf\_liverA, NM\_134156, Unigene45530\_Mf\_liverA, Unigene37153\_Mf\_liverA, Unigene30493\_Mf\_liverA, Unigene15592\_Mf\_liverA, Unigene12889\_Mf\_liverA, Unigene152\_Mf\_liverA, Unigene24613\_Mf\_liverA, CL532.Contig1\_Mf\_liverA, Unigene37076\_Mf\_liverA, Unigene6110\_Mf\_liverA, Unigene43107\_Mf\_liverA, Unigene31392\_Mf\_liverA, Unigene30288\_Mf\_liverA, Unigene14171\_Mf\_liverA, Unigene32436\_Mf\_liverA, CL2855.Contig1\_Mf\_liverA, NM\_007678 |
| nuclear part | NM\_176843, Unigene35884\_Mf\_liverA, Unigene38015\_Mf\_liverA, Unigene28142\_Mf\_liverA, NM\_025593, Unigene28899\_Mf\_liverA, NM\_018815, CL1575.Contig1\_Mf\_liverA, NM\_080638, CL1803.Contig1\_Mf\_liverA, Unigene29424\_Mf\_liverA, CL4160.Contig2\_Mf\_liverA, CL4105.Contig1\_Mf\_liverA, Unigene14916\_Mf\_liverA, CL4162.Contig1\_Mf\_liverA, Unigene35237\_Mf\_liverA, Unigene15064\_Mf\_liverA, Unigene5165\_Mf\_liverA, CL4757.Contig1\_Mf\_liverA, Unigene30707\_Mf\_liverA, Unigene29876\_Mf\_liverA, CL2478.Contig3\_Mf\_liverA, Unigene36414\_Mf\_liverA, Unigene37535\_Mf\_liverA, NM\_009609, CL1810.Contig1\_Mf\_liverA, CL4141.Contig1\_Mf\_liverA, Unigene35431\_Mf\_liverA, Unigene15703\_Mf\_liverA, Unigene35816\_Mf\_liverA, Unigene35935\_Mf\_liverA, CL3900.Contig1\_Mf\_liverA, Unigene13683\_Mf\_liverA, NM\_033444, CL2791.Contig1\_Mf\_liverA, Unigene9698\_Mf\_liverA, CL5268.Contig1\_Mf\_liverA, Unigene36420\_Mf\_liverA, Unigene802\_Mf\_liverA, Unigene24758\_Mf\_liverA, Unigene32294\_Mf\_liverA, Unigene24471\_Mf\_liverA, Unigene36417\_Mf\_liverA, NM\_009883, NM\_177093, Unigene14907\_Mf\_liverA, Unigene33512\_Mf\_liverA, Unigene25976\_Mf\_liverA, Unigene14270\_Mf\_liverA, CL1263.Contig1\_Mf\_liverA, Unigene31517\_Mf\_liverA, NM\_007622, NM\_011305, Unigene30731\_Mf\_liverA, Unigene36418\_Mf\_liverA, Unigene4841\_Mf\_liverA, Unigene37575\_Mf\_liverA, NM\_010158, Unigene33366\_Mf\_liverA, CL2355.Contig1\_Mf\_liverA, NM\_134156, Unigene45530\_Mf\_liverA, Unigene37153\_Mf\_liverA, Unigene32295\_Mf\_liverA, Unigene8132\_Mf\_liverA, CL5254.Contig1\_Mf\_liverA, Unigene12889\_Mf\_liverA, Unigene152\_Mf\_liverA, Unigene24613\_Mf\_liverA, NM\_001099634, Unigene37076\_Mf\_liverA, Unigene43107\_Mf\_liverA, Unigene31392\_Mf\_liverA, Unigene30288\_Mf\_liverA, Unigene32436\_Mf\_liverA, Unigene16891\_Mf\_liverA, NM\_007678 |
| nuclear lumen | Unigene24758\_Mf\_liverA, NM\_176843, Unigene35884\_Mf\_liverA, Unigene24471\_Mf\_liverA, Unigene38015\_Mf\_liverA, NM\_009883, Unigene36417\_Mf\_liverA, NM\_177093, NM\_025593, Unigene28899\_Mf\_liverA, Unigene33512\_Mf\_liverA, Unigene14907\_Mf\_liverA, CL1803.Contig1\_Mf\_liverA, Unigene29424\_Mf\_liverA, CL4160.Contig2\_Mf\_liverA, CL4105.Contig1\_Mf\_liverA, Unigene25976\_Mf\_liverA, Unigene14270\_Mf\_liverA, Unigene14916\_Mf\_liverA, CL4162.Contig1\_Mf\_liverA, Unigene31517\_Mf\_liverA, Unigene35237\_Mf\_liverA, NM\_007622, NM\_011305, Unigene30731\_Mf\_liverA, Unigene15064\_Mf\_liverA, Unigene36418\_Mf\_liverA, Unigene4841\_Mf\_liverA, Unigene5165\_Mf\_liverA, Unigene37575\_Mf\_liverA, CL4757.Contig1\_Mf\_liverA, Unigene30707\_Mf\_liverA, Unigene29876\_Mf\_liverA, NM\_010158, Unigene36414\_Mf\_liverA, Unigene33366\_Mf\_liverA, CL2355.Contig1\_Mf\_liverA, NM\_134156, Unigene37153\_Mf\_liverA, Unigene45530\_Mf\_liverA, NM\_009609, CL1810.Contig1\_Mf\_liverA, CL4141.Contig1\_Mf\_liverA, Unigene35431\_Mf\_liverA, Unigene35816\_Mf\_liverA, Unigene12889\_Mf\_liverA, Unigene35935\_Mf\_liverA, Unigene24613\_Mf\_liverA, Unigene152\_Mf\_liverA, Unigene37076\_Mf\_liverA, CL3900.Contig1\_Mf\_liverA, Unigene43107\_Mf\_liverA, Unigene31392\_Mf\_liverA, Unigene30288\_Mf\_liverA, Unigene13683\_Mf\_liverA, Unigene32436\_Mf\_liverA, CL2791.Contig1\_Mf\_liverA, Unigene9698\_Mf\_liverA, CL5268.Contig1\_Mf\_liverA, Unigene36420\_Mf\_liverA, NM\_007678, Unigene802\_Mf\_liverA |
|
