# Supplementary material for: De novo assembly and transcriptome characterization: novel insights into the natural resistance mechanisms of Microtus fortis against Schistosoma japonicum
Source: BMC Genomics. 2014 Jun 2;15(1):417. doi: 10.1186/1471-2164-15-417 (PMC4073500; doi:10.1186/1471-2164-15-417)
Supplement: Supplementary file 5 — Additional file 5: Dataset S3: GO terms for MfA-VS-MfA2W_C. (ZIP 64 KB) [file 12864_2013_6159_MOESM5_ESM.zip › 1990354100108772_add5.html]

Terms for MfA-VS-MfA2W\_C


## Terms for MfA-VS-MfA2W\_C

---


### Result Table

|  |
| --- |
| **Terms from the Component Ontology with p-value as good or better than 1** |

| Gene Ontology term | Cluster frequency | Genome frequency of use | Corrected P-value | Expression Profile |
| --- | --- | --- | --- | --- |
| extracellular space | 117 out of 946 genes, 12.4% | 1015 out of 37603 genes, 2.7% | 4.99e-41 | View Result |
| extracellular region | 207 out of 946 genes, 21.9% | 2987 out of 37603 genes, 7.9% | 2.70e-39 | View Result |
| cell periphery | 313 out of 946 genes, 33.1% | 6038 out of 37603 genes, 16.1% | 1.44e-36 | View Result |
| plasma membrane | 306 out of 946 genes, 32.3% | 5905 out of 37603 genes, 15.7% | 1.74e-35 | View Result |
| extracellular region part | 165 out of 946 genes, 17.4% | 2343 out of 37603 genes, 6.2% | 3.74e-31 | View Result |
| external side of plasma membrane | 52 out of 946 genes, 5.5% | 286 out of 37603 genes, 0.8% | 1.26e-26 | View Result |
| cytoplasm | 595 out of 946 genes, 62.9% | 17507 out of 37603 genes, 46.6% | 4.62e-22 | View Result |
| cell surface | 88 out of 946 genes, 9.3% | 1006 out of 37603 genes, 2.7% | 1.35e-21 | View Result |
| cytosol | 168 out of 946 genes, 17.8% | 2945 out of 37603 genes, 7.8% | 2.45e-21 | View Result |
| proteinaceous extracellular matrix | 60 out of 946 genes, 6.3% | 554 out of 37603 genes, 1.5% | 1.04e-18 | View Result |
| fibrillar collagen | 14 out of 946 genes, 1.5% | 16 out of 37603 genes, 0.0% | 1.69e-18 | View Result |
| extracellular matrix | 73 out of 946 genes, 7.7% | 854 out of 37603 genes, 2.3% | 4.70e-17 | View Result |
| collagen | 27 out of 946 genes, 2.9% | 112 out of 37603 genes, 0.3% | 1.54e-16 | View Result |
| collagen type I | 11 out of 946 genes, 1.2% | 11 out of 37603 genes, 0.0% | 9.65e-16 | View Result |
| lysosome | 53 out of 946 genes, 5.6% | 560 out of 37603 genes, 1.5% | 8.04e-14 | View Result |
| extracellular matrix part | 40 out of 946 genes, 4.2% | 335 out of 37603 genes, 0.9% | 1.80e-13 | View Result |
| plasma membrane part | 182 out of 946 genes, 19.2% | 4083 out of 37603 genes, 10.9% | 3.83e-12 | View Result |
| cytoplasmic part | 480 out of 946 genes, 50.7% | 14592 out of 37603 genes, 38.8% | 1.28e-11 | View Result |
| MHC protein complex | 18 out of 946 genes, 1.9% | 71 out of 37603 genes, 0.2% | 4.93e-11 | View Result |
| lytic vacuole | 55 out of 946 genes, 5.8% | 750 out of 37603 genes, 2.0% | 8.79e-10 | View Result |
| intrinsic to plasma membrane | 83 out of 946 genes, 8.8% | 1463 out of 37603 genes, 3.9% | 2.42e-09 | View Result |
| endoplasmic reticulum | 104 out of 946 genes, 11.0% | 2107 out of 37603 genes, 5.6% | 1.83e-08 | View Result |
| immunological synapse | 10 out of 946 genes, 1.1% | 22 out of 37603 genes, 0.1% | 1.90e-08 | View Result |
| integral to plasma membrane | 78 out of 946 genes, 8.2% | 1399 out of 37603 genes, 3.7% | 2.49e-08 | View Result |
| perinuclear region of cytoplasm | 50 out of 946 genes, 5.3% | 703 out of 37603 genes, 1.9% | 2.64e-08 | View Result |
| vacuole | 57 out of 946 genes, 6.0% | 875 out of 37603 genes, 2.3% | 3.66e-08 | View Result |
| membrane raft | 31 out of 946 genes, 3.3% | 335 out of 37603 genes, 0.9% | 2.51e-07 | View Result |
| lysosomal lumen | 14 out of 946 genes, 1.5% | 65 out of 37603 genes, 0.2% | 2.74e-07 | View Result |
| vacuolar lumen | 14 out of 946 genes, 1.5% | 66 out of 37603 genes, 0.2% | 3.40e-07 | View Result |
| MHC class II protein complex | 8 out of 946 genes, 0.8% | 15 out of 37603 genes, 0.0% | 3.42e-07 | View Result |
| vacuolar part | 29 out of 946 genes, 3.1% | 304 out of 37603 genes, 0.8% | 4.43e-07 | View Result |
| actin filament | 22 out of 946 genes, 2.3% | 183 out of 37603 genes, 0.5% | 6.36e-07 | View Result |
| integral to membrane | 230 out of 946 genes, 24.3% | 6371 out of 37603 genes, 16.9% | 1.32e-06 | View Result |
| symbiont-containing vacuole | 9 out of 946 genes, 1.0% | 27 out of 37603 genes, 0.1% | 4.85e-06 | View Result |
| actin cytoskeleton | 50 out of 946 genes, 5.3% | 830 out of 37603 genes, 2.2% | 6.79e-06 | View Result |
| endoplasmic reticulum lumen | 20 out of 946 genes, 2.1% | 174 out of 37603 genes, 0.5% | 7.54e-06 | View Result |
| host cell cytoplasm | 9 out of 946 genes, 1.0% | 30 out of 37603 genes, 0.1% | 1.38e-05 | View Result |
| host cell cytoplasm part | 9 out of 946 genes, 1.0% | 30 out of 37603 genes, 0.1% | 1.38e-05 | View Result |
| host intracellular part | 9 out of 946 genes, 1.0% | 32 out of 37603 genes, 0.1% | 2.59e-05 | View Result |
| intracellular region of host | 9 out of 946 genes, 1.0% | 32 out of 37603 genes, 0.1% | 2.59e-05 | View Result |
| integrin complex | 11 out of 946 genes, 1.2% | 53 out of 37603 genes, 0.1% | 2.80e-05 | View Result |
| host | 9 out of 946 genes, 1.0% | 34 out of 37603 genes, 0.1% | 4.63e-05 | View Result |
| host cell part | 9 out of 946 genes, 1.0% | 34 out of 37603 genes, 0.1% | 4.63e-05 | View Result |
| extraorganismal space | 9 out of 946 genes, 1.0% | 34 out of 37603 genes, 0.1% | 4.63e-05 | View Result |
| host cell | 9 out of 946 genes, 1.0% | 34 out of 37603 genes, 0.1% | 4.63e-05 | View Result |
| other organism | 9 out of 946 genes, 1.0% | 34 out of 37603 genes, 0.1% | 4.63e-05 | View Result |
| other organism cell | 9 out of 946 genes, 1.0% | 34 out of 37603 genes, 0.1% | 4.63e-05 | View Result |
| other organism part | 9 out of 946 genes, 1.0% | 34 out of 37603 genes, 0.1% | 4.63e-05 | View Result |
| extracellular organelle | 10 out of 946 genes, 1.1% | 45 out of 37603 genes, 0.1% | 5.56e-05 | View Result |
| extracellular membrane-bounded organelle | 10 out of 946 genes, 1.1% | 45 out of 37603 genes, 0.1% | 5.56e-05 | View Result |
| platelet alpha granule | 16 out of 946 genes, 1.7% | 135 out of 37603 genes, 0.4% | 0.00012 | View Result |
| symbiont-containing vacuole membrane | 7 out of 946 genes, 0.7% | 20 out of 37603 genes, 0.1% | 0.00014 | View Result |
| apical plasma membrane | 27 out of 946 genes, 2.9% | 360 out of 37603 genes, 1.0% | 0.00023 | View Result |
| uropod | 6 out of 946 genes, 0.6% | 14 out of 37603 genes, 0.0% | 0.00025 | View Result |
| endoplasmic reticulum part | 66 out of 946 genes, 7.0% | 1424 out of 37603 genes, 3.8% | 0.00069 | View Result |
| cortical cytoskeleton | 13 out of 946 genes, 1.4% | 103 out of 37603 genes, 0.3% | 0.00079 | View Result |
| multivesicular body | 8 out of 946 genes, 0.8% | 35 out of 37603 genes, 0.1% | 0.00080 | View Result |
| secretory granule | 36 out of 946 genes, 3.8% | 610 out of 37603 genes, 1.6% | 0.00108 | View Result |
| phagocytic vesicle | 10 out of 946 genes, 1.1% | 67 out of 37603 genes, 0.2% | 0.00262 | View Result |
| lysosomal membrane | 15 out of 946 genes, 1.6% | 151 out of 37603 genes, 0.4% | 0.00275 | View Result |
| trailing edge | 6 out of 946 genes, 0.6% | 21 out of 37603 genes, 0.1% | 0.00391 | View Result |
| cell cortex | 24 out of 946 genes, 2.5% | 358 out of 37603 genes, 1.0% | 0.00641 | View Result |
| apical part of cell | 29 out of 946 genes, 3.1% | 488 out of 37603 genes, 1.3% | 0.00867 | View Result |
| receptor complex | 21 out of 946 genes, 2.2% | 299 out of 37603 genes, 0.8% | 0.01081 | View Result |
| late endosome | 21 out of 946 genes, 2.2% | 301 out of 37603 genes, 0.8% | 0.01192 | View Result |
| anchored to membrane | 14 out of 946 genes, 1.5% | 151 out of 37603 genes, 0.4% | 0.01207 | View Result |
| cell cortex part | 15 out of 946 genes, 1.6% | 171 out of 37603 genes, 0.5% | 0.01222 | View Result |
| vacuolar membrane | 18 out of 946 genes, 1.9% | 247 out of 37603 genes, 0.7% | 0.02470 | View Result |
| phagocytic vesicle membrane | 7 out of 946 genes, 0.7% | 42 out of 37603 genes, 0.1% | 0.03122 | View Result |
| membrane | 487 out of 946 genes, 51.5% | 17065 out of 37603 genes, 45.4% | 0.03215 | View Result |
| platelet alpha granule lumen | 9 out of 946 genes, 1.0% | 74 out of 37603 genes, 0.2% | 0.03977 | View Result |
| lamellipodium | 15 out of 946 genes, 1.6% | 191 out of 37603 genes, 0.5% | 0.04329 | View Result |
| secretory granule lumen | 9 out of 946 genes, 1.0% | 75 out of 37603 genes, 0.2% | 0.04420 | View Result |
| MHC class I protein complex | 5 out of 946 genes, 0.5% | 20 out of 37603 genes, 0.1% | 0.04514 | View Result |
| cytoplasmic membrane-bounded vesicle lumen | 9 out of 946 genes, 1.0% | 77 out of 37603 genes, 0.2% | 0.05429 | View Result |
| endocytic vesicle membrane | 11 out of 946 genes, 1.2% | 113 out of 37603 genes, 0.3% | 0.05441 | View Result |
| cytoplasmic vesicle part | 32 out of 946 genes, 3.4% | 629 out of 37603 genes, 1.7% | 0.06421 | View Result |
| endocytic vesicle | 17 out of 946 genes, 1.8% | 252 out of 37603 genes, 0.7% | 0.09845 | View Result |
| intermediate filament | 12 out of 946 genes, 1.3% | 144 out of 37603 genes, 0.4% | 0.11959 | View Result |
| NADPH oxidase complex | 3 out of 946 genes, 0.3% | 6 out of 37603 genes, 0.0% | 0.11996 | View Result |
| dendrite | 32 out of 946 genes, 3.4% | 655 out of 37603 genes, 1.7% | 0.13094 | View Result |
| MCM complex | 5 out of 946 genes, 0.5% | 25 out of 37603 genes, 0.1% | 0.13939 | View Result |
| vesicle lumen | 9 out of 946 genes, 1.0% | 88 out of 37603 genes, 0.2% | 0.15049 | View Result |
| Golgi lumen | 7 out of 946 genes, 0.7% | 54 out of 37603 genes, 0.1% | 0.15783 | View Result |
| extrinsic to membrane | 17 out of 946 genes, 1.8% | 264 out of 37603 genes, 0.7% | 0.16866 | View Result |
| unconventional myosin complex | 4 out of 946 genes, 0.4% | 15 out of 37603 genes, 0.0% | 0.17412 | View Result |
| anchored to external side of plasma membrane | 4 out of 946 genes, 0.4% | 15 out of 37603 genes, 0.0% | 0.17412 | View Result |
| mitochondrion | 100 out of 946 genes, 10.6% | 2848 out of 37603 genes, 7.6% | 0.18737 | View Result |
| cytoplasmic membrane-bounded vesicle | 72 out of 946 genes, 7.6% | 1912 out of 37603 genes, 5.1% | 0.18805 | View Result |
| cytoskeletal part | 93 out of 946 genes, 9.8% | 2623 out of 37603 genes, 7.0% | 0.21813 | View Result |
| endosomal part | 21 out of 946 genes, 2.2% | 375 out of 37603 genes, 1.0% | 0.24597 | View Result |
| Bcl3/NF-kappaB2 complex | 2 out of 946 genes, 0.2% | 2 out of 37603 genes, 0.0% | 0.25290 | View Result |
| macrophage migration inhibitory factor receptor complex | 2 out of 946 genes, 0.2% | 2 out of 37603 genes, 0.0% | 0.25290 | View Result |
| NOS2-CD74 complex | 2 out of 946 genes, 0.2% | 2 out of 37603 genes, 0.0% | 0.25290 | View Result |
| actomyosin, actin part | 2 out of 946 genes, 0.2% | 2 out of 37603 genes, 0.0% | 0.25290 | View Result |
| protein complex | 208 out of 946 genes, 22.0% | 6728 out of 37603 genes, 17.9% | 0.26714 | View Result |
| high-density lipoprotein particle | 6 out of 946 genes, 0.6% | 43 out of 37603 genes, 0.1% | 0.27491 | View Result |
| endosome | 46 out of 946 genes, 4.9% | 1107 out of 37603 genes, 2.9% | 0.28413 | View Result |
| proteasome core complex | 5 out of 946 genes, 0.5% | 29 out of 37603 genes, 0.1% | 0.28670 | View Result |
| cytoplasmic vesicle | 81 out of 946 genes, 8.6% | 2245 out of 37603 genes, 6.0% | 0.30228 | View Result |
| basement membrane | 14 out of 946 genes, 1.5% | 206 out of 37603 genes, 0.5% | 0.31264 | View Result |
| cell-cell adherens junction | 11 out of 946 genes, 1.2% | 140 out of 37603 genes, 0.4% | 0.34471 | View Result |
| proteasome complex | 9 out of 946 genes, 1.0% | 100 out of 37603 genes, 0.3% | 0.38381 | View Result |
| late endosome membrane | 9 out of 946 genes, 1.0% | 100 out of 37603 genes, 0.3% | 0.38381 | View Result |
| myosin complex | 9 out of 946 genes, 1.0% | 101 out of 37603 genes, 0.3% | 0.41212 | View Result |
| membrane-bounded vesicle | 74 out of 946 genes, 7.8% | 2048 out of 37603 genes, 5.4% | 0.48793 | View Result |
| anchored to plasma membrane | 6 out of 946 genes, 0.6% | 48 out of 37603 genes, 0.1% | 0.49757 | View Result |
| endoplasmic reticulum membrane | 46 out of 946 genes, 4.9% | 1142 out of 37603 genes, 3.0% | 0.53247 | View Result |
| zymogen granule | 4 out of 946 genes, 0.4% | 20 out of 37603 genes, 0.1% | 0.55918 | View Result |
| cytoskeleton | 131 out of 946 genes, 13.8% | 4034 out of 37603 genes, 10.7% | 0.56274 | View Result |
| ruffle | 17 out of 946 genes, 1.8% | 296 out of 37603 genes, 0.8% | 0.59648 | View Result |
| adherens junction | 25 out of 946 genes, 2.6% | 518 out of 37603 genes, 1.4% | 0.66162 | View Result |
| intrinsic to external side of plasma membrane | 4 out of 946 genes, 0.4% | 21 out of 37603 genes, 0.1% | 0.67710 | View Result |
| complement component C1 complex | 2 out of 946 genes, 0.2% | 3 out of 37603 genes, 0.0% | 0.74600 | View Result |
| actomyosin contractile ring | 2 out of 946 genes, 0.2% | 3 out of 37603 genes, 0.0% | 0.74600 | View Result |
| proteasome activator complex | 2 out of 946 genes, 0.2% | 3 out of 37603 genes, 0.0% | 0.74600 | View Result |
| vesicle | 87 out of 946 genes, 9.2% | 2526 out of 37603 genes, 6.7% | 0.76290 | View Result |
| filamentous actin | 5 out of 946 genes, 0.5% | 37 out of 37603 genes, 0.1% | 0.89165 | View Result |
| nuclear outer membrane-endoplasmic reticulum membrane network | 46 out of 946 genes, 4.9% | 1173 out of 37603 genes, 3.1% | 0.89493 | View Result |
| Weibel-Palade body | 3 out of 946 genes, 0.3% | 11 out of 37603 genes, 0.0% | 0.90058 | View Result |
| pre-autophagosomal structure membrane | 3 out of 946 genes, 0.3% | 11 out of 37603 genes, 0.0% | 0.90058 | View Result |
| endosome membrane | 19 out of 946 genes, 2.0% | 366 out of 37603 genes, 1.0% | 1 | View Result |
| cortical actin cytoskeleton | 7 out of 946 genes, 0.7% | 75 out of 37603 genes, 0.2% | 1 | View Result |
| sarcolemma | 12 out of 946 genes, 1.3% | 193 out of 37603 genes, 0.5% | 1 | View Result |
| zymogen granule membrane | 3 out of 946 genes, 0.3% | 13 out of 37603 genes, 0.0% | 1 | View Result |
| actomyosin | 8 out of 946 genes, 0.8% | 100 out of 37603 genes, 0.3% | 1 | View Result |
| neuronal cell body | 23 out of 946 genes, 2.4% | 494 out of 37603 genes, 1.3% | 1 | View Result |
| endomembrane system | 112 out of 946 genes, 11.8% | 3480 out of 37603 genes, 9.3% | 1 | View Result |
| stress fiber | 7 out of 946 genes, 0.7% | 81 out of 37603 genes, 0.2% | 1 | View Result |
| contractile fiber | 21 out of 946 genes, 2.2% | 441 out of 37603 genes, 1.2% | 1 | View Result |
| cell leading edge | 32 out of 946 genes, 3.4% | 771 out of 37603 genes, 2.1% | 1 | View Result |
| intermediate filament cytoskeleton | 12 out of 946 genes, 1.3% | 197 out of 37603 genes, 0.5% | 1 | View Result |
| I-kappaB/NF-kappaB complex | 3 out of 946 genes, 0.3% | 14 out of 37603 genes, 0.0% | 1 | View Result |
| microvillus membrane | 4 out of 946 genes, 0.4% | 29 out of 37603 genes, 0.1% | 1 | View Result |
| secretory granule membrane | 10 out of 946 genes, 1.1% | 154 out of 37603 genes, 0.4% | 1 | View Result |
| varicosity | 2 out of 946 genes, 0.2% | 5 out of 37603 genes, 0.0% | 1 | View Result |
| focal adhesion | 12 out of 946 genes, 1.3% | 207 out of 37603 genes, 0.6% | 1 | View Result |
| anchoring junction | 25 out of 946 genes, 2.6% | 581 out of 37603 genes, 1.5% | 1 | View Result |
| neuron projection | 58 out of 946 genes, 6.1% | 1654 out of 37603 genes, 4.4% | 1 | View Result |
| filopodium | 8 out of 946 genes, 0.8% | 112 out of 37603 genes, 0.3% | 1 | View Result |
| ruffle membrane | 8 out of 946 genes, 0.8% | 112 out of 37603 genes, 0.3% | 1 | View Result |
| lateral plasma membrane | 6 out of 946 genes, 0.6% | 69 out of 37603 genes, 0.2% | 1 | View Result |
| growth cone | 10 out of 946 genes, 1.1% | 162 out of 37603 genes, 0.4% | 1 | View Result |
| contractile ring | 2 out of 946 genes, 0.2% | 6 out of 37603 genes, 0.0% | 1 | View Result |
| actin filament bundle | 10 out of 946 genes, 1.1% | 165 out of 37603 genes, 0.4% | 1 | View Result |
| anaphase-promoting complex | 4 out of 946 genes, 0.4% | 34 out of 37603 genes, 0.1% | 1 | View Result |
| myosin II complex | 4 out of 946 genes, 0.4% | 34 out of 37603 genes, 0.1% | 1 | View Result |
| cytoplasmic microtubule | 5 out of 946 genes, 0.5% | 53 out of 37603 genes, 0.1% | 1 | View Result |
| cell projection | 96 out of 946 genes, 10.1% | 3024 out of 37603 genes, 8.0% | 1 | View Result |
| Golgi apparatus | 64 out of 946 genes, 6.8% | 1902 out of 37603 genes, 5.1% | 1 | View Result |
| ULK1-ATG13-FIP200 complex | 2 out of 946 genes, 0.2% | 7 out of 37603 genes, 0.0% | 1 | View Result |
| costamere | 4 out of 946 genes, 0.4% | 36 out of 37603 genes, 0.1% | 1 | View Result |
| midbody | 8 out of 946 genes, 0.8% | 123 out of 37603 genes, 0.3% | 1 | View Result |
| cytoplasmic vesicle membrane | 23 out of 946 genes, 2.4% | 549 out of 37603 genes, 1.5% | 1 | View Result |
| microtubule | 21 out of 946 genes, 2.2% | 499 out of 37603 genes, 1.3% | 1 | View Result |
| microvillus | 9 out of 946 genes, 1.0% | 154 out of 37603 genes, 0.4% | 1 | View Result |
| nuclear matrix | 8 out of 946 genes, 0.8% | 132 out of 37603 genes, 0.4% | 1 | View Result |
| proteasome accessory complex | 3 out of 946 genes, 0.3% | 23 out of 37603 genes, 0.1% | 1 | View Result |
| chloride channel complex | 3 out of 946 genes, 0.3% | 23 out of 37603 genes, 0.1% | 1 | View Result |
| organelle membrane | 144 out of 946 genes, 15.2% | 4856 out of 37603 genes, 12.9% | 1 | View Result |
| type III intermediate filament | 2 out of 946 genes, 0.2% | 9 out of 37603 genes, 0.0% | 1 | View Result |
| cell part | 831 out of 946 genes, 87.8% | 32173 out of 37603 genes, 85.6% | 1 | View Result |
| cell | 831 out of 946 genes, 87.8% | 32174 out of 37603 genes, 85.6% | 1 | View Result |
| macromolecular complex | 240 out of 946 genes, 25.4% | 8498 out of 37603 genes, 22.6% | 1 | View Result |
| female germ cell nucleus | 2 out of 946 genes, 0.2% | 10 out of 37603 genes, 0.0% | 1 | View Result |
| muscle myosin complex | 2 out of 946 genes, 0.2% | 10 out of 37603 genes, 0.0% | 1 | View Result |
| H4/H2A histone acetyltransferase complex | 4 out of 946 genes, 0.4% | 45 out of 37603 genes, 0.1% | 1 | View Result |
| integral to mitochondrial membrane | 3 out of 946 genes, 0.3% | 26 out of 37603 genes, 0.1% | 1 | View Result |
| recycling endosome | 6 out of 946 genes, 0.6% | 91 out of 37603 genes, 0.2% | 1 | View Result |
| cyclin-dependent protein kinase holoenzyme complex | 3 out of 946 genes, 0.3% | 27 out of 37603 genes, 0.1% | 1 | View Result |
| male pronucleus | 2 out of 946 genes, 0.2% | 11 out of 37603 genes, 0.0% | 1 | View Result |
| axon | 21 out of 946 genes, 2.2% | 539 out of 37603 genes, 1.4% | 1 | View Result |
| nuclear envelope | 24 out of 946 genes, 2.5% | 638 out of 37603 genes, 1.7% | 1 | View Result |
| endosome lumen | 2 out of 946 genes, 0.2% | 13 out of 37603 genes, 0.0% | 1 | View Result |
| Z disc | 6 out of 946 genes, 0.6% | 101 out of 37603 genes, 0.3% | 1 | View Result |
| leading edge membrane | 10 out of 946 genes, 1.1% | 212 out of 37603 genes, 0.6% | 1 | View Result |
| myofibril | 16 out of 946 genes, 1.7% | 395 out of 37603 genes, 1.1% | 1 | View Result |
| melanosome | 6 out of 946 genes, 0.6% | 102 out of 37603 genes, 0.3% | 1 | View Result |
| extrinsic to plasma membrane | 8 out of 946 genes, 0.8% | 156 out of 37603 genes, 0.4% | 1 | View Result |
| microfibril | 2 out of 946 genes, 0.2% | 14 out of 37603 genes, 0.0% | 1 | View Result |
| collagen type IV | 2 out of 946 genes, 0.2% | 14 out of 37603 genes, 0.0% | 1 | View Result |
| intercellular canaliculus | 2 out of 946 genes, 0.2% | 14 out of 37603 genes, 0.0% | 1 | View Result |
| neuromuscular junction | 4 out of 946 genes, 0.4% | 55 out of 37603 genes, 0.1% | 1 | View Result |
| aggresome | 3 out of 946 genes, 0.3% | 33 out of 37603 genes, 0.1% | 1 | View Result |
| dystrophin-associated glycoprotein complex | 3 out of 946 genes, 0.3% | 34 out of 37603 genes, 0.1% | 1 | View Result |
| autophagic vacuole membrane | 2 out of 946 genes, 0.2% | 15 out of 37603 genes, 0.0% | 1 | View Result |
| female pronucleus | 2 out of 946 genes, 0.2% | 15 out of 37603 genes, 0.0% | 1 | View Result |
| keratin filament | 2 out of 946 genes, 0.2% | 15 out of 37603 genes, 0.0% | 1 | View Result |
| vesicle membrane | 24 out of 946 genes, 2.5% | 672 out of 37603 genes, 1.8% | 1 | View Result |
| Ada2/Gcn5/Ada3 transcription activator complex | 3 out of 946 genes, 0.3% | 35 out of 37603 genes, 0.1% | 1 | View Result |
| filopodium membrane | 2 out of 946 genes, 0.2% | 16 out of 37603 genes, 0.0% | 1 | View Result |
| spherical high-density lipoprotein particle | 2 out of 946 genes, 0.2% | 16 out of 37603 genes, 0.0% | 1 | View Result |
| T cell receptor complex | 2 out of 946 genes, 0.2% | 16 out of 37603 genes, 0.0% | 1 | View Result |
| pre-autophagosomal structure | 3 out of 946 genes, 0.3% | 36 out of 37603 genes, 0.1% | 1 | View Result |
| protein-lipid complex | 6 out of 946 genes, 0.6% | 112 out of 37603 genes, 0.3% | 1 | View Result |
| plasma lipoprotein particle | 6 out of 946 genes, 0.6% | 112 out of 37603 genes, 0.3% | 1 | View Result |
| recycling endosome membrane | 3 out of 946 genes, 0.3% | 38 out of 37603 genes, 0.1% | 1 | View Result |
| intracellular organelle part | 374 out of 946 genes, 39.5% | 13998 out of 37603 genes, 37.2% | 1 | View Result |
| sheet-forming collagen | 2 out of 946 genes, 0.2% | 18 out of 37603 genes, 0.0% | 1 | View Result |
| membrane part | 353 out of 946 genes, 37.3% | 13186 out of 37603 genes, 35.1% | 1 | View Result |
| I band | 9 out of 946 genes, 1.0% | 208 out of 37603 genes, 0.6% | 1 | View Result |
| nuclear lamina | 2 out of 946 genes, 0.2% | 19 out of 37603 genes, 0.1% | 1 | View Result |
| cell-cell junction | 25 out of 946 genes, 2.6% | 742 out of 37603 genes, 2.0% | 1 | View Result |
| caveola | 6 out of 946 genes, 0.6% | 122 out of 37603 genes, 0.3% | 1 | View Result |
| integral to endoplasmic reticulum membrane | 6 out of 946 genes, 0.6% | 122 out of 37603 genes, 0.3% | 1 | View Result |
| nuclear ubiquitin ligase complex | 4 out of 946 genes, 0.4% | 67 out of 37603 genes, 0.2% | 1 | View Result |
| interstitial matrix | 2 out of 946 genes, 0.2% | 20 out of 37603 genes, 0.1% | 1 | View Result |
| integral to lumenal side of endoplasmic reticulum membrane | 2 out of 946 genes, 0.2% | 20 out of 37603 genes, 0.1% | 1 | View Result |
| germ cell nucleus | 3 out of 946 genes, 0.3% | 44 out of 37603 genes, 0.1% | 1 | View Result |
| intrinsic to endoplasmic reticulum membrane | 9 out of 946 genes, 1.0% | 217 out of 37603 genes, 0.6% | 1 | View Result |
| lamellipodium membrane | 2 out of 946 genes, 0.2% | 22 out of 37603 genes, 0.1% | 1 | View Result |
| fibril | 2 out of 946 genes, 0.2% | 22 out of 37603 genes, 0.1% | 1 | View Result |
| nuclear periphery | 10 out of 946 genes, 1.1% | 252 out of 37603 genes, 0.7% | 1 | View Result |
| nucleus | 280 out of 946 genes, 29.6% | 10441 out of 37603 genes, 27.8% | 1 | View Result |
| cleavage furrow | 3 out of 946 genes, 0.3% | 46 out of 37603 genes, 0.1% | 1 | View Result |
| dendritic shaft | 3 out of 946 genes, 0.3% | 46 out of 37603 genes, 0.1% | 1 | View Result |
| integral to organelle membrane | 10 out of 946 genes, 1.1% | 257 out of 37603 genes, 0.7% | 1 | View Result |
| trans-Golgi network membrane | 2 out of 946 genes, 0.2% | 24 out of 37603 genes, 0.1% | 1 | View Result |
| cell-substrate adherens junction | 13 out of 946 genes, 1.4% | 359 out of 37603 genes, 1.0% | 1 | View Result |
| cytosolic ribosome | 4 out of 946 genes, 0.4% | 78 out of 37603 genes, 0.2% | 1 | View Result |
| signalosome | 2 out of 946 genes, 0.2% | 26 out of 37603 genes, 0.1% | 1 | View Result |
| main axon | 3 out of 946 genes, 0.3% | 52 out of 37603 genes, 0.1% | 1 | View Result |
| smooth endoplasmic reticulum | 2 out of 946 genes, 0.2% | 27 out of 37603 genes, 0.1% | 1 | View Result |
| contractile fiber part | 12 out of 946 genes, 1.3% | 339 out of 37603 genes, 0.9% | 1 | View Result |
| cytosolic small ribosomal subunit | 2 out of 946 genes, 0.2% | 28 out of 37603 genes, 0.1% | 1 | View Result |
| transport vesicle membrane | 5 out of 946 genes, 0.5% | 113 out of 37603 genes, 0.3% | 1 | View Result |
| site of polarized growth | 11 out of 946 genes, 1.2% | 311 out of 37603 genes, 0.8% | 1 | View Result |
| SCF ubiquitin ligase complex | 2 out of 946 genes, 0.2% | 29 out of 37603 genes, 0.1% | 1 | View Result |
| cell-substrate junction | 13 out of 946 genes, 1.4% | 381 out of 37603 genes, 1.0% | 1 | View Result |
| cell projection membrane | 12 out of 946 genes, 1.3% | 349 out of 37603 genes, 0.9% | 1 | View Result |
| phagocytic cup | 3 out of 946 genes, 0.3% | 57 out of 37603 genes, 0.2% | 1 | View Result |
| cell projection part | 33 out of 946 genes, 3.5% | 1106 out of 37603 genes, 2.9% | 1 | View Result |
| sarcoplasmic reticulum membrane | 2 out of 946 genes, 0.2% | 31 out of 37603 genes, 0.1% | 1 | View Result |
| neuron projection terminus | 3 out of 946 genes, 0.3% | 59 out of 37603 genes, 0.2% | 1 | View Result |
| lipid particle | 4 out of 946 genes, 0.4% | 89 out of 37603 genes, 0.2% | 1 | View Result |
| heterotrimeric G-protein complex | 2 out of 946 genes, 0.2% | 32 out of 37603 genes, 0.1% | 1 | View Result |
| non-membrane-bounded organelle | 210 out of 946 genes, 22.2% | 7901 out of 37603 genes, 21.0% | 1 | View Result |
| intracellular non-membrane-bounded organelle | 210 out of 946 genes, 22.2% | 7901 out of 37603 genes, 21.0% | 1 | View Result |
| cell body | 28 out of 946 genes, 3.0% | 936 out of 37603 genes, 2.5% | 1 | View Result |
| stereocilium | 2 out of 946 genes, 0.2% | 33 out of 37603 genes, 0.1% | 1 | View Result |
| basolateral plasma membrane | 21 out of 946 genes, 2.2% | 687 out of 37603 genes, 1.8% | 1 | View Result |
| cell junction | 43 out of 946 genes, 4.5% | 1502 out of 37603 genes, 4.0% | 1 | View Result |
| rough endoplasmic reticulum | 3 out of 946 genes, 0.3% | 64 out of 37603 genes, 0.2% | 1 | View Result |
| ER to Golgi transport vesicle membrane | 2 out of 946 genes, 0.2% | 35 out of 37603 genes, 0.1% | 1 | View Result |
| stereocilium bundle | 2 out of 946 genes, 0.2% | 35 out of 37603 genes, 0.1% | 1 | View Result |
| clathrin-coated endocytic vesicle membrane | 2 out of 946 genes, 0.2% | 36 out of 37603 genes, 0.1% | 1 | View Result |
| early endosome | 10 out of 946 genes, 1.1% | 301 out of 37603 genes, 0.8% | 1 | View Result |
| sarcomere | 10 out of 946 genes, 1.1% | 302 out of 37603 genes, 0.8% | 1 | View Result |
| histone acetyltransferase complex | 7 out of 946 genes, 0.7% | 198 out of 37603 genes, 0.5% | 1 | View Result |
| nuclear origin of replication recognition complex | 2 out of 946 genes, 0.2% | 37 out of 37603 genes, 0.1% | 1 | View Result |
| organelle envelope | 54 out of 946 genes, 5.7% | 1940 out of 37603 genes, 5.2% | 1 | View Result |
| organelle part | 384 out of 946 genes, 40.6% | 14834 out of 37603 genes, 39.4% | 1 | View Result |
| trans-Golgi network | 6 out of 946 genes, 0.6% | 167 out of 37603 genes, 0.4% | 1 | View Result |
| cell division site | 4 out of 946 genes, 0.4% | 102 out of 37603 genes, 0.3% | 1 | View Result |
| cell division site part | 4 out of 946 genes, 0.4% | 102 out of 37603 genes, 0.3% | 1 | View Result |
| centriole | 3 out of 946 genes, 0.3% | 71 out of 37603 genes, 0.2% | 1 | View Result |
| microtubule organizing center part | 5 out of 946 genes, 0.5% | 138 out of 37603 genes, 0.4% | 1 | View Result |
| cullin-RING ubiquitin ligase complex | 6 out of 946 genes, 0.6% | 173 out of 37603 genes, 0.5% | 1 | View Result |
| envelope | 54 out of 946 genes, 5.7% | 1966 out of 37603 genes, 5.2% | 1 | View Result |
| mitochondrial membrane | 27 out of 946 genes, 2.9% | 943 out of 37603 genes, 2.5% | 1 | View Result |
| clathrin-coated endocytic vesicle | 2 out of 946 genes, 0.2% | 42 out of 37603 genes, 0.1% | 1 | View Result |
| dendritic spine | 8 out of 946 genes, 0.8% | 248 out of 37603 genes, 0.7% | 1 | View Result |
| neuron spine | 8 out of 946 genes, 0.8% | 248 out of 37603 genes, 0.7% | 1 | View Result |
| mitochondrial outer membrane | 7 out of 946 genes, 0.7% | 214 out of 37603 genes, 0.6% | 1 | View Result |
| intrinsic to organelle membrane | 14 out of 946 genes, 1.5% | 470 out of 37603 genes, 1.2% | 1 | View Result |
| nuclear membrane | 9 out of 946 genes, 1.0% | 287 out of 37603 genes, 0.8% | 1 | View Result |
| replication fork | 3 out of 946 genes, 0.3% | 77 out of 37603 genes, 0.2% | 1 | View Result |
| pore complex | 4 out of 946 genes, 0.4% | 112 out of 37603 genes, 0.3% | 1 | View Result |
| nucleolus | 56 out of 946 genes, 5.9% | 2075 out of 37603 genes, 5.5% | 1 | View Result |
| cytosolic large ribosomal subunit | 2 out of 946 genes, 0.2% | 45 out of 37603 genes, 0.1% | 1 | View Result |
| synapse | 21 out of 946 genes, 2.2% | 741 out of 37603 genes, 2.0% | 1 | View Result |
| endoplasmic reticulum-Golgi intermediate compartment | 3 out of 946 genes, 0.3% | 79 out of 37603 genes, 0.2% | 1 | View Result |
| extrinsic to internal side of plasma membrane | 4 out of 946 genes, 0.4% | 114 out of 37603 genes, 0.3% | 1 | View Result |
| mitochondrial intermembrane space | 3 out of 946 genes, 0.3% | 80 out of 37603 genes, 0.2% | 1 | View Result |
| centrosome | 19 out of 946 genes, 2.0% | 672 out of 37603 genes, 1.8% | 1 | View Result |
| nucleosome | 2 out of 946 genes, 0.2% | 48 out of 37603 genes, 0.1% | 1 | View Result |
| ER to Golgi transport vesicle | 2 out of 946 genes, 0.2% | 48 out of 37603 genes, 0.1% | 1 | View Result |
| microtubule basal body | 3 out of 946 genes, 0.3% | 83 out of 37603 genes, 0.2% | 1 | View Result |
| histone methyltransferase complex | 5 out of 946 genes, 0.5% | 155 out of 37603 genes, 0.4% | 1 | View Result |
| origin recognition complex | 2 out of 946 genes, 0.2% | 50 out of 37603 genes, 0.1% | 1 | View Result |
| nuclear heterochromatin | 2 out of 946 genes, 0.2% | 50 out of 37603 genes, 0.1% | 1 | View Result |
| mitochondrial envelope | 29 out of 946 genes, 3.1% | 1070 out of 37603 genes, 2.8% | 1 | View Result |
| SWI/SNF-type complex | 2 out of 946 genes, 0.2% | 51 out of 37603 genes, 0.1% | 1 | View Result |
| mitochondrial matrix | 12 out of 946 genes, 1.3% | 422 out of 37603 genes, 1.1% | 1 | View Result |
| acrosomal membrane | 2 out of 946 genes, 0.2% | 52 out of 37603 genes, 0.1% | 1 | View Result |
| mediator complex | 2 out of 946 genes, 0.2% | 52 out of 37603 genes, 0.1% | 1 | View Result |
| mitochondrial inner membrane | 16 out of 946 genes, 1.7% | 577 out of 37603 genes, 1.5% | 1 | View Result |
| organelle envelope lumen | 3 out of 946 genes, 0.3% | 89 out of 37603 genes, 0.2% | 1 | View Result |
| perikaryon | 2 out of 946 genes, 0.2% | 54 out of 37603 genes, 0.1% | 1 | View Result |
| Golgi membrane | 22 out of 946 genes, 2.3% | 819 out of 37603 genes, 2.2% | 1 | View Result |
| nuclear inner membrane | 2 out of 946 genes, 0.2% | 56 out of 37603 genes, 0.1% | 1 | View Result |
| autophagic vacuole | 2 out of 946 genes, 0.2% | 56 out of 37603 genes, 0.1% | 1 | View Result |
| SAGA-type complex | 2 out of 946 genes, 0.2% | 56 out of 37603 genes, 0.1% | 1 | View Result |
| flagellum | 3 out of 946 genes, 0.3% | 93 out of 37603 genes, 0.2% | 1 | View Result |
| pigment granule | 6 out of 946 genes, 0.6% | 206 out of 37603 genes, 0.5% | 1 | View Result |
| intercalated disc | 2 out of 946 genes, 0.2% | 57 out of 37603 genes, 0.2% | 1 | View Result |
| Golgi apparatus part | 30 out of 946 genes, 3.2% | 1145 out of 37603 genes, 3.0% | 1 | View Result |
| sex chromosome | 2 out of 946 genes, 0.2% | 59 out of 37603 genes, 0.2% | 1 | View Result |
| sarcoplasmic reticulum | 2 out of 946 genes, 0.2% | 59 out of 37603 genes, 0.2% | 1 | View Result |
| heterochromatin | 4 out of 946 genes, 0.4% | 135 out of 37603 genes, 0.4% | 1 | View Result |
| organelle outer membrane | 7 out of 946 genes, 0.7% | 251 out of 37603 genes, 0.7% | 1 | View Result |
| methyltransferase complex | 5 out of 946 genes, 0.5% | 176 out of 37603 genes, 0.5% | 1 | View Result |
| inclusion body | 3 out of 946 genes, 0.3% | 101 out of 37603 genes, 0.3% | 1 | View Result |
| protein-DNA complex | 4 out of 946 genes, 0.4% | 141 out of 37603 genes, 0.4% | 1 | View Result |
| nucleoplasm | 69 out of 946 genes, 7.3% | 2713 out of 37603 genes, 7.2% | 1 | View Result |
| sarcoplasm | 2 out of 946 genes, 0.2% | 64 out of 37603 genes, 0.2% | 1 | View Result |
| pronucleus | 2 out of 946 genes, 0.2% | 64 out of 37603 genes, 0.2% | 1 | View Result |
| chromatin | 19 out of 946 genes, 2.0% | 737 out of 37603 genes, 2.0% | 1 | View Result |
| catalytic step 2 spliceosome | 3 out of 946 genes, 0.3% | 106 out of 37603 genes, 0.3% | 1 | View Result |
| basal plasma membrane | 2 out of 946 genes, 0.2% | 67 out of 37603 genes, 0.2% | 1 | View Result |
| nuclear chromosome part | 14 out of 946 genes, 1.5% | 547 out of 37603 genes, 1.5% | 1 | View Result |
| transport vesicle | 6 out of 946 genes, 0.6% | 228 out of 37603 genes, 0.6% | 1 | View Result |
| mitochondrial part | 40 out of 946 genes, 4.2% | 1586 out of 37603 genes, 4.2% | 1 | View Result |
| internal side of plasma membrane | 6 out of 946 genes, 0.6% | 229 out of 37603 genes, 0.6% | 1 | View Result |
| intracellular part | 724 out of 946 genes, 76.5% | 28781 out of 37603 genes, 76.5% | 1 | View Result |
| cell-cell contact zone | 2 out of 946 genes, 0.2% | 69 out of 37603 genes, 0.2% | 1 | View Result |
| outer membrane | 7 out of 946 genes, 0.7% | 271 out of 37603 genes, 0.7% | 1 | View Result |
| acrosomal vesicle | 4 out of 946 genes, 0.4% | 153 out of 37603 genes, 0.4% | 1 | View Result |
| intracellular | 736 out of 946 genes, 77.8% | 29285 out of 37603 genes, 77.9% | 1 | View Result |
| microtubule cytoskeleton | 52 out of 946 genes, 5.5% | 2086 out of 37603 genes, 5.5% | 1 | View Result |
| Cajal body | 2 out of 946 genes, 0.2% | 74 out of 37603 genes, 0.2% | 1 | View Result |
| tight junction | 4 out of 946 genes, 0.4% | 157 out of 37603 genes, 0.4% | 1 | View Result |
| occluding junction | 4 out of 946 genes, 0.4% | 157 out of 37603 genes, 0.4% | 1 | View Result |
| small nuclear ribonucleoprotein complex | 2 out of 946 genes, 0.2% | 75 out of 37603 genes, 0.2% | 1 | View Result |
| early endosome membrane | 2 out of 946 genes, 0.2% | 75 out of 37603 genes, 0.2% | 1 | View Result |
| small ribosomal subunit | 3 out of 946 genes, 0.3% | 120 out of 37603 genes, 0.3% | 1 | View Result |
| vesicle coat | 2 out of 946 genes, 0.2% | 78 out of 37603 genes, 0.2% | 1 | View Result |
| condensed chromosome kinetochore | 3 out of 946 genes, 0.3% | 121 out of 37603 genes, 0.3% | 1 | View Result |
| basal part of cell | 2 out of 946 genes, 0.2% | 79 out of 37603 genes, 0.2% | 1 | View Result |
| Golgi-associated vesicle membrane | 2 out of 946 genes, 0.2% | 80 out of 37603 genes, 0.2% | 1 | View Result |
| nuclear pore | 2 out of 946 genes, 0.2% | 81 out of 37603 genes, 0.2% | 1 | View Result |
| microtubule organizing center | 26 out of 946 genes, 2.7% | 1077 out of 37603 genes, 2.9% | 1 | View Result |
| basal lamina | 2 out of 946 genes, 0.2% | 82 out of 37603 genes, 0.2% | 1 | View Result |
| postsynaptic density | 4 out of 946 genes, 0.4% | 170 out of 37603 genes, 0.5% | 1 | View Result |
| dendritic spine head | 4 out of 946 genes, 0.4% | 170 out of 37603 genes, 0.5% | 1 | View Result |
| clathrin-coated vesicle | 10 out of 946 genes, 1.1% | 423 out of 37603 genes, 1.1% | 1 | View Result |
| transcriptional repressor complex | 3 out of 946 genes, 0.3% | 128 out of 37603 genes, 0.3% | 1 | View Result |
| proton-transporting two-sector ATPase complex | 2 out of 946 genes, 0.2% | 85 out of 37603 genes, 0.2% | 1 | View Result |
| cytosolic part | 6 out of 946 genes, 0.6% | 259 out of 37603 genes, 0.7% | 1 | View Result |
| condensed chromosome, centromeric region | 3 out of 946 genes, 0.3% | 130 out of 37603 genes, 0.3% | 1 | View Result |
| ubiquitin ligase complex | 7 out of 946 genes, 0.7% | 303 out of 37603 genes, 0.8% | 1 | View Result |
| nuclear chromatin | 8 out of 946 genes, 0.8% | 346 out of 37603 genes, 0.9% | 1 | View Result |
| membrane-enclosed lumen | 177 out of 946 genes, 18.7% | 7219 out of 37603 genes, 19.2% | 1 | View Result |
| myelin sheath | 2 out of 946 genes, 0.2% | 91 out of 37603 genes, 0.2% | 1 | View Result |
| DNA-directed RNA polymerase II, holoenzyme | 3 out of 946 genes, 0.3% | 140 out of 37603 genes, 0.4% | 1 | View Result |
| condensed chromosome | 7 out of 946 genes, 0.7% | 318 out of 37603 genes, 0.8% | 1 | View Result |
| organelle lumen | 174 out of 946 genes, 18.4% | 7139 out of 37603 genes, 19.0% | 1 | View Result |
| spindle | 11 out of 946 genes, 1.2% | 496 out of 37603 genes, 1.3% | 1 | View Result |
| ribosome | 9 out of 946 genes, 1.0% | 410 out of 37603 genes, 1.1% | 1 | View Result |
| clathrin coated vesicle membrane | 3 out of 946 genes, 0.3% | 145 out of 37603 genes, 0.4% | 1 | View Result |
| peroxisome | 6 out of 946 genes, 0.6% | 284 out of 37603 genes, 0.8% | 1 | View Result |
| spliceosomal complex | 7 out of 946 genes, 0.7% | 330 out of 37603 genes, 0.9% | 1 | View Result |
| nuclear chromosome | 15 out of 946 genes, 1.6% | 677 out of 37603 genes, 1.8% | 1 | View Result |
| organelle inner membrane | 21 out of 946 genes, 2.2% | 932 out of 37603 genes, 2.5% | 1 | View Result |
| PML body | 3 out of 946 genes, 0.3% | 150 out of 37603 genes, 0.4% | 1 | View Result |
| intrinsic to membrane | 263 out of 946 genes, 27.8% | 10799 out of 37603 genes, 28.7% | 1 | View Result |
| DNA-directed RNA polymerase complex | 3 out of 946 genes, 0.3% | 159 out of 37603 genes, 0.4% | 1 | View Result |
| nuclear DNA-directed RNA polymerase complex | 3 out of 946 genes, 0.3% | 159 out of 37603 genes, 0.4% | 1 | View Result |
| ion channel complex | 4 out of 946 genes, 0.4% | 208 out of 37603 genes, 0.6% | 1 | View Result |
| coated vesicle | 11 out of 946 genes, 1.2% | 526 out of 37603 genes, 1.4% | 1 | View Result |
| synaptic vesicle | 3 out of 946 genes, 0.3% | 161 out of 37603 genes, 0.4% | 1 | View Result |
| coated vesicle membrane | 4 out of 946 genes, 0.4% | 209 out of 37603 genes, 0.6% | 1 | View Result |
| axon part | 6 out of 946 genes, 0.6% | 304 out of 37603 genes, 0.8% | 1 | View Result |
| vesicular fraction | 10 out of 946 genes, 1.1% | 487 out of 37603 genes, 1.3% | 1 | View Result |
| RNA polymerase complex | 3 out of 946 genes, 0.3% | 164 out of 37603 genes, 0.4% | 1 | View Result |
| ribosomal subunit | 5 out of 946 genes, 0.5% | 261 out of 37603 genes, 0.7% | 1 | View Result |
| chromatin remodeling complex | 7 out of 946 genes, 0.7% | 368 out of 37603 genes, 1.0% | 1 | View Result |
| intracellular organelle lumen | 167 out of 946 genes, 17.7% | 7060 out of 37603 genes, 18.8% | 1 | View Result |
| intrinsic to Golgi membrane | 2 out of 946 genes, 0.2% | 128 out of 37603 genes, 0.3% | 1 | View Result |
| mitochondrial membrane part | 4 out of 946 genes, 0.4% | 234 out of 37603 genes, 0.6% | 1 | View Result |
| Golgi-associated vesicle | 2 out of 946 genes, 0.2% | 135 out of 37603 genes, 0.4% | 1 | View Result |
| kinetochore | 4 out of 946 genes, 0.4% | 242 out of 37603 genes, 0.6% | 1 | View Result |
| microbody | 6 out of 946 genes, 0.6% | 344 out of 37603 genes, 0.9% | 1 | View Result |
| synapse part | 8 out of 946 genes, 0.8% | 453 out of 37603 genes, 1.2% | 1 | View Result |
| large ribosomal subunit | 2 out of 946 genes, 0.2% | 147 out of 37603 genes, 0.4% | 1 | View Result |
| membrane fraction | 14 out of 946 genes, 1.5% | 745 out of 37603 genes, 2.0% | 1 | View Result |
| nuclear speck | 3 out of 946 genes, 0.3% | 213 out of 37603 genes, 0.6% | 1 | View Result |
| membrane coat | 2 out of 946 genes, 0.2% | 157 out of 37603 genes, 0.4% | 1 | View Result |
| coated membrane | 2 out of 946 genes, 0.2% | 157 out of 37603 genes, 0.4% | 1 | View Result |
| intracellular organelle | 607 out of 946 genes, 64.2% | 24874 out of 37603 genes, 66.1% | 1 | View Result |
| chromosome, telomeric region | 2 out of 946 genes, 0.2% | 158 out of 37603 genes, 0.4% | 1 | View Result |
| brush border | 2 out of 946 genes, 0.2% | 160 out of 37603 genes, 0.4% | 1 | View Result |
| condensed nuclear chromosome | 2 out of 946 genes, 0.2% | 162 out of 37603 genes, 0.4% | 1 | View Result |
| apical junction complex | 5 out of 946 genes, 0.5% | 339 out of 37603 genes, 0.9% | 1 | View Result |
| apicolateral plasma membrane | 5 out of 946 genes, 0.5% | 348 out of 37603 genes, 0.9% | 1 | View Result |
| microtubule associated complex | 2 out of 946 genes, 0.2% | 178 out of 37603 genes, 0.5% | 1 | View Result |
| transcription factor complex | 9 out of 946 genes, 1.0% | 555 out of 37603 genes, 1.5% | 1 | View Result |
| chromosomal part | 28 out of 946 genes, 3.0% | 1456 out of 37603 genes, 3.9% | 1 | View Result |
| organelle | 607 out of 946 genes, 64.2% | 25054 out of 37603 genes, 66.6% | 1 | View Result |
| ribonucleoprotein complex | 25 out of 946 genes, 2.6% | 1367 out of 37603 genes, 3.6% | 1 | View Result |
| chromosome, centromeric region | 4 out of 946 genes, 0.4% | 354 out of 37603 genes, 0.9% | 1 | View Result |
| membrane-bounded organelle | 541 out of 946 genes, 57.2% | 22716 out of 37603 genes, 60.4% | 1 | View Result |
| chromosome | 30 out of 946 genes, 3.2% | 1683 out of 37603 genes, 4.5% | 1 | View Result |
| nucleoplasm part | 33 out of 946 genes, 3.5% | 1841 out of 37603 genes, 4.9% | 1 | View Result |
| intracellular membrane-bounded organelle | 534 out of 946 genes, 56.4% | 22524 out of 37603 genes, 59.9% | 1 | View Result |
| nuclear part | 149 out of 946 genes, 15.8% | 7093 out of 37603 genes, 18.9% | 1 | View Result |
| nuclear lumen | 128 out of 946 genes, 13.5% | 6408 out of 37603 genes, 17.0% | 1 | View Result |
| nuclear body | 7 out of 946 genes, 0.7% | 729 out of 37603 genes, 1.9% | 1 | View Result |
| insoluble fraction | 18 out of 946 genes, 1.9% | 1517 out of 37603 genes, 4.0% | 1 | View Result |
| cell fraction | 27 out of 946 genes, 2.9% | 2030 out of 37603 genes, 5.4% | 1 | View Result |

| Gene Ontology term | Genes annotated to the term |
| --- | --- |
| extracellular space | Unigene29399\_Mf\_liverA, CL6018.Contig2\_Mf\_liverA, CL4086.Contig1\_Mf\_liverA, CL1811.Contig2\_Mf\_liverA, Unigene5852\_Mf\_liverA, Unigene8106\_Mf\_liverA, CL3669.Contig2\_Mf\_liverA, Unigene7612\_Mf\_liverA, Unigene42975\_Mf\_liverA, Unigene14536\_Mf\_liverA, Unigene15318\_Mf\_liverA, Unigene7950\_Mf\_liverA, Unigene14603\_Mf\_liverA, Unigene22980\_Mf\_liverA, Unigene9081\_Mf\_liverA, Unigene30815\_Mf\_liverA, Unigene10351\_Mf\_liverA, Unigene15914\_Mf\_liverA, Unigene15982\_Mf\_liverA, Unigene19049\_Mf\_liverA, CL81.Contig1\_Mf\_liverA, Unigene35237\_Mf\_liverA, Unigene5501\_Mf\_liverA, Unigene37259\_Mf\_liverA, Unigene30039\_Mf\_liverA, Unigene29231\_Mf\_liverA, Unigene37698\_Mf\_liverA, Unigene38689\_Mf\_liverA, Unigene1137\_Mf\_liverA, Unigene35958\_Mf\_liverA, Unigene36765\_Mf\_liverA, CL6038.Contig2\_Mf\_liverA, CL4583.Contig2\_Mf\_liverA, Unigene13525\_Mf\_liverA, Unigene14594\_Mf\_liverA, Unigene13894\_Mf\_liverA, Unigene37099\_Mf\_liverA, Unigene37904\_Mf\_liverA, Unigene22979\_Mf\_liverA, Unigene30495\_Mf\_liverA, Unigene28662\_Mf\_liverA, Unigene37263\_Mf\_liverA, Unigene38110\_Mf\_liverA, Unigene31333\_Mf\_liverA, NM\_010233, Unigene4630\_Mf\_liverA, Unigene29426\_Mf\_liverA, Unigene11544\_Mf\_liverA, Unigene30494\_Mf\_liverA, CL3575.Contig1\_Mf\_liverA, Unigene22978\_Mf\_liverA, Unigene1212\_Mf\_liverA, Unigene14050\_Mf\_liverA, CL787.Contig1\_Mf\_liverA, Unigene13106\_Mf\_liverA, Unigene19623\_Mf\_liverA, Unigene37454\_Mf\_liverA, Unigene29938\_Mf\_liverA, Unigene15529\_Mf\_liverA, Unigene33832\_Mf\_liverA, CL725.Contig1\_Mf\_liverA, Unigene37278\_Mf\_liverA, Unigene13616\_Mf\_liverA, Unigene21337\_Mf\_liverA, Unigene34034\_Mf\_liverA, CL1190.Contig3\_Mf\_liverA, Unigene34258\_Mf\_liverA, Unigene13143\_Mf\_liverA, Unigene25595\_Mf\_liverA, Unigene14582\_Mf\_liverA, Unigene45728\_Mf\_liverA, Unigene34746\_Mf\_liverA, Unigene36112\_Mf\_liverA, Unigene9990\_Mf\_liverA, Unigene941\_Mf\_liverA, Unigene560\_Mf\_liverA, Unigene27438\_Mf\_liverA, Unigene36643\_Mf\_liverA, NM\_011580, Unigene35494\_Mf\_liverA, CL3669.Contig1\_Mf\_liverA, Unigene27249\_Mf\_liverA, Unigene26521\_Mf\_liverA, Unigene26083\_Mf\_liverA, Unigene35059\_Mf\_liverA, CL4040.Contig2\_Mf\_liverA, Unigene36644\_Mf\_liverA, Unigene425\_Mf\_liverA, Unigene21693\_Mf\_liverA, Unigene22977\_Mf\_liverA, Unigene30493\_Mf\_liverA, Unigene677\_Mf\_liverA, Unigene15592\_Mf\_liverA, Unigene13296\_Mf\_liverA, Unigene27248\_Mf\_liverA, Unigene32716\_Mf\_liverA, Unigene21336\_Mf\_liverA, Unigene37729\_Mf\_liverA, Unigene8132\_Mf\_liverA, Unigene41583\_Mf\_liverA, Unigene29940\_Mf\_liverA, Unigene2195\_Mf\_liverA, Unigene43068\_Mf\_liverA, Unigene30814\_Mf\_liverA, Unigene24157\_Mf\_liverA, Unigene36667\_Mf\_liverA, Unigene18340\_Mf\_liverA, Unigene32058\_Mf\_liverA, CL3911.Contig2\_Mf\_liverA, Unigene18510\_Mf\_liverA, CL6018.Contig1\_Mf\_liverA, CL1347.Contig3\_Mf\_liverA, Unigene30786\_Mf\_liverA, Unigene38387\_Mf\_liverA, Unigene36426\_Mf\_liverA, Unigene17569\_Mf\_liverA, Unigene32059\_Mf\_liverA |
| extracellular region | Unigene29399\_Mf\_liverA, CL6018.Contig2\_Mf\_liverA, Unigene38831\_Mf\_liverA, Unigene5852\_Mf\_liverA, Unigene8106\_Mf\_liverA, CL3669.Contig2\_Mf\_liverA, Unigene7612\_Mf\_liverA, Unigene42975\_Mf\_liverA, Unigene14536\_Mf\_liverA, CL1828.Contig1\_Mf\_liverA, NM\_007737, Unigene7950\_Mf\_liverA, Unigene14603\_Mf\_liverA, Unigene9081\_Mf\_liverA, Unigene15914\_Mf\_liverA, CL482.Contig1\_Mf\_liverA, Unigene19049\_Mf\_liverA, CL81.Contig1\_Mf\_liverA, Unigene29405\_Mf\_liverA, Unigene37698\_Mf\_liverA, Unigene38689\_Mf\_liverA, CL33.Contig4\_Mf\_liverA, Unigene19719\_Mf\_liverA, Unigene34124\_Mf\_liverA, CL4790.Contig1\_Mf\_liverA, Unigene1137\_Mf\_liverA, Unigene36765\_Mf\_liverA, Unigene34810\_Mf\_liverA, Unigene27420\_Mf\_liverA, CL6038.Contig2\_Mf\_liverA, Unigene25261\_Mf\_liverA, Unigene14594\_Mf\_liverA, Unigene17397\_Mf\_liverA, Unigene37099\_Mf\_liverA, Unigene21255\_Mf\_liverA, Unigene38479\_Mf\_liverA, Unigene37904\_Mf\_liverA, Unigene30495\_Mf\_liverA, CL5978.Contig3\_Mf\_liverA, Unigene14276\_Mf\_liverA, Unigene37263\_Mf\_liverA, Unigene38110\_Mf\_liverA, Unigene31333\_Mf\_liverA, CL33.Contig3\_Mf\_liverA, NM\_010233, Unigene11\_Mf\_liverA, Unigene4630\_Mf\_liverA, CL376.Contig1\_Mf\_liverA, NM\_009776, Unigene11544\_Mf\_liverA, NM\_018780, Unigene18117\_Mf\_liverA, CL3575.Contig1\_Mf\_liverA, Unigene3174\_Mf\_liverA, Unigene17396\_Mf\_liverA, Unigene22978\_Mf\_liverA, Unigene14050\_Mf\_liverA, Unigene38657\_Mf\_liverA, Unigene23328\_Mf\_liverA, Unigene8054\_Mf\_liverA, Unigene36677\_Mf\_liverA, Unigene5134\_Mf\_liverA, CL787.Contig1\_Mf\_liverA, Unigene9150\_Mf\_liverA, Unigene19623\_Mf\_liverA, Unigene7608\_Mf\_liverA, Unigene37454\_Mf\_liverA, Unigene26380\_Mf\_liverA, Unigene37278\_Mf\_liverA, Unigene33459\_Mf\_liverA, Unigene34034\_Mf\_liverA, NM\_008808, Unigene34258\_Mf\_liverA, Unigene13143\_Mf\_liverA, Unigene30261\_Mf\_liverA, Unigene27419\_Mf\_liverA, Unigene14582\_Mf\_liverA, Unigene45728\_Mf\_liverA, Unigene34746\_Mf\_liverA, Unigene36112\_Mf\_liverA, Unigene941\_Mf\_liverA, Unigene9990\_Mf\_liverA, Unigene13945\_Mf\_liverA, Unigene33560\_Mf\_liverA, Unigene27438\_Mf\_liverA, Unigene35494\_Mf\_liverA, Unigene34123\_Mf\_liverA, Unigene15145\_Mf\_liverA, CL3411.Contig2\_Mf\_liverA, Unigene30584\_Mf\_liverA, CL3669.Contig1\_Mf\_liverA, Unigene27249\_Mf\_liverA, Unigene26521\_Mf\_liverA, Unigene35059\_Mf\_liverA, CL4040.Contig2\_Mf\_liverA, CL1736.Contig2\_Mf\_liverA, NM\_015734, Unigene425\_Mf\_liverA, Unigene5606\_Mf\_liverA, Unigene44992\_Mf\_liverA, Unigene22977\_Mf\_liverA, Unigene33271\_Mf\_liverA, Unigene30493\_Mf\_liverA, Unigene27248\_Mf\_liverA, Unigene32716\_Mf\_liverA, Unigene13296\_Mf\_liverA, Unigene37729\_Mf\_liverA, Unigene8132\_Mf\_liverA, Unigene29940\_Mf\_liverA, Unigene8246\_Mf\_liverA, CL3232.Contig1\_Mf\_liverA, Unigene24157\_Mf\_liverA, Unigene32058\_Mf\_liverA, NM\_009713, CL6018.Contig1\_Mf\_liverA, Unigene32791\_Mf\_liverA, Unigene21256\_Mf\_liverA, Unigene23055\_Mf\_liverA, CL442.Contig2\_Mf\_liverA, CL1347.Contig3\_Mf\_liverA, CL6039.Contig1\_Mf\_liverA, Unigene38893\_Mf\_liverA, CL3339.Contig1\_Mf\_liverA, CL5827.Contig1\_Mf\_liverA, Unigene17569\_Mf\_liverA, Unigene32059\_Mf\_liverA, NM\_009933, CL4086.Contig1\_Mf\_liverA, CL1811.Contig2\_Mf\_liverA, Unigene2162\_Mf\_liverA, Unigene15318\_Mf\_liverA, Unigene22980\_Mf\_liverA, Unigene30815\_Mf\_liverA, Unigene10351\_Mf\_liverA, Unigene1130\_Mf\_liverA, Unigene15982\_Mf\_liverA, Unigene30587\_Mf\_liverA, Unigene35237\_Mf\_liverA, Unigene36650\_Mf\_liverA, Unigene5501\_Mf\_liverA, Unigene37259\_Mf\_liverA, Unigene30039\_Mf\_liverA, Unigene29231\_Mf\_liverA, Unigene12230\_Mf\_liverA, CL3030.Contig2\_Mf\_liverA, Unigene4985\_Mf\_liverA, CL3122.Contig2\_Mf\_liverA, Unigene35958\_Mf\_liverA, Unigene43037\_Mf\_liverA, CL4583.Contig2\_Mf\_liverA, Unigene13525\_Mf\_liverA, Unigene13894\_Mf\_liverA, Unigene36328\_Mf\_liverA, NM\_008871, CL6039.Contig2\_Mf\_liverA, Unigene22979\_Mf\_liverA, Unigene28662\_Mf\_liverA, Unigene8033\_Mf\_liverA, Unigene29426\_Mf\_liverA, Unigene30494\_Mf\_liverA, Unigene36987\_Mf\_liverA, Unigene1212\_Mf\_liverA, Unigene30585\_Mf\_liverA, Unigene6854\_Mf\_liverA, Unigene38593\_Mf\_liverA, NM\_033325, Unigene13106\_Mf\_liverA, Unigene29938\_Mf\_liverA, Unigene15529\_Mf\_liverA, Unigene33832\_Mf\_liverA, CL725.Contig1\_Mf\_liverA, Unigene13616\_Mf\_liverA, Unigene21337\_Mf\_liverA, CL1190.Contig3\_Mf\_liverA, CL5978.Contig2\_Mf\_liverA, CL5293.Contig2\_Mf\_liverA, CL2251.Contig1\_Mf\_liverA, CL5293.Contig1\_Mf\_liverA, Unigene26381\_Mf\_liverA, Unigene25595\_Mf\_liverA, Unigene35090\_Mf\_liverA, Unigene560\_Mf\_liverA, Unigene36643\_Mf\_liverA, NM\_011580, Unigene26083\_Mf\_liverA, Unigene36644\_Mf\_liverA, Unigene36910\_Mf\_liverA, Unigene21693\_Mf\_liverA, Unigene677\_Mf\_liverA, Unigene15592\_Mf\_liverA, Unigene21336\_Mf\_liverA, Unigene5422\_Mf\_liverA, Unigene41583\_Mf\_liverA, Unigene30814\_Mf\_liverA, Unigene43068\_Mf\_liverA, Unigene2195\_Mf\_liverA, Unigene36667\_Mf\_liverA, Unigene36673\_Mf\_liverA, Unigene18340\_Mf\_liverA, CL3911.Contig2\_Mf\_liverA, Unigene18510\_Mf\_liverA, Unigene30786\_Mf\_liverA, NM\_009636, Unigene38237\_Mf\_liverA, Unigene38387\_Mf\_liverA, Unigene36426\_Mf\_liverA, Unigene31492\_Mf\_liverA |
| cell periphery | Unigene24506\_Mf\_liverA, NM\_009898, Unigene25721\_Mf\_liverA, Unigene24111\_Mf\_liverA, Unigene28186\_Mf\_liverA, NM\_010378, Unigene29424\_Mf\_liverA, Unigene34218\_Mf\_liverA, Unigene26053\_Mf\_liverA, Unigene31045\_Mf\_liverA, Unigene25090\_Mf\_liverA, Unigene16463\_Mf\_liverA, CL238.Contig1\_Mf\_liverA, Unigene38689\_Mf\_liverA, NM\_010877, Unigene38919\_Mf\_liverA, Unigene1137\_Mf\_liverA, Unigene24252\_Mf\_liverA, NR\_004446, CL5586.Contig1\_Mf\_liverA, Unigene15703\_Mf\_liverA, Unigene35816\_Mf\_liverA, Unigene28331\_Mf\_liverA, NM\_010233, CL593.Contig2\_Mf\_liverA, Unigene13940\_Mf\_liverA, Unigene5138\_Mf\_liverA, Unigene37150\_Mf\_liverA, Unigene5330\_Mf\_liverA, Unigene23869\_Mf\_liverA, Unigene5552\_Mf\_liverA, Unigene13363\_Mf\_liverA, Unigene26065\_Mf\_liverA, CL2855.Contig2\_Mf\_liverA, Unigene25091\_Mf\_liverA, Unigene18125\_Mf\_liverA, Unigene28226\_Mf\_liverA, Unigene13097\_Mf\_liverA, Unigene44317\_Mf\_liverA, Unigene25047\_Mf\_liverA, Unigene37278\_Mf\_liverA, Unigene2919\_Mf\_liverA, Unigene21561\_Mf\_liverA, Unigene4922\_Mf\_liverA, Unigene13913\_Mf\_liverA, Unigene30261\_Mf\_liverA, Unigene9475\_Mf\_liverA, Unigene36112\_Mf\_liverA, Unigene9990\_Mf\_liverA, Unigene30459\_Mf\_liverA, Unigene139\_Mf\_liverA, Unigene26941\_Mf\_liverA, Unigene33560\_Mf\_liverA, NM\_172509, NM\_001143689, CL3055.Contig2\_Mf\_liverA, Unigene37575\_Mf\_liverA, Unigene27249\_Mf\_liverA, Unigene43515\_Mf\_liverA, Unigene27547\_Mf\_liverA, Unigene21842\_Mf\_liverA, NM\_134156, Unigene32889\_Mf\_liverA, CL4156.Contig1\_Mf\_liverA, Unigene27248\_Mf\_liverA, Unigene32716\_Mf\_liverA, CL5254.Contig1\_Mf\_liverA, Unigene31852\_Mf\_liverA, Unigene5456\_Mf\_liverA, NM\_009713, Unigene37076\_Mf\_liverA, Unigene2678\_Mf\_liverA, CL1738.Contig1\_Mf\_liverA, Unigene2746\_Mf\_liverA, Unigene5169\_Mf\_liverA, Unigene16465\_Mf\_liverA, Unigene10496\_Mf\_liverA, Unigene28021\_Mf\_liverA, NM\_009933, Unigene31251\_Mf\_liverA, NM\_011503, Unigene40610\_Mf\_liverA, CL186.Contig3\_Mf\_liverA, Unigene2162\_Mf\_liverA, CL425.Contig1\_Mf\_liverA, CL3150.Contig1\_Mf\_liverA, Unigene4697\_Mf\_liverA, Unigene37243\_Mf\_liverA, Unigene27026\_Mf\_liverA, Unigene30587\_Mf\_liverA, Unigene39403\_Mf\_liverA, Unigene30039\_Mf\_liverA, Unigene29231\_Mf\_liverA, Unigene5693\_Mf\_liverA, Unigene50250\_Mf\_liverA, CL4057.Contig1\_Mf\_liverA, Unigene35958\_Mf\_liverA, Unigene1440\_Mf\_liverA, CL3800.Contig1\_Mf\_liverA, Unigene13525\_Mf\_liverA, Unigene25080\_Mf\_liverA, Unigene28687\_Mf\_liverA, Unigene4938\_Mf\_liverA, CL3055.Contig1\_Mf\_liverA, Unigene29885\_Mf\_liverA, Unigene5758\_Mf\_liverA, CL1810.Contig1\_Mf\_liverA, CL4033.Contig1\_Mf\_liverA, Unigene27483\_Mf\_liverA, Unigene28662\_Mf\_liverA, Unigene24547\_Mf\_liverA, Unigene4781\_Mf\_liverA, Unigene46615\_Mf\_liverA, CL3519.Contig1\_Mf\_liverA, Unigene29426\_Mf\_liverA, Unigene13462\_Mf\_liverA, Unigene28025\_Mf\_liverA, Unigene34446\_Mf\_liverA, CL1493.Contig1\_Mf\_liverA, Unigene11686\_Mf\_liverA, Unigene30585\_Mf\_liverA, CL5764.Contig1\_Mf\_liverA, Unigene39231\_Mf\_liverA, Unigene41226\_Mf\_liverA, Unigene4909\_Mf\_liverA, Unigene34197\_Mf\_liverA, Unigene24471\_Mf\_liverA, Unigene25092\_Mf\_liverA, Unigene29938\_Mf\_liverA, CL725.Contig1\_Mf\_liverA, Unigene30947\_Mf\_liverA, CL1165.Contig2\_Mf\_liverA, Unigene35090\_Mf\_liverA, CL1165.Contig4\_Mf\_liverA, Unigene39011\_Mf\_liverA, Unigene26250\_Mf\_liverA, Unigene20432\_Mf\_liverA, Unigene14330\_Mf\_liverA, Unigene37245\_Mf\_liverA, Unigene16671\_Mf\_liverA, Unigene26580\_Mf\_liverA, Unigene29302\_Mf\_liverA, Unigene5037\_Mf\_liverA, Unigene26336\_Mf\_liverA, Unigene33428\_Mf\_liverA, Unigene5422\_Mf\_liverA, Unigene36699\_Mf\_liverA, Unigene36667\_Mf\_liverA, Unigene18340\_Mf\_liverA, Unigene4983\_Mf\_liverA, CL5698.Contig1\_Mf\_liverA, CL591.Contig1\_Mf\_liverA, Unigene26055\_Mf\_liverA, CL5640.Contig1\_Mf\_liverA, NM\_007896, CL3835.Contig2\_Mf\_liverA, Unigene38831\_Mf\_liverA, Unigene42975\_Mf\_liverA, Unigene29308\_Mf\_liverA, Unigene14603\_Mf\_liverA, CL1362.Contig1\_Mf\_liverA, Unigene8473\_Mf\_liverA, Unigene29008\_Mf\_liverA, Unigene5194\_Mf\_liverA, Unigene19049\_Mf\_liverA, Unigene27593\_Mf\_liverA, Unigene22433\_Mf\_liverA, Unigene37132\_Mf\_liverA, Unigene48792\_Mf\_liverA, Unigene37698\_Mf\_liverA, Unigene14816\_Mf\_liverA, Unigene30878\_Mf\_liverA, Unigene15656\_Mf\_liverA, Unigene34234\_Mf\_liverA, CL6038.Contig2\_Mf\_liverA, Unigene24344\_Mf\_liverA, Unigene36889\_Mf\_liverA, Unigene5204\_Mf\_liverA, CL1256.Contig1\_Mf\_liverA, Unigene24112\_Mf\_liverA, Unigene34789\_Mf\_liverA, Unigene36853\_Mf\_liverA, Unigene38110\_Mf\_liverA, Unigene26194\_Mf\_liverA, Unigene31333\_Mf\_liverA, Unigene4630\_Mf\_liverA, Unigene11\_Mf\_liverA, Unigene4686\_Mf\_liverA, Unigene11544\_Mf\_liverA, CL1493.Contig2\_Mf\_liverA, CL3575.Contig1\_Mf\_liverA, Unigene18117\_Mf\_liverA, Unigene3174\_Mf\_liverA, CL2117.Contig1\_Mf\_liverA, Unigene22432\_Mf\_liverA, Unigene25046\_Mf\_liverA, Unigene36988\_Mf\_liverA, Unigene40824\_Mf\_liverA, Unigene28094\_Mf\_liverA, CL2574.Contig1\_Mf\_liverA, Unigene2745\_Mf\_liverA, Unigene25462\_Mf\_liverA, Unigene16684\_Mf\_liverA, CL4847.Contig1\_Mf\_liverA, Unigene28822\_Mf\_liverA, Unigene20128\_Mf\_liverA, Unigene31988\_Mf\_liverA, Unigene47203\_Mf\_liverA, Unigene33459\_Mf\_liverA, CL848.Contig2\_Mf\_liverA, Unigene23870\_Mf\_liverA, Unigene4768\_Mf\_liverA, Unigene13143\_Mf\_liverA, CL479.Contig1\_Mf\_liverA, Unigene14715\_Mf\_liverA, Unigene34746\_Mf\_liverA, Unigene24503\_Mf\_liverA, Unigene12200\_Mf\_liverA, CL3816.Contig1\_Mf\_liverA, CL2722.Contig1\_Mf\_liverA, CL5631.Contig1\_Mf\_liverA, Unigene20116\_Mf\_liverA, Unigene14896\_Mf\_liverA, Unigene30584\_Mf\_liverA, Unigene24284\_Mf\_liverA, Unigene431\_Mf\_liverA, Unigene26873\_Mf\_liverA, Unigene27260\_Mf\_liverA, NM\_010391, CL795.Contig1\_Mf\_liverA, Unigene27082\_Mf\_liverA, CL5039.Contig2\_Mf\_liverA, CL6039.Contig1\_Mf\_liverA, Unigene26214\_Mf\_liverA, NM\_010380, CL2855.Contig1\_Mf\_liverA, Unigene31206\_Mf\_liverA, Unigene17569\_Mf\_liverA, Unigene5648\_Mf\_liverA, CL4086.Contig1\_Mf\_liverA, CL507.Contig1\_Mf\_liverA, Unigene23082\_Mf\_liverA, Unigene15318\_Mf\_liverA, Unigene13414\_Mf\_liverA, Unigene29823\_Mf\_liverA, Unigene39385\_Mf\_liverA, Unigene15474\_Mf\_liverA, Unigene23448\_Mf\_liverA, Unigene15982\_Mf\_liverA, Unigene30154\_Mf\_liverA, Unigene37526\_Mf\_liverA, Unigene35237\_Mf\_liverA, CL5993.Contig3\_Mf\_liverA, Unigene12907\_Mf\_liverA, Unigene15681\_Mf\_liverA, Unigene7350\_Mf\_liverA, Unigene4703\_Mf\_liverA, Unigene37096\_Mf\_liverA, Unigene5165\_Mf\_liverA, Unigene30707\_Mf\_liverA, CL4583.Contig2\_Mf\_liverA, Unigene36543\_Mf\_liverA, Unigene36328\_Mf\_liverA, CL3002.Contig1\_Mf\_liverA, CL1222.Contig1\_Mf\_liverA, Unigene6895\_Mf\_liverA, CL2327.Contig1\_Mf\_liverA, CL6039.Contig2\_Mf\_liverA, Unigene37139\_Mf\_liverA, NM\_010393, Unigene5906\_Mf\_liverA, CL1662.Contig1\_Mf\_liverA, Unigene28731\_Mf\_liverA, Unigene4597\_Mf\_liverA, Unigene14170\_Mf\_liverA, Unigene24085\_Mf\_liverA, NM\_007986, CL4106.Contig1\_Mf\_liverA, Unigene22330\_Mf\_liverA, Unigene8016\_Mf\_liverA, Unigene5011\_Mf\_liverA, Unigene18796\_Mf\_liverA, Unigene21337\_Mf\_liverA, Unigene51055\_Mf\_liverA, Unigene28873\_Mf\_liverA, Unigene8740\_Mf\_liverA, Unigene560\_Mf\_liverA, Unigene39749\_Mf\_liverA, Unigene25052\_Mf\_liverA, Unigene21843\_Mf\_liverA, Unigene36910\_Mf\_liverA, CL2355.Contig1\_Mf\_liverA, Unigene21562\_Mf\_liverA, Unigene17632\_Mf\_liverA, Unigene5745\_Mf\_liverA, Unigene21336\_Mf\_liverA, Unigene12889\_Mf\_liverA, Unigene38514\_Mf\_liverA, Unigene93\_Mf\_liverA, Unigene30892\_Mf\_liverA, NM\_146007, CL4434.Contig1\_Mf\_liverA, CL3911.Contig2\_Mf\_liverA, Unigene13396\_Mf\_liverA, Unigene14765\_Mf\_liverA, Unigene4681\_Mf\_liverA, CL548.Contig1\_Mf\_liverA, Unigene16891\_Mf\_liverA, Unigene7733\_Mf\_liverA, Unigene37819\_Mf\_liverA, Unigene14284\_Mf\_liverA, Unigene26474\_Mf\_liverA, Unigene5886\_Mf\_liverA |
| plasma membrane | Unigene24506\_Mf\_liverA, NM\_009898, Unigene25721\_Mf\_liverA, Unigene24111\_Mf\_liverA, Unigene28186\_Mf\_liverA, NM\_010378, Unigene29424\_Mf\_liverA, Unigene34218\_Mf\_liverA, Unigene26053\_Mf\_liverA, Unigene31045\_Mf\_liverA, Unigene25090\_Mf\_liverA, Unigene16463\_Mf\_liverA, CL238.Contig1\_Mf\_liverA, Unigene38689\_Mf\_liverA, NM\_010877, Unigene38919\_Mf\_liverA, Unigene1137\_Mf\_liverA, NR\_004446, CL5586.Contig1\_Mf\_liverA, Unigene15703\_Mf\_liverA, Unigene35816\_Mf\_liverA, Unigene28331\_Mf\_liverA, NM\_010233, CL593.Contig2\_Mf\_liverA, Unigene13940\_Mf\_liverA, Unigene5138\_Mf\_liverA, Unigene37150\_Mf\_liverA, Unigene5330\_Mf\_liverA, Unigene23869\_Mf\_liverA, Unigene5552\_Mf\_liverA, Unigene13363\_Mf\_liverA, Unigene26065\_Mf\_liverA, CL2855.Contig2\_Mf\_liverA, Unigene25091\_Mf\_liverA, Unigene18125\_Mf\_liverA, Unigene28226\_Mf\_liverA, Unigene13097\_Mf\_liverA, Unigene25047\_Mf\_liverA, Unigene37278\_Mf\_liverA, Unigene2919\_Mf\_liverA, Unigene21561\_Mf\_liverA, Unigene4922\_Mf\_liverA, Unigene13913\_Mf\_liverA, Unigene30261\_Mf\_liverA, Unigene9475\_Mf\_liverA, Unigene36112\_Mf\_liverA, Unigene9990\_Mf\_liverA, Unigene30459\_Mf\_liverA, Unigene139\_Mf\_liverA, Unigene26941\_Mf\_liverA, Unigene33560\_Mf\_liverA, NM\_172509, NM\_001143689, CL3055.Contig2\_Mf\_liverA, Unigene37575\_Mf\_liverA, Unigene27249\_Mf\_liverA, Unigene43515\_Mf\_liverA, Unigene27547\_Mf\_liverA, Unigene21842\_Mf\_liverA, NM\_134156, Unigene32889\_Mf\_liverA, CL4156.Contig1\_Mf\_liverA, Unigene27248\_Mf\_liverA, Unigene32716\_Mf\_liverA, CL5254.Contig1\_Mf\_liverA, Unigene31852\_Mf\_liverA, Unigene5456\_Mf\_liverA, NM\_009713, Unigene37076\_Mf\_liverA, Unigene2678\_Mf\_liverA, CL1738.Contig1\_Mf\_liverA, Unigene2746\_Mf\_liverA, Unigene5169\_Mf\_liverA, Unigene16465\_Mf\_liverA, Unigene10496\_Mf\_liverA, Unigene28021\_Mf\_liverA, NM\_009933, Unigene31251\_Mf\_liverA, NM\_011503, Unigene40610\_Mf\_liverA, Unigene2162\_Mf\_liverA, CL425.Contig1\_Mf\_liverA, CL3150.Contig1\_Mf\_liverA, Unigene4697\_Mf\_liverA, Unigene37243\_Mf\_liverA, Unigene27026\_Mf\_liverA, Unigene30587\_Mf\_liverA, Unigene39403\_Mf\_liverA, Unigene30039\_Mf\_liverA, Unigene29231\_Mf\_liverA, Unigene5693\_Mf\_liverA, Unigene50250\_Mf\_liverA, CL4057.Contig1\_Mf\_liverA, Unigene1440\_Mf\_liverA, Unigene35958\_Mf\_liverA, CL3800.Contig1\_Mf\_liverA, Unigene13525\_Mf\_liverA, Unigene25080\_Mf\_liverA, Unigene28687\_Mf\_liverA, Unigene4938\_Mf\_liverA, CL3055.Contig1\_Mf\_liverA, Unigene29885\_Mf\_liverA, Unigene5758\_Mf\_liverA, CL1810.Contig1\_Mf\_liverA, CL4033.Contig1\_Mf\_liverA, Unigene27483\_Mf\_liverA, Unigene28662\_Mf\_liverA, Unigene24547\_Mf\_liverA, Unigene4781\_Mf\_liverA, Unigene46615\_Mf\_liverA, CL3519.Contig1\_Mf\_liverA, Unigene29426\_Mf\_liverA, Unigene13462\_Mf\_liverA, Unigene28025\_Mf\_liverA, Unigene34446\_Mf\_liverA, CL1493.Contig1\_Mf\_liverA, Unigene11686\_Mf\_liverA, Unigene30585\_Mf\_liverA, CL5764.Contig1\_Mf\_liverA, Unigene39231\_Mf\_liverA, Unigene41226\_Mf\_liverA, Unigene4909\_Mf\_liverA, Unigene34197\_Mf\_liverA, Unigene24471\_Mf\_liverA, Unigene25092\_Mf\_liverA, Unigene29938\_Mf\_liverA, CL725.Contig1\_Mf\_liverA, Unigene30947\_Mf\_liverA, CL1165.Contig2\_Mf\_liverA, Unigene35090\_Mf\_liverA, CL1165.Contig4\_Mf\_liverA, Unigene39011\_Mf\_liverA, Unigene26250\_Mf\_liverA, Unigene20432\_Mf\_liverA, Unigene14330\_Mf\_liverA, Unigene37245\_Mf\_liverA, Unigene16671\_Mf\_liverA, Unigene26580\_Mf\_liverA, Unigene29302\_Mf\_liverA, Unigene5037\_Mf\_liverA, Unigene26336\_Mf\_liverA, Unigene33428\_Mf\_liverA, Unigene5422\_Mf\_liverA, Unigene36699\_Mf\_liverA, Unigene36667\_Mf\_liverA, Unigene18340\_Mf\_liverA, Unigene4983\_Mf\_liverA, CL5698.Contig1\_Mf\_liverA, CL591.Contig1\_Mf\_liverA, Unigene26055\_Mf\_liverA, CL5640.Contig1\_Mf\_liverA, NM\_007896, CL3835.Contig2\_Mf\_liverA, Unigene38831\_Mf\_liverA, Unigene42975\_Mf\_liverA, Unigene29308\_Mf\_liverA, Unigene14603\_Mf\_liverA, CL1362.Contig1\_Mf\_liverA, Unigene8473\_Mf\_liverA, Unigene29008\_Mf\_liverA, Unigene5194\_Mf\_liverA, Unigene19049\_Mf\_liverA, Unigene27593\_Mf\_liverA, Unigene22433\_Mf\_liverA, Unigene37132\_Mf\_liverA, Unigene48792\_Mf\_liverA, Unigene37698\_Mf\_liverA, Unigene14816\_Mf\_liverA, Unigene30878\_Mf\_liverA, Unigene15656\_Mf\_liverA, Unigene34234\_Mf\_liverA, CL6038.Contig2\_Mf\_liverA, Unigene24344\_Mf\_liverA, Unigene36889\_Mf\_liverA, Unigene5204\_Mf\_liverA, CL1256.Contig1\_Mf\_liverA, Unigene24112\_Mf\_liverA, Unigene34789\_Mf\_liverA, Unigene36853\_Mf\_liverA, Unigene38110\_Mf\_liverA, Unigene26194\_Mf\_liverA, Unigene31333\_Mf\_liverA, Unigene4630\_Mf\_liverA, Unigene11\_Mf\_liverA, Unigene4686\_Mf\_liverA, Unigene11544\_Mf\_liverA, CL1493.Contig2\_Mf\_liverA, CL3575.Contig1\_Mf\_liverA, Unigene18117\_Mf\_liverA, Unigene3174\_Mf\_liverA, CL2117.Contig1\_Mf\_liverA, Unigene22432\_Mf\_liverA, Unigene25046\_Mf\_liverA, Unigene36988\_Mf\_liverA, Unigene40824\_Mf\_liverA, Unigene28094\_Mf\_liverA, CL2574.Contig1\_Mf\_liverA, Unigene2745\_Mf\_liverA, Unigene25462\_Mf\_liverA, Unigene16684\_Mf\_liverA, CL4847.Contig1\_Mf\_liverA, Unigene20128\_Mf\_liverA, Unigene31988\_Mf\_liverA, Unigene47203\_Mf\_liverA, Unigene33459\_Mf\_liverA, Unigene23870\_Mf\_liverA, Unigene13143\_Mf\_liverA, CL479.Contig1\_Mf\_liverA, Unigene34746\_Mf\_liverA, Unigene14715\_Mf\_liverA, Unigene24503\_Mf\_liverA, Unigene12200\_Mf\_liverA, CL3816.Contig1\_Mf\_liverA, CL2722.Contig1\_Mf\_liverA, CL5631.Contig1\_Mf\_liverA, Unigene20116\_Mf\_liverA, Unigene14896\_Mf\_liverA, Unigene30584\_Mf\_liverA, Unigene24284\_Mf\_liverA, Unigene431\_Mf\_liverA, Unigene26873\_Mf\_liverA, Unigene27260\_Mf\_liverA, NM\_010391, CL795.Contig1\_Mf\_liverA, Unigene27082\_Mf\_liverA, CL5039.Contig2\_Mf\_liverA, CL6039.Contig1\_Mf\_liverA, Unigene26214\_Mf\_liverA, NM\_010380, CL2855.Contig1\_Mf\_liverA, Unigene31206\_Mf\_liverA, Unigene17569\_Mf\_liverA, Unigene5648\_Mf\_liverA, CL4086.Contig1\_Mf\_liverA, CL507.Contig1\_Mf\_liverA, Unigene23082\_Mf\_liverA, Unigene15318\_Mf\_liverA, Unigene13414\_Mf\_liverA, Unigene29823\_Mf\_liverA, Unigene39385\_Mf\_liverA, Unigene15474\_Mf\_liverA, Unigene15982\_Mf\_liverA, Unigene30154\_Mf\_liverA, Unigene23448\_Mf\_liverA, Unigene37526\_Mf\_liverA, Unigene35237\_Mf\_liverA, CL5993.Contig3\_Mf\_liverA, Unigene12907\_Mf\_liverA, Unigene15681\_Mf\_liverA, Unigene7350\_Mf\_liverA, Unigene4703\_Mf\_liverA, Unigene37096\_Mf\_liverA, Unigene5165\_Mf\_liverA, Unigene30707\_Mf\_liverA, CL4583.Contig2\_Mf\_liverA, Unigene36543\_Mf\_liverA, Unigene36328\_Mf\_liverA, CL3002.Contig1\_Mf\_liverA, Unigene6895\_Mf\_liverA, CL2327.Contig1\_Mf\_liverA, CL6039.Contig2\_Mf\_liverA, Unigene37139\_Mf\_liverA, NM\_010393, Unigene5906\_Mf\_liverA, CL1662.Contig1\_Mf\_liverA, Unigene28731\_Mf\_liverA, Unigene4597\_Mf\_liverA, Unigene14170\_Mf\_liverA, Unigene24085\_Mf\_liverA, NM\_007986, CL4106.Contig1\_Mf\_liverA, Unigene22330\_Mf\_liverA, Unigene8016\_Mf\_liverA, Unigene5011\_Mf\_liverA, Unigene18796\_Mf\_liverA, Unigene21337\_Mf\_liverA, Unigene51055\_Mf\_liverA, Unigene28873\_Mf\_liverA, Unigene8740\_Mf\_liverA, Unigene560\_Mf\_liverA, Unigene39749\_Mf\_liverA, Unigene25052\_Mf\_liverA, Unigene21843\_Mf\_liverA, Unigene36910\_Mf\_liverA, CL2355.Contig1\_Mf\_liverA, Unigene21562\_Mf\_liverA, Unigene17632\_Mf\_liverA, Unigene5745\_Mf\_liverA, Unigene21336\_Mf\_liverA, Unigene12889\_Mf\_liverA, Unigene38514\_Mf\_liverA, Unigene93\_Mf\_liverA, Unigene30892\_Mf\_liverA, NM\_146007, CL4434.Contig1\_Mf\_liverA, CL3911.Contig2\_Mf\_liverA, Unigene13396\_Mf\_liverA, Unigene14765\_Mf\_liverA, Unigene4681\_Mf\_liverA, CL548.Contig1\_Mf\_liverA, Unigene16891\_Mf\_liverA, Unigene7733\_Mf\_liverA, Unigene37819\_Mf\_liverA, Unigene14284\_Mf\_liverA, Unigene26474\_Mf\_liverA, Unigene5886\_Mf\_liverA |
| extracellular region part | Unigene29399\_Mf\_liverA, CL6018.Contig2\_Mf\_liverA, Unigene5852\_Mf\_liverA, Unigene8106\_Mf\_liverA, CL3669.Contig2\_Mf\_liverA, Unigene7612\_Mf\_liverA, Unigene42975\_Mf\_liverA, Unigene14536\_Mf\_liverA, CL1828.Contig1\_Mf\_liverA, NM\_007737, Unigene7950\_Mf\_liverA, Unigene14603\_Mf\_liverA, Unigene9081\_Mf\_liverA, Unigene15914\_Mf\_liverA, CL482.Contig1\_Mf\_liverA, Unigene19049\_Mf\_liverA, CL81.Contig1\_Mf\_liverA, Unigene29405\_Mf\_liverA, Unigene37698\_Mf\_liverA, Unigene38689\_Mf\_liverA, Unigene34124\_Mf\_liverA, Unigene1137\_Mf\_liverA, Unigene36765\_Mf\_liverA, Unigene34810\_Mf\_liverA, CL6038.Contig2\_Mf\_liverA, Unigene14594\_Mf\_liverA, Unigene17397\_Mf\_liverA, Unigene37099\_Mf\_liverA, Unigene37904\_Mf\_liverA, Unigene30495\_Mf\_liverA, CL5978.Contig3\_Mf\_liverA, Unigene37263\_Mf\_liverA, Unigene38110\_Mf\_liverA, Unigene31333\_Mf\_liverA, NM\_010233, Unigene4630\_Mf\_liverA, CL376.Contig1\_Mf\_liverA, NM\_009776, Unigene11544\_Mf\_liverA, NM\_018780, Unigene18117\_Mf\_liverA, CL3575.Contig1\_Mf\_liverA, Unigene3174\_Mf\_liverA, Unigene17396\_Mf\_liverA, Unigene22978\_Mf\_liverA, Unigene14050\_Mf\_liverA, Unigene23328\_Mf\_liverA, Unigene8054\_Mf\_liverA, CL787.Contig1\_Mf\_liverA, Unigene19623\_Mf\_liverA, Unigene37454\_Mf\_liverA, Unigene37278\_Mf\_liverA, Unigene33459\_Mf\_liverA, Unigene34034\_Mf\_liverA, NM\_008808, Unigene34258\_Mf\_liverA, Unigene13143\_Mf\_liverA, Unigene30261\_Mf\_liverA, Unigene14582\_Mf\_liverA, Unigene45728\_Mf\_liverA, Unigene34746\_Mf\_liverA, Unigene36112\_Mf\_liverA, Unigene941\_Mf\_liverA, Unigene9990\_Mf\_liverA, Unigene27438\_Mf\_liverA, Unigene35494\_Mf\_liverA, Unigene34123\_Mf\_liverA, CL3411.Contig2\_Mf\_liverA, Unigene30584\_Mf\_liverA, CL3669.Contig1\_Mf\_liverA, Unigene27249\_Mf\_liverA, Unigene26521\_Mf\_liverA, Unigene35059\_Mf\_liverA, CL4040.Contig2\_Mf\_liverA, NM\_015734, Unigene425\_Mf\_liverA, Unigene5606\_Mf\_liverA, Unigene22977\_Mf\_liverA, Unigene33271\_Mf\_liverA, Unigene30493\_Mf\_liverA, Unigene27248\_Mf\_liverA, Unigene32716\_Mf\_liverA, Unigene13296\_Mf\_liverA, Unigene37729\_Mf\_liverA, Unigene8132\_Mf\_liverA, Unigene29940\_Mf\_liverA, Unigene8246\_Mf\_liverA, CL3232.Contig1\_Mf\_liverA, Unigene24157\_Mf\_liverA, Unigene32058\_Mf\_liverA, NM\_009713, CL6018.Contig1\_Mf\_liverA, Unigene32791\_Mf\_liverA, Unigene23055\_Mf\_liverA, CL442.Contig2\_Mf\_liverA, CL1347.Contig3\_Mf\_liverA, CL6039.Contig1\_Mf\_liverA, Unigene17569\_Mf\_liverA, Unigene32059\_Mf\_liverA, NM\_009933, CL4086.Contig1\_Mf\_liverA, CL1811.Contig2\_Mf\_liverA, Unigene15318\_Mf\_liverA, Unigene22980\_Mf\_liverA, Unigene30815\_Mf\_liverA, Unigene10351\_Mf\_liverA, Unigene15982\_Mf\_liverA, Unigene30587\_Mf\_liverA, Unigene35237\_Mf\_liverA, Unigene5501\_Mf\_liverA, Unigene37259\_Mf\_liverA, Unigene30039\_Mf\_liverA, Unigene12230\_Mf\_liverA, Unigene29231\_Mf\_liverA, Unigene4985\_Mf\_liverA, CL3122.Contig2\_Mf\_liverA, Unigene35958\_Mf\_liverA, CL4583.Contig2\_Mf\_liverA, Unigene13525\_Mf\_liverA, Unigene13894\_Mf\_liverA, Unigene36328\_Mf\_liverA, NM\_008871, CL6039.Contig2\_Mf\_liverA, Unigene22979\_Mf\_liverA, Unigene28662\_Mf\_liverA, Unigene8033\_Mf\_liverA, Unigene29426\_Mf\_liverA, Unigene30494\_Mf\_liverA, Unigene1212\_Mf\_liverA, Unigene30585\_Mf\_liverA, NM\_033325, Unigene13106\_Mf\_liverA, Unigene29938\_Mf\_liverA, Unigene15529\_Mf\_liverA, Unigene33832\_Mf\_liverA, CL725.Contig1\_Mf\_liverA, Unigene13616\_Mf\_liverA, Unigene21337\_Mf\_liverA, CL1190.Contig3\_Mf\_liverA, CL2251.Contig1\_Mf\_liverA, CL5978.Contig2\_Mf\_liverA, Unigene25595\_Mf\_liverA, Unigene35090\_Mf\_liverA, Unigene560\_Mf\_liverA, NM\_011580, Unigene36643\_Mf\_liverA, Unigene26083\_Mf\_liverA, Unigene36644\_Mf\_liverA, Unigene21693\_Mf\_liverA, Unigene677\_Mf\_liverA, Unigene15592\_Mf\_liverA, Unigene21336\_Mf\_liverA, Unigene41583\_Mf\_liverA, Unigene30814\_Mf\_liverA, Unigene43068\_Mf\_liverA, Unigene2195\_Mf\_liverA, Unigene36667\_Mf\_liverA, Unigene18340\_Mf\_liverA, CL3911.Contig2\_Mf\_liverA, Unigene18510\_Mf\_liverA, Unigene30786\_Mf\_liverA, NM\_009636, Unigene38387\_Mf\_liverA, Unigene36426\_Mf\_liverA, Unigene31492\_Mf\_liverA |
| external side of plasma membrane | Unigene24506\_Mf\_liverA, Unigene4909\_Mf\_liverA, Unigene5552\_Mf\_liverA, Unigene24471\_Mf\_liverA, CL4086.Contig1\_Mf\_liverA, Unigene40610\_Mf\_liverA, CL3835.Contig2\_Mf\_liverA, Unigene29938\_Mf\_liverA, Unigene26065\_Mf\_liverA, CL2855.Contig2\_Mf\_liverA, Unigene20128\_Mf\_liverA, Unigene18125\_Mf\_liverA, CL725.Contig1\_Mf\_liverA, Unigene37278\_Mf\_liverA, Unigene24111\_Mf\_liverA, Unigene28186\_Mf\_liverA, Unigene39385\_Mf\_liverA, Unigene21561\_Mf\_liverA, Unigene13143\_Mf\_liverA, Unigene15982\_Mf\_liverA, Unigene27593\_Mf\_liverA, Unigene22433\_Mf\_liverA, Unigene30587\_Mf\_liverA, Unigene34746\_Mf\_liverA, Unigene48792\_Mf\_liverA, Unigene24503\_Mf\_liverA, Unigene14816\_Mf\_liverA, Unigene38689\_Mf\_liverA, Unigene39749\_Mf\_liverA, Unigene1137\_Mf\_liverA, Unigene35958\_Mf\_liverA, Unigene30584\_Mf\_liverA, CL4583.Contig2\_Mf\_liverA, Unigene37245\_Mf\_liverA, Unigene21843\_Mf\_liverA, Unigene36889\_Mf\_liverA, Unigene21842\_Mf\_liverA, CL3002.Contig1\_Mf\_liverA, Unigene6895\_Mf\_liverA, Unigene24112\_Mf\_liverA, CL4156.Contig1\_Mf\_liverA, Unigene21562\_Mf\_liverA, Unigene5422\_Mf\_liverA, Unigene38110\_Mf\_liverA, Unigene4630\_Mf\_liverA, NM\_009713, Unigene11544\_Mf\_liverA, Unigene4983\_Mf\_liverA, Unigene22432\_Mf\_liverA, CL2855.Contig1\_Mf\_liverA, Unigene30585\_Mf\_liverA, Unigene24085\_Mf\_liverA |
| cytoplasm | Unigene34656\_Mf\_liverA, NM\_009898, Unigene7612\_Mf\_liverA, Unigene7950\_Mf\_liverA, Unigene28186\_Mf\_liverA, Unigene36622\_Mf\_liverA, NM\_010378, Unigene33138\_Mf\_liverA, Unigene16463\_Mf\_liverA, Unigene39886\_Mf\_liverA, CL33.Contig4\_Mf\_liverA, Unigene15064\_Mf\_liverA, Unigene34124\_Mf\_liverA, Unigene7510\_Mf\_liverA, Unigene24252\_Mf\_liverA, CL4757.Contig1\_Mf\_liverA, NR\_004446, Unigene36765\_Mf\_liverA, Unigene4557\_Mf\_liverA, Unigene30495\_Mf\_liverA, Unigene2\_Mf\_liverA, Unigene28331\_Mf\_liverA, CL33.Contig3\_Mf\_liverA, NM\_010233, Unigene13379\_Mf\_liverA, Unigene25594\_Mf\_liverA, Unigene779\_Mf\_liverA, Unigene14050\_Mf\_liverA, Unigene21857\_Mf\_liverA, Unigene8054\_Mf\_liverA, Unigene37454\_Mf\_liverA, Unigene743\_Mf\_liverA, Unigene36417\_Mf\_liverA, Unigene25091\_Mf\_liverA, NM\_177093, Unigene25047\_Mf\_liverA, Unigene7476\_Mf\_liverA, CL4332.Contig1\_Mf\_liverA, Unigene25976\_Mf\_liverA, Unigene30261\_Mf\_liverA, Unigene9475\_Mf\_liverA, NM\_145942, Unigene27895\_Mf\_liverA, Unigene36112\_Mf\_liverA, Unigene36641\_Mf\_liverA, Unigene13945\_Mf\_liverA, Unigene139\_Mf\_liverA, Unigene26941\_Mf\_liverA, Unigene995\_Mf\_liverA, Unigene34123\_Mf\_liverA, Unigene10756\_Mf\_liverA, CL3669.Contig1\_Mf\_liverA, Unigene27249\_Mf\_liverA, Unigene43515\_Mf\_liverA, Unigene11097\_Mf\_liverA, NM\_134156, Unigene4720\_Mf\_liverA, Unigene37389\_Mf\_liverA, Unigene27248\_Mf\_liverA, Unigene29253\_Mf\_liverA, Unigene34010\_Mf\_liverA, Unigene152\_Mf\_liverA, Unigene31852\_Mf\_liverA, Unigene37076\_Mf\_liverA, Unigene2678\_Mf\_liverA, Unigene1280\_Mf\_liverA, Unigene30288\_Mf\_liverA, CL1738.Contig1\_Mf\_liverA, CL3339.Contig1\_Mf\_liverA, Unigene2746\_Mf\_liverA, Unigene5236\_Mf\_liverA, Unigene28021\_Mf\_liverA, Unigene45904\_Mf\_liverA, Unigene37433\_Mf\_liverA, CL5528.Contig1\_Mf\_liverA, CL425.Contig1\_Mf\_liverA, CL2797.Contig2\_Mf\_liverA, Unigene37243\_Mf\_liverA, Unigene30587\_Mf\_liverA, Unigene5693\_Mf\_liverA, Unigene39970\_Mf\_liverA, CL3393.Contig1\_Mf\_liverA, Unigene13271\_Mf\_liverA, Unigene19821\_Mf\_liverA, Unigene29876\_Mf\_liverA, CL3800.Contig1\_Mf\_liverA, Unigene25080\_Mf\_liverA, Unigene28687\_Mf\_liverA, CL1988.Contig3\_Mf\_liverA, Unigene29885\_Mf\_liverA, CL3055.Contig1\_Mf\_liverA, NM\_028785, NM\_009609, CL1810.Contig1\_Mf\_liverA, CL4033.Contig1\_Mf\_liverA, Unigene24547\_Mf\_liverA, CL3519.Contig1\_Mf\_liverA, Unigene8033\_Mf\_liverA, CL1493.Contig1\_Mf\_liverA, CL3685.Contig1\_Mf\_liverA, Unigene39231\_Mf\_liverA, Unigene4909\_Mf\_liverA, Unigene34197\_Mf\_liverA, Unigene24471\_Mf\_liverA, NM\_026823, Unigene35168\_Mf\_liverA, Unigene15888\_Mf\_liverA, NM\_010162, Unigene15529\_Mf\_liverA, Unigene37262\_Mf\_liverA, Unigene12908\_Mf\_liverA, CL2251.Contig1\_Mf\_liverA, CL4490.Contig2\_Mf\_liverA, CL5293.Contig1\_Mf\_liverA, Unigene25595\_Mf\_liverA, Unigene14263\_Mf\_liverA, Unigene14286\_Mf\_liverA, Unigene39011\_Mf\_liverA, Unigene386\_Mf\_liverA, Unigene26250\_Mf\_liverA, Unigene30795\_Mf\_liverA, Unigene20432\_Mf\_liverA, Unigene48460\_Mf\_liverA, NM\_011580, Unigene35046\_Mf\_liverA, Unigene28459\_Mf\_liverA, Unigene37245\_Mf\_liverA, Unigene16671\_Mf\_liverA, Unigene21693\_Mf\_liverA, Unigene13535\_Mf\_liverA, Unigene13772\_Mf\_liverA, Unigene29302\_Mf\_liverA, Unigene33428\_Mf\_liverA, Unigene37574\_Mf\_liverA, Unigene36176\_Mf\_liverA, Unigene36699\_Mf\_liverA, Unigene18340\_Mf\_liverA, Unigene30429\_Mf\_liverA, Unigene30412\_Mf\_liverA, Unigene38317\_Mf\_liverA, Unigene5512\_Mf\_liverA, CL373.Contig7\_Mf\_liverA, Unigene29889\_Mf\_liverA, Unigene2939\_Mf\_liverA, Unigene36728\_Mf\_liverA, Unigene9466\_Mf\_liverA, Unigene35884\_Mf\_liverA, NM\_020559, CL5640.Contig1\_Mf\_liverA, Unigene28142\_Mf\_liverA, CL3835.Contig2\_Mf\_liverA, Unigene8473\_Mf\_liverA, Unigene29008\_Mf\_liverA, Unigene19049\_Mf\_liverA, Unigene33508\_Mf\_liverA, NM\_011072, Unigene21466\_Mf\_liverA, Unigene30303\_Mf\_liverA, Unigene34810\_Mf\_liverA, Unigene27420\_Mf\_liverA, CL6038.Contig2\_Mf\_liverA, Unigene13307\_Mf\_liverA, Unigene37999\_Mf\_liverA, Unigene40020\_Mf\_liverA, Unigene37904\_Mf\_liverA, CL3483.Contig1\_Mf\_liverA, Unigene34789\_Mf\_liverA, CL4141.Contig1\_Mf\_liverA, Unigene25057\_Mf\_liverA, CL5978.Contig3\_Mf\_liverA, CL5191.Contig2\_Mf\_liverA, Unigene13658\_Mf\_liverA, NM\_175260, Unigene4686\_Mf\_liverA, Unigene11\_Mf\_liverA, Unigene11544\_Mf\_liverA, Unigene18117\_Mf\_liverA, CL1493.Contig2\_Mf\_liverA, CL2117.Contig1\_Mf\_liverA, CL3565.Contig1\_Mf\_liverA, Unigene36988\_Mf\_liverA, Unigene28094\_Mf\_liverA, Unigene40824\_Mf\_liverA, CL2574.Contig1\_Mf\_liverA, Unigene38104\_Mf\_liverA, Unigene23328\_Mf\_liverA, Unigene25462\_Mf\_liverA, Unigene36626\_Mf\_liverA, Unigene28822\_Mf\_liverA, Unigene36691\_Mf\_liverA, NM\_019703, Unigene19297\_Mf\_liverA, Unigene151\_Mf\_liverA, CL848.Contig2\_Mf\_liverA, Unigene23870\_Mf\_liverA, NM\_008808, Unigene4768\_Mf\_liverA, CL3198.Contig1\_Mf\_liverA, Unigene25662\_Mf\_liverA, Unigene37460\_Mf\_liverA, Unigene31517\_Mf\_liverA, Unigene36628\_Mf\_liverA, Unigene31231\_Mf\_liverA, Unigene24323\_Mf\_liverA, NM\_008538, NM\_008610, CL3816.Contig1\_Mf\_liverA, Unigene36631\_Mf\_liverA, CL3803.Contig2\_Mf\_liverA, Unigene4510\_Mf\_liverA, Unigene24284\_Mf\_liverA, Unigene26873\_Mf\_liverA, Unigene27260\_Mf\_liverA, CL3268.Contig1\_Mf\_liverA, Unigene36845\_Mf\_liverA, Unigene37729\_Mf\_liverA, Unigene29940\_Mf\_liverA, CL795.Contig1\_Mf\_liverA, Unigene42812\_Mf\_liverA, Unigene5509\_Mf\_liverA, Unigene5325\_Mf\_liverA, Unigene1205\_Mf\_liverA, Unigene13841\_Mf\_liverA, CL1352.Contig1\_Mf\_liverA, CL6039.Contig1\_Mf\_liverA, Unigene24804\_Mf\_liverA, Unigene31206\_Mf\_liverA, Unigene40924\_Mf\_liverA, NM\_009128, Unigene7412\_Mf\_liverA, CL2240.Contig1\_Mf\_liverA, Unigene22980\_Mf\_liverA, Unigene15474\_Mf\_liverA, CL1125.Contig1\_Mf\_liverA, Unigene29510\_Mf\_liverA, Unigene15681\_Mf\_liverA, Unigene7350\_Mf\_liverA, Unigene19083\_Mf\_liverA, Unigene673\_Mf\_liverA, Unigene4703\_Mf\_liverA, Unigene37096\_Mf\_liverA, Unigene5165\_Mf\_liverA, Unigene30707\_Mf\_liverA, NM\_133838, Unigene7674\_Mf\_liverA, CL3002.Contig1\_Mf\_liverA, NM\_008871, Unigene15058\_Mf\_liverA, Unigene35491\_Mf\_liverA, Unigene5906\_Mf\_liverA, CL5688.Contig1\_Mf\_liverA, CL2791.Contig1\_Mf\_liverA, Unigene35269\_Mf\_liverA, Unigene10135\_Mf\_liverA, Unigene22330\_Mf\_liverA, Unigene5954\_Mf\_liverA, CL4127.Contig1\_Mf\_liverA, Unigene1327\_Mf\_liverA, Unigene33526\_Mf\_liverA, CL1190.Contig3\_Mf\_liverA, Unigene8740\_Mf\_liverA, CL5978.Contig2\_Mf\_liverA, NM\_008776, Unigene36177\_Mf\_liverA, Unigene24566\_Mf\_liverA, NM\_007478, Unigene15170\_Mf\_liverA, Unigene560\_Mf\_liverA, Unigene24477\_Mf\_liverA, Unigene30731\_Mf\_liverA, Unigene30983\_Mf\_liverA, Unigene36418\_Mf\_liverA, Unigene550\_Mf\_liverA, Unigene38311\_Mf\_liverA, CL2355.Contig1\_Mf\_liverA, Unigene36910\_Mf\_liverA, Unigene28314\_Mf\_liverA, Unigene37153\_Mf\_liverA, Unigene21562\_Mf\_liverA, Unigene15592\_Mf\_liverA, NM\_015767, Unigene37711\_Mf\_liverA, Unigene41583\_Mf\_liverA, Unigene5418\_Mf\_liverA, Unigene2195\_Mf\_liverA, Unigene93\_Mf\_liverA, Unigene30892\_Mf\_liverA, Unigene803\_Mf\_liverA, CL3911.Contig2\_Mf\_liverA, Unigene34866\_Mf\_liverA, Unigene31885\_Mf\_liverA, Unigene4681\_Mf\_liverA, CL548.Contig1\_Mf\_liverA, NM\_007392, Unigene24506\_Mf\_liverA, Unigene34609\_Mf\_liverA, Unigene25721\_Mf\_liverA, CL3669.Contig2\_Mf\_liverA, Unigene5852\_Mf\_liverA, Unigene36172\_Mf\_liverA, Unigene36851\_Mf\_liverA, Unigene7936\_Mf\_liverA, Unigene29424\_Mf\_liverA, Unigene26053\_Mf\_liverA, CL4105.Contig1\_Mf\_liverA, Unigene34218\_Mf\_liverA, Unigene25090\_Mf\_liverA, CL238.Contig1\_Mf\_liverA, CL2423.Contig1\_Mf\_liverA, CL5560.Contig1\_Mf\_liverA, Unigene22575\_Mf\_liverA, Unigene785\_Mf\_liverA, Unigene26422\_Mf\_liverA, Unigene29334\_Mf\_liverA, NM\_010877, NM\_021273, Unigene38919\_Mf\_liverA, CL5586.Contig1\_Mf\_liverA, Unigene21255\_Mf\_liverA, Unigene34341\_Mf\_liverA, Unigene30142\_Mf\_liverA, Unigene35816\_Mf\_liverA, Unigene13940\_Mf\_liverA, Unigene33054\_Mf\_liverA, NM\_009776, Unigene15205\_Mf\_liverA, Unigene5138\_Mf\_liverA, NM\_153193, Unigene13266\_Mf\_liverA, Unigene5330\_Mf\_liverA, NM\_177320, Unigene653\_Mf\_liverA, Unigene38657\_Mf\_liverA, Unigene23869\_Mf\_liverA, Unigene9150\_Mf\_liverA, Unigene5598\_Mf\_liverA, Unigene13363\_Mf\_liverA, CL2855.Contig2\_Mf\_liverA, Unigene36175\_Mf\_liverA, Unigene28226\_Mf\_liverA, Unigene44317\_Mf\_liverA, Unigene37278\_Mf\_liverA, Unigene21561\_Mf\_liverA, Unigene1479\_Mf\_liverA, Unigene4922\_Mf\_liverA, CL2159.Contig2\_Mf\_liverA, NM\_153505, Unigene37662\_Mf\_liverA, Unigene9990\_Mf\_liverA, Unigene4944\_Mf\_liverA, CL3055.Contig2\_Mf\_liverA, Unigene37575\_Mf\_liverA, Unigene25398\_Mf\_liverA, Unigene27547\_Mf\_liverA, CL1736.Contig2\_Mf\_liverA, Unigene5180\_Mf\_liverA, Unigene12909\_Mf\_liverA, Unigene32332\_Mf\_liverA, Unigene37470\_Mf\_liverA, CL5254.Contig1\_Mf\_liverA, Unigene37542\_Mf\_liverA, NM\_009713, Unigene35169\_Mf\_liverA, Unigene21256\_Mf\_liverA, Unigene16465\_Mf\_liverA, Unigene32059\_Mf\_liverA, Unigene5472\_Mf\_liverA, Unigene26515\_Mf\_liverA, Unigene31251\_Mf\_liverA, NM\_011503, CL186.Contig3\_Mf\_liverA, CL4736.Contig1\_Mf\_liverA, Unigene14916\_Mf\_liverA, Unigene30808\_Mf\_liverA, Unigene37442\_Mf\_liverA, Unigene50250\_Mf\_liverA, Unigene1440\_Mf\_liverA, Unigene35958\_Mf\_liverA, Unigene43037\_Mf\_liverA, Unigene13525\_Mf\_liverA, Unigene5815\_Mf\_liverA, Unigene1129\_Mf\_liverA, Unigene33168\_Mf\_liverA, Unigene27483\_Mf\_liverA, Unigene28662\_Mf\_liverA, CL3738.Contig1\_Mf\_liverA, CL2439.Contig1\_Mf\_liverA, Unigene4781\_Mf\_liverA, Unigene46615\_Mf\_liverA, Unigene30494\_Mf\_liverA, CL5049.Contig2\_Mf\_liverA, Unigene34446\_Mf\_liverA, Unigene13683\_Mf\_liverA, Unigene9698\_Mf\_liverA, Unigene24755\_Mf\_liverA, Unigene28499\_Mf\_liverA, Unigene30585\_Mf\_liverA, Unigene36420\_Mf\_liverA, Unigene542\_Mf\_liverA, Unigene38593\_Mf\_liverA, Unigene24758\_Mf\_liverA, Unigene25092\_Mf\_liverA, CL5828.Contig2\_Mf\_liverA, Unigene28527\_Mf\_liverA, Unigene35039\_Mf\_liverA, Unigene29938\_Mf\_liverA, CL2625.Contig2\_Mf\_liverA, CL725.Contig1\_Mf\_liverA, CL5293.Contig2\_Mf\_liverA, CL1165.Contig2\_Mf\_liverA, Unigene10335\_Mf\_liverA, Unigene34962\_Mf\_liverA, Unigene14212\_Mf\_liverA, CL1165.Contig4\_Mf\_liverA, CL4669.Contig1\_Mf\_liverA, NM\_028222, Unigene29558\_Mf\_liverA, Unigene23185\_Mf\_liverA, CL3692.Contig2\_Mf\_liverA, Unigene26083\_Mf\_liverA, NM\_013863, Unigene281\_Mf\_liverA, Unigene10313\_Mf\_liverA, Unigene29985\_Mf\_liverA, Unigene5687\_Mf\_liverA, CL3104.Contig1\_Mf\_liverA, Unigene36593\_Mf\_liverA, Unigene5037\_Mf\_liverA, Unigene5422\_Mf\_liverA, Unigene36673\_Mf\_liverA, CL5698.Contig1\_Mf\_liverA, Unigene4556\_Mf\_liverA, Unigene38237\_Mf\_liverA, Unigene26055\_Mf\_liverA, Unigene29399\_Mf\_liverA, Unigene38015\_Mf\_liverA, NM\_007896, Unigene24713\_Mf\_liverA, Unigene42975\_Mf\_liverA, CL1828.Contig1\_Mf\_liverA, Unigene29308\_Mf\_liverA, CL1362.Contig1\_Mf\_liverA, CL523.Contig1\_Mf\_liverA, Unigene15914\_Mf\_liverA, Unigene5194\_Mf\_liverA, CL1063.Contig1\_Mf\_liverA, CL482.Contig1\_Mf\_liverA, Unigene29405\_Mf\_liverA, Unigene32882\_Mf\_liverA, Unigene2633\_Mf\_liverA, Unigene30878\_Mf\_liverA, Unigene40289\_Mf\_liverA, Unigene21013\_Mf\_liverA, Unigene15656\_Mf\_liverA, Unigene34234\_Mf\_liverA, Unigene38331\_Mf\_liverA, Unigene5204\_Mf\_liverA, Unigene37099\_Mf\_liverA, Unigene25070\_Mf\_liverA, Unigene37263\_Mf\_liverA, Unigene36853\_Mf\_liverA, NM\_009896, CL1119.Contig1\_Mf\_liverA, Unigene26194\_Mf\_liverA, Unigene25596\_Mf\_liverA, Unigene3174\_Mf\_liverA, Unigene22978\_Mf\_liverA, Unigene25046\_Mf\_liverA, Unigene2745\_Mf\_liverA, NM\_001024205, CL4076.Contig1\_Mf\_liverA, Unigene1162\_Mf\_liverA, CL787.Contig1\_Mf\_liverA, Unigene25524\_Mf\_liverA, CL4847.Contig1\_Mf\_liverA, CL3196.Contig2\_Mf\_liverA, CL3750.Contig2\_Mf\_liverA, CL4722.Contig1\_Mf\_liverA, Unigene33459\_Mf\_liverA, Unigene26398\_Mf\_liverA, Unigene25012\_Mf\_liverA, Unigene34258\_Mf\_liverA, Unigene5632\_Mf\_liverA, CL479.Contig1\_Mf\_liverA, Unigene27419\_Mf\_liverA, Unigene14715\_Mf\_liverA, Unigene24503\_Mf\_liverA, Unigene37180\_Mf\_liverA, Unigene4523\_Mf\_liverA, CL2722.Contig1\_Mf\_liverA, Unigene27438\_Mf\_liverA, CL5631.Contig1\_Mf\_liverA, Unigene31427\_Mf\_liverA, Unigene14896\_Mf\_liverA, Unigene30584\_Mf\_liverA, Unigene19885\_Mf\_liverA, Unigene431\_Mf\_liverA, Unigene36510\_Mf\_liverA, NM\_009091, Unigene44992\_Mf\_liverA, Unigene10820\_Mf\_liverA, NM\_010391, Unigene21317\_Mf\_liverA, NM\_010227, Unigene30493\_Mf\_liverA, Unigene8132\_Mf\_liverA, Unigene27082\_Mf\_liverA, Unigene37178\_Mf\_liverA, Unigene32058\_Mf\_liverA, Unigene5712\_Mf\_liverA, CL442.Contig2\_Mf\_liverA, Unigene26214\_Mf\_liverA, NM\_033374, CL2855.Contig1\_Mf\_liverA, CL4411.Contig4\_Mf\_liverA, NM\_001025388, Unigene13233\_Mf\_liverA, Unigene5648\_Mf\_liverA, NM\_011099, NM\_009447, CL1537.Contig1\_Mf\_liverA, NM\_010481, Unigene392\_Mf\_liverA, CL4086.Contig1\_Mf\_liverA, CL507.Contig1\_Mf\_liverA, Unigene23082\_Mf\_liverA, Unigene5360\_Mf\_liverA, Unigene15318\_Mf\_liverA, NM\_009178, Unigene13414\_Mf\_liverA, NM\_008293, Unigene30154\_Mf\_liverA, Unigene1130\_Mf\_liverA, Unigene38208\_Mf\_liverA, CL4162.Contig1\_Mf\_liverA, Unigene35237\_Mf\_liverA, CL5993.Contig3\_Mf\_liverA, Unigene37259\_Mf\_liverA, Unigene12907\_Mf\_liverA, Unigene5260\_Mf\_liverA, Unigene15588\_Mf\_liverA, Unigene23013\_Mf\_liverA, CL4583.Contig2\_Mf\_liverA, Unigene36414\_Mf\_liverA, Unigene5175\_Mf\_liverA, Unigene33880\_Mf\_liverA, Unigene13894\_Mf\_liverA, Unigene36328\_Mf\_liverA, CL1222.Contig1\_Mf\_liverA, CL2327.Contig1\_Mf\_liverA, CL6039.Contig2\_Mf\_liverA, Unigene35037\_Mf\_liverA, Unigene36710\_Mf\_liverA, NM\_007393, Unigene28731\_Mf\_liverA, Unigene1212\_Mf\_liverA, Unigene24085\_Mf\_liverA, NM\_009706, CL3549.Contig1\_Mf\_liverA, CL4106.Contig1\_Mf\_liverA, CL3750.Contig1\_Mf\_liverA, CL1588.Contig3\_Mf\_liverA, Unigene8016\_Mf\_liverA, Unigene18796\_Mf\_liverA, CL5307.Contig1\_Mf\_liverA, Unigene51055\_Mf\_liverA, Unigene31730\_Mf\_liverA, Unigene4540\_Mf\_liverA, Unigene33525\_Mf\_liverA, Unigene24204\_Mf\_liverA, Unigene9406\_Mf\_liverA, Unigene15026\_Mf\_liverA, Unigene1221\_Mf\_liverA, CL1588.Contig1\_Mf\_liverA, Unigene39875\_Mf\_liverA, NM\_001081274, Unigene25238\_Mf\_liverA, Unigene17632\_Mf\_liverA, Unigene5745\_Mf\_liverA, Unigene12889\_Mf\_liverA, Unigene38514\_Mf\_liverA, Unigene665\_Mf\_liverA, Unigene35678\_Mf\_liverA, Unigene8560\_Mf\_liverA, CL4434.Contig1\_Mf\_liverA, Unigene13396\_Mf\_liverA, Unigene21359\_Mf\_liverA, Unigene36487\_Mf\_liverA, Unigene37474\_Mf\_liverA, Unigene16891\_Mf\_liverA, Unigene7733\_Mf\_liverA, Unigene37819\_Mf\_liverA, Unigene5886\_Mf\_liverA, Unigene27274\_Mf\_liverA, Unigene15493\_Mf\_liverA |
| cell surface | Unigene24506\_Mf\_liverA, Unigene29399\_Mf\_liverA, CL4086.Contig1\_Mf\_liverA, Unigene40610\_Mf\_liverA, CL3835.Contig2\_Mf\_liverA, Unigene8106\_Mf\_liverA, Unigene2162\_Mf\_liverA, Unigene24111\_Mf\_liverA, Unigene28186\_Mf\_liverA, Unigene39385\_Mf\_liverA, NM\_010378, Unigene29424\_Mf\_liverA, Unigene4697\_Mf\_liverA, Unigene15982\_Mf\_liverA, Unigene27593\_Mf\_liverA, Unigene27026\_Mf\_liverA, Unigene22433\_Mf\_liverA, Unigene30587\_Mf\_liverA, Unigene48792\_Mf\_liverA, Unigene30039\_Mf\_liverA, Unigene29231\_Mf\_liverA, Unigene14816\_Mf\_liverA, Unigene38689\_Mf\_liverA, Unigene1137\_Mf\_liverA, Unigene35958\_Mf\_liverA, NR\_004446, CL4583.Contig2\_Mf\_liverA, Unigene13525\_Mf\_liverA, NM\_011352, Unigene36889\_Mf\_liverA, Unigene5204\_Mf\_liverA, CL3002.Contig1\_Mf\_liverA, Unigene37904\_Mf\_liverA, Unigene6895\_Mf\_liverA, Unigene24112\_Mf\_liverA, Unigene38110\_Mf\_liverA, Unigene28331\_Mf\_liverA, Unigene31333\_Mf\_liverA, CL3519.Contig1\_Mf\_liverA, Unigene4630\_Mf\_liverA, Unigene11\_Mf\_liverA, NM\_010393, Unigene29426\_Mf\_liverA, Unigene11544\_Mf\_liverA, Unigene13462\_Mf\_liverA, Unigene37150\_Mf\_liverA, Unigene22432\_Mf\_liverA, Unigene30585\_Mf\_liverA, Unigene24085\_Mf\_liverA, Unigene4909\_Mf\_liverA, Unigene5552\_Mf\_liverA, Unigene24471\_Mf\_liverA, Unigene26065\_Mf\_liverA, Unigene29938\_Mf\_liverA, CL2855.Contig2\_Mf\_liverA, Unigene20128\_Mf\_liverA, Unigene18125\_Mf\_liverA, CL725.Contig1\_Mf\_liverA, Unigene37278\_Mf\_liverA, Unigene21561\_Mf\_liverA, Unigene1479\_Mf\_liverA, Unigene13143\_Mf\_liverA, Unigene34746\_Mf\_liverA, Unigene14582\_Mf\_liverA, Unigene24503\_Mf\_liverA, Unigene26250\_Mf\_liverA, NM\_011580, NM\_001143689, Unigene39749\_Mf\_liverA, Unigene27249\_Mf\_liverA, Unigene30584\_Mf\_liverA, Unigene35046\_Mf\_liverA, Unigene37245\_Mf\_liverA, Unigene21843\_Mf\_liverA, Unigene21842\_Mf\_liverA, NM\_010391, Unigene36845\_Mf\_liverA, Unigene33271\_Mf\_liverA, CL4156.Contig1\_Mf\_liverA, Unigene26580\_Mf\_liverA, Unigene21562\_Mf\_liverA, Unigene27248\_Mf\_liverA, Unigene5422\_Mf\_liverA, NM\_009713, Unigene4983\_Mf\_liverA, NM\_010380, Unigene14284\_Mf\_liverA, CL2855.Contig1\_Mf\_liverA |
| cytosol | Unigene35884\_Mf\_liverA, CL5640.Contig1\_Mf\_liverA, Unigene38015\_Mf\_liverA, Unigene24713\_Mf\_liverA, Unigene36172\_Mf\_liverA, Unigene36622\_Mf\_liverA, Unigene36851\_Mf\_liverA, Unigene29308\_Mf\_liverA, Unigene7936\_Mf\_liverA, Unigene34218\_Mf\_liverA, CL4105.Contig1\_Mf\_liverA, CL1063.Contig1\_Mf\_liverA, Unigene25090\_Mf\_liverA, Unigene32882\_Mf\_liverA, Unigene2633\_Mf\_liverA, Unigene39886\_Mf\_liverA, CL33.Contig4\_Mf\_liverA, Unigene29334\_Mf\_liverA, Unigene15064\_Mf\_liverA, CL4757.Contig1\_Mf\_liverA, Unigene13307\_Mf\_liverA, Unigene37099\_Mf\_liverA, Unigene40020\_Mf\_liverA, Unigene25070\_Mf\_liverA, CL4141.Contig1\_Mf\_liverA, Unigene25057\_Mf\_liverA, CL5191.Contig2\_Mf\_liverA, CL1119.Contig1\_Mf\_liverA, Unigene35816\_Mf\_liverA, Unigene26194\_Mf\_liverA, Unigene13658\_Mf\_liverA, Unigene28331\_Mf\_liverA, CL33.Contig3\_Mf\_liverA, Unigene13940\_Mf\_liverA, Unigene25596\_Mf\_liverA, Unigene15205\_Mf\_liverA, Unigene5138\_Mf\_liverA, Unigene779\_Mf\_liverA, Unigene14050\_Mf\_liverA, Unigene25046\_Mf\_liverA, Unigene36988\_Mf\_liverA, Unigene40824\_Mf\_liverA, Unigene38657\_Mf\_liverA, Unigene2745\_Mf\_liverA, Unigene25091\_Mf\_liverA, CL3196.Contig2\_Mf\_liverA, Unigene25047\_Mf\_liverA, Unigene151\_Mf\_liverA, CL848.Contig2\_Mf\_liverA, Unigene21561\_Mf\_liverA, CL3198.Contig1\_Mf\_liverA, CL2159.Contig2\_Mf\_liverA, Unigene30261\_Mf\_liverA, Unigene31231\_Mf\_liverA, Unigene37662\_Mf\_liverA, Unigene24323\_Mf\_liverA, Unigene13945\_Mf\_liverA, Unigene37180\_Mf\_liverA, CL5631.Contig1\_Mf\_liverA, Unigene31427\_Mf\_liverA, Unigene43515\_Mf\_liverA, Unigene431\_Mf\_liverA, Unigene24284\_Mf\_liverA, Unigene19885\_Mf\_liverA, Unigene5180\_Mf\_liverA, Unigene12909\_Mf\_liverA, Unigene27260\_Mf\_liverA, CL3268.Contig1\_Mf\_liverA, Unigene21317\_Mf\_liverA, CL5254.Contig1\_Mf\_liverA, Unigene37542\_Mf\_liverA, Unigene42812\_Mf\_liverA, Unigene152\_Mf\_liverA, Unigene37178\_Mf\_liverA, Unigene27082\_Mf\_liverA, Unigene31852\_Mf\_liverA, Unigene37076\_Mf\_liverA, Unigene5325\_Mf\_liverA, Unigene30288\_Mf\_liverA, CL442.Contig2\_Mf\_liverA, CL6039.Contig1\_Mf\_liverA, Unigene2746\_Mf\_liverA, Unigene24804\_Mf\_liverA, Unigene45904\_Mf\_liverA, Unigene37433\_Mf\_liverA, Unigene5472\_Mf\_liverA, Unigene31251\_Mf\_liverA, CL507.Contig1\_Mf\_liverA, CL186.Contig3\_Mf\_liverA, Unigene30154\_Mf\_liverA, Unigene38208\_Mf\_liverA, Unigene35237\_Mf\_liverA, Unigene30808\_Mf\_liverA, Unigene37259\_Mf\_liverA, Unigene15681\_Mf\_liverA, Unigene5693\_Mf\_liverA, Unigene7350\_Mf\_liverA, Unigene5260\_Mf\_liverA, Unigene13271\_Mf\_liverA, Unigene37096\_Mf\_liverA, Unigene19821\_Mf\_liverA, Unigene5165\_Mf\_liverA, Unigene1440\_Mf\_liverA, CL3800.Contig1\_Mf\_liverA, CL4583.Contig2\_Mf\_liverA, Unigene25080\_Mf\_liverA, Unigene28687\_Mf\_liverA, Unigene5815\_Mf\_liverA, CL1222.Contig1\_Mf\_liverA, Unigene29885\_Mf\_liverA, Unigene1129\_Mf\_liverA, CL1810.Contig1\_Mf\_liverA, CL6039.Contig2\_Mf\_liverA, Unigene24547\_Mf\_liverA, CL3738.Contig1\_Mf\_liverA, Unigene35037\_Mf\_liverA, Unigene36710\_Mf\_liverA, CL5049.Contig2\_Mf\_liverA, Unigene34446\_Mf\_liverA, CL1493.Contig1\_Mf\_liverA, Unigene1212\_Mf\_liverA, Unigene24755\_Mf\_liverA, Unigene28499\_Mf\_liverA, CL3685.Contig1\_Mf\_liverA, CL3549.Contig1\_Mf\_liverA, Unigene24085\_Mf\_liverA, Unigene24758\_Mf\_liverA, Unigene24471\_Mf\_liverA, Unigene28527\_Mf\_liverA, Unigene35039\_Mf\_liverA, Unigene5954\_Mf\_liverA, CL2625.Contig2\_Mf\_liverA, Unigene37262\_Mf\_liverA, Unigene51055\_Mf\_liverA, Unigene12908\_Mf\_liverA, CL1190.Contig3\_Mf\_liverA, CL2251.Contig1\_Mf\_liverA, Unigene8740\_Mf\_liverA, Unigene10335\_Mf\_liverA, Unigene4540\_Mf\_liverA, Unigene34962\_Mf\_liverA, Unigene26250\_Mf\_liverA, Unigene24477\_Mf\_liverA, CL4669.Contig1\_Mf\_liverA, Unigene30983\_Mf\_liverA, Unigene48460\_Mf\_liverA, Unigene15026\_Mf\_liverA, Unigene38311\_Mf\_liverA, Unigene39875\_Mf\_liverA, Unigene550\_Mf\_liverA, Unigene16671\_Mf\_liverA, CL2355.Contig1\_Mf\_liverA, Unigene37153\_Mf\_liverA, Unigene29302\_Mf\_liverA, CL3104.Contig1\_Mf\_liverA, Unigene21562\_Mf\_liverA, Unigene37711\_Mf\_liverA, Unigene12889\_Mf\_liverA, Unigene38514\_Mf\_liverA, Unigene665\_Mf\_liverA, Unigene36673\_Mf\_liverA, CL4434.Contig1\_Mf\_liverA, Unigene13396\_Mf\_liverA, CL5698.Contig1\_Mf\_liverA, Unigene16891\_Mf\_liverA, Unigene4556\_Mf\_liverA, Unigene2939\_Mf\_liverA, Unigene27274\_Mf\_liverA |
| proteinaceous extracellular matrix | NM\_009933, Unigene29399\_Mf\_liverA, Unigene13106\_Mf\_liverA, Unigene7612\_Mf\_liverA, CL1828.Contig1\_Mf\_liverA, NM\_007737, Unigene13616\_Mf\_liverA, Unigene22980\_Mf\_liverA, Unigene34258\_Mf\_liverA, Unigene15914\_Mf\_liverA, Unigene30261\_Mf\_liverA, Unigene29405\_Mf\_liverA, Unigene30587\_Mf\_liverA, Unigene14582\_Mf\_liverA, Unigene36112\_Mf\_liverA, Unigene5501\_Mf\_liverA, Unigene35090\_Mf\_liverA, Unigene560\_Mf\_liverA, Unigene12230\_Mf\_liverA, Unigene4985\_Mf\_liverA, Unigene34123\_Mf\_liverA, CL3122.Contig2\_Mf\_liverA, Unigene34124\_Mf\_liverA, CL3411.Contig2\_Mf\_liverA, Unigene30584\_Mf\_liverA, Unigene34810\_Mf\_liverA, Unigene35059\_Mf\_liverA, CL4040.Contig2\_Mf\_liverA, NM\_015734, Unigene5606\_Mf\_liverA, Unigene17397\_Mf\_liverA, Unigene37904\_Mf\_liverA, Unigene21693\_Mf\_liverA, Unigene22977\_Mf\_liverA, Unigene33271\_Mf\_liverA, Unigene30493\_Mf\_liverA, Unigene22979\_Mf\_liverA, Unigene32716\_Mf\_liverA, Unigene30495\_Mf\_liverA, Unigene37729\_Mf\_liverA, Unigene37263\_Mf\_liverA, Unigene8132\_Mf\_liverA, Unigene29940\_Mf\_liverA, Unigene8246\_Mf\_liverA, Unigene43068\_Mf\_liverA, CL3232.Contig1\_Mf\_liverA, NM\_010233, CL376.Contig1\_Mf\_liverA, Unigene30494\_Mf\_liverA, Unigene18510\_Mf\_liverA, Unigene18117\_Mf\_liverA, Unigene32791\_Mf\_liverA, Unigene3174\_Mf\_liverA, Unigene17396\_Mf\_liverA, Unigene23055\_Mf\_liverA, Unigene22978\_Mf\_liverA, Unigene30585\_Mf\_liverA, Unigene8054\_Mf\_liverA, Unigene23328\_Mf\_liverA, Unigene31492\_Mf\_liverA |
| fibrillar collagen | Unigene30494\_Mf\_liverA, Unigene5501\_Mf\_liverA, Unigene21693\_Mf\_liverA, Unigene7612\_Mf\_liverA, Unigene22977\_Mf\_liverA, Unigene22978\_Mf\_liverA, NM\_007737, Unigene30493\_Mf\_liverA, Unigene22979\_Mf\_liverA, Unigene30495\_Mf\_liverA, Unigene22980\_Mf\_liverA, Unigene23328\_Mf\_liverA, Unigene37729\_Mf\_liverA, Unigene15914\_Mf\_liverA |
| extracellular matrix | NM\_009933, Unigene29399\_Mf\_liverA, Unigene7612\_Mf\_liverA, NM\_007743, CL1828.Contig1\_Mf\_liverA, NM\_007737, Unigene22980\_Mf\_liverA, Unigene15914\_Mf\_liverA, Unigene19049\_Mf\_liverA, Unigene29405\_Mf\_liverA, Unigene30587\_Mf\_liverA, Unigene5501\_Mf\_liverA, Unigene12230\_Mf\_liverA, Unigene4985\_Mf\_liverA, CL3122.Contig2\_Mf\_liverA, Unigene34124\_Mf\_liverA, Unigene34810\_Mf\_liverA, Unigene24344\_Mf\_liverA, Unigene17397\_Mf\_liverA, Unigene37904\_Mf\_liverA, Unigene22979\_Mf\_liverA, Unigene30495\_Mf\_liverA, Unigene12843\_Mf\_liverA, Unigene37263\_Mf\_liverA, NM\_010233, CL376.Contig1\_Mf\_liverA, Unigene30494\_Mf\_liverA, Unigene18117\_Mf\_liverA, Unigene3174\_Mf\_liverA, Unigene17396\_Mf\_liverA, Unigene22978\_Mf\_liverA, NM\_153107, Unigene30585\_Mf\_liverA, Unigene23328\_Mf\_liverA, Unigene8054\_Mf\_liverA, Unigene13106\_Mf\_liverA, Unigene13616\_Mf\_liverA, CL1190.Contig3\_Mf\_liverA, Unigene34258\_Mf\_liverA, Unigene30261\_Mf\_liverA, Unigene14582\_Mf\_liverA, Unigene36112\_Mf\_liverA, NM\_008610, Unigene35090\_Mf\_liverA, Unigene560\_Mf\_liverA, Unigene34123\_Mf\_liverA, Unigene26521\_Mf\_liverA, Unigene30584\_Mf\_liverA, CL3411.Contig2\_Mf\_liverA, Unigene35059\_Mf\_liverA, CL4040.Contig2\_Mf\_liverA, NM\_015734, Unigene5606\_Mf\_liverA, Unigene425\_Mf\_liverA, Unigene21693\_Mf\_liverA, Unigene22977\_Mf\_liverA, Unigene33271\_Mf\_liverA, Unigene30493\_Mf\_liverA, Unigene32716\_Mf\_liverA, NM\_175643, Unigene8132\_Mf\_liverA, Unigene37729\_Mf\_liverA, Unigene43068\_Mf\_liverA, Unigene8246\_Mf\_liverA, Unigene29940\_Mf\_liverA, Unigene41583\_Mf\_liverA, CL3232.Contig1\_Mf\_liverA, NM\_146007, Unigene18510\_Mf\_liverA, Unigene32791\_Mf\_liverA, Unigene23055\_Mf\_liverA, Unigene31492\_Mf\_liverA, Unigene28021\_Mf\_liverA |
| collagen | Unigene35059\_Mf\_liverA, CL4040.Contig2\_Mf\_liverA, Unigene13106\_Mf\_liverA, NM\_015734, Unigene5606\_Mf\_liverA, Unigene17397\_Mf\_liverA, Unigene21693\_Mf\_liverA, Unigene7612\_Mf\_liverA, Unigene22977\_Mf\_liverA, NM\_007737, Unigene30493\_Mf\_liverA, Unigene22979\_Mf\_liverA, Unigene32716\_Mf\_liverA, Unigene30495\_Mf\_liverA, Unigene22980\_Mf\_liverA, Unigene37729\_Mf\_liverA, Unigene43068\_Mf\_liverA, Unigene15914\_Mf\_liverA, Unigene30494\_Mf\_liverA, Unigene5501\_Mf\_liverA, Unigene32791\_Mf\_liverA, Unigene35090\_Mf\_liverA, Unigene17396\_Mf\_liverA, Unigene22978\_Mf\_liverA, Unigene23328\_Mf\_liverA, Unigene34123\_Mf\_liverA, Unigene34124\_Mf\_liverA |
| collagen type I | Unigene30494\_Mf\_liverA, Unigene5501\_Mf\_liverA, Unigene21693\_Mf\_liverA, Unigene22977\_Mf\_liverA, Unigene22978\_Mf\_liverA, Unigene30493\_Mf\_liverA, Unigene22979\_Mf\_liverA, Unigene30495\_Mf\_liverA, Unigene22980\_Mf\_liverA, Unigene37729\_Mf\_liverA, Unigene15914\_Mf\_liverA |
| lysosome | Unigene25524\_Mf\_liverA, Unigene24471\_Mf\_liverA, NM\_026823, NM\_011503, CL3835.Contig2\_Mf\_liverA, CL2855.Contig2\_Mf\_liverA, CL3669.Contig2\_Mf\_liverA, Unigene15529\_Mf\_liverA, Unigene28226\_Mf\_liverA, CL725.Contig1\_Mf\_liverA, Unigene13414\_Mf\_liverA, Unigene36622\_Mf\_liverA, Unigene8740\_Mf\_liverA, Unigene1130\_Mf\_liverA, Unigene14715\_Mf\_liverA, Unigene36112\_Mf\_liverA, Unigene560\_Mf\_liverA, Unigene37259\_Mf\_liverA, Unigene39886\_Mf\_liverA, Unigene21466\_Mf\_liverA, Unigene30878\_Mf\_liverA, Unigene38919\_Mf\_liverA, Unigene35958\_Mf\_liverA, CL3055.Contig2\_Mf\_liverA, Unigene37575\_Mf\_liverA, Unigene29558\_Mf\_liverA, CL3669.Contig1\_Mf\_liverA, CL4583.Contig2\_Mf\_liverA, CL1736.Contig2\_Mf\_liverA, Unigene36328\_Mf\_liverA, Unigene5204\_Mf\_liverA, CL3002.Contig1\_Mf\_liverA, Unigene36910\_Mf\_liverA, Unigene27260\_Mf\_liverA, CL3055.Contig1\_Mf\_liverA, Unigene37389\_Mf\_liverA, Unigene32332\_Mf\_liverA, Unigene15592\_Mf\_liverA, Unigene5745\_Mf\_liverA, Unigene8132\_Mf\_liverA, Unigene5422\_Mf\_liverA, Unigene29940\_Mf\_liverA, Unigene46615\_Mf\_liverA, Unigene27082\_Mf\_liverA, Unigene5906\_Mf\_liverA, CL1493.Contig2\_Mf\_liverA, CL442.Contig2\_Mf\_liverA, Unigene4681\_Mf\_liverA, CL1493.Contig1\_Mf\_liverA, Unigene21857\_Mf\_liverA, CL2855.Contig1\_Mf\_liverA, Unigene5236\_Mf\_liverA, Unigene24085\_Mf\_liverA |
| extracellular matrix part | Unigene13106\_Mf\_liverA, Unigene7612\_Mf\_liverA, NM\_007737, Unigene22980\_Mf\_liverA, Unigene34258\_Mf\_liverA, Unigene15914\_Mf\_liverA, Unigene14582\_Mf\_liverA, Unigene5501\_Mf\_liverA, Unigene35090\_Mf\_liverA, Unigene560\_Mf\_liverA, Unigene12230\_Mf\_liverA, Unigene4985\_Mf\_liverA, Unigene34123\_Mf\_liverA, Unigene34124\_Mf\_liverA, CL3411.Contig2\_Mf\_liverA, Unigene35059\_Mf\_liverA, CL4040.Contig2\_Mf\_liverA, NM\_015734, Unigene17397\_Mf\_liverA, Unigene5606\_Mf\_liverA, Unigene21693\_Mf\_liverA, Unigene22977\_Mf\_liverA, Unigene33271\_Mf\_liverA, Unigene30493\_Mf\_liverA, Unigene32716\_Mf\_liverA, Unigene22979\_Mf\_liverA, Unigene30495\_Mf\_liverA, Unigene37729\_Mf\_liverA, Unigene8132\_Mf\_liverA, Unigene43068\_Mf\_liverA, CL3232.Contig1\_Mf\_liverA, CL376.Contig1\_Mf\_liverA, Unigene18510\_Mf\_liverA, Unigene30494\_Mf\_liverA, Unigene32791\_Mf\_liverA, Unigene17396\_Mf\_liverA, Unigene23055\_Mf\_liverA, Unigene22978\_Mf\_liverA, Unigene23328\_Mf\_liverA, Unigene31492\_Mf\_liverA |
| plasma membrane part | Unigene24506\_Mf\_liverA, NM\_007896, NM\_009898, CL3835.Contig2\_Mf\_liverA, Unigene42975\_Mf\_liverA, Unigene24111\_Mf\_liverA, Unigene28186\_Mf\_liverA, CL1362.Contig1\_Mf\_liverA, NM\_010378, Unigene29424\_Mf\_liverA, Unigene34218\_Mf\_liverA, Unigene5194\_Mf\_liverA, Unigene29008\_Mf\_liverA, Unigene27593\_Mf\_liverA, Unigene22433\_Mf\_liverA, Unigene25090\_Mf\_liverA, CL238.Contig1\_Mf\_liverA, Unigene48792\_Mf\_liverA, Unigene14816\_Mf\_liverA, Unigene38689\_Mf\_liverA, Unigene30878\_Mf\_liverA, NM\_010877, Unigene38919\_Mf\_liverA, Unigene1137\_Mf\_liverA, Unigene15656\_Mf\_liverA, NR\_004446, Unigene24344\_Mf\_liverA, CL5586.Contig1\_Mf\_liverA, Unigene36889\_Mf\_liverA, Unigene5204\_Mf\_liverA, CL1256.Contig1\_Mf\_liverA, Unigene34789\_Mf\_liverA, Unigene24112\_Mf\_liverA, Unigene15703\_Mf\_liverA, Unigene38110\_Mf\_liverA, Unigene35816\_Mf\_liverA, Unigene28331\_Mf\_liverA, NM\_010233, Unigene4686\_Mf\_liverA, Unigene4630\_Mf\_liverA, Unigene11544\_Mf\_liverA, Unigene5138\_Mf\_liverA, CL3575.Contig1\_Mf\_liverA, CL2117.Contig1\_Mf\_liverA, Unigene22432\_Mf\_liverA, Unigene5330\_Mf\_liverA, Unigene28094\_Mf\_liverA, Unigene40824\_Mf\_liverA, Unigene16684\_Mf\_liverA, Unigene23869\_Mf\_liverA, Unigene5552\_Mf\_liverA, CL4847.Contig1\_Mf\_liverA, Unigene13363\_Mf\_liverA, Unigene26065\_Mf\_liverA, CL2855.Contig2\_Mf\_liverA, Unigene20128\_Mf\_liverA, Unigene25091\_Mf\_liverA, Unigene47203\_Mf\_liverA, Unigene28226\_Mf\_liverA, Unigene18125\_Mf\_liverA, Unigene37278\_Mf\_liverA, Unigene2919\_Mf\_liverA, Unigene21561\_Mf\_liverA, Unigene23870\_Mf\_liverA, Unigene13913\_Mf\_liverA, Unigene13143\_Mf\_liverA, CL479.Contig1\_Mf\_liverA, Unigene9475\_Mf\_liverA, Unigene34746\_Mf\_liverA, Unigene36112\_Mf\_liverA, Unigene24503\_Mf\_liverA, Unigene139\_Mf\_liverA, Unigene26941\_Mf\_liverA, NM\_172509, NM\_001143689, CL5631.Contig1\_Mf\_liverA, Unigene37575\_Mf\_liverA, Unigene30584\_Mf\_liverA, Unigene43515\_Mf\_liverA, Unigene431\_Mf\_liverA, Unigene24284\_Mf\_liverA, Unigene21842\_Mf\_liverA, Unigene27260\_Mf\_liverA, NM\_134156, NM\_010391, CL4156.Contig1\_Mf\_liverA, CL795.Contig1\_Mf\_liverA, NM\_009713, Unigene27082\_Mf\_liverA, Unigene31852\_Mf\_liverA, Unigene2678\_Mf\_liverA, CL5039.Contig2\_Mf\_liverA, CL1738.Contig1\_Mf\_liverA, NM\_010380, Unigene26214\_Mf\_liverA, CL2855.Contig1\_Mf\_liverA, Unigene5169\_Mf\_liverA, Unigene17569\_Mf\_liverA, Unigene28021\_Mf\_liverA, CL4086.Contig1\_Mf\_liverA, NM\_011503, Unigene40610\_Mf\_liverA, CL507.Contig1\_Mf\_liverA, Unigene2162\_Mf\_liverA, Unigene39385\_Mf\_liverA, CL425.Contig1\_Mf\_liverA, CL3150.Contig1\_Mf\_liverA, Unigene15474\_Mf\_liverA, Unigene15982\_Mf\_liverA, Unigene37243\_Mf\_liverA, Unigene30587\_Mf\_liverA, Unigene35237\_Mf\_liverA, CL5993.Contig3\_Mf\_liverA, Unigene30039\_Mf\_liverA, Unigene29231\_Mf\_liverA, Unigene5693\_Mf\_liverA, Unigene7350\_Mf\_liverA, Unigene50250\_Mf\_liverA, Unigene1440\_Mf\_liverA, Unigene35958\_Mf\_liverA, Unigene30707\_Mf\_liverA, CL4583.Contig2\_Mf\_liverA, Unigene13525\_Mf\_liverA, Unigene25080\_Mf\_liverA, Unigene4938\_Mf\_liverA, Unigene36543\_Mf\_liverA, Unigene36328\_Mf\_liverA, CL3002.Contig1\_Mf\_liverA, Unigene6895\_Mf\_liverA, CL1810.Contig1\_Mf\_liverA, Unigene5758\_Mf\_liverA, CL4033.Contig1\_Mf\_liverA, Unigene27483\_Mf\_liverA, Unigene4781\_Mf\_liverA, Unigene37139\_Mf\_liverA, CL3519.Contig1\_Mf\_liverA, NM\_010393, Unigene29426\_Mf\_liverA, Unigene13462\_Mf\_liverA, Unigene5906\_Mf\_liverA, Unigene28025\_Mf\_liverA, Unigene4597\_Mf\_liverA, Unigene30585\_Mf\_liverA, Unigene24085\_Mf\_liverA, NM\_007986, Unigene41226\_Mf\_liverA, Unigene4909\_Mf\_liverA, Unigene34197\_Mf\_liverA, Unigene24471\_Mf\_liverA, Unigene29938\_Mf\_liverA, CL725.Contig1\_Mf\_liverA, Unigene30947\_Mf\_liverA, Unigene21337\_Mf\_liverA, Unigene51055\_Mf\_liverA, Unigene28873\_Mf\_liverA, Unigene8740\_Mf\_liverA, Unigene35090\_Mf\_liverA, Unigene39011\_Mf\_liverA, Unigene14330\_Mf\_liverA, Unigene39749\_Mf\_liverA, Unigene25052\_Mf\_liverA, Unigene37245\_Mf\_liverA, Unigene21843\_Mf\_liverA, Unigene16671\_Mf\_liverA, Unigene26580\_Mf\_liverA, Unigene29302\_Mf\_liverA, Unigene21562\_Mf\_liverA, Unigene17632\_Mf\_liverA, Unigene26336\_Mf\_liverA, Unigene21336\_Mf\_liverA, Unigene5422\_Mf\_liverA, Unigene12889\_Mf\_liverA, Unigene36667\_Mf\_liverA, Unigene30892\_Mf\_liverA, CL4434.Contig1\_Mf\_liverA, Unigene18340\_Mf\_liverA, Unigene14765\_Mf\_liverA, Unigene4983\_Mf\_liverA, Unigene4681\_Mf\_liverA, CL591.Contig1\_Mf\_liverA, Unigene14284\_Mf\_liverA, Unigene26474\_Mf\_liverA |
| cytoplasmic part | Unigene24506\_Mf\_liverA, Unigene34656\_Mf\_liverA, NM\_009898, Unigene25721\_Mf\_liverA, Unigene5852\_Mf\_liverA, CL3669.Contig2\_Mf\_liverA, Unigene7612\_Mf\_liverA, Unigene36172\_Mf\_liverA, Unigene28186\_Mf\_liverA, Unigene36622\_Mf\_liverA, Unigene36851\_Mf\_liverA, NM\_010378, Unigene7936\_Mf\_liverA, Unigene29424\_Mf\_liverA, Unigene34218\_Mf\_liverA, CL4105.Contig1\_Mf\_liverA, Unigene26053\_Mf\_liverA, Unigene33138\_Mf\_liverA, Unigene25090\_Mf\_liverA, CL2423.Contig1\_Mf\_liverA, CL5560.Contig1\_Mf\_liverA, Unigene22575\_Mf\_liverA, Unigene39886\_Mf\_liverA, Unigene26422\_Mf\_liverA, CL33.Contig4\_Mf\_liverA, Unigene29334\_Mf\_liverA, Unigene15064\_Mf\_liverA, NM\_010877, NM\_021273, Unigene34124\_Mf\_liverA, Unigene38919\_Mf\_liverA, Unigene7510\_Mf\_liverA, Unigene24252\_Mf\_liverA, CL4757.Contig1\_Mf\_liverA, NR\_004446, Unigene36765\_Mf\_liverA, CL5586.Contig1\_Mf\_liverA, Unigene4557\_Mf\_liverA, Unigene21255\_Mf\_liverA, Unigene34341\_Mf\_liverA, Unigene30142\_Mf\_liverA, Unigene2\_Mf\_liverA, Unigene35816\_Mf\_liverA, Unigene28331\_Mf\_liverA, CL33.Contig3\_Mf\_liverA, NM\_010233, Unigene13940\_Mf\_liverA, Unigene13379\_Mf\_liverA, NM\_009776, Unigene13266\_Mf\_liverA, NM\_153193, Unigene25594\_Mf\_liverA, Unigene5138\_Mf\_liverA, Unigene15205\_Mf\_liverA, Unigene779\_Mf\_liverA, Unigene14050\_Mf\_liverA, Unigene5330\_Mf\_liverA, Unigene21857\_Mf\_liverA, NM\_177320, Unigene38657\_Mf\_liverA, Unigene8054\_Mf\_liverA, Unigene5598\_Mf\_liverA, Unigene13363\_Mf\_liverA, Unigene743\_Mf\_liverA, CL2855.Contig2\_Mf\_liverA, Unigene25091\_Mf\_liverA, Unigene28226\_Mf\_liverA, NM\_177093, Unigene44317\_Mf\_liverA, Unigene25047\_Mf\_liverA, Unigene21561\_Mf\_liverA, Unigene7476\_Mf\_liverA, Unigene1479\_Mf\_liverA, Unigene4922\_Mf\_liverA, NM\_153505, CL2159.Contig2\_Mf\_liverA, Unigene30261\_Mf\_liverA, Unigene9475\_Mf\_liverA, NM\_145942, Unigene37662\_Mf\_liverA, Unigene27895\_Mf\_liverA, Unigene36112\_Mf\_liverA, Unigene36641\_Mf\_liverA, Unigene9990\_Mf\_liverA, Unigene13945\_Mf\_liverA, Unigene139\_Mf\_liverA, Unigene26941\_Mf\_liverA, Unigene995\_Mf\_liverA, Unigene34123\_Mf\_liverA, Unigene37575\_Mf\_liverA, CL3055.Contig2\_Mf\_liverA, CL3669.Contig1\_Mf\_liverA, Unigene27249\_Mf\_liverA, Unigene43515\_Mf\_liverA, Unigene25398\_Mf\_liverA, Unigene27547\_Mf\_liverA, CL1736.Contig2\_Mf\_liverA, Unigene11097\_Mf\_liverA, Unigene5180\_Mf\_liverA, Unigene12909\_Mf\_liverA, NM\_134156, Unigene4720\_Mf\_liverA, Unigene37389\_Mf\_liverA, Unigene32332\_Mf\_liverA, Unigene27248\_Mf\_liverA, CL5254.Contig1\_Mf\_liverA, Unigene37542\_Mf\_liverA, Unigene29253\_Mf\_liverA, Unigene34010\_Mf\_liverA, Unigene152\_Mf\_liverA, Unigene31852\_Mf\_liverA, NM\_009713, Unigene37076\_Mf\_liverA, Unigene2678\_Mf\_liverA, Unigene1280\_Mf\_liverA, Unigene21256\_Mf\_liverA, Unigene30288\_Mf\_liverA, CL1738.Contig1\_Mf\_liverA, CL3339.Contig1\_Mf\_liverA, Unigene2746\_Mf\_liverA, Unigene5236\_Mf\_liverA, Unigene32059\_Mf\_liverA, Unigene28021\_Mf\_liverA, Unigene45904\_Mf\_liverA, Unigene37433\_Mf\_liverA, Unigene5472\_Mf\_liverA, Unigene31251\_Mf\_liverA, NM\_011503, CL5528.Contig1\_Mf\_liverA, CL186.Contig3\_Mf\_liverA, CL425.Contig1\_Mf\_liverA, CL2797.Contig2\_Mf\_liverA, Unigene14916\_Mf\_liverA, Unigene37243\_Mf\_liverA, Unigene30587\_Mf\_liverA, Unigene30808\_Mf\_liverA, Unigene5693\_Mf\_liverA, Unigene50250\_Mf\_liverA, Unigene13271\_Mf\_liverA, Unigene19821\_Mf\_liverA, Unigene35958\_Mf\_liverA, Unigene1440\_Mf\_liverA, Unigene43037\_Mf\_liverA, Unigene29876\_Mf\_liverA, CL3800.Contig1\_Mf\_liverA, Unigene13525\_Mf\_liverA, Unigene25080\_Mf\_liverA, Unigene28687\_Mf\_liverA, Unigene5815\_Mf\_liverA, CL1988.Contig3\_Mf\_liverA, Unigene1129\_Mf\_liverA, Unigene29885\_Mf\_liverA, CL3055.Contig1\_Mf\_liverA, NM\_028785, NM\_009609, CL1810.Contig1\_Mf\_liverA, CL4033.Contig1\_Mf\_liverA, CL3738.Contig1\_Mf\_liverA, Unigene24547\_Mf\_liverA, Unigene28662\_Mf\_liverA, Unigene4781\_Mf\_liverA, Unigene46615\_Mf\_liverA, Unigene8033\_Mf\_liverA, CL5049.Contig2\_Mf\_liverA, Unigene34446\_Mf\_liverA, CL1493.Contig1\_Mf\_liverA, Unigene13683\_Mf\_liverA, Unigene24755\_Mf\_liverA, Unigene28499\_Mf\_liverA, CL3685.Contig1\_Mf\_liverA, Unigene30585\_Mf\_liverA, Unigene542\_Mf\_liverA, Unigene24758\_Mf\_liverA, Unigene4909\_Mf\_liverA, Unigene34197\_Mf\_liverA, Unigene24471\_Mf\_liverA, NM\_026823, CL5828.Contig2\_Mf\_liverA, Unigene28527\_Mf\_liverA, Unigene35039\_Mf\_liverA, Unigene15888\_Mf\_liverA, NM\_010162, Unigene15529\_Mf\_liverA, CL2625.Contig2\_Mf\_liverA, CL725.Contig1\_Mf\_liverA, Unigene37262\_Mf\_liverA, Unigene12908\_Mf\_liverA, CL2251.Contig1\_Mf\_liverA, Unigene10335\_Mf\_liverA, CL4490.Contig2\_Mf\_liverA, Unigene25595\_Mf\_liverA, Unigene14286\_Mf\_liverA, Unigene34962\_Mf\_liverA, Unigene39011\_Mf\_liverA, CL4669.Contig1\_Mf\_liverA, Unigene26250\_Mf\_liverA, Unigene30795\_Mf\_liverA, Unigene48460\_Mf\_liverA, NM\_028222, NM\_011580, Unigene23185\_Mf\_liverA, Unigene29558\_Mf\_liverA, CL3692.Contig2\_Mf\_liverA, Unigene35046\_Mf\_liverA, Unigene28459\_Mf\_liverA, NM\_013863, Unigene37245\_Mf\_liverA, Unigene16671\_Mf\_liverA, Unigene281\_Mf\_liverA, Unigene13535\_Mf\_liverA, Unigene10313\_Mf\_liverA, Unigene13772\_Mf\_liverA, CL3104.Contig1\_Mf\_liverA, Unigene29302\_Mf\_liverA, Unigene5687\_Mf\_liverA, Unigene36593\_Mf\_liverA, Unigene33428\_Mf\_liverA, Unigene5422\_Mf\_liverA, Unigene37574\_Mf\_liverA, Unigene36699\_Mf\_liverA, Unigene36673\_Mf\_liverA, CL5698.Contig1\_Mf\_liverA, Unigene5512\_Mf\_liverA, Unigene4556\_Mf\_liverA, Unigene38237\_Mf\_liverA, Unigene2939\_Mf\_liverA, Unigene36728\_Mf\_liverA, Unigene26055\_Mf\_liverA, Unigene29399\_Mf\_liverA, Unigene35884\_Mf\_liverA, NM\_020559, CL5640.Contig1\_Mf\_liverA, Unigene38015\_Mf\_liverA, NM\_007896, CL3835.Contig2\_Mf\_liverA, Unigene24713\_Mf\_liverA, CL1828.Contig1\_Mf\_liverA, Unigene29308\_Mf\_liverA, CL523.Contig1\_Mf\_liverA, Unigene29008\_Mf\_liverA, Unigene5194\_Mf\_liverA, CL1063.Contig1\_Mf\_liverA, CL482.Contig1\_Mf\_liverA, Unigene19049\_Mf\_liverA, Unigene29405\_Mf\_liverA, Unigene32882\_Mf\_liverA, NM\_011072, Unigene2633\_Mf\_liverA, Unigene21466\_Mf\_liverA, Unigene30303\_Mf\_liverA, Unigene30878\_Mf\_liverA, Unigene40289\_Mf\_liverA, Unigene15656\_Mf\_liverA, Unigene34234\_Mf\_liverA, Unigene34810\_Mf\_liverA, CL6038.Contig2\_Mf\_liverA, Unigene38331\_Mf\_liverA, Unigene5204\_Mf\_liverA, Unigene13307\_Mf\_liverA, Unigene37099\_Mf\_liverA, Unigene37999\_Mf\_liverA, Unigene40020\_Mf\_liverA, Unigene25070\_Mf\_liverA, Unigene34789\_Mf\_liverA, CL4141.Contig1\_Mf\_liverA, Unigene25057\_Mf\_liverA, CL5978.Contig3\_Mf\_liverA, CL5191.Contig2\_Mf\_liverA, Unigene37263\_Mf\_liverA, NM\_009896, CL1119.Contig1\_Mf\_liverA, Unigene26194\_Mf\_liverA, Unigene13658\_Mf\_liverA, NM\_175260, Unigene4686\_Mf\_liverA, Unigene11\_Mf\_liverA, Unigene11544\_Mf\_liverA, Unigene25596\_Mf\_liverA, Unigene18117\_Mf\_liverA, CL1493.Contig2\_Mf\_liverA, Unigene3174\_Mf\_liverA, CL2117.Contig1\_Mf\_liverA, Unigene22978\_Mf\_liverA, Unigene25046\_Mf\_liverA, Unigene36988\_Mf\_liverA, Unigene28094\_Mf\_liverA, Unigene40824\_Mf\_liverA, CL2574.Contig1\_Mf\_liverA, Unigene38104\_Mf\_liverA, Unigene23328\_Mf\_liverA, Unigene2745\_Mf\_liverA, NM\_001024205, Unigene25462\_Mf\_liverA, Unigene36626\_Mf\_liverA, CL787.Contig1\_Mf\_liverA, Unigene1162\_Mf\_liverA, Unigene25524\_Mf\_liverA, CL4847.Contig1\_Mf\_liverA, Unigene28822\_Mf\_liverA, Unigene36691\_Mf\_liverA, CL3196.Contig2\_Mf\_liverA, NM\_019703, CL3750.Contig2\_Mf\_liverA, Unigene151\_Mf\_liverA, Unigene33459\_Mf\_liverA, CL848.Contig2\_Mf\_liverA, NM\_008808, Unigene4768\_Mf\_liverA, Unigene26398\_Mf\_liverA, Unigene34258\_Mf\_liverA, CL3198.Contig1\_Mf\_liverA, Unigene5632\_Mf\_liverA, Unigene25662\_Mf\_liverA, CL479.Contig1\_Mf\_liverA, Unigene37460\_Mf\_liverA, Unigene31231\_Mf\_liverA, Unigene24323\_Mf\_liverA, Unigene14715\_Mf\_liverA, NM\_008538, Unigene24503\_Mf\_liverA, NM\_008610, Unigene37180\_Mf\_liverA, Unigene4523\_Mf\_liverA, CL3816.Contig1\_Mf\_liverA, Unigene27438\_Mf\_liverA, CL2722.Contig1\_Mf\_liverA, CL5631.Contig1\_Mf\_liverA, Unigene31427\_Mf\_liverA, Unigene30584\_Mf\_liverA, Unigene4510\_Mf\_liverA, Unigene431\_Mf\_liverA, Unigene24284\_Mf\_liverA, Unigene19885\_Mf\_liverA, Unigene36510\_Mf\_liverA, NM\_009091, Unigene27260\_Mf\_liverA, Unigene44992\_Mf\_liverA, CL3268.Contig1\_Mf\_liverA, Unigene36845\_Mf\_liverA, NM\_010391, NM\_010227, Unigene21317\_Mf\_liverA, Unigene30493\_Mf\_liverA, Unigene37729\_Mf\_liverA, Unigene8132\_Mf\_liverA, Unigene29940\_Mf\_liverA, CL795.Contig1\_Mf\_liverA, Unigene42812\_Mf\_liverA, Unigene32058\_Mf\_liverA, Unigene27082\_Mf\_liverA, Unigene37178\_Mf\_liverA, Unigene5712\_Mf\_liverA, Unigene5325\_Mf\_liverA, Unigene1205\_Mf\_liverA, CL442.Contig2\_Mf\_liverA, CL1352.Contig1\_Mf\_liverA, CL6039.Contig1\_Mf\_liverA, Unigene26214\_Mf\_liverA, NM\_033374, CL2855.Contig1\_Mf\_liverA, CL4411.Contig4\_Mf\_liverA, NM\_001025388, Unigene24804\_Mf\_liverA, Unigene31206\_Mf\_liverA, Unigene13233\_Mf\_liverA, NM\_011099, Unigene5648\_Mf\_liverA, NM\_009447, Unigene40924\_Mf\_liverA, CL1537.Contig1\_Mf\_liverA, NM\_010481, CL4086.Contig1\_Mf\_liverA, NM\_009128, CL507.Contig1\_Mf\_liverA, Unigene5360\_Mf\_liverA, Unigene15318\_Mf\_liverA, Unigene7412\_Mf\_liverA, NM\_009178, CL2240.Contig1\_Mf\_liverA, Unigene13414\_Mf\_liverA, NM\_008293, Unigene22980\_Mf\_liverA, Unigene15474\_Mf\_liverA, Unigene1130\_Mf\_liverA, Unigene30154\_Mf\_liverA, Unigene38208\_Mf\_liverA, CL1125.Contig1\_Mf\_liverA, CL4162.Contig1\_Mf\_liverA, Unigene35237\_Mf\_liverA, CL5993.Contig3\_Mf\_liverA, Unigene37259\_Mf\_liverA, Unigene12907\_Mf\_liverA, Unigene15681\_Mf\_liverA, Unigene7350\_Mf\_liverA, Unigene19083\_Mf\_liverA, Unigene673\_Mf\_liverA, Unigene5260\_Mf\_liverA, Unigene4703\_Mf\_liverA, Unigene37096\_Mf\_liverA, Unigene5165\_Mf\_liverA, Unigene23013\_Mf\_liverA, Unigene30707\_Mf\_liverA, CL4583.Contig2\_Mf\_liverA, NM\_133838, Unigene7674\_Mf\_liverA, Unigene36328\_Mf\_liverA, CL3002.Contig1\_Mf\_liverA, NM\_008871, CL1222.Contig1\_Mf\_liverA, Unigene35491\_Mf\_liverA, CL6039.Contig2\_Mf\_liverA, Unigene35037\_Mf\_liverA, Unigene36710\_Mf\_liverA, Unigene5906\_Mf\_liverA, NM\_007393, CL5688.Contig1\_Mf\_liverA, Unigene28731\_Mf\_liverA, Unigene1212\_Mf\_liverA, CL2791.Contig1\_Mf\_liverA, CL3549.Contig1\_Mf\_liverA, NM\_009706, Unigene24085\_Mf\_liverA, Unigene10135\_Mf\_liverA, CL4106.Contig1\_Mf\_liverA, Unigene22330\_Mf\_liverA, CL3750.Contig1\_Mf\_liverA, Unigene5954\_Mf\_liverA, CL4127.Contig1\_Mf\_liverA, CL5307.Contig1\_Mf\_liverA, Unigene31730\_Mf\_liverA, Unigene51055\_Mf\_liverA, CL1190.Contig3\_Mf\_liverA, Unigene8740\_Mf\_liverA, CL5978.Contig2\_Mf\_liverA, NM\_008776, Unigene4540\_Mf\_liverA, NM\_007478, Unigene15170\_Mf\_liverA, Unigene560\_Mf\_liverA, Unigene24477\_Mf\_liverA, Unigene30983\_Mf\_liverA, Unigene30731\_Mf\_liverA, Unigene15026\_Mf\_liverA, Unigene1221\_Mf\_liverA, Unigene550\_Mf\_liverA, Unigene38311\_Mf\_liverA, Unigene39875\_Mf\_liverA, CL2355.Contig1\_Mf\_liverA, Unigene36910\_Mf\_liverA, NM\_001081274, Unigene37153\_Mf\_liverA, Unigene21562\_Mf\_liverA, Unigene15592\_Mf\_liverA, Unigene17632\_Mf\_liverA, Unigene5745\_Mf\_liverA, Unigene37711\_Mf\_liverA, NM\_015767, Unigene5418\_Mf\_liverA, Unigene12889\_Mf\_liverA, Unigene2195\_Mf\_liverA, Unigene38514\_Mf\_liverA, Unigene665\_Mf\_liverA, Unigene35678\_Mf\_liverA, Unigene803\_Mf\_liverA, CL4434.Contig1\_Mf\_liverA, Unigene8560\_Mf\_liverA, CL3911.Contig2\_Mf\_liverA, Unigene34866\_Mf\_liverA, Unigene13396\_Mf\_liverA, Unigene31885\_Mf\_liverA, Unigene21359\_Mf\_liverA, Unigene36487\_Mf\_liverA, Unigene4681\_Mf\_liverA, Unigene37474\_Mf\_liverA, CL548.Contig1\_Mf\_liverA, NM\_007392, Unigene16891\_Mf\_liverA, Unigene37819\_Mf\_liverA, Unigene5886\_Mf\_liverA, Unigene27274\_Mf\_liverA |
| MHC protein complex | Unigene37245\_Mf\_liverA, Unigene36543\_Mf\_liverA, Unigene4938\_Mf\_liverA, Unigene24471\_Mf\_liverA, CL3835.Contig2\_Mf\_liverA, CL3002.Contig1\_Mf\_liverA, CL2855.Contig2\_Mf\_liverA, NM\_010391, NM\_010378, Unigene13143\_Mf\_liverA, CL5039.Contig2\_Mf\_liverA, Unigene4681\_Mf\_liverA, NM\_010380, CL2855.Contig1\_Mf\_liverA, Unigene30878\_Mf\_liverA, NM\_001143689, Unigene50250\_Mf\_liverA, NR\_004446 |
| lytic vacuole | Unigene25524\_Mf\_liverA, Unigene24471\_Mf\_liverA, NM\_026823, NM\_011503, CL3835.Contig2\_Mf\_liverA, CL2855.Contig2\_Mf\_liverA, CL3669.Contig2\_Mf\_liverA, Unigene15529\_Mf\_liverA, Unigene28226\_Mf\_liverA, CL725.Contig1\_Mf\_liverA, Unigene13414\_Mf\_liverA, Unigene36622\_Mf\_liverA, NM\_010378, Unigene8740\_Mf\_liverA, Unigene1130\_Mf\_liverA, Unigene14715\_Mf\_liverA, Unigene36112\_Mf\_liverA, Unigene37259\_Mf\_liverA, Unigene560\_Mf\_liverA, Unigene39886\_Mf\_liverA, Unigene21466\_Mf\_liverA, Unigene30878\_Mf\_liverA, Unigene38919\_Mf\_liverA, Unigene35958\_Mf\_liverA, CL3055.Contig2\_Mf\_liverA, Unigene37575\_Mf\_liverA, Unigene29558\_Mf\_liverA, CL3669.Contig1\_Mf\_liverA, CL4583.Contig2\_Mf\_liverA, CL1736.Contig2\_Mf\_liverA, Unigene36328\_Mf\_liverA, Unigene5204\_Mf\_liverA, CL3002.Contig1\_Mf\_liverA, Unigene36910\_Mf\_liverA, Unigene27260\_Mf\_liverA, CL3055.Contig1\_Mf\_liverA, Unigene37389\_Mf\_liverA, Unigene32332\_Mf\_liverA, Unigene15592\_Mf\_liverA, Unigene5745\_Mf\_liverA, Unigene8132\_Mf\_liverA, Unigene5422\_Mf\_liverA, Unigene29940\_Mf\_liverA, Unigene46615\_Mf\_liverA, Unigene27082\_Mf\_liverA, NM\_009713, Unigene5906\_Mf\_liverA, CL1493.Contig2\_Mf\_liverA, CL442.Contig2\_Mf\_liverA, Unigene4681\_Mf\_liverA, CL1493.Contig1\_Mf\_liverA, Unigene21857\_Mf\_liverA, CL2855.Contig1\_Mf\_liverA, Unigene5236\_Mf\_liverA, Unigene24085\_Mf\_liverA |
| intrinsic to plasma membrane | Unigene40610\_Mf\_liverA, CL507.Contig1\_Mf\_liverA, Unigene42975\_Mf\_liverA, Unigene2162\_Mf\_liverA, Unigene24111\_Mf\_liverA, CL425.Contig1\_Mf\_liverA, CL3150.Contig1\_Mf\_liverA, CL1362.Contig1\_Mf\_liverA, Unigene15474\_Mf\_liverA, Unigene5194\_Mf\_liverA, Unigene15982\_Mf\_liverA, Unigene27593\_Mf\_liverA, Unigene22433\_Mf\_liverA, CL238.Contig1\_Mf\_liverA, CL5993.Contig3\_Mf\_liverA, Unigene30039\_Mf\_liverA, Unigene29231\_Mf\_liverA, NM\_010877, Unigene38919\_Mf\_liverA, Unigene24344\_Mf\_liverA, Unigene13525\_Mf\_liverA, CL5586.Contig1\_Mf\_liverA, Unigene36889\_Mf\_liverA, Unigene5204\_Mf\_liverA, CL1256.Contig1\_Mf\_liverA, Unigene34789\_Mf\_liverA, Unigene5758\_Mf\_liverA, Unigene24112\_Mf\_liverA, Unigene15703\_Mf\_liverA, Unigene4781\_Mf\_liverA, Unigene28331\_Mf\_liverA, Unigene37139\_Mf\_liverA, Unigene4686\_Mf\_liverA, Unigene29426\_Mf\_liverA, Unigene13462\_Mf\_liverA, Unigene5906\_Mf\_liverA, CL3575.Contig1\_Mf\_liverA, Unigene28025\_Mf\_liverA, Unigene22432\_Mf\_liverA, Unigene5330\_Mf\_liverA, Unigene24085\_Mf\_liverA, Unigene16684\_Mf\_liverA, Unigene41226\_Mf\_liverA, Unigene4909\_Mf\_liverA, Unigene13363\_Mf\_liverA, Unigene26065\_Mf\_liverA, Unigene29938\_Mf\_liverA, CL2855.Contig2\_Mf\_liverA, Unigene20128\_Mf\_liverA, Unigene28226\_Mf\_liverA, Unigene18125\_Mf\_liverA, Unigene47203\_Mf\_liverA, Unigene30947\_Mf\_liverA, Unigene21337\_Mf\_liverA, Unigene21561\_Mf\_liverA, Unigene8740\_Mf\_liverA, Unigene13913\_Mf\_liverA, CL479.Contig1\_Mf\_liverA, Unigene36112\_Mf\_liverA, Unigene35090\_Mf\_liverA, Unigene26941\_Mf\_liverA, Unigene39011\_Mf\_liverA, NM\_172509, Unigene39749\_Mf\_liverA, Unigene14330\_Mf\_liverA, Unigene25052\_Mf\_liverA, Unigene37575\_Mf\_liverA, CL4156.Contig1\_Mf\_liverA, Unigene21562\_Mf\_liverA, Unigene17632\_Mf\_liverA, Unigene21336\_Mf\_liverA, Unigene5422\_Mf\_liverA, Unigene36667\_Mf\_liverA, Unigene18340\_Mf\_liverA, Unigene27082\_Mf\_liverA, Unigene14765\_Mf\_liverA, Unigene4983\_Mf\_liverA, Unigene14284\_Mf\_liverA, CL2855.Contig1\_Mf\_liverA, Unigene26474\_Mf\_liverA, Unigene5169\_Mf\_liverA, Unigene17569\_Mf\_liverA, Unigene28021\_Mf\_liverA |
| endoplasmic reticulum | Unigene40924\_Mf\_liverA, Unigene34656\_Mf\_liverA, NM\_009128, Unigene5852\_Mf\_liverA, Unigene7612\_Mf\_liverA, Unigene5360\_Mf\_liverA, Unigene13414\_Mf\_liverA, CL2240.Contig1\_Mf\_liverA, Unigene22980\_Mf\_liverA, CL2797.Contig2\_Mf\_liverA, CL523.Contig1\_Mf\_liverA, Unigene5194\_Mf\_liverA, CL482.Contig1\_Mf\_liverA, Unigene37243\_Mf\_liverA, Unigene29405\_Mf\_liverA, Unigene35237\_Mf\_liverA, Unigene30587\_Mf\_liverA, CL5560.Contig1\_Mf\_liverA, Unigene22575\_Mf\_liverA, Unigene21466\_Mf\_liverA, Unigene40289\_Mf\_liverA, Unigene34124\_Mf\_liverA, Unigene35958\_Mf\_liverA, CL4757.Contig1\_Mf\_liverA, Unigene23013\_Mf\_liverA, Unigene36765\_Mf\_liverA, CL4583.Contig2\_Mf\_liverA, Unigene36328\_Mf\_liverA, Unigene34341\_Mf\_liverA, CL1988.Contig3\_Mf\_liverA, Unigene29885\_Mf\_liverA, CL3055.Contig1\_Mf\_liverA, Unigene35491\_Mf\_liverA, Unigene37263\_Mf\_liverA, Unigene46615\_Mf\_liverA, Unigene28331\_Mf\_liverA, Unigene4686\_Mf\_liverA, Unigene25596\_Mf\_liverA, Unigene25594\_Mf\_liverA, CL1493.Contig2\_Mf\_liverA, Unigene28731\_Mf\_liverA, CL1493.Contig1\_Mf\_liverA, Unigene22978\_Mf\_liverA, Unigene779\_Mf\_liverA, CL2574.Contig1\_Mf\_liverA, Unigene30585\_Mf\_liverA, Unigene23328\_Mf\_liverA, Unigene25462\_Mf\_liverA, Unigene542\_Mf\_liverA, Unigene10135\_Mf\_liverA, CL787.Contig1\_Mf\_liverA, Unigene34197\_Mf\_liverA, Unigene5598\_Mf\_liverA, NM\_010162, Unigene15888\_Mf\_liverA, CL2855.Contig2\_Mf\_liverA, CL4127.Contig1\_Mf\_liverA, CL5307.Contig1\_Mf\_liverA, Unigene31730\_Mf\_liverA, Unigene21561\_Mf\_liverA, Unigene1479\_Mf\_liverA, CL1190.Contig3\_Mf\_liverA, Unigene26398\_Mf\_liverA, NM\_008808, Unigene8740\_Mf\_liverA, Unigene4922\_Mf\_liverA, Unigene5632\_Mf\_liverA, CL479.Contig1\_Mf\_liverA, CL4490.Contig2\_Mf\_liverA, Unigene25595\_Mf\_liverA, Unigene139\_Mf\_liverA, CL2722.Contig1\_Mf\_liverA, Unigene34123\_Mf\_liverA, Unigene23185\_Mf\_liverA, CL3055.Contig2\_Mf\_liverA, Unigene30584\_Mf\_liverA, Unigene35046\_Mf\_liverA, Unigene36510\_Mf\_liverA, Unigene11097\_Mf\_liverA, Unigene37389\_Mf\_liverA, Unigene13772\_Mf\_liverA, Unigene30493\_Mf\_liverA, CL3104.Contig1\_Mf\_liverA, Unigene36593\_Mf\_liverA, Unigene21562\_Mf\_liverA, Unigene8132\_Mf\_liverA, Unigene37729\_Mf\_liverA, Unigene12889\_Mf\_liverA, Unigene2195\_Mf\_liverA, Unigene36699\_Mf\_liverA, Unigene29253\_Mf\_liverA, Unigene32058\_Mf\_liverA, Unigene27082\_Mf\_liverA, CL3911.Contig2\_Mf\_liverA, Unigene5712\_Mf\_liverA, CL5698.Contig1\_Mf\_liverA, Unigene1280\_Mf\_liverA, Unigene1205\_Mf\_liverA, CL442.Contig2\_Mf\_liverA, CL2855.Contig1\_Mf\_liverA, Unigene5886\_Mf\_liverA, CL4411.Contig4\_Mf\_liverA, Unigene5648\_Mf\_liverA, Unigene32059\_Mf\_liverA |
| immunological synapse | CL4434.Contig1\_Mf\_liverA, Unigene22433\_Mf\_liverA, Unigene34746\_Mf\_liverA, CL507.Contig1\_Mf\_liverA, Unigene24112\_Mf\_liverA, Unigene22432\_Mf\_liverA, Unigene26580\_Mf\_liverA, Unigene24111\_Mf\_liverA, Unigene39749\_Mf\_liverA, Unigene29008\_Mf\_liverA |
| integral to plasma membrane | Unigene40610\_Mf\_liverA, CL507.Contig1\_Mf\_liverA, Unigene42975\_Mf\_liverA, Unigene2162\_Mf\_liverA, Unigene24111\_Mf\_liverA, CL425.Contig1\_Mf\_liverA, CL3150.Contig1\_Mf\_liverA, CL1362.Contig1\_Mf\_liverA, Unigene15474\_Mf\_liverA, Unigene5194\_Mf\_liverA, Unigene15982\_Mf\_liverA, Unigene27593\_Mf\_liverA, Unigene22433\_Mf\_liverA, CL238.Contig1\_Mf\_liverA, CL5993.Contig3\_Mf\_liverA, Unigene30039\_Mf\_liverA, Unigene29231\_Mf\_liverA, NM\_010877, Unigene38919\_Mf\_liverA, Unigene24344\_Mf\_liverA, Unigene13525\_Mf\_liverA, CL5586.Contig1\_Mf\_liverA, Unigene36889\_Mf\_liverA, Unigene5204\_Mf\_liverA, CL1256.Contig1\_Mf\_liverA, Unigene34789\_Mf\_liverA, Unigene5758\_Mf\_liverA, Unigene24112\_Mf\_liverA, Unigene15703\_Mf\_liverA, Unigene4781\_Mf\_liverA, Unigene28331\_Mf\_liverA, Unigene37139\_Mf\_liverA, Unigene4686\_Mf\_liverA, Unigene29426\_Mf\_liverA, Unigene13462\_Mf\_liverA, Unigene5906\_Mf\_liverA, Unigene28025\_Mf\_liverA, Unigene22432\_Mf\_liverA, Unigene5330\_Mf\_liverA, Unigene16684\_Mf\_liverA, Unigene41226\_Mf\_liverA, Unigene4909\_Mf\_liverA, Unigene13363\_Mf\_liverA, Unigene26065\_Mf\_liverA, Unigene29938\_Mf\_liverA, CL2855.Contig2\_Mf\_liverA, Unigene20128\_Mf\_liverA, Unigene28226\_Mf\_liverA, Unigene18125\_Mf\_liverA, Unigene47203\_Mf\_liverA, Unigene30947\_Mf\_liverA, Unigene21337\_Mf\_liverA, Unigene8740\_Mf\_liverA, Unigene13913\_Mf\_liverA, CL479.Contig1\_Mf\_liverA, Unigene36112\_Mf\_liverA, Unigene35090\_Mf\_liverA, Unigene26941\_Mf\_liverA, Unigene39011\_Mf\_liverA, NM\_172509, Unigene39749\_Mf\_liverA, Unigene14330\_Mf\_liverA, Unigene37575\_Mf\_liverA, Unigene25052\_Mf\_liverA, Unigene17632\_Mf\_liverA, Unigene21336\_Mf\_liverA, Unigene5422\_Mf\_liverA, Unigene36667\_Mf\_liverA, Unigene18340\_Mf\_liverA, Unigene27082\_Mf\_liverA, Unigene14765\_Mf\_liverA, Unigene4983\_Mf\_liverA, Unigene14284\_Mf\_liverA, CL2855.Contig1\_Mf\_liverA, Unigene26474\_Mf\_liverA, Unigene5169\_Mf\_liverA, Unigene17569\_Mf\_liverA, Unigene28021\_Mf\_liverA |
| perinuclear region of cytoplasm | Unigene22330\_Mf\_liverA, Unigene26055\_Mf\_liverA, CL1537.Contig1\_Mf\_liverA, Unigene34656\_Mf\_liverA, Unigene31251\_Mf\_liverA, CL4086.Contig1\_Mf\_liverA, Unigene28527\_Mf\_liverA, CL186.Contig3\_Mf\_liverA, CL2625.Contig2\_Mf\_liverA, CL725.Contig1\_Mf\_liverA, Unigene5360\_Mf\_liverA, Unigene36622\_Mf\_liverA, Unigene51055\_Mf\_liverA, Unigene1479\_Mf\_liverA, CL1190.Contig3\_Mf\_liverA, Unigene26053\_Mf\_liverA, CL4162.Contig1\_Mf\_liverA, Unigene39011\_Mf\_liverA, Unigene27438\_Mf\_liverA, Unigene48460\_Mf\_liverA, Unigene5165\_Mf\_liverA, Unigene27249\_Mf\_liverA, Unigene36765\_Mf\_liverA, CL4583.Contig2\_Mf\_liverA, Unigene7674\_Mf\_liverA, Unigene29885\_Mf\_liverA, Unigene30142\_Mf\_liverA, Unigene4720\_Mf\_liverA, Unigene5687\_Mf\_liverA, Unigene27248\_Mf\_liverA, Unigene8132\_Mf\_liverA, Unigene24547\_Mf\_liverA, Unigene35816\_Mf\_liverA, Unigene2195\_Mf\_liverA, Unigene28331\_Mf\_liverA, Unigene4686\_Mf\_liverA, Unigene11\_Mf\_liverA, Unigene34866\_Mf\_liverA, Unigene13396\_Mf\_liverA, Unigene5712\_Mf\_liverA, Unigene5138\_Mf\_liverA, Unigene37076\_Mf\_liverA, Unigene34446\_Mf\_liverA, CL1352.Contig1\_Mf\_liverA, Unigene5330\_Mf\_liverA, Unigene2746\_Mf\_liverA, Unigene5236\_Mf\_liverA, Unigene24085\_Mf\_liverA, Unigene2745\_Mf\_liverA, Unigene25462\_Mf\_liverA |
| vacuole | Unigene25524\_Mf\_liverA, Unigene24471\_Mf\_liverA, NM\_026823, NM\_011503, CL3835.Contig2\_Mf\_liverA, CL2855.Contig2\_Mf\_liverA, CL3669.Contig2\_Mf\_liverA, Unigene15529\_Mf\_liverA, Unigene28226\_Mf\_liverA, CL725.Contig1\_Mf\_liverA, Unigene13414\_Mf\_liverA, Unigene36622\_Mf\_liverA, NM\_010378, Unigene8740\_Mf\_liverA, Unigene1130\_Mf\_liverA, CL5993.Contig3\_Mf\_liverA, Unigene14715\_Mf\_liverA, Unigene36112\_Mf\_liverA, Unigene37259\_Mf\_liverA, Unigene560\_Mf\_liverA, Unigene39886\_Mf\_liverA, Unigene21466\_Mf\_liverA, Unigene30878\_Mf\_liverA, Unigene38919\_Mf\_liverA, Unigene35958\_Mf\_liverA, CL3055.Contig2\_Mf\_liverA, Unigene37575\_Mf\_liverA, Unigene29558\_Mf\_liverA, CL3669.Contig1\_Mf\_liverA, CL4583.Contig2\_Mf\_liverA, CL1736.Contig2\_Mf\_liverA, Unigene36328\_Mf\_liverA, Unigene5204\_Mf\_liverA, CL3002.Contig1\_Mf\_liverA, Unigene36910\_Mf\_liverA, Unigene27260\_Mf\_liverA, CL3055.Contig1\_Mf\_liverA, Unigene37389\_Mf\_liverA, Unigene32332\_Mf\_liverA, Unigene15592\_Mf\_liverA, Unigene5745\_Mf\_liverA, Unigene8132\_Mf\_liverA, Unigene5422\_Mf\_liverA, Unigene29940\_Mf\_liverA, Unigene46615\_Mf\_liverA, Unigene27082\_Mf\_liverA, NM\_009713, Unigene5906\_Mf\_liverA, CL1493.Contig2\_Mf\_liverA, CL442.Contig2\_Mf\_liverA, CL1493.Contig1\_Mf\_liverA, Unigene4681\_Mf\_liverA, Unigene21857\_Mf\_liverA, CL2855.Contig1\_Mf\_liverA, CL3685.Contig1\_Mf\_liverA, Unigene5236\_Mf\_liverA, Unigene24085\_Mf\_liverA |
| membrane raft | Unigene36889\_Mf\_liverA, Unigene25092\_Mf\_liverA, Unigene37268\_Mf\_liverA, Unigene27260\_Mf\_liverA, Unigene23082\_Mf\_liverA, Unigene20128\_Mf\_liverA, Unigene25091\_Mf\_liverA, Unigene4720\_Mf\_liverA, CL725.Contig1\_Mf\_liverA, CL1810.Contig1\_Mf\_liverA, Unigene21562\_Mf\_liverA, Unigene21561\_Mf\_liverA, Unigene12889\_Mf\_liverA, Unigene28331\_Mf\_liverA, Unigene31333\_Mf\_liverA, Unigene34218\_Mf\_liverA, Unigene33514\_Mf\_liverA, Unigene34866\_Mf\_liverA, Unigene27026\_Mf\_liverA, Unigene27082\_Mf\_liverA, Unigene35237\_Mf\_liverA, Unigene25090\_Mf\_liverA, CL5698.Contig1\_Mf\_liverA, Unigene9990\_Mf\_liverA, Unigene34446\_Mf\_liverA, Unigene37259\_Mf\_liverA, Unigene29231\_Mf\_liverA, CL3816.Contig1\_Mf\_liverA, Unigene14284\_Mf\_liverA, CL5631.Contig1\_Mf\_liverA, Unigene24085\_Mf\_liverA |
| lysosomal lumen | CL4583.Contig2\_Mf\_liverA, Unigene36112\_Mf\_liverA, CL2855.Contig2\_Mf\_liverA, Unigene560\_Mf\_liverA, Unigene21466\_Mf\_liverA, Unigene15592\_Mf\_liverA, CL2855.Contig1\_Mf\_liverA, Unigene5236\_Mf\_liverA, Unigene5422\_Mf\_liverA, Unigene29940\_Mf\_liverA, Unigene38919\_Mf\_liverA, Unigene37575\_Mf\_liverA, Unigene29558\_Mf\_liverA, Unigene35958\_Mf\_liverA |
| vacuolar lumen | CL4583.Contig2\_Mf\_liverA, Unigene36112\_Mf\_liverA, CL2855.Contig2\_Mf\_liverA, Unigene560\_Mf\_liverA, Unigene21466\_Mf\_liverA, Unigene15592\_Mf\_liverA, CL2855.Contig1\_Mf\_liverA, Unigene5236\_Mf\_liverA, Unigene5422\_Mf\_liverA, Unigene29940\_Mf\_liverA, Unigene38919\_Mf\_liverA, Unigene37575\_Mf\_liverA, Unigene29558\_Mf\_liverA, Unigene35958\_Mf\_liverA |
| MHC class II protein complex | Unigene37245\_Mf\_liverA, CL2855.Contig1\_Mf\_liverA, Unigene24471\_Mf\_liverA, Unigene30878\_Mf\_liverA, CL3835.Contig2\_Mf\_liverA, CL3002.Contig1\_Mf\_liverA, CL2855.Contig2\_Mf\_liverA, Unigene4681\_Mf\_liverA |
| vacuolar part | CL4583.Contig2\_Mf\_liverA, Unigene24471\_Mf\_liverA, Unigene36328\_Mf\_liverA, Unigene5204\_Mf\_liverA, CL3835.Contig2\_Mf\_liverA, CL3002.Contig1\_Mf\_liverA, CL2855.Contig2\_Mf\_liverA, CL3055.Contig1\_Mf\_liverA, Unigene32332\_Mf\_liverA, Unigene15592\_Mf\_liverA, Unigene5422\_Mf\_liverA, Unigene29940\_Mf\_liverA, Unigene5906\_Mf\_liverA, CL5993.Contig3\_Mf\_liverA, Unigene36112\_Mf\_liverA, Unigene560\_Mf\_liverA, Unigene4681\_Mf\_liverA, Unigene21466\_Mf\_liverA, Unigene39886\_Mf\_liverA, Unigene21857\_Mf\_liverA, CL2855.Contig1\_Mf\_liverA, Unigene30878\_Mf\_liverA, CL3685.Contig1\_Mf\_liverA, Unigene5236\_Mf\_liverA, Unigene38919\_Mf\_liverA, Unigene29558\_Mf\_liverA, Unigene37575\_Mf\_liverA, CL3055.Contig2\_Mf\_liverA, Unigene35958\_Mf\_liverA |
| actin filament | Unigene25080\_Mf\_liverA, Unigene34197\_Mf\_liverA, Unigene16671\_Mf\_liverA, Unigene4557\_Mf\_liverA, NM\_009898, Unigene28822\_Mf\_liverA, NM\_053214, CL1222.Contig1\_Mf\_liverA, NM\_009609, NM\_177093, Unigene25047\_Mf\_liverA, Unigene29308\_Mf\_liverA, Unigene29008\_Mf\_liverA, Unigene5138\_Mf\_liverA, CL5698.Contig1\_Mf\_liverA, CL5358.Contig1\_Mf\_liverA, Unigene37474\_Mf\_liverA, Unigene25046\_Mf\_liverA, Unigene26214\_Mf\_liverA, NM\_007392, Unigene21857\_Mf\_liverA, Unigene4556\_Mf\_liverA |
| integral to membrane | Unigene26055\_Mf\_liverA, Unigene24506\_Mf\_liverA, CL3835.Contig2\_Mf\_liverA, Unigene42975\_Mf\_liverA, Unigene24111\_Mf\_liverA, Unigene28186\_Mf\_liverA, CL1362.Contig1\_Mf\_liverA, Unigene5194\_Mf\_liverA, Unigene26053\_Mf\_liverA, Unigene27593\_Mf\_liverA, Unigene22433\_Mf\_liverA, Unigene33627\_Mf\_liverA, CL238.Contig1\_Mf\_liverA, Unigene48792\_Mf\_liverA, CL5560.Contig1\_Mf\_liverA, Unigene22575\_Mf\_liverA, Unigene14816\_Mf\_liverA, Unigene37698\_Mf\_liverA, Unigene39886\_Mf\_liverA, Unigene30878\_Mf\_liverA, NM\_010877, Unigene38919\_Mf\_liverA, Unigene24344\_Mf\_liverA, CL5586.Contig1\_Mf\_liverA, Unigene36889\_Mf\_liverA, Unigene5204\_Mf\_liverA, Unigene37268\_Mf\_liverA, CL1256.Contig1\_Mf\_liverA, Unigene34789\_Mf\_liverA, Unigene24112\_Mf\_liverA, Unigene25057\_Mf\_liverA, Unigene15703\_Mf\_liverA, Unigene2\_Mf\_liverA, Unigene28331\_Mf\_liverA, Unigene31333\_Mf\_liverA, CL593.Contig2\_Mf\_liverA, Unigene4686\_Mf\_liverA, Unigene13266\_Mf\_liverA, Unigene37150\_Mf\_liverA, Unigene26533\_Mf\_liverA, Unigene22432\_Mf\_liverA, Unigene5330\_Mf\_liverA, Unigene34143\_Mf\_liverA, Unigene28094\_Mf\_liverA, CL2574.Contig1\_Mf\_liverA, Unigene27218\_Mf\_liverA, Unigene8054\_Mf\_liverA, Unigene25462\_Mf\_liverA, Unigene16684\_Mf\_liverA, CL787.Contig1\_Mf\_liverA, Unigene5552\_Mf\_liverA, Unigene5598\_Mf\_liverA, Unigene13363\_Mf\_liverA, Unigene26065\_Mf\_liverA, Unigene13230\_Mf\_liverA, CL2855.Contig2\_Mf\_liverA, Unigene20128\_Mf\_liverA, CL3196.Contig2\_Mf\_liverA, Unigene47203\_Mf\_liverA, Unigene28226\_Mf\_liverA, Unigene18125\_Mf\_liverA, Unigene38406\_Mf\_liverA, Unigene1479\_Mf\_liverA, Unigene7519\_Mf\_liverA, Unigene4922\_Mf\_liverA, Unigene13913\_Mf\_liverA, Unigene5632\_Mf\_liverA, CL479.Contig1\_Mf\_liverA, NM\_153505, Unigene17213\_Mf\_liverA, Unigene36112\_Mf\_liverA, Unigene941\_Mf\_liverA, Unigene30459\_Mf\_liverA, Unigene24503\_Mf\_liverA, Unigene6340\_Mf\_liverA, Unigene12200\_Mf\_liverA, Unigene35838\_Mf\_liverA, Unigene26941\_Mf\_liverA, CL3816.Contig1\_Mf\_liverA, NM\_172509, CL2722.Contig1\_Mf\_liverA, Unigene995\_Mf\_liverA, Unigene37420\_Mf\_liverA, NM\_009320, Unigene20116\_Mf\_liverA, Unigene25852\_Mf\_liverA, Unigene19735\_Mf\_liverA, CL3055.Contig2\_Mf\_liverA, Unigene37575\_Mf\_liverA, Unigene27249\_Mf\_liverA, Unigene16271\_Mf\_liverA, Unigene25398\_Mf\_liverA, Unigene27547\_Mf\_liverA, Unigene32889\_Mf\_liverA, Unigene37389\_Mf\_liverA, Unigene14070\_Mf\_liverA, Unigene32332\_Mf\_liverA, Unigene33271\_Mf\_liverA, Unigene29960\_Mf\_liverA, Unigene27248\_Mf\_liverA, Unigene29253\_Mf\_liverA, Unigene27082\_Mf\_liverA, Unigene5712\_Mf\_liverA, CL5039.Contig2\_Mf\_liverA, CL1738.Contig1\_Mf\_liverA, Unigene1088\_Mf\_liverA, Unigene479\_Mf\_liverA, Unigene674\_Mf\_liverA, Unigene15184\_Mf\_liverA, CL2855.Contig1\_Mf\_liverA, Unigene33760\_Mf\_liverA, Unigene5169\_Mf\_liverA, Unigene13233\_Mf\_liverA, Unigene17569\_Mf\_liverA, Unigene5648\_Mf\_liverA, Unigene28021\_Mf\_liverA, Unigene40924\_Mf\_liverA, Unigene40610\_Mf\_liverA, CL507.Contig1\_Mf\_liverA, Unigene7889\_Mf\_liverA, Unigene15275\_Mf\_liverA, Unigene2162\_Mf\_liverA, Unigene15318\_Mf\_liverA, Unigene13414\_Mf\_liverA, Unigene39385\_Mf\_liverA, CL425.Contig1\_Mf\_liverA, Unigene29823\_Mf\_liverA, CL3150.Contig1\_Mf\_liverA, CL2797.Contig2\_Mf\_liverA, Unigene4697\_Mf\_liverA, Unigene15474\_Mf\_liverA, Unigene15982\_Mf\_liverA, Unigene38208\_Mf\_liverA, Unigene27026\_Mf\_liverA, Unigene37526\_Mf\_liverA, Unigene39403\_Mf\_liverA, CL5993.Contig3\_Mf\_liverA, Unigene30039\_Mf\_liverA, Unigene29231\_Mf\_liverA, Unigene50250\_Mf\_liverA, Unigene4703\_Mf\_liverA, CL4057.Contig1\_Mf\_liverA, Unigene35958\_Mf\_liverA, Unigene23013\_Mf\_liverA, CL4583.Contig2\_Mf\_liverA, Unigene13525\_Mf\_liverA, Unigene4938\_Mf\_liverA, Unigene36543\_Mf\_liverA, CL3002.Contig1\_Mf\_liverA, CL3055.Contig1\_Mf\_liverA, CL1988.Contig3\_Mf\_liverA, Unigene6895\_Mf\_liverA, CL2327.Contig1\_Mf\_liverA, Unigene5758\_Mf\_liverA, CL4033.Contig1\_Mf\_liverA, Unigene12843\_Mf\_liverA, Unigene4781\_Mf\_liverA, Unigene46615\_Mf\_liverA, Unigene37139\_Mf\_liverA, Unigene25994\_Mf\_liverA, Unigene29426\_Mf\_liverA, Unigene13462\_Mf\_liverA, Unigene5906\_Mf\_liverA, CL1662.Contig1\_Mf\_liverA, Unigene28025\_Mf\_liverA, Unigene28731\_Mf\_liverA, Unigene4597\_Mf\_liverA, NM\_033444, Unigene11686\_Mf\_liverA, Unigene28499\_Mf\_liverA, CL5764.Contig1\_Mf\_liverA, Unigene10135\_Mf\_liverA, Unigene39231\_Mf\_liverA, Unigene41226\_Mf\_liverA, CL4106.Contig1\_Mf\_liverA, Unigene4909\_Mf\_liverA, Unigene34197\_Mf\_liverA, Unigene24471\_Mf\_liverA, Unigene29938\_Mf\_liverA, Unigene15888\_Mf\_liverA, CL4127.Contig1\_Mf\_liverA, Unigene23255\_Mf\_liverA, CL5307.Contig1\_Mf\_liverA, Unigene30947\_Mf\_liverA, Unigene21337\_Mf\_liverA, Unigene28873\_Mf\_liverA, Unigene8740\_Mf\_liverA, CL4490.Contig2\_Mf\_liverA, Unigene35090\_Mf\_liverA, Unigene9406\_Mf\_liverA, Unigene39011\_Mf\_liverA, Unigene14330\_Mf\_liverA, Unigene39749\_Mf\_liverA, Unigene28930\_Mf\_liverA, Unigene25052\_Mf\_liverA, Unigene23185\_Mf\_liverA, Unigene35046\_Mf\_liverA, Unigene37245\_Mf\_liverA, Unigene21843\_Mf\_liverA, Unigene281\_Mf\_liverA, Unigene14240\_Mf\_liverA, Unigene677\_Mf\_liverA, Unigene26580\_Mf\_liverA, CL3104.Contig1\_Mf\_liverA, Unigene36593\_Mf\_liverA, NM\_023580, Unigene17632\_Mf\_liverA, Unigene26336\_Mf\_liverA, Unigene5745\_Mf\_liverA, Unigene21336\_Mf\_liverA, Unigene5422\_Mf\_liverA, Unigene36699\_Mf\_liverA, Unigene36667\_Mf\_liverA, Unigene35417\_Mf\_liverA, Unigene18340\_Mf\_liverA, Unigene34866\_Mf\_liverA, Unigene14765\_Mf\_liverA, Unigene4983\_Mf\_liverA, CL5698.Contig1\_Mf\_liverA, Unigene5512\_Mf\_liverA, Unigene4681\_Mf\_liverA, CL548.Contig1\_Mf\_liverA, Unigene37189\_Mf\_liverA, Unigene31080\_Mf\_liverA, Unigene979\_Mf\_liverA, Unigene7733\_Mf\_liverA, Unigene14284\_Mf\_liverA, Unigene37819\_Mf\_liverA, Unigene26474\_Mf\_liverA, Unigene5886\_Mf\_liverA |
| symbiont-containing vacuole | CL482.Contig1\_Mf\_liverA, Unigene36328\_Mf\_liverA, CL442.Contig2\_Mf\_liverA, CL6039.Contig1\_Mf\_liverA, CL6039.Contig2\_Mf\_liverA, Unigene33459\_Mf\_liverA, CL5978.Contig3\_Mf\_liverA, CL5978.Contig2\_Mf\_liverA, Unigene8033\_Mf\_liverA |
| actin cytoskeleton | Unigene5472\_Mf\_liverA, Unigene34197\_Mf\_liverA, NM\_009898, Unigene25721\_Mf\_liverA, CL507.Contig1\_Mf\_liverA, Unigene28822\_Mf\_liverA, NM\_053214, NM\_177093, Unigene25047\_Mf\_liverA, CL848.Contig2\_Mf\_liverA, Unigene29308\_Mf\_liverA, CL2251.Contig1\_Mf\_liverA, Unigene29008\_Mf\_liverA, Unigene14637\_Mf\_liverA, Unigene37662\_Mf\_liverA, CL5358.Contig1\_Mf\_liverA, Unigene24477\_Mf\_liverA, Unigene36631\_Mf\_liverA, Unigene14896\_Mf\_liverA, Unigene15656\_Mf\_liverA, Unigene30707\_Mf\_liverA, Unigene25080\_Mf\_liverA, Unigene16671\_Mf\_liverA, Unigene4557\_Mf\_liverA, Unigene21255\_Mf\_liverA, CL1222.Contig1\_Mf\_liverA, NM\_134156, Unigene40020\_Mf\_liverA, NM\_010227, NM\_009609, Unigene26309\_Mf\_liverA, Unigene25057\_Mf\_liverA, Unigene24379\_Mf\_liverA, Unigene2195\_Mf\_liverA, CL795.Contig1\_Mf\_liverA, NM\_175260, CL4434.Contig1\_Mf\_liverA, Unigene31852\_Mf\_liverA, Unigene5138\_Mf\_liverA, CL5698.Contig1\_Mf\_liverA, Unigene37474\_Mf\_liverA, Unigene25046\_Mf\_liverA, NM\_007392, CL5576.Contig1\_Mf\_liverA, Unigene21857\_Mf\_liverA, Unigene26214\_Mf\_liverA, Unigene24755\_Mf\_liverA, CL591.Contig1\_Mf\_liverA, Unigene28499\_Mf\_liverA, Unigene4556\_Mf\_liverA |
| endoplasmic reticulum lumen | Unigene11097\_Mf\_liverA, Unigene5852\_Mf\_liverA, Unigene7612\_Mf\_liverA, Unigene13414\_Mf\_liverA, Unigene30493\_Mf\_liverA, Unigene31730\_Mf\_liverA, Unigene22980\_Mf\_liverA, Unigene1479\_Mf\_liverA, Unigene37729\_Mf\_liverA, Unigene26398\_Mf\_liverA, CL3911.Contig2\_Mf\_liverA, Unigene25596\_Mf\_liverA, Unigene25595\_Mf\_liverA, Unigene29405\_Mf\_liverA, Unigene22978\_Mf\_liverA, Unigene21466\_Mf\_liverA, Unigene34123\_Mf\_liverA, Unigene23328\_Mf\_liverA, Unigene34124\_Mf\_liverA, Unigene35958\_Mf\_liverA |
| host cell cytoplasm | CL482.Contig1\_Mf\_liverA, Unigene36328\_Mf\_liverA, CL442.Contig2\_Mf\_liverA, CL6039.Contig1\_Mf\_liverA, CL6039.Contig2\_Mf\_liverA, Unigene33459\_Mf\_liverA, CL5978.Contig3\_Mf\_liverA, CL5978.Contig2\_Mf\_liverA, Unigene8033\_Mf\_liverA |
| host cell cytoplasm part | CL482.Contig1\_Mf\_liverA, Unigene36328\_Mf\_liverA, CL442.Contig2\_Mf\_liverA, CL6039.Contig1\_Mf\_liverA, CL6039.Contig2\_Mf\_liverA, Unigene33459\_Mf\_liverA, CL5978.Contig3\_Mf\_liverA, CL5978.Contig2\_Mf\_liverA, Unigene8033\_Mf\_liverA |
| host intracellular part | CL482.Contig1\_Mf\_liverA, Unigene36328\_Mf\_liverA, CL442.Contig2\_Mf\_liverA, CL6039.Contig1\_Mf\_liverA, CL6039.Contig2\_Mf\_liverA, Unigene33459\_Mf\_liverA, CL5978.Contig3\_Mf\_liverA, CL5978.Contig2\_Mf\_liverA, Unigene8033\_Mf\_liverA |
| intracellular region of host | CL482.Contig1\_Mf\_liverA, Unigene36328\_Mf\_liverA, CL442.Contig2\_Mf\_liverA, CL6039.Contig1\_Mf\_liverA, CL6039.Contig2\_Mf\_liverA, Unigene33459\_Mf\_liverA, CL5978.Contig3\_Mf\_liverA, CL5978.Contig2\_Mf\_liverA, Unigene8033\_Mf\_liverA |
| integrin complex | Unigene18340\_Mf\_liverA, Unigene22433\_Mf\_liverA, Unigene40610\_Mf\_liverA, Unigene4983\_Mf\_liverA, CL507.Contig1\_Mf\_liverA, Unigene29938\_Mf\_liverA, Unigene24112\_Mf\_liverA, Unigene22432\_Mf\_liverA, Unigene24111\_Mf\_liverA, Unigene14284\_Mf\_liverA, Unigene39749\_Mf\_liverA |
| host | CL482.Contig1\_Mf\_liverA, Unigene36328\_Mf\_liverA, CL442.Contig2\_Mf\_liverA, CL6039.Contig1\_Mf\_liverA, CL6039.Contig2\_Mf\_liverA, Unigene33459\_Mf\_liverA, CL5978.Contig3\_Mf\_liverA, CL5978.Contig2\_Mf\_liverA, Unigene8033\_Mf\_liverA |
| host cell part | CL482.Contig1\_Mf\_liverA, Unigene36328\_Mf\_liverA, CL442.Contig2\_Mf\_liverA, CL6039.Contig1\_Mf\_liverA, CL6039.Contig2\_Mf\_liverA, Unigene33459\_Mf\_liverA, CL5978.Contig3\_Mf\_liverA, CL5978.Contig2\_Mf\_liverA, Unigene8033\_Mf\_liverA |
| extraorganismal space | CL482.Contig1\_Mf\_liverA, Unigene36328\_Mf\_liverA, CL442.Contig2\_Mf\_liverA, CL6039.Contig1\_Mf\_liverA, CL6039.Contig2\_Mf\_liverA, Unigene33459\_Mf\_liverA, CL5978.Contig3\_Mf\_liverA, CL5978.Contig2\_Mf\_liverA, Unigene8033\_Mf\_liverA |
| host cell | CL482.Contig1\_Mf\_liverA, Unigene36328\_Mf\_liverA, CL442.Contig2\_Mf\_liverA, CL6039.Contig1\_Mf\_liverA, CL6039.Contig2\_Mf\_liverA, Unigene33459\_Mf\_liverA, CL5978.Contig3\_Mf\_liverA, CL5978.Contig2\_Mf\_liverA, Unigene8033\_Mf\_liverA |
| other organism | CL482.Contig1\_Mf\_liverA, Unigene36328\_Mf\_liverA, CL442.Contig2\_Mf\_liverA, CL6039.Contig1\_Mf\_liverA, CL6039.Contig2\_Mf\_liverA, Unigene33459\_Mf\_liverA, CL5978.Contig3\_Mf\_liverA, CL5978.Contig2\_Mf\_liverA, Unigene8033\_Mf\_liverA |
| other organism cell | CL482.Contig1\_Mf\_liverA, Unigene36328\_Mf\_liverA, CL442.Contig2\_Mf\_liverA, CL6039.Contig1\_Mf\_liverA, CL6039.Contig2\_Mf\_liverA, Unigene33459\_Mf\_liverA, CL5978.Contig3\_Mf\_liverA, CL5978.Contig2\_Mf\_liverA, Unigene8033\_Mf\_liverA |
| other organism part | CL482.Contig1\_Mf\_liverA, Unigene36328\_Mf\_liverA, CL442.Contig2\_Mf\_liverA, CL6039.Contig1\_Mf\_liverA, CL6039.Contig2\_Mf\_liverA, Unigene33459\_Mf\_liverA, CL5978.Contig3\_Mf\_liverA, CL5978.Contig2\_Mf\_liverA, Unigene8033\_Mf\_liverA |
| extracellular organelle | CL482.Contig1\_Mf\_liverA, Unigene36328\_Mf\_liverA, CL442.Contig2\_Mf\_liverA, CL6039.Contig1\_Mf\_liverA, CL6039.Contig2\_Mf\_liverA, Unigene33459\_Mf\_liverA, CL5978.Contig3\_Mf\_liverA, CL2251.Contig1\_Mf\_liverA, CL5978.Contig2\_Mf\_liverA, Unigene8033\_Mf\_liverA |
| extracellular membrane-bounded organelle | CL482.Contig1\_Mf\_liverA, Unigene36328\_Mf\_liverA, CL442.Contig2\_Mf\_liverA, CL6039.Contig1\_Mf\_liverA, CL6039.Contig2\_Mf\_liverA, Unigene33459\_Mf\_liverA, CL5978.Contig3\_Mf\_liverA, CL2251.Contig1\_Mf\_liverA, CL5978.Contig2\_Mf\_liverA, Unigene8033\_Mf\_liverA |
| platelet alpha granule | CL6038.Contig2\_Mf\_liverA, Unigene29399\_Mf\_liverA, Unigene4909\_Mf\_liverA, NM\_008871, NM\_134156, Unigene5852\_Mf\_liverA, Unigene15529\_Mf\_liverA, Unigene28662\_Mf\_liverA, NM\_008808, Unigene34258\_Mf\_liverA, CL2251.Contig1\_Mf\_liverA, Unigene36673\_Mf\_liverA, NM\_009776, Unigene19049\_Mf\_liverA, Unigene11544\_Mf\_liverA, NM\_011580 |
| symbiont-containing vacuole membrane | CL482.Contig1\_Mf\_liverA, CL6039.Contig2\_Mf\_liverA, CL6039.Contig1\_Mf\_liverA, Unigene33459\_Mf\_liverA, CL5978.Contig3\_Mf\_liverA, CL5978.Contig2\_Mf\_liverA, Unigene8033\_Mf\_liverA |
| apical plasma membrane | Unigene4909\_Mf\_liverA, Unigene25080\_Mf\_liverA, CL5586.Contig1\_Mf\_liverA, Unigene16671\_Mf\_liverA, Unigene42975\_Mf\_liverA, CL725.Contig1\_Mf\_liverA, Unigene21562\_Mf\_liverA, CL4033.Contig1\_Mf\_liverA, Unigene28186\_Mf\_liverA, Unigene21561\_Mf\_liverA, Unigene26336\_Mf\_liverA, Unigene28873\_Mf\_liverA, Unigene29424\_Mf\_liverA, Unigene35816\_Mf\_liverA, Unigene28331\_Mf\_liverA, CL3519.Contig1\_Mf\_liverA, Unigene30892\_Mf\_liverA, Unigene4686\_Mf\_liverA, Unigene27082\_Mf\_liverA, CL5993.Contig3\_Mf\_liverA, Unigene139\_Mf\_liverA, Unigene4597\_Mf\_liverA, Unigene26214\_Mf\_liverA, CL591.Contig1\_Mf\_liverA, Unigene24085\_Mf\_liverA, Unigene38919\_Mf\_liverA, Unigene43515\_Mf\_liverA |
| uropod | CL4434.Contig1\_Mf\_liverA, Unigene25080\_Mf\_liverA, Unigene16671\_Mf\_liverA, CL507.Contig1\_Mf\_liverA, Unigene5422\_Mf\_liverA, Unigene29424\_Mf\_liverA |
| endoplasmic reticulum part | Unigene34656\_Mf\_liverA, NM\_009128, Unigene5852\_Mf\_liverA, Unigene7612\_Mf\_liverA, Unigene13414\_Mf\_liverA, CL2240.Contig1\_Mf\_liverA, Unigene22980\_Mf\_liverA, CL2797.Contig2\_Mf\_liverA, CL523.Contig1\_Mf\_liverA, CL482.Contig1\_Mf\_liverA, Unigene29405\_Mf\_liverA, Unigene35237\_Mf\_liverA, CL5560.Contig1\_Mf\_liverA, Unigene22575\_Mf\_liverA, Unigene21466\_Mf\_liverA, Unigene34124\_Mf\_liverA, Unigene35958\_Mf\_liverA, Unigene23013\_Mf\_liverA, CL4583.Contig2\_Mf\_liverA, Unigene34341\_Mf\_liverA, CL1988.Contig3\_Mf\_liverA, Unigene4686\_Mf\_liverA, Unigene25596\_Mf\_liverA, Unigene28731\_Mf\_liverA, Unigene22978\_Mf\_liverA, CL2574.Contig1\_Mf\_liverA, Unigene23328\_Mf\_liverA, Unigene25462\_Mf\_liverA, Unigene10135\_Mf\_liverA, Unigene542\_Mf\_liverA, Unigene5598\_Mf\_liverA, NM\_010162, Unigene15888\_Mf\_liverA, CL2855.Contig2\_Mf\_liverA, CL4127.Contig1\_Mf\_liverA, CL5307.Contig1\_Mf\_liverA, Unigene31730\_Mf\_liverA, Unigene1479\_Mf\_liverA, Unigene26398\_Mf\_liverA, NM\_008808, Unigene5632\_Mf\_liverA, Unigene4922\_Mf\_liverA, CL4490.Contig2\_Mf\_liverA, Unigene25595\_Mf\_liverA, Unigene139\_Mf\_liverA, Unigene34123\_Mf\_liverA, Unigene23185\_Mf\_liverA, Unigene35046\_Mf\_liverA, Unigene36510\_Mf\_liverA, Unigene11097\_Mf\_liverA, Unigene37389\_Mf\_liverA, Unigene30493\_Mf\_liverA, Unigene13772\_Mf\_liverA, Unigene36593\_Mf\_liverA, Unigene37729\_Mf\_liverA, Unigene36699\_Mf\_liverA, Unigene29253\_Mf\_liverA, Unigene27082\_Mf\_liverA, CL3911.Contig2\_Mf\_liverA, Unigene5712\_Mf\_liverA, Unigene1280\_Mf\_liverA, CL5698.Contig1\_Mf\_liverA, Unigene1205\_Mf\_liverA, CL2855.Contig1\_Mf\_liverA, Unigene5886\_Mf\_liverA, Unigene5648\_Mf\_liverA |
| cortical cytoskeleton | CL4434.Contig1\_Mf\_liverA, Unigene30707\_Mf\_liverA, CL507.Contig1\_Mf\_liverA, Unigene28822\_Mf\_liverA, CL1222.Contig1\_Mf\_liverA, Unigene25046\_Mf\_liverA, Unigene26214\_Mf\_liverA, Unigene25047\_Mf\_liverA, CL848.Contig2\_Mf\_liverA, Unigene29308\_Mf\_liverA, CL795.Contig1\_Mf\_liverA, Unigene15656\_Mf\_liverA, Unigene29008\_Mf\_liverA |
| multivesicular body | Unigene37245\_Mf\_liverA, Unigene27082\_Mf\_liverA, CL2855.Contig1\_Mf\_liverA, Unigene30878\_Mf\_liverA, Unigene8132\_Mf\_liverA, CL3002.Contig1\_Mf\_liverA, CL2855.Contig2\_Mf\_liverA, Unigene4681\_Mf\_liverA |
| secretory granule | Unigene29399\_Mf\_liverA, Unigene4909\_Mf\_liverA, NM\_011503, Unigene5852\_Mf\_liverA, Unigene15529\_Mf\_liverA, CL1828.Contig1\_Mf\_liverA, CL1190.Contig3\_Mf\_liverA, Unigene29424\_Mf\_liverA, NM\_008808, Unigene34258\_Mf\_liverA, CL2251.Contig1\_Mf\_liverA, Unigene19049\_Mf\_liverA, Unigene30587\_Mf\_liverA, Unigene9990\_Mf\_liverA, Unigene26941\_Mf\_liverA, CL3816.Contig1\_Mf\_liverA, NM\_011580, NM\_010877, Unigene7510\_Mf\_liverA, Unigene35958\_Mf\_liverA, Unigene30584\_Mf\_liverA, Unigene34810\_Mf\_liverA, CL6038.Contig2\_Mf\_liverA, Unigene5204\_Mf\_liverA, NM\_134156, NM\_008871, Unigene28662\_Mf\_liverA, Unigene28331\_Mf\_liverA, Unigene36673\_Mf\_liverA, Unigene4686\_Mf\_liverA, NM\_009713, NM\_009776, CL3911.Contig2\_Mf\_liverA, Unigene11544\_Mf\_liverA, Unigene30585\_Mf\_liverA, Unigene8054\_Mf\_liverA |
| phagocytic vesicle | Unigene4686\_Mf\_liverA, Unigene36328\_Mf\_liverA, CL5993.Contig3\_Mf\_liverA, CL507.Contig1\_Mf\_liverA, Unigene37389\_Mf\_liverA, CL1828.Contig1\_Mf\_liverA, Unigene8054\_Mf\_liverA, Unigene8740\_Mf\_liverA, Unigene34810\_Mf\_liverA, Unigene29008\_Mf\_liverA |
| lysosomal membrane | CL4583.Contig2\_Mf\_liverA, Unigene24471\_Mf\_liverA, Unigene5204\_Mf\_liverA, Unigene5906\_Mf\_liverA, CL3835.Contig2\_Mf\_liverA, CL3002.Contig1\_Mf\_liverA, CL2855.Contig2\_Mf\_liverA, CL3055.Contig1\_Mf\_liverA, Unigene4681\_Mf\_liverA, Unigene32332\_Mf\_liverA, Unigene39886\_Mf\_liverA, Unigene21857\_Mf\_liverA, CL2855.Contig1\_Mf\_liverA, Unigene30878\_Mf\_liverA, CL3055.Contig2\_Mf\_liverA |
| trailing edge | CL4434.Contig1\_Mf\_liverA, Unigene25080\_Mf\_liverA, Unigene16671\_Mf\_liverA, CL507.Contig1\_Mf\_liverA, Unigene5422\_Mf\_liverA, Unigene29424\_Mf\_liverA |
| cell cortex | Unigene30707\_Mf\_liverA, Unigene25080\_Mf\_liverA, Unigene16671\_Mf\_liverA, CL507.Contig1\_Mf\_liverA, Unigene28822\_Mf\_liverA, CL1222.Contig1\_Mf\_liverA, Unigene44317\_Mf\_liverA, Unigene25047\_Mf\_liverA, CL848.Contig2\_Mf\_liverA, Unigene29308\_Mf\_liverA, Unigene4768\_Mf\_liverA, CL795.Contig1\_Mf\_liverA, Unigene28331\_Mf\_liverA, Unigene4922\_Mf\_liverA, Unigene29008\_Mf\_liverA, CL4434.Contig1\_Mf\_liverA, Unigene34446\_Mf\_liverA, CL2117.Contig1\_Mf\_liverA, Unigene25046\_Mf\_liverA, Unigene26214\_Mf\_liverA, Unigene5693\_Mf\_liverA, Unigene37575\_Mf\_liverA, Unigene15656\_Mf\_liverA, Unigene24252\_Mf\_liverA |
| apical part of cell | Unigene4909\_Mf\_liverA, Unigene25080\_Mf\_liverA, CL5586.Contig1\_Mf\_liverA, Unigene16671\_Mf\_liverA, Unigene42975\_Mf\_liverA, CL725.Contig1\_Mf\_liverA, Unigene21562\_Mf\_liverA, CL4033.Contig1\_Mf\_liverA, Unigene28186\_Mf\_liverA, Unigene21561\_Mf\_liverA, Unigene26336\_Mf\_liverA, Unigene28873\_Mf\_liverA, Unigene29424\_Mf\_liverA, Unigene2195\_Mf\_liverA, Unigene35816\_Mf\_liverA, Unigene28331\_Mf\_liverA, CL3519.Contig1\_Mf\_liverA, Unigene30892\_Mf\_liverA, Unigene4686\_Mf\_liverA, Unigene27082\_Mf\_liverA, CL5993.Contig3\_Mf\_liverA, Unigene139\_Mf\_liverA, Unigene4597\_Mf\_liverA, Unigene26214\_Mf\_liverA, CL591.Contig1\_Mf\_liverA, Unigene24085\_Mf\_liverA, Unigene38919\_Mf\_liverA, Unigene37575\_Mf\_liverA, Unigene43515\_Mf\_liverA |
| receptor complex | Unigene13525\_Mf\_liverA, CL4086.Contig1\_Mf\_liverA, Unigene40610\_Mf\_liverA, CL507.Contig1\_Mf\_liverA, Unigene29938\_Mf\_liverA, CL2855.Contig2\_Mf\_liverA, Unigene25091\_Mf\_liverA, Unigene24112\_Mf\_liverA, Unigene24111\_Mf\_liverA, Unigene31333\_Mf\_liverA, Unigene18340\_Mf\_liverA, Unigene15982\_Mf\_liverA, Unigene29426\_Mf\_liverA, Unigene22433\_Mf\_liverA, Unigene25090\_Mf\_liverA, Unigene4983\_Mf\_liverA, Unigene37150\_Mf\_liverA, Unigene22432\_Mf\_liverA, Unigene14284\_Mf\_liverA, CL2855.Contig1\_Mf\_liverA, Unigene39749\_Mf\_liverA |
| late endosome | Unigene37245\_Mf\_liverA, Unigene24471\_Mf\_liverA, NM\_026823, Unigene36328\_Mf\_liverA, Unigene5204\_Mf\_liverA, CL3835.Contig2\_Mf\_liverA, CL3002.Contig1\_Mf\_liverA, Unigene27260\_Mf\_liverA, CL2855.Contig2\_Mf\_liverA, CL3055.Contig1\_Mf\_liverA, Unigene8132\_Mf\_liverA, Unigene27082\_Mf\_liverA, CL1493.Contig2\_Mf\_liverA, CL442.Contig2\_Mf\_liverA, Unigene4681\_Mf\_liverA, CL1493.Contig1\_Mf\_liverA, Unigene21857\_Mf\_liverA, Unigene5693\_Mf\_liverA, CL2855.Contig1\_Mf\_liverA, Unigene30878\_Mf\_liverA, CL3055.Contig2\_Mf\_liverA |
| anchored to membrane | Unigene23448\_Mf\_liverA, CL3575.Contig1\_Mf\_liverA, Unigene36112\_Mf\_liverA, Unigene38831\_Mf\_liverA, Unigene20128\_Mf\_liverA, Unigene2162\_Mf\_liverA, Unigene13097\_Mf\_liverA, CL4156.Contig1\_Mf\_liverA, Unigene37698\_Mf\_liverA, Unigene21562\_Mf\_liverA, Unigene21561\_Mf\_liverA, Unigene5169\_Mf\_liverA, Unigene24085\_Mf\_liverA, Unigene31333\_Mf\_liverA |
| cell cortex part | CL4434.Contig1\_Mf\_liverA, Unigene30707\_Mf\_liverA, CL507.Contig1\_Mf\_liverA, Unigene28822\_Mf\_liverA, CL1222.Contig1\_Mf\_liverA, Unigene44317\_Mf\_liverA, Unigene25046\_Mf\_liverA, Unigene26214\_Mf\_liverA, Unigene25047\_Mf\_liverA, CL848.Contig2\_Mf\_liverA, Unigene29308\_Mf\_liverA, CL795.Contig1\_Mf\_liverA, Unigene37575\_Mf\_liverA, Unigene15656\_Mf\_liverA, Unigene29008\_Mf\_liverA |
| vacuolar membrane | CL4583.Contig2\_Mf\_liverA, Unigene24471\_Mf\_liverA, Unigene36328\_Mf\_liverA, Unigene5204\_Mf\_liverA, CL3835.Contig2\_Mf\_liverA, CL3002.Contig1\_Mf\_liverA, CL2855.Contig2\_Mf\_liverA, CL3055.Contig1\_Mf\_liverA, Unigene32332\_Mf\_liverA, CL5993.Contig3\_Mf\_liverA, Unigene5906\_Mf\_liverA, Unigene4681\_Mf\_liverA, Unigene21857\_Mf\_liverA, Unigene39886\_Mf\_liverA, CL2855.Contig1\_Mf\_liverA, Unigene30878\_Mf\_liverA, CL3685.Contig1\_Mf\_liverA, CL3055.Contig2\_Mf\_liverA |
| phagocytic vesicle membrane | CL1828.Contig1\_Mf\_liverA, Unigene4686\_Mf\_liverA, Unigene36328\_Mf\_liverA, Unigene8054\_Mf\_liverA, CL5993.Contig3\_Mf\_liverA, Unigene29008\_Mf\_liverA, Unigene34810\_Mf\_liverA |
| membrane | Unigene24506\_Mf\_liverA, NM\_176843, Unigene34656\_Mf\_liverA, NM\_009898, Unigene25721\_Mf\_liverA, Unigene8106\_Mf\_liverA, NM\_001025208, NM\_009592, NM\_010807, Unigene24111\_Mf\_liverA, Unigene28186\_Mf\_liverA, NM\_018815, NM\_010378, Unigene29424\_Mf\_liverA, Unigene34218\_Mf\_liverA, Unigene26053\_Mf\_liverA, Unigene31045\_Mf\_liverA, Unigene33627\_Mf\_liverA, Unigene25090\_Mf\_liverA, Unigene16463\_Mf\_liverA, CL238.Contig1\_Mf\_liverA, CL5560.Contig1\_Mf\_liverA, Unigene22575\_Mf\_liverA, Unigene39886\_Mf\_liverA, Unigene38689\_Mf\_liverA, NM\_010877, NM\_172723, NM\_021273, Unigene38919\_Mf\_liverA, Unigene1137\_Mf\_liverA, NR\_004446, CL5586.Contig1\_Mf\_liverA, Unigene21255\_Mf\_liverA, Unigene34341\_Mf\_liverA, Unigene30142\_Mf\_liverA, Unigene15703\_Mf\_liverA, Unigene2\_Mf\_liverA, Unigene35816\_Mf\_liverA, Unigene28331\_Mf\_liverA, NM\_010233, NM\_145474, CL593.Contig2\_Mf\_liverA, Unigene13940\_Mf\_liverA, Unigene13379\_Mf\_liverA, Unigene13266\_Mf\_liverA, NM\_153193, Unigene5138\_Mf\_liverA, Unigene37150\_Mf\_liverA, Unigene5330\_Mf\_liverA, Unigene21857\_Mf\_liverA, Unigene34143\_Mf\_liverA, Unigene748\_Mf\_liverA, Unigene27218\_Mf\_liverA, Unigene8054\_Mf\_liverA, Unigene23869\_Mf\_liverA, Unigene5552\_Mf\_liverA, Unigene5598\_Mf\_liverA, Unigene13363\_Mf\_liverA, Unigene26065\_Mf\_liverA, Unigene13230\_Mf\_liverA, CL2855.Contig2\_Mf\_liverA, Unigene25091\_Mf\_liverA, Unigene18125\_Mf\_liverA, Unigene28226\_Mf\_liverA, Unigene13097\_Mf\_liverA, Unigene25047\_Mf\_liverA, Unigene37278\_Mf\_liverA, Unigene2919\_Mf\_liverA, Unigene21561\_Mf\_liverA, Unigene1479\_Mf\_liverA, Unigene7519\_Mf\_liverA, Unigene4922\_Mf\_liverA, Unigene13913\_Mf\_liverA, NM\_153505, Unigene30261\_Mf\_liverA, Unigene9475\_Mf\_liverA, Unigene36112\_Mf\_liverA, Unigene36641\_Mf\_liverA, NM\_015803, Unigene941\_Mf\_liverA, Unigene9990\_Mf\_liverA, Unigene30459\_Mf\_liverA, NM\_001104531, Unigene139\_Mf\_liverA, NM\_153055, Unigene26941\_Mf\_liverA, Unigene35838\_Mf\_liverA, Unigene33560\_Mf\_liverA, NM\_172509, Unigene995\_Mf\_liverA, Unigene37420\_Mf\_liverA, NM\_001143689, Unigene19735\_Mf\_liverA, Unigene37575\_Mf\_liverA, CL3055.Contig2\_Mf\_liverA, Unigene27249\_Mf\_liverA, Unigene43515\_Mf\_liverA, Unigene16271\_Mf\_liverA, Unigene25398\_Mf\_liverA, Unigene27547\_Mf\_liverA, Unigene21842\_Mf\_liverA, NM\_134156, Unigene32889\_Mf\_liverA, Unigene4720\_Mf\_liverA, Unigene37389\_Mf\_liverA, Unigene14070\_Mf\_liverA, Unigene32332\_Mf\_liverA, Unigene33271\_Mf\_liverA, Unigene4723\_Mf\_liverA, Unigene29960\_Mf\_liverA, CL4156.Contig1\_Mf\_liverA, Unigene32716\_Mf\_liverA, Unigene27248\_Mf\_liverA, CL5254.Contig1\_Mf\_liverA, CL3232.Contig1\_Mf\_liverA, Unigene29253\_Mf\_liverA, Unigene34010\_Mf\_liverA, Unigene31852\_Mf\_liverA, Unigene5456\_Mf\_liverA, NM\_009713, Unigene37076\_Mf\_liverA, Unigene2678\_Mf\_liverA, Unigene1280\_Mf\_liverA, Unigene21256\_Mf\_liverA, CL1738.Contig1\_Mf\_liverA, Unigene479\_Mf\_liverA, Unigene674\_Mf\_liverA, Unigene15184\_Mf\_liverA, Unigene2746\_Mf\_liverA, NR\_003552, Unigene5169\_Mf\_liverA, Unigene16465\_Mf\_liverA, Unigene10496\_Mf\_liverA, Unigene28021\_Mf\_liverA, Unigene32202\_Mf\_liverA, Unigene26515\_Mf\_liverA, NM\_009933, Unigene31251\_Mf\_liverA, NM\_008365, NM\_011503, Unigene40610\_Mf\_liverA, NM\_001159724, Unigene2162\_Mf\_liverA, CL425.Contig1\_Mf\_liverA, CL3150.Contig1\_Mf\_liverA, CL2797.Contig2\_Mf\_liverA, Unigene4697\_Mf\_liverA, Unigene37243\_Mf\_liverA, Unigene27026\_Mf\_liverA, Unigene39403\_Mf\_liverA, Unigene30587\_Mf\_liverA, Unigene30039\_Mf\_liverA, Unigene29231\_Mf\_liverA, Unigene5693\_Mf\_liverA, Unigene50250\_Mf\_liverA, CL4057.Contig1\_Mf\_liverA, Unigene35958\_Mf\_liverA, Unigene1440\_Mf\_liverA, Unigene43037\_Mf\_liverA, CL3800.Contig1\_Mf\_liverA, Unigene13525\_Mf\_liverA, Unigene25080\_Mf\_liverA, Unigene28687\_Mf\_liverA, Unigene4938\_Mf\_liverA, CL1988.Contig3\_Mf\_liverA, Unigene29885\_Mf\_liverA, CL3055.Contig1\_Mf\_liverA, Unigene5758\_Mf\_liverA, CL1810.Contig1\_Mf\_liverA, CL4033.Contig1\_Mf\_liverA, Unigene12843\_Mf\_liverA, Unigene27483\_Mf\_liverA, Unigene24547\_Mf\_liverA, Unigene28662\_Mf\_liverA, Unigene4781\_Mf\_liverA, Unigene46615\_Mf\_liverA, CL3519.Contig1\_Mf\_liverA, Unigene29426\_Mf\_liverA, Unigene13462\_Mf\_liverA, Unigene28025\_Mf\_liverA, Unigene34446\_Mf\_liverA, CL1493.Contig1\_Mf\_liverA, Unigene11686\_Mf\_liverA, Unigene28499\_Mf\_liverA, CL3685.Contig1\_Mf\_liverA, Unigene30585\_Mf\_liverA, CL5764.Contig1\_Mf\_liverA, Unigene38593\_Mf\_liverA, Unigene39231\_Mf\_liverA, Unigene542\_Mf\_liverA, Unigene41226\_Mf\_liverA, Unigene4909\_Mf\_liverA, Unigene34197\_Mf\_liverA, Unigene24471\_Mf\_liverA, Unigene25092\_Mf\_liverA, Unigene15888\_Mf\_liverA, Unigene29938\_Mf\_liverA, NM\_010162, Unigene15529\_Mf\_liverA, CL725.Contig1\_Mf\_liverA, Unigene30947\_Mf\_liverA, CL1165.Contig2\_Mf\_liverA, CL2251.Contig1\_Mf\_liverA, CL4490.Contig2\_Mf\_liverA, Unigene33514\_Mf\_liverA, Unigene35090\_Mf\_liverA, CL1165.Contig4\_Mf\_liverA, Unigene39011\_Mf\_liverA, Unigene26250\_Mf\_liverA, Unigene30795\_Mf\_liverA, Unigene20432\_Mf\_liverA, Unigene14330\_Mf\_liverA, Unigene23185\_Mf\_liverA, Unigene35046\_Mf\_liverA, Unigene37245\_Mf\_liverA, Unigene16671\_Mf\_liverA, Unigene281\_Mf\_liverA, Unigene13535\_Mf\_liverA, Unigene13772\_Mf\_liverA, CL3104.Contig1\_Mf\_liverA, Unigene29302\_Mf\_liverA, Unigene26580\_Mf\_liverA, Unigene5687\_Mf\_liverA, Unigene36593\_Mf\_liverA, Unigene5037\_Mf\_liverA, Unigene26336\_Mf\_liverA, Unigene33428\_Mf\_liverA, Unigene5422\_Mf\_liverA, Unigene36699\_Mf\_liverA, Unigene36667\_Mf\_liverA, Unigene18340\_Mf\_liverA, NM\_029219, Unigene30412\_Mf\_liverA, Unigene4983\_Mf\_liverA, CL5698.Contig1\_Mf\_liverA, Unigene5512\_Mf\_liverA, CL373.Contig7\_Mf\_liverA, Unigene37189\_Mf\_liverA, CL591.Contig1\_Mf\_liverA, Unigene9466\_Mf\_liverA, Unigene26055\_Mf\_liverA, CL5640.Contig1\_Mf\_liverA, Unigene38015\_Mf\_liverA, NM\_007896, CL3835.Contig2\_Mf\_liverA, Unigene38831\_Mf\_liverA, Unigene42975\_Mf\_liverA, CL1828.Contig1\_Mf\_liverA, Unigene29308\_Mf\_liverA, Unigene14603\_Mf\_liverA, CL1362.Contig1\_Mf\_liverA, CL523.Contig1\_Mf\_liverA, Unigene8473\_Mf\_liverA, Unigene29008\_Mf\_liverA, Unigene5194\_Mf\_liverA, CL482.Contig1\_Mf\_liverA, Unigene27593\_Mf\_liverA, Unigene19049\_Mf\_liverA, Unigene22433\_Mf\_liverA, Unigene37132\_Mf\_liverA, Unigene48792\_Mf\_liverA, Unigene14816\_Mf\_liverA, Unigene37698\_Mf\_liverA, Unigene30303\_Mf\_liverA, Unigene30878\_Mf\_liverA, Unigene15656\_Mf\_liverA, Unigene34234\_Mf\_liverA, Unigene34810\_Mf\_liverA, NM\_172671, CL6038.Contig2\_Mf\_liverA, Unigene24344\_Mf\_liverA, Unigene36889\_Mf\_liverA, Unigene5204\_Mf\_liverA, Unigene13307\_Mf\_liverA, Unigene37268\_Mf\_liverA, Unigene40020\_Mf\_liverA, CL1256.Contig1\_Mf\_liverA, Unigene34789\_Mf\_liverA, Unigene24112\_Mf\_liverA, Unigene25057\_Mf\_liverA, CL5191.Contig2\_Mf\_liverA, Unigene36853\_Mf\_liverA, Unigene38110\_Mf\_liverA, Unigene26194\_Mf\_liverA, Unigene31333\_Mf\_liverA, NM\_175260, Unigene4686\_Mf\_liverA, Unigene4630\_Mf\_liverA, Unigene11\_Mf\_liverA, Unigene11544\_Mf\_liverA, NM\_018780, NM\_010763, Unigene18117\_Mf\_liverA, CL3575.Contig1\_Mf\_liverA, CL1493.Contig2\_Mf\_liverA, Unigene3174\_Mf\_liverA, CL2117.Contig1\_Mf\_liverA, Unigene26533\_Mf\_liverA, Unigene22432\_Mf\_liverA, Unigene25046\_Mf\_liverA, Unigene36988\_Mf\_liverA, Unigene28094\_Mf\_liverA, Unigene40824\_Mf\_liverA, CL2574.Contig1\_Mf\_liverA, Unigene2745\_Mf\_liverA, Unigene25462\_Mf\_liverA, Unigene16684\_Mf\_liverA, CL787.Contig1\_Mf\_liverA, Unigene1162\_Mf\_liverA, Unigene25524\_Mf\_liverA, CL4847.Contig1\_Mf\_liverA, NM\_029562, Unigene20128\_Mf\_liverA, CL3196.Contig2\_Mf\_liverA, Unigene31988\_Mf\_liverA, Unigene47203\_Mf\_liverA, Unigene38406\_Mf\_liverA, Unigene33459\_Mf\_liverA, Unigene23870\_Mf\_liverA, Unigene4768\_Mf\_liverA, CL3198.Contig1\_Mf\_liverA, Unigene13143\_Mf\_liverA, Unigene5632\_Mf\_liverA, CL479.Contig1\_Mf\_liverA, NM\_007811, Unigene17213\_Mf\_liverA, Unigene14715\_Mf\_liverA, Unigene34746\_Mf\_liverA, NM\_008538, Unigene24503\_Mf\_liverA, NM\_008610, Unigene6340\_Mf\_liverA, Unigene12200\_Mf\_liverA, CL3816.Contig1\_Mf\_liverA, CL2722.Contig1\_Mf\_liverA, NM\_009320, Unigene20116\_Mf\_liverA, CL5631.Contig1\_Mf\_liverA, Unigene25852\_Mf\_liverA, Unigene14896\_Mf\_liverA, Unigene30584\_Mf\_liverA, Unigene431\_Mf\_liverA, Unigene24284\_Mf\_liverA, Unigene36510\_Mf\_liverA, Unigene26873\_Mf\_liverA, Unigene27260\_Mf\_liverA, Unigene44992\_Mf\_liverA, Unigene36845\_Mf\_liverA, NM\_010391, NM\_010227, Unigene8132\_Mf\_liverA, CL795.Contig1\_Mf\_liverA, NM\_019717, Unigene27082\_Mf\_liverA, Unigene5712\_Mf\_liverA, CL5039.Contig2\_Mf\_liverA, Unigene1205\_Mf\_liverA, Unigene23055\_Mf\_liverA, CL442.Contig2\_Mf\_liverA, Unigene1088\_Mf\_liverA, CL6039.Contig1\_Mf\_liverA, Unigene26214\_Mf\_liverA, NM\_010380, CL2855.Contig1\_Mf\_liverA, Unigene33760\_Mf\_liverA, NM\_001025388, Unigene24804\_Mf\_liverA, Unigene31206\_Mf\_liverA, Unigene13233\_Mf\_liverA, NM\_011099, Unigene17569\_Mf\_liverA, Unigene5648\_Mf\_liverA, NM\_001100182, Unigene40924\_Mf\_liverA, CL4086.Contig1\_Mf\_liverA, NM\_009128, CL507.Contig1\_Mf\_liverA, Unigene23082\_Mf\_liverA, Unigene7889\_Mf\_liverA, Unigene15275\_Mf\_liverA, Unigene15318\_Mf\_liverA, NM\_009178, CL2240.Contig1\_Mf\_liverA, Unigene13414\_Mf\_liverA, Unigene39385\_Mf\_liverA, Unigene29823\_Mf\_liverA, NM\_008293, Unigene15474\_Mf\_liverA, Unigene23448\_Mf\_liverA, Unigene30154\_Mf\_liverA, Unigene15982\_Mf\_liverA, Unigene38208\_Mf\_liverA, CL1125.Contig1\_Mf\_liverA, Unigene37526\_Mf\_liverA, Unigene35237\_Mf\_liverA, CL5993.Contig3\_Mf\_liverA, Unigene37259\_Mf\_liverA, Unigene12907\_Mf\_liverA, Unigene15681\_Mf\_liverA, Unigene7350\_Mf\_liverA, Unigene4703\_Mf\_liverA, Unigene37096\_Mf\_liverA, Unigene5165\_Mf\_liverA, Unigene23013\_Mf\_liverA, Unigene30707\_Mf\_liverA, CL4583.Contig2\_Mf\_liverA, NM\_133838, Unigene36543\_Mf\_liverA, NM\_011352, Unigene36328\_Mf\_liverA, CL3002.Contig1\_Mf\_liverA, NM\_008871, Unigene6895\_Mf\_liverA, CL2327.Contig1\_Mf\_liverA, Unigene24250\_Mf\_liverA, Unigene35491\_Mf\_liverA, CL6039.Contig2\_Mf\_liverA, Unigene37139\_Mf\_liverA, Unigene25994\_Mf\_liverA, NM\_010393, Unigene5906\_Mf\_liverA, CL1662.Contig1\_Mf\_liverA, Unigene28731\_Mf\_liverA, NM\_033444, Unigene4597\_Mf\_liverA, Unigene14170\_Mf\_liverA, Unigene24085\_Mf\_liverA, Unigene10135\_Mf\_liverA, NM\_007986, CL4106.Contig1\_Mf\_liverA, Unigene22330\_Mf\_liverA, Unigene8016\_Mf\_liverA, Unigene5011\_Mf\_liverA, CL4127.Contig1\_Mf\_liverA, Unigene18796\_Mf\_liverA, Unigene23255\_Mf\_liverA, CL5307.Contig1\_Mf\_liverA, Unigene13616\_Mf\_liverA, Unigene21337\_Mf\_liverA, Unigene51055\_Mf\_liverA, CL1190.Contig3\_Mf\_liverA, Unigene2035\_Mf\_liverA, Unigene28873\_Mf\_liverA, Unigene8740\_Mf\_liverA, NM\_007478, Unigene14580\_Mf\_liverA, Unigene9406\_Mf\_liverA, Unigene560\_Mf\_liverA, Unigene39749\_Mf\_liverA, Unigene1221\_Mf\_liverA, Unigene28930\_Mf\_liverA, Unigene25052\_Mf\_liverA, Unigene550\_Mf\_liverA, Unigene21843\_Mf\_liverA, CL2355.Contig1\_Mf\_liverA, Unigene36910\_Mf\_liverA, Unigene14240\_Mf\_liverA, Unigene677\_Mf\_liverA, Unigene21562\_Mf\_liverA, NM\_023580, Unigene17632\_Mf\_liverA, Unigene5745\_Mf\_liverA, Unigene21336\_Mf\_liverA, Unigene12889\_Mf\_liverA, Unigene2195\_Mf\_liverA, Unigene38514\_Mf\_liverA, Unigene93\_Mf\_liverA, NM\_146007, Unigene30892\_Mf\_liverA, Unigene35417\_Mf\_liverA, CL4434.Contig1\_Mf\_liverA, CL3911.Contig2\_Mf\_liverA, Unigene34866\_Mf\_liverA, Unigene13396\_Mf\_liverA, Unigene14765\_Mf\_liverA, Unigene21359\_Mf\_liverA, NM\_001033481, Unigene4681\_Mf\_liverA, CL548.Contig1\_Mf\_liverA, Unigene31080\_Mf\_liverA, Unigene979\_Mf\_liverA, Unigene16891\_Mf\_liverA, Unigene7733\_Mf\_liverA, Unigene14284\_Mf\_liverA, Unigene37819\_Mf\_liverA, Unigene26474\_Mf\_liverA, Unigene5886\_Mf\_liverA |
| platelet alpha granule lumen | CL6038.Contig2\_Mf\_liverA, Unigene29399\_Mf\_liverA, Unigene19049\_Mf\_liverA, Unigene5852\_Mf\_liverA, Unigene15529\_Mf\_liverA, Unigene28662\_Mf\_liverA, CL2251.Contig1\_Mf\_liverA, Unigene34258\_Mf\_liverA, Unigene36673\_Mf\_liverA |
| lamellipodium | Unigene30707\_Mf\_liverA, Unigene31852\_Mf\_liverA, Unigene5138\_Mf\_liverA, Unigene31885\_Mf\_liverA, CL2117.Contig1\_Mf\_liverA, Unigene39011\_Mf\_liverA, CL1352.Contig1\_Mf\_liverA, Unigene29302\_Mf\_liverA, Unigene5693\_Mf\_liverA, Unigene40824\_Mf\_liverA, NM\_007986, CL795.Contig1\_Mf\_liverA, Unigene14896\_Mf\_liverA, Unigene29008\_Mf\_liverA, Unigene24252\_Mf\_liverA |
| secretory granule lumen | CL6038.Contig2\_Mf\_liverA, Unigene29399\_Mf\_liverA, Unigene19049\_Mf\_liverA, Unigene5852\_Mf\_liverA, Unigene15529\_Mf\_liverA, Unigene28662\_Mf\_liverA, CL2251.Contig1\_Mf\_liverA, Unigene34258\_Mf\_liverA, Unigene36673\_Mf\_liverA |
| MHC class I protein complex | Unigene36543\_Mf\_liverA, Unigene4938\_Mf\_liverA, Unigene50250\_Mf\_liverA, CL5039.Contig2\_Mf\_liverA, Unigene13143\_Mf\_liverA |
| cytoplasmic membrane-bounded vesicle lumen | CL6038.Contig2\_Mf\_liverA, Unigene29399\_Mf\_liverA, Unigene19049\_Mf\_liverA, Unigene5852\_Mf\_liverA, Unigene15529\_Mf\_liverA, Unigene28662\_Mf\_liverA, CL2251.Contig1\_Mf\_liverA, Unigene34258\_Mf\_liverA, Unigene36673\_Mf\_liverA |
| endocytic vesicle membrane | Unigene4686\_Mf\_liverA, Unigene36328\_Mf\_liverA, NM\_009898, CL5993.Contig3\_Mf\_liverA, CL2855.Contig2\_Mf\_liverA, CL1828.Contig1\_Mf\_liverA, CL2855.Contig1\_Mf\_liverA, Unigene8054\_Mf\_liverA, Unigene28331\_Mf\_liverA, Unigene34810\_Mf\_liverA, Unigene29008\_Mf\_liverA |
| cytoplasmic vesicle part | Unigene29399\_Mf\_liverA, Unigene4909\_Mf\_liverA, NM\_009898, CL2855.Contig2\_Mf\_liverA, Unigene5852\_Mf\_liverA, Unigene15529\_Mf\_liverA, CL1828.Contig1\_Mf\_liverA, Unigene29424\_Mf\_liverA, Unigene34258\_Mf\_liverA, CL2251.Contig1\_Mf\_liverA, Unigene4922\_Mf\_liverA, Unigene29008\_Mf\_liverA, Unigene5632\_Mf\_liverA, Unigene19049\_Mf\_liverA, CL5993.Contig3\_Mf\_liverA, Unigene9990\_Mf\_liverA, CL3816.Contig1\_Mf\_liverA, Unigene34234\_Mf\_liverA, Unigene34810\_Mf\_liverA, CL6038.Contig2\_Mf\_liverA, Unigene36328\_Mf\_liverA, Unigene5204\_Mf\_liverA, NM\_134156, Unigene28662\_Mf\_liverA, Unigene28331\_Mf\_liverA, Unigene36673\_Mf\_liverA, Unigene4686\_Mf\_liverA, Unigene27082\_Mf\_liverA, CL548.Contig1\_Mf\_liverA, CL2855.Contig1\_Mf\_liverA, Unigene28094\_Mf\_liverA, Unigene8054\_Mf\_liverA |
| endocytic vesicle | Unigene22330\_Mf\_liverA, Unigene4686\_Mf\_liverA, CL3911.Contig2\_Mf\_liverA, Unigene36328\_Mf\_liverA, NM\_009898, CL5993.Contig3\_Mf\_liverA, CL507.Contig1\_Mf\_liverA, CL2855.Contig2\_Mf\_liverA, Unigene37389\_Mf\_liverA, CL1828.Contig1\_Mf\_liverA, CL2855.Contig1\_Mf\_liverA, Unigene24085\_Mf\_liverA, Unigene8054\_Mf\_liverA, Unigene28331\_Mf\_liverA, Unigene8740\_Mf\_liverA, Unigene34810\_Mf\_liverA, Unigene29008\_Mf\_liverA |
| intermediate filament | Unigene27082\_Mf\_liverA, CL4162.Contig1\_Mf\_liverA, CL1537.Contig1\_Mf\_liverA, Unigene9475\_Mf\_liverA, CL4847.Contig1\_Mf\_liverA, Unigene37076\_Mf\_liverA, Unigene2678\_Mf\_liverA, Unigene5940\_Mf\_liverA, NM\_010579, Unigene34789\_Mf\_liverA, Unigene5941\_Mf\_liverA, Unigene5165\_Mf\_liverA |
| NADPH oxidase complex | Unigene4686\_Mf\_liverA, Unigene8740\_Mf\_liverA, Unigene5194\_Mf\_liverA |
| dendrite | Unigene30707\_Mf\_liverA, Unigene36414\_Mf\_liverA, Unigene27547\_Mf\_liverA, Unigene37488\_Mf\_liverA, Unigene31251\_Mf\_liverA, Unigene28527\_Mf\_liverA, Unigene13363\_Mf\_liverA, Unigene27260\_Mf\_liverA, CL1222.Contig1\_Mf\_liverA, NM\_134156, Unigene36417\_Mf\_liverA, Unigene34789\_Mf\_liverA, Unigene21562\_Mf\_liverA, Unigene5037\_Mf\_liverA, CL425.Contig1\_Mf\_liverA, Unigene21561\_Mf\_liverA, Unigene24547\_Mf\_liverA, Unigene8740\_Mf\_liverA, NM\_175260, Unigene5194\_Mf\_liverA, Unigene4686\_Mf\_liverA, CL479.Contig1\_Mf\_liverA, Unigene13396\_Mf\_liverA, Unigene9990\_Mf\_liverA, CL2117.Contig1\_Mf\_liverA, Unigene37259\_Mf\_liverA, CL1738.Contig1\_Mf\_liverA, Unigene36420\_Mf\_liverA, Unigene36418\_Mf\_liverA, Unigene7510\_Mf\_liverA, Unigene14896\_Mf\_liverA, Unigene24252\_Mf\_liverA |
| MCM complex | Unigene42855\_Mf\_liverA, Unigene23271\_Mf\_liverA, Unigene7969\_Mf\_liverA, Unigene23273\_Mf\_liverA, Unigene7968\_Mf\_liverA |
| vesicle lumen | CL6038.Contig2\_Mf\_liverA, Unigene29399\_Mf\_liverA, Unigene19049\_Mf\_liverA, Unigene5852\_Mf\_liverA, Unigene15529\_Mf\_liverA, Unigene28662\_Mf\_liverA, CL2251.Contig1\_Mf\_liverA, Unigene34258\_Mf\_liverA, Unigene36673\_Mf\_liverA |
| Golgi lumen | Unigene29399\_Mf\_liverA, Unigene5422\_Mf\_liverA, Unigene29940\_Mf\_liverA, Unigene36112\_Mf\_liverA, Unigene5852\_Mf\_liverA, Unigene28021\_Mf\_liverA, Unigene560\_Mf\_liverA |
| extrinsic to membrane | Unigene13525\_Mf\_liverA, Unigene25080\_Mf\_liverA, Unigene24284\_Mf\_liverA, Unigene16671\_Mf\_liverA, CL1828.Contig1\_Mf\_liverA, CL4156.Contig1\_Mf\_liverA, Unigene2919\_Mf\_liverA, Unigene51055\_Mf\_liverA, Unigene29424\_Mf\_liverA, Unigene34218\_Mf\_liverA, Unigene37243\_Mf\_liverA, CL373.Contig7\_Mf\_liverA, Unigene8054\_Mf\_liverA, NM\_001025388, Unigene1440\_Mf\_liverA, Unigene35958\_Mf\_liverA, Unigene34810\_Mf\_liverA |
| unconventional myosin complex | Unigene5472\_Mf\_liverA, Unigene34197\_Mf\_liverA, Unigene37662\_Mf\_liverA, Unigene28822\_Mf\_liverA |
| anchored to external side of plasma membrane | CL4156.Contig1\_Mf\_liverA, Unigene21562\_Mf\_liverA, Unigene21561\_Mf\_liverA, Unigene24085\_Mf\_liverA |
| mitochondrion | Unigene24506\_Mf\_liverA, NM\_020559, NM\_010481, CL3669.Contig2\_Mf\_liverA, CL5528.Contig1\_Mf\_liverA, CL425.Contig1\_Mf\_liverA, NM\_008293, CL2797.Contig2\_Mf\_liverA, Unigene5194\_Mf\_liverA, Unigene1130\_Mf\_liverA, Unigene38208\_Mf\_liverA, Unigene14916\_Mf\_liverA, CL1125.Contig1\_Mf\_liverA, CL2423.Contig1\_Mf\_liverA, CL5993.Contig3\_Mf\_liverA, Unigene32882\_Mf\_liverA, Unigene12907\_Mf\_liverA, Unigene21466\_Mf\_liverA, Unigene26422\_Mf\_liverA, Unigene5693\_Mf\_liverA, Unigene19083\_Mf\_liverA, Unigene30303\_Mf\_liverA, Unigene40289\_Mf\_liverA, CL4757.Contig1\_Mf\_liverA, Unigene29876\_Mf\_liverA, Unigene13525\_Mf\_liverA, Unigene38331\_Mf\_liverA, Unigene37999\_Mf\_liverA, Unigene29885\_Mf\_liverA, CL1810.Contig1\_Mf\_liverA, Unigene25057\_Mf\_liverA, Unigene2\_Mf\_liverA, Unigene35816\_Mf\_liverA, Unigene4781\_Mf\_liverA, Unigene13658\_Mf\_liverA, Unigene28331\_Mf\_liverA, Unigene4686\_Mf\_liverA, Unigene35037\_Mf\_liverA, Unigene13266\_Mf\_liverA, NM\_153193, Unigene18117\_Mf\_liverA, Unigene3174\_Mf\_liverA, CL2791.Contig1\_Mf\_liverA, Unigene38104\_Mf\_liverA, Unigene38657\_Mf\_liverA, Unigene25462\_Mf\_liverA, Unigene36626\_Mf\_liverA, Unigene1162\_Mf\_liverA, CL3750.Contig1\_Mf\_liverA, CL5828.Contig2\_Mf\_liverA, Unigene35039\_Mf\_liverA, Unigene743\_Mf\_liverA, Unigene36691\_Mf\_liverA, CL725.Contig1\_Mf\_liverA, CL3750.Contig2\_Mf\_liverA, CL4127.Contig1\_Mf\_liverA, Unigene7476\_Mf\_liverA, CL1190.Contig3\_Mf\_liverA, CL2251.Contig1\_Mf\_liverA, Unigene37460\_Mf\_liverA, CL4490.Contig2\_Mf\_liverA, Unigene30261\_Mf\_liverA, Unigene14286\_Mf\_liverA, Unigene24503\_Mf\_liverA, Unigene36641\_Mf\_liverA, CL3816.Contig1\_Mf\_liverA, CL2722.Contig1\_Mf\_liverA, Unigene995\_Mf\_liverA, Unigene30731\_Mf\_liverA, CL5631.Contig1\_Mf\_liverA, Unigene23185\_Mf\_liverA, Unigene37575\_Mf\_liverA, Unigene29558\_Mf\_liverA, CL3692.Contig2\_Mf\_liverA, Unigene4510\_Mf\_liverA, CL3669.Contig1\_Mf\_liverA, Unigene25398\_Mf\_liverA, Unigene28459\_Mf\_liverA, Unigene27547\_Mf\_liverA, CL1736.Contig2\_Mf\_liverA, Unigene27260\_Mf\_liverA, Unigene10313\_Mf\_liverA, Unigene36845\_Mf\_liverA, CL3104.Contig1\_Mf\_liverA, Unigene17632\_Mf\_liverA, Unigene37711\_Mf\_liverA, Unigene33428\_Mf\_liverA, Unigene12889\_Mf\_liverA, Unigene2195\_Mf\_liverA, Unigene5418\_Mf\_liverA, Unigene803\_Mf\_liverA, Unigene27082\_Mf\_liverA, Unigene21359\_Mf\_liverA, Unigene36487\_Mf\_liverA, Unigene5512\_Mf\_liverA, CL3339.Contig1\_Mf\_liverA, Unigene31206\_Mf\_liverA, Unigene13233\_Mf\_liverA, Unigene36728\_Mf\_liverA, Unigene37433\_Mf\_liverA |
| cytoplasmic membrane-bounded vesicle | Unigene29399\_Mf\_liverA, NM\_011503, NM\_009898, CL507.Contig1\_Mf\_liverA, Unigene5852\_Mf\_liverA, CL1828.Contig1\_Mf\_liverA, Unigene29424\_Mf\_liverA, Unigene29008\_Mf\_liverA, Unigene19049\_Mf\_liverA, Unigene30587\_Mf\_liverA, CL5993.Contig3\_Mf\_liverA, Unigene37259\_Mf\_liverA, NM\_010877, Unigene13271\_Mf\_liverA, Unigene38919\_Mf\_liverA, Unigene7510\_Mf\_liverA, Unigene35958\_Mf\_liverA, Unigene34234\_Mf\_liverA, Unigene34810\_Mf\_liverA, CL6038.Contig2\_Mf\_liverA, Unigene36328\_Mf\_liverA, Unigene5204\_Mf\_liverA, Unigene21255\_Mf\_liverA, NM\_008871, CL3055.Contig1\_Mf\_liverA, Unigene34789\_Mf\_liverA, Unigene28662\_Mf\_liverA, Unigene46615\_Mf\_liverA, Unigene28331\_Mf\_liverA, Unigene4686\_Mf\_liverA, NM\_009776, Unigene11544\_Mf\_liverA, Unigene28094\_Mf\_liverA, Unigene28499\_Mf\_liverA, CL3685.Contig1\_Mf\_liverA, Unigene30585\_Mf\_liverA, Unigene24085\_Mf\_liverA, Unigene8054\_Mf\_liverA, Unigene22330\_Mf\_liverA, Unigene4909\_Mf\_liverA, Unigene13363\_Mf\_liverA, Unigene15529\_Mf\_liverA, CL2855.Contig2\_Mf\_liverA, Unigene28226\_Mf\_liverA, CL725.Contig1\_Mf\_liverA, CL1190.Contig3\_Mf\_liverA, NM\_008808, CL2251.Contig1\_Mf\_liverA, Unigene8740\_Mf\_liverA, Unigene34258\_Mf\_liverA, Unigene4922\_Mf\_liverA, Unigene5632\_Mf\_liverA, Unigene9990\_Mf\_liverA, Unigene26941\_Mf\_liverA, CL3816.Contig1\_Mf\_liverA, NM\_011580, CL3055.Contig2\_Mf\_liverA, Unigene30584\_Mf\_liverA, Unigene24284\_Mf\_liverA, NM\_134156, Unigene37389\_Mf\_liverA, Unigene36673\_Mf\_liverA, Unigene27082\_Mf\_liverA, NM\_009713, CL3911.Contig2\_Mf\_liverA, Unigene21256\_Mf\_liverA, CL1738.Contig1\_Mf\_liverA, CL548.Contig1\_Mf\_liverA, Unigene37819\_Mf\_liverA, CL2855.Contig1\_Mf\_liverA, Unigene38237\_Mf\_liverA, Unigene28021\_Mf\_liverA |
| cytoskeletal part | Unigene5472\_Mf\_liverA, CL1537.Contig1\_Mf\_liverA, NM\_007896, NM\_009898, CL507.Contig1\_Mf\_liverA, NM\_145824, Unigene15318\_Mf\_liverA, Unigene7412\_Mf\_liverA, CL425.Contig1\_Mf\_liverA, Unigene29308\_Mf\_liverA, Unigene34218\_Mf\_liverA, Unigene29008\_Mf\_liverA, CL4162.Contig1\_Mf\_liverA, CL5358.Contig1\_Mf\_liverA, NM\_010579, CL33.Contig4\_Mf\_liverA, Unigene673\_Mf\_liverA, Unigene50250\_Mf\_liverA, Unigene5165\_Mf\_liverA, Unigene7510\_Mf\_liverA, Unigene15656\_Mf\_liverA, Unigene24252\_Mf\_liverA, Unigene30707\_Mf\_liverA, Unigene25080\_Mf\_liverA, Unigene34219\_Mf\_liverA, Unigene4557\_Mf\_liverA, Unigene21255\_Mf\_liverA, CL1222.Contig1\_Mf\_liverA, Unigene25070\_Mf\_liverA, Unigene34789\_Mf\_liverA, NM\_009609, CL1810.Contig1\_Mf\_liverA, Unigene25057\_Mf\_liverA, CL33.Contig3\_Mf\_liverA, NM\_175260, Unigene5138\_Mf\_liverA, Unigene5940\_Mf\_liverA, CL5688.Contig1\_Mf\_liverA, CL2117.Contig1\_Mf\_liverA, Unigene25046\_Mf\_liverA, Unigene21857\_Mf\_liverA, Unigene24755\_Mf\_liverA, Unigene5941\_Mf\_liverA, Unigene2745\_Mf\_liverA, Unigene23869\_Mf\_liverA, Unigene34197\_Mf\_liverA, CL4847.Contig1\_Mf\_liverA, NM\_026823, Unigene28527\_Mf\_liverA, Unigene28822\_Mf\_liverA, NM\_053214, Unigene44317\_Mf\_liverA, NM\_177093, CL4722.Contig1\_Mf\_liverA, Unigene25047\_Mf\_liverA, NM\_009448, CL848.Contig2\_Mf\_liverA, Unigene23870\_Mf\_liverA, Unigene4768\_Mf\_liverA, CL3198.Contig1\_Mf\_liverA, Unigene25662\_Mf\_liverA, CL479.Contig1\_Mf\_liverA, Unigene14637\_Mf\_liverA, Unigene9475\_Mf\_liverA, Unigene14263\_Mf\_liverA, Unigene37662\_Mf\_liverA, Unigene27895\_Mf\_liverA, NM\_008538, Unigene15170\_Mf\_liverA, Unigene26250\_Mf\_liverA, Unigene48460\_Mf\_liverA, Unigene14896\_Mf\_liverA, Unigene43515\_Mf\_liverA, CL3803.Contig2\_Mf\_liverA, Unigene27547\_Mf\_liverA, Unigene16671\_Mf\_liverA, Unigene27260\_Mf\_liverA, NM\_134156, Unigene21317\_Mf\_liverA, CL795.Contig1\_Mf\_liverA, CL4434.Contig1\_Mf\_liverA, Unigene27082\_Mf\_liverA, Unigene2678\_Mf\_liverA, Unigene37076\_Mf\_liverA, CL5698.Contig1\_Mf\_liverA, Unigene37474\_Mf\_liverA, NM\_007392, CL5576.Contig1\_Mf\_liverA, Unigene26214\_Mf\_liverA, CL591.Contig1\_Mf\_liverA, Unigene2746\_Mf\_liverA, Unigene4556\_Mf\_liverA, Unigene15493\_Mf\_liverA |
| endosomal part | Unigene22330\_Mf\_liverA, Unigene13525\_Mf\_liverA, NM\_133838, Unigene24471\_Mf\_liverA, Unigene38015\_Mf\_liverA, Unigene5204\_Mf\_liverA, CL3835.Contig2\_Mf\_liverA, CL3002.Contig1\_Mf\_liverA, CL3055.Contig1\_Mf\_liverA, Unigene15592\_Mf\_liverA, CL5993.Contig3\_Mf\_liverA, Unigene4681\_Mf\_liverA, Unigene21857\_Mf\_liverA, Unigene5693\_Mf\_liverA, Unigene2746\_Mf\_liverA, Unigene30878\_Mf\_liverA, CL3685.Contig1\_Mf\_liverA, Unigene2745\_Mf\_liverA, Unigene4703\_Mf\_liverA, Unigene1440\_Mf\_liverA, CL3055.Contig2\_Mf\_liverA |
| Bcl3/NF-kappaB2 complex | Unigene152\_Mf\_liverA, Unigene7674\_Mf\_liverA |
| macrophage migration inhibitory factor receptor complex | CL2855.Contig1\_Mf\_liverA, CL2855.Contig2\_Mf\_liverA |
| NOS2-CD74 complex | CL2855.Contig1\_Mf\_liverA, CL2855.Contig2\_Mf\_liverA |
| actomyosin, actin part | Unigene4556\_Mf\_liverA, Unigene4557\_Mf\_liverA |
| protein complex | Unigene9466\_Mf\_liverA, NM\_176843, Unigene35884\_Mf\_liverA, NM\_007896, NM\_009898, CL3835.Contig2\_Mf\_liverA, NM\_145824, Unigene30356\_Mf\_liverA, Unigene24111\_Mf\_liverA, Unigene28186\_Mf\_liverA, Unigene38124\_Mf\_liverA, Unigene36851\_Mf\_liverA, Unigene29308\_Mf\_liverA, Unigene14665\_Mf\_liverA, NM\_010378, Unigene34218\_Mf\_liverA, Unigene5194\_Mf\_liverA, Unigene29008\_Mf\_liverA, Unigene22433\_Mf\_liverA, Unigene25090\_Mf\_liverA, CL2423.Contig1\_Mf\_liverA, Unigene32882\_Mf\_liverA, CL5358.Contig1\_Mf\_liverA, Unigene31263\_Mf\_liverA, Unigene2633\_Mf\_liverA, Unigene5248\_Mf\_liverA, Unigene7969\_Mf\_liverA, Unigene26422\_Mf\_liverA, CL33.Contig4\_Mf\_liverA, Unigene29334\_Mf\_liverA, Unigene30878\_Mf\_liverA, Unigene7510\_Mf\_liverA, CL4757.Contig1\_Mf\_liverA, Unigene34234\_Mf\_liverA, NR\_004446, Unigene38331\_Mf\_liverA, Unigene36889\_Mf\_liverA, Unigene5204\_Mf\_liverA, Unigene13307\_Mf\_liverA, Unigene4557\_Mf\_liverA, Unigene21255\_Mf\_liverA, Unigene34789\_Mf\_liverA, Unigene24112\_Mf\_liverA, CL4141.Contig1\_Mf\_liverA, Unigene25057\_Mf\_liverA, Unigene23273\_Mf\_liverA, Unigene37263\_Mf\_liverA, CL1119.Contig1\_Mf\_liverA, Unigene35816\_Mf\_liverA, Unigene13658\_Mf\_liverA, Unigene28331\_Mf\_liverA, Unigene31333\_Mf\_liverA, CL33.Contig3\_Mf\_liverA, Unigene4686\_Mf\_liverA, Unigene33054\_Mf\_liverA, Unigene11544\_Mf\_liverA, Unigene5138\_Mf\_liverA, Unigene37150\_Mf\_liverA, CL2117.Contig1\_Mf\_liverA, Unigene22432\_Mf\_liverA, Unigene25046\_Mf\_liverA, Unigene21857\_Mf\_liverA, Unigene5941\_Mf\_liverA, Unigene38657\_Mf\_liverA, Unigene2745\_Mf\_liverA, Unigene23869\_Mf\_liverA, CL4847.Contig1\_Mf\_liverA, Unigene28822\_Mf\_liverA, NM\_053214, CL2855.Contig2\_Mf\_liverA, Unigene20128\_Mf\_liverA, Unigene25091\_Mf\_liverA, NM\_019703, Unigene44317\_Mf\_liverA, NM\_177093, Unigene25047\_Mf\_liverA, CL4722.Contig1\_Mf\_liverA, Unigene14907\_Mf\_liverA, Unigene2919\_Mf\_liverA, Unigene23870\_Mf\_liverA, Unigene7476\_Mf\_liverA, Unigene1479\_Mf\_liverA, CL2131.Contig4\_Mf\_liverA, Unigene25976\_Mf\_liverA, Unigene25662\_Mf\_liverA, Unigene13143\_Mf\_liverA, CL3198.Contig1\_Mf\_liverA, Unigene5632\_Mf\_liverA, NM\_153505, Unigene31517\_Mf\_liverA, Unigene9475\_Mf\_liverA, Unigene37662\_Mf\_liverA, Unigene30459\_Mf\_liverA, Unigene13945\_Mf\_liverA, Unigene4523\_Mf\_liverA, Unigene27438\_Mf\_liverA, NM\_001143689, CL5631.Contig1\_Mf\_liverA, Unigene37575\_Mf\_liverA, Unigene43515\_Mf\_liverA, CL3803.Contig2\_Mf\_liverA, Unigene27547\_Mf\_liverA, Unigene19885\_Mf\_liverA, CL4040.Contig2\_Mf\_liverA, Unigene36845\_Mf\_liverA, NM\_010391, Unigene32716\_Mf\_liverA, Unigene42812\_Mf\_liverA, Unigene152\_Mf\_liverA, Unigene27082\_Mf\_liverA, Unigene5509\_Mf\_liverA, Unigene37076\_Mf\_liverA, Unigene2678\_Mf\_liverA, CL5039.Contig2\_Mf\_liverA, Unigene30288\_Mf\_liverA, NM\_010380, Unigene26214\_Mf\_liverA, CL5576.Contig1\_Mf\_liverA, CL2855.Contig1\_Mf\_liverA, Unigene2746\_Mf\_liverA, NM\_001025388, Unigene17569\_Mf\_liverA, Unigene37433\_Mf\_liverA, Unigene5472\_Mf\_liverA, CL1537.Contig1\_Mf\_liverA, CL4086.Contig1\_Mf\_liverA, NM\_011503, Unigene40610\_Mf\_liverA, CL507.Contig1\_Mf\_liverA, Unigene7412\_Mf\_liverA, CL425.Contig1\_Mf\_liverA, Unigene15982\_Mf\_liverA, CL4162.Contig1\_Mf\_liverA, Unigene37526\_Mf\_liverA, CL5993.Contig3\_Mf\_liverA, NM\_010579, Unigene29231\_Mf\_liverA, Unigene19083\_Mf\_liverA, Unigene7350\_Mf\_liverA, Unigene50250\_Mf\_liverA, Unigene4985\_Mf\_liverA, Unigene5165\_Mf\_liverA, Unigene1440\_Mf\_liverA, CL3800.Contig1\_Mf\_liverA, Unigene30707\_Mf\_liverA, Unigene13525\_Mf\_liverA, Unigene25080\_Mf\_liverA, Unigene34219\_Mf\_liverA, Unigene4938\_Mf\_liverA, Unigene36543\_Mf\_liverA, Unigene7674\_Mf\_liverA, CL3002.Contig1\_Mf\_liverA, CL1222.Contig1\_Mf\_liverA, CL1810.Contig1\_Mf\_liverA, NM\_009609, Unigene5758\_Mf\_liverA, Unigene27483\_Mf\_liverA, Unigene35935\_Mf\_liverA, Unigene29426\_Mf\_liverA, Unigene5940\_Mf\_liverA, NM\_007393, Unigene34446\_Mf\_liverA, NM\_033444, Unigene13683\_Mf\_liverA, Unigene24755\_Mf\_liverA, Unigene23271\_Mf\_liverA, Unigene13106\_Mf\_liverA, Unigene34197\_Mf\_liverA, Unigene24471\_Mf\_liverA, Unigene28527\_Mf\_liverA, Unigene5954\_Mf\_liverA, Unigene29938\_Mf\_liverA, NM\_009448, Unigene8740\_Mf\_liverA, CL2251.Contig1\_Mf\_liverA, Unigene4540\_Mf\_liverA, Unigene14263\_Mf\_liverA, Unigene37310\_Mf\_liverA, NM\_016861, Unigene4604\_Mf\_liverA, NM\_011418, Unigene26250\_Mf\_liverA, Unigene48460\_Mf\_liverA, Unigene39749\_Mf\_liverA, Unigene37245\_Mf\_liverA, Unigene16671\_Mf\_liverA, CL2355.Contig1\_Mf\_liverA, Unigene13535\_Mf\_liverA, Unigene37153\_Mf\_liverA, CL3104.Contig1\_Mf\_liverA, Unigene5422\_Mf\_liverA, Unigene7968\_Mf\_liverA, Unigene36667\_Mf\_liverA, CL4434.Contig1\_Mf\_liverA, Unigene5147\_Mf\_liverA, Unigene18340\_Mf\_liverA, Unigene4983\_Mf\_liverA, CL5698.Contig1\_Mf\_liverA, Unigene31392\_Mf\_liverA, NM\_023256, Unigene4681\_Mf\_liverA, Unigene37474\_Mf\_liverA, Unigene42855\_Mf\_liverA, NM\_007392, Unigene14284\_Mf\_liverA, CL591.Contig1\_Mf\_liverA, Unigene4556\_Mf\_liverA, Unigene15493\_Mf\_liverA |
| high-density lipoprotein particle | CL3911.Contig2\_Mf\_liverA, Unigene9081\_Mf\_liverA, CL1190.Contig3\_Mf\_liverA, Unigene30815\_Mf\_liverA, Unigene30814\_Mf\_liverA, Unigene24157\_Mf\_liverA |
| endosome | Unigene22330\_Mf\_liverA, Unigene24506\_Mf\_liverA, Unigene24471\_Mf\_liverA, Unigene38015\_Mf\_liverA, NM\_026823, CL3835.Contig2\_Mf\_liverA, CL507.Contig1\_Mf\_liverA, CL2855.Contig2\_Mf\_liverA, Unigene13414\_Mf\_liverA, CL5993.Contig3\_Mf\_liverA, Unigene24503\_Mf\_liverA, Unigene5693\_Mf\_liverA, Unigene30878\_Mf\_liverA, Unigene50250\_Mf\_liverA, Unigene4703\_Mf\_liverA, Unigene35958\_Mf\_liverA, Unigene1440\_Mf\_liverA, CL3055.Contig2\_Mf\_liverA, NR\_004446, Unigene13525\_Mf\_liverA, Unigene37245\_Mf\_liverA, NM\_133838, Unigene36328\_Mf\_liverA, Unigene5204\_Mf\_liverA, CL3002.Contig1\_Mf\_liverA, Unigene27260\_Mf\_liverA, NM\_010391, CL3055.Contig1\_Mf\_liverA, Unigene37389\_Mf\_liverA, Unigene15592\_Mf\_liverA, Unigene5745\_Mf\_liverA, NM\_015767, Unigene8132\_Mf\_liverA, Unigene46615\_Mf\_liverA, Unigene28331\_Mf\_liverA, Unigene13379\_Mf\_liverA, Unigene27082\_Mf\_liverA, CL1493.Contig2\_Mf\_liverA, CL442.Contig2\_Mf\_liverA, Unigene4681\_Mf\_liverA, CL1493.Contig1\_Mf\_liverA, Unigene21857\_Mf\_liverA, CL2855.Contig1\_Mf\_liverA, Unigene2746\_Mf\_liverA, CL3685.Contig1\_Mf\_liverA, Unigene2745\_Mf\_liverA |
| proteasome core complex | Unigene35884\_Mf\_liverA, Unigene24471\_Mf\_liverA, CL3835.Contig2\_Mf\_liverA, CL3002.Contig1\_Mf\_liverA, Unigene37153\_Mf\_liverA |
| cytoplasmic vesicle | Unigene29399\_Mf\_liverA, NM\_011503, NM\_009898, CL507.Contig1\_Mf\_liverA, Unigene5852\_Mf\_liverA, CL1828.Contig1\_Mf\_liverA, Unigene29424\_Mf\_liverA, Unigene29008\_Mf\_liverA, Unigene19049\_Mf\_liverA, Unigene30587\_Mf\_liverA, CL5993.Contig3\_Mf\_liverA, Unigene37259\_Mf\_liverA, NM\_010877, Unigene13271\_Mf\_liverA, Unigene38919\_Mf\_liverA, Unigene7510\_Mf\_liverA, Unigene35958\_Mf\_liverA, Unigene34234\_Mf\_liverA, Unigene34810\_Mf\_liverA, CL6038.Contig2\_Mf\_liverA, Unigene36328\_Mf\_liverA, Unigene5204\_Mf\_liverA, Unigene21255\_Mf\_liverA, NM\_008871, CL3055.Contig1\_Mf\_liverA, Unigene34789\_Mf\_liverA, CL6039.Contig2\_Mf\_liverA, CL5978.Contig3\_Mf\_liverA, Unigene28662\_Mf\_liverA, Unigene46615\_Mf\_liverA, Unigene28331\_Mf\_liverA, Unigene8033\_Mf\_liverA, Unigene4686\_Mf\_liverA, NM\_009776, Unigene11544\_Mf\_liverA, CL1493.Contig2\_Mf\_liverA, CL1493.Contig1\_Mf\_liverA, Unigene28094\_Mf\_liverA, Unigene28499\_Mf\_liverA, CL3685.Contig1\_Mf\_liverA, Unigene30585\_Mf\_liverA, Unigene24085\_Mf\_liverA, Unigene8054\_Mf\_liverA, CL4106.Contig1\_Mf\_liverA, Unigene22330\_Mf\_liverA, Unigene4909\_Mf\_liverA, Unigene13363\_Mf\_liverA, CL2855.Contig2\_Mf\_liverA, Unigene15529\_Mf\_liverA, Unigene28226\_Mf\_liverA, CL725.Contig1\_Mf\_liverA, Unigene33459\_Mf\_liverA, CL1190.Contig3\_Mf\_liverA, NM\_008808, CL2251.Contig1\_Mf\_liverA, CL5978.Contig2\_Mf\_liverA, Unigene8740\_Mf\_liverA, Unigene34258\_Mf\_liverA, Unigene4922\_Mf\_liverA, Unigene5632\_Mf\_liverA, Unigene9990\_Mf\_liverA, Unigene26941\_Mf\_liverA, CL3816.Contig1\_Mf\_liverA, NM\_011580, CL3055.Contig2\_Mf\_liverA, Unigene30584\_Mf\_liverA, Unigene24284\_Mf\_liverA, NM\_134156, Unigene37389\_Mf\_liverA, Unigene36673\_Mf\_liverA, Unigene27082\_Mf\_liverA, NM\_009713, CL3911.Contig2\_Mf\_liverA, Unigene21256\_Mf\_liverA, CL1738.Contig1\_Mf\_liverA, CL548.Contig1\_Mf\_liverA, CL6039.Contig1\_Mf\_liverA, Unigene37819\_Mf\_liverA, CL2855.Contig1\_Mf\_liverA, Unigene38237\_Mf\_liverA, Unigene28021\_Mf\_liverA |
| basement membrane | CL376.Contig1\_Mf\_liverA, Unigene18510\_Mf\_liverA, Unigene14582\_Mf\_liverA, Unigene23055\_Mf\_liverA, Unigene560\_Mf\_liverA, Unigene33271\_Mf\_liverA, Unigene4985\_Mf\_liverA, Unigene8132\_Mf\_liverA, Unigene34123\_Mf\_liverA, Unigene34124\_Mf\_liverA, Unigene34258\_Mf\_liverA, CL3232.Contig1\_Mf\_liverA, CL3411.Contig2\_Mf\_liverA, Unigene31492\_Mf\_liverA |
| cell-cell adherens junction | CL4434.Contig1\_Mf\_liverA, Unigene27082\_Mf\_liverA, CL507.Contig1\_Mf\_liverA, NM\_134156, CL2117.Contig1\_Mf\_liverA, NM\_001159724, Unigene26214\_Mf\_liverA, Unigene7733\_Mf\_liverA, Unigene2746\_Mf\_liverA, Unigene2745\_Mf\_liverA, Unigene39231\_Mf\_liverA |
| proteasome complex | Unigene35884\_Mf\_liverA, Unigene24471\_Mf\_liverA, CL3835.Contig2\_Mf\_liverA, CL3002.Contig1\_Mf\_liverA, Unigene37153\_Mf\_liverA, Unigene26250\_Mf\_liverA, CL4141.Contig1\_Mf\_liverA, Unigene36851\_Mf\_liverA, CL1119.Contig1\_Mf\_liverA |
| late endosome membrane | Unigene24471\_Mf\_liverA, Unigene5204\_Mf\_liverA, CL3835.Contig2\_Mf\_liverA, CL3002.Contig1\_Mf\_liverA, CL3055.Contig1\_Mf\_liverA, Unigene21857\_Mf\_liverA, Unigene5693\_Mf\_liverA, Unigene30878\_Mf\_liverA, CL3055.Contig2\_Mf\_liverA |
| myosin complex | Unigene5472\_Mf\_liverA, CL4434.Contig1\_Mf\_liverA, Unigene34197\_Mf\_liverA, Unigene37662\_Mf\_liverA, CL507.Contig1\_Mf\_liverA, Unigene28822\_Mf\_liverA, NM\_053214, Unigene24755\_Mf\_liverA, CL591.Contig1\_Mf\_liverA |
| membrane-bounded vesicle | Unigene29399\_Mf\_liverA, NM\_011503, NM\_009898, CL507.Contig1\_Mf\_liverA, Unigene5852\_Mf\_liverA, CL1828.Contig1\_Mf\_liverA, Unigene29424\_Mf\_liverA, Unigene29008\_Mf\_liverA, Unigene19049\_Mf\_liverA, Unigene30587\_Mf\_liverA, CL5993.Contig3\_Mf\_liverA, Unigene37259\_Mf\_liverA, NM\_010877, Unigene13271\_Mf\_liverA, Unigene38919\_Mf\_liverA, Unigene7510\_Mf\_liverA, Unigene35958\_Mf\_liverA, Unigene34234\_Mf\_liverA, Unigene34810\_Mf\_liverA, CL6038.Contig2\_Mf\_liverA, Unigene36328\_Mf\_liverA, Unigene5204\_Mf\_liverA, Unigene21255\_Mf\_liverA, NM\_008871, Unigene40020\_Mf\_liverA, CL3055.Contig1\_Mf\_liverA, Unigene34789\_Mf\_liverA, Unigene28662\_Mf\_liverA, Unigene46615\_Mf\_liverA, Unigene28331\_Mf\_liverA, Unigene4686\_Mf\_liverA, NM\_009776, Unigene11544\_Mf\_liverA, Unigene28094\_Mf\_liverA, Unigene28499\_Mf\_liverA, CL3685.Contig1\_Mf\_liverA, Unigene30585\_Mf\_liverA, Unigene24085\_Mf\_liverA, Unigene8054\_Mf\_liverA, Unigene22330\_Mf\_liverA, Unigene4909\_Mf\_liverA, Unigene13363\_Mf\_liverA, CL2855.Contig2\_Mf\_liverA, Unigene15529\_Mf\_liverA, Unigene28226\_Mf\_liverA, CL725.Contig1\_Mf\_liverA, CL1190.Contig3\_Mf\_liverA, NM\_008808, CL2251.Contig1\_Mf\_liverA, Unigene8740\_Mf\_liverA, Unigene34258\_Mf\_liverA, Unigene4922\_Mf\_liverA, Unigene5632\_Mf\_liverA, Unigene9990\_Mf\_liverA, Unigene26941\_Mf\_liverA, CL3816.Contig1\_Mf\_liverA, NM\_011580, CL3055.Contig2\_Mf\_liverA, Unigene30584\_Mf\_liverA, Unigene24284\_Mf\_liverA, NM\_134156, Unigene37389\_Mf\_liverA, Unigene2195\_Mf\_liverA, Unigene36673\_Mf\_liverA, Unigene27082\_Mf\_liverA, NM\_009713, CL3911.Contig2\_Mf\_liverA, Unigene21256\_Mf\_liverA, CL1738.Contig1\_Mf\_liverA, CL548.Contig1\_Mf\_liverA, Unigene37819\_Mf\_liverA, CL2855.Contig1\_Mf\_liverA, Unigene38237\_Mf\_liverA, Unigene28021\_Mf\_liverA |
| anchored to plasma membrane | CL4156.Contig1\_Mf\_liverA, Unigene21562\_Mf\_liverA, Unigene21561\_Mf\_liverA, Unigene24085\_Mf\_liverA, CL3575.Contig1\_Mf\_liverA, Unigene20128\_Mf\_liverA |
| endoplasmic reticulum membrane | Unigene34656\_Mf\_liverA, Unigene5598\_Mf\_liverA, NM\_009128, Unigene15888\_Mf\_liverA, NM\_010162, CL2855.Contig2\_Mf\_liverA, CL4127.Contig1\_Mf\_liverA, Unigene13414\_Mf\_liverA, CL2240.Contig1\_Mf\_liverA, CL5307.Contig1\_Mf\_liverA, CL2797.Contig2\_Mf\_liverA, Unigene1479\_Mf\_liverA, CL523.Contig1\_Mf\_liverA, Unigene4922\_Mf\_liverA, Unigene5632\_Mf\_liverA, CL482.Contig1\_Mf\_liverA, CL4490.Contig2\_Mf\_liverA, Unigene35237\_Mf\_liverA, CL5560.Contig1\_Mf\_liverA, Unigene139\_Mf\_liverA, Unigene22575\_Mf\_liverA, Unigene23185\_Mf\_liverA, Unigene23013\_Mf\_liverA, Unigene35046\_Mf\_liverA, CL4583.Contig2\_Mf\_liverA, Unigene36510\_Mf\_liverA, Unigene34341\_Mf\_liverA, CL1988.Contig3\_Mf\_liverA, Unigene37389\_Mf\_liverA, Unigene13772\_Mf\_liverA, Unigene36593\_Mf\_liverA, Unigene36699\_Mf\_liverA, Unigene29253\_Mf\_liverA, Unigene27082\_Mf\_liverA, Unigene5712\_Mf\_liverA, CL5698.Contig1\_Mf\_liverA, Unigene1280\_Mf\_liverA, Unigene1205\_Mf\_liverA, Unigene28731\_Mf\_liverA, CL2855.Contig1\_Mf\_liverA, CL2574.Contig1\_Mf\_liverA, Unigene5886\_Mf\_liverA, Unigene10135\_Mf\_liverA, Unigene542\_Mf\_liverA, Unigene25462\_Mf\_liverA, Unigene5648\_Mf\_liverA |
| zymogen granule | CL1828.Contig1\_Mf\_liverA, Unigene8054\_Mf\_liverA, Unigene15529\_Mf\_liverA, Unigene34810\_Mf\_liverA |
| cytoskeleton | NM\_009450, NM\_007896, Unigene34609\_Mf\_liverA, NM\_009898, Unigene25721\_Mf\_liverA, NM\_145824, Unigene29308\_Mf\_liverA, Unigene29424\_Mf\_liverA, Unigene34218\_Mf\_liverA, Unigene29008\_Mf\_liverA, CL5358.Contig1\_Mf\_liverA, NM\_011072, CL33.Contig4\_Mf\_liverA, Unigene7510\_Mf\_liverA, Unigene15656\_Mf\_liverA, Unigene24252\_Mf\_liverA, Unigene4557\_Mf\_liverA, Unigene21255\_Mf\_liverA, Unigene40020\_Mf\_liverA, Unigene25070\_Mf\_liverA, Unigene34789\_Mf\_liverA, Unigene25057\_Mf\_liverA, CL5191.Contig2\_Mf\_liverA, Unigene24379\_Mf\_liverA, CL33.Contig3\_Mf\_liverA, NM\_175260, Unigene5138\_Mf\_liverA, CL2117.Contig1\_Mf\_liverA, Unigene25046\_Mf\_liverA, Unigene21857\_Mf\_liverA, Unigene40824\_Mf\_liverA, Unigene5941\_Mf\_liverA, Unigene13419\_Mf\_liverA, Unigene2745\_Mf\_liverA, Unigene23869\_Mf\_liverA, CL4847.Contig1\_Mf\_liverA, Unigene28822\_Mf\_liverA, NM\_053214, Unigene44317\_Mf\_liverA, NM\_177093, CL4722.Contig1\_Mf\_liverA, Unigene25047\_Mf\_liverA, CL848.Contig2\_Mf\_liverA, Unigene23870\_Mf\_liverA, Unigene4768\_Mf\_liverA, CL3198.Contig1\_Mf\_liverA, Unigene25662\_Mf\_liverA, CL479.Contig1\_Mf\_liverA, Unigene14637\_Mf\_liverA, Unigene9475\_Mf\_liverA, Unigene37662\_Mf\_liverA, Unigene27895\_Mf\_liverA, NM\_008538, Unigene36631\_Mf\_liverA, Unigene14896\_Mf\_liverA, CL3803.Contig2\_Mf\_liverA, Unigene43515\_Mf\_liverA, Unigene27547\_Mf\_liverA, Unigene24284\_Mf\_liverA, Unigene27260\_Mf\_liverA, NM\_134156, NM\_010227, Unigene21317\_Mf\_liverA, Unigene37470\_Mf\_liverA, CL795.Contig1\_Mf\_liverA, Unigene27082\_Mf\_liverA, Unigene31852\_Mf\_liverA, Unigene35169\_Mf\_liverA, Unigene2678\_Mf\_liverA, Unigene37076\_Mf\_liverA, CL1352.Contig1\_Mf\_liverA, CL5576.Contig1\_Mf\_liverA, Unigene26214\_Mf\_liverA, Unigene2746\_Mf\_liverA, NM\_009447, Unigene5472\_Mf\_liverA, CL1537.Contig1\_Mf\_liverA, CL507.Contig1\_Mf\_liverA, Unigene7412\_Mf\_liverA, Unigene15318\_Mf\_liverA, CL425.Contig1\_Mf\_liverA, CL4162.Contig1\_Mf\_liverA, NM\_010579, Unigene673\_Mf\_liverA, Unigene5693\_Mf\_liverA, Unigene13271\_Mf\_liverA, Unigene50250\_Mf\_liverA, Unigene5165\_Mf\_liverA, Unigene30707\_Mf\_liverA, Unigene25080\_Mf\_liverA, Unigene34219\_Mf\_liverA, CL1222.Contig1\_Mf\_liverA, CL1810.Contig1\_Mf\_liverA, NM\_009609, Unigene5940\_Mf\_liverA, NM\_007393, CL5688.Contig1\_Mf\_liverA, Unigene24755\_Mf\_liverA, Unigene28499\_Mf\_liverA, Unigene34197\_Mf\_liverA, Unigene35168\_Mf\_liverA, NM\_026823, Unigene8016\_Mf\_liverA, Unigene28527\_Mf\_liverA, NM\_009448, Unigene1327\_Mf\_liverA, CL2251.Contig1\_Mf\_liverA, Unigene14263\_Mf\_liverA, Unigene15170\_Mf\_liverA, Unigene26250\_Mf\_liverA, Unigene24477\_Mf\_liverA, Unigene20432\_Mf\_liverA, Unigene48460\_Mf\_liverA, NM\_017379, Unigene550\_Mf\_liverA, Unigene16671\_Mf\_liverA, Unigene29302\_Mf\_liverA, Unigene26309\_Mf\_liverA, Unigene2195\_Mf\_liverA, NM\_146016, Unigene36673\_Mf\_liverA, CL4434.Contig1\_Mf\_liverA, Unigene30412\_Mf\_liverA, Unigene31885\_Mf\_liverA, CL5698.Contig1\_Mf\_liverA, CL373.Contig7\_Mf\_liverA, Unigene37474\_Mf\_liverA, NM\_007392, CL591.Contig1\_Mf\_liverA, Unigene4556\_Mf\_liverA, Unigene15493\_Mf\_liverA |
| ruffle | Unigene30707\_Mf\_liverA, Unigene25080\_Mf\_liverA, Unigene5175\_Mf\_liverA, Unigene16671\_Mf\_liverA, CL507.Contig1\_Mf\_liverA, Unigene29938\_Mf\_liverA, Unigene51055\_Mf\_liverA, Unigene24379\_Mf\_liverA, Unigene4697\_Mf\_liverA, CL795.Contig1\_Mf\_liverA, CL4434.Contig1\_Mf\_liverA, Unigene5138\_Mf\_liverA, Unigene31885\_Mf\_liverA, CL2117.Contig1\_Mf\_liverA, Unigene5693\_Mf\_liverA, Unigene14896\_Mf\_liverA, Unigene24252\_Mf\_liverA |
| adherens junction | Unigene30707\_Mf\_liverA, Unigene25080\_Mf\_liverA, Unigene39875\_Mf\_liverA, Unigene24284\_Mf\_liverA, Unigene16671\_Mf\_liverA, CL507.Contig1\_Mf\_liverA, NM\_134156, Unigene29938\_Mf\_liverA, NM\_001159724, Unigene29302\_Mf\_liverA, Unigene27483\_Mf\_liverA, Unigene5422\_Mf\_liverA, Unigene29424\_Mf\_liverA, Unigene28331\_Mf\_liverA, CL4434.Contig1\_Mf\_liverA, Unigene27082\_Mf\_liverA, Unigene31852\_Mf\_liverA, CL2117.Contig1\_Mf\_liverA, Unigene26214\_Mf\_liverA, Unigene7733\_Mf\_liverA, Unigene40824\_Mf\_liverA, Unigene2746\_Mf\_liverA, Unigene2745\_Mf\_liverA, Unigene39231\_Mf\_liverA, Unigene15656\_Mf\_liverA |
| intrinsic to external side of plasma membrane | CL4156.Contig1\_Mf\_liverA, Unigene21562\_Mf\_liverA, Unigene21561\_Mf\_liverA, Unigene24085\_Mf\_liverA |
| complement component C1 complex | CL4040.Contig2\_Mf\_liverA, Unigene13106\_Mf\_liverA |
| actomyosin contractile ring | CL4434.Contig1\_Mf\_liverA, CL507.Contig1\_Mf\_liverA |
| proteasome activator complex | Unigene36851\_Mf\_liverA, CL1119.Contig1\_Mf\_liverA |
| vesicle | Unigene29399\_Mf\_liverA, NM\_011503, NM\_009898, CL507.Contig1\_Mf\_liverA, Unigene5852\_Mf\_liverA, CL1828.Contig1\_Mf\_liverA, Unigene15318\_Mf\_liverA, Unigene29424\_Mf\_liverA, Unigene29008\_Mf\_liverA, Unigene19049\_Mf\_liverA, Unigene30587\_Mf\_liverA, CL5993.Contig3\_Mf\_liverA, Unigene37259\_Mf\_liverA, NM\_010877, Unigene13271\_Mf\_liverA, Unigene38919\_Mf\_liverA, Unigene7510\_Mf\_liverA, Unigene35958\_Mf\_liverA, Unigene34234\_Mf\_liverA, Unigene34810\_Mf\_liverA, CL6038.Contig2\_Mf\_liverA, Unigene36328\_Mf\_liverA, Unigene5204\_Mf\_liverA, Unigene21255\_Mf\_liverA, NM\_008871, Unigene40020\_Mf\_liverA, CL3055.Contig1\_Mf\_liverA, Unigene34789\_Mf\_liverA, CL6039.Contig2\_Mf\_liverA, CL5978.Contig3\_Mf\_liverA, Unigene28662\_Mf\_liverA, Unigene46615\_Mf\_liverA, Unigene28331\_Mf\_liverA, Unigene8033\_Mf\_liverA, Unigene4686\_Mf\_liverA, NM\_009776, Unigene11544\_Mf\_liverA, CL1493.Contig2\_Mf\_liverA, CL1493.Contig1\_Mf\_liverA, Unigene28094\_Mf\_liverA, Unigene28499\_Mf\_liverA, CL3685.Contig1\_Mf\_liverA, Unigene30585\_Mf\_liverA, Unigene24085\_Mf\_liverA, Unigene8054\_Mf\_liverA, CL4106.Contig1\_Mf\_liverA, Unigene22330\_Mf\_liverA, Unigene4909\_Mf\_liverA, Unigene13363\_Mf\_liverA, CL2855.Contig2\_Mf\_liverA, Unigene15529\_Mf\_liverA, Unigene28226\_Mf\_liverA, CL725.Contig1\_Mf\_liverA, Unigene33459\_Mf\_liverA, CL1190.Contig3\_Mf\_liverA, NM\_008808, CL2251.Contig1\_Mf\_liverA, CL5978.Contig2\_Mf\_liverA, Unigene8740\_Mf\_liverA, Unigene34258\_Mf\_liverA, Unigene4922\_Mf\_liverA, Unigene5632\_Mf\_liverA, CL479.Contig1\_Mf\_liverA, Unigene9990\_Mf\_liverA, Unigene26941\_Mf\_liverA, CL3816.Contig1\_Mf\_liverA, NM\_011580, CL3055.Contig2\_Mf\_liverA, Unigene30584\_Mf\_liverA, Unigene24284\_Mf\_liverA, NM\_134156, Unigene37389\_Mf\_liverA, Unigene8132\_Mf\_liverA, Unigene2195\_Mf\_liverA, Unigene36673\_Mf\_liverA, Unigene27082\_Mf\_liverA, NM\_009713, CL3911.Contig2\_Mf\_liverA, Unigene21256\_Mf\_liverA, CL1738.Contig1\_Mf\_liverA, CL548.Contig1\_Mf\_liverA, CL6039.Contig1\_Mf\_liverA, Unigene16891\_Mf\_liverA, Unigene37819\_Mf\_liverA, CL2855.Contig1\_Mf\_liverA, Unigene38237\_Mf\_liverA, Unigene28021\_Mf\_liverA |
| filamentous actin | Unigene37474\_Mf\_liverA, Unigene26214\_Mf\_liverA, Unigene34197\_Mf\_liverA, Unigene28822\_Mf\_liverA, CL1222.Contig1\_Mf\_liverA |
| nuclear outer membrane-endoplasmic reticulum membrane network | Unigene34656\_Mf\_liverA, Unigene5598\_Mf\_liverA, NM\_009128, Unigene15888\_Mf\_liverA, NM\_010162, CL2855.Contig2\_Mf\_liverA, CL4127.Contig1\_Mf\_liverA, Unigene13414\_Mf\_liverA, CL2240.Contig1\_Mf\_liverA, CL5307.Contig1\_Mf\_liverA, CL2797.Contig2\_Mf\_liverA, Unigene1479\_Mf\_liverA, CL523.Contig1\_Mf\_liverA, Unigene4922\_Mf\_liverA, Unigene5632\_Mf\_liverA, CL482.Contig1\_Mf\_liverA, CL4490.Contig2\_Mf\_liverA, Unigene35237\_Mf\_liverA, CL5560.Contig1\_Mf\_liverA, Unigene139\_Mf\_liverA, Unigene22575\_Mf\_liverA, Unigene23185\_Mf\_liverA, Unigene23013\_Mf\_liverA, Unigene35046\_Mf\_liverA, CL4583.Contig2\_Mf\_liverA, Unigene36510\_Mf\_liverA, Unigene34341\_Mf\_liverA, CL1988.Contig3\_Mf\_liverA, Unigene37389\_Mf\_liverA, Unigene13772\_Mf\_liverA, Unigene36593\_Mf\_liverA, Unigene36699\_Mf\_liverA, Unigene29253\_Mf\_liverA, Unigene27082\_Mf\_liverA, Unigene5712\_Mf\_liverA, CL5698.Contig1\_Mf\_liverA, Unigene1280\_Mf\_liverA, Unigene1205\_Mf\_liverA, Unigene28731\_Mf\_liverA, CL2855.Contig1\_Mf\_liverA, CL2574.Contig1\_Mf\_liverA, Unigene5886\_Mf\_liverA, Unigene10135\_Mf\_liverA, Unigene542\_Mf\_liverA, Unigene25462\_Mf\_liverA, Unigene5648\_Mf\_liverA |
| Weibel-Palade body | Unigene30587\_Mf\_liverA, Unigene30585\_Mf\_liverA, Unigene30584\_Mf\_liverA |
| pre-autophagosomal structure membrane | CL3685.Contig1\_Mf\_liverA, Unigene13307\_Mf\_liverA, Unigene13535\_Mf\_liverA |
| endosome membrane | Unigene22330\_Mf\_liverA, NM\_133838, Unigene24471\_Mf\_liverA, Unigene38015\_Mf\_liverA, Unigene5204\_Mf\_liverA, CL3835.Contig2\_Mf\_liverA, CL3002.Contig1\_Mf\_liverA, CL3055.Contig1\_Mf\_liverA, CL5993.Contig3\_Mf\_liverA, Unigene4681\_Mf\_liverA, Unigene21857\_Mf\_liverA, Unigene5693\_Mf\_liverA, Unigene30878\_Mf\_liverA, CL3685.Contig1\_Mf\_liverA, Unigene2746\_Mf\_liverA, Unigene2745\_Mf\_liverA, Unigene4703\_Mf\_liverA, Unigene1440\_Mf\_liverA, CL3055.Contig2\_Mf\_liverA |
| cortical actin cytoskeleton | Unigene30707\_Mf\_liverA, Unigene26214\_Mf\_liverA, CL848.Contig2\_Mf\_liverA, Unigene28822\_Mf\_liverA, CL795.Contig1\_Mf\_liverA, Unigene15656\_Mf\_liverA, Unigene29008\_Mf\_liverA |
| sarcolemma | Unigene27547\_Mf\_liverA, Unigene9475\_Mf\_liverA, CL4847.Contig1\_Mf\_liverA, Unigene2678\_Mf\_liverA, CL725.Contig1\_Mf\_liverA, CL3816.Contig1\_Mf\_liverA, CL2722.Contig1\_Mf\_liverA, Unigene37278\_Mf\_liverA, Unigene28094\_Mf\_liverA, Unigene32716\_Mf\_liverA, CL425.Contig1\_Mf\_liverA, Unigene28331\_Mf\_liverA |
| zymogen granule membrane | CL1828.Contig1\_Mf\_liverA, Unigene8054\_Mf\_liverA, Unigene34810\_Mf\_liverA |
| actomyosin | CL4434.Contig1\_Mf\_liverA, Unigene14637\_Mf\_liverA, Unigene34197\_Mf\_liverA, Unigene24755\_Mf\_liverA, Unigene4556\_Mf\_liverA, Unigene4557\_Mf\_liverA, CL507.Contig1\_Mf\_liverA, Unigene14896\_Mf\_liverA |
| neuronal cell body | Unigene29399\_Mf\_liverA, Unigene13525\_Mf\_liverA, Unigene27547\_Mf\_liverA, Unigene28527\_Mf\_liverA, Unigene4720\_Mf\_liverA, Unigene34789\_Mf\_liverA, Unigene7950\_Mf\_liverA, Unigene5037\_Mf\_liverA, CL425.Contig1\_Mf\_liverA, Unigene8132\_Mf\_liverA, Unigene8740\_Mf\_liverA, Unigene5194\_Mf\_liverA, Unigene4686\_Mf\_liverA, Unigene34866\_Mf\_liverA, Unigene5712\_Mf\_liverA, Unigene5138\_Mf\_liverA, Unigene14582\_Mf\_liverA, Unigene9990\_Mf\_liverA, CL2117.Contig1\_Mf\_liverA, Unigene37259\_Mf\_liverA, Unigene26214\_Mf\_liverA, Unigene35269\_Mf\_liverA, Unigene38657\_Mf\_liverA |
| endomembrane system | Unigene26515\_Mf\_liverA, Unigene26055\_Mf\_liverA, Unigene28142\_Mf\_liverA, Unigene34656\_Mf\_liverA, NM\_009898, NM\_009128, CL1828.Contig1\_Mf\_liverA, Unigene13414\_Mf\_liverA, NM\_009178, CL2240.Contig1\_Mf\_liverA, NM\_018815, NM\_080638, CL2797.Contig2\_Mf\_liverA, CL523.Contig1\_Mf\_liverA, Unigene29424\_Mf\_liverA, Unigene15474\_Mf\_liverA, Unigene26053\_Mf\_liverA, Unigene29008\_Mf\_liverA, CL482.Contig1\_Mf\_liverA, CL4162.Contig1\_Mf\_liverA, Unigene35237\_Mf\_liverA, CL5993.Contig3\_Mf\_liverA, CL5560.Contig1\_Mf\_liverA, Unigene22575\_Mf\_liverA, Unigene23013\_Mf\_liverA, Unigene34234\_Mf\_liverA, Unigene43037\_Mf\_liverA, NR\_004446, Unigene34810\_Mf\_liverA, CL4583.Contig2\_Mf\_liverA, CL5586.Contig1\_Mf\_liverA, Unigene36328\_Mf\_liverA, Unigene5204\_Mf\_liverA, Unigene21255\_Mf\_liverA, Unigene40020\_Mf\_liverA, Unigene34341\_Mf\_liverA, CL1988.Contig3\_Mf\_liverA, Unigene25057\_Mf\_liverA, Unigene15703\_Mf\_liverA, CL4033.Contig1\_Mf\_liverA, Unigene28331\_Mf\_liverA, Unigene4686\_Mf\_liverA, NM\_010763, CL1493.Contig2\_Mf\_liverA, Unigene28731\_Mf\_liverA, CL2117.Contig1\_Mf\_liverA, CL1493.Contig1\_Mf\_liverA, NM\_033444, Unigene28094\_Mf\_liverA, Unigene28499\_Mf\_liverA, CL2574.Contig1\_Mf\_liverA, CL3685.Contig1\_Mf\_liverA, Unigene24085\_Mf\_liverA, Unigene8054\_Mf\_liverA, Unigene25462\_Mf\_liverA, Unigene542\_Mf\_liverA, Unigene10135\_Mf\_liverA, CL4106.Contig1\_Mf\_liverA, CL787.Contig1\_Mf\_liverA, Unigene4909\_Mf\_liverA, Unigene34197\_Mf\_liverA, Unigene5598\_Mf\_liverA, NM\_010162, Unigene15888\_Mf\_liverA, CL2855.Contig2\_Mf\_liverA, Unigene15529\_Mf\_liverA, Unigene38406\_Mf\_liverA, CL4127.Contig1\_Mf\_liverA, CL5307.Contig1\_Mf\_liverA, Unigene51055\_Mf\_liverA, Unigene1479\_Mf\_liverA, NM\_008808, Unigene4922\_Mf\_liverA, Unigene5632\_Mf\_liverA, CL4490.Contig2\_Mf\_liverA, NM\_007478, Unigene9990\_Mf\_liverA, Unigene14212\_Mf\_liverA, Unigene139\_Mf\_liverA, CL3816.Contig1\_Mf\_liverA, Unigene1221\_Mf\_liverA, Unigene23185\_Mf\_liverA, Unigene35046\_Mf\_liverA, Unigene550\_Mf\_liverA, Unigene36510\_Mf\_liverA, Unigene281\_Mf\_liverA, NM\_134156, NM\_010391, Unigene37389\_Mf\_liverA, Unigene13772\_Mf\_liverA, Unigene5687\_Mf\_liverA, Unigene36593\_Mf\_liverA, Unigene8132\_Mf\_liverA, CL5254.Contig1\_Mf\_liverA, Unigene36699\_Mf\_liverA, Unigene29253\_Mf\_liverA, Unigene34010\_Mf\_liverA, Unigene42812\_Mf\_liverA, NM\_181517, Unigene27082\_Mf\_liverA, Unigene5456\_Mf\_liverA, Unigene5712\_Mf\_liverA, Unigene30412\_Mf\_liverA, CL5698.Contig1\_Mf\_liverA, Unigene1280\_Mf\_liverA, Unigene21256\_Mf\_liverA, Unigene1205\_Mf\_liverA, CL548.Contig1\_Mf\_liverA, Unigene16891\_Mf\_liverA, CL2855.Contig1\_Mf\_liverA, Unigene5886\_Mf\_liverA, Unigene5648\_Mf\_liverA |
| stress fiber | CL4434.Contig1\_Mf\_liverA, Unigene14637\_Mf\_liverA, Unigene34197\_Mf\_liverA, Unigene24755\_Mf\_liverA, Unigene4556\_Mf\_liverA, CL507.Contig1\_Mf\_liverA, Unigene14896\_Mf\_liverA |
| contractile fiber | CL4847.Contig1\_Mf\_liverA, Unigene4557\_Mf\_liverA, CL1222.Contig1\_Mf\_liverA, NM\_134156, NM\_009609, NM\_177093, Unigene8132\_Mf\_liverA, CL2251.Contig1\_Mf\_liverA, Unigene27082\_Mf\_liverA, Unigene9475\_Mf\_liverA, NM\_007478, Unigene2678\_Mf\_liverA, Unigene37662\_Mf\_liverA, NM\_008610, Unigene26250\_Mf\_liverA, Unigene37474\_Mf\_liverA, CL1352.Contig1\_Mf\_liverA, NM\_007392, Unigene24755\_Mf\_liverA, Unigene4556\_Mf\_liverA, NM\_001025388 |
| cell leading edge | Unigene34197\_Mf\_liverA, NM\_011503, NM\_009898, CL507.Contig1\_Mf\_liverA, Unigene29938\_Mf\_liverA, Unigene51055\_Mf\_liverA, Unigene23870\_Mf\_liverA, Unigene4697\_Mf\_liverA, Unigene29008\_Mf\_liverA, Unigene39011\_Mf\_liverA, Unigene5693\_Mf\_liverA, Unigene5165\_Mf\_liverA, Unigene14896\_Mf\_liverA, Unigene24252\_Mf\_liverA, Unigene30707\_Mf\_liverA, Unigene25080\_Mf\_liverA, Unigene5175\_Mf\_liverA, Unigene16671\_Mf\_liverA, Unigene29302\_Mf\_liverA, Unigene24379\_Mf\_liverA, CL795.Contig1\_Mf\_liverA, CL4434.Contig1\_Mf\_liverA, Unigene31852\_Mf\_liverA, Unigene37076\_Mf\_liverA, Unigene5138\_Mf\_liverA, Unigene31885\_Mf\_liverA, NM\_053072, CL2117.Contig1\_Mf\_liverA, CL1352.Contig1\_Mf\_liverA, Unigene40824\_Mf\_liverA, NM\_007986, Unigene23869\_Mf\_liverA |
| intermediate filament cytoskeleton | Unigene27082\_Mf\_liverA, CL4162.Contig1\_Mf\_liverA, CL1537.Contig1\_Mf\_liverA, Unigene9475\_Mf\_liverA, CL4847.Contig1\_Mf\_liverA, Unigene37076\_Mf\_liverA, Unigene2678\_Mf\_liverA, Unigene5940\_Mf\_liverA, NM\_010579, Unigene34789\_Mf\_liverA, Unigene5941\_Mf\_liverA, Unigene5165\_Mf\_liverA |
| I-kappaB/NF-kappaB complex | CL3800.Contig1\_Mf\_liverA, Unigene152\_Mf\_liverA, Unigene7674\_Mf\_liverA |
| microvillus membrane | Unigene25080\_Mf\_liverA, Unigene34197\_Mf\_liverA, Unigene16671\_Mf\_liverA, Unigene29424\_Mf\_liverA |
| secretory granule membrane | Unigene4909\_Mf\_liverA, Unigene5204\_Mf\_liverA, NM\_134156, Unigene9990\_Mf\_liverA, CL3816.Contig1\_Mf\_liverA, CL1828.Contig1\_Mf\_liverA, Unigene8054\_Mf\_liverA, Unigene29424\_Mf\_liverA, Unigene28331\_Mf\_liverA, Unigene34810\_Mf\_liverA |
| varicosity | Unigene34866\_Mf\_liverA, Unigene4720\_Mf\_liverA |
| focal adhesion | Unigene25080\_Mf\_liverA, Unigene31852\_Mf\_liverA, Unigene24284\_Mf\_liverA, Unigene16671\_Mf\_liverA, Unigene29938\_Mf\_liverA, Unigene29302\_Mf\_liverA, Unigene40824\_Mf\_liverA, Unigene27483\_Mf\_liverA, Unigene5422\_Mf\_liverA, Unigene29424\_Mf\_liverA, Unigene15656\_Mf\_liverA, Unigene28331\_Mf\_liverA |
| anchoring junction | Unigene30707\_Mf\_liverA, Unigene25080\_Mf\_liverA, Unigene39875\_Mf\_liverA, Unigene24284\_Mf\_liverA, Unigene16671\_Mf\_liverA, CL507.Contig1\_Mf\_liverA, NM\_134156, Unigene29938\_Mf\_liverA, NM\_001159724, Unigene29302\_Mf\_liverA, Unigene27483\_Mf\_liverA, Unigene5422\_Mf\_liverA, Unigene29424\_Mf\_liverA, Unigene28331\_Mf\_liverA, CL4434.Contig1\_Mf\_liverA, Unigene27082\_Mf\_liverA, Unigene31852\_Mf\_liverA, CL2117.Contig1\_Mf\_liverA, Unigene26214\_Mf\_liverA, Unigene7733\_Mf\_liverA, Unigene40824\_Mf\_liverA, Unigene2746\_Mf\_liverA, Unigene2745\_Mf\_liverA, Unigene39231\_Mf\_liverA, Unigene15656\_Mf\_liverA |
| neuron projection | Unigene29399\_Mf\_liverA, Unigene34197\_Mf\_liverA, Unigene31251\_Mf\_liverA, Unigene28527\_Mf\_liverA, Unigene13363\_Mf\_liverA, Unigene36417\_Mf\_liverA, NM\_177093, CL4722.Contig1\_Mf\_liverA, CL425.Contig1\_Mf\_liverA, Unigene21561\_Mf\_liverA, Unigene23870\_Mf\_liverA, CL1190.Contig3\_Mf\_liverA, Unigene8740\_Mf\_liverA, Unigene5194\_Mf\_liverA, CL479.Contig1\_Mf\_liverA, Unigene14582\_Mf\_liverA, Unigene9990\_Mf\_liverA, Unigene13945\_Mf\_liverA, NM\_011305, Unigene37259\_Mf\_liverA, Unigene29231\_Mf\_liverA, Unigene36631\_Mf\_liverA, Unigene36418\_Mf\_liverA, Unigene5165\_Mf\_liverA, Unigene7510\_Mf\_liverA, Unigene14896\_Mf\_liverA, Unigene24252\_Mf\_liverA, Unigene30707\_Mf\_liverA, CL4583.Contig2\_Mf\_liverA, Unigene13525\_Mf\_liverA, Unigene36414\_Mf\_liverA, Unigene27547\_Mf\_liverA, Unigene37488\_Mf\_liverA, Unigene27260\_Mf\_liverA, CL1222.Contig1\_Mf\_liverA, NM\_134156, Unigene4720\_Mf\_liverA, CL1810.Contig1\_Mf\_liverA, NM\_009609, Unigene34789\_Mf\_liverA, Unigene21562\_Mf\_liverA, Unigene5037\_Mf\_liverA, Unigene8132\_Mf\_liverA, Unigene24547\_Mf\_liverA, NM\_175260, Unigene13940\_Mf\_liverA, Unigene4686\_Mf\_liverA, Unigene34866\_Mf\_liverA, Unigene13396\_Mf\_liverA, Unigene5712\_Mf\_liverA, Unigene37076\_Mf\_liverA, NM\_007393, CL2117.Contig1\_Mf\_liverA, CL1738.Contig1\_Mf\_liverA, CL1352.Contig1\_Mf\_liverA, Unigene36420\_Mf\_liverA, Unigene35269\_Mf\_liverA, Unigene23869\_Mf\_liverA |
| filopodium | Unigene30707\_Mf\_liverA, Unigene25080\_Mf\_liverA, Unigene31852\_Mf\_liverA, Unigene16671\_Mf\_liverA, Unigene39011\_Mf\_liverA, CL1352.Contig1\_Mf\_liverA, Unigene29424\_Mf\_liverA, Unigene14896\_Mf\_liverA |
| ruffle membrane | Unigene30707\_Mf\_liverA, Unigene25080\_Mf\_liverA, Unigene5693\_Mf\_liverA, Unigene16671\_Mf\_liverA, Unigene5138\_Mf\_liverA, Unigene29938\_Mf\_liverA, CL795.Contig1\_Mf\_liverA, CL2117.Contig1\_Mf\_liverA |
| lateral plasma membrane | Unigene27082\_Mf\_liverA, Unigene34197\_Mf\_liverA, Unigene23870\_Mf\_liverA, CL507.Contig1\_Mf\_liverA, CL2117.Contig1\_Mf\_liverA, Unigene23869\_Mf\_liverA |
| growth cone | Unigene13396\_Mf\_liverA, Unigene31251\_Mf\_liverA, Unigene14582\_Mf\_liverA, CL2117.Contig1\_Mf\_liverA, CL1352.Contig1\_Mf\_liverA, Unigene21562\_Mf\_liverA, Unigene21561\_Mf\_liverA, Unigene24547\_Mf\_liverA, CL1190.Contig3\_Mf\_liverA, Unigene14896\_Mf\_liverA |
| contractile ring | CL4434.Contig1\_Mf\_liverA, CL507.Contig1\_Mf\_liverA |
| actin filament bundle | CL4434.Contig1\_Mf\_liverA, Unigene14637\_Mf\_liverA, Unigene34197\_Mf\_liverA, CL507.Contig1\_Mf\_liverA, NM\_134156, NM\_007392, Unigene24755\_Mf\_liverA, Unigene4556\_Mf\_liverA, Unigene14896\_Mf\_liverA, NM\_175260 |
| anaphase-promoting complex | Unigene5248\_Mf\_liverA, Unigene48460\_Mf\_liverA, Unigene29334\_Mf\_liverA, Unigene5954\_Mf\_liverA |
| myosin II complex | CL4434.Contig1\_Mf\_liverA, Unigene24755\_Mf\_liverA, Unigene37662\_Mf\_liverA, CL507.Contig1\_Mf\_liverA |
| cytoplasmic microtubule | CL33.Contig4\_Mf\_liverA, Unigene7510\_Mf\_liverA, CL2117.Contig1\_Mf\_liverA, CL33.Contig3\_Mf\_liverA, Unigene43515\_Mf\_liverA |
| cell projection | Unigene29399\_Mf\_liverA, Unigene31251\_Mf\_liverA, CL507.Contig1\_Mf\_liverA, CL425.Contig1\_Mf\_liverA, Unigene29424\_Mf\_liverA, Unigene4697\_Mf\_liverA, Unigene5194\_Mf\_liverA, Unigene29008\_Mf\_liverA, Unigene37259\_Mf\_liverA, Unigene29231\_Mf\_liverA, Unigene5693\_Mf\_liverA, Unigene5165\_Mf\_liverA, Unigene7510\_Mf\_liverA, Unigene24252\_Mf\_liverA, Unigene30707\_Mf\_liverA, CL4583.Contig2\_Mf\_liverA, Unigene13525\_Mf\_liverA, Unigene36414\_Mf\_liverA, Unigene25080\_Mf\_liverA, Unigene5175\_Mf\_liverA, Unigene37488\_Mf\_liverA, Unigene36328\_Mf\_liverA, CL1222.Contig1\_Mf\_liverA, Unigene34789\_Mf\_liverA, NM\_009609, CL1810.Contig1\_Mf\_liverA, Unigene24379\_Mf\_liverA, Unigene24547\_Mf\_liverA, Unigene28331\_Mf\_liverA, Unigene13940\_Mf\_liverA, NM\_175260, Unigene4686\_Mf\_liverA, Unigene5138\_Mf\_liverA, NM\_007393, CL1493.Contig2\_Mf\_liverA, Unigene34446\_Mf\_liverA, CL2117.Contig1\_Mf\_liverA, CL1493.Contig1\_Mf\_liverA, NM\_033444, Unigene40824\_Mf\_liverA, Unigene35269\_Mf\_liverA, Unigene36420\_Mf\_liverA, Unigene24085\_Mf\_liverA, NM\_007986, Unigene23869\_Mf\_liverA, Unigene34197\_Mf\_liverA, Unigene13363\_Mf\_liverA, Unigene28527\_Mf\_liverA, Unigene29938\_Mf\_liverA, Unigene36417\_Mf\_liverA, NM\_177093, CL4722.Contig1\_Mf\_liverA, Unigene37278\_Mf\_liverA, Unigene51055\_Mf\_liverA, Unigene23870\_Mf\_liverA, Unigene21561\_Mf\_liverA, CL1190.Contig3\_Mf\_liverA, NM\_008808, CL2251.Contig1\_Mf\_liverA, Unigene8740\_Mf\_liverA, CL479.Contig1\_Mf\_liverA, Unigene14582\_Mf\_liverA, Unigene27895\_Mf\_liverA, Unigene13945\_Mf\_liverA, NM\_011305, Unigene9990\_Mf\_liverA, Unigene39011\_Mf\_liverA, Unigene36631\_Mf\_liverA, Unigene36418\_Mf\_liverA, Unigene14896\_Mf\_liverA, Unigene27249\_Mf\_liverA, Unigene27547\_Mf\_liverA, Unigene16671\_Mf\_liverA, Unigene27260\_Mf\_liverA, NM\_134156, Unigene4720\_Mf\_liverA, Unigene29302\_Mf\_liverA, Unigene21562\_Mf\_liverA, Unigene27248\_Mf\_liverA, Unigene5037\_Mf\_liverA, Unigene8132\_Mf\_liverA, Unigene5422\_Mf\_liverA, Unigene2195\_Mf\_liverA, CL795.Contig1\_Mf\_liverA, CL4434.Contig1\_Mf\_liverA, Unigene31852\_Mf\_liverA, Unigene34866\_Mf\_liverA, Unigene13396\_Mf\_liverA, Unigene5712\_Mf\_liverA, Unigene37076\_Mf\_liverA, Unigene31885\_Mf\_liverA, CL1738.Contig1\_Mf\_liverA, CL1352.Contig1\_Mf\_liverA, CL5576.Contig1\_Mf\_liverA, Unigene31206\_Mf\_liverA, NM\_011099 |
| Golgi apparatus | CL787.Contig1\_Mf\_liverA, Unigene24506\_Mf\_liverA, Unigene29399\_Mf\_liverA, Unigene25721\_Mf\_liverA, CL2855.Contig2\_Mf\_liverA, Unigene5852\_Mf\_liverA, CL3669.Contig2\_Mf\_liverA, Unigene15529\_Mf\_liverA, Unigene13414\_Mf\_liverA, NM\_009178, Unigene28186\_Mf\_liverA, Unigene8740\_Mf\_liverA, Unigene15474\_Mf\_liverA, Unigene4922\_Mf\_liverA, Unigene5194\_Mf\_liverA, Unigene5632\_Mf\_liverA, CL482.Contig1\_Mf\_liverA, Unigene36112\_Mf\_liverA, Unigene9990\_Mf\_liverA, Unigene24503\_Mf\_liverA, Unigene37259\_Mf\_liverA, Unigene560\_Mf\_liverA, Unigene30795\_Mf\_liverA, CL3816.Contig1\_Mf\_liverA, Unigene21466\_Mf\_liverA, Unigene1221\_Mf\_liverA, CL3669.Contig1\_Mf\_liverA, Unigene43037\_Mf\_liverA, Unigene37245\_Mf\_liverA, Unigene24284\_Mf\_liverA, CL5586.Contig1\_Mf\_liverA, Unigene5180\_Mf\_liverA, Unigene36328\_Mf\_liverA, CL3002.Contig1\_Mf\_liverA, Unigene281\_Mf\_liverA, Unigene44992\_Mf\_liverA, CL3268.Contig1\_Mf\_liverA, NM\_010227, Unigene37389\_Mf\_liverA, Unigene5687\_Mf\_liverA, CL4033.Contig1\_Mf\_liverA, Unigene5422\_Mf\_liverA, Unigene29940\_Mf\_liverA, Unigene37574\_Mf\_liverA, Unigene2195\_Mf\_liverA, Unigene28331\_Mf\_liverA, Unigene34010\_Mf\_liverA, Unigene4686\_Mf\_liverA, Unigene11\_Mf\_liverA, Unigene32058\_Mf\_liverA, Unigene27082\_Mf\_liverA, Unigene31885\_Mf\_liverA, CL1493.Contig2\_Mf\_liverA, Unigene34446\_Mf\_liverA, CL442.Contig2\_Mf\_liverA, CL1493.Contig1\_Mf\_liverA, Unigene779\_Mf\_liverA, CL2855.Contig1\_Mf\_liverA, Unigene28499\_Mf\_liverA, CL3685.Contig1\_Mf\_liverA, Unigene24085\_Mf\_liverA, Unigene25462\_Mf\_liverA, Unigene32059\_Mf\_liverA, Unigene28021\_Mf\_liverA |
| ULK1-ATG13-FIP200 complex | Unigene13307\_Mf\_liverA, Unigene13535\_Mf\_liverA |
| costamere | Unigene37474\_Mf\_liverA, Unigene9475\_Mf\_liverA, CL4847.Contig1\_Mf\_liverA, Unigene2678\_Mf\_liverA |
| midbody | Unigene5693\_Mf\_liverA, CL33.Contig4\_Mf\_liverA, Unigene23870\_Mf\_liverA, Unigene1479\_Mf\_liverA, Unigene43515\_Mf\_liverA, Unigene23869\_Mf\_liverA, CL33.Contig3\_Mf\_liverA, Unigene34218\_Mf\_liverA |
| cytoplasmic vesicle membrane | Unigene4909\_Mf\_liverA, Unigene36328\_Mf\_liverA, Unigene5204\_Mf\_liverA, NM\_009898, NM\_134156, CL2855.Contig2\_Mf\_liverA, CL1828.Contig1\_Mf\_liverA, Unigene29424\_Mf\_liverA, Unigene28331\_Mf\_liverA, Unigene4922\_Mf\_liverA, Unigene5632\_Mf\_liverA, Unigene29008\_Mf\_liverA, Unigene4686\_Mf\_liverA, Unigene27082\_Mf\_liverA, CL5993.Contig3\_Mf\_liverA, Unigene9990\_Mf\_liverA, CL3816.Contig1\_Mf\_liverA, CL548.Contig1\_Mf\_liverA, Unigene28094\_Mf\_liverA, CL2855.Contig1\_Mf\_liverA, Unigene8054\_Mf\_liverA, Unigene34810\_Mf\_liverA, Unigene34234\_Mf\_liverA |
| microtubule | Unigene27547\_Mf\_liverA, NM\_007896, Unigene28527\_Mf\_liverA, NM\_145824, NM\_009448, CL425.Contig1\_Mf\_liverA, Unigene23870\_Mf\_liverA, CL3198.Contig1\_Mf\_liverA, CL33.Contig3\_Mf\_liverA, Unigene14263\_Mf\_liverA, CL2117.Contig1\_Mf\_liverA, Unigene26214\_Mf\_liverA, CL33.Contig4\_Mf\_liverA, Unigene2746\_Mf\_liverA, Unigene2745\_Mf\_liverA, Unigene50250\_Mf\_liverA, Unigene15493\_Mf\_liverA, Unigene7510\_Mf\_liverA, Unigene23869\_Mf\_liverA, CL3803.Contig2\_Mf\_liverA, Unigene43515\_Mf\_liverA |
| microvillus | Unigene29399\_Mf\_liverA, Unigene25080\_Mf\_liverA, Unigene34197\_Mf\_liverA, Unigene16671\_Mf\_liverA, Unigene34446\_Mf\_liverA, CL1352.Contig1\_Mf\_liverA, Unigene24085\_Mf\_liverA, Unigene29424\_Mf\_liverA, Unigene14896\_Mf\_liverA |
| nuclear matrix | CL4162.Contig1\_Mf\_liverA, CL1537.Contig1\_Mf\_liverA, CL4847.Contig1\_Mf\_liverA, Unigene37076\_Mf\_liverA, Unigene29885\_Mf\_liverA, CL795.Contig1\_Mf\_liverA, Unigene5165\_Mf\_liverA, CL4757.Contig1\_Mf\_liverA |
| proteasome accessory complex | CL4141.Contig1\_Mf\_liverA, Unigene36851\_Mf\_liverA, CL1119.Contig1\_Mf\_liverA |
| chloride channel complex | Unigene5758\_Mf\_liverA, Unigene37526\_Mf\_liverA, Unigene30459\_Mf\_liverA |
| organelle membrane | Unigene38015\_Mf\_liverA, Unigene34656\_Mf\_liverA, NM\_009898, CL3835.Contig2\_Mf\_liverA, NM\_009592, CL1828.Contig1\_Mf\_liverA, NM\_018815, CL523.Contig1\_Mf\_liverA, Unigene29424\_Mf\_liverA, Unigene29008\_Mf\_liverA, CL482.Contig1\_Mf\_liverA, CL5560.Contig1\_Mf\_liverA, Unigene22575\_Mf\_liverA, Unigene39886\_Mf\_liverA, Unigene30303\_Mf\_liverA, Unigene30878\_Mf\_liverA, Unigene34234\_Mf\_liverA, Unigene34810\_Mf\_liverA, CL5586.Contig1\_Mf\_liverA, Unigene5204\_Mf\_liverA, Unigene21255\_Mf\_liverA, Unigene40020\_Mf\_liverA, Unigene34341\_Mf\_liverA, Unigene2\_Mf\_liverA, Unigene28331\_Mf\_liverA, NM\_145474, Unigene4686\_Mf\_liverA, Unigene13266\_Mf\_liverA, NM\_153193, Unigene18117\_Mf\_liverA, CL1493.Contig2\_Mf\_liverA, Unigene3174\_Mf\_liverA, CL2117.Contig1\_Mf\_liverA, Unigene21857\_Mf\_liverA, Unigene28094\_Mf\_liverA, CL2574.Contig1\_Mf\_liverA, Unigene2745\_Mf\_liverA, Unigene8054\_Mf\_liverA, Unigene25462\_Mf\_liverA, CL787.Contig1\_Mf\_liverA, Unigene1162\_Mf\_liverA, NM\_029562, Unigene5598\_Mf\_liverA, CL2855.Contig2\_Mf\_liverA, Unigene38406\_Mf\_liverA, Unigene1479\_Mf\_liverA, Unigene4922\_Mf\_liverA, Unigene5632\_Mf\_liverA, NM\_007811, Unigene9990\_Mf\_liverA, Unigene36641\_Mf\_liverA, Unigene139\_Mf\_liverA, NM\_001104531, NM\_153055, CL3816.Contig1\_Mf\_liverA, CL2722.Contig1\_Mf\_liverA, Unigene995\_Mf\_liverA, CL5631.Contig1\_Mf\_liverA, CL3055.Contig2\_Mf\_liverA, Unigene25398\_Mf\_liverA, Unigene36510\_Mf\_liverA, Unigene27260\_Mf\_liverA, NM\_134156, Unigene36845\_Mf\_liverA, Unigene37389\_Mf\_liverA, Unigene32332\_Mf\_liverA, Unigene8132\_Mf\_liverA, NM\_019717, Unigene34010\_Mf\_liverA, Unigene29253\_Mf\_liverA, Unigene27082\_Mf\_liverA, Unigene5456\_Mf\_liverA, Unigene5712\_Mf\_liverA, Unigene1280\_Mf\_liverA, Unigene21256\_Mf\_liverA, Unigene1205\_Mf\_liverA, CL442.Contig2\_Mf\_liverA, CL2855.Contig1\_Mf\_liverA, Unigene2746\_Mf\_liverA, NR\_003552, Unigene13233\_Mf\_liverA, Unigene5648\_Mf\_liverA, Unigene26515\_Mf\_liverA, NM\_001100182, NM\_009128, CL2240.Contig1\_Mf\_liverA, NM\_009178, Unigene13414\_Mf\_liverA, NM\_008293, CL2797.Contig2\_Mf\_liverA, CL1125.Contig1\_Mf\_liverA, Unigene38208\_Mf\_liverA, Unigene35237\_Mf\_liverA, CL5993.Contig3\_Mf\_liverA, Unigene5693\_Mf\_liverA, Unigene4703\_Mf\_liverA, Unigene1440\_Mf\_liverA, Unigene43037\_Mf\_liverA, Unigene23013\_Mf\_liverA, CL4583.Contig2\_Mf\_liverA, NM\_133838, Unigene36328\_Mf\_liverA, CL3002.Contig1\_Mf\_liverA, CL3055.Contig1\_Mf\_liverA, CL1988.Contig3\_Mf\_liverA, CL1810.Contig1\_Mf\_liverA, CL4033.Contig1\_Mf\_liverA, Unigene4781\_Mf\_liverA, Unigene46615\_Mf\_liverA, Unigene5906\_Mf\_liverA, Unigene28731\_Mf\_liverA, CL1493.Contig1\_Mf\_liverA, Unigene28499\_Mf\_liverA, CL3685.Contig1\_Mf\_liverA, Unigene24085\_Mf\_liverA, Unigene10135\_Mf\_liverA, Unigene542\_Mf\_liverA, CL4106.Contig1\_Mf\_liverA, Unigene22330\_Mf\_liverA, Unigene4909\_Mf\_liverA, Unigene24471\_Mf\_liverA, NM\_010162, Unigene15888\_Mf\_liverA, Unigene15529\_Mf\_liverA, CL4127.Contig1\_Mf\_liverA, CL5307.Contig1\_Mf\_liverA, CL1190.Contig3\_Mf\_liverA, CL4490.Contig2\_Mf\_liverA, Unigene1221\_Mf\_liverA, Unigene23185\_Mf\_liverA, Unigene35046\_Mf\_liverA, Unigene281\_Mf\_liverA, CL3104.Contig1\_Mf\_liverA, Unigene5687\_Mf\_liverA, Unigene13772\_Mf\_liverA, Unigene36593\_Mf\_liverA, Unigene5745\_Mf\_liverA, Unigene36699\_Mf\_liverA, Unigene21359\_Mf\_liverA, CL5698.Contig1\_Mf\_liverA, Unigene4681\_Mf\_liverA, Unigene5512\_Mf\_liverA, CL548.Contig1\_Mf\_liverA, Unigene5886\_Mf\_liverA |
| type III intermediate filament | Unigene37076\_Mf\_liverA, Unigene5165\_Mf\_liverA |
| cell part | NM\_176843, Unigene34656\_Mf\_liverA, NM\_009898, Unigene7612\_Mf\_liverA, Unigene7950\_Mf\_liverA, Unigene24111\_Mf\_liverA, Unigene28186\_Mf\_liverA, Unigene36622\_Mf\_liverA, NM\_010378, Unigene31045\_Mf\_liverA, Unigene33138\_Mf\_liverA, Unigene16463\_Mf\_liverA, Unigene39886\_Mf\_liverA, CL33.Contig4\_Mf\_liverA, Unigene15064\_Mf\_liverA, NM\_172723, Unigene34124\_Mf\_liverA, Unigene7510\_Mf\_liverA, Unigene24252\_Mf\_liverA, CL4757.Contig1\_Mf\_liverA, NR\_004446, Unigene36765\_Mf\_liverA, NM\_013866, Unigene37488\_Mf\_liverA, Unigene4557\_Mf\_liverA, Unigene35431\_Mf\_liverA, Unigene15703\_Mf\_liverA, Unigene30495\_Mf\_liverA, Unigene2\_Mf\_liverA, Unigene28331\_Mf\_liverA, CL33.Contig3\_Mf\_liverA, NM\_010233, Unigene37084\_Mf\_liverA, Unigene13379\_Mf\_liverA, Unigene25594\_Mf\_liverA, Unigene779\_Mf\_liverA, Unigene14050\_Mf\_liverA, Unigene21857\_Mf\_liverA, Unigene13419\_Mf\_liverA, Unigene8054\_Mf\_liverA, Unigene5552\_Mf\_liverA, CL2384.Contig1\_Mf\_liverA, Unigene37454\_Mf\_liverA, Unigene743\_Mf\_liverA, NM\_053214, Unigene36417\_Mf\_liverA, Unigene26065\_Mf\_liverA, Unigene25091\_Mf\_liverA, Unigene18125\_Mf\_liverA, Unigene13097\_Mf\_liverA, NM\_177093, Unigene25047\_Mf\_liverA, Unigene14907\_Mf\_liverA, Unigene7476\_Mf\_liverA, CL4332.Contig1\_Mf\_liverA, Unigene13913\_Mf\_liverA, Unigene25976\_Mf\_liverA, Unigene30261\_Mf\_liverA, Unigene9475\_Mf\_liverA, NM\_145942, Unigene27895\_Mf\_liverA, Unigene36112\_Mf\_liverA, Unigene36641\_Mf\_liverA, Unigene13945\_Mf\_liverA, Unigene30459\_Mf\_liverA, Unigene139\_Mf\_liverA, Unigene26941\_Mf\_liverA, NM\_172509, Unigene995\_Mf\_liverA, NM\_001143689, Unigene34123\_Mf\_liverA, Unigene10756\_Mf\_liverA, CL3669.Contig1\_Mf\_liverA, Unigene27249\_Mf\_liverA, Unigene43515\_Mf\_liverA, Unigene11097\_Mf\_liverA, NM\_134156, Unigene4720\_Mf\_liverA, Unigene37389\_Mf\_liverA, CL4156.Contig1\_Mf\_liverA, Unigene32716\_Mf\_liverA, Unigene27248\_Mf\_liverA, Unigene29253\_Mf\_liverA, Unigene34010\_Mf\_liverA, Unigene152\_Mf\_liverA, Unigene31852\_Mf\_liverA, Unigene5456\_Mf\_liverA, Unigene37076\_Mf\_liverA, Unigene2678\_Mf\_liverA, Unigene1280\_Mf\_liverA, Unigene30288\_Mf\_liverA, CL1738.Contig1\_Mf\_liverA, CL5576.Contig1\_Mf\_liverA, CL3339.Contig1\_Mf\_liverA, Unigene2746\_Mf\_liverA, Unigene4593\_Mf\_liverA, Unigene5236\_Mf\_liverA, Unigene10496\_Mf\_liverA, Unigene28021\_Mf\_liverA, Unigene45904\_Mf\_liverA, Unigene37433\_Mf\_liverA, CL1442.Contig1\_Mf\_liverA, NM\_009933, CL5528.Contig1\_Mf\_liverA, Unigene2162\_Mf\_liverA, CL425.Contig1\_Mf\_liverA, CL3150.Contig1\_Mf\_liverA, CL2797.Contig2\_Mf\_liverA, Unigene37243\_Mf\_liverA, Unigene39403\_Mf\_liverA, Unigene30587\_Mf\_liverA, Unigene5693\_Mf\_liverA, Unigene39970\_Mf\_liverA, Unigene13271\_Mf\_liverA, CL3393.Contig1\_Mf\_liverA, Unigene19821\_Mf\_liverA, CL4057.Contig1\_Mf\_liverA, Unigene29876\_Mf\_liverA, CL3800.Contig1\_Mf\_liverA, Unigene25080\_Mf\_liverA, Unigene28687\_Mf\_liverA, CL1988.Contig3\_Mf\_liverA, Unigene29885\_Mf\_liverA, CL3055.Contig1\_Mf\_liverA, NM\_028785, Unigene5758\_Mf\_liverA, NM\_009609, CL1810.Contig1\_Mf\_liverA, CL4033.Contig1\_Mf\_liverA, Unigene24547\_Mf\_liverA, CL3519.Contig1\_Mf\_liverA, Unigene8033\_Mf\_liverA, Unigene28025\_Mf\_liverA, CL1493.Contig1\_Mf\_liverA, Unigene24801\_Mf\_liverA, CL3685.Contig1\_Mf\_liverA, CL5764.Contig1\_Mf\_liverA, Unigene39231\_Mf\_liverA, Unigene41226\_Mf\_liverA, Unigene4909\_Mf\_liverA, Unigene34197\_Mf\_liverA, Unigene24471\_Mf\_liverA, NM\_026823, Unigene35168\_Mf\_liverA, Unigene15888\_Mf\_liverA, NM\_010162, Unigene15529\_Mf\_liverA, Unigene37262\_Mf\_liverA, Unigene12908\_Mf\_liverA, NM\_010469, CL2251.Contig1\_Mf\_liverA, CL4490.Contig2\_Mf\_liverA, CL5293.Contig1\_Mf\_liverA, Unigene25595\_Mf\_liverA, Unigene14263\_Mf\_liverA, Unigene14286\_Mf\_liverA, NM\_016861, Unigene39011\_Mf\_liverA, Unigene386\_Mf\_liverA, Unigene26250\_Mf\_liverA, Unigene30795\_Mf\_liverA, NM\_024452, Unigene20432\_Mf\_liverA, Unigene48460\_Mf\_liverA, NM\_011580, Unigene14330\_Mf\_liverA, Unigene35046\_Mf\_liverA, Unigene28459\_Mf\_liverA, Unigene37245\_Mf\_liverA, Unigene16671\_Mf\_liverA, Unigene21693\_Mf\_liverA, Unigene13535\_Mf\_liverA, Unigene13772\_Mf\_liverA, Unigene29302\_Mf\_liverA, Unigene33428\_Mf\_liverA, Unigene37574\_Mf\_liverA, Unigene36176\_Mf\_liverA, Unigene36699\_Mf\_liverA, Unigene7968\_Mf\_liverA, Unigene36667\_Mf\_liverA, Unigene18340\_Mf\_liverA, NM\_001164598, Unigene30429\_Mf\_liverA, Unigene30412\_Mf\_liverA, Unigene4983\_Mf\_liverA, Unigene38317\_Mf\_liverA, Unigene5512\_Mf\_liverA, CL373.Contig7\_Mf\_liverA, Unigene42855\_Mf\_liverA, Unigene29889\_Mf\_liverA, Unigene2939\_Mf\_liverA, Unigene36728\_Mf\_liverA, Unigene9466\_Mf\_liverA, Unigene35884\_Mf\_liverA, CL5640.Contig1\_Mf\_liverA, NM\_020559, Unigene28142\_Mf\_liverA, CL3835.Contig2\_Mf\_liverA, Unigene38831\_Mf\_liverA, NM\_145824, Unigene30356\_Mf\_liverA, Unigene38124\_Mf\_liverA, Unigene46870\_Mf\_liverA, Unigene14603\_Mf\_liverA, CL4336.Contig3\_Mf\_liverA, Unigene8473\_Mf\_liverA, Unigene29008\_Mf\_liverA, Unigene27593\_Mf\_liverA, Unigene19049\_Mf\_liverA, Unigene33508\_Mf\_liverA, Unigene37132\_Mf\_liverA, CL5358.Contig1\_Mf\_liverA, Unigene48792\_Mf\_liverA, Unigene31263\_Mf\_liverA, NM\_011072, Unigene14816\_Mf\_liverA, Unigene7969\_Mf\_liverA, Unigene21466\_Mf\_liverA, Unigene30303\_Mf\_liverA, NM\_173753, Unigene34810\_Mf\_liverA, Unigene27420\_Mf\_liverA, CL6038.Contig2\_Mf\_liverA, Unigene36889\_Mf\_liverA, Unigene13307\_Mf\_liverA, Unigene37999\_Mf\_liverA, Unigene40020\_Mf\_liverA, Unigene37904\_Mf\_liverA, CL3483.Contig1\_Mf\_liverA, Unigene34789\_Mf\_liverA, CL4141.Contig1\_Mf\_liverA, Unigene25057\_Mf\_liverA, NM\_145419, CL5978.Contig3\_Mf\_liverA, CL5191.Contig2\_Mf\_liverA, Unigene13658\_Mf\_liverA, NM\_175260, Unigene4686\_Mf\_liverA, Unigene11\_Mf\_liverA, Unigene4630\_Mf\_liverA, Unigene11544\_Mf\_liverA, NM\_018780, Unigene18117\_Mf\_liverA, CL3575.Contig1\_Mf\_liverA, CL1493.Contig2\_Mf\_liverA, CL2117.Contig1\_Mf\_liverA, CL3565.Contig1\_Mf\_liverA, Unigene36988\_Mf\_liverA, Unigene28094\_Mf\_liverA, Unigene40824\_Mf\_liverA, CL2574.Contig1\_Mf\_liverA, Unigene5941\_Mf\_liverA, Unigene38104\_Mf\_liverA, Unigene23328\_Mf\_liverA, Unigene25462\_Mf\_liverA, Unigene38065\_Mf\_liverA, Unigene16684\_Mf\_liverA, Unigene36626\_Mf\_liverA, NM\_001029934, NM\_029562, Unigene28822\_Mf\_liverA, Unigene36691\_Mf\_liverA, Unigene31988\_Mf\_liverA, NM\_019703, Unigene19297\_Mf\_liverA, CL2326.Contig1\_Mf\_liverA, Unigene151\_Mf\_liverA, CL848.Contig2\_Mf\_liverA, Unigene23870\_Mf\_liverA, NM\_008808, Unigene4768\_Mf\_liverA, Unigene10774\_Mf\_liverA, CL2131.Contig4\_Mf\_liverA, Unigene25662\_Mf\_liverA, CL3198.Contig1\_Mf\_liverA, Unigene13143\_Mf\_liverA, Unigene37460\_Mf\_liverA, Unigene31517\_Mf\_liverA, Unigene31231\_Mf\_liverA, Unigene36628\_Mf\_liverA, NM\_007811, Unigene24323\_Mf\_liverA, NM\_008538, NM\_011305, NM\_008610, Unigene12200\_Mf\_liverA, Unigene35859\_Mf\_liverA, CL3816.Contig1\_Mf\_liverA, Unigene36631\_Mf\_liverA, Unigene4510\_Mf\_liverA, CL3803.Contig2\_Mf\_liverA, Unigene24284\_Mf\_liverA, Unigene26873\_Mf\_liverA, Unigene27260\_Mf\_liverA, CL3268.Contig1\_Mf\_liverA, Unigene36845\_Mf\_liverA, Unigene14758\_Mf\_liverA, Unigene37729\_Mf\_liverA, NM\_010359, Unigene29940\_Mf\_liverA, CL795.Contig1\_Mf\_liverA, Unigene42812\_Mf\_liverA, Unigene5509\_Mf\_liverA, Unigene5325\_Mf\_liverA, CL5039.Contig2\_Mf\_liverA, Unigene1205\_Mf\_liverA, Unigene13841\_Mf\_liverA, CL1352.Contig1\_Mf\_liverA, CL6039.Contig1\_Mf\_liverA, Unigene24804\_Mf\_liverA, Unigene31206\_Mf\_liverA, NM\_011132, Unigene32412\_Mf\_liverA, Unigene17569\_Mf\_liverA, Unigene40924\_Mf\_liverA, NM\_009128, NM\_175472, Unigene7412\_Mf\_liverA, CL2240.Contig1\_Mf\_liverA, Unigene5783\_Mf\_liverA, Unigene29823\_Mf\_liverA, Unigene39385\_Mf\_liverA, Unigene22980\_Mf\_liverA, Unigene38339\_Mf\_liverA, Unigene15474\_Mf\_liverA, Unigene23448\_Mf\_liverA, CL1125.Contig1\_Mf\_liverA, Unigene37526\_Mf\_liverA, Unigene29510\_Mf\_liverA, NM\_010579, Unigene15681\_Mf\_liverA, Unigene7350\_Mf\_liverA, Unigene19083\_Mf\_liverA, Unigene673\_Mf\_liverA, Unigene4703\_Mf\_liverA, Unigene37096\_Mf\_liverA, Unigene5165\_Mf\_liverA, Unigene30707\_Mf\_liverA, NM\_133838, Unigene7674\_Mf\_liverA, CL3002.Contig1\_Mf\_liverA, NM\_008871, Unigene15058\_Mf\_liverA, Unigene35491\_Mf\_liverA, NM\_029872, Unigene37139\_Mf\_liverA, Unigene5906\_Mf\_liverA, Unigene34035\_Mf\_liverA, CL5688.Contig1\_Mf\_liverA, NM\_033444, Unigene4597\_Mf\_liverA, CL2791.Contig1\_Mf\_liverA, Unigene14170\_Mf\_liverA, Unigene35269\_Mf\_liverA, Unigene10135\_Mf\_liverA, Unigene22330\_Mf\_liverA, Unigene5011\_Mf\_liverA, Unigene5954\_Mf\_liverA, NM\_009883, CL4127.Contig1\_Mf\_liverA, Unigene33526\_Mf\_liverA, Unigene1327\_Mf\_liverA, CL1190.Contig3\_Mf\_liverA, Unigene8740\_Mf\_liverA, CL5978.Contig2\_Mf\_liverA, NM\_008776, Unigene36177\_Mf\_liverA, Unigene24566\_Mf\_liverA, NM\_007478, Unigene15170\_Mf\_liverA, Unigene4604\_Mf\_liverA, NM\_011418, Unigene560\_Mf\_liverA, Unigene24477\_Mf\_liverA, Unigene30731\_Mf\_liverA, Unigene30983\_Mf\_liverA, Unigene39749\_Mf\_liverA, Unigene36418\_Mf\_liverA, Unigene25052\_Mf\_liverA, Unigene550\_Mf\_liverA, Unigene38311\_Mf\_liverA, Unigene21843\_Mf\_liverA, CL2355.Contig1\_Mf\_liverA, Unigene36910\_Mf\_liverA, Unigene28314\_Mf\_liverA, Unigene37153\_Mf\_liverA, Unigene21562\_Mf\_liverA, Unigene15592\_Mf\_liverA, NM\_015767, Unigene37711\_Mf\_liverA, Unigene41583\_Mf\_liverA, Unigene5418\_Mf\_liverA, Unigene2195\_Mf\_liverA, Unigene93\_Mf\_liverA, NM\_146007, Unigene30892\_Mf\_liverA, Unigene803\_Mf\_liverA, CL3911.Contig2\_Mf\_liverA, Unigene34866\_Mf\_liverA, Unigene29292\_Mf\_liverA, Unigene14765\_Mf\_liverA, NM\_144848, Unigene31885\_Mf\_liverA, NM\_001033481, Unigene4681\_Mf\_liverA, CL548.Contig1\_Mf\_liverA, NM\_007392, Unigene14284\_Mf\_liverA, Unigene26474\_Mf\_liverA, Unigene25787\_Mf\_liverA, Unigene24506\_Mf\_liverA, NM\_009450, Unigene34609\_Mf\_liverA, Unigene25721\_Mf\_liverA, NM\_009592, CL3669.Contig2\_Mf\_liverA, Unigene8106\_Mf\_liverA, Unigene5852\_Mf\_liverA, Unigene36172\_Mf\_liverA, Unigene37334\_Mf\_liverA, NM\_018815, Unigene36851\_Mf\_liverA, Unigene14665\_Mf\_liverA, NM\_080638, Unigene7936\_Mf\_liverA, Unigene29424\_Mf\_liverA, Unigene34218\_Mf\_liverA, Unigene26053\_Mf\_liverA, CL4105.Contig1\_Mf\_liverA, CL4577.Contig1\_Mf\_liverA, Unigene25090\_Mf\_liverA, CL238.Contig1\_Mf\_liverA, CL2423.Contig1\_Mf\_liverA, CL5560.Contig1\_Mf\_liverA, Unigene22575\_Mf\_liverA, Unigene5248\_Mf\_liverA, Unigene785\_Mf\_liverA, Unigene26422\_Mf\_liverA, Unigene38689\_Mf\_liverA, Unigene29334\_Mf\_liverA, NM\_010877, NM\_021273, Unigene38919\_Mf\_liverA, Unigene1137\_Mf\_liverA, CL5586.Contig1\_Mf\_liverA, Unigene21255\_Mf\_liverA, Unigene34341\_Mf\_liverA, Unigene30142\_Mf\_liverA, Unigene35816\_Mf\_liverA, Unigene13940\_Mf\_liverA, CL593.Contig2\_Mf\_liverA, NM\_145474, Unigene33054\_Mf\_liverA, NM\_009776, Unigene13266\_Mf\_liverA, NM\_153193, Unigene15205\_Mf\_liverA, Unigene5138\_Mf\_liverA, Unigene37150\_Mf\_liverA, Unigene5330\_Mf\_liverA, NM\_177320, Unigene34143\_Mf\_liverA, Unigene38657\_Mf\_liverA, Unigene653\_Mf\_liverA, Unigene23869\_Mf\_liverA, Unigene9150\_Mf\_liverA, Unigene5598\_Mf\_liverA, Unigene13363\_Mf\_liverA, CL2855.Contig2\_Mf\_liverA, Unigene36175\_Mf\_liverA, Unigene28226\_Mf\_liverA, Unigene44317\_Mf\_liverA, Unigene37278\_Mf\_liverA, Unigene2919\_Mf\_liverA, Unigene21561\_Mf\_liverA, Unigene1479\_Mf\_liverA, Unigene4922\_Mf\_liverA, Unigene14637\_Mf\_liverA, NM\_153505, CL2159.Contig2\_Mf\_liverA, Unigene44882\_Mf\_liverA, Unigene37662\_Mf\_liverA, Unigene31654\_Mf\_liverA, Unigene9990\_Mf\_liverA, NM\_001104531, NM\_153055, Unigene4944\_Mf\_liverA, Unigene33560\_Mf\_liverA, Unigene23158\_Mf\_liverA, CL3055.Contig2\_Mf\_liverA, Unigene37575\_Mf\_liverA, Unigene25398\_Mf\_liverA, Unigene27547\_Mf\_liverA, CL1736.Contig2\_Mf\_liverA, Unigene5180\_Mf\_liverA, Unigene21842\_Mf\_liverA, Unigene12909\_Mf\_liverA, Unigene32889\_Mf\_liverA, Unigene4723\_Mf\_liverA, Unigene33271\_Mf\_liverA, Unigene32332\_Mf\_liverA, Unigene37470\_Mf\_liverA, CL5254.Contig1\_Mf\_liverA, Unigene37542\_Mf\_liverA, NM\_009713, NM\_009087, Unigene35169\_Mf\_liverA, Unigene21256\_Mf\_liverA, NR\_003552, Unigene5169\_Mf\_liverA, Unigene16465\_Mf\_liverA, Unigene32059\_Mf\_liverA, Unigene32202\_Mf\_liverA, Unigene5472\_Mf\_liverA, NM\_001025573, Unigene26515\_Mf\_liverA, Unigene31251\_Mf\_liverA, NM\_011503, Unigene40610\_Mf\_liverA, NM\_172121, CL186.Contig3\_Mf\_liverA, Unigene583\_Mf\_liverA, Unigene4697\_Mf\_liverA, CL4736.Contig1\_Mf\_liverA, Unigene27026\_Mf\_liverA, Unigene14916\_Mf\_liverA, Unigene35858\_Mf\_liverA, Unigene30808\_Mf\_liverA, Unigene30039\_Mf\_liverA, Unigene29231\_Mf\_liverA, Unigene37442\_Mf\_liverA, Unigene50250\_Mf\_liverA, Unigene1440\_Mf\_liverA, Unigene35958\_Mf\_liverA, Unigene43037\_Mf\_liverA, Unigene13525\_Mf\_liverA, Unigene24395\_Mf\_liverA, Unigene4938\_Mf\_liverA, Unigene5815\_Mf\_liverA, Unigene1129\_Mf\_liverA, Unigene33168\_Mf\_liverA, Unigene27483\_Mf\_liverA, Unigene28662\_Mf\_liverA, CL3738.Contig1\_Mf\_liverA, CL2439.Contig1\_Mf\_liverA, Unigene4781\_Mf\_liverA, Unigene46615\_Mf\_liverA, Unigene35935\_Mf\_liverA, Unigene29426\_Mf\_liverA, Unigene30494\_Mf\_liverA, Unigene13462\_Mf\_liverA, CL5049.Contig2\_Mf\_liverA, Unigene34446\_Mf\_liverA, Unigene13683\_Mf\_liverA, Unigene11686\_Mf\_liverA, Unigene24755\_Mf\_liverA, Unigene9698\_Mf\_liverA, Unigene28499\_Mf\_liverA, Unigene30585\_Mf\_liverA, Unigene36420\_Mf\_liverA, Unigene542\_Mf\_liverA, Unigene38593\_Mf\_liverA, NM\_033325, Unigene24758\_Mf\_liverA, Unigene23271\_Mf\_liverA, Unigene25092\_Mf\_liverA, CL5828.Contig2\_Mf\_liverA, Unigene28527\_Mf\_liverA, Unigene35039\_Mf\_liverA, Unigene29938\_Mf\_liverA, CL2625.Contig2\_Mf\_liverA, CL725.Contig1\_Mf\_liverA, Unigene30947\_Mf\_liverA, CL5293.Contig2\_Mf\_liverA, CL1165.Contig2\_Mf\_liverA, Unigene10335\_Mf\_liverA, Unigene35090\_Mf\_liverA, Unigene14212\_Mf\_liverA, Unigene34962\_Mf\_liverA, CL1165.Contig4\_Mf\_liverA, CL4669.Contig1\_Mf\_liverA, NM\_028222, Unigene23185\_Mf\_liverA, Unigene29558\_Mf\_liverA, CL3692.Contig2\_Mf\_liverA, Unigene26083\_Mf\_liverA, NM\_013863, Unigene281\_Mf\_liverA, Unigene10313\_Mf\_liverA, Unigene29985\_Mf\_liverA, Unigene26580\_Mf\_liverA, Unigene5687\_Mf\_liverA, CL3104.Contig1\_Mf\_liverA, Unigene36593\_Mf\_liverA, Unigene5037\_Mf\_liverA, Unigene26336\_Mf\_liverA, Unigene5422\_Mf\_liverA, NM\_146016, Unigene36673\_Mf\_liverA, NM\_181517, CL5698.Contig1\_Mf\_liverA, Unigene31392\_Mf\_liverA, NM\_023256, CL591.Contig1\_Mf\_liverA, NM\_009636, Unigene4556\_Mf\_liverA, Unigene38237\_Mf\_liverA, NM\_007763, Unigene26055\_Mf\_liverA, CL139.Contig2\_Mf\_liverA, Unigene29399\_Mf\_liverA, CL2520.Contig1\_Mf\_liverA, Unigene38015\_Mf\_liverA, NM\_007896, Unigene24713\_Mf\_liverA, Unigene42975\_Mf\_liverA, NM\_144907, CL1828.Contig1\_Mf\_liverA, Unigene29308\_Mf\_liverA, CL1362.Contig1\_Mf\_liverA, CL523.Contig1\_Mf\_liverA, Unigene15914\_Mf\_liverA, Unigene5194\_Mf\_liverA, CL1063.Contig1\_Mf\_liverA, CL482.Contig1\_Mf\_liverA, Unigene22433\_Mf\_liverA, NM\_001167705, Unigene29405\_Mf\_liverA, Unigene32882\_Mf\_liverA, Unigene21263\_Mf\_liverA, Unigene7619\_Mf\_liverA, Unigene37698\_Mf\_liverA, Unigene2633\_Mf\_liverA, Unigene30878\_Mf\_liverA, Unigene40289\_Mf\_liverA, Unigene21013\_Mf\_liverA, Unigene15656\_Mf\_liverA, Unigene34234\_Mf\_liverA, Unigene24344\_Mf\_liverA, Unigene38331\_Mf\_liverA, Unigene5204\_Mf\_liverA, CL5001.Contig2\_Mf\_liverA, Unigene37099\_Mf\_liverA, Unigene37268\_Mf\_liverA, CL1256.Contig1\_Mf\_liverA, Unigene25070\_Mf\_liverA, Unigene24112\_Mf\_liverA, Unigene23273\_Mf\_liverA, Unigene24379\_Mf\_liverA, Unigene37263\_Mf\_liverA, Unigene36853\_Mf\_liverA, Unigene38110\_Mf\_liverA, NM\_009896, CL1119.Contig1\_Mf\_liverA, Unigene26194\_Mf\_liverA, Unigene31333\_Mf\_liverA, Unigene41158\_Mf\_liverA, Unigene25596\_Mf\_liverA, NM\_010763, CL3778.Contig2\_Mf\_liverA, Unigene3174\_Mf\_liverA, Unigene22978\_Mf\_liverA, Unigene22432\_Mf\_liverA, Unigene25046\_Mf\_liverA, Unigene13918\_Mf\_liverA, Unigene2745\_Mf\_liverA, NM\_001024205, CL787.Contig1\_Mf\_liverA, Unigene1162\_Mf\_liverA, CL4076.Contig1\_Mf\_liverA, Unigene25524\_Mf\_liverA, CL4847.Contig1\_Mf\_liverA, Unigene20128\_Mf\_liverA, NM\_001162917, CL3196.Contig2\_Mf\_liverA, Unigene47203\_Mf\_liverA, Unigene38406\_Mf\_liverA, CL3750.Contig2\_Mf\_liverA, CL4722.Contig1\_Mf\_liverA, Unigene33459\_Mf\_liverA, Unigene26398\_Mf\_liverA, Unigene25012\_Mf\_liverA, Unigene34258\_Mf\_liverA, Unigene5632\_Mf\_liverA, NM\_001001806, CL479.Contig1\_Mf\_liverA, Unigene27419\_Mf\_liverA, Unigene14582\_Mf\_liverA, Unigene34746\_Mf\_liverA, Unigene14715\_Mf\_liverA, Unigene24503\_Mf\_liverA, NM\_134059, Unigene37180\_Mf\_liverA, Unigene4523\_Mf\_liverA, Unigene27438\_Mf\_liverA, CL2722.Contig1\_Mf\_liverA, Unigene20116\_Mf\_liverA, CL5631.Contig1\_Mf\_liverA, Unigene31427\_Mf\_liverA, Unigene14896\_Mf\_liverA, Unigene30584\_Mf\_liverA, Unigene431\_Mf\_liverA, Unigene19885\_Mf\_liverA, Unigene36510\_Mf\_liverA, NM\_009091, Unigene44992\_Mf\_liverA, Unigene10820\_Mf\_liverA, NM\_010391, NM\_008889, NM\_010227, Unigene21317\_Mf\_liverA, Unigene30493\_Mf\_liverA, Unigene8132\_Mf\_liverA, NM\_019717, Unigene32058\_Mf\_liverA, Unigene27082\_Mf\_liverA, Unigene37178\_Mf\_liverA, Unigene5712\_Mf\_liverA, Unigene584\_Mf\_liverA, CL2962.Contig1\_Mf\_liverA, Unigene23055\_Mf\_liverA, CL442.Contig2\_Mf\_liverA, Unigene26214\_Mf\_liverA, NM\_010380, NM\_033374, CL2855.Contig1\_Mf\_liverA, CL4411.Contig4\_Mf\_liverA, NM\_001025388, Unigene13233\_Mf\_liverA, NM\_011099, Unigene5648\_Mf\_liverA, NM\_009447, NM\_001100182, CL1537.Contig1\_Mf\_liverA, NM\_010481, Unigene392\_Mf\_liverA, CL4086.Contig1\_Mf\_liverA, CL507.Contig1\_Mf\_liverA, Unigene23082\_Mf\_liverA, Unigene5360\_Mf\_liverA, Unigene15318\_Mf\_liverA, NM\_009178, Unigene13414\_Mf\_liverA, NM\_008293, Unigene1130\_Mf\_liverA, Unigene30154\_Mf\_liverA, Unigene15982\_Mf\_liverA, Unigene38208\_Mf\_liverA, CL4162.Contig1\_Mf\_liverA, Unigene35237\_Mf\_liverA, CL5993.Contig3\_Mf\_liverA, Unigene37259\_Mf\_liverA, Unigene12907\_Mf\_liverA, Unigene5260\_Mf\_liverA, Unigene15588\_Mf\_liverA, Unigene23013\_Mf\_liverA, CL4583.Contig2\_Mf\_liverA, Unigene36414\_Mf\_liverA, Unigene13894\_Mf\_liverA, Unigene5175\_Mf\_liverA, Unigene34219\_Mf\_liverA, Unigene33880\_Mf\_liverA, Unigene36543\_Mf\_liverA, NM\_011352, Unigene36328\_Mf\_liverA, CL1222.Contig1\_Mf\_liverA, Unigene6895\_Mf\_liverA, CL2327.Contig1\_Mf\_liverA, CL2688.Contig1\_Mf\_liverA, CL6039.Contig2\_Mf\_liverA, NM\_010393, Unigene35037\_Mf\_liverA, Unigene36710\_Mf\_liverA, Unigene5940\_Mf\_liverA, NM\_007393, CL1662.Contig1\_Mf\_liverA, Unigene28731\_Mf\_liverA, NM\_053072, Unigene1212\_Mf\_liverA, CL3549.Contig1\_Mf\_liverA, NM\_009706, Unigene24085\_Mf\_liverA, NM\_007986, CL4106.Contig1\_Mf\_liverA, CL3750.Contig1\_Mf\_liverA, CL1588.Contig3\_Mf\_liverA, Unigene8016\_Mf\_liverA, Unigene18796\_Mf\_liverA, NM\_009448, CL5307.Contig1\_Mf\_liverA, Unigene21337\_Mf\_liverA, Unigene31730\_Mf\_liverA, Unigene51055\_Mf\_liverA, Unigene28873\_Mf\_liverA, CL4432.Contig2\_Mf\_liverA, Unigene33525\_Mf\_liverA, Unigene4540\_Mf\_liverA, Unigene24204\_Mf\_liverA, Unigene37310\_Mf\_liverA, Unigene9406\_Mf\_liverA, Unigene27803\_Mf\_liverA, Unigene15026\_Mf\_liverA, Unigene1221\_Mf\_liverA, CL1588.Contig1\_Mf\_liverA, NM\_017379, Unigene39875\_Mf\_liverA, NM\_001081274, Unigene25238\_Mf\_liverA, Unigene7224\_Mf\_liverA, Unigene26309\_Mf\_liverA, Unigene17632\_Mf\_liverA, Unigene5745\_Mf\_liverA, Unigene21336\_Mf\_liverA, Unigene12889\_Mf\_liverA, Unigene38514\_Mf\_liverA, Unigene665\_Mf\_liverA, Unigene35678\_Mf\_liverA, CL4434.Contig1\_Mf\_liverA, Unigene5147\_Mf\_liverA, Unigene8560\_Mf\_liverA, Unigene13396\_Mf\_liverA, Unigene41419\_Mf\_liverA, Unigene21359\_Mf\_liverA, Unigene36487\_Mf\_liverA, Unigene37474\_Mf\_liverA, Unigene16891\_Mf\_liverA, Unigene7733\_Mf\_liverA, Unigene37819\_Mf\_liverA, Unigene5886\_Mf\_liverA, Unigene27274\_Mf\_liverA, Unigene15493\_Mf\_liverA |
| cell | NM\_176843, Unigene34656\_Mf\_liverA, NM\_009898, Unigene7612\_Mf\_liverA, Unigene7950\_Mf\_liverA, Unigene24111\_Mf\_liverA, Unigene28186\_Mf\_liverA, Unigene36622\_Mf\_liverA, NM\_010378, Unigene31045\_Mf\_liverA, Unigene33138\_Mf\_liverA, Unigene16463\_Mf\_liverA, Unigene39886\_Mf\_liverA, CL33.Contig4\_Mf\_liverA, Unigene15064\_Mf\_liverA, NM\_172723, Unigene34124\_Mf\_liverA, Unigene7510\_Mf\_liverA, Unigene24252\_Mf\_liverA, CL4757.Contig1\_Mf\_liverA, NR\_004446, Unigene36765\_Mf\_liverA, NM\_013866, Unigene37488\_Mf\_liverA, Unigene4557\_Mf\_liverA, Unigene35431\_Mf\_liverA, Unigene15703\_Mf\_liverA, Unigene30495\_Mf\_liverA, Unigene2\_Mf\_liverA, Unigene28331\_Mf\_liverA, CL33.Contig3\_Mf\_liverA, NM\_010233, Unigene37084\_Mf\_liverA, Unigene13379\_Mf\_liverA, Unigene25594\_Mf\_liverA, Unigene779\_Mf\_liverA, Unigene14050\_Mf\_liverA, Unigene21857\_Mf\_liverA, Unigene13419\_Mf\_liverA, Unigene8054\_Mf\_liverA, Unigene5552\_Mf\_liverA, CL2384.Contig1\_Mf\_liverA, Unigene37454\_Mf\_liverA, Unigene743\_Mf\_liverA, NM\_053214, Unigene36417\_Mf\_liverA, Unigene26065\_Mf\_liverA, Unigene25091\_Mf\_liverA, Unigene18125\_Mf\_liverA, Unigene13097\_Mf\_liverA, NM\_177093, Unigene25047\_Mf\_liverA, Unigene14907\_Mf\_liverA, Unigene7476\_Mf\_liverA, CL4332.Contig1\_Mf\_liverA, Unigene13913\_Mf\_liverA, Unigene25976\_Mf\_liverA, Unigene30261\_Mf\_liverA, Unigene9475\_Mf\_liverA, NM\_145942, Unigene27895\_Mf\_liverA, Unigene36112\_Mf\_liverA, Unigene36641\_Mf\_liverA, Unigene13945\_Mf\_liverA, Unigene30459\_Mf\_liverA, Unigene139\_Mf\_liverA, Unigene26941\_Mf\_liverA, NM\_172509, Unigene995\_Mf\_liverA, NM\_001143689, Unigene34123\_Mf\_liverA, Unigene10756\_Mf\_liverA, CL3669.Contig1\_Mf\_liverA, Unigene27249\_Mf\_liverA, Unigene43515\_Mf\_liverA, Unigene11097\_Mf\_liverA, NM\_134156, Unigene4720\_Mf\_liverA, Unigene37389\_Mf\_liverA, CL4156.Contig1\_Mf\_liverA, Unigene32716\_Mf\_liverA, Unigene27248\_Mf\_liverA, Unigene29253\_Mf\_liverA, Unigene34010\_Mf\_liverA, Unigene152\_Mf\_liverA, Unigene31852\_Mf\_liverA, Unigene5456\_Mf\_liverA, Unigene37076\_Mf\_liverA, Unigene2678\_Mf\_liverA, Unigene1280\_Mf\_liverA, Unigene30288\_Mf\_liverA, CL1738.Contig1\_Mf\_liverA, CL5576.Contig1\_Mf\_liverA, CL3339.Contig1\_Mf\_liverA, Unigene2746\_Mf\_liverA, Unigene4593\_Mf\_liverA, Unigene5236\_Mf\_liverA, Unigene10496\_Mf\_liverA, Unigene28021\_Mf\_liverA, Unigene45904\_Mf\_liverA, Unigene37433\_Mf\_liverA, CL1442.Contig1\_Mf\_liverA, NM\_009933, CL5528.Contig1\_Mf\_liverA, Unigene2162\_Mf\_liverA, CL425.Contig1\_Mf\_liverA, CL3150.Contig1\_Mf\_liverA, CL2797.Contig2\_Mf\_liverA, Unigene37243\_Mf\_liverA, Unigene39403\_Mf\_liverA, Unigene30587\_Mf\_liverA, Unigene5693\_Mf\_liverA, Unigene39970\_Mf\_liverA, Unigene13271\_Mf\_liverA, CL3393.Contig1\_Mf\_liverA, Unigene19821\_Mf\_liverA, CL4057.Contig1\_Mf\_liverA, Unigene29876\_Mf\_liverA, CL3800.Contig1\_Mf\_liverA, Unigene25080\_Mf\_liverA, Unigene28687\_Mf\_liverA, CL1988.Contig3\_Mf\_liverA, Unigene29885\_Mf\_liverA, CL3055.Contig1\_Mf\_liverA, NM\_028785, Unigene5758\_Mf\_liverA, NM\_009609, CL1810.Contig1\_Mf\_liverA, CL4033.Contig1\_Mf\_liverA, Unigene24547\_Mf\_liverA, CL3519.Contig1\_Mf\_liverA, Unigene8033\_Mf\_liverA, Unigene28025\_Mf\_liverA, CL1493.Contig1\_Mf\_liverA, Unigene24801\_Mf\_liverA, CL3685.Contig1\_Mf\_liverA, CL5764.Contig1\_Mf\_liverA, Unigene39231\_Mf\_liverA, Unigene41226\_Mf\_liverA, Unigene4909\_Mf\_liverA, Unigene34197\_Mf\_liverA, Unigene24471\_Mf\_liverA, NM\_026823, Unigene35168\_Mf\_liverA, Unigene15888\_Mf\_liverA, NM\_010162, Unigene15529\_Mf\_liverA, Unigene37262\_Mf\_liverA, Unigene12908\_Mf\_liverA, NM\_010469, CL2251.Contig1\_Mf\_liverA, CL4490.Contig2\_Mf\_liverA, CL5293.Contig1\_Mf\_liverA, Unigene25595\_Mf\_liverA, Unigene14263\_Mf\_liverA, Unigene14286\_Mf\_liverA, NM\_016861, Unigene39011\_Mf\_liverA, Unigene386\_Mf\_liverA, Unigene26250\_Mf\_liverA, Unigene30795\_Mf\_liverA, NM\_024452, Unigene20432\_Mf\_liverA, Unigene48460\_Mf\_liverA, NM\_011580, Unigene14330\_Mf\_liverA, Unigene35046\_Mf\_liverA, Unigene28459\_Mf\_liverA, Unigene37245\_Mf\_liverA, Unigene16671\_Mf\_liverA, Unigene21693\_Mf\_liverA, Unigene13535\_Mf\_liverA, Unigene13772\_Mf\_liverA, Unigene29302\_Mf\_liverA, Unigene33428\_Mf\_liverA, Unigene37574\_Mf\_liverA, Unigene36176\_Mf\_liverA, Unigene36699\_Mf\_liverA, Unigene7968\_Mf\_liverA, Unigene36667\_Mf\_liverA, Unigene18340\_Mf\_liverA, NM\_001164598, Unigene30429\_Mf\_liverA, Unigene30412\_Mf\_liverA, Unigene4983\_Mf\_liverA, Unigene38317\_Mf\_liverA, Unigene5512\_Mf\_liverA, CL373.Contig7\_Mf\_liverA, Unigene42855\_Mf\_liverA, Unigene29889\_Mf\_liverA, Unigene2939\_Mf\_liverA, Unigene36728\_Mf\_liverA, Unigene9466\_Mf\_liverA, Unigene35884\_Mf\_liverA, CL5640.Contig1\_Mf\_liverA, NM\_020559, Unigene28142\_Mf\_liverA, CL3835.Contig2\_Mf\_liverA, Unigene38831\_Mf\_liverA, NM\_145824, Unigene30356\_Mf\_liverA, Unigene38124\_Mf\_liverA, Unigene46870\_Mf\_liverA, Unigene14603\_Mf\_liverA, CL4336.Contig3\_Mf\_liverA, Unigene8473\_Mf\_liverA, Unigene29008\_Mf\_liverA, Unigene27593\_Mf\_liverA, Unigene19049\_Mf\_liverA, Unigene33508\_Mf\_liverA, Unigene37132\_Mf\_liverA, CL5358.Contig1\_Mf\_liverA, Unigene48792\_Mf\_liverA, Unigene31263\_Mf\_liverA, NM\_011072, Unigene14816\_Mf\_liverA, Unigene7969\_Mf\_liverA, Unigene21466\_Mf\_liverA, Unigene30303\_Mf\_liverA, NM\_173753, Unigene34810\_Mf\_liverA, Unigene27420\_Mf\_liverA, CL6038.Contig2\_Mf\_liverA, Unigene36889\_Mf\_liverA, Unigene13307\_Mf\_liverA, Unigene37999\_Mf\_liverA, Unigene40020\_Mf\_liverA, Unigene37904\_Mf\_liverA, CL3483.Contig1\_Mf\_liverA, Unigene34789\_Mf\_liverA, CL4141.Contig1\_Mf\_liverA, Unigene25057\_Mf\_liverA, NM\_145419, CL5978.Contig3\_Mf\_liverA, CL5191.Contig2\_Mf\_liverA, Unigene13658\_Mf\_liverA, NM\_175260, Unigene4686\_Mf\_liverA, Unigene11\_Mf\_liverA, Unigene4630\_Mf\_liverA, Unigene11544\_Mf\_liverA, NM\_018780, Unigene18117\_Mf\_liverA, CL3575.Contig1\_Mf\_liverA, CL1493.Contig2\_Mf\_liverA, CL2117.Contig1\_Mf\_liverA, CL3565.Contig1\_Mf\_liverA, Unigene36988\_Mf\_liverA, Unigene28094\_Mf\_liverA, Unigene40824\_Mf\_liverA, CL2574.Contig1\_Mf\_liverA, Unigene5941\_Mf\_liverA, Unigene38104\_Mf\_liverA, Unigene23328\_Mf\_liverA, Unigene25462\_Mf\_liverA, Unigene38065\_Mf\_liverA, Unigene16684\_Mf\_liverA, Unigene36626\_Mf\_liverA, NM\_001029934, NM\_029562, Unigene28822\_Mf\_liverA, Unigene36691\_Mf\_liverA, Unigene31988\_Mf\_liverA, NM\_019703, Unigene19297\_Mf\_liverA, CL2326.Contig1\_Mf\_liverA, Unigene151\_Mf\_liverA, CL848.Contig2\_Mf\_liverA, Unigene23870\_Mf\_liverA, NM\_008808, Unigene4768\_Mf\_liverA, Unigene10774\_Mf\_liverA, CL2131.Contig4\_Mf\_liverA, Unigene25662\_Mf\_liverA, CL3198.Contig1\_Mf\_liverA, Unigene13143\_Mf\_liverA, Unigene37460\_Mf\_liverA, Unigene31517\_Mf\_liverA, Unigene31231\_Mf\_liverA, Unigene36628\_Mf\_liverA, NM\_007811, Unigene24323\_Mf\_liverA, NM\_008538, NM\_011305, NM\_008610, Unigene12200\_Mf\_liverA, Unigene35859\_Mf\_liverA, CL3816.Contig1\_Mf\_liverA, Unigene36631\_Mf\_liverA, Unigene4510\_Mf\_liverA, CL3803.Contig2\_Mf\_liverA, Unigene24284\_Mf\_liverA, Unigene26873\_Mf\_liverA, Unigene27260\_Mf\_liverA, CL3268.Contig1\_Mf\_liverA, Unigene36845\_Mf\_liverA, Unigene14758\_Mf\_liverA, Unigene37729\_Mf\_liverA, NM\_010359, Unigene29940\_Mf\_liverA, CL795.Contig1\_Mf\_liverA, Unigene42812\_Mf\_liverA, Unigene5509\_Mf\_liverA, Unigene5325\_Mf\_liverA, CL5039.Contig2\_Mf\_liverA, Unigene1205\_Mf\_liverA, Unigene13841\_Mf\_liverA, CL1352.Contig1\_Mf\_liverA, CL6039.Contig1\_Mf\_liverA, Unigene24804\_Mf\_liverA, Unigene31206\_Mf\_liverA, NM\_011132, Unigene32412\_Mf\_liverA, Unigene17569\_Mf\_liverA, Unigene40924\_Mf\_liverA, NM\_009128, NM\_175472, Unigene7412\_Mf\_liverA, CL2240.Contig1\_Mf\_liverA, Unigene5783\_Mf\_liverA, Unigene29823\_Mf\_liverA, Unigene39385\_Mf\_liverA, Unigene22980\_Mf\_liverA, Unigene38339\_Mf\_liverA, Unigene15474\_Mf\_liverA, Unigene23448\_Mf\_liverA, CL1125.Contig1\_Mf\_liverA, Unigene37526\_Mf\_liverA, Unigene29510\_Mf\_liverA, NM\_010579, Unigene15681\_Mf\_liverA, Unigene7350\_Mf\_liverA, Unigene19083\_Mf\_liverA, Unigene673\_Mf\_liverA, Unigene4703\_Mf\_liverA, Unigene37096\_Mf\_liverA, Unigene5165\_Mf\_liverA, Unigene30707\_Mf\_liverA, NM\_133838, Unigene7674\_Mf\_liverA, CL3002.Contig1\_Mf\_liverA, NM\_008871, Unigene15058\_Mf\_liverA, Unigene35491\_Mf\_liverA, NM\_029872, Unigene37139\_Mf\_liverA, Unigene5906\_Mf\_liverA, Unigene34035\_Mf\_liverA, CL5688.Contig1\_Mf\_liverA, NM\_033444, Unigene4597\_Mf\_liverA, CL2791.Contig1\_Mf\_liverA, Unigene14170\_Mf\_liverA, Unigene35269\_Mf\_liverA, Unigene10135\_Mf\_liverA, Unigene22330\_Mf\_liverA, Unigene5011\_Mf\_liverA, Unigene5954\_Mf\_liverA, NM\_009883, CL4127.Contig1\_Mf\_liverA, Unigene33526\_Mf\_liverA, Unigene1327\_Mf\_liverA, CL1190.Contig3\_Mf\_liverA, Unigene8740\_Mf\_liverA, CL5978.Contig2\_Mf\_liverA, NM\_008776, Unigene36177\_Mf\_liverA, Unigene24566\_Mf\_liverA, NM\_007478, Unigene15170\_Mf\_liverA, Unigene4604\_Mf\_liverA, NM\_011418, Unigene560\_Mf\_liverA, Unigene24477\_Mf\_liverA, Unigene30731\_Mf\_liverA, Unigene30983\_Mf\_liverA, Unigene39749\_Mf\_liverA, Unigene36418\_Mf\_liverA, Unigene25052\_Mf\_liverA, Unigene550\_Mf\_liverA, Unigene38311\_Mf\_liverA, Unigene21843\_Mf\_liverA, CL2355.Contig1\_Mf\_liverA, Unigene36910\_Mf\_liverA, Unigene28314\_Mf\_liverA, Unigene37153\_Mf\_liverA, Unigene21562\_Mf\_liverA, Unigene15592\_Mf\_liverA, NM\_015767, Unigene37711\_Mf\_liverA, Unigene41583\_Mf\_liverA, Unigene5418\_Mf\_liverA, Unigene2195\_Mf\_liverA, Unigene93\_Mf\_liverA, NM\_146007, Unigene30892\_Mf\_liverA, Unigene803\_Mf\_liverA, CL3911.Contig2\_Mf\_liverA, Unigene34866\_Mf\_liverA, Unigene29292\_Mf\_liverA, Unigene14765\_Mf\_liverA, NM\_144848, Unigene31885\_Mf\_liverA, NM\_001033481, Unigene4681\_Mf\_liverA, CL548.Contig1\_Mf\_liverA, NM\_007392, Unigene14284\_Mf\_liverA, Unigene26474\_Mf\_liverA, Unigene25787\_Mf\_liverA, Unigene24506\_Mf\_liverA, NM\_009450, Unigene34609\_Mf\_liverA, Unigene25721\_Mf\_liverA, NM\_009592, CL3669.Contig2\_Mf\_liverA, Unigene8106\_Mf\_liverA, Unigene5852\_Mf\_liverA, Unigene36172\_Mf\_liverA, Unigene37334\_Mf\_liverA, NM\_018815, Unigene36851\_Mf\_liverA, Unigene14665\_Mf\_liverA, NM\_080638, Unigene7936\_Mf\_liverA, Unigene29424\_Mf\_liverA, Unigene34218\_Mf\_liverA, Unigene26053\_Mf\_liverA, CL4105.Contig1\_Mf\_liverA, CL4577.Contig1\_Mf\_liverA, Unigene25090\_Mf\_liverA, CL238.Contig1\_Mf\_liverA, CL2423.Contig1\_Mf\_liverA, CL5560.Contig1\_Mf\_liverA, Unigene22575\_Mf\_liverA, Unigene5248\_Mf\_liverA, Unigene785\_Mf\_liverA, Unigene26422\_Mf\_liverA, Unigene38689\_Mf\_liverA, Unigene29334\_Mf\_liverA, NM\_010877, NM\_021273, Unigene38919\_Mf\_liverA, Unigene1137\_Mf\_liverA, CL5586.Contig1\_Mf\_liverA, Unigene21255\_Mf\_liverA, Unigene34341\_Mf\_liverA, Unigene30142\_Mf\_liverA, Unigene35816\_Mf\_liverA, Unigene13940\_Mf\_liverA, CL593.Contig2\_Mf\_liverA, NM\_145474, Unigene33054\_Mf\_liverA, NM\_009776, Unigene13266\_Mf\_liverA, NM\_153193, Unigene15205\_Mf\_liverA, Unigene5138\_Mf\_liverA, Unigene37150\_Mf\_liverA, Unigene5330\_Mf\_liverA, NM\_177320, Unigene34143\_Mf\_liverA, Unigene38657\_Mf\_liverA, Unigene653\_Mf\_liverA, Unigene23869\_Mf\_liverA, Unigene9150\_Mf\_liverA, Unigene5598\_Mf\_liverA, Unigene13363\_Mf\_liverA, CL2855.Contig2\_Mf\_liverA, Unigene36175\_Mf\_liverA, Unigene28226\_Mf\_liverA, Unigene44317\_Mf\_liverA, Unigene37278\_Mf\_liverA, Unigene2919\_Mf\_liverA, Unigene21561\_Mf\_liverA, Unigene1479\_Mf\_liverA, Unigene4922\_Mf\_liverA, Unigene14637\_Mf\_liverA, NM\_153505, CL2159.Contig2\_Mf\_liverA, Unigene44882\_Mf\_liverA, Unigene37662\_Mf\_liverA, Unigene31654\_Mf\_liverA, Unigene9990\_Mf\_liverA, NM\_001104531, NM\_153055, Unigene4944\_Mf\_liverA, Unigene33560\_Mf\_liverA, Unigene23158\_Mf\_liverA, CL3055.Contig2\_Mf\_liverA, Unigene37575\_Mf\_liverA, Unigene25398\_Mf\_liverA, Unigene27547\_Mf\_liverA, CL1736.Contig2\_Mf\_liverA, Unigene5180\_Mf\_liverA, Unigene21842\_Mf\_liverA, Unigene12909\_Mf\_liverA, Unigene32889\_Mf\_liverA, Unigene4723\_Mf\_liverA, Unigene33271\_Mf\_liverA, Unigene32332\_Mf\_liverA, Unigene37470\_Mf\_liverA, CL5254.Contig1\_Mf\_liverA, Unigene37542\_Mf\_liverA, NM\_009713, NM\_009087, Unigene35169\_Mf\_liverA, Unigene21256\_Mf\_liverA, NR\_003552, Unigene5169\_Mf\_liverA, Unigene16465\_Mf\_liverA, Unigene32059\_Mf\_liverA, Unigene32202\_Mf\_liverA, Unigene5472\_Mf\_liverA, NM\_001025573, Unigene26515\_Mf\_liverA, Unigene31251\_Mf\_liverA, NM\_011503, Unigene40610\_Mf\_liverA, NM\_172121, CL186.Contig3\_Mf\_liverA, Unigene583\_Mf\_liverA, Unigene4697\_Mf\_liverA, CL4736.Contig1\_Mf\_liverA, Unigene27026\_Mf\_liverA, Unigene14916\_Mf\_liverA, Unigene35858\_Mf\_liverA, Unigene30808\_Mf\_liverA, Unigene30039\_Mf\_liverA, Unigene29231\_Mf\_liverA, Unigene37442\_Mf\_liverA, Unigene50250\_Mf\_liverA, Unigene1440\_Mf\_liverA, Unigene35958\_Mf\_liverA, Unigene43037\_Mf\_liverA, Unigene13525\_Mf\_liverA, Unigene24395\_Mf\_liverA, Unigene4938\_Mf\_liverA, Unigene5815\_Mf\_liverA, Unigene1129\_Mf\_liverA, Unigene33168\_Mf\_liverA, Unigene27483\_Mf\_liverA, Unigene28662\_Mf\_liverA, CL3738.Contig1\_Mf\_liverA, CL2439.Contig1\_Mf\_liverA, Unigene4781\_Mf\_liverA, Unigene46615\_Mf\_liverA, Unigene35935\_Mf\_liverA, Unigene29426\_Mf\_liverA, Unigene30494\_Mf\_liverA, Unigene13462\_Mf\_liverA, CL5049.Contig2\_Mf\_liverA, Unigene34446\_Mf\_liverA, Unigene13683\_Mf\_liverA, Unigene11686\_Mf\_liverA, Unigene24755\_Mf\_liverA, Unigene9698\_Mf\_liverA, Unigene28499\_Mf\_liverA, Unigene30585\_Mf\_liverA, Unigene36420\_Mf\_liverA, Unigene542\_Mf\_liverA, Unigene38593\_Mf\_liverA, NM\_033325, Unigene24758\_Mf\_liverA, Unigene23271\_Mf\_liverA, Unigene25092\_Mf\_liverA, CL5828.Contig2\_Mf\_liverA, Unigene28527\_Mf\_liverA, Unigene35039\_Mf\_liverA, Unigene29938\_Mf\_liverA, CL2625.Contig2\_Mf\_liverA, CL725.Contig1\_Mf\_liverA, Unigene30947\_Mf\_liverA, CL5293.Contig2\_Mf\_liverA, CL1165.Contig2\_Mf\_liverA, Unigene10335\_Mf\_liverA, Unigene35090\_Mf\_liverA, Unigene14212\_Mf\_liverA, Unigene34962\_Mf\_liverA, CL1165.Contig4\_Mf\_liverA, CL4669.Contig1\_Mf\_liverA, NM\_028222, Unigene23185\_Mf\_liverA, Unigene29558\_Mf\_liverA, CL3692.Contig2\_Mf\_liverA, Unigene26083\_Mf\_liverA, NM\_013863, Unigene281\_Mf\_liverA, Unigene10313\_Mf\_liverA, Unigene29985\_Mf\_liverA, Unigene26580\_Mf\_liverA, Unigene5687\_Mf\_liverA, CL3104.Contig1\_Mf\_liverA, Unigene36593\_Mf\_liverA, Unigene5037\_Mf\_liverA, Unigene26336\_Mf\_liverA, Unigene5422\_Mf\_liverA, NM\_146016, Unigene36673\_Mf\_liverA, NM\_181517, CL5698.Contig1\_Mf\_liverA, Unigene31392\_Mf\_liverA, NM\_023256, CL591.Contig1\_Mf\_liverA, NM\_009636, Unigene4556\_Mf\_liverA, Unigene38237\_Mf\_liverA, NM\_007763, Unigene26055\_Mf\_liverA, CL139.Contig2\_Mf\_liverA, Unigene29399\_Mf\_liverA, CL2520.Contig1\_Mf\_liverA, Unigene38015\_Mf\_liverA, NM\_007896, Unigene24713\_Mf\_liverA, Unigene42975\_Mf\_liverA, NM\_144907, CL1828.Contig1\_Mf\_liverA, Unigene29308\_Mf\_liverA, CL1362.Contig1\_Mf\_liverA, CL523.Contig1\_Mf\_liverA, Unigene15914\_Mf\_liverA, Unigene5194\_Mf\_liverA, CL1063.Contig1\_Mf\_liverA, CL482.Contig1\_Mf\_liverA, Unigene22433\_Mf\_liverA, NM\_001167705, Unigene29405\_Mf\_liverA, Unigene32882\_Mf\_liverA, Unigene21263\_Mf\_liverA, Unigene7619\_Mf\_liverA, Unigene37698\_Mf\_liverA, Unigene2633\_Mf\_liverA, Unigene30878\_Mf\_liverA, Unigene40289\_Mf\_liverA, Unigene21013\_Mf\_liverA, Unigene15656\_Mf\_liverA, Unigene34234\_Mf\_liverA, Unigene24344\_Mf\_liverA, Unigene38331\_Mf\_liverA, Unigene5204\_Mf\_liverA, CL5001.Contig2\_Mf\_liverA, Unigene37099\_Mf\_liverA, Unigene37268\_Mf\_liverA, CL1256.Contig1\_Mf\_liverA, Unigene25070\_Mf\_liverA, Unigene24112\_Mf\_liverA, Unigene23273\_Mf\_liverA, Unigene24379\_Mf\_liverA, Unigene37263\_Mf\_liverA, Unigene36853\_Mf\_liverA, Unigene38110\_Mf\_liverA, NM\_009896, CL1119.Contig1\_Mf\_liverA, Unigene26194\_Mf\_liverA, Unigene31333\_Mf\_liverA, Unigene41158\_Mf\_liverA, Unigene25596\_Mf\_liverA, NM\_010763, CL3778.Contig2\_Mf\_liverA, Unigene3174\_Mf\_liverA, Unigene22978\_Mf\_liverA, Unigene22432\_Mf\_liverA, Unigene25046\_Mf\_liverA, Unigene13918\_Mf\_liverA, Unigene2745\_Mf\_liverA, NM\_001024205, CL787.Contig1\_Mf\_liverA, Unigene1162\_Mf\_liverA, CL4076.Contig1\_Mf\_liverA, Unigene25524\_Mf\_liverA, CL4847.Contig1\_Mf\_liverA, Unigene20128\_Mf\_liverA, NM\_001162917, CL3196.Contig2\_Mf\_liverA, Unigene47203\_Mf\_liverA, Unigene38406\_Mf\_liverA, CL3750.Contig2\_Mf\_liverA, CL4722.Contig1\_Mf\_liverA, Unigene33459\_Mf\_liverA, Unigene26398\_Mf\_liverA, Unigene25012\_Mf\_liverA, Unigene34258\_Mf\_liverA, Unigene5632\_Mf\_liverA, NM\_001001806, CL479.Contig1\_Mf\_liverA, Unigene27419\_Mf\_liverA, Unigene14582\_Mf\_liverA, Unigene34746\_Mf\_liverA, Unigene14715\_Mf\_liverA, Unigene24503\_Mf\_liverA, NM\_134059, Unigene37180\_Mf\_liverA, Unigene4523\_Mf\_liverA, Unigene27438\_Mf\_liverA, CL2722.Contig1\_Mf\_liverA, Unigene20116\_Mf\_liverA, CL5631.Contig1\_Mf\_liverA, Unigene31427\_Mf\_liverA, Unigene14896\_Mf\_liverA, Unigene30584\_Mf\_liverA, Unigene431\_Mf\_liverA, Unigene19885\_Mf\_liverA, Unigene36510\_Mf\_liverA, NM\_009091, Unigene44992\_Mf\_liverA, Unigene10820\_Mf\_liverA, NM\_010391, NM\_008889, NM\_010227, Unigene21317\_Mf\_liverA, Unigene30493\_Mf\_liverA, Unigene8132\_Mf\_liverA, NM\_019717, Unigene32058\_Mf\_liverA, Unigene27082\_Mf\_liverA, Unigene37178\_Mf\_liverA, Unigene5712\_Mf\_liverA, Unigene584\_Mf\_liverA, CL2962.Contig1\_Mf\_liverA, Unigene23055\_Mf\_liverA, CL442.Contig2\_Mf\_liverA, Unigene26214\_Mf\_liverA, NM\_010380, NM\_033374, CL2855.Contig1\_Mf\_liverA, CL4411.Contig4\_Mf\_liverA, NM\_001025388, Unigene13233\_Mf\_liverA, NM\_011099, Unigene5648\_Mf\_liverA, NM\_009447, NM\_001100182, CL1537.Contig1\_Mf\_liverA, NM\_010481, Unigene392\_Mf\_liverA, CL4086.Contig1\_Mf\_liverA, CL507.Contig1\_Mf\_liverA, Unigene23082\_Mf\_liverA, Unigene5360\_Mf\_liverA, Unigene15318\_Mf\_liverA, NM\_009178, Unigene13414\_Mf\_liverA, NM\_008293, Unigene1130\_Mf\_liverA, Unigene30154\_Mf\_liverA, Unigene15982\_Mf\_liverA, Unigene38208\_Mf\_liverA, CL4162.Contig1\_Mf\_liverA, Unigene35237\_Mf\_liverA, CL5993.Contig3\_Mf\_liverA, Unigene37259\_Mf\_liverA, Unigene12907\_Mf\_liverA, Unigene5260\_Mf\_liverA, Unigene15588\_Mf\_liverA, Unigene23013\_Mf\_liverA, CL4583.Contig2\_Mf\_liverA, Unigene36414\_Mf\_liverA, Unigene13894\_Mf\_liverA, Unigene5175\_Mf\_liverA, Unigene34219\_Mf\_liverA, Unigene33880\_Mf\_liverA, Unigene36543\_Mf\_liverA, NM\_011352, Unigene36328\_Mf\_liverA, CL1222.Contig1\_Mf\_liverA, Unigene6895\_Mf\_liverA, CL2327.Contig1\_Mf\_liverA, CL2688.Contig1\_Mf\_liverA, CL6039.Contig2\_Mf\_liverA, NM\_010393, Unigene35037\_Mf\_liverA, Unigene36710\_Mf\_liverA, Unigene5940\_Mf\_liverA, NM\_007393, CL1662.Contig1\_Mf\_liverA, Unigene28731\_Mf\_liverA, NM\_053072, Unigene1212\_Mf\_liverA, CL3549.Contig1\_Mf\_liverA, NM\_009706, Unigene24085\_Mf\_liverA, NM\_007986, CL4106.Contig1\_Mf\_liverA, CL3750.Contig1\_Mf\_liverA, CL1588.Contig3\_Mf\_liverA, Unigene8016\_Mf\_liverA, Unigene18796\_Mf\_liverA, NM\_009448, CL5307.Contig1\_Mf\_liverA, Unigene21337\_Mf\_liverA, Unigene31730\_Mf\_liverA, Unigene51055\_Mf\_liverA, Unigene28873\_Mf\_liverA, CL4432.Contig2\_Mf\_liverA, Unigene33525\_Mf\_liverA, Unigene4540\_Mf\_liverA, Unigene24204\_Mf\_liverA, Unigene37310\_Mf\_liverA, Unigene9406\_Mf\_liverA, Unigene27803\_Mf\_liverA, Unigene15026\_Mf\_liverA, Unigene1221\_Mf\_liverA, CL1588.Contig1\_Mf\_liverA, NM\_017379, Unigene39875\_Mf\_liverA, NM\_001081274, Unigene25238\_Mf\_liverA, Unigene7224\_Mf\_liverA, Unigene26309\_Mf\_liverA, Unigene17632\_Mf\_liverA, Unigene5745\_Mf\_liverA, Unigene21336\_Mf\_liverA, Unigene12889\_Mf\_liverA, Unigene38514\_Mf\_liverA, Unigene665\_Mf\_liverA, Unigene35678\_Mf\_liverA, CL4434.Contig1\_Mf\_liverA, Unigene5147\_Mf\_liverA, Unigene8560\_Mf\_liverA, Unigene13396\_Mf\_liverA, Unigene41419\_Mf\_liverA, Unigene21359\_Mf\_liverA, Unigene36487\_Mf\_liverA, Unigene37474\_Mf\_liverA, Unigene16891\_Mf\_liverA, Unigene7733\_Mf\_liverA, Unigene37819\_Mf\_liverA, Unigene5886\_Mf\_liverA, Unigene27274\_Mf\_liverA, Unigene15493\_Mf\_liverA |
| macromolecular complex | Unigene9466\_Mf\_liverA, NM\_176843, Unigene35884\_Mf\_liverA, NM\_009450, NM\_007896, NM\_009898, CL3835.Contig2\_Mf\_liverA, NM\_145824, Unigene30356\_Mf\_liverA, Unigene24111\_Mf\_liverA, Unigene28186\_Mf\_liverA, Unigene38124\_Mf\_liverA, Unigene36851\_Mf\_liverA, Unigene29308\_Mf\_liverA, Unigene9081\_Mf\_liverA, Unigene14665\_Mf\_liverA, NM\_080638, NM\_010378, Unigene34218\_Mf\_liverA, Unigene5194\_Mf\_liverA, Unigene29008\_Mf\_liverA, CL1063.Contig1\_Mf\_liverA, Unigene22433\_Mf\_liverA, Unigene33138\_Mf\_liverA, Unigene25090\_Mf\_liverA, CL2423.Contig1\_Mf\_liverA, Unigene32882\_Mf\_liverA, CL5358.Contig1\_Mf\_liverA, Unigene31263\_Mf\_liverA, CL5560.Contig1\_Mf\_liverA, Unigene2633\_Mf\_liverA, Unigene5248\_Mf\_liverA, Unigene7969\_Mf\_liverA, Unigene26422\_Mf\_liverA, CL33.Contig4\_Mf\_liverA, Unigene29334\_Mf\_liverA, Unigene30878\_Mf\_liverA, Unigene7510\_Mf\_liverA, CL4757.Contig1\_Mf\_liverA, Unigene34234\_Mf\_liverA, NR\_004446, Unigene38331\_Mf\_liverA, Unigene36889\_Mf\_liverA, Unigene5204\_Mf\_liverA, Unigene13307\_Mf\_liverA, Unigene4557\_Mf\_liverA, CL5001.Contig2\_Mf\_liverA, Unigene21255\_Mf\_liverA, Unigene34789\_Mf\_liverA, Unigene24112\_Mf\_liverA, CL4141.Contig1\_Mf\_liverA, Unigene35431\_Mf\_liverA, Unigene25057\_Mf\_liverA, Unigene23273\_Mf\_liverA, Unigene37263\_Mf\_liverA, CL1119.Contig1\_Mf\_liverA, Unigene35816\_Mf\_liverA, Unigene13658\_Mf\_liverA, Unigene28331\_Mf\_liverA, Unigene31333\_Mf\_liverA, CL33.Contig3\_Mf\_liverA, Unigene4686\_Mf\_liverA, Unigene33054\_Mf\_liverA, Unigene11544\_Mf\_liverA, Unigene5138\_Mf\_liverA, Unigene15205\_Mf\_liverA, Unigene18117\_Mf\_liverA, Unigene3174\_Mf\_liverA, Unigene37150\_Mf\_liverA, CL2117.Contig1\_Mf\_liverA, Unigene22432\_Mf\_liverA, Unigene25046\_Mf\_liverA, Unigene21857\_Mf\_liverA, Unigene5941\_Mf\_liverA, Unigene38657\_Mf\_liverA, Unigene2745\_Mf\_liverA, NM\_001024205, Unigene23869\_Mf\_liverA, CL4847.Contig1\_Mf\_liverA, Unigene28822\_Mf\_liverA, NM\_053214, CL2855.Contig2\_Mf\_liverA, Unigene20128\_Mf\_liverA, Unigene25091\_Mf\_liverA, NM\_019703, NM\_177093, CL2326.Contig1\_Mf\_liverA, Unigene44317\_Mf\_liverA, Unigene25047\_Mf\_liverA, CL4722.Contig1\_Mf\_liverA, Unigene14907\_Mf\_liverA, Unigene2919\_Mf\_liverA, Unigene23870\_Mf\_liverA, Unigene7476\_Mf\_liverA, Unigene1479\_Mf\_liverA, CL2131.Contig4\_Mf\_liverA, Unigene25976\_Mf\_liverA, Unigene25662\_Mf\_liverA, Unigene13143\_Mf\_liverA, CL3198.Contig1\_Mf\_liverA, Unigene5632\_Mf\_liverA, Unigene37460\_Mf\_liverA, NM\_153505, Unigene31517\_Mf\_liverA, Unigene9475\_Mf\_liverA, Unigene37662\_Mf\_liverA, Unigene13945\_Mf\_liverA, Unigene30459\_Mf\_liverA, NM\_134059, Unigene4523\_Mf\_liverA, Unigene27438\_Mf\_liverA, NM\_001143689, CL5631.Contig1\_Mf\_liverA, Unigene37575\_Mf\_liverA, Unigene43515\_Mf\_liverA, CL3803.Contig2\_Mf\_liverA, Unigene27547\_Mf\_liverA, Unigene19885\_Mf\_liverA, CL4040.Contig2\_Mf\_liverA, NM\_009091, CL3268.Contig1\_Mf\_liverA, Unigene36845\_Mf\_liverA, NM\_010391, Unigene32716\_Mf\_liverA, Unigene24157\_Mf\_liverA, Unigene42812\_Mf\_liverA, Unigene152\_Mf\_liverA, Unigene27082\_Mf\_liverA, Unigene5509\_Mf\_liverA, Unigene37076\_Mf\_liverA, Unigene2678\_Mf\_liverA, Unigene5325\_Mf\_liverA, CL5039.Contig2\_Mf\_liverA, Unigene30288\_Mf\_liverA, NM\_010380, Unigene26214\_Mf\_liverA, CL5576.Contig1\_Mf\_liverA, CL2855.Contig1\_Mf\_liverA, Unigene2746\_Mf\_liverA, NM\_001025388, Unigene17569\_Mf\_liverA, NM\_009447, Unigene37433\_Mf\_liverA, Unigene5472\_Mf\_liverA, CL1537.Contig1\_Mf\_liverA, CL4086.Contig1\_Mf\_liverA, NM\_011503, Unigene40610\_Mf\_liverA, CL507.Contig1\_Mf\_liverA, Unigene7412\_Mf\_liverA, CL425.Contig1\_Mf\_liverA, Unigene30815\_Mf\_liverA, Unigene15982\_Mf\_liverA, CL4162.Contig1\_Mf\_liverA, Unigene37526\_Mf\_liverA, CL5993.Contig3\_Mf\_liverA, NM\_010579, Unigene29231\_Mf\_liverA, Unigene7350\_Mf\_liverA, Unigene19083\_Mf\_liverA, Unigene50250\_Mf\_liverA, Unigene4985\_Mf\_liverA, Unigene19821\_Mf\_liverA, Unigene5165\_Mf\_liverA, CL4057.Contig1\_Mf\_liverA, Unigene1440\_Mf\_liverA, CL3800.Contig1\_Mf\_liverA, Unigene30707\_Mf\_liverA, Unigene13525\_Mf\_liverA, Unigene25080\_Mf\_liverA, Unigene34219\_Mf\_liverA, Unigene4938\_Mf\_liverA, Unigene36543\_Mf\_liverA, Unigene7674\_Mf\_liverA, CL3002.Contig1\_Mf\_liverA, CL1222.Contig1\_Mf\_liverA, CL1810.Contig1\_Mf\_liverA, NM\_009609, Unigene5758\_Mf\_liverA, NM\_029872, Unigene27483\_Mf\_liverA, CL2439.Contig1\_Mf\_liverA, Unigene35935\_Mf\_liverA, Unigene29426\_Mf\_liverA, Unigene36710\_Mf\_liverA, Unigene5940\_Mf\_liverA, NM\_007393, CL5049.Contig2\_Mf\_liverA, Unigene34446\_Mf\_liverA, NM\_033444, Unigene13683\_Mf\_liverA, Unigene24755\_Mf\_liverA, Unigene23271\_Mf\_liverA, Unigene13106\_Mf\_liverA, Unigene34197\_Mf\_liverA, Unigene24471\_Mf\_liverA, Unigene28527\_Mf\_liverA, Unigene5954\_Mf\_liverA, Unigene29938\_Mf\_liverA, NM\_009448, CL1190.Contig3\_Mf\_liverA, Unigene8740\_Mf\_liverA, CL2251.Contig1\_Mf\_liverA, CL4432.Contig2\_Mf\_liverA, Unigene4540\_Mf\_liverA, Unigene14263\_Mf\_liverA, Unigene37310\_Mf\_liverA, NM\_016861, Unigene4604\_Mf\_liverA, NM\_011418, Unigene26250\_Mf\_liverA, Unigene48460\_Mf\_liverA, Unigene39749\_Mf\_liverA, Unigene37245\_Mf\_liverA, NM\_017379, Unigene16671\_Mf\_liverA, CL2355.Contig1\_Mf\_liverA, Unigene13535\_Mf\_liverA, Unigene37153\_Mf\_liverA, CL3104.Contig1\_Mf\_liverA, Unigene5422\_Mf\_liverA, Unigene30814\_Mf\_liverA, Unigene7968\_Mf\_liverA, Unigene36667\_Mf\_liverA, CL4434.Contig1\_Mf\_liverA, Unigene5147\_Mf\_liverA, Unigene18340\_Mf\_liverA, CL3911.Contig2\_Mf\_liverA, Unigene4983\_Mf\_liverA, CL5698.Contig1\_Mf\_liverA, Unigene31392\_Mf\_liverA, NM\_023256, Unigene4681\_Mf\_liverA, Unigene37474\_Mf\_liverA, Unigene42855\_Mf\_liverA, NM\_007392, Unigene14284\_Mf\_liverA, CL591.Contig1\_Mf\_liverA, Unigene4556\_Mf\_liverA, Unigene15493\_Mf\_liverA |
| female germ cell nucleus | NM\_008538, Unigene4768\_Mf\_liverA |
| muscle myosin complex | Unigene24755\_Mf\_liverA, Unigene37662\_Mf\_liverA |
| H4/H2A histone acetyltransferase complex | NM\_009609, NM\_177093, NM\_007393, CL1222.Contig1\_Mf\_liverA |
| integral to mitochondrial membrane | Unigene38208\_Mf\_liverA, CL3104.Contig1\_Mf\_liverA, Unigene5512\_Mf\_liverA |
| recycling endosome | Unigene22330\_Mf\_liverA, Unigene24506\_Mf\_liverA, Unigene2746\_Mf\_liverA, Unigene2745\_Mf\_liverA, Unigene24503\_Mf\_liverA, Unigene1440\_Mf\_liverA |
| cyclin-dependent protein kinase holoenzyme complex | Unigene9466\_Mf\_liverA, Unigene19885\_Mf\_liverA, Unigene7350\_Mf\_liverA |
| male pronucleus | Unigene19297\_Mf\_liverA, Unigene10820\_Mf\_liverA |
| axon | CL4583.Contig2\_Mf\_liverA, Unigene29399\_Mf\_liverA, Unigene13525\_Mf\_liverA, Unigene36414\_Mf\_liverA, Unigene34197\_Mf\_liverA, Unigene13363\_Mf\_liverA, CL1222.Contig1\_Mf\_liverA, Unigene36417\_Mf\_liverA, Unigene4720\_Mf\_liverA, CL4722.Contig1\_Mf\_liverA, Unigene5037\_Mf\_liverA, Unigene8132\_Mf\_liverA, Unigene34866\_Mf\_liverA, Unigene37076\_Mf\_liverA, Unigene37259\_Mf\_liverA, CL1738.Contig1\_Mf\_liverA, CL2117.Contig1\_Mf\_liverA, Unigene29231\_Mf\_liverA, Unigene36420\_Mf\_liverA, Unigene36418\_Mf\_liverA, Unigene5165\_Mf\_liverA |
| nuclear envelope | Unigene26515\_Mf\_liverA, Unigene34197\_Mf\_liverA, Unigene28142\_Mf\_liverA, Unigene21255\_Mf\_liverA, Unigene38406\_Mf\_liverA, Unigene13414\_Mf\_liverA, Unigene25057\_Mf\_liverA, Unigene15703\_Mf\_liverA, NM\_018815, Unigene51055\_Mf\_liverA, NM\_080638, Unigene8132\_Mf\_liverA, CL5254.Contig1\_Mf\_liverA, Unigene42812\_Mf\_liverA, Unigene29253\_Mf\_liverA, NM\_181517, Unigene5456\_Mf\_liverA, CL4162.Contig1\_Mf\_liverA, Unigene21256\_Mf\_liverA, Unigene14212\_Mf\_liverA, CL2117.Contig1\_Mf\_liverA, NM\_033444, Unigene16891\_Mf\_liverA, CL4106.Contig1\_Mf\_liverA |
| endosome lumen | Unigene13525\_Mf\_liverA, Unigene15592\_Mf\_liverA |
| Z disc | Unigene26250\_Mf\_liverA, Unigene24755\_Mf\_liverA, Unigene9475\_Mf\_liverA, CL4847.Contig1\_Mf\_liverA, Unigene2678\_Mf\_liverA, CL2251.Contig1\_Mf\_liverA |
| leading edge membrane | Unigene30707\_Mf\_liverA, Unigene25080\_Mf\_liverA, Unigene31852\_Mf\_liverA, Unigene34197\_Mf\_liverA, Unigene16671\_Mf\_liverA, Unigene5138\_Mf\_liverA, Unigene29938\_Mf\_liverA, CL2117.Contig1\_Mf\_liverA, Unigene5693\_Mf\_liverA, CL795.Contig1\_Mf\_liverA |
| myofibril | NM\_007478, Unigene9475\_Mf\_liverA, CL4847.Contig1\_Mf\_liverA, Unigene2678\_Mf\_liverA, Unigene4557\_Mf\_liverA, CL1222.Contig1\_Mf\_liverA, NM\_134156, NM\_008610, Unigene37474\_Mf\_liverA, Unigene26250\_Mf\_liverA, CL1352.Contig1\_Mf\_liverA, NM\_007392, Unigene24755\_Mf\_liverA, Unigene4556\_Mf\_liverA, NM\_001025388, CL2251.Contig1\_Mf\_liverA |
| melanosome | CL725.Contig1\_Mf\_liverA, Unigene34789\_Mf\_liverA, CL3816.Contig1\_Mf\_liverA, Unigene21255\_Mf\_liverA, Unigene21256\_Mf\_liverA, Unigene28021\_Mf\_liverA |
| extrinsic to plasma membrane | CL4156.Contig1\_Mf\_liverA, Unigene24284\_Mf\_liverA, Unigene37243\_Mf\_liverA, Unigene2919\_Mf\_liverA, Unigene51055\_Mf\_liverA, Unigene35958\_Mf\_liverA, Unigene1440\_Mf\_liverA, Unigene34218\_Mf\_liverA |
| microfibril | Unigene12230\_Mf\_liverA, Unigene18510\_Mf\_liverA |
| collagen type IV | Unigene34123\_Mf\_liverA, Unigene34124\_Mf\_liverA |
| intercellular canaliculus | CL4033.Contig1\_Mf\_liverA, CL5586.Contig1\_Mf\_liverA |
| neuromuscular junction | CL4434.Contig1\_Mf\_liverA, Unigene7733\_Mf\_liverA, CL507.Contig1\_Mf\_liverA, Unigene42975\_Mf\_liverA |
| aggresome | CL1190.Contig3\_Mf\_liverA, Unigene35816\_Mf\_liverA, Unigene9406\_Mf\_liverA |
| dystrophin-associated glycoprotein complex | Unigene9475\_Mf\_liverA, CL4847.Contig1\_Mf\_liverA, Unigene2678\_Mf\_liverA |
| autophagic vacuole membrane | CL3685.Contig1\_Mf\_liverA, Unigene36328\_Mf\_liverA |
| female pronucleus | Unigene19297\_Mf\_liverA, Unigene10820\_Mf\_liverA |
| keratin filament | CL1537.Contig1\_Mf\_liverA, CL4847.Contig1\_Mf\_liverA |
| vesicle membrane | Unigene4909\_Mf\_liverA, Unigene36328\_Mf\_liverA, Unigene5204\_Mf\_liverA, NM\_009898, Unigene40020\_Mf\_liverA, NM\_134156, CL2855.Contig2\_Mf\_liverA, CL1828.Contig1\_Mf\_liverA, Unigene29424\_Mf\_liverA, Unigene28331\_Mf\_liverA, Unigene4922\_Mf\_liverA, Unigene5632\_Mf\_liverA, Unigene29008\_Mf\_liverA, Unigene4686\_Mf\_liverA, Unigene27082\_Mf\_liverA, CL5993.Contig3\_Mf\_liverA, Unigene9990\_Mf\_liverA, CL3816.Contig1\_Mf\_liverA, CL548.Contig1\_Mf\_liverA, Unigene28094\_Mf\_liverA, CL2855.Contig1\_Mf\_liverA, Unigene8054\_Mf\_liverA, Unigene34810\_Mf\_liverA, Unigene34234\_Mf\_liverA |
| Ada2/Gcn5/Ada3 transcription activator complex | Unigene34219\_Mf\_liverA, Unigene1479\_Mf\_liverA, Unigene30288\_Mf\_liverA |
| filopodium membrane | Unigene31852\_Mf\_liverA, Unigene29424\_Mf\_liverA |
| spherical high-density lipoprotein particle | CL3911.Contig2\_Mf\_liverA, CL1190.Contig3\_Mf\_liverA |
| T cell receptor complex | Unigene25090\_Mf\_liverA, Unigene25091\_Mf\_liverA |
| pre-autophagosomal structure | CL3685.Contig1\_Mf\_liverA, Unigene13307\_Mf\_liverA, Unigene13535\_Mf\_liverA |
| protein-lipid complex | CL3911.Contig2\_Mf\_liverA, Unigene9081\_Mf\_liverA, CL1190.Contig3\_Mf\_liverA, Unigene30815\_Mf\_liverA, Unigene30814\_Mf\_liverA, Unigene24157\_Mf\_liverA |
| plasma lipoprotein particle | CL3911.Contig2\_Mf\_liverA, Unigene9081\_Mf\_liverA, CL1190.Contig3\_Mf\_liverA, Unigene30815\_Mf\_liverA, Unigene30814\_Mf\_liverA, Unigene24157\_Mf\_liverA |
| recycling endosome membrane | Unigene22330\_Mf\_liverA, Unigene2746\_Mf\_liverA, Unigene2745\_Mf\_liverA |
| intracellular organelle part | NM\_176843, Unigene34656\_Mf\_liverA, NM\_009898, Unigene5852\_Mf\_liverA, NM\_009592, Unigene7612\_Mf\_liverA, NM\_018815, Unigene36851\_Mf\_liverA, Unigene14665\_Mf\_liverA, NM\_080638, Unigene29424\_Mf\_liverA, Unigene34218\_Mf\_liverA, CL4105.Contig1\_Mf\_liverA, CL5560.Contig1\_Mf\_liverA, Unigene22575\_Mf\_liverA, Unigene5248\_Mf\_liverA, Unigene39886\_Mf\_liverA, Unigene26422\_Mf\_liverA, CL33.Contig4\_Mf\_liverA, Unigene29334\_Mf\_liverA, Unigene15064\_Mf\_liverA, NM\_010877, Unigene34124\_Mf\_liverA, Unigene38919\_Mf\_liverA, Unigene7510\_Mf\_liverA, Unigene24252\_Mf\_liverA, CL4757.Contig1\_Mf\_liverA, CL5586.Contig1\_Mf\_liverA, Unigene4557\_Mf\_liverA, Unigene21255\_Mf\_liverA, Unigene34341\_Mf\_liverA, Unigene35431\_Mf\_liverA, Unigene15703\_Mf\_liverA, Unigene2\_Mf\_liverA, Unigene35816\_Mf\_liverA, Unigene28331\_Mf\_liverA, CL33.Contig3\_Mf\_liverA, Unigene13266\_Mf\_liverA, NM\_153193, Unigene5138\_Mf\_liverA, Unigene21857\_Mf\_liverA, Unigene8054\_Mf\_liverA, Unigene23869\_Mf\_liverA, Unigene5598\_Mf\_liverA, NM\_053214, Unigene36417\_Mf\_liverA, CL2855.Contig2\_Mf\_liverA, NM\_177093, Unigene44317\_Mf\_liverA, Unigene25047\_Mf\_liverA, Unigene14907\_Mf\_liverA, Unigene7476\_Mf\_liverA, Unigene1479\_Mf\_liverA, Unigene4922\_Mf\_liverA, Unigene25976\_Mf\_liverA, Unigene14637\_Mf\_liverA, Unigene9475\_Mf\_liverA, Unigene37662\_Mf\_liverA, Unigene27895\_Mf\_liverA, Unigene36112\_Mf\_liverA, Unigene36641\_Mf\_liverA, Unigene9990\_Mf\_liverA, Unigene139\_Mf\_liverA, Unigene995\_Mf\_liverA, Unigene34123\_Mf\_liverA, CL3055.Contig2\_Mf\_liverA, Unigene37575\_Mf\_liverA, Unigene43515\_Mf\_liverA, Unigene25398\_Mf\_liverA, Unigene27547\_Mf\_liverA, Unigene11097\_Mf\_liverA, NM\_134156, Unigene37389\_Mf\_liverA, Unigene32332\_Mf\_liverA, CL5254.Contig1\_Mf\_liverA, Unigene29253\_Mf\_liverA, Unigene34010\_Mf\_liverA, Unigene152\_Mf\_liverA, Unigene5456\_Mf\_liverA, NM\_009087, Unigene37076\_Mf\_liverA, Unigene2678\_Mf\_liverA, Unigene1280\_Mf\_liverA, Unigene21256\_Mf\_liverA, Unigene30288\_Mf\_liverA, CL5576.Contig1\_Mf\_liverA, Unigene2746\_Mf\_liverA, Unigene5236\_Mf\_liverA, Unigene28021\_Mf\_liverA, Unigene37433\_Mf\_liverA, Unigene5472\_Mf\_liverA, Unigene26515\_Mf\_liverA, CL425.Contig1\_Mf\_liverA, CL2797.Contig2\_Mf\_liverA, Unigene14916\_Mf\_liverA, Unigene30808\_Mf\_liverA, Unigene5693\_Mf\_liverA, Unigene50250\_Mf\_liverA, Unigene19821\_Mf\_liverA, CL4057.Contig1\_Mf\_liverA, Unigene35958\_Mf\_liverA, Unigene1440\_Mf\_liverA, Unigene43037\_Mf\_liverA, Unigene29876\_Mf\_liverA, Unigene13525\_Mf\_liverA, Unigene25080\_Mf\_liverA, CL1988.Contig3\_Mf\_liverA, Unigene1129\_Mf\_liverA, CL3055.Contig1\_Mf\_liverA, Unigene29885\_Mf\_liverA, NM\_009609, CL1810.Contig1\_Mf\_liverA, CL4033.Contig1\_Mf\_liverA, Unigene33168\_Mf\_liverA, Unigene27483\_Mf\_liverA, CL3738.Contig1\_Mf\_liverA, Unigene28662\_Mf\_liverA, Unigene4781\_Mf\_liverA, Unigene46615\_Mf\_liverA, Unigene35935\_Mf\_liverA, CL5049.Contig2\_Mf\_liverA, Unigene34446\_Mf\_liverA, CL1493.Contig1\_Mf\_liverA, Unigene13683\_Mf\_liverA, Unigene24801\_Mf\_liverA, Unigene24755\_Mf\_liverA, Unigene9698\_Mf\_liverA, Unigene28499\_Mf\_liverA, CL3685.Contig1\_Mf\_liverA, Unigene36420\_Mf\_liverA, Unigene542\_Mf\_liverA, Unigene24758\_Mf\_liverA, Unigene23271\_Mf\_liverA, Unigene4909\_Mf\_liverA, Unigene34197\_Mf\_liverA, Unigene24471\_Mf\_liverA, NM\_026823, Unigene28527\_Mf\_liverA, Unigene15888\_Mf\_liverA, NM\_010162, Unigene15529\_Mf\_liverA, CL2251.Contig1\_Mf\_liverA, CL4490.Contig2\_Mf\_liverA, Unigene25595\_Mf\_liverA, Unigene14263\_Mf\_liverA, Unigene14212\_Mf\_liverA, Unigene26250\_Mf\_liverA, Unigene48460\_Mf\_liverA, Unigene23185\_Mf\_liverA, Unigene29558\_Mf\_liverA, Unigene35046\_Mf\_liverA, Unigene16671\_Mf\_liverA, Unigene281\_Mf\_liverA, Unigene10313\_Mf\_liverA, Unigene13772\_Mf\_liverA, CL3104.Contig1\_Mf\_liverA, Unigene5687\_Mf\_liverA, Unigene36593\_Mf\_liverA, Unigene5422\_Mf\_liverA, Unigene7968\_Mf\_liverA, Unigene36699\_Mf\_liverA, Unigene36673\_Mf\_liverA, NM\_181517, CL5698.Contig1\_Mf\_liverA, Unigene31392\_Mf\_liverA, Unigene5512\_Mf\_liverA, Unigene42855\_Mf\_liverA, CL591.Contig1\_Mf\_liverA, Unigene4556\_Mf\_liverA, Unigene29399\_Mf\_liverA, NM\_020559, Unigene35884\_Mf\_liverA, Unigene28142\_Mf\_liverA, Unigene38015\_Mf\_liverA, NM\_007896, CL3835.Contig2\_Mf\_liverA, CL1828.Contig1\_Mf\_liverA, NM\_145824, Unigene30356\_Mf\_liverA, Unigene38124\_Mf\_liverA, Unigene29308\_Mf\_liverA, CL523.Contig1\_Mf\_liverA, Unigene29008\_Mf\_liverA, CL1063.Contig1\_Mf\_liverA, CL482.Contig1\_Mf\_liverA, Unigene19049\_Mf\_liverA, Unigene29405\_Mf\_liverA, Unigene32882\_Mf\_liverA, Unigene31263\_Mf\_liverA, CL5358.Contig1\_Mf\_liverA, Unigene7619\_Mf\_liverA, Unigene21466\_Mf\_liverA, Unigene7969\_Mf\_liverA, Unigene30303\_Mf\_liverA, Unigene30878\_Mf\_liverA, Unigene15656\_Mf\_liverA, Unigene34810\_Mf\_liverA, Unigene34234\_Mf\_liverA, CL6038.Contig2\_Mf\_liverA, Unigene5204\_Mf\_liverA, CL5001.Contig2\_Mf\_liverA, Unigene25070\_Mf\_liverA, Unigene34789\_Mf\_liverA, CL4141.Contig1\_Mf\_liverA, Unigene25057\_Mf\_liverA, NM\_145419, Unigene23273\_Mf\_liverA, CL1119.Contig1\_Mf\_liverA, Unigene13658\_Mf\_liverA, Unigene41158\_Mf\_liverA, NM\_175260, Unigene4686\_Mf\_liverA, Unigene25596\_Mf\_liverA, Unigene18117\_Mf\_liverA, CL1493.Contig2\_Mf\_liverA, Unigene3174\_Mf\_liverA, CL2117.Contig1\_Mf\_liverA, Unigene22978\_Mf\_liverA, Unigene25046\_Mf\_liverA, Unigene36988\_Mf\_liverA, Unigene28094\_Mf\_liverA, Unigene5941\_Mf\_liverA, CL2574.Contig1\_Mf\_liverA, Unigene23328\_Mf\_liverA, Unigene2745\_Mf\_liverA, Unigene25462\_Mf\_liverA, Unigene16684\_Mf\_liverA, Unigene1162\_Mf\_liverA, CL787.Contig1\_Mf\_liverA, CL4847.Contig1\_Mf\_liverA, Unigene28822\_Mf\_liverA, Unigene36691\_Mf\_liverA, Unigene38406\_Mf\_liverA, Unigene19297\_Mf\_liverA, CL2326.Contig1\_Mf\_liverA, CL4722.Contig1\_Mf\_liverA, CL848.Contig2\_Mf\_liverA, Unigene23870\_Mf\_liverA, Unigene4768\_Mf\_liverA, Unigene26398\_Mf\_liverA, NM\_008808, CL2131.Contig4\_Mf\_liverA, Unigene34258\_Mf\_liverA, CL3198.Contig1\_Mf\_liverA, Unigene5632\_Mf\_liverA, Unigene25662\_Mf\_liverA, CL479.Contig1\_Mf\_liverA, Unigene37460\_Mf\_liverA, Unigene31517\_Mf\_liverA, NM\_008538, NM\_134059, NM\_011305, CL3816.Contig1\_Mf\_liverA, CL2722.Contig1\_Mf\_liverA, Unigene27438\_Mf\_liverA, CL5631.Contig1\_Mf\_liverA, Unigene14896\_Mf\_liverA, CL3803.Contig2\_Mf\_liverA, Unigene19885\_Mf\_liverA, Unigene36510\_Mf\_liverA, Unigene26873\_Mf\_liverA, NM\_009091, Unigene27260\_Mf\_liverA, CL3268.Contig1\_Mf\_liverA, Unigene10820\_Mf\_liverA, Unigene36845\_Mf\_liverA, Unigene21317\_Mf\_liverA, NM\_010227, Unigene30493\_Mf\_liverA, Unigene37729\_Mf\_liverA, Unigene8132\_Mf\_liverA, Unigene29940\_Mf\_liverA, CL795.Contig1\_Mf\_liverA, Unigene42812\_Mf\_liverA, Unigene27082\_Mf\_liverA, Unigene5712\_Mf\_liverA, Unigene5325\_Mf\_liverA, Unigene1205\_Mf\_liverA, CL2962.Contig1\_Mf\_liverA, CL442.Contig2\_Mf\_liverA, Unigene26214\_Mf\_liverA, CL2855.Contig1\_Mf\_liverA, NM\_011132, Unigene13233\_Mf\_liverA, Unigene5648\_Mf\_liverA, CL1537.Contig1\_Mf\_liverA, NM\_010481, NM\_009128, CL507.Contig1\_Mf\_liverA, NM\_175472, Unigene15318\_Mf\_liverA, Unigene7412\_Mf\_liverA, NM\_009178, CL2240.Contig1\_Mf\_liverA, Unigene13414\_Mf\_liverA, NM\_008293, Unigene22980\_Mf\_liverA, Unigene38339\_Mf\_liverA, Unigene38208\_Mf\_liverA, CL1125.Contig1\_Mf\_liverA, CL4162.Contig1\_Mf\_liverA, Unigene35237\_Mf\_liverA, CL5993.Contig3\_Mf\_liverA, NM\_010579, Unigene673\_Mf\_liverA, Unigene7350\_Mf\_liverA, Unigene4703\_Mf\_liverA, Unigene5165\_Mf\_liverA, Unigene23013\_Mf\_liverA, Unigene30707\_Mf\_liverA, CL4583.Contig2\_Mf\_liverA, Unigene36414\_Mf\_liverA, NM\_133838, Unigene34219\_Mf\_liverA, Unigene33880\_Mf\_liverA, Unigene36328\_Mf\_liverA, CL3002.Contig1\_Mf\_liverA, CL1222.Contig1\_Mf\_liverA, Unigene15058\_Mf\_liverA, Unigene35491\_Mf\_liverA, NM\_029872, Unigene36710\_Mf\_liverA, Unigene5906\_Mf\_liverA, Unigene5940\_Mf\_liverA, NM\_007393, Unigene34035\_Mf\_liverA, Unigene28731\_Mf\_liverA, CL5688.Contig1\_Mf\_liverA, NM\_033444, CL2791.Contig1\_Mf\_liverA, Unigene24085\_Mf\_liverA, Unigene10135\_Mf\_liverA, CL4106.Contig1\_Mf\_liverA, Unigene22330\_Mf\_liverA, NM\_009883, Unigene5954\_Mf\_liverA, CL4127.Contig1\_Mf\_liverA, NM\_009448, CL5307.Contig1\_Mf\_liverA, Unigene31730\_Mf\_liverA, Unigene51055\_Mf\_liverA, CL1190.Contig3\_Mf\_liverA, CL4432.Contig2\_Mf\_liverA, Unigene4540\_Mf\_liverA, Unigene24566\_Mf\_liverA, Unigene37310\_Mf\_liverA, Unigene15170\_Mf\_liverA, NM\_011418, Unigene4604\_Mf\_liverA, Unigene560\_Mf\_liverA, Unigene27803\_Mf\_liverA, Unigene30731\_Mf\_liverA, Unigene36418\_Mf\_liverA, Unigene1221\_Mf\_liverA, CL2355.Contig1\_Mf\_liverA, Unigene37153\_Mf\_liverA, Unigene15592\_Mf\_liverA, Unigene5745\_Mf\_liverA, Unigene37711\_Mf\_liverA, Unigene12889\_Mf\_liverA, Unigene93\_Mf\_liverA, Unigene803\_Mf\_liverA, CL4434.Contig1\_Mf\_liverA, Unigene5147\_Mf\_liverA, CL3911.Contig2\_Mf\_liverA, Unigene21359\_Mf\_liverA, Unigene36487\_Mf\_liverA, Unigene4681\_Mf\_liverA, Unigene37474\_Mf\_liverA, CL548.Contig1\_Mf\_liverA, NM\_007392, Unigene16891\_Mf\_liverA, Unigene5886\_Mf\_liverA, Unigene15493\_Mf\_liverA |
| sheet-forming collagen | Unigene34123\_Mf\_liverA, Unigene34124\_Mf\_liverA |
| membrane part | Unigene24506\_Mf\_liverA, NM\_176843, Unigene34656\_Mf\_liverA, NM\_009898, NM\_001025208, NM\_009592, Unigene24111\_Mf\_liverA, Unigene28186\_Mf\_liverA, NM\_018815, NM\_010378, Unigene29424\_Mf\_liverA, Unigene34218\_Mf\_liverA, Unigene26053\_Mf\_liverA, Unigene33627\_Mf\_liverA, Unigene25090\_Mf\_liverA, CL238.Contig1\_Mf\_liverA, CL5560.Contig1\_Mf\_liverA, Unigene22575\_Mf\_liverA, Unigene39886\_Mf\_liverA, Unigene38689\_Mf\_liverA, NM\_010877, Unigene38919\_Mf\_liverA, Unigene1137\_Mf\_liverA, NR\_004446, CL5586.Contig1\_Mf\_liverA, Unigene34341\_Mf\_liverA, Unigene15703\_Mf\_liverA, Unigene2\_Mf\_liverA, Unigene35816\_Mf\_liverA, Unigene28331\_Mf\_liverA, NM\_010233, CL593.Contig2\_Mf\_liverA, Unigene13266\_Mf\_liverA, NM\_153193, Unigene5138\_Mf\_liverA, Unigene37150\_Mf\_liverA, Unigene5330\_Mf\_liverA, Unigene21857\_Mf\_liverA, Unigene34143\_Mf\_liverA, Unigene27218\_Mf\_liverA, Unigene8054\_Mf\_liverA, Unigene23869\_Mf\_liverA, Unigene5552\_Mf\_liverA, Unigene5598\_Mf\_liverA, Unigene13363\_Mf\_liverA, Unigene26065\_Mf\_liverA, Unigene13230\_Mf\_liverA, CL2855.Contig2\_Mf\_liverA, Unigene25091\_Mf\_liverA, Unigene18125\_Mf\_liverA, Unigene28226\_Mf\_liverA, Unigene13097\_Mf\_liverA, Unigene37278\_Mf\_liverA, Unigene2919\_Mf\_liverA, Unigene21561\_Mf\_liverA, Unigene1479\_Mf\_liverA, Unigene7519\_Mf\_liverA, Unigene4922\_Mf\_liverA, Unigene13913\_Mf\_liverA, NM\_153505, Unigene9475\_Mf\_liverA, Unigene36112\_Mf\_liverA, NM\_015803, Unigene941\_Mf\_liverA, Unigene9990\_Mf\_liverA, Unigene30459\_Mf\_liverA, Unigene139\_Mf\_liverA, NM\_153055, Unigene26941\_Mf\_liverA, Unigene35838\_Mf\_liverA, NM\_172509, Unigene995\_Mf\_liverA, Unigene37420\_Mf\_liverA, NM\_001143689, Unigene19735\_Mf\_liverA, CL3055.Contig2\_Mf\_liverA, Unigene37575\_Mf\_liverA, Unigene27249\_Mf\_liverA, Unigene43515\_Mf\_liverA, Unigene16271\_Mf\_liverA, Unigene25398\_Mf\_liverA, Unigene27547\_Mf\_liverA, Unigene21842\_Mf\_liverA, NM\_134156, Unigene32889\_Mf\_liverA, Unigene4720\_Mf\_liverA, Unigene37389\_Mf\_liverA, Unigene14070\_Mf\_liverA, Unigene32332\_Mf\_liverA, Unigene33271\_Mf\_liverA, Unigene29960\_Mf\_liverA, CL4156.Contig1\_Mf\_liverA, Unigene27248\_Mf\_liverA, Unigene29253\_Mf\_liverA, Unigene31852\_Mf\_liverA, NM\_009713, Unigene2678\_Mf\_liverA, Unigene1280\_Mf\_liverA, CL1738.Contig1\_Mf\_liverA, Unigene479\_Mf\_liverA, Unigene674\_Mf\_liverA, Unigene15184\_Mf\_liverA, Unigene5169\_Mf\_liverA, Unigene28021\_Mf\_liverA, NM\_008365, NM\_011503, Unigene40610\_Mf\_liverA, NM\_001159724, Unigene2162\_Mf\_liverA, CL425.Contig1\_Mf\_liverA, CL3150.Contig1\_Mf\_liverA, CL2797.Contig2\_Mf\_liverA, Unigene4697\_Mf\_liverA, Unigene37243\_Mf\_liverA, Unigene27026\_Mf\_liverA, Unigene30587\_Mf\_liverA, Unigene39403\_Mf\_liverA, Unigene30039\_Mf\_liverA, Unigene29231\_Mf\_liverA, Unigene5693\_Mf\_liverA, Unigene50250\_Mf\_liverA, CL4057.Contig1\_Mf\_liverA, Unigene35958\_Mf\_liverA, Unigene1440\_Mf\_liverA, Unigene13525\_Mf\_liverA, Unigene25080\_Mf\_liverA, Unigene4938\_Mf\_liverA, CL1988.Contig3\_Mf\_liverA, CL3055.Contig1\_Mf\_liverA, Unigene5758\_Mf\_liverA, CL1810.Contig1\_Mf\_liverA, CL4033.Contig1\_Mf\_liverA, Unigene12843\_Mf\_liverA, Unigene27483\_Mf\_liverA, Unigene4781\_Mf\_liverA, Unigene46615\_Mf\_liverA, CL3519.Contig1\_Mf\_liverA, Unigene29426\_Mf\_liverA, Unigene13462\_Mf\_liverA, Unigene28025\_Mf\_liverA, Unigene34446\_Mf\_liverA, Unigene11686\_Mf\_liverA, Unigene28499\_Mf\_liverA, Unigene30585\_Mf\_liverA, CL5764.Contig1\_Mf\_liverA, Unigene38593\_Mf\_liverA, Unigene39231\_Mf\_liverA, Unigene542\_Mf\_liverA, Unigene41226\_Mf\_liverA, Unigene4909\_Mf\_liverA, Unigene34197\_Mf\_liverA, Unigene24471\_Mf\_liverA, Unigene25092\_Mf\_liverA, Unigene15888\_Mf\_liverA, Unigene29938\_Mf\_liverA, NM\_010162, CL725.Contig1\_Mf\_liverA, Unigene30947\_Mf\_liverA, CL4490.Contig2\_Mf\_liverA, Unigene33514\_Mf\_liverA, Unigene35090\_Mf\_liverA, Unigene39011\_Mf\_liverA, Unigene14330\_Mf\_liverA, Unigene23185\_Mf\_liverA, Unigene35046\_Mf\_liverA, Unigene37245\_Mf\_liverA, Unigene16671\_Mf\_liverA, Unigene281\_Mf\_liverA, Unigene13772\_Mf\_liverA, CL3104.Contig1\_Mf\_liverA, Unigene26580\_Mf\_liverA, Unigene29302\_Mf\_liverA, Unigene36593\_Mf\_liverA, Unigene26336\_Mf\_liverA, Unigene5422\_Mf\_liverA, Unigene36699\_Mf\_liverA, Unigene36667\_Mf\_liverA, Unigene18340\_Mf\_liverA, NM\_029219, Unigene4983\_Mf\_liverA, CL5698.Contig1\_Mf\_liverA, Unigene5512\_Mf\_liverA, CL373.Contig7\_Mf\_liverA, Unigene37189\_Mf\_liverA, CL591.Contig1\_Mf\_liverA, Unigene26055\_Mf\_liverA, NM\_007896, CL3835.Contig2\_Mf\_liverA, Unigene38831\_Mf\_liverA, Unigene42975\_Mf\_liverA, CL1828.Contig1\_Mf\_liverA, CL1362.Contig1\_Mf\_liverA, CL523.Contig1\_Mf\_liverA, Unigene29008\_Mf\_liverA, Unigene5194\_Mf\_liverA, CL482.Contig1\_Mf\_liverA, Unigene27593\_Mf\_liverA, Unigene22433\_Mf\_liverA, Unigene48792\_Mf\_liverA, Unigene14816\_Mf\_liverA, Unigene37698\_Mf\_liverA, Unigene30303\_Mf\_liverA, Unigene30878\_Mf\_liverA, Unigene15656\_Mf\_liverA, Unigene34810\_Mf\_liverA, NM\_172671, Unigene34234\_Mf\_liverA, Unigene24344\_Mf\_liverA, Unigene36889\_Mf\_liverA, Unigene5204\_Mf\_liverA, Unigene37268\_Mf\_liverA, CL1256.Contig1\_Mf\_liverA, Unigene24112\_Mf\_liverA, Unigene34789\_Mf\_liverA, Unigene25057\_Mf\_liverA, Unigene38110\_Mf\_liverA, Unigene31333\_Mf\_liverA, Unigene4686\_Mf\_liverA, Unigene4630\_Mf\_liverA, Unigene11544\_Mf\_liverA, NM\_010763, CL3575.Contig1\_Mf\_liverA, CL2117.Contig1\_Mf\_liverA, Unigene22432\_Mf\_liverA, Unigene26533\_Mf\_liverA, Unigene28094\_Mf\_liverA, Unigene40824\_Mf\_liverA, CL2574.Contig1\_Mf\_liverA, Unigene25462\_Mf\_liverA, Unigene16684\_Mf\_liverA, CL787.Contig1\_Mf\_liverA, CL4847.Contig1\_Mf\_liverA, Unigene20128\_Mf\_liverA, Unigene47203\_Mf\_liverA, CL3196.Contig2\_Mf\_liverA, Unigene38406\_Mf\_liverA, Unigene23870\_Mf\_liverA, Unigene13143\_Mf\_liverA, Unigene5632\_Mf\_liverA, CL479.Contig1\_Mf\_liverA, Unigene34746\_Mf\_liverA, Unigene17213\_Mf\_liverA, Unigene24503\_Mf\_liverA, Unigene6340\_Mf\_liverA, Unigene12200\_Mf\_liverA, CL3816.Contig1\_Mf\_liverA, CL2722.Contig1\_Mf\_liverA, NM\_009320, CL5631.Contig1\_Mf\_liverA, Unigene20116\_Mf\_liverA, Unigene25852\_Mf\_liverA, Unigene30584\_Mf\_liverA, Unigene24284\_Mf\_liverA, Unigene431\_Mf\_liverA, Unigene36510\_Mf\_liverA, Unigene27260\_Mf\_liverA, Unigene36845\_Mf\_liverA, NM\_010391, CL795.Contig1\_Mf\_liverA, NM\_019717, Unigene27082\_Mf\_liverA, Unigene5712\_Mf\_liverA, CL5039.Contig2\_Mf\_liverA, Unigene1205\_Mf\_liverA, Unigene1088\_Mf\_liverA, Unigene26214\_Mf\_liverA, NM\_010380, CL2855.Contig1\_Mf\_liverA, Unigene33760\_Mf\_liverA, NM\_001025388, Unigene13233\_Mf\_liverA, Unigene17569\_Mf\_liverA, Unigene5648\_Mf\_liverA, Unigene40924\_Mf\_liverA, CL4086.Contig1\_Mf\_liverA, NM\_009128, CL507.Contig1\_Mf\_liverA, Unigene23082\_Mf\_liverA, Unigene7889\_Mf\_liverA, Unigene15275\_Mf\_liverA, Unigene15318\_Mf\_liverA, NM\_009178, CL2240.Contig1\_Mf\_liverA, Unigene13414\_Mf\_liverA, Unigene39385\_Mf\_liverA, Unigene29823\_Mf\_liverA, NM\_008293, Unigene15474\_Mf\_liverA, Unigene23448\_Mf\_liverA, Unigene15982\_Mf\_liverA, Unigene38208\_Mf\_liverA, Unigene37526\_Mf\_liverA, Unigene35237\_Mf\_liverA, CL5993.Contig3\_Mf\_liverA, Unigene37259\_Mf\_liverA, Unigene7350\_Mf\_liverA, Unigene4703\_Mf\_liverA, Unigene23013\_Mf\_liverA, Unigene30707\_Mf\_liverA, CL4583.Contig2\_Mf\_liverA, Unigene36543\_Mf\_liverA, NM\_011352, Unigene36328\_Mf\_liverA, CL3002.Contig1\_Mf\_liverA, Unigene6895\_Mf\_liverA, CL2327.Contig1\_Mf\_liverA, Unigene35491\_Mf\_liverA, Unigene37139\_Mf\_liverA, Unigene25994\_Mf\_liverA, NM\_010393, Unigene5906\_Mf\_liverA, CL1662.Contig1\_Mf\_liverA, Unigene28731\_Mf\_liverA, Unigene4597\_Mf\_liverA, NM\_033444, Unigene24085\_Mf\_liverA, Unigene10135\_Mf\_liverA, NM\_007986, CL4106.Contig1\_Mf\_liverA, CL4127.Contig1\_Mf\_liverA, Unigene23255\_Mf\_liverA, Unigene21337\_Mf\_liverA, CL5307.Contig1\_Mf\_liverA, Unigene51055\_Mf\_liverA, Unigene28873\_Mf\_liverA, Unigene8740\_Mf\_liverA, Unigene9406\_Mf\_liverA, Unigene39749\_Mf\_liverA, Unigene28930\_Mf\_liverA, Unigene25052\_Mf\_liverA, Unigene21843\_Mf\_liverA, Unigene14240\_Mf\_liverA, Unigene677\_Mf\_liverA, Unigene21562\_Mf\_liverA, NM\_023580, Unigene17632\_Mf\_liverA, Unigene5745\_Mf\_liverA, Unigene21336\_Mf\_liverA, Unigene12889\_Mf\_liverA, Unigene30892\_Mf\_liverA, Unigene35417\_Mf\_liverA, CL4434.Contig1\_Mf\_liverA, Unigene34866\_Mf\_liverA, Unigene14765\_Mf\_liverA, Unigene4681\_Mf\_liverA, CL548.Contig1\_Mf\_liverA, Unigene31080\_Mf\_liverA, Unigene979\_Mf\_liverA, Unigene7733\_Mf\_liverA, Unigene37819\_Mf\_liverA, Unigene14284\_Mf\_liverA, Unigene26474\_Mf\_liverA, Unigene5886\_Mf\_liverA |
| I band | Unigene9475\_Mf\_liverA, CL4847.Contig1\_Mf\_liverA, Unigene2678\_Mf\_liverA, Unigene4557\_Mf\_liverA, NM\_134156, Unigene26250\_Mf\_liverA, Unigene24755\_Mf\_liverA, Unigene4556\_Mf\_liverA, CL2251.Contig1\_Mf\_liverA |
| nuclear lamina | Unigene38406\_Mf\_liverA, CL4162.Contig1\_Mf\_liverA |
| cell-cell junction | CL5586.Contig1\_Mf\_liverA, CL507.Contig1\_Mf\_liverA, NM\_134156, Unigene29938\_Mf\_liverA, Unigene29885\_Mf\_liverA, NM\_001159724, Unigene37278\_Mf\_liverA, CL4033.Contig1\_Mf\_liverA, Unigene23870\_Mf\_liverA, CL4434.Contig1\_Mf\_liverA, Unigene27082\_Mf\_liverA, Unigene14637\_Mf\_liverA, Unigene14765\_Mf\_liverA, CL2117.Contig1\_Mf\_liverA, CL1738.Contig1\_Mf\_liverA, Unigene39011\_Mf\_liverA, Unigene26214\_Mf\_liverA, Unigene7733\_Mf\_liverA, Unigene5693\_Mf\_liverA, Unigene7350\_Mf\_liverA, Unigene2746\_Mf\_liverA, Unigene2745\_Mf\_liverA, Unigene39231\_Mf\_liverA, Unigene25052\_Mf\_liverA, Unigene23869\_Mf\_liverA |
| caveola | CL725.Contig1\_Mf\_liverA, Unigene35237\_Mf\_liverA, CL5631.Contig1\_Mf\_liverA, Unigene27260\_Mf\_liverA, Unigene12889\_Mf\_liverA, Unigene28331\_Mf\_liverA |
| integral to endoplasmic reticulum membrane | CL2855.Contig1\_Mf\_liverA, Unigene1479\_Mf\_liverA, Unigene15888\_Mf\_liverA, Unigene10135\_Mf\_liverA, CL2855.Contig2\_Mf\_liverA, CL5560.Contig1\_Mf\_liverA |
| nuclear ubiquitin ligase complex | Unigene5248\_Mf\_liverA, Unigene48460\_Mf\_liverA, Unigene29334\_Mf\_liverA, Unigene5954\_Mf\_liverA |
| interstitial matrix | CL376.Contig1\_Mf\_liverA, Unigene31492\_Mf\_liverA |
| integral to lumenal side of endoplasmic reticulum membrane | CL2855.Contig1\_Mf\_liverA, CL2855.Contig2\_Mf\_liverA |
| germ cell nucleus | CL2326.Contig1\_Mf\_liverA, NM\_008538, Unigene4768\_Mf\_liverA |
| intrinsic to endoplasmic reticulum membrane | Unigene5598\_Mf\_liverA, NM\_009128, Unigene15888\_Mf\_liverA, NM\_010162, CL2855.Contig2\_Mf\_liverA, CL5560.Contig1\_Mf\_liverA, CL2855.Contig1\_Mf\_liverA, Unigene1479\_Mf\_liverA, Unigene10135\_Mf\_liverA |
| lamellipodium membrane | Unigene31852\_Mf\_liverA, CL795.Contig1\_Mf\_liverA |
| fibril | Unigene12230\_Mf\_liverA, Unigene18510\_Mf\_liverA |
| nuclear periphery | CL4162.Contig1\_Mf\_liverA, CL1537.Contig1\_Mf\_liverA, CL4847.Contig1\_Mf\_liverA, Unigene37076\_Mf\_liverA, NM\_009883, Unigene29885\_Mf\_liverA, Unigene38406\_Mf\_liverA, CL795.Contig1\_Mf\_liverA, Unigene5165\_Mf\_liverA, CL4757.Contig1\_Mf\_liverA |
| nucleus | NM\_176843, Unigene28186\_Mf\_liverA, NM\_018815, Unigene36622\_Mf\_liverA, Unigene36851\_Mf\_liverA, Unigene14665\_Mf\_liverA, NM\_080638, Unigene7936\_Mf\_liverA, Unigene29424\_Mf\_liverA, Unigene34218\_Mf\_liverA, CL4105.Contig1\_Mf\_liverA, CL4577.Contig1\_Mf\_liverA, Unigene33138\_Mf\_liverA, CL2423.Contig1\_Mf\_liverA, Unigene5248\_Mf\_liverA, Unigene39886\_Mf\_liverA, Unigene26422\_Mf\_liverA, Unigene29334\_Mf\_liverA, Unigene15064\_Mf\_liverA, NM\_010877, Unigene38919\_Mf\_liverA, CL4757.Contig1\_Mf\_liverA, NM\_013866, Unigene21255\_Mf\_liverA, Unigene30142\_Mf\_liverA, Unigene35431\_Mf\_liverA, Unigene15703\_Mf\_liverA, Unigene35816\_Mf\_liverA, Unigene37084\_Mf\_liverA, Unigene33054\_Mf\_liverA, Unigene5138\_Mf\_liverA, Unigene15205\_Mf\_liverA, Unigene779\_Mf\_liverA, Unigene14050\_Mf\_liverA, Unigene5330\_Mf\_liverA, Unigene653\_Mf\_liverA, Unigene23869\_Mf\_liverA, CL2384.Contig1\_Mf\_liverA, Unigene36417\_Mf\_liverA, NM\_177093, Unigene14907\_Mf\_liverA, Unigene7476\_Mf\_liverA, Unigene1479\_Mf\_liverA, CL4332.Contig1\_Mf\_liverA, Unigene25976\_Mf\_liverA, Unigene44882\_Mf\_liverA, Unigene9990\_Mf\_liverA, Unigene13945\_Mf\_liverA, Unigene4944\_Mf\_liverA, Unigene23158\_Mf\_liverA, Unigene37575\_Mf\_liverA, Unigene43515\_Mf\_liverA, Unigene5180\_Mf\_liverA, NM\_134156, Unigene4720\_Mf\_liverA, CL5254.Contig1\_Mf\_liverA, Unigene29253\_Mf\_liverA, Unigene152\_Mf\_liverA, Unigene5456\_Mf\_liverA, NM\_009087, Unigene37076\_Mf\_liverA, Unigene21256\_Mf\_liverA, Unigene30288\_Mf\_liverA, CL3339.Contig1\_Mf\_liverA, Unigene2746\_Mf\_liverA, Unigene45904\_Mf\_liverA, Unigene37433\_Mf\_liverA, CL1442.Contig1\_Mf\_liverA, Unigene26515\_Mf\_liverA, Unigene40610\_Mf\_liverA, CL186.Contig3\_Mf\_liverA, Unigene14916\_Mf\_liverA, Unigene35858\_Mf\_liverA, Unigene30808\_Mf\_liverA, Unigene29231\_Mf\_liverA, Unigene5693\_Mf\_liverA, Unigene50250\_Mf\_liverA, Unigene19821\_Mf\_liverA, CL4057.Contig1\_Mf\_liverA, Unigene35958\_Mf\_liverA, Unigene29876\_Mf\_liverA, CL3800.Contig1\_Mf\_liverA, Unigene13525\_Mf\_liverA, Unigene25080\_Mf\_liverA, Unigene28687\_Mf\_liverA, Unigene24395\_Mf\_liverA, Unigene29885\_Mf\_liverA, Unigene1129\_Mf\_liverA, NM\_009609, CL1810.Contig1\_Mf\_liverA, Unigene33168\_Mf\_liverA, Unigene27483\_Mf\_liverA, CL3738.Contig1\_Mf\_liverA, CL2439.Contig1\_Mf\_liverA, Unigene35935\_Mf\_liverA, Unigene34446\_Mf\_liverA, Unigene13683\_Mf\_liverA, Unigene24801\_Mf\_liverA, Unigene9698\_Mf\_liverA, Unigene36420\_Mf\_liverA, Unigene24758\_Mf\_liverA, Unigene23271\_Mf\_liverA, Unigene34197\_Mf\_liverA, Unigene24471\_Mf\_liverA, Unigene28527\_Mf\_liverA, CL2625.Contig2\_Mf\_liverA, CL1165.Contig2\_Mf\_liverA, Unigene14212\_Mf\_liverA, CL1165.Contig4\_Mf\_liverA, Unigene39011\_Mf\_liverA, CL4669.Contig1\_Mf\_liverA, Unigene26250\_Mf\_liverA, Unigene48460\_Mf\_liverA, Unigene16671\_Mf\_liverA, Unigene10313\_Mf\_liverA, Unigene13535\_Mf\_liverA, Unigene29985\_Mf\_liverA, Unigene5037\_Mf\_liverA, Unigene7968\_Mf\_liverA, Unigene36673\_Mf\_liverA, NM\_181517, Unigene4983\_Mf\_liverA, Unigene31392\_Mf\_liverA, Unigene42855\_Mf\_liverA, Unigene9466\_Mf\_liverA, Unigene29399\_Mf\_liverA, CL2520.Contig1\_Mf\_liverA, Unigene35884\_Mf\_liverA, Unigene28142\_Mf\_liverA, Unigene38015\_Mf\_liverA, CL3835.Contig2\_Mf\_liverA, Unigene24713\_Mf\_liverA, Unigene30356\_Mf\_liverA, Unigene38124\_Mf\_liverA, Unigene46870\_Mf\_liverA, CL1362.Contig1\_Mf\_liverA, Unigene29008\_Mf\_liverA, CL1063.Contig1\_Mf\_liverA, CL482.Contig1\_Mf\_liverA, Unigene32882\_Mf\_liverA, Unigene31263\_Mf\_liverA, Unigene21263\_Mf\_liverA, Unigene7619\_Mf\_liverA, Unigene7969\_Mf\_liverA, Unigene30303\_Mf\_liverA, Unigene40289\_Mf\_liverA, Unigene21013\_Mf\_liverA, CL5001.Contig2\_Mf\_liverA, Unigene13307\_Mf\_liverA, Unigene37904\_Mf\_liverA, Unigene25070\_Mf\_liverA, Unigene25057\_Mf\_liverA, CL4141.Contig1\_Mf\_liverA, NM\_145419, Unigene23273\_Mf\_liverA, CL1119.Contig1\_Mf\_liverA, Unigene13658\_Mf\_liverA, Unigene26194\_Mf\_liverA, Unigene41158\_Mf\_liverA, CL3778.Contig2\_Mf\_liverA, Unigene18117\_Mf\_liverA, Unigene3174\_Mf\_liverA, CL2117.Contig1\_Mf\_liverA, Unigene36988\_Mf\_liverA, CL3565.Contig1\_Mf\_liverA, Unigene2745\_Mf\_liverA, Unigene38065\_Mf\_liverA, Unigene16684\_Mf\_liverA, Unigene1162\_Mf\_liverA, CL4847.Contig1\_Mf\_liverA, Unigene38406\_Mf\_liverA, Unigene19297\_Mf\_liverA, CL2326.Contig1\_Mf\_liverA, CL4722.Contig1\_Mf\_liverA, Unigene23870\_Mf\_liverA, Unigene4768\_Mf\_liverA, CL2131.Contig4\_Mf\_liverA, Unigene34258\_Mf\_liverA, Unigene10774\_Mf\_liverA, Unigene36628\_Mf\_liverA, Unigene31517\_Mf\_liverA, NM\_008538, Unigene14582\_Mf\_liverA, NM\_134059, NM\_011305, Unigene35859\_Mf\_liverA, Unigene27438\_Mf\_liverA, CL5631.Contig1\_Mf\_liverA, Unigene36631\_Mf\_liverA, Unigene19885\_Mf\_liverA, Unigene26873\_Mf\_liverA, NM\_009091, CL3268.Contig1\_Mf\_liverA, Unigene27260\_Mf\_liverA, Unigene10820\_Mf\_liverA, Unigene8132\_Mf\_liverA, CL795.Contig1\_Mf\_liverA, Unigene42812\_Mf\_liverA, Unigene37178\_Mf\_liverA, Unigene5509\_Mf\_liverA, Unigene584\_Mf\_liverA, Unigene13841\_Mf\_liverA, Unigene23055\_Mf\_liverA, CL2962.Contig1\_Mf\_liverA, Unigene31206\_Mf\_liverA, NM\_011132, Unigene32412\_Mf\_liverA, CL1537.Contig1\_Mf\_liverA, CL507.Contig1\_Mf\_liverA, NM\_175472, Unigene5360\_Mf\_liverA, Unigene15318\_Mf\_liverA, Unigene13414\_Mf\_liverA, Unigene38339\_Mf\_liverA, CL4162.Contig1\_Mf\_liverA, Unigene35237\_Mf\_liverA, Unigene29510\_Mf\_liverA, NM\_010579, Unigene37259\_Mf\_liverA, Unigene12907\_Mf\_liverA, Unigene673\_Mf\_liverA, Unigene7350\_Mf\_liverA, Unigene15588\_Mf\_liverA, Unigene5165\_Mf\_liverA, Unigene23013\_Mf\_liverA, Unigene30707\_Mf\_liverA, Unigene36414\_Mf\_liverA, Unigene5175\_Mf\_liverA, Unigene34219\_Mf\_liverA, Unigene33880\_Mf\_liverA, Unigene7674\_Mf\_liverA, CL3002.Contig1\_Mf\_liverA, Unigene15058\_Mf\_liverA, CL1222.Contig1\_Mf\_liverA, Unigene35491\_Mf\_liverA, NM\_029872, Unigene36710\_Mf\_liverA, NM\_007393, Unigene34035\_Mf\_liverA, CL5688.Contig1\_Mf\_liverA, NM\_033444, Unigene1212\_Mf\_liverA, CL2791.Contig1\_Mf\_liverA, CL4106.Contig1\_Mf\_liverA, NM\_009883, Unigene5954\_Mf\_liverA, Unigene18796\_Mf\_liverA, Unigene51055\_Mf\_liverA, CL1190.Contig3\_Mf\_liverA, CL4432.Contig2\_Mf\_liverA, Unigene4540\_Mf\_liverA, Unigene24566\_Mf\_liverA, Unigene37310\_Mf\_liverA, NM\_011418, Unigene4604\_Mf\_liverA, Unigene9406\_Mf\_liverA, Unigene24477\_Mf\_liverA, Unigene30731\_Mf\_liverA, Unigene15026\_Mf\_liverA, Unigene36418\_Mf\_liverA, Unigene39875\_Mf\_liverA, CL2355.Contig1\_Mf\_liverA, Unigene25238\_Mf\_liverA, Unigene37153\_Mf\_liverA, Unigene26309\_Mf\_liverA, Unigene37711\_Mf\_liverA, Unigene12889\_Mf\_liverA, Unigene665\_Mf\_liverA, Unigene41583\_Mf\_liverA, Unigene93\_Mf\_liverA, Unigene35678\_Mf\_liverA, Unigene8560\_Mf\_liverA, Unigene5147\_Mf\_liverA, CL4434.Contig1\_Mf\_liverA, Unigene803\_Mf\_liverA, Unigene34866\_Mf\_liverA, Unigene41419\_Mf\_liverA, Unigene16891\_Mf\_liverA, CL548.Contig1\_Mf\_liverA, Unigene25787\_Mf\_liverA |
| cleavage furrow | CL4434.Contig1\_Mf\_liverA, Unigene5693\_Mf\_liverA, CL507.Contig1\_Mf\_liverA |
| dendritic shaft | Unigene13396\_Mf\_liverA, Unigene31251\_Mf\_liverA, Unigene24547\_Mf\_liverA |
| integral to organelle membrane | CL787.Contig1\_Mf\_liverA, Unigene38208\_Mf\_liverA, Unigene15888\_Mf\_liverA, CL2855.Contig2\_Mf\_liverA, CL5560.Contig1\_Mf\_liverA, Unigene5512\_Mf\_liverA, CL3104.Contig1\_Mf\_liverA, CL2855.Contig1\_Mf\_liverA, Unigene1479\_Mf\_liverA, Unigene10135\_Mf\_liverA |
| trans-Golgi network membrane | CL2855.Contig1\_Mf\_liverA, CL2855.Contig2\_Mf\_liverA |
| cell-substrate adherens junction | Unigene25080\_Mf\_liverA, Unigene31852\_Mf\_liverA, Unigene24284\_Mf\_liverA, Unigene16671\_Mf\_liverA, NM\_134156, Unigene29938\_Mf\_liverA, Unigene29302\_Mf\_liverA, Unigene40824\_Mf\_liverA, Unigene27483\_Mf\_liverA, Unigene5422\_Mf\_liverA, Unigene29424\_Mf\_liverA, Unigene15656\_Mf\_liverA, Unigene28331\_Mf\_liverA |
| cytosolic ribosome | CL1063.Contig1\_Mf\_liverA, Unigene36710\_Mf\_liverA, Unigene5325\_Mf\_liverA, CL5049.Contig2\_Mf\_liverA |
| signalosome | CL507.Contig1\_Mf\_liverA, Unigene35816\_Mf\_liverA |
| main axon | Unigene34866\_Mf\_liverA, Unigene34197\_Mf\_liverA, Unigene4720\_Mf\_liverA |
| smooth endoplasmic reticulum | Unigene34197\_Mf\_liverA, Unigene23185\_Mf\_liverA |
| contractile fiber part | Unigene9475\_Mf\_liverA, CL4847.Contig1\_Mf\_liverA, Unigene37662\_Mf\_liverA, Unigene2678\_Mf\_liverA, Unigene4557\_Mf\_liverA, NM\_134156, Unigene37474\_Mf\_liverA, Unigene26250\_Mf\_liverA, NM\_007392, Unigene24755\_Mf\_liverA, Unigene4556\_Mf\_liverA, CL2251.Contig1\_Mf\_liverA |
| cytosolic small ribosomal subunit | CL1063.Contig1\_Mf\_liverA, CL5049.Contig2\_Mf\_liverA |
| transport vesicle membrane | CL2855.Contig1\_Mf\_liverA, Unigene5204\_Mf\_liverA, Unigene8054\_Mf\_liverA, Unigene9990\_Mf\_liverA, CL2855.Contig2\_Mf\_liverA |
| site of polarized growth | Unigene13396\_Mf\_liverA, Unigene31251\_Mf\_liverA, Unigene14582\_Mf\_liverA, CL2117.Contig1\_Mf\_liverA, CL1352.Contig1\_Mf\_liverA, Unigene21562\_Mf\_liverA, Unigene21561\_Mf\_liverA, Unigene24547\_Mf\_liverA, CL1190.Contig3\_Mf\_liverA, Unigene14896\_Mf\_liverA, NM\_175260 |
| SCF ubiquitin ligase complex | Unigene33054\_Mf\_liverA, Unigene13683\_Mf\_liverA |
| cell-substrate junction | Unigene25080\_Mf\_liverA, Unigene31852\_Mf\_liverA, Unigene24284\_Mf\_liverA, Unigene16671\_Mf\_liverA, NM\_134156, Unigene29938\_Mf\_liverA, Unigene29302\_Mf\_liverA, Unigene40824\_Mf\_liverA, Unigene27483\_Mf\_liverA, Unigene5422\_Mf\_liverA, Unigene29424\_Mf\_liverA, Unigene15656\_Mf\_liverA, Unigene28331\_Mf\_liverA |
| cell projection membrane | Unigene30707\_Mf\_liverA, Unigene25080\_Mf\_liverA, Unigene31852\_Mf\_liverA, Unigene34197\_Mf\_liverA, Unigene16671\_Mf\_liverA, Unigene5138\_Mf\_liverA, Unigene29938\_Mf\_liverA, CL2117.Contig1\_Mf\_liverA, Unigene5693\_Mf\_liverA, Unigene29424\_Mf\_liverA, NM\_007986, CL795.Contig1\_Mf\_liverA |
| phagocytic cup | Unigene36328\_Mf\_liverA, Unigene5138\_Mf\_liverA, Unigene29008\_Mf\_liverA |
| cell projection part | Unigene34197\_Mf\_liverA, Unigene31251\_Mf\_liverA, Unigene13363\_Mf\_liverA, Unigene29938\_Mf\_liverA, CL425.Contig1\_Mf\_liverA, Unigene21561\_Mf\_liverA, CL1190.Contig3\_Mf\_liverA, Unigene29424\_Mf\_liverA, CL479.Contig1\_Mf\_liverA, Unigene14582\_Mf\_liverA, Unigene27895\_Mf\_liverA, Unigene5693\_Mf\_liverA, Unigene14896\_Mf\_liverA, Unigene24252\_Mf\_liverA, Unigene30707\_Mf\_liverA, Unigene25080\_Mf\_liverA, Unigene27547\_Mf\_liverA, Unigene16671\_Mf\_liverA, CL1222.Contig1\_Mf\_liverA, Unigene27260\_Mf\_liverA, Unigene4720\_Mf\_liverA, Unigene5037\_Mf\_liverA, Unigene21562\_Mf\_liverA, Unigene8132\_Mf\_liverA, Unigene24547\_Mf\_liverA, CL795.Contig1\_Mf\_liverA, Unigene31852\_Mf\_liverA, Unigene34866\_Mf\_liverA, Unigene13396\_Mf\_liverA, Unigene5138\_Mf\_liverA, CL2117.Contig1\_Mf\_liverA, CL1352.Contig1\_Mf\_liverA, NM\_007986 |
| sarcoplasmic reticulum membrane | Unigene34656\_Mf\_liverA, Unigene29253\_Mf\_liverA |
| neuron projection terminus | Unigene5037\_Mf\_liverA, Unigene13363\_Mf\_liverA, CL2117.Contig1\_Mf\_liverA |
| lipid particle | Unigene25596\_Mf\_liverA, Unigene5418\_Mf\_liverA, Unigene28331\_Mf\_liverA, Unigene45904\_Mf\_liverA |
| heterotrimeric G-protein complex | Unigene2919\_Mf\_liverA, Unigene34218\_Mf\_liverA |
| non-membrane-bounded organelle | NM\_009450, NM\_007896, Unigene34609\_Mf\_liverA, NM\_009898, Unigene25721\_Mf\_liverA, NM\_145824, Unigene38124\_Mf\_liverA, Unigene29308\_Mf\_liverA, Unigene29424\_Mf\_liverA, Unigene34218\_Mf\_liverA, Unigene29008\_Mf\_liverA, CL1063.Contig1\_Mf\_liverA, Unigene33138\_Mf\_liverA, Unigene32882\_Mf\_liverA, CL5358.Contig1\_Mf\_liverA, CL5560.Contig1\_Mf\_liverA, NM\_011072, Unigene7969\_Mf\_liverA, CL33.Contig4\_Mf\_liverA, Unigene7510\_Mf\_liverA, Unigene15656\_Mf\_liverA, Unigene24252\_Mf\_liverA, CL4757.Contig1\_Mf\_liverA, Unigene4557\_Mf\_liverA, Unigene21255\_Mf\_liverA, Unigene40020\_Mf\_liverA, Unigene25070\_Mf\_liverA, Unigene34789\_Mf\_liverA, Unigene35431\_Mf\_liverA, Unigene25057\_Mf\_liverA, Unigene23273\_Mf\_liverA, Unigene24379\_Mf\_liverA, CL5191.Contig2\_Mf\_liverA, CL33.Contig3\_Mf\_liverA, NM\_175260, Unigene5138\_Mf\_liverA, CL2117.Contig1\_Mf\_liverA, Unigene25046\_Mf\_liverA, Unigene36988\_Mf\_liverA, Unigene21857\_Mf\_liverA, Unigene40824\_Mf\_liverA, Unigene5941\_Mf\_liverA, Unigene13419\_Mf\_liverA, Unigene2745\_Mf\_liverA, NM\_001024205, Unigene16684\_Mf\_liverA, Unigene23869\_Mf\_liverA, CL4847.Contig1\_Mf\_liverA, Unigene28822\_Mf\_liverA, NM\_053214, Unigene36417\_Mf\_liverA, CL2326.Contig1\_Mf\_liverA, Unigene44317\_Mf\_liverA, NM\_177093, Unigene25047\_Mf\_liverA, CL4722.Contig1\_Mf\_liverA, Unigene14907\_Mf\_liverA, CL848.Contig2\_Mf\_liverA, Unigene23870\_Mf\_liverA, Unigene1479\_Mf\_liverA, Unigene4768\_Mf\_liverA, CL2131.Contig4\_Mf\_liverA, Unigene25662\_Mf\_liverA, CL3198.Contig1\_Mf\_liverA, CL479.Contig1\_Mf\_liverA, Unigene14637\_Mf\_liverA, Unigene37460\_Mf\_liverA, Unigene31517\_Mf\_liverA, Unigene9475\_Mf\_liverA, Unigene37662\_Mf\_liverA, NM\_008538, Unigene27895\_Mf\_liverA, NM\_011305, NM\_008610, Unigene27438\_Mf\_liverA, Unigene36631\_Mf\_liverA, Unigene14896\_Mf\_liverA, Unigene37575\_Mf\_liverA, Unigene43515\_Mf\_liverA, CL3803.Contig2\_Mf\_liverA, Unigene27547\_Mf\_liverA, Unigene24284\_Mf\_liverA, Unigene26873\_Mf\_liverA, NM\_009091, Unigene27260\_Mf\_liverA, NM\_134156, CL3268.Contig1\_Mf\_liverA, NM\_010227, Unigene21317\_Mf\_liverA, Unigene37470\_Mf\_liverA, Unigene8132\_Mf\_liverA, CL795.Contig1\_Mf\_liverA, Unigene152\_Mf\_liverA, Unigene27082\_Mf\_liverA, Unigene31852\_Mf\_liverA, NM\_009087, Unigene35169\_Mf\_liverA, Unigene37076\_Mf\_liverA, Unigene2678\_Mf\_liverA, Unigene5325\_Mf\_liverA, Unigene21256\_Mf\_liverA, CL2962.Contig1\_Mf\_liverA, Unigene23055\_Mf\_liverA, CL1352.Contig1\_Mf\_liverA, NM\_033374, Unigene26214\_Mf\_liverA, CL5576.Contig1\_Mf\_liverA, Unigene2746\_Mf\_liverA, NM\_001025388, NM\_009447, Unigene5472\_Mf\_liverA, Unigene26515\_Mf\_liverA, CL1537.Contig1\_Mf\_liverA, CL507.Contig1\_Mf\_liverA, Unigene15318\_Mf\_liverA, Unigene7412\_Mf\_liverA, CL425.Contig1\_Mf\_liverA, Unigene38339\_Mf\_liverA, Unigene14916\_Mf\_liverA, CL4162.Contig1\_Mf\_liverA, Unigene35237\_Mf\_liverA, NM\_010579, Unigene673\_Mf\_liverA, Unigene5693\_Mf\_liverA, Unigene50250\_Mf\_liverA, Unigene13271\_Mf\_liverA, Unigene5165\_Mf\_liverA, CL4057.Contig1\_Mf\_liverA, Unigene35958\_Mf\_liverA, Unigene29876\_Mf\_liverA, Unigene30707\_Mf\_liverA, Unigene36414\_Mf\_liverA, Unigene25080\_Mf\_liverA, Unigene33880\_Mf\_liverA, Unigene34219\_Mf\_liverA, CL1222.Contig1\_Mf\_liverA, Unigene15058\_Mf\_liverA, CL1810.Contig1\_Mf\_liverA, NM\_009609, Unigene35491\_Mf\_liverA, Unigene33168\_Mf\_liverA, Unigene27483\_Mf\_liverA, Unigene36710\_Mf\_liverA, Unigene5940\_Mf\_liverA, NM\_007393, CL5049.Contig2\_Mf\_liverA, Unigene34035\_Mf\_liverA, Unigene34446\_Mf\_liverA, CL5688.Contig1\_Mf\_liverA, Unigene13683\_Mf\_liverA, NM\_053072, Unigene24801\_Mf\_liverA, CL2791.Contig1\_Mf\_liverA, Unigene24755\_Mf\_liverA, Unigene9698\_Mf\_liverA, Unigene28499\_Mf\_liverA, Unigene36420\_Mf\_liverA, CL4106.Contig1\_Mf\_liverA, Unigene24758\_Mf\_liverA, Unigene23271\_Mf\_liverA, Unigene34197\_Mf\_liverA, NM\_026823, Unigene35168\_Mf\_liverA, Unigene8016\_Mf\_liverA, Unigene28527\_Mf\_liverA, NM\_009883, NM\_009448, Unigene1327\_Mf\_liverA, CL2251.Contig1\_Mf\_liverA, CL4432.Contig2\_Mf\_liverA, Unigene4540\_Mf\_liverA, Unigene24566\_Mf\_liverA, NM\_007478, Unigene14263\_Mf\_liverA, NM\_016861, Unigene15170\_Mf\_liverA, Unigene14212\_Mf\_liverA, NM\_011418, Unigene26250\_Mf\_liverA, Unigene24477\_Mf\_liverA, Unigene20432\_Mf\_liverA, Unigene30731\_Mf\_liverA, Unigene48460\_Mf\_liverA, Unigene36418\_Mf\_liverA, NM\_017379, Unigene550\_Mf\_liverA, Unigene16671\_Mf\_liverA, Unigene10313\_Mf\_liverA, Unigene26309\_Mf\_liverA, Unigene29302\_Mf\_liverA, Unigene37711\_Mf\_liverA, Unigene2195\_Mf\_liverA, NM\_146016, Unigene7968\_Mf\_liverA, Unigene93\_Mf\_liverA, Unigene36673\_Mf\_liverA, CL4434.Contig1\_Mf\_liverA, Unigene5147\_Mf\_liverA, Unigene31885\_Mf\_liverA, Unigene30412\_Mf\_liverA, CL5698.Contig1\_Mf\_liverA, Unigene31392\_Mf\_liverA, CL373.Contig7\_Mf\_liverA, Unigene37474\_Mf\_liverA, Unigene42855\_Mf\_liverA, CL548.Contig1\_Mf\_liverA, NM\_007392, CL591.Contig1\_Mf\_liverA, Unigene4556\_Mf\_liverA, Unigene15493\_Mf\_liverA |
| intracellular non-membrane-bounded organelle | NM\_009450, NM\_007896, Unigene34609\_Mf\_liverA, NM\_009898, Unigene25721\_Mf\_liverA, NM\_145824, Unigene38124\_Mf\_liverA, Unigene29308\_Mf\_liverA, Unigene29424\_Mf\_liverA, Unigene34218\_Mf\_liverA, Unigene29008\_Mf\_liverA, CL1063.Contig1\_Mf\_liverA, Unigene33138\_Mf\_liverA, Unigene32882\_Mf\_liverA, CL5358.Contig1\_Mf\_liverA, CL5560.Contig1\_Mf\_liverA, NM\_011072, Unigene7969\_Mf\_liverA, CL33.Contig4\_Mf\_liverA, Unigene7510\_Mf\_liverA, Unigene15656\_Mf\_liverA, Unigene24252\_Mf\_liverA, CL4757.Contig1\_Mf\_liverA, Unigene4557\_Mf\_liverA, Unigene21255\_Mf\_liverA, Unigene40020\_Mf\_liverA, Unigene25070\_Mf\_liverA, Unigene34789\_Mf\_liverA, Unigene35431\_Mf\_liverA, Unigene25057\_Mf\_liverA, Unigene23273\_Mf\_liverA, Unigene24379\_Mf\_liverA, CL5191.Contig2\_Mf\_liverA, CL33.Contig3\_Mf\_liverA, NM\_175260, Unigene5138\_Mf\_liverA, CL2117.Contig1\_Mf\_liverA, Unigene25046\_Mf\_liverA, Unigene36988\_Mf\_liverA, Unigene21857\_Mf\_liverA, Unigene40824\_Mf\_liverA, Unigene5941\_Mf\_liverA, Unigene13419\_Mf\_liverA, Unigene2745\_Mf\_liverA, NM\_001024205, Unigene16684\_Mf\_liverA, Unigene23869\_Mf\_liverA, CL4847.Contig1\_Mf\_liverA, Unigene28822\_Mf\_liverA, NM\_053214, Unigene36417\_Mf\_liverA, CL2326.Contig1\_Mf\_liverA, Unigene44317\_Mf\_liverA, NM\_177093, Unigene25047\_Mf\_liverA, CL4722.Contig1\_Mf\_liverA, Unigene14907\_Mf\_liverA, CL848.Contig2\_Mf\_liverA, Unigene23870\_Mf\_liverA, Unigene1479\_Mf\_liverA, Unigene4768\_Mf\_liverA, CL2131.Contig4\_Mf\_liverA, Unigene25662\_Mf\_liverA, CL3198.Contig1\_Mf\_liverA, CL479.Contig1\_Mf\_liverA, Unigene14637\_Mf\_liverA, Unigene37460\_Mf\_liverA, Unigene31517\_Mf\_liverA, Unigene9475\_Mf\_liverA, Unigene37662\_Mf\_liverA, NM\_008538, Unigene27895\_Mf\_liverA, NM\_011305, NM\_008610, Unigene27438\_Mf\_liverA, Unigene36631\_Mf\_liverA, Unigene14896\_Mf\_liverA, Unigene37575\_Mf\_liverA, Unigene43515\_Mf\_liverA, CL3803.Contig2\_Mf\_liverA, Unigene27547\_Mf\_liverA, Unigene24284\_Mf\_liverA, Unigene26873\_Mf\_liverA, NM\_009091, Unigene27260\_Mf\_liverA, NM\_134156, CL3268.Contig1\_Mf\_liverA, NM\_010227, Unigene21317\_Mf\_liverA, Unigene37470\_Mf\_liverA, Unigene8132\_Mf\_liverA, CL795.Contig1\_Mf\_liverA, Unigene152\_Mf\_liverA, Unigene27082\_Mf\_liverA, Unigene31852\_Mf\_liverA, NM\_009087, Unigene35169\_Mf\_liverA, Unigene37076\_Mf\_liverA, Unigene2678\_Mf\_liverA, Unigene5325\_Mf\_liverA, Unigene21256\_Mf\_liverA, CL2962.Contig1\_Mf\_liverA, Unigene23055\_Mf\_liverA, CL1352.Contig1\_Mf\_liverA, NM\_033374, Unigene26214\_Mf\_liverA, CL5576.Contig1\_Mf\_liverA, Unigene2746\_Mf\_liverA, NM\_001025388, NM\_009447, Unigene5472\_Mf\_liverA, Unigene26515\_Mf\_liverA, CL1537.Contig1\_Mf\_liverA, CL507.Contig1\_Mf\_liverA, Unigene15318\_Mf\_liverA, Unigene7412\_Mf\_liverA, CL425.Contig1\_Mf\_liverA, Unigene38339\_Mf\_liverA, Unigene14916\_Mf\_liverA, CL4162.Contig1\_Mf\_liverA, Unigene35237\_Mf\_liverA, NM\_010579, Unigene673\_Mf\_liverA, Unigene5693\_Mf\_liverA, Unigene50250\_Mf\_liverA, Unigene13271\_Mf\_liverA, Unigene5165\_Mf\_liverA, CL4057.Contig1\_Mf\_liverA, Unigene35958\_Mf\_liverA, Unigene29876\_Mf\_liverA, Unigene30707\_Mf\_liverA, Unigene36414\_Mf\_liverA, Unigene25080\_Mf\_liverA, Unigene33880\_Mf\_liverA, Unigene34219\_Mf\_liverA, CL1222.Contig1\_Mf\_liverA, Unigene15058\_Mf\_liverA, CL1810.Contig1\_Mf\_liverA, NM\_009609, Unigene35491\_Mf\_liverA, Unigene33168\_Mf\_liverA, Unigene27483\_Mf\_liverA, Unigene36710\_Mf\_liverA, Unigene5940\_Mf\_liverA, NM\_007393, CL5049.Contig2\_Mf\_liverA, Unigene34035\_Mf\_liverA, Unigene34446\_Mf\_liverA, CL5688.Contig1\_Mf\_liverA, Unigene13683\_Mf\_liverA, NM\_053072, Unigene24801\_Mf\_liverA, CL2791.Contig1\_Mf\_liverA, Unigene24755\_Mf\_liverA, Unigene9698\_Mf\_liverA, Unigene28499\_Mf\_liverA, Unigene36420\_Mf\_liverA, CL4106.Contig1\_Mf\_liverA, Unigene24758\_Mf\_liverA, Unigene23271\_Mf\_liverA, Unigene34197\_Mf\_liverA, NM\_026823, Unigene35168\_Mf\_liverA, Unigene8016\_Mf\_liverA, Unigene28527\_Mf\_liverA, NM\_009883, NM\_009448, Unigene1327\_Mf\_liverA, CL2251.Contig1\_Mf\_liverA, CL4432.Contig2\_Mf\_liverA, Unigene4540\_Mf\_liverA, Unigene24566\_Mf\_liverA, NM\_007478, Unigene14263\_Mf\_liverA, NM\_016861, Unigene15170\_Mf\_liverA, Unigene14212\_Mf\_liverA, NM\_011418, Unigene26250\_Mf\_liverA, Unigene24477\_Mf\_liverA, Unigene20432\_Mf\_liverA, Unigene30731\_Mf\_liverA, Unigene48460\_Mf\_liverA, Unigene36418\_Mf\_liverA, NM\_017379, Unigene550\_Mf\_liverA, Unigene16671\_Mf\_liverA, Unigene10313\_Mf\_liverA, Unigene26309\_Mf\_liverA, Unigene29302\_Mf\_liverA, Unigene37711\_Mf\_liverA, Unigene2195\_Mf\_liverA, NM\_146016, Unigene7968\_Mf\_liverA, Unigene93\_Mf\_liverA, Unigene36673\_Mf\_liverA, CL4434.Contig1\_Mf\_liverA, Unigene5147\_Mf\_liverA, Unigene31885\_Mf\_liverA, Unigene30412\_Mf\_liverA, CL5698.Contig1\_Mf\_liverA, Unigene31392\_Mf\_liverA, CL373.Contig7\_Mf\_liverA, Unigene37474\_Mf\_liverA, Unigene42855\_Mf\_liverA, CL548.Contig1\_Mf\_liverA, NM\_007392, CL591.Contig1\_Mf\_liverA, Unigene4556\_Mf\_liverA, Unigene15493\_Mf\_liverA |
| cell body | Unigene29399\_Mf\_liverA, Unigene13525\_Mf\_liverA, Unigene27547\_Mf\_liverA, Unigene28527\_Mf\_liverA, Unigene4720\_Mf\_liverA, Unigene34789\_Mf\_liverA, CL1810.Contig1\_Mf\_liverA, Unigene7950\_Mf\_liverA, Unigene5037\_Mf\_liverA, CL425.Contig1\_Mf\_liverA, Unigene8132\_Mf\_liverA, Unigene8740\_Mf\_liverA, Unigene5194\_Mf\_liverA, NM\_175260, Unigene4686\_Mf\_liverA, Unigene34866\_Mf\_liverA, Unigene5712\_Mf\_liverA, Unigene5138\_Mf\_liverA, Unigene37076\_Mf\_liverA, Unigene14582\_Mf\_liverA, Unigene9990\_Mf\_liverA, CL2117.Contig1\_Mf\_liverA, Unigene37259\_Mf\_liverA, Unigene26214\_Mf\_liverA, Unigene35269\_Mf\_liverA, Unigene38657\_Mf\_liverA, Unigene5165\_Mf\_liverA, Unigene37433\_Mf\_liverA |
| stereocilium | CL1352.Contig1\_Mf\_liverA, Unigene34197\_Mf\_liverA |
| basolateral plasma membrane | Unigene431\_Mf\_liverA, Unigene25080\_Mf\_liverA, Unigene24284\_Mf\_liverA, Unigene34197\_Mf\_liverA, Unigene16671\_Mf\_liverA, NM\_134156, Unigene29938\_Mf\_liverA, CL1256.Contig1\_Mf\_liverA, Unigene29302\_Mf\_liverA, Unigene28186\_Mf\_liverA, CL3150.Contig1\_Mf\_liverA, Unigene27483\_Mf\_liverA, Unigene5422\_Mf\_liverA, Unigene29424\_Mf\_liverA, Unigene35816\_Mf\_liverA, Unigene28331\_Mf\_liverA, Unigene31852\_Mf\_liverA, CL238.Contig1\_Mf\_liverA, CL1738.Contig1\_Mf\_liverA, Unigene40824\_Mf\_liverA, Unigene15656\_Mf\_liverA |
| cell junction | CL507.Contig1\_Mf\_liverA, Unigene29938\_Mf\_liverA, NM\_001159724, Unigene37278\_Mf\_liverA, Unigene23870\_Mf\_liverA, Unigene29424\_Mf\_liverA, Unigene14637\_Mf\_liverA, Unigene39011\_Mf\_liverA, Unigene20432\_Mf\_liverA, Unigene7350\_Mf\_liverA, Unigene5693\_Mf\_liverA, Unigene25052\_Mf\_liverA, Unigene14896\_Mf\_liverA, Unigene15656\_Mf\_liverA, Unigene30707\_Mf\_liverA, Unigene24284\_Mf\_liverA, Unigene39875\_Mf\_liverA, Unigene25080\_Mf\_liverA, CL5586.Contig1\_Mf\_liverA, Unigene16671\_Mf\_liverA, Unigene36910\_Mf\_liverA, NM\_134156, Unigene29885\_Mf\_liverA, Unigene29302\_Mf\_liverA, CL4033.Contig1\_Mf\_liverA, Unigene27483\_Mf\_liverA, Unigene5422\_Mf\_liverA, Unigene28331\_Mf\_liverA, CL4434.Contig1\_Mf\_liverA, Unigene31852\_Mf\_liverA, Unigene27082\_Mf\_liverA, Unigene14765\_Mf\_liverA, CL2117.Contig1\_Mf\_liverA, CL1738.Contig1\_Mf\_liverA, CL548.Contig1\_Mf\_liverA, Unigene26214\_Mf\_liverA, CL5576.Contig1\_Mf\_liverA, Unigene7733\_Mf\_liverA, Unigene40824\_Mf\_liverA, Unigene2746\_Mf\_liverA, Unigene2745\_Mf\_liverA, Unigene39231\_Mf\_liverA, Unigene23869\_Mf\_liverA |
| rough endoplasmic reticulum | Unigene21466\_Mf\_liverA, Unigene8740\_Mf\_liverA, Unigene5194\_Mf\_liverA |
| ER to Golgi transport vesicle membrane | CL2855.Contig1\_Mf\_liverA, CL2855.Contig2\_Mf\_liverA |
| stereocilium bundle | CL1352.Contig1\_Mf\_liverA, Unigene34197\_Mf\_liverA |
| clathrin-coated endocytic vesicle membrane | CL2855.Contig1\_Mf\_liverA, CL2855.Contig2\_Mf\_liverA |
| early endosome | Unigene37245\_Mf\_liverA, Unigene27082\_Mf\_liverA, NM\_133838, CL3002.Contig1\_Mf\_liverA, NM\_010391, Unigene21857\_Mf\_liverA, Unigene4703\_Mf\_liverA, Unigene50250\_Mf\_liverA, Unigene1440\_Mf\_liverA, NR\_004446 |
| sarcomere | Unigene9475\_Mf\_liverA, CL4847.Contig1\_Mf\_liverA, Unigene2678\_Mf\_liverA, Unigene4557\_Mf\_liverA, NM\_134156, Unigene26250\_Mf\_liverA, NM\_007392, Unigene24755\_Mf\_liverA, Unigene4556\_Mf\_liverA, CL2251.Contig1\_Mf\_liverA |
| histone acetyltransferase complex | NM\_009609, NM\_177093, Unigene34219\_Mf\_liverA, Unigene1479\_Mf\_liverA, NM\_007393, CL1222.Contig1\_Mf\_liverA, Unigene30288\_Mf\_liverA |
| nuclear origin of replication recognition complex | Unigene23271\_Mf\_liverA, Unigene23273\_Mf\_liverA |
| organelle envelope | Unigene1162\_Mf\_liverA, Unigene26515\_Mf\_liverA, Unigene34197\_Mf\_liverA, Unigene28142\_Mf\_liverA, NM\_009592, Unigene38406\_Mf\_liverA, Unigene13414\_Mf\_liverA, NM\_018815, Unigene51055\_Mf\_liverA, NM\_008293, NM\_080638, CL2797.Contig2\_Mf\_liverA, CL1190.Contig3\_Mf\_liverA, Unigene38208\_Mf\_liverA, CL4490.Contig2\_Mf\_liverA, CL1125.Contig1\_Mf\_liverA, CL4162.Contig1\_Mf\_liverA, Unigene36641\_Mf\_liverA, Unigene14212\_Mf\_liverA, CL2722.Contig1\_Mf\_liverA, Unigene995\_Mf\_liverA, Unigene30303\_Mf\_liverA, CL5631.Contig1\_Mf\_liverA, Unigene23185\_Mf\_liverA, Unigene25398\_Mf\_liverA, Unigene21255\_Mf\_liverA, Unigene27260\_Mf\_liverA, Unigene36845\_Mf\_liverA, CL1810.Contig1\_Mf\_liverA, Unigene15703\_Mf\_liverA, Unigene25057\_Mf\_liverA, CL3104.Contig1\_Mf\_liverA, Unigene8132\_Mf\_liverA, Unigene2\_Mf\_liverA, CL5254.Contig1\_Mf\_liverA, Unigene4781\_Mf\_liverA, Unigene42812\_Mf\_liverA, Unigene29253\_Mf\_liverA, NM\_181517, Unigene27082\_Mf\_liverA, Unigene5456\_Mf\_liverA, NM\_153193, Unigene13266\_Mf\_liverA, Unigene21359\_Mf\_liverA, Unigene18117\_Mf\_liverA, Unigene3174\_Mf\_liverA, Unigene21256\_Mf\_liverA, CL2117.Contig1\_Mf\_liverA, Unigene5512\_Mf\_liverA, NM\_033444, Unigene16891\_Mf\_liverA, Unigene13233\_Mf\_liverA, Unigene25462\_Mf\_liverA, CL4106.Contig1\_Mf\_liverA |
| organelle part | NM\_176843, Unigene34656\_Mf\_liverA, NM\_009898, Unigene5852\_Mf\_liverA, NM\_009592, Unigene7612\_Mf\_liverA, NM\_018815, Unigene36851\_Mf\_liverA, Unigene14665\_Mf\_liverA, NM\_080638, Unigene29424\_Mf\_liverA, Unigene34218\_Mf\_liverA, CL4105.Contig1\_Mf\_liverA, CL5560.Contig1\_Mf\_liverA, Unigene22575\_Mf\_liverA, Unigene5248\_Mf\_liverA, Unigene39886\_Mf\_liverA, Unigene26422\_Mf\_liverA, CL33.Contig4\_Mf\_liverA, Unigene29334\_Mf\_liverA, Unigene15064\_Mf\_liverA, NM\_010877, Unigene34124\_Mf\_liverA, Unigene38919\_Mf\_liverA, Unigene7510\_Mf\_liverA, Unigene24252\_Mf\_liverA, CL4757.Contig1\_Mf\_liverA, CL5586.Contig1\_Mf\_liverA, Unigene4557\_Mf\_liverA, Unigene21255\_Mf\_liverA, Unigene34341\_Mf\_liverA, Unigene35431\_Mf\_liverA, Unigene15703\_Mf\_liverA, Unigene2\_Mf\_liverA, Unigene35816\_Mf\_liverA, Unigene28331\_Mf\_liverA, CL33.Contig3\_Mf\_liverA, NM\_145474, Unigene13266\_Mf\_liverA, NM\_153193, Unigene5138\_Mf\_liverA, Unigene21857\_Mf\_liverA, Unigene8054\_Mf\_liverA, Unigene23869\_Mf\_liverA, Unigene5598\_Mf\_liverA, NM\_053214, Unigene36417\_Mf\_liverA, CL2855.Contig2\_Mf\_liverA, NM\_177093, Unigene44317\_Mf\_liverA, Unigene25047\_Mf\_liverA, Unigene14907\_Mf\_liverA, Unigene7476\_Mf\_liverA, Unigene1479\_Mf\_liverA, Unigene4922\_Mf\_liverA, Unigene25976\_Mf\_liverA, Unigene14637\_Mf\_liverA, Unigene9475\_Mf\_liverA, Unigene37662\_Mf\_liverA, Unigene27895\_Mf\_liverA, Unigene36112\_Mf\_liverA, Unigene36641\_Mf\_liverA, Unigene9990\_Mf\_liverA, NM\_001104531, Unigene139\_Mf\_liverA, NM\_153055, Unigene995\_Mf\_liverA, Unigene34123\_Mf\_liverA, CL3055.Contig2\_Mf\_liverA, Unigene37575\_Mf\_liverA, Unigene43515\_Mf\_liverA, Unigene25398\_Mf\_liverA, Unigene27547\_Mf\_liverA, Unigene11097\_Mf\_liverA, NM\_134156, Unigene37389\_Mf\_liverA, Unigene32332\_Mf\_liverA, CL5254.Contig1\_Mf\_liverA, Unigene29253\_Mf\_liverA, Unigene34010\_Mf\_liverA, Unigene152\_Mf\_liverA, Unigene5456\_Mf\_liverA, NM\_009087, Unigene37076\_Mf\_liverA, Unigene2678\_Mf\_liverA, Unigene1280\_Mf\_liverA, Unigene21256\_Mf\_liverA, Unigene30288\_Mf\_liverA, CL5576.Contig1\_Mf\_liverA, Unigene2746\_Mf\_liverA, NR\_003552, Unigene5236\_Mf\_liverA, Unigene28021\_Mf\_liverA, Unigene37433\_Mf\_liverA, Unigene5472\_Mf\_liverA, Unigene26515\_Mf\_liverA, CL425.Contig1\_Mf\_liverA, CL2797.Contig2\_Mf\_liverA, Unigene14916\_Mf\_liverA, Unigene30808\_Mf\_liverA, Unigene5693\_Mf\_liverA, Unigene50250\_Mf\_liverA, Unigene19821\_Mf\_liverA, CL4057.Contig1\_Mf\_liverA, Unigene35958\_Mf\_liverA, Unigene1440\_Mf\_liverA, Unigene43037\_Mf\_liverA, Unigene29876\_Mf\_liverA, Unigene13525\_Mf\_liverA, Unigene25080\_Mf\_liverA, CL1988.Contig3\_Mf\_liverA, Unigene1129\_Mf\_liverA, CL3055.Contig1\_Mf\_liverA, Unigene29885\_Mf\_liverA, NM\_009609, CL1810.Contig1\_Mf\_liverA, CL4033.Contig1\_Mf\_liverA, Unigene33168\_Mf\_liverA, Unigene27483\_Mf\_liverA, CL3738.Contig1\_Mf\_liverA, Unigene28662\_Mf\_liverA, Unigene4781\_Mf\_liverA, Unigene46615\_Mf\_liverA, Unigene35935\_Mf\_liverA, CL5049.Contig2\_Mf\_liverA, Unigene34446\_Mf\_liverA, CL1493.Contig1\_Mf\_liverA, Unigene13683\_Mf\_liverA, Unigene24801\_Mf\_liverA, Unigene24755\_Mf\_liverA, Unigene9698\_Mf\_liverA, Unigene28499\_Mf\_liverA, CL3685.Contig1\_Mf\_liverA, Unigene36420\_Mf\_liverA, Unigene542\_Mf\_liverA, Unigene24758\_Mf\_liverA, Unigene23271\_Mf\_liverA, Unigene4909\_Mf\_liverA, Unigene34197\_Mf\_liverA, Unigene24471\_Mf\_liverA, NM\_026823, Unigene28527\_Mf\_liverA, Unigene15888\_Mf\_liverA, NM\_010162, Unigene15529\_Mf\_liverA, CL2251.Contig1\_Mf\_liverA, CL4490.Contig2\_Mf\_liverA, Unigene25595\_Mf\_liverA, Unigene14263\_Mf\_liverA, Unigene14212\_Mf\_liverA, Unigene26250\_Mf\_liverA, Unigene48460\_Mf\_liverA, Unigene23185\_Mf\_liverA, Unigene29558\_Mf\_liverA, Unigene35046\_Mf\_liverA, Unigene16671\_Mf\_liverA, Unigene281\_Mf\_liverA, Unigene10313\_Mf\_liverA, Unigene13772\_Mf\_liverA, CL3104.Contig1\_Mf\_liverA, Unigene5687\_Mf\_liverA, Unigene36593\_Mf\_liverA, Unigene5422\_Mf\_liverA, Unigene7968\_Mf\_liverA, Unigene36699\_Mf\_liverA, Unigene36673\_Mf\_liverA, NM\_181517, CL5698.Contig1\_Mf\_liverA, Unigene31392\_Mf\_liverA, Unigene5512\_Mf\_liverA, Unigene42855\_Mf\_liverA, CL591.Contig1\_Mf\_liverA, Unigene4556\_Mf\_liverA, Unigene29399\_Mf\_liverA, NM\_020559, Unigene35884\_Mf\_liverA, Unigene28142\_Mf\_liverA, Unigene38015\_Mf\_liverA, NM\_007896, CL3835.Contig2\_Mf\_liverA, CL1828.Contig1\_Mf\_liverA, NM\_145824, Unigene30356\_Mf\_liverA, Unigene38124\_Mf\_liverA, Unigene29308\_Mf\_liverA, CL523.Contig1\_Mf\_liverA, Unigene29008\_Mf\_liverA, CL1063.Contig1\_Mf\_liverA, CL482.Contig1\_Mf\_liverA, Unigene19049\_Mf\_liverA, Unigene29405\_Mf\_liverA, Unigene32882\_Mf\_liverA, Unigene31263\_Mf\_liverA, CL5358.Contig1\_Mf\_liverA, Unigene7619\_Mf\_liverA, Unigene21466\_Mf\_liverA, Unigene7969\_Mf\_liverA, Unigene30303\_Mf\_liverA, Unigene30878\_Mf\_liverA, Unigene15656\_Mf\_liverA, Unigene34810\_Mf\_liverA, Unigene34234\_Mf\_liverA, CL6038.Contig2\_Mf\_liverA, Unigene5204\_Mf\_liverA, CL5001.Contig2\_Mf\_liverA, Unigene40020\_Mf\_liverA, Unigene25070\_Mf\_liverA, Unigene34789\_Mf\_liverA, CL4141.Contig1\_Mf\_liverA, Unigene25057\_Mf\_liverA, NM\_145419, Unigene23273\_Mf\_liverA, CL1119.Contig1\_Mf\_liverA, Unigene13658\_Mf\_liverA, Unigene41158\_Mf\_liverA, NM\_175260, Unigene4686\_Mf\_liverA, Unigene25596\_Mf\_liverA, Unigene18117\_Mf\_liverA, CL1493.Contig2\_Mf\_liverA, Unigene3174\_Mf\_liverA, CL2117.Contig1\_Mf\_liverA, Unigene22978\_Mf\_liverA, Unigene25046\_Mf\_liverA, Unigene36988\_Mf\_liverA, Unigene28094\_Mf\_liverA, Unigene5941\_Mf\_liverA, CL2574.Contig1\_Mf\_liverA, Unigene23328\_Mf\_liverA, Unigene2745\_Mf\_liverA, Unigene25462\_Mf\_liverA, Unigene16684\_Mf\_liverA, Unigene1162\_Mf\_liverA, CL787.Contig1\_Mf\_liverA, CL4847.Contig1\_Mf\_liverA, NM\_029562, Unigene28822\_Mf\_liverA, Unigene36691\_Mf\_liverA, Unigene38406\_Mf\_liverA, Unigene19297\_Mf\_liverA, CL2326.Contig1\_Mf\_liverA, CL4722.Contig1\_Mf\_liverA, CL848.Contig2\_Mf\_liverA, Unigene23870\_Mf\_liverA, Unigene4768\_Mf\_liverA, Unigene26398\_Mf\_liverA, NM\_008808, CL2131.Contig4\_Mf\_liverA, Unigene34258\_Mf\_liverA, CL3198.Contig1\_Mf\_liverA, Unigene5632\_Mf\_liverA, Unigene25662\_Mf\_liverA, CL479.Contig1\_Mf\_liverA, Unigene37460\_Mf\_liverA, Unigene31517\_Mf\_liverA, NM\_007811, NM\_008538, NM\_134059, NM\_011305, CL3816.Contig1\_Mf\_liverA, CL2722.Contig1\_Mf\_liverA, Unigene27438\_Mf\_liverA, CL5631.Contig1\_Mf\_liverA, Unigene14896\_Mf\_liverA, CL3803.Contig2\_Mf\_liverA, Unigene19885\_Mf\_liverA, Unigene36510\_Mf\_liverA, Unigene26873\_Mf\_liverA, NM\_009091, Unigene27260\_Mf\_liverA, CL3268.Contig1\_Mf\_liverA, Unigene10820\_Mf\_liverA, Unigene36845\_Mf\_liverA, NM\_010227, Unigene21317\_Mf\_liverA, Unigene30493\_Mf\_liverA, Unigene37729\_Mf\_liverA, Unigene8132\_Mf\_liverA, Unigene29940\_Mf\_liverA, CL795.Contig1\_Mf\_liverA, NM\_019717, Unigene42812\_Mf\_liverA, Unigene27082\_Mf\_liverA, Unigene5712\_Mf\_liverA, Unigene5325\_Mf\_liverA, Unigene1205\_Mf\_liverA, CL2962.Contig1\_Mf\_liverA, CL442.Contig2\_Mf\_liverA, CL1352.Contig1\_Mf\_liverA, Unigene26214\_Mf\_liverA, CL2855.Contig1\_Mf\_liverA, Unigene13233\_Mf\_liverA, NM\_011132, Unigene5648\_Mf\_liverA, NM\_001100182, CL1537.Contig1\_Mf\_liverA, NM\_010481, NM\_009128, CL507.Contig1\_Mf\_liverA, NM\_175472, Unigene15318\_Mf\_liverA, Unigene7412\_Mf\_liverA, NM\_009178, CL2240.Contig1\_Mf\_liverA, Unigene13414\_Mf\_liverA, NM\_008293, Unigene22980\_Mf\_liverA, Unigene38339\_Mf\_liverA, Unigene38208\_Mf\_liverA, CL1125.Contig1\_Mf\_liverA, CL4162.Contig1\_Mf\_liverA, Unigene35237\_Mf\_liverA, CL5993.Contig3\_Mf\_liverA, NM\_010579, Unigene673\_Mf\_liverA, Unigene7350\_Mf\_liverA, Unigene4703\_Mf\_liverA, Unigene5165\_Mf\_liverA, Unigene23013\_Mf\_liverA, Unigene30707\_Mf\_liverA, CL4583.Contig2\_Mf\_liverA, Unigene36414\_Mf\_liverA, NM\_133838, Unigene34219\_Mf\_liverA, Unigene33880\_Mf\_liverA, Unigene36328\_Mf\_liverA, CL3002.Contig1\_Mf\_liverA, CL1222.Contig1\_Mf\_liverA, Unigene15058\_Mf\_liverA, Unigene35491\_Mf\_liverA, NM\_029872, Unigene36710\_Mf\_liverA, Unigene5906\_Mf\_liverA, Unigene5940\_Mf\_liverA, NM\_007393, Unigene34035\_Mf\_liverA, Unigene28731\_Mf\_liverA, CL5688.Contig1\_Mf\_liverA, NM\_033444, CL2791.Contig1\_Mf\_liverA, Unigene24085\_Mf\_liverA, Unigene10135\_Mf\_liverA, CL4106.Contig1\_Mf\_liverA, Unigene22330\_Mf\_liverA, NM\_009883, Unigene5954\_Mf\_liverA, CL4127.Contig1\_Mf\_liverA, NM\_009448, CL5307.Contig1\_Mf\_liverA, Unigene31730\_Mf\_liverA, Unigene51055\_Mf\_liverA, CL1190.Contig3\_Mf\_liverA, CL4432.Contig2\_Mf\_liverA, Unigene4540\_Mf\_liverA, Unigene24566\_Mf\_liverA, Unigene37310\_Mf\_liverA, Unigene15170\_Mf\_liverA, NM\_011418, Unigene4604\_Mf\_liverA, Unigene560\_Mf\_liverA, Unigene27803\_Mf\_liverA, Unigene30731\_Mf\_liverA, Unigene36418\_Mf\_liverA, Unigene1221\_Mf\_liverA, CL2355.Contig1\_Mf\_liverA, Unigene37153\_Mf\_liverA, Unigene15592\_Mf\_liverA, Unigene5745\_Mf\_liverA, Unigene37711\_Mf\_liverA, Unigene12889\_Mf\_liverA, Unigene93\_Mf\_liverA, Unigene803\_Mf\_liverA, CL4434.Contig1\_Mf\_liverA, Unigene5147\_Mf\_liverA, CL3911.Contig2\_Mf\_liverA, Unigene21359\_Mf\_liverA, Unigene36487\_Mf\_liverA, Unigene4681\_Mf\_liverA, Unigene37474\_Mf\_liverA, CL548.Contig1\_Mf\_liverA, NM\_007392, Unigene16891\_Mf\_liverA, Unigene5886\_Mf\_liverA, Unigene15493\_Mf\_liverA |
| trans-Golgi network | Unigene30795\_Mf\_liverA, CL2855.Contig1\_Mf\_liverA, CL3685.Contig1\_Mf\_liverA, CL2855.Contig2\_Mf\_liverA, Unigene15474\_Mf\_liverA, Unigene31988\_Mf\_liverA |
| cell division site | CL4434.Contig1\_Mf\_liverA, Unigene5693\_Mf\_liverA, CL507.Contig1\_Mf\_liverA, NM\_175260 |
| cell division site part | CL4434.Contig1\_Mf\_liverA, Unigene5693\_Mf\_liverA, CL507.Contig1\_Mf\_liverA, NM\_175260 |
| centriole | Unigene673\_Mf\_liverA, Unigene27895\_Mf\_liverA, Unigene43515\_Mf\_liverA |
| microtubule organizing center part | Unigene7412\_Mf\_liverA, CL1537.Contig1\_Mf\_liverA, Unigene673\_Mf\_liverA, Unigene27895\_Mf\_liverA, Unigene43515\_Mf\_liverA |
| cullin-RING ubiquitin ligase complex | Unigene5248\_Mf\_liverA, Unigene33054\_Mf\_liverA, Unigene48460\_Mf\_liverA, Unigene29334\_Mf\_liverA, Unigene5954\_Mf\_liverA, Unigene13683\_Mf\_liverA |
| envelope | Unigene1162\_Mf\_liverA, Unigene26515\_Mf\_liverA, Unigene34197\_Mf\_liverA, Unigene28142\_Mf\_liverA, NM\_009592, Unigene38406\_Mf\_liverA, Unigene13414\_Mf\_liverA, NM\_018815, Unigene51055\_Mf\_liverA, NM\_008293, NM\_080638, CL2797.Contig2\_Mf\_liverA, CL1190.Contig3\_Mf\_liverA, Unigene38208\_Mf\_liverA, CL4490.Contig2\_Mf\_liverA, CL1125.Contig1\_Mf\_liverA, CL4162.Contig1\_Mf\_liverA, Unigene36641\_Mf\_liverA, Unigene14212\_Mf\_liverA, CL2722.Contig1\_Mf\_liverA, Unigene995\_Mf\_liverA, Unigene30303\_Mf\_liverA, CL5631.Contig1\_Mf\_liverA, Unigene23185\_Mf\_liverA, Unigene25398\_Mf\_liverA, Unigene21255\_Mf\_liverA, Unigene27260\_Mf\_liverA, Unigene36845\_Mf\_liverA, CL1810.Contig1\_Mf\_liverA, Unigene15703\_Mf\_liverA, Unigene25057\_Mf\_liverA, CL3104.Contig1\_Mf\_liverA, Unigene8132\_Mf\_liverA, Unigene2\_Mf\_liverA, CL5254.Contig1\_Mf\_liverA, Unigene4781\_Mf\_liverA, Unigene42812\_Mf\_liverA, Unigene29253\_Mf\_liverA, NM\_181517, Unigene27082\_Mf\_liverA, Unigene5456\_Mf\_liverA, NM\_153193, Unigene13266\_Mf\_liverA, Unigene21359\_Mf\_liverA, Unigene18117\_Mf\_liverA, Unigene3174\_Mf\_liverA, Unigene21256\_Mf\_liverA, CL2117.Contig1\_Mf\_liverA, Unigene5512\_Mf\_liverA, NM\_033444, Unigene16891\_Mf\_liverA, Unigene13233\_Mf\_liverA, Unigene25462\_Mf\_liverA, CL4106.Contig1\_Mf\_liverA |
| mitochondrial membrane | Unigene1162\_Mf\_liverA, Unigene25398\_Mf\_liverA, Unigene27260\_Mf\_liverA, Unigene36845\_Mf\_liverA, CL1810.Contig1\_Mf\_liverA, CL3104.Contig1\_Mf\_liverA, CL2797.Contig2\_Mf\_liverA, CL1190.Contig3\_Mf\_liverA, Unigene2\_Mf\_liverA, Unigene4781\_Mf\_liverA, Unigene38208\_Mf\_liverA, Unigene27082\_Mf\_liverA, CL1125.Contig1\_Mf\_liverA, CL4490.Contig2\_Mf\_liverA, Unigene13266\_Mf\_liverA, Unigene21359\_Mf\_liverA, Unigene18117\_Mf\_liverA, Unigene36641\_Mf\_liverA, Unigene3174\_Mf\_liverA, Unigene5512\_Mf\_liverA, Unigene995\_Mf\_liverA, CL2722.Contig1\_Mf\_liverA, Unigene30303\_Mf\_liverA, CL5631.Contig1\_Mf\_liverA, Unigene13233\_Mf\_liverA, Unigene25462\_Mf\_liverA, Unigene23185\_Mf\_liverA |
| clathrin-coated endocytic vesicle | CL2855.Contig1\_Mf\_liverA, CL2855.Contig2\_Mf\_liverA |
| dendritic spine | CL479.Contig1\_Mf\_liverA, Unigene30707\_Mf\_liverA, Unigene27547\_Mf\_liverA, Unigene27260\_Mf\_liverA, CL1222.Contig1\_Mf\_liverA, CL425.Contig1\_Mf\_liverA, Unigene5037\_Mf\_liverA, Unigene24252\_Mf\_liverA |
| neuron spine | CL479.Contig1\_Mf\_liverA, Unigene30707\_Mf\_liverA, Unigene27547\_Mf\_liverA, Unigene27260\_Mf\_liverA, CL1222.Contig1\_Mf\_liverA, CL425.Contig1\_Mf\_liverA, Unigene5037\_Mf\_liverA, Unigene24252\_Mf\_liverA |
| mitochondrial outer membrane | CL1810.Contig1\_Mf\_liverA, CL3104.Contig1\_Mf\_liverA, Unigene995\_Mf\_liverA, Unigene27082\_Mf\_liverA, CL5631.Contig1\_Mf\_liverA, Unigene25462\_Mf\_liverA, Unigene4781\_Mf\_liverA |
| intrinsic to organelle membrane | CL787.Contig1\_Mf\_liverA, Unigene38208\_Mf\_liverA, Unigene5598\_Mf\_liverA, NM\_009128, Unigene15888\_Mf\_liverA, NM\_010162, CL2855.Contig2\_Mf\_liverA, CL5560.Contig1\_Mf\_liverA, Unigene5512\_Mf\_liverA, NM\_009178, CL3104.Contig1\_Mf\_liverA, CL2855.Contig1\_Mf\_liverA, Unigene1479\_Mf\_liverA, Unigene10135\_Mf\_liverA |
| nuclear membrane | Unigene26515\_Mf\_liverA, Unigene5456\_Mf\_liverA, Unigene21255\_Mf\_liverA, Unigene21256\_Mf\_liverA, CL2117.Contig1\_Mf\_liverA, Unigene38406\_Mf\_liverA, Unigene8132\_Mf\_liverA, CL4106.Contig1\_Mf\_liverA, Unigene29253\_Mf\_liverA |
| replication fork | CL2326.Contig1\_Mf\_liverA, Unigene31392\_Mf\_liverA, CL4757.Contig1\_Mf\_liverA |
| pore complex | CL3104.Contig1\_Mf\_liverA, Unigene25057\_Mf\_liverA, Unigene34197\_Mf\_liverA, Unigene36667\_Mf\_liverA |
| nucleolus | Unigene24758\_Mf\_liverA, CL1537.Contig1\_Mf\_liverA, Unigene34197\_Mf\_liverA, Unigene36417\_Mf\_liverA, CL4722.Contig1\_Mf\_liverA, Unigene1479\_Mf\_liverA, Unigene38339\_Mf\_liverA, Unigene29424\_Mf\_liverA, CL2131.Contig4\_Mf\_liverA, CL1063.Contig1\_Mf\_liverA, Unigene24566\_Mf\_liverA, Unigene14916\_Mf\_liverA, Unigene35237\_Mf\_liverA, Unigene32882\_Mf\_liverA, Unigene14212\_Mf\_liverA, Unigene27438\_Mf\_liverA, Unigene30731\_Mf\_liverA, Unigene36418\_Mf\_liverA, CL4057.Contig1\_Mf\_liverA, Unigene35958\_Mf\_liverA, Unigene37575\_Mf\_liverA, CL4757.Contig1\_Mf\_liverA, Unigene43515\_Mf\_liverA, Unigene29876\_Mf\_liverA, Unigene36414\_Mf\_liverA, Unigene25080\_Mf\_liverA, Unigene33880\_Mf\_liverA, Unigene34219\_Mf\_liverA, Unigene16671\_Mf\_liverA, Unigene26873\_Mf\_liverA, Unigene21255\_Mf\_liverA, CL3268.Contig1\_Mf\_liverA, Unigene10313\_Mf\_liverA, CL1810.Contig1\_Mf\_liverA, Unigene35491\_Mf\_liverA, Unigene35431\_Mf\_liverA, Unigene33168\_Mf\_liverA, Unigene27483\_Mf\_liverA, Unigene37711\_Mf\_liverA, Unigene152\_Mf\_liverA, NM\_009087, Unigene36710\_Mf\_liverA, Unigene34035\_Mf\_liverA, Unigene31392\_Mf\_liverA, Unigene21256\_Mf\_liverA, CL2962.Contig1\_Mf\_liverA, CL5688.Contig1\_Mf\_liverA, Unigene13683\_Mf\_liverA, Unigene24801\_Mf\_liverA, Unigene42855\_Mf\_liverA, CL548.Contig1\_Mf\_liverA, Unigene36988\_Mf\_liverA, CL2791.Contig1\_Mf\_liverA, Unigene9698\_Mf\_liverA, Unigene36420\_Mf\_liverA, Unigene16684\_Mf\_liverA |
| cytosolic large ribosomal subunit | Unigene36710\_Mf\_liverA, Unigene5325\_Mf\_liverA |
| synapse | Unigene30707\_Mf\_liverA, Unigene29705\_Mf\_liverA, Unigene28527\_Mf\_liverA, Unigene13363\_Mf\_liverA, CL507.Contig1\_Mf\_liverA, Unigene27260\_Mf\_liverA, CL1222.Contig1\_Mf\_liverA, Unigene29938\_Mf\_liverA, Unigene42975\_Mf\_liverA, NM\_175260, CL479.Contig1\_Mf\_liverA, CL4434.Contig1\_Mf\_liverA, Unigene9990\_Mf\_liverA, Unigene37259\_Mf\_liverA, CL1738.Contig1\_Mf\_liverA, CL2117.Contig1\_Mf\_liverA, Unigene7733\_Mf\_liverA, Unigene36631\_Mf\_liverA, Unigene24804\_Mf\_liverA, Unigene1221\_Mf\_liverA, Unigene24252\_Mf\_liverA |
| endoplasmic reticulum-Golgi intermediate compartment | Unigene31730\_Mf\_liverA, Unigene1479\_Mf\_liverA, Unigene5632\_Mf\_liverA |
| extrinsic to internal side of plasma membrane | Unigene24284\_Mf\_liverA, Unigene2919\_Mf\_liverA, Unigene51055\_Mf\_liverA, Unigene34218\_Mf\_liverA |
| mitochondrial intermembrane space | CL4490.Contig2\_Mf\_liverA, CL2797.Contig2\_Mf\_liverA, Unigene23185\_Mf\_liverA |
| centrosome | CL1537.Contig1\_Mf\_liverA, Unigene21255\_Mf\_liverA, Unigene21317\_Mf\_liverA, CL1810.Contig1\_Mf\_liverA, Unigene15318\_Mf\_liverA, Unigene7412\_Mf\_liverA, Unigene25057\_Mf\_liverA, Unigene4768\_Mf\_liverA, Unigene34218\_Mf\_liverA, Unigene25662\_Mf\_liverA, Unigene27895\_Mf\_liverA, Unigene15170\_Mf\_liverA, CL5688.Contig1\_Mf\_liverA, CL2117.Contig1\_Mf\_liverA, Unigene673\_Mf\_liverA, Unigene48460\_Mf\_liverA, Unigene2746\_Mf\_liverA, Unigene2745\_Mf\_liverA, Unigene43515\_Mf\_liverA |
| nucleosome | CL4432.Contig2\_Mf\_liverA, CL2326.Contig1\_Mf\_liverA |
| ER to Golgi transport vesicle | CL2855.Contig1\_Mf\_liverA, CL2855.Contig2\_Mf\_liverA |
| microtubule basal body | Unigene25080\_Mf\_liverA, Unigene16671\_Mf\_liverA, Unigene27895\_Mf\_liverA |
| histone methyltransferase complex | NM\_009609, NM\_177093, Unigene30356\_Mf\_liverA, NM\_007393, CL1222.Contig1\_Mf\_liverA |
| origin recognition complex | Unigene23271\_Mf\_liverA, Unigene23273\_Mf\_liverA |
| nuclear heterochromatin | Unigene34035\_Mf\_liverA, NM\_011418 |
| mitochondrial envelope | Unigene1162\_Mf\_liverA, Unigene25398\_Mf\_liverA, Unigene27260\_Mf\_liverA, Unigene36845\_Mf\_liverA, CL1810.Contig1\_Mf\_liverA, CL3104.Contig1\_Mf\_liverA, NM\_008293, CL2797.Contig2\_Mf\_liverA, CL1190.Contig3\_Mf\_liverA, Unigene2\_Mf\_liverA, Unigene4781\_Mf\_liverA, Unigene38208\_Mf\_liverA, Unigene27082\_Mf\_liverA, CL1125.Contig1\_Mf\_liverA, CL4490.Contig2\_Mf\_liverA, NM\_153193, Unigene13266\_Mf\_liverA, Unigene21359\_Mf\_liverA, Unigene18117\_Mf\_liverA, Unigene36641\_Mf\_liverA, Unigene3174\_Mf\_liverA, Unigene5512\_Mf\_liverA, Unigene995\_Mf\_liverA, CL2722.Contig1\_Mf\_liverA, Unigene30303\_Mf\_liverA, CL5631.Contig1\_Mf\_liverA, Unigene13233\_Mf\_liverA, Unigene25462\_Mf\_liverA, Unigene23185\_Mf\_liverA |
| SWI/SNF-type complex | NM\_011418, CL2131.Contig4\_Mf\_liverA |
| mitochondrial matrix | Unigene803\_Mf\_liverA, Unigene14916\_Mf\_liverA, CL1125.Contig1\_Mf\_liverA, Unigene37460\_Mf\_liverA, NM\_010481, Unigene21359\_Mf\_liverA, Unigene36487\_Mf\_liverA, Unigene36691\_Mf\_liverA, CL2791.Contig1\_Mf\_liverA, Unigene30731\_Mf\_liverA, Unigene7476\_Mf\_liverA, Unigene35816\_Mf\_liverA |
| acrosomal membrane | CL3816.Contig1\_Mf\_liverA, Unigene28331\_Mf\_liverA |
| mediator complex | Unigene31263\_Mf\_liverA, Unigene4604\_Mf\_liverA |
| mitochondrial inner membrane | Unigene25398\_Mf\_liverA, Unigene27260\_Mf\_liverA, Unigene36845\_Mf\_liverA, CL2797.Contig2\_Mf\_liverA, Unigene4781\_Mf\_liverA, CL1125.Contig1\_Mf\_liverA, CL4490.Contig2\_Mf\_liverA, Unigene36641\_Mf\_liverA, Unigene18117\_Mf\_liverA, Unigene21359\_Mf\_liverA, Unigene3174\_Mf\_liverA, Unigene5512\_Mf\_liverA, CL2722.Contig1\_Mf\_liverA, Unigene30303\_Mf\_liverA, Unigene13233\_Mf\_liverA, Unigene23185\_Mf\_liverA |
| organelle envelope lumen | CL4490.Contig2\_Mf\_liverA, CL2797.Contig2\_Mf\_liverA, Unigene23185\_Mf\_liverA |
| perikaryon | Unigene5138\_Mf\_liverA, Unigene9990\_Mf\_liverA |
| Golgi membrane | CL787.Contig1\_Mf\_liverA, CL5586.Contig1\_Mf\_liverA, Unigene36328\_Mf\_liverA, Unigene281\_Mf\_liverA, CL2855.Contig2\_Mf\_liverA, Unigene15529\_Mf\_liverA, Unigene37389\_Mf\_liverA, Unigene5687\_Mf\_liverA, NM\_009178, CL4033.Contig1\_Mf\_liverA, Unigene28331\_Mf\_liverA, Unigene4922\_Mf\_liverA, Unigene34010\_Mf\_liverA, Unigene5632\_Mf\_liverA, Unigene27082\_Mf\_liverA, CL3816.Contig1\_Mf\_liverA, CL2855.Contig1\_Mf\_liverA, Unigene28499\_Mf\_liverA, CL3685.Contig1\_Mf\_liverA, Unigene24085\_Mf\_liverA, Unigene1221\_Mf\_liverA, Unigene43037\_Mf\_liverA |
| nuclear inner membrane | Unigene38406\_Mf\_liverA, CL4106.Contig1\_Mf\_liverA |
| autophagic vacuole | CL3685.Contig1\_Mf\_liverA, Unigene36328\_Mf\_liverA |
| SAGA-type complex | Unigene34219\_Mf\_liverA, Unigene30288\_Mf\_liverA |
| flagellum | Unigene31206\_Mf\_liverA, CL2251.Contig1\_Mf\_liverA, Unigene28331\_Mf\_liverA |
| pigment granule | CL725.Contig1\_Mf\_liverA, Unigene34789\_Mf\_liverA, CL3816.Contig1\_Mf\_liverA, Unigene21255\_Mf\_liverA, Unigene21256\_Mf\_liverA, Unigene28021\_Mf\_liverA |
| intercalated disc | Unigene27082\_Mf\_liverA, Unigene37278\_Mf\_liverA |
| Golgi apparatus part | CL787.Contig1\_Mf\_liverA, Unigene29399\_Mf\_liverA, CL5586.Contig1\_Mf\_liverA, Unigene36328\_Mf\_liverA, Unigene281\_Mf\_liverA, CL2855.Contig2\_Mf\_liverA, Unigene5852\_Mf\_liverA, Unigene15529\_Mf\_liverA, NM\_010227, Unigene37389\_Mf\_liverA, Unigene5687\_Mf\_liverA, NM\_009178, CL4033.Contig1\_Mf\_liverA, Unigene5422\_Mf\_liverA, Unigene29940\_Mf\_liverA, Unigene28331\_Mf\_liverA, Unigene4922\_Mf\_liverA, Unigene34010\_Mf\_liverA, Unigene5632\_Mf\_liverA, Unigene27082\_Mf\_liverA, Unigene36112\_Mf\_liverA, Unigene560\_Mf\_liverA, CL3816.Contig1\_Mf\_liverA, CL2855.Contig1\_Mf\_liverA, Unigene28499\_Mf\_liverA, CL3685.Contig1\_Mf\_liverA, Unigene24085\_Mf\_liverA, Unigene1221\_Mf\_liverA, Unigene28021\_Mf\_liverA, Unigene43037\_Mf\_liverA |
| sex chromosome | CL2326.Contig1\_Mf\_liverA, NM\_011418 |
| sarcoplasmic reticulum | Unigene34656\_Mf\_liverA, Unigene29253\_Mf\_liverA |
| heterochromatin | Unigene31517\_Mf\_liverA, Unigene34035\_Mf\_liverA, NM\_011418, CL2131.Contig4\_Mf\_liverA |
| organelle outer membrane | CL1810.Contig1\_Mf\_liverA, CL3104.Contig1\_Mf\_liverA, Unigene995\_Mf\_liverA, Unigene27082\_Mf\_liverA, CL5631.Contig1\_Mf\_liverA, Unigene25462\_Mf\_liverA, Unigene4781\_Mf\_liverA |
| methyltransferase complex | NM\_009609, NM\_177093, Unigene30356\_Mf\_liverA, NM\_007393, CL1222.Contig1\_Mf\_liverA |
| inclusion body | CL1190.Contig3\_Mf\_liverA, Unigene35816\_Mf\_liverA, Unigene9406\_Mf\_liverA |
| protein-DNA complex | CL4432.Contig2\_Mf\_liverA, CL2326.Contig1\_Mf\_liverA, CL2439.Contig1\_Mf\_liverA, Unigene31392\_Mf\_liverA |
| nucleoplasm | NM\_176843, Unigene35884\_Mf\_liverA, Unigene38015\_Mf\_liverA, Unigene30356\_Mf\_liverA, Unigene38124\_Mf\_liverA, Unigene36851\_Mf\_liverA, Unigene14665\_Mf\_liverA, CL4105.Contig1\_Mf\_liverA, CL4162.Contig1\_Mf\_liverA, Unigene31263\_Mf\_liverA, Unigene30808\_Mf\_liverA, Unigene7619\_Mf\_liverA, Unigene7969\_Mf\_liverA, Unigene26422\_Mf\_liverA, Unigene7350\_Mf\_liverA, Unigene29334\_Mf\_liverA, Unigene15064\_Mf\_liverA, Unigene19821\_Mf\_liverA, CL4057.Contig1\_Mf\_liverA, CL4757.Contig1\_Mf\_liverA, Unigene30707\_Mf\_liverA, Unigene34219\_Mf\_liverA, CL5001.Contig2\_Mf\_liverA, CL1222.Contig1\_Mf\_liverA, Unigene1129\_Mf\_liverA, NM\_009609, CL4141.Contig1\_Mf\_liverA, Unigene35431\_Mf\_liverA, Unigene23273\_Mf\_liverA, CL3738.Contig1\_Mf\_liverA, Unigene35816\_Mf\_liverA, CL1119.Contig1\_Mf\_liverA, Unigene13658\_Mf\_liverA, Unigene41158\_Mf\_liverA, Unigene35935\_Mf\_liverA, NM\_007393, Unigene34035\_Mf\_liverA, Unigene24801\_Mf\_liverA, Unigene23271\_Mf\_liverA, Unigene24471\_Mf\_liverA, Unigene34197\_Mf\_liverA, CL4847.Contig1\_Mf\_liverA, Unigene5954\_Mf\_liverA, Unigene19297\_Mf\_liverA, CL2326.Contig1\_Mf\_liverA, NM\_177093, Unigene14907\_Mf\_liverA, CL4722.Contig1\_Mf\_liverA, Unigene1479\_Mf\_liverA, Unigene7476\_Mf\_liverA, Unigene25976\_Mf\_liverA, Unigene31517\_Mf\_liverA, Unigene37310\_Mf\_liverA, Unigene4604\_Mf\_liverA, Unigene14212\_Mf\_liverA, Unigene27438\_Mf\_liverA, Unigene48460\_Mf\_liverA, CL2355.Contig1\_Mf\_liverA, CL3268.Contig1\_Mf\_liverA, Unigene10820\_Mf\_liverA, Unigene37153\_Mf\_liverA, Unigene12889\_Mf\_liverA, Unigene7968\_Mf\_liverA, Unigene42812\_Mf\_liverA, Unigene152\_Mf\_liverA, Unigene31392\_Mf\_liverA, Unigene30288\_Mf\_liverA, Unigene42855\_Mf\_liverA, Unigene37433\_Mf\_liverA |
| sarcoplasm | Unigene34656\_Mf\_liverA, Unigene29253\_Mf\_liverA |
| pronucleus | Unigene19297\_Mf\_liverA, Unigene10820\_Mf\_liverA |
| chromatin | Unigene26515\_Mf\_liverA, Unigene23271\_Mf\_liverA, Unigene15058\_Mf\_liverA, NM\_009883, CL2326.Contig1\_Mf\_liverA, Unigene14907\_Mf\_liverA, Unigene38124\_Mf\_liverA, Unigene23273\_Mf\_liverA, Unigene93\_Mf\_liverA, CL2131.Contig4\_Mf\_liverA, CL4432.Contig2\_Mf\_liverA, Unigene5147\_Mf\_liverA, Unigene31517\_Mf\_liverA, Unigene34035\_Mf\_liverA, NM\_011305, Unigene34446\_Mf\_liverA, NM\_011418, CL4757.Contig1\_Mf\_liverA, CL4106.Contig1\_Mf\_liverA |
| catalytic step 2 spliceosome | CL5001.Contig2\_Mf\_liverA, CL3268.Contig1\_Mf\_liverA, CL4057.Contig1\_Mf\_liverA |
| basal plasma membrane | Unigene34197\_Mf\_liverA, Unigene28331\_Mf\_liverA |
| nuclear chromosome part | Unigene26515\_Mf\_liverA, Unigene23271\_Mf\_liverA, Unigene34035\_Mf\_liverA, Unigene31392\_Mf\_liverA, NM\_011418, CL2326.Contig1\_Mf\_liverA, Unigene42855\_Mf\_liverA, Unigene14907\_Mf\_liverA, Unigene7969\_Mf\_liverA, Unigene38124\_Mf\_liverA, Unigene23273\_Mf\_liverA, Unigene7968\_Mf\_liverA, CL2131.Contig4\_Mf\_liverA, CL4757.Contig1\_Mf\_liverA |
| transport vesicle | CL2855.Contig1\_Mf\_liverA, Unigene37819\_Mf\_liverA, Unigene5204\_Mf\_liverA, Unigene8054\_Mf\_liverA, Unigene9990\_Mf\_liverA, CL2855.Contig2\_Mf\_liverA |
| mitochondrial part | Unigene1162\_Mf\_liverA, NM\_020559, NM\_010481, Unigene36691\_Mf\_liverA, NM\_008293, CL2797.Contig2\_Mf\_liverA, Unigene7476\_Mf\_liverA, CL1190.Contig3\_Mf\_liverA, Unigene38208\_Mf\_liverA, Unigene37460\_Mf\_liverA, CL1125.Contig1\_Mf\_liverA, Unigene14916\_Mf\_liverA, CL4490.Contig2\_Mf\_liverA, Unigene36641\_Mf\_liverA, CL2722.Contig1\_Mf\_liverA, Unigene995\_Mf\_liverA, Unigene30731\_Mf\_liverA, Unigene30303\_Mf\_liverA, CL5631.Contig1\_Mf\_liverA, Unigene23185\_Mf\_liverA, Unigene25398\_Mf\_liverA, Unigene27260\_Mf\_liverA, Unigene36845\_Mf\_liverA, CL1810.Contig1\_Mf\_liverA, CL3104.Contig1\_Mf\_liverA, Unigene4781\_Mf\_liverA, Unigene35816\_Mf\_liverA, Unigene2\_Mf\_liverA, Unigene803\_Mf\_liverA, Unigene27082\_Mf\_liverA, NM\_153193, Unigene13266\_Mf\_liverA, Unigene21359\_Mf\_liverA, Unigene18117\_Mf\_liverA, Unigene3174\_Mf\_liverA, Unigene36487\_Mf\_liverA, Unigene5512\_Mf\_liverA, CL2791.Contig1\_Mf\_liverA, Unigene25462\_Mf\_liverA, Unigene13233\_Mf\_liverA |
| internal side of plasma membrane | Unigene24284\_Mf\_liverA, Unigene2919\_Mf\_liverA, Unigene51055\_Mf\_liverA, Unigene29424\_Mf\_liverA, Unigene37575\_Mf\_liverA, Unigene34218\_Mf\_liverA |
| intracellular part | NM\_176843, Unigene34656\_Mf\_liverA, NM\_009898, Unigene7612\_Mf\_liverA, Unigene7950\_Mf\_liverA, Unigene28186\_Mf\_liverA, Unigene36622\_Mf\_liverA, NM\_010378, Unigene33138\_Mf\_liverA, Unigene16463\_Mf\_liverA, Unigene39886\_Mf\_liverA, CL33.Contig4\_Mf\_liverA, Unigene15064\_Mf\_liverA, NM\_172723, Unigene34124\_Mf\_liverA, Unigene7510\_Mf\_liverA, Unigene24252\_Mf\_liverA, CL4757.Contig1\_Mf\_liverA, NR\_004446, Unigene36765\_Mf\_liverA, NM\_013866, Unigene4557\_Mf\_liverA, Unigene35431\_Mf\_liverA, Unigene15703\_Mf\_liverA, Unigene30495\_Mf\_liverA, Unigene2\_Mf\_liverA, Unigene28331\_Mf\_liverA, CL33.Contig3\_Mf\_liverA, NM\_010233, Unigene37084\_Mf\_liverA, Unigene13379\_Mf\_liverA, Unigene25594\_Mf\_liverA, Unigene779\_Mf\_liverA, Unigene14050\_Mf\_liverA, Unigene21857\_Mf\_liverA, Unigene13419\_Mf\_liverA, Unigene8054\_Mf\_liverA, CL2384.Contig1\_Mf\_liverA, Unigene37454\_Mf\_liverA, Unigene743\_Mf\_liverA, NM\_053214, Unigene36417\_Mf\_liverA, Unigene25091\_Mf\_liverA, NM\_177093, Unigene25047\_Mf\_liverA, Unigene14907\_Mf\_liverA, Unigene7476\_Mf\_liverA, CL4332.Contig1\_Mf\_liverA, Unigene25976\_Mf\_liverA, Unigene30261\_Mf\_liverA, Unigene9475\_Mf\_liverA, NM\_145942, Unigene27895\_Mf\_liverA, Unigene36112\_Mf\_liverA, Unigene36641\_Mf\_liverA, Unigene13945\_Mf\_liverA, Unigene139\_Mf\_liverA, Unigene26941\_Mf\_liverA, Unigene995\_Mf\_liverA, Unigene34123\_Mf\_liverA, Unigene10756\_Mf\_liverA, CL3669.Contig1\_Mf\_liverA, Unigene27249\_Mf\_liverA, Unigene43515\_Mf\_liverA, Unigene11097\_Mf\_liverA, NM\_134156, Unigene4720\_Mf\_liverA, Unigene37389\_Mf\_liverA, Unigene27248\_Mf\_liverA, Unigene29253\_Mf\_liverA, Unigene34010\_Mf\_liverA, Unigene152\_Mf\_liverA, Unigene31852\_Mf\_liverA, Unigene5456\_Mf\_liverA, Unigene37076\_Mf\_liverA, Unigene2678\_Mf\_liverA, Unigene1280\_Mf\_liverA, Unigene30288\_Mf\_liverA, CL1738.Contig1\_Mf\_liverA, CL5576.Contig1\_Mf\_liverA, CL3339.Contig1\_Mf\_liverA, Unigene2746\_Mf\_liverA, Unigene5236\_Mf\_liverA, Unigene28021\_Mf\_liverA, Unigene45904\_Mf\_liverA, Unigene37433\_Mf\_liverA, CL1442.Contig1\_Mf\_liverA, CL5528.Contig1\_Mf\_liverA, CL425.Contig1\_Mf\_liverA, CL2797.Contig2\_Mf\_liverA, Unigene37243\_Mf\_liverA, Unigene30587\_Mf\_liverA, Unigene5693\_Mf\_liverA, Unigene39970\_Mf\_liverA, Unigene13271\_Mf\_liverA, CL3393.Contig1\_Mf\_liverA, Unigene19821\_Mf\_liverA, CL4057.Contig1\_Mf\_liverA, Unigene29876\_Mf\_liverA, CL3800.Contig1\_Mf\_liverA, Unigene25080\_Mf\_liverA, Unigene28687\_Mf\_liverA, CL1988.Contig3\_Mf\_liverA, Unigene29885\_Mf\_liverA, CL3055.Contig1\_Mf\_liverA, NM\_028785, NM\_009609, CL1810.Contig1\_Mf\_liverA, CL4033.Contig1\_Mf\_liverA, Unigene24547\_Mf\_liverA, CL3519.Contig1\_Mf\_liverA, Unigene8033\_Mf\_liverA, CL1493.Contig1\_Mf\_liverA, Unigene24801\_Mf\_liverA, CL3685.Contig1\_Mf\_liverA, Unigene39231\_Mf\_liverA, Unigene4909\_Mf\_liverA, Unigene34197\_Mf\_liverA, Unigene24471\_Mf\_liverA, NM\_026823, Unigene35168\_Mf\_liverA, Unigene15888\_Mf\_liverA, NM\_010162, Unigene15529\_Mf\_liverA, Unigene37262\_Mf\_liverA, Unigene12908\_Mf\_liverA, NM\_010469, CL2251.Contig1\_Mf\_liverA, CL4490.Contig2\_Mf\_liverA, CL5293.Contig1\_Mf\_liverA, Unigene25595\_Mf\_liverA, Unigene14263\_Mf\_liverA, Unigene14286\_Mf\_liverA, NM\_016861, Unigene39011\_Mf\_liverA, Unigene386\_Mf\_liverA, Unigene26250\_Mf\_liverA, Unigene30795\_Mf\_liverA, NM\_024452, Unigene20432\_Mf\_liverA, Unigene48460\_Mf\_liverA, NM\_011580, Unigene35046\_Mf\_liverA, Unigene28459\_Mf\_liverA, Unigene37245\_Mf\_liverA, Unigene16671\_Mf\_liverA, Unigene21693\_Mf\_liverA, Unigene13535\_Mf\_liverA, Unigene13772\_Mf\_liverA, Unigene29302\_Mf\_liverA, Unigene33428\_Mf\_liverA, Unigene37574\_Mf\_liverA, Unigene36176\_Mf\_liverA, Unigene36699\_Mf\_liverA, Unigene7968\_Mf\_liverA, Unigene18340\_Mf\_liverA, NM\_001164598, Unigene30429\_Mf\_liverA, Unigene30412\_Mf\_liverA, Unigene4983\_Mf\_liverA, Unigene38317\_Mf\_liverA, Unigene5512\_Mf\_liverA, CL373.Contig7\_Mf\_liverA, Unigene42855\_Mf\_liverA, Unigene29889\_Mf\_liverA, Unigene2939\_Mf\_liverA, Unigene36728\_Mf\_liverA, Unigene9466\_Mf\_liverA, Unigene35884\_Mf\_liverA, NM\_020559, CL5640.Contig1\_Mf\_liverA, Unigene28142\_Mf\_liverA, CL3835.Contig2\_Mf\_liverA, NM\_145824, Unigene30356\_Mf\_liverA, Unigene38124\_Mf\_liverA, Unigene46870\_Mf\_liverA, CL4336.Contig3\_Mf\_liverA, Unigene8473\_Mf\_liverA, Unigene29008\_Mf\_liverA, Unigene19049\_Mf\_liverA, Unigene33508\_Mf\_liverA, CL5358.Contig1\_Mf\_liverA, Unigene31263\_Mf\_liverA, NM\_011072, Unigene21466\_Mf\_liverA, Unigene7969\_Mf\_liverA, Unigene30303\_Mf\_liverA, NM\_173753, Unigene34810\_Mf\_liverA, Unigene27420\_Mf\_liverA, CL6038.Contig2\_Mf\_liverA, Unigene13307\_Mf\_liverA, Unigene37999\_Mf\_liverA, Unigene40020\_Mf\_liverA, Unigene37904\_Mf\_liverA, CL3483.Contig1\_Mf\_liverA, Unigene34789\_Mf\_liverA, CL4141.Contig1\_Mf\_liverA, Unigene25057\_Mf\_liverA, NM\_145419, CL5978.Contig3\_Mf\_liverA, CL5191.Contig2\_Mf\_liverA, Unigene13658\_Mf\_liverA, NM\_175260, Unigene4686\_Mf\_liverA, Unigene11\_Mf\_liverA, Unigene11544\_Mf\_liverA, NM\_018780, Unigene18117\_Mf\_liverA, CL1493.Contig2\_Mf\_liverA, CL2117.Contig1\_Mf\_liverA, CL3565.Contig1\_Mf\_liverA, Unigene36988\_Mf\_liverA, Unigene28094\_Mf\_liverA, Unigene40824\_Mf\_liverA, CL2574.Contig1\_Mf\_liverA, Unigene5941\_Mf\_liverA, Unigene38104\_Mf\_liverA, Unigene23328\_Mf\_liverA, Unigene25462\_Mf\_liverA, Unigene38065\_Mf\_liverA, Unigene16684\_Mf\_liverA, Unigene36626\_Mf\_liverA, NM\_001029934, NM\_029562, Unigene28822\_Mf\_liverA, Unigene36691\_Mf\_liverA, NM\_019703, Unigene31988\_Mf\_liverA, Unigene19297\_Mf\_liverA, CL2326.Contig1\_Mf\_liverA, Unigene151\_Mf\_liverA, CL848.Contig2\_Mf\_liverA, Unigene23870\_Mf\_liverA, NM\_008808, Unigene4768\_Mf\_liverA, Unigene10774\_Mf\_liverA, CL2131.Contig4\_Mf\_liverA, Unigene25662\_Mf\_liverA, CL3198.Contig1\_Mf\_liverA, Unigene37460\_Mf\_liverA, Unigene31517\_Mf\_liverA, Unigene31231\_Mf\_liverA, Unigene36628\_Mf\_liverA, NM\_007811, Unigene24323\_Mf\_liverA, NM\_008538, NM\_011305, NM\_008610, Unigene35859\_Mf\_liverA, CL3816.Contig1\_Mf\_liverA, Unigene36631\_Mf\_liverA, Unigene4510\_Mf\_liverA, CL3803.Contig2\_Mf\_liverA, Unigene24284\_Mf\_liverA, Unigene26873\_Mf\_liverA, Unigene27260\_Mf\_liverA, CL3268.Contig1\_Mf\_liverA, Unigene36845\_Mf\_liverA, Unigene37729\_Mf\_liverA, NM\_010359, Unigene29940\_Mf\_liverA, CL795.Contig1\_Mf\_liverA, Unigene42812\_Mf\_liverA, Unigene5509\_Mf\_liverA, Unigene5325\_Mf\_liverA, Unigene1205\_Mf\_liverA, Unigene13841\_Mf\_liverA, CL1352.Contig1\_Mf\_liverA, CL6039.Contig1\_Mf\_liverA, Unigene24804\_Mf\_liverA, Unigene31206\_Mf\_liverA, NM\_011132, Unigene32412\_Mf\_liverA, Unigene17569\_Mf\_liverA, Unigene40924\_Mf\_liverA, NM\_009128, NM\_175472, Unigene7412\_Mf\_liverA, CL2240.Contig1\_Mf\_liverA, Unigene5783\_Mf\_liverA, Unigene22980\_Mf\_liverA, Unigene38339\_Mf\_liverA, Unigene15474\_Mf\_liverA, CL1125.Contig1\_Mf\_liverA, Unigene29510\_Mf\_liverA, NM\_010579, Unigene15681\_Mf\_liverA, Unigene7350\_Mf\_liverA, Unigene19083\_Mf\_liverA, Unigene673\_Mf\_liverA, Unigene4703\_Mf\_liverA, Unigene37096\_Mf\_liverA, Unigene5165\_Mf\_liverA, Unigene30707\_Mf\_liverA, NM\_133838, Unigene7674\_Mf\_liverA, CL3002.Contig1\_Mf\_liverA, NM\_008871, Unigene15058\_Mf\_liverA, Unigene35491\_Mf\_liverA, NM\_029872, Unigene5906\_Mf\_liverA, Unigene34035\_Mf\_liverA, CL5688.Contig1\_Mf\_liverA, NM\_033444, CL2791.Contig1\_Mf\_liverA, Unigene35269\_Mf\_liverA, Unigene10135\_Mf\_liverA, Unigene22330\_Mf\_liverA, Unigene5954\_Mf\_liverA, NM\_009883, CL4127.Contig1\_Mf\_liverA, Unigene33526\_Mf\_liverA, Unigene1327\_Mf\_liverA, CL1190.Contig3\_Mf\_liverA, Unigene8740\_Mf\_liverA, CL5978.Contig2\_Mf\_liverA, NM\_008776, Unigene36177\_Mf\_liverA, Unigene24566\_Mf\_liverA, NM\_007478, Unigene15170\_Mf\_liverA, Unigene4604\_Mf\_liverA, NM\_011418, Unigene560\_Mf\_liverA, Unigene24477\_Mf\_liverA, Unigene30731\_Mf\_liverA, Unigene30983\_Mf\_liverA, Unigene36418\_Mf\_liverA, Unigene550\_Mf\_liverA, Unigene38311\_Mf\_liverA, CL2355.Contig1\_Mf\_liverA, Unigene36910\_Mf\_liverA, Unigene28314\_Mf\_liverA, Unigene37153\_Mf\_liverA, Unigene21562\_Mf\_liverA, Unigene15592\_Mf\_liverA, NM\_015767, Unigene37711\_Mf\_liverA, Unigene41583\_Mf\_liverA, Unigene5418\_Mf\_liverA, Unigene2195\_Mf\_liverA, Unigene93\_Mf\_liverA, Unigene30892\_Mf\_liverA, Unigene803\_Mf\_liverA, CL3911.Contig2\_Mf\_liverA, Unigene34866\_Mf\_liverA, NM\_144848, Unigene31885\_Mf\_liverA, NM\_001033481, Unigene4681\_Mf\_liverA, CL548.Contig1\_Mf\_liverA, NM\_007392, Unigene25787\_Mf\_liverA, Unigene24506\_Mf\_liverA, NM\_009450, Unigene34609\_Mf\_liverA, Unigene25721\_Mf\_liverA, NM\_009592, CL3669.Contig2\_Mf\_liverA, Unigene5852\_Mf\_liverA, Unigene36172\_Mf\_liverA, Unigene37334\_Mf\_liverA, NM\_018815, Unigene36851\_Mf\_liverA, Unigene14665\_Mf\_liverA, NM\_080638, Unigene7936\_Mf\_liverA, Unigene29424\_Mf\_liverA, Unigene26053\_Mf\_liverA, CL4105.Contig1\_Mf\_liverA, Unigene34218\_Mf\_liverA, CL4577.Contig1\_Mf\_liverA, Unigene25090\_Mf\_liverA, CL238.Contig1\_Mf\_liverA, CL2423.Contig1\_Mf\_liverA, CL5560.Contig1\_Mf\_liverA, Unigene22575\_Mf\_liverA, Unigene5248\_Mf\_liverA, Unigene785\_Mf\_liverA, Unigene26422\_Mf\_liverA, Unigene29334\_Mf\_liverA, NM\_010877, NM\_021273, Unigene38919\_Mf\_liverA, CL5586.Contig1\_Mf\_liverA, Unigene21255\_Mf\_liverA, Unigene34341\_Mf\_liverA, Unigene30142\_Mf\_liverA, Unigene35816\_Mf\_liverA, Unigene13940\_Mf\_liverA, NM\_145474, Unigene33054\_Mf\_liverA, NM\_009776, Unigene15205\_Mf\_liverA, Unigene5138\_Mf\_liverA, NM\_153193, Unigene13266\_Mf\_liverA, Unigene5330\_Mf\_liverA, NM\_177320, Unigene653\_Mf\_liverA, Unigene38657\_Mf\_liverA, Unigene23869\_Mf\_liverA, Unigene9150\_Mf\_liverA, Unigene5598\_Mf\_liverA, Unigene13363\_Mf\_liverA, CL2855.Contig2\_Mf\_liverA, Unigene36175\_Mf\_liverA, Unigene28226\_Mf\_liverA, Unigene44317\_Mf\_liverA, Unigene37278\_Mf\_liverA, Unigene2919\_Mf\_liverA, Unigene21561\_Mf\_liverA, Unigene1479\_Mf\_liverA, Unigene4922\_Mf\_liverA, Unigene14637\_Mf\_liverA, Unigene44882\_Mf\_liverA, CL2159.Contig2\_Mf\_liverA, NM\_153505, Unigene37662\_Mf\_liverA, Unigene9990\_Mf\_liverA, NM\_001104531, NM\_153055, Unigene4944\_Mf\_liverA, Unigene23158\_Mf\_liverA, CL3055.Contig2\_Mf\_liverA, Unigene37575\_Mf\_liverA, Unigene25398\_Mf\_liverA, Unigene27547\_Mf\_liverA, CL1736.Contig2\_Mf\_liverA, Unigene5180\_Mf\_liverA, Unigene12909\_Mf\_liverA, Unigene4723\_Mf\_liverA, Unigene32332\_Mf\_liverA, Unigene37470\_Mf\_liverA, CL5254.Contig1\_Mf\_liverA, Unigene37542\_Mf\_liverA, NM\_009713, NM\_009087, Unigene35169\_Mf\_liverA, Unigene21256\_Mf\_liverA, NR\_003552, Unigene16465\_Mf\_liverA, Unigene32059\_Mf\_liverA, Unigene5472\_Mf\_liverA, Unigene26515\_Mf\_liverA, Unigene31251\_Mf\_liverA, NM\_011503, Unigene40610\_Mf\_liverA, NM\_172121, CL186.Contig3\_Mf\_liverA, CL4736.Contig1\_Mf\_liverA, Unigene14916\_Mf\_liverA, Unigene35858\_Mf\_liverA, Unigene30808\_Mf\_liverA, Unigene29231\_Mf\_liverA, Unigene37442\_Mf\_liverA, Unigene50250\_Mf\_liverA, Unigene1440\_Mf\_liverA, Unigene35958\_Mf\_liverA, Unigene43037\_Mf\_liverA, Unigene13525\_Mf\_liverA, Unigene24395\_Mf\_liverA, Unigene5815\_Mf\_liverA, Unigene1129\_Mf\_liverA, Unigene33168\_Mf\_liverA, Unigene27483\_Mf\_liverA, Unigene28662\_Mf\_liverA, CL3738.Contig1\_Mf\_liverA, CL2439.Contig1\_Mf\_liverA, Unigene4781\_Mf\_liverA, Unigene46615\_Mf\_liverA, Unigene35935\_Mf\_liverA, Unigene29426\_Mf\_liverA, Unigene30494\_Mf\_liverA, CL5049.Contig2\_Mf\_liverA, Unigene34446\_Mf\_liverA, Unigene13683\_Mf\_liverA, Unigene9698\_Mf\_liverA, Unigene24755\_Mf\_liverA, Unigene28499\_Mf\_liverA, Unigene30585\_Mf\_liverA, Unigene36420\_Mf\_liverA, Unigene542\_Mf\_liverA, Unigene38593\_Mf\_liverA, Unigene24758\_Mf\_liverA, Unigene23271\_Mf\_liverA, Unigene25092\_Mf\_liverA, CL5828.Contig2\_Mf\_liverA, Unigene28527\_Mf\_liverA, Unigene35039\_Mf\_liverA, Unigene29938\_Mf\_liverA, CL2625.Contig2\_Mf\_liverA, CL725.Contig1\_Mf\_liverA, CL5293.Contig2\_Mf\_liverA, CL1165.Contig2\_Mf\_liverA, Unigene10335\_Mf\_liverA, Unigene34962\_Mf\_liverA, Unigene14212\_Mf\_liverA, CL1165.Contig4\_Mf\_liverA, CL4669.Contig1\_Mf\_liverA, NM\_028222, Unigene29558\_Mf\_liverA, Unigene23185\_Mf\_liverA, CL3692.Contig2\_Mf\_liverA, Unigene26083\_Mf\_liverA, NM\_013863, Unigene281\_Mf\_liverA, Unigene10313\_Mf\_liverA, Unigene29985\_Mf\_liverA, Unigene5687\_Mf\_liverA, CL3104.Contig1\_Mf\_liverA, Unigene36593\_Mf\_liverA, Unigene5037\_Mf\_liverA, Unigene5422\_Mf\_liverA, NM\_146016, Unigene36673\_Mf\_liverA, NM\_181517, CL5698.Contig1\_Mf\_liverA, Unigene31392\_Mf\_liverA, NM\_023256, CL591.Contig1\_Mf\_liverA, NM\_009636, Unigene4556\_Mf\_liverA, Unigene38237\_Mf\_liverA, NM\_007763, Unigene26055\_Mf\_liverA, CL139.Contig2\_Mf\_liverA, Unigene29399\_Mf\_liverA, CL2520.Contig1\_Mf\_liverA, Unigene38015\_Mf\_liverA, NM\_007896, Unigene24713\_Mf\_liverA, Unigene42975\_Mf\_liverA, NM\_144907, CL1828.Contig1\_Mf\_liverA, Unigene29308\_Mf\_liverA, CL1362.Contig1\_Mf\_liverA, CL523.Contig1\_Mf\_liverA, Unigene15914\_Mf\_liverA, Unigene5194\_Mf\_liverA, CL1063.Contig1\_Mf\_liverA, CL482.Contig1\_Mf\_liverA, Unigene29405\_Mf\_liverA, Unigene32882\_Mf\_liverA, Unigene21263\_Mf\_liverA, Unigene7619\_Mf\_liverA, Unigene2633\_Mf\_liverA, Unigene30878\_Mf\_liverA, Unigene40289\_Mf\_liverA, Unigene21013\_Mf\_liverA, Unigene15656\_Mf\_liverA, Unigene34234\_Mf\_liverA, Unigene38331\_Mf\_liverA, Unigene5204\_Mf\_liverA, Unigene37099\_Mf\_liverA, CL5001.Contig2\_Mf\_liverA, Unigene25070\_Mf\_liverA, Unigene24379\_Mf\_liverA, Unigene37263\_Mf\_liverA, Unigene36853\_Mf\_liverA, Unigene23273\_Mf\_liverA, NM\_009896, CL1119.Contig1\_Mf\_liverA, Unigene26194\_Mf\_liverA, Unigene41158\_Mf\_liverA, Unigene25596\_Mf\_liverA, CL3778.Contig2\_Mf\_liverA, NM\_010763, Unigene3174\_Mf\_liverA, Unigene22978\_Mf\_liverA, Unigene25046\_Mf\_liverA, Unigene2745\_Mf\_liverA, NM\_001024205, CL4076.Contig1\_Mf\_liverA, Unigene1162\_Mf\_liverA, CL787.Contig1\_Mf\_liverA, Unigene25524\_Mf\_liverA, CL4847.Contig1\_Mf\_liverA, NM\_001162917, CL3196.Contig2\_Mf\_liverA, Unigene38406\_Mf\_liverA, CL3750.Contig2\_Mf\_liverA, CL4722.Contig1\_Mf\_liverA, Unigene33459\_Mf\_liverA, Unigene26398\_Mf\_liverA, Unigene25012\_Mf\_liverA, Unigene34258\_Mf\_liverA, Unigene5632\_Mf\_liverA, NM\_001001806, CL479.Contig1\_Mf\_liverA, Unigene27419\_Mf\_liverA, Unigene14715\_Mf\_liverA, Unigene14582\_Mf\_liverA, Unigene24503\_Mf\_liverA, NM\_134059, Unigene37180\_Mf\_liverA, Unigene4523\_Mf\_liverA, CL2722.Contig1\_Mf\_liverA, Unigene27438\_Mf\_liverA, CL5631.Contig1\_Mf\_liverA, Unigene31427\_Mf\_liverA, Unigene14896\_Mf\_liverA, Unigene30584\_Mf\_liverA, Unigene19885\_Mf\_liverA, Unigene431\_Mf\_liverA, Unigene36510\_Mf\_liverA, NM\_009091, Unigene44992\_Mf\_liverA, Unigene10820\_Mf\_liverA, NM\_010391, NM\_008889, Unigene21317\_Mf\_liverA, NM\_010227, Unigene30493\_Mf\_liverA, Unigene8132\_Mf\_liverA, NM\_019717, Unigene27082\_Mf\_liverA, Unigene37178\_Mf\_liverA, Unigene32058\_Mf\_liverA, Unigene5712\_Mf\_liverA, Unigene584\_Mf\_liverA, CL2962.Contig1\_Mf\_liverA, Unigene23055\_Mf\_liverA, CL442.Contig2\_Mf\_liverA, Unigene26214\_Mf\_liverA, NM\_033374, CL2855.Contig1\_Mf\_liverA, CL4411.Contig4\_Mf\_liverA, NM\_001025388, Unigene13233\_Mf\_liverA, NM\_011099, Unigene5648\_Mf\_liverA, NM\_009447, NM\_001100182, CL1537.Contig1\_Mf\_liverA, NM\_010481, Unigene392\_Mf\_liverA, CL4086.Contig1\_Mf\_liverA, CL507.Contig1\_Mf\_liverA, Unigene23082\_Mf\_liverA, Unigene5360\_Mf\_liverA, Unigene15318\_Mf\_liverA, NM\_009178, Unigene13414\_Mf\_liverA, NM\_008293, Unigene1130\_Mf\_liverA, Unigene30154\_Mf\_liverA, Unigene15982\_Mf\_liverA, Unigene38208\_Mf\_liverA, CL4162.Contig1\_Mf\_liverA, Unigene35237\_Mf\_liverA, CL5993.Contig3\_Mf\_liverA, Unigene37259\_Mf\_liverA, Unigene12907\_Mf\_liverA, Unigene5260\_Mf\_liverA, Unigene15588\_Mf\_liverA, Unigene23013\_Mf\_liverA, CL4583.Contig2\_Mf\_liverA, Unigene36414\_Mf\_liverA, Unigene13894\_Mf\_liverA, Unigene5175\_Mf\_liverA, Unigene34219\_Mf\_liverA, Unigene33880\_Mf\_liverA, Unigene36328\_Mf\_liverA, CL1222.Contig1\_Mf\_liverA, CL2327.Contig1\_Mf\_liverA, CL6039.Contig2\_Mf\_liverA, Unigene35037\_Mf\_liverA, Unigene36710\_Mf\_liverA, Unigene5940\_Mf\_liverA, NM\_007393, Unigene28731\_Mf\_liverA, NM\_053072, Unigene1212\_Mf\_liverA, CL3549.Contig1\_Mf\_liverA, NM\_009706, Unigene24085\_Mf\_liverA, CL4106.Contig1\_Mf\_liverA, CL3750.Contig1\_Mf\_liverA, CL1588.Contig3\_Mf\_liverA, Unigene8016\_Mf\_liverA, Unigene18796\_Mf\_liverA, NM\_009448, CL5307.Contig1\_Mf\_liverA, Unigene31730\_Mf\_liverA, Unigene51055\_Mf\_liverA, CL4432.Contig2\_Mf\_liverA, Unigene33525\_Mf\_liverA, Unigene4540\_Mf\_liverA, Unigene24204\_Mf\_liverA, Unigene37310\_Mf\_liverA, Unigene9406\_Mf\_liverA, Unigene27803\_Mf\_liverA, Unigene15026\_Mf\_liverA, Unigene1221\_Mf\_liverA, CL1588.Contig1\_Mf\_liverA, NM\_017379, Unigene39875\_Mf\_liverA, NM\_001081274, Unigene25238\_Mf\_liverA, Unigene26309\_Mf\_liverA, Unigene17632\_Mf\_liverA, Unigene5745\_Mf\_liverA, Unigene12889\_Mf\_liverA, Unigene38514\_Mf\_liverA, Unigene665\_Mf\_liverA, Unigene35678\_Mf\_liverA, CL4434.Contig1\_Mf\_liverA, Unigene5147\_Mf\_liverA, Unigene8560\_Mf\_liverA, Unigene13396\_Mf\_liverA, Unigene41419\_Mf\_liverA, Unigene21359\_Mf\_liverA, Unigene36487\_Mf\_liverA, Unigene37474\_Mf\_liverA, Unigene16891\_Mf\_liverA, Unigene7733\_Mf\_liverA, Unigene37819\_Mf\_liverA, Unigene5886\_Mf\_liverA, Unigene27274\_Mf\_liverA, Unigene15493\_Mf\_liverA |
| cell-cell contact zone | Unigene27082\_Mf\_liverA, Unigene37278\_Mf\_liverA |
| outer membrane | CL1810.Contig1\_Mf\_liverA, CL3104.Contig1\_Mf\_liverA, Unigene995\_Mf\_liverA, Unigene27082\_Mf\_liverA, CL5631.Contig1\_Mf\_liverA, Unigene25462\_Mf\_liverA, Unigene4781\_Mf\_liverA |
| acrosomal vesicle | CL3816.Contig1\_Mf\_liverA, Unigene35958\_Mf\_liverA, Unigene28331\_Mf\_liverA, Unigene26941\_Mf\_liverA |
| intracellular | NM\_176843, Unigene34656\_Mf\_liverA, NM\_009898, Unigene7612\_Mf\_liverA, Unigene7950\_Mf\_liverA, Unigene28186\_Mf\_liverA, Unigene36622\_Mf\_liverA, NM\_010378, Unigene33138\_Mf\_liverA, Unigene16463\_Mf\_liverA, Unigene39886\_Mf\_liverA, CL33.Contig4\_Mf\_liverA, Unigene15064\_Mf\_liverA, NM\_172723, Unigene34124\_Mf\_liverA, Unigene7510\_Mf\_liverA, Unigene24252\_Mf\_liverA, CL4757.Contig1\_Mf\_liverA, NR\_004446, Unigene36765\_Mf\_liverA, NM\_013866, Unigene4557\_Mf\_liverA, Unigene35431\_Mf\_liverA, Unigene15703\_Mf\_liverA, Unigene30495\_Mf\_liverA, Unigene2\_Mf\_liverA, Unigene28331\_Mf\_liverA, CL33.Contig3\_Mf\_liverA, NM\_010233, Unigene37084\_Mf\_liverA, Unigene13379\_Mf\_liverA, Unigene25594\_Mf\_liverA, Unigene779\_Mf\_liverA, Unigene14050\_Mf\_liverA, Unigene21857\_Mf\_liverA, Unigene13419\_Mf\_liverA, Unigene8054\_Mf\_liverA, CL2384.Contig1\_Mf\_liverA, Unigene37454\_Mf\_liverA, Unigene743\_Mf\_liverA, NM\_053214, Unigene36417\_Mf\_liverA, Unigene25091\_Mf\_liverA, NM\_177093, Unigene25047\_Mf\_liverA, Unigene14907\_Mf\_liverA, Unigene7476\_Mf\_liverA, CL4332.Contig1\_Mf\_liverA, Unigene25976\_Mf\_liverA, Unigene30261\_Mf\_liverA, Unigene9475\_Mf\_liverA, NM\_145942, Unigene27895\_Mf\_liverA, Unigene36112\_Mf\_liverA, Unigene36641\_Mf\_liverA, Unigene13945\_Mf\_liverA, Unigene139\_Mf\_liverA, Unigene26941\_Mf\_liverA, Unigene995\_Mf\_liverA, Unigene34123\_Mf\_liverA, Unigene10756\_Mf\_liverA, CL3669.Contig1\_Mf\_liverA, Unigene27249\_Mf\_liverA, Unigene43515\_Mf\_liverA, Unigene11097\_Mf\_liverA, NM\_134156, Unigene4720\_Mf\_liverA, Unigene37389\_Mf\_liverA, Unigene27248\_Mf\_liverA, Unigene29253\_Mf\_liverA, Unigene34010\_Mf\_liverA, Unigene152\_Mf\_liverA, Unigene31852\_Mf\_liverA, Unigene5456\_Mf\_liverA, Unigene37076\_Mf\_liverA, Unigene2678\_Mf\_liverA, Unigene1280\_Mf\_liverA, Unigene30288\_Mf\_liverA, CL1738.Contig1\_Mf\_liverA, CL5576.Contig1\_Mf\_liverA, CL3339.Contig1\_Mf\_liverA, Unigene2746\_Mf\_liverA, Unigene4593\_Mf\_liverA, Unigene5236\_Mf\_liverA, Unigene28021\_Mf\_liverA, Unigene45904\_Mf\_liverA, Unigene37433\_Mf\_liverA, CL1442.Contig1\_Mf\_liverA, CL5528.Contig1\_Mf\_liverA, CL425.Contig1\_Mf\_liverA, CL2797.Contig2\_Mf\_liverA, Unigene37243\_Mf\_liverA, Unigene30587\_Mf\_liverA, Unigene5693\_Mf\_liverA, Unigene39970\_Mf\_liverA, Unigene13271\_Mf\_liverA, CL3393.Contig1\_Mf\_liverA, Unigene19821\_Mf\_liverA, CL4057.Contig1\_Mf\_liverA, Unigene29876\_Mf\_liverA, CL3800.Contig1\_Mf\_liverA, Unigene25080\_Mf\_liverA, Unigene28687\_Mf\_liverA, CL1988.Contig3\_Mf\_liverA, Unigene29885\_Mf\_liverA, CL3055.Contig1\_Mf\_liverA, NM\_028785, NM\_009609, CL1810.Contig1\_Mf\_liverA, CL4033.Contig1\_Mf\_liverA, Unigene24547\_Mf\_liverA, CL3519.Contig1\_Mf\_liverA, Unigene8033\_Mf\_liverA, CL1493.Contig1\_Mf\_liverA, Unigene24801\_Mf\_liverA, CL3685.Contig1\_Mf\_liverA, Unigene39231\_Mf\_liverA, Unigene4909\_Mf\_liverA, Unigene34197\_Mf\_liverA, Unigene24471\_Mf\_liverA, NM\_026823, Unigene35168\_Mf\_liverA, Unigene15888\_Mf\_liverA, NM\_010162, Unigene15529\_Mf\_liverA, Unigene37262\_Mf\_liverA, Unigene12908\_Mf\_liverA, NM\_010469, CL2251.Contig1\_Mf\_liverA, CL4490.Contig2\_Mf\_liverA, CL5293.Contig1\_Mf\_liverA, Unigene25595\_Mf\_liverA, Unigene14263\_Mf\_liverA, Unigene14286\_Mf\_liverA, NM\_016861, Unigene39011\_Mf\_liverA, Unigene386\_Mf\_liverA, Unigene26250\_Mf\_liverA, Unigene30795\_Mf\_liverA, NM\_024452, Unigene20432\_Mf\_liverA, Unigene48460\_Mf\_liverA, NM\_011580, Unigene35046\_Mf\_liverA, Unigene28459\_Mf\_liverA, Unigene37245\_Mf\_liverA, Unigene16671\_Mf\_liverA, Unigene21693\_Mf\_liverA, Unigene13535\_Mf\_liverA, Unigene13772\_Mf\_liverA, Unigene29302\_Mf\_liverA, Unigene33428\_Mf\_liverA, Unigene37574\_Mf\_liverA, Unigene36176\_Mf\_liverA, Unigene36699\_Mf\_liverA, Unigene7968\_Mf\_liverA, Unigene18340\_Mf\_liverA, NM\_001164598, Unigene30429\_Mf\_liverA, Unigene30412\_Mf\_liverA, Unigene4983\_Mf\_liverA, Unigene38317\_Mf\_liverA, Unigene5512\_Mf\_liverA, CL373.Contig7\_Mf\_liverA, Unigene42855\_Mf\_liverA, Unigene29889\_Mf\_liverA, Unigene2939\_Mf\_liverA, Unigene36728\_Mf\_liverA, Unigene9466\_Mf\_liverA, Unigene35884\_Mf\_liverA, NM\_020559, CL5640.Contig1\_Mf\_liverA, Unigene28142\_Mf\_liverA, CL3835.Contig2\_Mf\_liverA, NM\_145824, Unigene30356\_Mf\_liverA, Unigene38124\_Mf\_liverA, Unigene46870\_Mf\_liverA, CL4336.Contig3\_Mf\_liverA, Unigene8473\_Mf\_liverA, Unigene29008\_Mf\_liverA, Unigene19049\_Mf\_liverA, Unigene33508\_Mf\_liverA, CL5358.Contig1\_Mf\_liverA, Unigene31263\_Mf\_liverA, NM\_011072, Unigene21466\_Mf\_liverA, Unigene7969\_Mf\_liverA, Unigene30303\_Mf\_liverA, NM\_173753, Unigene34810\_Mf\_liverA, Unigene27420\_Mf\_liverA, CL6038.Contig2\_Mf\_liverA, Unigene13307\_Mf\_liverA, Unigene37999\_Mf\_liverA, Unigene40020\_Mf\_liverA, Unigene37904\_Mf\_liverA, CL3483.Contig1\_Mf\_liverA, Unigene34789\_Mf\_liverA, CL4141.Contig1\_Mf\_liverA, Unigene25057\_Mf\_liverA, NM\_145419, CL5978.Contig3\_Mf\_liverA, CL5191.Contig2\_Mf\_liverA, Unigene13658\_Mf\_liverA, NM\_175260, Unigene4686\_Mf\_liverA, Unigene11\_Mf\_liverA, Unigene11544\_Mf\_liverA, NM\_018780, Unigene18117\_Mf\_liverA, CL1493.Contig2\_Mf\_liverA, CL2117.Contig1\_Mf\_liverA, CL3565.Contig1\_Mf\_liverA, Unigene36988\_Mf\_liverA, Unigene28094\_Mf\_liverA, Unigene40824\_Mf\_liverA, CL2574.Contig1\_Mf\_liverA, Unigene5941\_Mf\_liverA, Unigene38104\_Mf\_liverA, Unigene23328\_Mf\_liverA, Unigene25462\_Mf\_liverA, Unigene38065\_Mf\_liverA, Unigene16684\_Mf\_liverA, Unigene36626\_Mf\_liverA, NM\_001029934, NM\_029562, Unigene28822\_Mf\_liverA, Unigene36691\_Mf\_liverA, Unigene31988\_Mf\_liverA, NM\_019703, Unigene19297\_Mf\_liverA, CL2326.Contig1\_Mf\_liverA, Unigene151\_Mf\_liverA, CL848.Contig2\_Mf\_liverA, Unigene23870\_Mf\_liverA, NM\_008808, Unigene4768\_Mf\_liverA, Unigene10774\_Mf\_liverA, CL2131.Contig4\_Mf\_liverA, Unigene25662\_Mf\_liverA, CL3198.Contig1\_Mf\_liverA, Unigene37460\_Mf\_liverA, Unigene31517\_Mf\_liverA, Unigene31231\_Mf\_liverA, Unigene36628\_Mf\_liverA, NM\_007811, Unigene24323\_Mf\_liverA, NM\_008538, NM\_011305, NM\_008610, Unigene35859\_Mf\_liverA, CL3816.Contig1\_Mf\_liverA, Unigene36631\_Mf\_liverA, Unigene4510\_Mf\_liverA, CL3803.Contig2\_Mf\_liverA, Unigene24284\_Mf\_liverA, Unigene26873\_Mf\_liverA, Unigene27260\_Mf\_liverA, CL3268.Contig1\_Mf\_liverA, Unigene36845\_Mf\_liverA, Unigene14758\_Mf\_liverA, Unigene37729\_Mf\_liverA, NM\_010359, Unigene29940\_Mf\_liverA, CL795.Contig1\_Mf\_liverA, Unigene42812\_Mf\_liverA, Unigene5509\_Mf\_liverA, Unigene5325\_Mf\_liverA, Unigene1205\_Mf\_liverA, Unigene13841\_Mf\_liverA, CL1352.Contig1\_Mf\_liverA, CL6039.Contig1\_Mf\_liverA, Unigene24804\_Mf\_liverA, Unigene31206\_Mf\_liverA, NM\_011132, Unigene32412\_Mf\_liverA, Unigene17569\_Mf\_liverA, Unigene40924\_Mf\_liverA, NM\_009128, NM\_175472, Unigene7412\_Mf\_liverA, CL2240.Contig1\_Mf\_liverA, Unigene5783\_Mf\_liverA, Unigene22980\_Mf\_liverA, Unigene38339\_Mf\_liverA, Unigene15474\_Mf\_liverA, CL1125.Contig1\_Mf\_liverA, Unigene29510\_Mf\_liverA, NM\_010579, Unigene15681\_Mf\_liverA, Unigene7350\_Mf\_liverA, Unigene19083\_Mf\_liverA, Unigene673\_Mf\_liverA, Unigene4703\_Mf\_liverA, Unigene37096\_Mf\_liverA, Unigene5165\_Mf\_liverA, Unigene30707\_Mf\_liverA, NM\_133838, Unigene7674\_Mf\_liverA, CL3002.Contig1\_Mf\_liverA, NM\_008871, Unigene15058\_Mf\_liverA, Unigene35491\_Mf\_liverA, NM\_029872, Unigene37139\_Mf\_liverA, Unigene5906\_Mf\_liverA, Unigene34035\_Mf\_liverA, CL5688.Contig1\_Mf\_liverA, NM\_033444, CL2791.Contig1\_Mf\_liverA, Unigene35269\_Mf\_liverA, Unigene10135\_Mf\_liverA, Unigene22330\_Mf\_liverA, Unigene5954\_Mf\_liverA, NM\_009883, CL4127.Contig1\_Mf\_liverA, Unigene33526\_Mf\_liverA, Unigene1327\_Mf\_liverA, CL1190.Contig3\_Mf\_liverA, Unigene8740\_Mf\_liverA, CL5978.Contig2\_Mf\_liverA, NM\_008776, Unigene36177\_Mf\_liverA, Unigene24566\_Mf\_liverA, NM\_007478, Unigene15170\_Mf\_liverA, Unigene4604\_Mf\_liverA, NM\_011418, Unigene560\_Mf\_liverA, Unigene24477\_Mf\_liverA, Unigene30731\_Mf\_liverA, Unigene30983\_Mf\_liverA, Unigene36418\_Mf\_liverA, Unigene550\_Mf\_liverA, Unigene38311\_Mf\_liverA, CL2355.Contig1\_Mf\_liverA, Unigene36910\_Mf\_liverA, Unigene28314\_Mf\_liverA, Unigene37153\_Mf\_liverA, Unigene21562\_Mf\_liverA, Unigene15592\_Mf\_liverA, NM\_015767, Unigene37711\_Mf\_liverA, Unigene41583\_Mf\_liverA, Unigene5418\_Mf\_liverA, Unigene2195\_Mf\_liverA, Unigene93\_Mf\_liverA, Unigene30892\_Mf\_liverA, Unigene803\_Mf\_liverA, CL3911.Contig2\_Mf\_liverA, Unigene34866\_Mf\_liverA, Unigene29292\_Mf\_liverA, NM\_144848, Unigene31885\_Mf\_liverA, NM\_001033481, Unigene4681\_Mf\_liverA, CL548.Contig1\_Mf\_liverA, NM\_007392, Unigene25787\_Mf\_liverA, Unigene24506\_Mf\_liverA, NM\_009450, Unigene34609\_Mf\_liverA, Unigene25721\_Mf\_liverA, NM\_009592, CL3669.Contig2\_Mf\_liverA, Unigene5852\_Mf\_liverA, Unigene36172\_Mf\_liverA, Unigene37334\_Mf\_liverA, NM\_018815, Unigene36851\_Mf\_liverA, Unigene14665\_Mf\_liverA, NM\_080638, Unigene7936\_Mf\_liverA, Unigene29424\_Mf\_liverA, Unigene26053\_Mf\_liverA, CL4105.Contig1\_Mf\_liverA, Unigene34218\_Mf\_liverA, CL4577.Contig1\_Mf\_liverA, Unigene25090\_Mf\_liverA, CL238.Contig1\_Mf\_liverA, CL2423.Contig1\_Mf\_liverA, CL5560.Contig1\_Mf\_liverA, Unigene22575\_Mf\_liverA, Unigene5248\_Mf\_liverA, Unigene785\_Mf\_liverA, Unigene26422\_Mf\_liverA, Unigene29334\_Mf\_liverA, NM\_010877, NM\_021273, Unigene38919\_Mf\_liverA, CL5586.Contig1\_Mf\_liverA, Unigene21255\_Mf\_liverA, Unigene34341\_Mf\_liverA, Unigene30142\_Mf\_liverA, Unigene35816\_Mf\_liverA, Unigene13940\_Mf\_liverA, NM\_145474, Unigene33054\_Mf\_liverA, NM\_009776, Unigene15205\_Mf\_liverA, Unigene5138\_Mf\_liverA, NM\_153193, Unigene13266\_Mf\_liverA, Unigene5330\_Mf\_liverA, NM\_177320, Unigene34143\_Mf\_liverA, Unigene653\_Mf\_liverA, Unigene38657\_Mf\_liverA, Unigene23869\_Mf\_liverA, Unigene9150\_Mf\_liverA, Unigene5598\_Mf\_liverA, Unigene13363\_Mf\_liverA, CL2855.Contig2\_Mf\_liverA, Unigene36175\_Mf\_liverA, Unigene28226\_Mf\_liverA, Unigene44317\_Mf\_liverA, Unigene37278\_Mf\_liverA, Unigene2919\_Mf\_liverA, Unigene21561\_Mf\_liverA, Unigene1479\_Mf\_liverA, Unigene4922\_Mf\_liverA, Unigene14637\_Mf\_liverA, Unigene44882\_Mf\_liverA, CL2159.Contig2\_Mf\_liverA, NM\_153505, Unigene37662\_Mf\_liverA, Unigene31654\_Mf\_liverA, Unigene9990\_Mf\_liverA, NM\_001104531, NM\_153055, Unigene4944\_Mf\_liverA, Unigene23158\_Mf\_liverA, CL3055.Contig2\_Mf\_liverA, Unigene37575\_Mf\_liverA, Unigene25398\_Mf\_liverA, Unigene27547\_Mf\_liverA, CL1736.Contig2\_Mf\_liverA, Unigene5180\_Mf\_liverA, Unigene12909\_Mf\_liverA, Unigene4723\_Mf\_liverA, Unigene32332\_Mf\_liverA, Unigene37470\_Mf\_liverA, CL5254.Contig1\_Mf\_liverA, Unigene37542\_Mf\_liverA, NM\_009713, NM\_009087, Unigene35169\_Mf\_liverA, Unigene21256\_Mf\_liverA, NR\_003552, Unigene16465\_Mf\_liverA, Unigene32059\_Mf\_liverA, Unigene32202\_Mf\_liverA, Unigene5472\_Mf\_liverA, Unigene26515\_Mf\_liverA, Unigene31251\_Mf\_liverA, NM\_011503, Unigene40610\_Mf\_liverA, NM\_172121, CL186.Contig3\_Mf\_liverA, Unigene583\_Mf\_liverA, CL4736.Contig1\_Mf\_liverA, Unigene27026\_Mf\_liverA, Unigene14916\_Mf\_liverA, Unigene35858\_Mf\_liverA, Unigene30808\_Mf\_liverA, Unigene29231\_Mf\_liverA, Unigene37442\_Mf\_liverA, Unigene50250\_Mf\_liverA, Unigene1440\_Mf\_liverA, Unigene35958\_Mf\_liverA, Unigene43037\_Mf\_liverA, Unigene13525\_Mf\_liverA, Unigene24395\_Mf\_liverA, Unigene5815\_Mf\_liverA, Unigene1129\_Mf\_liverA, Unigene33168\_Mf\_liverA, Unigene27483\_Mf\_liverA, Unigene28662\_Mf\_liverA, CL3738.Contig1\_Mf\_liverA, CL2439.Contig1\_Mf\_liverA, Unigene4781\_Mf\_liverA, Unigene46615\_Mf\_liverA, Unigene35935\_Mf\_liverA, Unigene29426\_Mf\_liverA, Unigene30494\_Mf\_liverA, CL5049.Contig2\_Mf\_liverA, Unigene34446\_Mf\_liverA, Unigene13683\_Mf\_liverA, Unigene9698\_Mf\_liverA, Unigene24755\_Mf\_liverA, Unigene28499\_Mf\_liverA, Unigene30585\_Mf\_liverA, Unigene36420\_Mf\_liverA, Unigene542\_Mf\_liverA, Unigene38593\_Mf\_liverA, Unigene24758\_Mf\_liverA, Unigene23271\_Mf\_liverA, Unigene25092\_Mf\_liverA, CL5828.Contig2\_Mf\_liverA, Unigene28527\_Mf\_liverA, Unigene35039\_Mf\_liverA, Unigene29938\_Mf\_liverA, CL2625.Contig2\_Mf\_liverA, CL725.Contig1\_Mf\_liverA, CL5293.Contig2\_Mf\_liverA, CL1165.Contig2\_Mf\_liverA, Unigene10335\_Mf\_liverA, Unigene34962\_Mf\_liverA, Unigene14212\_Mf\_liverA, CL1165.Contig4\_Mf\_liverA, CL4669.Contig1\_Mf\_liverA, NM\_028222, Unigene29558\_Mf\_liverA, Unigene23185\_Mf\_liverA, CL3692.Contig2\_Mf\_liverA, Unigene26083\_Mf\_liverA, NM\_013863, Unigene281\_Mf\_liverA, Unigene10313\_Mf\_liverA, Unigene29985\_Mf\_liverA, Unigene5687\_Mf\_liverA, CL3104.Contig1\_Mf\_liverA, Unigene36593\_Mf\_liverA, Unigene5037\_Mf\_liverA, Unigene5422\_Mf\_liverA, NM\_146016, Unigene36673\_Mf\_liverA, NM\_181517, CL5698.Contig1\_Mf\_liverA, Unigene31392\_Mf\_liverA, NM\_023256, CL591.Contig1\_Mf\_liverA, NM\_009636, Unigene4556\_Mf\_liverA, Unigene38237\_Mf\_liverA, NM\_007763, Unigene26055\_Mf\_liverA, CL139.Contig2\_Mf\_liverA, Unigene29399\_Mf\_liverA, CL2520.Contig1\_Mf\_liverA, Unigene38015\_Mf\_liverA, NM\_007896, Unigene24713\_Mf\_liverA, Unigene42975\_Mf\_liverA, NM\_144907, CL1828.Contig1\_Mf\_liverA, Unigene29308\_Mf\_liverA, CL1362.Contig1\_Mf\_liverA, CL523.Contig1\_Mf\_liverA, Unigene15914\_Mf\_liverA, Unigene5194\_Mf\_liverA, CL1063.Contig1\_Mf\_liverA, CL482.Contig1\_Mf\_liverA, Unigene29405\_Mf\_liverA, Unigene32882\_Mf\_liverA, Unigene21263\_Mf\_liverA, Unigene7619\_Mf\_liverA, Unigene2633\_Mf\_liverA, Unigene30878\_Mf\_liverA, Unigene40289\_Mf\_liverA, Unigene21013\_Mf\_liverA, Unigene15656\_Mf\_liverA, Unigene34234\_Mf\_liverA, Unigene38331\_Mf\_liverA, Unigene5204\_Mf\_liverA, Unigene37099\_Mf\_liverA, CL5001.Contig2\_Mf\_liverA, Unigene25070\_Mf\_liverA, Unigene24379\_Mf\_liverA, Unigene37263\_Mf\_liverA, Unigene36853\_Mf\_liverA, Unigene23273\_Mf\_liverA, NM\_009896, CL1119.Contig1\_Mf\_liverA, Unigene26194\_Mf\_liverA, Unigene41158\_Mf\_liverA, Unigene25596\_Mf\_liverA, CL3778.Contig2\_Mf\_liverA, NM\_010763, Unigene3174\_Mf\_liverA, Unigene22978\_Mf\_liverA, Unigene25046\_Mf\_liverA, Unigene13918\_Mf\_liverA, Unigene2745\_Mf\_liverA, NM\_001024205, CL4076.Contig1\_Mf\_liverA, Unigene1162\_Mf\_liverA, CL787.Contig1\_Mf\_liverA, Unigene25524\_Mf\_liverA, CL4847.Contig1\_Mf\_liverA, NM\_001162917, CL3196.Contig2\_Mf\_liverA, Unigene38406\_Mf\_liverA, CL3750.Contig2\_Mf\_liverA, CL4722.Contig1\_Mf\_liverA, Unigene33459\_Mf\_liverA, Unigene26398\_Mf\_liverA, Unigene25012\_Mf\_liverA, Unigene34258\_Mf\_liverA, Unigene5632\_Mf\_liverA, NM\_001001806, CL479.Contig1\_Mf\_liverA, Unigene27419\_Mf\_liverA, Unigene14582\_Mf\_liverA, Unigene14715\_Mf\_liverA, Unigene24503\_Mf\_liverA, NM\_134059, Unigene37180\_Mf\_liverA, Unigene4523\_Mf\_liverA, Unigene27438\_Mf\_liverA, CL2722.Contig1\_Mf\_liverA, CL5631.Contig1\_Mf\_liverA, Unigene31427\_Mf\_liverA, Unigene14896\_Mf\_liverA, Unigene30584\_Mf\_liverA, Unigene431\_Mf\_liverA, Unigene19885\_Mf\_liverA, Unigene36510\_Mf\_liverA, NM\_009091, Unigene44992\_Mf\_liverA, Unigene10820\_Mf\_liverA, NM\_010391, NM\_008889, NM\_010227, Unigene21317\_Mf\_liverA, Unigene30493\_Mf\_liverA, Unigene8132\_Mf\_liverA, NM\_019717, Unigene32058\_Mf\_liverA, Unigene27082\_Mf\_liverA, Unigene37178\_Mf\_liverA, Unigene5712\_Mf\_liverA, Unigene584\_Mf\_liverA, CL2962.Contig1\_Mf\_liverA, Unigene23055\_Mf\_liverA, CL442.Contig2\_Mf\_liverA, Unigene26214\_Mf\_liverA, NM\_033374, CL2855.Contig1\_Mf\_liverA, CL4411.Contig4\_Mf\_liverA, NM\_001025388, Unigene13233\_Mf\_liverA, NM\_011099, Unigene5648\_Mf\_liverA, NM\_009447, NM\_001100182, CL1537.Contig1\_Mf\_liverA, NM\_010481, Unigene392\_Mf\_liverA, CL4086.Contig1\_Mf\_liverA, CL507.Contig1\_Mf\_liverA, Unigene23082\_Mf\_liverA, Unigene5360\_Mf\_liverA, Unigene15318\_Mf\_liverA, NM\_009178, Unigene13414\_Mf\_liverA, NM\_008293, Unigene1130\_Mf\_liverA, Unigene30154\_Mf\_liverA, Unigene15982\_Mf\_liverA, Unigene38208\_Mf\_liverA, CL4162.Contig1\_Mf\_liverA, Unigene35237\_Mf\_liverA, CL5993.Contig3\_Mf\_liverA, Unigene37259\_Mf\_liverA, Unigene12907\_Mf\_liverA, Unigene5260\_Mf\_liverA, Unigene15588\_Mf\_liverA, Unigene23013\_Mf\_liverA, CL4583.Contig2\_Mf\_liverA, Unigene36414\_Mf\_liverA, Unigene13894\_Mf\_liverA, Unigene5175\_Mf\_liverA, Unigene34219\_Mf\_liverA, Unigene33880\_Mf\_liverA, Unigene36328\_Mf\_liverA, CL1222.Contig1\_Mf\_liverA, CL2327.Contig1\_Mf\_liverA, CL2688.Contig1\_Mf\_liverA, CL6039.Contig2\_Mf\_liverA, Unigene35037\_Mf\_liverA, Unigene36710\_Mf\_liverA, Unigene5940\_Mf\_liverA, NM\_007393, Unigene28731\_Mf\_liverA, NM\_053072, Unigene1212\_Mf\_liverA, CL3549.Contig1\_Mf\_liverA, NM\_009706, Unigene24085\_Mf\_liverA, CL4106.Contig1\_Mf\_liverA, CL3750.Contig1\_Mf\_liverA, CL1588.Contig3\_Mf\_liverA, Unigene8016\_Mf\_liverA, Unigene18796\_Mf\_liverA, NM\_009448, CL5307.Contig1\_Mf\_liverA, Unigene31730\_Mf\_liverA, Unigene51055\_Mf\_liverA, CL4432.Contig2\_Mf\_liverA, Unigene33525\_Mf\_liverA, Unigene4540\_Mf\_liverA, Unigene24204\_Mf\_liverA, Unigene37310\_Mf\_liverA, Unigene9406\_Mf\_liverA, Unigene27803\_Mf\_liverA, Unigene15026\_Mf\_liverA, Unigene1221\_Mf\_liverA, CL1588.Contig1\_Mf\_liverA, NM\_017379, Unigene39875\_Mf\_liverA, NM\_001081274, Unigene25238\_Mf\_liverA, Unigene7224\_Mf\_liverA, Unigene26309\_Mf\_liverA, Unigene17632\_Mf\_liverA, Unigene5745\_Mf\_liverA, Unigene12889\_Mf\_liverA, Unigene38514\_Mf\_liverA, Unigene665\_Mf\_liverA, Unigene35678\_Mf\_liverA, CL4434.Contig1\_Mf\_liverA, Unigene5147\_Mf\_liverA, Unigene8560\_Mf\_liverA, Unigene13396\_Mf\_liverA, Unigene41419\_Mf\_liverA, Unigene21359\_Mf\_liverA, Unigene36487\_Mf\_liverA, Unigene37474\_Mf\_liverA, Unigene16891\_Mf\_liverA, Unigene7733\_Mf\_liverA, Unigene37819\_Mf\_liverA, Unigene5886\_Mf\_liverA, Unigene27274\_Mf\_liverA, Unigene15493\_Mf\_liverA |
| microtubule cytoskeleton | CL1537.Contig1\_Mf\_liverA, NM\_009450, NM\_007896, Unigene28527\_Mf\_liverA, CL507.Contig1\_Mf\_liverA, NM\_145824, Unigene44317\_Mf\_liverA, Unigene15318\_Mf\_liverA, Unigene7412\_Mf\_liverA, CL4722.Contig1\_Mf\_liverA, NM\_009448, CL425.Contig1\_Mf\_liverA, Unigene23870\_Mf\_liverA, Unigene4768\_Mf\_liverA, Unigene34218\_Mf\_liverA, Unigene25662\_Mf\_liverA, CL3198.Contig1\_Mf\_liverA, Unigene14263\_Mf\_liverA, NM\_008538, Unigene27895\_Mf\_liverA, Unigene15170\_Mf\_liverA, Unigene26250\_Mf\_liverA, CL33.Contig4\_Mf\_liverA, Unigene48460\_Mf\_liverA, Unigene673\_Mf\_liverA, Unigene50250\_Mf\_liverA, Unigene7510\_Mf\_liverA, CL3803.Contig2\_Mf\_liverA, Unigene43515\_Mf\_liverA, Unigene27547\_Mf\_liverA, Unigene24284\_Mf\_liverA, NM\_017379, Unigene25080\_Mf\_liverA, Unigene34219\_Mf\_liverA, Unigene16671\_Mf\_liverA, Unigene21255\_Mf\_liverA, Unigene21317\_Mf\_liverA, Unigene25070\_Mf\_liverA, CL1810.Contig1\_Mf\_liverA, Unigene25057\_Mf\_liverA, NM\_146016, NM\_175260, CL33.Contig3\_Mf\_liverA, CL4434.Contig1\_Mf\_liverA, CL5688.Contig1\_Mf\_liverA, CL2117.Contig1\_Mf\_liverA, Unigene26214\_Mf\_liverA, Unigene2746\_Mf\_liverA, Unigene2745\_Mf\_liverA, Unigene15493\_Mf\_liverA, NM\_009447, Unigene23869\_Mf\_liverA |
| Cajal body | CL3268.Contig1\_Mf\_liverA, CL4057.Contig1\_Mf\_liverA |
| tight junction | Unigene26214\_Mf\_liverA, Unigene7350\_Mf\_liverA, Unigene14765\_Mf\_liverA, CL2117.Contig1\_Mf\_liverA |
| occluding junction | Unigene26214\_Mf\_liverA, Unigene7350\_Mf\_liverA, Unigene14765\_Mf\_liverA, CL2117.Contig1\_Mf\_liverA |
| small nuclear ribonucleoprotein complex | CL5001.Contig2\_Mf\_liverA, Unigene19821\_Mf\_liverA |
| early endosome membrane | Unigene4703\_Mf\_liverA, Unigene1440\_Mf\_liverA |
| small ribosomal subunit | CL1063.Contig1\_Mf\_liverA, NM\_009091, CL5049.Contig2\_Mf\_liverA |
| vesicle coat | Unigene34234\_Mf\_liverA, Unigene5632\_Mf\_liverA |
| condensed chromosome kinetochore | Unigene4540\_Mf\_liverA, Unigene25057\_Mf\_liverA, Unigene43515\_Mf\_liverA |
| basal part of cell | Unigene34197\_Mf\_liverA, Unigene28331\_Mf\_liverA |
| Golgi-associated vesicle membrane | Unigene27082\_Mf\_liverA, Unigene5632\_Mf\_liverA |
| nuclear pore | Unigene25057\_Mf\_liverA, Unigene34197\_Mf\_liverA |
| microtubule organizing center | Unigene25080\_Mf\_liverA, CL1537.Contig1\_Mf\_liverA, NM\_007896, Unigene16671\_Mf\_liverA, Unigene21255\_Mf\_liverA, Unigene21317\_Mf\_liverA, Unigene25070\_Mf\_liverA, CL1810.Contig1\_Mf\_liverA, Unigene44317\_Mf\_liverA, Unigene15318\_Mf\_liverA, Unigene7412\_Mf\_liverA, Unigene25057\_Mf\_liverA, Unigene4768\_Mf\_liverA, Unigene34218\_Mf\_liverA, Unigene25662\_Mf\_liverA, Unigene27895\_Mf\_liverA, NM\_008538, Unigene15170\_Mf\_liverA, CL5688.Contig1\_Mf\_liverA, CL2117.Contig1\_Mf\_liverA, Unigene673\_Mf\_liverA, Unigene48460\_Mf\_liverA, Unigene2746\_Mf\_liverA, Unigene50250\_Mf\_liverA, Unigene2745\_Mf\_liverA, Unigene43515\_Mf\_liverA |
| basal lamina | Unigene4985\_Mf\_liverA, Unigene560\_Mf\_liverA |
| postsynaptic density | CL479.Contig1\_Mf\_liverA, Unigene27260\_Mf\_liverA, CL1222.Contig1\_Mf\_liverA, Unigene24252\_Mf\_liverA |
| dendritic spine head | CL479.Contig1\_Mf\_liverA, Unigene27260\_Mf\_liverA, CL1222.Contig1\_Mf\_liverA, Unigene24252\_Mf\_liverA |
| clathrin-coated vesicle | Unigene30587\_Mf\_liverA, Unigene13363\_Mf\_liverA, CL2855.Contig2\_Mf\_liverA, CL1738.Contig1\_Mf\_liverA, Unigene37259\_Mf\_liverA, CL2855.Contig1\_Mf\_liverA, Unigene30585\_Mf\_liverA, CL3685.Contig1\_Mf\_liverA, Unigene34234\_Mf\_liverA, Unigene30584\_Mf\_liverA |
| transcriptional repressor complex | Unigene31517\_Mf\_liverA, CL2355.Contig1\_Mf\_liverA, Unigene37433\_Mf\_liverA |
| proton-transporting two-sector ATPase complex | CL5993.Contig3\_Mf\_liverA, Unigene36845\_Mf\_liverA |
| cytosolic part | CL1063.Contig1\_Mf\_liverA, Unigene2633\_Mf\_liverA, Unigene36710\_Mf\_liverA, Unigene5325\_Mf\_liverA, Unigene38657\_Mf\_liverA, CL5049.Contig2\_Mf\_liverA |
| condensed chromosome, centromeric region | Unigene4540\_Mf\_liverA, Unigene25057\_Mf\_liverA, Unigene43515\_Mf\_liverA |
| ubiquitin ligase complex | Unigene5248\_Mf\_liverA, Unigene33054\_Mf\_liverA, Unigene48460\_Mf\_liverA, Unigene29334\_Mf\_liverA, Unigene5954\_Mf\_liverA, Unigene34446\_Mf\_liverA, Unigene13683\_Mf\_liverA |
| nuclear chromatin | Unigene26515\_Mf\_liverA, CL2326.Contig1\_Mf\_liverA, Unigene14907\_Mf\_liverA, Unigene38124\_Mf\_liverA, Unigene34035\_Mf\_liverA, NM\_011418, CL2131.Contig4\_Mf\_liverA, CL4757.Contig1\_Mf\_liverA |
| membrane-enclosed lumen | Unigene29399\_Mf\_liverA, NM\_176843, Unigene35884\_Mf\_liverA, Unigene38015\_Mf\_liverA, Unigene5852\_Mf\_liverA, Unigene7612\_Mf\_liverA, Unigene30356\_Mf\_liverA, Unigene38124\_Mf\_liverA, Unigene36851\_Mf\_liverA, Unigene14665\_Mf\_liverA, Unigene29424\_Mf\_liverA, CL4105.Contig1\_Mf\_liverA, CL1063.Contig1\_Mf\_liverA, Unigene19049\_Mf\_liverA, Unigene29405\_Mf\_liverA, Unigene32882\_Mf\_liverA, Unigene31263\_Mf\_liverA, Unigene7619\_Mf\_liverA, Unigene7969\_Mf\_liverA, Unigene21466\_Mf\_liverA, Unigene26422\_Mf\_liverA, Unigene29334\_Mf\_liverA, Unigene15064\_Mf\_liverA, NM\_010877, Unigene34124\_Mf\_liverA, Unigene38919\_Mf\_liverA, CL4757.Contig1\_Mf\_liverA, CL6038.Contig2\_Mf\_liverA, CL5001.Contig2\_Mf\_liverA, Unigene21255\_Mf\_liverA, CL4141.Contig1\_Mf\_liverA, Unigene35431\_Mf\_liverA, NM\_145419, Unigene23273\_Mf\_liverA, CL1119.Contig1\_Mf\_liverA, Unigene35816\_Mf\_liverA, Unigene13658\_Mf\_liverA, Unigene41158\_Mf\_liverA, Unigene25596\_Mf\_liverA, Unigene22978\_Mf\_liverA, Unigene36988\_Mf\_liverA, Unigene23328\_Mf\_liverA, Unigene16684\_Mf\_liverA, CL4847.Contig1\_Mf\_liverA, Unigene36417\_Mf\_liverA, CL2855.Contig2\_Mf\_liverA, Unigene36691\_Mf\_liverA, Unigene38406\_Mf\_liverA, CL2326.Contig1\_Mf\_liverA, Unigene19297\_Mf\_liverA, NM\_177093, CL4722.Contig1\_Mf\_liverA, Unigene14907\_Mf\_liverA, Unigene7476\_Mf\_liverA, Unigene1479\_Mf\_liverA, Unigene26398\_Mf\_liverA, Unigene34258\_Mf\_liverA, CL2131.Contig4\_Mf\_liverA, Unigene25976\_Mf\_liverA, Unigene37460\_Mf\_liverA, Unigene31517\_Mf\_liverA, Unigene36112\_Mf\_liverA, NM\_134059, NM\_011305, Unigene27438\_Mf\_liverA, Unigene34123\_Mf\_liverA, Unigene37575\_Mf\_liverA, Unigene43515\_Mf\_liverA, Unigene11097\_Mf\_liverA, Unigene26873\_Mf\_liverA, NM\_009091, NM\_134156, CL3268.Contig1\_Mf\_liverA, Unigene10820\_Mf\_liverA, Unigene30493\_Mf\_liverA, Unigene37729\_Mf\_liverA, Unigene29940\_Mf\_liverA, CL795.Contig1\_Mf\_liverA, Unigene42812\_Mf\_liverA, Unigene152\_Mf\_liverA, NM\_009087, Unigene37076\_Mf\_liverA, Unigene21256\_Mf\_liverA, Unigene30288\_Mf\_liverA, CL2962.Contig1\_Mf\_liverA, CL2855.Contig1\_Mf\_liverA, Unigene5236\_Mf\_liverA, NM\_011132, Unigene28021\_Mf\_liverA, Unigene37433\_Mf\_liverA, Unigene26515\_Mf\_liverA, CL1537.Contig1\_Mf\_liverA, NM\_010481, NM\_175472, Unigene13414\_Mf\_liverA, Unigene22980\_Mf\_liverA, CL2797.Contig2\_Mf\_liverA, Unigene38339\_Mf\_liverA, CL1125.Contig1\_Mf\_liverA, Unigene14916\_Mf\_liverA, CL4162.Contig1\_Mf\_liverA, Unigene35237\_Mf\_liverA, Unigene30808\_Mf\_liverA, NM\_010579, Unigene7350\_Mf\_liverA, Unigene19821\_Mf\_liverA, Unigene5165\_Mf\_liverA, CL4057.Contig1\_Mf\_liverA, Unigene35958\_Mf\_liverA, Unigene29876\_Mf\_liverA, Unigene30707\_Mf\_liverA, CL4583.Contig2\_Mf\_liverA, Unigene13525\_Mf\_liverA, Unigene25080\_Mf\_liverA, Unigene36414\_Mf\_liverA, Unigene33880\_Mf\_liverA, Unigene34219\_Mf\_liverA, CL1222.Contig1\_Mf\_liverA, Unigene29885\_Mf\_liverA, Unigene1129\_Mf\_liverA, CL1810.Contig1\_Mf\_liverA, NM\_009609, Unigene35491\_Mf\_liverA, NM\_029872, Unigene33168\_Mf\_liverA, Unigene27483\_Mf\_liverA, Unigene28662\_Mf\_liverA, CL3738.Contig1\_Mf\_liverA, Unigene35935\_Mf\_liverA, Unigene36710\_Mf\_liverA, NM\_007393, Unigene34035\_Mf\_liverA, CL5688.Contig1\_Mf\_liverA, Unigene13683\_Mf\_liverA, Unigene24801\_Mf\_liverA, CL2791.Contig1\_Mf\_liverA, Unigene9698\_Mf\_liverA, Unigene36420\_Mf\_liverA, Unigene24758\_Mf\_liverA, Unigene23271\_Mf\_liverA, Unigene34197\_Mf\_liverA, Unigene24471\_Mf\_liverA, NM\_009883, Unigene5954\_Mf\_liverA, Unigene15529\_Mf\_liverA, Unigene31730\_Mf\_liverA, CL2251.Contig1\_Mf\_liverA, CL4490.Contig2\_Mf\_liverA, Unigene24566\_Mf\_liverA, Unigene25595\_Mf\_liverA, Unigene37310\_Mf\_liverA, NM\_011418, Unigene4604\_Mf\_liverA, Unigene14212\_Mf\_liverA, Unigene560\_Mf\_liverA, Unigene30731\_Mf\_liverA, Unigene48460\_Mf\_liverA, Unigene36418\_Mf\_liverA, Unigene29558\_Mf\_liverA, Unigene23185\_Mf\_liverA, Unigene16671\_Mf\_liverA, CL2355.Contig1\_Mf\_liverA, Unigene10313\_Mf\_liverA, Unigene37153\_Mf\_liverA, Unigene15592\_Mf\_liverA, Unigene37711\_Mf\_liverA, Unigene5422\_Mf\_liverA, Unigene12889\_Mf\_liverA, Unigene7968\_Mf\_liverA, Unigene36673\_Mf\_liverA, Unigene803\_Mf\_liverA, CL3911.Contig2\_Mf\_liverA, Unigene21359\_Mf\_liverA, Unigene31392\_Mf\_liverA, Unigene36487\_Mf\_liverA, Unigene42855\_Mf\_liverA, CL548.Contig1\_Mf\_liverA |
| myelin sheath | Unigene5712\_Mf\_liverA, Unigene37268\_Mf\_liverA |
| DNA-directed RNA polymerase II, holoenzyme | NM\_176843, Unigene34219\_Mf\_liverA, CL4757.Contig1\_Mf\_liverA |
| condensed chromosome | Unigene26515\_Mf\_liverA, CL2326.Contig1\_Mf\_liverA, Unigene27438\_Mf\_liverA, Unigene4540\_Mf\_liverA, Unigene25057\_Mf\_liverA, Unigene21255\_Mf\_liverA, Unigene43515\_Mf\_liverA |
| organelle lumen | Unigene29399\_Mf\_liverA, NM\_176843, Unigene35884\_Mf\_liverA, Unigene38015\_Mf\_liverA, Unigene5852\_Mf\_liverA, Unigene7612\_Mf\_liverA, Unigene30356\_Mf\_liverA, Unigene38124\_Mf\_liverA, Unigene36851\_Mf\_liverA, Unigene14665\_Mf\_liverA, Unigene29424\_Mf\_liverA, CL4105.Contig1\_Mf\_liverA, CL1063.Contig1\_Mf\_liverA, Unigene19049\_Mf\_liverA, Unigene29405\_Mf\_liverA, Unigene32882\_Mf\_liverA, Unigene31263\_Mf\_liverA, Unigene7619\_Mf\_liverA, Unigene7969\_Mf\_liverA, Unigene21466\_Mf\_liverA, Unigene26422\_Mf\_liverA, Unigene29334\_Mf\_liverA, Unigene15064\_Mf\_liverA, NM\_010877, Unigene34124\_Mf\_liverA, Unigene38919\_Mf\_liverA, CL4757.Contig1\_Mf\_liverA, CL6038.Contig2\_Mf\_liverA, CL5001.Contig2\_Mf\_liverA, Unigene21255\_Mf\_liverA, CL4141.Contig1\_Mf\_liverA, Unigene35431\_Mf\_liverA, NM\_145419, Unigene23273\_Mf\_liverA, CL1119.Contig1\_Mf\_liverA, Unigene35816\_Mf\_liverA, Unigene13658\_Mf\_liverA, Unigene41158\_Mf\_liverA, Unigene25596\_Mf\_liverA, Unigene22978\_Mf\_liverA, Unigene36988\_Mf\_liverA, Unigene23328\_Mf\_liverA, Unigene16684\_Mf\_liverA, CL4847.Contig1\_Mf\_liverA, Unigene36417\_Mf\_liverA, CL2855.Contig2\_Mf\_liverA, Unigene36691\_Mf\_liverA, Unigene38406\_Mf\_liverA, CL2326.Contig1\_Mf\_liverA, Unigene19297\_Mf\_liverA, NM\_177093, CL4722.Contig1\_Mf\_liverA, Unigene14907\_Mf\_liverA, Unigene7476\_Mf\_liverA, Unigene1479\_Mf\_liverA, Unigene26398\_Mf\_liverA, Unigene34258\_Mf\_liverA, CL2131.Contig4\_Mf\_liverA, Unigene25976\_Mf\_liverA, Unigene37460\_Mf\_liverA, Unigene31517\_Mf\_liverA, Unigene36112\_Mf\_liverA, NM\_134059, NM\_011305, Unigene27438\_Mf\_liverA, Unigene34123\_Mf\_liverA, Unigene37575\_Mf\_liverA, Unigene43515\_Mf\_liverA, Unigene11097\_Mf\_liverA, Unigene26873\_Mf\_liverA, NM\_009091, NM\_134156, CL3268.Contig1\_Mf\_liverA, Unigene10820\_Mf\_liverA, Unigene30493\_Mf\_liverA, Unigene37729\_Mf\_liverA, Unigene29940\_Mf\_liverA, CL795.Contig1\_Mf\_liverA, Unigene42812\_Mf\_liverA, Unigene152\_Mf\_liverA, NM\_009087, Unigene37076\_Mf\_liverA, Unigene21256\_Mf\_liverA, Unigene30288\_Mf\_liverA, CL2962.Contig1\_Mf\_liverA, CL2855.Contig1\_Mf\_liverA, Unigene5236\_Mf\_liverA, NM\_011132, Unigene28021\_Mf\_liverA, Unigene37433\_Mf\_liverA, Unigene26515\_Mf\_liverA, CL1537.Contig1\_Mf\_liverA, NM\_010481, NM\_175472, Unigene13414\_Mf\_liverA, Unigene22980\_Mf\_liverA, Unigene38339\_Mf\_liverA, CL1125.Contig1\_Mf\_liverA, Unigene14916\_Mf\_liverA, CL4162.Contig1\_Mf\_liverA, Unigene35237\_Mf\_liverA, Unigene30808\_Mf\_liverA, NM\_010579, Unigene7350\_Mf\_liverA, Unigene19821\_Mf\_liverA, Unigene5165\_Mf\_liverA, CL4057.Contig1\_Mf\_liverA, Unigene35958\_Mf\_liverA, Unigene29876\_Mf\_liverA, Unigene30707\_Mf\_liverA, CL4583.Contig2\_Mf\_liverA, Unigene13525\_Mf\_liverA, Unigene25080\_Mf\_liverA, Unigene36414\_Mf\_liverA, Unigene33880\_Mf\_liverA, Unigene34219\_Mf\_liverA, CL1222.Contig1\_Mf\_liverA, Unigene29885\_Mf\_liverA, Unigene1129\_Mf\_liverA, CL1810.Contig1\_Mf\_liverA, NM\_009609, Unigene35491\_Mf\_liverA, NM\_029872, Unigene33168\_Mf\_liverA, Unigene27483\_Mf\_liverA, Unigene28662\_Mf\_liverA, CL3738.Contig1\_Mf\_liverA, Unigene35935\_Mf\_liverA, Unigene36710\_Mf\_liverA, NM\_007393, Unigene34035\_Mf\_liverA, CL5688.Contig1\_Mf\_liverA, Unigene13683\_Mf\_liverA, Unigene24801\_Mf\_liverA, CL2791.Contig1\_Mf\_liverA, Unigene9698\_Mf\_liverA, Unigene36420\_Mf\_liverA, Unigene24758\_Mf\_liverA, Unigene23271\_Mf\_liverA, Unigene34197\_Mf\_liverA, Unigene24471\_Mf\_liverA, NM\_009883, Unigene5954\_Mf\_liverA, Unigene15529\_Mf\_liverA, Unigene31730\_Mf\_liverA, CL2251.Contig1\_Mf\_liverA, Unigene24566\_Mf\_liverA, Unigene25595\_Mf\_liverA, Unigene37310\_Mf\_liverA, NM\_011418, Unigene4604\_Mf\_liverA, Unigene14212\_Mf\_liverA, Unigene560\_Mf\_liverA, Unigene30731\_Mf\_liverA, Unigene48460\_Mf\_liverA, Unigene36418\_Mf\_liverA, Unigene29558\_Mf\_liverA, Unigene16671\_Mf\_liverA, CL2355.Contig1\_Mf\_liverA, Unigene10313\_Mf\_liverA, Unigene37153\_Mf\_liverA, Unigene15592\_Mf\_liverA, Unigene37711\_Mf\_liverA, Unigene5422\_Mf\_liverA, Unigene12889\_Mf\_liverA, Unigene7968\_Mf\_liverA, Unigene36673\_Mf\_liverA, Unigene803\_Mf\_liverA, CL3911.Contig2\_Mf\_liverA, Unigene21359\_Mf\_liverA, Unigene31392\_Mf\_liverA, Unigene36487\_Mf\_liverA, Unigene42855\_Mf\_liverA, CL548.Contig1\_Mf\_liverA |
| spindle | CL4434.Contig1\_Mf\_liverA, Unigene34219\_Mf\_liverA, Unigene28527\_Mf\_liverA, CL507.Contig1\_Mf\_liverA, Unigene26250\_Mf\_liverA, Unigene7412\_Mf\_liverA, Unigene25057\_Mf\_liverA, CL4722.Contig1\_Mf\_liverA, Unigene48460\_Mf\_liverA, Unigene50250\_Mf\_liverA, Unigene43515\_Mf\_liverA |
| ribosome | CL1063.Contig1\_Mf\_liverA, Unigene37460\_Mf\_liverA, Unigene36710\_Mf\_liverA, Unigene33138\_Mf\_liverA, Unigene5325\_Mf\_liverA, NM\_009091, CL5049.Contig2\_Mf\_liverA, CL5560.Contig1\_Mf\_liverA, NM\_001024205 |
| clathrin coated vesicle membrane | CL2855.Contig1\_Mf\_liverA, CL2855.Contig2\_Mf\_liverA, Unigene34234\_Mf\_liverA |
| peroxisome | CL1125.Contig1\_Mf\_liverA, Unigene37076\_Mf\_liverA, Unigene743\_Mf\_liverA, Unigene5165\_Mf\_liverA, Unigene35678\_Mf\_liverA, Unigene28331\_Mf\_liverA |
| spliceosomal complex | CL5001.Contig2\_Mf\_liverA, Unigene18117\_Mf\_liverA, Unigene19821\_Mf\_liverA, NM\_134059, CL3268.Contig1\_Mf\_liverA, Unigene3174\_Mf\_liverA, CL4057.Contig1\_Mf\_liverA |
| nuclear chromosome | Unigene26515\_Mf\_liverA, Unigene23271\_Mf\_liverA, Unigene34035\_Mf\_liverA, Unigene31392\_Mf\_liverA, NM\_011418, CL2326.Contig1\_Mf\_liverA, Unigene42855\_Mf\_liverA, Unigene14907\_Mf\_liverA, Unigene7969\_Mf\_liverA, Unigene38124\_Mf\_liverA, Unigene23273\_Mf\_liverA, CL2131.Contig4\_Mf\_liverA, Unigene7968\_Mf\_liverA, CL4757.Contig1\_Mf\_liverA, Unigene43515\_Mf\_liverA |
| organelle inner membrane | Unigene25398\_Mf\_liverA, Unigene27260\_Mf\_liverA, NM\_009592, Unigene36845\_Mf\_liverA, Unigene38406\_Mf\_liverA, NM\_008293, CL2797.Contig2\_Mf\_liverA, Unigene4781\_Mf\_liverA, CL1125.Contig1\_Mf\_liverA, CL4490.Contig2\_Mf\_liverA, NM\_153193, Unigene21359\_Mf\_liverA, Unigene36641\_Mf\_liverA, Unigene18117\_Mf\_liverA, Unigene3174\_Mf\_liverA, Unigene5512\_Mf\_liverA, CL2722.Contig1\_Mf\_liverA, Unigene30303\_Mf\_liverA, Unigene13233\_Mf\_liverA, Unigene23185\_Mf\_liverA, CL4106.Contig1\_Mf\_liverA |
| PML body | Unigene7619\_Mf\_liverA, CL2355.Contig1\_Mf\_liverA, CL4757.Contig1\_Mf\_liverA |
[truncated: 80,737 more chars]
