# Supplementary material for: De novo assembly and transcriptome characterization: novel insights into the natural resistance mechanisms of Microtus fortis against Schistosoma japonicum
Source: BMC Genomics. 2014 Jun 2;15(1):417. doi: 10.1186/1471-2164-15-417 (PMC4073500; doi:10.1186/1471-2164-15-417)
Supplement: Supplementary file 6 — Additional file 6: Dataset S4: GO terms for MfA-VS-MfA3W_C. (ZIP 42 KB) [file 12864_2013_6159_MOESM6_ESM.zip › 1990354100108772_add6.html]

Terms for MfA-VS-MfA3W\_C


## Terms for MfA-VS-MfA3W\_C

---


### Result Table

|  |
| --- |
| **Terms from the Component Ontology with p-value as good or better than 1** |

| Gene Ontology term | Cluster frequency | Genome frequency of use | Corrected P-value | Expression Profile |
| --- | --- | --- | --- | --- |
| extracellular space | 70 out of 628 genes, 11.1% | 1015 out of 37603 genes, 2.7% | 3.57e-21 | View Result |
| extracellular region | 127 out of 628 genes, 20.2% | 2987 out of 37603 genes, 7.9% | 2.43e-20 | View Result |
| cytoplasm | 408 out of 628 genes, 65.0% | 17507 out of 37603 genes, 46.6% | 1.86e-18 | View Result |
| endoplasmic reticulum | 98 out of 628 genes, 15.6% | 2107 out of 37603 genes, 5.6% | 1.08e-17 | View Result |
| endoplasmic reticulum part | 76 out of 628 genes, 12.1% | 1424 out of 37603 genes, 3.8% | 1.55e-16 | View Result |
| integral to membrane | 195 out of 628 genes, 31.1% | 6371 out of 37603 genes, 16.9% | 4.47e-16 | View Result |
| cell periphery | 183 out of 628 genes, 29.1% | 6038 out of 37603 genes, 16.1% | 2.38e-14 | View Result |
| plasma membrane | 178 out of 628 genes, 28.3% | 5905 out of 37603 genes, 15.7% | 1.31e-13 | View Result |
| extracellular region part | 92 out of 628 genes, 14.6% | 2343 out of 37603 genes, 6.2% | 6.31e-12 | View Result |
| endoplasmic reticulum membrane | 59 out of 628 genes, 9.4% | 1142 out of 37603 genes, 3.0% | 7.47e-12 | View Result |
| MHC protein complex | 16 out of 628 genes, 2.5% | 71 out of 37603 genes, 0.2% | 1.25e-11 | View Result |
| nuclear outer membrane-endoplasmic reticulum membrane network | 59 out of 628 genes, 9.4% | 1173 out of 37603 genes, 3.1% | 2.34e-11 | View Result |
| cytoplasmic part | 333 out of 628 genes, 53.0% | 14592 out of 37603 genes, 38.8% | 7.34e-11 | View Result |
| integral to plasma membrane | 64 out of 628 genes, 10.2% | 1399 out of 37603 genes, 3.7% | 1.37e-10 | View Result |
| external side of plasma membrane | 27 out of 628 genes, 4.3% | 286 out of 37603 genes, 0.8% | 1.72e-10 | View Result |
| cell surface | 51 out of 628 genes, 8.1% | 1006 out of 37603 genes, 2.7% | 9.41e-10 | View Result |
| intrinsic to plasma membrane | 64 out of 628 genes, 10.2% | 1463 out of 37603 genes, 3.9% | 9.64e-10 | View Result |
| MHC class II protein complex | 8 out of 628 genes, 1.3% | 15 out of 37603 genes, 0.0% | 1.01e-08 | View Result |
| cytosol | 98 out of 628 genes, 15.6% | 2945 out of 37603 genes, 7.8% | 1.33e-08 | View Result |
| multivesicular body | 9 out of 628 genes, 1.4% | 35 out of 37603 genes, 0.1% | 1.38e-06 | View Result |
| symbiont-containing vacuole | 8 out of 628 genes, 1.3% | 27 out of 37603 genes, 0.1% | 2.93e-06 | View Result |
| plasma membrane part | 115 out of 628 genes, 18.3% | 4083 out of 37603 genes, 10.9% | 4.39e-06 | View Result |
| host cell cytoplasm | 8 out of 628 genes, 1.3% | 30 out of 37603 genes, 0.1% | 7.40e-06 | View Result |
| host cell cytoplasm part | 8 out of 628 genes, 1.3% | 30 out of 37603 genes, 0.1% | 7.40e-06 | View Result |
| host intracellular part | 8 out of 628 genes, 1.3% | 32 out of 37603 genes, 0.1% | 1.29e-05 | View Result |
| intracellular region of host | 8 out of 628 genes, 1.3% | 32 out of 37603 genes, 0.1% | 1.29e-05 | View Result |
| endoplasmic reticulum lumen | 16 out of 628 genes, 2.5% | 174 out of 37603 genes, 0.5% | 1.33e-05 | View Result |
| host | 8 out of 628 genes, 1.3% | 34 out of 37603 genes, 0.1% | 2.16e-05 | View Result |
| host cell part | 8 out of 628 genes, 1.3% | 34 out of 37603 genes, 0.1% | 2.16e-05 | View Result |
| extraorganismal space | 8 out of 628 genes, 1.3% | 34 out of 37603 genes, 0.1% | 2.16e-05 | View Result |
| host cell | 8 out of 628 genes, 1.3% | 34 out of 37603 genes, 0.1% | 2.16e-05 | View Result |
| other organism | 8 out of 628 genes, 1.3% | 34 out of 37603 genes, 0.1% | 2.16e-05 | View Result |
| other organism cell | 8 out of 628 genes, 1.3% | 34 out of 37603 genes, 0.1% | 2.16e-05 | View Result |
| other organism part | 8 out of 628 genes, 1.3% | 34 out of 37603 genes, 0.1% | 2.16e-05 | View Result |
| lysosome | 29 out of 628 genes, 4.6% | 560 out of 37603 genes, 1.5% | 3.19e-05 | View Result |
| extracellular organelle | 8 out of 628 genes, 1.3% | 45 out of 37603 genes, 0.1% | 0.00021 | View Result |
| extracellular membrane-bounded organelle | 8 out of 628 genes, 1.3% | 45 out of 37603 genes, 0.1% | 0.00021 | View Result |
| organelle membrane | 124 out of 628 genes, 19.7% | 4856 out of 37603 genes, 12.9% | 0.00024 | View Result |
| endosome | 42 out of 628 genes, 6.7% | 1107 out of 37603 genes, 2.9% | 0.00027 | View Result |
| late endosome | 19 out of 628 genes, 3.0% | 301 out of 37603 genes, 0.8% | 0.00028 | View Result |
| endomembrane system | 95 out of 628 genes, 15.1% | 3480 out of 37603 genes, 9.3% | 0.00039 | View Result |
| lytic vacuole | 31 out of 628 genes, 4.9% | 750 out of 37603 genes, 2.0% | 0.00140 | View Result |
| high-density lipoprotein particle | 7 out of 628 genes, 1.1% | 43 out of 37603 genes, 0.1% | 0.00202 | View Result |
| symbiont-containing vacuole membrane | 5 out of 628 genes, 0.8% | 20 out of 37603 genes, 0.1% | 0.00486 | View Result |
| MHC class I protein complex | 5 out of 628 genes, 0.8% | 20 out of 37603 genes, 0.1% | 0.00486 | View Result |
| vacuole | 32 out of 628 genes, 5.1% | 875 out of 37603 genes, 2.3% | 0.01119 | View Result |
| SREBP-SCAP-Insig complex | 3 out of 628 genes, 0.5% | 5 out of 37603 genes, 0.0% | 0.01365 | View Result |
| lysosomal membrane | 11 out of 628 genes, 1.8% | 151 out of 37603 genes, 0.4% | 0.01522 | View Result |
| vacuolar part | 16 out of 628 genes, 2.5% | 304 out of 37603 genes, 0.8% | 0.01869 | View Result |
| perinuclear region of cytoplasm | 27 out of 628 genes, 4.3% | 703 out of 37603 genes, 1.9% | 0.02000 | View Result |
| lysosomal lumen | 7 out of 628 genes, 1.1% | 65 out of 37603 genes, 0.2% | 0.03185 | View Result |
| proteasome core complex | 5 out of 628 genes, 0.8% | 29 out of 37603 genes, 0.1% | 0.03290 | View Result |
| vacuolar lumen | 7 out of 628 genes, 1.1% | 66 out of 37603 genes, 0.2% | 0.03512 | View Result |
| integral to organelle membrane | 14 out of 628 genes, 2.2% | 257 out of 37603 genes, 0.7% | 0.03696 | View Result |
| macrophage migration inhibitory factor receptor complex | 2 out of 628 genes, 0.3% | 2 out of 37603 genes, 0.0% | 0.08410 | View Result |
| NOS2-CD74 complex | 2 out of 628 genes, 0.3% | 2 out of 37603 genes, 0.0% | 0.08410 | View Result |
| proteasome complex | 8 out of 628 genes, 1.3% | 100 out of 37603 genes, 0.3% | 0.08457 | View Result |
| Golgi apparatus | 52 out of 628 genes, 8.3% | 1902 out of 37603 genes, 5.1% | 0.11430 | View Result |
| intrinsic to membrane | 218 out of 628 genes, 34.7% | 10799 out of 37603 genes, 28.7% | 0.17403 | View Result |
| membrane | 325 out of 628 genes, 51.8% | 17065 out of 37603 genes, 45.4% | 0.21814 | View Result |
| proteasome activator complex | 2 out of 628 genes, 0.3% | 3 out of 37603 genes, 0.0% | 0.24950 | View Result |
| vacuolar membrane | 12 out of 628 genes, 1.9% | 247 out of 37603 genes, 0.7% | 0.30304 | View Result |
| integral to endoplasmic reticulum membrane | 8 out of 628 genes, 1.3% | 122 out of 37603 genes, 0.3% | 0.31823 | View Result |
| cytoplasmic vesicle | 57 out of 628 genes, 9.1% | 2245 out of 37603 genes, 6.0% | 0.35506 | View Result |
| proteinaceous extracellular matrix | 20 out of 628 genes, 3.2% | 554 out of 37603 genes, 1.5% | 0.36118 | View Result |
| endosome membrane | 15 out of 628 genes, 2.4% | 366 out of 37603 genes, 1.0% | 0.42757 | View Result |
| membrane part | 256 out of 628 genes, 40.8% | 13186 out of 37603 genes, 35.1% | 0.48377 | View Result |
| membrane raft | 14 out of 628 genes, 2.2% | 335 out of 37603 genes, 0.9% | 0.50785 | View Result |
| endosomal part | 15 out of 628 genes, 2.4% | 375 out of 37603 genes, 1.0% | 0.54127 | View Result |
| early endosome | 13 out of 628 genes, 2.1% | 301 out of 37603 genes, 0.8% | 0.54974 | View Result |
| hemoglobin complex | 2 out of 628 genes, 0.3% | 5 out of 37603 genes, 0.0% | 0.81335 | View Result |
| chromaffin granule membrane | 2 out of 628 genes, 0.3% | 5 out of 37603 genes, 0.0% | 0.81335 | View Result |
| protein-lipid complex | 7 out of 628 genes, 1.1% | 112 out of 37603 genes, 0.3% | 0.84734 | View Result |
| plasma lipoprotein particle | 7 out of 628 genes, 1.1% | 112 out of 37603 genes, 0.3% | 0.84734 | View Result |
| apical plasma membrane | 14 out of 628 genes, 2.2% | 360 out of 37603 genes, 1.0% | 0.97489 | View Result |
| apical part of cell | 17 out of 628 genes, 2.7% | 488 out of 37603 genes, 1.3% | 1 | View Result |
| neuronal cell body | 17 out of 628 genes, 2.7% | 494 out of 37603 genes, 1.3% | 1 | View Result |
| intrinsic to organelle membrane | 16 out of 628 genes, 2.5% | 470 out of 37603 genes, 1.2% | 1 | View Result |
| proteasome accessory complex | 3 out of 628 genes, 0.5% | 23 out of 37603 genes, 0.1% | 1 | View Result |
| chromaffin granule | 2 out of 628 genes, 0.3% | 8 out of 37603 genes, 0.0% | 1 | View Result |
| melanosome | 6 out of 628 genes, 1.0% | 102 out of 37603 genes, 0.3% | 1 | View Result |
| secretory granule | 19 out of 628 genes, 3.0% | 610 out of 37603 genes, 1.6% | 1 | View Result |
| cytoplasmic membrane-bounded vesicle | 46 out of 628 genes, 7.3% | 1912 out of 37603 genes, 5.1% | 1 | View Result |
| extracellular matrix | 24 out of 628 genes, 3.8% | 854 out of 37603 genes, 2.3% | 1 | View Result |
| endocytic vesicle | 10 out of 628 genes, 1.6% | 252 out of 37603 genes, 0.7% | 1 | View Result |
| intrinsic to endoplasmic reticulum membrane | 9 out of 628 genes, 1.4% | 217 out of 37603 genes, 0.6% | 1 | View Result |
| endocytic vesicle membrane | 6 out of 628 genes, 1.0% | 113 out of 37603 genes, 0.3% | 1 | View Result |
| vesicle | 57 out of 628 genes, 9.1% | 2526 out of 37603 genes, 6.7% | 1 | View Result |
| dendrite | 19 out of 628 genes, 3.0% | 655 out of 37603 genes, 1.7% | 1 | View Result |
| membrane-bounded vesicle | 47 out of 628 genes, 7.5% | 2048 out of 37603 genes, 5.4% | 1 | View Result |
| clathrin-coated endocytic vesicle membrane | 3 out of 628 genes, 0.5% | 36 out of 37603 genes, 0.1% | 1 | View Result |
| membrane attack complex | 2 out of 628 genes, 0.3% | 14 out of 37603 genes, 0.0% | 1 | View Result |
| glycogen granule | 2 out of 628 genes, 0.3% | 14 out of 37603 genes, 0.0% | 1 | View Result |
| pigment granule | 8 out of 628 genes, 1.3% | 206 out of 37603 genes, 0.5% | 1 | View Result |
| coated vesicle membrane | 8 out of 628 genes, 1.3% | 209 out of 37603 genes, 0.6% | 1 | View Result |
| late endosome membrane | 5 out of 628 genes, 0.8% | 100 out of 37603 genes, 0.3% | 1 | View Result |
| clathrin-coated endocytic vesicle | 3 out of 628 genes, 0.5% | 42 out of 37603 genes, 0.1% | 1 | View Result |
| clathrin coated vesicle membrane | 6 out of 628 genes, 1.0% | 145 out of 37603 genes, 0.4% | 1 | View Result |
| vesicular fraction | 14 out of 628 genes, 2.2% | 487 out of 37603 genes, 1.3% | 1 | View Result |
| cytoplasmic vesicle part | 17 out of 628 genes, 2.7% | 629 out of 37603 genes, 1.7% | 1 | View Result |
| axon | 15 out of 628 genes, 2.4% | 539 out of 37603 genes, 1.4% | 1 | View Result |
| collagen | 5 out of 628 genes, 0.8% | 112 out of 37603 genes, 0.3% | 1 | View Result |
| pore complex | 5 out of 628 genes, 0.8% | 112 out of 37603 genes, 0.3% | 1 | View Result |
| dendritic shaft | 3 out of 628 genes, 0.5% | 46 out of 37603 genes, 0.1% | 1 | View Result |
| integral to lumenal side of endoplasmic reticulum membrane | 2 out of 628 genes, 0.3% | 20 out of 37603 genes, 0.1% | 1 | View Result |
| extracellular matrix part | 10 out of 628 genes, 1.6% | 335 out of 37603 genes, 0.9% | 1 | View Result |
| vesicle lumen | 4 out of 628 genes, 0.6% | 88 out of 37603 genes, 0.2% | 1 | View Result |
| trans-Golgi network membrane | 2 out of 628 genes, 0.3% | 24 out of 37603 genes, 0.1% | 1 | View Result |
| receptor complex | 9 out of 628 genes, 1.4% | 299 out of 37603 genes, 0.8% | 1 | View Result |
| mitochondrion | 58 out of 628 genes, 9.2% | 2848 out of 37603 genes, 7.6% | 1 | View Result |
| neuron projection terminus | 3 out of 628 genes, 0.5% | 59 out of 37603 genes, 0.2% | 1 | View Result |
| platelet alpha granule | 5 out of 628 genes, 0.8% | 135 out of 37603 genes, 0.4% | 1 | View Result |
| excitatory synapse | 2 out of 628 genes, 0.3% | 28 out of 37603 genes, 0.1% | 1 | View Result |
| cytoplasmic vesicle membrane | 14 out of 628 genes, 2.2% | 549 out of 37603 genes, 1.5% | 1 | View Result |
| SCF ubiquitin ligase complex | 2 out of 628 genes, 0.3% | 29 out of 37603 genes, 0.1% | 1 | View Result |
| integral to Golgi membrane | 3 out of 628 genes, 0.5% | 64 out of 37603 genes, 0.2% | 1 | View Result |
| neuron projection | 35 out of 628 genes, 5.6% | 1654 out of 37603 genes, 4.4% | 1 | View Result |
| cell part | 549 out of 628 genes, 87.4% | 32173 out of 37603 genes, 85.6% | 1 | View Result |
| cell | 549 out of 628 genes, 87.4% | 32174 out of 37603 genes, 85.6% | 1 | View Result |
| phagocytic vesicle | 3 out of 628 genes, 0.5% | 67 out of 37603 genes, 0.2% | 1 | View Result |
| anchored to membrane | 5 out of 628 genes, 0.8% | 151 out of 37603 genes, 0.4% | 1 | View Result |
| COPI-coated vesicle | 2 out of 628 genes, 0.3% | 34 out of 37603 genes, 0.1% | 1 | View Result |
| acrosomal vesicle | 5 out of 628 genes, 0.8% | 153 out of 37603 genes, 0.4% | 1 | View Result |
| Ada2/Gcn5/Ada3 transcription activator complex | 2 out of 628 genes, 0.3% | 35 out of 37603 genes, 0.1% | 1 | View Result |
| ER to Golgi transport vesicle membrane | 2 out of 628 genes, 0.3% | 35 out of 37603 genes, 0.1% | 1 | View Result |
| basolateral plasma membrane | 16 out of 628 genes, 2.5% | 687 out of 37603 genes, 1.8% | 1 | View Result |
| microvillus | 5 out of 628 genes, 0.8% | 154 out of 37603 genes, 0.4% | 1 | View Result |
| secretory granule membrane | 5 out of 628 genes, 0.8% | 154 out of 37603 genes, 0.4% | 1 | View Result |
| dendritic spine | 7 out of 628 genes, 1.1% | 248 out of 37603 genes, 0.7% | 1 | View Result |
| neuron spine | 7 out of 628 genes, 1.1% | 248 out of 37603 genes, 0.7% | 1 | View Result |
| basement membrane | 6 out of 628 genes, 1.0% | 206 out of 37603 genes, 0.5% | 1 | View Result |
| cell projection membrane | 9 out of 628 genes, 1.4% | 349 out of 37603 genes, 0.9% | 1 | View Result |
| focal adhesion | 6 out of 628 genes, 1.0% | 207 out of 37603 genes, 0.6% | 1 | View Result |
| cytoplasmic membrane-bounded vesicle lumen | 3 out of 628 genes, 0.5% | 77 out of 37603 genes, 0.2% | 1 | View Result |
| vesicle coat | 3 out of 628 genes, 0.5% | 78 out of 37603 genes, 0.2% | 1 | View Result |
| very-low-density lipoprotein particle | 2 out of 628 genes, 0.3% | 40 out of 37603 genes, 0.1% | 1 | View Result |
| basal part of cell | 3 out of 628 genes, 0.5% | 79 out of 37603 genes, 0.2% | 1 | View Result |
| Golgi membrane | 18 out of 628 genes, 2.9% | 819 out of 37603 genes, 2.2% | 1 | View Result |
| caveola | 4 out of 628 genes, 0.6% | 122 out of 37603 genes, 0.3% | 1 | View Result |
| trans-Golgi network | 5 out of 628 genes, 0.8% | 167 out of 37603 genes, 0.4% | 1 | View Result |
| intracellular part | 492 out of 628 genes, 78.3% | 28781 out of 37603 genes, 76.5% | 1 | View Result |
| basal lamina | 3 out of 628 genes, 0.5% | 82 out of 37603 genes, 0.2% | 1 | View Result |
| cell body | 20 out of 628 genes, 3.2% | 936 out of 37603 genes, 2.5% | 1 | View Result |
| vesicle membrane | 15 out of 628 genes, 2.4% | 672 out of 37603 genes, 1.8% | 1 | View Result |
| intrinsic to Golgi membrane | 4 out of 628 genes, 0.6% | 128 out of 37603 genes, 0.3% | 1 | View Result |
| synaptic vesicle membrane | 2 out of 628 genes, 0.3% | 44 out of 37603 genes, 0.1% | 1 | View Result |
| membrane fraction | 16 out of 628 genes, 2.5% | 745 out of 37603 genes, 2.0% | 1 | View Result |
| ER to Golgi transport vesicle | 2 out of 628 genes, 0.3% | 48 out of 37603 genes, 0.1% | 1 | View Result |
| axon terminus | 2 out of 628 genes, 0.3% | 48 out of 37603 genes, 0.1% | 1 | View Result |
| intracellular | 498 out of 628 genes, 79.3% | 29285 out of 37603 genes, 77.9% | 1 | View Result |
| Golgi apparatus part | 23 out of 628 genes, 3.7% | 1145 out of 37603 genes, 3.0% | 1 | View Result |
| lamellipodium | 5 out of 628 genes, 0.8% | 191 out of 37603 genes, 0.5% | 1 | View Result |
| intermediate filament | 4 out of 628 genes, 0.6% | 144 out of 37603 genes, 0.4% | 1 | View Result |
| cell projection | 56 out of 628 genes, 8.9% | 3024 out of 37603 genes, 8.0% | 1 | View Result |
| Golgi lumen | 2 out of 628 genes, 0.3% | 54 out of 37603 genes, 0.1% | 1 | View Result |
| perikaryon | 2 out of 628 genes, 0.3% | 54 out of 37603 genes, 0.1% | 1 | View Result |
| synapse part | 10 out of 628 genes, 1.6% | 453 out of 37603 genes, 1.2% | 1 | View Result |
| neuromuscular junction | 2 out of 628 genes, 0.3% | 55 out of 37603 genes, 0.1% | 1 | View Result |
| intercalated disc | 2 out of 628 genes, 0.3% | 57 out of 37603 genes, 0.2% | 1 | View Result |
| coated vesicle | 11 out of 628 genes, 1.8% | 526 out of 37603 genes, 1.4% | 1 | View Result |
| clathrin-coated vesicle | 9 out of 628 genes, 1.4% | 423 out of 37603 genes, 1.1% | 1 | View Result |
| brush border | 4 out of 628 genes, 0.6% | 160 out of 37603 genes, 0.4% | 1 | View Result |
| synaptic vesicle | 4 out of 628 genes, 0.6% | 161 out of 37603 genes, 0.4% | 1 | View Result |
| filopodium | 3 out of 628 genes, 0.5% | 112 out of 37603 genes, 0.3% | 1 | View Result |
| sarcoplasm | 2 out of 628 genes, 0.3% | 64 out of 37603 genes, 0.2% | 1 | View Result |
| triglyceride-rich lipoprotein particle | 2 out of 628 genes, 0.3% | 64 out of 37603 genes, 0.2% | 1 | View Result |
| transport vesicle membrane | 3 out of 628 genes, 0.5% | 113 out of 37603 genes, 0.3% | 1 | View Result |
| cell projection part | 21 out of 628 genes, 3.3% | 1106 out of 37603 genes, 2.9% | 1 | View Result |
| basal plasma membrane | 2 out of 628 genes, 0.3% | 67 out of 37603 genes, 0.2% | 1 | View Result |
| brush border membrane | 2 out of 628 genes, 0.3% | 68 out of 37603 genes, 0.2% | 1 | View Result |
| postsynaptic density | 4 out of 628 genes, 0.6% | 170 out of 37603 genes, 0.5% | 1 | View Result |
| dendritic spine head | 4 out of 628 genes, 0.6% | 170 out of 37603 genes, 0.5% | 1 | View Result |
| cell-cell contact zone | 2 out of 628 genes, 0.3% | 69 out of 37603 genes, 0.2% | 1 | View Result |
| centriole | 2 out of 628 genes, 0.3% | 71 out of 37603 genes, 0.2% | 1 | View Result |
| transport vesicle | 5 out of 628 genes, 0.8% | 228 out of 37603 genes, 0.6% | 1 | View Result |
| internal side of plasma membrane | 5 out of 628 genes, 0.8% | 229 out of 37603 genes, 0.6% | 1 | View Result |
| midbody | 3 out of 628 genes, 0.5% | 123 out of 37603 genes, 0.3% | 1 | View Result |
| peroxisome | 6 out of 628 genes, 1.0% | 284 out of 37603 genes, 0.8% | 1 | View Result |
| ribonucleoprotein granule | 3 out of 628 genes, 0.5% | 125 out of 37603 genes, 0.3% | 1 | View Result |
| platelet alpha granule lumen | 2 out of 628 genes, 0.3% | 74 out of 37603 genes, 0.2% | 1 | View Result |
| cortical actin cytoskeleton | 2 out of 628 genes, 0.3% | 75 out of 37603 genes, 0.2% | 1 | View Result |
| secretory granule lumen | 2 out of 628 genes, 0.3% | 75 out of 37603 genes, 0.2% | 1 | View Result |
| synapse | 14 out of 628 genes, 2.2% | 741 out of 37603 genes, 2.0% | 1 | View Result |
| cell-cell junction | 14 out of 628 genes, 2.2% | 742 out of 37603 genes, 2.0% | 1 | View Result |
| actin filament | 4 out of 628 genes, 0.6% | 183 out of 37603 genes, 0.5% | 1 | View Result |
| endoplasmic reticulum-Golgi intermediate compartment | 2 out of 628 genes, 0.3% | 79 out of 37603 genes, 0.2% | 1 | View Result |
| mitochondrial intermembrane space | 2 out of 628 genes, 0.3% | 80 out of 37603 genes, 0.2% | 1 | View Result |
| Golgi-associated vesicle membrane | 2 out of 628 genes, 0.3% | 80 out of 37603 genes, 0.2% | 1 | View Result |
| neuron projection membrane | 2 out of 628 genes, 0.3% | 80 out of 37603 genes, 0.2% | 1 | View Result |
| Golgi-associated vesicle | 3 out of 628 genes, 0.5% | 135 out of 37603 genes, 0.4% | 1 | View Result |
| nuclear pore | 2 out of 628 genes, 0.3% | 81 out of 37603 genes, 0.2% | 1 | View Result |
| cell-substrate adherens junction | 7 out of 628 genes, 1.1% | 359 out of 37603 genes, 1.0% | 1 | View Result |
| sarcolemma | 4 out of 628 genes, 0.6% | 193 out of 37603 genes, 0.5% | 1 | View Result |
| DNA-directed RNA polymerase II, holoenzyme | 3 out of 628 genes, 0.5% | 140 out of 37603 genes, 0.4% | 1 | View Result |
| intermediate filament cytoskeleton | 4 out of 628 genes, 0.6% | 197 out of 37603 genes, 0.5% | 1 | View Result |
| lipid particle | 2 out of 628 genes, 0.3% | 89 out of 37603 genes, 0.2% | 1 | View Result |
| organelle envelope lumen | 2 out of 628 genes, 0.3% | 89 out of 37603 genes, 0.2% | 1 | View Result |
| recycling endosome | 2 out of 628 genes, 0.3% | 91 out of 37603 genes, 0.2% | 1 | View Result |
| cell-substrate junction | 7 out of 628 genes, 1.1% | 381 out of 37603 genes, 1.0% | 1 | View Result |
| cell junction | 26 out of 628 genes, 4.1% | 1502 out of 37603 genes, 4.0% | 1 | View Result |
| coated pit | 2 out of 628 genes, 0.3% | 95 out of 37603 genes, 0.3% | 1 | View Result |
| leading edge membrane | 4 out of 628 genes, 0.6% | 212 out of 37603 genes, 0.6% | 1 | View Result |
| tight junction | 3 out of 628 genes, 0.5% | 157 out of 37603 genes, 0.4% | 1 | View Result |
| membrane coat | 3 out of 628 genes, 0.5% | 157 out of 37603 genes, 0.4% | 1 | View Result |
| coated membrane | 3 out of 628 genes, 0.5% | 157 out of 37603 genes, 0.4% | 1 | View Result |
| occluding junction | 3 out of 628 genes, 0.5% | 157 out of 37603 genes, 0.4% | 1 | View Result |
| DNA-directed RNA polymerase complex | 3 out of 628 genes, 0.5% | 159 out of 37603 genes, 0.4% | 1 | View Result |
| nuclear DNA-directed RNA polymerase complex | 3 out of 628 genes, 0.5% | 159 out of 37603 genes, 0.4% | 1 | View Result |
| adherens junction | 9 out of 628 genes, 1.4% | 518 out of 37603 genes, 1.4% | 1 | View Result |
| actomyosin | 2 out of 628 genes, 0.3% | 100 out of 37603 genes, 0.3% | 1 | View Result |
| myosin complex | 2 out of 628 genes, 0.3% | 101 out of 37603 genes, 0.3% | 1 | View Result |
| cell division site | 2 out of 628 genes, 0.3% | 102 out of 37603 genes, 0.3% | 1 | View Result |
| cell division site part | 2 out of 628 genes, 0.3% | 102 out of 37603 genes, 0.3% | 1 | View Result |
| microbody | 6 out of 628 genes, 1.0% | 344 out of 37603 genes, 0.9% | 1 | View Result |
| cortical cytoskeleton | 2 out of 628 genes, 0.3% | 103 out of 37603 genes, 0.3% | 1 | View Result |
| RNA polymerase complex | 3 out of 628 genes, 0.5% | 164 out of 37603 genes, 0.4% | 1 | View Result |
| ruffle | 5 out of 628 genes, 0.8% | 296 out of 37603 genes, 0.8% | 1 | View Result |
| mitochondrial matrix | 7 out of 628 genes, 1.1% | 422 out of 37603 genes, 1.1% | 1 | View Result |
| ruffle membrane | 2 out of 628 genes, 0.3% | 112 out of 37603 genes, 0.3% | 1 | View Result |
| synaptic membrane | 3 out of 628 genes, 0.5% | 184 out of 37603 genes, 0.5% | 1 | View Result |
| anchoring junction | 9 out of 628 genes, 1.4% | 581 out of 37603 genes, 1.5% | 1 | View Result |
| nuclear matrix | 2 out of 628 genes, 0.3% | 132 out of 37603 genes, 0.4% | 1 | View Result |
| microtubule organizing center part | 2 out of 628 genes, 0.3% | 138 out of 37603 genes, 0.4% | 1 | View Result |
| ion channel complex | 3 out of 628 genes, 0.5% | 208 out of 37603 genes, 0.6% | 1 | View Result |
| protein-DNA complex | 2 out of 628 genes, 0.3% | 141 out of 37603 genes, 0.4% | 1 | View Result |
| mitochondrial outer membrane | 3 out of 628 genes, 0.5% | 214 out of 37603 genes, 0.6% | 1 | View Result |
| nuclear membrane | 4 out of 628 genes, 0.6% | 287 out of 37603 genes, 0.8% | 1 | View Result |
| cell cortex | 5 out of 628 genes, 0.8% | 358 out of 37603 genes, 1.0% | 1 | View Result |
| PML body | 2 out of 628 genes, 0.3% | 150 out of 37603 genes, 0.4% | 1 | View Result |
| actin cytoskeleton | 12 out of 628 genes, 1.9% | 830 out of 37603 genes, 2.2% | 1 | View Result |
| Golgi stack | 2 out of 628 genes, 0.3% | 155 out of 37603 genes, 0.4% | 1 | View Result |
| extrinsic to plasma membrane | 2 out of 628 genes, 0.3% | 156 out of 37603 genes, 0.4% | 1 | View Result |
| postsynaptic membrane | 2 out of 628 genes, 0.3% | 157 out of 37603 genes, 0.4% | 1 | View Result |
| cell leading edge | 11 out of 628 genes, 1.8% | 771 out of 37603 genes, 2.1% | 1 | View Result |
| ubiquitin ligase complex | 4 out of 628 genes, 0.6% | 303 out of 37603 genes, 0.8% | 1 | View Result |
| axon part | 4 out of 628 genes, 0.6% | 304 out of 37603 genes, 0.8% | 1 | View Result |
| growth cone | 2 out of 628 genes, 0.3% | 162 out of 37603 genes, 0.4% | 1 | View Result |
| actin filament bundle | 2 out of 628 genes, 0.3% | 165 out of 37603 genes, 0.4% | 1 | View Result |
| cell cortex part | 2 out of 628 genes, 0.3% | 171 out of 37603 genes, 0.5% | 1 | View Result |
| cullin-RING ubiquitin ligase complex | 2 out of 628 genes, 0.3% | 173 out of 37603 genes, 0.5% | 1 | View Result |
| organelle outer membrane | 3 out of 628 genes, 0.5% | 251 out of 37603 genes, 0.7% | 1 | View Result |
| nuclear periphery | 3 out of 628 genes, 0.5% | 252 out of 37603 genes, 0.7% | 1 | View Result |
| apical junction complex | 4 out of 628 genes, 0.6% | 339 out of 37603 genes, 0.9% | 1 | View Result |
| outer membrane | 3 out of 628 genes, 0.5% | 271 out of 37603 genes, 0.7% | 1 | View Result |
| apicolateral plasma membrane | 4 out of 628 genes, 0.6% | 348 out of 37603 genes, 0.9% | 1 | View Result |
| nuclear envelope | 8 out of 628 genes, 1.3% | 638 out of 37603 genes, 1.7% | 1 | View Result |
| histone acetyltransferase complex | 2 out of 628 genes, 0.3% | 198 out of 37603 genes, 0.5% | 1 | View Result |
| microbody part | 2 out of 628 genes, 0.3% | 201 out of 37603 genes, 0.5% | 1 | View Result |
| peroxisomal part | 2 out of 628 genes, 0.3% | 201 out of 37603 genes, 0.5% | 1 | View Result |
| contractile fiber | 5 out of 628 genes, 0.8% | 441 out of 37603 genes, 1.2% | 1 | View Result |
| nucleolus | 29 out of 628 genes, 4.6% | 2075 out of 37603 genes, 5.5% | 1 | View Result |
| intracellular organelle part | 221 out of 628 genes, 35.2% | 13998 out of 37603 genes, 37.2% | 1 | View Result |
| nuclear speck | 2 out of 628 genes, 0.3% | 213 out of 37603 genes, 0.6% | 1 | View Result |
| organelle part | 234 out of 628 genes, 37.3% | 14834 out of 37603 genes, 39.4% | 1 | View Result |
| membrane-bounded organelle | 365 out of 628 genes, 58.1% | 22716 out of 37603 genes, 60.4% | 1 | View Result |
| intracellular membrane-bounded organelle | 360 out of 628 genes, 57.3% | 22524 out of 37603 genes, 59.9% | 1 | View Result |
| mitochondrial inner membrane | 6 out of 628 genes, 1.0% | 577 out of 37603 genes, 1.5% | 1 | View Result |
| contractile fiber part | 3 out of 628 genes, 0.5% | 339 out of 37603 genes, 0.9% | 1 | View Result |
| cytosolic part | 2 out of 628 genes, 0.3% | 259 out of 37603 genes, 0.7% | 1 | View Result |
| extrinsic to membrane | 2 out of 628 genes, 0.3% | 264 out of 37603 genes, 0.7% | 1 | View Result |
| mitochondrial envelope | 12 out of 628 genes, 1.9% | 1070 out of 37603 genes, 2.8% | 1 | View Result |
| insoluble fraction | 18 out of 628 genes, 2.9% | 1517 out of 37603 genes, 4.0% | 1 | View Result |
| mitochondrial membrane | 10 out of 628 genes, 1.6% | 943 out of 37603 genes, 2.5% | 1 | View Result |
| myofibril | 3 out of 628 genes, 0.5% | 395 out of 37603 genes, 1.1% | 1 | View Result |
| sarcomere | 2 out of 628 genes, 0.3% | 302 out of 37603 genes, 0.8% | 1 | View Result |
| site of polarized growth | 2 out of 628 genes, 0.3% | 311 out of 37603 genes, 0.8% | 1 | View Result |
| microtubule | 4 out of 628 genes, 0.6% | 499 out of 37603 genes, 1.3% | 1 | View Result |
| intracellular organelle | 394 out of 628 genes, 62.7% | 24874 out of 37603 genes, 66.1% | 1 | View Result |
| ribosome | 3 out of 628 genes, 0.5% | 410 out of 37603 genes, 1.1% | 1 | View Result |
| protein complex | 95 out of 628 genes, 15.1% | 6728 out of 37603 genes, 17.9% | 1 | View Result |
| spliceosomal complex | 2 out of 628 genes, 0.3% | 330 out of 37603 genes, 0.9% | 1 | View Result |
| nuclear chromatin | 2 out of 628 genes, 0.3% | 346 out of 37603 genes, 0.9% | 1 | View Result |
| mitochondrial part | 17 out of 628 genes, 2.7% | 1586 out of 37603 genes, 4.2% | 1 | View Result |
| organelle | 394 out of 628 genes, 62.7% | 25054 out of 37603 genes, 66.6% | 1 | View Result |
| cell fraction | 23 out of 628 genes, 3.7% | 2030 out of 37603 genes, 5.4% | 1 | View Result |
| centrosome | 5 out of 628 genes, 0.8% | 672 out of 37603 genes, 1.8% | 1 | View Result |
| organelle inner membrane | 8 out of 628 genes, 1.3% | 932 out of 37603 genes, 2.5% | 1 | View Result |
| organelle envelope | 21 out of 628 genes, 3.3% | 1940 out of 37603 genes, 5.2% | 1 | View Result |
| envelope | 21 out of 628 genes, 3.3% | 1966 out of 37603 genes, 5.2% | 1 | View Result |
| cytoskeletal part | 29 out of 628 genes, 4.6% | 2623 out of 37603 genes, 7.0% | 1 | View Result |
| nuclear chromosome part | 3 out of 628 genes, 0.5% | 547 out of 37603 genes, 1.5% | 1 | View Result |
| membrane-enclosed lumen | 94 out of 628 genes, 15.0% | 7219 out of 37603 genes, 19.2% | 1 | View Result |
| macromolecular complex | 113 out of 628 genes, 18.0% | 8498 out of 37603 genes, 22.6% | 1 | View Result |
| organelle lumen | 92 out of 628 genes, 14.6% | 7139 out of 37603 genes, 19.0% | 1 | View Result |
| chromatin | 4 out of 628 genes, 0.6% | 737 out of 37603 genes, 2.0% | 1 | View Result |
| cytoskeleton | 46 out of 628 genes, 7.3% | 4034 out of 37603 genes, 10.7% | 1 | View Result |
| nucleus | 141 out of 628 genes, 22.5% | 10441 out of 37603 genes, 27.8% | 1 | View Result |
| microtubule organizing center | 7 out of 628 genes, 1.1% | 1077 out of 37603 genes, 2.9% | 1 | View Result |
| transcription factor complex | 2 out of 628 genes, 0.3% | 555 out of 37603 genes, 1.5% | 1 | View Result |
| nuclear chromosome | 3 out of 628 genes, 0.5% | 677 out of 37603 genes, 1.8% | 1 | View Result |
| intracellular organelle lumen | 88 out of 628 genes, 14.0% | 7060 out of 37603 genes, 18.8% | 1 | View Result |
| nucleoplasm | 26 out of 628 genes, 4.1% | 2713 out of 37603 genes, 7.2% | 1 | View Result |
| nuclear body | 3 out of 628 genes, 0.5% | 729 out of 37603 genes, 1.9% | 1 | View Result |
| ribonucleoprotein complex | 9 out of 628 genes, 1.4% | 1367 out of 37603 genes, 3.6% | 1 | View Result |
| microtubule cytoskeleton | 14 out of 628 genes, 2.2% | 2086 out of 37603 genes, 5.5% | 1 | View Result |
| non-membrane-bounded organelle | 89 out of 628 genes, 14.2% | 7901 out of 37603 genes, 21.0% | 1 | View Result |
| intracellular non-membrane-bounded organelle | 89 out of 628 genes, 14.2% | 7901 out of 37603 genes, 21.0% | 1 | View Result |
| nucleoplasm part | 10 out of 628 genes, 1.6% | 1841 out of 37603 genes, 4.9% | 1 | View Result |
| chromosomal part | 6 out of 628 genes, 1.0% | 1456 out of 37603 genes, 3.9% | 1 | View Result |
| nuclear lumen | 62 out of 628 genes, 9.9% | 6408 out of 37603 genes, 17.0% | 1 | View Result |
| chromosome | 6 out of 628 genes, 1.0% | 1683 out of 37603 genes, 4.5% | 1 | View Result |
| nuclear part | 70 out of 628 genes, 11.1% | 7093 out of 37603 genes, 18.9% | 1 | View Result |

| Gene Ontology term | Genes annotated to the term |
| --- | --- |
| extracellular space | Unigene33441\_Mf\_liverA, CL3669.Contig2\_Mf\_liverA, Unigene28899\_Mf\_liverA, Unigene14603\_Mf\_liverA, Unigene9081\_Mf\_liverA, Unigene30815\_Mf\_liverA, Unigene34754\_Mf\_liverA, Unigene10351\_Mf\_liverA, Unigene19687\_Mf\_liverA, CL2339.Contig1\_Mf\_liverA, Unigene28143\_Mf\_liverA, Unigene1351\_Mf\_liverA, CL81.Contig1\_Mf\_liverA, Unigene37698\_Mf\_liverA, Unigene28564\_Mf\_liverA, Unigene1137\_Mf\_liverA, Unigene35958\_Mf\_liverA, Unigene36765\_Mf\_liverA, CL1175.Contig1\_Mf\_liverA, CL6038.Contig2\_Mf\_liverA, CL4583.Contig2\_Mf\_liverA, Unigene13894\_Mf\_liverA, Unigene38280\_Mf\_liverA, Unigene34375\_Mf\_liverA, Unigene37904\_Mf\_liverA, Unigene34983\_Mf\_liverA, Unigene28662\_Mf\_liverA, CL4299.Contig2\_Mf\_liverA, Unigene31333\_Mf\_liverA, NM\_010233, Unigene4630\_Mf\_liverA, Unigene29426\_Mf\_liverA, Unigene35283\_Mf\_liverA, CL2697.Contig4\_Mf\_liverA, Unigene1212\_Mf\_liverA, Unigene33683\_Mf\_liverA, Unigene32110\_Mf\_liverA, CL1372.Contig1\_Mf\_liverA, CL787.Contig1\_Mf\_liverA, Unigene33080\_Mf\_liverA, Unigene37454\_Mf\_liverA, Unigene33832\_Mf\_liverA, CL4338.Contig1\_Mf\_liverA, Unigene13616\_Mf\_liverA, Unigene34034\_Mf\_liverA, Unigene13143\_Mf\_liverA, Unigene4574\_Mf\_liverA, Unigene36698\_Mf\_liverA, Unigene25595\_Mf\_liverA, Unigene13498\_Mf\_liverA, Unigene13950\_Mf\_liverA, Unigene17579\_Mf\_liverA, Unigene41586\_Mf\_liverA, Unigene32789\_Mf\_liverA, Unigene37063\_Mf\_liverA, CL2333.Contig1\_Mf\_liverA, Unigene15290\_Mf\_liverA, Unigene32335\_Mf\_liverA, Unigene13296\_Mf\_liverA, Unigene30814\_Mf\_liverA, Unigene24157\_Mf\_liverA, CL5808.Contig1\_Mf\_liverA, CL3911.Contig2\_Mf\_liverA, Unigene32058\_Mf\_liverA, Unigene14541\_Mf\_liverA, CL6018.Contig1\_Mf\_liverA, CL4650.Contig2\_Mf\_liverA, Unigene38387\_Mf\_liverA, Unigene17569\_Mf\_liverA, Unigene32059\_Mf\_liverA |
| extracellular region | Unigene33441\_Mf\_liverA, NM\_177033, Unigene29628\_Mf\_liverA, Unigene4363\_Mf\_liverA, CL3669.Contig2\_Mf\_liverA, Unigene28899\_Mf\_liverA, Unigene14603\_Mf\_liverA, Unigene9081\_Mf\_liverA, Unigene30815\_Mf\_liverA, Unigene34754\_Mf\_liverA, Unigene10351\_Mf\_liverA, Unigene19687\_Mf\_liverA, Unigene33746\_Mf\_liverA, CL482.Contig1\_Mf\_liverA, CL2339.Contig1\_Mf\_liverA, Unigene28143\_Mf\_liverA, Unigene1351\_Mf\_liverA, CL81.Contig1\_Mf\_liverA, Unigene36762\_Mf\_liverA, Unigene37698\_Mf\_liverA, Unigene28564\_Mf\_liverA, Unigene34124\_Mf\_liverA, Unigene1137\_Mf\_liverA, Unigene35958\_Mf\_liverA, Unigene36765\_Mf\_liverA, CL1175.Contig1\_Mf\_liverA, CL6038.Contig2\_Mf\_liverA, CL4583.Contig2\_Mf\_liverA, CL485.Contig1\_Mf\_liverA, Unigene13894\_Mf\_liverA, CL4910.Contig1\_Mf\_liverA, Unigene32803\_Mf\_liverA, Unigene36328\_Mf\_liverA, Unigene38280\_Mf\_liverA, Unigene34375\_Mf\_liverA, Unigene37904\_Mf\_liverA, Unigene29082\_Mf\_liverA, Unigene20512\_Mf\_liverA, CL5978.Contig3\_Mf\_liverA, Unigene34983\_Mf\_liverA, Unigene14276\_Mf\_liverA, Unigene28662\_Mf\_liverA, CL4299.Contig2\_Mf\_liverA, Unigene31333\_Mf\_liverA, NM\_010233, Unigene11\_Mf\_liverA, Unigene4630\_Mf\_liverA, Unigene29426\_Mf\_liverA, NM\_009776, Unigene14609\_Mf\_liverA, Unigene30003\_Mf\_liverA, Unigene20371\_Mf\_liverA, Unigene35283\_Mf\_liverA, Unigene36430\_Mf\_liverA, Unigene393\_Mf\_liverA, Unigene14810\_Mf\_liverA, Unigene36987\_Mf\_liverA, CL2697.Contig4\_Mf\_liverA, Unigene1212\_Mf\_liverA, Unigene33683\_Mf\_liverA, Unigene30002\_Mf\_liverA, Unigene23328\_Mf\_liverA, Unigene36677\_Mf\_liverA, Unigene5134\_Mf\_liverA, CL787.Contig1\_Mf\_liverA, Unigene9150\_Mf\_liverA, Unigene32110\_Mf\_liverA, Unigene15055\_Mf\_liverA, CL1372.Contig1\_Mf\_liverA, CL3154.Contig1\_Mf\_liverA, Unigene33080\_Mf\_liverA, Unigene37454\_Mf\_liverA, Unigene33832\_Mf\_liverA, Unigene26380\_Mf\_liverA, CL4338.Contig1\_Mf\_liverA, Unigene13616\_Mf\_liverA, Unigene34034\_Mf\_liverA, CL4456.Contig1\_Mf\_liverA, CL5978.Contig2\_Mf\_liverA, Unigene13143\_Mf\_liverA, NM\_008483, Unigene4574\_Mf\_liverA, CL5293.Contig1\_Mf\_liverA, Unigene36698\_Mf\_liverA, Unigene26381\_Mf\_liverA, Unigene25595\_Mf\_liverA, Unigene27419\_Mf\_liverA, CL442.Contig5\_Mf\_liverA, Unigene32434\_Mf\_liverA, Unigene13498\_Mf\_liverA, Unigene28315\_Mf\_liverA, Unigene13950\_Mf\_liverA, Unigene30584\_Mf\_liverA, CL842.Contig1\_Mf\_liverA, Unigene17579\_Mf\_liverA, NM\_001081372, Unigene15290\_Mf\_liverA, CL2333.Contig1\_Mf\_liverA, Unigene37063\_Mf\_liverA, Unigene32789\_Mf\_liverA, Unigene41586\_Mf\_liverA, Unigene32908\_Mf\_liverA, Unigene36898\_Mf\_liverA, Unigene20372\_Mf\_liverA, Unigene32335\_Mf\_liverA, Unigene37104\_Mf\_liverA, Unigene14809\_Mf\_liverA, Unigene13296\_Mf\_liverA, Unigene30814\_Mf\_liverA, Unigene30839\_Mf\_liverA, Unigene138\_Mf\_liverA, CL5808.Contig1\_Mf\_liverA, Unigene24157\_Mf\_liverA, Unigene32058\_Mf\_liverA, CL3911.Contig2\_Mf\_liverA, Unigene14541\_Mf\_liverA, CL6018.Contig1\_Mf\_liverA, NM\_010776, CL4650.Contig2\_Mf\_liverA, CL442.Contig2\_Mf\_liverA, CL6039.Contig1\_Mf\_liverA, CL5827.Contig1\_Mf\_liverA, Unigene38387\_Mf\_liverA, Unigene17569\_Mf\_liverA, Unigene32059\_Mf\_liverA, Unigene37559\_Mf\_liverA, Unigene31492\_Mf\_liverA |
| cytoplasm | Unigene21684\_Mf\_liverA, Unigene27730\_Mf\_liverA, NM\_144940, CL3669.Contig2\_Mf\_liverA, Unigene29011\_Mf\_liverA, Unigene36172\_Mf\_liverA, Unigene36851\_Mf\_liverA, NM\_010378, NM\_011082, Unigene19687\_Mf\_liverA, CL854.Contig1\_Mf\_liverA, CL4105.Contig1\_Mf\_liverA, Unigene26053\_Mf\_liverA, Unigene7336\_Mf\_liverA, Unigene35507\_Mf\_liverA, CL238.Contig1\_Mf\_liverA, CL3617.Contig1\_Mf\_liverA, Unigene785\_Mf\_liverA, Unigene39886\_Mf\_liverA, Unigene15064\_Mf\_liverA, Unigene32920\_Mf\_liverA, Unigene34124\_Mf\_liverA, NR\_004446, Unigene36765\_Mf\_liverA, Unigene36434\_Mf\_liverA, Unigene34341\_Mf\_liverA, Unigene30153\_Mf\_liverA, Unigene30142\_Mf\_liverA, CL695.Contig1\_Mf\_liverA, Unigene34847\_Mf\_liverA, Unigene29082\_Mf\_liverA, NM\_011921, Unigene2\_Mf\_liverA, CL4919.Contig1\_Mf\_liverA, Unigene31623\_Mf\_liverA, CL2266.Contig1\_Mf\_liverA, NM\_010233, NM\_009776, Unigene33054\_Mf\_liverA, Unigene34394\_Mf\_liverA, Unigene30003\_Mf\_liverA, NM\_031165, Unigene25594\_Mf\_liverA, CL497.Contig2\_Mf\_liverA, Unigene36430\_Mf\_liverA, CL6.Contig1\_Mf\_liverA, Unigene18848\_Mf\_liverA, Unigene30002\_Mf\_liverA, NM\_016697, Unigene802\_Mf\_liverA, CL4816.Contig1\_Mf\_liverA, Unigene9150\_Mf\_liverA, Unigene15055\_Mf\_liverA, Unigene5639\_Mf\_liverA, CL3154.Contig1\_Mf\_liverA, CL5807.Contig1\_Mf\_liverA, Unigene32594\_Mf\_liverA, Unigene13363\_Mf\_liverA, Unigene743\_Mf\_liverA, Unigene37454\_Mf\_liverA, Unigene36417\_Mf\_liverA, CL2855.Contig2\_Mf\_liverA, CL4263.Contig1\_Mf\_liverA, NM\_021278, Unigene36175\_Mf\_liverA, CL4293.Contig1\_Mf\_liverA, Unigene1479\_Mf\_liverA, Unigene25976\_Mf\_liverA, NM\_009338, Unigene36698\_Mf\_liverA, NM\_145942, Unigene139\_Mf\_liverA, Unigene15572\_Mf\_liverA, Unigene31625\_Mf\_liverA, Unigene37018\_Mf\_liverA, NM\_022324, Unigene10756\_Mf\_liverA, Unigene34032\_Mf\_liverA, Unigene5057\_Mf\_liverA, Unigene27547\_Mf\_liverA, Unigene15290\_Mf\_liverA, Unigene24090\_Mf\_liverA, Unigene32789\_Mf\_liverA, Unigene12909\_Mf\_liverA, Unigene36898\_Mf\_liverA, CL5316.Contig1\_Mf\_liverA, Unigene35664\_Mf\_liverA, CL1688.Contig3\_Mf\_liverA, Unigene37389\_Mf\_liverA, Unigene32332\_Mf\_liverA, NR\_003630, CL3930.Contig1\_Mf\_liverA, Unigene41957\_Mf\_liverA, Unigene30839\_Mf\_liverA, Unigene24613\_Mf\_liverA, Unigene26611\_Mf\_liverA, CL532.Contig1\_Mf\_liverA, Unigene15477\_Mf\_liverA, Unigene35169\_Mf\_liverA, Unigene1280\_Mf\_liverA, Unigene30288\_Mf\_liverA, CL3166.Contig4\_Mf\_liverA, NM\_027959, Unigene34972\_Mf\_liverA, Unigene32059\_Mf\_liverA, Unigene4363\_Mf\_liverA, Unigene836\_Mf\_liverA, CL186.Contig3\_Mf\_liverA, CL425.Contig1\_Mf\_liverA, CL2797.Contig2\_Mf\_liverA, Unigene30832\_Mf\_liverA, CL4736.Contig1\_Mf\_liverA, Unigene28143\_Mf\_liverA, Unigene14916\_Mf\_liverA, Unigene37243\_Mf\_liverA, Unigene30217\_Mf\_liverA, Unigene15077\_Mf\_liverA, Unigene2050\_Mf\_liverA, CL4600.Contig1\_Mf\_liverA, Unigene16184\_Mf\_liverA, Unigene5693\_Mf\_liverA, CL3382.Contig1\_Mf\_liverA, Unigene35958\_Mf\_liverA, Unigene29876\_Mf\_liverA, Unigene28687\_Mf\_liverA, Unigene32803\_Mf\_liverA, Unigene5815\_Mf\_liverA, Unigene34375\_Mf\_liverA, CL1988.Contig3\_Mf\_liverA, CL1810.Contig1\_Mf\_liverA, CL4033.Contig1\_Mf\_liverA, Unigene34983\_Mf\_liverA, Unigene28662\_Mf\_liverA, CL2439.Contig1\_Mf\_liverA, CL3519.Contig1\_Mf\_liverA, Unigene33330\_Mf\_liverA, CL3933.Contig1\_Mf\_liverA, Unigene21026\_Mf\_liverA, Unigene20371\_Mf\_liverA, CL1325.Contig1\_Mf\_liverA, CL1493.Contig1\_Mf\_liverA, Unigene13683\_Mf\_liverA, Unigene13009\_Mf\_liverA, Unigene9698\_Mf\_liverA, Unigene36420\_Mf\_liverA, Unigene30004\_Mf\_liverA, Unigene542\_Mf\_liverA, Unigene35476\_Mf\_liverA, Unigene24758\_Mf\_liverA, Unigene24471\_Mf\_liverA, Unigene31571\_Mf\_liverA, CL5828.Contig2\_Mf\_liverA, Unigene35168\_Mf\_liverA, Unigene33080\_Mf\_liverA, Unigene15888\_Mf\_liverA, CL4220.Contig1\_Mf\_liverA, Unigene37262\_Mf\_liverA, CL4338.Contig1\_Mf\_liverA, Unigene12908\_Mf\_liverA, CL5052.Contig1\_Mf\_liverA, NM\_019817, Unigene5750\_Mf\_liverA, NM\_019879, NR\_033215, CL5293.Contig1\_Mf\_liverA, Unigene25595\_Mf\_liverA, Unigene14263\_Mf\_liverA, Unigene15470\_Mf\_liverA, Unigene14286\_Mf\_liverA, Unigene32434\_Mf\_liverA, CL4816.Contig3\_Mf\_liverA, Unigene34962\_Mf\_liverA, CL114.Contig1\_Mf\_liverA, Unigene39011\_Mf\_liverA, Unigene24225\_Mf\_liverA, Unigene37344\_Mf\_liverA, CL1588.Contig2\_Mf\_liverA, CL5275.Contig2\_Mf\_liverA, Unigene36189\_Mf\_liverA, NM\_008183, Unigene29558\_Mf\_liverA, Unigene13950\_Mf\_liverA, Unigene28459\_Mf\_liverA, Unigene37245\_Mf\_liverA, Unigene32133\_Mf\_liverA, Unigene281\_Mf\_liverA, NM\_177652, NM\_011978, Unigene10313\_Mf\_liverA, Unigene20372\_Mf\_liverA, Unigene37104\_Mf\_liverA, CL3104.Contig1\_Mf\_liverA, Unigene13018\_Mf\_liverA, Unigene36593\_Mf\_liverA, NM\_027406, Unigene36176\_Mf\_liverA, Unigene16529\_Mf\_liverA, Unigene37232\_Mf\_liverA, Unigene28609\_Mf\_liverA, CL5007.Contig1\_Mf\_liverA, CL5698.Contig1\_Mf\_liverA, Unigene14461\_Mf\_liverA, Unigene29889\_Mf\_liverA, Unigene2939\_Mf\_liverA, Unigene35618\_Mf\_liverA, CL5640.Contig1\_Mf\_liverA, Unigene35884\_Mf\_liverA, Unigene38015\_Mf\_liverA, NM\_028291, CL3835.Contig2\_Mf\_liverA, Unigene31199\_Mf\_liverA, Unigene6959\_Mf\_liverA, CL4550.Contig2\_Mf\_liverA, Unigene28899\_Mf\_liverA, Unigene28244\_Mf\_liverA, CL523.Contig1\_Mf\_liverA, NM\_008277, Unigene8473\_Mf\_liverA, CL482.Contig1\_Mf\_liverA, Unigene27071\_Mf\_liverA, Unigene36762\_Mf\_liverA, Unigene35382\_Mf\_liverA, Unigene600\_Mf\_liverA, NM\_011072, Unigene18795\_Mf\_liverA, Unigene28564\_Mf\_liverA, Unigene30878\_Mf\_liverA, Unigene40289\_Mf\_liverA, CL6038.Contig2\_Mf\_liverA, Unigene32570\_Mf\_liverA, Unigene37904\_Mf\_liverA, Unigene5333\_Mf\_liverA, Unigene15295\_Mf\_liverA, CL3483.Contig1\_Mf\_liverA, Unigene12999\_Mf\_liverA, Unigene34789\_Mf\_liverA, CL4141.Contig1\_Mf\_liverA, Unigene34184\_Mf\_liverA, CL5978.Contig3\_Mf\_liverA, CL1119.Contig1\_Mf\_liverA, Unigene13658\_Mf\_liverA, Unigene13593\_Mf\_liverA, Unigene11\_Mf\_liverA, Unigene32546\_Mf\_liverA, CL2622.Contig2\_Mf\_liverA, Unigene14609\_Mf\_liverA, Unigene843\_Mf\_liverA, Unigene25596\_Mf\_liverA, Unigene12997\_Mf\_liverA, CL1493.Contig2\_Mf\_liverA, CL2697.Contig4\_Mf\_liverA, CL2574.Contig1\_Mf\_liverA, NM\_024434, Unigene38104\_Mf\_liverA, Unigene23328\_Mf\_liverA, Unigene36626\_Mf\_liverA, CL787.Contig1\_Mf\_liverA, CL4076.Contig1\_Mf\_liverA, Unigene29490\_Mf\_liverA, CL3897.Contig5\_Mf\_liverA, Unigene36691\_Mf\_liverA, Unigene12967\_Mf\_liverA, CL2389.Contig1\_Mf\_liverA, CL3750.Contig2\_Mf\_liverA, CL335.Contig1\_Mf\_liverA, CL3599.Contig1\_Mf\_liverA, Unigene151\_Mf\_liverA, CL848.Contig2\_Mf\_liverA, CL784.Contig3\_Mf\_liverA, Unigene31639\_Mf\_liverA, CL838.Contig3\_Mf\_liverA, Unigene28520\_Mf\_liverA, Unigene5632\_Mf\_liverA, CL1263.Contig1\_Mf\_liverA, CL4550.Contig3\_Mf\_liverA, Unigene21816\_Mf\_liverA, CL258.Contig1\_Mf\_liverA, Unigene27419\_Mf\_liverA, NM\_008538, Unigene24323\_Mf\_liverA, CL2142.Contig2\_Mf\_liverA, Unigene21741\_Mf\_liverA, Unigene21742\_Mf\_liverA, Unigene13498\_Mf\_liverA, Unigene31427\_Mf\_liverA, CL529.Contig2\_Mf\_liverA, Unigene40069\_Mf\_liverA, Unigene30584\_Mf\_liverA, Unigene4510\_Mf\_liverA, Unigene17579\_Mf\_liverA, NM\_001081372, CL913.Contig1\_Mf\_liverA, Unigene34406\_Mf\_liverA, Unigene7195\_Mf\_liverA, Unigene35506\_Mf\_liverA, Unigene32058\_Mf\_liverA, Unigene27082\_Mf\_liverA, Unigene37178\_Mf\_liverA, Unigene5712\_Mf\_liverA, Unigene1205\_Mf\_liverA, Unigene36772\_Mf\_liverA, CL442.Contig2\_Mf\_liverA, CL6039.Contig1\_Mf\_liverA, CL2855.Contig1\_Mf\_liverA, CL4411.Contig4\_Mf\_liverA, NM\_013821, Unigene40796\_Mf\_liverA, Unigene31051\_Mf\_liverA, Unigene392\_Mf\_liverA, NM\_011732, NM\_009178, Unigene49971\_Mf\_liverA, Unigene38307\_Mf\_liverA, Unigene36190\_Mf\_liverA, Unigene34754\_Mf\_liverA, Unigene17042\_Mf\_liverA, Unigene34811\_Mf\_liverA, Unigene30154\_Mf\_liverA, Unigene3789\_Mf\_liverA, Unigene33746\_Mf\_liverA, CL1125.Contig1\_Mf\_liverA, CL4162.Contig1\_Mf\_liverA, Unigene1000\_Mf\_liverA, Unigene27922\_Mf\_liverA, Unigene15681\_Mf\_liverA, Unigene673\_Mf\_liverA, Unigene15476\_Mf\_liverA, CL1100.Contig1\_Mf\_liverA, Unigene32093\_Mf\_liverA, CL4583.Contig2\_Mf\_liverA, Unigene36414\_Mf\_liverA, CL485.Contig1\_Mf\_liverA, CL1988.Contig1\_Mf\_liverA, Unigene13894\_Mf\_liverA, Unigene5175\_Mf\_liverA, Unigene36328\_Mf\_liverA, Unigene38280\_Mf\_liverA, CL4745.Contig1\_Mf\_liverA, CL5309.Contig1\_Mf\_liverA, CL3002.Contig1\_Mf\_liverA, Unigene36315\_Mf\_liverA, Unigene31267\_Mf\_liverA, CL2327.Contig1\_Mf\_liverA, Unigene36949\_Mf\_liverA, CL887.Contig2\_Mf\_liverA, Unigene5488\_Mf\_liverA, CL2597.Contig1\_Mf\_liverA, Unigene33736\_Mf\_liverA, Unigene33523\_Mf\_liverA, Unigene35037\_Mf\_liverA, Unigene14810\_Mf\_liverA, CL3684.Contig2\_Mf\_liverA, CL1656.Contig1\_Mf\_liverA, Unigene1212\_Mf\_liverA, Unigene32233\_Mf\_liverA, CL838.Contig6\_Mf\_liverA, Unigene5607\_Mf\_liverA, CL4770.Contig1\_Mf\_liverA, Unigene24536\_Mf\_liverA, CL3750.Contig1\_Mf\_liverA, Unigene13196\_Mf\_liverA, CL114.Contig2\_Mf\_liverA, Unigene14449\_Mf\_liverA, CL1076.Contig1\_Mf\_liverA, Unigene39520\_Mf\_liverA, Unigene601\_Mf\_liverA, Unigene18796\_Mf\_liverA, Unigene22423\_Mf\_liverA, CL5307.Contig1\_Mf\_liverA, Unigene1327\_Mf\_liverA, Unigene31670\_Mf\_liverA, CL5978.Contig2\_Mf\_liverA, NM\_172203, CL442.Contig5\_Mf\_liverA, Unigene120\_Mf\_liverA, Unigene36418\_Mf\_liverA, Unigene15026\_Mf\_liverA, CL4007.Contig1\_Mf\_liverA, NM\_022997, NM\_001081116, Unigene550\_Mf\_liverA, CL55.Contig1\_Mf\_liverA, Unigene28489\_Mf\_liverA, Unigene37120\_Mf\_liverA, Unigene31198\_Mf\_liverA, CL2355.Contig1\_Mf\_liverA, Unigene4863\_Mf\_liverA, CL4984.Contig1\_Mf\_liverA, NM\_001081274, Unigene24537\_Mf\_liverA, Unigene32335\_Mf\_liverA, Unigene37153\_Mf\_liverA, Unigene14809\_Mf\_liverA, Unigene5489\_Mf\_liverA, Unigene34444\_Mf\_liverA, Unigene7399\_Mf\_liverA, Unigene35678\_Mf\_liverA, Unigene138\_Mf\_liverA, Unigene8560\_Mf\_liverA, CL3911.Contig2\_Mf\_liverA, Unigene5761\_Mf\_liverA, Unigene4681\_Mf\_liverA, Unigene12519\_Mf\_liverA, NM\_008851, Unigene37819\_Mf\_liverA, Unigene5886\_Mf\_liverA, CL336.Contig3\_Mf\_liverA, CL1198.Contig1\_Mf\_liverA |
| endoplasmic reticulum | Unigene29011\_Mf\_liverA, CL4550.Contig2\_Mf\_liverA, CL2797.Contig2\_Mf\_liverA, CL523.Contig1\_Mf\_liverA, Unigene30832\_Mf\_liverA, Unigene7336\_Mf\_liverA, CL482.Contig1\_Mf\_liverA, Unigene37243\_Mf\_liverA, Unigene30217\_Mf\_liverA, Unigene27922\_Mf\_liverA, Unigene35507\_Mf\_liverA, Unigene600\_Mf\_liverA, Unigene40289\_Mf\_liverA, Unigene34124\_Mf\_liverA, CL1100.Contig1\_Mf\_liverA, Unigene35958\_Mf\_liverA, Unigene36765\_Mf\_liverA, CL4583.Contig2\_Mf\_liverA, CL1988.Contig1\_Mf\_liverA, Unigene36328\_Mf\_liverA, Unigene34375\_Mf\_liverA, CL5309.Contig1\_Mf\_liverA, Unigene36315\_Mf\_liverA, Unigene34341\_Mf\_liverA, CL1988.Contig3\_Mf\_liverA, CL695.Contig1\_Mf\_liverA, Unigene36949\_Mf\_liverA, Unigene5488\_Mf\_liverA, CL887.Contig2\_Mf\_liverA, Unigene25596\_Mf\_liverA, Unigene21026\_Mf\_liverA, Unigene25594\_Mf\_liverA, CL1493.Contig2\_Mf\_liverA, CL1493.Contig1\_Mf\_liverA, CL6.Contig1\_Mf\_liverA, Unigene5607\_Mf\_liverA, CL2574.Contig1\_Mf\_liverA, Unigene23328\_Mf\_liverA, Unigene542\_Mf\_liverA, CL4816.Contig1\_Mf\_liverA, CL787.Contig1\_Mf\_liverA, Unigene15055\_Mf\_liverA, Unigene13196\_Mf\_liverA, CL114.Contig2\_Mf\_liverA, CL1076.Contig1\_Mf\_liverA, Unigene32594\_Mf\_liverA, Unigene15888\_Mf\_liverA, CL2855.Contig2\_Mf\_liverA, CL4263.Contig1\_Mf\_liverA, Unigene12967\_Mf\_liverA, CL4220.Contig1\_Mf\_liverA, CL5307.Contig1\_Mf\_liverA, CL784.Contig3\_Mf\_liverA, Unigene1479\_Mf\_liverA, CL838.Contig3\_Mf\_liverA, Unigene5632\_Mf\_liverA, CL4550.Contig3\_Mf\_liverA, Unigene25595\_Mf\_liverA, CL2142.Contig2\_Mf\_liverA, CL4816.Contig3\_Mf\_liverA, Unigene139\_Mf\_liverA, CL114.Contig1\_Mf\_liverA, Unigene37344\_Mf\_liverA, Unigene13498\_Mf\_liverA, NM\_022324, CL4007.Contig1\_Mf\_liverA, CL529.Contig2\_Mf\_liverA, Unigene30584\_Mf\_liverA, NM\_001081372, Unigene24090\_Mf\_liverA, Unigene32335\_Mf\_liverA, NM\_177652, Unigene37389\_Mf\_liverA, CL913.Contig1\_Mf\_liverA, Unigene34406\_Mf\_liverA, CL3104.Contig1\_Mf\_liverA, Unigene36593\_Mf\_liverA, Unigene5489\_Mf\_liverA, Unigene7195\_Mf\_liverA, Unigene34444\_Mf\_liverA, Unigene35506\_Mf\_liverA, Unigene32058\_Mf\_liverA, Unigene27082\_Mf\_liverA, CL3911.Contig2\_Mf\_liverA, Unigene5712\_Mf\_liverA, CL5698.Contig1\_Mf\_liverA, Unigene1280\_Mf\_liverA, Unigene1205\_Mf\_liverA, CL442.Contig2\_Mf\_liverA, CL3166.Contig4\_Mf\_liverA, CL2855.Contig1\_Mf\_liverA, Unigene5886\_Mf\_liverA, NM\_027959, CL4411.Contig4\_Mf\_liverA, CL336.Contig3\_Mf\_liverA, Unigene40796\_Mf\_liverA, Unigene35618\_Mf\_liverA, Unigene32059\_Mf\_liverA |
| endoplasmic reticulum part | Unigene29011\_Mf\_liverA, CL4550.Contig2\_Mf\_liverA, CL2797.Contig2\_Mf\_liverA, CL523.Contig1\_Mf\_liverA, Unigene7336\_Mf\_liverA, CL482.Contig1\_Mf\_liverA, Unigene30217\_Mf\_liverA, Unigene35507\_Mf\_liverA, Unigene600\_Mf\_liverA, Unigene34124\_Mf\_liverA, CL1100.Contig1\_Mf\_liverA, Unigene35958\_Mf\_liverA, CL4583.Contig2\_Mf\_liverA, CL1988.Contig1\_Mf\_liverA, Unigene34375\_Mf\_liverA, CL5309.Contig1\_Mf\_liverA, Unigene36315\_Mf\_liverA, Unigene34341\_Mf\_liverA, CL1988.Contig3\_Mf\_liverA, CL695.Contig1\_Mf\_liverA, Unigene36949\_Mf\_liverA, Unigene5488\_Mf\_liverA, Unigene25596\_Mf\_liverA, Unigene21026\_Mf\_liverA, CL6.Contig1\_Mf\_liverA, Unigene5607\_Mf\_liverA, CL2574.Contig1\_Mf\_liverA, Unigene23328\_Mf\_liverA, Unigene542\_Mf\_liverA, Unigene15055\_Mf\_liverA, CL4816.Contig1\_Mf\_liverA, CL114.Contig2\_Mf\_liverA, Unigene13196\_Mf\_liverA, CL1076.Contig1\_Mf\_liverA, Unigene15888\_Mf\_liverA, CL2855.Contig2\_Mf\_liverA, CL4263.Contig1\_Mf\_liverA, Unigene12967\_Mf\_liverA, CL4220.Contig1\_Mf\_liverA, CL5307.Contig1\_Mf\_liverA, CL784.Contig3\_Mf\_liverA, Unigene1479\_Mf\_liverA, Unigene5632\_Mf\_liverA, CL4550.Contig3\_Mf\_liverA, Unigene25595\_Mf\_liverA, CL4816.Contig3\_Mf\_liverA, Unigene139\_Mf\_liverA, CL114.Contig1\_Mf\_liverA, Unigene37344\_Mf\_liverA, Unigene13498\_Mf\_liverA, CL4007.Contig1\_Mf\_liverA, CL529.Contig2\_Mf\_liverA, NM\_001081372, Unigene24090\_Mf\_liverA, NM\_177652, Unigene32335\_Mf\_liverA, CL913.Contig1\_Mf\_liverA, Unigene37389\_Mf\_liverA, Unigene34406\_Mf\_liverA, Unigene36593\_Mf\_liverA, Unigene5489\_Mf\_liverA, Unigene7195\_Mf\_liverA, Unigene34444\_Mf\_liverA, Unigene35506\_Mf\_liverA, Unigene27082\_Mf\_liverA, CL3911.Contig2\_Mf\_liverA, Unigene5712\_Mf\_liverA, CL5698.Contig1\_Mf\_liverA, Unigene1280\_Mf\_liverA, Unigene1205\_Mf\_liverA, CL3166.Contig4\_Mf\_liverA, CL2855.Contig1\_Mf\_liverA, NM\_027959, Unigene5886\_Mf\_liverA, CL336.Contig3\_Mf\_liverA, Unigene40796\_Mf\_liverA |
| integral to membrane | NM\_007396, Unigene13808\_Mf\_liverA, Unigene21684\_Mf\_liverA, CL3835.Contig2\_Mf\_liverA, Unigene32891\_Mf\_liverA, Unigene26532\_Mf\_liverA, Unigene6959\_Mf\_liverA, CL4550.Contig2\_Mf\_liverA, Unigene15961\_Mf\_liverA, Unigene28244\_Mf\_liverA, Unigene24883\_Mf\_liverA, NM\_011082, Unigene19687\_Mf\_liverA, Unigene36849\_Mf\_liverA, Unigene26053\_Mf\_liverA, Unigene25055\_Mf\_liverA, Unigene7336\_Mf\_liverA, CL2339.Contig1\_Mf\_liverA, Unigene36762\_Mf\_liverA, Unigene35507\_Mf\_liverA, CL238.Contig1\_Mf\_liverA, Unigene35407\_Mf\_liverA, Unigene14816\_Mf\_liverA, Unigene37698\_Mf\_liverA, Unigene39886\_Mf\_liverA, Unigene30878\_Mf\_liverA, Unigene32920\_Mf\_liverA, Unigene27294\_Mf\_liverA, Unigene18500\_Mf\_liverA, CL4910.Contig1\_Mf\_liverA, CL1256.Contig1\_Mf\_liverA, Unigene34789\_Mf\_liverA, CL695.Contig1\_Mf\_liverA, NM\_008029, Unigene2\_Mf\_liverA, Unigene31333\_Mf\_liverA, Unigene13593\_Mf\_liverA, Unigene30369\_Mf\_liverA, Unigene33598\_Mf\_liverA, Unigene34394\_Mf\_liverA, Unigene31694\_Mf\_liverA, Unigene14609\_Mf\_liverA, Unigene843\_Mf\_liverA, NM\_001004357, Unigene28852\_Mf\_liverA, CL2697.Contig4\_Mf\_liverA, Unigene34143\_Mf\_liverA, CL2574.Contig1\_Mf\_liverA, Unigene27218\_Mf\_liverA, NM\_016697, CL2411.Contig1\_Mf\_liverA, CL787.Contig1\_Mf\_liverA, Unigene15055\_Mf\_liverA, CL3154.Contig1\_Mf\_liverA, CL5807.Contig1\_Mf\_liverA, Unigene13363\_Mf\_liverA, CL2855.Contig2\_Mf\_liverA, CL4293.Contig1\_Mf\_liverA, CL784.Contig3\_Mf\_liverA, Unigene1479\_Mf\_liverA, CL4456.Contig1\_Mf\_liverA, CL838.Contig3\_Mf\_liverA, Unigene5632\_Mf\_liverA, CL4550.Contig3\_Mf\_liverA, Unigene21816\_Mf\_liverA, CL2142.Contig2\_Mf\_liverA, Unigene7264\_Mf\_liverA, Unigene37420\_Mf\_liverA, CL153.Contig2\_Mf\_liverA, NM\_153589, CL4095.Contig4\_Mf\_liverA, CL529.Contig2\_Mf\_liverA, Unigene24166\_Mf\_liverA, Unigene25388\_Mf\_liverA, CL4283.Contig1\_Mf\_liverA, Unigene34032\_Mf\_liverA, Unigene27547\_Mf\_liverA, Unigene24090\_Mf\_liverA, Unigene32789\_Mf\_liverA, Unigene36898\_Mf\_liverA, CL5316.Contig1\_Mf\_liverA, Unigene37389\_Mf\_liverA, Unigene14070\_Mf\_liverA, Unigene32332\_Mf\_liverA, Unigene860\_Mf\_liverA, Unigene35506\_Mf\_liverA, Unigene32852\_Mf\_liverA, Unigene27082\_Mf\_liverA, Unigene5712\_Mf\_liverA, Unigene14541\_Mf\_liverA, Unigene1288\_Mf\_liverA, CL275.Contig5\_Mf\_liverA, Unigene15184\_Mf\_liverA, CL2855.Contig1\_Mf\_liverA, Unigene30697\_Mf\_liverA, Unigene33760\_Mf\_liverA, Unigene5169\_Mf\_liverA, Unigene40796\_Mf\_liverA, Unigene17569\_Mf\_liverA, Unigene31051\_Mf\_liverA, Unigene4363\_Mf\_liverA, Unigene42606\_Mf\_liverA, CL4228.Contig1\_Mf\_liverA, Unigene49971\_Mf\_liverA, CL425.Contig1\_Mf\_liverA, Unigene38307\_Mf\_liverA, CL3150.Contig1\_Mf\_liverA, CL2797.Contig2\_Mf\_liverA, Unigene34754\_Mf\_liverA, Unigene4697\_Mf\_liverA, Unigene33746\_Mf\_liverA, Unigene28143\_Mf\_liverA, Unigene37526\_Mf\_liverA, Unigene30217\_Mf\_liverA, Unigene28853\_Mf\_liverA, Unigene27922\_Mf\_liverA, CL4600.Contig1\_Mf\_liverA, CL5349.Contig1\_Mf\_liverA, CL3382.Contig1\_Mf\_liverA, CL1100.Contig1\_Mf\_liverA, Unigene35958\_Mf\_liverA, NM\_016689, CL4583.Contig2\_Mf\_liverA, CL485.Contig1\_Mf\_liverA, CL1988.Contig1\_Mf\_liverA, Unigene4938\_Mf\_liverA, Unigene32803\_Mf\_liverA, Unigene5292\_Mf\_liverA, NM\_010141, CL3002.Contig1\_Mf\_liverA, Unigene36315\_Mf\_liverA, Unigene31267\_Mf\_liverA, CL1988.Contig3\_Mf\_liverA, CL2327.Contig1\_Mf\_liverA, Unigene37224\_Mf\_liverA, Unigene36949\_Mf\_liverA, CL4033.Contig1\_Mf\_liverA, CL887.Contig2\_Mf\_liverA, Unigene5488\_Mf\_liverA, Unigene25994\_Mf\_liverA, Unigene33736\_Mf\_liverA, Unigene17916\_Mf\_liverA, Unigene29426\_Mf\_liverA, Unigene21026\_Mf\_liverA, Unigene35406\_Mf\_liverA, Unigene20371\_Mf\_liverA, CL1656.Contig1\_Mf\_liverA, CL838.Contig6\_Mf\_liverA, Unigene10915\_Mf\_liverA, Unigene24471\_Mf\_liverA, CL1076.Contig1\_Mf\_liverA, Unigene33080\_Mf\_liverA, Unigene15888\_Mf\_liverA, CL4220.Contig1\_Mf\_liverA, Unigene39437\_Mf\_liverA, CL4338.Contig1\_Mf\_liverA, Unigene15433\_Mf\_liverA, CL5307.Contig1\_Mf\_liverA, Unigene5750\_Mf\_liverA, Unigene31670\_Mf\_liverA, NM\_178405, Unigene13624\_Mf\_liverA, NM\_172203, Unigene30547\_Mf\_liverA, Unigene15470\_Mf\_liverA, Unigene39011\_Mf\_liverA, Unigene24225\_Mf\_liverA, Unigene37344\_Mf\_liverA, CL4007.Contig1\_Mf\_liverA, Unigene25052\_Mf\_liverA, Unigene13950\_Mf\_liverA, Unigene37245\_Mf\_liverA, Unigene33624\_Mf\_liverA, CL4984.Contig1\_Mf\_liverA, Unigene281\_Mf\_liverA, Unigene32335\_Mf\_liverA, Unigene20372\_Mf\_liverA, Unigene26951\_Mf\_liverA, Unigene30548\_Mf\_liverA, Unigene17842\_Mf\_liverA, CL3104.Contig1\_Mf\_liverA, Unigene36593\_Mf\_liverA, Unigene5489\_Mf\_liverA, Unigene34444\_Mf\_liverA, Unigene16529\_Mf\_liverA, Unigene37232\_Mf\_liverA, Unigene33720\_Mf\_liverA, CL5007.Contig1\_Mf\_liverA, CL5698.Contig1\_Mf\_liverA, Unigene37366\_Mf\_liverA, Unigene4681\_Mf\_liverA, CL2884.Contig2\_Mf\_liverA, Unigene37819\_Mf\_liverA, Unigene5886\_Mf\_liverA, Unigene35618\_Mf\_liverA |
| cell periphery | Unigene21684\_Mf\_liverA, CL5640.Contig1\_Mf\_liverA, CL4302.Contig1\_Mf\_liverA, Unigene29628\_Mf\_liverA, CL3835.Contig2\_Mf\_liverA, Unigene6959\_Mf\_liverA, Unigene28899\_Mf\_liverA, Unigene28244\_Mf\_liverA, Unigene24883\_Mf\_liverA, Unigene14603\_Mf\_liverA, NM\_010378, Unigene880\_Mf\_liverA, Unigene19687\_Mf\_liverA, Unigene36849\_Mf\_liverA, Unigene8473\_Mf\_liverA, Unigene26053\_Mf\_liverA, Unigene25055\_Mf\_liverA, CL2339.Contig1\_Mf\_liverA, Unigene36762\_Mf\_liverA, CL238.Contig1\_Mf\_liverA, CL3617.Contig1\_Mf\_liverA, Unigene18795\_Mf\_liverA, Unigene14816\_Mf\_liverA, Unigene37698\_Mf\_liverA, Unigene30878\_Mf\_liverA, Unigene1137\_Mf\_liverA, Unigene27294\_Mf\_liverA, NR\_004446, CL6038.Contig2\_Mf\_liverA, Unigene18500\_Mf\_liverA, Unigene32570\_Mf\_liverA, CL4910.Contig1\_Mf\_liverA, CL1256.Contig1\_Mf\_liverA, Unigene30153\_Mf\_liverA, Unigene15295\_Mf\_liverA, Unigene34789\_Mf\_liverA, Unigene31623\_Mf\_liverA, Unigene31333\_Mf\_liverA, Unigene13593\_Mf\_liverA, NM\_010233, Unigene30369\_Mf\_liverA, Unigene33598\_Mf\_liverA, Unigene11\_Mf\_liverA, Unigene4630\_Mf\_liverA, CL2622.Contig2\_Mf\_liverA, Unigene34394\_Mf\_liverA, Unigene14609\_Mf\_liverA, Unigene843\_Mf\_liverA, CL497.Contig2\_Mf\_liverA, NM\_001004357, Unigene28852\_Mf\_liverA, CL1493.Contig2\_Mf\_liverA, CL2697.Contig4\_Mf\_liverA, CL2574.Contig1\_Mf\_liverA, Unigene802\_Mf\_liverA, Unigene15055\_Mf\_liverA, CL3154.Contig1\_Mf\_liverA, CL5807.Contig1\_Mf\_liverA, Unigene13363\_Mf\_liverA, CL2855.Contig2\_Mf\_liverA, Unigene31988\_Mf\_liverA, Unigene13097\_Mf\_liverA, CL4293.Contig1\_Mf\_liverA, CL848.Contig2\_Mf\_liverA, CL4456.Contig1\_Mf\_liverA, CL838.Contig3\_Mf\_liverA, Unigene13143\_Mf\_liverA, CL1263.Contig1\_Mf\_liverA, CL258.Contig1\_Mf\_liverA, Unigene139\_Mf\_liverA, Unigene7264\_Mf\_liverA, CL153.Contig2\_Mf\_liverA, Unigene15572\_Mf\_liverA, Unigene31625\_Mf\_liverA, CL4095.Contig4\_Mf\_liverA, Unigene30584\_Mf\_liverA, CL4283.Contig1\_Mf\_liverA, Unigene17579\_Mf\_liverA, Unigene34032\_Mf\_liverA, Unigene27547\_Mf\_liverA, Unigene15290\_Mf\_liverA, Unigene32908\_Mf\_liverA, Unigene32789\_Mf\_liverA, Unigene36898\_Mf\_liverA, CL3930.Contig1\_Mf\_liverA, Unigene32852\_Mf\_liverA, Unigene24613\_Mf\_liverA, Unigene27082\_Mf\_liverA, Unigene26611\_Mf\_liverA, Unigene1288\_Mf\_liverA, NM\_010380, CL6039.Contig1\_Mf\_liverA, CL275.Contig5\_Mf\_liverA, CL2855.Contig1\_Mf\_liverA, Unigene30697\_Mf\_liverA, Unigene5169\_Mf\_liverA, Unigene17569\_Mf\_liverA, Unigene42606\_Mf\_liverA, CL186.Contig3\_Mf\_liverA, CL4228.Contig1\_Mf\_liverA, Unigene49971\_Mf\_liverA, CL425.Contig1\_Mf\_liverA, Unigene38307\_Mf\_liverA, Unigene36190\_Mf\_liverA, CL3150.Contig1\_Mf\_liverA, Unigene34754\_Mf\_liverA, Unigene4697\_Mf\_liverA, Unigene30154\_Mf\_liverA, Unigene33746\_Mf\_liverA, Unigene28143\_Mf\_liverA, Unigene37243\_Mf\_liverA, Unigene37526\_Mf\_liverA, Unigene28853\_Mf\_liverA, Unigene27922\_Mf\_liverA, CL4600.Contig1\_Mf\_liverA, Unigene15681\_Mf\_liverA, Unigene5693\_Mf\_liverA, Unigene35958\_Mf\_liverA, CL4583.Contig2\_Mf\_liverA, NM\_021423, CL485.Contig1\_Mf\_liverA, Unigene28687\_Mf\_liverA, Unigene4938\_Mf\_liverA, Unigene32803\_Mf\_liverA, Unigene36328\_Mf\_liverA, CL3002.Contig1\_Mf\_liverA, Unigene31267\_Mf\_liverA, CL1810.Contig1\_Mf\_liverA, CL2327.Contig1\_Mf\_liverA, Unigene37224\_Mf\_liverA, CL4033.Contig1\_Mf\_liverA, Unigene34983\_Mf\_liverA, Unigene28662\_Mf\_liverA, CL887.Contig2\_Mf\_liverA, Unigene33736\_Mf\_liverA, CL3519.Contig1\_Mf\_liverA, Unigene29426\_Mf\_liverA, Unigene35406\_Mf\_liverA, Unigene20371\_Mf\_liverA, CL1656.Contig1\_Mf\_liverA, CL1493.Contig1\_Mf\_liverA, CL838.Contig6\_Mf\_liverA, Unigene10915\_Mf\_liverA, Unigene24471\_Mf\_liverA, Unigene33080\_Mf\_liverA, Unigene39437\_Mf\_liverA, Unigene18796\_Mf\_liverA, CL4338.Contig1\_Mf\_liverA, Unigene22423\_Mf\_liverA, Unigene5750\_Mf\_liverA, NM\_178405, NM\_172203, Unigene30547\_Mf\_liverA, Unigene15470\_Mf\_liverA, Unigene39011\_Mf\_liverA, Unigene120\_Mf\_liverA, NM\_011207, Unigene36189\_Mf\_liverA, CL4007.Contig1\_Mf\_liverA, Unigene25052\_Mf\_liverA, Unigene13950\_Mf\_liverA, Unigene37245\_Mf\_liverA, CL2355.Contig1\_Mf\_liverA, Unigene32335\_Mf\_liverA, Unigene20372\_Mf\_liverA, Unigene30548\_Mf\_liverA, Unigene16529\_Mf\_liverA, Unigene37232\_Mf\_liverA, NM\_026313, CL3911.Contig2\_Mf\_liverA, Unigene44057\_Mf\_liverA, CL5007.Contig1\_Mf\_liverA, CL5698.Contig1\_Mf\_liverA, Unigene37366\_Mf\_liverA, Unigene4681\_Mf\_liverA, Unigene12519\_Mf\_liverA, CL2884.Contig2\_Mf\_liverA, CL591.Contig1\_Mf\_liverA, Unigene37819\_Mf\_liverA, Unigene5886\_Mf\_liverA, CL336.Contig3\_Mf\_liverA, Unigene35618\_Mf\_liverA, CL1198.Contig1\_Mf\_liverA |
| plasma membrane | Unigene21684\_Mf\_liverA, CL5640.Contig1\_Mf\_liverA, CL4302.Contig1\_Mf\_liverA, CL3835.Contig2\_Mf\_liverA, Unigene6959\_Mf\_liverA, Unigene28244\_Mf\_liverA, Unigene24883\_Mf\_liverA, Unigene14603\_Mf\_liverA, NM\_010378, Unigene880\_Mf\_liverA, Unigene19687\_Mf\_liverA, Unigene36849\_Mf\_liverA, Unigene8473\_Mf\_liverA, Unigene26053\_Mf\_liverA, Unigene25055\_Mf\_liverA, CL2339.Contig1\_Mf\_liverA, Unigene36762\_Mf\_liverA, CL238.Contig1\_Mf\_liverA, CL3617.Contig1\_Mf\_liverA, Unigene18795\_Mf\_liverA, Unigene14816\_Mf\_liverA, Unigene37698\_Mf\_liverA, Unigene30878\_Mf\_liverA, Unigene1137\_Mf\_liverA, Unigene27294\_Mf\_liverA, NR\_004446, CL6038.Contig2\_Mf\_liverA, Unigene18500\_Mf\_liverA, Unigene32570\_Mf\_liverA, CL4910.Contig1\_Mf\_liverA, CL1256.Contig1\_Mf\_liverA, Unigene30153\_Mf\_liverA, Unigene15295\_Mf\_liverA, Unigene34789\_Mf\_liverA, Unigene31623\_Mf\_liverA, Unigene31333\_Mf\_liverA, Unigene13593\_Mf\_liverA, NM\_010233, Unigene30369\_Mf\_liverA, Unigene33598\_Mf\_liverA, Unigene11\_Mf\_liverA, Unigene4630\_Mf\_liverA, CL2622.Contig2\_Mf\_liverA, Unigene34394\_Mf\_liverA, Unigene14609\_Mf\_liverA, Unigene843\_Mf\_liverA, CL497.Contig2\_Mf\_liverA, NM\_001004357, Unigene28852\_Mf\_liverA, CL1493.Contig2\_Mf\_liverA, CL2697.Contig4\_Mf\_liverA, CL2574.Contig1\_Mf\_liverA, Unigene802\_Mf\_liverA, Unigene15055\_Mf\_liverA, CL3154.Contig1\_Mf\_liverA, CL5807.Contig1\_Mf\_liverA, Unigene13363\_Mf\_liverA, CL2855.Contig2\_Mf\_liverA, Unigene31988\_Mf\_liverA, Unigene13097\_Mf\_liverA, CL4293.Contig1\_Mf\_liverA, CL4456.Contig1\_Mf\_liverA, CL838.Contig3\_Mf\_liverA, Unigene13143\_Mf\_liverA, CL1263.Contig1\_Mf\_liverA, CL258.Contig1\_Mf\_liverA, Unigene139\_Mf\_liverA, Unigene7264\_Mf\_liverA, CL153.Contig2\_Mf\_liverA, Unigene15572\_Mf\_liverA, Unigene31625\_Mf\_liverA, CL4095.Contig4\_Mf\_liverA, Unigene30584\_Mf\_liverA, CL4283.Contig1\_Mf\_liverA, Unigene17579\_Mf\_liverA, Unigene34032\_Mf\_liverA, Unigene27547\_Mf\_liverA, Unigene15290\_Mf\_liverA, Unigene32789\_Mf\_liverA, Unigene36898\_Mf\_liverA, CL3930.Contig1\_Mf\_liverA, Unigene32852\_Mf\_liverA, Unigene24613\_Mf\_liverA, Unigene27082\_Mf\_liverA, Unigene26611\_Mf\_liverA, Unigene1288\_Mf\_liverA, NM\_010380, CL6039.Contig1\_Mf\_liverA, CL275.Contig5\_Mf\_liverA, CL2855.Contig1\_Mf\_liverA, Unigene30697\_Mf\_liverA, Unigene5169\_Mf\_liverA, Unigene17569\_Mf\_liverA, Unigene42606\_Mf\_liverA, CL4228.Contig1\_Mf\_liverA, Unigene49971\_Mf\_liverA, CL425.Contig1\_Mf\_liverA, Unigene38307\_Mf\_liverA, Unigene36190\_Mf\_liverA, CL3150.Contig1\_Mf\_liverA, Unigene34754\_Mf\_liverA, Unigene4697\_Mf\_liverA, Unigene30154\_Mf\_liverA, Unigene33746\_Mf\_liverA, Unigene28143\_Mf\_liverA, Unigene37243\_Mf\_liverA, Unigene37526\_Mf\_liverA, Unigene28853\_Mf\_liverA, Unigene27922\_Mf\_liverA, CL4600.Contig1\_Mf\_liverA, Unigene15681\_Mf\_liverA, Unigene5693\_Mf\_liverA, Unigene35958\_Mf\_liverA, CL4583.Contig2\_Mf\_liverA, NM\_021423, CL485.Contig1\_Mf\_liverA, Unigene28687\_Mf\_liverA, Unigene4938\_Mf\_liverA, Unigene32803\_Mf\_liverA, Unigene36328\_Mf\_liverA, CL3002.Contig1\_Mf\_liverA, Unigene31267\_Mf\_liverA, CL1810.Contig1\_Mf\_liverA, CL2327.Contig1\_Mf\_liverA, Unigene37224\_Mf\_liverA, CL4033.Contig1\_Mf\_liverA, Unigene34983\_Mf\_liverA, Unigene28662\_Mf\_liverA, CL887.Contig2\_Mf\_liverA, Unigene33736\_Mf\_liverA, CL3519.Contig1\_Mf\_liverA, Unigene29426\_Mf\_liverA, Unigene35406\_Mf\_liverA, Unigene20371\_Mf\_liverA, CL1656.Contig1\_Mf\_liverA, CL1493.Contig1\_Mf\_liverA, CL838.Contig6\_Mf\_liverA, Unigene10915\_Mf\_liverA, Unigene24471\_Mf\_liverA, Unigene33080\_Mf\_liverA, Unigene39437\_Mf\_liverA, Unigene18796\_Mf\_liverA, CL4338.Contig1\_Mf\_liverA, Unigene22423\_Mf\_liverA, Unigene5750\_Mf\_liverA, NM\_178405, NM\_172203, Unigene30547\_Mf\_liverA, Unigene15470\_Mf\_liverA, Unigene39011\_Mf\_liverA, Unigene120\_Mf\_liverA, NM\_011207, Unigene36189\_Mf\_liverA, CL4007.Contig1\_Mf\_liverA, Unigene25052\_Mf\_liverA, Unigene13950\_Mf\_liverA, Unigene37245\_Mf\_liverA, CL2355.Contig1\_Mf\_liverA, Unigene32335\_Mf\_liverA, Unigene20372\_Mf\_liverA, Unigene30548\_Mf\_liverA, Unigene16529\_Mf\_liverA, Unigene37232\_Mf\_liverA, NM\_026313, CL3911.Contig2\_Mf\_liverA, Unigene44057\_Mf\_liverA, CL5007.Contig1\_Mf\_liverA, CL5698.Contig1\_Mf\_liverA, Unigene37366\_Mf\_liverA, Unigene4681\_Mf\_liverA, Unigene12519\_Mf\_liverA, CL2884.Contig2\_Mf\_liverA, CL591.Contig1\_Mf\_liverA, Unigene37819\_Mf\_liverA, Unigene5886\_Mf\_liverA, CL336.Contig3\_Mf\_liverA, Unigene35618\_Mf\_liverA, CL1198.Contig1\_Mf\_liverA |
| extracellular region part | Unigene33441\_Mf\_liverA, NM\_177033, Unigene29628\_Mf\_liverA, CL3669.Contig2\_Mf\_liverA, Unigene28899\_Mf\_liverA, Unigene14603\_Mf\_liverA, Unigene9081\_Mf\_liverA, Unigene30815\_Mf\_liverA, Unigene34754\_Mf\_liverA, Unigene10351\_Mf\_liverA, Unigene19687\_Mf\_liverA, CL482.Contig1\_Mf\_liverA, CL2339.Contig1\_Mf\_liverA, Unigene28143\_Mf\_liverA, Unigene1351\_Mf\_liverA, CL81.Contig1\_Mf\_liverA, Unigene37698\_Mf\_liverA, Unigene28564\_Mf\_liverA, Unigene34124\_Mf\_liverA, Unigene1137\_Mf\_liverA, Unigene35958\_Mf\_liverA, Unigene36765\_Mf\_liverA, CL1175.Contig1\_Mf\_liverA, CL6038.Contig2\_Mf\_liverA, CL4583.Contig2\_Mf\_liverA, Unigene13894\_Mf\_liverA, Unigene36328\_Mf\_liverA, Unigene38280\_Mf\_liverA, Unigene34375\_Mf\_liverA, Unigene37904\_Mf\_liverA, Unigene29082\_Mf\_liverA, Unigene20512\_Mf\_liverA, CL5978.Contig3\_Mf\_liverA, Unigene34983\_Mf\_liverA, Unigene28662\_Mf\_liverA, CL4299.Contig2\_Mf\_liverA, Unigene31333\_Mf\_liverA, NM\_010233, Unigene4630\_Mf\_liverA, Unigene29426\_Mf\_liverA, NM\_009776, Unigene35283\_Mf\_liverA, CL2697.Contig4\_Mf\_liverA, Unigene1212\_Mf\_liverA, Unigene33683\_Mf\_liverA, Unigene23328\_Mf\_liverA, Unigene32110\_Mf\_liverA, CL1372.Contig1\_Mf\_liverA, CL787.Contig1\_Mf\_liverA, Unigene33080\_Mf\_liverA, Unigene37454\_Mf\_liverA, Unigene33832\_Mf\_liverA, CL4338.Contig1\_Mf\_liverA, Unigene13616\_Mf\_liverA, Unigene34034\_Mf\_liverA, CL5978.Contig2\_Mf\_liverA, Unigene13143\_Mf\_liverA, NM\_008483, Unigene4574\_Mf\_liverA, Unigene36698\_Mf\_liverA, Unigene25595\_Mf\_liverA, CL442.Contig5\_Mf\_liverA, Unigene32434\_Mf\_liverA, Unigene13498\_Mf\_liverA, Unigene13950\_Mf\_liverA, Unigene30584\_Mf\_liverA, Unigene17579\_Mf\_liverA, NM\_001081372, Unigene15290\_Mf\_liverA, Unigene32789\_Mf\_liverA, Unigene37063\_Mf\_liverA, CL2333.Contig1\_Mf\_liverA, Unigene41586\_Mf\_liverA, Unigene32908\_Mf\_liverA, Unigene36898\_Mf\_liverA, Unigene32335\_Mf\_liverA, Unigene13296\_Mf\_liverA, Unigene30814\_Mf\_liverA, CL5808.Contig1\_Mf\_liverA, Unigene24157\_Mf\_liverA, Unigene32058\_Mf\_liverA, CL3911.Contig2\_Mf\_liverA, Unigene14541\_Mf\_liverA, CL6018.Contig1\_Mf\_liverA, NM\_010776, CL4650.Contig2\_Mf\_liverA, CL442.Contig2\_Mf\_liverA, CL6039.Contig1\_Mf\_liverA, Unigene38387\_Mf\_liverA, Unigene17569\_Mf\_liverA, Unigene32059\_Mf\_liverA, Unigene31492\_Mf\_liverA |
| endoplasmic reticulum membrane | CL4816.Contig1\_Mf\_liverA, Unigene15055\_Mf\_liverA, Unigene13196\_Mf\_liverA, CL114.Contig2\_Mf\_liverA, Unigene15888\_Mf\_liverA, CL2855.Contig2\_Mf\_liverA, Unigene12967\_Mf\_liverA, CL4220.Contig1\_Mf\_liverA, CL4550.Contig2\_Mf\_liverA, CL5307.Contig1\_Mf\_liverA, CL784.Contig3\_Mf\_liverA, CL2797.Contig2\_Mf\_liverA, Unigene1479\_Mf\_liverA, CL523.Contig1\_Mf\_liverA, Unigene5632\_Mf\_liverA, Unigene7336\_Mf\_liverA, CL482.Contig1\_Mf\_liverA, CL4550.Contig3\_Mf\_liverA, Unigene30217\_Mf\_liverA, Unigene35507\_Mf\_liverA, CL4816.Contig3\_Mf\_liverA, Unigene139\_Mf\_liverA, CL114.Contig1\_Mf\_liverA, Unigene37344\_Mf\_liverA, CL4007.Contig1\_Mf\_liverA, CL1100.Contig1\_Mf\_liverA, CL529.Contig2\_Mf\_liverA, CL4583.Contig2\_Mf\_liverA, CL1988.Contig1\_Mf\_liverA, Unigene24090\_Mf\_liverA, Unigene36315\_Mf\_liverA, Unigene34341\_Mf\_liverA, Unigene32335\_Mf\_liverA, NM\_177652, CL1988.Contig3\_Mf\_liverA, Unigene37389\_Mf\_liverA, CL695.Contig1\_Mf\_liverA, Unigene36593\_Mf\_liverA, Unigene36949\_Mf\_liverA, Unigene5489\_Mf\_liverA, Unigene7195\_Mf\_liverA, Unigene34444\_Mf\_liverA, Unigene5488\_Mf\_liverA, Unigene35506\_Mf\_liverA, Unigene27082\_Mf\_liverA, Unigene21026\_Mf\_liverA, Unigene5712\_Mf\_liverA, CL5698.Contig1\_Mf\_liverA, Unigene1280\_Mf\_liverA, Unigene1205\_Mf\_liverA, CL3166.Contig4\_Mf\_liverA, CL6.Contig1\_Mf\_liverA, Unigene5607\_Mf\_liverA, CL2855.Contig1\_Mf\_liverA, Unigene5886\_Mf\_liverA, CL2574.Contig1\_Mf\_liverA, CL336.Contig3\_Mf\_liverA, Unigene40796\_Mf\_liverA, Unigene542\_Mf\_liverA |
| MHC protein complex | Unigene37245\_Mf\_liverA, Unigene4938\_Mf\_liverA, Unigene24471\_Mf\_liverA, CL3835.Contig2\_Mf\_liverA, CL3002.Contig1\_Mf\_liverA, CL2855.Contig2\_Mf\_liverA, Unigene28244\_Mf\_liverA, NM\_010378, Unigene36849\_Mf\_liverA, Unigene13143\_Mf\_liverA, Unigene4681\_Mf\_liverA, CL275.Contig5\_Mf\_liverA, NM\_010380, CL2855.Contig1\_Mf\_liverA, Unigene30878\_Mf\_liverA, NR\_004446 |
| nuclear outer membrane-endoplasmic reticulum membrane network | CL4816.Contig1\_Mf\_liverA, Unigene15055\_Mf\_liverA, Unigene13196\_Mf\_liverA, CL114.Contig2\_Mf\_liverA, Unigene15888\_Mf\_liverA, CL2855.Contig2\_Mf\_liverA, Unigene12967\_Mf\_liverA, CL4220.Contig1\_Mf\_liverA, CL4550.Contig2\_Mf\_liverA, CL5307.Contig1\_Mf\_liverA, CL784.Contig3\_Mf\_liverA, CL2797.Contig2\_Mf\_liverA, Unigene1479\_Mf\_liverA, CL523.Contig1\_Mf\_liverA, Unigene5632\_Mf\_liverA, Unigene7336\_Mf\_liverA, CL482.Contig1\_Mf\_liverA, CL4550.Contig3\_Mf\_liverA, Unigene30217\_Mf\_liverA, Unigene35507\_Mf\_liverA, CL4816.Contig3\_Mf\_liverA, Unigene139\_Mf\_liverA, CL114.Contig1\_Mf\_liverA, Unigene37344\_Mf\_liverA, CL4007.Contig1\_Mf\_liverA, CL1100.Contig1\_Mf\_liverA, CL529.Contig2\_Mf\_liverA, CL4583.Contig2\_Mf\_liverA, CL1988.Contig1\_Mf\_liverA, Unigene24090\_Mf\_liverA, Unigene36315\_Mf\_liverA, Unigene34341\_Mf\_liverA, Unigene32335\_Mf\_liverA, NM\_177652, CL1988.Contig3\_Mf\_liverA, Unigene37389\_Mf\_liverA, CL695.Contig1\_Mf\_liverA, Unigene36593\_Mf\_liverA, Unigene36949\_Mf\_liverA, Unigene5489\_Mf\_liverA, Unigene7195\_Mf\_liverA, Unigene34444\_Mf\_liverA, Unigene5488\_Mf\_liverA, Unigene35506\_Mf\_liverA, Unigene27082\_Mf\_liverA, Unigene21026\_Mf\_liverA, Unigene5712\_Mf\_liverA, CL5698.Contig1\_Mf\_liverA, Unigene1280\_Mf\_liverA, Unigene1205\_Mf\_liverA, CL3166.Contig4\_Mf\_liverA, CL6.Contig1\_Mf\_liverA, Unigene5607\_Mf\_liverA, CL2855.Contig1\_Mf\_liverA, Unigene5886\_Mf\_liverA, CL2574.Contig1\_Mf\_liverA, CL336.Contig3\_Mf\_liverA, Unigene40796\_Mf\_liverA, Unigene542\_Mf\_liverA |
| cytoplasmic part | Unigene21684\_Mf\_liverA, Unigene27730\_Mf\_liverA, NM\_144940, CL3669.Contig2\_Mf\_liverA, Unigene29011\_Mf\_liverA, Unigene36172\_Mf\_liverA, Unigene36851\_Mf\_liverA, NM\_010378, NM\_011082, Unigene19687\_Mf\_liverA, CL854.Contig1\_Mf\_liverA, CL4105.Contig1\_Mf\_liverA, Unigene26053\_Mf\_liverA, Unigene7336\_Mf\_liverA, Unigene35507\_Mf\_liverA, Unigene39886\_Mf\_liverA, Unigene15064\_Mf\_liverA, Unigene32920\_Mf\_liverA, Unigene34124\_Mf\_liverA, NR\_004446, Unigene36765\_Mf\_liverA, Unigene34341\_Mf\_liverA, Unigene30142\_Mf\_liverA, CL695.Contig1\_Mf\_liverA, Unigene29082\_Mf\_liverA, NM\_011921, Unigene2\_Mf\_liverA, CL4919.Contig1\_Mf\_liverA, Unigene31623\_Mf\_liverA, NM\_010233, NM\_009776, Unigene34394\_Mf\_liverA, Unigene30003\_Mf\_liverA, NM\_031165, Unigene25594\_Mf\_liverA, CL497.Contig2\_Mf\_liverA, Unigene36430\_Mf\_liverA, CL6.Contig1\_Mf\_liverA, Unigene18848\_Mf\_liverA, Unigene30002\_Mf\_liverA, NM\_016697, CL4816.Contig1\_Mf\_liverA, Unigene15055\_Mf\_liverA, Unigene5639\_Mf\_liverA, CL3154.Contig1\_Mf\_liverA, CL5807.Contig1\_Mf\_liverA, Unigene32594\_Mf\_liverA, Unigene13363\_Mf\_liverA, Unigene743\_Mf\_liverA, CL2855.Contig2\_Mf\_liverA, CL4263.Contig1\_Mf\_liverA, NM\_021278, Unigene1479\_Mf\_liverA, NM\_009338, Unigene36698\_Mf\_liverA, NM\_145942, Unigene139\_Mf\_liverA, Unigene15572\_Mf\_liverA, Unigene31625\_Mf\_liverA, Unigene37018\_Mf\_liverA, NM\_022324, Unigene34032\_Mf\_liverA, Unigene5057\_Mf\_liverA, Unigene27547\_Mf\_liverA, Unigene15290\_Mf\_liverA, Unigene24090\_Mf\_liverA, Unigene32789\_Mf\_liverA, Unigene12909\_Mf\_liverA, Unigene36898\_Mf\_liverA, CL5316.Contig1\_Mf\_liverA, Unigene35664\_Mf\_liverA, CL1688.Contig3\_Mf\_liverA, Unigene37389\_Mf\_liverA, Unigene32332\_Mf\_liverA, NR\_003630, CL3930.Contig1\_Mf\_liverA, Unigene26611\_Mf\_liverA, CL532.Contig1\_Mf\_liverA, Unigene15477\_Mf\_liverA, Unigene1280\_Mf\_liverA, Unigene30288\_Mf\_liverA, CL3166.Contig4\_Mf\_liverA, NM\_027959, Unigene32059\_Mf\_liverA, Unigene4363\_Mf\_liverA, Unigene836\_Mf\_liverA, CL186.Contig3\_Mf\_liverA, CL425.Contig1\_Mf\_liverA, CL2797.Contig2\_Mf\_liverA, Unigene30832\_Mf\_liverA, Unigene28143\_Mf\_liverA, Unigene14916\_Mf\_liverA, Unigene37243\_Mf\_liverA, Unigene30217\_Mf\_liverA, Unigene15077\_Mf\_liverA, CL4600.Contig1\_Mf\_liverA, CL3382.Contig1\_Mf\_liverA, Unigene5693\_Mf\_liverA, Unigene35958\_Mf\_liverA, Unigene29876\_Mf\_liverA, Unigene28687\_Mf\_liverA, Unigene5815\_Mf\_liverA, Unigene34375\_Mf\_liverA, CL1988.Contig3\_Mf\_liverA, CL1810.Contig1\_Mf\_liverA, CL4033.Contig1\_Mf\_liverA, Unigene28662\_Mf\_liverA, Unigene33330\_Mf\_liverA, CL3933.Contig1\_Mf\_liverA, Unigene21026\_Mf\_liverA, Unigene20371\_Mf\_liverA, CL1493.Contig1\_Mf\_liverA, Unigene13683\_Mf\_liverA, Unigene13009\_Mf\_liverA, Unigene30004\_Mf\_liverA, Unigene542\_Mf\_liverA, Unigene35476\_Mf\_liverA, Unigene24758\_Mf\_liverA, Unigene24471\_Mf\_liverA, CL5828.Contig2\_Mf\_liverA, Unigene33080\_Mf\_liverA, Unigene15888\_Mf\_liverA, CL4220.Contig1\_Mf\_liverA, Unigene37262\_Mf\_liverA, CL4338.Contig1\_Mf\_liverA, Unigene12908\_Mf\_liverA, NM\_019817, CL5052.Contig1\_Mf\_liverA, Unigene5750\_Mf\_liverA, NM\_019879, NR\_033215, Unigene25595\_Mf\_liverA, Unigene15470\_Mf\_liverA, Unigene14286\_Mf\_liverA, CL4816.Contig3\_Mf\_liverA, Unigene34962\_Mf\_liverA, CL114.Contig1\_Mf\_liverA, Unigene39011\_Mf\_liverA, Unigene24225\_Mf\_liverA, Unigene37344\_Mf\_liverA, CL5275.Contig2\_Mf\_liverA, Unigene36189\_Mf\_liverA, NM\_008183, Unigene29558\_Mf\_liverA, Unigene13950\_Mf\_liverA, Unigene28459\_Mf\_liverA, Unigene37245\_Mf\_liverA, Unigene32133\_Mf\_liverA, Unigene281\_Mf\_liverA, NM\_177652, Unigene20372\_Mf\_liverA, Unigene10313\_Mf\_liverA, NM\_011978, Unigene37104\_Mf\_liverA, CL3104.Contig1\_Mf\_liverA, Unigene36593\_Mf\_liverA, NM\_027406, Unigene16529\_Mf\_liverA, Unigene37232\_Mf\_liverA, CL5007.Contig1\_Mf\_liverA, CL5698.Contig1\_Mf\_liverA, Unigene14461\_Mf\_liverA, Unigene2939\_Mf\_liverA, Unigene35618\_Mf\_liverA, CL5640.Contig1\_Mf\_liverA, Unigene35884\_Mf\_liverA, Unigene38015\_Mf\_liverA, NM\_028291, CL3835.Contig2\_Mf\_liverA, Unigene31199\_Mf\_liverA, Unigene6959\_Mf\_liverA, CL4550.Contig2\_Mf\_liverA, Unigene28899\_Mf\_liverA, Unigene28244\_Mf\_liverA, CL523.Contig1\_Mf\_liverA, NM\_008277, CL482.Contig1\_Mf\_liverA, Unigene27071\_Mf\_liverA, Unigene35382\_Mf\_liverA, Unigene36762\_Mf\_liverA, Unigene600\_Mf\_liverA, NM\_011072, Unigene30878\_Mf\_liverA, Unigene40289\_Mf\_liverA, CL6038.Contig2\_Mf\_liverA, Unigene32570\_Mf\_liverA, Unigene5333\_Mf\_liverA, Unigene15295\_Mf\_liverA, Unigene12999\_Mf\_liverA, Unigene34789\_Mf\_liverA, CL4141.Contig1\_Mf\_liverA, Unigene34184\_Mf\_liverA, CL5978.Contig3\_Mf\_liverA, CL1119.Contig1\_Mf\_liverA, Unigene13658\_Mf\_liverA, Unigene13593\_Mf\_liverA, Unigene11\_Mf\_liverA, Unigene14609\_Mf\_liverA, Unigene843\_Mf\_liverA, Unigene25596\_Mf\_liverA, CL1493.Contig2\_Mf\_liverA, CL2697.Contig4\_Mf\_liverA, CL2574.Contig1\_Mf\_liverA, NM\_024434, Unigene38104\_Mf\_liverA, Unigene23328\_Mf\_liverA, Unigene36626\_Mf\_liverA, CL787.Contig1\_Mf\_liverA, Unigene29490\_Mf\_liverA, CL3897.Contig5\_Mf\_liverA, Unigene36691\_Mf\_liverA, Unigene12967\_Mf\_liverA, CL2389.Contig1\_Mf\_liverA, CL3750.Contig2\_Mf\_liverA, CL3599.Contig1\_Mf\_liverA, Unigene151\_Mf\_liverA, CL848.Contig2\_Mf\_liverA, CL784.Contig3\_Mf\_liverA, Unigene31639\_Mf\_liverA, CL838.Contig3\_Mf\_liverA, Unigene5632\_Mf\_liverA, CL1263.Contig1\_Mf\_liverA, CL4550.Contig3\_Mf\_liverA, CL258.Contig1\_Mf\_liverA, NM\_008538, Unigene24323\_Mf\_liverA, CL2142.Contig2\_Mf\_liverA, Unigene21741\_Mf\_liverA, Unigene21742\_Mf\_liverA, Unigene13498\_Mf\_liverA, Unigene31427\_Mf\_liverA, CL529.Contig2\_Mf\_liverA, Unigene40069\_Mf\_liverA, Unigene4510\_Mf\_liverA, Unigene30584\_Mf\_liverA, NM\_001081372, CL913.Contig1\_Mf\_liverA, Unigene34406\_Mf\_liverA, Unigene7195\_Mf\_liverA, Unigene35506\_Mf\_liverA, Unigene27082\_Mf\_liverA, Unigene37178\_Mf\_liverA, Unigene32058\_Mf\_liverA, Unigene5712\_Mf\_liverA, Unigene1205\_Mf\_liverA, Unigene36772\_Mf\_liverA, CL442.Contig2\_Mf\_liverA, CL6039.Contig1\_Mf\_liverA, CL2855.Contig1\_Mf\_liverA, CL4411.Contig4\_Mf\_liverA, NM\_013821, Unigene40796\_Mf\_liverA, Unigene31051\_Mf\_liverA, NM\_011732, NM\_009178, Unigene49971\_Mf\_liverA, Unigene38307\_Mf\_liverA, Unigene36190\_Mf\_liverA, Unigene34754\_Mf\_liverA, Unigene34811\_Mf\_liverA, Unigene33746\_Mf\_liverA, Unigene30154\_Mf\_liverA, CL1125.Contig1\_Mf\_liverA, CL4162.Contig1\_Mf\_liverA, Unigene1000\_Mf\_liverA, Unigene27922\_Mf\_liverA, Unigene15681\_Mf\_liverA, Unigene673\_Mf\_liverA, Unigene15476\_Mf\_liverA, CL1100.Contig1\_Mf\_liverA, Unigene32093\_Mf\_liverA, CL4583.Contig2\_Mf\_liverA, CL1988.Contig1\_Mf\_liverA, Unigene36328\_Mf\_liverA, Unigene38280\_Mf\_liverA, CL4745.Contig1\_Mf\_liverA, CL3002.Contig1\_Mf\_liverA, CL5309.Contig1\_Mf\_liverA, Unigene36315\_Mf\_liverA, Unigene31267\_Mf\_liverA, Unigene36949\_Mf\_liverA, CL887.Contig2\_Mf\_liverA, Unigene5488\_Mf\_liverA, Unigene33736\_Mf\_liverA, Unigene33523\_Mf\_liverA, Unigene35037\_Mf\_liverA, Unigene14810\_Mf\_liverA, CL3684.Contig2\_Mf\_liverA, Unigene1212\_Mf\_liverA, Unigene32233\_Mf\_liverA, CL838.Contig6\_Mf\_liverA, Unigene5607\_Mf\_liverA, Unigene24536\_Mf\_liverA, CL3750.Contig1\_Mf\_liverA, CL114.Contig2\_Mf\_liverA, Unigene13196\_Mf\_liverA, Unigene601\_Mf\_liverA, CL1076.Contig1\_Mf\_liverA, Unigene22423\_Mf\_liverA, CL5307.Contig1\_Mf\_liverA, Unigene31670\_Mf\_liverA, CL5978.Contig2\_Mf\_liverA, NM\_172203, CL442.Contig5\_Mf\_liverA, Unigene120\_Mf\_liverA, Unigene15026\_Mf\_liverA, CL4007.Contig1\_Mf\_liverA, NM\_022997, NM\_001081116, CL55.Contig1\_Mf\_liverA, Unigene550\_Mf\_liverA, Unigene31198\_Mf\_liverA, Unigene37120\_Mf\_liverA, CL4984.Contig1\_Mf\_liverA, NM\_001081274, Unigene24537\_Mf\_liverA, Unigene4863\_Mf\_liverA, CL2355.Contig1\_Mf\_liverA, Unigene32335\_Mf\_liverA, Unigene37153\_Mf\_liverA, Unigene14809\_Mf\_liverA, Unigene5489\_Mf\_liverA, Unigene34444\_Mf\_liverA, Unigene35678\_Mf\_liverA, Unigene138\_Mf\_liverA, Unigene8560\_Mf\_liverA, CL3911.Contig2\_Mf\_liverA, Unigene5761\_Mf\_liverA, Unigene4681\_Mf\_liverA, NM\_008851, Unigene37819\_Mf\_liverA, Unigene5886\_Mf\_liverA, CL336.Contig3\_Mf\_liverA |
| integral to plasma membrane | Unigene42606\_Mf\_liverA, CL4228.Contig1\_Mf\_liverA, CL425.Contig1\_Mf\_liverA, Unigene49971\_Mf\_liverA, Unigene38307\_Mf\_liverA, Unigene24883\_Mf\_liverA, CL3150.Contig1\_Mf\_liverA, Unigene25055\_Mf\_liverA, Unigene33746\_Mf\_liverA, CL2339.Contig1\_Mf\_liverA, Unigene36762\_Mf\_liverA, Unigene28853\_Mf\_liverA, CL238.Contig1\_Mf\_liverA, CL4600.Contig1\_Mf\_liverA, Unigene18500\_Mf\_liverA, CL485.Contig1\_Mf\_liverA, CL4910.Contig1\_Mf\_liverA, Unigene32803\_Mf\_liverA, CL1256.Contig1\_Mf\_liverA, Unigene34789\_Mf\_liverA, Unigene37224\_Mf\_liverA, Unigene13593\_Mf\_liverA, Unigene30369\_Mf\_liverA, Unigene33736\_Mf\_liverA, Unigene33598\_Mf\_liverA, Unigene29426\_Mf\_liverA, Unigene14609\_Mf\_liverA, Unigene843\_Mf\_liverA, Unigene35406\_Mf\_liverA, Unigene28852\_Mf\_liverA, NM\_001004357, Unigene20371\_Mf\_liverA, CL1656.Contig1\_Mf\_liverA, CL838.Contig6\_Mf\_liverA, Unigene15055\_Mf\_liverA, CL3154.Contig1\_Mf\_liverA, Unigene13363\_Mf\_liverA, CL2855.Contig2\_Mf\_liverA, CL4293.Contig1\_Mf\_liverA, CL4338.Contig1\_Mf\_liverA, CL4456.Contig1\_Mf\_liverA, Unigene5750\_Mf\_liverA, CL838.Contig3\_Mf\_liverA, NM\_178405, NM\_172203, Unigene30547\_Mf\_liverA, Unigene15470\_Mf\_liverA, Unigene39011\_Mf\_liverA, CL153.Contig2\_Mf\_liverA, Unigene25052\_Mf\_liverA, Unigene13950\_Mf\_liverA, Unigene36898\_Mf\_liverA, Unigene20372\_Mf\_liverA, Unigene16529\_Mf\_liverA, Unigene37232\_Mf\_liverA, Unigene27082\_Mf\_liverA, Unigene37366\_Mf\_liverA, Unigene1288\_Mf\_liverA, CL2884.Contig2\_Mf\_liverA, Unigene30697\_Mf\_liverA, CL2855.Contig1\_Mf\_liverA, Unigene5169\_Mf\_liverA, Unigene35618\_Mf\_liverA, Unigene17569\_Mf\_liverA |
| external side of plasma membrane | CL4583.Contig2\_Mf\_liverA, Unigene37245\_Mf\_liverA, Unigene24471\_Mf\_liverA, Unigene32789\_Mf\_liverA, CL3835.Contig2\_Mf\_liverA, Unigene33080\_Mf\_liverA, CL3002.Contig1\_Mf\_liverA, CL2855.Contig2\_Mf\_liverA, Unigene6959\_Mf\_liverA, Unigene5750\_Mf\_liverA, CL887.Contig2\_Mf\_liverA, Unigene34754\_Mf\_liverA, Unigene19687\_Mf\_liverA, Unigene13143\_Mf\_liverA, Unigene4630\_Mf\_liverA, Unigene33746\_Mf\_liverA, Unigene14609\_Mf\_liverA, Unigene30547\_Mf\_liverA, CL1656.Contig1\_Mf\_liverA, Unigene14816\_Mf\_liverA, CL275.Contig5\_Mf\_liverA, CL2855.Contig1\_Mf\_liverA, CL2884.Contig2\_Mf\_liverA, Unigene10915\_Mf\_liverA, Unigene1137\_Mf\_liverA, Unigene35958\_Mf\_liverA, Unigene30584\_Mf\_liverA |
| cell surface | Unigene15055\_Mf\_liverA, Unigene24471\_Mf\_liverA, CL3835.Contig2\_Mf\_liverA, Unigene33080\_Mf\_liverA, CL2855.Contig2\_Mf\_liverA, Unigene6959\_Mf\_liverA, Unigene1479\_Mf\_liverA, Unigene5750\_Mf\_liverA, NM\_010378, Unigene34754\_Mf\_liverA, CL838.Contig3\_Mf\_liverA, Unigene4697\_Mf\_liverA, Unigene19687\_Mf\_liverA, Unigene13143\_Mf\_liverA, Unigene33746\_Mf\_liverA, Unigene30547\_Mf\_liverA, Unigene36698\_Mf\_liverA, Unigene27922\_Mf\_liverA, Unigene14816\_Mf\_liverA, Unigene13498\_Mf\_liverA, Unigene1137\_Mf\_liverA, Unigene35958\_Mf\_liverA, Unigene30584\_Mf\_liverA, NR\_004446, CL4583.Contig2\_Mf\_liverA, Unigene37245\_Mf\_liverA, Unigene32803\_Mf\_liverA, Unigene32789\_Mf\_liverA, CL3002.Contig1\_Mf\_liverA, Unigene37904\_Mf\_liverA, Unigene32335\_Mf\_liverA, CL887.Contig2\_Mf\_liverA, Unigene31333\_Mf\_liverA, Unigene33736\_Mf\_liverA, Unigene16529\_Mf\_liverA, CL3519.Contig1\_Mf\_liverA, Unigene32852\_Mf\_liverA, Unigene37232\_Mf\_liverA, Unigene11\_Mf\_liverA, Unigene4630\_Mf\_liverA, Unigene29426\_Mf\_liverA, Unigene14609\_Mf\_liverA, Unigene20371\_Mf\_liverA, CL1656.Contig1\_Mf\_liverA, CL2697.Contig4\_Mf\_liverA, CL838.Contig6\_Mf\_liverA, CL275.Contig5\_Mf\_liverA, NM\_010380, CL2855.Contig1\_Mf\_liverA, CL2884.Contig2\_Mf\_liverA, Unigene10915\_Mf\_liverA |
| intrinsic to plasma membrane | Unigene42606\_Mf\_liverA, CL4228.Contig1\_Mf\_liverA, CL425.Contig1\_Mf\_liverA, Unigene49971\_Mf\_liverA, Unigene38307\_Mf\_liverA, Unigene24883\_Mf\_liverA, CL3150.Contig1\_Mf\_liverA, Unigene25055\_Mf\_liverA, Unigene33746\_Mf\_liverA, CL2339.Contig1\_Mf\_liverA, Unigene36762\_Mf\_liverA, Unigene28853\_Mf\_liverA, CL238.Contig1\_Mf\_liverA, CL4600.Contig1\_Mf\_liverA, Unigene18500\_Mf\_liverA, CL485.Contig1\_Mf\_liverA, CL4910.Contig1\_Mf\_liverA, Unigene32803\_Mf\_liverA, CL1256.Contig1\_Mf\_liverA, Unigene34789\_Mf\_liverA, Unigene37224\_Mf\_liverA, Unigene13593\_Mf\_liverA, Unigene30369\_Mf\_liverA, Unigene33736\_Mf\_liverA, Unigene33598\_Mf\_liverA, Unigene29426\_Mf\_liverA, Unigene14609\_Mf\_liverA, Unigene843\_Mf\_liverA, Unigene35406\_Mf\_liverA, Unigene28852\_Mf\_liverA, NM\_001004357, Unigene20371\_Mf\_liverA, CL1656.Contig1\_Mf\_liverA, CL838.Contig6\_Mf\_liverA, Unigene15055\_Mf\_liverA, CL3154.Contig1\_Mf\_liverA, Unigene13363\_Mf\_liverA, CL2855.Contig2\_Mf\_liverA, CL4293.Contig1\_Mf\_liverA, CL4338.Contig1\_Mf\_liverA, CL4456.Contig1\_Mf\_liverA, Unigene5750\_Mf\_liverA, CL838.Contig3\_Mf\_liverA, NM\_178405, NM\_172203, Unigene30547\_Mf\_liverA, Unigene15470\_Mf\_liverA, Unigene39011\_Mf\_liverA, CL153.Contig2\_Mf\_liverA, Unigene25052\_Mf\_liverA, Unigene13950\_Mf\_liverA, Unigene36898\_Mf\_liverA, Unigene20372\_Mf\_liverA, Unigene16529\_Mf\_liverA, Unigene37232\_Mf\_liverA, Unigene27082\_Mf\_liverA, Unigene37366\_Mf\_liverA, Unigene1288\_Mf\_liverA, CL2884.Contig2\_Mf\_liverA, Unigene30697\_Mf\_liverA, CL2855.Contig1\_Mf\_liverA, Unigene5169\_Mf\_liverA, Unigene35618\_Mf\_liverA, Unigene17569\_Mf\_liverA |
| MHC class II protein complex | Unigene37245\_Mf\_liverA, CL2855.Contig1\_Mf\_liverA, Unigene24471\_Mf\_liverA, Unigene30878\_Mf\_liverA, CL3835.Contig2\_Mf\_liverA, CL3002.Contig1\_Mf\_liverA, CL2855.Contig2\_Mf\_liverA, Unigene4681\_Mf\_liverA |
| cytosol | Unigene35884\_Mf\_liverA, CL5640.Contig1\_Mf\_liverA, Unigene27730\_Mf\_liverA, Unigene38015\_Mf\_liverA, Unigene836\_Mf\_liverA, CL186.Contig3\_Mf\_liverA, Unigene36172\_Mf\_liverA, Unigene28899\_Mf\_liverA, Unigene36851\_Mf\_liverA, Unigene36190\_Mf\_liverA, Unigene34811\_Mf\_liverA, Unigene30832\_Mf\_liverA, CL4105.Contig1\_Mf\_liverA, Unigene30154\_Mf\_liverA, Unigene7336\_Mf\_liverA, Unigene35382\_Mf\_liverA, Unigene1000\_Mf\_liverA, Unigene39886\_Mf\_liverA, Unigene15681\_Mf\_liverA, Unigene5693\_Mf\_liverA, Unigene15064\_Mf\_liverA, Unigene15476\_Mf\_liverA, CL4583.Contig2\_Mf\_liverA, Unigene28687\_Mf\_liverA, Unigene32570\_Mf\_liverA, Unigene5815\_Mf\_liverA, CL4745.Contig1\_Mf\_liverA, Unigene5333\_Mf\_liverA, Unigene15295\_Mf\_liverA, Unigene12999\_Mf\_liverA, CL1810.Contig1\_Mf\_liverA, CL4141.Contig1\_Mf\_liverA, Unigene34184\_Mf\_liverA, CL1119.Contig1\_Mf\_liverA, Unigene13658\_Mf\_liverA, Unigene33523\_Mf\_liverA, Unigene35037\_Mf\_liverA, CL3933.Contig1\_Mf\_liverA, Unigene30003\_Mf\_liverA, Unigene843\_Mf\_liverA, Unigene25596\_Mf\_liverA, Unigene36430\_Mf\_liverA, CL3684.Contig2\_Mf\_liverA, CL1493.Contig1\_Mf\_liverA, Unigene1212\_Mf\_liverA, Unigene13009\_Mf\_liverA, Unigene18848\_Mf\_liverA, Unigene30002\_Mf\_liverA, Unigene30004\_Mf\_liverA, Unigene35476\_Mf\_liverA, Unigene24758\_Mf\_liverA, Unigene24471\_Mf\_liverA, Unigene29490\_Mf\_liverA, Unigene601\_Mf\_liverA, CL2389.Contig1\_Mf\_liverA, Unigene151\_Mf\_liverA, Unigene37262\_Mf\_liverA, Unigene22423\_Mf\_liverA, CL848.Contig2\_Mf\_liverA, CL5052.Contig1\_Mf\_liverA, Unigene12908\_Mf\_liverA, CL1263.Contig1\_Mf\_liverA, CL258.Contig1\_Mf\_liverA, CL442.Contig5\_Mf\_liverA, Unigene24323\_Mf\_liverA, CL2142.Contig2\_Mf\_liverA, Unigene21741\_Mf\_liverA, Unigene34962\_Mf\_liverA, Unigene21742\_Mf\_liverA, Unigene120\_Mf\_liverA, Unigene36189\_Mf\_liverA, Unigene31427\_Mf\_liverA, Unigene15026\_Mf\_liverA, Unigene37018\_Mf\_liverA, Unigene40069\_Mf\_liverA, Unigene550\_Mf\_liverA, Unigene32133\_Mf\_liverA, Unigene37120\_Mf\_liverA, Unigene15290\_Mf\_liverA, Unigene12909\_Mf\_liverA, CL2355.Contig1\_Mf\_liverA, Unigene35664\_Mf\_liverA, Unigene37153\_Mf\_liverA, CL1688.Contig3\_Mf\_liverA, CL3104.Contig1\_Mf\_liverA, Unigene37104\_Mf\_liverA, CL3930.Contig1\_Mf\_liverA, Unigene16529\_Mf\_liverA, Unigene26611\_Mf\_liverA, Unigene27082\_Mf\_liverA, Unigene37178\_Mf\_liverA, Unigene15477\_Mf\_liverA, CL5698.Contig1\_Mf\_liverA, Unigene30288\_Mf\_liverA, Unigene36772\_Mf\_liverA, CL442.Contig2\_Mf\_liverA, CL6039.Contig1\_Mf\_liverA, Unigene2939\_Mf\_liverA |
| multivesicular body | Unigene37245\_Mf\_liverA, Unigene27082\_Mf\_liverA, Unigene21026\_Mf\_liverA, Unigene15470\_Mf\_liverA, CL3002.Contig1\_Mf\_liverA, CL2855.Contig2\_Mf\_liverA, Unigene4681\_Mf\_liverA, CL2855.Contig1\_Mf\_liverA, Unigene30878\_Mf\_liverA |
| symbiont-containing vacuole | CL482.Contig1\_Mf\_liverA, CL6039.Contig1\_Mf\_liverA, Unigene36328\_Mf\_liverA, CL5978.Contig3\_Mf\_liverA, CL442.Contig5\_Mf\_liverA, Unigene36898\_Mf\_liverA, CL5978.Contig2\_Mf\_liverA, CL442.Contig2\_Mf\_liverA |
| plasma membrane part | CL4302.Contig1\_Mf\_liverA, CL3835.Contig2\_Mf\_liverA, Unigene42606\_Mf\_liverA, CL4228.Contig1\_Mf\_liverA, Unigene6959\_Mf\_liverA, CL425.Contig1\_Mf\_liverA, Unigene49971\_Mf\_liverA, Unigene38307\_Mf\_liverA, Unigene28244\_Mf\_liverA, Unigene24883\_Mf\_liverA, CL3150.Contig1\_Mf\_liverA, NM\_010378, Unigene34754\_Mf\_liverA, Unigene880\_Mf\_liverA, Unigene19687\_Mf\_liverA, Unigene36849\_Mf\_liverA, Unigene25055\_Mf\_liverA, Unigene33746\_Mf\_liverA, CL2339.Contig1\_Mf\_liverA, Unigene37243\_Mf\_liverA, Unigene36762\_Mf\_liverA, Unigene28853\_Mf\_liverA, Unigene27922\_Mf\_liverA, CL4600.Contig1\_Mf\_liverA, CL238.Contig1\_Mf\_liverA, Unigene14816\_Mf\_liverA, Unigene5693\_Mf\_liverA, Unigene30878\_Mf\_liverA, Unigene1137\_Mf\_liverA, Unigene35958\_Mf\_liverA, NR\_004446, CL4583.Contig2\_Mf\_liverA, Unigene18500\_Mf\_liverA, CL485.Contig1\_Mf\_liverA, NM\_021423, Unigene4938\_Mf\_liverA, CL4910.Contig1\_Mf\_liverA, Unigene32803\_Mf\_liverA, Unigene36328\_Mf\_liverA, CL3002.Contig1\_Mf\_liverA, CL1256.Contig1\_Mf\_liverA, Unigene31267\_Mf\_liverA, Unigene34789\_Mf\_liverA, Unigene37224\_Mf\_liverA, CL1810.Contig1\_Mf\_liverA, CL4033.Contig1\_Mf\_liverA, CL887.Contig2\_Mf\_liverA, Unigene13593\_Mf\_liverA, CL3519.Contig1\_Mf\_liverA, NM\_010233, Unigene30369\_Mf\_liverA, Unigene33736\_Mf\_liverA, Unigene33598\_Mf\_liverA, Unigene4630\_Mf\_liverA, Unigene29426\_Mf\_liverA, Unigene14609\_Mf\_liverA, Unigene843\_Mf\_liverA, Unigene35406\_Mf\_liverA, NM\_001004357, Unigene28852\_Mf\_liverA, Unigene20371\_Mf\_liverA, CL1656.Contig1\_Mf\_liverA, CL838.Contig6\_Mf\_liverA, Unigene10915\_Mf\_liverA, Unigene15055\_Mf\_liverA, CL3154.Contig1\_Mf\_liverA, Unigene24471\_Mf\_liverA, Unigene13363\_Mf\_liverA, Unigene33080\_Mf\_liverA, CL2855.Contig2\_Mf\_liverA, CL4293.Contig1\_Mf\_liverA, Unigene22423\_Mf\_liverA, CL4338.Contig1\_Mf\_liverA, Unigene5750\_Mf\_liverA, CL4456.Contig1\_Mf\_liverA, CL838.Contig3\_Mf\_liverA, Unigene13143\_Mf\_liverA, NM\_178405, Unigene30547\_Mf\_liverA, NM\_172203, Unigene15470\_Mf\_liverA, Unigene139\_Mf\_liverA, Unigene39011\_Mf\_liverA, NM\_011207, CL153.Contig2\_Mf\_liverA, Unigene15572\_Mf\_liverA, CL4007.Contig1\_Mf\_liverA, CL4095.Contig4\_Mf\_liverA, Unigene25052\_Mf\_liverA, Unigene13950\_Mf\_liverA, Unigene30584\_Mf\_liverA, Unigene37245\_Mf\_liverA, Unigene32789\_Mf\_liverA, Unigene36898\_Mf\_liverA, Unigene32335\_Mf\_liverA, Unigene20372\_Mf\_liverA, Unigene16529\_Mf\_liverA, Unigene37232\_Mf\_liverA, NM\_026313, Unigene27082\_Mf\_liverA, Unigene44057\_Mf\_liverA, CL5007.Contig1\_Mf\_liverA, Unigene37366\_Mf\_liverA, Unigene1288\_Mf\_liverA, Unigene4681\_Mf\_liverA, CL275.Contig5\_Mf\_liverA, NM\_010380, CL2855.Contig1\_Mf\_liverA, CL2884.Contig2\_Mf\_liverA, CL591.Contig1\_Mf\_liverA, Unigene30697\_Mf\_liverA, CL336.Contig3\_Mf\_liverA, Unigene5169\_Mf\_liverA, Unigene35618\_Mf\_liverA, Unigene17569\_Mf\_liverA |
| host cell cytoplasm | CL482.Contig1\_Mf\_liverA, CL6039.Contig1\_Mf\_liverA, Unigene36328\_Mf\_liverA, CL5978.Contig3\_Mf\_liverA, CL442.Contig5\_Mf\_liverA, Unigene36898\_Mf\_liverA, CL5978.Contig2\_Mf\_liverA, CL442.Contig2\_Mf\_liverA |
| host cell cytoplasm part | CL482.Contig1\_Mf\_liverA, CL6039.Contig1\_Mf\_liverA, Unigene36328\_Mf\_liverA, CL5978.Contig3\_Mf\_liverA, CL442.Contig5\_Mf\_liverA, Unigene36898\_Mf\_liverA, CL5978.Contig2\_Mf\_liverA, CL442.Contig2\_Mf\_liverA |
| host intracellular part | CL482.Contig1\_Mf\_liverA, CL6039.Contig1\_Mf\_liverA, Unigene36328\_Mf\_liverA, CL5978.Contig3\_Mf\_liverA, CL442.Contig5\_Mf\_liverA, Unigene36898\_Mf\_liverA, CL5978.Contig2\_Mf\_liverA, CL442.Contig2\_Mf\_liverA |
| intracellular region of host | CL482.Contig1\_Mf\_liverA, CL6039.Contig1\_Mf\_liverA, Unigene36328\_Mf\_liverA, CL5978.Contig3\_Mf\_liverA, CL442.Contig5\_Mf\_liverA, Unigene36898\_Mf\_liverA, CL5978.Contig2\_Mf\_liverA, CL442.Contig2\_Mf\_liverA |
| endoplasmic reticulum lumen | CL3911.Contig2\_Mf\_liverA, Unigene25595\_Mf\_liverA, Unigene25596\_Mf\_liverA, CL1076.Contig1\_Mf\_liverA, Unigene34375\_Mf\_liverA, CL5309.Contig1\_Mf\_liverA, Unigene600\_Mf\_liverA, CL4263.Contig1\_Mf\_liverA, CL913.Contig1\_Mf\_liverA, Unigene29011\_Mf\_liverA, Unigene34406\_Mf\_liverA, Unigene13498\_Mf\_liverA, Unigene1479\_Mf\_liverA, Unigene23328\_Mf\_liverA, Unigene34124\_Mf\_liverA, Unigene35958\_Mf\_liverA |
| host | CL482.Contig1\_Mf\_liverA, CL6039.Contig1\_Mf\_liverA, Unigene36328\_Mf\_liverA, CL5978.Contig3\_Mf\_liverA, CL442.Contig5\_Mf\_liverA, Unigene36898\_Mf\_liverA, CL5978.Contig2\_Mf\_liverA, CL442.Contig2\_Mf\_liverA |
| host cell part | CL482.Contig1\_Mf\_liverA, CL6039.Contig1\_Mf\_liverA, Unigene36328\_Mf\_liverA, CL5978.Contig3\_Mf\_liverA, CL442.Contig5\_Mf\_liverA, Unigene36898\_Mf\_liverA, CL5978.Contig2\_Mf\_liverA, CL442.Contig2\_Mf\_liverA |
| extraorganismal space | CL482.Contig1\_Mf\_liverA, CL6039.Contig1\_Mf\_liverA, Unigene36328\_Mf\_liverA, CL5978.Contig3\_Mf\_liverA, CL442.Contig5\_Mf\_liverA, Unigene36898\_Mf\_liverA, CL5978.Contig2\_Mf\_liverA, CL442.Contig2\_Mf\_liverA |
| host cell | CL482.Contig1\_Mf\_liverA, CL6039.Contig1\_Mf\_liverA, Unigene36328\_Mf\_liverA, CL5978.Contig3\_Mf\_liverA, CL442.Contig5\_Mf\_liverA, Unigene36898\_Mf\_liverA, CL5978.Contig2\_Mf\_liverA, CL442.Contig2\_Mf\_liverA |
| other organism | CL482.Contig1\_Mf\_liverA, CL6039.Contig1\_Mf\_liverA, Unigene36328\_Mf\_liverA, CL5978.Contig3\_Mf\_liverA, CL442.Contig5\_Mf\_liverA, Unigene36898\_Mf\_liverA, CL5978.Contig2\_Mf\_liverA, CL442.Contig2\_Mf\_liverA |
| other organism cell | CL482.Contig1\_Mf\_liverA, CL6039.Contig1\_Mf\_liverA, Unigene36328\_Mf\_liverA, CL5978.Contig3\_Mf\_liverA, CL442.Contig5\_Mf\_liverA, Unigene36898\_Mf\_liverA, CL5978.Contig2\_Mf\_liverA, CL442.Contig2\_Mf\_liverA |
| other organism part | CL482.Contig1\_Mf\_liverA, CL6039.Contig1\_Mf\_liverA, Unigene36328\_Mf\_liverA, CL5978.Contig3\_Mf\_liverA, CL442.Contig5\_Mf\_liverA, Unigene36898\_Mf\_liverA, CL5978.Contig2\_Mf\_liverA, CL442.Contig2\_Mf\_liverA |
| lysosome | Unigene34032\_Mf\_liverA, CL4583.Contig2\_Mf\_liverA, Unigene24471\_Mf\_liverA, Unigene36328\_Mf\_liverA, Unigene38280\_Mf\_liverA, CL3835.Contig2\_Mf\_liverA, CL3002.Contig1\_Mf\_liverA, CL2855.Contig2\_Mf\_liverA, CL3669.Contig2\_Mf\_liverA, Unigene37389\_Mf\_liverA, Unigene32332\_Mf\_liverA, Unigene29082\_Mf\_liverA, Unigene31670\_Mf\_liverA, CL838.Contig3\_Mf\_liverA, CL854.Contig1\_Mf\_liverA, Unigene138\_Mf\_liverA, Unigene37232\_Mf\_liverA, Unigene27082\_Mf\_liverA, CL1493.Contig2\_Mf\_liverA, CL442.Contig2\_Mf\_liverA, CL1493.Contig1\_Mf\_liverA, Unigene4681\_Mf\_liverA, Unigene39886\_Mf\_liverA, CL838.Contig6\_Mf\_liverA, CL2855.Contig1\_Mf\_liverA, Unigene30878\_Mf\_liverA, Unigene35618\_Mf\_liverA, Unigene29558\_Mf\_liverA, Unigene35958\_Mf\_liverA |
| extracellular organelle | CL482.Contig1\_Mf\_liverA, CL6039.Contig1\_Mf\_liverA, Unigene36328\_Mf\_liverA, CL5978.Contig3\_Mf\_liverA, CL442.Contig5\_Mf\_liverA, Unigene36898\_Mf\_liverA, CL5978.Contig2\_Mf\_liverA, CL442.Contig2\_Mf\_liverA |
| extracellular membrane-bounded organelle | CL482.Contig1\_Mf\_liverA, CL6039.Contig1\_Mf\_liverA, Unigene36328\_Mf\_liverA, CL5978.Contig3\_Mf\_liverA, CL442.Contig5\_Mf\_liverA, Unigene36898\_Mf\_liverA, CL5978.Contig2\_Mf\_liverA, CL442.Contig2\_Mf\_liverA |
| organelle membrane | NM\_001100182, Unigene31051\_Mf\_liverA, Unigene38015\_Mf\_liverA, NM\_206537, CL3835.Contig2\_Mf\_liverA, CL4550.Contig2\_Mf\_liverA, NM\_009178, CL2797.Contig2\_Mf\_liverA, CL523.Contig1\_Mf\_liverA, NM\_008277, NM\_011082, Unigene7336\_Mf\_liverA, CL482.Contig1\_Mf\_liverA, Unigene28143\_Mf\_liverA, CL1125.Contig1\_Mf\_liverA, Unigene30217\_Mf\_liverA, Unigene15077\_Mf\_liverA, CL4600.Contig1\_Mf\_liverA, Unigene35507\_Mf\_liverA, Unigene39886\_Mf\_liverA, Unigene5693\_Mf\_liverA, CL3382.Contig1\_Mf\_liverA, NM\_029582, Unigene30878\_Mf\_liverA, Unigene32920\_Mf\_liverA, CL1100.Contig1\_Mf\_liverA, CL4583.Contig2\_Mf\_liverA, CL1988.Contig1\_Mf\_liverA, Unigene36328\_Mf\_liverA, CL3002.Contig1\_Mf\_liverA, Unigene36315\_Mf\_liverA, Unigene34341\_Mf\_liverA, CL1988.Contig3\_Mf\_liverA, CL695.Contig1\_Mf\_liverA, CL1810.Contig1\_Mf\_liverA, Unigene36949\_Mf\_liverA, CL4033.Contig1\_Mf\_liverA, Unigene5488\_Mf\_liverA, Unigene2\_Mf\_liverA, NM\_145474, NM\_134127, Unigene33736\_Mf\_liverA, Unigene34394\_Mf\_liverA, Unigene21026\_Mf\_liverA, NM\_031165, CL497.Contig2\_Mf\_liverA, CL1493.Contig2\_Mf\_liverA, CL1493.Contig1\_Mf\_liverA, CL6.Contig1\_Mf\_liverA, CL2697.Contig4\_Mf\_liverA, NM\_001100181, Unigene5607\_Mf\_liverA, CL2574.Contig1\_Mf\_liverA, Unigene542\_Mf\_liverA, CL4816.Contig1\_Mf\_liverA, CL787.Contig1\_Mf\_liverA, Unigene15055\_Mf\_liverA, Unigene13196\_Mf\_liverA, CL114.Contig2\_Mf\_liverA, CL5807.Contig1\_Mf\_liverA, Unigene24471\_Mf\_liverA, Unigene29490\_Mf\_liverA, CL1076.Contig1\_Mf\_liverA, Unigene15888\_Mf\_liverA, CL2855.Contig2\_Mf\_liverA, Unigene12967\_Mf\_liverA, CL4220.Contig1\_Mf\_liverA, CL3599.Contig1\_Mf\_liverA, Unigene15433\_Mf\_liverA, CL5307.Contig1\_Mf\_liverA, NM\_019817, CL784.Contig3\_Mf\_liverA, Unigene1479\_Mf\_liverA, Unigene5750\_Mf\_liverA, NM\_019879, Unigene31670\_Mf\_liverA, NM\_010001, Unigene5632\_Mf\_liverA, NM\_007822, CL4550.Contig3\_Mf\_liverA, Unigene21816\_Mf\_liverA, NM\_007811, CL4816.Contig3\_Mf\_liverA, Unigene139\_Mf\_liverA, CL114.Contig1\_Mf\_liverA, Unigene24225\_Mf\_liverA, Unigene37344\_Mf\_liverA, CL4007.Contig1\_Mf\_liverA, CL529.Contig2\_Mf\_liverA, CL55.Contig1\_Mf\_liverA, Unigene32789\_Mf\_liverA, Unigene24090\_Mf\_liverA, CL4984.Contig1\_Mf\_liverA, Unigene36898\_Mf\_liverA, Unigene281\_Mf\_liverA, CL5316.Contig1\_Mf\_liverA, Unigene32335\_Mf\_liverA, NM\_011978, NM\_177652, Unigene37389\_Mf\_liverA, Unigene32332\_Mf\_liverA, CL3104.Contig1\_Mf\_liverA, Unigene36593\_Mf\_liverA, Unigene5489\_Mf\_liverA, Unigene7195\_Mf\_liverA, NM\_010002, Unigene34444\_Mf\_liverA, Unigene35506\_Mf\_liverA, Unigene16529\_Mf\_liverA, Unigene27082\_Mf\_liverA, Unigene5712\_Mf\_liverA, CL5007.Contig1\_Mf\_liverA, CL5698.Contig1\_Mf\_liverA, Unigene1280\_Mf\_liverA, Unigene1205\_Mf\_liverA, CL442.Contig2\_Mf\_liverA, CL3166.Contig4\_Mf\_liverA, Unigene4681\_Mf\_liverA, NM\_008851, CL2855.Contig1\_Mf\_liverA, Unigene5886\_Mf\_liverA, CL336.Contig3\_Mf\_liverA, NM\_013821, Unigene40796\_Mf\_liverA |
| endosome | Unigene24471\_Mf\_liverA, Unigene38015\_Mf\_liverA, Unigene29490\_Mf\_liverA, CL3835.Contig2\_Mf\_liverA, CL2855.Contig2\_Mf\_liverA, Unigene836\_Mf\_liverA, CL4338.Contig1\_Mf\_liverA, Unigene49971\_Mf\_liverA, Unigene28244\_Mf\_liverA, Unigene34754\_Mf\_liverA, Unigene31670\_Mf\_liverA, NM\_011082, Unigene33746\_Mf\_liverA, Unigene36698\_Mf\_liverA, NM\_172203, Unigene15077\_Mf\_liverA, Unigene27922\_Mf\_liverA, Unigene15470\_Mf\_liverA, Unigene5693\_Mf\_liverA, Unigene30878\_Mf\_liverA, Unigene32093\_Mf\_liverA, Unigene35958\_Mf\_liverA, NR\_004446, Unigene37245\_Mf\_liverA, CL55.Contig1\_Mf\_liverA, Unigene32570\_Mf\_liverA, Unigene36328\_Mf\_liverA, CL3002.Contig1\_Mf\_liverA, Unigene32335\_Mf\_liverA, Unigene37389\_Mf\_liverA, Unigene33736\_Mf\_liverA, Unigene16529\_Mf\_liverA, Unigene27082\_Mf\_liverA, Unigene14609\_Mf\_liverA, Unigene21026\_Mf\_liverA, CL497.Contig2\_Mf\_liverA, CL1493.Contig2\_Mf\_liverA, CL442.Contig2\_Mf\_liverA, Unigene4681\_Mf\_liverA, CL1493.Contig1\_Mf\_liverA, CL2855.Contig1\_Mf\_liverA, Unigene35618\_Mf\_liverA |
| late endosome | Unigene37245\_Mf\_liverA, Unigene24471\_Mf\_liverA, Unigene36328\_Mf\_liverA, CL3835.Contig2\_Mf\_liverA, CL3002.Contig1\_Mf\_liverA, CL2855.Contig2\_Mf\_liverA, Unigene31670\_Mf\_liverA, Unigene27082\_Mf\_liverA, Unigene36698\_Mf\_liverA, Unigene21026\_Mf\_liverA, Unigene15470\_Mf\_liverA, CL1493.Contig2\_Mf\_liverA, CL442.Contig2\_Mf\_liverA, Unigene4681\_Mf\_liverA, CL1493.Contig1\_Mf\_liverA, Unigene5693\_Mf\_liverA, CL2855.Contig1\_Mf\_liverA, Unigene30878\_Mf\_liverA, Unigene32093\_Mf\_liverA |
| endomembrane system | Unigene31051\_Mf\_liverA, Unigene29011\_Mf\_liverA, CL4550.Contig2\_Mf\_liverA, NM\_009178, NM\_080638, CL2797.Contig2\_Mf\_liverA, CL523.Contig1\_Mf\_liverA, NM\_008277, Unigene26053\_Mf\_liverA, Unigene7336\_Mf\_liverA, CL482.Contig1\_Mf\_liverA, Unigene28143\_Mf\_liverA, CL4162.Contig1\_Mf\_liverA, Unigene30217\_Mf\_liverA, Unigene35507\_Mf\_liverA, CL4600.Contig1\_Mf\_liverA, Unigene32920\_Mf\_liverA, CL1100.Contig1\_Mf\_liverA, NR\_004446, CL4583.Contig2\_Mf\_liverA, CL1988.Contig1\_Mf\_liverA, Unigene36328\_Mf\_liverA, Unigene36315\_Mf\_liverA, Unigene34341\_Mf\_liverA, CL1988.Contig3\_Mf\_liverA, CL695.Contig1\_Mf\_liverA, Unigene36949\_Mf\_liverA, CL4033.Contig1\_Mf\_liverA, Unigene5488\_Mf\_liverA, Unigene31623\_Mf\_liverA, Unigene34394\_Mf\_liverA, Unigene21026\_Mf\_liverA, NM\_031165, CL1493.Contig2\_Mf\_liverA, CL1493.Contig1\_Mf\_liverA, CL2697.Contig4\_Mf\_liverA, CL6.Contig1\_Mf\_liverA, Unigene5607\_Mf\_liverA, CL2574.Contig1\_Mf\_liverA, Unigene542\_Mf\_liverA, CL4816.Contig1\_Mf\_liverA, Unigene15055\_Mf\_liverA, CL787.Contig1\_Mf\_liverA, Unigene13196\_Mf\_liverA, CL114.Contig2\_Mf\_liverA, CL5807.Contig1\_Mf\_liverA, Unigene15888\_Mf\_liverA, CL2855.Contig2\_Mf\_liverA, Unigene12967\_Mf\_liverA, CL4220.Contig1\_Mf\_liverA, Unigene15433\_Mf\_liverA, CL5307.Contig1\_Mf\_liverA, NM\_019817, CL784.Contig3\_Mf\_liverA, Unigene5750\_Mf\_liverA, Unigene1479\_Mf\_liverA, Unigene5632\_Mf\_liverA, CL1263.Contig1\_Mf\_liverA, CL4550.Contig3\_Mf\_liverA, Unigene21816\_Mf\_liverA, CL4816.Contig3\_Mf\_liverA, Unigene139\_Mf\_liverA, CL114.Contig1\_Mf\_liverA, Unigene37344\_Mf\_liverA, CL4007.Contig1\_Mf\_liverA, Unigene31625\_Mf\_liverA, CL529.Contig2\_Mf\_liverA, Unigene550\_Mf\_liverA, Unigene32789\_Mf\_liverA, Unigene24090\_Mf\_liverA, Unigene36898\_Mf\_liverA, Unigene281\_Mf\_liverA, Unigene32335\_Mf\_liverA, NM\_177652, Unigene37389\_Mf\_liverA, CL1688.Contig3\_Mf\_liverA, Unigene36593\_Mf\_liverA, Unigene5489\_Mf\_liverA, Unigene7195\_Mf\_liverA, Unigene34444\_Mf\_liverA, Unigene35506\_Mf\_liverA, Unigene16529\_Mf\_liverA, Unigene37232\_Mf\_liverA, Unigene27082\_Mf\_liverA, CL5007.Contig1\_Mf\_liverA, Unigene5712\_Mf\_liverA, CL5698.Contig1\_Mf\_liverA, Unigene1280\_Mf\_liverA, Unigene1205\_Mf\_liverA, CL3166.Contig4\_Mf\_liverA, NM\_008851, CL2855.Contig1\_Mf\_liverA, Unigene5886\_Mf\_liverA, CL336.Contig3\_Mf\_liverA, Unigene40796\_Mf\_liverA |
| lytic vacuole | Unigene34032\_Mf\_liverA, CL4583.Contig2\_Mf\_liverA, Unigene24471\_Mf\_liverA, Unigene36328\_Mf\_liverA, Unigene38280\_Mf\_liverA, CL3835.Contig2\_Mf\_liverA, CL3002.Contig1\_Mf\_liverA, CL2855.Contig2\_Mf\_liverA, CL3669.Contig2\_Mf\_liverA, Unigene37389\_Mf\_liverA, Unigene32332\_Mf\_liverA, Unigene29082\_Mf\_liverA, NM\_010378, Unigene31670\_Mf\_liverA, CL838.Contig3\_Mf\_liverA, CL854.Contig1\_Mf\_liverA, Unigene138\_Mf\_liverA, Unigene37232\_Mf\_liverA, Unigene27082\_Mf\_liverA, CL1493.Contig2\_Mf\_liverA, CL442.Contig2\_Mf\_liverA, CL1493.Contig1\_Mf\_liverA, Unigene4681\_Mf\_liverA, CL838.Contig6\_Mf\_liverA, Unigene39886\_Mf\_liverA, CL2855.Contig1\_Mf\_liverA, Unigene30878\_Mf\_liverA, Unigene35618\_Mf\_liverA, NM\_016697, Unigene35958\_Mf\_liverA, Unigene29558\_Mf\_liverA |
| high-density lipoprotein particle | CL3911.Contig2\_Mf\_liverA, Unigene36698\_Mf\_liverA, Unigene13498\_Mf\_liverA, Unigene9081\_Mf\_liverA, Unigene30815\_Mf\_liverA, Unigene30814\_Mf\_liverA, Unigene24157\_Mf\_liverA |
| symbiont-containing vacuole membrane | CL482.Contig1\_Mf\_liverA, CL6039.Contig1\_Mf\_liverA, CL5978.Contig3\_Mf\_liverA, Unigene36898\_Mf\_liverA, CL5978.Contig2\_Mf\_liverA |
| MHC class I protein complex | CL275.Contig5\_Mf\_liverA, Unigene4938\_Mf\_liverA, Unigene28244\_Mf\_liverA, Unigene36849\_Mf\_liverA, Unigene13143\_Mf\_liverA |
| vacuole | Unigene34032\_Mf\_liverA, CL4583.Contig2\_Mf\_liverA, Unigene24471\_Mf\_liverA, Unigene36328\_Mf\_liverA, Unigene38280\_Mf\_liverA, CL3835.Contig2\_Mf\_liverA, CL3002.Contig1\_Mf\_liverA, CL2855.Contig2\_Mf\_liverA, CL3669.Contig2\_Mf\_liverA, Unigene37389\_Mf\_liverA, Unigene32332\_Mf\_liverA, Unigene29082\_Mf\_liverA, Unigene31639\_Mf\_liverA, NM\_010378, Unigene31670\_Mf\_liverA, CL838.Contig3\_Mf\_liverA, CL854.Contig1\_Mf\_liverA, Unigene138\_Mf\_liverA, Unigene37232\_Mf\_liverA, Unigene27082\_Mf\_liverA, CL1493.Contig2\_Mf\_liverA, CL442.Contig2\_Mf\_liverA, CL1493.Contig1\_Mf\_liverA, Unigene4681\_Mf\_liverA, CL838.Contig6\_Mf\_liverA, Unigene39886\_Mf\_liverA, CL2855.Contig1\_Mf\_liverA, Unigene30878\_Mf\_liverA, Unigene35618\_Mf\_liverA, NM\_016697, Unigene35958\_Mf\_liverA, Unigene29558\_Mf\_liverA |
| SREBP-SCAP-Insig complex | Unigene5489\_Mf\_liverA, Unigene34444\_Mf\_liverA, Unigene5488\_Mf\_liverA |
| lysosomal membrane | CL4583.Contig2\_Mf\_liverA, Unigene24471\_Mf\_liverA, CL3835.Contig2\_Mf\_liverA, CL3002.Contig1\_Mf\_liverA, CL2855.Contig2\_Mf\_liverA, Unigene4681\_Mf\_liverA, Unigene32332\_Mf\_liverA, Unigene39886\_Mf\_liverA, CL2855.Contig1\_Mf\_liverA, Unigene30878\_Mf\_liverA, Unigene31670\_Mf\_liverA |
| vacuolar part | Unigene34032\_Mf\_liverA, CL4583.Contig2\_Mf\_liverA, Unigene24471\_Mf\_liverA, Unigene36328\_Mf\_liverA, CL3835.Contig2\_Mf\_liverA, CL3002.Contig1\_Mf\_liverA, CL2855.Contig2\_Mf\_liverA, Unigene4681\_Mf\_liverA, Unigene32332\_Mf\_liverA, Unigene29082\_Mf\_liverA, Unigene39886\_Mf\_liverA, CL2855.Contig1\_Mf\_liverA, Unigene30878\_Mf\_liverA, Unigene31670\_Mf\_liverA, Unigene35958\_Mf\_liverA, Unigene29558\_Mf\_liverA |
| perinuclear region of cytoplasm | Unigene21684\_Mf\_liverA, CL4583.Contig2\_Mf\_liverA, Unigene15290\_Mf\_liverA, Unigene31267\_Mf\_liverA, Unigene32335\_Mf\_liverA, Unigene20372\_Mf\_liverA, Unigene15295\_Mf\_liverA, CL186.Contig3\_Mf\_liverA, Unigene30142\_Mf\_liverA, Unigene28244\_Mf\_liverA, Unigene1479\_Mf\_liverA, CL838.Contig3\_Mf\_liverA, CL4919.Contig1\_Mf\_liverA, Unigene26053\_Mf\_liverA, Unigene11\_Mf\_liverA, CL4162.Contig1\_Mf\_liverA, Unigene21026\_Mf\_liverA, Unigene5712\_Mf\_liverA, CL497.Contig2\_Mf\_liverA, Unigene20371\_Mf\_liverA, CL2142.Contig2\_Mf\_liverA, Unigene14461\_Mf\_liverA, CL2697.Contig4\_Mf\_liverA, Unigene39011\_Mf\_liverA, CL838.Contig6\_Mf\_liverA, Unigene15572\_Mf\_liverA, Unigene36765\_Mf\_liverA |
| lysosomal lumen | Unigene34032\_Mf\_liverA, Unigene29082\_Mf\_liverA, CL4583.Contig2\_Mf\_liverA, CL2855.Contig1\_Mf\_liverA, CL2855.Contig2\_Mf\_liverA, Unigene35958\_Mf\_liverA, Unigene29558\_Mf\_liverA |
| proteasome core complex | Unigene35884\_Mf\_liverA, Unigene24471\_Mf\_liverA, CL3835.Contig2\_Mf\_liverA, CL3002.Contig1\_Mf\_liverA, Unigene37153\_Mf\_liverA |
| vacuolar lumen | Unigene34032\_Mf\_liverA, Unigene29082\_Mf\_liverA, CL4583.Contig2\_Mf\_liverA, CL2855.Contig1\_Mf\_liverA, CL2855.Contig2\_Mf\_liverA, Unigene35958\_Mf\_liverA, Unigene29558\_Mf\_liverA |
| integral to organelle membrane | CL787.Contig1\_Mf\_liverA, Unigene31051\_Mf\_liverA, CL1076.Contig1\_Mf\_liverA, Unigene15888\_Mf\_liverA, CL2855.Contig2\_Mf\_liverA, CL695.Contig1\_Mf\_liverA, CL3104.Contig1\_Mf\_liverA, CL2855.Contig1\_Mf\_liverA, CL3382.Contig1\_Mf\_liverA, Unigene5489\_Mf\_liverA, Unigene34444\_Mf\_liverA, Unigene5488\_Mf\_liverA, Unigene1479\_Mf\_liverA, Unigene32920\_Mf\_liverA |
| macrophage migration inhibitory factor receptor complex | CL2855.Contig1\_Mf\_liverA, CL2855.Contig2\_Mf\_liverA |
| NOS2-CD74 complex | CL2855.Contig1\_Mf\_liverA, CL2855.Contig2\_Mf\_liverA |
| proteasome complex | CL4141.Contig1\_Mf\_liverA, Unigene35884\_Mf\_liverA, Unigene24471\_Mf\_liverA, Unigene36851\_Mf\_liverA, CL3835.Contig2\_Mf\_liverA, CL3002.Contig1\_Mf\_liverA, CL1119.Contig1\_Mf\_liverA, Unigene37153\_Mf\_liverA |
| Golgi apparatus | CL787.Contig1\_Mf\_liverA, Unigene15055\_Mf\_liverA, CL3154.Contig1\_Mf\_liverA, Unigene31051\_Mf\_liverA, Unigene4363\_Mf\_liverA, CL2855.Contig2\_Mf\_liverA, CL3669.Contig2\_Mf\_liverA, NM\_009178, CL4338.Contig1\_Mf\_liverA, Unigene36190\_Mf\_liverA, NM\_019817, Unigene5632\_Mf\_liverA, CL482.Contig1\_Mf\_liverA, CL1263.Contig1\_Mf\_liverA, CL442.Contig5\_Mf\_liverA, CL4600.Contig1\_Mf\_liverA, CL5275.Contig2\_Mf\_liverA, Unigene36189\_Mf\_liverA, Unigene31625\_Mf\_liverA, Unigene32920\_Mf\_liverA, Unigene32093\_Mf\_liverA, Unigene34032\_Mf\_liverA, Unigene37245\_Mf\_liverA, CL55.Contig1\_Mf\_liverA, Unigene15290\_Mf\_liverA, Unigene36328\_Mf\_liverA, CL3002.Contig1\_Mf\_liverA, Unigene281\_Mf\_liverA, Unigene24537\_Mf\_liverA, Unigene4863\_Mf\_liverA, Unigene32335\_Mf\_liverA, Unigene37389\_Mf\_liverA, CL1688.Contig3\_Mf\_liverA, Unigene29082\_Mf\_liverA, CL4033.Contig1\_Mf\_liverA, CL887.Contig2\_Mf\_liverA, Unigene31623\_Mf\_liverA, CL4919.Contig1\_Mf\_liverA, Unigene16529\_Mf\_liverA, Unigene11\_Mf\_liverA, Unigene32058\_Mf\_liverA, Unigene27082\_Mf\_liverA, CL5007.Contig1\_Mf\_liverA, CL1493.Contig2\_Mf\_liverA, CL442.Contig2\_Mf\_liverA, CL1493.Contig1\_Mf\_liverA, Unigene32233\_Mf\_liverA, NM\_008851, CL2855.Contig1\_Mf\_liverA, NM\_024434, Unigene24536\_Mf\_liverA, Unigene32059\_Mf\_liverA |
| intrinsic to membrane | NM\_011256, NM\_007396, Unigene13808\_Mf\_liverA, Unigene21684\_Mf\_liverA, NM\_176843, CL3835.Contig2\_Mf\_liverA, Unigene32891\_Mf\_liverA, Unigene26532\_Mf\_liverA, Unigene6959\_Mf\_liverA, CL4550.Contig2\_Mf\_liverA, Unigene15961\_Mf\_liverA, Unigene28244\_Mf\_liverA, Unigene24883\_Mf\_liverA, NM\_010378, NM\_011082, Unigene19687\_Mf\_liverA, Unigene36849\_Mf\_liverA, Unigene26053\_Mf\_liverA, Unigene25055\_Mf\_liverA, Unigene7336\_Mf\_liverA, CL2339.Contig1\_Mf\_liverA, Unigene36762\_Mf\_liverA, Unigene35507\_Mf\_liverA, CL238.Contig1\_Mf\_liverA, Unigene35407\_Mf\_liverA, Unigene14816\_Mf\_liverA, Unigene37698\_Mf\_liverA, NM\_015800, Unigene39886\_Mf\_liverA, Unigene30878\_Mf\_liverA, NM\_001039371, Unigene32920\_Mf\_liverA, Unigene27294\_Mf\_liverA, NR\_004446, Unigene18500\_Mf\_liverA, CL4910.Contig1\_Mf\_liverA, CL1256.Contig1\_Mf\_liverA, Unigene34789\_Mf\_liverA, CL695.Contig1\_Mf\_liverA, NM\_133718, NM\_008029, Unigene2\_Mf\_liverA, Unigene31333\_Mf\_liverA, Unigene13593\_Mf\_liverA, Unigene30369\_Mf\_liverA, NM\_134127, Unigene33598\_Mf\_liverA, Unigene34394\_Mf\_liverA, Unigene31694\_Mf\_liverA, Unigene14609\_Mf\_liverA, Unigene843\_Mf\_liverA, NM\_001004357, Unigene28852\_Mf\_liverA, CL2697.Contig4\_Mf\_liverA, Unigene34143\_Mf\_liverA, CL2574.Contig1\_Mf\_liverA, Unigene27218\_Mf\_liverA, NM\_016697, CL2411.Contig1\_Mf\_liverA, CL787.Contig1\_Mf\_liverA, Unigene15055\_Mf\_liverA, CL3154.Contig1\_Mf\_liverA, CL5807.Contig1\_Mf\_liverA, Unigene13363\_Mf\_liverA, CL2855.Contig2\_Mf\_liverA, Unigene13097\_Mf\_liverA, CL4293.Contig1\_Mf\_liverA, CL784.Contig3\_Mf\_liverA, Unigene1479\_Mf\_liverA, CL4456.Contig1\_Mf\_liverA, CL838.Contig3\_Mf\_liverA, Unigene5632\_Mf\_liverA, CL4550.Contig3\_Mf\_liverA, Unigene21816\_Mf\_liverA, CL2142.Contig2\_Mf\_liverA, Unigene7264\_Mf\_liverA, Unigene37420\_Mf\_liverA, CL153.Contig2\_Mf\_liverA, NM\_153589, CL4095.Contig4\_Mf\_liverA, CL529.Contig2\_Mf\_liverA, Unigene24166\_Mf\_liverA, Unigene25388\_Mf\_liverA, CL4283.Contig1\_Mf\_liverA, Unigene34032\_Mf\_liverA, Unigene27547\_Mf\_liverA, Unigene24090\_Mf\_liverA, Unigene32789\_Mf\_liverA, Unigene36898\_Mf\_liverA, CL5316.Contig1\_Mf\_liverA, Unigene37389\_Mf\_liverA, Unigene14070\_Mf\_liverA, Unigene32332\_Mf\_liverA, Unigene860\_Mf\_liverA, Unigene35506\_Mf\_liverA, Unigene32852\_Mf\_liverA, Unigene27082\_Mf\_liverA, Unigene5712\_Mf\_liverA, Unigene14541\_Mf\_liverA, Unigene1288\_Mf\_liverA, NM\_010380, CL275.Contig5\_Mf\_liverA, Unigene15184\_Mf\_liverA, CL2855.Contig1\_Mf\_liverA, Unigene30697\_Mf\_liverA, Unigene33760\_Mf\_liverA, Unigene5169\_Mf\_liverA, NM\_013821, Unigene40796\_Mf\_liverA, Unigene17569\_Mf\_liverA, Unigene31051\_Mf\_liverA, NM\_008365, Unigene4363\_Mf\_liverA, Unigene42606\_Mf\_liverA, CL4228.Contig1\_Mf\_liverA, NM\_009178, Unigene49971\_Mf\_liverA, CL425.Contig1\_Mf\_liverA, Unigene38307\_Mf\_liverA, CL3150.Contig1\_Mf\_liverA, CL2797.Contig2\_Mf\_liverA, Unigene34754\_Mf\_liverA, Unigene4697\_Mf\_liverA, Unigene33746\_Mf\_liverA, Unigene28143\_Mf\_liverA, Unigene37526\_Mf\_liverA, Unigene30217\_Mf\_liverA, Unigene28853\_Mf\_liverA, Unigene27922\_Mf\_liverA, CL4600.Contig1\_Mf\_liverA, CL5349.Contig1\_Mf\_liverA, CL3382.Contig1\_Mf\_liverA, NM\_029582, CL1100.Contig1\_Mf\_liverA, Unigene35958\_Mf\_liverA, NM\_016689, CL4583.Contig2\_Mf\_liverA, CL485.Contig1\_Mf\_liverA, CL1988.Contig1\_Mf\_liverA, Unigene4938\_Mf\_liverA, Unigene32803\_Mf\_liverA, Unigene5292\_Mf\_liverA, NM\_010141, CL3002.Contig1\_Mf\_liverA, Unigene36315\_Mf\_liverA, Unigene31267\_Mf\_liverA, CL1988.Contig3\_Mf\_liverA, CL2327.Contig1\_Mf\_liverA, NM\_172679, Unigene37224\_Mf\_liverA, Unigene36949\_Mf\_liverA, CL4033.Contig1\_Mf\_liverA, NM\_001033304, CL887.Contig2\_Mf\_liverA, Unigene5488\_Mf\_liverA, Unigene25994\_Mf\_liverA, Unigene17916\_Mf\_liverA, Unigene33736\_Mf\_liverA, CL3519.Contig1\_Mf\_liverA, Unigene29426\_Mf\_liverA, Unigene21026\_Mf\_liverA, Unigene35406\_Mf\_liverA, Unigene20371\_Mf\_liverA, CL1656.Contig1\_Mf\_liverA, CL838.Contig6\_Mf\_liverA, Unigene10915\_Mf\_liverA, Unigene24471\_Mf\_liverA, CL1076.Contig1\_Mf\_liverA, Unigene33080\_Mf\_liverA, Unigene15888\_Mf\_liverA, NM\_198865, CL4220.Contig1\_Mf\_liverA, Unigene39437\_Mf\_liverA, CL4338.Contig1\_Mf\_liverA, Unigene15433\_Mf\_liverA, CL5307.Contig1\_Mf\_liverA, Unigene5750\_Mf\_liverA, Unigene31670\_Mf\_liverA, NM\_178405, NM\_007822, Unigene13624\_Mf\_liverA, NM\_018797, NM\_172203, Unigene30547\_Mf\_liverA, Unigene15470\_Mf\_liverA, Unigene39011\_Mf\_liverA, Unigene24225\_Mf\_liverA, Unigene37344\_Mf\_liverA, NM\_145218, CL4007.Contig1\_Mf\_liverA, Unigene25052\_Mf\_liverA, Unigene13950\_Mf\_liverA, Unigene37245\_Mf\_liverA, Unigene33624\_Mf\_liverA, CL4984.Contig1\_Mf\_liverA, Unigene281\_Mf\_liverA, Unigene32335\_Mf\_liverA, Unigene20372\_Mf\_liverA, NM\_011978, NM\_177652, Unigene30548\_Mf\_liverA, Unigene26951\_Mf\_liverA, Unigene17842\_Mf\_liverA, CL3104.Contig1\_Mf\_liverA, Unigene36593\_Mf\_liverA, Unigene5489\_Mf\_liverA, Unigene34444\_Mf\_liverA, Unigene16529\_Mf\_liverA, Unigene37232\_Mf\_liverA, Unigene33720\_Mf\_liverA, CL5007.Contig1\_Mf\_liverA, CL5698.Contig1\_Mf\_liverA, Unigene37366\_Mf\_liverA, Unigene4681\_Mf\_liverA, CL2884.Contig2\_Mf\_liverA, Unigene37819\_Mf\_liverA, Unigene5886\_Mf\_liverA, Unigene35618\_Mf\_liverA |
| membrane | NM\_007396, Unigene13808\_Mf\_liverA, Unigene21684\_Mf\_liverA, NM\_176843, CL4302.Contig1\_Mf\_liverA, Unigene32891\_Mf\_liverA, Unigene26532\_Mf\_liverA, Unigene15961\_Mf\_liverA, NM\_010378, NM\_011082, Unigene19687\_Mf\_liverA, Unigene26053\_Mf\_liverA, Unigene7336\_Mf\_liverA, Unigene35507\_Mf\_liverA, CL238.Contig1\_Mf\_liverA, CL3617.Contig1\_Mf\_liverA, Unigene39886\_Mf\_liverA, Unigene32920\_Mf\_liverA, Unigene1137\_Mf\_liverA, Unigene27294\_Mf\_liverA, NR\_004446, Unigene34341\_Mf\_liverA, Unigene30153\_Mf\_liverA, Unigene30142\_Mf\_liverA, CL695.Contig1\_Mf\_liverA, Unigene2\_Mf\_liverA, Unigene31623\_Mf\_liverA, NM\_010233, NM\_145474, Unigene34394\_Mf\_liverA, Unigene31694\_Mf\_liverA, NM\_031165, CL497.Contig2\_Mf\_liverA, CL6.Contig1\_Mf\_liverA, Unigene34143\_Mf\_liverA, Unigene27218\_Mf\_liverA, NM\_016697, Unigene802\_Mf\_liverA, CL4816.Contig1\_Mf\_liverA, Unigene15055\_Mf\_liverA, CL3154.Contig1\_Mf\_liverA, CL5807.Contig1\_Mf\_liverA, Unigene13363\_Mf\_liverA, CL2855.Contig2\_Mf\_liverA, Unigene28162\_Mf\_liverA, Unigene13097\_Mf\_liverA, CL4293.Contig1\_Mf\_liverA, Unigene1479\_Mf\_liverA, CL4456.Contig1\_Mf\_liverA, NM\_010001, Unigene139\_Mf\_liverA, Unigene7264\_Mf\_liverA, Unigene37420\_Mf\_liverA, Unigene15572\_Mf\_liverA, Unigene31625\_Mf\_liverA, Unigene25388\_Mf\_liverA, Unigene24166\_Mf\_liverA, Unigene34032\_Mf\_liverA, Unigene27547\_Mf\_liverA, Unigene15290\_Mf\_liverA, Unigene24090\_Mf\_liverA, Unigene32789\_Mf\_liverA, Unigene36898\_Mf\_liverA, CL5316.Contig1\_Mf\_liverA, CL1688.Contig3\_Mf\_liverA, Unigene37389\_Mf\_liverA, Unigene14070\_Mf\_liverA, Unigene32332\_Mf\_liverA, Unigene4723\_Mf\_liverA, CL3930.Contig1\_Mf\_liverA, Unigene32852\_Mf\_liverA, Unigene24613\_Mf\_liverA, Unigene26611\_Mf\_liverA, Unigene1280\_Mf\_liverA, Unigene1288\_Mf\_liverA, CL3166.Contig4\_Mf\_liverA, CL275.Contig5\_Mf\_liverA, Unigene15184\_Mf\_liverA, Unigene30697\_Mf\_liverA, NM\_027959, Unigene5169\_Mf\_liverA, NM\_008365, Unigene4363\_Mf\_liverA, Unigene42606\_Mf\_liverA, CL425.Contig1\_Mf\_liverA, CL3150.Contig1\_Mf\_liverA, CL2797.Contig2\_Mf\_liverA, Unigene4697\_Mf\_liverA, Unigene28143\_Mf\_liverA, Unigene37243\_Mf\_liverA, Unigene30217\_Mf\_liverA, Unigene15077\_Mf\_liverA, CL4600.Contig1\_Mf\_liverA, CL5349.Contig1\_Mf\_liverA, CL3382.Contig1\_Mf\_liverA, Unigene5693\_Mf\_liverA, NM\_029582, Unigene35958\_Mf\_liverA, NM\_016689, Unigene28687\_Mf\_liverA, Unigene4938\_Mf\_liverA, Unigene32803\_Mf\_liverA, Unigene5292\_Mf\_liverA, CL1988.Contig3\_Mf\_liverA, Unigene37224\_Mf\_liverA, CL1810.Contig1\_Mf\_liverA, NM\_172679, CL4033.Contig1\_Mf\_liverA, NM\_001033304, Unigene34983\_Mf\_liverA, Unigene28662\_Mf\_liverA, Unigene17916\_Mf\_liverA, CL3519.Contig1\_Mf\_liverA, Unigene29426\_Mf\_liverA, Unigene21026\_Mf\_liverA, Unigene20371\_Mf\_liverA, CL1493.Contig1\_Mf\_liverA, Unigene542\_Mf\_liverA, Unigene24471\_Mf\_liverA, Unigene33080\_Mf\_liverA, NM\_198865, Unigene15888\_Mf\_liverA, CL4220.Contig1\_Mf\_liverA, Unigene15433\_Mf\_liverA, CL4338.Contig1\_Mf\_liverA, NM\_019817, Unigene5750\_Mf\_liverA, NM\_019879, NM\_007822, Unigene15470\_Mf\_liverA, CL4816.Contig3\_Mf\_liverA, CL114.Contig1\_Mf\_liverA, Unigene39011\_Mf\_liverA, Unigene24225\_Mf\_liverA, Unigene37344\_Mf\_liverA, NM\_011207, NM\_145218, Unigene36189\_Mf\_liverA, NM\_008183, Unigene13950\_Mf\_liverA, Unigene37245\_Mf\_liverA, Unigene33624\_Mf\_liverA, Unigene281\_Mf\_liverA, NM\_177652, Unigene20372\_Mf\_liverA, NM\_011978, Unigene30548\_Mf\_liverA, CL3104.Contig1\_Mf\_liverA, Unigene36593\_Mf\_liverA, NM\_010002, Unigene16529\_Mf\_liverA, Unigene37232\_Mf\_liverA, NM\_026313, CL5007.Contig1\_Mf\_liverA, CL5698.Contig1\_Mf\_liverA, CL591.Contig1\_Mf\_liverA, Unigene35618\_Mf\_liverA, NM\_011256, CL5640.Contig1\_Mf\_liverA, Unigene38015\_Mf\_liverA, CL3835.Contig2\_Mf\_liverA, NM\_206537, Unigene6959\_Mf\_liverA, CL4550.Contig2\_Mf\_liverA, Unigene28244\_Mf\_liverA, Unigene14603\_Mf\_liverA, Unigene24883\_Mf\_liverA, CL523.Contig1\_Mf\_liverA, NM\_008277, Unigene880\_Mf\_liverA, Unigene8473\_Mf\_liverA, Unigene36849\_Mf\_liverA, Unigene25055\_Mf\_liverA, CL482.Contig1\_Mf\_liverA, CL2339.Contig1\_Mf\_liverA, Unigene36762\_Mf\_liverA, Unigene35407\_Mf\_liverA, Unigene18795\_Mf\_liverA, Unigene14816\_Mf\_liverA, NM\_015800, Unigene37698\_Mf\_liverA, Unigene11922\_Mf\_liverA, Unigene30878\_Mf\_liverA, NM\_001039371, CL6038.Contig2\_Mf\_liverA, Unigene2798\_Mf\_liverA, Unigene18500\_Mf\_liverA, Unigene32570\_Mf\_liverA, CL4910.Contig1\_Mf\_liverA, CL1256.Contig1\_Mf\_liverA, Unigene15295\_Mf\_liverA, Unigene34789\_Mf\_liverA, NM\_133718, NM\_008029, Unigene31333\_Mf\_liverA, Unigene13593\_Mf\_liverA, NM\_134127, Unigene30369\_Mf\_liverA, Unigene33598\_Mf\_liverA, Unigene11\_Mf\_liverA, Unigene4630\_Mf\_liverA, CL2622.Contig2\_Mf\_liverA, Unigene14609\_Mf\_liverA, Unigene843\_Mf\_liverA, Unigene28852\_Mf\_liverA, NM\_001004357, CL1493.Contig2\_Mf\_liverA, CL2697.Contig4\_Mf\_liverA, CL2574.Contig1\_Mf\_liverA, CL2411.Contig1\_Mf\_liverA, CL787.Contig1\_Mf\_liverA, Unigene29490\_Mf\_liverA, Unigene12967\_Mf\_liverA, Unigene31988\_Mf\_liverA, CL3599.Contig1\_Mf\_liverA, CL784.Contig3\_Mf\_liverA, CL838.Contig3\_Mf\_liverA, Unigene13143\_Mf\_liverA, Unigene5632\_Mf\_liverA, CL1263.Contig1\_Mf\_liverA, CL4550.Contig3\_Mf\_liverA, Unigene21816\_Mf\_liverA, CL258.Contig1\_Mf\_liverA, NM\_007811, NM\_008538, CL2142.Contig2\_Mf\_liverA, CL153.Contig2\_Mf\_liverA, NM\_153589, CL4095.Contig4\_Mf\_liverA, CL529.Contig2\_Mf\_liverA, CL4283.Contig1\_Mf\_liverA, Unigene30584\_Mf\_liverA, Unigene17579\_Mf\_liverA, Unigene860\_Mf\_liverA, Unigene7195\_Mf\_liverA, Unigene35506\_Mf\_liverA, Unigene27082\_Mf\_liverA, Unigene14541\_Mf\_liverA, Unigene5712\_Mf\_liverA, CL4650.Contig2\_Mf\_liverA, Unigene1205\_Mf\_liverA, CL442.Contig2\_Mf\_liverA, CL6039.Contig1\_Mf\_liverA, NM\_010380, CL2855.Contig1\_Mf\_liverA, Unigene33760\_Mf\_liverA, NM\_013821, Unigene40796\_Mf\_liverA, Unigene17569\_Mf\_liverA, NM\_001100182, Unigene31051\_Mf\_liverA, CL4228.Contig1\_Mf\_liverA, NM\_009178, Unigene49971\_Mf\_liverA, Unigene38307\_Mf\_liverA, Unigene36190\_Mf\_liverA, Unigene34754\_Mf\_liverA, Unigene33746\_Mf\_liverA, Unigene30154\_Mf\_liverA, CL1125.Contig1\_Mf\_liverA, Unigene37526\_Mf\_liverA, Unigene28853\_Mf\_liverA, Unigene27922\_Mf\_liverA, Unigene15681\_Mf\_liverA, CL1100.Contig1\_Mf\_liverA, CL4583.Contig2\_Mf\_liverA, NM\_021423, CL485.Contig1\_Mf\_liverA, CL1988.Contig1\_Mf\_liverA, Unigene36328\_Mf\_liverA, NM\_010141, CL3002.Contig1\_Mf\_liverA, Unigene36315\_Mf\_liverA, Unigene31267\_Mf\_liverA, CL2327.Contig1\_Mf\_liverA, Unigene36949\_Mf\_liverA, CL887.Contig2\_Mf\_liverA, Unigene5488\_Mf\_liverA, Unigene25994\_Mf\_liverA, Unigene33736\_Mf\_liverA, Unigene35406\_Mf\_liverA, CL1656.Contig1\_Mf\_liverA, NM\_001100181, CL838.Contig6\_Mf\_liverA, Unigene5607\_Mf\_liverA, Unigene10915\_Mf\_liverA, CL114.Contig2\_Mf\_liverA, Unigene13196\_Mf\_liverA, CL1076.Contig1\_Mf\_liverA, Unigene39437\_Mf\_liverA, Unigene22423\_Mf\_liverA, Unigene18796\_Mf\_liverA, Unigene13616\_Mf\_liverA, CL5307.Contig1\_Mf\_liverA, Unigene31670\_Mf\_liverA, NM\_178405, Unigene13624\_Mf\_liverA, NM\_018797, NM\_172203, Unigene30547\_Mf\_liverA, Unigene120\_Mf\_liverA, CL4007.Contig1\_Mf\_liverA, Unigene25052\_Mf\_liverA, CL55.Contig1\_Mf\_liverA, Unigene550\_Mf\_liverA, CL4984.Contig1\_Mf\_liverA, Unigene4863\_Mf\_liverA, CL2355.Contig1\_Mf\_liverA, Unigene32335\_Mf\_liverA, Unigene26951\_Mf\_liverA, Unigene17842\_Mf\_liverA, Unigene5489\_Mf\_liverA, Unigene34444\_Mf\_liverA, Unigene33720\_Mf\_liverA, CL3911.Contig2\_Mf\_liverA, Unigene44057\_Mf\_liverA, Unigene37366\_Mf\_liverA, Unigene4681\_Mf\_liverA, Unigene12519\_Mf\_liverA, NM\_008851, CL2884.Contig2\_Mf\_liverA, Unigene37819\_Mf\_liverA, Unigene5886\_Mf\_liverA, CL336.Contig3\_Mf\_liverA, CL1198.Contig1\_Mf\_liverA |
| proteasome activator complex | Unigene36851\_Mf\_liverA, CL1119.Contig1\_Mf\_liverA |
| vacuolar membrane | CL4583.Contig2\_Mf\_liverA, Unigene24471\_Mf\_liverA, Unigene36328\_Mf\_liverA, CL3835.Contig2\_Mf\_liverA, CL3002.Contig1\_Mf\_liverA, CL2855.Contig2\_Mf\_liverA, Unigene4681\_Mf\_liverA, Unigene32332\_Mf\_liverA, Unigene39886\_Mf\_liverA, CL2855.Contig1\_Mf\_liverA, Unigene30878\_Mf\_liverA, Unigene31670\_Mf\_liverA |
| integral to endoplasmic reticulum membrane | Unigene15888\_Mf\_liverA, CL2855.Contig2\_Mf\_liverA, CL695.Contig1\_Mf\_liverA, Unigene5489\_Mf\_liverA, CL2855.Contig1\_Mf\_liverA, Unigene5488\_Mf\_liverA, Unigene1479\_Mf\_liverA, Unigene34444\_Mf\_liverA |
| cytoplasmic vesicle | Unigene15055\_Mf\_liverA, CL5807.Contig1\_Mf\_liverA, Unigene33080\_Mf\_liverA, Unigene13363\_Mf\_liverA, CL2855.Contig2\_Mf\_liverA, CL4263.Contig1\_Mf\_liverA, NM\_021278, Unigene6959\_Mf\_liverA, Unigene38307\_Mf\_liverA, Unigene28244\_Mf\_liverA, Unigene36190\_Mf\_liverA, NM\_019817, Unigene5750\_Mf\_liverA, CL838.Contig3\_Mf\_liverA, NM\_011082, Unigene19687\_Mf\_liverA, CL5978.Contig2\_Mf\_liverA, Unigene5632\_Mf\_liverA, Unigene33746\_Mf\_liverA, Unigene28143\_Mf\_liverA, Unigene36762\_Mf\_liverA, Unigene27922\_Mf\_liverA, Unigene15470\_Mf\_liverA, Unigene600\_Mf\_liverA, Unigene36189\_Mf\_liverA, Unigene32093\_Mf\_liverA, Unigene35958\_Mf\_liverA, Unigene13950\_Mf\_liverA, Unigene30584\_Mf\_liverA, CL6038.Contig2\_Mf\_liverA, Unigene32789\_Mf\_liverA, Unigene36328\_Mf\_liverA, Unigene38280\_Mf\_liverA, Unigene36898\_Mf\_liverA, Unigene32335\_Mf\_liverA, Unigene37389\_Mf\_liverA, Unigene34789\_Mf\_liverA, Unigene14809\_Mf\_liverA, CL5978.Contig3\_Mf\_liverA, Unigene28662\_Mf\_liverA, CL4919.Contig1\_Mf\_liverA, CL3911.Contig2\_Mf\_liverA, Unigene27082\_Mf\_liverA, NM\_009776, Unigene34394\_Mf\_liverA, Unigene14609\_Mf\_liverA, CL5007.Contig1\_Mf\_liverA, NM\_031165, CL497.Contig2\_Mf\_liverA, CL1493.Contig2\_Mf\_liverA, Unigene14810\_Mf\_liverA, CL2697.Contig4\_Mf\_liverA, CL1493.Contig1\_Mf\_liverA, CL6039.Contig1\_Mf\_liverA, CL2855.Contig1\_Mf\_liverA, Unigene37819\_Mf\_liverA, NM\_027959 |
| proteinaceous extracellular matrix | Unigene33441\_Mf\_liverA, Unigene32110\_Mf\_liverA, NM\_177033, Unigene29628\_Mf\_liverA, Unigene15290\_Mf\_liverA, Unigene32908\_Mf\_liverA, Unigene34375\_Mf\_liverA, Unigene37904\_Mf\_liverA, Unigene29082\_Mf\_liverA, Unigene28899\_Mf\_liverA, Unigene20512\_Mf\_liverA, Unigene13616\_Mf\_liverA, NM\_010233, NM\_008483, Unigene32434\_Mf\_liverA, Unigene28564\_Mf\_liverA, Unigene23328\_Mf\_liverA, Unigene34124\_Mf\_liverA, Unigene31492\_Mf\_liverA, Unigene30584\_Mf\_liverA |
| endosome membrane | CL55.Contig1\_Mf\_liverA, Unigene24471\_Mf\_liverA, Unigene38015\_Mf\_liverA, Unigene15077\_Mf\_liverA, Unigene29490\_Mf\_liverA, CL497.Contig2\_Mf\_liverA, CL3835.Contig2\_Mf\_liverA, CL3002.Contig1\_Mf\_liverA, Unigene32335\_Mf\_liverA, Unigene4681\_Mf\_liverA, Unigene5693\_Mf\_liverA, Unigene30878\_Mf\_liverA, NM\_011082, Unigene16529\_Mf\_liverA, Unigene33736\_Mf\_liverA |
| membrane part | NM\_011256, NM\_007396, Unigene13808\_Mf\_liverA, Unigene21684\_Mf\_liverA, NM\_176843, CL4302.Contig1\_Mf\_liverA, CL3835.Contig2\_Mf\_liverA, Unigene32891\_Mf\_liverA, Unigene26532\_Mf\_liverA, Unigene6959\_Mf\_liverA, CL4550.Contig2\_Mf\_liverA, Unigene15961\_Mf\_liverA, Unigene28244\_Mf\_liverA, Unigene24883\_Mf\_liverA, NM\_010378, CL523.Contig1\_Mf\_liverA, Unigene880\_Mf\_liverA, NM\_011082, Unigene19687\_Mf\_liverA, Unigene36849\_Mf\_liverA, Unigene26053\_Mf\_liverA, Unigene25055\_Mf\_liverA, Unigene7336\_Mf\_liverA, CL482.Contig1\_Mf\_liverA, CL2339.Contig1\_Mf\_liverA, Unigene36762\_Mf\_liverA, Unigene35507\_Mf\_liverA, CL238.Contig1\_Mf\_liverA, Unigene35407\_Mf\_liverA, Unigene14816\_Mf\_liverA, Unigene37698\_Mf\_liverA, NM\_015800, Unigene39886\_Mf\_liverA, Unigene30878\_Mf\_liverA, NM\_001039371, Unigene32920\_Mf\_liverA, Unigene1137\_Mf\_liverA, Unigene27294\_Mf\_liverA, NR\_004446, Unigene18500\_Mf\_liverA, CL4910.Contig1\_Mf\_liverA, CL1256.Contig1\_Mf\_liverA, Unigene34341\_Mf\_liverA, Unigene34789\_Mf\_liverA, CL695.Contig1\_Mf\_liverA, NM\_133718, NM\_008029, Unigene2\_Mf\_liverA, Unigene31333\_Mf\_liverA, Unigene13593\_Mf\_liverA, NM\_010233, Unigene30369\_Mf\_liverA, NM\_134127, Unigene33598\_Mf\_liverA, Unigene4630\_Mf\_liverA, Unigene34394\_Mf\_liverA, Unigene31694\_Mf\_liverA, Unigene14609\_Mf\_liverA, Unigene843\_Mf\_liverA, NM\_001004357, Unigene28852\_Mf\_liverA, CL6.Contig1\_Mf\_liverA, CL2697.Contig4\_Mf\_liverA, Unigene34143\_Mf\_liverA, CL2574.Contig1\_Mf\_liverA, Unigene27218\_Mf\_liverA, NM\_016697, CL2411.Contig1\_Mf\_liverA, CL4816.Contig1\_Mf\_liverA, CL787.Contig1\_Mf\_liverA, Unigene15055\_Mf\_liverA, CL3154.Contig1\_Mf\_liverA, CL5807.Contig1\_Mf\_liverA, Unigene13363\_Mf\_liverA, CL2855.Contig2\_Mf\_liverA, Unigene12967\_Mf\_liverA, Unigene13097\_Mf\_liverA, CL4293.Contig1\_Mf\_liverA, CL784.Contig3\_Mf\_liverA, Unigene1479\_Mf\_liverA, CL4456.Contig1\_Mf\_liverA, CL838.Contig3\_Mf\_liverA, Unigene13143\_Mf\_liverA, Unigene5632\_Mf\_liverA, CL4550.Contig3\_Mf\_liverA, Unigene21816\_Mf\_liverA, CL2142.Contig2\_Mf\_liverA, Unigene139\_Mf\_liverA, Unigene7264\_Mf\_liverA, Unigene37420\_Mf\_liverA, CL153.Contig2\_Mf\_liverA, Unigene15572\_Mf\_liverA, NM\_153589, CL4095.Contig4\_Mf\_liverA, CL529.Contig2\_Mf\_liverA, Unigene25388\_Mf\_liverA, Unigene24166\_Mf\_liverA, Unigene30584\_Mf\_liverA, CL4283.Contig1\_Mf\_liverA, Unigene34032\_Mf\_liverA, Unigene27547\_Mf\_liverA, Unigene24090\_Mf\_liverA, Unigene32789\_Mf\_liverA, Unigene36898\_Mf\_liverA, CL5316.Contig1\_Mf\_liverA, Unigene37389\_Mf\_liverA, Unigene14070\_Mf\_liverA, Unigene32332\_Mf\_liverA, Unigene860\_Mf\_liverA, Unigene7195\_Mf\_liverA, Unigene35506\_Mf\_liverA, Unigene32852\_Mf\_liverA, Unigene27082\_Mf\_liverA, Unigene5712\_Mf\_liverA, Unigene14541\_Mf\_liverA, Unigene1280\_Mf\_liverA, Unigene1205\_Mf\_liverA, Unigene1288\_Mf\_liverA, CL3166.Contig4\_Mf\_liverA, NM\_010380, CL275.Contig5\_Mf\_liverA, Unigene15184\_Mf\_liverA, CL2855.Contig1\_Mf\_liverA, Unigene30697\_Mf\_liverA, Unigene33760\_Mf\_liverA, Unigene5169\_Mf\_liverA, NM\_013821, Unigene40796\_Mf\_liverA, Unigene17569\_Mf\_liverA, Unigene31051\_Mf\_liverA, NM\_008365, Unigene4363\_Mf\_liverA, Unigene42606\_Mf\_liverA, CL4228.Contig1\_Mf\_liverA, NM\_009178, Unigene49971\_Mf\_liverA, CL425.Contig1\_Mf\_liverA, Unigene38307\_Mf\_liverA, CL3150.Contig1\_Mf\_liverA, CL2797.Contig2\_Mf\_liverA, Unigene34754\_Mf\_liverA, Unigene4697\_Mf\_liverA, Unigene33746\_Mf\_liverA, Unigene28143\_Mf\_liverA, Unigene37243\_Mf\_liverA, Unigene37526\_Mf\_liverA, Unigene30217\_Mf\_liverA, Unigene28853\_Mf\_liverA, Unigene27922\_Mf\_liverA, CL4600.Contig1\_Mf\_liverA, CL5349.Contig1\_Mf\_liverA, CL3382.Contig1\_Mf\_liverA, Unigene5693\_Mf\_liverA, NM\_029582, CL1100.Contig1\_Mf\_liverA, Unigene35958\_Mf\_liverA, NM\_016689, CL4583.Contig2\_Mf\_liverA, NM\_021423, CL485.Contig1\_Mf\_liverA, CL1988.Contig1\_Mf\_liverA, Unigene4938\_Mf\_liverA, Unigene32803\_Mf\_liverA, Unigene5292\_Mf\_liverA, Unigene36328\_Mf\_liverA, NM\_010141, CL3002.Contig1\_Mf\_liverA, Unigene36315\_Mf\_liverA, Unigene31267\_Mf\_liverA, CL1988.Contig3\_Mf\_liverA, CL1810.Contig1\_Mf\_liverA, CL2327.Contig1\_Mf\_liverA, NM\_172679, Unigene37224\_Mf\_liverA, Unigene36949\_Mf\_liverA, CL4033.Contig1\_Mf\_liverA, NM\_001033304, Unigene5488\_Mf\_liverA, CL887.Contig2\_Mf\_liverA, Unigene25994\_Mf\_liverA, Unigene17916\_Mf\_liverA, CL3519.Contig1\_Mf\_liverA, Unigene33736\_Mf\_liverA, Unigene29426\_Mf\_liverA, Unigene21026\_Mf\_liverA, Unigene35406\_Mf\_liverA, Unigene20371\_Mf\_liverA, CL1656.Contig1\_Mf\_liverA, CL838.Contig6\_Mf\_liverA, Unigene5607\_Mf\_liverA, Unigene10915\_Mf\_liverA, Unigene542\_Mf\_liverA, Unigene13196\_Mf\_liverA, CL114.Contig2\_Mf\_liverA, Unigene24471\_Mf\_liverA, CL1076.Contig1\_Mf\_liverA, Unigene33080\_Mf\_liverA, NM\_198865, Unigene15888\_Mf\_liverA, CL4220.Contig1\_Mf\_liverA, Unigene39437\_Mf\_liverA, Unigene22423\_Mf\_liverA, CL4338.Contig1\_Mf\_liverA, Unigene15433\_Mf\_liverA, CL5307.Contig1\_Mf\_liverA, NM\_019817, Unigene5750\_Mf\_liverA, Unigene31670\_Mf\_liverA, NM\_178405, NM\_007822, Unigene13624\_Mf\_liverA, NM\_018797, Unigene30547\_Mf\_liverA, NM\_172203, Unigene15470\_Mf\_liverA, CL4816.Contig3\_Mf\_liverA, CL114.Contig1\_Mf\_liverA, Unigene39011\_Mf\_liverA, Unigene24225\_Mf\_liverA, Unigene37344\_Mf\_liverA, NM\_011207, NM\_145218, CL4007.Contig1\_Mf\_liverA, Unigene25052\_Mf\_liverA, Unigene13950\_Mf\_liverA, Unigene37245\_Mf\_liverA, Unigene33624\_Mf\_liverA, CL4984.Contig1\_Mf\_liverA, Unigene281\_Mf\_liverA, Unigene20372\_Mf\_liverA, NM\_011978, Unigene32335\_Mf\_liverA, NM\_177652, Unigene30548\_Mf\_liverA, Unigene26951\_Mf\_liverA, Unigene17842\_Mf\_liverA, CL3104.Contig1\_Mf\_liverA, Unigene36593\_Mf\_liverA, Unigene5489\_Mf\_liverA, Unigene34444\_Mf\_liverA, Unigene16529\_Mf\_liverA, Unigene37232\_Mf\_liverA, Unigene33720\_Mf\_liverA, NM\_026313, Unigene44057\_Mf\_liverA, CL5007.Contig1\_Mf\_liverA, CL5698.Contig1\_Mf\_liverA, Unigene37366\_Mf\_liverA, Unigene4681\_Mf\_liverA, Unigene12519\_Mf\_liverA, CL2884.Contig2\_Mf\_liverA, CL591.Contig1\_Mf\_liverA, Unigene37819\_Mf\_liverA, Unigene5886\_Mf\_liverA, CL336.Contig3\_Mf\_liverA, Unigene35618\_Mf\_liverA |
| membrane raft | Unigene27082\_Mf\_liverA, CL3154.Contig1\_Mf\_liverA, Unigene32803\_Mf\_liverA, Unigene32789\_Mf\_liverA, Unigene27922\_Mf\_liverA, CL5698.Contig1\_Mf\_liverA, Unigene31267\_Mf\_liverA, Unigene32335\_Mf\_liverA, CL1810.Contig1\_Mf\_liverA, Unigene39437\_Mf\_liverA, Unigene12519\_Mf\_liverA, CL887.Contig2\_Mf\_liverA, Unigene31333\_Mf\_liverA, Unigene13950\_Mf\_liverA |
| endosomal part | CL55.Contig1\_Mf\_liverA, Unigene24471\_Mf\_liverA, Unigene38015\_Mf\_liverA, Unigene15077\_Mf\_liverA, Unigene29490\_Mf\_liverA, CL497.Contig2\_Mf\_liverA, CL3835.Contig2\_Mf\_liverA, CL3002.Contig1\_Mf\_liverA, Unigene32335\_Mf\_liverA, Unigene4681\_Mf\_liverA, Unigene5693\_Mf\_liverA, Unigene30878\_Mf\_liverA, NM\_011082, Unigene16529\_Mf\_liverA, Unigene33736\_Mf\_liverA |
| early endosome | Unigene33746\_Mf\_liverA, Unigene37245\_Mf\_liverA, Unigene27082\_Mf\_liverA, Unigene14609\_Mf\_liverA, Unigene36698\_Mf\_liverA, Unigene15077\_Mf\_liverA, Unigene27922\_Mf\_liverA, CL3002.Contig1\_Mf\_liverA, Unigene836\_Mf\_liverA, Unigene49971\_Mf\_liverA, Unigene28244\_Mf\_liverA, Unigene32093\_Mf\_liverA, NR\_004446 |
| hemoglobin complex | Unigene12999\_Mf\_liverA, CL3684.Contig2\_Mf\_liverA |
| chromaffin granule membrane | Unigene34394\_Mf\_liverA, CL5807.Contig1\_Mf\_liverA |
| protein-lipid complex | CL3911.Contig2\_Mf\_liverA, Unigene36698\_Mf\_liverA, Unigene13498\_Mf\_liverA, Unigene9081\_Mf\_liverA, Unigene30815\_Mf\_liverA, Unigene30814\_Mf\_liverA, Unigene24157\_Mf\_liverA |
| plasma lipoprotein particle | CL3911.Contig2\_Mf\_liverA, Unigene36698\_Mf\_liverA, Unigene13498\_Mf\_liverA, Unigene9081\_Mf\_liverA, Unigene30815\_Mf\_liverA, Unigene30814\_Mf\_liverA, Unigene24157\_Mf\_liverA |
| apical plasma membrane | Unigene27082\_Mf\_liverA, Unigene32803\_Mf\_liverA, Unigene36898\_Mf\_liverA, Unigene139\_Mf\_liverA, Unigene32335\_Mf\_liverA, Unigene1288\_Mf\_liverA, CL4033.Contig1\_Mf\_liverA, CL591.Contig1\_Mf\_liverA, CL336.Contig3\_Mf\_liverA, Unigene5750\_Mf\_liverA, CL4007.Contig1\_Mf\_liverA, Unigene880\_Mf\_liverA, CL4095.Contig4\_Mf\_liverA, CL3519.Contig1\_Mf\_liverA |
| apical part of cell | Unigene32803\_Mf\_liverA, Unigene36898\_Mf\_liverA, Unigene32335\_Mf\_liverA, CL4033.Contig1\_Mf\_liverA, Unigene28244\_Mf\_liverA, Unigene5750\_Mf\_liverA, CL887.Contig2\_Mf\_liverA, Unigene880\_Mf\_liverA, Unigene13593\_Mf\_liverA, CL3519.Contig1\_Mf\_liverA, Unigene27082\_Mf\_liverA, Unigene1288\_Mf\_liverA, Unigene139\_Mf\_liverA, CL591.Contig1\_Mf\_liverA, CL4007.Contig1\_Mf\_liverA, CL336.Contig3\_Mf\_liverA, CL4095.Contig4\_Mf\_liverA |
| neuronal cell body | Unigene18500\_Mf\_liverA, Unigene27547\_Mf\_liverA, Unigene36898\_Mf\_liverA, Unigene34789\_Mf\_liverA, CL425.Contig1\_Mf\_liverA, Unigene13593\_Mf\_liverA, Unigene16529\_Mf\_liverA, Unigene33746\_Mf\_liverA, Unigene26611\_Mf\_liverA, Unigene14609\_Mf\_liverA, Unigene5712\_Mf\_liverA, Unigene27922\_Mf\_liverA, Unigene14461\_Mf\_liverA, Unigene16184\_Mf\_liverA, CL2697.Contig4\_Mf\_liverA, CL3166.Contig4\_Mf\_liverA, Unigene28564\_Mf\_liverA |
| intrinsic to organelle membrane | CL787.Contig1\_Mf\_liverA, Unigene31051\_Mf\_liverA, CL1076.Contig1\_Mf\_liverA, Unigene15888\_Mf\_liverA, CL2855.Contig2\_Mf\_liverA, CL695.Contig1\_Mf\_liverA, NM\_009178, CL3104.Contig1\_Mf\_liverA, Unigene5489\_Mf\_liverA, CL784.Contig3\_Mf\_liverA, Unigene34444\_Mf\_liverA, Unigene5488\_Mf\_liverA, Unigene1479\_Mf\_liverA, CL3382.Contig1\_Mf\_liverA, CL2855.Contig1\_Mf\_liverA, Unigene32920\_Mf\_liverA |
| proteasome accessory complex | CL4141.Contig1\_Mf\_liverA, Unigene36851\_Mf\_liverA, CL1119.Contig1\_Mf\_liverA |
| chromaffin granule | Unigene34394\_Mf\_liverA, CL5807.Contig1\_Mf\_liverA |
| melanosome | Unigene34789\_Mf\_liverA, Unigene36762\_Mf\_liverA, Unigene36190\_Mf\_liverA, Unigene36189\_Mf\_liverA, Unigene600\_Mf\_liverA, CL4263.Contig1\_Mf\_liverA |
| secretory granule | Unigene15055\_Mf\_liverA, CL6038.Contig2\_Mf\_liverA, CL5807.Contig1\_Mf\_liverA, Unigene32789\_Mf\_liverA, Unigene36898\_Mf\_liverA, Unigene33080\_Mf\_liverA, NM\_021278, Unigene14809\_Mf\_liverA, Unigene28662\_Mf\_liverA, Unigene19687\_Mf\_liverA, Unigene34394\_Mf\_liverA, NM\_009776, CL3911.Contig2\_Mf\_liverA, CL5007.Contig1\_Mf\_liverA, Unigene14810\_Mf\_liverA, CL2697.Contig4\_Mf\_liverA, Unigene35958\_Mf\_liverA, Unigene13950\_Mf\_liverA, Unigene30584\_Mf\_liverA |
| cytoplasmic membrane-bounded vesicle | Unigene15055\_Mf\_liverA, CL5807.Contig1\_Mf\_liverA, Unigene33080\_Mf\_liverA, Unigene13363\_Mf\_liverA, CL2855.Contig2\_Mf\_liverA, CL4263.Contig1\_Mf\_liverA, NM\_021278, Unigene36190\_Mf\_liverA, NM\_019817, CL838.Contig3\_Mf\_liverA, NM\_011082, Unigene19687\_Mf\_liverA, Unigene5632\_Mf\_liverA, Unigene33746\_Mf\_liverA, Unigene28143\_Mf\_liverA, Unigene36762\_Mf\_liverA, Unigene15470\_Mf\_liverA, Unigene600\_Mf\_liverA, Unigene36189\_Mf\_liverA, Unigene32093\_Mf\_liverA, Unigene35958\_Mf\_liverA, Unigene13950\_Mf\_liverA, Unigene30584\_Mf\_liverA, CL6038.Contig2\_Mf\_liverA, Unigene32789\_Mf\_liverA, Unigene36328\_Mf\_liverA, Unigene36898\_Mf\_liverA, Unigene32335\_Mf\_liverA, Unigene37389\_Mf\_liverA, Unigene34789\_Mf\_liverA, Unigene14809\_Mf\_liverA, Unigene28662\_Mf\_liverA, CL4919.Contig1\_Mf\_liverA, Unigene27082\_Mf\_liverA, NM\_009776, Unigene34394\_Mf\_liverA, CL3911.Contig2\_Mf\_liverA, Unigene14609\_Mf\_liverA, NM\_031165, CL5007.Contig1\_Mf\_liverA, CL497.Contig2\_Mf\_liverA, Unigene14810\_Mf\_liverA, CL2697.Contig4\_Mf\_liverA, CL2855.Contig1\_Mf\_liverA, Unigene37819\_Mf\_liverA, NM\_027959 |
| extracellular matrix | Unigene33441\_Mf\_liverA, CL1372.Contig1\_Mf\_liverA, Unigene32110\_Mf\_liverA, NM\_177033, Unigene29628\_Mf\_liverA, Unigene15290\_Mf\_liverA, Unigene32908\_Mf\_liverA, Unigene34375\_Mf\_liverA, Unigene37904\_Mf\_liverA, Unigene29082\_Mf\_liverA, Unigene28899\_Mf\_liverA, Unigene20512\_Mf\_liverA, Unigene13616\_Mf\_liverA, NM\_010233, NM\_145584, NM\_008483, Unigene32434\_Mf\_liverA, Unigene33683\_Mf\_liverA, Unigene28564\_Mf\_liverA, Unigene23328\_Mf\_liverA, Unigene34124\_Mf\_liverA, NM\_016697, Unigene30584\_Mf\_liverA, Unigene31492\_Mf\_liverA |
| endocytic vesicle | CL3911.Contig2\_Mf\_liverA, Unigene34394\_Mf\_liverA, CL5807.Contig1\_Mf\_liverA, Unigene36328\_Mf\_liverA, CL497.Contig2\_Mf\_liverA, CL2855.Contig2\_Mf\_liverA, Unigene32335\_Mf\_liverA, Unigene37389\_Mf\_liverA, Unigene14809\_Mf\_liverA, CL2855.Contig1\_Mf\_liverA |
| intrinsic to endoplasmic reticulum membrane | Unigene15888\_Mf\_liverA, CL2855.Contig2\_Mf\_liverA, CL695.Contig1\_Mf\_liverA, Unigene5489\_Mf\_liverA, CL2855.Contig1\_Mf\_liverA, CL784.Contig3\_Mf\_liverA, Unigene5488\_Mf\_liverA, Unigene1479\_Mf\_liverA, Unigene34444\_Mf\_liverA |
| endocytic vesicle membrane | Unigene34394\_Mf\_liverA, CL2855.Contig1\_Mf\_liverA, CL5807.Contig1\_Mf\_liverA, Unigene36328\_Mf\_liverA, CL2855.Contig2\_Mf\_liverA, Unigene32335\_Mf\_liverA |
| vesicle | Unigene15055\_Mf\_liverA, CL5807.Contig1\_Mf\_liverA, Unigene33080\_Mf\_liverA, Unigene13363\_Mf\_liverA, CL2855.Contig2\_Mf\_liverA, CL4263.Contig1\_Mf\_liverA, NM\_021278, Unigene6959\_Mf\_liverA, Unigene38307\_Mf\_liverA, Unigene28244\_Mf\_liverA, Unigene36190\_Mf\_liverA, NM\_019817, Unigene5750\_Mf\_liverA, CL838.Contig3\_Mf\_liverA, NM\_011082, Unigene19687\_Mf\_liverA, CL5978.Contig2\_Mf\_liverA, Unigene5632\_Mf\_liverA, Unigene33746\_Mf\_liverA, Unigene28143\_Mf\_liverA, Unigene36762\_Mf\_liverA, Unigene27922\_Mf\_liverA, Unigene15470\_Mf\_liverA, Unigene600\_Mf\_liverA, Unigene36189\_Mf\_liverA, Unigene32093\_Mf\_liverA, Unigene35958\_Mf\_liverA, Unigene13950\_Mf\_liverA, Unigene30584\_Mf\_liverA, CL6038.Contig2\_Mf\_liverA, Unigene32789\_Mf\_liverA, Unigene36328\_Mf\_liverA, Unigene38280\_Mf\_liverA, Unigene36898\_Mf\_liverA, Unigene32335\_Mf\_liverA, Unigene37389\_Mf\_liverA, Unigene34789\_Mf\_liverA, Unigene14809\_Mf\_liverA, CL5978.Contig3\_Mf\_liverA, Unigene28662\_Mf\_liverA, CL4919.Contig1\_Mf\_liverA, CL3911.Contig2\_Mf\_liverA, Unigene27082\_Mf\_liverA, NM\_009776, Unigene34394\_Mf\_liverA, Unigene14609\_Mf\_liverA, CL5007.Contig1\_Mf\_liverA, NM\_031165, CL497.Contig2\_Mf\_liverA, CL1493.Contig2\_Mf\_liverA, Unigene14810\_Mf\_liverA, CL2697.Contig4\_Mf\_liverA, CL1493.Contig1\_Mf\_liverA, CL6039.Contig1\_Mf\_liverA, CL2855.Contig1\_Mf\_liverA, Unigene37819\_Mf\_liverA, NM\_027959 |
| dendrite | Unigene36414\_Mf\_liverA, Unigene18500\_Mf\_liverA, Unigene27547\_Mf\_liverA, Unigene32570\_Mf\_liverA, Unigene13363\_Mf\_liverA, Unigene36417\_Mf\_liverA, Unigene34789\_Mf\_liverA, CL425.Contig1\_Mf\_liverA, Unigene24883\_Mf\_liverA, Unigene13593\_Mf\_liverA, NM\_178405, Unigene16529\_Mf\_liverA, Unigene33330\_Mf\_liverA, Unigene26611\_Mf\_liverA, Unigene14461\_Mf\_liverA, Unigene16184\_Mf\_liverA, Unigene28564\_Mf\_liverA, Unigene36420\_Mf\_liverA, Unigene36418\_Mf\_liverA |
| membrane-bounded vesicle | Unigene15055\_Mf\_liverA, CL5807.Contig1\_Mf\_liverA, Unigene33080\_Mf\_liverA, Unigene13363\_Mf\_liverA, CL2855.Contig2\_Mf\_liverA, CL4263.Contig1\_Mf\_liverA, NM\_021278, Unigene36190\_Mf\_liverA, NM\_019817, Unigene5750\_Mf\_liverA, CL838.Contig3\_Mf\_liverA, NM\_011082, Unigene19687\_Mf\_liverA, Unigene5632\_Mf\_liverA, Unigene33746\_Mf\_liverA, Unigene28143\_Mf\_liverA, Unigene36762\_Mf\_liverA, Unigene15470\_Mf\_liverA, Unigene600\_Mf\_liverA, Unigene36189\_Mf\_liverA, Unigene32093\_Mf\_liverA, Unigene35958\_Mf\_liverA, Unigene13950\_Mf\_liverA, Unigene30584\_Mf\_liverA, CL6038.Contig2\_Mf\_liverA, Unigene32789\_Mf\_liverA, Unigene36328\_Mf\_liverA, Unigene36898\_Mf\_liverA, Unigene32335\_Mf\_liverA, Unigene37389\_Mf\_liverA, Unigene34789\_Mf\_liverA, Unigene14809\_Mf\_liverA, Unigene28662\_Mf\_liverA, CL4919.Contig1\_Mf\_liverA, Unigene27082\_Mf\_liverA, NM\_009776, Unigene34394\_Mf\_liverA, CL3911.Contig2\_Mf\_liverA, Unigene14609\_Mf\_liverA, CL5007.Contig1\_Mf\_liverA, NM\_031165, CL497.Contig2\_Mf\_liverA, Unigene14810\_Mf\_liverA, CL2697.Contig4\_Mf\_liverA, CL2855.Contig1\_Mf\_liverA, Unigene37819\_Mf\_liverA, NM\_027959 |
| clathrin-coated endocytic vesicle membrane | CL2855.Contig1\_Mf\_liverA, CL2855.Contig2\_Mf\_liverA, Unigene32335\_Mf\_liverA |
| membrane attack complex | CL2339.Contig1\_Mf\_liverA, CL4456.Contig1\_Mf\_liverA |
| glycogen granule | Unigene36190\_Mf\_liverA, Unigene36189\_Mf\_liverA |
| pigment granule | Unigene34789\_Mf\_liverA, Unigene36762\_Mf\_liverA, NM\_031165, Unigene36190\_Mf\_liverA, NM\_027959, Unigene36189\_Mf\_liverA, Unigene600\_Mf\_liverA, CL4263.Contig1\_Mf\_liverA |
| coated vesicle membrane | Unigene34394\_Mf\_liverA, CL5807.Contig1\_Mf\_liverA, NM\_031165, CL2855.Contig2\_Mf\_liverA, Unigene32335\_Mf\_liverA, CL2855.Contig1\_Mf\_liverA, NM\_019817, Unigene5632\_Mf\_liverA |
| late endosome membrane | Unigene24471\_Mf\_liverA, Unigene5693\_Mf\_liverA, Unigene30878\_Mf\_liverA, CL3835.Contig2\_Mf\_liverA, CL3002.Contig1\_Mf\_liverA |
| clathrin-coated endocytic vesicle | CL2855.Contig1\_Mf\_liverA, CL2855.Contig2\_Mf\_liverA, Unigene32335\_Mf\_liverA |
| clathrin coated vesicle membrane | Unigene34394\_Mf\_liverA, CL2855.Contig1\_Mf\_liverA, CL5807.Contig1\_Mf\_liverA, NM\_031165, CL2855.Contig2\_Mf\_liverA, Unigene32335\_Mf\_liverA |
| vesicular fraction | NM\_007822, NM\_001100182, NM\_001081372, NM\_007811, NM\_206537, NM\_001081274, NM\_011978, NM\_001100181, NM\_010002, NM\_013821, NM\_010001, NM\_134127, NM\_178405, NM\_145474 |
| cytoplasmic vesicle part | CL6038.Contig2\_Mf\_liverA, CL5807.Contig1\_Mf\_liverA, Unigene36328\_Mf\_liverA, Unigene32789\_Mf\_liverA, Unigene36898\_Mf\_liverA, CL2855.Contig2\_Mf\_liverA, Unigene32335\_Mf\_liverA, Unigene14809\_Mf\_liverA, NM\_019817, Unigene28662\_Mf\_liverA, Unigene5632\_Mf\_liverA, Unigene34394\_Mf\_liverA, Unigene27082\_Mf\_liverA, Unigene28143\_Mf\_liverA, NM\_031165, CL2697.Contig4\_Mf\_liverA, CL2855.Contig1\_Mf\_liverA |
| axon | Unigene33330\_Mf\_liverA, Unigene34032\_Mf\_liverA, CL4583.Contig2\_Mf\_liverA, Unigene36414\_Mf\_liverA, Unigene18500\_Mf\_liverA, NM\_001004357, Unigene36898\_Mf\_liverA, Unigene13363\_Mf\_liverA, Unigene36417\_Mf\_liverA, CL3166.Contig4\_Mf\_liverA, Unigene28564\_Mf\_liverA, Unigene36420\_Mf\_liverA, Unigene24883\_Mf\_liverA, Unigene36418\_Mf\_liverA, Unigene16529\_Mf\_liverA |
| collagen | Unigene33441\_Mf\_liverA, Unigene32110\_Mf\_liverA, Unigene34375\_Mf\_liverA, Unigene23328\_Mf\_liverA, Unigene34124\_Mf\_liverA |
| pore complex | CL2339.Contig1\_Mf\_liverA, CL3104.Contig1\_Mf\_liverA, Unigene15433\_Mf\_liverA, Unigene21816\_Mf\_liverA, CL4456.Contig1\_Mf\_liverA |
| dendritic shaft | Unigene18500\_Mf\_liverA, Unigene24883\_Mf\_liverA, Unigene13593\_Mf\_liverA |
| integral to lumenal side of endoplasmic reticulum membrane | CL2855.Contig1\_Mf\_liverA, CL2855.Contig2\_Mf\_liverA |
| extracellular matrix part | NM\_008483, Unigene33441\_Mf\_liverA, Unigene32110\_Mf\_liverA, Unigene29628\_Mf\_liverA, Unigene32908\_Mf\_liverA, Unigene34375\_Mf\_liverA, Unigene32434\_Mf\_liverA, Unigene23328\_Mf\_liverA, Unigene34124\_Mf\_liverA, Unigene31492\_Mf\_liverA |
| vesicle lumen | CL6038.Contig2\_Mf\_liverA, Unigene14809\_Mf\_liverA, Unigene5750\_Mf\_liverA, Unigene28662\_Mf\_liverA |
| trans-Golgi network membrane | CL2855.Contig1\_Mf\_liverA, CL2855.Contig2\_Mf\_liverA |
| receptor complex | Unigene37232\_Mf\_liverA, NM\_007396, Unigene29426\_Mf\_liverA, Unigene843\_Mf\_liverA, Unigene27922\_Mf\_liverA, CL2855.Contig2\_Mf\_liverA, Unigene6959\_Mf\_liverA, CL2855.Contig1\_Mf\_liverA, Unigene31333\_Mf\_liverA |
| mitochondrion | Unigene35476\_Mf\_liverA, CL3750.Contig1\_Mf\_liverA, CL114.Contig2\_Mf\_liverA, CL5828.Contig2\_Mf\_liverA, Unigene743\_Mf\_liverA, Unigene31199\_Mf\_liverA, Unigene36691\_Mf\_liverA, CL3669.Contig2\_Mf\_liverA, CL3750.Contig2\_Mf\_liverA, CL2389.Contig1\_Mf\_liverA, CL3599.Contig1\_Mf\_liverA, CL425.Contig1\_Mf\_liverA, Unigene31639\_Mf\_liverA, CL5052.Contig1\_Mf\_liverA, Unigene36190\_Mf\_liverA, CL2797.Contig2\_Mf\_liverA, NM\_019879, Unigene30832\_Mf\_liverA, Unigene14916\_Mf\_liverA, CL1125.Contig1\_Mf\_liverA, Unigene27071\_Mf\_liverA, Unigene14286\_Mf\_liverA, CL114.Contig1\_Mf\_liverA, Unigene24225\_Mf\_liverA, CL5275.Contig2\_Mf\_liverA, Unigene5693\_Mf\_liverA, Unigene36189\_Mf\_liverA, Unigene40289\_Mf\_liverA, Unigene29558\_Mf\_liverA, Unigene4510\_Mf\_liverA, Unigene29876\_Mf\_liverA, Unigene28459\_Mf\_liverA, Unigene27547\_Mf\_liverA, Unigene32570\_Mf\_liverA, Unigene37120\_Mf\_liverA, Unigene31198\_Mf\_liverA, CL4984.Contig1\_Mf\_liverA, CL5316.Contig1\_Mf\_liverA, Unigene10313\_Mf\_liverA, Unigene35664\_Mf\_liverA, CL1688.Contig3\_Mf\_liverA, Unigene34406\_Mf\_liverA, CL1810.Contig1\_Mf\_liverA, CL3104.Contig1\_Mf\_liverA, CL887.Contig2\_Mf\_liverA, Unigene2\_Mf\_liverA, Unigene13658\_Mf\_liverA, Unigene27082\_Mf\_liverA, CL532.Contig1\_Mf\_liverA, Unigene35037\_Mf\_liverA, CL3933.Contig1\_Mf\_liverA, Unigene5761\_Mf\_liverA, CL3166.Contig4\_Mf\_liverA, Unigene5607\_Mf\_liverA, Unigene18848\_Mf\_liverA, Unigene38104\_Mf\_liverA, NM\_013821, Unigene36626\_Mf\_liverA |
| neuron projection terminus | Unigene36898\_Mf\_liverA, Unigene13363\_Mf\_liverA, Unigene16529\_Mf\_liverA |
| platelet alpha granule | CL6038.Contig2\_Mf\_liverA, NM\_009776, Unigene32789\_Mf\_liverA, Unigene28662\_Mf\_liverA, NM\_021278 |
| excitatory synapse | Unigene13593\_Mf\_liverA, Unigene16529\_Mf\_liverA |
| cytoplasmic vesicle membrane | Unigene28143\_Mf\_liverA, Unigene27082\_Mf\_liverA, Unigene34394\_Mf\_liverA, CL5807.Contig1\_Mf\_liverA, Unigene36328\_Mf\_liverA, NM\_031165, Unigene32789\_Mf\_liverA, Unigene36898\_Mf\_liverA, CL2855.Contig2\_Mf\_liverA, Unigene32335\_Mf\_liverA, CL2697.Contig4\_Mf\_liverA, CL2855.Contig1\_Mf\_liverA, NM\_019817, Unigene5632\_Mf\_liverA |
| SCF ubiquitin ligase complex | Unigene33054\_Mf\_liverA, Unigene13683\_Mf\_liverA |
| integral to Golgi membrane | CL787.Contig1\_Mf\_liverA, Unigene31051\_Mf\_liverA, Unigene32920\_Mf\_liverA |
| neuron projection | Unigene13363\_Mf\_liverA, NM\_011732, Unigene36417\_Mf\_liverA, CL425.Contig1\_Mf\_liverA, Unigene24883\_Mf\_liverA, NM\_178405, Unigene33746\_Mf\_liverA, Unigene36762\_Mf\_liverA, NM\_011305, Unigene16184\_Mf\_liverA, Unigene28564\_Mf\_liverA, Unigene36418\_Mf\_liverA, Unigene13950\_Mf\_liverA, Unigene34032\_Mf\_liverA, CL4583.Contig2\_Mf\_liverA, NM\_021423, Unigene27547\_Mf\_liverA, Unigene18500\_Mf\_liverA, Unigene36414\_Mf\_liverA, Unigene32570\_Mf\_liverA, NM\_010141, Unigene36898\_Mf\_liverA, CL1810.Contig1\_Mf\_liverA, Unigene34789\_Mf\_liverA, Unigene16529\_Mf\_liverA, Unigene13593\_Mf\_liverA, Unigene33330\_Mf\_liverA, Unigene26611\_Mf\_liverA, Unigene14609\_Mf\_liverA, Unigene5712\_Mf\_liverA, NM\_001004357, Unigene14461\_Mf\_liverA, CL2697.Contig4\_Mf\_liverA, CL3166.Contig4\_Mf\_liverA, Unigene36420\_Mf\_liverA |
| cell part | Unigene21684\_Mf\_liverA, NM\_176843, CL4302.Contig1\_Mf\_liverA, Unigene27730\_Mf\_liverA, Unigene29628\_Mf\_liverA, NM\_025593, NM\_010378, Unigene15553\_Mf\_liverA, CL854.Contig1\_Mf\_liverA, Unigene39886\_Mf\_liverA, Unigene15064\_Mf\_liverA, Unigene34124\_Mf\_liverA, NR\_004446, Unigene36765\_Mf\_liverA, CL695.Contig1\_Mf\_liverA, Unigene34847\_Mf\_liverA, Unigene29082\_Mf\_liverA, Unigene2\_Mf\_liverA, CL4919.Contig1\_Mf\_liverA, Unigene31623\_Mf\_liverA, CL2266.Contig1\_Mf\_liverA, NM\_010233, Unigene37084\_Mf\_liverA, Unigene20874\_Mf\_liverA, Unigene34394\_Mf\_liverA, Unigene25594\_Mf\_liverA, Unigene36430\_Mf\_liverA, CL3154.Contig1\_Mf\_liverA, CL5807.Contig1\_Mf\_liverA, Unigene743\_Mf\_liverA, Unigene37454\_Mf\_liverA, Unigene36417\_Mf\_liverA, CL4263.Contig1\_Mf\_liverA, Unigene13097\_Mf\_liverA, CL4293.Contig1\_Mf\_liverA, Unigene14907\_Mf\_liverA, CL4456.Contig1\_Mf\_liverA, Unigene25976\_Mf\_liverA, NM\_009338, Unigene36698\_Mf\_liverA, NM\_145942, Unigene139\_Mf\_liverA, Unigene15572\_Mf\_liverA, Unigene37018\_Mf\_liverA, NM\_022324, Unigene10756\_Mf\_liverA, Unigene15290\_Mf\_liverA, Unigene32789\_Mf\_liverA, Unigene36898\_Mf\_liverA, Unigene35664\_Mf\_liverA, NM\_027660, Unigene37389\_Mf\_liverA, Unigene41957\_Mf\_liverA, Unigene30839\_Mf\_liverA, Unigene32852\_Mf\_liverA, Unigene24613\_Mf\_liverA, Unigene26611\_Mf\_liverA, CL532.Contig1\_Mf\_liverA, Unigene15477\_Mf\_liverA, Unigene1280\_Mf\_liverA, Unigene30288\_Mf\_liverA, CL275.Contig5\_Mf\_liverA, Unigene30697\_Mf\_liverA, NM\_027959, Unigene34972\_Mf\_liverA, Unigene34140\_Mf\_liverA, Unigene4363\_Mf\_liverA, Unigene836\_Mf\_liverA, Unigene42606\_Mf\_liverA, CL425.Contig1\_Mf\_liverA, CL3150.Contig1\_Mf\_liverA, CL2797.Contig2\_Mf\_liverA, Unigene28143\_Mf\_liverA, Unigene37243\_Mf\_liverA, Unigene15077\_Mf\_liverA, Unigene5693\_Mf\_liverA, CL3382.Contig1\_Mf\_liverA, Unigene29876\_Mf\_liverA, Unigene28687\_Mf\_liverA, Unigene32803\_Mf\_liverA, CL1988.Contig3\_Mf\_liverA, NM\_172679, CL1810.Contig1\_Mf\_liverA, CL4033.Contig1\_Mf\_liverA, Unigene34983\_Mf\_liverA, CL3519.Contig1\_Mf\_liverA, Unigene33330\_Mf\_liverA, NM\_011221, CL1493.Contig1\_Mf\_liverA, Unigene30004\_Mf\_liverA, Unigene35476\_Mf\_liverA, Unigene24471\_Mf\_liverA, Unigene31571\_Mf\_liverA, Unigene35168\_Mf\_liverA, Unigene15888\_Mf\_liverA, CL4220.Contig1\_Mf\_liverA, Unigene37262\_Mf\_liverA, CL4338.Contig1\_Mf\_liverA, Unigene12908\_Mf\_liverA, CL5052.Contig1\_Mf\_liverA, Unigene26251\_Mf\_liverA, NM\_010469, Unigene5750\_Mf\_liverA, NR\_033215, NM\_007822, CL5293.Contig1\_Mf\_liverA, Unigene25595\_Mf\_liverA, Unigene14263\_Mf\_liverA, Unigene14286\_Mf\_liverA, CL4816.Contig3\_Mf\_liverA, CL114.Contig1\_Mf\_liverA, Unigene39011\_Mf\_liverA, Unigene8218\_Mf\_liverA, NM\_008183, Unigene13950\_Mf\_liverA, Unigene28459\_Mf\_liverA, Unigene37245\_Mf\_liverA, NM\_011978, Unigene37104\_Mf\_liverA, NM\_027406, Unigene36176\_Mf\_liverA, NM\_026313, Unigene28609\_Mf\_liverA, Unigene29889\_Mf\_liverA, Unigene2939\_Mf\_liverA, Unigene35884\_Mf\_liverA, CL5640.Contig1\_Mf\_liverA, NM\_028291, CL3835.Contig2\_Mf\_liverA, Unigene28899\_Mf\_liverA, CL4550.Contig2\_Mf\_liverA, Unigene24883\_Mf\_liverA, Unigene14603\_Mf\_liverA, Unigene46870\_Mf\_liverA, NM\_019651, Unigene880\_Mf\_liverA, Unigene36849\_Mf\_liverA, Unigene8473\_Mf\_liverA, Unigene25055\_Mf\_liverA, CL2339.Contig1\_Mf\_liverA, Unigene27071\_Mf\_liverA, NM\_011072, Unigene14816\_Mf\_liverA, Unigene28564\_Mf\_liverA, NM\_173753, CL6038.Contig2\_Mf\_liverA, Unigene37904\_Mf\_liverA, CL3483.Contig1\_Mf\_liverA, Unigene34789\_Mf\_liverA, CL4141.Contig1\_Mf\_liverA, Unigene24221\_Mf\_liverA, NM\_145419, Unigene34184\_Mf\_liverA, CL5978.Contig3\_Mf\_liverA, Unigene13658\_Mf\_liverA, Unigene13593\_Mf\_liverA, NM\_134127, Unigene32546\_Mf\_liverA, Unigene4630\_Mf\_liverA, Unigene11\_Mf\_liverA, CL2622.Contig2\_Mf\_liverA, Unigene14609\_Mf\_liverA, Unigene12997\_Mf\_liverA, Unigene5994\_Mf\_liverA, CL1493.Contig2\_Mf\_liverA, CL2697.Contig4\_Mf\_liverA, NM\_008261, NM\_024434, CL2574.Contig1\_Mf\_liverA, Unigene5941\_Mf\_liverA, Unigene38104\_Mf\_liverA, Unigene23328\_Mf\_liverA, Unigene36626\_Mf\_liverA, CL3897.Contig5\_Mf\_liverA, Unigene12967\_Mf\_liverA, Unigene36691\_Mf\_liverA, Unigene31988\_Mf\_liverA, CL3599.Contig1\_Mf\_liverA, Unigene151\_Mf\_liverA, CL848.Contig2\_Mf\_liverA, Unigene31639\_Mf\_liverA, Unigene10774\_Mf\_liverA, Unigene13143\_Mf\_liverA, CL1263.Contig1\_Mf\_liverA, CL258.Contig1\_Mf\_liverA, NM\_007811, Unigene24323\_Mf\_liverA, NM\_008538, CL2142.Contig2\_Mf\_liverA, Unigene30428\_Mf\_liverA, NM\_011305, Unigene21741\_Mf\_liverA, CL153.Contig2\_Mf\_liverA, Unigene13498\_Mf\_liverA, CL529.Contig2\_Mf\_liverA, Unigene40069\_Mf\_liverA, Unigene4510\_Mf\_liverA, Unigene37148\_Mf\_liverA, Unigene34406\_Mf\_liverA, Unigene7195\_Mf\_liverA, Unigene35506\_Mf\_liverA, CL2919.Contig1\_Mf\_liverA, Unigene1205\_Mf\_liverA, CL6039.Contig1\_Mf\_liverA, Unigene33420\_Mf\_liverA, Unigene32412\_Mf\_liverA, Unigene17569\_Mf\_liverA, Unigene30426\_Mf\_liverA, Unigene17042\_Mf\_liverA, Unigene34811\_Mf\_liverA, Unigene3789\_Mf\_liverA, Unigene33746\_Mf\_liverA, CL1125.Contig1\_Mf\_liverA, Unigene37526\_Mf\_liverA, Unigene1000\_Mf\_liverA, Unigene28853\_Mf\_liverA, Unigene15681\_Mf\_liverA, Unigene673\_Mf\_liverA, NM\_021423, Unigene15552\_Mf\_liverA, Unigene38280\_Mf\_liverA, CL5309.Contig1\_Mf\_liverA, CL3002.Contig1\_Mf\_liverA, Unigene31483\_Mf\_liverA, Unigene31267\_Mf\_liverA, Unigene36949\_Mf\_liverA, Unigene5488\_Mf\_liverA, CL2597.Contig1\_Mf\_liverA, Unigene33523\_Mf\_liverA, Unigene35406\_Mf\_liverA, Unigene14810\_Mf\_liverA, NM\_001100181, Unigene5607\_Mf\_liverA, CL4770.Contig1\_Mf\_liverA, CL114.Contig2\_Mf\_liverA, Unigene14449\_Mf\_liverA, Unigene39520\_Mf\_liverA, NM\_009883, Unigene39437\_Mf\_liverA, Unigene22423\_Mf\_liverA, Unigene1327\_Mf\_liverA, CL5978.Contig2\_Mf\_liverA, Unigene30547\_Mf\_liverA, CL442.Contig5\_Mf\_liverA, Unigene36418\_Mf\_liverA, Unigene25052\_Mf\_liverA, NM\_022997, Unigene550\_Mf\_liverA, CL55.Contig1\_Mf\_liverA, Unigene37120\_Mf\_liverA, Unigene31198\_Mf\_liverA, CL2355.Contig1\_Mf\_liverA, Unigene4863\_Mf\_liverA, Unigene37153\_Mf\_liverA, Unigene14809\_Mf\_liverA, Unigene5489\_Mf\_liverA, Unigene138\_Mf\_liverA, NM\_009011, CL3911.Contig2\_Mf\_liverA, Unigene37366\_Mf\_liverA, Unigene25597\_Mf\_liverA, Unigene4681\_Mf\_liverA, NM\_008851, CL2884.Contig2\_Mf\_liverA, NM\_007396, NM\_009450, NM\_144940, CL3669.Contig2\_Mf\_liverA, Unigene29011\_Mf\_liverA, Unigene37334\_Mf\_liverA, Unigene36172\_Mf\_liverA, Unigene36851\_Mf\_liverA, NM\_080638, NM\_011082, Unigene19687\_Mf\_liverA, Unigene26053\_Mf\_liverA, CL4105.Contig1\_Mf\_liverA, Unigene7336\_Mf\_liverA, CL238.Contig1\_Mf\_liverA, Unigene35507\_Mf\_liverA, Unigene6465\_Mf\_liverA, CL3617.Contig1\_Mf\_liverA, Unigene785\_Mf\_liverA, Unigene32920\_Mf\_liverA, Unigene27294\_Mf\_liverA, NM\_199027, Unigene1137\_Mf\_liverA, Unigene36434\_Mf\_liverA, Unigene34341\_Mf\_liverA, Unigene30142\_Mf\_liverA, Unigene30153\_Mf\_liverA, NM\_011921, Unigene7897\_Mf\_liverA, NM\_145474, Unigene33054\_Mf\_liverA, NM\_009776, Unigene30003\_Mf\_liverA, NM\_031165, CL497.Contig2\_Mf\_liverA, CL6.Contig1\_Mf\_liverA, Unigene34143\_Mf\_liverA, Unigene18848\_Mf\_liverA, Unigene30002\_Mf\_liverA, NM\_016697, Unigene802\_Mf\_liverA, Unigene15055\_Mf\_liverA, Unigene9150\_Mf\_liverA, CL4816.Contig1\_Mf\_liverA, Unigene5639\_Mf\_liverA, Unigene32594\_Mf\_liverA, Unigene13363\_Mf\_liverA, Unigene36175\_Mf\_liverA, NM\_021278, CL2855.Contig2\_Mf\_liverA, Unigene1479\_Mf\_liverA, NM\_010001, CL4249.Contig1\_Mf\_liverA, Unigene44882\_Mf\_liverA, Unigene7264\_Mf\_liverA, Unigene31625\_Mf\_liverA, Unigene5057\_Mf\_liverA, Unigene34032\_Mf\_liverA, Unigene27547\_Mf\_liverA, Unigene32908\_Mf\_liverA, Unigene24090\_Mf\_liverA, Unigene12909\_Mf\_liverA, CL5316.Contig1\_Mf\_liverA, CL1688.Contig3\_Mf\_liverA, Unigene4723\_Mf\_liverA, Unigene32332\_Mf\_liverA, NR\_003630, CL3930.Contig1\_Mf\_liverA, Unigene35169\_Mf\_liverA, Unigene1288\_Mf\_liverA, CL3166.Contig4\_Mf\_liverA, Unigene5169\_Mf\_liverA, Unigene32059\_Mf\_liverA, CL186.Contig3\_Mf\_liverA, Unigene30832\_Mf\_liverA, Unigene4697\_Mf\_liverA, CL4736.Contig1\_Mf\_liverA, Unigene14916\_Mf\_liverA, Unigene30217\_Mf\_liverA, CL4600.Contig1\_Mf\_liverA, Unigene2050\_Mf\_liverA, Unigene16184\_Mf\_liverA, NM\_029582, Unigene29134\_Mf\_liverA, Unigene35958\_Mf\_liverA, NM\_016689, Unigene4938\_Mf\_liverA, Unigene5815\_Mf\_liverA, Unigene34375\_Mf\_liverA, Unigene37224\_Mf\_liverA, Unigene28662\_Mf\_liverA, CL2439.Contig1\_Mf\_liverA, Unigene29426\_Mf\_liverA, Unigene6464\_Mf\_liverA, CL3933.Contig1\_Mf\_liverA, Unigene21026\_Mf\_liverA, Unigene20371\_Mf\_liverA, Unigene13683\_Mf\_liverA, CL1325.Contig1\_Mf\_liverA, Unigene13009\_Mf\_liverA, Unigene9698\_Mf\_liverA, Unigene36420\_Mf\_liverA, Unigene542\_Mf\_liverA, Unigene24758\_Mf\_liverA, CL5828.Contig2\_Mf\_liverA, Unigene33080\_Mf\_liverA, Unigene15433\_Mf\_liverA, NM\_019817, NM\_019879, Unigene15470\_Mf\_liverA, Unigene32434\_Mf\_liverA, Unigene34962\_Mf\_liverA, Unigene24225\_Mf\_liverA, CL1588.Contig2\_Mf\_liverA, Unigene37344\_Mf\_liverA, NM\_011207, CL5275.Contig2\_Mf\_liverA, NM\_145218, Unigene36189\_Mf\_liverA, Unigene29558\_Mf\_liverA, Unigene32133\_Mf\_liverA, Unigene281\_Mf\_liverA, Unigene20372\_Mf\_liverA, Unigene10313\_Mf\_liverA, NM\_177652, Unigene30548\_Mf\_liverA, Unigene13018\_Mf\_liverA, CL3104.Contig1\_Mf\_liverA, Unigene36593\_Mf\_liverA, NM\_013842, NM\_010002, Unigene16529\_Mf\_liverA, Unigene37232\_Mf\_liverA, CL5007.Contig1\_Mf\_liverA, Unigene14461\_Mf\_liverA, CL5698.Contig1\_Mf\_liverA, Unigene31392\_Mf\_liverA, NR\_024097, CL591.Contig1\_Mf\_liverA, Unigene35618\_Mf\_liverA, NM\_011256, Unigene38015\_Mf\_liverA, NM\_173038, NM\_206537, Unigene31199\_Mf\_liverA, Unigene6959\_Mf\_liverA, Unigene28244\_Mf\_liverA, CL523.Contig1\_Mf\_liverA, NM\_008277, CL482.Contig1\_Mf\_liverA, Unigene35382\_Mf\_liverA, Unigene36762\_Mf\_liverA, Unigene600\_Mf\_liverA, Unigene18795\_Mf\_liverA, Unigene37698\_Mf\_liverA, Unigene30878\_Mf\_liverA, Unigene40289\_Mf\_liverA, NM\_134012, Unigene18500\_Mf\_liverA, Unigene32570\_Mf\_liverA, CL4910.Contig1\_Mf\_liverA, CL1256.Contig1\_Mf\_liverA, Unigene5333\_Mf\_liverA, Unigene15295\_Mf\_liverA, Unigene12999\_Mf\_liverA, CL1119.Contig1\_Mf\_liverA, Unigene31333\_Mf\_liverA, Unigene30369\_Mf\_liverA, Unigene33598\_Mf\_liverA, Unigene25596\_Mf\_liverA, Unigene843\_Mf\_liverA, Unigene37109\_Mf\_liverA, Unigene28852\_Mf\_liverA, NM\_001004357, CL3778.Contig2\_Mf\_liverA, CL4076.Contig1\_Mf\_liverA, CL787.Contig1\_Mf\_liverA, Unigene29490\_Mf\_liverA, NM\_001162917, CL2389.Contig1\_Mf\_liverA, CL3750.Contig2\_Mf\_liverA, CL335.Contig1\_Mf\_liverA, Unigene39141\_Mf\_liverA, CL784.Contig3\_Mf\_liverA, CL838.Contig3\_Mf\_liverA, Unigene5632\_Mf\_liverA, Unigene28520\_Mf\_liverA, CL4550.Contig3\_Mf\_liverA, Unigene21816\_Mf\_liverA, Unigene27419\_Mf\_liverA, Unigene21742\_Mf\_liverA, Unigene31427\_Mf\_liverA, CL4095.Contig4\_Mf\_liverA, CL4283.Contig1\_Mf\_liverA, Unigene30584\_Mf\_liverA, Unigene17579\_Mf\_liverA, NM\_001081372, CL913.Contig1\_Mf\_liverA, NM\_021525, Unigene27082\_Mf\_liverA, Unigene37178\_Mf\_liverA, Unigene32058\_Mf\_liverA, Unigene5712\_Mf\_liverA, Unigene36772\_Mf\_liverA, CL442.Contig2\_Mf\_liverA, NM\_010380, CL2855.Contig1\_Mf\_liverA, CL4411.Contig4\_Mf\_liverA, NM\_013821, Unigene40796\_Mf\_liverA, NM\_001100182, NM\_025706, Unigene31051\_Mf\_liverA, Unigene392\_Mf\_liverA, NM\_011732, CL4228.Contig1\_Mf\_liverA, NM\_009178, Unigene49971\_Mf\_liverA, Unigene38307\_Mf\_liverA, Unigene36190\_Mf\_liverA, Unigene27406\_Mf\_liverA, Unigene34754\_Mf\_liverA, Unigene30154\_Mf\_liverA, CL4162.Contig1\_Mf\_liverA, Unigene27922\_Mf\_liverA, Unigene15476\_Mf\_liverA, CL1100.Contig1\_Mf\_liverA, Unigene32093\_Mf\_liverA, CL4583.Contig2\_Mf\_liverA, CL485.Contig1\_Mf\_liverA, Unigene36414\_Mf\_liverA, CL1988.Contig1\_Mf\_liverA, Unigene5175\_Mf\_liverA, Unigene13894\_Mf\_liverA, Unigene36328\_Mf\_liverA, NM\_010141, CL4745.Contig1\_Mf\_liverA, Unigene36315\_Mf\_liverA, CL2327.Contig1\_Mf\_liverA, CL887.Contig2\_Mf\_liverA, Unigene33736\_Mf\_liverA, Unigene35037\_Mf\_liverA, CL1656.Contig1\_Mf\_liverA, CL3684.Contig2\_Mf\_liverA, Unigene1212\_Mf\_liverA, Unigene32233\_Mf\_liverA, CL838.Contig6\_Mf\_liverA, Unigene10915\_Mf\_liverA, Unigene32143\_Mf\_liverA, Unigene24536\_Mf\_liverA, CL3750.Contig1\_Mf\_liverA, Unigene13196\_Mf\_liverA, Unigene601\_Mf\_liverA, CL1076.Contig1\_Mf\_liverA, Unigene18796\_Mf\_liverA, NM\_009448, CL5307.Contig1\_Mf\_liverA, Unigene31670\_Mf\_liverA, NM\_178405, NM\_008483, NM\_172203, Unigene120\_Mf\_liverA, Unigene15026\_Mf\_liverA, CL4007.Contig1\_Mf\_liverA, NM\_001081116, NM\_017379, Unigene28489\_Mf\_liverA, CL4984.Contig1\_Mf\_liverA, NM\_001081274, Unigene24537\_Mf\_liverA, Unigene32335\_Mf\_liverA, Unigene7224\_Mf\_liverA, NM\_027604, Unigene34444\_Mf\_liverA, Unigene7399\_Mf\_liverA, Unigene35678\_Mf\_liverA, Unigene33993\_Mf\_liverA, Unigene8560\_Mf\_liverA, Unigene44057\_Mf\_liverA, Unigene5761\_Mf\_liverA, Unigene12519\_Mf\_liverA, Unigene37819\_Mf\_liverA, Unigene5886\_Mf\_liverA, CL336.Contig3\_Mf\_liverA, CL1198.Contig1\_Mf\_liverA |
| cell | Unigene21684\_Mf\_liverA, NM\_176843, CL4302.Contig1\_Mf\_liverA, Unigene27730\_Mf\_liverA, Unigene29628\_Mf\_liverA, NM\_025593, NM\_010378, Unigene15553\_Mf\_liverA, CL854.Contig1\_Mf\_liverA, Unigene39886\_Mf\_liverA, Unigene15064\_Mf\_liverA, Unigene34124\_Mf\_liverA, NR\_004446, Unigene36765\_Mf\_liverA, CL695.Contig1\_Mf\_liverA, Unigene34847\_Mf\_liverA, Unigene29082\_Mf\_liverA, Unigene2\_Mf\_liverA, CL4919.Contig1\_Mf\_liverA, Unigene31623\_Mf\_liverA, CL2266.Contig1\_Mf\_liverA, NM\_010233, Unigene37084\_Mf\_liverA, Unigene20874\_Mf\_liverA, Unigene34394\_Mf\_liverA, Unigene25594\_Mf\_liverA, Unigene36430\_Mf\_liverA, CL3154.Contig1\_Mf\_liverA, CL5807.Contig1\_Mf\_liverA, Unigene743\_Mf\_liverA, Unigene37454\_Mf\_liverA, Unigene36417\_Mf\_liverA, CL4263.Contig1\_Mf\_liverA, Unigene13097\_Mf\_liverA, CL4293.Contig1\_Mf\_liverA, Unigene14907\_Mf\_liverA, CL4456.Contig1\_Mf\_liverA, Unigene25976\_Mf\_liverA, NM\_009338, Unigene36698\_Mf\_liverA, NM\_145942, Unigene139\_Mf\_liverA, Unigene15572\_Mf\_liverA, Unigene37018\_Mf\_liverA, NM\_022324, Unigene10756\_Mf\_liverA, Unigene15290\_Mf\_liverA, Unigene32789\_Mf\_liverA, Unigene36898\_Mf\_liverA, Unigene35664\_Mf\_liverA, NM\_027660, Unigene37389\_Mf\_liverA, Unigene41957\_Mf\_liverA, Unigene30839\_Mf\_liverA, Unigene32852\_Mf\_liverA, Unigene24613\_Mf\_liverA, Unigene26611\_Mf\_liverA, CL532.Contig1\_Mf\_liverA, Unigene15477\_Mf\_liverA, Unigene1280\_Mf\_liverA, Unigene30288\_Mf\_liverA, CL275.Contig5\_Mf\_liverA, Unigene30697\_Mf\_liverA, NM\_027959, Unigene34972\_Mf\_liverA, Unigene34140\_Mf\_liverA, Unigene4363\_Mf\_liverA, Unigene836\_Mf\_liverA, Unigene42606\_Mf\_liverA, CL425.Contig1\_Mf\_liverA, CL3150.Contig1\_Mf\_liverA, CL2797.Contig2\_Mf\_liverA, Unigene28143\_Mf\_liverA, Unigene37243\_Mf\_liverA, Unigene15077\_Mf\_liverA, Unigene5693\_Mf\_liverA, CL3382.Contig1\_Mf\_liverA, Unigene29876\_Mf\_liverA, Unigene28687\_Mf\_liverA, Unigene32803\_Mf\_liverA, CL1988.Contig3\_Mf\_liverA, NM\_172679, CL1810.Contig1\_Mf\_liverA, CL4033.Contig1\_Mf\_liverA, Unigene34983\_Mf\_liverA, CL3519.Contig1\_Mf\_liverA, Unigene33330\_Mf\_liverA, NM\_011221, CL1493.Contig1\_Mf\_liverA, Unigene30004\_Mf\_liverA, Unigene35476\_Mf\_liverA, Unigene24471\_Mf\_liverA, Unigene31571\_Mf\_liverA, Unigene35168\_Mf\_liverA, Unigene15888\_Mf\_liverA, CL4220.Contig1\_Mf\_liverA, Unigene37262\_Mf\_liverA, CL4338.Contig1\_Mf\_liverA, Unigene12908\_Mf\_liverA, CL5052.Contig1\_Mf\_liverA, Unigene26251\_Mf\_liverA, NM\_010469, Unigene5750\_Mf\_liverA, NR\_033215, NM\_007822, CL5293.Contig1\_Mf\_liverA, Unigene25595\_Mf\_liverA, Unigene14263\_Mf\_liverA, Unigene14286\_Mf\_liverA, CL4816.Contig3\_Mf\_liverA, CL114.Contig1\_Mf\_liverA, Unigene39011\_Mf\_liverA, Unigene8218\_Mf\_liverA, NM\_008183, Unigene13950\_Mf\_liverA, Unigene28459\_Mf\_liverA, Unigene37245\_Mf\_liverA, NM\_011978, Unigene37104\_Mf\_liverA, NM\_027406, Unigene36176\_Mf\_liverA, NM\_026313, Unigene28609\_Mf\_liverA, Unigene29889\_Mf\_liverA, Unigene2939\_Mf\_liverA, Unigene35884\_Mf\_liverA, CL5640.Contig1\_Mf\_liverA, NM\_028291, CL3835.Contig2\_Mf\_liverA, Unigene28899\_Mf\_liverA, CL4550.Contig2\_Mf\_liverA, Unigene24883\_Mf\_liverA, Unigene14603\_Mf\_liverA, Unigene46870\_Mf\_liverA, NM\_019651, Unigene880\_Mf\_liverA, Unigene36849\_Mf\_liverA, Unigene8473\_Mf\_liverA, Unigene25055\_Mf\_liverA, CL2339.Contig1\_Mf\_liverA, Unigene27071\_Mf\_liverA, NM\_011072, Unigene14816\_Mf\_liverA, Unigene28564\_Mf\_liverA, NM\_173753, CL6038.Contig2\_Mf\_liverA, Unigene37904\_Mf\_liverA, CL3483.Contig1\_Mf\_liverA, Unigene34789\_Mf\_liverA, CL4141.Contig1\_Mf\_liverA, Unigene24221\_Mf\_liverA, NM\_145419, Unigene34184\_Mf\_liverA, CL5978.Contig3\_Mf\_liverA, Unigene13658\_Mf\_liverA, Unigene13593\_Mf\_liverA, NM\_134127, Unigene32546\_Mf\_liverA, Unigene4630\_Mf\_liverA, Unigene11\_Mf\_liverA, CL2622.Contig2\_Mf\_liverA, Unigene14609\_Mf\_liverA, Unigene12997\_Mf\_liverA, Unigene5994\_Mf\_liverA, CL1493.Contig2\_Mf\_liverA, CL2697.Contig4\_Mf\_liverA, NM\_008261, NM\_024434, CL2574.Contig1\_Mf\_liverA, Unigene5941\_Mf\_liverA, Unigene38104\_Mf\_liverA, Unigene23328\_Mf\_liverA, Unigene36626\_Mf\_liverA, CL3897.Contig5\_Mf\_liverA, Unigene12967\_Mf\_liverA, Unigene36691\_Mf\_liverA, Unigene31988\_Mf\_liverA, CL3599.Contig1\_Mf\_liverA, Unigene151\_Mf\_liverA, CL848.Contig2\_Mf\_liverA, Unigene31639\_Mf\_liverA, Unigene10774\_Mf\_liverA, Unigene13143\_Mf\_liverA, CL1263.Contig1\_Mf\_liverA, CL258.Contig1\_Mf\_liverA, NM\_007811, Unigene24323\_Mf\_liverA, NM\_008538, CL2142.Contig2\_Mf\_liverA, Unigene30428\_Mf\_liverA, NM\_011305, Unigene21741\_Mf\_liverA, CL153.Contig2\_Mf\_liverA, Unigene13498\_Mf\_liverA, CL529.Contig2\_Mf\_liverA, Unigene40069\_Mf\_liverA, Unigene4510\_Mf\_liverA, Unigene37148\_Mf\_liverA, Unigene34406\_Mf\_liverA, Unigene7195\_Mf\_liverA, Unigene35506\_Mf\_liverA, CL2919.Contig1\_Mf\_liverA, Unigene1205\_Mf\_liverA, CL6039.Contig1\_Mf\_liverA, Unigene33420\_Mf\_liverA, Unigene32412\_Mf\_liverA, Unigene17569\_Mf\_liverA, Unigene30426\_Mf\_liverA, Unigene17042\_Mf\_liverA, Unigene34811\_Mf\_liverA, Unigene3789\_Mf\_liverA, Unigene33746\_Mf\_liverA, CL1125.Contig1\_Mf\_liverA, Unigene37526\_Mf\_liverA, Unigene1000\_Mf\_liverA, Unigene28853\_Mf\_liverA, Unigene15681\_Mf\_liverA, Unigene673\_Mf\_liverA, NM\_021423, Unigene15552\_Mf\_liverA, Unigene38280\_Mf\_liverA, CL5309.Contig1\_Mf\_liverA, CL3002.Contig1\_Mf\_liverA, Unigene31483\_Mf\_liverA, Unigene31267\_Mf\_liverA, Unigene36949\_Mf\_liverA, Unigene5488\_Mf\_liverA, CL2597.Contig1\_Mf\_liverA, Unigene33523\_Mf\_liverA, Unigene35406\_Mf\_liverA, Unigene14810\_Mf\_liverA, NM\_001100181, Unigene5607\_Mf\_liverA, CL4770.Contig1\_Mf\_liverA, CL114.Contig2\_Mf\_liverA, Unigene14449\_Mf\_liverA, Unigene39520\_Mf\_liverA, NM\_009883, Unigene39437\_Mf\_liverA, Unigene22423\_Mf\_liverA, Unigene1327\_Mf\_liverA, CL5978.Contig2\_Mf\_liverA, Unigene30547\_Mf\_liverA, CL442.Contig5\_Mf\_liverA, Unigene36418\_Mf\_liverA, Unigene25052\_Mf\_liverA, NM\_022997, Unigene550\_Mf\_liverA, CL55.Contig1\_Mf\_liverA, Unigene37120\_Mf\_liverA, Unigene31198\_Mf\_liverA, CL2355.Contig1\_Mf\_liverA, Unigene4863\_Mf\_liverA, Unigene37153\_Mf\_liverA, Unigene14809\_Mf\_liverA, Unigene5489\_Mf\_liverA, Unigene138\_Mf\_liverA, NM\_009011, CL3911.Contig2\_Mf\_liverA, Unigene37366\_Mf\_liverA, Unigene25597\_Mf\_liverA, Unigene4681\_Mf\_liverA, NM\_008851, CL2884.Contig2\_Mf\_liverA, NM\_007396, NM\_009450, NM\_144940, CL3669.Contig2\_Mf\_liverA, Unigene29011\_Mf\_liverA, Unigene37334\_Mf\_liverA, Unigene36172\_Mf\_liverA, Unigene36851\_Mf\_liverA, NM\_080638, NM\_011082, Unigene19687\_Mf\_liverA, Unigene26053\_Mf\_liverA, CL4105.Contig1\_Mf\_liverA, Unigene7336\_Mf\_liverA, CL238.Contig1\_Mf\_liverA, Unigene35507\_Mf\_liverA, Unigene6465\_Mf\_liverA, CL3617.Contig1\_Mf\_liverA, Unigene785\_Mf\_liverA, Unigene32920\_Mf\_liverA, Unigene27294\_Mf\_liverA, NM\_199027, Unigene1137\_Mf\_liverA, Unigene36434\_Mf\_liverA, Unigene34341\_Mf\_liverA, Unigene30142\_Mf\_liverA, Unigene30153\_Mf\_liverA, NM\_011921, Unigene7897\_Mf\_liverA, NM\_145474, Unigene33054\_Mf\_liverA, NM\_009776, Unigene30003\_Mf\_liverA, NM\_031165, CL497.Contig2\_Mf\_liverA, CL6.Contig1\_Mf\_liverA, Unigene34143\_Mf\_liverA, Unigene18848\_Mf\_liverA, Unigene30002\_Mf\_liverA, NM\_016697, Unigene802\_Mf\_liverA, Unigene15055\_Mf\_liverA, Unigene9150\_Mf\_liverA, CL4816.Contig1\_Mf\_liverA, Unigene5639\_Mf\_liverA, Unigene32594\_Mf\_liverA, Unigene13363\_Mf\_liverA, Unigene36175\_Mf\_liverA, NM\_021278, CL2855.Contig2\_Mf\_liverA, Unigene1479\_Mf\_liverA, NM\_010001, CL4249.Contig1\_Mf\_liverA, Unigene44882\_Mf\_liverA, Unigene7264\_Mf\_liverA, Unigene31625\_Mf\_liverA, Unigene5057\_Mf\_liverA, Unigene34032\_Mf\_liverA, Unigene27547\_Mf\_liverA, Unigene32908\_Mf\_liverA, Unigene24090\_Mf\_liverA, Unigene12909\_Mf\_liverA, CL5316.Contig1\_Mf\_liverA, CL1688.Contig3\_Mf\_liverA, Unigene4723\_Mf\_liverA, Unigene32332\_Mf\_liverA, NR\_003630, CL3930.Contig1\_Mf\_liverA, Unigene35169\_Mf\_liverA, Unigene1288\_Mf\_liverA, CL3166.Contig4\_Mf\_liverA, Unigene5169\_Mf\_liverA, Unigene32059\_Mf\_liverA, CL186.Contig3\_Mf\_liverA, Unigene30832\_Mf\_liverA, Unigene4697\_Mf\_liverA, CL4736.Contig1\_Mf\_liverA, Unigene14916\_Mf\_liverA, Unigene30217\_Mf\_liverA, CL4600.Contig1\_Mf\_liverA, Unigene2050\_Mf\_liverA, Unigene16184\_Mf\_liverA, NM\_029582, Unigene29134\_Mf\_liverA, Unigene35958\_Mf\_liverA, NM\_016689, Unigene4938\_Mf\_liverA, Unigene5815\_Mf\_liverA, Unigene34375\_Mf\_liverA, Unigene37224\_Mf\_liverA, Unigene28662\_Mf\_liverA, CL2439.Contig1\_Mf\_liverA, Unigene29426\_Mf\_liverA, Unigene6464\_Mf\_liverA, CL3933.Contig1\_Mf\_liverA, Unigene21026\_Mf\_liverA, Unigene20371\_Mf\_liverA, Unigene13683\_Mf\_liverA, CL1325.Contig1\_Mf\_liverA, Unigene13009\_Mf\_liverA, Unigene9698\_Mf\_liverA, Unigene36420\_Mf\_liverA, Unigene542\_Mf\_liverA, Unigene24758\_Mf\_liverA, CL5828.Contig2\_Mf\_liverA, Unigene33080\_Mf\_liverA, Unigene15433\_Mf\_liverA, NM\_019817, NM\_019879, Unigene15470\_Mf\_liverA, Unigene32434\_Mf\_liverA, Unigene34962\_Mf\_liverA, Unigene24225\_Mf\_liverA, CL1588.Contig2\_Mf\_liverA, Unigene37344\_Mf\_liverA, NM\_011207, CL5275.Contig2\_Mf\_liverA, NM\_145218, Unigene36189\_Mf\_liverA, Unigene29558\_Mf\_liverA, Unigene32133\_Mf\_liverA, Unigene281\_Mf\_liverA, Unigene20372\_Mf\_liverA, Unigene10313\_Mf\_liverA, NM\_177652, Unigene30548\_Mf\_liverA, Unigene13018\_Mf\_liverA, CL3104.Contig1\_Mf\_liverA, Unigene36593\_Mf\_liverA, NM\_013842, NM\_010002, Unigene16529\_Mf\_liverA, Unigene37232\_Mf\_liverA, CL5007.Contig1\_Mf\_liverA, Unigene14461\_Mf\_liverA, CL5698.Contig1\_Mf\_liverA, Unigene31392\_Mf\_liverA, NR\_024097, CL591.Contig1\_Mf\_liverA, Unigene35618\_Mf\_liverA, NM\_011256, Unigene38015\_Mf\_liverA, NM\_173038, NM\_206537, Unigene31199\_Mf\_liverA, Unigene6959\_Mf\_liverA, Unigene28244\_Mf\_liverA, CL523.Contig1\_Mf\_liverA, NM\_008277, CL482.Contig1\_Mf\_liverA, Unigene35382\_Mf\_liverA, Unigene36762\_Mf\_liverA, Unigene600\_Mf\_liverA, Unigene18795\_Mf\_liverA, Unigene37698\_Mf\_liverA, Unigene30878\_Mf\_liverA, Unigene40289\_Mf\_liverA, NM\_134012, Unigene18500\_Mf\_liverA, Unigene32570\_Mf\_liverA, CL4910.Contig1\_Mf\_liverA, CL1256.Contig1\_Mf\_liverA, Unigene5333\_Mf\_liverA, Unigene15295\_Mf\_liverA, Unigene12999\_Mf\_liverA, CL1119.Contig1\_Mf\_liverA, Unigene31333\_Mf\_liverA, Unigene30369\_Mf\_liverA, Unigene33598\_Mf\_liverA, Unigene25596\_Mf\_liverA, Unigene843\_Mf\_liverA, Unigene37109\_Mf\_liverA, Unigene28852\_Mf\_liverA, NM\_001004357, CL3778.Contig2\_Mf\_liverA, CL4076.Contig1\_Mf\_liverA, CL787.Contig1\_Mf\_liverA, Unigene29490\_Mf\_liverA, NM\_001162917, CL2389.Contig1\_Mf\_liverA, CL3750.Contig2\_Mf\_liverA, CL335.Contig1\_Mf\_liverA, Unigene39141\_Mf\_liverA, CL784.Contig3\_Mf\_liverA, CL838.Contig3\_Mf\_liverA, Unigene5632\_Mf\_liverA, Unigene28520\_Mf\_liverA, CL4550.Contig3\_Mf\_liverA, Unigene21816\_Mf\_liverA, Unigene27419\_Mf\_liverA, Unigene21742\_Mf\_liverA, Unigene31427\_Mf\_liverA, CL4095.Contig4\_Mf\_liverA, CL4283.Contig1\_Mf\_liverA, Unigene30584\_Mf\_liverA, Unigene17579\_Mf\_liverA, NM\_001081372, CL913.Contig1\_Mf\_liverA, NM\_021525, Unigene27082\_Mf\_liverA, Unigene37178\_Mf\_liverA, Unigene32058\_Mf\_liverA, Unigene5712\_Mf\_liverA, Unigene36772\_Mf\_liverA, CL442.Contig2\_Mf\_liverA, NM\_010380, CL2855.Contig1\_Mf\_liverA, CL4411.Contig4\_Mf\_liverA, NM\_013821, Unigene40796\_Mf\_liverA, NM\_001100182, NM\_025706, Unigene31051\_Mf\_liverA, Unigene392\_Mf\_liverA, NM\_011732, CL4228.Contig1\_Mf\_liverA, NM\_009178, Unigene49971\_Mf\_liverA, Unigene38307\_Mf\_liverA, Unigene36190\_Mf\_liverA, Unigene27406\_Mf\_liverA, Unigene34754\_Mf\_liverA, Unigene30154\_Mf\_liverA, CL4162.Contig1\_Mf\_liverA, Unigene27922\_Mf\_liverA, Unigene15476\_Mf\_liverA, CL1100.Contig1\_Mf\_liverA, Unigene32093\_Mf\_liverA, CL4583.Contig2\_Mf\_liverA, CL485.Contig1\_Mf\_liverA, Unigene36414\_Mf\_liverA, CL1988.Contig1\_Mf\_liverA, Unigene5175\_Mf\_liverA, Unigene13894\_Mf\_liverA, Unigene36328\_Mf\_liverA, NM\_010141, CL4745.Contig1\_Mf\_liverA, Unigene36315\_Mf\_liverA, CL2327.Contig1\_Mf\_liverA, CL887.Contig2\_Mf\_liverA, Unigene33736\_Mf\_liverA, Unigene35037\_Mf\_liverA, CL1656.Contig1\_Mf\_liverA, CL3684.Contig2\_Mf\_liverA, Unigene1212\_Mf\_liverA, Unigene32233\_Mf\_liverA, CL838.Contig6\_Mf\_liverA, Unigene10915\_Mf\_liverA, Unigene32143\_Mf\_liverA, Unigene24536\_Mf\_liverA, CL3750.Contig1\_Mf\_liverA, Unigene13196\_Mf\_liverA, Unigene601\_Mf\_liverA, CL1076.Contig1\_Mf\_liverA, Unigene18796\_Mf\_liverA, NM\_009448, CL5307.Contig1\_Mf\_liverA, Unigene31670\_Mf\_liverA, NM\_178405, NM\_008483, NM\_172203, Unigene120\_Mf\_liverA, Unigene15026\_Mf\_liverA, CL4007.Contig1\_Mf\_liverA, NM\_001081116, NM\_017379, Unigene28489\_Mf\_liverA, CL4984.Contig1\_Mf\_liverA, NM\_001081274, Unigene24537\_Mf\_liverA, Unigene32335\_Mf\_liverA, Unigene7224\_Mf\_liverA, NM\_027604, Unigene34444\_Mf\_liverA, Unigene7399\_Mf\_liverA, Unigene35678\_Mf\_liverA, Unigene33993\_Mf\_liverA, Unigene8560\_Mf\_liverA, Unigene44057\_Mf\_liverA, Unigene5761\_Mf\_liverA, Unigene12519\_Mf\_liverA, Unigene37819\_Mf\_liverA, Unigene5886\_Mf\_liverA, CL336.Contig3\_Mf\_liverA, CL1198.Contig1\_Mf\_liverA |
| phagocytic vesicle | Unigene14809\_Mf\_liverA, Unigene36328\_Mf\_liverA, Unigene37389\_Mf\_liverA |
| anchored to membrane | Unigene13097\_Mf\_liverA, Unigene37698\_Mf\_liverA, Unigene5169\_Mf\_liverA, NM\_016697, Unigene31333\_Mf\_liverA |
| COPI-coated vesicle | NM\_019817, Unigene5632\_Mf\_liverA |
| acrosomal vesicle | Unigene15055\_Mf\_liverA, Unigene33080\_Mf\_liverA, Unigene19687\_Mf\_liverA, Unigene35958\_Mf\_liverA, Unigene13950\_Mf\_liverA |
| Ada2/Gcn5/Ada3 transcription activator complex | Unigene1479\_Mf\_liverA, Unigene30288\_Mf\_liverA |
| ER to Golgi transport vesicle membrane | CL2855.Contig1\_Mf\_liverA, CL2855.Contig2\_Mf\_liverA |
| basolateral plasma membrane | NM\_026313, Unigene32803\_Mf\_liverA, CL5007.Contig1\_Mf\_liverA, Unigene36898\_Mf\_liverA, CL238.Contig1\_Mf\_liverA, CL1256.Contig1\_Mf\_liverA, Unigene32335\_Mf\_liverA, Unigene1288\_Mf\_liverA, Unigene42606\_Mf\_liverA, CL4228.Contig1\_Mf\_liverA, Unigene22423\_Mf\_liverA, Unigene15572\_Mf\_liverA, CL3150.Contig1\_Mf\_liverA, Unigene880\_Mf\_liverA, CL838.Contig3\_Mf\_liverA, Unigene13950\_Mf\_liverA |
| microvillus | Unigene36698\_Mf\_liverA, Unigene28244\_Mf\_liverA, Unigene32803\_Mf\_liverA, Unigene36898\_Mf\_liverA, Unigene1288\_Mf\_liverA |
| secretory granule membrane | Unigene34394\_Mf\_liverA, CL5807.Contig1\_Mf\_liverA, Unigene32789\_Mf\_liverA, Unigene36898\_Mf\_liverA, CL2697.Contig4\_Mf\_liverA |
| dendritic spine | Unigene27547\_Mf\_liverA, Unigene26611\_Mf\_liverA, CL425.Contig1\_Mf\_liverA, Unigene32570\_Mf\_liverA, Unigene24883\_Mf\_liverA, Unigene13593\_Mf\_liverA, Unigene16529\_Mf\_liverA |
| neuron spine | Unigene27547\_Mf\_liverA, Unigene26611\_Mf\_liverA, CL425.Contig1\_Mf\_liverA, Unigene32570\_Mf\_liverA, Unigene24883\_Mf\_liverA, Unigene13593\_Mf\_liverA, Unigene16529\_Mf\_liverA |
| basement membrane | NM\_008483, Unigene29628\_Mf\_liverA, Unigene32908\_Mf\_liverA, Unigene32434\_Mf\_liverA, Unigene34124\_Mf\_liverA, Unigene31492\_Mf\_liverA |
| cell projection membrane | NM\_172203, NM\_001004357, Unigene36898\_Mf\_liverA, Unigene42606\_Mf\_liverA, CL4228.Contig1\_Mf\_liverA, Unigene49971\_Mf\_liverA, Unigene5693\_Mf\_liverA, Unigene24883\_Mf\_liverA, Unigene28244\_Mf\_liverA |
| focal adhesion | CL4228.Contig1\_Mf\_liverA, Unigene22423\_Mf\_liverA, Unigene32803\_Mf\_liverA, CL5007.Contig1\_Mf\_liverA, Unigene15572\_Mf\_liverA, CL838.Contig3\_Mf\_liverA |
| cytoplasmic membrane-bounded vesicle lumen | CL6038.Contig2\_Mf\_liverA, Unigene14809\_Mf\_liverA, Unigene28662\_Mf\_liverA |
| vesicle coat | NM\_019817, Unigene32335\_Mf\_liverA, Unigene5632\_Mf\_liverA |
| very-low-density lipoprotein particle | CL3911.Contig2\_Mf\_liverA, Unigene13498\_Mf\_liverA |
| basal part of cell | Unigene28244\_Mf\_liverA, Unigene32803\_Mf\_liverA, Unigene36898\_Mf\_liverA |
| Golgi membrane | CL787.Contig1\_Mf\_liverA, Unigene15055\_Mf\_liverA, Unigene31051\_Mf\_liverA, Unigene36328\_Mf\_liverA, Unigene281\_Mf\_liverA, CL2855.Contig2\_Mf\_liverA, Unigene32335\_Mf\_liverA, Unigene37389\_Mf\_liverA, NM\_009178, CL4033.Contig1\_Mf\_liverA, Unigene16529\_Mf\_liverA, Unigene5632\_Mf\_liverA, Unigene27082\_Mf\_liverA, CL5007.Contig1\_Mf\_liverA, CL4600.Contig1\_Mf\_liverA, CL2855.Contig1\_Mf\_liverA, NM\_008851, Unigene32920\_Mf\_liverA |
| caveola | CL3154.Contig1\_Mf\_liverA, Unigene27922\_Mf\_liverA, CL887.Contig2\_Mf\_liverA, Unigene31267\_Mf\_liverA |
| trans-Golgi network | CL2855.Contig1\_Mf\_liverA, CL4919.Contig1\_Mf\_liverA, CL2855.Contig2\_Mf\_liverA, Unigene31988\_Mf\_liverA, CL2697.Contig4\_Mf\_liverA |
| intracellular part | NM\_007396, Unigene21684\_Mf\_liverA, NM\_176843, CL4302.Contig1\_Mf\_liverA, NM\_009450, Unigene27730\_Mf\_liverA, NM\_144940, CL3669.Contig2\_Mf\_liverA, Unigene29011\_Mf\_liverA, NM\_025593, Unigene36172\_Mf\_liverA, Unigene37334\_Mf\_liverA, Unigene36851\_Mf\_liverA, NM\_080638, NM\_010378, Unigene15553\_Mf\_liverA, NM\_011082, Unigene19687\_Mf\_liverA, CL854.Contig1\_Mf\_liverA, CL4105.Contig1\_Mf\_liverA, Unigene26053\_Mf\_liverA, Unigene7336\_Mf\_liverA, Unigene35507\_Mf\_liverA, CL238.Contig1\_Mf\_liverA, Unigene6465\_Mf\_liverA, CL3617.Contig1\_Mf\_liverA, Unigene785\_Mf\_liverA, Unigene39886\_Mf\_liverA, Unigene15064\_Mf\_liverA, Unigene32920\_Mf\_liverA, Unigene34124\_Mf\_liverA, NR\_004446, Unigene36765\_Mf\_liverA, Unigene36434\_Mf\_liverA, Unigene34341\_Mf\_liverA, Unigene30153\_Mf\_liverA, Unigene30142\_Mf\_liverA, CL695.Contig1\_Mf\_liverA, Unigene34847\_Mf\_liverA, Unigene29082\_Mf\_liverA, NM\_011921, Unigene2\_Mf\_liverA, CL4919.Contig1\_Mf\_liverA, Unigene31623\_Mf\_liverA, Unigene7897\_Mf\_liverA, CL2266.Contig1\_Mf\_liverA, NM\_010233, NM\_145474, Unigene37084\_Mf\_liverA, Unigene20874\_Mf\_liverA, NM\_009776, Unigene33054\_Mf\_liverA, Unigene34394\_Mf\_liverA, Unigene30003\_Mf\_liverA, NM\_031165, Unigene25594\_Mf\_liverA, CL497.Contig2\_Mf\_liverA, Unigene36430\_Mf\_liverA, CL6.Contig1\_Mf\_liverA, Unigene18848\_Mf\_liverA, Unigene30002\_Mf\_liverA, NM\_016697, Unigene802\_Mf\_liverA, CL4816.Contig1\_Mf\_liverA, Unigene9150\_Mf\_liverA, Unigene15055\_Mf\_liverA, Unigene5639\_Mf\_liverA, CL3154.Contig1\_Mf\_liverA, CL5807.Contig1\_Mf\_liverA, Unigene13363\_Mf\_liverA, Unigene32594\_Mf\_liverA, Unigene743\_Mf\_liverA, Unigene37454\_Mf\_liverA, Unigene36417\_Mf\_liverA, CL2855.Contig2\_Mf\_liverA, CL4263.Contig1\_Mf\_liverA, NM\_021278, Unigene36175\_Mf\_liverA, CL4293.Contig1\_Mf\_liverA, Unigene14907\_Mf\_liverA, Unigene1479\_Mf\_liverA, CL4249.Contig1\_Mf\_liverA, NM\_010001, Unigene25976\_Mf\_liverA, NM\_009338, Unigene44882\_Mf\_liverA, Unigene36698\_Mf\_liverA, NM\_145942, Unigene139\_Mf\_liverA, Unigene15572\_Mf\_liverA, Unigene31625\_Mf\_liverA, Unigene37018\_Mf\_liverA, NM\_022324, Unigene10756\_Mf\_liverA, Unigene34032\_Mf\_liverA, Unigene5057\_Mf\_liverA, Unigene27547\_Mf\_liverA, Unigene15290\_Mf\_liverA, Unigene24090\_Mf\_liverA, Unigene32789\_Mf\_liverA, Unigene12909\_Mf\_liverA, Unigene36898\_Mf\_liverA, CL5316.Contig1\_Mf\_liverA, Unigene35664\_Mf\_liverA, CL1688.Contig3\_Mf\_liverA, NM\_027660, Unigene37389\_Mf\_liverA, Unigene32332\_Mf\_liverA, Unigene4723\_Mf\_liverA, NR\_003630, CL3930.Contig1\_Mf\_liverA, Unigene41957\_Mf\_liverA, Unigene30839\_Mf\_liverA, Unigene32852\_Mf\_liverA, Unigene24613\_Mf\_liverA, Unigene26611\_Mf\_liverA, CL532.Contig1\_Mf\_liverA, Unigene15477\_Mf\_liverA, Unigene35169\_Mf\_liverA, Unigene1280\_Mf\_liverA, Unigene30288\_Mf\_liverA, CL3166.Contig4\_Mf\_liverA, NM\_027959, Unigene34972\_Mf\_liverA, Unigene32059\_Mf\_liverA, Unigene34140\_Mf\_liverA, Unigene4363\_Mf\_liverA, Unigene836\_Mf\_liverA, CL186.Contig3\_Mf\_liverA, CL425.Contig1\_Mf\_liverA, CL2797.Contig2\_Mf\_liverA, Unigene30832\_Mf\_liverA, CL4736.Contig1\_Mf\_liverA, Unigene28143\_Mf\_liverA, Unigene14916\_Mf\_liverA, Unigene37243\_Mf\_liverA, Unigene30217\_Mf\_liverA, Unigene15077\_Mf\_liverA, Unigene2050\_Mf\_liverA, CL4600.Contig1\_Mf\_liverA, Unigene16184\_Mf\_liverA, Unigene5693\_Mf\_liverA, CL3382.Contig1\_Mf\_liverA, NM\_029582, Unigene29134\_Mf\_liverA, Unigene35958\_Mf\_liverA, NM\_016689, Unigene29876\_Mf\_liverA, Unigene28687\_Mf\_liverA, Unigene32803\_Mf\_liverA, Unigene5815\_Mf\_liverA, Unigene34375\_Mf\_liverA, CL1988.Contig3\_Mf\_liverA, NM\_172679, CL1810.Contig1\_Mf\_liverA, CL4033.Contig1\_Mf\_liverA, Unigene34983\_Mf\_liverA, Unigene28662\_Mf\_liverA, CL2439.Contig1\_Mf\_liverA, CL3519.Contig1\_Mf\_liverA, Unigene33330\_Mf\_liverA, Unigene6464\_Mf\_liverA, Unigene29426\_Mf\_liverA, CL3933.Contig1\_Mf\_liverA, Unigene21026\_Mf\_liverA, NM\_011221, Unigene20371\_Mf\_liverA, CL1325.Contig1\_Mf\_liverA, CL1493.Contig1\_Mf\_liverA, Unigene13683\_Mf\_liverA, Unigene13009\_Mf\_liverA, Unigene9698\_Mf\_liverA, Unigene36420\_Mf\_liverA, Unigene30004\_Mf\_liverA, Unigene542\_Mf\_liverA, Unigene35476\_Mf\_liverA, Unigene24758\_Mf\_liverA, Unigene24471\_Mf\_liverA, Unigene31571\_Mf\_liverA, CL5828.Contig2\_Mf\_liverA, Unigene35168\_Mf\_liverA, Unigene33080\_Mf\_liverA, Unigene15888\_Mf\_liverA, CL4220.Contig1\_Mf\_liverA, Unigene15433\_Mf\_liverA, Unigene37262\_Mf\_liverA, CL4338.Contig1\_Mf\_liverA, Unigene12908\_Mf\_liverA, CL5052.Contig1\_Mf\_liverA, NM\_019817, NM\_010469, Unigene5750\_Mf\_liverA, NM\_019879, NR\_033215, NM\_007822, CL5293.Contig1\_Mf\_liverA, Unigene25595\_Mf\_liverA, Unigene14263\_Mf\_liverA, Unigene15470\_Mf\_liverA, Unigene14286\_Mf\_liverA, Unigene32434\_Mf\_liverA, CL4816.Contig3\_Mf\_liverA, Unigene34962\_Mf\_liverA, CL114.Contig1\_Mf\_liverA, Unigene39011\_Mf\_liverA, Unigene24225\_Mf\_liverA, Unigene37344\_Mf\_liverA, Unigene8218\_Mf\_liverA, CL1588.Contig2\_Mf\_liverA, CL5275.Contig2\_Mf\_liverA, NM\_011207, NM\_145218, Unigene36189\_Mf\_liverA, NM\_008183, Unigene29558\_Mf\_liverA, Unigene13950\_Mf\_liverA, Unigene28459\_Mf\_liverA, Unigene37245\_Mf\_liverA, Unigene32133\_Mf\_liverA, Unigene281\_Mf\_liverA, NM\_177652, NM\_011978, Unigene10313\_Mf\_liverA, Unigene20372\_Mf\_liverA, Unigene37104\_Mf\_liverA, CL3104.Contig1\_Mf\_liverA, Unigene13018\_Mf\_liverA, Unigene36593\_Mf\_liverA, NM\_010002, NM\_013842, NM\_027406, Unigene36176\_Mf\_liverA, Unigene16529\_Mf\_liverA, Unigene37232\_Mf\_liverA, NM\_026313, Unigene28609\_Mf\_liverA, CL5007.Contig1\_Mf\_liverA, CL5698.Contig1\_Mf\_liverA, Unigene14461\_Mf\_liverA, Unigene31392\_Mf\_liverA, CL591.Contig1\_Mf\_liverA, Unigene29889\_Mf\_liverA, Unigene2939\_Mf\_liverA, Unigene35618\_Mf\_liverA, NM\_011256, Unigene35884\_Mf\_liverA, CL5640.Contig1\_Mf\_liverA, Unigene38015\_Mf\_liverA, NM\_028291, NM\_173038, NM\_206537, CL3835.Contig2\_Mf\_liverA, Unigene31199\_Mf\_liverA, Unigene6959\_Mf\_liverA, Unigene28899\_Mf\_liverA, CL4550.Contig2\_Mf\_liverA, Unigene28244\_Mf\_liverA, Unigene46870\_Mf\_liverA, NM\_019651, CL523.Contig1\_Mf\_liverA, NM\_008277, Unigene8473\_Mf\_liverA, CL482.Contig1\_Mf\_liverA, Unigene27071\_Mf\_liverA, Unigene36762\_Mf\_liverA, Unigene35382\_Mf\_liverA, Unigene600\_Mf\_liverA, NM\_011072, Unigene18795\_Mf\_liverA, Unigene28564\_Mf\_liverA, Unigene30878\_Mf\_liverA, Unigene40289\_Mf\_liverA, NM\_173753, NM\_134012, CL6038.Contig2\_Mf\_liverA, Unigene32570\_Mf\_liverA, Unigene37904\_Mf\_liverA, Unigene5333\_Mf\_liverA, CL3483.Contig1\_Mf\_liverA, Unigene15295\_Mf\_liverA, Unigene34789\_Mf\_liverA, Unigene12999\_Mf\_liverA, CL4141.Contig1\_Mf\_liverA, Unigene24221\_Mf\_liverA, NM\_145419, Unigene34184\_Mf\_liverA, CL5978.Contig3\_Mf\_liverA, CL1119.Contig1\_Mf\_liverA, Unigene13658\_Mf\_liverA, Unigene13593\_Mf\_liverA, NM\_134127, Unigene32546\_Mf\_liverA, Unigene11\_Mf\_liverA, CL2622.Contig2\_Mf\_liverA, Unigene14609\_Mf\_liverA, Unigene843\_Mf\_liverA, Unigene25596\_Mf\_liverA, Unigene37109\_Mf\_liverA, CL3778.Contig2\_Mf\_liverA, Unigene12997\_Mf\_liverA, Unigene5994\_Mf\_liverA, CL1493.Contig2\_Mf\_liverA, CL2697.Contig4\_Mf\_liverA, NM\_008261, NM\_024434, CL2574.Contig1\_Mf\_liverA, Unigene5941\_Mf\_liverA, Unigene38104\_Mf\_liverA, Unigene23328\_Mf\_liverA, Unigene36626\_Mf\_liverA, CL787.Contig1\_Mf\_liverA, CL4076.Contig1\_Mf\_liverA, Unigene29490\_Mf\_liverA, CL3897.Contig5\_Mf\_liverA, Unigene12967\_Mf\_liverA, Unigene36691\_Mf\_liverA, NM\_001162917, Unigene31988\_Mf\_liverA, CL2389.Contig1\_Mf\_liverA, CL3750.Contig2\_Mf\_liverA, CL335.Contig1\_Mf\_liverA, CL3599.Contig1\_Mf\_liverA, Unigene151\_Mf\_liverA, Unigene39141\_Mf\_liverA, CL848.Contig2\_Mf\_liverA, Unigene31639\_Mf\_liverA, CL784.Contig3\_Mf\_liverA, CL838.Contig3\_Mf\_liverA, Unigene10774\_Mf\_liverA, Unigene28520\_Mf\_liverA, Unigene5632\_Mf\_liverA, CL1263.Contig1\_Mf\_liverA, CL4550.Contig3\_Mf\_liverA, Unigene21816\_Mf\_liverA, CL258.Contig1\_Mf\_liverA, Unigene27419\_Mf\_liverA, NM\_007811, Unigene24323\_Mf\_liverA, NM\_008538, CL2142.Contig2\_Mf\_liverA, Unigene30428\_Mf\_liverA, NM\_011305, Unigene21741\_Mf\_liverA, Unigene21742\_Mf\_liverA, Unigene13498\_Mf\_liverA, Unigene31427\_Mf\_liverA, CL529.Contig2\_Mf\_liverA, Unigene40069\_Mf\_liverA, Unigene30584\_Mf\_liverA, Unigene4510\_Mf\_liverA, Unigene17579\_Mf\_liverA, NM\_001081372, Unigene37148\_Mf\_liverA, CL913.Contig1\_Mf\_liverA, Unigene34406\_Mf\_liverA, NM\_021525, Unigene7195\_Mf\_liverA, Unigene35506\_Mf\_liverA, Unigene32058\_Mf\_liverA, Unigene27082\_Mf\_liverA, Unigene37178\_Mf\_liverA, Unigene5712\_Mf\_liverA, CL2919.Contig1\_Mf\_liverA, Unigene1205\_Mf\_liverA, Unigene36772\_Mf\_liverA, CL442.Contig2\_Mf\_liverA, CL6039.Contig1\_Mf\_liverA, CL2855.Contig1\_Mf\_liverA, CL4411.Contig4\_Mf\_liverA, NM\_013821, Unigene40796\_Mf\_liverA, Unigene33420\_Mf\_liverA, Unigene32412\_Mf\_liverA, Unigene17569\_Mf\_liverA, NM\_001100182, Unigene31051\_Mf\_liverA, NM\_025706, Unigene392\_Mf\_liverA, NM\_011732, Unigene30426\_Mf\_liverA, NM\_009178, Unigene49971\_Mf\_liverA, Unigene38307\_Mf\_liverA, Unigene36190\_Mf\_liverA, Unigene27406\_Mf\_liverA, Unigene34754\_Mf\_liverA, Unigene17042\_Mf\_liverA, Unigene34811\_Mf\_liverA, Unigene30154\_Mf\_liverA, Unigene3789\_Mf\_liverA, Unigene33746\_Mf\_liverA, CL1125.Contig1\_Mf\_liverA, CL4162.Contig1\_Mf\_liverA, Unigene1000\_Mf\_liverA, Unigene27922\_Mf\_liverA, Unigene15681\_Mf\_liverA, Unigene673\_Mf\_liverA, Unigene15476\_Mf\_liverA, CL1100.Contig1\_Mf\_liverA, Unigene32093\_Mf\_liverA, CL4583.Contig2\_Mf\_liverA, Unigene36414\_Mf\_liverA, NM\_021423, CL485.Contig1\_Mf\_liverA, CL1988.Contig1\_Mf\_liverA, Unigene13894\_Mf\_liverA, Unigene5175\_Mf\_liverA, Unigene36328\_Mf\_liverA, Unigene15552\_Mf\_liverA, Unigene38280\_Mf\_liverA, CL4745.Contig1\_Mf\_liverA, CL5309.Contig1\_Mf\_liverA, CL3002.Contig1\_Mf\_liverA, Unigene31483\_Mf\_liverA, Unigene36315\_Mf\_liverA, Unigene31267\_Mf\_liverA, CL2327.Contig1\_Mf\_liverA, Unigene36949\_Mf\_liverA, Unigene5488\_Mf\_liverA, CL887.Contig2\_Mf\_liverA, CL2597.Contig1\_Mf\_liverA, Unigene33736\_Mf\_liverA, Unigene33523\_Mf\_liverA, Unigene35037\_Mf\_liverA, Unigene14810\_Mf\_liverA, CL3684.Contig2\_Mf\_liverA, CL1656.Contig1\_Mf\_liverA, Unigene1212\_Mf\_liverA, NM\_001100181, Unigene32233\_Mf\_liverA, CL838.Contig6\_Mf\_liverA, Unigene5607\_Mf\_liverA, CL4770.Contig1\_Mf\_liverA, Unigene32143\_Mf\_liverA, Unigene24536\_Mf\_liverA, CL3750.Contig1\_Mf\_liverA, Unigene13196\_Mf\_liverA, CL114.Contig2\_Mf\_liverA, Unigene14449\_Mf\_liverA, CL1076.Contig1\_Mf\_liverA, Unigene39520\_Mf\_liverA, Unigene601\_Mf\_liverA, NM\_009883, Unigene18796\_Mf\_liverA, Unigene22423\_Mf\_liverA, NM\_009448, CL5307.Contig1\_Mf\_liverA, Unigene1327\_Mf\_liverA, Unigene31670\_Mf\_liverA, CL5978.Contig2\_Mf\_liverA, NM\_178405, NM\_172203, CL442.Contig5\_Mf\_liverA, Unigene120\_Mf\_liverA, Unigene36418\_Mf\_liverA, Unigene15026\_Mf\_liverA, CL4007.Contig1\_Mf\_liverA, NM\_022997, NM\_001081116, Unigene550\_Mf\_liverA, NM\_017379, CL55.Contig1\_Mf\_liverA, Unigene28489\_Mf\_liverA, Unigene37120\_Mf\_liverA, Unigene31198\_Mf\_liverA, CL2355.Contig1\_Mf\_liverA, Unigene4863\_Mf\_liverA, Unigene24537\_Mf\_liverA, NM\_001081274, CL4984.Contig1\_Mf\_liverA, Unigene32335\_Mf\_liverA, Unigene37153\_Mf\_liverA, Unigene14809\_Mf\_liverA, NM\_027604, Unigene5489\_Mf\_liverA, Unigene34444\_Mf\_liverA, Unigene7399\_Mf\_liverA, Unigene35678\_Mf\_liverA, Unigene33993\_Mf\_liverA, Unigene138\_Mf\_liverA, NM\_009011, Unigene8560\_Mf\_liverA, CL3911.Contig2\_Mf\_liverA, Unigene44057\_Mf\_liverA, Unigene5761\_Mf\_liverA, Unigene4681\_Mf\_liverA, Unigene12519\_Mf\_liverA, NM\_008851, Unigene37819\_Mf\_liverA, Unigene5886\_Mf\_liverA, CL336.Contig3\_Mf\_liverA, CL1198.Contig1\_Mf\_liverA |
| basal lamina | NM\_008483, Unigene29628\_Mf\_liverA, Unigene32908\_Mf\_liverA |
| cell body | Unigene18500\_Mf\_liverA, Unigene27547\_Mf\_liverA, NM\_010141, Unigene36898\_Mf\_liverA, Unigene34789\_Mf\_liverA, CL1810.Contig1\_Mf\_liverA, CL425.Contig1\_Mf\_liverA, Unigene13593\_Mf\_liverA, Unigene16529\_Mf\_liverA, Unigene33746\_Mf\_liverA, Unigene26611\_Mf\_liverA, Unigene14609\_Mf\_liverA, Unigene5712\_Mf\_liverA, Unigene27922\_Mf\_liverA, Unigene14461\_Mf\_liverA, Unigene16184\_Mf\_liverA, CL3166.Contig4\_Mf\_liverA, CL2697.Contig4\_Mf\_liverA, Unigene28564\_Mf\_liverA, Unigene13950\_Mf\_liverA |
| vesicle membrane | Unigene28143\_Mf\_liverA, Unigene27082\_Mf\_liverA, Unigene34394\_Mf\_liverA, CL5807.Contig1\_Mf\_liverA, Unigene36328\_Mf\_liverA, NM\_031165, Unigene32789\_Mf\_liverA, Unigene36898\_Mf\_liverA, CL2855.Contig2\_Mf\_liverA, Unigene32335\_Mf\_liverA, CL2697.Contig4\_Mf\_liverA, CL2855.Contig1\_Mf\_liverA, NM\_019817, Unigene5750\_Mf\_liverA, Unigene5632\_Mf\_liverA |
| intrinsic to Golgi membrane | CL787.Contig1\_Mf\_liverA, NM\_009178, Unigene31051\_Mf\_liverA, Unigene32920\_Mf\_liverA |
| synaptic vesicle membrane | Unigene34394\_Mf\_liverA, CL5807.Contig1\_Mf\_liverA |
| membrane fraction | NM\_001100182, NM\_001081372, NM\_206537, NM\_001081274, NM\_011978, NM\_010002, NM\_010001, NM\_134127, NM\_178405, NM\_145474, NM\_008483, NM\_007822, NM\_007811, NM\_001100181, NM\_011072, NM\_013821 |
| ER to Golgi transport vesicle | CL2855.Contig1\_Mf\_liverA, CL2855.Contig2\_Mf\_liverA |
| axon terminus | Unigene36898\_Mf\_liverA, Unigene16529\_Mf\_liverA |
| intracellular | NM\_007396, Unigene21684\_Mf\_liverA, NM\_176843, CL4302.Contig1\_Mf\_liverA, NM\_009450, Unigene27730\_Mf\_liverA, NM\_144940, CL3669.Contig2\_Mf\_liverA, Unigene29011\_Mf\_liverA, NM\_025593, Unigene36172\_Mf\_liverA, Unigene37334\_Mf\_liverA, Unigene36851\_Mf\_liverA, NM\_080638, NM\_010378, Unigene15553\_Mf\_liverA, NM\_011082, Unigene19687\_Mf\_liverA, CL854.Contig1\_Mf\_liverA, CL4105.Contig1\_Mf\_liverA, Unigene26053\_Mf\_liverA, Unigene7336\_Mf\_liverA, Unigene35507\_Mf\_liverA, CL238.Contig1\_Mf\_liverA, Unigene6465\_Mf\_liverA, CL3617.Contig1\_Mf\_liverA, Unigene785\_Mf\_liverA, Unigene39886\_Mf\_liverA, Unigene15064\_Mf\_liverA, Unigene32920\_Mf\_liverA, Unigene34124\_Mf\_liverA, NR\_004446, Unigene36765\_Mf\_liverA, Unigene36434\_Mf\_liverA, Unigene34341\_Mf\_liverA, Unigene30153\_Mf\_liverA, Unigene30142\_Mf\_liverA, CL695.Contig1\_Mf\_liverA, Unigene34847\_Mf\_liverA, Unigene29082\_Mf\_liverA, NM\_011921, Unigene2\_Mf\_liverA, CL4919.Contig1\_Mf\_liverA, Unigene31623\_Mf\_liverA, Unigene7897\_Mf\_liverA, CL2266.Contig1\_Mf\_liverA, NM\_010233, NM\_145474, Unigene37084\_Mf\_liverA, Unigene20874\_Mf\_liverA, NM\_009776, Unigene33054\_Mf\_liverA, Unigene34394\_Mf\_liverA, Unigene30003\_Mf\_liverA, NM\_031165, Unigene25594\_Mf\_liverA, CL497.Contig2\_Mf\_liverA, Unigene36430\_Mf\_liverA, CL6.Contig1\_Mf\_liverA, Unigene34143\_Mf\_liverA, Unigene18848\_Mf\_liverA, Unigene30002\_Mf\_liverA, NM\_016697, Unigene802\_Mf\_liverA, CL4816.Contig1\_Mf\_liverA, Unigene9150\_Mf\_liverA, Unigene15055\_Mf\_liverA, Unigene5639\_Mf\_liverA, CL3154.Contig1\_Mf\_liverA, CL5807.Contig1\_Mf\_liverA, Unigene13363\_Mf\_liverA, Unigene32594\_Mf\_liverA, Unigene743\_Mf\_liverA, Unigene37454\_Mf\_liverA, Unigene36417\_Mf\_liverA, CL2855.Contig2\_Mf\_liverA, CL4263.Contig1\_Mf\_liverA, NM\_021278, Unigene36175\_Mf\_liverA, CL4293.Contig1\_Mf\_liverA, Unigene14907\_Mf\_liverA, Unigene1479\_Mf\_liverA, CL4249.Contig1\_Mf\_liverA, NM\_010001, Unigene25976\_Mf\_liverA, NM\_009338, Unigene44882\_Mf\_liverA, Unigene36698\_Mf\_liverA, NM\_145942, Unigene139\_Mf\_liverA, Unigene15572\_Mf\_liverA, Unigene31625\_Mf\_liverA, Unigene37018\_Mf\_liverA, NM\_022324, Unigene10756\_Mf\_liverA, Unigene34032\_Mf\_liverA, Unigene5057\_Mf\_liverA, Unigene27547\_Mf\_liverA, Unigene15290\_Mf\_liverA, Unigene24090\_Mf\_liverA, Unigene32789\_Mf\_liverA, Unigene12909\_Mf\_liverA, Unigene36898\_Mf\_liverA, CL5316.Contig1\_Mf\_liverA, Unigene35664\_Mf\_liverA, CL1688.Contig3\_Mf\_liverA, NM\_027660, Unigene37389\_Mf\_liverA, Unigene32332\_Mf\_liverA, Unigene4723\_Mf\_liverA, NR\_003630, CL3930.Contig1\_Mf\_liverA, Unigene41957\_Mf\_liverA, Unigene30839\_Mf\_liverA, Unigene32852\_Mf\_liverA, Unigene24613\_Mf\_liverA, Unigene26611\_Mf\_liverA, CL532.Contig1\_Mf\_liverA, Unigene15477\_Mf\_liverA, Unigene35169\_Mf\_liverA, Unigene1280\_Mf\_liverA, Unigene30288\_Mf\_liverA, CL3166.Contig4\_Mf\_liverA, NM\_027959, Unigene34972\_Mf\_liverA, Unigene32059\_Mf\_liverA, Unigene34140\_Mf\_liverA, Unigene4363\_Mf\_liverA, Unigene836\_Mf\_liverA, CL186.Contig3\_Mf\_liverA, CL425.Contig1\_Mf\_liverA, CL2797.Contig2\_Mf\_liverA, Unigene30832\_Mf\_liverA, CL4736.Contig1\_Mf\_liverA, Unigene28143\_Mf\_liverA, Unigene14916\_Mf\_liverA, Unigene37243\_Mf\_liverA, Unigene30217\_Mf\_liverA, Unigene15077\_Mf\_liverA, Unigene2050\_Mf\_liverA, CL4600.Contig1\_Mf\_liverA, Unigene16184\_Mf\_liverA, Unigene5693\_Mf\_liverA, CL3382.Contig1\_Mf\_liverA, NM\_029582, Unigene29134\_Mf\_liverA, Unigene35958\_Mf\_liverA, NM\_016689, Unigene29876\_Mf\_liverA, Unigene28687\_Mf\_liverA, Unigene32803\_Mf\_liverA, Unigene5815\_Mf\_liverA, Unigene34375\_Mf\_liverA, CL1988.Contig3\_Mf\_liverA, Unigene37224\_Mf\_liverA, NM\_172679, CL1810.Contig1\_Mf\_liverA, CL4033.Contig1\_Mf\_liverA, Unigene34983\_Mf\_liverA, Unigene28662\_Mf\_liverA, CL2439.Contig1\_Mf\_liverA, CL3519.Contig1\_Mf\_liverA, Unigene33330\_Mf\_liverA, Unigene6464\_Mf\_liverA, Unigene29426\_Mf\_liverA, CL3933.Contig1\_Mf\_liverA, Unigene21026\_Mf\_liverA, NM\_011221, Unigene20371\_Mf\_liverA, CL1325.Contig1\_Mf\_liverA, CL1493.Contig1\_Mf\_liverA, Unigene13683\_Mf\_liverA, Unigene13009\_Mf\_liverA, Unigene9698\_Mf\_liverA, Unigene36420\_Mf\_liverA, Unigene30004\_Mf\_liverA, Unigene542\_Mf\_liverA, Unigene35476\_Mf\_liverA, Unigene24758\_Mf\_liverA, Unigene24471\_Mf\_liverA, Unigene31571\_Mf\_liverA, CL5828.Contig2\_Mf\_liverA, Unigene35168\_Mf\_liverA, Unigene33080\_Mf\_liverA, Unigene15888\_Mf\_liverA, CL4220.Contig1\_Mf\_liverA, Unigene15433\_Mf\_liverA, Unigene37262\_Mf\_liverA, CL4338.Contig1\_Mf\_liverA, Unigene12908\_Mf\_liverA, CL5052.Contig1\_Mf\_liverA, Unigene26251\_Mf\_liverA, NM\_019817, NM\_010469, Unigene5750\_Mf\_liverA, NM\_019879, NR\_033215, NM\_007822, CL5293.Contig1\_Mf\_liverA, Unigene25595\_Mf\_liverA, Unigene14263\_Mf\_liverA, Unigene15470\_Mf\_liverA, Unigene14286\_Mf\_liverA, Unigene32434\_Mf\_liverA, CL4816.Contig3\_Mf\_liverA, Unigene34962\_Mf\_liverA, CL114.Contig1\_Mf\_liverA, Unigene39011\_Mf\_liverA, Unigene24225\_Mf\_liverA, Unigene37344\_Mf\_liverA, Unigene8218\_Mf\_liverA, CL1588.Contig2\_Mf\_liverA, CL5275.Contig2\_Mf\_liverA, NM\_011207, NM\_145218, Unigene36189\_Mf\_liverA, NM\_008183, Unigene29558\_Mf\_liverA, Unigene13950\_Mf\_liverA, Unigene28459\_Mf\_liverA, Unigene37245\_Mf\_liverA, Unigene32133\_Mf\_liverA, Unigene281\_Mf\_liverA, NM\_177652, NM\_011978, Unigene10313\_Mf\_liverA, Unigene20372\_Mf\_liverA, Unigene37104\_Mf\_liverA, CL3104.Contig1\_Mf\_liverA, Unigene13018\_Mf\_liverA, Unigene36593\_Mf\_liverA, NM\_010002, NM\_013842, NM\_027406, Unigene36176\_Mf\_liverA, Unigene16529\_Mf\_liverA, Unigene37232\_Mf\_liverA, NM\_026313, Unigene28609\_Mf\_liverA, CL5007.Contig1\_Mf\_liverA, CL5698.Contig1\_Mf\_liverA, Unigene14461\_Mf\_liverA, Unigene31392\_Mf\_liverA, CL591.Contig1\_Mf\_liverA, Unigene29889\_Mf\_liverA, Unigene2939\_Mf\_liverA, Unigene35618\_Mf\_liverA, NM\_011256, Unigene35884\_Mf\_liverA, CL5640.Contig1\_Mf\_liverA, Unigene38015\_Mf\_liverA, NM\_028291, NM\_173038, NM\_206537, CL3835.Contig2\_Mf\_liverA, Unigene31199\_Mf\_liverA, Unigene6959\_Mf\_liverA, Unigene28899\_Mf\_liverA, CL4550.Contig2\_Mf\_liverA, Unigene28244\_Mf\_liverA, Unigene46870\_Mf\_liverA, NM\_019651, CL523.Contig1\_Mf\_liverA, NM\_008277, Unigene8473\_Mf\_liverA, CL482.Contig1\_Mf\_liverA, Unigene27071\_Mf\_liverA, Unigene36762\_Mf\_liverA, Unigene35382\_Mf\_liverA, Unigene600\_Mf\_liverA, NM\_011072, Unigene18795\_Mf\_liverA, Unigene28564\_Mf\_liverA, Unigene30878\_Mf\_liverA, Unigene40289\_Mf\_liverA, NM\_173753, NM\_134012, CL6038.Contig2\_Mf\_liverA, Unigene32570\_Mf\_liverA, Unigene37904\_Mf\_liverA, Unigene5333\_Mf\_liverA, CL3483.Contig1\_Mf\_liverA, Unigene15295\_Mf\_liverA, Unigene34789\_Mf\_liverA, Unigene12999\_Mf\_liverA, CL4141.Contig1\_Mf\_liverA, Unigene24221\_Mf\_liverA, NM\_145419, Unigene34184\_Mf\_liverA, CL5978.Contig3\_Mf\_liverA, CL1119.Contig1\_Mf\_liverA, Unigene13658\_Mf\_liverA, Unigene13593\_Mf\_liverA, NM\_134127, Unigene32546\_Mf\_liverA, Unigene11\_Mf\_liverA, CL2622.Contig2\_Mf\_liverA, Unigene14609\_Mf\_liverA, Unigene843\_Mf\_liverA, Unigene25596\_Mf\_liverA, Unigene37109\_Mf\_liverA, CL3778.Contig2\_Mf\_liverA, Unigene12997\_Mf\_liverA, Unigene5994\_Mf\_liverA, CL1493.Contig2\_Mf\_liverA, CL2697.Contig4\_Mf\_liverA, NM\_008261, NM\_024434, CL2574.Contig1\_Mf\_liverA, Unigene5941\_Mf\_liverA, Unigene38104\_Mf\_liverA, Unigene23328\_Mf\_liverA, Unigene36626\_Mf\_liverA, CL787.Contig1\_Mf\_liverA, CL4076.Contig1\_Mf\_liverA, Unigene29490\_Mf\_liverA, CL3897.Contig5\_Mf\_liverA, Unigene12967\_Mf\_liverA, Unigene36691\_Mf\_liverA, NM\_001162917, Unigene31988\_Mf\_liverA, CL2389.Contig1\_Mf\_liverA, CL3750.Contig2\_Mf\_liverA, CL335.Contig1\_Mf\_liverA, CL3599.Contig1\_Mf\_liverA, Unigene151\_Mf\_liverA, Unigene39141\_Mf\_liverA, CL848.Contig2\_Mf\_liverA, Unigene31639\_Mf\_liverA, CL784.Contig3\_Mf\_liverA, CL838.Contig3\_Mf\_liverA, Unigene10774\_Mf\_liverA, Unigene28520\_Mf\_liverA, Unigene5632\_Mf\_liverA, CL1263.Contig1\_Mf\_liverA, CL4550.Contig3\_Mf\_liverA, Unigene21816\_Mf\_liverA, CL258.Contig1\_Mf\_liverA, Unigene27419\_Mf\_liverA, NM\_007811, Unigene24323\_Mf\_liverA, NM\_008538, CL2142.Contig2\_Mf\_liverA, Unigene30428\_Mf\_liverA, NM\_011305, Unigene21741\_Mf\_liverA, Unigene21742\_Mf\_liverA, CL153.Contig2\_Mf\_liverA, Unigene13498\_Mf\_liverA, Unigene31427\_Mf\_liverA, CL529.Contig2\_Mf\_liverA, Unigene40069\_Mf\_liverA, Unigene30584\_Mf\_liverA, Unigene4510\_Mf\_liverA, Unigene17579\_Mf\_liverA, NM\_001081372, Unigene37148\_Mf\_liverA, CL913.Contig1\_Mf\_liverA, Unigene34406\_Mf\_liverA, NM\_021525, Unigene7195\_Mf\_liverA, Unigene35506\_Mf\_liverA, Unigene32058\_Mf\_liverA, Unigene27082\_Mf\_liverA, Unigene37178\_Mf\_liverA, Unigene5712\_Mf\_liverA, CL2919.Contig1\_Mf\_liverA, Unigene1205\_Mf\_liverA, Unigene36772\_Mf\_liverA, CL442.Contig2\_Mf\_liverA, CL6039.Contig1\_Mf\_liverA, CL2855.Contig1\_Mf\_liverA, CL4411.Contig4\_Mf\_liverA, NM\_013821, Unigene40796\_Mf\_liverA, Unigene33420\_Mf\_liverA, Unigene32412\_Mf\_liverA, Unigene17569\_Mf\_liverA, NM\_001100182, Unigene31051\_Mf\_liverA, NM\_025706, Unigene392\_Mf\_liverA, NM\_011732, Unigene30426\_Mf\_liverA, NM\_009178, Unigene49971\_Mf\_liverA, Unigene38307\_Mf\_liverA, Unigene36190\_Mf\_liverA, Unigene27406\_Mf\_liverA, Unigene34754\_Mf\_liverA, Unigene17042\_Mf\_liverA, Unigene34811\_Mf\_liverA, Unigene30154\_Mf\_liverA, Unigene3789\_Mf\_liverA, Unigene33746\_Mf\_liverA, CL1125.Contig1\_Mf\_liverA, CL4162.Contig1\_Mf\_liverA, Unigene1000\_Mf\_liverA, Unigene27922\_Mf\_liverA, Unigene15681\_Mf\_liverA, Unigene673\_Mf\_liverA, Unigene15476\_Mf\_liverA, CL1100.Contig1\_Mf\_liverA, Unigene32093\_Mf\_liverA, CL4583.Contig2\_Mf\_liverA, Unigene36414\_Mf\_liverA, NM\_021423, CL485.Contig1\_Mf\_liverA, CL1988.Contig1\_Mf\_liverA, Unigene13894\_Mf\_liverA, Unigene5175\_Mf\_liverA, Unigene36328\_Mf\_liverA, Unigene15552\_Mf\_liverA, Unigene38280\_Mf\_liverA, CL4745.Contig1\_Mf\_liverA, CL5309.Contig1\_Mf\_liverA, CL3002.Contig1\_Mf\_liverA, Unigene31483\_Mf\_liverA, Unigene36315\_Mf\_liverA, Unigene31267\_Mf\_liverA, CL2327.Contig1\_Mf\_liverA, Unigene36949\_Mf\_liverA, Unigene5488\_Mf\_liverA, CL887.Contig2\_Mf\_liverA, CL2597.Contig1\_Mf\_liverA, Unigene33736\_Mf\_liverA, Unigene33523\_Mf\_liverA, Unigene35037\_Mf\_liverA, Unigene14810\_Mf\_liverA, CL3684.Contig2\_Mf\_liverA, CL1656.Contig1\_Mf\_liverA, Unigene1212\_Mf\_liverA, NM\_001100181, Unigene32233\_Mf\_liverA, CL838.Contig6\_Mf\_liverA, Unigene5607\_Mf\_liverA, CL4770.Contig1\_Mf\_liverA, Unigene32143\_Mf\_liverA, Unigene24536\_Mf\_liverA, CL3750.Contig1\_Mf\_liverA, Unigene13196\_Mf\_liverA, CL114.Contig2\_Mf\_liverA, Unigene14449\_Mf\_liverA, CL1076.Contig1\_Mf\_liverA, Unigene39520\_Mf\_liverA, Unigene601\_Mf\_liverA, NM\_009883, Unigene18796\_Mf\_liverA, Unigene22423\_Mf\_liverA, NM\_009448, CL5307.Contig1\_Mf\_liverA, Unigene1327\_Mf\_liverA, Unigene31670\_Mf\_liverA, CL5978.Contig2\_Mf\_liverA, NM\_178405, NM\_172203, CL442.Contig5\_Mf\_liverA, Unigene120\_Mf\_liverA, Unigene36418\_Mf\_liverA, CL4007.Contig1\_Mf\_liverA, Unigene15026\_Mf\_liverA, NM\_022997, NM\_001081116, Unigene550\_Mf\_liverA, NM\_017379, CL55.Contig1\_Mf\_liverA, Unigene28489\_Mf\_liverA, Unigene37120\_Mf\_liverA, Unigene31198\_Mf\_liverA, CL2355.Contig1\_Mf\_liverA, Unigene4863\_Mf\_liverA, Unigene24537\_Mf\_liverA, NM\_001081274, CL4984.Contig1\_Mf\_liverA, Unigene32335\_Mf\_liverA, Unigene37153\_Mf\_liverA, Unigene7224\_Mf\_liverA, Unigene14809\_Mf\_liverA, NM\_027604, Unigene5489\_Mf\_liverA, Unigene34444\_Mf\_liverA, Unigene7399\_Mf\_liverA, Unigene35678\_Mf\_liverA, Unigene33993\_Mf\_liverA, Unigene138\_Mf\_liverA, NM\_009011, Unigene8560\_Mf\_liverA, CL3911.Contig2\_Mf\_liverA, Unigene44057\_Mf\_liverA, Unigene5761\_Mf\_liverA, Unigene25597\_Mf\_liverA, Unigene4681\_Mf\_liverA, Unigene12519\_Mf\_liverA, NM\_008851, Unigene37819\_Mf\_liverA, Unigene5886\_Mf\_liverA, CL336.Contig3\_Mf\_liverA, CL1198.Contig1\_Mf\_liverA |
| Golgi apparatus part | CL787.Contig1\_Mf\_liverA, Unigene34032\_Mf\_liverA, Unigene15055\_Mf\_liverA, Unigene31051\_Mf\_liverA, Unigene36328\_Mf\_liverA, Unigene281\_Mf\_liverA, CL2855.Contig2\_Mf\_liverA, Unigene32335\_Mf\_liverA, Unigene37389\_Mf\_liverA, Unigene29082\_Mf\_liverA, NM\_009178, CL4033.Contig1\_Mf\_liverA, NM\_019817, Unigene16529\_Mf\_liverA, Unigene5632\_Mf\_liverA, Unigene27082\_Mf\_liverA, CL5007.Contig1\_Mf\_liverA, CL4600.Contig1\_Mf\_liverA, NM\_008851, CL2855.Contig1\_Mf\_liverA, CL5275.Contig2\_Mf\_liverA, NM\_024434, Unigene32920\_Mf\_liverA |
| lamellipodium | CL4228.Contig1\_Mf\_liverA, Unigene39011\_Mf\_liverA, Unigene21684\_Mf\_liverA, Unigene22423\_Mf\_liverA, Unigene5693\_Mf\_liverA |
| intermediate filament | Unigene34789\_Mf\_liverA, Unigene27082\_Mf\_liverA, CL4162.Contig1\_Mf\_liverA, Unigene5941\_Mf\_liverA |
| cell projection | Unigene21684\_Mf\_liverA, Unigene13363\_Mf\_liverA, NM\_011732, Unigene36417\_Mf\_liverA, Unigene42606\_Mf\_liverA, CL4228.Contig1\_Mf\_liverA, Unigene22423\_Mf\_liverA, CL425.Contig1\_Mf\_liverA, Unigene49971\_Mf\_liverA, Unigene28244\_Mf\_liverA, Unigene24883\_Mf\_liverA, Unigene5750\_Mf\_liverA, Unigene4697\_Mf\_liverA, NM\_178405, Unigene33746\_Mf\_liverA, Unigene36698\_Mf\_liverA, NM\_172203, Unigene36762\_Mf\_liverA, Unigene2050\_Mf\_liverA, NM\_011305, Unigene16184\_Mf\_liverA, Unigene39011\_Mf\_liverA, Unigene28564\_Mf\_liverA, Unigene5693\_Mf\_liverA, Unigene36418\_Mf\_liverA, Unigene13950\_Mf\_liverA, Unigene5057\_Mf\_liverA, Unigene34032\_Mf\_liverA, CL4583.Contig2\_Mf\_liverA, Unigene36414\_Mf\_liverA, Unigene18500\_Mf\_liverA, Unigene27547\_Mf\_liverA, NM\_021423, Unigene32570\_Mf\_liverA, Unigene5175\_Mf\_liverA, Unigene32803\_Mf\_liverA, Unigene36328\_Mf\_liverA, NM\_010141, Unigene36898\_Mf\_liverA, NM\_027660, CL1810.Contig1\_Mf\_liverA, Unigene34789\_Mf\_liverA, Unigene13593\_Mf\_liverA, Unigene16529\_Mf\_liverA, Unigene33330\_Mf\_liverA, Unigene26611\_Mf\_liverA, Unigene14609\_Mf\_liverA, Unigene5712\_Mf\_liverA, NM\_001004357, Unigene14461\_Mf\_liverA, CL1493.Contig2\_Mf\_liverA, Unigene1288\_Mf\_liverA, CL3166.Contig4\_Mf\_liverA, CL2697.Contig4\_Mf\_liverA, CL1493.Contig1\_Mf\_liverA, Unigene36420\_Mf\_liverA |
| Golgi lumen | Unigene34032\_Mf\_liverA, Unigene29082\_Mf\_liverA |
| perikaryon | Unigene28564\_Mf\_liverA, CL2697.Contig4\_Mf\_liverA |
| synapse part | Unigene26611\_Mf\_liverA, CL3154.Contig1\_Mf\_liverA, Unigene34394\_Mf\_liverA, Unigene32570\_Mf\_liverA, CL5807.Contig1\_Mf\_liverA, Unigene15470\_Mf\_liverA, Unigene13363\_Mf\_liverA, Unigene24883\_Mf\_liverA, Unigene13593\_Mf\_liverA, Unigene16529\_Mf\_liverA |
| neuromuscular junction | CL3154.Contig1\_Mf\_liverA, Unigene13950\_Mf\_liverA |
| intercalated disc | Unigene27082\_Mf\_liverA, Unigene13950\_Mf\_liverA |
| coated vesicle | Unigene34394\_Mf\_liverA, CL5807.Contig1\_Mf\_liverA, NM\_031165, Unigene15470\_Mf\_liverA, Unigene13363\_Mf\_liverA, CL2855.Contig2\_Mf\_liverA, Unigene32335\_Mf\_liverA, CL2855.Contig1\_Mf\_liverA, NM\_019817, Unigene30584\_Mf\_liverA, Unigene5632\_Mf\_liverA |
| clathrin-coated vesicle | Unigene34394\_Mf\_liverA, CL5807.Contig1\_Mf\_liverA, NM\_031165, Unigene15470\_Mf\_liverA, Unigene13363\_Mf\_liverA, CL2855.Contig2\_Mf\_liverA, Unigene32335\_Mf\_liverA, CL2855.Contig1\_Mf\_liverA, Unigene30584\_Mf\_liverA |
| brush border | Unigene5750\_Mf\_liverA, Unigene36898\_Mf\_liverA, Unigene13363\_Mf\_liverA, Unigene42606\_Mf\_liverA |
| synaptic vesicle | Unigene34394\_Mf\_liverA, CL5807.Contig1\_Mf\_liverA, Unigene15470\_Mf\_liverA, Unigene13363\_Mf\_liverA |
| filopodium | Unigene39011\_Mf\_liverA, Unigene21684\_Mf\_liverA, Unigene13950\_Mf\_liverA |
| sarcoplasm | Unigene30832\_Mf\_liverA, NM\_177652 |
| triglyceride-rich lipoprotein particle | CL3911.Contig2\_Mf\_liverA, Unigene13498\_Mf\_liverA |
| transport vesicle membrane | CL2855.Contig1\_Mf\_liverA, CL2855.Contig2\_Mf\_liverA, CL2697.Contig4\_Mf\_liverA |
| cell projection part | Unigene5057\_Mf\_liverA, Unigene18500\_Mf\_liverA, Unigene27547\_Mf\_liverA, Unigene32570\_Mf\_liverA, Unigene36898\_Mf\_liverA, Unigene13363\_Mf\_liverA, NM\_027660, Unigene42606\_Mf\_liverA, CL4228.Contig1\_Mf\_liverA, CL425.Contig1\_Mf\_liverA, Unigene49971\_Mf\_liverA, Unigene28244\_Mf\_liverA, Unigene24883\_Mf\_liverA, Unigene13593\_Mf\_liverA, Unigene16529\_Mf\_liverA, Unigene33330\_Mf\_liverA, Unigene26611\_Mf\_liverA, NM\_172203, NM\_001004357, Unigene5693\_Mf\_liverA, Unigene13950\_Mf\_liverA |
| basal plasma membrane | Unigene32803\_Mf\_liverA, Unigene36898\_Mf\_liverA |
| brush border membrane | Unigene36898\_Mf\_liverA, Unigene42606\_Mf\_liverA |
| postsynaptic density | Unigene26611\_Mf\_liverA, Unigene32570\_Mf\_liverA, Unigene13593\_Mf\_liverA, Unigene16529\_Mf\_liverA |
| dendritic spine head | Unigene26611\_Mf\_liverA, Unigene32570\_Mf\_liverA, Unigene13593\_Mf\_liverA, Unigene16529\_Mf\_liverA |
| cell-cell contact zone | Unigene27082\_Mf\_liverA, Unigene13950\_Mf\_liverA |
| centriole | Unigene5057\_Mf\_liverA, Unigene673\_Mf\_liverA |
| transport vesicle | CL2855.Contig1\_Mf\_liverA, Unigene37819\_Mf\_liverA, Unigene32093\_Mf\_liverA, CL2855.Contig2\_Mf\_liverA, CL2697.Contig4\_Mf\_liverA |
| internal side of plasma membrane | CL838.Contig6\_Mf\_liverA, Unigene843\_Mf\_liverA, Unigene33080\_Mf\_liverA, CL838.Contig3\_Mf\_liverA, Unigene19687\_Mf\_liverA |
| midbody | Unigene5057\_Mf\_liverA, Unigene5693\_Mf\_liverA, Unigene1479\_Mf\_liverA |
| peroxisome | Unigene37104\_Mf\_liverA, CL1125.Contig1\_Mf\_liverA, CL3382.Contig1\_Mf\_liverA, Unigene743\_Mf\_liverA, Unigene35678\_Mf\_liverA, NM\_011978 |
| ribonucleoprotein granule | Unigene33330\_Mf\_liverA, CL4745.Contig1\_Mf\_liverA, Unigene13683\_Mf\_liverA |
| platelet alpha granule lumen | CL6038.Contig2\_Mf\_liverA, Unigene28662\_Mf\_liverA |
| cortical actin cytoskeleton | Unigene26611\_Mf\_liverA, CL848.Contig2\_Mf\_liverA |
| secretory granule lumen | CL6038.Contig2\_Mf\_liverA, Unigene28662\_Mf\_liverA |
| synapse | Unigene18500\_Mf\_liverA, Unigene26611\_Mf\_liverA, CL3154.Contig1\_Mf\_liverA, Unigene34394\_Mf\_liverA, Unigene32570\_Mf\_liverA, CL5807.Contig1\_Mf\_liverA, NM\_010141, Unigene27922\_Mf\_liverA, Unigene15470\_Mf\_liverA, Unigene13363\_Mf\_liverA, Unigene24883\_Mf\_liverA, Unigene13593\_Mf\_liverA, Unigene13950\_Mf\_liverA, Unigene16529\_Mf\_liverA |
| cell-cell junction | Unigene25055\_Mf\_liverA, Unigene32546\_Mf\_liverA, Unigene21684\_Mf\_liverA, CL4302.Contig1\_Mf\_liverA, Unigene27082\_Mf\_liverA, Unigene44057\_Mf\_liverA, Unigene32803\_Mf\_liverA, Unigene28853\_Mf\_liverA, Unigene28852\_Mf\_liverA, Unigene39011\_Mf\_liverA, Unigene5693\_Mf\_liverA, CL4033.Contig1\_Mf\_liverA, Unigene25052\_Mf\_liverA, Unigene13950\_Mf\_liverA |
| actin filament | Unigene26611\_Mf\_liverA, Unigene32803\_Mf\_liverA, Unigene2050\_Mf\_liverA, CL5698.Contig1\_Mf\_liverA |
| endoplasmic reticulum-Golgi intermediate compartment | Unigene1479\_Mf\_liverA, Unigene5632\_Mf\_liverA |
| mitochondrial intermembrane space | Unigene35476\_Mf\_liverA, CL2797.Contig2\_Mf\_liverA |
| Golgi-associated vesicle membrane | Unigene27082\_Mf\_liverA, Unigene5632\_Mf\_liverA |
| neuron projection membrane | Unigene24883\_Mf\_liverA, NM\_001004357 |
| Golgi-associated vesicle | Unigene27082\_Mf\_liverA, NM\_019817, Unigene5632\_Mf\_liverA |
| nuclear pore | Unigene15433\_Mf\_liverA, Unigene21816\_Mf\_liverA |
| cell-substrate adherens junction | CL4228.Contig1\_Mf\_liverA, NM\_026313, Unigene22423\_Mf\_liverA, Unigene32803\_Mf\_liverA, CL5007.Contig1\_Mf\_liverA, Unigene15572\_Mf\_liverA, CL838.Contig3\_Mf\_liverA |
| sarcolemma | Unigene27547\_Mf\_liverA, CL425.Contig1\_Mf\_liverA, Unigene36898\_Mf\_liverA, CL887.Contig2\_Mf\_liverA |
| DNA-directed RNA polymerase II, holoenzyme | NM\_025593, NM\_176843, CL4249.Contig1\_Mf\_liverA |
| intermediate filament cytoskeleton | Unigene34789\_Mf\_liverA, Unigene27082\_Mf\_liverA, CL4162.Contig1\_Mf\_liverA, Unigene5941\_Mf\_liverA |
| lipid particle | CL55.Contig1\_Mf\_liverA, Unigene25596\_Mf\_liverA |
| organelle envelope lumen | Unigene35476\_Mf\_liverA, CL2797.Contig2\_Mf\_liverA |
| recycling endosome | Unigene28244\_Mf\_liverA, CL497.Contig2\_Mf\_liverA |
| cell-substrate junction | CL4228.Contig1\_Mf\_liverA, NM\_026313, Unigene22423\_Mf\_liverA, Unigene32803\_Mf\_liverA, CL5007.Contig1\_Mf\_liverA, Unigene15572\_Mf\_liverA, CL838.Contig3\_Mf\_liverA |
| cell junction | Unigene21684\_Mf\_liverA, CL4302.Contig1\_Mf\_liverA, CL5807.Contig1\_Mf\_liverA, Unigene32803\_Mf\_liverA, Unigene32789\_Mf\_liverA, CL4228.Contig1\_Mf\_liverA, Unigene22423\_Mf\_liverA, CL4033.Contig1\_Mf\_liverA, Unigene49971\_Mf\_liverA, CL838.Contig3\_Mf\_liverA, Unigene13593\_Mf\_liverA, Unigene25055\_Mf\_liverA, Unigene32546\_Mf\_liverA, NM\_026313, Unigene34394\_Mf\_liverA, Unigene27082\_Mf\_liverA, Unigene44057\_Mf\_liverA, CL5007.Contig1\_Mf\_liverA, Unigene28853\_Mf\_liverA, Unigene28852\_Mf\_liverA, Unigene39011\_Mf\_liverA, Unigene5693\_Mf\_liverA, Unigene15572\_Mf\_liverA, Unigene25052\_Mf\_liverA, Unigene13950\_Mf\_liverA, NM\_016689 |
| coated pit | Unigene37232\_Mf\_liverA, Unigene32335\_Mf\_liverA |
| leading edge membrane | CL4228.Contig1\_Mf\_liverA, Unigene5693\_Mf\_liverA, Unigene24883\_Mf\_liverA, NM\_001004357 |
| tight junction | CL4302.Contig1\_Mf\_liverA, Unigene44057\_Mf\_liverA, Unigene13950\_Mf\_liverA |
| membrane coat | NM\_019817, Unigene32335\_Mf\_liverA, Unigene5632\_Mf\_liverA |
| coated membrane | NM\_019817, Unigene32335\_Mf\_liverA, Unigene5632\_Mf\_liverA |
| occluding junction | CL4302.Contig1\_Mf\_liverA, Unigene44057\_Mf\_liverA, Unigene13950\_Mf\_liverA |
| DNA-directed RNA polymerase complex | NM\_025593, NM\_176843, CL4249.Contig1\_Mf\_liverA |
| nuclear DNA-directed RNA polymerase complex | NM\_025593, NM\_176843, CL4249.Contig1\_Mf\_liverA |
| adherens junction | NM\_026313, Unigene27082\_Mf\_liverA, Unigene32803\_Mf\_liverA, CL5007.Contig1\_Mf\_liverA, CL4228.Contig1\_Mf\_liverA, Unigene22423\_Mf\_liverA, Unigene15572\_Mf\_liverA, CL838.Contig3\_Mf\_liverA, Unigene13950\_Mf\_liverA |
| actomyosin | Unigene32546\_Mf\_liverA, Unigene32803\_Mf\_liverA |
| myosin complex | CL4302.Contig1\_Mf\_liverA, CL591.Contig1\_Mf\_liverA |
| cell division site | NM\_008851, Unigene5693\_Mf\_liverA |
| cell division site part | NM\_008851, Unigene5693\_Mf\_liverA |
| microbody | Unigene37104\_Mf\_liverA, CL1125.Contig1\_Mf\_liverA, CL3382.Contig1\_Mf\_liverA, Unigene743\_Mf\_liverA, Unigene35678\_Mf\_liverA, NM\_011978 |
| cortical cytoskeleton | Unigene26611\_Mf\_liverA, CL848.Contig2\_Mf\_liverA |
| RNA polymerase complex | NM\_025593, NM\_176843, CL4249.Contig1\_Mf\_liverA |
| ruffle | CL4228.Contig1\_Mf\_liverA, Unigene5693\_Mf\_liverA, Unigene5175\_Mf\_liverA, Unigene2050\_Mf\_liverA, Unigene4697\_Mf\_liverA |
| mitochondrial matrix | CL3599.Contig1\_Mf\_liverA, Unigene14916\_Mf\_liverA, CL1125.Contig1\_Mf\_liverA, CL532.Contig1\_Mf\_liverA, CL5052.Contig1\_Mf\_liverA, NM\_019879, Unigene36691\_Mf\_liverA |
| ruffle membrane | CL4228.Contig1\_Mf\_liverA, Unigene5693\_Mf\_liverA |
| synaptic membrane | CL3154.Contig1\_Mf\_liverA, Unigene24883\_Mf\_liverA, Unigene13593\_Mf\_liverA |
| anchoring junction | NM\_026313, Unigene27082\_Mf\_liverA, Unigene32803\_Mf\_liverA, CL5007.Contig1\_Mf\_liverA, CL4228.Contig1\_Mf\_liverA, Unigene22423\_Mf\_liverA, Unigene15572\_Mf\_liverA, CL838.Contig3\_Mf\_liverA, Unigene13950\_Mf\_liverA |
| nuclear matrix | CL4162.Contig1\_Mf\_liverA, Unigene21816\_Mf\_liverA |
| microtubule organizing center part | Unigene5057\_Mf\_liverA, Unigene673\_Mf\_liverA |
| ion channel complex | Unigene18500\_Mf\_liverA, Unigene37526\_Mf\_liverA, NM\_153589 |
| protein-DNA complex | CL2439.Contig1\_Mf\_liverA, Unigene31392\_Mf\_liverA |
| mitochondrial outer membrane | CL1810.Contig1\_Mf\_liverA, CL3104.Contig1\_Mf\_liverA, Unigene27082\_Mf\_liverA |
| nuclear membrane | Unigene15433\_Mf\_liverA, Unigene21816\_Mf\_liverA, Unigene36898\_Mf\_liverA, Unigene32335\_Mf\_liverA |
| cell cortex | Unigene28899\_Mf\_liverA, Unigene26611\_Mf\_liverA, Unigene5693\_Mf\_liverA, CL848.Contig2\_Mf\_liverA, Unigene15290\_Mf\_liverA |
| PML body | Unigene5057\_Mf\_liverA, CL2355.Contig1\_Mf\_liverA |
| actin cytoskeleton | Unigene32546\_Mf\_liverA, Unigene5639\_Mf\_liverA, Unigene26611\_Mf\_liverA, CL4302.Contig1\_Mf\_liverA, Unigene44057\_Mf\_liverA, Unigene32803\_Mf\_liverA, Unigene2050\_Mf\_liverA, CL5698.Contig1\_Mf\_liverA, CL591.Contig1\_Mf\_liverA, CL848.Contig2\_Mf\_liverA, Unigene31625\_Mf\_liverA, Unigene31623\_Mf\_liverA |
| Golgi stack | CL5275.Contig2\_Mf\_liverA, Unigene5632\_Mf\_liverA |
| extrinsic to plasma membrane | Unigene37243\_Mf\_liverA, Unigene35958\_Mf\_liverA |
| postsynaptic membrane | CL3154.Contig1\_Mf\_liverA, Unigene13593\_Mf\_liverA |
| cell leading edge | Unigene32546\_Mf\_liverA, Unigene21684\_Mf\_liverA, Unigene5175\_Mf\_liverA, Unigene2050\_Mf\_liverA, NM\_001004357, CL4228.Contig1\_Mf\_liverA, Unigene39011\_Mf\_liverA, Unigene22423\_Mf\_liverA, Unigene5693\_Mf\_liverA, Unigene24883\_Mf\_liverA, Unigene4697\_Mf\_liverA |
| ubiquitin ligase complex | Unigene33054\_Mf\_liverA, Unigene8218\_Mf\_liverA, Unigene843\_Mf\_liverA, Unigene13683\_Mf\_liverA |
| axon part | Unigene24883\_Mf\_liverA, NM\_001004357, Unigene36898\_Mf\_liverA, Unigene16529\_Mf\_liverA |
| growth cone | Unigene13950\_Mf\_liverA, Unigene16529\_Mf\_liverA |
| actin filament bundle | Unigene44057\_Mf\_liverA, Unigene32803\_Mf\_liverA |
| cell cortex part | Unigene26611\_Mf\_liverA, CL848.Contig2\_Mf\_liverA |
| cullin-RING ubiquitin ligase complex | Unigene33054\_Mf\_liverA, Unigene13683\_Mf\_liverA |
| organelle outer membrane | CL1810.Contig1\_Mf\_liverA, CL3104.Contig1\_Mf\_liverA, Unigene27082\_Mf\_liverA |
| nuclear periphery | CL4162.Contig1\_Mf\_liverA, Unigene21816\_Mf\_liverA, NM\_009883 |
| apical junction complex | CL4302.Contig1\_Mf\_liverA, Unigene5693\_Mf\_liverA, Unigene44057\_Mf\_liverA, Unigene13950\_Mf\_liverA |
| outer membrane | CL1810.Contig1\_Mf\_liverA, CL3104.Contig1\_Mf\_liverA, Unigene27082\_Mf\_liverA |
| apicolateral plasma membrane | CL4302.Contig1\_Mf\_liverA, Unigene5693\_Mf\_liverA, Unigene44057\_Mf\_liverA, Unigene13950\_Mf\_liverA |
| nuclear envelope | CL1263.Contig1\_Mf\_liverA, CL4162.Contig1\_Mf\_liverA, Unigene21816\_Mf\_liverA, Unigene36898\_Mf\_liverA, Unigene32335\_Mf\_liverA, Unigene29011\_Mf\_liverA, Unigene15433\_Mf\_liverA, NM\_080638 |
| histone acetyltransferase complex | Unigene1479\_Mf\_liverA, Unigene30288\_Mf\_liverA |
| microbody part | CL3382.Contig1\_Mf\_liverA, NM\_011978 |
| peroxisomal part | CL3382.Contig1\_Mf\_liverA, NM\_011978 |
| contractile fiber | Unigene5639\_Mf\_liverA, Unigene26611\_Mf\_liverA, Unigene27082\_Mf\_liverA, Unigene32789\_Mf\_liverA, Unigene13593\_Mf\_liverA |
| nucleolus | Unigene29876\_Mf\_liverA, Unigene24758\_Mf\_liverA, Unigene36414\_Mf\_liverA, Unigene14449\_Mf\_liverA, Unigene24537\_Mf\_liverA, Unigene30426\_Mf\_liverA, Unigene36417\_Mf\_liverA, Unigene10313\_Mf\_liverA, CL1810.Contig1\_Mf\_liverA, Unigene28899\_Mf\_liverA, CL4338.Contig1\_Mf\_liverA, Unigene31639\_Mf\_liverA, Unigene1479\_Mf\_liverA, Unigene7399\_Mf\_liverA, Unigene24613\_Mf\_liverA, Unigene14916\_Mf\_liverA, Unigene20874\_Mf\_liverA, Unigene37109\_Mf\_liverA, Unigene30428\_Mf\_liverA, Unigene31392\_Mf\_liverA, Unigene13683\_Mf\_liverA, Unigene32233\_Mf\_liverA, Unigene9698\_Mf\_liverA, Unigene36420\_Mf\_liverA, Unigene36418\_Mf\_liverA, Unigene24536\_Mf\_liverA, Unigene32093\_Mf\_liverA, Unigene802\_Mf\_liverA, Unigene35958\_Mf\_liverA |
| intracellular organelle part | NM\_176843, Unigene35884\_Mf\_liverA, CL4302.Contig1\_Mf\_liverA, Unigene38015\_Mf\_liverA, NM\_028291, CL3835.Contig2\_Mf\_liverA, Unigene29011\_Mf\_liverA, NM\_025593, Unigene28899\_Mf\_liverA, CL4550.Contig2\_Mf\_liverA, Unigene36851\_Mf\_liverA, NM\_080638, CL523.Contig1\_Mf\_liverA, NM\_011082, CL4105.Contig1\_Mf\_liverA, Unigene7336\_Mf\_liverA, CL482.Contig1\_Mf\_liverA, Unigene35507\_Mf\_liverA, Unigene600\_Mf\_liverA, Unigene39886\_Mf\_liverA, Unigene15064\_Mf\_liverA, Unigene30878\_Mf\_liverA, Unigene32920\_Mf\_liverA, Unigene34124\_Mf\_liverA, CL6038.Contig2\_Mf\_liverA, Unigene32570\_Mf\_liverA, Unigene34341\_Mf\_liverA, Unigene15295\_Mf\_liverA, Unigene34789\_Mf\_liverA, CL695.Contig1\_Mf\_liverA, Unigene12999\_Mf\_liverA, Unigene29082\_Mf\_liverA, CL4141.Contig1\_Mf\_liverA, NM\_145419, Unigene2\_Mf\_liverA, CL1119.Contig1\_Mf\_liverA, Unigene13658\_Mf\_liverA, Unigene13593\_Mf\_liverA, Unigene32546\_Mf\_liverA, Unigene20874\_Mf\_liverA, Unigene34394\_Mf\_liverA, Unigene25596\_Mf\_liverA, Unigene37109\_Mf\_liverA, NM\_031165, CL497.Contig2\_Mf\_liverA, CL1493.Contig2\_Mf\_liverA, CL6.Contig1\_Mf\_liverA, CL2697.Contig4\_Mf\_liverA, NM\_008261, NM\_024434, CL2574.Contig1\_Mf\_liverA, Unigene5941\_Mf\_liverA, Unigene23328\_Mf\_liverA, Unigene802\_Mf\_liverA, CL4816.Contig1\_Mf\_liverA, CL787.Contig1\_Mf\_liverA, Unigene15055\_Mf\_liverA, Unigene5639\_Mf\_liverA, CL5807.Contig1\_Mf\_liverA, Unigene29490\_Mf\_liverA, Unigene32594\_Mf\_liverA, Unigene36417\_Mf\_liverA, CL2855.Contig2\_Mf\_liverA, Unigene12967\_Mf\_liverA, Unigene36691\_Mf\_liverA, CL4263.Contig1\_Mf\_liverA, CL3599.Contig1\_Mf\_liverA, Unigene14907\_Mf\_liverA, CL848.Contig2\_Mf\_liverA, Unigene31639\_Mf\_liverA, CL784.Contig3\_Mf\_liverA, Unigene1479\_Mf\_liverA, CL4249.Contig1\_Mf\_liverA, Unigene25976\_Mf\_liverA, Unigene5632\_Mf\_liverA, NM\_009338, CL1263.Contig1\_Mf\_liverA, CL4550.Contig3\_Mf\_liverA, Unigene21816\_Mf\_liverA, NM\_008538, NM\_011305, Unigene30428\_Mf\_liverA, Unigene139\_Mf\_liverA, Unigene13498\_Mf\_liverA, CL529.Contig2\_Mf\_liverA, Unigene34032\_Mf\_liverA, Unigene5057\_Mf\_liverA, Unigene27547\_Mf\_liverA, NM\_001081372, Unigene24090\_Mf\_liverA, Unigene32789\_Mf\_liverA, Unigene36898\_Mf\_liverA, CL5316.Contig1\_Mf\_liverA, CL913.Contig1\_Mf\_liverA, Unigene37389\_Mf\_liverA, Unigene34406\_Mf\_liverA, Unigene32332\_Mf\_liverA, NM\_021525, CL3930.Contig1\_Mf\_liverA, Unigene7195\_Mf\_liverA, Unigene35506\_Mf\_liverA, Unigene24613\_Mf\_liverA, Unigene26611\_Mf\_liverA, CL532.Contig1\_Mf\_liverA, Unigene27082\_Mf\_liverA, Unigene5712\_Mf\_liverA, Unigene1280\_Mf\_liverA, Unigene1205\_Mf\_liverA, Unigene30288\_Mf\_liverA, CL442.Contig2\_Mf\_liverA, CL3166.Contig4\_Mf\_liverA, CL2855.Contig1\_Mf\_liverA, NM\_027959, NM\_013821, Unigene40796\_Mf\_liverA, Unigene31051\_Mf\_liverA, NM\_011732, Unigene30426\_Mf\_liverA, NM\_009178, CL425.Contig1\_Mf\_liverA, CL2797.Contig2\_Mf\_liverA, Unigene28143\_Mf\_liverA, CL1125.Contig1\_Mf\_liverA, Unigene14916\_Mf\_liverA, CL4162.Contig1\_Mf\_liverA, Unigene15077\_Mf\_liverA, Unigene30217\_Mf\_liverA, Unigene2050\_Mf\_liverA, CL4600.Contig1\_Mf\_liverA, Unigene673\_Mf\_liverA, CL3382.Contig1\_Mf\_liverA, Unigene5693\_Mf\_liverA, Unigene29134\_Mf\_liverA, CL1100.Contig1\_Mf\_liverA, Unigene32093\_Mf\_liverA, Unigene35958\_Mf\_liverA, Unigene29876\_Mf\_liverA, CL4583.Contig2\_Mf\_liverA, Unigene36414\_Mf\_liverA, NM\_021423, CL1988.Contig1\_Mf\_liverA, Unigene32803\_Mf\_liverA, Unigene36328\_Mf\_liverA, Unigene34375\_Mf\_liverA, CL5309.Contig1\_Mf\_liverA, CL3002.Contig1\_Mf\_liverA, Unigene36315\_Mf\_liverA, CL1988.Contig3\_Mf\_liverA, CL1810.Contig1\_Mf\_liverA, Unigene36949\_Mf\_liverA, CL4033.Contig1\_Mf\_liverA, Unigene5488\_Mf\_liverA, Unigene28662\_Mf\_liverA, Unigene33736\_Mf\_liverA, Unigene21026\_Mf\_liverA, Unigene13683\_Mf\_liverA, CL1493.Contig1\_Mf\_liverA, Unigene32233\_Mf\_liverA, Unigene5607\_Mf\_liverA, Unigene9698\_Mf\_liverA, Unigene36420\_Mf\_liverA, Unigene24536\_Mf\_liverA, Unigene542\_Mf\_liverA, Unigene35476\_Mf\_liverA, Unigene24758\_Mf\_liverA, Unigene13196\_Mf\_liverA, CL114.Contig2\_Mf\_liverA, Unigene14449\_Mf\_liverA, Unigene24471\_Mf\_liverA, CL1076.Contig1\_Mf\_liverA, NM\_009883, Unigene15888\_Mf\_liverA, CL4220.Contig1\_Mf\_liverA, CL4338.Contig1\_Mf\_liverA, Unigene15433\_Mf\_liverA, NM\_009448, CL5307.Contig1\_Mf\_liverA, NM\_019817, CL5052.Contig1\_Mf\_liverA, NM\_019879, Unigene31670\_Mf\_liverA, Unigene25595\_Mf\_liverA, Unigene14263\_Mf\_liverA, CL4816.Contig3\_Mf\_liverA, CL114.Contig1\_Mf\_liverA, Unigene24225\_Mf\_liverA, Unigene37344\_Mf\_liverA, CL5275.Contig2\_Mf\_liverA, Unigene36418\_Mf\_liverA, CL4007.Contig1\_Mf\_liverA, Unigene29558\_Mf\_liverA, CL55.Contig1\_Mf\_liverA, CL2355.Contig1\_Mf\_liverA, CL4984.Contig1\_Mf\_liverA, Unigene281\_Mf\_liverA, Unigene24537\_Mf\_liverA, Unigene32335\_Mf\_liverA, Unigene10313\_Mf\_liverA, NM\_011978, NM\_177652, Unigene37153\_Mf\_liverA, Unigene14809\_Mf\_liverA, CL3104.Contig1\_Mf\_liverA, Unigene36593\_Mf\_liverA, Unigene5489\_Mf\_liverA, Unigene34444\_Mf\_liverA, Unigene7399\_Mf\_liverA, Unigene33993\_Mf\_liverA, NM\_009011, Unigene16529\_Mf\_liverA, NM\_026313, CL3911.Contig2\_Mf\_liverA, Unigene44057\_Mf\_liverA, CL5007.Contig1\_Mf\_liverA, CL5698.Contig1\_Mf\_liverA, Unigene31392\_Mf\_liverA, Unigene4681\_Mf\_liverA, NM\_008851, CL591.Contig1\_Mf\_liverA, Unigene5886\_Mf\_liverA, CL336.Contig3\_Mf\_liverA |
| nuclear speck | Unigene5057\_Mf\_liverA, CL2355.Contig1\_Mf\_liverA |
| organelle part | NM\_176843, Unigene35884\_Mf\_liverA, CL4302.Contig1\_Mf\_liverA, Unigene38015\_Mf\_liverA, NM\_028291, NM\_206537, CL3835.Contig2\_Mf\_liverA, Unigene29011\_Mf\_liverA, NM\_025593, Unigene28899\_Mf\_liverA, CL4550.Contig2\_Mf\_liverA, Unigene36851\_Mf\_liverA, NM\_080638, CL523.Contig1\_Mf\_liverA, NM\_008277, NM\_011082, CL4105.Contig1\_Mf\_liverA, Unigene7336\_Mf\_liverA, CL482.Contig1\_Mf\_liverA, Unigene35507\_Mf\_liverA, Unigene600\_Mf\_liverA, Unigene39886\_Mf\_liverA, Unigene15064\_Mf\_liverA, Unigene30878\_Mf\_liverA, Unigene32920\_Mf\_liverA, Unigene34124\_Mf\_liverA, CL6038.Contig2\_Mf\_liverA, Unigene32570\_Mf\_liverA, Unigene34341\_Mf\_liverA, Unigene15295\_Mf\_liverA, Unigene34789\_Mf\_liverA, CL695.Contig1\_Mf\_liverA, Unigene12999\_Mf\_liverA, Unigene29082\_Mf\_liverA, CL4141.Contig1\_Mf\_liverA, NM\_145419, Unigene2\_Mf\_liverA, CL1119.Contig1\_Mf\_liverA, Unigene13658\_Mf\_liverA, Unigene13593\_Mf\_liverA, NM\_145474, NM\_134127, Unigene32546\_Mf\_liverA, Unigene20874\_Mf\_liverA, Unigene34394\_Mf\_liverA, Unigene25596\_Mf\_liverA, Unigene37109\_Mf\_liverA, NM\_031165, CL497.Contig2\_Mf\_liverA, CL1493.Contig2\_Mf\_liverA, CL6.Contig1\_Mf\_liverA, CL2697.Contig4\_Mf\_liverA, NM\_008261, NM\_024434, CL2574.Contig1\_Mf\_liverA, Unigene5941\_Mf\_liverA, Unigene23328\_Mf\_liverA, Unigene802\_Mf\_liverA, CL4816.Contig1\_Mf\_liverA, CL787.Contig1\_Mf\_liverA, Unigene15055\_Mf\_liverA, Unigene5639\_Mf\_liverA, CL5807.Contig1\_Mf\_liverA, Unigene29490\_Mf\_liverA, Unigene32594\_Mf\_liverA, Unigene36417\_Mf\_liverA, CL2855.Contig2\_Mf\_liverA, Unigene12967\_Mf\_liverA, Unigene36691\_Mf\_liverA, CL4263.Contig1\_Mf\_liverA, CL3599.Contig1\_Mf\_liverA, Unigene14907\_Mf\_liverA, CL848.Contig2\_Mf\_liverA, Unigene31639\_Mf\_liverA, CL784.Contig3\_Mf\_liverA, Unigene1479\_Mf\_liverA, CL4249.Contig1\_Mf\_liverA, NM\_010001, Unigene25976\_Mf\_liverA, Unigene5632\_Mf\_liverA, NM\_009338, CL1263.Contig1\_Mf\_liverA, CL4550.Contig3\_Mf\_liverA, Unigene21816\_Mf\_liverA, NM\_007811, NM\_008538, NM\_011305, Unigene30428\_Mf\_liverA, Unigene139\_Mf\_liverA, Unigene13498\_Mf\_liverA, CL529.Contig2\_Mf\_liverA, Unigene34032\_Mf\_liverA, Unigene5057\_Mf\_liverA, Unigene27547\_Mf\_liverA, NM\_001081372, Unigene24090\_Mf\_liverA, Unigene32789\_Mf\_liverA, Unigene36898\_Mf\_liverA, CL5316.Contig1\_Mf\_liverA, CL913.Contig1\_Mf\_liverA, NM\_027660, Unigene37389\_Mf\_liverA, Unigene34406\_Mf\_liverA, Unigene32332\_Mf\_liverA, NM\_021525, CL3930.Contig1\_Mf\_liverA, Unigene7195\_Mf\_liverA, Unigene35506\_Mf\_liverA, Unigene24613\_Mf\_liverA, Unigene26611\_Mf\_liverA, CL532.Contig1\_Mf\_liverA, Unigene27082\_Mf\_liverA, Unigene5712\_Mf\_liverA, Unigene1280\_Mf\_liverA, Unigene1205\_Mf\_liverA, Unigene30288\_Mf\_liverA, CL442.Contig2\_Mf\_liverA, CL3166.Contig4\_Mf\_liverA, CL2855.Contig1\_Mf\_liverA, NM\_027959, NM\_013821, Unigene40796\_Mf\_liverA, NM\_001100182, Unigene31051\_Mf\_liverA, NM\_011732, Unigene30426\_Mf\_liverA, NM\_009178, CL425.Contig1\_Mf\_liverA, CL2797.Contig2\_Mf\_liverA, Unigene28143\_Mf\_liverA, CL1125.Contig1\_Mf\_liverA, Unigene14916\_Mf\_liverA, CL4162.Contig1\_Mf\_liverA, Unigene15077\_Mf\_liverA, Unigene30217\_Mf\_liverA, Unigene2050\_Mf\_liverA, CL4600.Contig1\_Mf\_liverA, Unigene673\_Mf\_liverA, CL3382.Contig1\_Mf\_liverA, Unigene5693\_Mf\_liverA, NM\_029582, Unigene29134\_Mf\_liverA, CL1100.Contig1\_Mf\_liverA, Unigene32093\_Mf\_liverA, Unigene35958\_Mf\_liverA, Unigene29876\_Mf\_liverA, CL4583.Contig2\_Mf\_liverA, Unigene36414\_Mf\_liverA, NM\_021423, CL1988.Contig1\_Mf\_liverA, Unigene32803\_Mf\_liverA, Unigene36328\_Mf\_liverA, Unigene34375\_Mf\_liverA, CL5309.Contig1\_Mf\_liverA, CL3002.Contig1\_Mf\_liverA, Unigene36315\_Mf\_liverA, CL1988.Contig3\_Mf\_liverA, CL1810.Contig1\_Mf\_liverA, Unigene36949\_Mf\_liverA, CL4033.Contig1\_Mf\_liverA, Unigene5488\_Mf\_liverA, Unigene28662\_Mf\_liverA, Unigene33736\_Mf\_liverA, Unigene21026\_Mf\_liverA, CL1493.Contig1\_Mf\_liverA, Unigene13683\_Mf\_liverA, NM\_001100181, Unigene32233\_Mf\_liverA, Unigene5607\_Mf\_liverA, Unigene9698\_Mf\_liverA, Unigene36420\_Mf\_liverA, Unigene24536\_Mf\_liverA, Unigene542\_Mf\_liverA, Unigene35476\_Mf\_liverA, Unigene24758\_Mf\_liverA, Unigene13196\_Mf\_liverA, CL114.Contig2\_Mf\_liverA, Unigene14449\_Mf\_liverA, Unigene24471\_Mf\_liverA, CL1076.Contig1\_Mf\_liverA, Unigene15888\_Mf\_liverA, NM\_009883, CL4220.Contig1\_Mf\_liverA, CL4338.Contig1\_Mf\_liverA, Unigene15433\_Mf\_liverA, NM\_009448, CL5307.Contig1\_Mf\_liverA, NM\_019817, CL5052.Contig1\_Mf\_liverA, Unigene5750\_Mf\_liverA, NM\_019879, Unigene31670\_Mf\_liverA, NM\_007822, Unigene25595\_Mf\_liverA, Unigene14263\_Mf\_liverA, CL4816.Contig3\_Mf\_liverA, CL114.Contig1\_Mf\_liverA, Unigene24225\_Mf\_liverA, Unigene37344\_Mf\_liverA, CL5275.Contig2\_Mf\_liverA, Unigene36418\_Mf\_liverA, CL4007.Contig1\_Mf\_liverA, Unigene29558\_Mf\_liverA, CL55.Contig1\_Mf\_liverA, CL2355.Contig1\_Mf\_liverA, CL4984.Contig1\_Mf\_liverA, Unigene281\_Mf\_liverA, Unigene24537\_Mf\_liverA, Unigene32335\_Mf\_liverA, Unigene10313\_Mf\_liverA, NM\_011978, NM\_177652, Unigene37153\_Mf\_liverA, Unigene14809\_Mf\_liverA, CL3104.Contig1\_Mf\_liverA, Unigene36593\_Mf\_liverA, Unigene5489\_Mf\_liverA, NM\_010002, Unigene34444\_Mf\_liverA, Unigene7399\_Mf\_liverA, Unigene33993\_Mf\_liverA, NM\_009011, Unigene16529\_Mf\_liverA, NM\_026313, CL3911.Contig2\_Mf\_liverA, Unigene44057\_Mf\_liverA, CL5007.Contig1\_Mf\_liverA, CL5698.Contig1\_Mf\_liverA, Unigene31392\_Mf\_liverA, Unigene4681\_Mf\_liverA, NM\_008851, CL591.Contig1\_Mf\_liverA, Unigene5886\_Mf\_liverA, CL336.Contig3\_Mf\_liverA |
| membrane-bounded organelle | Unigene21684\_Mf\_liverA, NM\_176843, CL3669.Contig2\_Mf\_liverA, Unigene29011\_Mf\_liverA, NM\_025593, Unigene36851\_Mf\_liverA, NM\_080638, NM\_010378, Unigene15553\_Mf\_liverA, NM\_011082, Unigene19687\_Mf\_liverA, CL854.Contig1\_Mf\_liverA, CL4105.Contig1\_Mf\_liverA, Unigene7336\_Mf\_liverA, Unigene35507\_Mf\_liverA, Unigene6465\_Mf\_liverA, CL3617.Contig1\_Mf\_liverA, Unigene39886\_Mf\_liverA, Unigene15064\_Mf\_liverA, Unigene32920\_Mf\_liverA, Unigene34124\_Mf\_liverA, NR\_004446, Unigene36765\_Mf\_liverA, Unigene34341\_Mf\_liverA, Unigene30142\_Mf\_liverA, CL695.Contig1\_Mf\_liverA, Unigene29082\_Mf\_liverA, Unigene2\_Mf\_liverA, CL4919.Contig1\_Mf\_liverA, Unigene31623\_Mf\_liverA, Unigene7897\_Mf\_liverA, NM\_145474, Unigene37084\_Mf\_liverA, Unigene20874\_Mf\_liverA, NM\_009776, Unigene33054\_Mf\_liverA, Unigene34394\_Mf\_liverA, NM\_031165, Unigene25594\_Mf\_liverA, CL497.Contig2\_Mf\_liverA, Unigene36430\_Mf\_liverA, CL6.Contig1\_Mf\_liverA, Unigene18848\_Mf\_liverA, NM\_016697, Unigene802\_Mf\_liverA, CL4816.Contig1\_Mf\_liverA, Unigene15055\_Mf\_liverA, CL3154.Contig1\_Mf\_liverA, CL5807.Contig1\_Mf\_liverA, Unigene32594\_Mf\_liverA, Unigene13363\_Mf\_liverA, Unigene743\_Mf\_liverA, Unigene36417\_Mf\_liverA, CL2855.Contig2\_Mf\_liverA, CL4263.Contig1\_Mf\_liverA, NM\_021278, Unigene14907\_Mf\_liverA, Unigene1479\_Mf\_liverA, CL4249.Contig1\_Mf\_liverA, NM\_010001, Unigene25976\_Mf\_liverA, NM\_009338, Unigene44882\_Mf\_liverA, Unigene36698\_Mf\_liverA, Unigene139\_Mf\_liverA, Unigene31625\_Mf\_liverA, NM\_022324, Unigene34032\_Mf\_liverA, Unigene5057\_Mf\_liverA, Unigene27547\_Mf\_liverA, Unigene15290\_Mf\_liverA, Unigene24090\_Mf\_liverA, Unigene32789\_Mf\_liverA, Unigene36898\_Mf\_liverA, CL5316.Contig1\_Mf\_liverA, Unigene35664\_Mf\_liverA, CL1688.Contig3\_Mf\_liverA, NM\_027660, Unigene37389\_Mf\_liverA, Unigene32332\_Mf\_liverA, Unigene4723\_Mf\_liverA, NR\_003630, CL3930.Contig1\_Mf\_liverA, Unigene32852\_Mf\_liverA, Unigene24613\_Mf\_liverA, CL532.Contig1\_Mf\_liverA, Unigene1280\_Mf\_liverA, Unigene30288\_Mf\_liverA, CL3166.Contig4\_Mf\_liverA, NM\_027959, Unigene32059\_Mf\_liverA, Unigene34140\_Mf\_liverA, Unigene4363\_Mf\_liverA, Unigene836\_Mf\_liverA, CL186.Contig3\_Mf\_liverA, CL425.Contig1\_Mf\_liverA, CL2797.Contig2\_Mf\_liverA, Unigene30832\_Mf\_liverA, Unigene28143\_Mf\_liverA, Unigene14916\_Mf\_liverA, Unigene37243\_Mf\_liverA, Unigene30217\_Mf\_liverA, Unigene15077\_Mf\_liverA, CL4600.Contig1\_Mf\_liverA, CL3382.Contig1\_Mf\_liverA, Unigene5693\_Mf\_liverA, NM\_029582, Unigene29134\_Mf\_liverA, Unigene35958\_Mf\_liverA, Unigene29876\_Mf\_liverA, Unigene28687\_Mf\_liverA, Unigene34375\_Mf\_liverA, CL1988.Contig3\_Mf\_liverA, CL1810.Contig1\_Mf\_liverA, NM\_172679, CL4033.Contig1\_Mf\_liverA, Unigene34983\_Mf\_liverA, Unigene28662\_Mf\_liverA, CL2439.Contig1\_Mf\_liverA, Unigene33330\_Mf\_liverA, Unigene6464\_Mf\_liverA, CL3933.Contig1\_Mf\_liverA, Unigene21026\_Mf\_liverA, NM\_011221, CL1493.Contig1\_Mf\_liverA, Unigene13683\_Mf\_liverA, Unigene9698\_Mf\_liverA, Unigene36420\_Mf\_liverA, Unigene542\_Mf\_liverA, Unigene35476\_Mf\_liverA, Unigene24758\_Mf\_liverA, Unigene24471\_Mf\_liverA, CL5828.Contig2\_Mf\_liverA, Unigene33080\_Mf\_liverA, Unigene15888\_Mf\_liverA, CL4220.Contig1\_Mf\_liverA, Unigene15433\_Mf\_liverA, CL4338.Contig1\_Mf\_liverA, NM\_019817, CL5052.Contig1\_Mf\_liverA, NM\_010469, Unigene5750\_Mf\_liverA, NM\_019879, NR\_033215, NM\_007822, Unigene25595\_Mf\_liverA, Unigene15470\_Mf\_liverA, Unigene14286\_Mf\_liverA, CL4816.Contig3\_Mf\_liverA, CL114.Contig1\_Mf\_liverA, Unigene39011\_Mf\_liverA, Unigene24225\_Mf\_liverA, Unigene37344\_Mf\_liverA, CL5275.Contig2\_Mf\_liverA, NM\_145218, Unigene36189\_Mf\_liverA, Unigene29558\_Mf\_liverA, Unigene13950\_Mf\_liverA, Unigene28459\_Mf\_liverA, Unigene37245\_Mf\_liverA, Unigene281\_Mf\_liverA, NM\_177652, Unigene10313\_Mf\_liverA, NM\_011978, Unigene37104\_Mf\_liverA, CL3104.Contig1\_Mf\_liverA, Unigene36593\_Mf\_liverA, NM\_010002, NM\_013842, NM\_027406, Unigene16529\_Mf\_liverA, Unigene37232\_Mf\_liverA, NM\_026313, Unigene28609\_Mf\_liverA, CL5007.Contig1\_Mf\_liverA, CL5698.Contig1\_Mf\_liverA, Unigene14461\_Mf\_liverA, Unigene31392\_Mf\_liverA, Unigene35618\_Mf\_liverA, NM\_011256, Unigene35884\_Mf\_liverA, Unigene38015\_Mf\_liverA, CL3835.Contig2\_Mf\_liverA, NM\_206537, Unigene31199\_Mf\_liverA, CL4550.Contig2\_Mf\_liverA, Unigene28899\_Mf\_liverA, Unigene28244\_Mf\_liverA, Unigene46870\_Mf\_liverA, CL523.Contig1\_Mf\_liverA, NM\_008277, CL482.Contig1\_Mf\_liverA, Unigene27071\_Mf\_liverA, Unigene36762\_Mf\_liverA, Unigene600\_Mf\_liverA, NM\_011072, Unigene18795\_Mf\_liverA, Unigene30878\_Mf\_liverA, Unigene40289\_Mf\_liverA, NM\_134012, CL6038.Contig2\_Mf\_liverA, Unigene32570\_Mf\_liverA, Unigene37904\_Mf\_liverA, Unigene15295\_Mf\_liverA, Unigene34789\_Mf\_liverA, CL4141.Contig1\_Mf\_liverA, Unigene24221\_Mf\_liverA, NM\_145419, CL5978.Contig3\_Mf\_liverA, CL1119.Contig1\_Mf\_liverA, Unigene13658\_Mf\_liverA, NM\_134127, Unigene11\_Mf\_liverA, Unigene14609\_Mf\_liverA, Unigene25596\_Mf\_liverA, Unigene37109\_Mf\_liverA, CL3778.Contig2\_Mf\_liverA, Unigene5994\_Mf\_liverA, CL1493.Contig2\_Mf\_liverA, CL2697.Contig4\_Mf\_liverA, CL2574.Contig1\_Mf\_liverA, NM\_024434, NM\_008261, Unigene38104\_Mf\_liverA, Unigene23328\_Mf\_liverA, Unigene36626\_Mf\_liverA, CL787.Contig1\_Mf\_liverA, Unigene29490\_Mf\_liverA, Unigene36691\_Mf\_liverA, Unigene12967\_Mf\_liverA, NM\_001162917, Unigene31988\_Mf\_liverA, CL2389.Contig1\_Mf\_liverA, CL3750.Contig2\_Mf\_liverA, CL3599.Contig1\_Mf\_liverA, Unigene39141\_Mf\_liverA, CL784.Contig3\_Mf\_liverA, Unigene31639\_Mf\_liverA, CL838.Contig3\_Mf\_liverA, Unigene10774\_Mf\_liverA, Unigene5632\_Mf\_liverA, CL1263.Contig1\_Mf\_liverA, CL4550.Contig3\_Mf\_liverA, Unigene21816\_Mf\_liverA, NM\_007811, NM\_008538, CL2142.Contig2\_Mf\_liverA, Unigene30428\_Mf\_liverA, NM\_011305, Unigene13498\_Mf\_liverA, CL529.Contig2\_Mf\_liverA, Unigene30584\_Mf\_liverA, Unigene4510\_Mf\_liverA, Unigene17579\_Mf\_liverA, NM\_001081372, CL913.Contig1\_Mf\_liverA, Unigene37148\_Mf\_liverA, Unigene34406\_Mf\_liverA, NM\_021525, Unigene7195\_Mf\_liverA, Unigene35506\_Mf\_liverA, Unigene32058\_Mf\_liverA, Unigene27082\_Mf\_liverA, Unigene37178\_Mf\_liverA, Unigene5712\_Mf\_liverA, CL2919.Contig1\_Mf\_liverA, Unigene1205\_Mf\_liverA, CL442.Contig2\_Mf\_liverA, CL6039.Contig1\_Mf\_liverA, CL2855.Contig1\_Mf\_liverA, CL4411.Contig4\_Mf\_liverA, NM\_013821, Unigene40796\_Mf\_liverA, Unigene33420\_Mf\_liverA, Unigene32412\_Mf\_liverA, NM\_001100182, Unigene31051\_Mf\_liverA, NM\_011732, Unigene30426\_Mf\_liverA, NM\_009178, Unigene49971\_Mf\_liverA, Unigene38307\_Mf\_liverA, Unigene36190\_Mf\_liverA, Unigene27406\_Mf\_liverA, Unigene34754\_Mf\_liverA, Unigene17042\_Mf\_liverA, Unigene33746\_Mf\_liverA, CL1125.Contig1\_Mf\_liverA, CL4162.Contig1\_Mf\_liverA, Unigene27922\_Mf\_liverA, Unigene673\_Mf\_liverA, CL1100.Contig1\_Mf\_liverA, Unigene32093\_Mf\_liverA, CL4583.Contig2\_Mf\_liverA, Unigene36414\_Mf\_liverA, CL485.Contig1\_Mf\_liverA, CL1988.Contig1\_Mf\_liverA, Unigene5175\_Mf\_liverA, Unigene36328\_Mf\_liverA, Unigene15552\_Mf\_liverA, Unigene38280\_Mf\_liverA, CL4745.Contig1\_Mf\_liverA, CL3002.Contig1\_Mf\_liverA, CL5309.Contig1\_Mf\_liverA, Unigene36315\_Mf\_liverA, Unigene31483\_Mf\_liverA, Unigene36949\_Mf\_liverA, CL887.Contig2\_Mf\_liverA, Unigene5488\_Mf\_liverA, CL2597.Contig1\_Mf\_liverA, Unigene33736\_Mf\_liverA, Unigene35037\_Mf\_liverA, Unigene14810\_Mf\_liverA, Unigene1212\_Mf\_liverA, NM\_001100181, Unigene32233\_Mf\_liverA, CL838.Contig6\_Mf\_liverA, Unigene5607\_Mf\_liverA, CL4770.Contig1\_Mf\_liverA, Unigene32143\_Mf\_liverA, Unigene24536\_Mf\_liverA, CL3750.Contig1\_Mf\_liverA, Unigene13196\_Mf\_liverA, CL114.Contig2\_Mf\_liverA, Unigene14449\_Mf\_liverA, CL1076.Contig1\_Mf\_liverA, NM\_009883, Unigene18796\_Mf\_liverA, CL5307.Contig1\_Mf\_liverA, Unigene31670\_Mf\_liverA, CL5978.Contig2\_Mf\_liverA, NM\_178405, NM\_172203, CL442.Contig5\_Mf\_liverA, Unigene120\_Mf\_liverA, Unigene36418\_Mf\_liverA, Unigene15026\_Mf\_liverA, CL4007.Contig1\_Mf\_liverA, NM\_022997, CL55.Contig1\_Mf\_liverA, Unigene37120\_Mf\_liverA, Unigene31198\_Mf\_liverA, CL4984.Contig1\_Mf\_liverA, Unigene24537\_Mf\_liverA, Unigene4863\_Mf\_liverA, CL2355.Contig1\_Mf\_liverA, Unigene32335\_Mf\_liverA, Unigene37153\_Mf\_liverA, Unigene14809\_Mf\_liverA, NM\_027604, Unigene5489\_Mf\_liverA, Unigene34444\_Mf\_liverA, Unigene7399\_Mf\_liverA, Unigene35678\_Mf\_liverA, Unigene33993\_Mf\_liverA, Unigene138\_Mf\_liverA, NM\_009011, Unigene8560\_Mf\_liverA, CL3911.Contig2\_Mf\_liverA, Unigene5761\_Mf\_liverA, Unigene4681\_Mf\_liverA, NM\_008851, Unigene37819\_Mf\_liverA, Unigene5886\_Mf\_liverA, CL336.Contig3\_Mf\_liverA |
| intracellular membrane-bounded organelle | Unigene21684\_Mf\_liverA, NM\_176843, CL3669.Contig2\_Mf\_liverA, Unigene29011\_Mf\_liverA, NM\_025593, Unigene36851\_Mf\_liverA, NM\_080638, NM\_010378, Unigene15553\_Mf\_liverA, NM\_011082, Unigene19687\_Mf\_liverA, CL854.Contig1\_Mf\_liverA, CL4105.Contig1\_Mf\_liverA, Unigene7336\_Mf\_liverA, Unigene35507\_Mf\_liverA, Unigene6465\_Mf\_liverA, CL3617.Contig1\_Mf\_liverA, Unigene39886\_Mf\_liverA, Unigene15064\_Mf\_liverA, Unigene32920\_Mf\_liverA, Unigene34124\_Mf\_liverA, NR\_004446, Unigene36765\_Mf\_liverA, Unigene34341\_Mf\_liverA, Unigene30142\_Mf\_liverA, CL695.Contig1\_Mf\_liverA, Unigene29082\_Mf\_liverA, Unigene2\_Mf\_liverA, CL4919.Contig1\_Mf\_liverA, Unigene31623\_Mf\_liverA, Unigene7897\_Mf\_liverA, NM\_145474, Unigene37084\_Mf\_liverA, Unigene20874\_Mf\_liverA, NM\_009776, Unigene33054\_Mf\_liverA, Unigene34394\_Mf\_liverA, NM\_031165, Unigene25594\_Mf\_liverA, CL497.Contig2\_Mf\_liverA, Unigene36430\_Mf\_liverA, CL6.Contig1\_Mf\_liverA, Unigene18848\_Mf\_liverA, NM\_016697, Unigene802\_Mf\_liverA, CL4816.Contig1\_Mf\_liverA, Unigene15055\_Mf\_liverA, CL3154.Contig1\_Mf\_liverA, CL5807.Contig1\_Mf\_liverA, Unigene32594\_Mf\_liverA, Unigene13363\_Mf\_liverA, Unigene743\_Mf\_liverA, Unigene36417\_Mf\_liverA, CL2855.Contig2\_Mf\_liverA, CL4263.Contig1\_Mf\_liverA, NM\_021278, Unigene14907\_Mf\_liverA, Unigene1479\_Mf\_liverA, CL4249.Contig1\_Mf\_liverA, NM\_010001, Unigene25976\_Mf\_liverA, NM\_009338, Unigene44882\_Mf\_liverA, Unigene36698\_Mf\_liverA, Unigene139\_Mf\_liverA, Unigene31625\_Mf\_liverA, NM\_022324, Unigene34032\_Mf\_liverA, Unigene5057\_Mf\_liverA, Unigene27547\_Mf\_liverA, Unigene15290\_Mf\_liverA, Unigene24090\_Mf\_liverA, Unigene32789\_Mf\_liverA, Unigene36898\_Mf\_liverA, CL5316.Contig1\_Mf\_liverA, Unigene35664\_Mf\_liverA, CL1688.Contig3\_Mf\_liverA, NM\_027660, Unigene37389\_Mf\_liverA, Unigene32332\_Mf\_liverA, Unigene4723\_Mf\_liverA, NR\_003630, CL3930.Contig1\_Mf\_liverA, Unigene32852\_Mf\_liverA, Unigene24613\_Mf\_liverA, CL532.Contig1\_Mf\_liverA, Unigene1280\_Mf\_liverA, Unigene30288\_Mf\_liverA, CL3166.Contig4\_Mf\_liverA, NM\_027959, Unigene32059\_Mf\_liverA, Unigene34140\_Mf\_liverA, Unigene4363\_Mf\_liverA, Unigene836\_Mf\_liverA, CL186.Contig3\_Mf\_liverA, CL425.Contig1\_Mf\_liverA, CL2797.Contig2\_Mf\_liverA, Unigene30832\_Mf\_liverA, Unigene28143\_Mf\_liverA, Unigene14916\_Mf\_liverA, Unigene37243\_Mf\_liverA, Unigene30217\_Mf\_liverA, Unigene15077\_Mf\_liverA, CL4600.Contig1\_Mf\_liverA, CL3382.Contig1\_Mf\_liverA, Unigene5693\_Mf\_liverA, NM\_029582, Unigene29134\_Mf\_liverA, Unigene35958\_Mf\_liverA, Unigene29876\_Mf\_liverA, Unigene28687\_Mf\_liverA, Unigene34375\_Mf\_liverA, CL1988.Contig3\_Mf\_liverA, CL1810.Contig1\_Mf\_liverA, NM\_172679, CL4033.Contig1\_Mf\_liverA, Unigene34983\_Mf\_liverA, Unigene28662\_Mf\_liverA, CL2439.Contig1\_Mf\_liverA, Unigene33330\_Mf\_liverA, Unigene6464\_Mf\_liverA, CL3933.Contig1\_Mf\_liverA, Unigene21026\_Mf\_liverA, NM\_011221, CL1493.Contig1\_Mf\_liverA, Unigene13683\_Mf\_liverA, Unigene9698\_Mf\_liverA, Unigene36420\_Mf\_liverA, Unigene542\_Mf\_liverA, Unigene35476\_Mf\_liverA, Unigene24758\_Mf\_liverA, Unigene24471\_Mf\_liverA, CL5828.Contig2\_Mf\_liverA, Unigene33080\_Mf\_liverA, Unigene15888\_Mf\_liverA, CL4220.Contig1\_Mf\_liverA, Unigene15433\_Mf\_liverA, CL4338.Contig1\_Mf\_liverA, NM\_019817, CL5052.Contig1\_Mf\_liverA, NM\_010469, NM\_019879, NR\_033215, NM\_007822, Unigene25595\_Mf\_liverA, Unigene15470\_Mf\_liverA, Unigene14286\_Mf\_liverA, CL4816.Contig3\_Mf\_liverA, CL114.Contig1\_Mf\_liverA, Unigene39011\_Mf\_liverA, Unigene24225\_Mf\_liverA, Unigene37344\_Mf\_liverA, CL5275.Contig2\_Mf\_liverA, NM\_145218, Unigene36189\_Mf\_liverA, Unigene29558\_Mf\_liverA, Unigene13950\_Mf\_liverA, Unigene28459\_Mf\_liverA, Unigene37245\_Mf\_liverA, Unigene281\_Mf\_liverA, NM\_177652, Unigene10313\_Mf\_liverA, NM\_011978, Unigene37104\_Mf\_liverA, CL3104.Contig1\_Mf\_liverA, Unigene36593\_Mf\_liverA, NM\_010002, NM\_013842, NM\_027406, Unigene16529\_Mf\_liverA, Unigene37232\_Mf\_liverA, NM\_026313, Unigene28609\_Mf\_liverA, CL5007.Contig1\_Mf\_liverA, CL5698.Contig1\_Mf\_liverA, Unigene14461\_Mf\_liverA, Unigene31392\_Mf\_liverA, Unigene35618\_Mf\_liverA, Unigene35884\_Mf\_liverA, Unigene38015\_Mf\_liverA, CL3835.Contig2\_Mf\_liverA, NM\_206537, Unigene31199\_Mf\_liverA, CL4550.Contig2\_Mf\_liverA, Unigene28899\_Mf\_liverA, Unigene28244\_Mf\_liverA, Unigene46870\_Mf\_liverA, CL523.Contig1\_Mf\_liverA, NM\_008277, CL482.Contig1\_Mf\_liverA, Unigene27071\_Mf\_liverA, Unigene36762\_Mf\_liverA, Unigene600\_Mf\_liverA, NM\_011072, Unigene18795\_Mf\_liverA, Unigene30878\_Mf\_liverA, Unigene40289\_Mf\_liverA, NM\_134012, CL6038.Contig2\_Mf\_liverA, Unigene32570\_Mf\_liverA, Unigene37904\_Mf\_liverA, Unigene15295\_Mf\_liverA, Unigene34789\_Mf\_liverA, CL4141.Contig1\_Mf\_liverA, Unigene24221\_Mf\_liverA, NM\_145419, CL1119.Contig1\_Mf\_liverA, Unigene13658\_Mf\_liverA, NM\_134127, Unigene11\_Mf\_liverA, Unigene14609\_Mf\_liverA, Unigene25596\_Mf\_liverA, Unigene37109\_Mf\_liverA, CL3778.Contig2\_Mf\_liverA, Unigene5994\_Mf\_liverA, CL1493.Contig2\_Mf\_liverA, CL2697.Contig4\_Mf\_liverA, CL2574.Contig1\_Mf\_liverA, NM\_024434, NM\_008261, Unigene38104\_Mf\_liverA, Unigene23328\_Mf\_liverA, Unigene36626\_Mf\_liverA, CL787.Contig1\_Mf\_liverA, Unigene29490\_Mf\_liverA, Unigene36691\_Mf\_liverA, Unigene12967\_Mf\_liverA, NM\_001162917, Unigene31988\_Mf\_liverA, CL2389.Contig1\_Mf\_liverA, CL3750.Contig2\_Mf\_liverA, CL3599.Contig1\_Mf\_liverA, Unigene39141\_Mf\_liverA, CL784.Contig3\_Mf\_liverA, Unigene31639\_Mf\_liverA, CL838.Contig3\_Mf\_liverA, Unigene10774\_Mf\_liverA, Unigene5632\_Mf\_liverA, CL1263.Contig1\_Mf\_liverA, CL4550.Contig3\_Mf\_liverA, Unigene21816\_Mf\_liverA, NM\_007811, NM\_008538, CL2142.Contig2\_Mf\_liverA, Unigene30428\_Mf\_liverA, NM\_011305, Unigene13498\_Mf\_liverA, CL529.Contig2\_Mf\_liverA, Unigene4510\_Mf\_liverA, Unigene30584\_Mf\_liverA, Unigene17579\_Mf\_liverA, NM\_001081372, CL913.Contig1\_Mf\_liverA, Unigene37148\_Mf\_liverA, Unigene34406\_Mf\_liverA, NM\_021525, Unigene7195\_Mf\_liverA, Unigene35506\_Mf\_liverA, Unigene32058\_Mf\_liverA, Unigene27082\_Mf\_liverA, Unigene37178\_Mf\_liverA, Unigene5712\_Mf\_liverA, CL2919.Contig1\_Mf\_liverA, Unigene1205\_Mf\_liverA, CL442.Contig2\_Mf\_liverA, CL2855.Contig1\_Mf\_liverA, CL4411.Contig4\_Mf\_liverA, NM\_013821, Unigene40796\_Mf\_liverA, Unigene33420\_Mf\_liverA, Unigene32412\_Mf\_liverA, NM\_001100182, Unigene31051\_Mf\_liverA, NM\_011732, Unigene30426\_Mf\_liverA, NM\_009178, Unigene49971\_Mf\_liverA, Unigene38307\_Mf\_liverA, Unigene36190\_Mf\_liverA, Unigene27406\_Mf\_liverA, Unigene34754\_Mf\_liverA, Unigene17042\_Mf\_liverA, Unigene33746\_Mf\_liverA, CL1125.Contig1\_Mf\_liverA, CL4162.Contig1\_Mf\_liverA, Unigene27922\_Mf\_liverA, Unigene673\_Mf\_liverA, CL1100.Contig1\_Mf\_liverA, Unigene32093\_Mf\_liverA, CL4583.Contig2\_Mf\_liverA, Unigene36414\_Mf\_liverA, CL485.Contig1\_Mf\_liverA, CL1988.Contig1\_Mf\_liverA, Unigene5175\_Mf\_liverA, Unigene36328\_Mf\_liverA, Unigene15552\_Mf\_liverA, Unigene38280\_Mf\_liverA, CL4745.Contig1\_Mf\_liverA, CL3002.Contig1\_Mf\_liverA, CL5309.Contig1\_Mf\_liverA, Unigene36315\_Mf\_liverA, Unigene31483\_Mf\_liverA, Unigene36949\_Mf\_liverA, CL887.Contig2\_Mf\_liverA, Unigene5488\_Mf\_liverA, CL2597.Contig1\_Mf\_liverA, Unigene33736\_Mf\_liverA, Unigene35037\_Mf\_liverA, Unigene14810\_Mf\_liverA, Unigene1212\_Mf\_liverA, NM\_001100181, Unigene32233\_Mf\_liverA, CL838.Contig6\_Mf\_liverA, Unigene5607\_Mf\_liverA, CL4770.Contig1\_Mf\_liverA, Unigene32143\_Mf\_liverA, Unigene24536\_Mf\_liverA, CL3750.Contig1\_Mf\_liverA, Unigene13196\_Mf\_liverA, CL114.Contig2\_Mf\_liverA, Unigene14449\_Mf\_liverA, CL1076.Contig1\_Mf\_liverA, NM\_009883, Unigene18796\_Mf\_liverA, CL5307.Contig1\_Mf\_liverA, Unigene31670\_Mf\_liverA, NM\_178405, NM\_172203, CL442.Contig5\_Mf\_liverA, Unigene120\_Mf\_liverA, Unigene36418\_Mf\_liverA, Unigene15026\_Mf\_liverA, CL4007.Contig1\_Mf\_liverA, NM\_022997, CL55.Contig1\_Mf\_liverA, Unigene37120\_Mf\_liverA, Unigene31198\_Mf\_liverA, CL4984.Contig1\_Mf\_liverA, Unigene24537\_Mf\_liverA, Unigene4863\_Mf\_liverA, CL2355.Contig1\_Mf\_liverA, Unigene32335\_Mf\_liverA, Unigene37153\_Mf\_liverA, Unigene14809\_Mf\_liverA, NM\_027604, Unigene5489\_Mf\_liverA, Unigene34444\_Mf\_liverA, Unigene7399\_Mf\_liverA, Unigene35678\_Mf\_liverA, Unigene33993\_Mf\_liverA, Unigene138\_Mf\_liverA, NM\_009011, Unigene8560\_Mf\_liverA, CL3911.Contig2\_Mf\_liverA, Unigene5761\_Mf\_liverA, Unigene4681\_Mf\_liverA, NM\_008851, Unigene37819\_Mf\_liverA, Unigene5886\_Mf\_liverA, CL336.Contig3\_Mf\_liverA |
| mitochondrial inner membrane | Unigene24225\_Mf\_liverA, CL3599.Contig1\_Mf\_liverA, CL1125.Contig1\_Mf\_liverA, CL2797.Contig2\_Mf\_liverA, CL4984.Contig1\_Mf\_liverA, CL5316.Contig1\_Mf\_liverA |
| contractile fiber part | Unigene5639\_Mf\_liverA, Unigene32789\_Mf\_liverA, Unigene13593\_Mf\_liverA |
| cytosolic part | Unigene12999\_Mf\_liverA, CL3684.Contig2\_Mf\_liverA |
| extrinsic to membrane | Unigene37243\_Mf\_liverA, Unigene35958\_Mf\_liverA |
| mitochondrial envelope | Unigene35476\_Mf\_liverA, CL1125.Contig1\_Mf\_liverA, Unigene27082\_Mf\_liverA, CL4984.Contig1\_Mf\_liverA, CL5316.Contig1\_Mf\_liverA, CL1810.Contig1\_Mf\_liverA, Unigene24225\_Mf\_liverA, CL3599.Contig1\_Mf\_liverA, CL3104.Contig1\_Mf\_liverA, NM\_013821, CL2797.Contig2\_Mf\_liverA, Unigene2\_Mf\_liverA |
| insoluble fraction | NM\_001100182, NM\_001081372, NM\_206537, NM\_001081274, NM\_011978, NM\_010002, NM\_010001, NM\_134127, NM\_178405, NM\_145474, NM\_008483, NM\_007822, NM\_007811, NM\_001100181, NM\_011072, NM\_008851, NR\_024097, NM\_013821 |
| mitochondrial membrane | CL1125.Contig1\_Mf\_liverA, Unigene27082\_Mf\_liverA, CL4984.Contig1\_Mf\_liverA, CL5316.Contig1\_Mf\_liverA, CL1810.Contig1\_Mf\_liverA, Unigene24225\_Mf\_liverA, CL3599.Contig1\_Mf\_liverA, CL3104.Contig1\_Mf\_liverA, CL2797.Contig2\_Mf\_liverA, Unigene2\_Mf\_liverA |
| myofibril | Unigene5639\_Mf\_liverA, Unigene26611\_Mf\_liverA, Unigene13593\_Mf\_liverA |
| sarcomere | Unigene5639\_Mf\_liverA, Unigene13593\_Mf\_liverA |
| site of polarized growth | Unigene13950\_Mf\_liverA, Unigene16529\_Mf\_liverA |
| microtubule | Unigene27547\_Mf\_liverA, NM\_009448, CL425.Contig1\_Mf\_liverA, Unigene14263\_Mf\_liverA |
| intracellular organelle | Unigene21684\_Mf\_liverA, NM\_176843, CL4302.Contig1\_Mf\_liverA, NM\_009450, CL3669.Contig2\_Mf\_liverA, Unigene29011\_Mf\_liverA, NM\_025593, Unigene36851\_Mf\_liverA, NM\_080638, NM\_010378, Unigene15553\_Mf\_liverA, NM\_011082, Unigene19687\_Mf\_liverA, CL854.Contig1\_Mf\_liverA, CL4105.Contig1\_Mf\_liverA, Unigene7336\_Mf\_liverA, Unigene35507\_Mf\_liverA, Unigene6465\_Mf\_liverA, CL3617.Contig1\_Mf\_liverA, Unigene39886\_Mf\_liverA, Unigene15064\_Mf\_liverA, Unigene32920\_Mf\_liverA, Unigene34124\_Mf\_liverA, NR\_004446, Unigene36765\_Mf\_liverA, Unigene34341\_Mf\_liverA, Unigene30142\_Mf\_liverA, CL695.Contig1\_Mf\_liverA, Unigene34847\_Mf\_liverA, Unigene29082\_Mf\_liverA, Unigene2\_Mf\_liverA, CL4919.Contig1\_Mf\_liverA, Unigene31623\_Mf\_liverA, Unigene7897\_Mf\_liverA, NM\_145474, Unigene37084\_Mf\_liverA, Unigene20874\_Mf\_liverA, NM\_009776, Unigene33054\_Mf\_liverA, Unigene34394\_Mf\_liverA, NM\_031165, Unigene25594\_Mf\_liverA, CL497.Contig2\_Mf\_liverA, Unigene36430\_Mf\_liverA, CL6.Contig1\_Mf\_liverA, Unigene18848\_Mf\_liverA, NM\_016697, Unigene802\_Mf\_liverA, CL4816.Contig1\_Mf\_liverA, Unigene15055\_Mf\_liverA, Unigene5639\_Mf\_liverA, CL3154.Contig1\_Mf\_liverA, CL5807.Contig1\_Mf\_liverA, Unigene32594\_Mf\_liverA, Unigene13363\_Mf\_liverA, Unigene743\_Mf\_liverA, Unigene36417\_Mf\_liverA, CL2855.Contig2\_Mf\_liverA, CL4263.Contig1\_Mf\_liverA, NM\_021278, Unigene14907\_Mf\_liverA, Unigene1479\_Mf\_liverA, CL4249.Contig1\_Mf\_liverA, NM\_010001, Unigene25976\_Mf\_liverA, NM\_009338, Unigene44882\_Mf\_liverA, Unigene36698\_Mf\_liverA, Unigene139\_Mf\_liverA, Unigene31625\_Mf\_liverA, NM\_022324, Unigene34032\_Mf\_liverA, Unigene5057\_Mf\_liverA, Unigene27547\_Mf\_liverA, Unigene15290\_Mf\_liverA, Unigene24090\_Mf\_liverA, Unigene32789\_Mf\_liverA, Unigene36898\_Mf\_liverA, CL5316.Contig1\_Mf\_liverA, Unigene35664\_Mf\_liverA, CL1688.Contig3\_Mf\_liverA, NM\_027660, Unigene37389\_Mf\_liverA, Unigene32332\_Mf\_liverA, Unigene4723\_Mf\_liverA, NR\_003630, CL3930.Contig1\_Mf\_liverA, Unigene32852\_Mf\_liverA, Unigene24613\_Mf\_liverA, Unigene26611\_Mf\_liverA, CL532.Contig1\_Mf\_liverA, Unigene35169\_Mf\_liverA, Unigene1280\_Mf\_liverA, Unigene30288\_Mf\_liverA, CL3166.Contig4\_Mf\_liverA, NM\_027959, Unigene32059\_Mf\_liverA, Unigene34140\_Mf\_liverA, Unigene4363\_Mf\_liverA, Unigene836\_Mf\_liverA, CL186.Contig3\_Mf\_liverA, CL425.Contig1\_Mf\_liverA, CL2797.Contig2\_Mf\_liverA, Unigene30832\_Mf\_liverA, Unigene28143\_Mf\_liverA, Unigene14916\_Mf\_liverA, Unigene37243\_Mf\_liverA, Unigene30217\_Mf\_liverA, Unigene15077\_Mf\_liverA, Unigene2050\_Mf\_liverA, CL4600.Contig1\_Mf\_liverA, Unigene16184\_Mf\_liverA, CL3382.Contig1\_Mf\_liverA, Unigene5693\_Mf\_liverA, NM\_029582, Unigene29134\_Mf\_liverA, Unigene35958\_Mf\_liverA, Unigene29876\_Mf\_liverA, Unigene28687\_Mf\_liverA, Unigene32803\_Mf\_liverA, Unigene34375\_Mf\_liverA, CL1988.Contig3\_Mf\_liverA, CL1810.Contig1\_Mf\_liverA, NM\_172679, CL4033.Contig1\_Mf\_liverA, Unigene34983\_Mf\_liverA, Unigene28662\_Mf\_liverA, CL2439.Contig1\_Mf\_liverA, Unigene33330\_Mf\_liverA, Unigene6464\_Mf\_liverA, CL3933.Contig1\_Mf\_liverA, Unigene21026\_Mf\_liverA, NM\_011221, CL1493.Contig1\_Mf\_liverA, Unigene13683\_Mf\_liverA, Unigene9698\_Mf\_liverA, Unigene36420\_Mf\_liverA, Unigene542\_Mf\_liverA, Unigene35476\_Mf\_liverA, Unigene24758\_Mf\_liverA, Unigene24471\_Mf\_liverA, CL5828.Contig2\_Mf\_liverA, Unigene35168\_Mf\_liverA, Unigene33080\_Mf\_liverA, Unigene15888\_Mf\_liverA, CL4220.Contig1\_Mf\_liverA, Unigene15433\_Mf\_liverA, CL4338.Contig1\_Mf\_liverA, NM\_019817, CL5052.Contig1\_Mf\_liverA, NM\_010469, Unigene5750\_Mf\_liverA, NM\_019879, NR\_033215, NM\_007822, Unigene25595\_Mf\_liverA, Unigene14263\_Mf\_liverA, Unigene15470\_Mf\_liverA, Unigene14286\_Mf\_liverA, CL4816.Contig3\_Mf\_liverA, CL114.Contig1\_Mf\_liverA, Unigene39011\_Mf\_liverA, Unigene24225\_Mf\_liverA, Unigene37344\_Mf\_liverA, CL5275.Contig2\_Mf\_liverA, NM\_011207, NM\_145218, Unigene36189\_Mf\_liverA, Unigene29558\_Mf\_liverA, Unigene13950\_Mf\_liverA, Unigene28459\_Mf\_liverA, Unigene37245\_Mf\_liverA, Unigene281\_Mf\_liverA, NM\_177652, NM\_011978, Unigene10313\_Mf\_liverA, Unigene37104\_Mf\_liverA, CL3104.Contig1\_Mf\_liverA, Unigene36593\_Mf\_liverA, NM\_010002, NM\_013842, NM\_027406, Unigene16529\_Mf\_liverA, Unigene37232\_Mf\_liverA, NM\_026313, Unigene28609\_Mf\_liverA, CL5007.Contig1\_Mf\_liverA, CL5698.Contig1\_Mf\_liverA, Unigene14461\_Mf\_liverA, Unigene31392\_Mf\_liverA, CL591.Contig1\_Mf\_liverA, Unigene35618\_Mf\_liverA, NM\_011256, Unigene35884\_Mf\_liverA, Unigene38015\_Mf\_liverA, NM\_028291, NM\_173038, NM\_206537, CL3835.Contig2\_Mf\_liverA, Unigene31199\_Mf\_liverA, Unigene6959\_Mf\_liverA, CL4550.Contig2\_Mf\_liverA, Unigene28899\_Mf\_liverA, Unigene28244\_Mf\_liverA, Unigene46870\_Mf\_liverA, CL523.Contig1\_Mf\_liverA, NM\_008277, CL482.Contig1\_Mf\_liverA, Unigene27071\_Mf\_liverA, Unigene36762\_Mf\_liverA, Unigene600\_Mf\_liverA, NM\_011072, Unigene18795\_Mf\_liverA, Unigene30878\_Mf\_liverA, Unigene40289\_Mf\_liverA, NM\_134012, CL6038.Contig2\_Mf\_liverA, Unigene32570\_Mf\_liverA, Unigene37904\_Mf\_liverA, Unigene15295\_Mf\_liverA, Unigene12999\_Mf\_liverA, Unigene34789\_Mf\_liverA, CL4141.Contig1\_Mf\_liverA, Unigene24221\_Mf\_liverA, NM\_145419, CL5978.Contig3\_Mf\_liverA, CL1119.Contig1\_Mf\_liverA, Unigene13658\_Mf\_liverA, Unigene13593\_Mf\_liverA, NM\_134127, Unigene11\_Mf\_liverA, Unigene32546\_Mf\_liverA, Unigene14609\_Mf\_liverA, Unigene25596\_Mf\_liverA, Unigene37109\_Mf\_liverA, CL3778.Contig2\_Mf\_liverA, Unigene5994\_Mf\_liverA, CL1493.Contig2\_Mf\_liverA, CL2697.Contig4\_Mf\_liverA, Unigene5941\_Mf\_liverA, CL2574.Contig1\_Mf\_liverA, NM\_024434, NM\_008261, Unigene38104\_Mf\_liverA, Unigene23328\_Mf\_liverA, Unigene36626\_Mf\_liverA, CL787.Contig1\_Mf\_liverA, Unigene29490\_Mf\_liverA, Unigene36691\_Mf\_liverA, Unigene12967\_Mf\_liverA, NM\_001162917, Unigene31988\_Mf\_liverA, CL2389.Contig1\_Mf\_liverA, CL3750.Contig2\_Mf\_liverA, CL3599.Contig1\_Mf\_liverA, Unigene39141\_Mf\_liverA, CL848.Contig2\_Mf\_liverA, CL784.Contig3\_Mf\_liverA, Unigene31639\_Mf\_liverA, CL838.Contig3\_Mf\_liverA, Unigene10774\_Mf\_liverA, Unigene5632\_Mf\_liverA, CL1263.Contig1\_Mf\_liverA, CL4550.Contig3\_Mf\_liverA, Unigene21816\_Mf\_liverA, NM\_007811, NM\_008538, CL2142.Contig2\_Mf\_liverA, Unigene30428\_Mf\_liverA, NM\_011305, Unigene13498\_Mf\_liverA, CL529.Contig2\_Mf\_liverA, Unigene30584\_Mf\_liverA, Unigene4510\_Mf\_liverA, Unigene17579\_Mf\_liverA, NM\_001081372, CL913.Contig1\_Mf\_liverA, Unigene37148\_Mf\_liverA, Unigene34406\_Mf\_liverA, NM\_021525, Unigene7195\_Mf\_liverA, Unigene35506\_Mf\_liverA, Unigene32058\_Mf\_liverA, Unigene27082\_Mf\_liverA, Unigene37178\_Mf\_liverA, Unigene5712\_Mf\_liverA, CL2919.Contig1\_Mf\_liverA, Unigene1205\_Mf\_liverA, CL442.Contig2\_Mf\_liverA, CL6039.Contig1\_Mf\_liverA, CL2855.Contig1\_Mf\_liverA, CL4411.Contig4\_Mf\_liverA, NM\_013821, Unigene40796\_Mf\_liverA, Unigene33420\_Mf\_liverA, Unigene32412\_Mf\_liverA, NM\_001100182, Unigene31051\_Mf\_liverA, NM\_011732, Unigene30426\_Mf\_liverA, NM\_009178, Unigene49971\_Mf\_liverA, Unigene38307\_Mf\_liverA, Unigene36190\_Mf\_liverA, Unigene27406\_Mf\_liverA, Unigene34754\_Mf\_liverA, Unigene17042\_Mf\_liverA, Unigene3789\_Mf\_liverA, Unigene33746\_Mf\_liverA, CL1125.Contig1\_Mf\_liverA, CL4162.Contig1\_Mf\_liverA, Unigene27922\_Mf\_liverA, Unigene673\_Mf\_liverA, CL1100.Contig1\_Mf\_liverA, Unigene32093\_Mf\_liverA, CL4583.Contig2\_Mf\_liverA, Unigene36414\_Mf\_liverA, NM\_021423, CL485.Contig1\_Mf\_liverA, CL1988.Contig1\_Mf\_liverA, Unigene5175\_Mf\_liverA, Unigene36328\_Mf\_liverA, Unigene15552\_Mf\_liverA, Unigene38280\_Mf\_liverA, CL4745.Contig1\_Mf\_liverA, CL5309.Contig1\_Mf\_liverA, CL3002.Contig1\_Mf\_liverA, Unigene36315\_Mf\_liverA, Unigene31483\_Mf\_liverA, Unigene36949\_Mf\_liverA, CL887.Contig2\_Mf\_liverA, Unigene5488\_Mf\_liverA, CL2597.Contig1\_Mf\_liverA, Unigene33736\_Mf\_liverA, Unigene35037\_Mf\_liverA, Unigene14810\_Mf\_liverA, Unigene1212\_Mf\_liverA, NM\_001100181, Unigene32233\_Mf\_liverA, CL838.Contig6\_Mf\_liverA, Unigene5607\_Mf\_liverA, CL4770.Contig1\_Mf\_liverA, Unigene32143\_Mf\_liverA, Unigene24536\_Mf\_liverA, CL3750.Contig1\_Mf\_liverA, Unigene13196\_Mf\_liverA, CL114.Contig2\_Mf\_liverA, Unigene14449\_Mf\_liverA, CL1076.Contig1\_Mf\_liverA, NM\_009883, Unigene18796\_Mf\_liverA, NM\_009448, CL5307.Contig1\_Mf\_liverA, Unigene1327\_Mf\_liverA, Unigene31670\_Mf\_liverA, CL5978.Contig2\_Mf\_liverA, NM\_178405, NM\_172203, CL442.Contig5\_Mf\_liverA, Unigene120\_Mf\_liverA, Unigene36418\_Mf\_liverA, Unigene15026\_Mf\_liverA, CL4007.Contig1\_Mf\_liverA, NM\_022997, NM\_017379, CL55.Contig1\_Mf\_liverA, Unigene550\_Mf\_liverA, Unigene28489\_Mf\_liverA, Unigene37120\_Mf\_liverA, Unigene31198\_Mf\_liverA, CL4984.Contig1\_Mf\_liverA, Unigene24537\_Mf\_liverA, Unigene4863\_Mf\_liverA, CL2355.Contig1\_Mf\_liverA, Unigene32335\_Mf\_liverA, Unigene37153\_Mf\_liverA, Unigene14809\_Mf\_liverA, NM\_027604, Unigene5489\_Mf\_liverA, Unigene34444\_Mf\_liverA, Unigene7399\_Mf\_liverA, Unigene35678\_Mf\_liverA, Unigene33993\_Mf\_liverA, Unigene138\_Mf\_liverA, NM\_009011, Unigene8560\_Mf\_liverA, CL3911.Contig2\_Mf\_liverA, Unigene44057\_Mf\_liverA, Unigene5761\_Mf\_liverA, Unigene4681\_Mf\_liverA, NM\_008851, Unigene37819\_Mf\_liverA, Unigene5886\_Mf\_liverA, CL336.Contig3\_Mf\_liverA |
| ribosome | Unigene12999\_Mf\_liverA, Unigene31198\_Mf\_liverA, Unigene31199\_Mf\_liverA |
| protein complex | NM\_007396, NM\_176843, Unigene35884\_Mf\_liverA, CL4302.Contig1\_Mf\_liverA, CL3835.Contig2\_Mf\_liverA, NM\_011732, Unigene6959\_Mf\_liverA, NM\_025593, CL425.Contig1\_Mf\_liverA, Unigene49971\_Mf\_liverA, Unigene28244\_Mf\_liverA, Unigene36851\_Mf\_liverA, NM\_010378, Unigene36849\_Mf\_liverA, CL2339.Contig1\_Mf\_liverA, CL4162.Contig1\_Mf\_liverA, Unigene37526\_Mf\_liverA, Unigene27922\_Mf\_liverA, Unigene2050\_Mf\_liverA, CL3382.Contig1\_Mf\_liverA, Unigene30878\_Mf\_liverA, NR\_004446, Unigene18500\_Mf\_liverA, Unigene4938\_Mf\_liverA, Unigene32803\_Mf\_liverA, CL3002.Contig1\_Mf\_liverA, Unigene15295\_Mf\_liverA, Unigene34789\_Mf\_liverA, Unigene12999\_Mf\_liverA, CL1810.Contig1\_Mf\_liverA, CL4141.Contig1\_Mf\_liverA, Unigene5488\_Mf\_liverA, CL887.Contig2\_Mf\_liverA, CL1119.Contig1\_Mf\_liverA, Unigene13658\_Mf\_liverA, Unigene31333\_Mf\_liverA, Unigene29426\_Mf\_liverA, Unigene33054\_Mf\_liverA, Unigene843\_Mf\_liverA, NM\_011221, CL3684.Contig2\_Mf\_liverA, Unigene13683\_Mf\_liverA, Unigene33683\_Mf\_liverA, NM\_008261, Unigene5941\_Mf\_liverA, Unigene15055\_Mf\_liverA, Unigene5639\_Mf\_liverA, Unigene24471\_Mf\_liverA, CL3897.Contig5\_Mf\_liverA, Unigene32594\_Mf\_liverA, CL2855.Contig2\_Mf\_liverA, Unigene14907\_Mf\_liverA, Unigene15433\_Mf\_liverA, NM\_009448, NM\_019817, CL4456.Contig1\_Mf\_liverA, Unigene1479\_Mf\_liverA, NM\_019879, CL4249.Contig1\_Mf\_liverA, Unigene13143\_Mf\_liverA, Unigene5632\_Mf\_liverA, Unigene25976\_Mf\_liverA, NM\_008483, Unigene21816\_Mf\_liverA, Unigene14263\_Mf\_liverA, Unigene8218\_Mf\_liverA, Unigene15572\_Mf\_liverA, NM\_153589, Unigene13950\_Mf\_liverA, Unigene5057\_Mf\_liverA, Unigene34032\_Mf\_liverA, Unigene37245\_Mf\_liverA, Unigene27547\_Mf\_liverA, CL2355.Contig1\_Mf\_liverA, Unigene32335\_Mf\_liverA, Unigene37153\_Mf\_liverA, CL3104.Contig1\_Mf\_liverA, Unigene5489\_Mf\_liverA, Unigene34444\_Mf\_liverA, CL5808.Contig1\_Mf\_liverA, NM\_009011, Unigene37232\_Mf\_liverA, Unigene26611\_Mf\_liverA, Unigene27082\_Mf\_liverA, CL532.Contig1\_Mf\_liverA, CL5698.Contig1\_Mf\_liverA, Unigene31392\_Mf\_liverA, Unigene30288\_Mf\_liverA, Unigene1288\_Mf\_liverA, Unigene4681\_Mf\_liverA, CL275.Contig5\_Mf\_liverA, NM\_010380, CL2855.Contig1\_Mf\_liverA, CL591.Contig1\_Mf\_liverA, Unigene17569\_Mf\_liverA |
| spliceosomal complex | NM\_011732, Unigene33993\_Mf\_liverA |
| nuclear chromatin | Unigene14907\_Mf\_liverA, Unigene32594\_Mf\_liverA |
| mitochondrial part | Unigene35476\_Mf\_liverA, CL4984.Contig1\_Mf\_liverA, CL5316.Contig1\_Mf\_liverA, Unigene36691\_Mf\_liverA, CL1810.Contig1\_Mf\_liverA, CL3599.Contig1\_Mf\_liverA, CL3104.Contig1\_Mf\_liverA, CL5052.Contig1\_Mf\_liverA, CL2797.Contig2\_Mf\_liverA, NM\_019879, Unigene2\_Mf\_liverA, CL532.Contig1\_Mf\_liverA, Unigene27082\_Mf\_liverA, CL1125.Contig1\_Mf\_liverA, Unigene14916\_Mf\_liverA, Unigene24225\_Mf\_liverA, NM\_013821 |
| organelle | Unigene21684\_Mf\_liverA, NM\_176843, CL4302.Contig1\_Mf\_liverA, NM\_009450, CL3669.Contig2\_Mf\_liverA, Unigene29011\_Mf\_liverA, NM\_025593, Unigene36851\_Mf\_liverA, NM\_080638, NM\_010378, Unigene15553\_Mf\_liverA, NM\_011082, Unigene19687\_Mf\_liverA, CL854.Contig1\_Mf\_liverA, CL4105.Contig1\_Mf\_liverA, Unigene7336\_Mf\_liverA, Unigene35507\_Mf\_liverA, Unigene6465\_Mf\_liverA, CL3617.Contig1\_Mf\_liverA, Unigene39886\_Mf\_liverA, Unigene15064\_Mf\_liverA, Unigene32920\_Mf\_liverA, Unigene34124\_Mf\_liverA, NR\_004446, Unigene36765\_Mf\_liverA, Unigene34341\_Mf\_liverA, Unigene30142\_Mf\_liverA, CL695.Contig1\_Mf\_liverA, Unigene34847\_Mf\_liverA, Unigene29082\_Mf\_liverA, Unigene2\_Mf\_liverA, CL4919.Contig1\_Mf\_liverA, Unigene31623\_Mf\_liverA, Unigene7897\_Mf\_liverA, NM\_145474, Unigene37084\_Mf\_liverA, Unigene20874\_Mf\_liverA, NM\_009776, Unigene33054\_Mf\_liverA, Unigene34394\_Mf\_liverA, NM\_031165, Unigene25594\_Mf\_liverA, CL497.Contig2\_Mf\_liverA, Unigene36430\_Mf\_liverA, CL6.Contig1\_Mf\_liverA, Unigene18848\_Mf\_liverA, NM\_016697, Unigene802\_Mf\_liverA, CL4816.Contig1\_Mf\_liverA, Unigene15055\_Mf\_liverA, Unigene5639\_Mf\_liverA, CL3154.Contig1\_Mf\_liverA, CL5807.Contig1\_Mf\_liverA, Unigene32594\_Mf\_liverA, Unigene13363\_Mf\_liverA, Unigene743\_Mf\_liverA, Unigene36417\_Mf\_liverA, CL2855.Contig2\_Mf\_liverA, CL4263.Contig1\_Mf\_liverA, NM\_021278, Unigene14907\_Mf\_liverA, Unigene1479\_Mf\_liverA, CL4249.Contig1\_Mf\_liverA, NM\_010001, Unigene25976\_Mf\_liverA, NM\_009338, Unigene44882\_Mf\_liverA, Unigene36698\_Mf\_liverA, Unigene139\_Mf\_liverA, Unigene31625\_Mf\_liverA, NM\_022324, Unigene34032\_Mf\_liverA, Unigene5057\_Mf\_liverA, Unigene27547\_Mf\_liverA, Unigene15290\_Mf\_liverA, Unigene24090\_Mf\_liverA, Unigene32789\_Mf\_liverA, Unigene36898\_Mf\_liverA, CL5316.Contig1\_Mf\_liverA, Unigene35664\_Mf\_liverA, CL1688.Contig3\_Mf\_liverA, NM\_027660, Unigene37389\_Mf\_liverA, Unigene32332\_Mf\_liverA, Unigene4723\_Mf\_liverA, NR\_003630, CL3930.Contig1\_Mf\_liverA, Unigene32852\_Mf\_liverA, Unigene24613\_Mf\_liverA, Unigene26611\_Mf\_liverA, CL532.Contig1\_Mf\_liverA, Unigene35169\_Mf\_liverA, Unigene1280\_Mf\_liverA, Unigene30288\_Mf\_liverA, CL3166.Contig4\_Mf\_liverA, NM\_027959, Unigene32059\_Mf\_liverA, Unigene34140\_Mf\_liverA, Unigene4363\_Mf\_liverA, Unigene836\_Mf\_liverA, CL186.Contig3\_Mf\_liverA, CL425.Contig1\_Mf\_liverA, CL2797.Contig2\_Mf\_liverA, Unigene30832\_Mf\_liverA, Unigene28143\_Mf\_liverA, Unigene14916\_Mf\_liverA, Unigene37243\_Mf\_liverA, Unigene30217\_Mf\_liverA, Unigene15077\_Mf\_liverA, Unigene2050\_Mf\_liverA, CL4600.Contig1\_Mf\_liverA, Unigene16184\_Mf\_liverA, CL3382.Contig1\_Mf\_liverA, Unigene5693\_Mf\_liverA, NM\_029582, Unigene29134\_Mf\_liverA, Unigene35958\_Mf\_liverA, Unigene29876\_Mf\_liverA, Unigene28687\_Mf\_liverA, Unigene32803\_Mf\_liverA, Unigene34375\_Mf\_liverA, CL1988.Contig3\_Mf\_liverA, CL1810.Contig1\_Mf\_liverA, NM\_172679, CL4033.Contig1\_Mf\_liverA, Unigene34983\_Mf\_liverA, Unigene28662\_Mf\_liverA, CL2439.Contig1\_Mf\_liverA, Unigene33330\_Mf\_liverA, Unigene6464\_Mf\_liverA, CL3933.Contig1\_Mf\_liverA, Unigene21026\_Mf\_liverA, NM\_011221, CL1493.Contig1\_Mf\_liverA, Unigene13683\_Mf\_liverA, Unigene9698\_Mf\_liverA, Unigene36420\_Mf\_liverA, Unigene542\_Mf\_liverA, Unigene35476\_Mf\_liverA, Unigene24758\_Mf\_liverA, Unigene24471\_Mf\_liverA, CL5828.Contig2\_Mf\_liverA, Unigene35168\_Mf\_liverA, Unigene33080\_Mf\_liverA, Unigene15888\_Mf\_liverA, CL4220.Contig1\_Mf\_liverA, Unigene15433\_Mf\_liverA, CL4338.Contig1\_Mf\_liverA, NM\_019817, CL5052.Contig1\_Mf\_liverA, NM\_010469, Unigene5750\_Mf\_liverA, NM\_019879, NR\_033215, NM\_007822, Unigene25595\_Mf\_liverA, Unigene14263\_Mf\_liverA, Unigene15470\_Mf\_liverA, Unigene14286\_Mf\_liverA, CL4816.Contig3\_Mf\_liverA, CL114.Contig1\_Mf\_liverA, Unigene39011\_Mf\_liverA, Unigene24225\_Mf\_liverA, Unigene37344\_Mf\_liverA, CL5275.Contig2\_Mf\_liverA, NM\_011207, NM\_145218, Unigene36189\_Mf\_liverA, Unigene29558\_Mf\_liverA, Unigene13950\_Mf\_liverA, Unigene28459\_Mf\_liverA, Unigene37245\_Mf\_liverA, Unigene281\_Mf\_liverA, NM\_177652, NM\_011978, Unigene10313\_Mf\_liverA, Unigene37104\_Mf\_liverA, CL3104.Contig1\_Mf\_liverA, Unigene36593\_Mf\_liverA, NM\_010002, NM\_013842, NM\_027406, Unigene16529\_Mf\_liverA, Unigene37232\_Mf\_liverA, NM\_026313, Unigene28609\_Mf\_liverA, CL5007.Contig1\_Mf\_liverA, CL5698.Contig1\_Mf\_liverA, Unigene14461\_Mf\_liverA, Unigene31392\_Mf\_liverA, CL591.Contig1\_Mf\_liverA, Unigene35618\_Mf\_liverA, NM\_011256, Unigene35884\_Mf\_liverA, Unigene38015\_Mf\_liverA, NM\_028291, NM\_173038, NM\_206537, CL3835.Contig2\_Mf\_liverA, Unigene31199\_Mf\_liverA, Unigene6959\_Mf\_liverA, CL4550.Contig2\_Mf\_liverA, Unigene28899\_Mf\_liverA, Unigene28244\_Mf\_liverA, Unigene46870\_Mf\_liverA, CL523.Contig1\_Mf\_liverA, NM\_008277, CL482.Contig1\_Mf\_liverA, Unigene27071\_Mf\_liverA, Unigene36762\_Mf\_liverA, Unigene600\_Mf\_liverA, NM\_011072, Unigene18795\_Mf\_liverA, Unigene30878\_Mf\_liverA, Unigene40289\_Mf\_liverA, NM\_134012, CL6038.Contig2\_Mf\_liverA, Unigene32570\_Mf\_liverA, Unigene37904\_Mf\_liverA, Unigene15295\_Mf\_liverA, Unigene12999\_Mf\_liverA, Unigene34789\_Mf\_liverA, CL4141.Contig1\_Mf\_liverA, Unigene24221\_Mf\_liverA, NM\_145419, CL5978.Contig3\_Mf\_liverA, CL1119.Contig1\_Mf\_liverA, Unigene13658\_Mf\_liverA, Unigene13593\_Mf\_liverA, NM\_134127, Unigene11\_Mf\_liverA, Unigene32546\_Mf\_liverA, Unigene14609\_Mf\_liverA, Unigene25596\_Mf\_liverA, Unigene37109\_Mf\_liverA, CL3778.Contig2\_Mf\_liverA, Unigene5994\_Mf\_liverA, CL1493.Contig2\_Mf\_liverA, CL2697.Contig4\_Mf\_liverA, Unigene5941\_Mf\_liverA, CL2574.Contig1\_Mf\_liverA, NM\_024434, NM\_008261, Unigene38104\_Mf\_liverA, Unigene23328\_Mf\_liverA, Unigene36626\_Mf\_liverA, CL787.Contig1\_Mf\_liverA, Unigene29490\_Mf\_liverA, Unigene36691\_Mf\_liverA, Unigene12967\_Mf\_liverA, NM\_001162917, Unigene31988\_Mf\_liverA, CL2389.Contig1\_Mf\_liverA, CL3750.Contig2\_Mf\_liverA, CL3599.Contig1\_Mf\_liverA, Unigene39141\_Mf\_liverA, CL848.Contig2\_Mf\_liverA, CL784.Contig3\_Mf\_liverA, Unigene31639\_Mf\_liverA, CL838.Contig3\_Mf\_liverA, Unigene10774\_Mf\_liverA, Unigene5632\_Mf\_liverA, CL1263.Contig1\_Mf\_liverA, CL4550.Contig3\_Mf\_liverA, Unigene21816\_Mf\_liverA, NM\_007811, NM\_008538, CL2142.Contig2\_Mf\_liverA, Unigene30428\_Mf\_liverA, NM\_011305, Unigene13498\_Mf\_liverA, CL529.Contig2\_Mf\_liverA, Unigene30584\_Mf\_liverA, Unigene4510\_Mf\_liverA, Unigene17579\_Mf\_liverA, NM\_001081372, CL913.Contig1\_Mf\_liverA, Unigene37148\_Mf\_liverA, Unigene34406\_Mf\_liverA, NM\_021525, Unigene7195\_Mf\_liverA, Unigene35506\_Mf\_liverA, Unigene32058\_Mf\_liverA, Unigene27082\_Mf\_liverA, Unigene37178\_Mf\_liverA, Unigene5712\_Mf\_liverA, CL2919.Contig1\_Mf\_liverA, Unigene1205\_Mf\_liverA, CL442.Contig2\_Mf\_liverA, CL6039.Contig1\_Mf\_liverA, CL2855.Contig1\_Mf\_liverA, CL4411.Contig4\_Mf\_liverA, NM\_013821, Unigene40796\_Mf\_liverA, Unigene33420\_Mf\_liverA, Unigene32412\_Mf\_liverA, NM\_001100182, Unigene31051\_Mf\_liverA, NM\_011732, Unigene30426\_Mf\_liverA, NM\_009178, Unigene49971\_Mf\_liverA, Unigene38307\_Mf\_liverA, Unigene36190\_Mf\_liverA, Unigene27406\_Mf\_liverA, Unigene34754\_Mf\_liverA, Unigene17042\_Mf\_liverA, Unigene3789\_Mf\_liverA, Unigene33746\_Mf\_liverA, CL1125.Contig1\_Mf\_liverA, CL4162.Contig1\_Mf\_liverA, Unigene27922\_Mf\_liverA, Unigene673\_Mf\_liverA, CL1100.Contig1\_Mf\_liverA, Unigene32093\_Mf\_liverA, CL4583.Contig2\_Mf\_liverA, Unigene36414\_Mf\_liverA, NM\_021423, CL485.Contig1\_Mf\_liverA, CL1988.Contig1\_Mf\_liverA, Unigene5175\_Mf\_liverA, Unigene36328\_Mf\_liverA, Unigene15552\_Mf\_liverA, Unigene38280\_Mf\_liverA, CL4745.Contig1\_Mf\_liverA, CL5309.Contig1\_Mf\_liverA, CL3002.Contig1\_Mf\_liverA, Unigene36315\_Mf\_liverA, Unigene31483\_Mf\_liverA, Unigene36949\_Mf\_liverA, CL887.Contig2\_Mf\_liverA, Unigene5488\_Mf\_liverA, CL2597.Contig1\_Mf\_liverA, Unigene33736\_Mf\_liverA, Unigene35037\_Mf\_liverA, Unigene14810\_Mf\_liverA, Unigene1212\_Mf\_liverA, NM\_001100181, Unigene32233\_Mf\_liverA, CL838.Contig6\_Mf\_liverA, Unigene5607\_Mf\_liverA, CL4770.Contig1\_Mf\_liverA, Unigene32143\_Mf\_liverA, Unigene24536\_Mf\_liverA, CL3750.Contig1\_Mf\_liverA, Unigene13196\_Mf\_liverA, CL114.Contig2\_Mf\_liverA, Unigene14449\_Mf\_liverA, CL1076.Contig1\_Mf\_liverA, NM\_009883, Unigene18796\_Mf\_liverA, NM\_009448, CL5307.Contig1\_Mf\_liverA, Unigene1327\_Mf\_liverA, Unigene31670\_Mf\_liverA, CL5978.Contig2\_Mf\_liverA, NM\_178405, NM\_172203, CL442.Contig5\_Mf\_liverA, Unigene120\_Mf\_liverA, Unigene36418\_Mf\_liverA, Unigene15026\_Mf\_liverA, CL4007.Contig1\_Mf\_liverA, NM\_022997, NM\_017379, CL55.Contig1\_Mf\_liverA, Unigene550\_Mf\_liverA, Unigene28489\_Mf\_liverA, Unigene37120\_Mf\_liverA, Unigene31198\_Mf\_liverA, CL4984.Contig1\_Mf\_liverA, Unigene24537\_Mf\_liverA, Unigene4863\_Mf\_liverA, CL2355.Contig1\_Mf\_liverA, Unigene32335\_Mf\_liverA, Unigene37153\_Mf\_liverA, Unigene14809\_Mf\_liverA, NM\_027604, Unigene5489\_Mf\_liverA, Unigene34444\_Mf\_liverA, Unigene7399\_Mf\_liverA, Unigene35678\_Mf\_liverA, Unigene33993\_Mf\_liverA, Unigene138\_Mf\_liverA, NM\_009011, Unigene8560\_Mf\_liverA, CL3911.Contig2\_Mf\_liverA, Unigene44057\_Mf\_liverA, Unigene5761\_Mf\_liverA, Unigene4681\_Mf\_liverA, NM\_008851, Unigene37819\_Mf\_liverA, Unigene5886\_Mf\_liverA, CL336.Contig3\_Mf\_liverA |
| cell fraction | NM\_011256, NM\_001100182, NM\_001081372, NM\_206537, NM\_001081274, NM\_011978, NR\_003630, NM\_010002, NM\_011921, NM\_010001, NM\_134127, NM\_178405, NM\_145474, NM\_008483, NM\_007822, NM\_031165, NM\_007811, NM\_011072, NM\_001100181, NR\_024097, NM\_008851, NM\_013821, NM\_008183 |
| centrosome | Unigene5057\_Mf\_liverA, CL1810.Contig1\_Mf\_liverA, Unigene673\_Mf\_liverA, Unigene13498\_Mf\_liverA, Unigene15295\_Mf\_liverA |
| organelle inner membrane | Unigene24225\_Mf\_liverA, CL3599.Contig1\_Mf\_liverA, CL1125.Contig1\_Mf\_liverA, CL2797.Contig2\_Mf\_liverA, NM\_013821, CL4984.Contig1\_Mf\_liverA, NM\_019879, CL5316.Contig1\_Mf\_liverA |
| organelle envelope | Unigene35476\_Mf\_liverA, Unigene36898\_Mf\_liverA, CL4984.Contig1\_Mf\_liverA, CL5316.Contig1\_Mf\_liverA, Unigene32335\_Mf\_liverA, Unigene29011\_Mf\_liverA, CL1810.Contig1\_Mf\_liverA, CL3599.Contig1\_Mf\_liverA, CL3104.Contig1\_Mf\_liverA, Unigene15433\_Mf\_liverA, NM\_080638, CL2797.Contig2\_Mf\_liverA, NM\_019879, Unigene2\_Mf\_liverA, Unigene27082\_Mf\_liverA, CL1125.Contig1\_Mf\_liverA, CL1263.Contig1\_Mf\_liverA, CL4162.Contig1\_Mf\_liverA, Unigene21816\_Mf\_liverA, Unigene24225\_Mf\_liverA, NM\_013821 |
| envelope | Unigene35476\_Mf\_liverA, Unigene36898\_Mf\_liverA, CL4984.Contig1\_Mf\_liverA, CL5316.Contig1\_Mf\_liverA, Unigene32335\_Mf\_liverA, Unigene29011\_Mf\_liverA, CL1810.Contig1\_Mf\_liverA, CL3599.Contig1\_Mf\_liverA, CL3104.Contig1\_Mf\_liverA, Unigene15433\_Mf\_liverA, NM\_080638, CL2797.Contig2\_Mf\_liverA, NM\_019879, Unigene2\_Mf\_liverA, Unigene27082\_Mf\_liverA, CL1125.Contig1\_Mf\_liverA, CL1263.Contig1\_Mf\_liverA, CL4162.Contig1\_Mf\_liverA, Unigene21816\_Mf\_liverA, Unigene24225\_Mf\_liverA, NM\_013821 |
| cytoskeletal part | Unigene5057\_Mf\_liverA, Unigene27547\_Mf\_liverA, Unigene5639\_Mf\_liverA, NM\_021423, CL4302.Contig1\_Mf\_liverA, Unigene32570\_Mf\_liverA, NM\_028291, Unigene32803\_Mf\_liverA, Unigene15295\_Mf\_liverA, Unigene34789\_Mf\_liverA, CL1810.Contig1\_Mf\_liverA, NM\_009448, CL425.Contig1\_Mf\_liverA, CL848.Contig2\_Mf\_liverA, Unigene13593\_Mf\_liverA, Unigene16529\_Mf\_liverA, Unigene32546\_Mf\_liverA, Unigene27082\_Mf\_liverA, Unigene26611\_Mf\_liverA, CL4162.Contig1\_Mf\_liverA, Unigene44057\_Mf\_liverA, Unigene14263\_Mf\_liverA, Unigene2050\_Mf\_liverA, NM\_008538, CL5698.Contig1\_Mf\_liverA, Unigene673\_Mf\_liverA, CL591.Contig1\_Mf\_liverA, Unigene13498\_Mf\_liverA, Unigene5941\_Mf\_liverA |
| nuclear chromosome part | Unigene14907\_Mf\_liverA, Unigene32594\_Mf\_liverA, Unigene31392\_Mf\_liverA |
| membrane-enclosed lumen | NM\_176843, Unigene35884\_Mf\_liverA, Unigene38015\_Mf\_liverA, Unigene30426\_Mf\_liverA, Unigene29011\_Mf\_liverA, NM\_025593, Unigene28899\_Mf\_liverA, Unigene36851\_Mf\_liverA, CL2797.Contig2\_Mf\_liverA, CL4105.Contig1\_Mf\_liverA, Unigene14916\_Mf\_liverA, CL1125.Contig1\_Mf\_liverA, CL4162.Contig1\_Mf\_liverA, Unigene600\_Mf\_liverA, Unigene15064\_Mf\_liverA, Unigene29134\_Mf\_liverA, Unigene34124\_Mf\_liverA, Unigene32093\_Mf\_liverA, Unigene35958\_Mf\_liverA, Unigene29876\_Mf\_liverA, CL6038.Contig2\_Mf\_liverA, CL4583.Contig2\_Mf\_liverA, Unigene36414\_Mf\_liverA, Unigene34375\_Mf\_liverA, CL5309.Contig1\_Mf\_liverA, CL1810.Contig1\_Mf\_liverA, Unigene29082\_Mf\_liverA, CL4141.Contig1\_Mf\_liverA, NM\_145419, Unigene28662\_Mf\_liverA, CL1119.Contig1\_Mf\_liverA, Unigene13658\_Mf\_liverA, Unigene20874\_Mf\_liverA, Unigene25596\_Mf\_liverA, Unigene37109\_Mf\_liverA, Unigene13683\_Mf\_liverA, Unigene32233\_Mf\_liverA, Unigene9698\_Mf\_liverA, NM\_008261, NM\_024434, Unigene36420\_Mf\_liverA, Unigene23328\_Mf\_liverA, Unigene24536\_Mf\_liverA, Unigene802\_Mf\_liverA, Unigene35476\_Mf\_liverA, Unigene15055\_Mf\_liverA, Unigene24758\_Mf\_liverA, Unigene14449\_Mf\_liverA, Unigene24471\_Mf\_liverA, CL1076.Contig1\_Mf\_liverA, Unigene32594\_Mf\_liverA, NM\_009883, Unigene36417\_Mf\_liverA, CL2855.Contig2\_Mf\_liverA, CL4263.Contig1\_Mf\_liverA, Unigene36691\_Mf\_liverA, CL3599.Contig1\_Mf\_liverA, CL4338.Contig1\_Mf\_liverA, Unigene14907\_Mf\_liverA, CL5052.Contig1\_Mf\_liverA, Unigene31639\_Mf\_liverA, Unigene1479\_Mf\_liverA, Unigene5750\_Mf\_liverA, NM\_019879, CL4249.Contig1\_Mf\_liverA, Unigene25976\_Mf\_liverA, NM\_009338, Unigene21816\_Mf\_liverA, Unigene25595\_Mf\_liverA, Unigene30428\_Mf\_liverA, NM\_011305, Unigene13498\_Mf\_liverA, Unigene36418\_Mf\_liverA, Unigene29558\_Mf\_liverA, Unigene5057\_Mf\_liverA, Unigene34032\_Mf\_liverA, Unigene24537\_Mf\_liverA, CL2355.Contig1\_Mf\_liverA, Unigene10313\_Mf\_liverA, Unigene37153\_Mf\_liverA, CL913.Contig1\_Mf\_liverA, Unigene34406\_Mf\_liverA, NM\_021525, Unigene14809\_Mf\_liverA, CL3930.Contig1\_Mf\_liverA, Unigene7399\_Mf\_liverA, NM\_009011, Unigene24613\_Mf\_liverA, NM\_026313, CL532.Contig1\_Mf\_liverA, CL3911.Contig2\_Mf\_liverA, Unigene31392\_Mf\_liverA, Unigene30288\_Mf\_liverA, CL2855.Contig1\_Mf\_liverA |
| macromolecular complex | NM\_007396, NM\_176843, Unigene35884\_Mf\_liverA, CL4302.Contig1\_Mf\_liverA, NM\_009450, CL3835.Contig2\_Mf\_liverA, NM\_011732, Unigene31199\_Mf\_liverA, Unigene6959\_Mf\_liverA, NM\_025593, CL425.Contig1\_Mf\_liverA, Unigene49971\_Mf\_liverA, Unigene28244\_Mf\_liverA, Unigene36851\_Mf\_liverA, Unigene9081\_Mf\_liverA, NM\_080638, NM\_010378, Unigene30815\_Mf\_liverA, Unigene36849\_Mf\_liverA, CL2339.Contig1\_Mf\_liverA, CL4162.Contig1\_Mf\_liverA, Unigene37526\_Mf\_liverA, Unigene27922\_Mf\_liverA, Unigene2050\_Mf\_liverA, CL3382.Contig1\_Mf\_liverA, Unigene30878\_Mf\_liverA, NR\_004446, Unigene18500\_Mf\_liverA, Unigene4938\_Mf\_liverA, Unigene32803\_Mf\_liverA, CL3002.Contig1\_Mf\_liverA, CL4745.Contig1\_Mf\_liverA, Unigene15295\_Mf\_liverA, Unigene34789\_Mf\_liverA, Unigene12999\_Mf\_liverA, CL1810.Contig1\_Mf\_liverA, CL4141.Contig1\_Mf\_liverA, Unigene5488\_Mf\_liverA, CL887.Contig2\_Mf\_liverA, CL2439.Contig1\_Mf\_liverA, CL1119.Contig1\_Mf\_liverA, Unigene13658\_Mf\_liverA, Unigene31333\_Mf\_liverA, Unigene33330\_Mf\_liverA, Unigene29426\_Mf\_liverA, Unigene33054\_Mf\_liverA, Unigene843\_Mf\_liverA, NM\_011221, NM\_031165, CL3684.Contig2\_Mf\_liverA, Unigene13683\_Mf\_liverA, Unigene33683\_Mf\_liverA, NM\_008261, Unigene5941\_Mf\_liverA, Unigene15055\_Mf\_liverA, Unigene5639\_Mf\_liverA, Unigene24471\_Mf\_liverA, CL3897.Contig5\_Mf\_liverA, Unigene32594\_Mf\_liverA, CL2855.Contig2\_Mf\_liverA, Unigene14907\_Mf\_liverA, Unigene15433\_Mf\_liverA, NM\_009448, NM\_019817, Unigene1479\_Mf\_liverA, CL4456.Contig1\_Mf\_liverA, NM\_019879, CL4249.Contig1\_Mf\_liverA, Unigene13143\_Mf\_liverA, Unigene5632\_Mf\_liverA, Unigene25976\_Mf\_liverA, NM\_008483, Unigene21816\_Mf\_liverA, Unigene36698\_Mf\_liverA, Unigene14263\_Mf\_liverA, Unigene8218\_Mf\_liverA, Unigene13498\_Mf\_liverA, Unigene15572\_Mf\_liverA, NM\_153589, Unigene13950\_Mf\_liverA, Unigene5057\_Mf\_liverA, Unigene34032\_Mf\_liverA, Unigene37245\_Mf\_liverA, Unigene27547\_Mf\_liverA, NM\_017379, Unigene31198\_Mf\_liverA, CL2355.Contig1\_Mf\_liverA, Unigene32335\_Mf\_liverA, Unigene37153\_Mf\_liverA, CL3104.Contig1\_Mf\_liverA, Unigene5489\_Mf\_liverA, Unigene34444\_Mf\_liverA, Unigene30814\_Mf\_liverA, NM\_027406, Unigene33993\_Mf\_liverA, CL5808.Contig1\_Mf\_liverA, Unigene24157\_Mf\_liverA, NM\_009011, Unigene37232\_Mf\_liverA, CL3911.Contig2\_Mf\_liverA, Unigene27082\_Mf\_liverA, CL532.Contig1\_Mf\_liverA, Unigene26611\_Mf\_liverA, CL5698.Contig1\_Mf\_liverA, Unigene31392\_Mf\_liverA, Unigene30288\_Mf\_liverA, Unigene1288\_Mf\_liverA, Unigene4681\_Mf\_liverA, CL275.Contig5\_Mf\_liverA, NM\_010380, CL2855.Contig1\_Mf\_liverA, CL591.Contig1\_Mf\_liverA, Unigene17569\_Mf\_liverA |
| organelle lumen | NM\_176843, Unigene35884\_Mf\_liverA, Unigene38015\_Mf\_liverA, Unigene30426\_Mf\_liverA, Unigene29011\_Mf\_liverA, NM\_025593, Unigene28899\_Mf\_liverA, Unigene36851\_Mf\_liverA, CL4105.Contig1\_Mf\_liverA, Unigene14916\_Mf\_liverA, CL1125.Contig1\_Mf\_liverA, CL4162.Contig1\_Mf\_liverA, Unigene600\_Mf\_liverA, Unigene15064\_Mf\_liverA, Unigene29134\_Mf\_liverA, Unigene34124\_Mf\_liverA, Unigene32093\_Mf\_liverA, Unigene35958\_Mf\_liverA, Unigene29876\_Mf\_liverA, CL6038.Contig2\_Mf\_liverA, CL4583.Contig2\_Mf\_liverA, Unigene36414\_Mf\_liverA, Unigene34375\_Mf\_liverA, CL5309.Contig1\_Mf\_liverA, CL1810.Contig1\_Mf\_liverA, Unigene29082\_Mf\_liverA, CL4141.Contig1\_Mf\_liverA, NM\_145419, Unigene28662\_Mf\_liverA, CL1119.Contig1\_Mf\_liverA, Unigene13658\_Mf\_liverA, Unigene20874\_Mf\_liverA, Unigene25596\_Mf\_liverA, Unigene37109\_Mf\_liverA, Unigene13683\_Mf\_liverA, Unigene32233\_Mf\_liverA, Unigene9698\_Mf\_liverA, NM\_008261, NM\_024434, Unigene36420\_Mf\_liverA, Unigene23328\_Mf\_liverA, Unigene24536\_Mf\_liverA, Unigene802\_Mf\_liverA, Unigene15055\_Mf\_liverA, Unigene24758\_Mf\_liverA, Unigene14449\_Mf\_liverA, Unigene24471\_Mf\_liverA, CL1076.Contig1\_Mf\_liverA, Unigene32594\_Mf\_liverA, NM\_009883, Unigene36417\_Mf\_liverA, CL2855.Contig2\_Mf\_liverA, CL4263.Contig1\_Mf\_liverA, Unigene36691\_Mf\_liverA, CL3599.Contig1\_Mf\_liverA, CL4338.Contig1\_Mf\_liverA, Unigene14907\_Mf\_liverA, CL5052.Contig1\_Mf\_liverA, Unigene31639\_Mf\_liverA, Unigene1479\_Mf\_liverA, Unigene5750\_Mf\_liverA, NM\_019879, CL4249.Contig1\_Mf\_liverA, Unigene25976\_Mf\_liverA, NM\_009338, Unigene21816\_Mf\_liverA, Unigene25595\_Mf\_liverA, Unigene30428\_Mf\_liverA, NM\_011305, Unigene13498\_Mf\_liverA, Unigene36418\_Mf\_liverA, Unigene29558\_Mf\_liverA, Unigene5057\_Mf\_liverA, Unigene34032\_Mf\_liverA, Unigene24537\_Mf\_liverA, CL2355.Contig1\_Mf\_liverA, Unigene10313\_Mf\_liverA, Unigene37153\_Mf\_liverA, CL913.Contig1\_Mf\_liverA, Unigene34406\_Mf\_liverA, NM\_021525, Unigene14809\_Mf\_liverA, CL3930.Contig1\_Mf\_liverA, Unigene7399\_Mf\_liverA, NM\_009011, Unigene24613\_Mf\_liverA, NM\_026313, CL532.Contig1\_Mf\_liverA, CL3911.Contig2\_Mf\_liverA, Unigene31392\_Mf\_liverA, Unigene30288\_Mf\_liverA, CL2855.Contig1\_Mf\_liverA |
| chromatin | Unigene14907\_Mf\_liverA, Unigene32594\_Mf\_liverA, NM\_009883, NM\_011305 |
| cytoskeleton | Unigene5639\_Mf\_liverA, CL4302.Contig1\_Mf\_liverA, NM\_009450, NM\_028291, Unigene35168\_Mf\_liverA, NM\_009448, CL425.Contig1\_Mf\_liverA, Unigene1327\_Mf\_liverA, CL848.Contig2\_Mf\_liverA, Unigene3789\_Mf\_liverA, CL4162.Contig1\_Mf\_liverA, Unigene14263\_Mf\_liverA, Unigene2050\_Mf\_liverA, NM\_008538, Unigene16184\_Mf\_liverA, NM\_011072, Unigene5693\_Mf\_liverA, Unigene673\_Mf\_liverA, Unigene13498\_Mf\_liverA, Unigene31625\_Mf\_liverA, Unigene5057\_Mf\_liverA, NM\_017379, NM\_021423, Unigene550\_Mf\_liverA, Unigene27547\_Mf\_liverA, Unigene28489\_Mf\_liverA, Unigene32570\_Mf\_liverA, Unigene32803\_Mf\_liverA, Unigene4863\_Mf\_liverA, Unigene15295\_Mf\_liverA, NM\_027660, CL1810.Contig1\_Mf\_liverA, Unigene34847\_Mf\_liverA, Unigene34789\_Mf\_liverA, Unigene31623\_Mf\_liverA, Unigene13593\_Mf\_liverA, Unigene16529\_Mf\_liverA, Unigene32546\_Mf\_liverA, Unigene26611\_Mf\_liverA, Unigene27082\_Mf\_liverA, Unigene44057\_Mf\_liverA, Unigene35169\_Mf\_liverA, CL5698.Contig1\_Mf\_liverA, Unigene36430\_Mf\_liverA, CL591.Contig1\_Mf\_liverA, Unigene5941\_Mf\_liverA |
| nucleus | Unigene21684\_Mf\_liverA, NM\_176843, Unigene35884\_Mf\_liverA, Unigene38015\_Mf\_liverA, CL3835.Contig2\_Mf\_liverA, Unigene29011\_Mf\_liverA, NM\_025593, Unigene28899\_Mf\_liverA, Unigene36851\_Mf\_liverA, Unigene46870\_Mf\_liverA, NM\_080638, Unigene15553\_Mf\_liverA, Unigene19687\_Mf\_liverA, CL4105.Contig1\_Mf\_liverA, CL482.Contig1\_Mf\_liverA, Unigene6465\_Mf\_liverA, CL3617.Contig1\_Mf\_liverA, Unigene18795\_Mf\_liverA, Unigene39886\_Mf\_liverA, Unigene15064\_Mf\_liverA, Unigene40289\_Mf\_liverA, Unigene37904\_Mf\_liverA, Unigene30142\_Mf\_liverA, Unigene15295\_Mf\_liverA, CL4141.Contig1\_Mf\_liverA, Unigene24221\_Mf\_liverA, NM\_145419, CL1119.Contig1\_Mf\_liverA, Unigene13658\_Mf\_liverA, Unigene7897\_Mf\_liverA, Unigene37084\_Mf\_liverA, Unigene20874\_Mf\_liverA, Unigene33054\_Mf\_liverA, Unigene14609\_Mf\_liverA, Unigene37109\_Mf\_liverA, CL497.Contig2\_Mf\_liverA, CL3778.Contig2\_Mf\_liverA, Unigene36430\_Mf\_liverA, Unigene5994\_Mf\_liverA, NM\_008261, NM\_024434, Unigene802\_Mf\_liverA, Unigene15055\_Mf\_liverA, Unigene32594\_Mf\_liverA, Unigene36417\_Mf\_liverA, Unigene14907\_Mf\_liverA, Unigene39141\_Mf\_liverA, Unigene31639\_Mf\_liverA, Unigene1479\_Mf\_liverA, CL838.Contig3\_Mf\_liverA, Unigene10774\_Mf\_liverA, CL4249.Contig1\_Mf\_liverA, Unigene25976\_Mf\_liverA, NM\_009338, CL1263.Contig1\_Mf\_liverA, Unigene21816\_Mf\_liverA, Unigene44882\_Mf\_liverA, NM\_008538, Unigene30428\_Mf\_liverA, NM\_011305, Unigene17579\_Mf\_liverA, Unigene5057\_Mf\_liverA, Unigene36898\_Mf\_liverA, Unigene37148\_Mf\_liverA, NM\_021525, CL3930.Contig1\_Mf\_liverA, Unigene32852\_Mf\_liverA, Unigene24613\_Mf\_liverA, Unigene37178\_Mf\_liverA, CL2919.Contig1\_Mf\_liverA, Unigene30288\_Mf\_liverA, Unigene33420\_Mf\_liverA, Unigene32412\_Mf\_liverA, Unigene34140\_Mf\_liverA, NM\_011732, Unigene30426\_Mf\_liverA, Unigene836\_Mf\_liverA, CL186.Contig3\_Mf\_liverA, Unigene38307\_Mf\_liverA, Unigene27406\_Mf\_liverA, Unigene17042\_Mf\_liverA, Unigene33746\_Mf\_liverA, Unigene14916\_Mf\_liverA, CL4162.Contig1\_Mf\_liverA, Unigene673\_Mf\_liverA, Unigene5693\_Mf\_liverA, Unigene29134\_Mf\_liverA, Unigene32093\_Mf\_liverA, Unigene35958\_Mf\_liverA, Unigene29876\_Mf\_liverA, CL485.Contig1\_Mf\_liverA, Unigene36414\_Mf\_liverA, Unigene5175\_Mf\_liverA, Unigene28687\_Mf\_liverA, CL3002.Contig1\_Mf\_liverA, CL4745.Contig1\_Mf\_liverA, Unigene15552\_Mf\_liverA, Unigene31483\_Mf\_liverA, CL1810.Contig1\_Mf\_liverA, Unigene34983\_Mf\_liverA, CL2439.Contig1\_Mf\_liverA, CL2597.Contig1\_Mf\_liverA, Unigene33330\_Mf\_liverA, Unigene6464\_Mf\_liverA, Unigene13683\_Mf\_liverA, Unigene1212\_Mf\_liverA, Unigene32233\_Mf\_liverA, CL838.Contig6\_Mf\_liverA, Unigene9698\_Mf\_liverA, CL4770.Contig1\_Mf\_liverA, Unigene36420\_Mf\_liverA, Unigene32143\_Mf\_liverA, Unigene24536\_Mf\_liverA, Unigene24758\_Mf\_liverA, Unigene35476\_Mf\_liverA, Unigene24471\_Mf\_liverA, Unigene14449\_Mf\_liverA, Unigene33080\_Mf\_liverA, NM\_009883, Unigene15433\_Mf\_liverA, CL4338.Contig1\_Mf\_liverA, Unigene18796\_Mf\_liverA, Unigene39011\_Mf\_liverA, Unigene15026\_Mf\_liverA, Unigene36418\_Mf\_liverA, Unigene13950\_Mf\_liverA, Unigene24537\_Mf\_liverA, Unigene4863\_Mf\_liverA, CL2355.Contig1\_Mf\_liverA, Unigene32335\_Mf\_liverA, Unigene10313\_Mf\_liverA, Unigene37153\_Mf\_liverA, Unigene7399\_Mf\_liverA, Unigene35678\_Mf\_liverA, Unigene33993\_Mf\_liverA, NM\_009011, Unigene8560\_Mf\_liverA, NM\_026313, Unigene28609\_Mf\_liverA, Unigene14461\_Mf\_liverA, Unigene31392\_Mf\_liverA |
| microtubule organizing center | Unigene5057\_Mf\_liverA, CL1810.Contig1\_Mf\_liverA, Unigene673\_Mf\_liverA, NM\_028291, Unigene13498\_Mf\_liverA, NM\_008538, Unigene15295\_Mf\_liverA |
| transcription factor complex | Unigene14907\_Mf\_liverA, Unigene13658\_Mf\_liverA |
| nuclear chromosome | Unigene14907\_Mf\_liverA, Unigene32594\_Mf\_liverA, Unigene31392\_Mf\_liverA |
| intracellular organelle lumen | NM\_176843, Unigene35884\_Mf\_liverA, Unigene38015\_Mf\_liverA, Unigene30426\_Mf\_liverA, Unigene29011\_Mf\_liverA, NM\_025593, Unigene28899\_Mf\_liverA, Unigene36851\_Mf\_liverA, CL4105.Contig1\_Mf\_liverA, Unigene14916\_Mf\_liverA, CL1125.Contig1\_Mf\_liverA, CL4162.Contig1\_Mf\_liverA, Unigene600\_Mf\_liverA, Unigene15064\_Mf\_liverA, Unigene29134\_Mf\_liverA, Unigene34124\_Mf\_liverA, Unigene32093\_Mf\_liverA, Unigene35958\_Mf\_liverA, Unigene29876\_Mf\_liverA, CL4583.Contig2\_Mf\_liverA, Unigene36414\_Mf\_liverA, Unigene34375\_Mf\_liverA, CL5309.Contig1\_Mf\_liverA, CL1810.Contig1\_Mf\_liverA, Unigene29082\_Mf\_liverA, CL4141.Contig1\_Mf\_liverA, NM\_145419, CL1119.Contig1\_Mf\_liverA, Unigene13658\_Mf\_liverA, Unigene20874\_Mf\_liverA, Unigene25596\_Mf\_liverA, Unigene37109\_Mf\_liverA, Unigene13683\_Mf\_liverA, Unigene32233\_Mf\_liverA, Unigene9698\_Mf\_liverA, NM\_008261, NM\_024434, Unigene36420\_Mf\_liverA, Unigene23328\_Mf\_liverA, Unigene24536\_Mf\_liverA, Unigene802\_Mf\_liverA, Unigene15055\_Mf\_liverA, Unigene24758\_Mf\_liverA, Unigene14449\_Mf\_liverA, Unigene24471\_Mf\_liverA, CL1076.Contig1\_Mf\_liverA, Unigene32594\_Mf\_liverA, NM\_009883, Unigene36417\_Mf\_liverA, CL2855.Contig2\_Mf\_liverA, CL4263.Contig1\_Mf\_liverA, Unigene36691\_Mf\_liverA, CL3599.Contig1\_Mf\_liverA, CL4338.Contig1\_Mf\_liverA, Unigene14907\_Mf\_liverA, CL5052.Contig1\_Mf\_liverA, Unigene31639\_Mf\_liverA, Unigene1479\_Mf\_liverA, NM\_019879, CL4249.Contig1\_Mf\_liverA, Unigene25976\_Mf\_liverA, NM\_009338, Unigene21816\_Mf\_liverA, Unigene25595\_Mf\_liverA, Unigene30428\_Mf\_liverA, NM\_011305, Unigene13498\_Mf\_liverA, Unigene36418\_Mf\_liverA, Unigene29558\_Mf\_liverA, Unigene5057\_Mf\_liverA, Unigene34032\_Mf\_liverA, Unigene24537\_Mf\_liverA, CL2355.Contig1\_Mf\_liverA, Unigene10313\_Mf\_liverA, Unigene37153\_Mf\_liverA, CL913.Contig1\_Mf\_liverA, Unigene34406\_Mf\_liverA, NM\_021525, CL3930.Contig1\_Mf\_liverA, Unigene7399\_Mf\_liverA, NM\_009011, Unigene24613\_Mf\_liverA, NM\_026313, CL532.Contig1\_Mf\_liverA, CL3911.Contig2\_Mf\_liverA, Unigene31392\_Mf\_liverA, Unigene30288\_Mf\_liverA, CL2855.Contig1\_Mf\_liverA |
| nucleoplasm | Unigene5057\_Mf\_liverA, Unigene15055\_Mf\_liverA, NM\_176843, Unigene35884\_Mf\_liverA, Unigene24471\_Mf\_liverA, Unigene38015\_Mf\_liverA, CL2355.Contig1\_Mf\_liverA, Unigene32594\_Mf\_liverA, Unigene37153\_Mf\_liverA, NM\_025593, CL4141.Contig1\_Mf\_liverA, Unigene14907\_Mf\_liverA, CL3930.Contig1\_Mf\_liverA, Unigene36851\_Mf\_liverA, Unigene1479\_Mf\_liverA, CL1119.Contig1\_Mf\_liverA, CL4249.Contig1\_Mf\_liverA, Unigene13658\_Mf\_liverA, CL4105.Contig1\_Mf\_liverA, Unigene25976\_Mf\_liverA, NM\_026313, CL4162.Contig1\_Mf\_liverA, Unigene31392\_Mf\_liverA, Unigene30288\_Mf\_liverA, Unigene15064\_Mf\_liverA, Unigene29134\_Mf\_liverA |
| nuclear body | Unigene5057\_Mf\_liverA, NM\_026313, CL2355.Contig1\_Mf\_liverA |
| ribonucleoprotein complex | Unigene33330\_Mf\_liverA, Unigene31198\_Mf\_liverA, CL4745.Contig1\_Mf\_liverA, NM\_011732, Unigene31199\_Mf\_liverA, Unigene13683\_Mf\_liverA, CL1810.Contig1\_Mf\_liverA, Unigene12999\_Mf\_liverA, Unigene33993\_Mf\_liverA |
| microtubule cytoskeleton | Unigene5057\_Mf\_liverA, Unigene27547\_Mf\_liverA, NM\_017379, NM\_009450, NM\_028291, Unigene14263\_Mf\_liverA, NM\_008538, NM\_027660, Unigene15295\_Mf\_liverA, CL1810.Contig1\_Mf\_liverA, NM\_009448, Unigene673\_Mf\_liverA, CL425.Contig1\_Mf\_liverA, Unigene13498\_Mf\_liverA |
| non-membrane-bounded organelle | NM\_011256, CL4302.Contig1\_Mf\_liverA, NM\_009450, NM\_028291, NM\_173038, Unigene30426\_Mf\_liverA, Unigene31199\_Mf\_liverA, Unigene28899\_Mf\_liverA, CL425.Contig1\_Mf\_liverA, Unigene3789\_Mf\_liverA, Unigene14916\_Mf\_liverA, CL4162.Contig1\_Mf\_liverA, Unigene2050\_Mf\_liverA, Unigene16184\_Mf\_liverA, NM\_011072, Unigene5693\_Mf\_liverA, Unigene673\_Mf\_liverA, Unigene32093\_Mf\_liverA, Unigene35958\_Mf\_liverA, Unigene29876\_Mf\_liverA, Unigene36414\_Mf\_liverA, NM\_021423, Unigene32570\_Mf\_liverA, Unigene32803\_Mf\_liverA, CL4745.Contig1\_Mf\_liverA, Unigene15295\_Mf\_liverA, Unigene34789\_Mf\_liverA, Unigene34847\_Mf\_liverA, Unigene12999\_Mf\_liverA, CL1810.Contig1\_Mf\_liverA, Unigene31623\_Mf\_liverA, Unigene13593\_Mf\_liverA, Unigene33330\_Mf\_liverA, Unigene32546\_Mf\_liverA, Unigene20874\_Mf\_liverA, Unigene37109\_Mf\_liverA, Unigene36430\_Mf\_liverA, Unigene13683\_Mf\_liverA, Unigene32233\_Mf\_liverA, Unigene9698\_Mf\_liverA, Unigene36420\_Mf\_liverA, Unigene5941\_Mf\_liverA, Unigene24536\_Mf\_liverA, Unigene802\_Mf\_liverA, Unigene24758\_Mf\_liverA, Unigene5639\_Mf\_liverA, Unigene14449\_Mf\_liverA, Unigene35168\_Mf\_liverA, Unigene32594\_Mf\_liverA, NM\_009883, Unigene36417\_Mf\_liverA, NM\_021278, CL4338.Contig1\_Mf\_liverA, Unigene14907\_Mf\_liverA, NM\_009448, Unigene1327\_Mf\_liverA, CL848.Contig2\_Mf\_liverA, Unigene31639\_Mf\_liverA, Unigene1479\_Mf\_liverA, Unigene21816\_Mf\_liverA, Unigene14263\_Mf\_liverA, NM\_008538, Unigene30428\_Mf\_liverA, NM\_011305, NM\_011207, Unigene13498\_Mf\_liverA, Unigene36418\_Mf\_liverA, Unigene31625\_Mf\_liverA, Unigene5057\_Mf\_liverA, NM\_017379, Unigene550\_Mf\_liverA, Unigene27547\_Mf\_liverA, Unigene28489\_Mf\_liverA, Unigene31198\_Mf\_liverA, Unigene32789\_Mf\_liverA, Unigene24537\_Mf\_liverA, Unigene4863\_Mf\_liverA, Unigene10313\_Mf\_liverA, NM\_027660, Unigene7399\_Mf\_liverA, Unigene16529\_Mf\_liverA, Unigene24613\_Mf\_liverA, Unigene26611\_Mf\_liverA, Unigene27082\_Mf\_liverA, Unigene44057\_Mf\_liverA, Unigene35169\_Mf\_liverA, CL5698.Contig1\_Mf\_liverA, Unigene31392\_Mf\_liverA, CL591.Contig1\_Mf\_liverA |
| intracellular non-membrane-bounded organelle | NM\_011256, CL4302.Contig1\_Mf\_liverA, NM\_009450, NM\_028291, NM\_173038, Unigene30426\_Mf\_liverA, Unigene31199\_Mf\_liverA, Unigene28899\_Mf\_liverA, CL425.Contig1\_Mf\_liverA, Unigene3789\_Mf\_liverA, Unigene14916\_Mf\_liverA, CL4162.Contig1\_Mf\_liverA, Unigene2050\_Mf\_liverA, Unigene16184\_Mf\_liverA, NM\_011072, Unigene5693\_Mf\_liverA, Unigene673\_Mf\_liverA, Unigene32093\_Mf\_liverA, Unigene35958\_Mf\_liverA, Unigene29876\_Mf\_liverA, Unigene36414\_Mf\_liverA, NM\_021423, Unigene32570\_Mf\_liverA, Unigene32803\_Mf\_liverA, CL4745.Contig1\_Mf\_liverA, Unigene15295\_Mf\_liverA, Unigene34789\_Mf\_liverA, Unigene34847\_Mf\_liverA, Unigene12999\_Mf\_liverA, CL1810.Contig1\_Mf\_liverA, Unigene31623\_Mf\_liverA, Unigene13593\_Mf\_liverA, Unigene33330\_Mf\_liverA, Unigene32546\_Mf\_liverA, Unigene20874\_Mf\_liverA, Unigene37109\_Mf\_liverA, Unigene36430\_Mf\_liverA, Unigene13683\_Mf\_liverA, Unigene32233\_Mf\_liverA, Unigene9698\_Mf\_liverA, Unigene36420\_Mf\_liverA, Unigene5941\_Mf\_liverA, Unigene24536\_Mf\_liverA, Unigene802\_Mf\_liverA, Unigene24758\_Mf\_liverA, Unigene5639\_Mf\_liverA, Unigene14449\_Mf\_liverA, Unigene35168\_Mf\_liverA, Unigene32594\_Mf\_liverA, NM\_009883, Unigene36417\_Mf\_liverA, NM\_021278, CL4338.Contig1\_Mf\_liverA, Unigene14907\_Mf\_liverA, NM\_009448, Unigene1327\_Mf\_liverA, CL848.Contig2\_Mf\_liverA, Unigene31639\_Mf\_liverA, Unigene1479\_Mf\_liverA, Unigene21816\_Mf\_liverA, Unigene14263\_Mf\_liverA, NM\_008538, Unigene30428\_Mf\_liverA, NM\_011305, NM\_011207, Unigene13498\_Mf\_liverA, Unigene36418\_Mf\_liverA, Unigene31625\_Mf\_liverA, Unigene5057\_Mf\_liverA, NM\_017379, Unigene550\_Mf\_liverA, Unigene27547\_Mf\_liverA, Unigene28489\_Mf\_liverA, Unigene31198\_Mf\_liverA, Unigene32789\_Mf\_liverA, Unigene24537\_Mf\_liverA, Unigene4863\_Mf\_liverA, Unigene10313\_Mf\_liverA, NM\_027660, Unigene7399\_Mf\_liverA, Unigene16529\_Mf\_liverA, Unigene24613\_Mf\_liverA, Unigene26611\_Mf\_liverA, Unigene27082\_Mf\_liverA, Unigene44057\_Mf\_liverA, Unigene35169\_Mf\_liverA, CL5698.Contig1\_Mf\_liverA, Unigene31392\_Mf\_liverA, CL591.Contig1\_Mf\_liverA |
| nucleoplasm part | Unigene5057\_Mf\_liverA, NM\_026313, NM\_176843, CL2355.Contig1\_Mf\_liverA, Unigene30288\_Mf\_liverA, NM\_025593, Unigene14907\_Mf\_liverA, Unigene1479\_Mf\_liverA, Unigene13658\_Mf\_liverA, CL4249.Contig1\_Mf\_liverA |
| chromosomal part | Unigene14907\_Mf\_liverA, Unigene21816\_Mf\_liverA, Unigene32594\_Mf\_liverA, NM\_009883, NM\_011305, Unigene31392\_Mf\_liverA |
| nuclear lumen | Unigene24758\_Mf\_liverA, Unigene15055\_Mf\_liverA, NM\_176843, Unigene35884\_Mf\_liverA, Unigene24471\_Mf\_liverA, Unigene38015\_Mf\_liverA, Unigene14449\_Mf\_liverA, Unigene32594\_Mf\_liverA, Unigene30426\_Mf\_liverA, Unigene36417\_Mf\_liverA, NM\_009883, NM\_025593, Unigene28899\_Mf\_liverA, Unigene14907\_Mf\_liverA, CL4338.Contig1\_Mf\_liverA, Unigene31639\_Mf\_liverA, Unigene36851\_Mf\_liverA, Unigene1479\_Mf\_liverA, CL4249.Contig1\_Mf\_liverA, CL4105.Contig1\_Mf\_liverA, Unigene25976\_Mf\_liverA, NM\_009338, Unigene14916\_Mf\_liverA, CL4162.Contig1\_Mf\_liverA, Unigene21816\_Mf\_liverA, NM\_011305, Unigene30428\_Mf\_liverA, Unigene15064\_Mf\_liverA, Unigene36418\_Mf\_liverA, Unigene29134\_Mf\_liverA, Unigene32093\_Mf\_liverA, Unigene35958\_Mf\_liverA, Unigene29876\_Mf\_liverA, Unigene5057\_Mf\_liverA, Unigene36414\_Mf\_liverA, CL2355.Contig1\_Mf\_liverA, Unigene24537\_Mf\_liverA, Unigene10313\_Mf\_liverA, Unigene37153\_Mf\_liverA, CL1810.Contig1\_Mf\_liverA, NM\_021525, CL4141.Contig1\_Mf\_liverA, NM\_145419, CL3930.Contig1\_Mf\_liverA, CL1119.Contig1\_Mf\_liverA, Unigene7399\_Mf\_liverA, Unigene13658\_Mf\_liverA, NM\_009011, Unigene24613\_Mf\_liverA, NM\_026313, Unigene20874\_Mf\_liverA, Unigene37109\_Mf\_liverA, Unigene31392\_Mf\_liverA, Unigene30288\_Mf\_liverA, Unigene13683\_Mf\_liverA, Unigene32233\_Mf\_liverA, Unigene9698\_Mf\_liverA, NM\_008261, NM\_024434, Unigene36420\_Mf\_liverA, Unigene24536\_Mf\_liverA, Unigene802\_Mf\_liverA |
| chromosome | Unigene14907\_Mf\_liverA, Unigene21816\_Mf\_liverA, Unigene32594\_Mf\_liverA, NM\_009883, NM\_011305, Unigene31392\_Mf\_liverA |
| nuclear part | NM\_176843, Unigene35884\_Mf\_liverA, Unigene38015\_Mf\_liverA, NM\_011732, Unigene30426\_Mf\_liverA, Unigene29011\_Mf\_liverA, NM\_025593, Unigene28899\_Mf\_liverA, Unigene36851\_Mf\_liverA, NM\_080638, CL4105.Contig1\_Mf\_liverA, Unigene14916\_Mf\_liverA, CL4162.Contig1\_Mf\_liverA, Unigene15064\_Mf\_liverA, Unigene29134\_Mf\_liverA, Unigene32093\_Mf\_liverA, Unigene35958\_Mf\_liverA, Unigene29876\_Mf\_liverA, Unigene36414\_Mf\_liverA, CL1810.Contig1\_Mf\_liverA, CL4141.Contig1\_Mf\_liverA, NM\_145419, CL1119.Contig1\_Mf\_liverA, Unigene13658\_Mf\_liverA, Unigene20874\_Mf\_liverA, Unigene37109\_Mf\_liverA, Unigene13683\_Mf\_liverA, Unigene32233\_Mf\_liverA, Unigene9698\_Mf\_liverA, NM\_008261, NM\_024434, Unigene36420\_Mf\_liverA, Unigene24536\_Mf\_liverA, Unigene802\_Mf\_liverA, Unigene15055\_Mf\_liverA, Unigene24758\_Mf\_liverA, Unigene14449\_Mf\_liverA, Unigene24471\_Mf\_liverA, Unigene32594\_Mf\_liverA, NM\_009883, Unigene36417\_Mf\_liverA, CL4338.Contig1\_Mf\_liverA, Unigene14907\_Mf\_liverA, Unigene15433\_Mf\_liverA, Unigene31639\_Mf\_liverA, Unigene1479\_Mf\_liverA, CL4249.Contig1\_Mf\_liverA, Unigene25976\_Mf\_liverA, NM\_009338, CL1263.Contig1\_Mf\_liverA, Unigene21816\_Mf\_liverA, Unigene30428\_Mf\_liverA, NM\_011305, Unigene36418\_Mf\_liverA, Unigene5057\_Mf\_liverA, Unigene36898\_Mf\_liverA, Unigene24537\_Mf\_liverA, CL2355.Contig1\_Mf\_liverA, Unigene32335\_Mf\_liverA, Unigene10313\_Mf\_liverA, Unigene37153\_Mf\_liverA, NM\_021525, CL3930.Contig1\_Mf\_liverA, Unigene7399\_Mf\_liverA, Unigene33993\_Mf\_liverA, NM\_009011, Unigene24613\_Mf\_liverA, NM\_026313, Unigene31392\_Mf\_liverA, Unigene30288\_Mf\_liverA |
|
