# Supplementary material for: De novo assembly and transcriptome characterization: novel insights into the natural resistance mechanisms of Microtus fortis against Schistosoma japonicum
Source: BMC Genomics. 2014 Jun 2;15(1):417. doi: 10.1186/1471-2164-15-417 (PMC4073500; doi:10.1186/1471-2164-15-417)
Supplement: Supplementary file 7 — Additional file 7: Dataset S5: GO terms for MfA-VS-MfA4W_C. (ZIP 26 KB) [file 12864_2013_6159_MOESM7_ESM.zip › 1990354100108772_add7.html]

Terms for MfA-VS-MfA4W\_C


## Terms for MfA-VS-MfA4W\_C

---


### Result Table

|  |
| --- |
| **Terms from the Component Ontology with p-value as good or better than 1** |

| Gene Ontology term | Cluster frequency | Genome frequency of use | Corrected P-value | Expression Profile |
| --- | --- | --- | --- | --- |
| extracellular space | 55 out of 412 genes, 13.3% | 1015 out of 37603 genes, 2.7% | 3.82e-20 | View Result |
| extracellular region | 90 out of 412 genes, 21.8% | 2987 out of 37603 genes, 7.9% | 1.98e-16 | View Result |
| nuclear outer membrane-endoplasmic reticulum membrane network | 47 out of 412 genes, 11.4% | 1173 out of 37603 genes, 3.1% | 5.90e-12 | View Result |
| endoplasmic reticulum part | 52 out of 412 genes, 12.6% | 1424 out of 37603 genes, 3.8% | 8.70e-12 | View Result |
| endoplasmic reticulum membrane | 46 out of 412 genes, 11.2% | 1142 out of 37603 genes, 3.0% | 9.43e-12 | View Result |
| cytoplasm | 265 out of 412 genes, 64.3% | 17507 out of 37603 genes, 46.6% | 5.48e-11 | View Result |
| endoplasmic reticulum | 62 out of 412 genes, 15.0% | 2107 out of 37603 genes, 5.6% | 3.48e-10 | View Result |
| extracellular region part | 65 out of 412 genes, 15.8% | 2343 out of 37603 genes, 6.2% | 1.22e-09 | View Result |
| integral to membrane | 126 out of 412 genes, 30.6% | 6371 out of 37603 genes, 16.9% | 1.25e-09 | View Result |
| cell periphery | 116 out of 412 genes, 28.2% | 6038 out of 37603 genes, 16.1% | 8.00e-08 | View Result |
| plasma membrane | 114 out of 412 genes, 27.7% | 5905 out of 37603 genes, 15.7% | 9.09e-08 | View Result |
| apical plasma membrane | 21 out of 412 genes, 5.1% | 360 out of 37603 genes, 1.0% | 1.69e-07 | View Result |
| cytoplasmic part | 218 out of 412 genes, 52.9% | 14592 out of 37603 genes, 38.8% | 9.06e-07 | View Result |
| apical part of cell | 23 out of 412 genes, 5.6% | 488 out of 37603 genes, 1.3% | 1.56e-06 | View Result |
| organelle membrane | 94 out of 412 genes, 22.8% | 4856 out of 37603 genes, 12.9% | 4.82e-06 | View Result |
| intercellular canaliculus | 5 out of 412 genes, 1.2% | 14 out of 37603 genes, 0.0% | 6.71e-05 | View Result |
| cell surface | 29 out of 412 genes, 7.0% | 1006 out of 37603 genes, 2.7% | 0.00065 | View Result |
| endomembrane system | 67 out of 412 genes, 16.3% | 3480 out of 37603 genes, 9.3% | 0.00097 | View Result |
| integral to plasma membrane | 35 out of 412 genes, 8.5% | 1399 out of 37603 genes, 3.7% | 0.00143 | View Result |
| intrinsic to plasma membrane | 36 out of 412 genes, 8.7% | 1463 out of 37603 genes, 3.9% | 0.00150 | View Result |
| multivesicular body | 5 out of 412 genes, 1.2% | 35 out of 37603 genes, 0.1% | 0.00900 | View Result |
| lysosome | 18 out of 412 genes, 4.4% | 560 out of 37603 genes, 1.5% | 0.01326 | View Result |
| plasma membrane part | 71 out of 412 genes, 17.2% | 4083 out of 37603 genes, 10.9% | 0.01425 | View Result |
| condensed nuclear chromosome kinetochore | 3 out of 412 genes, 0.7% | 8 out of 37603 genes, 0.0% | 0.01656 | View Result |
| cytosol | 55 out of 412 genes, 13.3% | 2945 out of 37603 genes, 7.8% | 0.01790 | View Result |
| high-density lipoprotein particle | 5 out of 412 genes, 1.2% | 43 out of 37603 genes, 0.1% | 0.02485 | View Result |
| mitochondrion | 53 out of 412 genes, 12.9% | 2848 out of 37603 genes, 7.6% | 0.02677 | View Result |
| condensed nuclear chromosome outer kinetochore | 2 out of 412 genes, 0.5% | 2 out of 37603 genes, 0.0% | 0.02826 | View Result |
| anchored to membrane | 8 out of 412 genes, 1.9% | 151 out of 37603 genes, 0.4% | 0.06513 | View Result |
| external side of plasma membrane | 11 out of 412 genes, 2.7% | 286 out of 37603 genes, 0.8% | 0.08337 | View Result |
| vesicular fraction | 15 out of 412 genes, 3.6% | 487 out of 37603 genes, 1.3% | 0.08515 | View Result |
| condensed nuclear chromosome, centromeric region | 3 out of 412 genes, 0.7% | 15 out of 37603 genes, 0.0% | 0.12712 | View Result |
| MHC class II protein complex | 3 out of 412 genes, 0.7% | 15 out of 37603 genes, 0.0% | 0.12712 | View Result |
| endoplasmic reticulum lumen | 8 out of 412 genes, 1.9% | 174 out of 37603 genes, 0.5% | 0.16717 | View Result |
| perinuclear region of cytoplasm | 18 out of 412 genes, 4.4% | 703 out of 37603 genes, 1.9% | 0.20443 | View Result |
| MHC protein complex | 5 out of 412 genes, 1.2% | 71 out of 37603 genes, 0.2% | 0.26169 | View Result |
| hemoglobin complex | 2 out of 412 genes, 0.5% | 5 out of 37603 genes, 0.0% | 0.27651 | View Result |
| Ndc80 complex | 2 out of 412 genes, 0.5% | 5 out of 37603 genes, 0.0% | 0.27651 | View Result |
| membrane | 218 out of 412 genes, 52.9% | 17065 out of 37603 genes, 45.4% | 0.28849 | View Result |
| lysosomal membrane | 7 out of 412 genes, 1.7% | 151 out of 37603 genes, 0.4% | 0.33998 | View Result |
| protein-lipid complex | 6 out of 412 genes, 1.5% | 112 out of 37603 genes, 0.3% | 0.35494 | View Result |
| plasma lipoprotein particle | 6 out of 412 genes, 1.5% | 112 out of 37603 genes, 0.3% | 0.35494 | View Result |
| lytic vacuole | 18 out of 412 genes, 4.4% | 750 out of 37603 genes, 2.0% | 0.41798 | View Result |
| membrane part | 172 out of 412 genes, 41.7% | 13186 out of 37603 genes, 35.1% | 0.64967 | View Result |
| membrane fraction | 17 out of 412 genes, 4.1% | 745 out of 37603 genes, 2.0% | 0.92110 | View Result |
| Ada2/Gcn5/Ada3 transcription activator complex | 3 out of 412 genes, 0.7% | 35 out of 37603 genes, 0.1% | 1 | View Result |
| vacuolar part | 9 out of 412 genes, 2.2% | 304 out of 37603 genes, 0.8% | 1 | View Result |
| extracellular matrix | 18 out of 412 genes, 4.4% | 854 out of 37603 genes, 2.3% | 1 | View Result |
| mitochondrial matrix | 11 out of 412 genes, 2.7% | 422 out of 37603 genes, 1.1% | 1 | View Result |
| filopodium | 5 out of 412 genes, 1.2% | 112 out of 37603 genes, 0.3% | 1 | View Result |
| vacuole | 18 out of 412 genes, 4.4% | 875 out of 37603 genes, 2.3% | 1 | View Result |
| proteinaceous extracellular matrix | 13 out of 412 genes, 3.2% | 554 out of 37603 genes, 1.5% | 1 | View Result |
| very-low-density lipoprotein particle | 3 out of 412 genes, 0.7% | 40 out of 37603 genes, 0.1% | 1 | View Result |
| glycogen granule | 2 out of 412 genes, 0.5% | 14 out of 37603 genes, 0.0% | 1 | View Result |
| intrinsic to membrane | 140 out of 412 genes, 34.0% | 10799 out of 37603 genes, 28.7% | 1 | View Result |
| peroxisome | 8 out of 412 genes, 1.9% | 284 out of 37603 genes, 0.8% | 1 | View Result |
| voltage-gated potassium channel complex | 2 out of 412 genes, 0.5% | 17 out of 37603 genes, 0.0% | 1 | View Result |
| potassium channel complex | 2 out of 412 genes, 0.5% | 17 out of 37603 genes, 0.0% | 1 | View Result |
| replisome | 2 out of 412 genes, 0.5% | 18 out of 37603 genes, 0.0% | 1 | View Result |
| nuclear replisome | 2 out of 412 genes, 0.5% | 18 out of 37603 genes, 0.0% | 1 | View Result |
| nucleotide-excision repair complex | 2 out of 412 genes, 0.5% | 19 out of 37603 genes, 0.1% | 1 | View Result |
| late endosome | 8 out of 412 genes, 1.9% | 301 out of 37603 genes, 0.8% | 1 | View Result |
| vacuolar membrane | 7 out of 412 genes, 1.7% | 247 out of 37603 genes, 0.7% | 1 | View Result |
| interstitial matrix | 2 out of 412 genes, 0.5% | 20 out of 37603 genes, 0.1% | 1 | View Result |
| MHC class I protein complex | 2 out of 412 genes, 0.5% | 20 out of 37603 genes, 0.1% | 1 | View Result |
| microbody part | 6 out of 412 genes, 1.5% | 201 out of 37603 genes, 0.5% | 1 | View Result |
| peroxisomal part | 6 out of 412 genes, 1.5% | 201 out of 37603 genes, 0.5% | 1 | View Result |
| condensed chromosome outer kinetochore | 2 out of 412 genes, 0.5% | 22 out of 37603 genes, 0.1% | 1 | View Result |
| proteasome complex | 4 out of 412 genes, 1.0% | 100 out of 37603 genes, 0.3% | 1 | View Result |
| peroxisomal membrane | 4 out of 412 genes, 1.0% | 101 out of 37603 genes, 0.3% | 1 | View Result |
| microbody membrane | 4 out of 412 genes, 1.0% | 101 out of 37603 genes, 0.3% | 1 | View Result |
| DNA polymerase complex | 2 out of 412 genes, 0.5% | 25 out of 37603 genes, 0.1% | 1 | View Result |
| triglyceride-rich lipoprotein particle | 3 out of 412 genes, 0.7% | 64 out of 37603 genes, 0.2% | 1 | View Result |
| lysosomal lumen | 3 out of 412 genes, 0.7% | 65 out of 37603 genes, 0.2% | 1 | View Result |
| cyclin-dependent protein kinase holoenzyme complex | 2 out of 412 genes, 0.5% | 27 out of 37603 genes, 0.1% | 1 | View Result |
| vacuolar lumen | 3 out of 412 genes, 0.7% | 66 out of 37603 genes, 0.2% | 1 | View Result |
| microbody | 8 out of 412 genes, 1.9% | 344 out of 37603 genes, 0.9% | 1 | View Result |
| brush border membrane | 3 out of 412 genes, 0.7% | 68 out of 37603 genes, 0.2% | 1 | View Result |
| proteasome core complex | 2 out of 412 genes, 0.5% | 29 out of 37603 genes, 0.1% | 1 | View Result |
| chylomicron | 2 out of 412 genes, 0.5% | 30 out of 37603 genes, 0.1% | 1 | View Result |
| nuclear replication fork | 2 out of 412 genes, 0.5% | 30 out of 37603 genes, 0.1% | 1 | View Result |
| condensed chromosome kinetochore | 4 out of 412 genes, 1.0% | 121 out of 37603 genes, 0.3% | 1 | View Result |
| nuclear envelope | 12 out of 412 genes, 2.9% | 638 out of 37603 genes, 1.7% | 1 | View Result |
| cytoplasmic membrane-bounded vesicle lumen | 3 out of 412 genes, 0.7% | 77 out of 37603 genes, 0.2% | 1 | View Result |
| condensed chromosome, centromeric region | 4 out of 412 genes, 1.0% | 130 out of 37603 genes, 0.3% | 1 | View Result |
| organelle envelope | 29 out of 412 genes, 7.0% | 1940 out of 37603 genes, 5.2% | 1 | View Result |
| mitochondrial intermembrane space | 3 out of 412 genes, 0.7% | 80 out of 37603 genes, 0.2% | 1 | View Result |
| nuclear outer membrane | 2 out of 412 genes, 0.5% | 37 out of 37603 genes, 0.1% | 1 | View Result |
| sarcolemma | 5 out of 412 genes, 1.2% | 193 out of 37603 genes, 0.5% | 1 | View Result |
| envelope | 29 out of 412 genes, 7.0% | 1966 out of 37603 genes, 5.2% | 1 | View Result |
| histone acetyltransferase complex | 5 out of 412 genes, 1.2% | 198 out of 37603 genes, 0.5% | 1 | View Result |
| mitochondrial part | 24 out of 412 genes, 5.8% | 1586 out of 37603 genes, 4.2% | 1 | View Result |
| vesicle lumen | 3 out of 412 genes, 0.7% | 88 out of 37603 genes, 0.2% | 1 | View Result |
| lipid particle | 3 out of 412 genes, 0.7% | 89 out of 37603 genes, 0.2% | 1 | View Result |
| organelle envelope lumen | 3 out of 412 genes, 0.7% | 89 out of 37603 genes, 0.2% | 1 | View Result |
| myelin sheath | 3 out of 412 genes, 0.7% | 91 out of 37603 genes, 0.2% | 1 | View Result |
| H4/H2A histone acetyltransferase complex | 2 out of 412 genes, 0.5% | 45 out of 37603 genes, 0.1% | 1 | View Result |
| nuclear membrane | 6 out of 412 genes, 1.5% | 287 out of 37603 genes, 0.8% | 1 | View Result |
| late endosome membrane | 3 out of 412 genes, 0.7% | 100 out of 37603 genes, 0.3% | 1 | View Result |
| cell part | 362 out of 412 genes, 87.9% | 32173 out of 37603 genes, 85.6% | 1 | View Result |
| cell | 362 out of 412 genes, 87.9% | 32174 out of 37603 genes, 85.6% | 1 | View Result |
| melanosome | 3 out of 412 genes, 0.7% | 102 out of 37603 genes, 0.3% | 1 | View Result |
| endosome membrane | 7 out of 412 genes, 1.7% | 366 out of 37603 genes, 1.0% | 1 | View Result |
| contractile fiber | 8 out of 412 genes, 1.9% | 441 out of 37603 genes, 1.2% | 1 | View Result |
| Golgi lumen | 2 out of 412 genes, 0.5% | 54 out of 37603 genes, 0.1% | 1 | View Result |
| endosomal part | 7 out of 412 genes, 1.7% | 375 out of 37603 genes, 1.0% | 1 | View Result |
| neuromuscular junction | 2 out of 412 genes, 0.5% | 55 out of 37603 genes, 0.1% | 1 | View Result |
| collagen | 3 out of 412 genes, 0.7% | 112 out of 37603 genes, 0.3% | 1 | View Result |
| cation channel complex | 3 out of 412 genes, 0.7% | 113 out of 37603 genes, 0.3% | 1 | View Result |
| mitochondrial envelope | 16 out of 412 genes, 3.9% | 1070 out of 37603 genes, 2.8% | 1 | View Result |
| endosome | 16 out of 412 genes, 3.9% | 1107 out of 37603 genes, 2.9% | 1 | View Result |
| extracellular matrix part | 6 out of 412 genes, 1.5% | 335 out of 37603 genes, 0.9% | 1 | View Result |
| phagocytic vesicle | 2 out of 412 genes, 0.5% | 67 out of 37603 genes, 0.2% | 1 | View Result |
| organelle part | 172 out of 412 genes, 41.7% | 14834 out of 37603 genes, 39.4% | 1 | View Result |
| platelet alpha granule | 3 out of 412 genes, 0.7% | 135 out of 37603 genes, 0.4% | 1 | View Result |
| mitochondrial inner membrane | 9 out of 412 genes, 2.2% | 577 out of 37603 genes, 1.5% | 1 | View Result |
| pigment granule | 4 out of 412 genes, 1.0% | 206 out of 37603 genes, 0.5% | 1 | View Result |
| cell-cell junction | 11 out of 412 genes, 2.7% | 742 out of 37603 genes, 2.0% | 1 | View Result |
| platelet alpha granule lumen | 2 out of 412 genes, 0.5% | 74 out of 37603 genes, 0.2% | 1 | View Result |
| ion channel complex | 4 out of 412 genes, 1.0% | 208 out of 37603 genes, 0.6% | 1 | View Result |
| secretory granule lumen | 2 out of 412 genes, 0.5% | 75 out of 37603 genes, 0.2% | 1 | View Result |
| protein-DNA complex | 3 out of 412 genes, 0.7% | 141 out of 37603 genes, 0.4% | 1 | View Result |
| replication fork | 2 out of 412 genes, 0.5% | 77 out of 37603 genes, 0.2% | 1 | View Result |
| intrinsic to endoplasmic reticulum membrane | 4 out of 412 genes, 1.0% | 217 out of 37603 genes, 0.6% | 1 | View Result |
| early endosome | 5 out of 412 genes, 1.2% | 301 out of 37603 genes, 0.8% | 1 | View Result |
| brush border | 3 out of 412 genes, 0.7% | 160 out of 37603 genes, 0.4% | 1 | View Result |
| condensed nuclear chromosome | 3 out of 412 genes, 0.7% | 162 out of 37603 genes, 0.4% | 1 | View Result |
| intracellular organelle part | 160 out of 412 genes, 38.8% | 13998 out of 37603 genes, 37.2% | 1 | View Result |
| kinetochore | 4 out of 412 genes, 1.0% | 242 out of 37603 genes, 0.6% | 1 | View Result |
| neuron projection | 21 out of 412 genes, 5.1% | 1654 out of 37603 genes, 4.4% | 1 | View Result |
| coated pit | 2 out of 412 genes, 0.5% | 95 out of 37603 genes, 0.3% | 1 | View Result |
| dendritic spine | 4 out of 412 genes, 1.0% | 248 out of 37603 genes, 0.7% | 1 | View Result |
| neuron spine | 4 out of 412 genes, 1.0% | 248 out of 37603 genes, 0.7% | 1 | View Result |
| membrane raft | 5 out of 412 genes, 1.2% | 335 out of 37603 genes, 0.9% | 1 | View Result |
| actin filament | 3 out of 412 genes, 0.7% | 183 out of 37603 genes, 0.5% | 1 | View Result |
| cell projection | 36 out of 412 genes, 8.7% | 3024 out of 37603 genes, 8.0% | 1 | View Result |
| extrinsic to membrane | 4 out of 412 genes, 1.0% | 264 out of 37603 genes, 0.7% | 1 | View Result |
| cell projection part | 14 out of 412 genes, 3.4% | 1106 out of 37603 genes, 2.9% | 1 | View Result |
| cell projection membrane | 5 out of 412 genes, 1.2% | 349 out of 37603 genes, 0.9% | 1 | View Result |
| mitochondrial membrane | 12 out of 412 genes, 2.9% | 943 out of 37603 genes, 2.5% | 1 | View Result |
| basolateral plasma membrane | 9 out of 412 genes, 2.2% | 687 out of 37603 genes, 1.8% | 1 | View Result |
| lamellipodium | 3 out of 412 genes, 0.7% | 191 out of 37603 genes, 0.5% | 1 | View Result |
| secretory granule | 8 out of 412 genes, 1.9% | 610 out of 37603 genes, 1.6% | 1 | View Result |
| cell fraction | 24 out of 412 genes, 5.8% | 2030 out of 37603 genes, 5.4% | 1 | View Result |
| integral to endoplasmic reticulum membrane | 2 out of 412 genes, 0.5% | 122 out of 37603 genes, 0.3% | 1 | View Result |
| nuclear chromosome part | 7 out of 412 genes, 1.7% | 547 out of 37603 genes, 1.5% | 1 | View Result |
| intracellular membrane-bounded organelle | 250 out of 412 genes, 60.7% | 22524 out of 37603 genes, 59.9% | 1 | View Result |
| basement membrane | 3 out of 412 genes, 0.7% | 206 out of 37603 genes, 0.5% | 1 | View Result |
| insoluble fraction | 18 out of 412 genes, 4.4% | 1517 out of 37603 genes, 4.0% | 1 | View Result |
| transcriptional repressor complex | 2 out of 412 genes, 0.5% | 128 out of 37603 genes, 0.3% | 1 | View Result |
| intrinsic to organelle membrane | 6 out of 412 genes, 1.5% | 470 out of 37603 genes, 1.2% | 1 | View Result |
| nuclear speck | 3 out of 412 genes, 0.7% | 213 out of 37603 genes, 0.6% | 1 | View Result |
| myofibril | 5 out of 412 genes, 1.2% | 395 out of 37603 genes, 1.1% | 1 | View Result |
| neuronal cell body | 6 out of 412 genes, 1.5% | 494 out of 37603 genes, 1.3% | 1 | View Result |
| internal side of plasma membrane | 3 out of 412 genes, 0.7% | 229 out of 37603 genes, 0.6% | 1 | View Result |
| condensed chromosome | 4 out of 412 genes, 1.0% | 318 out of 37603 genes, 0.8% | 1 | View Result |
| intermediate filament | 2 out of 412 genes, 0.5% | 144 out of 37603 genes, 0.4% | 1 | View Result |
| membrane-bounded organelle | 250 out of 412 genes, 60.7% | 22716 out of 37603 genes, 60.4% | 1 | View Result |
| microvillus | 2 out of 412 genes, 0.5% | 154 out of 37603 genes, 0.4% | 1 | View Result |
| histone methyltransferase complex | 2 out of 412 genes, 0.5% | 155 out of 37603 genes, 0.4% | 1 | View Result |
| extrinsic to plasma membrane | 2 out of 412 genes, 0.5% | 156 out of 37603 genes, 0.4% | 1 | View Result |
| tight junction | 2 out of 412 genes, 0.5% | 157 out of 37603 genes, 0.4% | 1 | View Result |
| postsynaptic membrane | 2 out of 412 genes, 0.5% | 157 out of 37603 genes, 0.4% | 1 | View Result |
| occluding junction | 2 out of 412 genes, 0.5% | 157 out of 37603 genes, 0.4% | 1 | View Result |
| Golgi apparatus | 21 out of 412 genes, 5.1% | 1902 out of 37603 genes, 5.1% | 1 | View Result |
| endocytic vesicle | 3 out of 412 genes, 0.7% | 252 out of 37603 genes, 0.7% | 1 | View Result |
| integral to organelle membrane | 3 out of 412 genes, 0.7% | 257 out of 37603 genes, 0.7% | 1 | View Result |
| intracellular part | 315 out of 412 genes, 76.5% | 28781 out of 37603 genes, 76.5% | 1 | View Result |
| chromosome, centromeric region | 4 out of 412 genes, 1.0% | 354 out of 37603 genes, 0.9% | 1 | View Result |
| postsynaptic density | 2 out of 412 genes, 0.5% | 170 out of 37603 genes, 0.5% | 1 | View Result |
| dendritic spine head | 2 out of 412 genes, 0.5% | 170 out of 37603 genes, 0.5% | 1 | View Result |
| intracellular | 320 out of 412 genes, 77.7% | 29285 out of 37603 genes, 77.9% | 1 | View Result |
| organelle inner membrane | 10 out of 412 genes, 2.4% | 932 out of 37603 genes, 2.5% | 1 | View Result |
| methyltransferase complex | 2 out of 412 genes, 0.5% | 176 out of 37603 genes, 0.5% | 1 | View Result |
| cell body | 10 out of 412 genes, 2.4% | 936 out of 37603 genes, 2.5% | 1 | View Result |
| dendrite | 7 out of 412 genes, 1.7% | 655 out of 37603 genes, 1.7% | 1 | View Result |
| synaptic membrane | 2 out of 412 genes, 0.5% | 184 out of 37603 genes, 0.5% | 1 | View Result |
| nuclear chromosome | 7 out of 412 genes, 1.7% | 677 out of 37603 genes, 1.8% | 1 | View Result |
| intermediate filament cytoskeleton | 2 out of 412 genes, 0.5% | 197 out of 37603 genes, 0.5% | 1 | View Result |
| sarcomere | 3 out of 412 genes, 0.7% | 302 out of 37603 genes, 0.8% | 1 | View Result |
| I band | 2 out of 412 genes, 0.5% | 208 out of 37603 genes, 0.6% | 1 | View Result |
| Golgi membrane | 8 out of 412 genes, 1.9% | 819 out of 37603 genes, 2.2% | 1 | View Result |
| synapse | 7 out of 412 genes, 1.7% | 741 out of 37603 genes, 2.0% | 1 | View Result |
| contractile fiber part | 3 out of 412 genes, 0.7% | 339 out of 37603 genes, 0.9% | 1 | View Result |
| organelle outer membrane | 2 out of 412 genes, 0.5% | 251 out of 37603 genes, 0.7% | 1 | View Result |
| nuclear periphery | 2 out of 412 genes, 0.5% | 252 out of 37603 genes, 0.7% | 1 | View Result |
| chromatin remodeling complex | 3 out of 412 genes, 0.7% | 368 out of 37603 genes, 1.0% | 1 | View Result |
| cytoplasmic membrane-bounded vesicle | 18 out of 412 genes, 4.4% | 1912 out of 37603 genes, 5.1% | 1 | View Result |
| cytosolic part | 2 out of 412 genes, 0.5% | 259 out of 37603 genes, 0.7% | 1 | View Result |
| outer membrane | 2 out of 412 genes, 0.5% | 271 out of 37603 genes, 0.7% | 1 | View Result |
| membrane-bounded vesicle | 19 out of 412 genes, 4.6% | 2048 out of 37603 genes, 5.4% | 1 | View Result |
| actin cytoskeleton | 7 out of 412 genes, 1.7% | 830 out of 37603 genes, 2.2% | 1 | View Result |
| Golgi apparatus part | 10 out of 412 genes, 2.4% | 1145 out of 37603 genes, 3.0% | 1 | View Result |
| cytoplasmic vesicle part | 5 out of 412 genes, 1.2% | 629 out of 37603 genes, 1.7% | 1 | View Result |
| nucleoplasm | 25 out of 412 genes, 6.1% | 2713 out of 37603 genes, 7.2% | 1 | View Result |
| cell junction | 13 out of 412 genes, 3.2% | 1502 out of 37603 genes, 4.0% | 1 | View Result |
| axon | 4 out of 412 genes, 1.0% | 539 out of 37603 genes, 1.4% | 1 | View Result |
| transcription factor complex | 4 out of 412 genes, 1.0% | 555 out of 37603 genes, 1.5% | 1 | View Result |
| synapse part | 3 out of 412 genes, 0.7% | 453 out of 37603 genes, 1.2% | 1 | View Result |
| chromosomal part | 12 out of 412 genes, 2.9% | 1456 out of 37603 genes, 3.9% | 1 | View Result |
| apical junction complex | 2 out of 412 genes, 0.5% | 339 out of 37603 genes, 0.9% | 1 | View Result |
| apicolateral plasma membrane | 2 out of 412 genes, 0.5% | 348 out of 37603 genes, 0.9% | 1 | View Result |
| cytoplasmic vesicle | 19 out of 412 genes, 4.6% | 2245 out of 37603 genes, 6.0% | 1 | View Result |
| chromatin | 5 out of 412 genes, 1.2% | 737 out of 37603 genes, 2.0% | 1 | View Result |
| microtubule | 3 out of 412 genes, 0.7% | 499 out of 37603 genes, 1.3% | 1 | View Result |
| intracellular organelle | 260 out of 412 genes, 63.1% | 24874 out of 37603 genes, 66.1% | 1 | View Result |
| nucleolus | 17 out of 412 genes, 4.1% | 2075 out of 37603 genes, 5.5% | 1 | View Result |
| vesicle | 21 out of 412 genes, 5.1% | 2526 out of 37603 genes, 6.7% | 1 | View Result |
| organelle | 261 out of 412 genes, 63.3% | 25054 out of 37603 genes, 66.6% | 1 | View Result |
| clathrin-coated vesicle | 2 out of 412 genes, 0.5% | 423 out of 37603 genes, 1.1% | 1 | View Result |
| membrane-enclosed lumen | 66 out of 412 genes, 16.0% | 7219 out of 37603 genes, 19.2% | 1 | View Result |
| chromosome | 12 out of 412 genes, 2.9% | 1683 out of 37603 genes, 4.5% | 1 | View Result |
| nucleus | 99 out of 412 genes, 24.0% | 10441 out of 37603 genes, 27.8% | 1 | View Result |
| nucleoplasm part | 13 out of 412 genes, 3.2% | 1841 out of 37603 genes, 4.9% | 1 | View Result |
| protein complex | 60 out of 412 genes, 14.6% | 6728 out of 37603 genes, 17.9% | 1 | View Result |
| cell leading edge | 4 out of 412 genes, 1.0% | 771 out of 37603 genes, 2.1% | 1 | View Result |
| spindle | 2 out of 412 genes, 0.5% | 496 out of 37603 genes, 1.3% | 1 | View Result |
| adherens junction | 2 out of 412 genes, 0.5% | 518 out of 37603 genes, 1.4% | 1 | View Result |
| organelle lumen | 63 out of 412 genes, 15.3% | 7139 out of 37603 genes, 19.0% | 1 | View Result |
| coated vesicle | 2 out of 412 genes, 0.5% | 526 out of 37603 genes, 1.4% | 1 | View Result |
| cytoplasmic vesicle membrane | 2 out of 412 genes, 0.5% | 549 out of 37603 genes, 1.5% | 1 | View Result |
| nuclear body | 3 out of 412 genes, 0.7% | 729 out of 37603 genes, 1.9% | 1 | View Result |
| anchoring junction | 2 out of 412 genes, 0.5% | 581 out of 37603 genes, 1.5% | 1 | View Result |
| cytoskeletal part | 18 out of 412 genes, 4.4% | 2623 out of 37603 genes, 7.0% | 1 | View Result |
| intracellular organelle lumen | 60 out of 412 genes, 14.6% | 7060 out of 37603 genes, 18.8% | 1 | View Result |
| centrosome | 2 out of 412 genes, 0.5% | 672 out of 37603 genes, 1.8% | 1 | View Result |
| vesicle membrane | 2 out of 412 genes, 0.5% | 672 out of 37603 genes, 1.8% | 1 | View Result |
| macromolecular complex | 71 out of 412 genes, 17.2% | 8498 out of 37603 genes, 22.6% | 1 | View Result |
| nuclear part | 56 out of 412 genes, 13.6% | 7093 out of 37603 genes, 18.9% | 1 | View Result |
| microtubule organizing center | 3 out of 412 genes, 0.7% | 1077 out of 37603 genes, 2.9% | 1 | View Result |
| microtubule cytoskeleton | 9 out of 412 genes, 2.2% | 2086 out of 37603 genes, 5.5% | 1 | View Result |
| cytoskeleton | 24 out of 412 genes, 5.8% | 4034 out of 37603 genes, 10.7% | 1 | View Result |
| ribonucleoprotein complex | 4 out of 412 genes, 1.0% | 1367 out of 37603 genes, 3.6% | 1 | View Result |
| nuclear lumen | 44 out of 412 genes, 10.7% | 6408 out of 37603 genes, 17.0% | 1 | View Result |
| non-membrane-bounded organelle | 55 out of 412 genes, 13.3% | 7901 out of 37603 genes, 21.0% | 1 | View Result |
| intracellular non-membrane-bounded organelle | 55 out of 412 genes, 13.3% | 7901 out of 37603 genes, 21.0% | 1 | View Result |

| Gene Ontology term | Genes annotated to the term |
| --- | --- |
| extracellular space | CL1372.Contig1\_Mf\_liverA, CL1509.Contig3\_Mf\_liverA, Unigene13337\_Mf\_liverA, CL1811.Contig2\_Mf\_liverA, Unigene36404\_Mf\_liverA, Unigene14536\_Mf\_liverA, Unigene5382\_Mf\_liverA, Unigene12959\_Mf\_liverA, Unigene34034\_Mf\_liverA, Unigene10351\_Mf\_liverA, CL2339.Contig1\_Mf\_liverA, CL81.Contig1\_Mf\_liverA, Unigene36698\_Mf\_liverA, Unigene25595\_Mf\_liverA, CL3312.Contig1\_Mf\_liverA, Unigene36112\_Mf\_liverA, Unigene941\_Mf\_liverA, Unigene31065\_Mf\_liverA, CL1372.Contig2\_Mf\_liverA, Unigene30039\_Mf\_liverA, Unigene30322\_Mf\_liverA, CL4299.Contig1\_Mf\_liverA, Unigene37698\_Mf\_liverA, Unigene19973\_Mf\_liverA, Unigene13498\_Mf\_liverA, Unigene13950\_Mf\_liverA, Unigene27249\_Mf\_liverA, Unigene46931\_Mf\_liverA, Unigene17579\_Mf\_liverA, CL4583.Contig2\_Mf\_liverA, Unigene13894\_Mf\_liverA, Unigene24974\_Mf\_liverA, Unigene34477\_Mf\_liverA, Unigene38280\_Mf\_liverA, Unigene34375\_Mf\_liverA, CL634.Contig1\_Mf\_liverA, Unigene27248\_Mf\_liverA, Unigene34983\_Mf\_liverA, Unigene8132\_Mf\_liverA, CL4299.Contig2\_Mf\_liverA, NM\_010233, Unigene32316\_Mf\_liverA, CL3911.Contig2\_Mf\_liverA, Unigene14541\_Mf\_liverA, Unigene559\_Mf\_liverA, CL6018.Contig1\_Mf\_liverA, CL3575.Contig1\_Mf\_liverA, CL1811.Contig1\_Mf\_liverA, Unigene30321\_Mf\_liverA, Unigene33683\_Mf\_liverA, Unigene36439\_Mf\_liverA, CL1509.Contig2\_Mf\_liverA, CL4650.Contig1\_Mf\_liverA, Unigene36426\_Mf\_liverA, Unigene32059\_Mf\_liverA |
| extracellular region | CL1509.Contig3\_Mf\_liverA, Unigene13337\_Mf\_liverA, Unigene38831\_Mf\_liverA, CL1811.Contig2\_Mf\_liverA, Unigene36404\_Mf\_liverA, Unigene36908\_Mf\_liverA, Unigene14536\_Mf\_liverA, Unigene12959\_Mf\_liverA, Unigene10351\_Mf\_liverA, CL2339.Contig1\_Mf\_liverA, CL81.Contig1\_Mf\_liverA, Unigene36762\_Mf\_liverA, Unigene31065\_Mf\_liverA, CL1372.Contig2\_Mf\_liverA, Unigene30039\_Mf\_liverA, CL4299.Contig1\_Mf\_liverA, Unigene37698\_Mf\_liverA, Unigene46931\_Mf\_liverA, CL3569.Contig2\_Mf\_liverA, CL4583.Contig2\_Mf\_liverA, Unigene13894\_Mf\_liverA, Unigene24974\_Mf\_liverA, Unigene34477\_Mf\_liverA, Unigene38280\_Mf\_liverA, Unigene34375\_Mf\_liverA, Unigene20512\_Mf\_liverA, Unigene34983\_Mf\_liverA, Unigene14276\_Mf\_liverA, CL4343.Contig2\_Mf\_liverA, CL4299.Contig2\_Mf\_liverA, CL2285.Contig2\_Mf\_liverA, CL4343.Contig3\_Mf\_liverA, NM\_010233, Unigene32316\_Mf\_liverA, NM\_009776, Unigene30003\_Mf\_liverA, Unigene559\_Mf\_liverA, Unigene20371\_Mf\_liverA, CL3575.Contig1\_Mf\_liverA, CL1811.Contig1\_Mf\_liverA, Unigene393\_Mf\_liverA, Unigene14810\_Mf\_liverA, Unigene33683\_Mf\_liverA, Unigene30002\_Mf\_liverA, Unigene8054\_Mf\_liverA, Unigene23328\_Mf\_liverA, CL4650.Contig1\_Mf\_liverA, Unigene5134\_Mf\_liverA, CL1372.Contig1\_Mf\_liverA, Unigene24887\_Mf\_liverA, CL3112.Contig7\_Mf\_liverA, Unigene5382\_Mf\_liverA, Unigene26380\_Mf\_liverA, Unigene34034\_Mf\_liverA, NM\_007585, CL5293.Contig1\_Mf\_liverA, Unigene36698\_Mf\_liverA, Unigene25595\_Mf\_liverA, CL3312.Contig1\_Mf\_liverA, Unigene36112\_Mf\_liverA, Unigene941\_Mf\_liverA, Unigene35090\_Mf\_liverA, Unigene33560\_Mf\_liverA, Unigene30322\_Mf\_liverA, Unigene19973\_Mf\_liverA, Unigene13498\_Mf\_liverA, Unigene28315\_Mf\_liverA, Unigene13950\_Mf\_liverA, Unigene27249\_Mf\_liverA, Unigene17579\_Mf\_liverA, CL1736.Contig2\_Mf\_liverA, Unigene32908\_Mf\_liverA, Unigene20372\_Mf\_liverA, CL634.Contig1\_Mf\_liverA, Unigene14809\_Mf\_liverA, Unigene27248\_Mf\_liverA, Unigene8132\_Mf\_liverA, Unigene5355\_Mf\_liverA, Unigene30839\_Mf\_liverA, CL3911.Contig2\_Mf\_liverA, Unigene14541\_Mf\_liverA, CL6018.Contig1\_Mf\_liverA, Unigene30321\_Mf\_liverA, Unigene36439\_Mf\_liverA, CL1509.Contig2\_Mf\_liverA, NM\_146120, Unigene36426\_Mf\_liverA, Unigene32059\_Mf\_liverA, Unigene31492\_Mf\_liverA, Unigene37559\_Mf\_liverA |
| nuclear outer membrane-endoplasmic reticulum membrane network | CL4816.Contig1\_Mf\_liverA, NM\_023868, Unigene10136\_Mf\_liverA, Unigene5755\_Mf\_liverA, NM\_009128, CL4220.Contig1\_Mf\_liverA, Unigene13414\_Mf\_liverA, CL2240.Contig1\_Mf\_liverA, CL5307.Contig1\_Mf\_liverA, Unigene1479\_Mf\_liverA, CL523.Contig1\_Mf\_liverA, Unigene172\_Mf\_liverA, CL4550.Contig3\_Mf\_liverA, Unigene35507\_Mf\_liverA, Unigene34628\_Mf\_liverA, Unigene5335\_Mf\_liverA, Unigene139\_Mf\_liverA, CL5986.Contig1\_Mf\_liverA, Unigene5428\_Mf\_liverA, Unigene35609\_Mf\_liverA, CL4007.Contig1\_Mf\_liverA, Unigene35046\_Mf\_liverA, CL4220.Contig2\_Mf\_liverA, CL4583.Contig2\_Mf\_liverA, CL2478.Contig3\_Mf\_liverA, Unigene36315\_Mf\_liverA, Unigene36784\_Mf\_liverA, Unigene34341\_Mf\_liverA, Unigene36514\_Mf\_liverA, NM\_130450, Unigene34630\_Mf\_liverA, Unigene36593\_Mf\_liverA, Unigene7195\_Mf\_liverA, CL4816.Contig2\_Mf\_liverA, Unigene36508\_Mf\_liverA, Unigene22500\_Mf\_liverA, Unigene35506\_Mf\_liverA, Unigene3439\_Mf\_liverA, Unigene21026\_Mf\_liverA, Unigene5712\_Mf\_liverA, CL5698.Contig1\_Mf\_liverA, Unigene36937\_Mf\_liverA, Unigene1205\_Mf\_liverA, Unigene5294\_Mf\_liverA, CL336.Contig3\_Mf\_liverA, Unigene29882\_Mf\_liverA, Unigene10135\_Mf\_liverA |
| endoplasmic reticulum part | CL4816.Contig1\_Mf\_liverA, NM\_023868, Unigene10136\_Mf\_liverA, Unigene5755\_Mf\_liverA, NM\_009128, CL4220.Contig1\_Mf\_liverA, Unigene13414\_Mf\_liverA, CL2240.Contig1\_Mf\_liverA, CL5307.Contig1\_Mf\_liverA, Unigene1479\_Mf\_liverA, CL523.Contig1\_Mf\_liverA, Unigene172\_Mf\_liverA, CL4550.Contig3\_Mf\_liverA, Unigene25595\_Mf\_liverA, Unigene35507\_Mf\_liverA, Unigene34628\_Mf\_liverA, Unigene5335\_Mf\_liverA, Unigene139\_Mf\_liverA, CL5986.Contig1\_Mf\_liverA, Unigene5428\_Mf\_liverA, Unigene35609\_Mf\_liverA, Unigene13498\_Mf\_liverA, CL4007.Contig1\_Mf\_liverA, Unigene35046\_Mf\_liverA, CL4220.Contig2\_Mf\_liverA, CL4583.Contig2\_Mf\_liverA, Unigene34375\_Mf\_liverA, Unigene36315\_Mf\_liverA, Unigene36784\_Mf\_liverA, Unigene34341\_Mf\_liverA, Unigene36514\_Mf\_liverA, NM\_130450, Unigene34630\_Mf\_liverA, Unigene36593\_Mf\_liverA, Unigene7195\_Mf\_liverA, CL4816.Contig2\_Mf\_liverA, Unigene36508\_Mf\_liverA, Unigene22500\_Mf\_liverA, Unigene35506\_Mf\_liverA, Unigene3439\_Mf\_liverA, CL3911.Contig2\_Mf\_liverA, Unigene25596\_Mf\_liverA, Unigene21026\_Mf\_liverA, Unigene5712\_Mf\_liverA, CL5698.Contig1\_Mf\_liverA, Unigene36937\_Mf\_liverA, Unigene1205\_Mf\_liverA, Unigene5294\_Mf\_liverA, CL336.Contig3\_Mf\_liverA, Unigene23328\_Mf\_liverA, Unigene29882\_Mf\_liverA, Unigene10135\_Mf\_liverA |
| endoplasmic reticulum membrane | CL4816.Contig1\_Mf\_liverA, NM\_023868, Unigene10136\_Mf\_liverA, Unigene5755\_Mf\_liverA, NM\_009128, CL4220.Contig1\_Mf\_liverA, Unigene13414\_Mf\_liverA, CL2240.Contig1\_Mf\_liverA, CL5307.Contig1\_Mf\_liverA, Unigene1479\_Mf\_liverA, CL523.Contig1\_Mf\_liverA, Unigene172\_Mf\_liverA, CL4550.Contig3\_Mf\_liverA, Unigene35507\_Mf\_liverA, Unigene34628\_Mf\_liverA, Unigene5335\_Mf\_liverA, Unigene139\_Mf\_liverA, CL5986.Contig1\_Mf\_liverA, Unigene5428\_Mf\_liverA, Unigene35609\_Mf\_liverA, CL4007.Contig1\_Mf\_liverA, Unigene35046\_Mf\_liverA, CL4220.Contig2\_Mf\_liverA, CL4583.Contig2\_Mf\_liverA, Unigene36315\_Mf\_liverA, Unigene36784\_Mf\_liverA, Unigene34341\_Mf\_liverA, Unigene36514\_Mf\_liverA, NM\_130450, Unigene34630\_Mf\_liverA, Unigene36593\_Mf\_liverA, Unigene7195\_Mf\_liverA, CL4816.Contig2\_Mf\_liverA, Unigene36508\_Mf\_liverA, Unigene22500\_Mf\_liverA, Unigene35506\_Mf\_liverA, Unigene3439\_Mf\_liverA, Unigene21026\_Mf\_liverA, Unigene5712\_Mf\_liverA, CL5698.Contig1\_Mf\_liverA, Unigene36937\_Mf\_liverA, Unigene1205\_Mf\_liverA, Unigene5294\_Mf\_liverA, CL336.Contig3\_Mf\_liverA, Unigene29882\_Mf\_liverA, Unigene10135\_Mf\_liverA |
| cytoplasm | Unigene21684\_Mf\_liverA, Unigene172\_Mf\_liverA, NM\_011082, Unigene35507\_Mf\_liverA, CL2982.Contig1\_Mf\_liverA, Unigene39886\_Mf\_liverA, Unigene27339\_Mf\_liverA, Unigene4732\_Mf\_liverA, CL2790.Contig1\_Mf\_liverA, CL5586.Contig1\_Mf\_liverA, Unigene47548\_Mf\_liverA, Unigene34341\_Mf\_liverA, Unigene30153\_Mf\_liverA, Unigene36662\_Mf\_liverA, NM\_011921, Unigene35816\_Mf\_liverA, NM\_010233, Unigene13940\_Mf\_liverA, Unigene4725\_Mf\_liverA, NM\_009776, Unigene30003\_Mf\_liverA, Unigene25594\_Mf\_liverA, Unigene29461\_Mf\_liverA, Unigene5328\_Mf\_liverA, Unigene30002\_Mf\_liverA, Unigene8054\_Mf\_liverA, Unigene14589\_Mf\_liverA, CL4816.Contig1\_Mf\_liverA, Unigene5639\_Mf\_liverA, Unigene17048\_Mf\_liverA, Unigene4684\_Mf\_liverA, Unigene743\_Mf\_liverA, Unigene36175\_Mf\_liverA, NM\_177093, Unigene33512\_Mf\_liverA, Unigene34591\_Mf\_liverA, Unigene1479\_Mf\_liverA, CL4332.Contig1\_Mf\_liverA, NM\_080639, Unigene36698\_Mf\_liverA, Unigene27895\_Mf\_liverA, Unigene36112\_Mf\_liverA, Unigene139\_Mf\_liverA, Unigene27249\_Mf\_liverA, Unigene27547\_Mf\_liverA, CL1736.Contig2\_Mf\_liverA, Unigene4731\_Mf\_liverA, Unigene33366\_Mf\_liverA, Unigene12909\_Mf\_liverA, CL5316.Contig1\_Mf\_liverA, Unigene45530\_Mf\_liverA, Unigene32332\_Mf\_liverA, Unigene14588\_Mf\_liverA, Unigene34630\_Mf\_liverA, NR\_003630, Unigene27248\_Mf\_liverA, Unigene36508\_Mf\_liverA, Unigene30839\_Mf\_liverA, Unigene37485\_Mf\_liverA, Unigene30288\_Mf\_liverA, Unigene30528\_Mf\_liverA, Unigene29882\_Mf\_liverA, Unigene32059\_Mf\_liverA, Unigene45904\_Mf\_liverA, Unigene25962\_Mf\_liverA, NM\_023868, Unigene29727\_Mf\_liverA, Unigene38913\_Mf\_liverA, CL4925.Contig1\_Mf\_liverA, CL186.Contig3\_Mf\_liverA, Unigene12959\_Mf\_liverA, CL425.Contig1\_Mf\_liverA, CL1803.Contig1\_Mf\_liverA, CL4736.Contig1\_Mf\_liverA, Unigene37243\_Mf\_liverA, Unigene14916\_Mf\_liverA, Unigene15077\_Mf\_liverA, CL3800.Contig1\_Mf\_liverA, CL2478.Contig3\_Mf\_liverA, Unigene34375\_Mf\_liverA, CL3055.Contig1\_Mf\_liverA, NM\_028785, NM\_009609, CL4033.Contig1\_Mf\_liverA, NM\_007409, Unigene34983\_Mf\_liverA, CL2439.Contig1\_Mf\_liverA, Unigene3439\_Mf\_liverA, Unigene37238\_Mf\_liverA, Unigene21026\_Mf\_liverA, Unigene20371\_Mf\_liverA, Unigene36937\_Mf\_liverA, Unigene13683\_Mf\_liverA, Unigene30004\_Mf\_liverA, Unigene35476\_Mf\_liverA, CL5828.Contig2\_Mf\_liverA, Unigene24858\_Mf\_liverA, CL4220.Contig1\_Mf\_liverA, Unigene37262\_Mf\_liverA, Unigene12908\_Mf\_liverA, NR\_033215, CL2702.Contig2\_Mf\_liverA, Unigene24466\_Mf\_liverA, CL1165.Contig2\_Mf\_liverA, CL5293.Contig1\_Mf\_liverA, Unigene25595\_Mf\_liverA, CL3312.Contig1\_Mf\_liverA, Unigene15470\_Mf\_liverA, Unigene14286\_Mf\_liverA, Unigene34628\_Mf\_liverA, Unigene14212\_Mf\_liverA, Unigene34962\_Mf\_liverA, Unigene39011\_Mf\_liverA, Unigene26250\_Mf\_liverA, CL1588.Contig2\_Mf\_liverA, Unigene35609\_Mf\_liverA, Unigene36189\_Mf\_liverA, Unigene33580\_Mf\_liverA, Unigene13950\_Mf\_liverA, Unigene35046\_Mf\_liverA, Unigene28459\_Mf\_liverA, NM\_013863, Unigene37245\_Mf\_liverA, Unigene281\_Mf\_liverA, Unigene20372\_Mf\_liverA, NM\_011978, NM\_130450, Unigene29985\_Mf\_liverA, Unigene36593\_Mf\_liverA, NM\_027406, Unigene36176\_Mf\_liverA, Unigene37232\_Mf\_liverA, Unigene37239\_Mf\_liverA, Unigene37028\_Mf\_liverA, CL5698.Contig1\_Mf\_liverA, NM\_020559, Unigene28142\_Mf\_liverA, Unigene38015\_Mf\_liverA, CL3835.Contig2\_Mf\_liverA, Unigene36735\_Mf\_liverA, CL523.Contig1\_Mf\_liverA, Unigene36762\_Mf\_liverA, Unigene31065\_Mf\_liverA, NM\_011072, Unigene20853\_Mf\_liverA, Unigene5428\_Mf\_liverA, Unigene27592\_Mf\_liverA, Unigene24351\_Mf\_liverA, Unigene37999\_Mf\_liverA, CL3483.Contig1\_Mf\_liverA, Unigene12999\_Mf\_liverA, CL4141.Contig1\_Mf\_liverA, Unigene13658\_Mf\_liverA, Unigene32316\_Mf\_liverA, CL2622.Contig2\_Mf\_liverA, Unigene25596\_Mf\_liverA, Unigene28548\_Mf\_liverA, Unigene5294\_Mf\_liverA, CL5268.Contig1\_Mf\_liverA, Unigene23328\_Mf\_liverA, Unigene35171\_Mf\_liverA, Unigene36626\_Mf\_liverA, Unigene39507\_Mf\_liverA, Unigene5755\_Mf\_liverA, CL3750.Contig2\_Mf\_liverA, Unigene151\_Mf\_liverA, Unigene5382\_Mf\_liverA, CL3599.Contig1\_Mf\_liverA, CL1595.Contig1\_Mf\_liverA, CL838.Contig3\_Mf\_liverA, CL4550.Contig3\_Mf\_liverA, CL1263.Contig1\_Mf\_liverA, NM\_029692, CL258.Contig1\_Mf\_liverA, Unigene36628\_Mf\_liverA, Unigene31231\_Mf\_liverA, Unigene12786\_Mf\_liverA, Unigene31517\_Mf\_liverA, NM\_008538, CL1716.Contig2\_Mf\_liverA, Unigene4523\_Mf\_liverA, Unigene13498\_Mf\_liverA, Unigene4510\_Mf\_liverA, CL4220.Contig2\_Mf\_liverA, Unigene17579\_Mf\_liverA, Unigene19885\_Mf\_liverA, Unigene7195\_Mf\_liverA, Unigene8132\_Mf\_liverA, Unigene9664\_Mf\_liverA, Unigene5355\_Mf\_liverA, Unigene35506\_Mf\_liverA, Unigene5712\_Mf\_liverA, Unigene1205\_Mf\_liverA, Unigene28587\_Mf\_liverA, CL1509.Contig2\_Mf\_liverA, Unigene13233\_Mf\_liverA, NM\_010481, Unigene10136\_Mf\_liverA, NM\_009128, NM\_011732, CL2240.Contig1\_Mf\_liverA, Unigene13414\_Mf\_liverA, Unigene49971\_Mf\_liverA, Unigene36190\_Mf\_liverA, Unigene38208\_Mf\_liverA, CL4162.Contig1\_Mf\_liverA, Unigene112\_Mf\_liverA, CL2517.Contig1\_Mf\_liverA, Unigene29510\_Mf\_liverA, CL5986.Contig1\_Mf\_liverA, Unigene15681\_Mf\_liverA, Unigene7350\_Mf\_liverA, Unigene25751\_Mf\_liverA, CL3569.Contig2\_Mf\_liverA, CL4583.Contig2\_Mf\_liverA, Unigene13894\_Mf\_liverA, CL3002.Contig1\_Mf\_liverA, Unigene38280\_Mf\_liverA, Unigene36315\_Mf\_liverA, Unigene36784\_Mf\_liverA, CL2327.Contig1\_Mf\_liverA, Unigene36514\_Mf\_liverA, CL887.Contig2\_Mf\_liverA, Unigene29593\_Mf\_liverA, CL4816.Contig2\_Mf\_liverA, NM\_023294, Unigene33523\_Mf\_liverA, Unigene35037\_Mf\_liverA, CL3684.Contig2\_Mf\_liverA, Unigene14810\_Mf\_liverA, Unigene15252\_Mf\_liverA, CL838.Contig6\_Mf\_liverA, CL2791.Contig1\_Mf\_liverA, CL4770.Contig1\_Mf\_liverA, CL3549.Contig1\_Mf\_liverA, Unigene10135\_Mf\_liverA, CL3750.Contig1\_Mf\_liverA, CL1588.Contig3\_Mf\_liverA, Unigene25101\_Mf\_liverA, CL3112.Contig7\_Mf\_liverA, CL1518.Contig1\_Mf\_liverA, CL5307.Contig1\_Mf\_liverA, NM\_008180, NM\_007585, Unigene36177\_Mf\_liverA, Unigene33525\_Mf\_liverA, NM\_172203, Unigene5335\_Mf\_liverA, Unigene15402\_Mf\_liverA, Unigene30731\_Mf\_liverA, CL912.Contig2\_Mf\_liverA, CL4007.Contig1\_Mf\_liverA, CL1588.Contig1\_Mf\_liverA, CL2355.Contig1\_Mf\_liverA, Unigene28314\_Mf\_liverA, Unigene14809\_Mf\_liverA, Unigene22500\_Mf\_liverA, Unigene30892\_Mf\_liverA, CL3911.Contig2\_Mf\_liverA, Unigene21359\_Mf\_liverA, Unigene12519\_Mf\_liverA, CL336.Contig3\_Mf\_liverA, Unigene28989\_Mf\_liverA, Unigene19954\_Mf\_liverA, NM\_146120 |
| endoplasmic reticulum | CL4816.Contig1\_Mf\_liverA, NM\_023868, Unigene10136\_Mf\_liverA, Unigene5755\_Mf\_liverA, NM\_009128, CL4220.Contig1\_Mf\_liverA, Unigene13414\_Mf\_liverA, CL2240.Contig1\_Mf\_liverA, CL5307.Contig1\_Mf\_liverA, CL1595.Contig1\_Mf\_liverA, Unigene1479\_Mf\_liverA, CL523.Contig1\_Mf\_liverA, Unigene172\_Mf\_liverA, CL838.Contig3\_Mf\_liverA, Unigene37243\_Mf\_liverA, CL4550.Contig3\_Mf\_liverA, Unigene25595\_Mf\_liverA, Unigene35507\_Mf\_liverA, Unigene34628\_Mf\_liverA, Unigene5335\_Mf\_liverA, Unigene31065\_Mf\_liverA, Unigene139\_Mf\_liverA, CL5986.Contig1\_Mf\_liverA, Unigene5428\_Mf\_liverA, Unigene35609\_Mf\_liverA, Unigene13498\_Mf\_liverA, CL4007.Contig1\_Mf\_liverA, Unigene35046\_Mf\_liverA, CL4220.Contig2\_Mf\_liverA, CL4583.Contig2\_Mf\_liverA, Unigene34375\_Mf\_liverA, Unigene36315\_Mf\_liverA, Unigene36784\_Mf\_liverA, Unigene34341\_Mf\_liverA, CL3055.Contig1\_Mf\_liverA, Unigene36514\_Mf\_liverA, NM\_130450, Unigene34630\_Mf\_liverA, Unigene36593\_Mf\_liverA, Unigene7195\_Mf\_liverA, Unigene36662\_Mf\_liverA, CL4816.Contig2\_Mf\_liverA, Unigene8132\_Mf\_liverA, CL887.Contig2\_Mf\_liverA, Unigene36508\_Mf\_liverA, Unigene22500\_Mf\_liverA, Unigene35506\_Mf\_liverA, Unigene3439\_Mf\_liverA, CL3911.Contig2\_Mf\_liverA, Unigene25596\_Mf\_liverA, Unigene21026\_Mf\_liverA, Unigene5712\_Mf\_liverA, Unigene25594\_Mf\_liverA, CL5698.Contig1\_Mf\_liverA, Unigene36937\_Mf\_liverA, Unigene1205\_Mf\_liverA, Unigene5294\_Mf\_liverA, CL336.Contig3\_Mf\_liverA, Unigene23328\_Mf\_liverA, Unigene29882\_Mf\_liverA, Unigene10135\_Mf\_liverA, Unigene32059\_Mf\_liverA |
| extracellular region part | CL1509.Contig3\_Mf\_liverA, Unigene13337\_Mf\_liverA, CL1811.Contig2\_Mf\_liverA, Unigene36404\_Mf\_liverA, Unigene14536\_Mf\_liverA, Unigene12959\_Mf\_liverA, Unigene10351\_Mf\_liverA, CL2339.Contig1\_Mf\_liverA, CL81.Contig1\_Mf\_liverA, Unigene31065\_Mf\_liverA, CL1372.Contig2\_Mf\_liverA, Unigene30039\_Mf\_liverA, CL4299.Contig1\_Mf\_liverA, Unigene37698\_Mf\_liverA, Unigene46931\_Mf\_liverA, CL4583.Contig2\_Mf\_liverA, Unigene13894\_Mf\_liverA, Unigene24974\_Mf\_liverA, Unigene34477\_Mf\_liverA, Unigene38280\_Mf\_liverA, Unigene34375\_Mf\_liverA, Unigene20512\_Mf\_liverA, Unigene34983\_Mf\_liverA, CL4299.Contig2\_Mf\_liverA, NM\_010233, Unigene32316\_Mf\_liverA, NM\_009776, Unigene559\_Mf\_liverA, CL3575.Contig1\_Mf\_liverA, CL1811.Contig1\_Mf\_liverA, Unigene33683\_Mf\_liverA, Unigene8054\_Mf\_liverA, Unigene23328\_Mf\_liverA, CL4650.Contig1\_Mf\_liverA, CL1372.Contig1\_Mf\_liverA, Unigene24887\_Mf\_liverA, Unigene5382\_Mf\_liverA, Unigene34034\_Mf\_liverA, NM\_007585, Unigene36698\_Mf\_liverA, Unigene25595\_Mf\_liverA, CL3312.Contig1\_Mf\_liverA, Unigene941\_Mf\_liverA, Unigene36112\_Mf\_liverA, Unigene35090\_Mf\_liverA, Unigene30322\_Mf\_liverA, Unigene19973\_Mf\_liverA, Unigene13498\_Mf\_liverA, Unigene27249\_Mf\_liverA, Unigene13950\_Mf\_liverA, Unigene17579\_Mf\_liverA, Unigene32908\_Mf\_liverA, CL634.Contig1\_Mf\_liverA, Unigene27248\_Mf\_liverA, Unigene8132\_Mf\_liverA, CL3911.Contig2\_Mf\_liverA, Unigene14541\_Mf\_liverA, CL6018.Contig1\_Mf\_liverA, Unigene30321\_Mf\_liverA, Unigene36439\_Mf\_liverA, CL1509.Contig2\_Mf\_liverA, NM\_146120, Unigene36426\_Mf\_liverA, Unigene31492\_Mf\_liverA, Unigene32059\_Mf\_liverA |
| integral to membrane | Unigene25962\_Mf\_liverA, Unigene21684\_Mf\_liverA, NM\_023868, CL3835.Contig2\_Mf\_liverA, Unigene38913\_Mf\_liverA, Unigene42606\_Mf\_liverA, Unigene13414\_Mf\_liverA, Unigene17310\_Mf\_liverA, Unigene36735\_Mf\_liverA, CL425.Contig1\_Mf\_liverA, Unigene49971\_Mf\_liverA, NM\_011082, CL2339.Contig1\_Mf\_liverA, Unigene38208\_Mf\_liverA, Unigene37526\_Mf\_liverA, Unigene33627\_Mf\_liverA, Unigene36762\_Mf\_liverA, Unigene112\_Mf\_liverA, Unigene35507\_Mf\_liverA, Unigene31065\_Mf\_liverA, Unigene30039\_Mf\_liverA, Unigene37698\_Mf\_liverA, Unigene39886\_Mf\_liverA, Unigene5428\_Mf\_liverA, Unigene4732\_Mf\_liverA, CL4583.Contig2\_Mf\_liverA, Unigene18500\_Mf\_liverA, CL1555.Contig1\_Mf\_liverA, Unigene4938\_Mf\_liverA, CL5586.Contig1\_Mf\_liverA, Unigene5292\_Mf\_liverA, NM\_010141, CL3002.Contig1\_Mf\_liverA, Unigene37268\_Mf\_liverA, Unigene47548\_Mf\_liverA, Unigene36315\_Mf\_liverA, CL1256.Contig1\_Mf\_liverA, Unigene25862\_Mf\_liverA, CL3055.Contig1\_Mf\_liverA, Unigene23503\_Mf\_liverA, CL2327.Contig1\_Mf\_liverA, Unigene25426\_Mf\_liverA, CL4033.Contig1\_Mf\_liverA, Unigene36662\_Mf\_liverA, Unigene29593\_Mf\_liverA, CL887.Contig2\_Mf\_liverA, CL2260.Contig1\_Mf\_liverA, Unigene4725\_Mf\_liverA, Unigene30369\_Mf\_liverA, Unigene21026\_Mf\_liverA, Unigene20371\_Mf\_liverA, Unigene36937\_Mf\_liverA, Unigene4597\_Mf\_liverA, CL838.Contig6\_Mf\_liverA, Unigene5294\_Mf\_liverA, Unigene8054\_Mf\_liverA, Unigene35171\_Mf\_liverA, Unigene10135\_Mf\_liverA, CL2971.Contig1\_Mf\_liverA, Unigene17048\_Mf\_liverA, Unigene16261\_Mf\_liverA, Unigene5755\_Mf\_liverA, CL4220.Contig1\_Mf\_liverA, CL3330.Contig1\_Mf\_liverA, CL5307.Contig1\_Mf\_liverA, Unigene24209\_Mf\_liverA, Unigene1479\_Mf\_liverA, Unigene28873\_Mf\_liverA, Unigene17373\_Mf\_liverA, CL838.Contig3\_Mf\_liverA, NM\_178405, CL4550.Contig3\_Mf\_liverA, NM\_172203, Unigene12786\_Mf\_liverA, Unigene15470\_Mf\_liverA, Unigene34628\_Mf\_liverA, Unigene36112\_Mf\_liverA, Unigene13356\_Mf\_liverA, Unigene941\_Mf\_liverA, Unigene35090\_Mf\_liverA, Unigene15583\_Mf\_liverA, Unigene39011\_Mf\_liverA, CL5062.Contig2\_Mf\_liverA, Unigene33517\_Mf\_liverA, Unigene7264\_Mf\_liverA, Unigene37420\_Mf\_liverA, Unigene35609\_Mf\_liverA, CL4007.Contig1\_Mf\_liverA, Unigene19735\_Mf\_liverA, CL4095.Contig4\_Mf\_liverA, Unigene13950\_Mf\_liverA, CL4283.Contig1\_Mf\_liverA, Unigene27249\_Mf\_liverA, Unigene35046\_Mf\_liverA, CL4220.Contig2\_Mf\_liverA, Unigene37245\_Mf\_liverA, Unigene27547\_Mf\_liverA, Unigene4731\_Mf\_liverA, Unigene281\_Mf\_liverA, Unigene28860\_Mf\_liverA, CL5316.Contig1\_Mf\_liverA, Unigene20372\_Mf\_liverA, CL634.Contig1\_Mf\_liverA, Unigene14070\_Mf\_liverA, Unigene32332\_Mf\_liverA, Unigene860\_Mf\_liverA, Unigene14588\_Mf\_liverA, Unigene34630\_Mf\_liverA, Unigene36593\_Mf\_liverA, Unigene36477\_Mf\_liverA, Unigene27248\_Mf\_liverA, Unigene26336\_Mf\_liverA, Unigene5355\_Mf\_liverA, Unigene22500\_Mf\_liverA, Unigene35506\_Mf\_liverA, Unigene37232\_Mf\_liverA, Unigene5712\_Mf\_liverA, Unigene14541\_Mf\_liverA, Unigene37485\_Mf\_liverA, CL5698.Contig1\_Mf\_liverA, Unigene32943\_Mf\_liverA, CL275.Contig5\_Mf\_liverA, CL2884.Contig2\_Mf\_liverA, Unigene5169\_Mf\_liverA, Unigene29882\_Mf\_liverA, Unigene13233\_Mf\_liverA |
| cell periphery | Unigene25962\_Mf\_liverA, Unigene21684\_Mf\_liverA, CL3835.Contig2\_Mf\_liverA, Unigene38831\_Mf\_liverA, Unigene36404\_Mf\_liverA, Unigene38913\_Mf\_liverA, CL186.Contig3\_Mf\_liverA, Unigene42606\_Mf\_liverA, Unigene13414\_Mf\_liverA, CL425.Contig1\_Mf\_liverA, Unigene49971\_Mf\_liverA, Unigene36190\_Mf\_liverA, CL2339.Contig1\_Mf\_liverA, Unigene37243\_Mf\_liverA, Unigene37526\_Mf\_liverA, Unigene36762\_Mf\_liverA, Unigene112\_Mf\_liverA, Unigene30039\_Mf\_liverA, Unigene37698\_Mf\_liverA, Unigene15681\_Mf\_liverA, Unigene7350\_Mf\_liverA, Unigene5428\_Mf\_liverA, Unigene27592\_Mf\_liverA, Unigene4732\_Mf\_liverA, CL3800.Contig1\_Mf\_liverA, CL4583.Contig2\_Mf\_liverA, Unigene18500\_Mf\_liverA, CL1555.Contig1\_Mf\_liverA, Unigene4938\_Mf\_liverA, CL5586.Contig1\_Mf\_liverA, Unigene24974\_Mf\_liverA, Unigene34477\_Mf\_liverA, CL3002.Contig1\_Mf\_liverA, CL1256.Contig1\_Mf\_liverA, Unigene25862\_Mf\_liverA, CL3055.Contig1\_Mf\_liverA, Unigene30153\_Mf\_liverA, Unigene23503\_Mf\_liverA, CL2327.Contig1\_Mf\_liverA, CL4033.Contig1\_Mf\_liverA, Unigene36662\_Mf\_liverA, Unigene34983\_Mf\_liverA, Unigene29593\_Mf\_liverA, CL887.Contig2\_Mf\_liverA, Unigene35816\_Mf\_liverA, CL2260.Contig1\_Mf\_liverA, NM\_010233, Unigene13940\_Mf\_liverA, Unigene4725\_Mf\_liverA, Unigene30369\_Mf\_liverA, CL2622.Contig2\_Mf\_liverA, Unigene20371\_Mf\_liverA, CL3575.Contig1\_Mf\_liverA, Unigene4597\_Mf\_liverA, CL1907.Contig1\_Mf\_liverA, CL838.Contig6\_Mf\_liverA, Unigene14589\_Mf\_liverA, CL3112.Contig7\_Mf\_liverA, Unigene13097\_Mf\_liverA, Unigene24209\_Mf\_liverA, Unigene28873\_Mf\_liverA, NM\_007585, CL838.Contig3\_Mf\_liverA, CL1165.Contig2\_Mf\_liverA, NM\_178405, CL1263.Contig1\_Mf\_liverA, CL258.Contig1\_Mf\_liverA, NM\_172203, Unigene12786\_Mf\_liverA, Unigene15470\_Mf\_liverA, Unigene36112\_Mf\_liverA, Unigene13356\_Mf\_liverA, Unigene35090\_Mf\_liverA, Unigene139\_Mf\_liverA, Unigene15583\_Mf\_liverA, Unigene33560\_Mf\_liverA, Unigene39011\_Mf\_liverA, Unigene33517\_Mf\_liverA, Unigene30322\_Mf\_liverA, Unigene26250\_Mf\_liverA, Unigene7264\_Mf\_liverA, Unigene35609\_Mf\_liverA, Unigene36189\_Mf\_liverA, CL4007.Contig1\_Mf\_liverA, CL4095.Contig4\_Mf\_liverA, Unigene13950\_Mf\_liverA, Unigene27249\_Mf\_liverA, CL4283.Contig1\_Mf\_liverA, Unigene17579\_Mf\_liverA, CL4220.Contig2\_Mf\_liverA, Unigene37245\_Mf\_liverA, Unigene27547\_Mf\_liverA, Unigene31364\_Mf\_liverA, Unigene4731\_Mf\_liverA, Unigene32908\_Mf\_liverA, CL2355.Contig1\_Mf\_liverA, Unigene20372\_Mf\_liverA, CL634.Contig1\_Mf\_liverA, Unigene36477\_Mf\_liverA, Unigene27248\_Mf\_liverA, Unigene26336\_Mf\_liverA, Unigene5355\_Mf\_liverA, Unigene4636\_Mf\_liverA, Unigene30892\_Mf\_liverA, Unigene37232\_Mf\_liverA, CL3911.Contig2\_Mf\_liverA, Unigene37485\_Mf\_liverA, CL5698.Contig1\_Mf\_liverA, Unigene30321\_Mf\_liverA, CL275.Contig5\_Mf\_liverA, Unigene12519\_Mf\_liverA, CL2884.Contig2\_Mf\_liverA, CL336.Contig3\_Mf\_liverA, Unigene5169\_Mf\_liverA, Unigene28989\_Mf\_liverA, CL1509.Contig2\_Mf\_liverA |
| plasma membrane | Unigene25962\_Mf\_liverA, Unigene21684\_Mf\_liverA, CL3835.Contig2\_Mf\_liverA, Unigene38831\_Mf\_liverA, Unigene36404\_Mf\_liverA, Unigene38913\_Mf\_liverA, Unigene42606\_Mf\_liverA, Unigene13414\_Mf\_liverA, CL425.Contig1\_Mf\_liverA, Unigene49971\_Mf\_liverA, Unigene36190\_Mf\_liverA, CL2339.Contig1\_Mf\_liverA, Unigene37243\_Mf\_liverA, Unigene37526\_Mf\_liverA, Unigene36762\_Mf\_liverA, Unigene112\_Mf\_liverA, Unigene30039\_Mf\_liverA, Unigene37698\_Mf\_liverA, Unigene15681\_Mf\_liverA, Unigene7350\_Mf\_liverA, Unigene5428\_Mf\_liverA, Unigene27592\_Mf\_liverA, Unigene4732\_Mf\_liverA, CL3800.Contig1\_Mf\_liverA, CL4583.Contig2\_Mf\_liverA, Unigene18500\_Mf\_liverA, CL1555.Contig1\_Mf\_liverA, Unigene4938\_Mf\_liverA, CL5586.Contig1\_Mf\_liverA, Unigene24974\_Mf\_liverA, Unigene34477\_Mf\_liverA, CL3002.Contig1\_Mf\_liverA, CL1256.Contig1\_Mf\_liverA, Unigene25862\_Mf\_liverA, CL3055.Contig1\_Mf\_liverA, Unigene30153\_Mf\_liverA, Unigene23503\_Mf\_liverA, CL2327.Contig1\_Mf\_liverA, CL4033.Contig1\_Mf\_liverA, Unigene36662\_Mf\_liverA, Unigene34983\_Mf\_liverA, Unigene29593\_Mf\_liverA, CL887.Contig2\_Mf\_liverA, Unigene35816\_Mf\_liverA, CL2260.Contig1\_Mf\_liverA, NM\_010233, Unigene13940\_Mf\_liverA, Unigene4725\_Mf\_liverA, Unigene30369\_Mf\_liverA, CL2622.Contig2\_Mf\_liverA, Unigene20371\_Mf\_liverA, CL3575.Contig1\_Mf\_liverA, Unigene4597\_Mf\_liverA, CL1907.Contig1\_Mf\_liverA, CL838.Contig6\_Mf\_liverA, Unigene14589\_Mf\_liverA, CL3112.Contig7\_Mf\_liverA, Unigene13097\_Mf\_liverA, Unigene24209\_Mf\_liverA, Unigene28873\_Mf\_liverA, NM\_007585, CL838.Contig3\_Mf\_liverA, CL1165.Contig2\_Mf\_liverA, NM\_178405, CL1263.Contig1\_Mf\_liverA, CL258.Contig1\_Mf\_liverA, NM\_172203, Unigene12786\_Mf\_liverA, Unigene15470\_Mf\_liverA, Unigene36112\_Mf\_liverA, Unigene13356\_Mf\_liverA, Unigene35090\_Mf\_liverA, Unigene139\_Mf\_liverA, Unigene15583\_Mf\_liverA, Unigene33560\_Mf\_liverA, Unigene39011\_Mf\_liverA, Unigene33517\_Mf\_liverA, Unigene30322\_Mf\_liverA, Unigene26250\_Mf\_liverA, Unigene7264\_Mf\_liverA, Unigene35609\_Mf\_liverA, Unigene36189\_Mf\_liverA, CL4007.Contig1\_Mf\_liverA, CL4095.Contig4\_Mf\_liverA, Unigene13950\_Mf\_liverA, CL4283.Contig1\_Mf\_liverA, Unigene27249\_Mf\_liverA, Unigene17579\_Mf\_liverA, CL4220.Contig2\_Mf\_liverA, Unigene37245\_Mf\_liverA, Unigene27547\_Mf\_liverA, Unigene31364\_Mf\_liverA, Unigene4731\_Mf\_liverA, CL2355.Contig1\_Mf\_liverA, Unigene20372\_Mf\_liverA, CL634.Contig1\_Mf\_liverA, Unigene36477\_Mf\_liverA, Unigene27248\_Mf\_liverA, Unigene26336\_Mf\_liverA, Unigene5355\_Mf\_liverA, Unigene4636\_Mf\_liverA, Unigene30892\_Mf\_liverA, Unigene37232\_Mf\_liverA, CL3911.Contig2\_Mf\_liverA, Unigene37485\_Mf\_liverA, CL5698.Contig1\_Mf\_liverA, Unigene30321\_Mf\_liverA, CL275.Contig5\_Mf\_liverA, Unigene12519\_Mf\_liverA, CL2884.Contig2\_Mf\_liverA, CL336.Contig3\_Mf\_liverA, Unigene5169\_Mf\_liverA, Unigene28989\_Mf\_liverA, CL1509.Contig2\_Mf\_liverA |
| apical plasma membrane | Unigene4732\_Mf\_liverA, CL4220.Contig2\_Mf\_liverA, CL5586.Contig1\_Mf\_liverA, Unigene4731\_Mf\_liverA, Unigene38913\_Mf\_liverA, CL4033.Contig1\_Mf\_liverA, Unigene26336\_Mf\_liverA, Unigene28873\_Mf\_liverA, Unigene35816\_Mf\_liverA, Unigene30892\_Mf\_liverA, Unigene4725\_Mf\_liverA, Unigene12786\_Mf\_liverA, Unigene37485\_Mf\_liverA, Unigene112\_Mf\_liverA, Unigene139\_Mf\_liverA, Unigene4597\_Mf\_liverA, Unigene15583\_Mf\_liverA, Unigene35609\_Mf\_liverA, CL336.Contig3\_Mf\_liverA, CL4007.Contig1\_Mf\_liverA, CL4095.Contig4\_Mf\_liverA |
| cytoplasmic part | Unigene21684\_Mf\_liverA, NM\_020559, Unigene38015\_Mf\_liverA, CL3835.Contig2\_Mf\_liverA, Unigene36735\_Mf\_liverA, CL523.Contig1\_Mf\_liverA, Unigene172\_Mf\_liverA, NM\_011082, Unigene36762\_Mf\_liverA, Unigene35507\_Mf\_liverA, Unigene31065\_Mf\_liverA, NM\_011072, Unigene39886\_Mf\_liverA, Unigene5428\_Mf\_liverA, Unigene27339\_Mf\_liverA, Unigene27592\_Mf\_liverA, Unigene4732\_Mf\_liverA, CL5586.Contig1\_Mf\_liverA, Unigene24351\_Mf\_liverA, Unigene37999\_Mf\_liverA, Unigene34341\_Mf\_liverA, Unigene12999\_Mf\_liverA, CL4141.Contig1\_Mf\_liverA, Unigene36662\_Mf\_liverA, NM\_011921, Unigene35816\_Mf\_liverA, Unigene13658\_Mf\_liverA, NM\_010233, Unigene13940\_Mf\_liverA, Unigene4725\_Mf\_liverA, Unigene32316\_Mf\_liverA, NM\_009776, Unigene30003\_Mf\_liverA, Unigene25596\_Mf\_liverA, Unigene25594\_Mf\_liverA, Unigene29461\_Mf\_liverA, Unigene28548\_Mf\_liverA, CL5268.Contig1\_Mf\_liverA, Unigene5294\_Mf\_liverA, Unigene30002\_Mf\_liverA, Unigene23328\_Mf\_liverA, Unigene8054\_Mf\_liverA, Unigene35171\_Mf\_liverA, Unigene14589\_Mf\_liverA, Unigene36626\_Mf\_liverA, CL4816.Contig1\_Mf\_liverA, Unigene5639\_Mf\_liverA, Unigene17048\_Mf\_liverA, Unigene4684\_Mf\_liverA, Unigene5755\_Mf\_liverA, Unigene743\_Mf\_liverA, CL3750.Contig2\_Mf\_liverA, NM\_177093, CL3599.Contig1\_Mf\_liverA, Unigene5382\_Mf\_liverA, Unigene151\_Mf\_liverA, Unigene34591\_Mf\_liverA, CL1595.Contig1\_Mf\_liverA, Unigene1479\_Mf\_liverA, CL838.Contig3\_Mf\_liverA, NM\_080639, NM\_029692, CL1263.Contig1\_Mf\_liverA, CL4550.Contig3\_Mf\_liverA, Unigene12786\_Mf\_liverA, Unigene31231\_Mf\_liverA, Unigene36698\_Mf\_liverA, CL258.Contig1\_Mf\_liverA, CL1716.Contig2\_Mf\_liverA, NM\_008538, Unigene27895\_Mf\_liverA, Unigene36112\_Mf\_liverA, Unigene139\_Mf\_liverA, Unigene4523\_Mf\_liverA, Unigene13498\_Mf\_liverA, Unigene27249\_Mf\_liverA, Unigene4510\_Mf\_liverA, CL4220.Contig2\_Mf\_liverA, Unigene27547\_Mf\_liverA, Unigene19885\_Mf\_liverA, CL1736.Contig2\_Mf\_liverA, Unigene4731\_Mf\_liverA, Unigene12909\_Mf\_liverA, CL5316.Contig1\_Mf\_liverA, Unigene45530\_Mf\_liverA, Unigene32332\_Mf\_liverA, Unigene14588\_Mf\_liverA, Unigene34630\_Mf\_liverA, NR\_003630, Unigene27248\_Mf\_liverA, Unigene7195\_Mf\_liverA, Unigene5355\_Mf\_liverA, Unigene36508\_Mf\_liverA, Unigene8132\_Mf\_liverA, Unigene35506\_Mf\_liverA, Unigene5712\_Mf\_liverA, Unigene37485\_Mf\_liverA, Unigene1205\_Mf\_liverA, Unigene30288\_Mf\_liverA, Unigene28587\_Mf\_liverA, Unigene30528\_Mf\_liverA, Unigene29882\_Mf\_liverA, Unigene13233\_Mf\_liverA, Unigene32059\_Mf\_liverA, Unigene45904\_Mf\_liverA, Unigene25962\_Mf\_liverA, NM\_023868, NM\_010481, Unigene10136\_Mf\_liverA, NM\_009128, NM\_011732, Unigene29727\_Mf\_liverA, Unigene38913\_Mf\_liverA, CL4925.Contig1\_Mf\_liverA, CL186.Contig3\_Mf\_liverA, CL2240.Contig1\_Mf\_liverA, Unigene13414\_Mf\_liverA, Unigene12959\_Mf\_liverA, Unigene49971\_Mf\_liverA, CL425.Contig1\_Mf\_liverA, Unigene36190\_Mf\_liverA, Unigene38208\_Mf\_liverA, Unigene37243\_Mf\_liverA, Unigene14916\_Mf\_liverA, CL4162.Contig1\_Mf\_liverA, Unigene15077\_Mf\_liverA, Unigene112\_Mf\_liverA, CL2517.Contig1\_Mf\_liverA, CL5986.Contig1\_Mf\_liverA, Unigene15681\_Mf\_liverA, Unigene7350\_Mf\_liverA, CL3800.Contig1\_Mf\_liverA, CL4583.Contig2\_Mf\_liverA, CL2478.Contig3\_Mf\_liverA, Unigene38280\_Mf\_liverA, Unigene34375\_Mf\_liverA, CL3002.Contig1\_Mf\_liverA, Unigene36315\_Mf\_liverA, Unigene36784\_Mf\_liverA, CL3055.Contig1\_Mf\_liverA, NM\_028785, Unigene36514\_Mf\_liverA, NM\_009609, NM\_007409, CL4033.Contig1\_Mf\_liverA, CL4816.Contig2\_Mf\_liverA, Unigene29593\_Mf\_liverA, CL887.Contig2\_Mf\_liverA, Unigene33523\_Mf\_liverA, NM\_023294, Unigene3439\_Mf\_liverA, Unigene35037\_Mf\_liverA, Unigene37238\_Mf\_liverA, Unigene21026\_Mf\_liverA, Unigene20371\_Mf\_liverA, Unigene36937\_Mf\_liverA, Unigene14810\_Mf\_liverA, CL3684.Contig2\_Mf\_liverA, Unigene13683\_Mf\_liverA, Unigene15252\_Mf\_liverA, CL2791.Contig1\_Mf\_liverA, CL838.Contig6\_Mf\_liverA, CL3549.Contig1\_Mf\_liverA, Unigene30004\_Mf\_liverA, Unigene10135\_Mf\_liverA, Unigene35476\_Mf\_liverA, CL3750.Contig1\_Mf\_liverA, Unigene25101\_Mf\_liverA, CL5828.Contig2\_Mf\_liverA, CL4220.Contig1\_Mf\_liverA, CL1518.Contig1\_Mf\_liverA, Unigene37262\_Mf\_liverA, CL5307.Contig1\_Mf\_liverA, Unigene12908\_Mf\_liverA, NM\_008180, NM\_007585, CL2702.Contig2\_Mf\_liverA, NR\_033215, NM\_172203, Unigene25595\_Mf\_liverA, Unigene15470\_Mf\_liverA, Unigene14286\_Mf\_liverA, Unigene34628\_Mf\_liverA, Unigene5335\_Mf\_liverA, Unigene34962\_Mf\_liverA, Unigene39011\_Mf\_liverA, Unigene26250\_Mf\_liverA, Unigene15402\_Mf\_liverA, Unigene30731\_Mf\_liverA, Unigene35609\_Mf\_liverA, Unigene36189\_Mf\_liverA, CL912.Contig2\_Mf\_liverA, CL4007.Contig1\_Mf\_liverA, Unigene33580\_Mf\_liverA, Unigene13950\_Mf\_liverA, Unigene35046\_Mf\_liverA, Unigene28459\_Mf\_liverA, NM\_013863, Unigene37245\_Mf\_liverA, CL2355.Contig1\_Mf\_liverA, Unigene281\_Mf\_liverA, Unigene20372\_Mf\_liverA, NM\_011978, NM\_130450, Unigene14809\_Mf\_liverA, Unigene36593\_Mf\_liverA, NM\_027406, Unigene22500\_Mf\_liverA, Unigene37232\_Mf\_liverA, CL3911.Contig2\_Mf\_liverA, Unigene37239\_Mf\_liverA, Unigene37028\_Mf\_liverA, Unigene21359\_Mf\_liverA, CL5698.Contig1\_Mf\_liverA, CL336.Contig3\_Mf\_liverA, Unigene28989\_Mf\_liverA, NM\_146120, Unigene19954\_Mf\_liverA |
| apical part of cell | Unigene4732\_Mf\_liverA, CL4220.Contig2\_Mf\_liverA, CL5586.Contig1\_Mf\_liverA, Unigene4731\_Mf\_liverA, Unigene38913\_Mf\_liverA, CL634.Contig1\_Mf\_liverA, CL4033.Contig1\_Mf\_liverA, Unigene26336\_Mf\_liverA, CL887.Contig2\_Mf\_liverA, Unigene28873\_Mf\_liverA, Unigene35816\_Mf\_liverA, Unigene4725\_Mf\_liverA, Unigene30892\_Mf\_liverA, Unigene12786\_Mf\_liverA, Unigene37485\_Mf\_liverA, Unigene112\_Mf\_liverA, Unigene139\_Mf\_liverA, Unigene4597\_Mf\_liverA, Unigene15583\_Mf\_liverA, Unigene35609\_Mf\_liverA, CL336.Contig3\_Mf\_liverA, CL4007.Contig1\_Mf\_liverA, CL4095.Contig4\_Mf\_liverA |
| organelle membrane | NM\_023868, Unigene38015\_Mf\_liverA, Unigene10136\_Mf\_liverA, CL3835.Contig2\_Mf\_liverA, NM\_009128, Unigene29727\_Mf\_liverA, Unigene38913\_Mf\_liverA, CL4925.Contig1\_Mf\_liverA, Unigene13414\_Mf\_liverA, CL2240.Contig1\_Mf\_liverA, Unigene36735\_Mf\_liverA, CL523.Contig1\_Mf\_liverA, Unigene172\_Mf\_liverA, NM\_011082, Unigene38208\_Mf\_liverA, Unigene112\_Mf\_liverA, Unigene15077\_Mf\_liverA, CL2517.Contig1\_Mf\_liverA, Unigene35507\_Mf\_liverA, Unigene31065\_Mf\_liverA, CL5986.Contig1\_Mf\_liverA, NM\_007820, Unigene39886\_Mf\_liverA, Unigene5428\_Mf\_liverA, CL4583.Contig2\_Mf\_liverA, CL2478.Contig3\_Mf\_liverA, CL5586.Contig1\_Mf\_liverA, CL3002.Contig1\_Mf\_liverA, Unigene36784\_Mf\_liverA, Unigene36315\_Mf\_liverA, Unigene34341\_Mf\_liverA, CL3055.Contig1\_Mf\_liverA, Unigene36514\_Mf\_liverA, CL4033.Contig1\_Mf\_liverA, Unigene36662\_Mf\_liverA, CL4816.Contig2\_Mf\_liverA, NM\_145474, Unigene4725\_Mf\_liverA, Unigene3439\_Mf\_liverA, Unigene21026\_Mf\_liverA, Unigene36937\_Mf\_liverA, NM\_001100181, Unigene5294\_Mf\_liverA, Unigene8054\_Mf\_liverA, Unigene10135\_Mf\_liverA, CL4816.Contig1\_Mf\_liverA, CL2971.Contig1\_Mf\_liverA, Unigene17048\_Mf\_liverA, NM\_029562, Unigene5755\_Mf\_liverA, CL4220.Contig1\_Mf\_liverA, CL3599.Contig1\_Mf\_liverA, CL5307.Contig1\_Mf\_liverA, Unigene1479\_Mf\_liverA, CL2702.Contig2\_Mf\_liverA, NM\_010001, NM\_007822, CL4550.Contig3\_Mf\_liverA, Unigene12786\_Mf\_liverA, NM\_007811, Unigene34628\_Mf\_liverA, Unigene5335\_Mf\_liverA, Unigene139\_Mf\_liverA, NM\_001104531, NM\_025336, Unigene35609\_Mf\_liverA, CL4007.Contig1\_Mf\_liverA, Unigene35046\_Mf\_liverA, CL4220.Contig2\_Mf\_liverA, Unigene281\_Mf\_liverA, CL5316.Contig1\_Mf\_liverA, NM\_011978, NM\_130450, Unigene32332\_Mf\_liverA, Unigene34630\_Mf\_liverA, Unigene36593\_Mf\_liverA, Unigene7195\_Mf\_liverA, Unigene8132\_Mf\_liverA, Unigene36508\_Mf\_liverA, Unigene22500\_Mf\_liverA, Unigene35506\_Mf\_liverA, NM\_019717, Unigene5712\_Mf\_liverA, Unigene37485\_Mf\_liverA, Unigene21359\_Mf\_liverA, CL5698.Contig1\_Mf\_liverA, Unigene1205\_Mf\_liverA, Unigene32943\_Mf\_liverA, NR\_003552, CL336.Contig3\_Mf\_liverA, Unigene28989\_Mf\_liverA, Unigene29882\_Mf\_liverA, Unigene13233\_Mf\_liverA, NM\_007819 |
| intercellular canaliculus | CL4033.Contig1\_Mf\_liverA, Unigene12786\_Mf\_liverA, CL5586.Contig1\_Mf\_liverA, Unigene112\_Mf\_liverA, Unigene38913\_Mf\_liverA |
| cell surface | Unigene35046\_Mf\_liverA, CL4583.Contig2\_Mf\_liverA, Unigene37245\_Mf\_liverA, Unigene31364\_Mf\_liverA, Unigene34477\_Mf\_liverA, CL3835.Contig2\_Mf\_liverA, Unigene5755\_Mf\_liverA, CL3002.Contig1\_Mf\_liverA, Unigene38913\_Mf\_liverA, CL634.Contig1\_Mf\_liverA, Unigene27248\_Mf\_liverA, Unigene1479\_Mf\_liverA, Unigene36508\_Mf\_liverA, CL887.Contig2\_Mf\_liverA, CL838.Contig3\_Mf\_liverA, Unigene37232\_Mf\_liverA, Unigene36698\_Mf\_liverA, Unigene12786\_Mf\_liverA, CL3312.Contig1\_Mf\_liverA, Unigene20371\_Mf\_liverA, Unigene30321\_Mf\_liverA, Unigene30039\_Mf\_liverA, Unigene30322\_Mf\_liverA, Unigene26250\_Mf\_liverA, CL275.Contig5\_Mf\_liverA, CL838.Contig6\_Mf\_liverA, CL2884.Contig2\_Mf\_liverA, Unigene13498\_Mf\_liverA, Unigene27249\_Mf\_liverA |
| endomembrane system | NM\_023868, Unigene28142\_Mf\_liverA, Unigene10136\_Mf\_liverA, NM\_009128, Unigene29727\_Mf\_liverA, Unigene38913\_Mf\_liverA, CL4925.Contig1\_Mf\_liverA, Unigene13414\_Mf\_liverA, CL2240.Contig1\_Mf\_liverA, CL523.Contig1\_Mf\_liverA, Unigene172\_Mf\_liverA, CL4162.Contig1\_Mf\_liverA, Unigene112\_Mf\_liverA, Unigene35507\_Mf\_liverA, CL5986.Contig1\_Mf\_liverA, Unigene5428\_Mf\_liverA, Unigene27592\_Mf\_liverA, CL4583.Contig2\_Mf\_liverA, CL2478.Contig3\_Mf\_liverA, CL5586.Contig1\_Mf\_liverA, Unigene36315\_Mf\_liverA, Unigene36784\_Mf\_liverA, Unigene34341\_Mf\_liverA, Unigene36514\_Mf\_liverA, CL4033.Contig1\_Mf\_liverA, CL4816.Contig2\_Mf\_liverA, Unigene3439\_Mf\_liverA, Unigene21026\_Mf\_liverA, Unigene36937\_Mf\_liverA, Unigene5294\_Mf\_liverA, Unigene8054\_Mf\_liverA, Unigene10135\_Mf\_liverA, CL4816.Contig1\_Mf\_liverA, CL2971.Contig1\_Mf\_liverA, Unigene5755\_Mf\_liverA, CL4220.Contig1\_Mf\_liverA, CL5307.Contig1\_Mf\_liverA, Unigene1479\_Mf\_liverA, CL2098.Contig1\_Mf\_liverA, CL4550.Contig3\_Mf\_liverA, CL1263.Contig1\_Mf\_liverA, Unigene12786\_Mf\_liverA, Unigene5335\_Mf\_liverA, Unigene34628\_Mf\_liverA, Unigene139\_Mf\_liverA, Unigene14212\_Mf\_liverA, Unigene35609\_Mf\_liverA, CL4007.Contig1\_Mf\_liverA, CL4220.Contig2\_Mf\_liverA, Unigene35046\_Mf\_liverA, Unigene281\_Mf\_liverA, NM\_130450, Unigene34630\_Mf\_liverA, Unigene36593\_Mf\_liverA, Unigene7195\_Mf\_liverA, Unigene36508\_Mf\_liverA, Unigene8132\_Mf\_liverA, Unigene22500\_Mf\_liverA, Unigene35506\_Mf\_liverA, Unigene37232\_Mf\_liverA, Unigene5712\_Mf\_liverA, CL5698.Contig1\_Mf\_liverA, Unigene1205\_Mf\_liverA, Unigene32943\_Mf\_liverA, CL336.Contig3\_Mf\_liverA, Unigene28989\_Mf\_liverA, Unigene29882\_Mf\_liverA |
| integral to plasma membrane | Unigene25962\_Mf\_liverA, Unigene42606\_Mf\_liverA, CL425.Contig1\_Mf\_liverA, Unigene49971\_Mf\_liverA, Unigene24209\_Mf\_liverA, CL838.Contig3\_Mf\_liverA, NM\_178405, CL2339.Contig1\_Mf\_liverA, NM\_172203, Unigene36762\_Mf\_liverA, Unigene15470\_Mf\_liverA, Unigene36112\_Mf\_liverA, Unigene13356\_Mf\_liverA, Unigene35090\_Mf\_liverA, Unigene30039\_Mf\_liverA, Unigene39011\_Mf\_liverA, Unigene33517\_Mf\_liverA, Unigene13950\_Mf\_liverA, Unigene4732\_Mf\_liverA, Unigene18500\_Mf\_liverA, CL5586.Contig1\_Mf\_liverA, Unigene4731\_Mf\_liverA, CL1256.Contig1\_Mf\_liverA, Unigene20372\_Mf\_liverA, Unigene25862\_Mf\_liverA, Unigene23503\_Mf\_liverA, Unigene36477\_Mf\_liverA, Unigene29593\_Mf\_liverA, Unigene5355\_Mf\_liverA, Unigene30369\_Mf\_liverA, Unigene37232\_Mf\_liverA, Unigene20371\_Mf\_liverA, CL838.Contig6\_Mf\_liverA, CL2884.Contig2\_Mf\_liverA, Unigene5169\_Mf\_liverA |
| intrinsic to plasma membrane | Unigene25962\_Mf\_liverA, Unigene42606\_Mf\_liverA, CL425.Contig1\_Mf\_liverA, Unigene49971\_Mf\_liverA, Unigene24209\_Mf\_liverA, CL838.Contig3\_Mf\_liverA, NM\_178405, CL2339.Contig1\_Mf\_liverA, NM\_172203, Unigene36762\_Mf\_liverA, Unigene15470\_Mf\_liverA, Unigene36112\_Mf\_liverA, Unigene13356\_Mf\_liverA, Unigene35090\_Mf\_liverA, Unigene30039\_Mf\_liverA, Unigene39011\_Mf\_liverA, Unigene33517\_Mf\_liverA, Unigene13950\_Mf\_liverA, Unigene4732\_Mf\_liverA, Unigene18500\_Mf\_liverA, CL5586.Contig1\_Mf\_liverA, Unigene4731\_Mf\_liverA, CL1256.Contig1\_Mf\_liverA, Unigene20372\_Mf\_liverA, Unigene25862\_Mf\_liverA, Unigene23503\_Mf\_liverA, Unigene36477\_Mf\_liverA, Unigene29593\_Mf\_liverA, Unigene5355\_Mf\_liverA, Unigene30369\_Mf\_liverA, Unigene37232\_Mf\_liverA, Unigene20371\_Mf\_liverA, CL3575.Contig1\_Mf\_liverA, CL838.Contig6\_Mf\_liverA, CL2884.Contig2\_Mf\_liverA, Unigene5169\_Mf\_liverA |
| multivesicular body | Unigene37245\_Mf\_liverA, Unigene21026\_Mf\_liverA, Unigene15470\_Mf\_liverA, Unigene8132\_Mf\_liverA, CL3002.Contig1\_Mf\_liverA |
| lysosome | CL4583.Contig2\_Mf\_liverA, CL1736.Contig2\_Mf\_liverA, Unigene38280\_Mf\_liverA, CL3835.Contig2\_Mf\_liverA, CL3002.Contig1\_Mf\_liverA, CL3055.Contig1\_Mf\_liverA, CL4925.Contig1\_Mf\_liverA, Unigene32332\_Mf\_liverA, Unigene5382\_Mf\_liverA, Unigene13414\_Mf\_liverA, Unigene36662\_Mf\_liverA, Unigene8132\_Mf\_liverA, CL838.Contig3\_Mf\_liverA, Unigene37232\_Mf\_liverA, Unigene36112\_Mf\_liverA, Unigene39886\_Mf\_liverA, CL838.Contig6\_Mf\_liverA, Unigene30528\_Mf\_liverA |
| plasma membrane part | Unigene25962\_Mf\_liverA, CL3835.Contig2\_Mf\_liverA, Unigene38913\_Mf\_liverA, Unigene42606\_Mf\_liverA, CL425.Contig1\_Mf\_liverA, Unigene49971\_Mf\_liverA, CL2339.Contig1\_Mf\_liverA, Unigene37243\_Mf\_liverA, Unigene36762\_Mf\_liverA, Unigene112\_Mf\_liverA, Unigene30039\_Mf\_liverA, Unigene7350\_Mf\_liverA, Unigene4732\_Mf\_liverA, CL4583.Contig2\_Mf\_liverA, Unigene18500\_Mf\_liverA, CL5586.Contig1\_Mf\_liverA, Unigene4938\_Mf\_liverA, CL3002.Contig1\_Mf\_liverA, CL1256.Contig1\_Mf\_liverA, Unigene25862\_Mf\_liverA, Unigene23503\_Mf\_liverA, CL4033.Contig1\_Mf\_liverA, Unigene29593\_Mf\_liverA, CL887.Contig2\_Mf\_liverA, Unigene35816\_Mf\_liverA, CL2260.Contig1\_Mf\_liverA, NM\_010233, Unigene30369\_Mf\_liverA, Unigene4725\_Mf\_liverA, Unigene20371\_Mf\_liverA, CL3575.Contig1\_Mf\_liverA, Unigene4597\_Mf\_liverA, CL838.Contig6\_Mf\_liverA, Unigene24209\_Mf\_liverA, Unigene28873\_Mf\_liverA, NM\_007585, CL838.Contig3\_Mf\_liverA, NM\_178405, NM\_172203, Unigene12786\_Mf\_liverA, Unigene15470\_Mf\_liverA, Unigene13356\_Mf\_liverA, Unigene36112\_Mf\_liverA, Unigene35090\_Mf\_liverA, Unigene139\_Mf\_liverA, Unigene15583\_Mf\_liverA, Unigene33517\_Mf\_liverA, Unigene30322\_Mf\_liverA, Unigene39011\_Mf\_liverA, Unigene35609\_Mf\_liverA, CL4007.Contig1\_Mf\_liverA, CL4095.Contig4\_Mf\_liverA, Unigene13950\_Mf\_liverA, CL4220.Contig2\_Mf\_liverA, Unigene31364\_Mf\_liverA, Unigene37245\_Mf\_liverA, Unigene4731\_Mf\_liverA, Unigene20372\_Mf\_liverA, CL634.Contig1\_Mf\_liverA, Unigene36477\_Mf\_liverA, Unigene26336\_Mf\_liverA, Unigene4636\_Mf\_liverA, Unigene5355\_Mf\_liverA, Unigene30892\_Mf\_liverA, Unigene37232\_Mf\_liverA, Unigene37485\_Mf\_liverA, Unigene30321\_Mf\_liverA, CL275.Contig5\_Mf\_liverA, CL2884.Contig2\_Mf\_liverA, CL336.Contig3\_Mf\_liverA, Unigene5169\_Mf\_liverA |
| condensed nuclear chromosome kinetochore | CL5268.Contig1\_Mf\_liverA, Unigene45530\_Mf\_liverA, NM\_023294 |
| cytosol | Unigene35476\_Mf\_liverA, Unigene4684\_Mf\_liverA, Unigene38015\_Mf\_liverA, CL186.Contig3\_Mf\_liverA, CL4925.Contig1\_Mf\_liverA, Unigene37262\_Mf\_liverA, Unigene151\_Mf\_liverA, Unigene12959\_Mf\_liverA, Unigene12908\_Mf\_liverA, Unigene36190\_Mf\_liverA, CL1263.Contig1\_Mf\_liverA, Unigene38208\_Mf\_liverA, Unigene31231\_Mf\_liverA, CL258.Contig1\_Mf\_liverA, Unigene34962\_Mf\_liverA, Unigene26250\_Mf\_liverA, Unigene15402\_Mf\_liverA, Unigene15681\_Mf\_liverA, Unigene39886\_Mf\_liverA, Unigene7350\_Mf\_liverA, Unigene36189\_Mf\_liverA, Unigene33580\_Mf\_liverA, Unigene27339\_Mf\_liverA, Unigene27592\_Mf\_liverA, CL3800.Contig1\_Mf\_liverA, CL4583.Contig2\_Mf\_liverA, Unigene19885\_Mf\_liverA, CL2478.Contig3\_Mf\_liverA, Unigene24351\_Mf\_liverA, Unigene12909\_Mf\_liverA, CL2355.Contig1\_Mf\_liverA, Unigene45530\_Mf\_liverA, Unigene12999\_Mf\_liverA, CL4141.Contig1\_Mf\_liverA, Unigene36662\_Mf\_liverA, Unigene35816\_Mf\_liverA, Unigene13658\_Mf\_liverA, Unigene33523\_Mf\_liverA, Unigene13940\_Mf\_liverA, Unigene32316\_Mf\_liverA, Unigene35037\_Mf\_liverA, Unigene25596\_Mf\_liverA, Unigene30003\_Mf\_liverA, Unigene37238\_Mf\_liverA, CL5698.Contig1\_Mf\_liverA, Unigene30288\_Mf\_liverA, Unigene28587\_Mf\_liverA, CL3684.Contig2\_Mf\_liverA, Unigene28548\_Mf\_liverA, CL5268.Contig1\_Mf\_liverA, CL3549.Contig1\_Mf\_liverA, Unigene30004\_Mf\_liverA, Unigene30002\_Mf\_liverA, Unigene14589\_Mf\_liverA, Unigene45904\_Mf\_liverA |
| high-density lipoprotein particle | Unigene46931\_Mf\_liverA, CL3911.Contig2\_Mf\_liverA, Unigene36698\_Mf\_liverA, Unigene13498\_Mf\_liverA, Unigene559\_Mf\_liverA |
| mitochondrion | Unigene35476\_Mf\_liverA, CL3750.Contig1\_Mf\_liverA, Unigene17048\_Mf\_liverA, NM\_020559, NM\_010481, CL5828.Contig2\_Mf\_liverA, Unigene10136\_Mf\_liverA, Unigene743\_Mf\_liverA, Unigene29727\_Mf\_liverA, CL1518.Contig1\_Mf\_liverA, CL3750.Contig2\_Mf\_liverA, CL3599.Contig1\_Mf\_liverA, Unigene36735\_Mf\_liverA, CL425.Contig1\_Mf\_liverA, Unigene34591\_Mf\_liverA, Unigene36190\_Mf\_liverA, CL2702.Contig2\_Mf\_liverA, Unigene38208\_Mf\_liverA, Unigene14916\_Mf\_liverA, CL1716.Contig2\_Mf\_liverA, CL2517.Contig1\_Mf\_liverA, Unigene14286\_Mf\_liverA, Unigene31065\_Mf\_liverA, Unigene30731\_Mf\_liverA, Unigene36189\_Mf\_liverA, Unigene4510\_Mf\_liverA, Unigene4732\_Mf\_liverA, Unigene28459\_Mf\_liverA, Unigene27547\_Mf\_liverA, CL1736.Contig2\_Mf\_liverA, Unigene4731\_Mf\_liverA, Unigene37999\_Mf\_liverA, CL5316.Contig1\_Mf\_liverA, Unigene14588\_Mf\_liverA, Unigene36662\_Mf\_liverA, CL887.Contig2\_Mf\_liverA, Unigene29593\_Mf\_liverA, Unigene35816\_Mf\_liverA, Unigene13658\_Mf\_liverA, Unigene3439\_Mf\_liverA, Unigene37239\_Mf\_liverA, Unigene35037\_Mf\_liverA, Unigene37028\_Mf\_liverA, Unigene37485\_Mf\_liverA, Unigene21359\_Mf\_liverA, Unigene29461\_Mf\_liverA, Unigene15252\_Mf\_liverA, CL2791.Contig1\_Mf\_liverA, Unigene28548\_Mf\_liverA, Unigene13233\_Mf\_liverA, Unigene35171\_Mf\_liverA, Unigene19954\_Mf\_liverA, Unigene36626\_Mf\_liverA |
| condensed nuclear chromosome outer kinetochore | CL5268.Contig1\_Mf\_liverA, Unigene45530\_Mf\_liverA |
| anchored to membrane | Unigene13097\_Mf\_liverA, Unigene37698\_Mf\_liverA, Unigene34477\_Mf\_liverA, Unigene5169\_Mf\_liverA, CL3575.Contig1\_Mf\_liverA, Unigene36112\_Mf\_liverA, Unigene38831\_Mf\_liverA, CL634.Contig1\_Mf\_liverA |
| external side of plasma membrane | CL4583.Contig2\_Mf\_liverA, Unigene37245\_Mf\_liverA, Unigene31364\_Mf\_liverA, CL3835.Contig2\_Mf\_liverA, CL3002.Contig1\_Mf\_liverA, Unigene30321\_Mf\_liverA, CL634.Contig1\_Mf\_liverA, Unigene30322\_Mf\_liverA, CL275.Contig5\_Mf\_liverA, CL2884.Contig2\_Mf\_liverA, CL887.Contig2\_Mf\_liverA |
| vesicular fraction | NM\_007822, NM\_023868, NM\_029562, NM\_007811, NM\_009128, NM\_001104531, NM\_011978, NM\_130450, NM\_007820, NM\_001100181, NR\_003552, NM\_007819, NM\_010001, NM\_145474, NM\_178405 |
| condensed nuclear chromosome, centromeric region | CL5268.Contig1\_Mf\_liverA, Unigene45530\_Mf\_liverA, NM\_023294 |
| MHC class II protein complex | Unigene37245\_Mf\_liverA, CL3835.Contig2\_Mf\_liverA, CL3002.Contig1\_Mf\_liverA |
| endoplasmic reticulum lumen | CL3911.Contig2\_Mf\_liverA, Unigene25595\_Mf\_liverA, Unigene25596\_Mf\_liverA, Unigene34375\_Mf\_liverA, Unigene13414\_Mf\_liverA, Unigene13498\_Mf\_liverA, Unigene23328\_Mf\_liverA, Unigene1479\_Mf\_liverA |
| perinuclear region of cytoplasm | Unigene32316\_Mf\_liverA, Unigene25962\_Mf\_liverA, Unigene21684\_Mf\_liverA, CL4583.Contig2\_Mf\_liverA, CL4162.Contig1\_Mf\_liverA, Unigene21026\_Mf\_liverA, Unigene5712\_Mf\_liverA, Unigene20371\_Mf\_liverA, Unigene20372\_Mf\_liverA, CL186.Contig3\_Mf\_liverA, Unigene39011\_Mf\_liverA, CL838.Contig6\_Mf\_liverA, Unigene27248\_Mf\_liverA, Unigene1479\_Mf\_liverA, Unigene8132\_Mf\_liverA, Unigene35816\_Mf\_liverA, CL838.Contig3\_Mf\_liverA, Unigene27249\_Mf\_liverA |
| MHC protein complex | Unigene37245\_Mf\_liverA, CL275.Contig5\_Mf\_liverA, Unigene4938\_Mf\_liverA, CL3835.Contig2\_Mf\_liverA, CL3002.Contig1\_Mf\_liverA |
| hemoglobin complex | Unigene12999\_Mf\_liverA, CL3684.Contig2\_Mf\_liverA |
| Ndc80 complex | CL5268.Contig1\_Mf\_liverA, Unigene45530\_Mf\_liverA |
| membrane | Unigene21684\_Mf\_liverA, Unigene38015\_Mf\_liverA, CL3835.Contig2\_Mf\_liverA, Unigene13337\_Mf\_liverA, Unigene38831\_Mf\_liverA, NM\_178050, Unigene36404\_Mf\_liverA, Unigene17310\_Mf\_liverA, Unigene36735\_Mf\_liverA, CL523.Contig1\_Mf\_liverA, Unigene172\_Mf\_liverA, NM\_011082, CL2339.Contig1\_Mf\_liverA, Unigene33627\_Mf\_liverA, Unigene36762\_Mf\_liverA, Unigene35507\_Mf\_liverA, Unigene31065\_Mf\_liverA, NM\_007820, Unigene37698\_Mf\_liverA, Unigene39886\_Mf\_liverA, Unigene5428\_Mf\_liverA, Unigene27592\_Mf\_liverA, Unigene4732\_Mf\_liverA, Unigene18500\_Mf\_liverA, CL1555.Contig1\_Mf\_liverA, CL5586.Contig1\_Mf\_liverA, Unigene37268\_Mf\_liverA, Unigene47548\_Mf\_liverA, CL1256.Contig1\_Mf\_liverA, Unigene34341\_Mf\_liverA, Unigene25862\_Mf\_liverA, Unigene30153\_Mf\_liverA, Unigene25426\_Mf\_liverA, Unigene36662\_Mf\_liverA, Unigene35816\_Mf\_liverA, CL2260.Contig1\_Mf\_liverA, NM\_010233, NM\_145474, Unigene13940\_Mf\_liverA, Unigene4725\_Mf\_liverA, Unigene30369\_Mf\_liverA, NM\_172926, CL2622.Contig2\_Mf\_liverA, CL3575.Contig1\_Mf\_liverA, Unigene5294\_Mf\_liverA, Unigene8054\_Mf\_liverA, Unigene35171\_Mf\_liverA, CL4650.Contig1\_Mf\_liverA, Unigene14589\_Mf\_liverA, CL4816.Contig1\_Mf\_liverA, CL2971.Contig1\_Mf\_liverA, Unigene16261\_Mf\_liverA, Unigene17048\_Mf\_liverA, NM\_029562, Unigene5755\_Mf\_liverA, Unigene13097\_Mf\_liverA, CL3599.Contig1\_Mf\_liverA, CL3330.Contig1\_Mf\_liverA, Unigene24209\_Mf\_liverA, Unigene1479\_Mf\_liverA, CL838.Contig3\_Mf\_liverA, Unigene17373\_Mf\_liverA, NM\_010001, CL1263.Contig1\_Mf\_liverA, CL4550.Contig3\_Mf\_liverA, Unigene12786\_Mf\_liverA, CL258.Contig1\_Mf\_liverA, NM\_007811, NM\_008538, Unigene36112\_Mf\_liverA, Unigene941\_Mf\_liverA, NM\_001104531, Unigene139\_Mf\_liverA, Unigene15583\_Mf\_liverA, Unigene33560\_Mf\_liverA, Unigene7264\_Mf\_liverA, Unigene37420\_Mf\_liverA, Unigene19735\_Mf\_liverA, CL4095.Contig4\_Mf\_liverA, Unigene27249\_Mf\_liverA, CL4283.Contig1\_Mf\_liverA, Unigene17579\_Mf\_liverA, CL4220.Contig2\_Mf\_liverA, Unigene27547\_Mf\_liverA, Unigene31364\_Mf\_liverA, Unigene4731\_Mf\_liverA, CL5316.Contig1\_Mf\_liverA, Unigene14070\_Mf\_liverA, Unigene32332\_Mf\_liverA, Unigene4723\_Mf\_liverA, Unigene860\_Mf\_liverA, Unigene14588\_Mf\_liverA, Unigene34630\_Mf\_liverA, Unigene36477\_Mf\_liverA, Unigene27248\_Mf\_liverA, Unigene7195\_Mf\_liverA, Unigene5355\_Mf\_liverA, Unigene36508\_Mf\_liverA, Unigene8132\_Mf\_liverA, Unigene35506\_Mf\_liverA, NM\_019717, Unigene5712\_Mf\_liverA, Unigene37485\_Mf\_liverA, Unigene14541\_Mf\_liverA, Unigene1205\_Mf\_liverA, Unigene30321\_Mf\_liverA, Unigene32943\_Mf\_liverA, CL275.Contig5\_Mf\_liverA, NR\_003552, Unigene5169\_Mf\_liverA, CL1509.Contig2\_Mf\_liverA, Unigene29882\_Mf\_liverA, Unigene13233\_Mf\_liverA, Unigene32202\_Mf\_liverA, Unigene25962\_Mf\_liverA, NM\_023868, Unigene10136\_Mf\_liverA, NM\_009128, Unigene29727\_Mf\_liverA, Unigene38913\_Mf\_liverA, Unigene42606\_Mf\_liverA, CL4925.Contig1\_Mf\_liverA, CL2240.Contig1\_Mf\_liverA, Unigene13414\_Mf\_liverA, Unigene49971\_Mf\_liverA, CL425.Contig1\_Mf\_liverA, Unigene36190\_Mf\_liverA, Unigene38208\_Mf\_liverA, Unigene37243\_Mf\_liverA, Unigene37526\_Mf\_liverA, Unigene15077\_Mf\_liverA, Unigene112\_Mf\_liverA, CL2517.Contig1\_Mf\_liverA, Unigene30039\_Mf\_liverA, CL5986.Contig1\_Mf\_liverA, Unigene15681\_Mf\_liverA, Unigene7350\_Mf\_liverA, CL3800.Contig1\_Mf\_liverA, CL4583.Contig2\_Mf\_liverA, CL2478.Contig3\_Mf\_liverA, Unigene4938\_Mf\_liverA, Unigene24974\_Mf\_liverA, Unigene34477\_Mf\_liverA, Unigene5292\_Mf\_liverA, NM\_010141, CL3002.Contig1\_Mf\_liverA, Unigene36315\_Mf\_liverA, Unigene36784\_Mf\_liverA, CL3055.Contig1\_Mf\_liverA, Unigene23503\_Mf\_liverA, Unigene36514\_Mf\_liverA, CL2327.Contig1\_Mf\_liverA, CL4033.Contig1\_Mf\_liverA, CL4816.Contig2\_Mf\_liverA, Unigene34983\_Mf\_liverA, Unigene29593\_Mf\_liverA, CL887.Contig2\_Mf\_liverA, Unigene3439\_Mf\_liverA, Unigene21026\_Mf\_liverA, Unigene20371\_Mf\_liverA, Unigene36937\_Mf\_liverA, Unigene4597\_Mf\_liverA, CL1907.Contig1\_Mf\_liverA, NM\_001100181, CL838.Contig6\_Mf\_liverA, Unigene10135\_Mf\_liverA, CL4220.Contig1\_Mf\_liverA, CL3112.Contig7\_Mf\_liverA, CL5307.Contig1\_Mf\_liverA, NM\_008180, Unigene28873\_Mf\_liverA, NM\_007585, CL2702.Contig2\_Mf\_liverA, CL1165.Contig2\_Mf\_liverA, NM\_178405, CL2098.Contig1\_Mf\_liverA, NM\_007822, NM\_172203, Unigene15470\_Mf\_liverA, Unigene34628\_Mf\_liverA, Unigene13356\_Mf\_liverA, Unigene5335\_Mf\_liverA, Unigene35090\_Mf\_liverA, Unigene39011\_Mf\_liverA, Unigene30322\_Mf\_liverA, Unigene33517\_Mf\_liverA, CL5062.Contig2\_Mf\_liverA, Unigene26250\_Mf\_liverA, NM\_025336, Unigene35609\_Mf\_liverA, NM\_145218, Unigene36189\_Mf\_liverA, CL4007.Contig1\_Mf\_liverA, Unigene13950\_Mf\_liverA, Unigene35046\_Mf\_liverA, Unigene37245\_Mf\_liverA, CL2355.Contig1\_Mf\_liverA, Unigene281\_Mf\_liverA, Unigene28860\_Mf\_liverA, Unigene20372\_Mf\_liverA, NM\_011978, CL634.Contig1\_Mf\_liverA, NM\_130450, Unigene36593\_Mf\_liverA, Unigene26336\_Mf\_liverA, Unigene4636\_Mf\_liverA, Unigene22500\_Mf\_liverA, Unigene30892\_Mf\_liverA, Unigene37232\_Mf\_liverA, CL3911.Contig2\_Mf\_liverA, Unigene21359\_Mf\_liverA, CL5698.Contig1\_Mf\_liverA, Unigene12519\_Mf\_liverA, CL2884.Contig2\_Mf\_liverA, CL336.Contig3\_Mf\_liverA, Unigene28989\_Mf\_liverA, NM\_007819, NM\_153388 |
| lysosomal membrane | Unigene32332\_Mf\_liverA, CL4583.Contig2\_Mf\_liverA, Unigene39886\_Mf\_liverA, CL3835.Contig2\_Mf\_liverA, CL3002.Contig1\_Mf\_liverA, CL3055.Contig1\_Mf\_liverA, CL4925.Contig1\_Mf\_liverA |
| protein-lipid complex | Unigene46931\_Mf\_liverA, CL3911.Contig2\_Mf\_liverA, Unigene36698\_Mf\_liverA, Unigene34477\_Mf\_liverA, Unigene13498\_Mf\_liverA, Unigene559\_Mf\_liverA |
| plasma lipoprotein particle | Unigene46931\_Mf\_liverA, CL3911.Contig2\_Mf\_liverA, Unigene36698\_Mf\_liverA, Unigene34477\_Mf\_liverA, Unigene13498\_Mf\_liverA, Unigene559\_Mf\_liverA |
| lytic vacuole | CL4583.Contig2\_Mf\_liverA, CL1736.Contig2\_Mf\_liverA, Unigene38280\_Mf\_liverA, CL3835.Contig2\_Mf\_liverA, CL3002.Contig1\_Mf\_liverA, CL3055.Contig1\_Mf\_liverA, CL4925.Contig1\_Mf\_liverA, Unigene32332\_Mf\_liverA, Unigene5382\_Mf\_liverA, Unigene13414\_Mf\_liverA, Unigene36662\_Mf\_liverA, Unigene8132\_Mf\_liverA, CL838.Contig3\_Mf\_liverA, Unigene37232\_Mf\_liverA, Unigene36112\_Mf\_liverA, Unigene39886\_Mf\_liverA, CL838.Contig6\_Mf\_liverA, Unigene30528\_Mf\_liverA |
| membrane part | Unigene21684\_Mf\_liverA, CL3835.Contig2\_Mf\_liverA, Unigene38831\_Mf\_liverA, NM\_178050, Unigene17310\_Mf\_liverA, Unigene36735\_Mf\_liverA, CL523.Contig1\_Mf\_liverA, Unigene172\_Mf\_liverA, NM\_011082, CL2339.Contig1\_Mf\_liverA, Unigene33627\_Mf\_liverA, Unigene36762\_Mf\_liverA, Unigene35507\_Mf\_liverA, Unigene31065\_Mf\_liverA, Unigene37698\_Mf\_liverA, Unigene39886\_Mf\_liverA, Unigene5428\_Mf\_liverA, Unigene4732\_Mf\_liverA, Unigene18500\_Mf\_liverA, CL1555.Contig1\_Mf\_liverA, CL5586.Contig1\_Mf\_liverA, Unigene37268\_Mf\_liverA, Unigene47548\_Mf\_liverA, CL1256.Contig1\_Mf\_liverA, Unigene34341\_Mf\_liverA, Unigene25862\_Mf\_liverA, Unigene25426\_Mf\_liverA, Unigene36662\_Mf\_liverA, Unigene35816\_Mf\_liverA, CL2260.Contig1\_Mf\_liverA, NM\_010233, Unigene4725\_Mf\_liverA, Unigene30369\_Mf\_liverA, NM\_172926, CL3575.Contig1\_Mf\_liverA, Unigene5294\_Mf\_liverA, Unigene8054\_Mf\_liverA, Unigene35171\_Mf\_liverA, CL4816.Contig1\_Mf\_liverA, CL2971.Contig1\_Mf\_liverA, Unigene16261\_Mf\_liverA, Unigene17048\_Mf\_liverA, Unigene5755\_Mf\_liverA, Unigene13097\_Mf\_liverA, CL3330.Contig1\_Mf\_liverA, Unigene24209\_Mf\_liverA, Unigene1479\_Mf\_liverA, CL838.Contig3\_Mf\_liverA, Unigene17373\_Mf\_liverA, CL4550.Contig3\_Mf\_liverA, Unigene12786\_Mf\_liverA, Unigene36112\_Mf\_liverA, Unigene941\_Mf\_liverA, Unigene139\_Mf\_liverA, Unigene15583\_Mf\_liverA, Unigene7264\_Mf\_liverA, Unigene37420\_Mf\_liverA, Unigene19735\_Mf\_liverA, CL4095.Contig4\_Mf\_liverA, Unigene27249\_Mf\_liverA, CL4283.Contig1\_Mf\_liverA, CL4220.Contig2\_Mf\_liverA, Unigene27547\_Mf\_liverA, Unigene31364\_Mf\_liverA, Unigene4731\_Mf\_liverA, CL5316.Contig1\_Mf\_liverA, Unigene14070\_Mf\_liverA, Unigene32332\_Mf\_liverA, Unigene860\_Mf\_liverA, Unigene14588\_Mf\_liverA, Unigene34630\_Mf\_liverA, Unigene36477\_Mf\_liverA, Unigene27248\_Mf\_liverA, Unigene7195\_Mf\_liverA, Unigene5355\_Mf\_liverA, Unigene36508\_Mf\_liverA, Unigene35506\_Mf\_liverA, NM\_019717, Unigene5712\_Mf\_liverA, Unigene37485\_Mf\_liverA, Unigene14541\_Mf\_liverA, Unigene1205\_Mf\_liverA, Unigene30321\_Mf\_liverA, Unigene32943\_Mf\_liverA, CL275.Contig5\_Mf\_liverA, Unigene5169\_Mf\_liverA, Unigene29882\_Mf\_liverA, Unigene13233\_Mf\_liverA, Unigene25962\_Mf\_liverA, NM\_023868, Unigene10136\_Mf\_liverA, NM\_009128, Unigene38913\_Mf\_liverA, Unigene42606\_Mf\_liverA, CL4925.Contig1\_Mf\_liverA, CL2240.Contig1\_Mf\_liverA, Unigene13414\_Mf\_liverA, Unigene49971\_Mf\_liverA, CL425.Contig1\_Mf\_liverA, Unigene37243\_Mf\_liverA, Unigene38208\_Mf\_liverA, Unigene37526\_Mf\_liverA, Unigene112\_Mf\_liverA, Unigene30039\_Mf\_liverA, CL5986.Contig1\_Mf\_liverA, Unigene7350\_Mf\_liverA, CL4583.Contig2\_Mf\_liverA, CL2478.Contig3\_Mf\_liverA, Unigene4938\_Mf\_liverA, Unigene5292\_Mf\_liverA, Unigene34477\_Mf\_liverA, NM\_010141, CL3002.Contig1\_Mf\_liverA, Unigene36315\_Mf\_liverA, Unigene36784\_Mf\_liverA, CL3055.Contig1\_Mf\_liverA, Unigene23503\_Mf\_liverA, Unigene36514\_Mf\_liverA, CL2327.Contig1\_Mf\_liverA, CL4033.Contig1\_Mf\_liverA, CL4816.Contig2\_Mf\_liverA, CL887.Contig2\_Mf\_liverA, Unigene29593\_Mf\_liverA, Unigene3439\_Mf\_liverA, Unigene21026\_Mf\_liverA, Unigene20371\_Mf\_liverA, Unigene36937\_Mf\_liverA, Unigene4597\_Mf\_liverA, CL838.Contig6\_Mf\_liverA, Unigene10135\_Mf\_liverA, CL4220.Contig1\_Mf\_liverA, CL5307.Contig1\_Mf\_liverA, NM\_008180, Unigene28873\_Mf\_liverA, NM\_007585, NM\_178405, CL2098.Contig1\_Mf\_liverA, NM\_007822, NM\_172203, Unigene15470\_Mf\_liverA, Unigene34628\_Mf\_liverA, Unigene13356\_Mf\_liverA, Unigene5335\_Mf\_liverA, Unigene35090\_Mf\_liverA, Unigene39011\_Mf\_liverA, Unigene30322\_Mf\_liverA, Unigene33517\_Mf\_liverA, CL5062.Contig2\_Mf\_liverA, Unigene35609\_Mf\_liverA, NM\_145218, CL4007.Contig1\_Mf\_liverA, Unigene13950\_Mf\_liverA, Unigene35046\_Mf\_liverA, Unigene37245\_Mf\_liverA, Unigene281\_Mf\_liverA, Unigene28860\_Mf\_liverA, NM\_011978, Unigene20372\_Mf\_liverA, CL634.Contig1\_Mf\_liverA, NM\_130450, Unigene36593\_Mf\_liverA, Unigene26336\_Mf\_liverA, Unigene4636\_Mf\_liverA, Unigene22500\_Mf\_liverA, Unigene30892\_Mf\_liverA, Unigene37232\_Mf\_liverA, CL5698.Contig1\_Mf\_liverA, Unigene12519\_Mf\_liverA, CL2884.Contig2\_Mf\_liverA, CL336.Contig3\_Mf\_liverA, NM\_007819, NM\_153388 |
| membrane fraction | NM\_013863, NM\_023868, NM\_029562, NM\_009128, NM\_011978, NM\_130450, NM\_010001, NM\_145474, NM\_178405, NM\_007822, NM\_007811, NM\_001104531, NM\_001100181, NM\_007820, NM\_011072, NR\_003552, NM\_007819 |
| Ada2/Gcn5/Ada3 transcription activator complex | Unigene21163\_Mf\_liverA, Unigene1479\_Mf\_liverA, Unigene30288\_Mf\_liverA |
| vacuolar part | CL4583.Contig2\_Mf\_liverA, CL3835.Contig2\_Mf\_liverA, CL3002.Contig1\_Mf\_liverA, Unigene36112\_Mf\_liverA, CL3055.Contig1\_Mf\_liverA, CL4925.Contig1\_Mf\_liverA, Unigene32332\_Mf\_liverA, Unigene39886\_Mf\_liverA, Unigene5382\_Mf\_liverA |
| extracellular matrix | CL1372.Contig1\_Mf\_liverA, Unigene34477\_Mf\_liverA, Unigene32908\_Mf\_liverA, Unigene34375\_Mf\_liverA, Unigene24887\_Mf\_liverA, Unigene5382\_Mf\_liverA, Unigene20512\_Mf\_liverA, Unigene8132\_Mf\_liverA, NM\_007585, NM\_080639, NM\_010233, Unigene36112\_Mf\_liverA, Unigene35090\_Mf\_liverA, CL1372.Contig2\_Mf\_liverA, Unigene33683\_Mf\_liverA, Unigene23328\_Mf\_liverA, Unigene8054\_Mf\_liverA, Unigene31492\_Mf\_liverA |
| mitochondrial matrix | Unigene14916\_Mf\_liverA, NM\_010481, Unigene37028\_Mf\_liverA, CL2517.Contig1\_Mf\_liverA, Unigene21359\_Mf\_liverA, Unigene15252\_Mf\_liverA, CL3599.Contig1\_Mf\_liverA, CL2791.Contig1\_Mf\_liverA, Unigene30731\_Mf\_liverA, Unigene35816\_Mf\_liverA, CL2702.Contig2\_Mf\_liverA |
| filopodium | Unigene39011\_Mf\_liverA, Unigene25962\_Mf\_liverA, Unigene21684\_Mf\_liverA, CL634.Contig1\_Mf\_liverA, Unigene13950\_Mf\_liverA |
| vacuole | CL4583.Contig2\_Mf\_liverA, CL1736.Contig2\_Mf\_liverA, Unigene38280\_Mf\_liverA, CL3835.Contig2\_Mf\_liverA, CL3002.Contig1\_Mf\_liverA, CL3055.Contig1\_Mf\_liverA, CL4925.Contig1\_Mf\_liverA, Unigene32332\_Mf\_liverA, Unigene5382\_Mf\_liverA, Unigene13414\_Mf\_liverA, Unigene36662\_Mf\_liverA, Unigene8132\_Mf\_liverA, CL838.Contig3\_Mf\_liverA, Unigene37232\_Mf\_liverA, Unigene36112\_Mf\_liverA, Unigene39886\_Mf\_liverA, CL838.Contig6\_Mf\_liverA, Unigene30528\_Mf\_liverA |
| proteinaceous extracellular matrix | Unigene32908\_Mf\_liverA, Unigene34375\_Mf\_liverA, Unigene36112\_Mf\_liverA, Unigene24887\_Mf\_liverA, Unigene35090\_Mf\_liverA, Unigene5382\_Mf\_liverA, Unigene20512\_Mf\_liverA, Unigene8132\_Mf\_liverA, Unigene8054\_Mf\_liverA, Unigene23328\_Mf\_liverA, NM\_007585, Unigene31492\_Mf\_liverA, NM\_010233 |
| very-low-density lipoprotein particle | CL3911.Contig2\_Mf\_liverA, Unigene34477\_Mf\_liverA, Unigene13498\_Mf\_liverA |
| glycogen granule | Unigene36190\_Mf\_liverA, Unigene36189\_Mf\_liverA |
| intrinsic to membrane | Unigene21684\_Mf\_liverA, CL3835.Contig2\_Mf\_liverA, Unigene38831\_Mf\_liverA, NM\_178050, Unigene17310\_Mf\_liverA, Unigene36735\_Mf\_liverA, NM\_011082, CL2339.Contig1\_Mf\_liverA, Unigene33627\_Mf\_liverA, Unigene36762\_Mf\_liverA, Unigene35507\_Mf\_liverA, Unigene31065\_Mf\_liverA, Unigene37698\_Mf\_liverA, Unigene39886\_Mf\_liverA, Unigene5428\_Mf\_liverA, Unigene4732\_Mf\_liverA, Unigene18500\_Mf\_liverA, CL1555.Contig1\_Mf\_liverA, CL5586.Contig1\_Mf\_liverA, Unigene37268\_Mf\_liverA, Unigene47548\_Mf\_liverA, CL1256.Contig1\_Mf\_liverA, Unigene25862\_Mf\_liverA, Unigene25426\_Mf\_liverA, Unigene36662\_Mf\_liverA, CL2260.Contig1\_Mf\_liverA, Unigene4725\_Mf\_liverA, Unigene30369\_Mf\_liverA, NM\_172926, CL3575.Contig1\_Mf\_liverA, Unigene5294\_Mf\_liverA, Unigene8054\_Mf\_liverA, Unigene35171\_Mf\_liverA, CL2971.Contig1\_Mf\_liverA, Unigene17048\_Mf\_liverA, Unigene16261\_Mf\_liverA, Unigene5755\_Mf\_liverA, Unigene13097\_Mf\_liverA, CL3330.Contig1\_Mf\_liverA, Unigene24209\_Mf\_liverA, Unigene1479\_Mf\_liverA, CL838.Contig3\_Mf\_liverA, Unigene17373\_Mf\_liverA, CL4550.Contig3\_Mf\_liverA, Unigene12786\_Mf\_liverA, Unigene36112\_Mf\_liverA, Unigene941\_Mf\_liverA, Unigene15583\_Mf\_liverA, Unigene7264\_Mf\_liverA, Unigene37420\_Mf\_liverA, Unigene19735\_Mf\_liverA, CL4095.Contig4\_Mf\_liverA, Unigene27249\_Mf\_liverA, CL4283.Contig1\_Mf\_liverA, CL4220.Contig2\_Mf\_liverA, Unigene27547\_Mf\_liverA, Unigene4731\_Mf\_liverA, CL5316.Contig1\_Mf\_liverA, Unigene14070\_Mf\_liverA, Unigene32332\_Mf\_liverA, Unigene860\_Mf\_liverA, Unigene14588\_Mf\_liverA, Unigene34630\_Mf\_liverA, Unigene36477\_Mf\_liverA, Unigene27248\_Mf\_liverA, Unigene5355\_Mf\_liverA, Unigene35506\_Mf\_liverA, NM\_019717, Unigene5712\_Mf\_liverA, Unigene14541\_Mf\_liverA, Unigene37485\_Mf\_liverA, Unigene32943\_Mf\_liverA, CL275.Contig5\_Mf\_liverA, Unigene5169\_Mf\_liverA, Unigene29882\_Mf\_liverA, Unigene13233\_Mf\_liverA, Unigene25962\_Mf\_liverA, NM\_023868, NM\_009128, Unigene38913\_Mf\_liverA, Unigene42606\_Mf\_liverA, Unigene13414\_Mf\_liverA, Unigene49971\_Mf\_liverA, CL425.Contig1\_Mf\_liverA, Unigene38208\_Mf\_liverA, Unigene37526\_Mf\_liverA, Unigene112\_Mf\_liverA, Unigene30039\_Mf\_liverA, CL4583.Contig2\_Mf\_liverA, Unigene4938\_Mf\_liverA, Unigene5292\_Mf\_liverA, Unigene34477\_Mf\_liverA, NM\_010141, CL3002.Contig1\_Mf\_liverA, Unigene36315\_Mf\_liverA, CL3055.Contig1\_Mf\_liverA, CL2327.Contig1\_Mf\_liverA, Unigene23503\_Mf\_liverA, CL4033.Contig1\_Mf\_liverA, CL887.Contig2\_Mf\_liverA, Unigene29593\_Mf\_liverA, Unigene21026\_Mf\_liverA, Unigene36937\_Mf\_liverA, Unigene20371\_Mf\_liverA, Unigene4597\_Mf\_liverA, CL838.Contig6\_Mf\_liverA, Unigene10135\_Mf\_liverA, CL4220.Contig1\_Mf\_liverA, CL5307.Contig1\_Mf\_liverA, NM\_008180, Unigene28873\_Mf\_liverA, NM\_178405, NM\_007822, NM\_172203, Unigene15470\_Mf\_liverA, Unigene13356\_Mf\_liverA, Unigene34628\_Mf\_liverA, Unigene35090\_Mf\_liverA, Unigene33517\_Mf\_liverA, CL5062.Contig2\_Mf\_liverA, Unigene39011\_Mf\_liverA, NM\_145218, Unigene35609\_Mf\_liverA, CL4007.Contig1\_Mf\_liverA, Unigene13950\_Mf\_liverA, Unigene35046\_Mf\_liverA, Unigene37245\_Mf\_liverA, Unigene281\_Mf\_liverA, Unigene28860\_Mf\_liverA, NM\_011978, Unigene20372\_Mf\_liverA, CL634.Contig1\_Mf\_liverA, NM\_130450, Unigene36593\_Mf\_liverA, Unigene26336\_Mf\_liverA, Unigene22500\_Mf\_liverA, Unigene37232\_Mf\_liverA, CL5698.Contig1\_Mf\_liverA, CL2884.Contig2\_Mf\_liverA, NM\_153388 |
| peroxisome | Unigene25101\_Mf\_liverA, Unigene37485\_Mf\_liverA, CL1716.Contig2\_Mf\_liverA, Unigene743\_Mf\_liverA, Unigene29727\_Mf\_liverA, NM\_011978, Unigene36662\_Mf\_liverA, Unigene22500\_Mf\_liverA |
| voltage-gated potassium channel complex | Unigene18500\_Mf\_liverA, Unigene29593\_Mf\_liverA |
| potassium channel complex | Unigene18500\_Mf\_liverA, Unigene29593\_Mf\_liverA |
| replisome | Unigene21163\_Mf\_liverA, Unigene31392\_Mf\_liverA |
| nuclear replisome | Unigene21163\_Mf\_liverA, Unigene31392\_Mf\_liverA |
| nucleotide-excision repair complex | Unigene21163\_Mf\_liverA, NM\_009011 |
| late endosome | Unigene37245\_Mf\_liverA, Unigene36698\_Mf\_liverA, Unigene21026\_Mf\_liverA, Unigene15470\_Mf\_liverA, CL3835.Contig2\_Mf\_liverA, Unigene8132\_Mf\_liverA, CL3002.Contig1\_Mf\_liverA, CL3055.Contig1\_Mf\_liverA |
| vacuolar membrane | Unigene32332\_Mf\_liverA, CL4583.Contig2\_Mf\_liverA, Unigene39886\_Mf\_liverA, CL3835.Contig2\_Mf\_liverA, CL3002.Contig1\_Mf\_liverA, CL3055.Contig1\_Mf\_liverA, CL4925.Contig1\_Mf\_liverA |
| interstitial matrix | Unigene24887\_Mf\_liverA, Unigene31492\_Mf\_liverA |
| MHC class I protein complex | CL275.Contig5\_Mf\_liverA, Unigene4938\_Mf\_liverA |
| microbody part | Unigene36662\_Mf\_liverA, Unigene37485\_Mf\_liverA, CL1716.Contig2\_Mf\_liverA, Unigene29727\_Mf\_liverA, Unigene22500\_Mf\_liverA, NM\_011978 |
| peroxisomal part | Unigene36662\_Mf\_liverA, Unigene37485\_Mf\_liverA, CL1716.Contig2\_Mf\_liverA, Unigene29727\_Mf\_liverA, Unigene22500\_Mf\_liverA, NM\_011978 |
| condensed chromosome outer kinetochore | CL5268.Contig1\_Mf\_liverA, Unigene45530\_Mf\_liverA |
| proteasome complex | Unigene26250\_Mf\_liverA, CL4141.Contig1\_Mf\_liverA, CL3835.Contig2\_Mf\_liverA, CL3002.Contig1\_Mf\_liverA |
| peroxisomal membrane | Unigene36662\_Mf\_liverA, Unigene37485\_Mf\_liverA, Unigene29727\_Mf\_liverA, Unigene22500\_Mf\_liverA |
| microbody membrane | Unigene36662\_Mf\_liverA, Unigene37485\_Mf\_liverA, Unigene29727\_Mf\_liverA, Unigene22500\_Mf\_liverA |
| DNA polymerase complex | Unigene21163\_Mf\_liverA, Unigene31392\_Mf\_liverA |
| triglyceride-rich lipoprotein particle | CL3911.Contig2\_Mf\_liverA, Unigene34477\_Mf\_liverA, Unigene13498\_Mf\_liverA |
| lysosomal lumen | CL4583.Contig2\_Mf\_liverA, Unigene5382\_Mf\_liverA, Unigene36112\_Mf\_liverA |
| cyclin-dependent protein kinase holoenzyme complex | Unigene19885\_Mf\_liverA, Unigene7350\_Mf\_liverA |
| vacuolar lumen | CL4583.Contig2\_Mf\_liverA, Unigene5382\_Mf\_liverA, Unigene36112\_Mf\_liverA |
| microbody | Unigene25101\_Mf\_liverA, Unigene37485\_Mf\_liverA, CL1716.Contig2\_Mf\_liverA, Unigene743\_Mf\_liverA, Unigene29727\_Mf\_liverA, NM\_011978, Unigene36662\_Mf\_liverA, Unigene22500\_Mf\_liverA |
| brush border membrane | Unigene36477\_Mf\_liverA, Unigene37485\_Mf\_liverA, Unigene42606\_Mf\_liverA |
| proteasome core complex | CL3835.Contig2\_Mf\_liverA, CL3002.Contig1\_Mf\_liverA |
| chylomicron | Unigene34477\_Mf\_liverA, Unigene13498\_Mf\_liverA |
| nuclear replication fork | Unigene21163\_Mf\_liverA, Unigene31392\_Mf\_liverA |
| condensed chromosome kinetochore | Unigene4684\_Mf\_liverA, CL5268.Contig1\_Mf\_liverA, Unigene45530\_Mf\_liverA, NM\_023294 |
| nuclear envelope | CL1263.Contig1\_Mf\_liverA, CL2971.Contig1\_Mf\_liverA, CL2478.Contig3\_Mf\_liverA, CL4162.Contig1\_Mf\_liverA, Unigene28142\_Mf\_liverA, Unigene29727\_Mf\_liverA, Unigene14212\_Mf\_liverA, Unigene32943\_Mf\_liverA, Unigene13414\_Mf\_liverA, Unigene8132\_Mf\_liverA, Unigene27592\_Mf\_liverA, Unigene172\_Mf\_liverA |
| cytoplasmic membrane-bounded vesicle lumen | Unigene14809\_Mf\_liverA, Unigene12959\_Mf\_liverA, Unigene5355\_Mf\_liverA |
| condensed chromosome, centromeric region | Unigene4684\_Mf\_liverA, CL5268.Contig1\_Mf\_liverA, Unigene45530\_Mf\_liverA, NM\_023294 |
| organelle envelope | Unigene35476\_Mf\_liverA, CL2971.Contig1\_Mf\_liverA, CL2478.Contig3\_Mf\_liverA, Unigene17048\_Mf\_liverA, Unigene28142\_Mf\_liverA, Unigene10136\_Mf\_liverA, Unigene29727\_Mf\_liverA, CL5316.Contig1\_Mf\_liverA, CL3599.Contig1\_Mf\_liverA, Unigene13414\_Mf\_liverA, Unigene36735\_Mf\_liverA, Unigene36662\_Mf\_liverA, Unigene8132\_Mf\_liverA, Unigene172\_Mf\_liverA, CL2702.Contig2\_Mf\_liverA, Unigene3439\_Mf\_liverA, CL1263.Contig1\_Mf\_liverA, Unigene38208\_Mf\_liverA, CL4162.Contig1\_Mf\_liverA, CL2517.Contig1\_Mf\_liverA, Unigene21359\_Mf\_liverA, Unigene29461\_Mf\_liverA, Unigene31065\_Mf\_liverA, Unigene14212\_Mf\_liverA, Unigene32943\_Mf\_liverA, Unigene28548\_Mf\_liverA, NM\_025336, Unigene13233\_Mf\_liverA, Unigene27592\_Mf\_liverA |
| mitochondrial intermembrane space | Unigene35476\_Mf\_liverA, Unigene28548\_Mf\_liverA, Unigene36662\_Mf\_liverA |
| nuclear outer membrane | CL2478.Contig3\_Mf\_liverA, Unigene172\_Mf\_liverA |
| sarcolemma | Unigene27547\_Mf\_liverA, CL425.Contig1\_Mf\_liverA, Unigene29593\_Mf\_liverA, CL887.Contig2\_Mf\_liverA, CL634.Contig1\_Mf\_liverA |
| envelope | Unigene35476\_Mf\_liverA, CL2971.Contig1\_Mf\_liverA, CL2478.Contig3\_Mf\_liverA, Unigene17048\_Mf\_liverA, Unigene28142\_Mf\_liverA, Unigene10136\_Mf\_liverA, Unigene29727\_Mf\_liverA, CL5316.Contig1\_Mf\_liverA, CL3599.Contig1\_Mf\_liverA, Unigene13414\_Mf\_liverA, Unigene36735\_Mf\_liverA, Unigene36662\_Mf\_liverA, Unigene8132\_Mf\_liverA, Unigene172\_Mf\_liverA, CL2702.Contig2\_Mf\_liverA, Unigene3439\_Mf\_liverA, CL1263.Contig1\_Mf\_liverA, Unigene38208\_Mf\_liverA, CL4162.Contig1\_Mf\_liverA, CL2517.Contig1\_Mf\_liverA, Unigene21359\_Mf\_liverA, Unigene29461\_Mf\_liverA, Unigene31065\_Mf\_liverA, Unigene14212\_Mf\_liverA, Unigene32943\_Mf\_liverA, Unigene28548\_Mf\_liverA, NM\_025336, Unigene13233\_Mf\_liverA, Unigene27592\_Mf\_liverA |
| histone acetyltransferase complex | NM\_009609, NM\_177093, Unigene21163\_Mf\_liverA, Unigene1479\_Mf\_liverA, Unigene30288\_Mf\_liverA |
| mitochondrial part | Unigene35476\_Mf\_liverA, Unigene17048\_Mf\_liverA, NM\_020559, NM\_010481, Unigene10136\_Mf\_liverA, CL5316.Contig1\_Mf\_liverA, CL3599.Contig1\_Mf\_liverA, Unigene36735\_Mf\_liverA, Unigene36662\_Mf\_liverA, CL2702.Contig2\_Mf\_liverA, Unigene35816\_Mf\_liverA, Unigene3439\_Mf\_liverA, Unigene38208\_Mf\_liverA, Unigene14916\_Mf\_liverA, Unigene37028\_Mf\_liverA, CL2517.Contig1\_Mf\_liverA, Unigene21359\_Mf\_liverA, Unigene29461\_Mf\_liverA, Unigene31065\_Mf\_liverA, Unigene15252\_Mf\_liverA, CL2791.Contig1\_Mf\_liverA, Unigene30731\_Mf\_liverA, Unigene28548\_Mf\_liverA, Unigene13233\_Mf\_liverA |
| vesicle lumen | Unigene14809\_Mf\_liverA, Unigene12959\_Mf\_liverA, Unigene5355\_Mf\_liverA |
| lipid particle | Unigene25596\_Mf\_liverA, Unigene29461\_Mf\_liverA, Unigene45904\_Mf\_liverA |
| organelle envelope lumen | Unigene35476\_Mf\_liverA, Unigene28548\_Mf\_liverA, Unigene36662\_Mf\_liverA |
| myelin sheath | Unigene5712\_Mf\_liverA, Unigene37268\_Mf\_liverA, CL2260.Contig1\_Mf\_liverA |
| H4/H2A histone acetyltransferase complex | NM\_009609, NM\_177093 |
| nuclear membrane | Unigene32943\_Mf\_liverA, CL2971.Contig1\_Mf\_liverA, CL2478.Contig3\_Mf\_liverA, Unigene8132\_Mf\_liverA, Unigene29727\_Mf\_liverA, Unigene172\_Mf\_liverA |
| late endosome membrane | CL3835.Contig2\_Mf\_liverA, CL3002.Contig1\_Mf\_liverA, CL3055.Contig1\_Mf\_liverA |
| cell part | Unigene21684\_Mf\_liverA, NM\_178050, NM\_021508, Unigene15553\_Mf\_liverA, Unigene172\_Mf\_liverA, NM\_011082, CL4577.Contig1\_Mf\_liverA, Unigene35507\_Mf\_liverA, Unigene6465\_Mf\_liverA, CL2982.Contig1\_Mf\_liverA, NM\_025613, Unigene39886\_Mf\_liverA, Unigene27339\_Mf\_liverA, Unigene4732\_Mf\_liverA, CL2790.Contig1\_Mf\_liverA, CL1555.Contig1\_Mf\_liverA, CL5586.Contig1\_Mf\_liverA, Unigene47548\_Mf\_liverA, CL5851.Contig1\_Mf\_liverA, Unigene34341\_Mf\_liverA, Unigene30153\_Mf\_liverA, Unigene36662\_Mf\_liverA, NM\_011921, Unigene35816\_Mf\_liverA, CL2260.Contig1\_Mf\_liverA, NM\_010233, NM\_145474, Unigene13940\_Mf\_liverA, Unigene4725\_Mf\_liverA, NM\_009776, Unigene30003\_Mf\_liverA, NM\_172409, Unigene25594\_Mf\_liverA, Unigene29461\_Mf\_liverA, Unigene5328\_Mf\_liverA, CL4995.Contig1\_Mf\_liverA, Unigene30002\_Mf\_liverA, Unigene8054\_Mf\_liverA, Unigene14589\_Mf\_liverA, CL4816.Contig1\_Mf\_liverA, CL2971.Contig1\_Mf\_liverA, Unigene5639\_Mf\_liverA, Unigene17048\_Mf\_liverA, Unigene4684\_Mf\_liverA, Unigene24092\_Mf\_liverA, Unigene743\_Mf\_liverA, Unigene36175\_Mf\_liverA, Unigene13097\_Mf\_liverA, NM\_177093, Unigene33512\_Mf\_liverA, Unigene34591\_Mf\_liverA, Unigene24209\_Mf\_liverA, Unigene1479\_Mf\_liverA, CL4332.Contig1\_Mf\_liverA, NM\_010001, NM\_080639, Unigene36698\_Mf\_liverA, Unigene27895\_Mf\_liverA, Unigene36112\_Mf\_liverA, NM\_001104531, Unigene139\_Mf\_liverA, Unigene33560\_Mf\_liverA, Unigene7264\_Mf\_liverA, Unigene23158\_Mf\_liverA, Unigene27249\_Mf\_liverA, Unigene27547\_Mf\_liverA, Unigene31364\_Mf\_liverA, CL1736.Contig2\_Mf\_liverA, Unigene4731\_Mf\_liverA, Unigene33366\_Mf\_liverA, Unigene32908\_Mf\_liverA, Unigene12909\_Mf\_liverA, Unigene33898\_Mf\_liverA, CL5316.Contig1\_Mf\_liverA, Unigene45530\_Mf\_liverA, Unigene32332\_Mf\_liverA, Unigene4723\_Mf\_liverA, Unigene14588\_Mf\_liverA, Unigene34630\_Mf\_liverA, NR\_003630, Unigene36477\_Mf\_liverA, Unigene27248\_Mf\_liverA, Unigene36508\_Mf\_liverA, Unigene30839\_Mf\_liverA, Unigene37485\_Mf\_liverA, Unigene30288\_Mf\_liverA, Unigene32943\_Mf\_liverA, CL275.Contig5\_Mf\_liverA, NR\_003552, Unigene5169\_Mf\_liverA, Unigene30528\_Mf\_liverA, Unigene29882\_Mf\_liverA, Unigene32059\_Mf\_liverA, Unigene45904\_Mf\_liverA, Unigene32202\_Mf\_liverA, Unigene25962\_Mf\_liverA, NM\_023868, Unigene29727\_Mf\_liverA, Unigene38913\_Mf\_liverA, Unigene42606\_Mf\_liverA, CL4925.Contig1\_Mf\_liverA, CL186.Contig3\_Mf\_liverA, Unigene12959\_Mf\_liverA, CL425.Contig1\_Mf\_liverA, CL1803.Contig1\_Mf\_liverA, CL4736.Contig1\_Mf\_liverA, Unigene14916\_Mf\_liverA, Unigene37243\_Mf\_liverA, Unigene15077\_Mf\_liverA, Unigene30039\_Mf\_liverA, Unigene29134\_Mf\_liverA, CL3800.Contig1\_Mf\_liverA, CL2478.Contig3\_Mf\_liverA, Unigene4938\_Mf\_liverA, Unigene24974\_Mf\_liverA, Unigene34375\_Mf\_liverA, CL3055.Contig1\_Mf\_liverA, Unigene23503\_Mf\_liverA, NM\_028785, NM\_009609, CL4033.Contig1\_Mf\_liverA, NM\_007409, Unigene34983\_Mf\_liverA, CL2439.Contig1\_Mf\_liverA, Unigene3439\_Mf\_liverA, Unigene35935\_Mf\_liverA, Unigene33980\_Mf\_liverA, Unigene37238\_Mf\_liverA, Unigene21026\_Mf\_liverA, Unigene20371\_Mf\_liverA, Unigene36937\_Mf\_liverA, Unigene13683\_Mf\_liverA, CL1907.Contig1\_Mf\_liverA, Unigene30004\_Mf\_liverA, CL480.Contig1\_Mf\_liverA, Unigene35476\_Mf\_liverA, CL5828.Contig2\_Mf\_liverA, Unigene24858\_Mf\_liverA, CL4220.Contig1\_Mf\_liverA, Unigene37262\_Mf\_liverA, Unigene12908\_Mf\_liverA, NR\_033215, CL2702.Contig2\_Mf\_liverA, CL1165.Contig2\_Mf\_liverA, Unigene24466\_Mf\_liverA, CL2098.Contig1\_Mf\_liverA, NM\_007822, CL5293.Contig1\_Mf\_liverA, Unigene25595\_Mf\_liverA, CL3312.Contig1\_Mf\_liverA, Unigene15470\_Mf\_liverA, Unigene14286\_Mf\_liverA, Unigene34628\_Mf\_liverA, Unigene13356\_Mf\_liverA, Unigene35090\_Mf\_liverA, Unigene14212\_Mf\_liverA, Unigene34962\_Mf\_liverA, Unigene39011\_Mf\_liverA, Unigene33517\_Mf\_liverA, Unigene26250\_Mf\_liverA, CL1588.Contig2\_Mf\_liverA, NM\_025336, Unigene35609\_Mf\_liverA, NM\_145218, Unigene36189\_Mf\_liverA, Unigene33580\_Mf\_liverA, Unigene13950\_Mf\_liverA, Unigene35046\_Mf\_liverA, Unigene28459\_Mf\_liverA, NM\_013863, Unigene37245\_Mf\_liverA, Unigene281\_Mf\_liverA, NM\_011978, Unigene20372\_Mf\_liverA, CL634.Contig1\_Mf\_liverA, Unigene29985\_Mf\_liverA, NM\_130450, Unigene36593\_Mf\_liverA, Unigene26336\_Mf\_liverA, NM\_027406, Unigene36176\_Mf\_liverA, Unigene37232\_Mf\_liverA, Unigene37239\_Mf\_liverA, Unigene37028\_Mf\_liverA, CL5698.Contig1\_Mf\_liverA, Unigene31392\_Mf\_liverA, NM\_020559, Unigene28142\_Mf\_liverA, Unigene38015\_Mf\_liverA, CL3835.Contig2\_Mf\_liverA, Unigene38831\_Mf\_liverA, Unigene36404\_Mf\_liverA, Unigene36735\_Mf\_liverA, NM\_019651, CL523.Contig1\_Mf\_liverA, CL2339.Contig1\_Mf\_liverA, Unigene36762\_Mf\_liverA, Unigene31065\_Mf\_liverA, Unigene38035\_Mf\_liverA, NM\_011072, NM\_007820, Unigene37698\_Mf\_liverA, Unigene20853\_Mf\_liverA, Unigene5428\_Mf\_liverA, Unigene27592\_Mf\_liverA, Unigene18500\_Mf\_liverA, Unigene24351\_Mf\_liverA, Unigene37268\_Mf\_liverA, Unigene37999\_Mf\_liverA, CL1256.Contig1\_Mf\_liverA, Unigene25862\_Mf\_liverA, CL3483.Contig1\_Mf\_liverA, Unigene12999\_Mf\_liverA, CL4141.Contig1\_Mf\_liverA, Unigene13658\_Mf\_liverA, Unigene30369\_Mf\_liverA, Unigene32316\_Mf\_liverA, CL2622.Contig2\_Mf\_liverA, Unigene25596\_Mf\_liverA, CL3575.Contig1\_Mf\_liverA, Unigene28548\_Mf\_liverA, CL5268.Contig1\_Mf\_liverA, Unigene5294\_Mf\_liverA, Unigene23328\_Mf\_liverA, Unigene38065\_Mf\_liverA, Unigene35171\_Mf\_liverA, Unigene36626\_Mf\_liverA, Unigene39507\_Mf\_liverA, NM\_029562, Unigene5755\_Mf\_liverA, CL3750.Contig2\_Mf\_liverA, CL3599.Contig1\_Mf\_liverA, Unigene151\_Mf\_liverA, Unigene39141\_Mf\_liverA, Unigene5382\_Mf\_liverA, CL1595.Contig1\_Mf\_liverA, CL838.Contig3\_Mf\_liverA, NM\_029692, CL1263.Contig1\_Mf\_liverA, CL4550.Contig3\_Mf\_liverA, CL258.Contig1\_Mf\_liverA, Unigene36628\_Mf\_liverA, Unigene31231\_Mf\_liverA, Unigene12786\_Mf\_liverA, Unigene31517\_Mf\_liverA, NM\_007811, NM\_008538, CL1716.Contig2\_Mf\_liverA, NM\_011305, Unigene15583\_Mf\_liverA, Unigene4523\_Mf\_liverA, Unigene13498\_Mf\_liverA, CL4095.Contig4\_Mf\_liverA, CL4283.Contig1\_Mf\_liverA, Unigene4510\_Mf\_liverA, Unigene17579\_Mf\_liverA, CL4220.Contig2\_Mf\_liverA, Unigene19885\_Mf\_liverA, CL2550.Contig1\_Mf\_liverA, Unigene7195\_Mf\_liverA, Unigene5355\_Mf\_liverA, Unigene8132\_Mf\_liverA, Unigene9664\_Mf\_liverA, Unigene35506\_Mf\_liverA, NM\_019717, Unigene5712\_Mf\_liverA, Unigene1205\_Mf\_liverA, Unigene30321\_Mf\_liverA, Unigene28587\_Mf\_liverA, CL1509.Contig2\_Mf\_liverA, Unigene13233\_Mf\_liverA, NM\_010481, Unigene10136\_Mf\_liverA, NM\_009128, NM\_011732, CL2240.Contig1\_Mf\_liverA, Unigene13414\_Mf\_liverA, Unigene49971\_Mf\_liverA, Unigene36190\_Mf\_liverA, Unigene5210\_Mf\_liverA, Unigene38208\_Mf\_liverA, CL4162.Contig1\_Mf\_liverA, Unigene37526\_Mf\_liverA, Unigene112\_Mf\_liverA, CL2517.Contig1\_Mf\_liverA, Unigene24931\_Mf\_liverA, Unigene29510\_Mf\_liverA, CL5986.Contig1\_Mf\_liverA, Unigene15681\_Mf\_liverA, Unigene7350\_Mf\_liverA, Unigene25751\_Mf\_liverA, CL3569.Contig2\_Mf\_liverA, CL4583.Contig2\_Mf\_liverA, Unigene13894\_Mf\_liverA, Unigene34477\_Mf\_liverA, NM\_010141, Unigene38280\_Mf\_liverA, CL3002.Contig1\_Mf\_liverA, Unigene36315\_Mf\_liverA, Unigene36784\_Mf\_liverA, Unigene31483\_Mf\_liverA, Unigene40262\_Mf\_liverA, CL2327.Contig1\_Mf\_liverA, Unigene36514\_Mf\_liverA, CL4816.Contig2\_Mf\_liverA, CL887.Contig2\_Mf\_liverA, Unigene29593\_Mf\_liverA, NM\_023294, Unigene33523\_Mf\_liverA, Unigene35037\_Mf\_liverA, Unigene14810\_Mf\_liverA, CL3684.Contig2\_Mf\_liverA, Unigene4597\_Mf\_liverA, Unigene15252\_Mf\_liverA, NM\_001100181, CL838.Contig6\_Mf\_liverA, CL2791.Contig1\_Mf\_liverA, CL4770.Contig1\_Mf\_liverA, CL3549.Contig1\_Mf\_liverA, Unigene10135\_Mf\_liverA, CL3750.Contig1\_Mf\_liverA, CL1588.Contig3\_Mf\_liverA, Unigene25101\_Mf\_liverA, NM\_009883, Unigene26138\_Mf\_liverA, CL3112.Contig7\_Mf\_liverA, CL1518.Contig1\_Mf\_liverA, CL5307.Contig1\_Mf\_liverA, NM\_008180, Unigene28873\_Mf\_liverA, NM\_007585, NM\_178405, Unigene33525\_Mf\_liverA, Unigene21163\_Mf\_liverA, Unigene36177\_Mf\_liverA, NM\_172203, Unigene37100\_Mf\_liverA, Unigene5335\_Mf\_liverA, Unigene30322\_Mf\_liverA, Unigene15402\_Mf\_liverA, Unigene30731\_Mf\_liverA, CL912.Contig2\_Mf\_liverA, CL4007.Contig1\_Mf\_liverA, CL1588.Contig1\_Mf\_liverA, NM\_017379, CL2355.Contig1\_Mf\_liverA, Unigene28314\_Mf\_liverA, Unigene14809\_Mf\_liverA, Unigene39686\_Mf\_liverA, Unigene4636\_Mf\_liverA, Unigene22500\_Mf\_liverA, NM\_009011, Unigene30892\_Mf\_liverA, CL3911.Contig2\_Mf\_liverA, Unigene21359\_Mf\_liverA, Unigene12519\_Mf\_liverA, CL2884.Contig2\_Mf\_liverA, CL336.Contig3\_Mf\_liverA, Unigene28989\_Mf\_liverA, NM\_146120, NM\_007819, Unigene19954\_Mf\_liverA |
| cell | Unigene21684\_Mf\_liverA, NM\_178050, NM\_021508, Unigene15553\_Mf\_liverA, Unigene172\_Mf\_liverA, NM\_011082, CL4577.Contig1\_Mf\_liverA, Unigene35507\_Mf\_liverA, Unigene6465\_Mf\_liverA, CL2982.Contig1\_Mf\_liverA, NM\_025613, Unigene39886\_Mf\_liverA, Unigene27339\_Mf\_liverA, Unigene4732\_Mf\_liverA, CL2790.Contig1\_Mf\_liverA, CL1555.Contig1\_Mf\_liverA, CL5586.Contig1\_Mf\_liverA, Unigene47548\_Mf\_liverA, CL5851.Contig1\_Mf\_liverA, Unigene34341\_Mf\_liverA, Unigene30153\_Mf\_liverA, Unigene36662\_Mf\_liverA, NM\_011921, Unigene35816\_Mf\_liverA, CL2260.Contig1\_Mf\_liverA, NM\_010233, NM\_145474, Unigene13940\_Mf\_liverA, Unigene4725\_Mf\_liverA, NM\_009776, Unigene30003\_Mf\_liverA, NM\_172409, Unigene25594\_Mf\_liverA, Unigene29461\_Mf\_liverA, Unigene5328\_Mf\_liverA, CL4995.Contig1\_Mf\_liverA, Unigene30002\_Mf\_liverA, Unigene8054\_Mf\_liverA, Unigene14589\_Mf\_liverA, CL4816.Contig1\_Mf\_liverA, CL2971.Contig1\_Mf\_liverA, Unigene5639\_Mf\_liverA, Unigene17048\_Mf\_liverA, Unigene4684\_Mf\_liverA, Unigene24092\_Mf\_liverA, Unigene743\_Mf\_liverA, Unigene36175\_Mf\_liverA, Unigene13097\_Mf\_liverA, NM\_177093, Unigene33512\_Mf\_liverA, Unigene34591\_Mf\_liverA, Unigene24209\_Mf\_liverA, Unigene1479\_Mf\_liverA, CL4332.Contig1\_Mf\_liverA, NM\_010001, NM\_080639, Unigene36698\_Mf\_liverA, Unigene27895\_Mf\_liverA, Unigene36112\_Mf\_liverA, NM\_001104531, Unigene139\_Mf\_liverA, Unigene33560\_Mf\_liverA, Unigene7264\_Mf\_liverA, Unigene23158\_Mf\_liverA, Unigene27249\_Mf\_liverA, Unigene27547\_Mf\_liverA, Unigene31364\_Mf\_liverA, CL1736.Contig2\_Mf\_liverA, Unigene4731\_Mf\_liverA, Unigene33366\_Mf\_liverA, Unigene32908\_Mf\_liverA, Unigene12909\_Mf\_liverA, Unigene33898\_Mf\_liverA, CL5316.Contig1\_Mf\_liverA, Unigene45530\_Mf\_liverA, Unigene32332\_Mf\_liverA, Unigene4723\_Mf\_liverA, Unigene14588\_Mf\_liverA, Unigene34630\_Mf\_liverA, NR\_003630, Unigene36477\_Mf\_liverA, Unigene27248\_Mf\_liverA, Unigene36508\_Mf\_liverA, Unigene30839\_Mf\_liverA, Unigene37485\_Mf\_liverA, Unigene30288\_Mf\_liverA, Unigene32943\_Mf\_liverA, CL275.Contig5\_Mf\_liverA, NR\_003552, Unigene5169\_Mf\_liverA, Unigene30528\_Mf\_liverA, Unigene29882\_Mf\_liverA, Unigene32059\_Mf\_liverA, Unigene45904\_Mf\_liverA, Unigene32202\_Mf\_liverA, Unigene25962\_Mf\_liverA, NM\_023868, Unigene29727\_Mf\_liverA, Unigene38913\_Mf\_liverA, Unigene42606\_Mf\_liverA, CL4925.Contig1\_Mf\_liverA, CL186.Contig3\_Mf\_liverA, Unigene12959\_Mf\_liverA, CL425.Contig1\_Mf\_liverA, CL1803.Contig1\_Mf\_liverA, CL4736.Contig1\_Mf\_liverA, Unigene14916\_Mf\_liverA, Unigene37243\_Mf\_liverA, Unigene15077\_Mf\_liverA, Unigene30039\_Mf\_liverA, Unigene29134\_Mf\_liverA, CL3800.Contig1\_Mf\_liverA, CL2478.Contig3\_Mf\_liverA, Unigene4938\_Mf\_liverA, Unigene24974\_Mf\_liverA, Unigene34375\_Mf\_liverA, CL3055.Contig1\_Mf\_liverA, Unigene23503\_Mf\_liverA, NM\_028785, NM\_009609, CL4033.Contig1\_Mf\_liverA, NM\_007409, Unigene34983\_Mf\_liverA, CL2439.Contig1\_Mf\_liverA, Unigene3439\_Mf\_liverA, Unigene35935\_Mf\_liverA, Unigene33980\_Mf\_liverA, Unigene37238\_Mf\_liverA, Unigene21026\_Mf\_liverA, Unigene20371\_Mf\_liverA, Unigene36937\_Mf\_liverA, Unigene13683\_Mf\_liverA, CL1907.Contig1\_Mf\_liverA, Unigene30004\_Mf\_liverA, CL480.Contig1\_Mf\_liverA, Unigene35476\_Mf\_liverA, CL5828.Contig2\_Mf\_liverA, Unigene24858\_Mf\_liverA, CL4220.Contig1\_Mf\_liverA, Unigene37262\_Mf\_liverA, Unigene12908\_Mf\_liverA, NR\_033215, CL2702.Contig2\_Mf\_liverA, CL1165.Contig2\_Mf\_liverA, Unigene24466\_Mf\_liverA, CL2098.Contig1\_Mf\_liverA, NM\_007822, CL5293.Contig1\_Mf\_liverA, Unigene25595\_Mf\_liverA, CL3312.Contig1\_Mf\_liverA, Unigene15470\_Mf\_liverA, Unigene14286\_Mf\_liverA, Unigene34628\_Mf\_liverA, Unigene13356\_Mf\_liverA, Unigene35090\_Mf\_liverA, Unigene14212\_Mf\_liverA, Unigene34962\_Mf\_liverA, Unigene39011\_Mf\_liverA, Unigene33517\_Mf\_liverA, Unigene26250\_Mf\_liverA, CL1588.Contig2\_Mf\_liverA, NM\_025336, Unigene35609\_Mf\_liverA, NM\_145218, Unigene36189\_Mf\_liverA, Unigene33580\_Mf\_liverA, Unigene13950\_Mf\_liverA, Unigene35046\_Mf\_liverA, Unigene28459\_Mf\_liverA, NM\_013863, Unigene37245\_Mf\_liverA, Unigene281\_Mf\_liverA, NM\_011978, Unigene20372\_Mf\_liverA, CL634.Contig1\_Mf\_liverA, Unigene29985\_Mf\_liverA, NM\_130450, Unigene36593\_Mf\_liverA, Unigene26336\_Mf\_liverA, NM\_027406, Unigene36176\_Mf\_liverA, Unigene37232\_Mf\_liverA, Unigene37239\_Mf\_liverA, Unigene37028\_Mf\_liverA, CL5698.Contig1\_Mf\_liverA, Unigene31392\_Mf\_liverA, NM\_020559, Unigene28142\_Mf\_liverA, Unigene38015\_Mf\_liverA, CL3835.Contig2\_Mf\_liverA, Unigene38831\_Mf\_liverA, Unigene36404\_Mf\_liverA, Unigene36735\_Mf\_liverA, NM\_019651, CL523.Contig1\_Mf\_liverA, CL2339.Contig1\_Mf\_liverA, Unigene36762\_Mf\_liverA, Unigene31065\_Mf\_liverA, Unigene38035\_Mf\_liverA, NM\_011072, NM\_007820, Unigene37698\_Mf\_liverA, Unigene20853\_Mf\_liverA, Unigene5428\_Mf\_liverA, Unigene27592\_Mf\_liverA, Unigene18500\_Mf\_liverA, Unigene24351\_Mf\_liverA, Unigene37268\_Mf\_liverA, Unigene37999\_Mf\_liverA, CL1256.Contig1\_Mf\_liverA, Unigene25862\_Mf\_liverA, CL3483.Contig1\_Mf\_liverA, Unigene12999\_Mf\_liverA, CL4141.Contig1\_Mf\_liverA, Unigene13658\_Mf\_liverA, Unigene30369\_Mf\_liverA, Unigene32316\_Mf\_liverA, CL2622.Contig2\_Mf\_liverA, Unigene25596\_Mf\_liverA, CL3575.Contig1\_Mf\_liverA, Unigene28548\_Mf\_liverA, CL5268.Contig1\_Mf\_liverA, Unigene5294\_Mf\_liverA, Unigene23328\_Mf\_liverA, Unigene38065\_Mf\_liverA, Unigene35171\_Mf\_liverA, Unigene36626\_Mf\_liverA, Unigene39507\_Mf\_liverA, NM\_029562, Unigene5755\_Mf\_liverA, CL3750.Contig2\_Mf\_liverA, CL3599.Contig1\_Mf\_liverA, Unigene151\_Mf\_liverA, Unigene39141\_Mf\_liverA, Unigene5382\_Mf\_liverA, CL1595.Contig1\_Mf\_liverA, CL838.Contig3\_Mf\_liverA, NM\_029692, CL1263.Contig1\_Mf\_liverA, CL4550.Contig3\_Mf\_liverA, CL258.Contig1\_Mf\_liverA, Unigene36628\_Mf\_liverA, Unigene31231\_Mf\_liverA, Unigene12786\_Mf\_liverA, Unigene31517\_Mf\_liverA, NM\_007811, NM\_008538, CL1716.Contig2\_Mf\_liverA, NM\_011305, Unigene15583\_Mf\_liverA, Unigene4523\_Mf\_liverA, Unigene13498\_Mf\_liverA, CL4095.Contig4\_Mf\_liverA, CL4283.Contig1\_Mf\_liverA, Unigene4510\_Mf\_liverA, Unigene17579\_Mf\_liverA, CL4220.Contig2\_Mf\_liverA, Unigene19885\_Mf\_liverA, CL2550.Contig1\_Mf\_liverA, Unigene7195\_Mf\_liverA, Unigene5355\_Mf\_liverA, Unigene8132\_Mf\_liverA, Unigene9664\_Mf\_liverA, Unigene35506\_Mf\_liverA, NM\_019717, Unigene5712\_Mf\_liverA, Unigene1205\_Mf\_liverA, Unigene30321\_Mf\_liverA, Unigene28587\_Mf\_liverA, CL1509.Contig2\_Mf\_liverA, Unigene13233\_Mf\_liverA, NM\_010481, Unigene10136\_Mf\_liverA, NM\_009128, NM\_011732, CL2240.Contig1\_Mf\_liverA, Unigene13414\_Mf\_liverA, Unigene49971\_Mf\_liverA, Unigene36190\_Mf\_liverA, Unigene5210\_Mf\_liverA, Unigene38208\_Mf\_liverA, CL4162.Contig1\_Mf\_liverA, Unigene37526\_Mf\_liverA, Unigene112\_Mf\_liverA, CL2517.Contig1\_Mf\_liverA, Unigene24931\_Mf\_liverA, Unigene29510\_Mf\_liverA, CL5986.Contig1\_Mf\_liverA, Unigene15681\_Mf\_liverA, Unigene7350\_Mf\_liverA, Unigene25751\_Mf\_liverA, CL3569.Contig2\_Mf\_liverA, CL4583.Contig2\_Mf\_liverA, Unigene13894\_Mf\_liverA, Unigene34477\_Mf\_liverA, NM\_010141, Unigene38280\_Mf\_liverA, CL3002.Contig1\_Mf\_liverA, Unigene36315\_Mf\_liverA, Unigene36784\_Mf\_liverA, Unigene31483\_Mf\_liverA, Unigene40262\_Mf\_liverA, CL2327.Contig1\_Mf\_liverA, Unigene36514\_Mf\_liverA, CL4816.Contig2\_Mf\_liverA, CL887.Contig2\_Mf\_liverA, Unigene29593\_Mf\_liverA, NM\_023294, Unigene33523\_Mf\_liverA, Unigene35037\_Mf\_liverA, Unigene14810\_Mf\_liverA, CL3684.Contig2\_Mf\_liverA, Unigene4597\_Mf\_liverA, Unigene15252\_Mf\_liverA, NM\_001100181, CL838.Contig6\_Mf\_liverA, CL2791.Contig1\_Mf\_liverA, CL4770.Contig1\_Mf\_liverA, CL3549.Contig1\_Mf\_liverA, Unigene10135\_Mf\_liverA, CL3750.Contig1\_Mf\_liverA, CL1588.Contig3\_Mf\_liverA, Unigene25101\_Mf\_liverA, NM\_009883, Unigene26138\_Mf\_liverA, CL3112.Contig7\_Mf\_liverA, CL1518.Contig1\_Mf\_liverA, CL5307.Contig1\_Mf\_liverA, NM\_008180, Unigene28873\_Mf\_liverA, NM\_007585, NM\_178405, Unigene33525\_Mf\_liverA, Unigene21163\_Mf\_liverA, Unigene36177\_Mf\_liverA, NM\_172203, Unigene37100\_Mf\_liverA, Unigene5335\_Mf\_liverA, Unigene30322\_Mf\_liverA, Unigene15402\_Mf\_liverA, Unigene30731\_Mf\_liverA, CL912.Contig2\_Mf\_liverA, CL4007.Contig1\_Mf\_liverA, CL1588.Contig1\_Mf\_liverA, NM\_017379, CL2355.Contig1\_Mf\_liverA, Unigene28314\_Mf\_liverA, Unigene14809\_Mf\_liverA, Unigene39686\_Mf\_liverA, Unigene4636\_Mf\_liverA, Unigene22500\_Mf\_liverA, NM\_009011, Unigene30892\_Mf\_liverA, CL3911.Contig2\_Mf\_liverA, Unigene21359\_Mf\_liverA, Unigene12519\_Mf\_liverA, CL2884.Contig2\_Mf\_liverA, CL336.Contig3\_Mf\_liverA, Unigene28989\_Mf\_liverA, NM\_146120, NM\_007819, Unigene19954\_Mf\_liverA |
| melanosome | Unigene36762\_Mf\_liverA, Unigene36190\_Mf\_liverA, Unigene36189\_Mf\_liverA |
| endosome membrane | Unigene38015\_Mf\_liverA, Unigene15077\_Mf\_liverA, CL3835.Contig2\_Mf\_liverA, CL3002.Contig1\_Mf\_liverA, NM\_011082, CL3055.Contig1\_Mf\_liverA, Unigene4725\_Mf\_liverA |
| contractile fiber | NM\_009609, Unigene26250\_Mf\_liverA, NM\_177093, Unigene5639\_Mf\_liverA, NM\_023868, Unigene29593\_Mf\_liverA, Unigene8132\_Mf\_liverA, NM\_080639 |
| Golgi lumen | Unigene5382\_Mf\_liverA, Unigene36112\_Mf\_liverA |
| endosomal part | Unigene38015\_Mf\_liverA, Unigene15077\_Mf\_liverA, CL3835.Contig2\_Mf\_liverA, CL3002.Contig1\_Mf\_liverA, NM\_011082, CL3055.Contig1\_Mf\_liverA, Unigene4725\_Mf\_liverA |
| neuromuscular junction | Unigene5355\_Mf\_liverA, Unigene13950\_Mf\_liverA |
| collagen | Unigene34375\_Mf\_liverA, Unigene23328\_Mf\_liverA, Unigene35090\_Mf\_liverA |
| cation channel complex | Unigene18500\_Mf\_liverA, NM\_023868, Unigene29593\_Mf\_liverA |
| mitochondrial envelope | Unigene35476\_Mf\_liverA, Unigene17048\_Mf\_liverA, Unigene10136\_Mf\_liverA, CL5316.Contig1\_Mf\_liverA, CL3599.Contig1\_Mf\_liverA, Unigene36735\_Mf\_liverA, Unigene36662\_Mf\_liverA, CL2702.Contig2\_Mf\_liverA, Unigene3439\_Mf\_liverA, Unigene38208\_Mf\_liverA, CL2517.Contig1\_Mf\_liverA, Unigene29461\_Mf\_liverA, Unigene21359\_Mf\_liverA, Unigene31065\_Mf\_liverA, Unigene28548\_Mf\_liverA, Unigene13233\_Mf\_liverA |
| endosome | Unigene37245\_Mf\_liverA, Unigene38015\_Mf\_liverA, CL3835.Contig2\_Mf\_liverA, CL3002.Contig1\_Mf\_liverA, CL3055.Contig1\_Mf\_liverA, Unigene13414\_Mf\_liverA, Unigene49971\_Mf\_liverA, Unigene8132\_Mf\_liverA, NM\_007585, NM\_011082, Unigene4725\_Mf\_liverA, Unigene36698\_Mf\_liverA, NM\_172203, Unigene15077\_Mf\_liverA, Unigene21026\_Mf\_liverA, Unigene15470\_Mf\_liverA |
| extracellular matrix part | Unigene32908\_Mf\_liverA, Unigene34375\_Mf\_liverA, Unigene23328\_Mf\_liverA, Unigene8132\_Mf\_liverA, Unigene35090\_Mf\_liverA, Unigene31492\_Mf\_liverA |
| phagocytic vesicle | Unigene14809\_Mf\_liverA, Unigene8054\_Mf\_liverA |
| organelle part | NM\_020559, Unigene38015\_Mf\_liverA, Unigene28142\_Mf\_liverA, CL3835.Contig2\_Mf\_liverA, Unigene36735\_Mf\_liverA, CL523.Contig1\_Mf\_liverA, Unigene172\_Mf\_liverA, NM\_011082, Unigene35507\_Mf\_liverA, Unigene31065\_Mf\_liverA, NM\_007820, Unigene39886\_Mf\_liverA, Unigene5428\_Mf\_liverA, Unigene27592\_Mf\_liverA, Unigene4732\_Mf\_liverA, CL2790.Contig1\_Mf\_liverA, CL5586.Contig1\_Mf\_liverA, Unigene24351\_Mf\_liverA, Unigene34341\_Mf\_liverA, Unigene12999\_Mf\_liverA, CL4141.Contig1\_Mf\_liverA, Unigene36662\_Mf\_liverA, Unigene35816\_Mf\_liverA, Unigene13658\_Mf\_liverA, NM\_145474, Unigene4725\_Mf\_liverA, Unigene25596\_Mf\_liverA, Unigene29461\_Mf\_liverA, Unigene28548\_Mf\_liverA, CL5268.Contig1\_Mf\_liverA, Unigene5294\_Mf\_liverA, Unigene23328\_Mf\_liverA, Unigene8054\_Mf\_liverA, CL4816.Contig1\_Mf\_liverA, CL2971.Contig1\_Mf\_liverA, Unigene5639\_Mf\_liverA, Unigene17048\_Mf\_liverA, Unigene4684\_Mf\_liverA, NM\_029562, Unigene5755\_Mf\_liverA, Unigene24092\_Mf\_liverA, NM\_177093, CL3599.Contig1\_Mf\_liverA, Unigene5382\_Mf\_liverA, Unigene33512\_Mf\_liverA, Unigene1479\_Mf\_liverA, NM\_010001, NM\_029692, CL1263.Contig1\_Mf\_liverA, CL4550.Contig3\_Mf\_liverA, Unigene31517\_Mf\_liverA, Unigene12786\_Mf\_liverA, NM\_007811, CL1716.Contig2\_Mf\_liverA, Unigene27895\_Mf\_liverA, NM\_008538, Unigene36112\_Mf\_liverA, NM\_011305, NM\_001104531, Unigene139\_Mf\_liverA, Unigene13498\_Mf\_liverA, CL4220.Contig2\_Mf\_liverA, Unigene27547\_Mf\_liverA, Unigene19885\_Mf\_liverA, Unigene4731\_Mf\_liverA, CL2550.Contig1\_Mf\_liverA, Unigene33366\_Mf\_liverA, CL5316.Contig1\_Mf\_liverA, Unigene45530\_Mf\_liverA, Unigene32332\_Mf\_liverA, Unigene34630\_Mf\_liverA, Unigene7195\_Mf\_liverA, Unigene5355\_Mf\_liverA, Unigene36508\_Mf\_liverA, Unigene8132\_Mf\_liverA, Unigene35506\_Mf\_liverA, NM\_019717, Unigene5712\_Mf\_liverA, Unigene37485\_Mf\_liverA, Unigene1205\_Mf\_liverA, Unigene30288\_Mf\_liverA, Unigene32943\_Mf\_liverA, NR\_003552, Unigene29882\_Mf\_liverA, Unigene13233\_Mf\_liverA, NM\_023868, NM\_010481, Unigene10136\_Mf\_liverA, NM\_009128, NM\_011732, Unigene29727\_Mf\_liverA, Unigene38913\_Mf\_liverA, CL4925.Contig1\_Mf\_liverA, CL2240.Contig1\_Mf\_liverA, Unigene13414\_Mf\_liverA, Unigene12959\_Mf\_liverA, CL425.Contig1\_Mf\_liverA, Unigene5210\_Mf\_liverA, CL1803.Contig1\_Mf\_liverA, Unigene14916\_Mf\_liverA, Unigene38208\_Mf\_liverA, CL4162.Contig1\_Mf\_liverA, Unigene15077\_Mf\_liverA, Unigene112\_Mf\_liverA, CL2517.Contig1\_Mf\_liverA, CL5986.Contig1\_Mf\_liverA, Unigene7350\_Mf\_liverA, Unigene29134\_Mf\_liverA, CL3569.Contig2\_Mf\_liverA, CL4583.Contig2\_Mf\_liverA, CL2478.Contig3\_Mf\_liverA, CL3002.Contig1\_Mf\_liverA, Unigene34375\_Mf\_liverA, Unigene36315\_Mf\_liverA, Unigene36784\_Mf\_liverA, Unigene40262\_Mf\_liverA, CL3055.Contig1\_Mf\_liverA, Unigene36514\_Mf\_liverA, NM\_009609, CL4033.Contig1\_Mf\_liverA, CL4816.Contig2\_Mf\_liverA, NM\_023294, Unigene35935\_Mf\_liverA, Unigene3439\_Mf\_liverA, Unigene33980\_Mf\_liverA, Unigene21026\_Mf\_liverA, Unigene36937\_Mf\_liverA, Unigene13683\_Mf\_liverA, CL1907.Contig1\_Mf\_liverA, Unigene15252\_Mf\_liverA, NM\_001100181, CL2791.Contig1\_Mf\_liverA, Unigene10135\_Mf\_liverA, Unigene35476\_Mf\_liverA, Unigene24858\_Mf\_liverA, NM\_009883, Unigene26138\_Mf\_liverA, CL4220.Contig1\_Mf\_liverA, CL5307.Contig1\_Mf\_liverA, NM\_007585, CL2702.Contig2\_Mf\_liverA, Unigene24466\_Mf\_liverA, NM\_007822, Unigene21163\_Mf\_liverA, Unigene25595\_Mf\_liverA, Unigene34628\_Mf\_liverA, Unigene5335\_Mf\_liverA, Unigene14212\_Mf\_liverA, Unigene26250\_Mf\_liverA, Unigene30731\_Mf\_liverA, NM\_025336, Unigene35609\_Mf\_liverA, CL4007.Contig1\_Mf\_liverA, Unigene35046\_Mf\_liverA, Unigene281\_Mf\_liverA, CL2355.Contig1\_Mf\_liverA, NM\_011978, CL634.Contig1\_Mf\_liverA, NM\_130450, Unigene14809\_Mf\_liverA, Unigene39686\_Mf\_liverA, Unigene36593\_Mf\_liverA, Unigene22500\_Mf\_liverA, NM\_009011, CL3911.Contig2\_Mf\_liverA, Unigene37028\_Mf\_liverA, Unigene21359\_Mf\_liverA, CL5698.Contig1\_Mf\_liverA, Unigene31392\_Mf\_liverA, CL336.Contig3\_Mf\_liverA, Unigene28989\_Mf\_liverA, NM\_007819 |
| platelet alpha granule | NM\_009776, Unigene12959\_Mf\_liverA, Unigene5355\_Mf\_liverA |
| mitochondrial inner membrane | Unigene17048\_Mf\_liverA, CL2517.Contig1\_Mf\_liverA, Unigene21359\_Mf\_liverA, CL5316.Contig1\_Mf\_liverA, Unigene31065\_Mf\_liverA, CL3599.Contig1\_Mf\_liverA, Unigene36735\_Mf\_liverA, CL2702.Contig2\_Mf\_liverA, Unigene13233\_Mf\_liverA |
| pigment granule | Unigene36762\_Mf\_liverA, Unigene36190\_Mf\_liverA, Unigene36189\_Mf\_liverA, NM\_007585 |
| cell-cell junction | Unigene25962\_Mf\_liverA, Unigene21684\_Mf\_liverA, Unigene12786\_Mf\_liverA, CL5586.Contig1\_Mf\_liverA, Unigene112\_Mf\_liverA, Unigene38913\_Mf\_liverA, Unigene39011\_Mf\_liverA, CL4033.Contig1\_Mf\_liverA, Unigene7350\_Mf\_liverA, CL2260.Contig1\_Mf\_liverA, Unigene13950\_Mf\_liverA |
| platelet alpha granule lumen | Unigene12959\_Mf\_liverA, Unigene5355\_Mf\_liverA |
| ion channel complex | Unigene18500\_Mf\_liverA, NM\_023868, Unigene37526\_Mf\_liverA, Unigene29593\_Mf\_liverA |
| secretory granule lumen | Unigene12959\_Mf\_liverA, Unigene5355\_Mf\_liverA |
| protein-DNA complex | Unigene21163\_Mf\_liverA, CL2439.Contig1\_Mf\_liverA, Unigene31392\_Mf\_liverA |
| replication fork | Unigene21163\_Mf\_liverA, Unigene31392\_Mf\_liverA |
| intrinsic to endoplasmic reticulum membrane | NM\_130450, Unigene1479\_Mf\_liverA, NM\_009128, Unigene10135\_Mf\_liverA |
| early endosome | Unigene37245\_Mf\_liverA, Unigene49971\_Mf\_liverA, Unigene36698\_Mf\_liverA, Unigene15077\_Mf\_liverA, CL3002.Contig1\_Mf\_liverA |
| brush border | Unigene36477\_Mf\_liverA, Unigene37485\_Mf\_liverA, Unigene42606\_Mf\_liverA |
| condensed nuclear chromosome | CL5268.Contig1\_Mf\_liverA, Unigene45530\_Mf\_liverA, NM\_023294 |
| intracellular organelle part | NM\_020559, Unigene38015\_Mf\_liverA, Unigene28142\_Mf\_liverA, CL3835.Contig2\_Mf\_liverA, Unigene36735\_Mf\_liverA, CL523.Contig1\_Mf\_liverA, Unigene172\_Mf\_liverA, NM\_011082, Unigene35507\_Mf\_liverA, Unigene31065\_Mf\_liverA, Unigene39886\_Mf\_liverA, Unigene5428\_Mf\_liverA, Unigene27592\_Mf\_liverA, Unigene4732\_Mf\_liverA, CL2790.Contig1\_Mf\_liverA, CL5586.Contig1\_Mf\_liverA, Unigene24351\_Mf\_liverA, Unigene34341\_Mf\_liverA, Unigene12999\_Mf\_liverA, CL4141.Contig1\_Mf\_liverA, Unigene36662\_Mf\_liverA, Unigene35816\_Mf\_liverA, Unigene13658\_Mf\_liverA, Unigene4725\_Mf\_liverA, Unigene25596\_Mf\_liverA, Unigene29461\_Mf\_liverA, Unigene28548\_Mf\_liverA, CL5268.Contig1\_Mf\_liverA, Unigene5294\_Mf\_liverA, Unigene23328\_Mf\_liverA, Unigene8054\_Mf\_liverA, CL4816.Contig1\_Mf\_liverA, CL2971.Contig1\_Mf\_liverA, Unigene5639\_Mf\_liverA, Unigene17048\_Mf\_liverA, Unigene4684\_Mf\_liverA, Unigene5755\_Mf\_liverA, Unigene24092\_Mf\_liverA, NM\_177093, CL3599.Contig1\_Mf\_liverA, Unigene5382\_Mf\_liverA, Unigene33512\_Mf\_liverA, Unigene1479\_Mf\_liverA, NM\_029692, CL1263.Contig1\_Mf\_liverA, CL4550.Contig3\_Mf\_liverA, Unigene31517\_Mf\_liverA, Unigene12786\_Mf\_liverA, CL1716.Contig2\_Mf\_liverA, Unigene27895\_Mf\_liverA, NM\_008538, Unigene36112\_Mf\_liverA, NM\_011305, Unigene139\_Mf\_liverA, Unigene13498\_Mf\_liverA, CL4220.Contig2\_Mf\_liverA, Unigene27547\_Mf\_liverA, Unigene19885\_Mf\_liverA, Unigene4731\_Mf\_liverA, CL2550.Contig1\_Mf\_liverA, Unigene33366\_Mf\_liverA, CL5316.Contig1\_Mf\_liverA, Unigene45530\_Mf\_liverA, Unigene32332\_Mf\_liverA, Unigene34630\_Mf\_liverA, Unigene7195\_Mf\_liverA, Unigene5355\_Mf\_liverA, Unigene36508\_Mf\_liverA, Unigene8132\_Mf\_liverA, Unigene35506\_Mf\_liverA, Unigene5712\_Mf\_liverA, Unigene37485\_Mf\_liverA, Unigene1205\_Mf\_liverA, Unigene30288\_Mf\_liverA, Unigene32943\_Mf\_liverA, Unigene29882\_Mf\_liverA, Unigene13233\_Mf\_liverA, NM\_023868, NM\_010481, Unigene10136\_Mf\_liverA, NM\_009128, NM\_011732, Unigene29727\_Mf\_liverA, Unigene38913\_Mf\_liverA, CL4925.Contig1\_Mf\_liverA, CL2240.Contig1\_Mf\_liverA, Unigene13414\_Mf\_liverA, Unigene12959\_Mf\_liverA, CL425.Contig1\_Mf\_liverA, Unigene5210\_Mf\_liverA, CL1803.Contig1\_Mf\_liverA, Unigene14916\_Mf\_liverA, Unigene38208\_Mf\_liverA, CL4162.Contig1\_Mf\_liverA, Unigene15077\_Mf\_liverA, Unigene112\_Mf\_liverA, CL2517.Contig1\_Mf\_liverA, CL5986.Contig1\_Mf\_liverA, Unigene7350\_Mf\_liverA, Unigene29134\_Mf\_liverA, CL3569.Contig2\_Mf\_liverA, CL4583.Contig2\_Mf\_liverA, CL2478.Contig3\_Mf\_liverA, CL3002.Contig1\_Mf\_liverA, Unigene34375\_Mf\_liverA, Unigene36315\_Mf\_liverA, Unigene36784\_Mf\_liverA, CL3055.Contig1\_Mf\_liverA, Unigene36514\_Mf\_liverA, NM\_009609, CL4033.Contig1\_Mf\_liverA, CL4816.Contig2\_Mf\_liverA, NM\_023294, Unigene35935\_Mf\_liverA, Unigene3439\_Mf\_liverA, Unigene33980\_Mf\_liverA, Unigene21026\_Mf\_liverA, Unigene36937\_Mf\_liverA, Unigene13683\_Mf\_liverA, CL1907.Contig1\_Mf\_liverA, Unigene15252\_Mf\_liverA, CL2791.Contig1\_Mf\_liverA, Unigene10135\_Mf\_liverA, Unigene35476\_Mf\_liverA, NM\_009883, Unigene24858\_Mf\_liverA, Unigene26138\_Mf\_liverA, CL4220.Contig1\_Mf\_liverA, CL5307.Contig1\_Mf\_liverA, NM\_007585, CL2702.Contig2\_Mf\_liverA, Unigene24466\_Mf\_liverA, Unigene21163\_Mf\_liverA, Unigene25595\_Mf\_liverA, Unigene5335\_Mf\_liverA, Unigene34628\_Mf\_liverA, Unigene14212\_Mf\_liverA, Unigene26250\_Mf\_liverA, Unigene30731\_Mf\_liverA, NM\_025336, Unigene35609\_Mf\_liverA, CL4007.Contig1\_Mf\_liverA, Unigene35046\_Mf\_liverA, Unigene281\_Mf\_liverA, CL2355.Contig1\_Mf\_liverA, NM\_011978, CL634.Contig1\_Mf\_liverA, NM\_130450, Unigene14809\_Mf\_liverA, Unigene39686\_Mf\_liverA, Unigene36593\_Mf\_liverA, Unigene22500\_Mf\_liverA, NM\_009011, CL3911.Contig2\_Mf\_liverA, Unigene37028\_Mf\_liverA, Unigene21359\_Mf\_liverA, CL5698.Contig1\_Mf\_liverA, Unigene31392\_Mf\_liverA, CL336.Contig3\_Mf\_liverA, Unigene28989\_Mf\_liverA |
| kinetochore | Unigene4684\_Mf\_liverA, CL5268.Contig1\_Mf\_liverA, Unigene45530\_Mf\_liverA, NM\_023294 |
| neuron projection | Unigene4732\_Mf\_liverA, CL4583.Contig2\_Mf\_liverA, Unigene18500\_Mf\_liverA, Unigene27547\_Mf\_liverA, Unigene4684\_Mf\_liverA, Unigene4731\_Mf\_liverA, NM\_010141, NM\_011732, NM\_009609, NM\_177093, CL425.Contig1\_Mf\_liverA, Unigene8132\_Mf\_liverA, Unigene4636\_Mf\_liverA, NM\_178405, Unigene13940\_Mf\_liverA, Unigene32316\_Mf\_liverA, Unigene36762\_Mf\_liverA, Unigene5712\_Mf\_liverA, NM\_011305, Unigene33580\_Mf\_liverA, Unigene13950\_Mf\_liverA |
| coated pit | Unigene37232\_Mf\_liverA, CL2098.Contig1\_Mf\_liverA |
| dendritic spine | Unigene4732\_Mf\_liverA, Unigene27547\_Mf\_liverA, CL425.Contig1\_Mf\_liverA, Unigene4731\_Mf\_liverA |
| neuron spine | Unigene4732\_Mf\_liverA, Unigene27547\_Mf\_liverA, CL425.Contig1\_Mf\_liverA, Unigene4731\_Mf\_liverA |
| membrane raft | Unigene12519\_Mf\_liverA, CL887.Contig2\_Mf\_liverA, Unigene37268\_Mf\_liverA, CL5698.Contig1\_Mf\_liverA, Unigene13950\_Mf\_liverA |
| actin filament | NM\_009609, NM\_177093, CL5698.Contig1\_Mf\_liverA |
| cell projection | Unigene25962\_Mf\_liverA, Unigene21684\_Mf\_liverA, Unigene4684\_Mf\_liverA, NM\_011732, Unigene42606\_Mf\_liverA, NM\_177093, NM\_021508, CL425.Contig1\_Mf\_liverA, Unigene49971\_Mf\_liverA, NM\_178405, Unigene36698\_Mf\_liverA, NM\_172203, Unigene36762\_Mf\_liverA, Unigene27895\_Mf\_liverA, NM\_011305, Unigene39011\_Mf\_liverA, Unigene33580\_Mf\_liverA, Unigene13950\_Mf\_liverA, Unigene27249\_Mf\_liverA, Unigene4732\_Mf\_liverA, CL4583.Contig2\_Mf\_liverA, Unigene27547\_Mf\_liverA, Unigene18500\_Mf\_liverA, Unigene4731\_Mf\_liverA, NM\_010141, Unigene40262\_Mf\_liverA, CL634.Contig1\_Mf\_liverA, NM\_009609, Unigene27248\_Mf\_liverA, Unigene36477\_Mf\_liverA, Unigene8132\_Mf\_liverA, Unigene4636\_Mf\_liverA, Unigene13940\_Mf\_liverA, Unigene32316\_Mf\_liverA, Unigene37485\_Mf\_liverA, Unigene5712\_Mf\_liverA |
| extrinsic to membrane | Unigene37243\_Mf\_liverA, Unigene4636\_Mf\_liverA, Unigene8054\_Mf\_liverA, NM\_007585 |
| cell projection part | Unigene4732\_Mf\_liverA, Unigene18500\_Mf\_liverA, Unigene27547\_Mf\_liverA, NM\_172203, Unigene4731\_Mf\_liverA, Unigene37485\_Mf\_liverA, Unigene27895\_Mf\_liverA, Unigene40262\_Mf\_liverA, Unigene42606\_Mf\_liverA, Unigene49971\_Mf\_liverA, CL425.Contig1\_Mf\_liverA, Unigene36477\_Mf\_liverA, Unigene8132\_Mf\_liverA, Unigene13950\_Mf\_liverA |
| cell projection membrane | Unigene36477\_Mf\_liverA, NM\_172203, Unigene49971\_Mf\_liverA, Unigene37485\_Mf\_liverA, Unigene42606\_Mf\_liverA |
| mitochondrial membrane | Unigene3439\_Mf\_liverA, Unigene38208\_Mf\_liverA, Unigene17048\_Mf\_liverA, Unigene10136\_Mf\_liverA, CL2517.Contig1\_Mf\_liverA, Unigene21359\_Mf\_liverA, CL5316.Contig1\_Mf\_liverA, Unigene31065\_Mf\_liverA, CL3599.Contig1\_Mf\_liverA, Unigene36735\_Mf\_liverA, CL2702.Contig2\_Mf\_liverA, Unigene13233\_Mf\_liverA |
| basolateral plasma membrane | Unigene4732\_Mf\_liverA, Unigene4731\_Mf\_liverA, Unigene37485\_Mf\_liverA, Unigene13356\_Mf\_liverA, CL1256.Contig1\_Mf\_liverA, Unigene42606\_Mf\_liverA, Unigene35816\_Mf\_liverA, CL838.Contig3\_Mf\_liverA, Unigene13950\_Mf\_liverA |
| lamellipodium | Unigene39011\_Mf\_liverA, Unigene25962\_Mf\_liverA, Unigene21684\_Mf\_liverA |
| secretory granule | CL3911.Contig2\_Mf\_liverA, NM\_009776, Unigene14810\_Mf\_liverA, Unigene14809\_Mf\_liverA, Unigene12959\_Mf\_liverA, Unigene8054\_Mf\_liverA, Unigene5355\_Mf\_liverA, Unigene13950\_Mf\_liverA |
| cell fraction | NM\_013863, NM\_023868, NM\_029562, NM\_009128, NM\_011978, NM\_009609, NM\_130450, NM\_177093, NR\_003630, NM\_007409, NM\_011921, NM\_008180, NM\_007585, NM\_010001, NM\_145474, NM\_178405, NM\_007822, NM\_007811, NM\_001104531, NM\_011072, NM\_007820, NM\_001100181, NR\_003552, NM\_007819 |
| integral to endoplasmic reticulum membrane | Unigene1479\_Mf\_liverA, Unigene10135\_Mf\_liverA |
| nuclear chromosome part | Unigene21163\_Mf\_liverA, CL5268.Contig1\_Mf\_liverA, CL2550.Contig1\_Mf\_liverA, Unigene24858\_Mf\_liverA, Unigene31392\_Mf\_liverA, Unigene45530\_Mf\_liverA, NM\_023294 |
| intracellular membrane-bounded organelle | Unigene21684\_Mf\_liverA, NM\_020559, Unigene38015\_Mf\_liverA, Unigene28142\_Mf\_liverA, CL3835.Contig2\_Mf\_liverA, NM\_178050, NM\_021508, Unigene36735\_Mf\_liverA, CL523.Contig1\_Mf\_liverA, Unigene15553\_Mf\_liverA, Unigene172\_Mf\_liverA, NM\_011082, CL4577.Contig1\_Mf\_liverA, Unigene36762\_Mf\_liverA, Unigene35507\_Mf\_liverA, Unigene6465\_Mf\_liverA, Unigene31065\_Mf\_liverA, CL2982.Contig1\_Mf\_liverA, NM\_011072, NM\_025613, NM\_007820, Unigene39886\_Mf\_liverA, Unigene20853\_Mf\_liverA, Unigene5428\_Mf\_liverA, Unigene27592\_Mf\_liverA, Unigene4732\_Mf\_liverA, CL2790.Contig1\_Mf\_liverA, CL5586.Contig1\_Mf\_liverA, Unigene24351\_Mf\_liverA, Unigene37999\_Mf\_liverA, Unigene34341\_Mf\_liverA, CL4141.Contig1\_Mf\_liverA, Unigene36662\_Mf\_liverA, Unigene35816\_Mf\_liverA, Unigene13658\_Mf\_liverA, NM\_145474, Unigene4725\_Mf\_liverA, Unigene32316\_Mf\_liverA, NM\_009776, Unigene25596\_Mf\_liverA, Unigene25594\_Mf\_liverA, Unigene29461\_Mf\_liverA, Unigene28548\_Mf\_liverA, CL5268.Contig1\_Mf\_liverA, CL4995.Contig1\_Mf\_liverA, Unigene5294\_Mf\_liverA, Unigene23328\_Mf\_liverA, Unigene8054\_Mf\_liverA, Unigene35171\_Mf\_liverA, Unigene38065\_Mf\_liverA, Unigene36626\_Mf\_liverA, CL4816.Contig1\_Mf\_liverA, CL2971.Contig1\_Mf\_liverA, Unigene39507\_Mf\_liverA, Unigene17048\_Mf\_liverA, Unigene4684\_Mf\_liverA, NM\_029562, Unigene5755\_Mf\_liverA, Unigene24092\_Mf\_liverA, Unigene743\_Mf\_liverA, CL3750.Contig2\_Mf\_liverA, NM\_177093, CL3599.Contig1\_Mf\_liverA, Unigene5382\_Mf\_liverA, Unigene39141\_Mf\_liverA, Unigene33512\_Mf\_liverA, Unigene34591\_Mf\_liverA, CL1595.Contig1\_Mf\_liverA, Unigene1479\_Mf\_liverA, CL838.Contig3\_Mf\_liverA, CL4332.Contig1\_Mf\_liverA, NM\_010001, CL1263.Contig1\_Mf\_liverA, CL4550.Contig3\_Mf\_liverA, Unigene31517\_Mf\_liverA, Unigene12786\_Mf\_liverA, Unigene36698\_Mf\_liverA, Unigene36628\_Mf\_liverA, NM\_007811, CL1716.Contig2\_Mf\_liverA, NM\_008538, Unigene36112\_Mf\_liverA, NM\_011305, NM\_001104531, Unigene139\_Mf\_liverA, Unigene13498\_Mf\_liverA, Unigene23158\_Mf\_liverA, Unigene4510\_Mf\_liverA, Unigene17579\_Mf\_liverA, CL4220.Contig2\_Mf\_liverA, Unigene27547\_Mf\_liverA, Unigene19885\_Mf\_liverA, CL1736.Contig2\_Mf\_liverA, Unigene4731\_Mf\_liverA, CL2550.Contig1\_Mf\_liverA, Unigene33366\_Mf\_liverA, Unigene33898\_Mf\_liverA, CL5316.Contig1\_Mf\_liverA, Unigene45530\_Mf\_liverA, Unigene32332\_Mf\_liverA, Unigene4723\_Mf\_liverA, Unigene14588\_Mf\_liverA, Unigene34630\_Mf\_liverA, NR\_003630, Unigene7195\_Mf\_liverA, Unigene5355\_Mf\_liverA, Unigene36508\_Mf\_liverA, Unigene8132\_Mf\_liverA, Unigene35506\_Mf\_liverA, NM\_019717, Unigene5712\_Mf\_liverA, Unigene37485\_Mf\_liverA, Unigene1205\_Mf\_liverA, Unigene30288\_Mf\_liverA, Unigene32943\_Mf\_liverA, NR\_003552, CL1509.Contig2\_Mf\_liverA, Unigene30528\_Mf\_liverA, Unigene29882\_Mf\_liverA, Unigene13233\_Mf\_liverA, Unigene32059\_Mf\_liverA, Unigene45904\_Mf\_liverA, Unigene25962\_Mf\_liverA, NM\_023868, NM\_010481, Unigene10136\_Mf\_liverA, NM\_009128, NM\_011732, Unigene29727\_Mf\_liverA, Unigene38913\_Mf\_liverA, CL4925.Contig1\_Mf\_liverA, CL186.Contig3\_Mf\_liverA, Unigene13414\_Mf\_liverA, CL2240.Contig1\_Mf\_liverA, Unigene12959\_Mf\_liverA, Unigene49971\_Mf\_liverA, CL425.Contig1\_Mf\_liverA, Unigene5210\_Mf\_liverA, Unigene36190\_Mf\_liverA, CL1803.Contig1\_Mf\_liverA, Unigene38208\_Mf\_liverA, Unigene37243\_Mf\_liverA, Unigene14916\_Mf\_liverA, CL4162.Contig1\_Mf\_liverA, Unigene112\_Mf\_liverA, Unigene15077\_Mf\_liverA, CL2517.Contig1\_Mf\_liverA, Unigene29510\_Mf\_liverA, Unigene24931\_Mf\_liverA, CL5986.Contig1\_Mf\_liverA, Unigene7350\_Mf\_liverA, Unigene29134\_Mf\_liverA, CL3569.Contig2\_Mf\_liverA, CL3800.Contig1\_Mf\_liverA, CL4583.Contig2\_Mf\_liverA, CL2478.Contig3\_Mf\_liverA, Unigene38280\_Mf\_liverA, Unigene34375\_Mf\_liverA, CL3002.Contig1\_Mf\_liverA, Unigene31483\_Mf\_liverA, Unigene36784\_Mf\_liverA, Unigene36315\_Mf\_liverA, Unigene40262\_Mf\_liverA, CL3055.Contig1\_Mf\_liverA, NM\_009609, Unigene36514\_Mf\_liverA, CL4033.Contig1\_Mf\_liverA, NM\_007409, CL4816.Contig2\_Mf\_liverA, Unigene34983\_Mf\_liverA, Unigene29593\_Mf\_liverA, CL887.Contig2\_Mf\_liverA, CL2439.Contig1\_Mf\_liverA, NM\_023294, Unigene3439\_Mf\_liverA, Unigene35935\_Mf\_liverA, Unigene33980\_Mf\_liverA, Unigene35037\_Mf\_liverA, Unigene21026\_Mf\_liverA, Unigene36937\_Mf\_liverA, Unigene14810\_Mf\_liverA, Unigene13683\_Mf\_liverA, Unigene15252\_Mf\_liverA, CL1907.Contig1\_Mf\_liverA, NM\_001100181, CL2791.Contig1\_Mf\_liverA, CL838.Contig6\_Mf\_liverA, CL4770.Contig1\_Mf\_liverA, Unigene10135\_Mf\_liverA, Unigene35476\_Mf\_liverA, CL3750.Contig1\_Mf\_liverA, Unigene25101\_Mf\_liverA, CL5828.Contig2\_Mf\_liverA, Unigene24858\_Mf\_liverA, NM\_009883, Unigene26138\_Mf\_liverA, CL4220.Contig1\_Mf\_liverA, CL1518.Contig1\_Mf\_liverA, CL5307.Contig1\_Mf\_liverA, NM\_008180, NR\_033215, NM\_007585, CL2702.Contig2\_Mf\_liverA, Unigene24466\_Mf\_liverA, CL1165.Contig2\_Mf\_liverA, NM\_178405, NM\_007822, Unigene21163\_Mf\_liverA, NM\_172203, Unigene25595\_Mf\_liverA, CL3312.Contig1\_Mf\_liverA, Unigene15470\_Mf\_liverA, Unigene14286\_Mf\_liverA, Unigene34628\_Mf\_liverA, Unigene5335\_Mf\_liverA, Unigene14212\_Mf\_liverA, Unigene39011\_Mf\_liverA, Unigene26250\_Mf\_liverA, Unigene30731\_Mf\_liverA, NM\_025336, Unigene35609\_Mf\_liverA, NM\_145218, Unigene36189\_Mf\_liverA, CL4007.Contig1\_Mf\_liverA, Unigene13950\_Mf\_liverA, Unigene35046\_Mf\_liverA, Unigene28459\_Mf\_liverA, Unigene37245\_Mf\_liverA, CL2355.Contig1\_Mf\_liverA, Unigene281\_Mf\_liverA, NM\_011978, NM\_130450, Unigene29985\_Mf\_liverA, Unigene14809\_Mf\_liverA, Unigene39686\_Mf\_liverA, Unigene36593\_Mf\_liverA, Unigene22500\_Mf\_liverA, NM\_027406, NM\_009011, Unigene37232\_Mf\_liverA, CL3911.Contig2\_Mf\_liverA, Unigene37239\_Mf\_liverA, Unigene37028\_Mf\_liverA, Unigene21359\_Mf\_liverA, CL5698.Contig1\_Mf\_liverA, Unigene31392\_Mf\_liverA, CL336.Contig3\_Mf\_liverA, Unigene28989\_Mf\_liverA, NM\_007819, Unigene19954\_Mf\_liverA |
| basement membrane | Unigene32908\_Mf\_liverA, Unigene8132\_Mf\_liverA, Unigene31492\_Mf\_liverA |
| insoluble fraction | NM\_013863, NM\_023868, NM\_029562, NM\_009128, NM\_011978, NM\_130450, NM\_007585, NM\_010001, NM\_145474, NM\_178405, NM\_007822, NM\_007811, NM\_001104531, NM\_001100181, NM\_007820, NM\_011072, NR\_003552, NM\_007819 |
| transcriptional repressor complex | Unigene31517\_Mf\_liverA, CL2355.Contig1\_Mf\_liverA |
| intrinsic to organelle membrane | NM\_130450, Unigene38208\_Mf\_liverA, Unigene37485\_Mf\_liverA, Unigene1479\_Mf\_liverA, NM\_009128, Unigene10135\_Mf\_liverA |
| nuclear speck | Unigene39686\_Mf\_liverA, CL2355.Contig1\_Mf\_liverA, Unigene35816\_Mf\_liverA |
| myofibril | Unigene26250\_Mf\_liverA, Unigene5639\_Mf\_liverA, NM\_023868, Unigene29593\_Mf\_liverA, NM\_080639 |
| neuronal cell body | Unigene18500\_Mf\_liverA, Unigene27547\_Mf\_liverA, CL425.Contig1\_Mf\_liverA, Unigene5712\_Mf\_liverA, Unigene8132\_Mf\_liverA, Unigene40262\_Mf\_liverA |
| internal side of plasma membrane | CL838.Contig6\_Mf\_liverA, Unigene4636\_Mf\_liverA, CL838.Contig3\_Mf\_liverA |
| condensed chromosome | Unigene4684\_Mf\_liverA, CL5268.Contig1\_Mf\_liverA, Unigene45530\_Mf\_liverA, NM\_023294 |
| intermediate filament | NM\_029692, CL4162.Contig1\_Mf\_liverA |
| membrane-bounded organelle | Unigene21684\_Mf\_liverA, NM\_020559, Unigene38015\_Mf\_liverA, Unigene28142\_Mf\_liverA, CL3835.Contig2\_Mf\_liverA, NM\_178050, NM\_021508, Unigene36735\_Mf\_liverA, CL523.Contig1\_Mf\_liverA, Unigene15553\_Mf\_liverA, Unigene172\_Mf\_liverA, NM\_011082, CL4577.Contig1\_Mf\_liverA, Unigene36762\_Mf\_liverA, Unigene35507\_Mf\_liverA, Unigene6465\_Mf\_liverA, Unigene31065\_Mf\_liverA, CL2982.Contig1\_Mf\_liverA, NM\_011072, NM\_025613, NM\_007820, Unigene39886\_Mf\_liverA, Unigene20853\_Mf\_liverA, Unigene5428\_Mf\_liverA, Unigene27592\_Mf\_liverA, Unigene4732\_Mf\_liverA, CL2790.Contig1\_Mf\_liverA, CL5586.Contig1\_Mf\_liverA, Unigene24351\_Mf\_liverA, Unigene37999\_Mf\_liverA, Unigene34341\_Mf\_liverA, CL4141.Contig1\_Mf\_liverA, Unigene36662\_Mf\_liverA, Unigene35816\_Mf\_liverA, Unigene13658\_Mf\_liverA, NM\_145474, Unigene4725\_Mf\_liverA, Unigene32316\_Mf\_liverA, NM\_009776, Unigene25596\_Mf\_liverA, Unigene25594\_Mf\_liverA, Unigene29461\_Mf\_liverA, Unigene28548\_Mf\_liverA, CL5268.Contig1\_Mf\_liverA, CL4995.Contig1\_Mf\_liverA, Unigene5294\_Mf\_liverA, Unigene23328\_Mf\_liverA, Unigene8054\_Mf\_liverA, Unigene35171\_Mf\_liverA, Unigene38065\_Mf\_liverA, Unigene36626\_Mf\_liverA, CL4816.Contig1\_Mf\_liverA, CL2971.Contig1\_Mf\_liverA, Unigene39507\_Mf\_liverA, Unigene17048\_Mf\_liverA, Unigene4684\_Mf\_liverA, NM\_029562, Unigene5755\_Mf\_liverA, Unigene24092\_Mf\_liverA, Unigene743\_Mf\_liverA, CL3750.Contig2\_Mf\_liverA, NM\_177093, CL3599.Contig1\_Mf\_liverA, Unigene5382\_Mf\_liverA, Unigene39141\_Mf\_liverA, Unigene33512\_Mf\_liverA, Unigene34591\_Mf\_liverA, CL1595.Contig1\_Mf\_liverA, Unigene1479\_Mf\_liverA, CL838.Contig3\_Mf\_liverA, CL4332.Contig1\_Mf\_liverA, NM\_010001, CL1263.Contig1\_Mf\_liverA, CL4550.Contig3\_Mf\_liverA, Unigene31517\_Mf\_liverA, Unigene12786\_Mf\_liverA, Unigene36698\_Mf\_liverA, Unigene36628\_Mf\_liverA, NM\_007811, CL1716.Contig2\_Mf\_liverA, NM\_008538, Unigene36112\_Mf\_liverA, NM\_011305, NM\_001104531, Unigene139\_Mf\_liverA, Unigene13498\_Mf\_liverA, Unigene23158\_Mf\_liverA, Unigene4510\_Mf\_liverA, Unigene17579\_Mf\_liverA, CL4220.Contig2\_Mf\_liverA, Unigene27547\_Mf\_liverA, Unigene19885\_Mf\_liverA, CL1736.Contig2\_Mf\_liverA, Unigene4731\_Mf\_liverA, CL2550.Contig1\_Mf\_liverA, Unigene33366\_Mf\_liverA, Unigene33898\_Mf\_liverA, CL5316.Contig1\_Mf\_liverA, Unigene45530\_Mf\_liverA, Unigene32332\_Mf\_liverA, Unigene4723\_Mf\_liverA, Unigene14588\_Mf\_liverA, Unigene34630\_Mf\_liverA, NR\_003630, Unigene7195\_Mf\_liverA, Unigene5355\_Mf\_liverA, Unigene36508\_Mf\_liverA, Unigene8132\_Mf\_liverA, Unigene35506\_Mf\_liverA, NM\_019717, Unigene5712\_Mf\_liverA, Unigene37485\_Mf\_liverA, Unigene1205\_Mf\_liverA, Unigene30288\_Mf\_liverA, Unigene32943\_Mf\_liverA, NR\_003552, CL1509.Contig2\_Mf\_liverA, Unigene30528\_Mf\_liverA, Unigene29882\_Mf\_liverA, Unigene13233\_Mf\_liverA, Unigene32059\_Mf\_liverA, Unigene45904\_Mf\_liverA, Unigene25962\_Mf\_liverA, NM\_023868, NM\_010481, Unigene10136\_Mf\_liverA, NM\_009128, NM\_011732, Unigene29727\_Mf\_liverA, Unigene38913\_Mf\_liverA, CL4925.Contig1\_Mf\_liverA, CL186.Contig3\_Mf\_liverA, Unigene13414\_Mf\_liverA, CL2240.Contig1\_Mf\_liverA, Unigene12959\_Mf\_liverA, Unigene49971\_Mf\_liverA, CL425.Contig1\_Mf\_liverA, Unigene5210\_Mf\_liverA, Unigene36190\_Mf\_liverA, CL1803.Contig1\_Mf\_liverA, Unigene38208\_Mf\_liverA, Unigene37243\_Mf\_liverA, Unigene14916\_Mf\_liverA, CL4162.Contig1\_Mf\_liverA, Unigene112\_Mf\_liverA, Unigene15077\_Mf\_liverA, CL2517.Contig1\_Mf\_liverA, Unigene29510\_Mf\_liverA, Unigene24931\_Mf\_liverA, CL5986.Contig1\_Mf\_liverA, Unigene7350\_Mf\_liverA, Unigene29134\_Mf\_liverA, CL3569.Contig2\_Mf\_liverA, CL3800.Contig1\_Mf\_liverA, CL4583.Contig2\_Mf\_liverA, CL2478.Contig3\_Mf\_liverA, Unigene38280\_Mf\_liverA, Unigene34375\_Mf\_liverA, CL3002.Contig1\_Mf\_liverA, Unigene31483\_Mf\_liverA, Unigene36784\_Mf\_liverA, Unigene36315\_Mf\_liverA, Unigene40262\_Mf\_liverA, CL3055.Contig1\_Mf\_liverA, NM\_009609, Unigene36514\_Mf\_liverA, CL4033.Contig1\_Mf\_liverA, NM\_007409, CL4816.Contig2\_Mf\_liverA, Unigene34983\_Mf\_liverA, Unigene29593\_Mf\_liverA, CL887.Contig2\_Mf\_liverA, CL2439.Contig1\_Mf\_liverA, NM\_023294, Unigene3439\_Mf\_liverA, Unigene35935\_Mf\_liverA, Unigene33980\_Mf\_liverA, Unigene35037\_Mf\_liverA, Unigene21026\_Mf\_liverA, Unigene36937\_Mf\_liverA, Unigene14810\_Mf\_liverA, Unigene13683\_Mf\_liverA, Unigene15252\_Mf\_liverA, CL1907.Contig1\_Mf\_liverA, NM\_001100181, CL2791.Contig1\_Mf\_liverA, CL838.Contig6\_Mf\_liverA, CL4770.Contig1\_Mf\_liverA, Unigene10135\_Mf\_liverA, Unigene35476\_Mf\_liverA, CL3750.Contig1\_Mf\_liverA, Unigene25101\_Mf\_liverA, CL5828.Contig2\_Mf\_liverA, Unigene24858\_Mf\_liverA, NM\_009883, Unigene26138\_Mf\_liverA, CL4220.Contig1\_Mf\_liverA, CL1518.Contig1\_Mf\_liverA, CL5307.Contig1\_Mf\_liverA, NM\_008180, NR\_033215, NM\_007585, CL2702.Contig2\_Mf\_liverA, Unigene24466\_Mf\_liverA, CL1165.Contig2\_Mf\_liverA, NM\_178405, NM\_007822, Unigene21163\_Mf\_liverA, NM\_172203, Unigene25595\_Mf\_liverA, CL3312.Contig1\_Mf\_liverA, Unigene15470\_Mf\_liverA, Unigene14286\_Mf\_liverA, Unigene34628\_Mf\_liverA, Unigene5335\_Mf\_liverA, Unigene14212\_Mf\_liverA, Unigene39011\_Mf\_liverA, Unigene26250\_Mf\_liverA, Unigene30731\_Mf\_liverA, NM\_025336, Unigene35609\_Mf\_liverA, NM\_145218, Unigene36189\_Mf\_liverA, CL4007.Contig1\_Mf\_liverA, Unigene13950\_Mf\_liverA, Unigene35046\_Mf\_liverA, Unigene28459\_Mf\_liverA, Unigene37245\_Mf\_liverA, CL2355.Contig1\_Mf\_liverA, Unigene281\_Mf\_liverA, NM\_011978, NM\_130450, Unigene29985\_Mf\_liverA, Unigene14809\_Mf\_liverA, Unigene39686\_Mf\_liverA, Unigene36593\_Mf\_liverA, Unigene22500\_Mf\_liverA, NM\_027406, NM\_009011, Unigene37232\_Mf\_liverA, CL3911.Contig2\_Mf\_liverA, Unigene37239\_Mf\_liverA, Unigene37028\_Mf\_liverA, Unigene21359\_Mf\_liverA, CL5698.Contig1\_Mf\_liverA, Unigene31392\_Mf\_liverA, CL336.Contig3\_Mf\_liverA, Unigene28989\_Mf\_liverA, NM\_007819, Unigene19954\_Mf\_liverA |
| microvillus | Unigene36698\_Mf\_liverA, CL634.Contig1\_Mf\_liverA |
| histone methyltransferase complex | NM\_009609, NM\_177093 |
| extrinsic to plasma membrane | Unigene37243\_Mf\_liverA, Unigene4636\_Mf\_liverA |
| tight junction | Unigene7350\_Mf\_liverA, Unigene13950\_Mf\_liverA |
| postsynaptic membrane | Unigene4732\_Mf\_liverA, Unigene4731\_Mf\_liverA |
| occluding junction | Unigene7350\_Mf\_liverA, Unigene13950\_Mf\_liverA |
| Golgi apparatus | Unigene37245\_Mf\_liverA, CL5586.Contig1\_Mf\_liverA, Unigene281\_Mf\_liverA, CL3002.Contig1\_Mf\_liverA, Unigene38913\_Mf\_liverA, CL4925.Contig1\_Mf\_liverA, Unigene5382\_Mf\_liverA, Unigene13414\_Mf\_liverA, CL4033.Contig1\_Mf\_liverA, Unigene36190\_Mf\_liverA, Unigene36662\_Mf\_liverA, CL887.Contig2\_Mf\_liverA, CL1263.Contig1\_Mf\_liverA, Unigene12786\_Mf\_liverA, Unigene112\_Mf\_liverA, Unigene36112\_Mf\_liverA, Unigene31065\_Mf\_liverA, Unigene36189\_Mf\_liverA, Unigene28989\_Mf\_liverA, Unigene27592\_Mf\_liverA, Unigene32059\_Mf\_liverA |
| endocytic vesicle | CL3911.Contig2\_Mf\_liverA, Unigene14809\_Mf\_liverA, Unigene8054\_Mf\_liverA |
| integral to organelle membrane | Unigene38208\_Mf\_liverA, Unigene1479\_Mf\_liverA, Unigene10135\_Mf\_liverA |
| intracellular part | Unigene21684\_Mf\_liverA, NM\_178050, NM\_021508, Unigene15553\_Mf\_liverA, Unigene172\_Mf\_liverA, NM\_011082, CL4577.Contig1\_Mf\_liverA, Unigene35507\_Mf\_liverA, Unigene6465\_Mf\_liverA, CL2982.Contig1\_Mf\_liverA, NM\_025613, Unigene39886\_Mf\_liverA, Unigene27339\_Mf\_liverA, Unigene4732\_Mf\_liverA, CL2790.Contig1\_Mf\_liverA, CL5586.Contig1\_Mf\_liverA, Unigene47548\_Mf\_liverA, Unigene34341\_Mf\_liverA, Unigene30153\_Mf\_liverA, Unigene36662\_Mf\_liverA, NM\_011921, Unigene35816\_Mf\_liverA, NM\_010233, NM\_145474, Unigene13940\_Mf\_liverA, Unigene4725\_Mf\_liverA, NM\_009776, Unigene30003\_Mf\_liverA, NM\_172409, Unigene25594\_Mf\_liverA, Unigene29461\_Mf\_liverA, Unigene5328\_Mf\_liverA, CL4995.Contig1\_Mf\_liverA, Unigene30002\_Mf\_liverA, Unigene8054\_Mf\_liverA, Unigene14589\_Mf\_liverA, CL4816.Contig1\_Mf\_liverA, CL2971.Contig1\_Mf\_liverA, Unigene5639\_Mf\_liverA, Unigene17048\_Mf\_liverA, Unigene4684\_Mf\_liverA, Unigene24092\_Mf\_liverA, Unigene743\_Mf\_liverA, Unigene36175\_Mf\_liverA, NM\_177093, Unigene33512\_Mf\_liverA, Unigene34591\_Mf\_liverA, Unigene1479\_Mf\_liverA, CL4332.Contig1\_Mf\_liverA, NM\_010001, NM\_080639, Unigene36698\_Mf\_liverA, Unigene27895\_Mf\_liverA, Unigene36112\_Mf\_liverA, NM\_001104531, Unigene139\_Mf\_liverA, Unigene23158\_Mf\_liverA, Unigene27249\_Mf\_liverA, Unigene27547\_Mf\_liverA, CL1736.Contig2\_Mf\_liverA, Unigene4731\_Mf\_liverA, Unigene33366\_Mf\_liverA, Unigene12909\_Mf\_liverA, Unigene33898\_Mf\_liverA, CL5316.Contig1\_Mf\_liverA, Unigene45530\_Mf\_liverA, Unigene32332\_Mf\_liverA, Unigene4723\_Mf\_liverA, Unigene14588\_Mf\_liverA, Unigene34630\_Mf\_liverA, NR\_003630, Unigene27248\_Mf\_liverA, Unigene36508\_Mf\_liverA, Unigene30839\_Mf\_liverA, Unigene37485\_Mf\_liverA, Unigene30288\_Mf\_liverA, Unigene32943\_Mf\_liverA, NR\_003552, Unigene30528\_Mf\_liverA, Unigene29882\_Mf\_liverA, Unigene32059\_Mf\_liverA, Unigene45904\_Mf\_liverA, Unigene25962\_Mf\_liverA, NM\_023868, Unigene29727\_Mf\_liverA, Unigene38913\_Mf\_liverA, CL4925.Contig1\_Mf\_liverA, CL186.Contig3\_Mf\_liverA, Unigene12959\_Mf\_liverA, CL425.Contig1\_Mf\_liverA, CL1803.Contig1\_Mf\_liverA, CL4736.Contig1\_Mf\_liverA, Unigene14916\_Mf\_liverA, Unigene37243\_Mf\_liverA, Unigene15077\_Mf\_liverA, Unigene29134\_Mf\_liverA, CL3800.Contig1\_Mf\_liverA, CL2478.Contig3\_Mf\_liverA, Unigene34375\_Mf\_liverA, CL3055.Contig1\_Mf\_liverA, NM\_028785, NM\_009609, CL4033.Contig1\_Mf\_liverA, NM\_007409, Unigene34983\_Mf\_liverA, CL2439.Contig1\_Mf\_liverA, Unigene3439\_Mf\_liverA, Unigene35935\_Mf\_liverA, Unigene33980\_Mf\_liverA, Unigene37238\_Mf\_liverA, Unigene21026\_Mf\_liverA, Unigene20371\_Mf\_liverA, Unigene36937\_Mf\_liverA, Unigene13683\_Mf\_liverA, CL1907.Contig1\_Mf\_liverA, Unigene30004\_Mf\_liverA, Unigene35476\_Mf\_liverA, CL5828.Contig2\_Mf\_liverA, Unigene24858\_Mf\_liverA, CL4220.Contig1\_Mf\_liverA, Unigene37262\_Mf\_liverA, Unigene12908\_Mf\_liverA, NR\_033215, CL2702.Contig2\_Mf\_liverA, CL1165.Contig2\_Mf\_liverA, Unigene24466\_Mf\_liverA, NM\_007822, CL5293.Contig1\_Mf\_liverA, Unigene25595\_Mf\_liverA, CL3312.Contig1\_Mf\_liverA, Unigene15470\_Mf\_liverA, Unigene14286\_Mf\_liverA, Unigene34628\_Mf\_liverA, Unigene14212\_Mf\_liverA, Unigene34962\_Mf\_liverA, Unigene39011\_Mf\_liverA, Unigene26250\_Mf\_liverA, CL1588.Contig2\_Mf\_liverA, NM\_025336, Unigene35609\_Mf\_liverA, NM\_145218, Unigene36189\_Mf\_liverA, Unigene33580\_Mf\_liverA, Unigene13950\_Mf\_liverA, Unigene35046\_Mf\_liverA, Unigene28459\_Mf\_liverA, NM\_013863, Unigene37245\_Mf\_liverA, Unigene281\_Mf\_liverA, Unigene20372\_Mf\_liverA, NM\_011978, CL634.Contig1\_Mf\_liverA, NM\_130450, Unigene29985\_Mf\_liverA, Unigene36593\_Mf\_liverA, NM\_027406, Unigene36176\_Mf\_liverA, Unigene37232\_Mf\_liverA, Unigene37239\_Mf\_liverA, Unigene37028\_Mf\_liverA, CL5698.Contig1\_Mf\_liverA, Unigene31392\_Mf\_liverA, NM\_020559, Unigene28142\_Mf\_liverA, Unigene38015\_Mf\_liverA, CL3835.Contig2\_Mf\_liverA, Unigene36735\_Mf\_liverA, NM\_019651, CL523.Contig1\_Mf\_liverA, Unigene36762\_Mf\_liverA, Unigene31065\_Mf\_liverA, NM\_011072, NM\_007820, Unigene20853\_Mf\_liverA, Unigene5428\_Mf\_liverA, Unigene27592\_Mf\_liverA, Unigene24351\_Mf\_liverA, Unigene37999\_Mf\_liverA, CL3483.Contig1\_Mf\_liverA, Unigene12999\_Mf\_liverA, CL4141.Contig1\_Mf\_liverA, Unigene13658\_Mf\_liverA, Unigene32316\_Mf\_liverA, CL2622.Contig2\_Mf\_liverA, Unigene25596\_Mf\_liverA, Unigene28548\_Mf\_liverA, CL5268.Contig1\_Mf\_liverA, Unigene5294\_Mf\_liverA, Unigene23328\_Mf\_liverA, Unigene38065\_Mf\_liverA, Unigene35171\_Mf\_liverA, Unigene36626\_Mf\_liverA, Unigene39507\_Mf\_liverA, NM\_029562, Unigene5755\_Mf\_liverA, CL3750.Contig2\_Mf\_liverA, CL3599.Contig1\_Mf\_liverA, Unigene151\_Mf\_liverA, Unigene39141\_Mf\_liverA, Unigene5382\_Mf\_liverA, CL1595.Contig1\_Mf\_liverA, CL838.Contig3\_Mf\_liverA, NM\_029692, CL1263.Contig1\_Mf\_liverA, CL4550.Contig3\_Mf\_liverA, CL258.Contig1\_Mf\_liverA, Unigene36628\_Mf\_liverA, Unigene31231\_Mf\_liverA, Unigene12786\_Mf\_liverA, Unigene31517\_Mf\_liverA, NM\_007811, NM\_008538, CL1716.Contig2\_Mf\_liverA, NM\_011305, Unigene4523\_Mf\_liverA, Unigene13498\_Mf\_liverA, Unigene4510\_Mf\_liverA, CL4220.Contig2\_Mf\_liverA, Unigene17579\_Mf\_liverA, Unigene19885\_Mf\_liverA, CL2550.Contig1\_Mf\_liverA, Unigene7195\_Mf\_liverA, Unigene8132\_Mf\_liverA, Unigene9664\_Mf\_liverA, Unigene5355\_Mf\_liverA, Unigene35506\_Mf\_liverA, NM\_019717, Unigene5712\_Mf\_liverA, Unigene1205\_Mf\_liverA, Unigene28587\_Mf\_liverA, CL1509.Contig2\_Mf\_liverA, Unigene13233\_Mf\_liverA, NM\_010481, Unigene10136\_Mf\_liverA, NM\_009128, NM\_011732, CL2240.Contig1\_Mf\_liverA, Unigene13414\_Mf\_liverA, Unigene49971\_Mf\_liverA, Unigene36190\_Mf\_liverA, Unigene5210\_Mf\_liverA, Unigene38208\_Mf\_liverA, CL4162.Contig1\_Mf\_liverA, Unigene112\_Mf\_liverA, CL2517.Contig1\_Mf\_liverA, Unigene24931\_Mf\_liverA, Unigene29510\_Mf\_liverA, CL5986.Contig1\_Mf\_liverA, Unigene15681\_Mf\_liverA, Unigene7350\_Mf\_liverA, Unigene25751\_Mf\_liverA, CL3569.Contig2\_Mf\_liverA, CL4583.Contig2\_Mf\_liverA, Unigene13894\_Mf\_liverA, Unigene38280\_Mf\_liverA, CL3002.Contig1\_Mf\_liverA, Unigene36315\_Mf\_liverA, Unigene36784\_Mf\_liverA, Unigene31483\_Mf\_liverA, Unigene40262\_Mf\_liverA, CL2327.Contig1\_Mf\_liverA, Unigene36514\_Mf\_liverA, CL4816.Contig2\_Mf\_liverA, CL887.Contig2\_Mf\_liverA, Unigene29593\_Mf\_liverA, NM\_023294, Unigene33523\_Mf\_liverA, Unigene35037\_Mf\_liverA, Unigene14810\_Mf\_liverA, CL3684.Contig2\_Mf\_liverA, NM\_001100181, Unigene15252\_Mf\_liverA, CL838.Contig6\_Mf\_liverA, CL2791.Contig1\_Mf\_liverA, CL4770.Contig1\_Mf\_liverA, CL3549.Contig1\_Mf\_liverA, Unigene10135\_Mf\_liverA, CL3750.Contig1\_Mf\_liverA, CL1588.Contig3\_Mf\_liverA, Unigene25101\_Mf\_liverA, NM\_009883, Unigene26138\_Mf\_liverA, CL3112.Contig7\_Mf\_liverA, CL1518.Contig1\_Mf\_liverA, CL5307.Contig1\_Mf\_liverA, NM\_008180, NM\_007585, NM\_178405, Unigene21163\_Mf\_liverA, Unigene36177\_Mf\_liverA, Unigene33525\_Mf\_liverA, NM\_172203, Unigene5335\_Mf\_liverA, Unigene15402\_Mf\_liverA, Unigene30731\_Mf\_liverA, CL912.Contig2\_Mf\_liverA, CL4007.Contig1\_Mf\_liverA, CL1588.Contig1\_Mf\_liverA, NM\_017379, CL2355.Contig1\_Mf\_liverA, Unigene28314\_Mf\_liverA, Unigene14809\_Mf\_liverA, Unigene39686\_Mf\_liverA, Unigene4636\_Mf\_liverA, Unigene22500\_Mf\_liverA, Unigene30892\_Mf\_liverA, NM\_009011, CL3911.Contig2\_Mf\_liverA, Unigene21359\_Mf\_liverA, Unigene12519\_Mf\_liverA, CL336.Contig3\_Mf\_liverA, Unigene28989\_Mf\_liverA, Unigene19954\_Mf\_liverA, NM\_007819, NM\_146120 |
| chromosome, centromeric region | Unigene4684\_Mf\_liverA, CL5268.Contig1\_Mf\_liverA, Unigene45530\_Mf\_liverA, NM\_023294 |
| postsynaptic density | Unigene4732\_Mf\_liverA, Unigene4731\_Mf\_liverA |
| dendritic spine head | Unigene4732\_Mf\_liverA, Unigene4731\_Mf\_liverA |
| intracellular | Unigene21684\_Mf\_liverA, NM\_178050, NM\_021508, Unigene15553\_Mf\_liverA, Unigene172\_Mf\_liverA, NM\_011082, CL4577.Contig1\_Mf\_liverA, Unigene35507\_Mf\_liverA, Unigene6465\_Mf\_liverA, CL2982.Contig1\_Mf\_liverA, NM\_025613, Unigene39886\_Mf\_liverA, Unigene27339\_Mf\_liverA, Unigene4732\_Mf\_liverA, CL2790.Contig1\_Mf\_liverA, CL5586.Contig1\_Mf\_liverA, Unigene47548\_Mf\_liverA, CL5851.Contig1\_Mf\_liverA, Unigene34341\_Mf\_liverA, Unigene30153\_Mf\_liverA, Unigene36662\_Mf\_liverA, NM\_011921, Unigene35816\_Mf\_liverA, NM\_010233, NM\_145474, Unigene13940\_Mf\_liverA, Unigene4725\_Mf\_liverA, NM\_009776, Unigene30003\_Mf\_liverA, NM\_172409, Unigene25594\_Mf\_liverA, Unigene29461\_Mf\_liverA, Unigene5328\_Mf\_liverA, CL4995.Contig1\_Mf\_liverA, Unigene30002\_Mf\_liverA, Unigene8054\_Mf\_liverA, Unigene14589\_Mf\_liverA, CL4816.Contig1\_Mf\_liverA, CL2971.Contig1\_Mf\_liverA, Unigene5639\_Mf\_liverA, Unigene17048\_Mf\_liverA, Unigene4684\_Mf\_liverA, Unigene24092\_Mf\_liverA, Unigene743\_Mf\_liverA, Unigene36175\_Mf\_liverA, NM\_177093, Unigene33512\_Mf\_liverA, Unigene34591\_Mf\_liverA, Unigene1479\_Mf\_liverA, CL4332.Contig1\_Mf\_liverA, NM\_010001, NM\_080639, Unigene36698\_Mf\_liverA, Unigene27895\_Mf\_liverA, Unigene36112\_Mf\_liverA, NM\_001104531, Unigene139\_Mf\_liverA, Unigene23158\_Mf\_liverA, Unigene27249\_Mf\_liverA, Unigene27547\_Mf\_liverA, CL1736.Contig2\_Mf\_liverA, Unigene4731\_Mf\_liverA, Unigene33366\_Mf\_liverA, Unigene12909\_Mf\_liverA, Unigene33898\_Mf\_liverA, CL5316.Contig1\_Mf\_liverA, Unigene45530\_Mf\_liverA, Unigene32332\_Mf\_liverA, Unigene4723\_Mf\_liverA, Unigene14588\_Mf\_liverA, Unigene34630\_Mf\_liverA, NR\_003630, Unigene27248\_Mf\_liverA, Unigene36508\_Mf\_liverA, Unigene30839\_Mf\_liverA, Unigene37485\_Mf\_liverA, Unigene30288\_Mf\_liverA, Unigene32943\_Mf\_liverA, NR\_003552, Unigene30528\_Mf\_liverA, Unigene29882\_Mf\_liverA, Unigene32059\_Mf\_liverA, Unigene45904\_Mf\_liverA, Unigene32202\_Mf\_liverA, Unigene25962\_Mf\_liverA, NM\_023868, Unigene29727\_Mf\_liverA, Unigene38913\_Mf\_liverA, CL4925.Contig1\_Mf\_liverA, CL186.Contig3\_Mf\_liverA, Unigene12959\_Mf\_liverA, CL425.Contig1\_Mf\_liverA, CL1803.Contig1\_Mf\_liverA, CL4736.Contig1\_Mf\_liverA, Unigene14916\_Mf\_liverA, Unigene37243\_Mf\_liverA, Unigene15077\_Mf\_liverA, Unigene29134\_Mf\_liverA, CL3800.Contig1\_Mf\_liverA, CL2478.Contig3\_Mf\_liverA, Unigene34375\_Mf\_liverA, CL3055.Contig1\_Mf\_liverA, NM\_028785, NM\_009609, CL4033.Contig1\_Mf\_liverA, NM\_007409, Unigene34983\_Mf\_liverA, CL2439.Contig1\_Mf\_liverA, Unigene3439\_Mf\_liverA, Unigene35935\_Mf\_liverA, Unigene33980\_Mf\_liverA, Unigene37238\_Mf\_liverA, Unigene21026\_Mf\_liverA, Unigene20371\_Mf\_liverA, Unigene36937\_Mf\_liverA, Unigene13683\_Mf\_liverA, CL1907.Contig1\_Mf\_liverA, Unigene30004\_Mf\_liverA, CL480.Contig1\_Mf\_liverA, Unigene35476\_Mf\_liverA, CL5828.Contig2\_Mf\_liverA, Unigene24858\_Mf\_liverA, CL4220.Contig1\_Mf\_liverA, Unigene37262\_Mf\_liverA, Unigene12908\_Mf\_liverA, NR\_033215, CL2702.Contig2\_Mf\_liverA, CL1165.Contig2\_Mf\_liverA, Unigene24466\_Mf\_liverA, NM\_007822, CL5293.Contig1\_Mf\_liverA, Unigene25595\_Mf\_liverA, CL3312.Contig1\_Mf\_liverA, Unigene15470\_Mf\_liverA, Unigene14286\_Mf\_liverA, Unigene34628\_Mf\_liverA, Unigene14212\_Mf\_liverA, Unigene34962\_Mf\_liverA, Unigene39011\_Mf\_liverA, Unigene26250\_Mf\_liverA, CL1588.Contig2\_Mf\_liverA, NM\_025336, Unigene35609\_Mf\_liverA, NM\_145218, Unigene36189\_Mf\_liverA, Unigene33580\_Mf\_liverA, Unigene13950\_Mf\_liverA, Unigene35046\_Mf\_liverA, Unigene28459\_Mf\_liverA, NM\_013863, Unigene37245\_Mf\_liverA, Unigene281\_Mf\_liverA, Unigene20372\_Mf\_liverA, NM\_011978, CL634.Contig1\_Mf\_liverA, NM\_130450, Unigene29985\_Mf\_liverA, Unigene36593\_Mf\_liverA, NM\_027406, Unigene36176\_Mf\_liverA, Unigene37232\_Mf\_liverA, Unigene37239\_Mf\_liverA, Unigene37028\_Mf\_liverA, CL5698.Contig1\_Mf\_liverA, Unigene31392\_Mf\_liverA, NM\_020559, Unigene28142\_Mf\_liverA, Unigene38015\_Mf\_liverA, CL3835.Contig2\_Mf\_liverA, Unigene36735\_Mf\_liverA, NM\_019651, CL523.Contig1\_Mf\_liverA, Unigene36762\_Mf\_liverA, Unigene31065\_Mf\_liverA, Unigene38035\_Mf\_liverA, NM\_011072, NM\_007820, Unigene20853\_Mf\_liverA, Unigene5428\_Mf\_liverA, Unigene27592\_Mf\_liverA, Unigene24351\_Mf\_liverA, Unigene37999\_Mf\_liverA, CL3483.Contig1\_Mf\_liverA, Unigene12999\_Mf\_liverA, CL4141.Contig1\_Mf\_liverA, Unigene13658\_Mf\_liverA, Unigene32316\_Mf\_liverA, CL2622.Contig2\_Mf\_liverA, Unigene25596\_Mf\_liverA, Unigene28548\_Mf\_liverA, CL5268.Contig1\_Mf\_liverA, Unigene5294\_Mf\_liverA, Unigene23328\_Mf\_liverA, Unigene38065\_Mf\_liverA, Unigene35171\_Mf\_liverA, Unigene36626\_Mf\_liverA, Unigene39507\_Mf\_liverA, NM\_029562, Unigene5755\_Mf\_liverA, CL3750.Contig2\_Mf\_liverA, CL3599.Contig1\_Mf\_liverA, Unigene151\_Mf\_liverA, Unigene39141\_Mf\_liverA, Unigene5382\_Mf\_liverA, CL1595.Contig1\_Mf\_liverA, CL838.Contig3\_Mf\_liverA, NM\_029692, CL1263.Contig1\_Mf\_liverA, CL4550.Contig3\_Mf\_liverA, CL258.Contig1\_Mf\_liverA, Unigene36628\_Mf\_liverA, Unigene31231\_Mf\_liverA, Unigene12786\_Mf\_liverA, Unigene31517\_Mf\_liverA, NM\_007811, NM\_008538, CL1716.Contig2\_Mf\_liverA, NM\_011305, Unigene4523\_Mf\_liverA, Unigene13498\_Mf\_liverA, Unigene4510\_Mf\_liverA, CL4220.Contig2\_Mf\_liverA, Unigene17579\_Mf\_liverA, Unigene19885\_Mf\_liverA, CL2550.Contig1\_Mf\_liverA, Unigene7195\_Mf\_liverA, Unigene8132\_Mf\_liverA, Unigene9664\_Mf\_liverA, Unigene5355\_Mf\_liverA, Unigene35506\_Mf\_liverA, NM\_019717, Unigene5712\_Mf\_liverA, Unigene1205\_Mf\_liverA, Unigene28587\_Mf\_liverA, CL1509.Contig2\_Mf\_liverA, Unigene13233\_Mf\_liverA, NM\_010481, Unigene10136\_Mf\_liverA, NM\_009128, NM\_011732, CL2240.Contig1\_Mf\_liverA, Unigene13414\_Mf\_liverA, Unigene49971\_Mf\_liverA, Unigene36190\_Mf\_liverA, Unigene5210\_Mf\_liverA, Unigene38208\_Mf\_liverA, CL4162.Contig1\_Mf\_liverA, Unigene112\_Mf\_liverA, CL2517.Contig1\_Mf\_liverA, Unigene24931\_Mf\_liverA, Unigene29510\_Mf\_liverA, CL5986.Contig1\_Mf\_liverA, Unigene15681\_Mf\_liverA, Unigene7350\_Mf\_liverA, Unigene25751\_Mf\_liverA, CL3569.Contig2\_Mf\_liverA, CL4583.Contig2\_Mf\_liverA, Unigene13894\_Mf\_liverA, Unigene38280\_Mf\_liverA, CL3002.Contig1\_Mf\_liverA, Unigene36315\_Mf\_liverA, Unigene36784\_Mf\_liverA, Unigene31483\_Mf\_liverA, Unigene40262\_Mf\_liverA, CL2327.Contig1\_Mf\_liverA, Unigene36514\_Mf\_liverA, CL4816.Contig2\_Mf\_liverA, CL887.Contig2\_Mf\_liverA, Unigene29593\_Mf\_liverA, NM\_023294, Unigene33523\_Mf\_liverA, Unigene35037\_Mf\_liverA, Unigene14810\_Mf\_liverA, CL3684.Contig2\_Mf\_liverA, NM\_001100181, Unigene15252\_Mf\_liverA, CL838.Contig6\_Mf\_liverA, CL2791.Contig1\_Mf\_liverA, CL4770.Contig1\_Mf\_liverA, CL3549.Contig1\_Mf\_liverA, Unigene10135\_Mf\_liverA, CL3750.Contig1\_Mf\_liverA, CL1588.Contig3\_Mf\_liverA, Unigene25101\_Mf\_liverA, NM\_009883, Unigene26138\_Mf\_liverA, CL3112.Contig7\_Mf\_liverA, CL1518.Contig1\_Mf\_liverA, CL5307.Contig1\_Mf\_liverA, NM\_008180, NM\_007585, NM\_178405, Unigene21163\_Mf\_liverA, Unigene36177\_Mf\_liverA, Unigene33525\_Mf\_liverA, NM\_172203, Unigene37100\_Mf\_liverA, Unigene5335\_Mf\_liverA, Unigene15402\_Mf\_liverA, Unigene30731\_Mf\_liverA, CL912.Contig2\_Mf\_liverA, CL4007.Contig1\_Mf\_liverA, CL1588.Contig1\_Mf\_liverA, NM\_017379, CL2355.Contig1\_Mf\_liverA, Unigene28314\_Mf\_liverA, Unigene14809\_Mf\_liverA, Unigene39686\_Mf\_liverA, Unigene4636\_Mf\_liverA, Unigene22500\_Mf\_liverA, Unigene30892\_Mf\_liverA, NM\_009011, CL3911.Contig2\_Mf\_liverA, Unigene21359\_Mf\_liverA, Unigene12519\_Mf\_liverA, CL336.Contig3\_Mf\_liverA, Unigene28989\_Mf\_liverA, NM\_007819, NM\_146120, Unigene19954\_Mf\_liverA |
| organelle inner membrane | Unigene17048\_Mf\_liverA, CL2517.Contig1\_Mf\_liverA, Unigene21359\_Mf\_liverA, CL5316.Contig1\_Mf\_liverA, Unigene31065\_Mf\_liverA, CL3599.Contig1\_Mf\_liverA, Unigene36735\_Mf\_liverA, NM\_025336, CL2702.Contig2\_Mf\_liverA, Unigene13233\_Mf\_liverA |
| methyltransferase complex | NM\_009609, NM\_177093 |
| cell body | Unigene4732\_Mf\_liverA, Unigene18500\_Mf\_liverA, Unigene27547\_Mf\_liverA, Unigene4731\_Mf\_liverA, Unigene5712\_Mf\_liverA, NM\_010141, Unigene40262\_Mf\_liverA, CL425.Contig1\_Mf\_liverA, Unigene8132\_Mf\_liverA, Unigene13950\_Mf\_liverA |
| dendrite | Unigene4732\_Mf\_liverA, Unigene18500\_Mf\_liverA, Unigene27547\_Mf\_liverA, CL425.Contig1\_Mf\_liverA, Unigene4684\_Mf\_liverA, Unigene4731\_Mf\_liverA, NM\_178405 |
| synaptic membrane | Unigene4732\_Mf\_liverA, Unigene4731\_Mf\_liverA |
| nuclear chromosome | Unigene21163\_Mf\_liverA, CL5268.Contig1\_Mf\_liverA, CL2550.Contig1\_Mf\_liverA, Unigene24858\_Mf\_liverA, Unigene31392\_Mf\_liverA, Unigene45530\_Mf\_liverA, NM\_023294 |
| intermediate filament cytoskeleton | NM\_029692, CL4162.Contig1\_Mf\_liverA |
| sarcomere | Unigene26250\_Mf\_liverA, Unigene5639\_Mf\_liverA, NM\_023868 |
| I band | Unigene26250\_Mf\_liverA, NM\_023868 |
| Golgi membrane | Unigene12786\_Mf\_liverA, CL5586.Contig1\_Mf\_liverA, Unigene112\_Mf\_liverA, Unigene281\_Mf\_liverA, Unigene38913\_Mf\_liverA, CL4925.Contig1\_Mf\_liverA, CL4033.Contig1\_Mf\_liverA, Unigene28989\_Mf\_liverA |
| synapse | Unigene4732\_Mf\_liverA, Unigene18500\_Mf\_liverA, Unigene4731\_Mf\_liverA, NM\_010141, Unigene5355\_Mf\_liverA, Unigene15470\_Mf\_liverA, Unigene13950\_Mf\_liverA |
| contractile fiber part | Unigene26250\_Mf\_liverA, Unigene5639\_Mf\_liverA, NM\_023868 |
| organelle outer membrane | CL2478.Contig3\_Mf\_liverA, Unigene172\_Mf\_liverA |
| nuclear periphery | CL4162.Contig1\_Mf\_liverA, NM\_009883 |
| chromatin remodeling complex | NM\_009609, NM\_177093, Unigene31517\_Mf\_liverA |
| cytoplasmic membrane-bounded vesicle | CL3055.Contig1\_Mf\_liverA, CL4925.Contig1\_Mf\_liverA, Unigene14809\_Mf\_liverA, Unigene12959\_Mf\_liverA, Unigene36190\_Mf\_liverA, Unigene5355\_Mf\_liverA, CL838.Contig3\_Mf\_liverA, NM\_011082, NM\_007585, Unigene4725\_Mf\_liverA, NM\_009776, CL3911.Contig2\_Mf\_liverA, Unigene36762\_Mf\_liverA, Unigene15470\_Mf\_liverA, Unigene14810\_Mf\_liverA, Unigene36189\_Mf\_liverA, Unigene8054\_Mf\_liverA, Unigene13950\_Mf\_liverA |
| cytosolic part | Unigene12999\_Mf\_liverA, CL3684.Contig2\_Mf\_liverA |
| outer membrane | CL2478.Contig3\_Mf\_liverA, Unigene172\_Mf\_liverA |
| membrane-bounded vesicle | CL1555.Contig1\_Mf\_liverA, CL3055.Contig1\_Mf\_liverA, CL4925.Contig1\_Mf\_liverA, Unigene14809\_Mf\_liverA, Unigene12959\_Mf\_liverA, Unigene36190\_Mf\_liverA, Unigene5355\_Mf\_liverA, CL838.Contig3\_Mf\_liverA, NM\_011082, NM\_007585, Unigene4725\_Mf\_liverA, NM\_009776, CL3911.Contig2\_Mf\_liverA, Unigene36762\_Mf\_liverA, Unigene15470\_Mf\_liverA, Unigene14810\_Mf\_liverA, Unigene36189\_Mf\_liverA, Unigene8054\_Mf\_liverA, Unigene13950\_Mf\_liverA |
| actin cytoskeleton | NM\_009609, NM\_177093, Unigene5639\_Mf\_liverA, Unigene37238\_Mf\_liverA, CL5698.Contig1\_Mf\_liverA, NM\_007585, CL634.Contig1\_Mf\_liverA |
| Golgi apparatus part | Unigene12786\_Mf\_liverA, CL5586.Contig1\_Mf\_liverA, Unigene112\_Mf\_liverA, Unigene281\_Mf\_liverA, Unigene36112\_Mf\_liverA, Unigene38913\_Mf\_liverA, CL4925.Contig1\_Mf\_liverA, Unigene5382\_Mf\_liverA, CL4033.Contig1\_Mf\_liverA, Unigene28989\_Mf\_liverA |
| cytoplasmic vesicle part | Unigene14809\_Mf\_liverA, Unigene12959\_Mf\_liverA, Unigene5355\_Mf\_liverA, Unigene8054\_Mf\_liverA, CL4925.Contig1\_Mf\_liverA |
| nucleoplasm | CL2790.Contig1\_Mf\_liverA, Unigene38015\_Mf\_liverA, Unigene33366\_Mf\_liverA, CL2355.Contig1\_Mf\_liverA, Unigene24092\_Mf\_liverA, NM\_009609, NM\_177093, CL4141.Contig1\_Mf\_liverA, Unigene33512\_Mf\_liverA, Unigene39686\_Mf\_liverA, Unigene5210\_Mf\_liverA, Unigene1479\_Mf\_liverA, Unigene35816\_Mf\_liverA, Unigene13658\_Mf\_liverA, Unigene35935\_Mf\_liverA, Unigene33980\_Mf\_liverA, Unigene21163\_Mf\_liverA, CL4162.Contig1\_Mf\_liverA, Unigene31517\_Mf\_liverA, Unigene31392\_Mf\_liverA, Unigene30288\_Mf\_liverA, Unigene14212\_Mf\_liverA, CL1907.Contig1\_Mf\_liverA, Unigene7350\_Mf\_liverA, Unigene29134\_Mf\_liverA |
| cell junction | Unigene25962\_Mf\_liverA, Unigene21684\_Mf\_liverA, Unigene12786\_Mf\_liverA, CL5586.Contig1\_Mf\_liverA, Unigene112\_Mf\_liverA, Unigene38913\_Mf\_liverA, Unigene39011\_Mf\_liverA, Unigene7350\_Mf\_liverA, Unigene49971\_Mf\_liverA, CL4033.Contig1\_Mf\_liverA, CL838.Contig3\_Mf\_liverA, CL2260.Contig1\_Mf\_liverA, Unigene13950\_Mf\_liverA |
| axon | Unigene32316\_Mf\_liverA, CL4583.Contig2\_Mf\_liverA, Unigene18500\_Mf\_liverA, Unigene8132\_Mf\_liverA |
| transcription factor complex | Unigene35935\_Mf\_liverA, CL2790.Contig1\_Mf\_liverA, Unigene33366\_Mf\_liverA, Unigene13658\_Mf\_liverA |
| synapse part | Unigene4732\_Mf\_liverA, Unigene4731\_Mf\_liverA, Unigene15470\_Mf\_liverA |
| chromosomal part | Unigene21163\_Mf\_liverA, Unigene31517\_Mf\_liverA, Unigene4684\_Mf\_liverA, CL2550.Contig1\_Mf\_liverA, Unigene24092\_Mf\_liverA, NM\_009883, NM\_011305, Unigene24858\_Mf\_liverA, Unigene31392\_Mf\_liverA, Unigene45530\_Mf\_liverA, CL5268.Contig1\_Mf\_liverA, NM\_023294 |
| apical junction complex | Unigene7350\_Mf\_liverA, Unigene13950\_Mf\_liverA |
| apicolateral plasma membrane | Unigene7350\_Mf\_liverA, Unigene13950\_Mf\_liverA |
| cytoplasmic vesicle | Unigene38280\_Mf\_liverA, CL3055.Contig1\_Mf\_liverA, CL4925.Contig1\_Mf\_liverA, Unigene14809\_Mf\_liverA, Unigene12959\_Mf\_liverA, Unigene36190\_Mf\_liverA, Unigene5355\_Mf\_liverA, CL838.Contig3\_Mf\_liverA, NM\_011082, NM\_007585, Unigene4725\_Mf\_liverA, NM\_009776, CL3911.Contig2\_Mf\_liverA, Unigene36762\_Mf\_liverA, Unigene15470\_Mf\_liverA, Unigene14810\_Mf\_liverA, Unigene36189\_Mf\_liverA, Unigene8054\_Mf\_liverA, Unigene13950\_Mf\_liverA |
| chromatin | Unigene31517\_Mf\_liverA, Unigene24092\_Mf\_liverA, NM\_009883, NM\_011305, Unigene24858\_Mf\_liverA |
| microtubule | CL3569.Contig2\_Mf\_liverA, Unigene27547\_Mf\_liverA, CL425.Contig1\_Mf\_liverA |
| intracellular organelle | Unigene21684\_Mf\_liverA, NM\_178050, NM\_021508, Unigene15553\_Mf\_liverA, Unigene172\_Mf\_liverA, NM\_011082, CL4577.Contig1\_Mf\_liverA, Unigene35507\_Mf\_liverA, Unigene6465\_Mf\_liverA, CL2982.Contig1\_Mf\_liverA, NM\_025613, Unigene39886\_Mf\_liverA, Unigene4732\_Mf\_liverA, CL2790.Contig1\_Mf\_liverA, CL5586.Contig1\_Mf\_liverA, Unigene34341\_Mf\_liverA, Unigene36662\_Mf\_liverA, Unigene35816\_Mf\_liverA, NM\_145474, Unigene4725\_Mf\_liverA, NM\_009776, Unigene25594\_Mf\_liverA, Unigene29461\_Mf\_liverA, CL4995.Contig1\_Mf\_liverA, Unigene8054\_Mf\_liverA, CL4816.Contig1\_Mf\_liverA, CL2971.Contig1\_Mf\_liverA, Unigene5639\_Mf\_liverA, Unigene17048\_Mf\_liverA, Unigene4684\_Mf\_liverA, Unigene24092\_Mf\_liverA, Unigene743\_Mf\_liverA, NM\_177093, Unigene33512\_Mf\_liverA, Unigene34591\_Mf\_liverA, Unigene1479\_Mf\_liverA, CL4332.Contig1\_Mf\_liverA, NM\_010001, NM\_080639, Unigene36698\_Mf\_liverA, Unigene27895\_Mf\_liverA, Unigene36112\_Mf\_liverA, NM\_001104531, Unigene139\_Mf\_liverA, Unigene23158\_Mf\_liverA, Unigene27547\_Mf\_liverA, CL1736.Contig2\_Mf\_liverA, Unigene4731\_Mf\_liverA, Unigene33366\_Mf\_liverA, Unigene33898\_Mf\_liverA, CL5316.Contig1\_Mf\_liverA, Unigene45530\_Mf\_liverA, Unigene32332\_Mf\_liverA, Unigene4723\_Mf\_liverA, Unigene14588\_Mf\_liverA, Unigene34630\_Mf\_liverA, NR\_003630, Unigene36508\_Mf\_liverA, Unigene37485\_Mf\_liverA, Unigene30288\_Mf\_liverA, Unigene32943\_Mf\_liverA, NR\_003552, Unigene30528\_Mf\_liverA, Unigene29882\_Mf\_liverA, Unigene32059\_Mf\_liverA, Unigene45904\_Mf\_liverA, Unigene25962\_Mf\_liverA, NM\_023868, Unigene29727\_Mf\_liverA, Unigene38913\_Mf\_liverA, CL4925.Contig1\_Mf\_liverA, CL186.Contig3\_Mf\_liverA, Unigene12959\_Mf\_liverA, CL425.Contig1\_Mf\_liverA, CL1803.Contig1\_Mf\_liverA, Unigene37243\_Mf\_liverA, Unigene14916\_Mf\_liverA, Unigene15077\_Mf\_liverA, Unigene29134\_Mf\_liverA, CL3800.Contig1\_Mf\_liverA, CL2478.Contig3\_Mf\_liverA, Unigene34375\_Mf\_liverA, CL3055.Contig1\_Mf\_liverA, NM\_009609, CL4033.Contig1\_Mf\_liverA, NM\_007409, Unigene34983\_Mf\_liverA, CL2439.Contig1\_Mf\_liverA, Unigene3439\_Mf\_liverA, Unigene35935\_Mf\_liverA, Unigene33980\_Mf\_liverA, Unigene37238\_Mf\_liverA, Unigene21026\_Mf\_liverA, Unigene36937\_Mf\_liverA, Unigene13683\_Mf\_liverA, CL1907.Contig1\_Mf\_liverA, Unigene35476\_Mf\_liverA, CL5828.Contig2\_Mf\_liverA, Unigene24858\_Mf\_liverA, CL4220.Contig1\_Mf\_liverA, NR\_033215, CL2702.Contig2\_Mf\_liverA, Unigene24466\_Mf\_liverA, CL1165.Contig2\_Mf\_liverA, NM\_007822, Unigene25595\_Mf\_liverA, CL3312.Contig1\_Mf\_liverA, Unigene15470\_Mf\_liverA, Unigene14286\_Mf\_liverA, Unigene34628\_Mf\_liverA, Unigene14212\_Mf\_liverA, Unigene39011\_Mf\_liverA, Unigene26250\_Mf\_liverA, NM\_025336, Unigene35609\_Mf\_liverA, NM\_145218, Unigene36189\_Mf\_liverA, Unigene13950\_Mf\_liverA, Unigene35046\_Mf\_liverA, Unigene28459\_Mf\_liverA, Unigene37245\_Mf\_liverA, Unigene281\_Mf\_liverA, NM\_011978, CL634.Contig1\_Mf\_liverA, NM\_130450, Unigene29985\_Mf\_liverA, Unigene36593\_Mf\_liverA, NM\_027406, Unigene37232\_Mf\_liverA, Unigene37239\_Mf\_liverA, Unigene37028\_Mf\_liverA, CL5698.Contig1\_Mf\_liverA, Unigene31392\_Mf\_liverA, NM\_020559, Unigene28142\_Mf\_liverA, Unigene38015\_Mf\_liverA, CL3835.Contig2\_Mf\_liverA, Unigene36735\_Mf\_liverA, CL523.Contig1\_Mf\_liverA, Unigene36762\_Mf\_liverA, Unigene31065\_Mf\_liverA, NM\_007820, NM\_011072, Unigene20853\_Mf\_liverA, Unigene5428\_Mf\_liverA, Unigene27592\_Mf\_liverA, Unigene24351\_Mf\_liverA, Unigene37999\_Mf\_liverA, Unigene12999\_Mf\_liverA, CL4141.Contig1\_Mf\_liverA, Unigene13658\_Mf\_liverA, Unigene32316\_Mf\_liverA, Unigene25596\_Mf\_liverA, Unigene28548\_Mf\_liverA, Unigene5294\_Mf\_liverA, CL5268.Contig1\_Mf\_liverA, Unigene23328\_Mf\_liverA, Unigene38065\_Mf\_liverA, Unigene35171\_Mf\_liverA, Unigene36626\_Mf\_liverA, Unigene39507\_Mf\_liverA, NM\_029562, Unigene5755\_Mf\_liverA, CL3750.Contig2\_Mf\_liverA, Unigene39141\_Mf\_liverA, Unigene5382\_Mf\_liverA, CL3599.Contig1\_Mf\_liverA, CL1595.Contig1\_Mf\_liverA, CL838.Contig3\_Mf\_liverA, CL4550.Contig3\_Mf\_liverA, CL1263.Contig1\_Mf\_liverA, NM\_029692, Unigene36628\_Mf\_liverA, Unigene12786\_Mf\_liverA, Unigene31517\_Mf\_liverA, NM\_007811, NM\_008538, CL1716.Contig2\_Mf\_liverA, NM\_011305, Unigene13498\_Mf\_liverA, Unigene4510\_Mf\_liverA, CL4220.Contig2\_Mf\_liverA, Unigene17579\_Mf\_liverA, Unigene19885\_Mf\_liverA, CL2550.Contig1\_Mf\_liverA, Unigene7195\_Mf\_liverA, Unigene8132\_Mf\_liverA, Unigene5355\_Mf\_liverA, Unigene35506\_Mf\_liverA, NM\_019717, Unigene5712\_Mf\_liverA, Unigene1205\_Mf\_liverA, CL1509.Contig2\_Mf\_liverA, Unigene13233\_Mf\_liverA, NM\_010481, Unigene10136\_Mf\_liverA, NM\_009128, NM\_011732, CL2240.Contig1\_Mf\_liverA, Unigene13414\_Mf\_liverA, Unigene49971\_Mf\_liverA, Unigene36190\_Mf\_liverA, Unigene5210\_Mf\_liverA, Unigene38208\_Mf\_liverA, CL4162.Contig1\_Mf\_liverA, Unigene112\_Mf\_liverA, CL2517.Contig1\_Mf\_liverA, Unigene24931\_Mf\_liverA, Unigene29510\_Mf\_liverA, CL5986.Contig1\_Mf\_liverA, Unigene7350\_Mf\_liverA, Unigene25751\_Mf\_liverA, CL3569.Contig2\_Mf\_liverA, CL4583.Contig2\_Mf\_liverA, CL3002.Contig1\_Mf\_liverA, Unigene38280\_Mf\_liverA, Unigene36315\_Mf\_liverA, Unigene36784\_Mf\_liverA, Unigene31483\_Mf\_liverA, Unigene40262\_Mf\_liverA, Unigene36514\_Mf\_liverA, CL887.Contig2\_Mf\_liverA, Unigene29593\_Mf\_liverA, CL4816.Contig2\_Mf\_liverA, NM\_023294, Unigene35037\_Mf\_liverA, Unigene14810\_Mf\_liverA, NM\_001100181, Unigene15252\_Mf\_liverA, CL838.Contig6\_Mf\_liverA, CL2791.Contig1\_Mf\_liverA, CL4770.Contig1\_Mf\_liverA, Unigene10135\_Mf\_liverA, CL3750.Contig1\_Mf\_liverA, Unigene25101\_Mf\_liverA, NM\_009883, Unigene26138\_Mf\_liverA, CL1518.Contig1\_Mf\_liverA, CL5307.Contig1\_Mf\_liverA, NM\_008180, NM\_007585, NM\_178405, Unigene21163\_Mf\_liverA, NM\_172203, Unigene5335\_Mf\_liverA, Unigene30731\_Mf\_liverA, CL4007.Contig1\_Mf\_liverA, NM\_017379, CL2355.Contig1\_Mf\_liverA, Unigene14809\_Mf\_liverA, Unigene39686\_Mf\_liverA, Unigene22500\_Mf\_liverA, NM\_009011, CL3911.Contig2\_Mf\_liverA, Unigene21359\_Mf\_liverA, CL336.Contig3\_Mf\_liverA, Unigene28989\_Mf\_liverA, Unigene19954\_Mf\_liverA, NM\_007819, NM\_146120 |
| nucleolus | CL3569.Contig2\_Mf\_liverA, Unigene24351\_Mf\_liverA, Unigene24092\_Mf\_liverA, Unigene26138\_Mf\_liverA, Unigene39686\_Mf\_liverA, Unigene1479\_Mf\_liverA, CL1803.Contig1\_Mf\_liverA, CL2702.Contig2\_Mf\_liverA, Unigene24466\_Mf\_liverA, Unigene14916\_Mf\_liverA, Unigene37028\_Mf\_liverA, Unigene14212\_Mf\_liverA, Unigene31392\_Mf\_liverA, Unigene13683\_Mf\_liverA, CL1907.Contig1\_Mf\_liverA, CL2791.Contig1\_Mf\_liverA, Unigene30731\_Mf\_liverA |
| vesicle | CL1555.Contig1\_Mf\_liverA, Unigene38280\_Mf\_liverA, CL3055.Contig1\_Mf\_liverA, CL4925.Contig1\_Mf\_liverA, Unigene14809\_Mf\_liverA, Unigene12959\_Mf\_liverA, Unigene36190\_Mf\_liverA, Unigene5355\_Mf\_liverA, Unigene8132\_Mf\_liverA, CL838.Contig3\_Mf\_liverA, NM\_007585, NM\_011082, Unigene4725\_Mf\_liverA, NM\_009776, CL3911.Contig2\_Mf\_liverA, Unigene36762\_Mf\_liverA, Unigene15470\_Mf\_liverA, Unigene14810\_Mf\_liverA, Unigene36189\_Mf\_liverA, Unigene8054\_Mf\_liverA, Unigene13950\_Mf\_liverA |
| organelle | Unigene21684\_Mf\_liverA, NM\_178050, NM\_021508, Unigene15553\_Mf\_liverA, Unigene172\_Mf\_liverA, NM\_011082, CL4577.Contig1\_Mf\_liverA, Unigene35507\_Mf\_liverA, Unigene6465\_Mf\_liverA, CL2982.Contig1\_Mf\_liverA, NM\_025613, Unigene39886\_Mf\_liverA, Unigene4732\_Mf\_liverA, CL2790.Contig1\_Mf\_liverA, CL1555.Contig1\_Mf\_liverA, CL5586.Contig1\_Mf\_liverA, Unigene34341\_Mf\_liverA, Unigene36662\_Mf\_liverA, Unigene35816\_Mf\_liverA, NM\_145474, Unigene4725\_Mf\_liverA, NM\_009776, Unigene25594\_Mf\_liverA, Unigene29461\_Mf\_liverA, CL4995.Contig1\_Mf\_liverA, Unigene8054\_Mf\_liverA, CL4816.Contig1\_Mf\_liverA, CL2971.Contig1\_Mf\_liverA, Unigene5639\_Mf\_liverA, Unigene17048\_Mf\_liverA, Unigene4684\_Mf\_liverA, Unigene24092\_Mf\_liverA, Unigene743\_Mf\_liverA, NM\_177093, Unigene33512\_Mf\_liverA, Unigene34591\_Mf\_liverA, Unigene1479\_Mf\_liverA, CL4332.Contig1\_Mf\_liverA, NM\_010001, NM\_080639, Unigene36698\_Mf\_liverA, Unigene27895\_Mf\_liverA, Unigene36112\_Mf\_liverA, NM\_001104531, Unigene139\_Mf\_liverA, Unigene23158\_Mf\_liverA, Unigene27547\_Mf\_liverA, CL1736.Contig2\_Mf\_liverA, Unigene4731\_Mf\_liverA, Unigene33366\_Mf\_liverA, Unigene33898\_Mf\_liverA, CL5316.Contig1\_Mf\_liverA, Unigene45530\_Mf\_liverA, Unigene32332\_Mf\_liverA, Unigene4723\_Mf\_liverA, Unigene14588\_Mf\_liverA, Unigene34630\_Mf\_liverA, NR\_003630, Unigene36508\_Mf\_liverA, Unigene37485\_Mf\_liverA, Unigene30288\_Mf\_liverA, Unigene32943\_Mf\_liverA, NR\_003552, Unigene30528\_Mf\_liverA, Unigene29882\_Mf\_liverA, Unigene32059\_Mf\_liverA, Unigene45904\_Mf\_liverA, Unigene25962\_Mf\_liverA, NM\_023868, Unigene29727\_Mf\_liverA, Unigene38913\_Mf\_liverA, CL4925.Contig1\_Mf\_liverA, CL186.Contig3\_Mf\_liverA, Unigene12959\_Mf\_liverA, CL425.Contig1\_Mf\_liverA, CL1803.Contig1\_Mf\_liverA, Unigene37243\_Mf\_liverA, Unigene14916\_Mf\_liverA, Unigene15077\_Mf\_liverA, Unigene29134\_Mf\_liverA, CL3800.Contig1\_Mf\_liverA, CL2478.Contig3\_Mf\_liverA, Unigene34375\_Mf\_liverA, CL3055.Contig1\_Mf\_liverA, NM\_009609, CL4033.Contig1\_Mf\_liverA, NM\_007409, Unigene34983\_Mf\_liverA, CL2439.Contig1\_Mf\_liverA, Unigene3439\_Mf\_liverA, Unigene35935\_Mf\_liverA, Unigene33980\_Mf\_liverA, Unigene37238\_Mf\_liverA, Unigene21026\_Mf\_liverA, Unigene36937\_Mf\_liverA, Unigene13683\_Mf\_liverA, CL1907.Contig1\_Mf\_liverA, Unigene35476\_Mf\_liverA, CL5828.Contig2\_Mf\_liverA, Unigene24858\_Mf\_liverA, CL4220.Contig1\_Mf\_liverA, NR\_033215, CL2702.Contig2\_Mf\_liverA, Unigene24466\_Mf\_liverA, CL1165.Contig2\_Mf\_liverA, NM\_007822, Unigene25595\_Mf\_liverA, CL3312.Contig1\_Mf\_liverA, Unigene15470\_Mf\_liverA, Unigene14286\_Mf\_liverA, Unigene34628\_Mf\_liverA, Unigene14212\_Mf\_liverA, Unigene39011\_Mf\_liverA, Unigene26250\_Mf\_liverA, NM\_025336, Unigene35609\_Mf\_liverA, NM\_145218, Unigene36189\_Mf\_liverA, Unigene13950\_Mf\_liverA, Unigene35046\_Mf\_liverA, Unigene28459\_Mf\_liverA, Unigene37245\_Mf\_liverA, Unigene281\_Mf\_liverA, NM\_011978, CL634.Contig1\_Mf\_liverA, NM\_130450, Unigene29985\_Mf\_liverA, Unigene36593\_Mf\_liverA, NM\_027406, Unigene37232\_Mf\_liverA, Unigene37239\_Mf\_liverA, Unigene37028\_Mf\_liverA, CL5698.Contig1\_Mf\_liverA, Unigene31392\_Mf\_liverA, NM\_020559, Unigene28142\_Mf\_liverA, Unigene38015\_Mf\_liverA, CL3835.Contig2\_Mf\_liverA, Unigene36735\_Mf\_liverA, CL523.Contig1\_Mf\_liverA, Unigene36762\_Mf\_liverA, Unigene31065\_Mf\_liverA, NM\_007820, NM\_011072, Unigene20853\_Mf\_liverA, Unigene5428\_Mf\_liverA, Unigene27592\_Mf\_liverA, Unigene24351\_Mf\_liverA, Unigene37999\_Mf\_liverA, Unigene12999\_Mf\_liverA, CL4141.Contig1\_Mf\_liverA, Unigene13658\_Mf\_liverA, Unigene32316\_Mf\_liverA, Unigene25596\_Mf\_liverA, Unigene28548\_Mf\_liverA, Unigene5294\_Mf\_liverA, CL5268.Contig1\_Mf\_liverA, Unigene23328\_Mf\_liverA, Unigene38065\_Mf\_liverA, Unigene35171\_Mf\_liverA, Unigene36626\_Mf\_liverA, Unigene39507\_Mf\_liverA, NM\_029562, Unigene5755\_Mf\_liverA, CL3750.Contig2\_Mf\_liverA, Unigene39141\_Mf\_liverA, Unigene5382\_Mf\_liverA, CL3599.Contig1\_Mf\_liverA, CL1595.Contig1\_Mf\_liverA, CL838.Contig3\_Mf\_liverA, CL4550.Contig3\_Mf\_liverA, CL1263.Contig1\_Mf\_liverA, NM\_029692, Unigene36628\_Mf\_liverA, Unigene12786\_Mf\_liverA, Unigene31517\_Mf\_liverA, NM\_007811, NM\_008538, CL1716.Contig2\_Mf\_liverA, NM\_011305, Unigene13498\_Mf\_liverA, Unigene4510\_Mf\_liverA, CL4220.Contig2\_Mf\_liverA, Unigene17579\_Mf\_liverA, Unigene19885\_Mf\_liverA, CL2550.Contig1\_Mf\_liverA, Unigene7195\_Mf\_liverA, Unigene8132\_Mf\_liverA, Unigene5355\_Mf\_liverA, Unigene35506\_Mf\_liverA, NM\_019717, Unigene5712\_Mf\_liverA, Unigene1205\_Mf\_liverA, CL1509.Contig2\_Mf\_liverA, Unigene13233\_Mf\_liverA, NM\_010481, Unigene10136\_Mf\_liverA, NM\_009128, NM\_011732, CL2240.Contig1\_Mf\_liverA, Unigene13414\_Mf\_liverA, Unigene49971\_Mf\_liverA, Unigene36190\_Mf\_liverA, Unigene5210\_Mf\_liverA, Unigene38208\_Mf\_liverA, CL4162.Contig1\_Mf\_liverA, Unigene112\_Mf\_liverA, CL2517.Contig1\_Mf\_liverA, Unigene24931\_Mf\_liverA, Unigene29510\_Mf\_liverA, CL5986.Contig1\_Mf\_liverA, Unigene7350\_Mf\_liverA, Unigene25751\_Mf\_liverA, CL3569.Contig2\_Mf\_liverA, CL4583.Contig2\_Mf\_liverA, CL3002.Contig1\_Mf\_liverA, Unigene38280\_Mf\_liverA, Unigene36315\_Mf\_liverA, Unigene36784\_Mf\_liverA, Unigene31483\_Mf\_liverA, Unigene40262\_Mf\_liverA, Unigene36514\_Mf\_liverA, CL887.Contig2\_Mf\_liverA, Unigene29593\_Mf\_liverA, CL4816.Contig2\_Mf\_liverA, NM\_023294, Unigene35037\_Mf\_liverA, Unigene14810\_Mf\_liverA, NM\_001100181, Unigene15252\_Mf\_liverA, CL838.Contig6\_Mf\_liverA, CL2791.Contig1\_Mf\_liverA, CL4770.Contig1\_Mf\_liverA, Unigene10135\_Mf\_liverA, CL3750.Contig1\_Mf\_liverA, Unigene25101\_Mf\_liverA, NM\_009883, Unigene26138\_Mf\_liverA, CL1518.Contig1\_Mf\_liverA, CL5307.Contig1\_Mf\_liverA, NM\_008180, NM\_007585, NM\_178405, Unigene21163\_Mf\_liverA, NM\_172203, Unigene5335\_Mf\_liverA, Unigene30731\_Mf\_liverA, CL4007.Contig1\_Mf\_liverA, NM\_017379, CL2355.Contig1\_Mf\_liverA, Unigene14809\_Mf\_liverA, Unigene39686\_Mf\_liverA, Unigene22500\_Mf\_liverA, NM\_009011, CL3911.Contig2\_Mf\_liverA, Unigene21359\_Mf\_liverA, CL336.Contig3\_Mf\_liverA, Unigene28989\_Mf\_liverA, Unigene19954\_Mf\_liverA, NM\_007819, NM\_146120 |
| clathrin-coated vesicle | Unigene15470\_Mf\_liverA, CL4925.Contig1\_Mf\_liverA |
| membrane-enclosed lumen | Unigene35476\_Mf\_liverA, NM\_010481, Unigene38015\_Mf\_liverA, Unigene24092\_Mf\_liverA, NM\_009883, Unigene24858\_Mf\_liverA, Unigene26138\_Mf\_liverA, NM\_177093, CL3599.Contig1\_Mf\_liverA, Unigene5382\_Mf\_liverA, Unigene13414\_Mf\_liverA, Unigene33512\_Mf\_liverA, Unigene12959\_Mf\_liverA, Unigene5210\_Mf\_liverA, Unigene1479\_Mf\_liverA, CL2702.Contig2\_Mf\_liverA, CL1803.Contig1\_Mf\_liverA, Unigene24466\_Mf\_liverA, Unigene14916\_Mf\_liverA, Unigene21163\_Mf\_liverA, CL4162.Contig1\_Mf\_liverA, Unigene31517\_Mf\_liverA, Unigene25595\_Mf\_liverA, CL1716.Contig2\_Mf\_liverA, CL2517.Contig1\_Mf\_liverA, Unigene36112\_Mf\_liverA, NM\_011305, Unigene14212\_Mf\_liverA, Unigene7350\_Mf\_liverA, Unigene30731\_Mf\_liverA, Unigene13498\_Mf\_liverA, Unigene29134\_Mf\_liverA, CL3569.Contig2\_Mf\_liverA, CL2790.Contig1\_Mf\_liverA, CL4583.Contig2\_Mf\_liverA, Unigene24351\_Mf\_liverA, CL2550.Contig1\_Mf\_liverA, Unigene33366\_Mf\_liverA, Unigene34375\_Mf\_liverA, CL2355.Contig1\_Mf\_liverA, Unigene45530\_Mf\_liverA, NM\_009609, CL4141.Contig1\_Mf\_liverA, Unigene14809\_Mf\_liverA, Unigene39686\_Mf\_liverA, Unigene36662\_Mf\_liverA, Unigene5355\_Mf\_liverA, Unigene35816\_Mf\_liverA, Unigene13658\_Mf\_liverA, NM\_009011, NM\_023294, Unigene35935\_Mf\_liverA, Unigene33980\_Mf\_liverA, CL3911.Contig2\_Mf\_liverA, Unigene25596\_Mf\_liverA, Unigene37028\_Mf\_liverA, Unigene21359\_Mf\_liverA, Unigene31392\_Mf\_liverA, Unigene30288\_Mf\_liverA, Unigene13683\_Mf\_liverA, CL1907.Contig1\_Mf\_liverA, Unigene15252\_Mf\_liverA, CL2791.Contig1\_Mf\_liverA, Unigene28548\_Mf\_liverA, CL5268.Contig1\_Mf\_liverA, Unigene23328\_Mf\_liverA |
| chromosome | Unigene21163\_Mf\_liverA, Unigene31517\_Mf\_liverA, Unigene4684\_Mf\_liverA, CL2550.Contig1\_Mf\_liverA, Unigene24092\_Mf\_liverA, NM\_009883, NM\_011305, Unigene24858\_Mf\_liverA, Unigene31392\_Mf\_liverA, Unigene45530\_Mf\_liverA, CL5268.Contig1\_Mf\_liverA, NM\_023294 |
| nucleus | Unigene25962\_Mf\_liverA, Unigene21684\_Mf\_liverA, Unigene38015\_Mf\_liverA, Unigene28142\_Mf\_liverA, CL3835.Contig2\_Mf\_liverA, NM\_011732, Unigene29727\_Mf\_liverA, CL186.Contig3\_Mf\_liverA, Unigene13414\_Mf\_liverA, Unigene5210\_Mf\_liverA, Unigene15553\_Mf\_liverA, Unigene172\_Mf\_liverA, CL1803.Contig1\_Mf\_liverA, Unigene14916\_Mf\_liverA, CL4162.Contig1\_Mf\_liverA, CL4577.Contig1\_Mf\_liverA, Unigene29510\_Mf\_liverA, Unigene24931\_Mf\_liverA, Unigene6465\_Mf\_liverA, CL2982.Contig1\_Mf\_liverA, Unigene39886\_Mf\_liverA, Unigene7350\_Mf\_liverA, Unigene20853\_Mf\_liverA, Unigene29134\_Mf\_liverA, Unigene27592\_Mf\_liverA, CL3569.Contig2\_Mf\_liverA, CL2790.Contig1\_Mf\_liverA, CL3800.Contig1\_Mf\_liverA, CL2478.Contig3\_Mf\_liverA, Unigene24351\_Mf\_liverA, CL3002.Contig1\_Mf\_liverA, Unigene31483\_Mf\_liverA, NM\_009609, CL4141.Contig1\_Mf\_liverA, Unigene34983\_Mf\_liverA, CL2439.Contig1\_Mf\_liverA, Unigene35816\_Mf\_liverA, Unigene13658\_Mf\_liverA, NM\_023294, Unigene32316\_Mf\_liverA, Unigene35935\_Mf\_liverA, Unigene33980\_Mf\_liverA, Unigene29461\_Mf\_liverA, Unigene13683\_Mf\_liverA, CL1907.Contig1\_Mf\_liverA, CL2791.Contig1\_Mf\_liverA, CL838.Contig6\_Mf\_liverA, CL4770.Contig1\_Mf\_liverA, CL5268.Contig1\_Mf\_liverA, CL4995.Contig1\_Mf\_liverA, Unigene38065\_Mf\_liverA, Unigene35476\_Mf\_liverA, CL2971.Contig1\_Mf\_liverA, Unigene39507\_Mf\_liverA, Unigene4684\_Mf\_liverA, Unigene24092\_Mf\_liverA, Unigene24858\_Mf\_liverA, NM\_009883, Unigene26138\_Mf\_liverA, NM\_177093, Unigene33512\_Mf\_liverA, Unigene39141\_Mf\_liverA, Unigene1479\_Mf\_liverA, CL2702.Contig2\_Mf\_liverA, CL838.Contig3\_Mf\_liverA, CL4332.Contig1\_Mf\_liverA, Unigene24466\_Mf\_liverA, CL1165.Contig2\_Mf\_liverA, CL1263.Contig1\_Mf\_liverA, Unigene21163\_Mf\_liverA, Unigene36628\_Mf\_liverA, Unigene31517\_Mf\_liverA, CL3312.Contig1\_Mf\_liverA, NM\_008538, NM\_011305, Unigene14212\_Mf\_liverA, Unigene39011\_Mf\_liverA, Unigene26250\_Mf\_liverA, Unigene30731\_Mf\_liverA, Unigene23158\_Mf\_liverA, Unigene13950\_Mf\_liverA, Unigene17579\_Mf\_liverA, Unigene19885\_Mf\_liverA, CL2550.Contig1\_Mf\_liverA, Unigene33366\_Mf\_liverA, CL2355.Contig1\_Mf\_liverA, Unigene33898\_Mf\_liverA, Unigene45530\_Mf\_liverA, Unigene29985\_Mf\_liverA, Unigene39686\_Mf\_liverA, Unigene8132\_Mf\_liverA, NM\_009011, Unigene37028\_Mf\_liverA, Unigene31392\_Mf\_liverA, Unigene30288\_Mf\_liverA, Unigene32943\_Mf\_liverA, CL1509.Contig2\_Mf\_liverA, Unigene19954\_Mf\_liverA, Unigene45904\_Mf\_liverA |
| nucleoplasm part | Unigene35935\_Mf\_liverA, CL2790.Contig1\_Mf\_liverA, Unigene21163\_Mf\_liverA, Unigene31517\_Mf\_liverA, Unigene33366\_Mf\_liverA, CL2355.Contig1\_Mf\_liverA, Unigene30288\_Mf\_liverA, NM\_177093, NM\_009609, Unigene39686\_Mf\_liverA, Unigene1479\_Mf\_liverA, Unigene35816\_Mf\_liverA, Unigene13658\_Mf\_liverA |
| protein complex | Unigene5639\_Mf\_liverA, Unigene16261\_Mf\_liverA, NM\_023868, Unigene4684\_Mf\_liverA, CL3835.Contig2\_Mf\_liverA, NM\_011732, CL4925.Contig1\_Mf\_liverA, NM\_177093, CL425.Contig1\_Mf\_liverA, Unigene49971\_Mf\_liverA, Unigene1479\_Mf\_liverA, CL2702.Contig2\_Mf\_liverA, NM\_029692, CL2339.Contig1\_Mf\_liverA, Unigene21163\_Mf\_liverA, CL4162.Contig1\_Mf\_liverA, Unigene37526\_Mf\_liverA, Unigene31517\_Mf\_liverA, Unigene15583\_Mf\_liverA, Unigene4523\_Mf\_liverA, Unigene26250\_Mf\_liverA, Unigene7350\_Mf\_liverA, Unigene13950\_Mf\_liverA, CL3800.Contig1\_Mf\_liverA, CL2790.Contig1\_Mf\_liverA, CL3569.Contig2\_Mf\_liverA, Unigene37245\_Mf\_liverA, Unigene18500\_Mf\_liverA, Unigene27547\_Mf\_liverA, Unigene19885\_Mf\_liverA, Unigene4938\_Mf\_liverA, CL2550.Contig1\_Mf\_liverA, Unigene33366\_Mf\_liverA, CL2355.Contig1\_Mf\_liverA, CL3002.Contig1\_Mf\_liverA, CL634.Contig1\_Mf\_liverA, Unigene45530\_Mf\_liverA, Unigene12999\_Mf\_liverA, NM\_009609, CL4141.Contig1\_Mf\_liverA, Unigene29593\_Mf\_liverA, CL887.Contig2\_Mf\_liverA, Unigene4636\_Mf\_liverA, Unigene35816\_Mf\_liverA, Unigene13658\_Mf\_liverA, CL2260.Contig1\_Mf\_liverA, NM\_009011, NM\_023294, Unigene37232\_Mf\_liverA, Unigene35935\_Mf\_liverA, Unigene37028\_Mf\_liverA, CL5698.Contig1\_Mf\_liverA, Unigene31392\_Mf\_liverA, Unigene30288\_Mf\_liverA, CL3684.Contig2\_Mf\_liverA, Unigene13683\_Mf\_liverA, Unigene33683\_Mf\_liverA, CL275.Contig5\_Mf\_liverA, CL5268.Contig1\_Mf\_liverA, Unigene5294\_Mf\_liverA |
| cell leading edge | Unigene39011\_Mf\_liverA, Unigene25962\_Mf\_liverA, Unigene21684\_Mf\_liverA, NM\_146120 |
| spindle | Unigene26250\_Mf\_liverA, Unigene33366\_Mf\_liverA |
| adherens junction | CL838.Contig3\_Mf\_liverA, Unigene13950\_Mf\_liverA |
| organelle lumen | NM\_010481, Unigene38015\_Mf\_liverA, Unigene24092\_Mf\_liverA, NM\_009883, Unigene24858\_Mf\_liverA, Unigene26138\_Mf\_liverA, NM\_177093, CL3599.Contig1\_Mf\_liverA, Unigene5382\_Mf\_liverA, Unigene13414\_Mf\_liverA, Unigene33512\_Mf\_liverA, Unigene12959\_Mf\_liverA, Unigene5210\_Mf\_liverA, Unigene1479\_Mf\_liverA, CL2702.Contig2\_Mf\_liverA, CL1803.Contig1\_Mf\_liverA, Unigene24466\_Mf\_liverA, Unigene14916\_Mf\_liverA, Unigene21163\_Mf\_liverA, CL4162.Contig1\_Mf\_liverA, Unigene31517\_Mf\_liverA, Unigene25595\_Mf\_liverA, CL1716.Contig2\_Mf\_liverA, CL2517.Contig1\_Mf\_liverA, Unigene36112\_Mf\_liverA, NM\_011305, Unigene14212\_Mf\_liverA, Unigene7350\_Mf\_liverA, Unigene30731\_Mf\_liverA, Unigene13498\_Mf\_liverA, Unigene29134\_Mf\_liverA, CL3569.Contig2\_Mf\_liverA, CL2790.Contig1\_Mf\_liverA, CL4583.Contig2\_Mf\_liverA, Unigene24351\_Mf\_liverA, CL2550.Contig1\_Mf\_liverA, Unigene33366\_Mf\_liverA, Unigene34375\_Mf\_liverA, CL2355.Contig1\_Mf\_liverA, Unigene45530\_Mf\_liverA, NM\_009609, CL4141.Contig1\_Mf\_liverA, Unigene14809\_Mf\_liverA, Unigene39686\_Mf\_liverA, Unigene5355\_Mf\_liverA, Unigene35816\_Mf\_liverA, Unigene13658\_Mf\_liverA, NM\_009011, NM\_023294, Unigene35935\_Mf\_liverA, Unigene33980\_Mf\_liverA, CL3911.Contig2\_Mf\_liverA, Unigene25596\_Mf\_liverA, Unigene37028\_Mf\_liverA, Unigene21359\_Mf\_liverA, Unigene31392\_Mf\_liverA, Unigene30288\_Mf\_liverA, Unigene13683\_Mf\_liverA, CL1907.Contig1\_Mf\_liverA, Unigene15252\_Mf\_liverA, CL2791.Contig1\_Mf\_liverA, CL5268.Contig1\_Mf\_liverA, Unigene23328\_Mf\_liverA |
| coated vesicle | Unigene15470\_Mf\_liverA, CL4925.Contig1\_Mf\_liverA |
| cytoplasmic vesicle membrane | Unigene8054\_Mf\_liverA, CL4925.Contig1\_Mf\_liverA |
| nuclear body | Unigene39686\_Mf\_liverA, CL2355.Contig1\_Mf\_liverA, Unigene35816\_Mf\_liverA |
| anchoring junction | CL838.Contig3\_Mf\_liverA, Unigene13950\_Mf\_liverA |
| cytoskeletal part | Unigene4732\_Mf\_liverA, CL3569.Contig2\_Mf\_liverA, Unigene27547\_Mf\_liverA, Unigene5639\_Mf\_liverA, Unigene4731\_Mf\_liverA, Unigene33366\_Mf\_liverA, CL634.Contig1\_Mf\_liverA, NM\_009609, NM\_177093, CL425.Contig1\_Mf\_liverA, NM\_007585, NM\_029692, CL4162.Contig1\_Mf\_liverA, Unigene27895\_Mf\_liverA, NM\_008538, CL5698.Contig1\_Mf\_liverA, Unigene26250\_Mf\_liverA, Unigene13498\_Mf\_liverA |
| intracellular organelle lumen | NM\_010481, Unigene38015\_Mf\_liverA, Unigene24092\_Mf\_liverA, NM\_009883, Unigene24858\_Mf\_liverA, Unigene26138\_Mf\_liverA, NM\_177093, CL3599.Contig1\_Mf\_liverA, Unigene5382\_Mf\_liverA, Unigene13414\_Mf\_liverA, Unigene33512\_Mf\_liverA, Unigene5210\_Mf\_liverA, Unigene1479\_Mf\_liverA, CL2702.Contig2\_Mf\_liverA, CL1803.Contig1\_Mf\_liverA, Unigene24466\_Mf\_liverA, Unigene14916\_Mf\_liverA, Unigene21163\_Mf\_liverA, CL4162.Contig1\_Mf\_liverA, Unigene31517\_Mf\_liverA, Unigene25595\_Mf\_liverA, CL1716.Contig2\_Mf\_liverA, CL2517.Contig1\_Mf\_liverA, Unigene36112\_Mf\_liverA, NM\_011305, Unigene14212\_Mf\_liverA, Unigene7350\_Mf\_liverA, Unigene30731\_Mf\_liverA, Unigene13498\_Mf\_liverA, Unigene29134\_Mf\_liverA, CL2790.Contig1\_Mf\_liverA, CL3569.Contig2\_Mf\_liverA, CL4583.Contig2\_Mf\_liverA, Unigene24351\_Mf\_liverA, CL2550.Contig1\_Mf\_liverA, Unigene33366\_Mf\_liverA, Unigene34375\_Mf\_liverA, CL2355.Contig1\_Mf\_liverA, Unigene45530\_Mf\_liverA, NM\_009609, CL4141.Contig1\_Mf\_liverA, Unigene39686\_Mf\_liverA, Unigene35816\_Mf\_liverA, Unigene13658\_Mf\_liverA, NM\_009011, NM\_023294, Unigene35935\_Mf\_liverA, Unigene33980\_Mf\_liverA, CL3911.Contig2\_Mf\_liverA, Unigene25596\_Mf\_liverA, Unigene37028\_Mf\_liverA, Unigene21359\_Mf\_liverA, Unigene31392\_Mf\_liverA, Unigene30288\_Mf\_liverA, Unigene13683\_Mf\_liverA, CL1907.Contig1\_Mf\_liverA, Unigene15252\_Mf\_liverA, CL2791.Contig1\_Mf\_liverA, CL5268.Contig1\_Mf\_liverA, Unigene23328\_Mf\_liverA |
| centrosome | Unigene13498\_Mf\_liverA, Unigene27895\_Mf\_liverA |
| vesicle membrane | Unigene8054\_Mf\_liverA, CL4925.Contig1\_Mf\_liverA |
| macromolecular complex | NM\_023868, CL3835.Contig2\_Mf\_liverA, NM\_011732, CL4925.Contig1\_Mf\_liverA, CL425.Contig1\_Mf\_liverA, Unigene49971\_Mf\_liverA, CL2339.Contig1\_Mf\_liverA, CL4162.Contig1\_Mf\_liverA, Unigene37526\_Mf\_liverA, Unigene7350\_Mf\_liverA, Unigene46931\_Mf\_liverA, CL3569.Contig2\_Mf\_liverA, CL3800.Contig1\_Mf\_liverA, CL2790.Contig1\_Mf\_liverA, Unigene18500\_Mf\_liverA, Unigene4938\_Mf\_liverA, Unigene34477\_Mf\_liverA, CL3002.Contig1\_Mf\_liverA, NM\_009609, Unigene12999\_Mf\_liverA, CL4141.Contig1\_Mf\_liverA, Unigene29593\_Mf\_liverA, CL887.Contig2\_Mf\_liverA, CL2439.Contig1\_Mf\_liverA, Unigene35816\_Mf\_liverA, Unigene13658\_Mf\_liverA, CL2260.Contig1\_Mf\_liverA, NM\_023294, Unigene35935\_Mf\_liverA, Unigene559\_Mf\_liverA, CL3684.Contig2\_Mf\_liverA, Unigene13683\_Mf\_liverA, Unigene33683\_Mf\_liverA, CL5268.Contig1\_Mf\_liverA, Unigene5294\_Mf\_liverA, Unigene16261\_Mf\_liverA, Unigene5639\_Mf\_liverA, Unigene4684\_Mf\_liverA, NM\_177093, Unigene1479\_Mf\_liverA, NM\_007585, CL2702.Contig2\_Mf\_liverA, NM\_029692, Unigene21163\_Mf\_liverA, Unigene36698\_Mf\_liverA, Unigene31517\_Mf\_liverA, Unigene15583\_Mf\_liverA, Unigene4523\_Mf\_liverA, Unigene26250\_Mf\_liverA, Unigene13498\_Mf\_liverA, Unigene13950\_Mf\_liverA, NM\_017379, Unigene19885\_Mf\_liverA, Unigene27547\_Mf\_liverA, Unigene37245\_Mf\_liverA, Unigene33366\_Mf\_liverA, CL2550.Contig1\_Mf\_liverA, CL2355.Contig1\_Mf\_liverA, Unigene45530\_Mf\_liverA, CL634.Contig1\_Mf\_liverA, Unigene4636\_Mf\_liverA, NM\_027406, NM\_009011, Unigene37232\_Mf\_liverA, CL3911.Contig2\_Mf\_liverA, Unigene37028\_Mf\_liverA, CL5698.Contig1\_Mf\_liverA, Unigene30288\_Mf\_liverA, Unigene31392\_Mf\_liverA, CL275.Contig5\_Mf\_liverA, NM\_146120 |
| nuclear part | CL2971.Contig1\_Mf\_liverA, Unigene38015\_Mf\_liverA, Unigene28142\_Mf\_liverA, Unigene24092\_Mf\_liverA, NM\_011732, Unigene29727\_Mf\_liverA, NM\_009883, Unigene24858\_Mf\_liverA, Unigene26138\_Mf\_liverA, NM\_177093, Unigene13414\_Mf\_liverA, Unigene33512\_Mf\_liverA, Unigene5210\_Mf\_liverA, Unigene1479\_Mf\_liverA, Unigene172\_Mf\_liverA, CL2702.Contig2\_Mf\_liverA, CL1803.Contig1\_Mf\_liverA, Unigene24466\_Mf\_liverA, CL1263.Contig1\_Mf\_liverA, Unigene14916\_Mf\_liverA, Unigene21163\_Mf\_liverA, CL4162.Contig1\_Mf\_liverA, Unigene31517\_Mf\_liverA, NM\_011305, Unigene14212\_Mf\_liverA, Unigene7350\_Mf\_liverA, Unigene30731\_Mf\_liverA, Unigene29134\_Mf\_liverA, Unigene27592\_Mf\_liverA, CL2790.Contig1\_Mf\_liverA, CL3569.Contig2\_Mf\_liverA, Unigene19885\_Mf\_liverA, CL2478.Contig3\_Mf\_liverA, Unigene24351\_Mf\_liverA, CL2550.Contig1\_Mf\_liverA, Unigene33366\_Mf\_liverA, CL2355.Contig1\_Mf\_liverA, Unigene45530\_Mf\_liverA, NM\_009609, CL4141.Contig1\_Mf\_liverA, Unigene39686\_Mf\_liverA, Unigene8132\_Mf\_liverA, Unigene35816\_Mf\_liverA, Unigene13658\_Mf\_liverA, NM\_009011, NM\_023294, Unigene35935\_Mf\_liverA, Unigene33980\_Mf\_liverA, Unigene37028\_Mf\_liverA, Unigene31392\_Mf\_liverA, Unigene30288\_Mf\_liverA, Unigene13683\_Mf\_liverA, Unigene32943\_Mf\_liverA, CL1907.Contig1\_Mf\_liverA, CL2791.Contig1\_Mf\_liverA, CL5268.Contig1\_Mf\_liverA |
| microtubule organizing center | Unigene13498\_Mf\_liverA, NM\_008538, Unigene27895\_Mf\_liverA |
| microtubule cytoskeleton | CL3569.Contig2\_Mf\_liverA, Unigene27547\_Mf\_liverA, NM\_017379, Unigene33366\_Mf\_liverA, NM\_008538, Unigene27895\_Mf\_liverA, Unigene26250\_Mf\_liverA, CL425.Contig1\_Mf\_liverA, Unigene13498\_Mf\_liverA |
| cytoskeleton | Unigene4732\_Mf\_liverA, CL3569.Contig2\_Mf\_liverA, Unigene27547\_Mf\_liverA, Unigene5639\_Mf\_liverA, NM\_017379, Unigene4731\_Mf\_liverA, Unigene33366\_Mf\_liverA, CL634.Contig1\_Mf\_liverA, NM\_009609, NM\_177093, NM\_021508, CL425.Contig1\_Mf\_liverA, NM\_007585, NM\_029692, CL4162.Contig1\_Mf\_liverA, Unigene37238\_Mf\_liverA, Unigene27895\_Mf\_liverA, NM\_008538, CL5698.Contig1\_Mf\_liverA, NM\_011072, Unigene26250\_Mf\_liverA, Unigene25751\_Mf\_liverA, Unigene13498\_Mf\_liverA, NM\_146120 |
| ribonucleoprotein complex | Unigene12999\_Mf\_liverA, NM\_011732, Unigene35816\_Mf\_liverA, Unigene13683\_Mf\_liverA |
| nuclear lumen | Unigene38015\_Mf\_liverA, Unigene24092\_Mf\_liverA, NM\_009883, Unigene24858\_Mf\_liverA, Unigene26138\_Mf\_liverA, NM\_177093, Unigene33512\_Mf\_liverA, Unigene5210\_Mf\_liverA, Unigene1479\_Mf\_liverA, CL2702.Contig2\_Mf\_liverA, CL1803.Contig1\_Mf\_liverA, Unigene24466\_Mf\_liverA, Unigene21163\_Mf\_liverA, Unigene14916\_Mf\_liverA, CL4162.Contig1\_Mf\_liverA, Unigene31517\_Mf\_liverA, NM\_011305, Unigene14212\_Mf\_liverA, Unigene7350\_Mf\_liverA, Unigene30731\_Mf\_liverA, Unigene29134\_Mf\_liverA, CL2790.Contig1\_Mf\_liverA, CL3569.Contig2\_Mf\_liverA, Unigene24351\_Mf\_liverA, Unigene33366\_Mf\_liverA, CL2550.Contig1\_Mf\_liverA, CL2355.Contig1\_Mf\_liverA, Unigene45530\_Mf\_liverA, NM\_009609, CL4141.Contig1\_Mf\_liverA, Unigene39686\_Mf\_liverA, Unigene35816\_Mf\_liverA, Unigene13658\_Mf\_liverA, NM\_023294, NM\_009011, Unigene35935\_Mf\_liverA, Unigene33980\_Mf\_liverA, Unigene37028\_Mf\_liverA, Unigene31392\_Mf\_liverA, Unigene30288\_Mf\_liverA, Unigene13683\_Mf\_liverA, CL1907.Contig1\_Mf\_liverA, CL2791.Contig1\_Mf\_liverA, CL5268.Contig1\_Mf\_liverA |
| non-membrane-bounded organelle | Unigene5639\_Mf\_liverA, NM\_023868, Unigene4684\_Mf\_liverA, Unigene24092\_Mf\_liverA, NM\_009883, Unigene24858\_Mf\_liverA, Unigene26138\_Mf\_liverA, NM\_177093, NM\_021508, CL425.Contig1\_Mf\_liverA, Unigene1479\_Mf\_liverA, CL2702.Contig2\_Mf\_liverA, NM\_007585, CL1803.Contig1\_Mf\_liverA, Unigene24466\_Mf\_liverA, NM\_080639, NM\_029692, Unigene14916\_Mf\_liverA, Unigene21163\_Mf\_liverA, CL4162.Contig1\_Mf\_liverA, Unigene31517\_Mf\_liverA, NM\_008538, Unigene27895\_Mf\_liverA, NM\_011305, Unigene14212\_Mf\_liverA, NM\_011072, Unigene26250\_Mf\_liverA, Unigene30731\_Mf\_liverA, Unigene25751\_Mf\_liverA, Unigene13498\_Mf\_liverA, Unigene4732\_Mf\_liverA, CL3569.Contig2\_Mf\_liverA, Unigene27547\_Mf\_liverA, NM\_017379, Unigene4731\_Mf\_liverA, Unigene24351\_Mf\_liverA, CL2550.Contig1\_Mf\_liverA, Unigene33366\_Mf\_liverA, CL634.Contig1\_Mf\_liverA, Unigene45530\_Mf\_liverA, Unigene12999\_Mf\_liverA, NM\_009609, Unigene39686\_Mf\_liverA, Unigene29593\_Mf\_liverA, Unigene8132\_Mf\_liverA, NM\_023294, Unigene37238\_Mf\_liverA, Unigene37028\_Mf\_liverA, CL5698.Contig1\_Mf\_liverA, Unigene31392\_Mf\_liverA, Unigene13683\_Mf\_liverA, CL1907.Contig1\_Mf\_liverA, CL2791.Contig1\_Mf\_liverA, CL5268.Contig1\_Mf\_liverA, NM\_146120 |
| intracellular non-membrane-bounded organelle | Unigene5639\_Mf\_liverA, NM\_023868, Unigene4684\_Mf\_liverA, Unigene24092\_Mf\_liverA, NM\_009883, Unigene24858\_Mf\_liverA, Unigene26138\_Mf\_liverA, NM\_177093, NM\_021508, CL425.Contig1\_Mf\_liverA, Unigene1479\_Mf\_liverA, CL2702.Contig2\_Mf\_liverA, NM\_007585, CL1803.Contig1\_Mf\_liverA, Unigene24466\_Mf\_liverA, NM\_080639, NM\_029692, Unigene14916\_Mf\_liverA, Unigene21163\_Mf\_liverA, CL4162.Contig1\_Mf\_liverA, Unigene31517\_Mf\_liverA, NM\_008538, Unigene27895\_Mf\_liverA, NM\_011305, Unigene14212\_Mf\_liverA, NM\_011072, Unigene26250\_Mf\_liverA, Unigene30731\_Mf\_liverA, Unigene25751\_Mf\_liverA, Unigene13498\_Mf\_liverA, Unigene4732\_Mf\_liverA, CL3569.Contig2\_Mf\_liverA, Unigene27547\_Mf\_liverA, NM\_017379, Unigene4731\_Mf\_liverA, Unigene24351\_Mf\_liverA, CL2550.Contig1\_Mf\_liverA, Unigene33366\_Mf\_liverA, CL634.Contig1\_Mf\_liverA, Unigene45530\_Mf\_liverA, Unigene12999\_Mf\_liverA, NM\_009609, Unigene39686\_Mf\_liverA, Unigene29593\_Mf\_liverA, Unigene8132\_Mf\_liverA, NM\_023294, Unigene37238\_Mf\_liverA, Unigene37028\_Mf\_liverA, CL5698.Contig1\_Mf\_liverA, Unigene31392\_Mf\_liverA, Unigene13683\_Mf\_liverA, CL1907.Contig1\_Mf\_liverA, CL2791.Contig1\_Mf\_liverA, CL5268.Contig1\_Mf\_liverA, NM\_146120 |
|
